# A novel oncogenic seRNA promotes nasopharyngeal carcinoma metastasis

**DOI:** 10.1038/s41419-022-04846-1

**Published:** 2022-04-23

**Authors:** Yuan Tan, Chonghua Jiang, Qunying Jia, Jing Wang, Ge Huang, Faqing Tang

**Affiliations:** 1 Clinical Laboratory of Hunan Cancer Hospital and the Affiliated Cancer Hospital of Xiangya School of Medicine, Central South University, Hunan Key Laboratory of Oncotarget Gene, Changsha, China; 2 Institute of Medical Technology, Peking University Health Science Center, Beijing, China; 3 Affiliated Haikou Hospital of Xiangya Medical College, Central South University, Haikou, China

**Keywords:** Metastasis, Oncogenes

## Abstract

Nasopharyngeal carcinoma (NPC) is a common malignant cancer in southern China that has highly invasive and metastatic features and causes high mortality, but the underlying mechanisms of this malignancy remain unclear. In this study, we utilized ChIP-Seq to identify metastasis-specific super enhancers (SEs) and found that the SE of LOC100506178 existed only in metastatic NPC cells and powerfully aggravated NPC metastasis. This metastatic SE transcribed into lncRNA LOC100506178, and it was verified as a seRNA through GRO-Seq. Furthermore, SE-derived seRNA LOC100506178 was found to be highly expressed in metastatic NPC cells and NPC lymph node metastatic tissues. Knockdown of seRNA LOC100506178 arrested the invasion and metastasis of NPC cells in vitro and in vivo, demonstrating that seRNA LOC100506178 accelerates the acquisition of NPC malignant phenotype. Mechanistic studies revealed that seRNA LOC100506178 specifically interacted with the transcription factor hnRNPK and modulated the expression of hnRNPK. Further, hnRNPK in combination with the promoter region of MICAL2 increased *Mical2* transcription. Knockdown of seRNA LOC100506178 or hnRNPK markedly repressed MICAL2, Vimentin and Snail expression and upregulated E-cadherin expression. Overexpression of seRNA LOC100506178 or hnRNPK markedly increased MICAL2, Vimentin and Snail expression and decreased E-cadherin expression. Therefore, seRNA LOC100506178 may promote MICAL2 expression by upregulating hnRNPK, subsequently enhancing EMT process and accelerating the invasion and metastasis of NPC cells. seRNA LOC100506178 has the potential to serve as a novel prognostic biomarker and therapeutic target in NPC patients.

## Introduction

Clinically, nasopharyngeal carcinoma (NPC) has highly invasive and metastatic features, resulting in high mortality [1]. Although there have been some significant advancements in systemic therapies for NPC, the 5-year survival rate has not markedly increased due to highly metastatic clinicopathological features [2, 3]. Therefore, the molecular mechanisms of NPC metastasis urgently need to be identified.

Super enhancers (SEs) are enriched with large clusters of enhancers, which are vital for facilitating cancer progression [4, 5]. SEs are mainly determined by ChIP-Seq (chromatin immunoprecipitation followed by sequence analysis) dependent on the high histone H3 lysine 27 acetylation (H3K27ac) or RNA polymerase (Pol) II signaling levels [6]. SEs drive much higher levels of transcription and exhibit much greater lineage- and tissue-specificity than typical enhancers (TEs) [7, 8]. Importantly, SEs transcribe into noncoding RNAs (ncRNAs), termed seRNAs, and the production rates of seRNAs rely mainly on the degree of RNA Pol II enrichment [9]. SE-derived seRNAs, including circular RNAs, long noncoding RNAs (lncRNAs), and microRNAs, are crucial modulators of gene expression and numerous pathological processes [10–12] and participate in the progression of various cancers by regulating oncogenic pathways [13, 14]. For instance, the SE-induced seRNA HCCL5 promotes hepatocellular carcinoma (HCC) cells viability, migration and epithelial-to-mesenchymal transition (EMT) by upregulating Snail, Slug, ZEB1, and Twist1 [15]. In squamous cell carcinomas (SCCs), the SCC-specific SE region co-occupied by TP63 and SOX2 enhances seRNA CCAT1 (colon cancer associated transcript 1) transcription, and the TP63/SOX2/CCAT1 complex promotes tumorigenesis by activating EGFR (epidermal growth factor receptor) transcription [16].

EMT is indispensable for triggering NPC invasion and metastasis by endowing cancer cells with the capacity to spread to neighboring and distant tissues, and the process might be regulated by seRNAs [17, 18]. In this study, we performed epigenomic profiling to characterize metastatic SEs and found that SE regulating LOC100506178 was a metastasis-specific SE and its transcribed seRNA accelerated the NPC malignant phenotype. Mechanistic experiments showed that seRNA LOC100506178 bound to hnRNPK-MICAL2 to mediate EMT and exacerbated NPC metastasis, rendering it a novel prognostic biomarker and a therapeutic target for NPC patients.

## Materials and methods

### Cell culture

Human NPC cell lines, S18, S26, 5–8 F and 6-10B were purchased from Shanghai Cell Center (Shanghai, China). S18 and 5–8 F cells have a high-metastatic ability, and S26 and 6-10B cells have a low-metastatic ability [19]. These cells were cultured in RPMI 1640 supplemented with 10% fetal bovine serum (FBS) in a humidified atmosphere of 5% CO_2_. Puromycin (Sigma-Aldrich, St. Louis, MO) was used to select stably transfected cells. JQ1 (Tocris Bioscience, UK) was dissolved in dimethyl sulfoxide (DMSO), and an appropriate amount of JQ1 was added.

### Western blotting

Western blotting was performed as described previously [17]. 1 × 10^6^ cells were lysed with RIPA buffer (Beyotime, Shanghai, China). Cell lysates were obtained, and the protein concentrations of lysates were measured using BCA protein quantitative kit (Beyotime). Forty microgram supernatant protein was separated by SDS-PAGE and transferred onto a PVDF (polyvinylidene fluoride) membrane, and the membrane was blocked with 5% non-fat milk. The proteins were incubated with the following primary antibodies: anti-hnRNPK (ab52600, Abcam, Cambridge, MA), anti-E-cadherin (orb378741, Biorbyt, UK), anti-Vimentin (orb379309, Biobyt), anti-Snail (#3879, Cell Signaling Technology, Beverly, MA), anti-N-cadherin (#13116, Cell Signaling Technology), anti-Slug (#9585, Cell Signaling Technology), anti-MICAL2 (13965-1-AP, Proteintech, Wuhan, China) or anti-β-actin (66009-1-Ig, Proteintech). After incubation with the appropriate secondary antibody, signals were detected using an ECL kit (Thermo Scientific, Rockford, IL).

### Transwell migration and matrigel invasion assays

Transwell migration and Matrigel invasion assays were performed as described previously [17]. For the invasion assay, Matrigel (8–12 mg/ml, Corning, USA) was added to the upper chamber, and 1 × 10^4^ cells in 100 μl serum-free medium was seeded into the upper chamber. 20% FBS-RPMI 1640 was added to the lower chamber, then incubated for 48 h at 37 °C. The invaded cells were fixed with 4% paraformaldehyde and stained with crystal violet, then the number of invaded cells were counted under a light microscope. The motility assay was performed as described in the invasive assay without Matrigel.

### Wound healing assay

A wound-healing assay was performed as described previously [15]. 1.5 × 10^5^ cells were seeded on 6-well plates and incubated at 37 °C. Then the cell monolayer was wounded with a 10 µl pipette tip when cells reached ~90% confluency. Cells were cultured in RPMI 1640 with 1% FBS, and the scratches were photographed at 0, 24, and 48 h. The percent wound of closure was calculated.

### RT-qPCR

RT-qPCR was performed as described previously [20]. Total RNA from cells was extracted with a total RNA kit (OMEGA, USA). 1 µg RNA was reverse transcribed using RevertAid First Strand cDNA Synthesis (Thermo Scientific). qPCR was performed with FastStart Essential DNA Green Master Mix (Roche, Basel, Switzerland). The primer sequences are shown in Supplementary Table 1. RNA expression of target genes was normalized to GAPDH.

### Lentivirus packaging

Six short hairpins (sh) seRNAs for LOC100506178 and one scramble control (shscramble) were designed by GeneKai Company (Shanghai, China), and the sequences are shown in Supplementary Table 2. pSIN-seRNA for LOC100506178 and negative control (NC) were designed by GeneKai Company. 1 × 10^6^ 293 T cells were seeded on 10 cm-culture plates and incubated at 37 °C. Then 293 T cells were co-transfected with the 20 μg plasmids, 15 µg pHelper 1.0, and 10 µg pHelper 2.0 when cells were cultivated to 60–70% confluence. After 8 h, removing the media containing the transfection reagent and adding 10 ml fresh 10% FBS-DMEM per plate. Finally, the supernatant virus was collected after 48 and 72 h.

### Establishing stable cells

Stable cells were selected following the manufacturer’s protocol. 4–5 × 10^4^ cells were seeded on 6-well plates, then transfected with the appropriate virus when cells were cultured to 20–30% confluency. After 12 h, removing the media containing the virus and adding fresh 10% FBS-RPMI 1640. After 48–72 h, puromycin was used to select cells for 3–4 days. Then, collecting cells, extracting RNA, and performing RT-qPCR to detect knockdown efficiencies that are shown in Supplementary Table 2. shseRNA#3 showed the effective knockdown. Ultimately, shseRNA LOC100506178 (shseRNA#3) and shscramble, pSIN-seRNA, and NC vector-transfected stable cells were obtained.

### siRNA interference

The small interfering RNAs (siRNAs) for hnRNPK and MICAL2 were designed by GeneKai Company, and the sequences are shown in Supplementary Table 3. Cells were cultivated to 70–80% confluence at transfection. Diluting LipofectamineTM 3000 transfection reagent (Thermo Scientific) in serum-free with 3:100 ratio. Diluting DNA (5 μg/μl) in Opti-MEM, then add P3000TM reagent. Add diluted DNA to each diluted LipofectamineTM 3000 transfection reagent with 1:1 ratio and incubate for 15 min at room temperature. The final concentration of plasmid DNA transfection was 0.5 μg/μl. After 8 h, removing the media containing the transfection reagent and adding fresh 10% FBS-RPMI 1640, cells were incubated for 48–72 h.

### RNA pull-down

Plasmid DNA for seRNA LOC100506178 was designed by Sangon Biotech (Shanghai, China), PCR was used to amplify DNA template, and PCR primer sequences are shown in Supplementary Table 4. PCR products served as the template for transcription in vitro, then biotin-labeled LOC100506178 truncation probes were transcribed with a Pierce RNA 3' end desthiobiotinylation kit (Thermo Scientific). After incubation of LOC100506178 probes with streptavidin-coated magnetic beads, the complex incubated with the total cell lysate (1 × 10^8^ cells) to capture proteins (Pierce^TM^ Magnetic RNA-protein pull-down kit, Thermo Scientific) [21]. The obtained proteins were eluted and verified by mass spectrometry (MS) for three replicates and were ranked by protein-score. Ultimately, the specific protein was detected with Western blotting.

### ChIP

ChIP was performed as described previously [7]. 1 × 10^7^ cells were cross-linked with 1% paraformaldehyde for 10 min and suspended in 0.125 M glycine solution for 5 min at room. Then, cell lysates were sonicated, and soluble chromatin was incubated with anti-hnRNPK (ab39975, Abcam) or anti-H3K27ac (ab177178, Abcam) antibody to precipitate immunocomplexes and obtain DNA. ChIP DNA was sequenced on Illumina HiSeq2000 or identified by qPCR. ROSE (Rank Ordering of SE) was utilized to defined SEs and TEs based on the density of H3K27ac signal [5, 22]. In detail, ROSE was run without promoter exclusion, closely spaced MACS peaks (except those within 2 kb of TSS) within 12.5 kb were merged, followed by the measurement of input and H3K27ac signals; these merged peaks were ranked by H3K27ac signal, then, an inflection point was obtained to establish the cutoff for distinguishing SEs and TEs. Both SEs and TEs were assigned to the Ensemble genes.

### RNA-Seq

RNA-Seq was performed as described previously [23, 24]. Briefly, total RNA from 3 × 10^6^ cells were extracted and purified, and the purified RNA products were reverse transcribed into cDNA. cDNA was amplified to construct library. HTseq count tool was applied to calculate the read counts mapped to the genome [25]. Differentially expressed genes were filtered using DESeq2 algorithm based on the raw read counts [26, 27]. The criterion for distinguishing the differential genes includes: |log2 (Fold Change)| *>* 1, and false discovery rate (FDR) < 0.05. FPKM (fragments per kilobase million reads) was used to normalize the read depth and gene length, and the pheatmap R-package was used to draw the heatmaps based on the FPKM normalized read counts in the Paired-End RNA-Seq [28].

### Chromatin isolation by RNA purification

ChIRP (chromatin isolation by RNA purification) was performed as described previously [23]. Biotin-labeled antisense oligos were designed for seRNA LOC100506178, and the probe sequences are described in Supplementary Table 5. 2 × 10^8^ cells were cross-linked in 1% glutaraldehyde for 10 min at room and suspended in 0.125 M glycine solution for 5 min at room, then lysates were sonicated. After incubation of biotin-labeled probes with streptavidin-coated magnetic beads, the probes hybridized with the lysates to target lncRNA-DNA complex. After chromatin complex purification, lncRNA-bound DNA was eluted. qPCR or Illumina HiSeq2000 was performed to confirm DNA retrieval.

### Metastatic models

Nude mouse lung and abdominal cavity metastatic models were generated as described previously [29]. Experiments involving animals were approved by the Ethical Approval Form from Institution Animal Care and Use Committees of The Affiliated Cancer Hospital of Xiangya School of Medicine, Central South University. Nude female mice aged 4–5 weeks were obtained from Hunan SJA Laboratory Animal Co., Ltd. (Changsha, China). 100 μl cell suspension was injected into the lateral tail vein and abdominal cavity of mice (3 × 10^6^ cells/animal). At 4–6 weeks, the luminescence intensity was observed using animal imaging technology, and metastatic nodules were counted.

### Fluorescence in situ hybridization

FISH (fluorescence in situ hybridization) was performed as described previously using a FISH kit (Wellbio, Shanghai, China) [29]. The digoxin-labeled LOC100506178 probe (5-cy3-AACACAACGUAGGGUUCCAUUAUGGCGAUGAGAC-3) was designed by Sangon Biotech. 1 × 10^5^ cells were plated on 6-well plates. Slides were fixed with 4% paraformaldehyde for 30 min and penetrated with proteinase K for 30 min. After blocking for 30 min at 37 °C, the slides were hybridized with the diluted probes in the dark at 37 °C overnight. Finally, the slides stained with DAPI and sealed with 90% glycerin.

### RNA FISH and immunofluorescence microscopy

RNA FISH was carried out as previously described [29]. 1 × 10^5^ cells were plated on 6-well plates. Slides were fixed with 4% paraformaldehyde for 30 min and penetrated with proteinase K for 30 min. After being blocked for 30 min at 37 °C, the slides were hybridized with the diluted probes in the dark at 37 °C overnight. For co-localization studies, cells were co-stained with rabbit anti-hnRNPK (ab52600, Abcam) and CoraLite488-conjugated Affinipure Goat Anti-Rabbit IgG (H + L) (SA00013-2, Proteintech). The nuclei were counterstained with DAPI, then sealed with 90% glycerin.

### Immunohistochemistry

IHC (immunohistochemistry) was performed using an SP Rabbit & Mouse HRP kit (CWBIO, Beijing, China). The tissue slices were baked at 6 °C for 12 h, then were placed into xylene I, II, 100%, 95%, 85%, and 75% alcohol for 5 min at each stage. Next, the slices were placed into sodium citrate solution and boiled for 10 min to repair antigens. After blocked with 3% hydrogen peroxide, the slices were incubated with the primary antibody, horseradish peroxidase-conjugated secondary antibody, dyed with DAB, redyed with hematoxylin. Ultimately, the slices were placed into 75%, 85%, 95%, 100% alcohol, xylene II and I for 5 min at each stage, and imaged under a microscope.

### 5' and 3' rapid amplification of cDNA Ends

5' and 3' RACE (rapid amplification of cDNA ends) was conducted using a GeneRacerTM Kit (Thermo Scientific) as described previously [30]. The primers for 5', 3' and intermediate fragments were designed by Sangon Biotech and are shown in Supplementary Table 6. Briefly, total RNA was extracted using a TRIzol^®^ Plus RNA purification kit (Thermo Scientific) and was inverse transcribed into cDNA. PCR amplification was performed using Platinum^®^ PCR SuperMix High Fidelity enzyme (Thermo Scientific). PCR products were isolated by 1.5% agarose gel electrophoresis and purified. The target fragment was cloned into a pGM-T PCR cloning vector, then transformed into DH5α cells for sequencing.

### Confocal laser scanning microscopy

CLSM (confocal laser scanning microscopy) was used to observe the location of seRNA LOC100506178 as described previously [31]. A Zeiss LSM 510 confocal microscope (Carl Zeiss) was set at the 560 nm excitation/emission wavelength for Cy3. CLSM images were acquired by Zen V.2 software (Carl Zeiss).

### Global run-on sequencing

GRO-Seq (global run-on sequencing) was performed as described previously [7]. Cells (1 × 10^8^) were collected, and the nuclei were extracted to perform nuclear run-on reactions and collect RNA products. After purification, RNA products were treated with Antarctic phosphatase, DNase, polynucleotide kinase, and modified with a poly-A tail. After cDNA synthesis and PCR amplification, PCR products were isolated with 10% PAGE. DNA libraries were clustered using Illumina and sequenced with oligo NTI202. The optimal size range of GRO-Seq libraries was 200–250 bp, and the reads were mapped to human genome hg38.

### Cytoplasmic and nuclear RNA isolation and detection

Cytoplasmic and nuclear RNA were isolated and purified according to the cytoplasmic & nuclear RNA purification kit (NGB-21000, Norgen Biotek). After lysing cells with lysis buffer on ice for 5 min, the cytoplasmic and nuclear RNA were isolated through centrifugation. After being washed and eluted, the obtained cytoplasmic and nuclear RNA were reverse transcribed into cDNA, and qPCR was performed according to the RT-qPCR.

### RNA binding protein immunoprecipitation

RIP (RNA binding protein immunoprecipitation) assay was performed with EZ Magna RIP kit (Millipore, MA, USA) as described previously [32]. Briefly, 1 × 10^8^ cells was lysed with the RIP lysis buffer containing protease inhibitor cocktail and RNase inhibitor. Then, protein A/G magnetic beads were washed with RIP wash buffer and incubated with 10 μg of purified antibodies for 30 min at room temperature. Further, the protein A/G magnetic beads-antibodies complexes were incubated with the cell lysate overnight at 4 °C. Finally, RNA was purified using proteinase K buffer, and the purified RNA products were detected with RT-qPCR. The IgG antibody was used as negative control.

### Luciferase activity reporter assay

The wild-type (wt) promoter of MICAL2 was amplified from genomic DNA and inserted into pGL3 firefly luciferase reporter plasmid (Promega, Madison, USA) to obtain MICAL2 promoter-wt. Site-direct mutagenesis kit (Takara) was used to generate mutant (mt) MICAL2 luciferase vector version. HEK293 cells were co-transfected with MICAL2 promoter-wt or MICAL2 promoter-mt and hnRNPK-overexpression (OE) or hnRNPK-NC using Lipofectamine 2000. 48 h after co-transfection, relative luciferase activity was measured using dual‐luciferase activity reporter assay system (Promega).

### Patient specimens

Ten pairs of NPC lymph node metastatic and primary tissues from ten patients were collected from Xiangya Hospital, Central South University, between December 1st, 2014, and May 31st, 2020. The use of patient materials was approved by the local ethics committee of Xiangya Hospital, Central South University. The patients’ clinical characteristics are shown in Supplementary Table 7.

### Statistical analysis

Data are presented as mean ± S.E.M. of three independent experiments and were statistically analyzed by two-tailed unpaired *t*-test. Differences between groups were considered *P < 0.05; **P < 0.01; ***P < 0.001; ****P < 0.0001; ns, *P* > 0.05. All statistical data were calculated using GraphPad Prism 7.0 software.

## Results

### The identification of metastasis-specific SEs

**Fig. 1:**
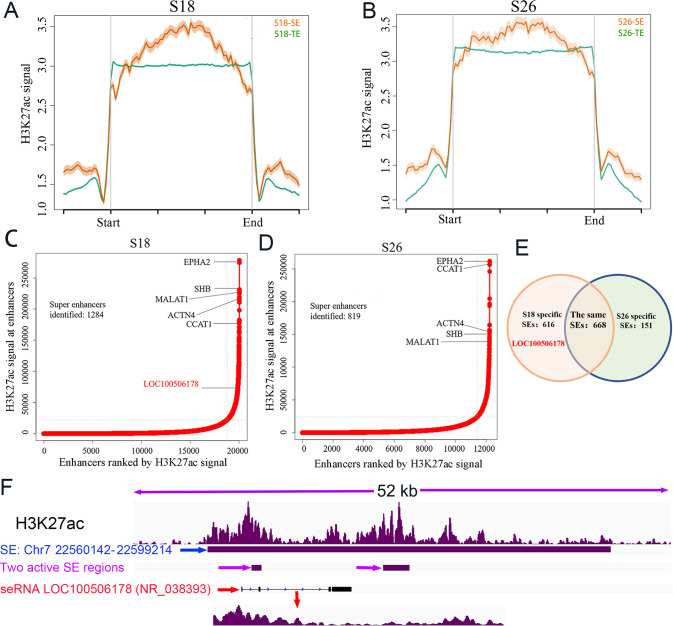
Comparative analysis of SEs in S18 and S26 cells. **A**, **B** H3K27ac signal in SE and TE regions in S18 and S26 cells, respectively; ChIP-seq was performed three replicates. **C**, **D** The numbers of identified SEs and the H3K27ac signal at enhancers in both S18 and S26 cells; the SE regulating LOC100506178 existed only in S18 cells in this study. **E** The numbers of identical and distinct SEs in S18 and S26 cells were analyzed. **F** The locations of the S18-specific SE, seRNA LOC100506178, and the active SE regions were visualized by IGV, and the active SE regions transcribed into seRNA LOC100506178 (NR_038393). TE typical enhancer; SE super enhancer. ROSE (Rank Ordering of SE) was utilized to define SEs and TEs.

SEs have powerful regulatory functions on their target genes, and oncogenic SEs further trigger cancer cells invasion and metastasis [33]. Specific SEs might enhance the metastasis and invasion of NPC cells. Hence, ChIP-Seq was used to screen distinct SEs from high-metastatic S18 and low-metastatic S26 cells based on the H3K27ac signal intensity (Supplementary Data 1, Data 2). The results showed that H3K27ac signals in SE regions were dramatically higher than those in TE regions in S18 and S26 cells (Fig. 1A, B). The numbers of SEs in S18 and S26 cells were 1284 and 819, respectively, and there were 661 identical SEs (Fig. 1C–E). Thus, S18 cells emitted higher H3K27ac signals and had more SEs than S26 cells. Most importantly, the active H3K27ac signal for LOC100506178 existed only in S18 cells but not in S26 cells (Fig. 1C, D), indicating that the SE regulating LOC100506178 was a S18-specific SE. IGV visually indicated that seLOC100506178 was located at chr7:22560142-22599214 (Fig. 1F).

### A specific SE transcribed into seRNA LOC100506178

**Fig. 2:**
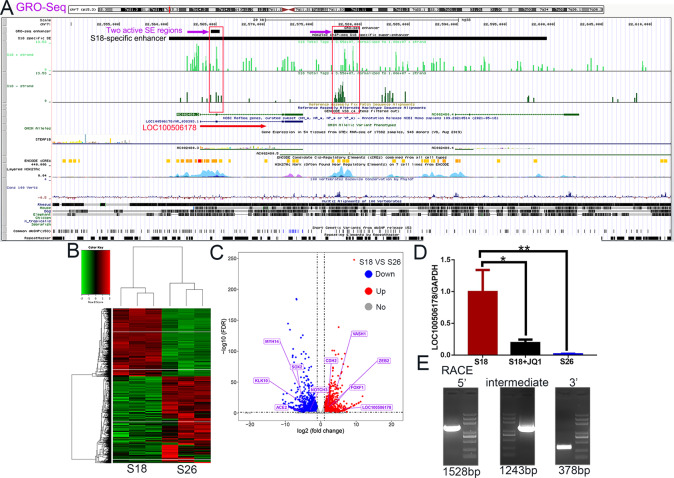
A specific SE transcribed into seRNA LOC100506178 and its sequences. **A** The locations of the S18-specific SE, seRNA LOC100506178 and active SE regions were visualized using UCSC, and the red squares represented the transcription-active SE regions that transcribed into seRNA LOC100506178 (NR_038393). **B** Heatmap showed the normalized expression of significantly deregulated genes between S18 and S26 cells (*n* = 3). **C** Log2 (fold change) versus log (FDR) of the differential genes expression between S18 and S26 cells. **D** The transcription of the seRNA LOC100506178 in S18, JQ1-treated S18, and S26 cells was detected with RT-qPCR. **E** Cloning products of seRNA LOC100506178 were identified using RACE (left to right: 5', intermediate fragment, and 3'). **P < 0.01; *P < 0.05.

To investigate the biological function of seLOC100506178 in NPC, GRO-Seq was used to identify whether this SE produced a transcript, and the results showed that the specific SE held two transcription-active regions and that the SE transcribed into seRNA LOC100506178 (NR_038393) on the basis of these transcription-active regions (Figs. 1F, 2A; Supplementary Data 3). Then, RNA-Seq was used to confirm the differences in transcript levels, and the results indicated 1754 upregulated and 2229 downregulated genes in S18 cells compared with S26 cells (Supplementary Data 4, Fig. 2B, C). Notably, seRNA LOC100506178 was significantly increased in S18 cells (Fig. 2C). RT-qPCR also showed that seRNA LOC100506178 expression was 53.6-fold higher in S18 cells (Fig. 2D). Furthermore, 3' and 5' RACE verified seRNA LOC100506178 as a novel transcript consisting of 2645 bp (Refseq database; ENSG00000232759) (Fig. 2E; Supplementary Data 5).

### seRNA LOC100506178 was overexpressed in high-metastatic NPC cells

**Fig. 3:**
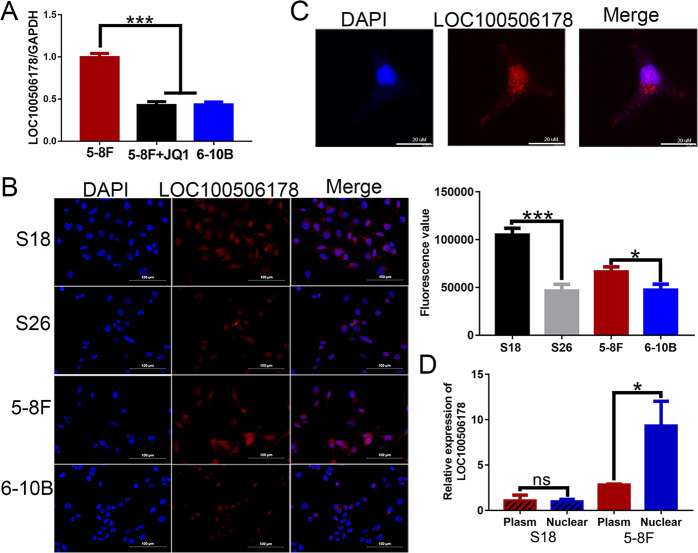
The expression of seRNA LOC100506178 in NPC cells. **A** The transcription of seRNA LOC100506178 was detected with RT-qPCR in 5–8 F, JQ1-treated 5–8 F, and 6-10B cells. **B** RNA FISH was carried out to verify the location and relative levels of seRNA LOC100506178 in S18, S26, 5–8 F, and 6-10B cells; the fluorescence intensity was presented as the means ± S.E.M of three independent experiments. **C** The location of seRNA LOC100506178 was detected using CLSM. **D** The cytoplasmic and nuclear RNA in S18 and 5–8 F cells was isolated and detected. ***P < 0.001; *P < 0.05; ns, *P* > 0.05.

To verify whether seRNA LOC100506178 was also highly expressed in other high-metastatic NPC cells, LOC100506178 expression was detected in high-metastatic 5–8 F and low-metastatic 6-10B cells. The results demonstrated that LOC100506178 was highly expressed in 5–8 F cells and lowly expressed in 6-10B cells (Fig. 3A). Correspondingly, RNA FISH showed that LOC100506178 was dramatically overexpressed in S18 and 5–8 F cells (Fig. 3B). CLSM showed that it simultaneously accumulated in both the nucleus and cytoplasm (Fig. 3C). The cytoplasmic and nuclear RNA in S18 and 5–8 F cells were isolated and detected, indicating that the expression level of cytoplasmic and nuclear LOC100506178 displayed no difference between nuclear and plasm in S18 cells, but the level of nuclear RNA was significantly higher than cytoplasmic RNA in 5–8 F cells (Fig. 3D). These data implied that seRNA LOC100506178 might facilitate NPC cells metastasis.

Bromodomain-containing protein 4 (BRD4) is an important SE constituent that binds to H3K27ac and enables transcript elongation [34]. The small-molecule BRD4 inhibitor JQ1 can block the interaction of BRD4 with H3K27ac to suppress the activity of SE and SE-triggered seRNA transcription [35, 36]. In this study, the level of LOC100506178 was remarkably reduced after JQ1 treatment (Figs. 2E and 3A). LOC100506178 transcription was sensitive to BRD4 perturbation, suggesting that SE was essential for seRNA LOC100506178 transcription.

### seRNA LOC100506178 promoted the invasion and metastasis of NPC cells in vitro

**Fig. 4:**
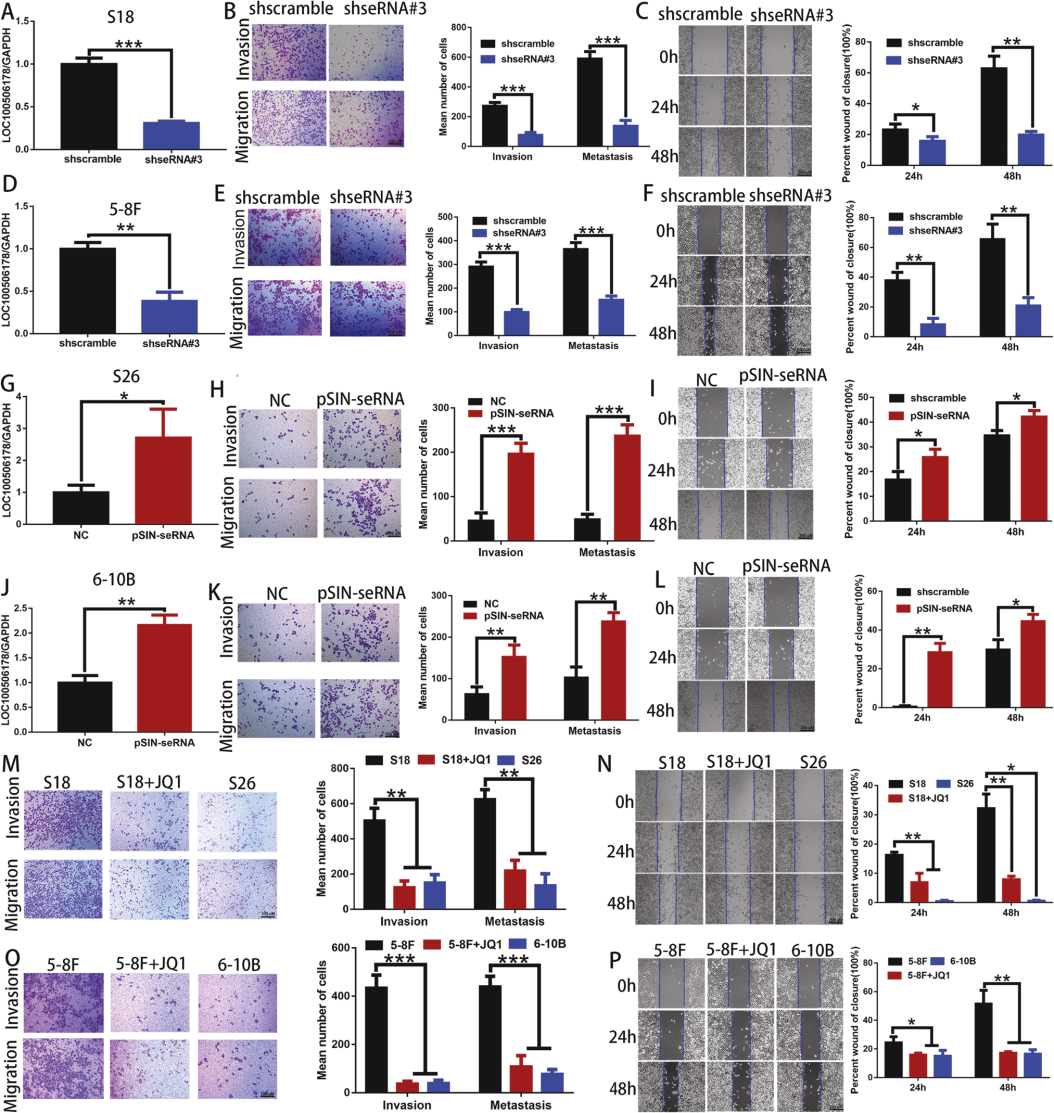
seRNA LOC100506178 promoted NPC progression in vitro. **A**, **D** RT-qPCR validated that the knockdown efficiencies of seRNA LOC100506178 in S18 and 5–8 F cells were 68.9% and 61.5%, respectively. **B**, **E** Transwell migration and invasion assays were used to detect the migratory and invasive capabilities of S18- and 5-8F-shseRNA#3 cells in the absence and presence of Matrigel, respectively; the number of migrated and invaded cells were presented as the means ± S.E.M. **C**, **F** A wound-healing assay was used to detect the migratory capabilities of S18- and 5-8F-shseRNA#3 cells, the percent wound of closure was presented as the means ± S.E.M. **G**, **J** RT-qPCR validated that the overexpression efficiencies of seRNA LOC100506178 in S26 and 6-10B cells were 2.72- and 2.17-fold, respectively. **H**, **K** Transwell migration and invasion assays were used to detect the migratory and invasive capabilities of S26- and 6-10B-pSIN-seRNA cells in the absence and presence of Matrigel, respectively, the migrated and invaded cells were presented as the means ± S.E.M. **I**, **L** A wound-healing assay was used to detect the migratory capabilities of S26- and 6-10B-pSIN-seRNA cells, the percent wound of closure was presented as the means ± S.E.M. **M**, **O** Transwell migration and invasion assays were used to detect the migratory and invasive capabilities of S18 and 5–8 F cells after treatment with JQ1 in the absence and presence of Matrigel, respectively; the number of migrated and invaded cells were presented as the means ± S.E.M. **N**, **P** A wound-healing assay was used to detect the migratory capabilities of S18 and 5–8 F cells after treatment with JQ1, the percent wound of closure was presented as the means ± S.E.M. NC negative control, ***P < 0.001; **P < 0.01; *P < 0.05.

To confirm the role of seRNA LOC100506178 in NPC metastasis, shseRNAs were designed to block LOC100506178 transcription (Fig. 4A, D). S18 and 5–8 F cells were stably transfected with shseRNA#3 LOC100506178 to observe NPC cells metastasis; the migration and invasion of S18 and 5–8 F cells were obviously impaired (Fig. 4B, C, E, F). Consistently, JQ1 treatment reduced the migratory and invasive properties of S18 and 5–8 F cells by interrupting SE activation and downregulating LOC100506178 (Fig. 4M–P). In addition, S26 and 6-10B cells were stably transfected with pSIN-seRNA LOC100506178 (Fig. 4G, J). The migratory and invasive capabilities of S26 and 6-10B cells were significantly increased (Fig. 4H, I, K, L). Collectively, these results highlighted that seRNA LOC100506178 truly strengthened the invasive and metastatic properties of NPC cells in vitro.

### seRNA LOC100506178 specifically interacted with hnRNPK and regulated the expression of hnRNPK

**Fig. 5:**
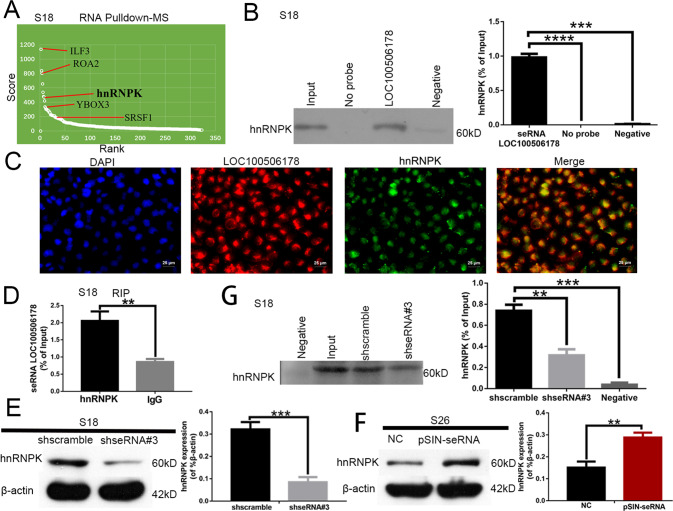
seRNA LOC100506178 specifically regulated hnRNPK. **A** Biotin-labeled RNA pull-down-MS assay was used to detect the interaction of seRNA LOC100506178 with hnRNPK in S18 cells; antisense RNA for LOC100506178 was used as a negative control. **B** The interaction of seRNA LOC100506178 with hnRNPK was confirmed by RNA pull-down western blotting, and the enrichment level of hnRNPK was analyzed; antisense RNA for LOC100506178 was used as a negative control, no probe served as a blank control. **C** Co-localization between LOC100506178 and hnRNPK was verified with RNA FISH and immunofluorescence microscopy. **D** hnRNPK RIP and RT-qPCR assays were performed to verify the enrichment level of hnRNPK with LOC100506178 in S18 cells; rabbit normal IgG antibody was used as a negative control. **E**, **F** The levels of hnRNPK in S18-shseRNA#3 and S26-pSIN-seRNA cells was detected by Western blotting, and the abundances were expressed as the mean ± S.E.M. of three independent experiments; β-actin served as a loading control. **G** The enrichment levels of hnRNPK in S18-shseRNA#3 and shscramble cells were detected by RNA pull-down western blotting, and the abundances were analyzed; antisense RNA for LOC100506178 was used as a negative control. NC negative control, ****P < 0.0001; ***P < 0.001; **P < 0.01.

Generally, lncRNAs can bind with RNA or proteins to modulate downstream genes [13, 14]. To further clarify the mechanisms of seRNA LOC100506178 regulating NPC metastasis, RNA pull-down-MS was used to explore LOC100506178-binding proteins: ILF3, ROA2, hnRNPK, SRSF1, and YBOX3 (Fig. 5A; Supplementary Data 6). HnRNPK (heterogeneous nuclear ribonucleoprotein K) had a high a higher protein-score and showed a strong binding with LOC100506178. Most importantly, hnRNPK is a master transcription factor (TF) that exacerbates EMT, and EMT contributes to the increased mobility and invasion of NPC cells [18]. Furthermore, the RIP data showed that the enrichment level of LOC100506178 with hnRNPK in S18 cells was more than two-fold (Fig. 5D). Thus, hnRNPK was the focus of this study. Consistently, RNA pull-down-Western blotting and co-localization between LOC100506178 and hnRNPK showed that hnRNPK interacted with LOC100506178 (Fig. 5B, C).

Next, we detected the expression of hnRNPK in S26-pSIN-seRNA and S18-shseRNA#3 stable cells to observe the effect of LOC100506178 on hnRNPK expression. The results supported that knockdown of LOC100506178 downregulated hnRNPK and overexpression of LOC100506178 upregulated hnRNPK (Fig. 5E, F). Also, there was a reduced pull-down of hnRNPK in the S18-shseRNA#3 group (Fig. 5G). Therefore, seRNA LOC100506178 and hnRNPK might form a ribonucleoprotein (RNP) complex, and LOC100506178 further positively regulated the level of hnRNPK to mediate EMT in NPC metastasis.

### seRNA LOC100506178/hnRNPK complex regulated MICAL2 expression and EMT

**Fig. 6:**
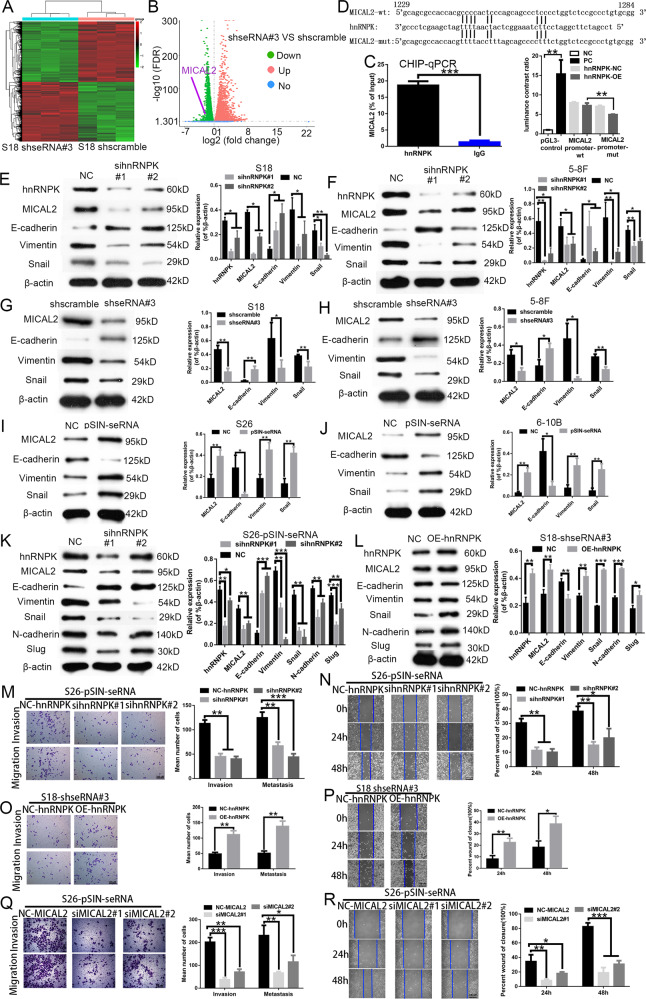
seRNA LOC100506178 regulated MICAL2 and EMT by upregulating hnRNPK. **A** Heatmap showed the normalized expression of significantly deregulated genes between S18-shseRNA#3 and shscramble cells (*n* = 3). **B** Log2 (fold change) versus log (FDR) of the differential genes expression between S18-shseRNA#3 and shscramble cells. **C** ChIP-qPCR was used to verify the enrichment level of hnRNPK with the promoter of MICAL2 in S18 cells; rabbit normal IgG antibody was used as a negative control. **D** Schematic representation of the hnRNPK target sequence within the promoter of MICAL2; the luciferase activities of MICAL2 promoter-wt and MICAL2 promoter-mut was measured after co-transfection with hnRNPK‐OE or hnRNPK‐NC; relative luciferase activity was expressed as the mean ± S.E.M. of three independent experiments. **E**, **F** The expression of hnRNPK, MICAL2, E-cadherin, Vimentin and Snail was determined by Western blotting after hnRNPK knockdown in S18 and 5–8 F cells, the abundances were expressed as the mean ± S.E.M. of three independent experiments. **G**, **H** The expression of MICAL2, E-cadherin, Vimentin, and Snail was determined by Western blotting after LOC100506178 knockdown in S18 and 5–8 F cells, the abundances were expressed as the mean ± S.E.M of three independent experiments. **I**, **J** The expression of MICAL2, E-cadherin, Vimentin, and Snail was detected by Western blotting after overexpression of LOC100506178 in S26 and 6-10B cells, the abundances were expressed as the mean ± S.E.M. of three independent experiments. **K**, **L** The expression of hnRNPK, MICAL2, E-cadherin, Vimentin, Snail, N-cadherin, and Slug was detected in S26-pSIN-seRNA and S18-shseRNA#3 cells after knockdown of hnRNPK and overexpression of hnRNPK, the abundances were expressed as the mean ± S.E.M. of three independent experiments; β-actin served as a loading control. **M**, **O**, **Q** Transwell migration and invasion assays were used to detect the migratory and invasive capabilities of S26-pSIN-seRNA and S18-shseRNA#3 cells after knockdown of hnRNPK or MICAL2 and overexpression of hnRNPK in the absence and presence of Matrigel, respectively, the number of migrated and invaded cells were presented as the means ± S.E.M. **N**, **P**, **R** A wound-healing assay was used to detect the migratory capabilities of S26-pSIN-seRNA and S18-shseRNA#3 cells after knockdown of hnRNPK or MICAL2 and overexpression of hnRNPK, the percent wound of closure was presented as the means ± S.E.M. NC negative control, PC positive control, wt wild-type, mut mutant, ***P < 0.001; **P < 0.01; *P < 0.05.

To explore the downstream genes of the seRNA LOC100506178/hnRNPK complex, we performed RNA-Seq to screen different genes in S18-shseRNA#3- and shscramble-transfected cells. The data showed that LOC100506178 knockdown caused 5340 upregulated and 5257 downregulated genes (Fig. 6A, B; Supplementary Data 7). MICAL2 (molecules interacting with CasL 2) was one of the downregulated genes (Fig. 6B), and it has been found to exacerbate cells invasion and migration in various cancers [37]. Significantly, the ChIP-Seq and ChIP-qPCR with anti-hnRNPK showed that hnRNPK directly bound to the promoter of MICAL2 (Supplementary Data 9), and the enrichment of the MICAL2 promoter was 13.7-fold high during transcription (Fig. 6C). Also, the results of the luciferase reporter assay disclosed that hnRNPK-OE introduction increased luciferase activity of cells transfected with MICAL2 promoter-wt but not MICAL2 promoter-mt (Fig. 6D). Therefore, MICAL2 was the focus in seRNA LOC100506178/hnRNPK-mediated NPC metastasis. Nevertheless, the ChIRP-Seq and ChIRP-qPCR showed that LOC100506178 did not combine with MICAL2 (Supplementary Data 8). seRNA LOC100506178 might indirectly affect MICAL2 expression by upregulating hnRNPK.

Consistently, we observed the reduced MICAL2 expression after LOC100506178 knockdown and the increased MICAL2 expression after overexpression of LOC100506178 (Fig. 6G–J). To explore the role of hnRNPK in seRNA LOC100506178-mediated MICAL2 expression, we constructed siRNA hnRNPK#1 and #2 in S18 and 5–8 F cells and found the decreased MICAL2 expression after knockdown of hnRNPK (Fig. 6E, F). Furthermore, knockdown of hnRNPK in S26-pSIN-seRNA cells downregulated MICAL2 (Fig. 6K), overexpression of hnRNPK in S18-shseRNA#3 cells upregulated MICAL2 (Fig. 6L). The combined analysis strongly indicated that hnRNPK upregulated MICAL2 and that seRNA LOC100506178 indirectly enhanced MICAL2 expression by increasing hnRNPK expression.

E-cadherin, Vimentin, and Snail are notable hallmarks of EMT and are also regulated by MICAL2 [38]. Therefore, we detected the levels of MICAL2, E-cadherin, Vimentin, and Snail after seRNA LOC100506178 knockdown or overexpression. In shseRNA#3-transfected cells, we found the upregulation of E-cadherin and the downregulation of Vimentin, Snail and MICAL2 (Fig. 6G, H). In pSIN-seRNA cells, overexpressed LOC100506178 attenuated E-cadherin and promoted Vimentin, Snail and MICAL2 expression (Fig. 6I, J). Additionally, hnRNPK knockdown in S18 and 5–8 F cells contributed to an increase in E-cadherin and reductions in Vimentin, Snail, and MICAL2 (Fig. 6E, F). In line with these findings, in S26-pSIN-seRNA cells, knockdown of hnRNPK downregulated MICAL2, Vimentin, Snail, N-cadherin, and Slug, and upregulated E-cadherin (Fig. 6K). In S18-shseRNA#3 cells, overexpression of hnRNPK upregulated MICAL2, Vimentin, Snail, N-cadherin, and Slug, downregulated E-cadherin (Fig. 6L).

Meanwhile, we observed that knockdown of hnRNPK or MICAL2 in S26-pSIN-seRNA cells inhibited the metastasis and invasion of NPC cells (Fig. 6M, N, Q, R), and overexpression of hnRNPK in S18-shseRNA#3 cells promoted the metastasis and invasion of NPC cells (Fig. 6O, P). Thereby, both hnRNPK and MICAL2 promoted the metastasis and invasion of NPC cells. Taken together, seRNA LOC100506178 indirectly modulated MICAL2 and EMT genes expression through upregulating hnRNPK, further enhancing the EMT process of NPC cells.

### seRNA LOC100506178 promoted NPC progression in vivo

**Fig. 7:**
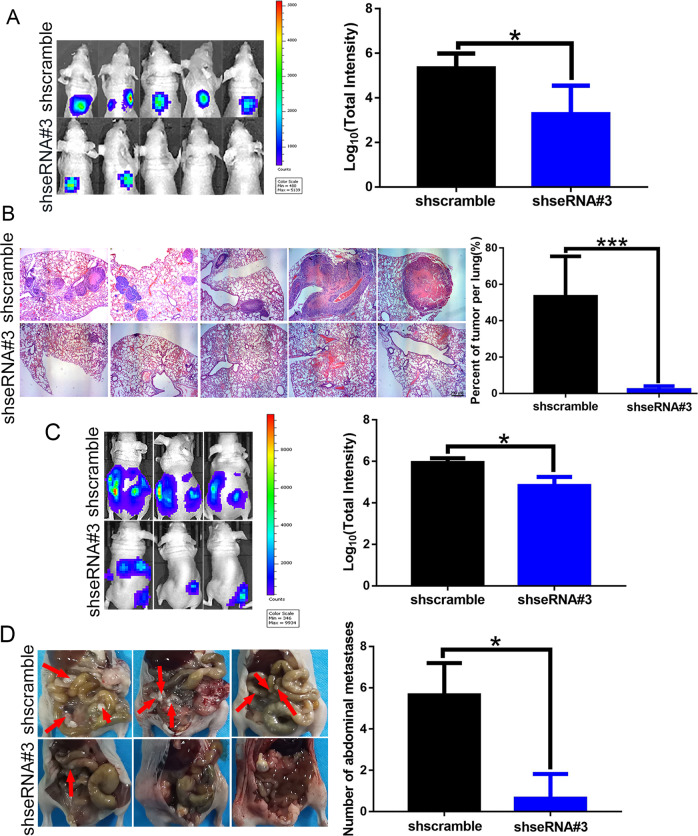
seRNA LOC100506178 promoted the invasion and metastasis of NPC cells in vivo. **A** A total of ten nude mice were randomly allocated to the S18-shseRNA#3 group (*n* = 5) or the shscramble group (*n* = 5). Cells were injected into the tail vein of nude mice, and the luminescence intensity of lung metastases was analyzed in vivo using an in vivo small animal imaging system. **B** Hematoxylin-eosin (HE) staining was used to detect the percent of tumor metastases per lung. **C** A total of six nude mice were randomly divided into the S18-shseRNA#3 group (*n* = 3) and the shscramble group (*n* = 3). Cells were injected into the abdominal cavity of nude mice, and the luminescence intensity was analyzed. **D** The number of abdominal cavity metastases was analyzed. ***P < 0.001; *P < 0.05.

Next, we tested the function of seRNA LOC100506178 in strengthening NPC cells invasion and metastasis in vivo. S18-shseRNA#3 and shscramble cells were injected into the lateral tail vein of nude mice, and the luminescence intensity of lung metastases and the percent of tumor metastases per lung were decreased in the LOC100506178-knockdown group at 41 days (Fig. 7A, B). Similarly, we injected S18-shseRNA#3 and shscramble cells into the mouse abdominal cavity and observed weaker luminescence intensity and fewer tumor metastases in the LOC100506178-knockdown group at 32 days (Fig. 7C, D). Thus, seRNA LOC100506178 triggered EMT and strengthened the metastatic and invasive abilities of NPC cells.

### Clinical significance of seRNA LOC100506178 in NPC patients

**Fig. 8:**
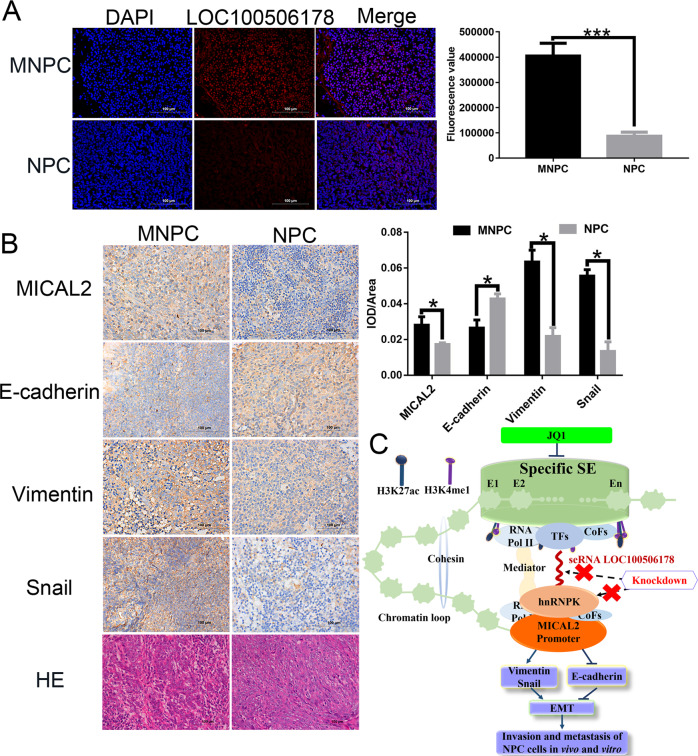
The clinical significance of seRNA LOC100506178 in human NPC patients. **A** FISH detected seRNA LOC100506178 expression in NPC lymph node metastatic tissues (*n* = 10) and NPC primary tissues (*n* = 10), the fluorescence intensity was presented as the means ± S.E.M of three independent experiments. **B** The expression of MICAL2, E-cadherin, Vimentin, and Snail in NPC lymph node metastatic tissues and NPC primary tissues were measured by IHC staining; the IOD/area values were counted in IHC-stained slides. **C** Specific SEs were enriched with clusters of enhancers and transcribed into seRNAs by binding with abundant TFs, CoFs, RNA Pol II, and H3K4me1 and H3K27ac. seRNAs exerted a powerful function in stabilizing the chromatin loop between the SE and the promoter in cooperation with the mediator and cohesin complex. In our research, seRNA LOC100506178 indirectly promoted MICAL2, Vimentin, and Snail expression and inhibits E-cadherin expression by specifically interacting with hnRNPK and upregulating hnRNPK, subsequently accelerating EMT and the invasion and metastasis of NPC cells in vivo and in vitro. Knockdown of seRNA LOC100506178 or hnRNPK markedly repressed MICAL2, Vimentin, and Snail expression, and increasing E-cadherin expression. Additionally, JQ1 suppressed seRNA transcription and blocked the invasion and metastasis of NPC cells by inhibiting the activation of SE. H3K4me1 histone H3 lysine 4 monomethylation, H3K27ac histone H3 lysine 27 acetylation, CoFs cofactors, TFs transcription factors, MNPC NPC lymph node metastasis tissues, NPC NPC primary tissues. ***P < 0.001; *P < 0.05.

Clinically, ten pairs of NPC samples were collected to detect the expression of seRNA LOC100506178 and downstream proteins. FISH showed that the positive rate of LOC100506178 was significantly higher in NPC lymph node metastatic tissues than that in primary tissues (Fig. 8A), suggesting that LOC100506178 positively modulated NPC metastasis. IHC showed that the expression of MICAL2, Vimentin, and Snail was increased, and the expression of E-cadherin was decreased in NPC lymph node metastatic tissues (Fig. 8B). These results indicated that seRNA LOC100506178 caused the downregulation of E-cadherin and the upregulation of MICAL2, Vimentin, and Snail by interacting with hnRNPK, consequently accelerating NPC metastasis (Fig. 8C).

## Discussion

SEs often drive oncogene expression to modulate cancer development [39]. SE inhibitors, such as the BRD4 inhibitor JQ1 [40], cyclin-dependent kinase (CDK)4/6 inhibitor LEE011 [41], and CDK8 inhibitor cortistatin A [42], have shown great potential for suppressing cancer progression by interfering with SE activity. In general, SEs are noncoding elements. Nevertheless, recent studies have indicated that oncogenic SEs can transcribe into seRNAs to mediate carcinogenesis [43], including polyadenylating and non-polyadenylating seRNAs [44]. In addition, seRNAs are categorized as *cis*-acting and *trans*-acting seRNAs according to their distinct functions [45]. In this research, S18-specific SE was identified with H3K27ac ChIP-Seq, and GRO-Seq confirmed that the specific SE transcribed into seRNA LOC100506178. The production of seRNA LOC100506178 relied on the transcription-active regions within the SE region, but the mechanisms of SE-producing seRNA remain to be investigated.

seRNAs govern oncogenic pathways to influence cells proliferation [16], autophagy [23], apoptosis [46], EMT [15], therapy resistance [47], extracellular matrix (ECM) remodeling [48] and angiogenesis [49]. EMT is critical for cells migration and invasion, which leads to high mortality and poor survival of NPC patients [50]. Because of the remarkably increased seRNA LOC100506178 expression in S18 and 5–8 F cells and NPC lymph node metastatic tissues, we selectively focused on the relationship between LOC100506178 and the properties of invasion and metastasis. Functionally, we observed that LOC100506178 knockdown prohibited the invasion and metastasis of NPC cells both in vitro and in vivo and that overexpressed LOC100506178 accelerated the invasion and metastasis of NPC cells. Mechanistically, seRNA LOC100506178 interacted with abundant hnRNPK and significantly enhanced hnRNPK expression. HnRNPK is highly expressed in numerous cancer tissues and can promote EMT [51–53]. In our study, knockdown of hnRNPK attenuated the migration and invasion of NPC cells, and overexpression of hnRNPK enhances the malignancy of NPC cells. Therefore, the seRNA LOC100506178/hnRNPK complex might boost the invasion and metastasis of NPC cells.

MICAL2 is enriched in the nucleus in human cells [54] and is a microtubule-associated monooxygenase and a novel cancer-promoting factor involved in angiogenesis, cells proliferation, and deformation [54, 55]. Additionally, MICAL2 boosts EMT by regulating SRF (serum response factor)/MRTF-A (myocardin-related transcription factor A), EGF/EGFR/P38 and Wnt/β-catenin signaling [55, 56]. Moreover, the MICAL2-boosted EMT is characterized by the upregulation of Vimentin and β-catenin and the downregulation of E-cadherin [37]. In agreement with these findings, our results demonstrated that knockdown of LOC100506178 or hnRNPK markedly blocked MICAL2, Vimentin, and Snail expression and facilitated E-cadherin expression, overexpression of LOC100506178 or hnRNPK significantly downregulated E-cadherin and upregulated MICAL2, Vimentin, and Snail, and knockdown of MICAL2 significantly inhibited the metastasis and invasion of NPC cells. So, MICAL2 accelerated the metastasis and invasion of NPC cells that was characterized with the upregulation of Vimentin and Snail and the downregulation of E-cadherin. HnRNPK occupied the promoter of MICAL2, but LOC100506178 did not bind with MICAL2, demonstrating that seRNA LOC100506178 recruited local hnRNPK to indirectly modulate the expression of MICAL2 and EMT genes, subsequently boosting EMT in NPC metastasis. Taken together, these findings showed that the SE-induced seRNA LOC100506178/hnRNPK/MICAL2/EMT signaling aggravates the invasion and mobility of NPC cells.

In the clinic, overexpressed seRNA LOC100506178 was detected in NPC lymph node metastatic tissues, suggesting that LOC100506178 might heighten cancer cells metastasis in NPC patients. These results highlighted the oncogenic clinical significance of LOC100506178 in NPC patients. However, more clinical specimens need to be collected to verify this conclusion, and the feasibility of seRNA LOC100506178 as a therapeutic target for NPC requires further investigation.

In summary, our study revealed that seRNA LOC100506178 indirectly enhances MICAL2, Vimentin, and Snail expression, and decreasing E-cadherin expression by specifically upregulating hnRNPK, which promotes EMT and NPC malignancy (Fig. 8C). Therefore, seRNA LOC100506178 in NPC patients has the potential to serve as a novel diagnostic or prognostic biomarker and a therapeutic target.

## Supplementary information

Supplementary Material 1

Supplementary Material 2

Supplementary Material 3

Supplementary Material 4

Supplementary Material 5

Reproducibility checklist of CDDIS-21-2671

## Data Availability

All data generated or analyzed during this study are included in this published article and its supplementary Data.
