# Supplementary material for: A novel oncogenic seRNA promotes nasopharyngeal carcinoma metastasis
Source: Cell Death Dis. 2022 Apr 23;13(4):401. doi: 10.1038/s41419-022-04846-1 (PMC9035166; doi:10.1038/s41419-022-04846-1)

Supplementary Table 1. The primer sequences for qPCR

Primers	Sequences（5′ to 3′）
LOC100506178-F	GGAGATGTGGCAACAGAGTGGATG
LOC100506178-R	CACTTCCTCCGCCTTCAGCTTG
GAPDH-F	TGACTTCAACAGCGACACCCA
GAPDH-R	CACCCTGTTGCTGTAGCCAAA
Luciferase-F	CTGGGACGAAGACGAACAC
Luciferase-R	GAAGACCTGCGACACCTG
hnRNPK-F	CTCCGTACAGACTACAATGCC
hnRNPK-R	ATGAATCAACAGCCTCAACTCG
MICAL2-F	AAGCCTGTAGGGCCCCG
MICAL2-R	TGGCTGGTGACTTCATCCTTC
PCNA-F	TAGCTCCAGCGGTGTAAACCT
PCNA-R	ACTTTCTCCTGGTTTGGTGCTT

Supplementary Table 2. Target sequences for shseRNA LOC100506178

shseRNA LOC100506178	Target sequences	Knockdown efficiency (%)	
		S18	5-8F
#1	TAGGAAGGAATCGGAAACCAA	31.2	22.3
#2	CTCCGATGTGCTAGGTACTAT	47.3	41.9
#3	ATGCCATATAACGTGCTACAT	68.9	61.5
#4	GACCAAACACTTCTACACATT	-122.7	-80.5
#5	TCCCAGTTCTCATAATCTCAT	-10.3	3.6
#6	GTGGTAACAAACTCAAGCTAA	-119.8	-66.7

Supplementary Table 3. siRNA sequences for hnRNPK and MICAL2

**Target genes**	**siRNAs**	**Sequences (5'-3')**
hnRNPK	#1	GCCUUUAGAAGGAUCCGAATT
	#2	GCAUUCUGCUUCAGAGCAATT
	#3	CCCAUGCCUCCAUCUAGAATT
MICAL2	#1	GCUCGACAAAGGUGUCAUCAUTT
	#2	GCUUGGCCAAAUCAUCCAUUUTT
	#3	CGACACGUGUUACUUCUGUAATT

Supplementary Table 4. The PCR primer sequences for plasmid DNA targeting LOC100506178

Primers	Sequences（5′ to 3′）
F	GTAAAACGACGGCCAGT
R	CAGGAAACAGCTATGAC

Supplementary Table 5. The probe sequences in ChIRP

**Probe number**	**Probe sequences (5'to3')**
2004105A-P1	ctgacagcaagtgggtaagg
2004105A-P2	gaacagcccattcttaaagc
2004105A-P3	actgtttggacacctgtatg
2004105A-P4	cctgaattcttctgtctgat
2004105A-P5	tttcatcacatgctgaccaa
2004105A-P6	ctcttccatgcatctttttg
2004105A-P7	tcatcacagctctgacaact
2004105A-P8	gattcaggggatttccgaag
2004105A-P9	gagcttgtcaccagaaatgc
2004105A-P10	ctcagagatctatgttaacc
2004105A-P11	aacgtagggttccattatgg
2004105A-P12	tgtgcgtgagtccatatttc
2004105A-P13	tctgcctgtggatatatgag
2004105A-P14	catttgcacaaccgtaagca
2004105A-P15	ctcccaacagtatgcttatg
2004105A-P16	gctaattctctggactttgt
2004105A-P17	attgacacaggcctcatttg
2004105A-P18	gaagccgtccagttttgaag
2004105A-P19	agccctcagatcgaactgac
2004105A-P20	tgaaggcacaggaaggatgc
2004105A-P21	ttcagatgttgaggtcctag
2004105A-P22	aagcgagcatgtgattcctt
2004105A-P23	caaggagatggagcttcaga

Supplementary Table 6. The primer sequences for 5' and 3' RACE

Position	Primers	Sequences（5′ to 3′）
Intermediate fragment	LOC100506178-F	CTGGAAGCCAGAAGTCCAA
	LOC100506178-R	CTGGACTTTGTACCCATCCAA
3'	LOC100506178-F1	CCACAGGCAGATGGAATTTACAAGTGGAA
	LOC100506178-F2	CGGTTGTGCAAATGCTTGCATTGGT
5'	LOC100506178-R1	CCTTGGGCACTTATCCCTTCCTTCTT
	LOC100506178-R2	CATCCACCCACCCTGAATTCAGATG

Supplementary Table 7. Clinical characteristics of NPC patients

Name	Gender	Age	Pathology number	Pathologic diagnosis	TNM	Status
Xx Xiao	Man	45	988339<br>986603	Undifferentiated nonkeratinized squamous cell carcinoma	T1N2M0	Alive
Xx Liu	Man	50	1309658①<br>1309658④	Undifferentiated nonkeratinized squamous cell carcinoma	-	Alive
Xx Zhou	Man	55	1003794<br>1309326④	Differentiated nonkeratinized squamous cell carcinoma	T3N2M0	Alive
Xx Li	Woman	44	1094343<br>1305882①	Differentiated nonkeratinized squamous cell carcinoma	T3N2M0	Alive
Xx Yang	Man	35	1294094<br>1293700③	Undifferentiated nonkeratinized squamous cell carcinoma	-	Alive
Xx Yin	Man	44	1194600<br>1299272①	Differentiated nonkeratinized squamous cell carcinoma	T2N2M0	Alive
Xx Xu	Man	47	1287635<br>1285901⑤	Differentiated nonkeratinized squamous cell carcinoma	T1N3M0	Alive
Xx Wu	Woman	41	1137614<br>1274102②	Undifferentiated nonkeratinized squamous cell carcinoma	T3N3M0	Alive
Xx Pang	Man	39	1182573<br>1185859	Undifferentiated nonkeratinized squamous cell carcinoma	T2N3M0	Alive
Xx Zhu	Man	27	1087300<br>1130991②	Undifferentiated nonkeratinized squamous cell carcinoma	T4N3M1	Alive

Figure 5E Actin

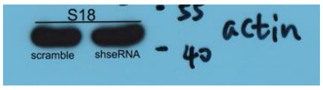

Figure 5E hnRNPK

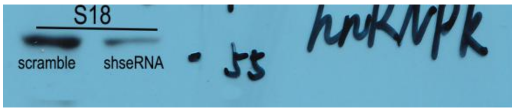

Figure 5F Actin

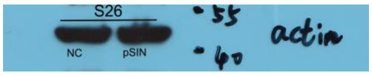

Figure 5F hnRNPK

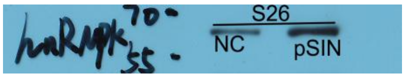

Figure 6E Actin

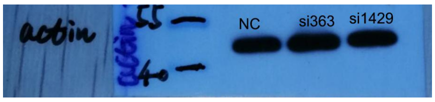

Figure 6E E-cadherin

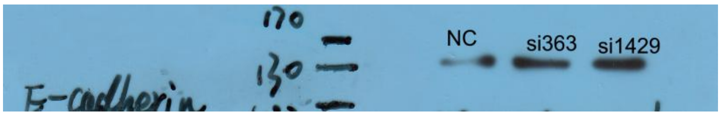

Figure 6E hnRNPK

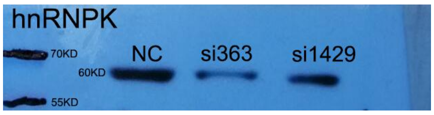

Figure 6E MICAL2

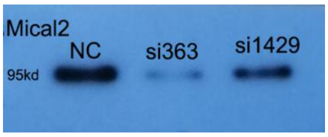

Figure 6E Snail

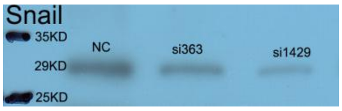

Figure 6E Vimentin

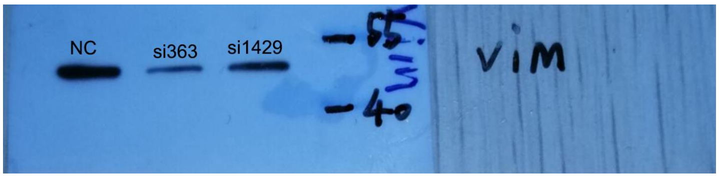

Figure 6F Actin

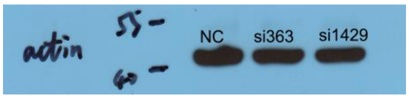

Figure 6F E-cadherin

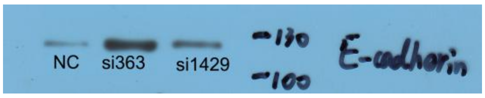

Figure 6F hnRNPK

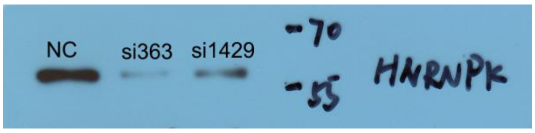

Figure 6F MICAL2

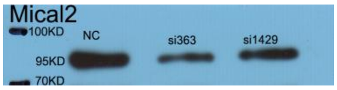

Figure 6F Snail

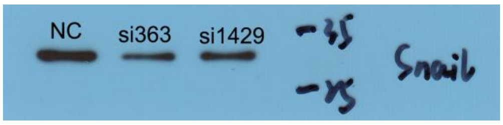

Figure 6F Vimentin

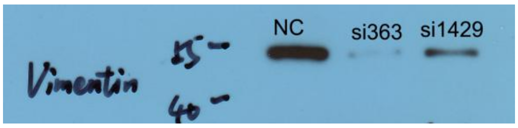

Figure 6G Actin

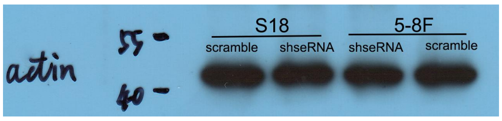

Figure 6G E-cadherin

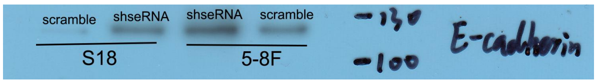

Figure 6G MICAL2

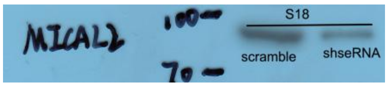

Figure 6G Snail

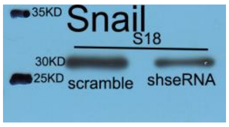

Figure 6G Vimentin

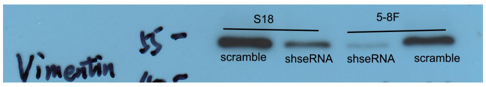

Figure 6H Actin

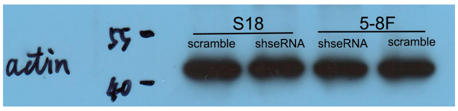

Figure 6H E-cadherin

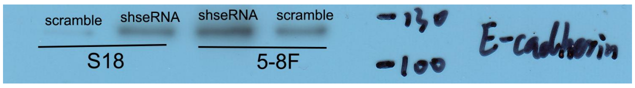

Figure 6H MICAL2

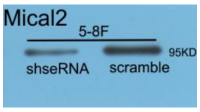

Figure 6H Snail

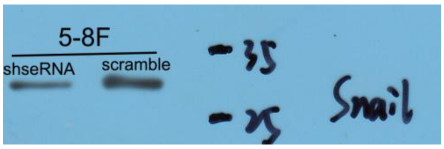

Figure 6H Vimentin

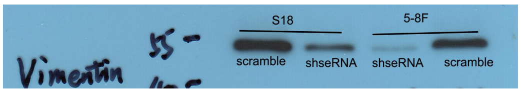

Figure 6I Actin

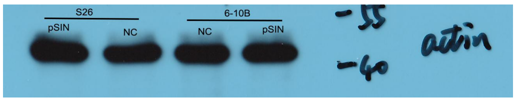

Figure 6I E-cadherin

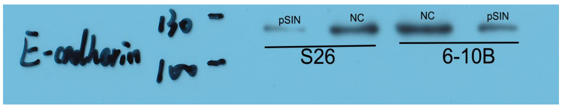

Figure 6I MICAL2

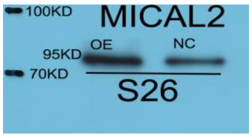

Figure 6I Snail

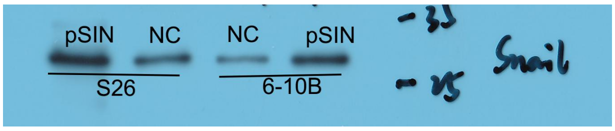

Figure 6I Vimentin

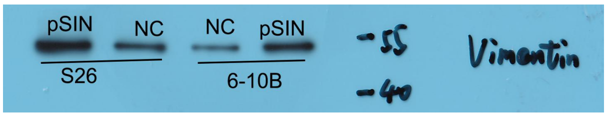

Figure 6J Actin

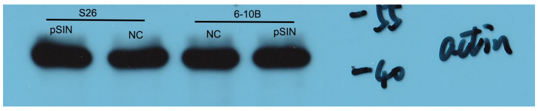

Figure 6J E-cadherin

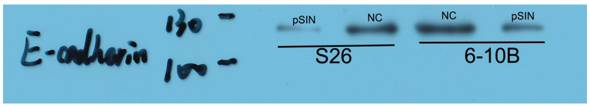

Figure 6J MICAL2

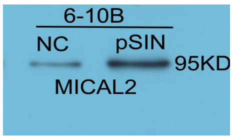

Figure 6J Snail

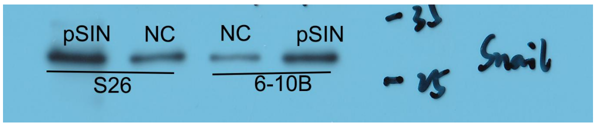

Figure 6J Vimentin

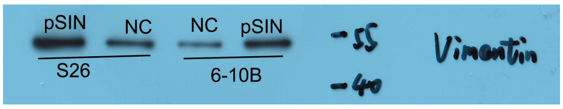

Figure 6K Actin

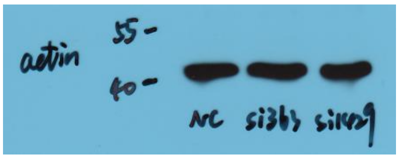

Figure 6K E-cadherin

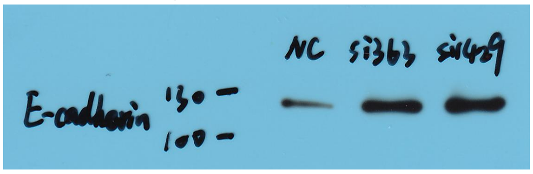

Figure 6K hnRNPK

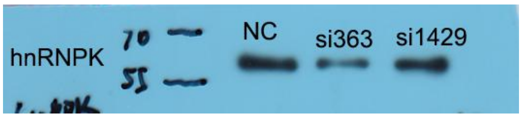

Figure 6K MICAL2

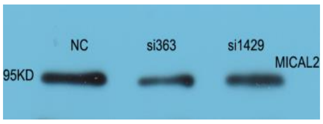

Figure 6K N-cadherin

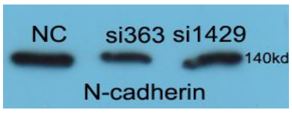

Figure 6K Slug

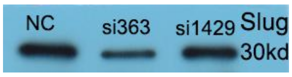

Figure 6K Snail

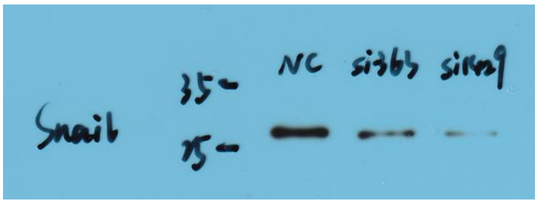

Figure 6K Vimentin

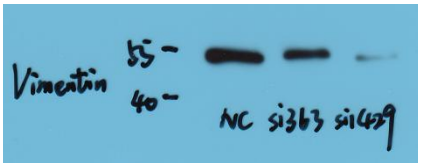

Figure 6L Actin

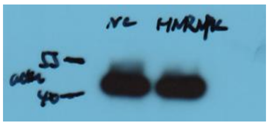

Figure 6L E-cadherin

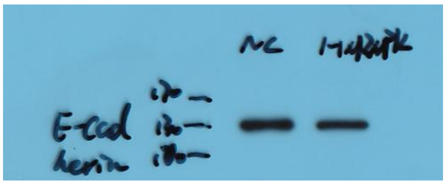

Figure 6L hnRNPK

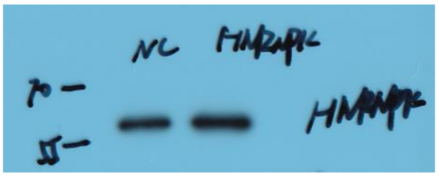

Figure 6L MICAL2

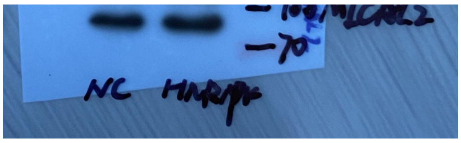

Figure 6L N-cadherin

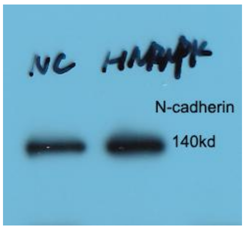

Figure 6L Slug

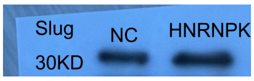

Figure 6L Slug-2

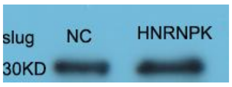

Figure 6L Snail

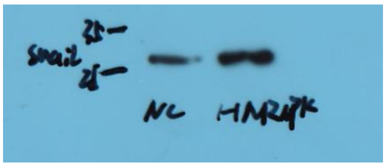

Figure 6L Vimentin-2

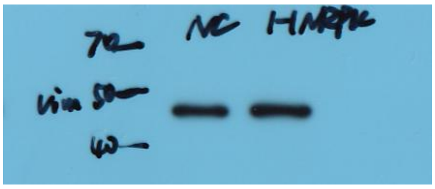

Supplement: Supplementary file 1 — Supplementary Material 1 [file 41419_2022_4846_MOESM1_ESM.pdf]
