# Supplementary material for: A novel oncogenic seRNA promotes nasopharyngeal carcinoma metastasis
Source: Cell Death Dis. 2022 Apr 23;13(4):401. doi: 10.1038/s41419-022-04846-1 (PMC9035166; doi:10.1038/s41419-022-04846-1)
Supplement: Supplementary file 2 — Supplementary Material 2 [file 41419_2022_4846_MOESM2_ESM.pdf]

Supplementary Data 1, H3K27ac ChIP-Seq data of S18 cells

PeakID	Nearest Ensembl	Gene Name	tag
9_S18-150mM_peak_35507_lociStitched	ENSG00000261924	LOC400627	23405.32
19_S18-150mM_peak_75085_lociStitched	ENSG00000164951	PDP1	28551.16
S18-150mM_peak_19962	ENSG00000139725	RHOF	22833.47
5_S18-150mM_peak_38168_lociStitched	ENSG00000104880	ARHGEF18	22826.17
6_S18-150mM_peak_43798_lociStitched	ENSG00000169564	PCBP1	24814.21
S18-150mM_peak_23684	ENSG00000156030	ELMSAN1	22576.3
8_S18-150mM_peak_78871_lociStitched	ENSG00000138835	RGS3	25711.05
3_S18-150mM_peak_34949_lociStitched	ENSG00000167889	MGAT5B	22877.35
6_S18-150mM_peak_50629_lociStitched		LINC00111	23671.68
5_S18-150mM_peak_21888_lociStitched	ENSG00000043355	ZIC2	24364.74
13_S18-150mM_peak_56801_lociStitched	ENSG00000114126	TFDP2	27292.64
7_S18-150mM_peak_19782_lociStitched	ENSG00000135108	FBXO21	24455.36
S18-150mM_peak_64552	ENSG00000113719	ERGIC1	22882.93
10_S18-150mM_peak_16729_lociStitched	ENSG00000139112	GABARAPL1	27025.43
13_S18-150mM_peak_81556_lociStitched	ENSG00000207725	MIR222	24477.88
S18-150mM_peak_38689	ENSG00000160888	IER2	22494.64
5_S18-150mM_peak_61640_lociStitched		FLJ32255	23178.63
9_S18-150mM_peak_71732_lociStitched	ENSG00000160813	PPP1R35	24077.42
S18-150mM_peak_54936	ENSG00000145040	UCN2	23697.8
13_S18-150mM_peak_33068_lociStitched	ENSG00000175832	ETV4	25468.21
S18-150mM_peak_13794	ENSG00000126432	PRDX5	22952.48
S18-150mM_peak_51734	ENSG00000232530	LIF-AS1	23610.29
S18-150mM_peak_53455	ENSG00000196576	PLXNB2	22904.49
13_S18-150mM_peak_79991_lociStitched	ENSG00000196979	LOC401554	26768.05
4_S18-150mM_peak_61655_lociStitched	ENSG00000177721	ANXA2R	23738.72
5_S18-150mM_peak_59109_lociStitched	ENSG00000109220	CHIC2	25816.99
12_S18-150mM_peak_14518_lociStitched	ENSG00000265539	MIR3164	26817.21
23_S18-150mM_peak_22332_lociStitched	ENSG00000185989	RASA3	24303.97
S18-150mM_peak_72390		LINC00513	23061.17
7_S18-150mM_peak_45644_lociStitched	ENSG00000162992	NEUROD1	24267.69
5_S18-150mM_peak_63043_lociStitched	ENSG00000131435	PDLIM4	26440.14
13_S18-150mM_peak_35650_lociStitched	ENSG00000182446	NPLOC4	25068.26
3_S18-150mM_peak_66389_lociStitched	ENSG00000168477	TNXB	23456.98
6_S18-150mM_peak_68676_lociStitched	ENSG00000135577	NMBR	23660.8
6_S18-150mM_peak_34985_lociStitched	ENSG00000184640	9-Sep	24030.65
8_S18-150mM_peak_16235_lociStitched	ENSG00000281097	LINC01395	26300.79
6_S18-150mM_peak_54464_lociStitched	ENSG00000172939	OXSR1	24453.17
12_S18-150mM_peak_53537_lociStitched	ENSG00000235947	EGOT	28148.42
8_S18-150mM_peak_29162_lociStitched	ENSG00000102900	NUP93	24764.96
3_S18-150mM_peak_77689_lociStitched	ENSG00000119138	KLF9	23552.09
8_S18-150mM_peak_12614_lociStitched	ENSG00000273912	MIR8068	26385.56

9_S18-150mM_peak_49267_lociStitched	ENSG00000026559	KCNG1	25538
8_S18-150mM_peak_28871_lociStitched	ENSG00000102870	ZNF629	25279.66
S18-150mM_peak_51024	ENSG00000235374	SSR4P1	23499.17
S18-150mM_peak_14072	ENSG00000173327	MAP3K11	23229.08
11_S18-150mM_peak_18144_lociStitched	ENSG00000258137	LOC102724050	26783.47
6_S18-150mM_peak_11428_lociStitched	ENSG00000240707	LINC01168	23219.08
3_S18-150mM_peak_70988_lociStitched	ENSG00000146648	EGFR	24507.75
8_S18-150mM_peak_33039_lociStitched	ENSG00000284344	MIR2117	27035.04
S18-150mM_peak_42492	ENSG00000171303	KCNK3	23345.98
13_S18-150mM_peak_47453_lociStitched	ENSG00000088826	SMOX	24793.94
7_S18-150mM_peak_42839_lociStitched	ENSG00000171055	FEZ2	27315.34
S18-150mM_peak_44629		FLJ42351	23492.27
7_S18-150mM_peak_25247_lociStitched	ENSG00000138594	TMOD3	25511.24
8_S18-150mM_peak_12352_lociStitched	ENSG00000133794	ARNTL	25270.52
9_S18-150mM_peak_5803_lociStitched	ENSG00000157064	NMNAT2	24630.04
13_S18-150mM_peak_68305_lociStitched	ENSG00000146376	ARHGAP18	28534.33
12_S18-150mM_peak_63509_lociStitched	ENSG00000113068	PFDN1	26284.63
6_S18-150mM_peak_78014_lociStitched	ENSG00000130045	NXNL2	24841.38
2_S18-150mM_peak_24623_lociStitched		LINC02298	23499.26
14_S18-150mM_peak_31984_lociStitched	ENSG00000109046	WSB1	27880.9
7_S18-150mM_peak_38796_lociStitched	ENSG00000123146	ADGRE5	24305.97
S18-150mM_peak_14353	ENSG00000172932	ANKRD13D	23289.55
11_S18-150mM_peak_8476_lociStitched	ENSG00000150093	ITGB1	25408.26
6_S18-150mM_peak_8719_lociStitched	ENSG00000225830	ERCC6	26022.4
2_S18-150mM_peak_79055_lociStitched	ENSG00000119403	PHF19	23912.55
8_S18-150mM_peak_26661_lociStitched	ENSG00000185033	SEMA4B	25012.79
11_S18-150mM_peak_78060_lociStitched		LOC100129316	24955.57
8_S18-150mM_peak_5315_lociStitched	ENSG00000158793	NIT1	24788.65
8_S18-150mM_peak_40260_lociStitched	ENSG00000028277	POU2F2	25417.87
2_S18-150mM_peak_63400_lociStitched	ENSG00000113013	HSPA9	23648.8
4_S18-150mM_peak_838_lociStitched	ENSG00000266079	SNORA59B	24558.86
15_S18-150mM_peak_21628_lociStitched	ENSG00000136153	LMO7	27866.61
17_S18-150mM_peak_34234_lociStitched	ENSG00000261371	PECAM1	28567.31
8_S18-150mM_peak_30218_lociStitched	ENSG00000153786	ZDHHC7	24419.79
9_S18-150mM_peak_53912_lociStitched	ENSG00000163517	HDAC11	25585.51
13_S18-150mM_peak_22043_lociStitched	ENSG00000231428	LINC00396	26563.97
18_S18-150mM_peak_7734_lociStitched	ENSG00000234962	LINC00700	25541.55
S18-150mM_peak_40873	ENSG00000104936	DMPK	24087.22
19_S18-150mM_peak_12472_lociStitched	ENSG00000166833	NAV2	27815.15
S18-150mM_peak_2414	ENSG00000196182	STK40	24031.46
3_S18-150mM_peak_3997_lociStitched	ENSG00000137962	ARHGAP29	25123.92
S18-150mM_peak_72386	ENSG00000231721	LINC-PINT	24062.61
S18-150mM_peak_56640	ENSG00000240006	LINC02004	24446.61
S18-150mM_peak_9322	ENSG00000138303	ASCC1	24569.57

7_S18-150mM_peak_52448_lociStitched	ENSG00000100226	GTPBP1	25781.7
26_S18-150mM_peak_11247_lociStitched	ENSG00000175029	CTBP2	30468.65
1_S18-150mM_peak_44856_lociStitched	ENSG00000144230	GPR17	23923.18
12_S18-150mM_peak_11890_lociStitched	ENSG00000021762	OSBPL5	28067.4
S18-150mM_peak_27120	ENSG00000103326	CAPN15	23804.64
7_S18-150mM_peak_60401_lociStitched	ENSG00000181381	DDX60L	24951.54
11_S18-150mM_peak_81618_lociStitched	ENSG00000147124	ZNF41	24862.41
S18-150mM_peak_13011	ENSG00000149091	DGKZ	24456.72
15_S18-150mM_peak_4260_lociStitched	ENSG00000184371	CSF1	28854.1
11_S18-150mM_peak_16504_lociStitched	ENSG00000008323	PLEKHG6	25299.9
7_S18-150mM_peak_74889_lociStitched	ENSG00000076554	TPD52	26899.86
11_S18-150mM_peak_70716_lociStitched	ENSG00000122515	ZMIZ2	24748.83
5_S18-150mM_peak_4203_lociStitched	ENSG00000134222	PSRC1	24731.97
13_S18-150mM_peak_7771_lociStitched	ENSG00000067057	PFKP	24566.1
12_S18-150mM_peak_15809_lociStitched	ENSG00000110400	NECTIN1	27115.48
10_S18-150mM_peak_62202_lociStitched	ENSG00000049860	HEXB	24876.63
13_S18-150mM_peak_39527_lociStitched	ENSG00000105717	PBX4	26966.01
4_S18-150mM_peak_79140_lociStitched	ENSG00000119522	DENND1A	25635.34
3_S18-150mM_peak_32308_lociStitched	ENSG00000092871	RFFL	25384.83
19_S18-150mM_peak_2273_lociStitched	ENSG00000116514	RNF19B	26809.15
13_S18-150mM_peak_57849_lociStitched		TMEM44-AS1	28336.58
10_S18-150mM_peak_7388_lociStitched	ENSG00000227630	LINC01132	26636.47
17_S18-150mM_peak_80013_lociStitched	ENSG00000107130	NCS1	26976.35
6_S18-150mM_peak_41959_lociStitched	ENSG00000151694	ADAM17	25528.29
13_S18-150mM_peak_41683_lociStitched	ENSG00000142409	ZNF787	26038.33
S18-150mM_peak_16787	ENSG00000178878	APOLD1	23834.36
8_S18-150mM_peak_32841_lociStitched	ENSG00000168259	DNAJC7	26307.1
S18-150mM_peak_11397		C10orf91	23996.5
15_S18-150mM_peak_41208_lociStitched	ENSG00000088002	SULT2B1	27198.08
4_S18-150mM_peak_44779_lociStitched	ENSG00000074047	GLI2	24730.64
11_S18-150mM_peak_13321_lociStitched	ENSG00000172742	OR4D9	26033.55
5_S18-150mM_peak_69729_lociStitched	ENSG00000146535	GNA12	25814.67
5_S18-150mM_peak_63455_lociStitched	ENSG00000184584	TMEM173	26827.8
8_S18-150mM_peak_78642_lociStitched	ENSG00000030304	MUSK	26147.37
9_S18-150mM_peak_8789_lociStitched	ENSG00000231131	LINC01468	26551.93
10_S18-150mM_peak_37096_lociStitched	ENSG00000099821	POLRMT	26798.37
4_S18-150mM_peak_76295_lociStitched	ENSG00000008513	ST3GAL1	25035.78
20_S18-150mM_peak_4400_lociStitched	ENSG00000134198	TSPAN2	26882.85
2_S18-150mM_peak_14426_lociStitched	ENSG00000167797	CDK2AP2	24839.93
S18-150mM_peak_56373	ENSG00000163870	TPRA1	25069.48
4_S18-150mM_peak_47907_lociStitched	ENSG00000171456	ASXL1	25797.82
S18-150mM_peak_3911	ENSG00000137936	BCAR3	24249.36
7_S18-150mM_peak_23333_lociStitched	ENSG00000182185	RAD51B	26077.62
S18-150mM_peak_56423	ENSG00000074416	MGLL	24871.52

11_S18-150mM_peak_15440_lociStitched	ENSG00000110660	SLC35F2	29705.58
9_S18-150mM_peak_47185_lociStitched	ENSG00000063660	GPC1	25253.64
1_S18-150mM_peak_77395_lociStitched	ENSG00000198853	RUSC2	24377.83
14_S18-150mM_peak_62003_lociStitched	ENSG00000112851	ERBIN	29005.46
8_S18-150mM_peak_49589_lociStitched	ENSG00000124225	PMEPA1	28106.52
S18-150mM_peak_35112	ENSG00000184557	SOCS3	24782.71
S18-150mM_peak_16126	ENSG00000110080	ST3GAL4	24855.99
8_S18-150mM_peak_6135_lociStitched	ENSG00000143858	SYT2	26357.74
15_S18-150mM_peak_54037_lociStitched	ENSG00000224660	SH3BP5-AS1	30095.09
10_S18-150mM_peak_48026_lociStitched	ENSG00000078747	ITCH	31215.22
S18-150mM_peak_16633	ENSG00000111676	ATN1	24665.36
9_S18-150mM_peak_49462_lociStitched		SUMO1P1	27994.3
2_S18-150mM_peak_18849_lociStitched	ENSG00000177425	PAWR	24981.45
2_S18-150mM_peak_63854_lociStitched	ENSG00000169252	ADRB2	25350.32
13_S18-150mM_peak_46814_lociStitched	ENSG00000072080	SPP2	26049.21
22_S18-150mM_peak_1643_lociStitched	ENSG00000157978	LDLRAP1	28129.96
10_S18-150mM_peak_73904_lociStitched	ENSG00000240694	PNMA2	26277.56
1_S18-150mM_peak_35928_lociStitched	ENSG00000177426	TGIF1	24366.01
S18-150mM_peak_16827	ENSG00000213782	DDX47	24915.25
S18-150mM_peak_37139	ENSG00000011304	PTBP1	24694.48
9_S18-150mM_peak_73790_lociStitched	ENSG00000180053	NKX2-6	26762.14
7_S18-150mM_peak_19521_lociStitched	ENSG00000174456	C12orf76	26324.02
14_S18-150mM_peak_80118_lociStitched	ENSG00000160539	PLPP7	27086.91
20_S18-150mM_peak_58061_lociStitched		LINC02012	29053.27
5_S18-150mM_peak_28104_lociStitched	ENSG00000199130	MIR365A	25985.13
7_S18-150mM_peak_77327_lociStitched	ENSG00000107341	UBE2R2	28480.57
S18-150mM_peak_72801	ENSG00000265810	MIR3907	25481.84
2_S18-150mM_peak_41267_lociStitched	ENSG00000087074	PPP1R15A	24919.13
1_S18-150mM_peak_45472_lociStitched	ENSG00000236651	DLX2-DT	25310.33
S18-150mM_peak_48630	ENSG00000124116	WFDC3	25138.04
7_S18-150mM_peak_19657_lociStitched	ENSG00000139405	RITA1	28451.12
17_S18-150mM_peak_73225_lociStitched	ENSG00000253958	CLDN23	32216.39
6_S18-150mM_peak_25133_lociStitched	ENSG00000284386	MIR147B	25501.02
8_S18-150mM_peak_81550_lociStitched	ENSG00000229563	LINC01204	25504.1
8_S18-150mM_peak_70225_lociStitched	ENSG00000136244	IL6	26334.25
6_S18-150mM_peak_36198_lociStitched	ENSG00000101558	VAPA	29451.49
5_S18-150mM_peak_11711_lociStitched	ENSG00000184545	DUSP8	25887.72
15_S18-150mM_peak_58445_lociStitched	ENSG00000071127	WDR1	27163.35
7_S18-150mM_peak_15836_lociStitched	ENSG00000184232	OAF	25991.05
18_S18-150mM_peak_67481_lociStitched	ENSG00000207827	MIR30A	29627.5
S18-150mM_peak_48754		LINC01754	25358.33
S18-150mM_peak_27298	ENSG00000206053	JPT2	26187.19
1_S18-150mM_peak_41776_lociStitched	ENSG00000130724	CHMP2A	25166.88
8_S18-150mM_peak_49346_lociStitched	ENSG00000101096	NFATC2	28421.59

S18-150mM_peak_51821	ENSG00000184792	OSBP2	25553.73
15_S18-150mM_peak_70958_lociStitched	ENSG00000146648	EGFR	30728.17
24_S18-150mM_peak_12729_lociStitched	ENSG00000166016	ABTB2	31464.75
S18-150mM_peak_14303	ENSG00000173227	SYT12	26762.77
11_S18-150mM_peak_232_lociStitched	ENSG00000197921	HES5	26721.88
12_S18-150mM_peak_46640_lociStitched	ENSG00000135932	CAB39	27890.53
S18-150mM_peak_47840	ENSG00000125968	ID1	25529.73
8_S18-150mM_peak_32880_lociStitched	ENSG00000126561	STAT5A	28682.08
S18-150mM_peak_66434	ENSG00000204310	AGPAT1	25682.38
9_S18-150mM_peak_57564_lociStitched	ENSG00000163915	IGF2BP2-AS1	28075.58
11_S18-150mM_peak_40848_lociStitched	ENSG00000177051	FBXO46	27238.64
9_S18-150mM_peak_41093_lociStitched	ENSG00000063169	BICRA	27349.24
5_S18-150mM_peak_58413_lociStitched	ENSG00000125089	SH3TC1	26067.73
5_S18-150mM_peak_73360_lociStitched	ENSG00000136573	BLK	27378.36
12_S18-150mM_peak_79550_lociStitched	ENSG00000148339	SLC25A25	31512.44
3_S18-150mM_peak_43105_lociStitched	ENSG00000152518	ZFP36L2	25802.59
16_S18-150mM_peak_16240_lociStitched	ENSG00000151715	TMEM45B	29413.98
5_S18-150mM_peak_24294_lociStitched	ENSG00000185559	DLK1	26058.49
9_S18-150mM_peak_54333_lociStitched	ENSG00000163513	TGFBR2	29690.29
10_S18-150mM_peak_63146_lociStitched	ENSG00000113575	PPP2CA	30249.1
1_S18-150mM_peak_52672_lociStitched	ENSG00000183864	TOB2	25476
14_S18-150mM_peak_11735_lociStitched	ENSG00000117984	CTSD	27345.28
13_S18-150mM_peak_44686_lociStitched	ENSG00000189223	PAX8-AS1	29650.16
25_S18-150mM_peak_60858_lociStitched	ENSG00000113504	SLC12A7	37503.96
13_S18-150mM_peak_29192_lociStitched	ENSG00000140853	NLRC5	29503.35
18_S18-150mM_peak_2664_lociStitched	ENSG00000127129	EDN2	29529.53
13_S18-150mM_peak_11217_lociStitched	ENSG00000264572	MIR4296	28492.38
S18-150mM_peak_546	ENSG00000227634	LINC01714	25932.02
1_S18-150mM_peak_25876_lociStitched	ENSG00000140350	ANP32A	25719.26
7_S18-150mM_peak_43806_lociStitched	ENSG00000231327	LINC01816	26933.71
18_S18-150mM_peak_70114_lociStitched	ENSG00000236039	LOC101927630	31732.32
16_S18-150mM_peak_76275_lociStitched	ENSG00000104419	NDRG1	38060.68
5_S18-150mM_peak_9987_lociStitched	ENSG00000138134	STAMBPL1	28266.53
11_S18-150mM_peak_7828_lociStitched	ENSG00000230573	LOC105376365	27768.76
10_S18-150mM_peak_21551_lociStitched		LINC00392	29731.68
8_S18-150mM_peak_65820_lociStitched	ENSG00000168405	CMAHP	30431.97
8_S18-150mM_peak_770_lociStitched	ENSG00000177000	MTHFR	27538.42
17_S18-150mM_peak_14570_lociStitched		LOC102724265	31161.38
S18-150mM_peak_30790	ENSG00000167716	WDR81	25970.88
16_S18-150mM_peak_60918_lociStitched	ENSG00000153395	LPCAT1	33898.66
16_S18-150mM_peak_27511_lociStitched	ENSG00000059122	FLYWCH1	29892.09
S18-150mM_peak_64974	ENSG00000204628	RACK1	26210.86
11_S18-150mM_peak_63361_lociStitched	ENSG00000152377	SPOCK1	30096.25
4_S18-150mM_peak_13157_lociStitched	ENSG00000109920	FNBP4	26875.99

14_S18-150mM_peak_54608_lociStitched	ENSG00000144647	POMGNT2	29837.44
2_S18-150mM_peak_56944_lociStitched	ENSG00000196428	TSC22D2	25994.89
8_S18-150mM_peak_53870_lociStitched	ENSG00000088726	TMEM40	27858.94
10_S18-150mM_peak_21508_lociStitched	ENSG00000102554	KLF5	27649.51
3_S18-150mM_peak_35629_lociStitched	ENSG00000184009	ACTG1	26471.46
S18-150mM_peak_30445		LOC102724467	26325.49
11_S18-150mM_peak_21274_lociStitched		TPT1-AS1	27932.26
S18-150mM_peak_54948	ENSG00000114270	COL7A1	26715.8
5_S18-150mM_peak_8888_lociStitched	ENSG00000150347	ARID5B	26862.03
18_S18-150mM_peak_43234_lociStitched	ENSG00000116016	EPAS1	30178.97
4_S18-150mM_peak_71150_lociStitched	ENSG00000265724	MIR4284	26896.17
9_S18-150mM_peak_13676_lociStitched	ENSG00000184743	ATL3	31458.09
11_S18-150mM_peak_81351_lociStitched	ENSG00000102172	SMS	27698.63
11_S18-150mM_peak_44379_lociStitched	ENSG00000170485	NPAS2	27724.64
7_S18-150mM_peak_55029_lociStitched	ENSG00000172037	LAMB2	27810.89
5_S18-150mM_peak_15280_lociStitched	ENSG00000248027	LOC100128386	28759.67
13_S18-150mM_peak_76199_lociStitched	ENSG00000264653	MIR5194	31548.43
S18-150mM_peak_69840	ENSG00000075618	FSCN1	27143.58
6_S18-150mM_peak_47988_lociStitched	ENSG00000230753	ZNF341-AS1	28472.41
12_S18-150mM_peak_43979_lociStitched	ENSG00000159399	HK2	29933.04
S18-150mM_peak_10327	ENSG00000155254	MARVELD1	27021.96
S18-150mM_peak_273	ENSG00000130762	ARHGEF16	26595.05
21_S18-150mM_peak_9471_lociStitched	ENSG00000035403	VCL	35955.81
13_S18-150mM_peak_45342_lociStitched	ENSG00000115221	ITGB6	31246.71
14_S18-150mM_peak_42629_lociStitched	ENSG00000223522	LOC100505716	31038.64
S18-150mM_peak_35104	ENSG00000204277	LINC01993	26913.04
10_S18-150mM_peak_79433_lociStitched	ENSG00000160404	TOR2A	29280.37
S18-150mM_peak_54493	ENSG00000144655	CSRNP1	27029.6
9_S18-150mM_peak_26156_lociStitched	ENSG00000137868	STRA6	28948.97
9_S18-150mM_peak_66642_lociStitched	ENSG00000007866	TEAD3	28000.5
S18-150mM_peak_40542	ENSG00000104783	KCNN4	26969.44
14_S18-150mM_peak_7516_lociStitched	ENSG00000143669	LYST	28744.17
14_S18-150mM_peak_69932_lociStitched	ENSG00000008256	CYTH3	31715.45
14_S18-150mM_peak_53680_lociStitched	ENSG00000187288	CIDEC	34275.19
3_S18-150mM_peak_57513_lociStitched	ENSG00000212158	SNORD66	28079.48
19_S18-150mM_peak_40149_lociStitched	ENSG00000233622	CYP2T1P	31826.75
13_S18-150mM_peak_73101_lociStitched	ENSG00000231419	LINC00689	28278.67
2_S18-150mM_peak_72425_lociStitched	ENSG00000128585	MKLN1	26832.06
9_S18-150mM_peak_42422_lociStitched	ENSG00000205639	MFSD2B	27581.72
11_S18-150mM_peak_73533_lociStitched	ENSG00000147408	CSGALNACT1	28341.68
15_S18-150mM_peak_67676_lociStitched	ENSG00000228290	TBX18-AS1	31241.29
2_S18-150mM_peak_29491_lociStitched	ENSG00000135723	FHOD1	27090.18
19_S18-150mM_peak_19732_lociStitched	ENSG00000264037	MIR4472-2	34290.67
11_S18-150mM_peak_35039_lociStitched	ENSG00000184640	9-Sep	29163.32

14_S18-150mM_peak_75291_lociStitched	ENSG00000120963	ZNF706	32198.82
8_S18-150mM_peak_9048_lociStitched	ENSG00000122862	SRGN	28269
6_S18-150mM_peak_18161_lociStitched	ENSG00000135404	CD63	28102.35
S18-150mM_peak_9332	ENSG00000168209	DDIT4	27286.91
4_S18-150mM_peak_56670_lociStitched	ENSG00000182923	CEP63	29508.65
17_S18-150mM_peak_38324_lociStitched	ENSG00000170929	OR1M1	30185.43
8_S18-150mM_peak_2314_lociStitched	ENSG00000142920	AZIN2	28425.45
7_S18-150mM_peak_42593_lociStitched	ENSG00000243147	MRPL33	28357.74
12_S18-150mM_peak_73260_lociStitched		SNORD3I	30593.8
6_S18-150mM_peak_19288_lociStitched	ENSG00000198431	TXNRD1	27823.37
14_S18-150mM_peak_65395_lociStitched		HULC	33829.05
13_S18-150mM_peak_26754_lociStitched	ENSG00000140564	FURIN	30365.25
17_S18-150mM_peak_36542_lociStitched	ENSG00000141622	RNF165	30320.66
S18-150mM_peak_49772	ENSG00000101187	SLCO4A1	27279.89
10_S18-150mM_peak_9514_lociStitched	ENSG00000165637	VDAC2	30878
3_S18-150mM_peak_70258_lociStitched	ENSG00000136231	IGF2BP3	26962.95
6_S18-150mM_peak_39712_lociStitched	ENSG00000012124	CD22	28206.8
12_S18-150mM_peak_34039_lociStitched	ENSG00000284190	MIR21	29219.78
6_S18-150mM_peak_40393_lociStitched	ENSG00000204941	PSG5	28974.13
S18-150mM_peak_73657	ENSG00000241852	C8orf58	27052.96
4_S18-150mM_peak_68850_lociStitched	ENSG00000164520	RAET1E	29273.61
S18-150mM_peak_76691	ENSG00000178209	PLEC	27586.15
15_S18-150mM_peak_26797_lociStitched	ENSG00000176463	SLCO3A1	29415.15
16_S18-150mM_peak_6608_lociStitched		LINC01693	31211.5
11_S18-150mM_peak_39431_lociStitched	ENSG00000105662	CRTC1	30884.36
5_S18-150mM_peak_64628_lociStitched	ENSG00000274994	MIR8056	30003.36
S18-150mM_peak_16810	ENSG00000213782	DDX47	27814.58
3_S18-150mM_peak_373_lociStitched	ENSG00000171680	PLEKHG5	27928.31
S18-150mM_peak_48724	ENSG00000267882	LOC100131496	28914.28
S18-150mM_peak_27491	ENSG00000172460	PRSS30P	27421.86
8_S18-150mM_peak_40126_lociStitched	ENSG00000188493	C19orf54	28932.02
14_S18-150mM_peak_80622_lociStitched	ENSG00000169692	AGPAT2	30011.37
11_S18-150mM_peak_68288_lociStitched	ENSG00000146376	ARHGAP18	29751.94
3_S18-150mM_peak_4328_lociStitched	ENSG00000155366	RHOC	28061.36
18_S18-150mM_peak_48076_lociStitched	ENSG00000100983	GSS	35485.33
13_S18-150mM_peak_52113_lociStitched	ENSG00000100348	TXN2	28380.05
S18-150mM_peak_28888	ENSG00000099385	BCL7C	27575.98
S18-150mM_peak_49135	ENSG00000196396	PTPN1	28282.64
6_S18-150mM_peak_12046_lociStitched	ENSG00000205339	IPO7	28319.81
20_S18-150mM_peak_66839_lociStitched	ENSG00000164626	KCNK5	31255.02
19_S18-150mM_peak_74472_lociStitched	ENSG00000164808	SPIDR	36310.78
S18-150mM_peak_27393	ENSG00000131653	TRAF7	27824.02
4_S18-150mM_peak_66262_lociStitched	ENSG00000206344	HCG27	28722.63
S18-150mM_peak_3941	ENSG00000230439	MIG7	28209.87

S18-150mM_peak_35964		DLGAP1-AS2	27875.69
S18-150mM_peak_39133	ENSG00000130311	DDA1	27805.56
15_S18-150mM_peak_63019_lociStitched	ENSG00000164400	CSF2	34390.09
S18-150mM_peak_35946		DLGAP1-AS2	27563.92
S18-150mM_peak_80590		NALT1	28037.47
4_S18-150mM_peak_17334_lociStitched	ENSG00000111371	SLC38A1	28356.99
12_S18-150mM_peak_62558_lociStitched	ENSG00000207578	MIR583	34473.79
17_S18-150mM_peak_59451_lociStitched	ENSG00000173085	COQ2	29620.88
8_S18-150mM_peak_22849_lociStitched	ENSG00000165527	ARF6	30238.02
S18-150mM_peak_64548	ENSG00000113719	ERGIC1	28297.12
15_S18-150mM_peak_26270_lociStitched	ENSG00000138621	PPCDC	31924.51
1_S18-150mM_peak_16055_lociStitched	ENSG00000154133	ROBO4	28686.68
S18-150mM_peak_75937	ENSG00000283710	MIR1204	28792.67
14_S18-150mM_peak_36478_lociStitched	ENSG00000075643	MOCOS	30434.16
4_S18-150mM_peak_51702_lociStitched	ENSG00000186575	NF2	29041.23
15_S18-150mM_peak_37081_lociStitched	ENSG00000172270	BSG	29587.59
5_S18-150mM_peak_4757_lociStitched	ENSG00000143398	PIP5K1A	28585.38
12_S18-150mM_peak_35333_lociStitched	ENSG00000141582	CBX4	29599.58
7_S18-150mM_peak_74241_lociStitched		LINC01605	29038.46
5_S18-150mM_peak_79076_lociStitched	ENSG00000148180	GSN	30317.84
10_S18-150mM_peak_45584_lociStitched	ENSG00000265396	MIR3128	30741.41
13_S18-150mM_peak_80525_lociStitched	ENSG00000148411	NACC2	31477.94
12_S18-150mM_peak_12757_lociStitched	ENSG00000135373	EHF	32129.1
S18-150mM_peak_6884	ENSG00000162909	CAPN2	28396.25
10_S18-150mM_peak_64923_lociStitched	ENSG00000131459	GFPT2	29803
12_S18-150mM_peak_40446_lociStitched	ENSG00000105755	ETHE1	33085.63
17_S18-150mM_peak_21324_lociStitched	ENSG00000152207	CYSLTR2	30983.43
6_S18-150mM_peak_63954_lociStitched	ENSG00000113721	PDGFRB	30964.39
S18-150mM_peak_33245	ENSG00000214447	FAM187A	28597.21
5_S18-150mM_peak_61571_lociStitched		OSMR-AS1	35482.82
11_S18-150mM_peak_49484_lociStitched		SUMO1P1	31688.14
11_S18-150mM_peak_16884_lociStitched	ENSG00000134531	EMP1	33862.49
12_S18-150mM_peak_49360_lociStitched	ENSG00000054793	ATP9A	30572.69
7_S18-150mM_peak_51238_lociStitched	ENSG00000100075	SLC25A1	29989.47
4_S18-150mM_peak_40_lociStitched	ENSG00000237330	RNF223	29030.61
S18-150mM_peak_11657	ENSG00000214063	TSPAN4	28421.74
8_S18-150mM_peak_17454_lociStitched		LOC105369747	30735.14
S18-150mM_peak_65064		SERPINB9P1	28795.01
6_S18-150mM_peak_47403_lociStitched	ENSG00000275491	LINC01730	28938.25
16_S18-150mM_peak_24351_lociStitched	ENSG00000258512	LINC00239	31104.04
8_S18-150mM_peak_19349_lociStitched	ENSG00000264295	MIR3922	31353.52
18_S18-150mM_peak_56559_lociStitched	ENSG00000004399	PLXND1	32536.32
13_S18-150mM_peak_11403_lociStitched	ENSG00000068383	INPP5A	31445.97
10_S18-150mM_peak_75312_lociStitched	ENSG00000083307	GRHL2	32594.01

6_S18-150mM_peak_19123_lociStitched	ENSG00000180263	FGD6	30227.98
13_S18-150mM_peak_38726_lociStitched	ENSG00000132003	ZSWIM4	30551.82
S18-150mM_peak_7143	ENSG00000198835	GJC2	28669.52
8_S18-150mM_peak_68358_lociStitched		LINC01312	32698.01
S18-150mM_peak_44622		FLJ42351	29077.94
12_S18-150mM_peak_50756_lociStitched	ENSG00000160191	PDE9A	31254.82
14_S18-150mM_peak_2520_lociStitched	ENSG00000127603	MACF1	32138.18
12_S18-150mM_peak_64444_lociStitched	ENSG00000072786	STK10	31165.21
S18-150mM_peak_20367	ENSG00000196498	NCOR2	29563.88
12_S18-150mM_peak_18466_lociStitched	ENSG00000196935	SRGAP1	31281.64
8_S18-150mM_peak_57151_lociStitched	ENSG00000179674	ARL14	31755.37
10_S18-150mM_peak_2103_lociStitched	ENSG00000142910	TINAGL1	30838.92
8_S18-150mM_peak_72895_lociStitched		LOC644090	32589.23
S18-150mM_peak_40775	ENSG00000125753	VASP	29074.77
22_S18-150mM_peak_64460_lociStitched	ENSG00000174705	SH3PXD2B	33582.32
17_S18-150mM_peak_43865_lociStitched	ENSG00000003137	CYP26B1	31484.94
S18-150mM_peak_19484	ENSG00000207622	MIR619	29372.51
5_S18-150mM_peak_66228_lociStitched	ENSG00000204580	DDR1	31697.34
15_S18-150mM_peak_62825_lociStitched	ENSG00000172901	LVRN	33942.25
13_S18-150mM_peak_78683_lociStitched	ENSG00000165181	C9orf84	31492.91
16_S18-150mM_peak_13208_lociStitched	ENSG00000149177	PTPRJ	36977.39
2_S18-150mM_peak_67690_lociStitched	ENSG00000234155	LINC02535	29931.14
S18-150mM_peak_64828	ENSG00000196923	PDLIM7	29134.27
8_S18-150mM_peak_61080_lociStitched	ENSG00000164236	ANKRD33B	33220.13
S18-150mM_peak_35786	ENSG00000265692	LINC01970	29745.74
S18-150mM_peak_40830	ENSG00000125746	EML2	29565.45
27_S18-150mM_peak_78695_lociStitched	ENSG00000165181	C9orf84	39829.53
11_S18-150mM_peak_79207_lociStitched	ENSG00000136930	PSMB7	33494.1
S18-150mM_peak_20354	ENSG00000196498	NCOR2	30572.57
5_S18-150mM_peak_78996_lociStitched	ENSG00000228714	LOC101928775	30431.39
5_S18-150mM_peak_65879_lociStitched	ENSG00000158373	HIST1H2BD	32048.29
12_S18-150mM_peak_41108_lociStitched	ENSG00000063169	BICRA	33137.64
3_S18-150mM_peak_14453_lociStitched	ENSG00000110721	CHKA	31171.85
4_S18-150mM_peak_16342_lociStitched		LOC105369595	29677.87
18_S18-150mM_peak_5571_lociStitched	ENSG00000120337	TNFSF18	35278.96
11_S18-150mM_peak_25877_lociStitched	ENSG00000255346	NOX5	32820.06
10_S18-150mM_peak_42687_lociStitched	ENSG00000115295	CLIP4	31914.4
14_S18-150mM_peak_30258_lociStitched	ENSG00000131149	GSE1	32079.27
S18-150mM_peak_31288	ENSG00000132510	KDM6B	29244.19
S18-150mM_peak_35935		DLGAP1-AS1	29666.86
10_S18-150mM_peak_47202_lociStitched	ENSG00000142327	RNPEPL1	30706.73
S18-150mM_peak_34	ENSG00000188157	AGRN	29479.53
12_S18-150mM_peak_1228_lociStitched	ENSG00000169991	IFFO2	32213.72
S18-150mM_peak_76406	ENSG00000169398	PTK2	29850.48

11_S18-150mM_peak_30266_lociStitched	ENSG00000131149	GSE1	30499.91
S18-150mM_peak_32114	ENSG00000076604	TRAF4	29765.26
13_S18-150mM_peak_26591_lociStitched	ENSG00000140526	ABHD2	33289.72
10_S18-150mM_peak_29651_lociStitched	ENSG00000103018	CYB5B	31965.69
S18-150mM_peak_66333	ENSG00000204394	VARS	29895.67
1_S18-150mM_peak_57123_lociStitched		IQCJ-SCHIP1-AS1	29642.62
9_S18-150mM_peak_1385_lociStitched	ENSG00000142798	HSPG2	31461.13
14_S18-150mM_peak_80941_lociStitched	ENSG00000177138	FAM9B	34801.56
S18-150mM_peak_76469	ENSG00000105339	DENND3	30985.27
21_S18-150mM_peak_33780_lociStitched	ENSG00000141232	TOB1	35701.94
4_S18-150mM_peak_43539_lociStitched	ENSG00000028116	VRK2	30340.92
1_S18-150mM_peak_14799_lociStitched	ENSG00000054967	RELT	31079.38
11_S18-150mM_peak_28936_lociStitched	ENSG00000167394	ZNF668	32163.03
S18-150mM_peak_40738	ENSG00000012061	ERCC1	30310.65
3_S18-150mM_peak_62539_lociStitched	ENSG00000236882	LINC01554	30967.1
12_S18-150mM_peak_39952_lociStitched	ENSG00000183760	ACP7	33228.21
2_S18-150mM_peak_62473_lociStitched	ENSG00000113369	ARRDC3	32754.75
13_S18-150mM_peak_52018_lociStitched	ENSG00000198125	MB	31548.72
4_S18-150mM_peak_50591_lociStitched	ENSG00000232837	LINC01700	34008.14
9_S18-150mM_peak_47268_lociStitched	ENSG00000232002	LINC01880	30458.58
13_S18-150mM_peak_37847_lociStitched	ENSG00000167680	SEMA6B	32202.23
6_S18-150mM_peak_69264_lociStitched	ENSG00000112541	PDE10A	32876.27
S18-150mM_peak_6882	ENSG00000162909	CAPN2	30454.45
S18-150mM_peak_40534	ENSG00000105771	SMG9	31105.45
S18-150mM_peak_20447	ENSG00000265345	MIR5188	30227.2
14_S18-150mM_peak_67855_lociStitched	ENSG00000085377	PREP	33503.23
3_S18-150mM_peak_3403_lociStitched	ENSG00000079739	PGM1	30499.94
15_S18-150mM_peak_35980_lociStitched	ENSG00000170579	DLGAP1	34456.78
10_S18-150mM_peak_64143_lociStitched	ENSG00000263361	MIR378H	36183.58
14_S18-150mM_peak_64702_lociStitched	ENSG00000249306	LINC01411	36775.41
8_S18-150mM_peak_53897_lociStitched	ENSG00000132182	NUP210	32596.48
S18-150mM_peak_79189	ENSG00000119408	NEK6	31407.73
14_S18-150mM_peak_73419_lociStitched	ENSG00000164741	DLC1	33121.29
16_S18-150mM_peak_52277_lociStitched	ENSG00000100097	LGALS1	31783.43
5_S18-150mM_peak_57378_lociStitched	ENSG00000114346	ECT2	31506.12
7_S18-150mM_peak_61261_lociStitched	ENSG00000250822	LINC02111	38579.03
15_S18-150mM_peak_69966_lociStitched	ENSG00000164535	DAGLB	33433.88
9_S18-150mM_peak_69700_lociStitched	ENSG00000146535	GNA12	37071.35
17_S18-150mM_peak_50956_lociStitched	ENSG00000183255	PTTG1IP	33780.91
17_S18-150mM_peak_60758_lociStitched	ENSG00000221990	EXOC3-AS1	42590.24
S18-150mM_peak_72384	ENSG00000283797	MIR29B1	31476.91
5_S18-150mM_peak_15039_lociStitched	ENSG00000073921	PICALM	31674.04
6_S18-150mM_peak_57970_lociStitched	ENSG00000145107	TM4SF19	33142.87
16_S18-150mM_peak_35404_lociStitched	ENSG00000171246	NPTX1	34572.88

8_S18-150mM_peak_10197_lociStitched	ENSG00000173124	ACSM6	32668.2
7_S18-150mM_peak_18264_lociStitched	ENSG00000076067	RBMS2	32526.39
S18-150mM_peak_49904	ENSG00000101197	BIRC7	31286.52
2_S18-150mM_peak_30738_lociStitched	ENSG00000197879	MYO1C	30779.75
S18-150mM_peak_3951	ENSG00000230439	MIG7	30792.84
21_S18-150mM_peak_13511_lociStitched	ENSG00000124942	AHNAK	38503.01
20_S18-150mM_peak_69120_lociStitched	ENSG00000265558	MIR3918	34164.33
7_S18-150mM_peak_15831_lociStitched	ENSG00000137699	TRIM29	34181.88
6_S18-150mM_peak_58649_lociStitched	ENSG00000109819	PPARGC1A	31789.52
20_S18-150mM_peak_82348_lociStitched	ENSG00000102034	ELF4	32969.03
17_S18-150mM_peak_35720_lociStitched	ENSG00000169696	ASPSCR1	33146.17
19_S18-150mM_peak_14795_lociStitched	ENSG00000257038	LOC100287837	34669.29
4_S18-150mM_peak_28848_lociStitched	ENSG00000156860	FBRS	31869.67
S18-150mM_peak_69316	ENSG00000223414	LINC00473	31356.03
S18-150mM_peak_53458	ENSG00000196576	PLXNB2	31076.93
21_S18-150mM_peak_81021_lociStitched	ENSG00000047644	WWC3	37050.95
9_S18-150mM_peak_19084_lociStitched	ENSG00000258274	LOC101928731	35251.85
11_S18-150mM_peak_53822_lociStitched	ENSG00000157150	TIMP4	34479.38
13_S18-150mM_peak_4233_lociStitched	ENSG00000198758	EPS8L3	35751.05
S18-150mM_peak_28711	ENSG00000013364	MVP	31899.82
10_S18-150mM_peak_11499_lociStitched	ENSG00000182272	B4GALNT4	33286.48
S18-150mM_peak_35936		DLGAP1-AS2	31384.59
S18-150mM_peak_52701	ENSG00000100138	SNU13	31652.3
20_S18-150mM_peak_51467_lociStitched	ENSG00000230701	FBXW4P1	35682.93
10_S18-150mM_peak_63789_lociStitched	ENSG00000266478	MIR5197	35358.11
18_S18-150mM_peak_78084_lociStitched	ENSG00000229694	LINC00484	35428.8
15_S18-150mM_peak_29789_lociStitched	ENSG00000214353	VAC14-AS1	33798.86
18_S18-150mM_peak_6167_lociStitched	ENSG00000188770	OPTC	35081.65
9_S18-150mM_peak_39837_lociStitched	ENSG00000167641	PPP1R14A	34559.25
4_S18-150mM_peak_24894_lociStitched	ENSG00000188549	CCDC9B	31792.92
13_S18-150mM_peak_66866_lociStitched	ENSG00000112561	TFEB	36807.7
S18-150mM_peak_31034	ENSG00000108518	PFN1	31314.79
3_S18-150mM_peak_27187_lociStitched	ENSG00000102854	MSLN	31793.26
S18-150mM_peak_38177		LOC100128573	31705.9
S18-150mM_peak_13806	ENSG00000162302	RPS6KA4	31556.32
S18-150mM_peak_63212	ENSG00000043143	JADE2	32275.29
7_S18-150mM_peak_58826_lociStitched	ENSG00000121895	TMEM156	33258.16
S18-150mM_peak_69322	ENSG00000223414	LINC00473	31753.74
25_S18-150mM_peak_29229_lociStitched	ENSG00000102934	PLLP	37010.08
15_S18-150mM_peak_6652_lociStitched	ENSG00000228067	LINC01740	36376.04
8_S18-150mM_peak_22897_lociStitched	ENSG00000214900	LINC01588	35295.12
S18-150mM_peak_53240	ENSG00000197182	MIRLET7BHG	31751.32
13_S18-150mM_peak_16191_lociStitched	ENSG00000134954	ETS1	36806.78
14_S18-150mM_peak_56001_lociStitched		SNORD155	33582.04

15_S18-150mM_peak_60041_lociStitched	ENSG00000151012	SLC7A11	33049.12
9_S18-150mM_peak_37106_lociStitched	ENSG00000070404	FSTL3	33580
S18-150mM_peak_75990	ENSG00000280055	TMEM75	33274.94
13_S18-150mM_peak_6685_lociStitched	ENSG00000162772	ATF3	36505.91
9_S18-150mM_peak_47139_lociStitched	ENSG00000265215	MIR4269	33171.28
S18-150mM_peak_34733	ENSG00000125458	NT5C	32203.35
11_S18-150mM_peak_60830_lociStitched	ENSG00000215246	LOC100506688	35265.8
S18-150mM_peak_63440	ENSG00000170476	MZB1	32582.37
13_S18-150mM_peak_25586_lociStitched	ENSG00000140455	USP3	36718.75
15_S18-150mM_peak_53941_lociStitched	ENSG00000154764	WNT7A	36118.95
1_S18-150mM_peak_17328_lociStitched	ENSG00000189079	ARID2	31969.25
S18-150mM_peak_4862		LOC101928034	33108.7
S18-150mM_peak_13465	ENSG00000167996	FTH1	32086.27
5_S18-150mM_peak_18663_lociStitched	ENSG00000153233	PTPRR	34971.97
10_S18-150mM_peak_51087_lociStitched	ENSG00000237664	LINC00316	33752
4_S18-150mM_peak_71614_lociStitched	ENSG00000164713	BRI3	32072.36
17_S18-150mM_peak_37788_lociStitched	ENSG00000126934	MAP2K2	36880.34
7_S18-150mM_peak_52355_lociStitched	ENSG00000184381	PLA2G6	33229.35
4_S18-150mM_peak_56457_lociStitched	ENSG00000179348	GATA2	34247.55
8_S18-150mM_peak_58257_lociStitched	ENSG00000087266	SH3BP2	32366
20_S18-150mM_peak_3537_lociStitched	ENSG00000116717	GADD45A	39396.98
9_S18-150mM_peak_17985_lociStitched	ENSG00000172819	RARG	34937.83
7_S18-150mM_peak_61485_lociStitched	ENSG00000168685	IL7R	39792.51
24_S18-150mM_peak_80955_lociStitched	ENSG00000101849	TBL1X	39542.93
S18-150mM_peak_69594		LOC100128653	32505.99
5_S18-150mM_peak_41153_lociStitched	ENSG00000142227	EMP3	33260.57
9_S18-150mM_peak_29426_lociStitched	ENSG00000166548	TK2	35979.84
4_S18-150mM_peak_65030_lociStitched		LINC02521	35311.05
17_S18-150mM_peak_43493_lociStitched	ENSG00000115310	RTN4	35572.55
15_S18-150mM_peak_46990_lociStitched	ENSG00000115648	MLPH	36095.34
24_S18-150mM_peak_65927_lociStitched	ENSG00000158406	HIST1H4H	42313.8
14_S18-150mM_peak_58980_lociStitched	ENSG00000064042	LIMCH1	41367.02
16_S18-150mM_peak_37177_lociStitched	ENSG00000175221	MED16	34889.9
2_S18-150mM_peak_24466_lociStitched	ENSG00000198752	CDC42BPB	32965.92
S18-150mM_peak_23189	ENSG00000250548	LINC01303	33046.08
4_S18-150mM_peak_37364_lociStitched	ENSG00000205922	ONECUT3	32666.64
S18-150mM_peak_14233	ENSG00000173621	LRFN4	33167.75
S18-150mM_peak_79644	ENSG00000119335	SET	33300.58
S18-150mM_peak_35120	ENSG00000184557	SOCS3	32937
S18-150mM_peak_27706	ENSG00000126603	GLIS2	33522.77
18_S18-150mM_peak_40804_lociStitched	ENSG00000125741	OPA3	39343.73
15_S18-150mM_peak_23609_lociStitched	ENSG00000197555	SIPA1L1	35683.99
5_S18-150mM_peak_57792_lociStitched	ENSG00000114315	HES1	33485.82
21_S18-150mM_peak_35854_lociStitched	ENSG00000176845	METRNL	36069.76

9_S18-150mM_peak_40918_lociStitched		IGFL2-AS1	36995.14
S18-150mM_peak_40531	ENSG00000105771	SMG9	33381.61
5_S18-150mM_peak_69675_lociStitched	ENSG00000264357	MIR4648	33882.12
3_S18-150mM_peak_27209_lociStitched	ENSG00000162009	SSTR5	34278.3
10_S18-150mM_peak_75835_lociStitched	ENSG00000168672	FAM84B	40103.7
14_S18-150mM_peak_2294_lociStitched	ENSG00000004455	AK2	35493.32
2_S18-150mM_peak_68567_lociStitched	ENSG00000164442	CITED2	33700.08
S18-150mM_peak_69821	ENSG00000075624	ACTB	33512.05
7_S18-150mM_peak_56132_lociStitched	ENSG00000239523	MYLK-AS1	38670.41
12_S18-150mM_peak_28232_lociStitched	ENSG00000072864	NDE1	37490
6_S18-150mM_peak_63279_lociStitched	ENSG00000249647	C5orf66-AS2	37729.89
S18-150mM_peak_77283	ENSG00000165272	AQP3	34415.16
14_S18-150mM_peak_74633_lociStitched	ENSG00000035681	NSMAF	37955.11
S18-150mM_peak_32911	ENSG00000283929	MIR5010	33818.93
S18-150mM_peak_10468	ENSG00000235823	OLMALINC	33433.09
11_S18-150mM_peak_13167_lociStitched	ENSG00000030066	NUP160	38911.33
11_S18-150mM_peak_57409_lociStitched	ENSG00000203645	LINC00501	35718.35
17_S18-150mM_peak_10084_lociStitched		HECTD2-AS1	38165.52
18_S18-150mM_peak_10888_lociStitched		SNORA87	37474.04
8_S18-150mM_peak_61587_lociStitched	ENSG00000164327	RICTOR	39089.54
16_S18-150mM_peak_72162_lociStitched	ENSG00000105976	MET	40342.36
7_S18-150mM_peak_15709_lociStitched	ENSG00000019144	PHLDB1	36080.87
S18-150mM_peak_66375	ENSG00000243649	CFB	34976.95
9_S18-150mM_peak_43448_lociStitched	ENSG00000115306	SPTBN1	35037.47
11_S18-150mM_peak_72316_lociStitched	ENSG00000186591	UBE2H	37202.87
13_S18-150mM_peak_11853_lociStitched	ENSG00000110619	CARS	38002.3
S18-150mM_peak_40056	ENSG00000197019	SERTAD1	34240.55
11_S18-150mM_peak_63689_lociStitched	ENSG00000226272	ARHGAP26-AS1	40624.76
5_S18-150mM_peak_745_lociStitched	ENSG00000175262	C1orf127	34971.46
S18-150mM_peak_47844	ENSG00000264395	MIR3193	34382.48
14_S18-150mM_peak_73844_lociStitched	ENSG00000147459	DOCK5	36325.8
10_S18-150mM_peak_8683_lociStitched		LOC107001062	36663.17
S18-150mM_peak_17860	ENSG00000257671	KRT7-AS	35217.49
7_S18-150mM_peak_46249_lociStitched	ENSG00000135926	TMBIM1	36499.13
1_S18-150mM_peak_4738_lociStitched	ENSG00000143418	CERS2	34072.92
S18-150mM_peak_51010	ENSG00000275874	PICSAR	34088.66
4_S18-150mM_peak_31398_lociStitched	ENSG00000065325	GLP2R	35362.87
18_S18-150mM_peak_23427_lociStitched		LOC100996664	36923.99
21_S18-150mM_peak_2614_lociStitched	ENSG00000171793	CTPS1	41498.71
5_S18-150mM_peak_53921_lociStitched	ENSG00000163520	FBLN2	35968.1
19_S18-150mM_peak_30172_lociStitched	ENSG00000103187	COTL1	37753.91
14_S18-150mM_peak_44056_lociStitched	ENSG00000176407	KCMF1	36722.24
10_S18-150mM_peak_60998_lociStitched	ENSG00000112941	TENT4A	40173.96
7_S18-150mM_peak_65694_lociStitched	ENSG00000124788	ATXN1	36886.05

8_S18-150mM_peak_49934_lociStitched	ENSG00000101213	PTK6	35411.76
8_S18-150mM_peak_62060_lociStitched	ENSG00000197822	OCLN	38291.13
8_S18-150mM_peak_28404_lociStitched	ENSG00000266758	MIR3680-2	35542.14
S18-150mM_peak_37938	ENSG00000276043	UHRF1	34750.02
23_S18-150mM_peak_40975_lociStitched	ENSG00000042753	AP2S1	41024.32
8_S18-150mM_peak_79245_lociStitched	ENSG00000136950	ARPC5L	35902.31
S18-150mM_peak_53556	ENSG00000134107	BHLHE40	34324.96
S18-150mM_peak_77445	ENSG00000196196	HRCT1	34751.44
S18-150mM_peak_23675	ENSG00000264741	MIR4505	35326.29
S18-150mM_peak_2454	ENSG00000283724	MIR6732	34573.28
15_S18-150mM_peak_28188_lociStitched	ENSG00000166780	C16orf45	39922.07
13_S18-150mM_peak_37449_lociStitched	ENSG00000104885	DOT1L	36240.55
8_S18-150mM_peak_1759_lociStitched	ENSG00000117713	ARID1A	37626.87
17_S18-150mM_peak_35607_lociStitched	ENSG00000266392	MIR4740	36317.44
S18-150mM_peak_37918	ENSG00000105355	PLIN3	34870.61
21_S18-150mM_peak_64613_lociStitched	ENSG00000113739	STC2	39850.13
8_S18-150mM_peak_35790_lociStitched	ENSG00000141574	SECTM1	36342.06
21_S18-150mM_peak_52868_lociStitched	ENSG00000100266	PACSIN2	45234.22
20_S18-150mM_peak_39497_lociStitched	ENSG00000167491	GATAD2A	37697.46
13_S18-150mM_peak_52155_lociStitched	ENSG00000128340	RAC2	37325.46
13_S18-150mM_peak_39200_lociStitched	ENSG00000179913	B3GNT3	38953.26
17_S18-150mM_peak_80085_lociStitched	ENSG00000130720	FIBCD1	40997.39
S18-150mM_peak_39990	ENSG00000128016	ZFP36	34953.56
S18-150mM_peak_69587	ENSG00000164880	INTS1	35856.74
S18-150mM_peak_75935	ENSG00000283710	MIR1204	36136.17
S18-150mM_peak_13466	ENSG00000167996	FTH1	34870.3
13_S18-150mM_peak_38102_lociStitched	ENSG00000125657	TNFSF9	39237.44
S18-150mM_peak_27320	ENSG00000265820	MIR3177	35001.89
S18-150mM_peak_9918		AGAP11	35080.95
S18-150mM_peak_12234	ENSG00000275373	MIR6124	35950.3
5_S18-150mM_peak_13728_lociStitched	ENSG00000126500	FLRT1	35512.35
13_S18-150mM_peak_44399_lociStitched	ENSG00000204634	TBC1D8	36383.15
14_S18-150mM_peak_41118_lociStitched	ENSG00000105373	NOP53	36299.13
15_S18-150mM_peak_27852_lociStitched	ENSG00000153048	CARHSP1	40343.42
13_S18-150mM_peak_33610_lociStitched	ENSG00000277478	MIR6165	37931.5
16_S18-150mM_peak_15058_lociStitched	ENSG00000074266	EED	43888.36
5_S18-150mM_peak_10391_lociStitched	ENSG00000023839	ABCC2	36802.8
8_S18-150mM_peak_29890_lociStitched	ENSG00000168404	MLKL	37771.35
12_S18-150mM_peak_26580_lociStitched	ENSG00000172183	ISG20	38286.12
S18-150mM_peak_38826	ENSG00000132002	DNAJB1	36227.29
S18-150mM_peak_32651	ENSG00000131759	RARA	36228.59
S18-150mM_peak_53220	ENSG00000280424	LOC730668	36090.15
1_S18-150mM_peak_30958_lociStitched	ENSG00000004660	CAMKK1	35674.73
6_S18-150mM_peak_50305_lociStitched	ENSG00000159128	IFNGR2	39712.95

13_S18-150mM_peak_75814_lociStitched	ENSG00000168672	FAM84B	41676.97
S18-150mM_peak_17347		LOC100288798	36263.11
6_S18-150mM_peak_76329_lociStitched	ENSG00000167632	TRAPPC9	39273.15
5_S18-150mM_peak_49027_lociStitched	ENSG00000277449	CEBPB-AS1	36870.78
8_S18-150mM_peak_28554_lociStitched	ENSG00000077238	IL4R	38669.88
15_S18-150mM_peak_1800_lociStitched	ENSG00000175793	SFN	38346.73
12_S18-150mM_peak_13245_lociStitched	ENSG00000149115	TNKS1BP1	38615.78
3_S18-150mM_peak_5196_lociStitched	ENSG00000187800	PEAR1	37872.38
15_S18-150mM_peak_6308_lociStitched	ENSG00000158615	PPP1R15B	40523.14
19_S18-150mM_peak_72451_lociStitched	ENSG00000236753	MKLN1-AS	39656.19
2_S18-150mM_peak_16869_lociStitched	ENSG00000111305	GSG1	37472.14
S18-150mM_peak_4861	ENSG00000197956	S100A6	36249.2
6_S18-150mM_peak_28976_lociStitched	ENSG00000140691	ARMC5	37479.98
8_S18-150mM_peak_35385_lociStitched	ENSG00000173821	RNF213	38393.96
11_S18-150mM_peak_64485_lociStitched		LOC101928093	39686.98
S18-150mM_peak_17506	ENSG00000061273	HDAC7	37622.7
15_S18-150mM_peak_9656_lociStitched	ENSG00000224596	ZMIZ1-AS1	38556.29
6_S18-150mM_peak_56257_lociStitched	ENSG00000144908	ALDH1L1	39234.08
14_S18-150mM_peak_39008_lociStitched	ENSG00000127526	SLC35E1	41428.2
S18-150mM_peak_75960	ENSG00000283710	MIR1204	37147.94
13_S18-150mM_peak_68897_lociStitched	ENSG00000120254	MTHFD1L	42166.48
21_S18-150mM_peak_65283_lociStitched	ENSG00000216863	LY86-AS1	44650.7
12_S18-150mM_peak_30761_lociStitched	ENSG00000174238	PITPNA	39641.67
17_S18-150mM_peak_9214_lociStitched	ENSG00000197746	PSAP	41467.88
11_S18-150mM_peak_16417_lociStitched	ENSG00000197905	TEAD4	42367.14
5_S18-150mM_peak_56773_lociStitched	ENSG00000177311	ZBTB38	38349.16
S18-150mM_peak_56655	ENSG00000114019	AMOTL2	37468.42
5_S18-150mM_peak_80662_lociStitched	ENSG00000266507	MIR4479	38638.44
20_S18-150mM_peak_50865_lociStitched	ENSG00000278433	MIR6070	42154.6
4_S18-150mM_peak_80793_lociStitched	ENSG00000187609	EXD3	37699.65
S18-150mM_peak_47826	ENSG00000230613	HM13-AS1	37921.24
8_S18-150mM_peak_4839_lociStitched	ENSG00000163191	S100A11	39197.75
22_S18-150mM_peak_54564_lociStitched	ENSG00000182606	TRAK1	47820.3
24_S18-150mM_peak_71325_lociStitched	ENSG00000106211	HSPB1	41265.79
7_S18-150mM_peak_66750_lociStitched	ENSG00000137193	PIM1	38148.04
S18-150mM_peak_13756	ENSG00000256940	LOC105369340	37460.09
3_S18-150mM_peak_40120_lociStitched	ENSG00000123815	COQ8B	38017.11
S18-150mM_peak_63200	ENSG00000043143	JADE2	37773.54
6_S18-150mM_peak_40970_lociStitched	ENSG00000105281	SLC1A5	38633.79
4_S18-150mM_peak_30720_lociStitched	ENSG00000159842	ABR	37963.42
20_S18-150mM_peak_16167_lociStitched	ENSG00000272575	LINC02098	43363.59
15_S18-150mM_peak_33946_lociStitched	ENSG00000180891	CUEDC1	43072.42
16_S18-150mM_peak_36059_lociStitched	ENSG00000168502	MTCL1	40941.6
24_S18-150mM_peak_60427_lociStitched	ENSG00000129116	PALLD	42349.41

9_S18-150mM_peak_70903_lociStitched	ENSG00000183696	UPP1	39601.71
11_S18-150mM_peak_27055_lociStitched	ENSG00000007384	RHBDF1	40769.29
6_S18-150mM_peak_11695_lociStitched	ENSG00000215182	MUC5AC	38440.01
5_S18-150mM_peak_3094_lociStitched	ENSG00000123080	CDKN2C	38456.77
6_S18-150mM_peak_31522_lociStitched	ENSG00000221355	MIR1288	41501.97
14_S18-150mM_peak_15127_lociStitched	ENSG00000150687	PRSS23	44335
18_S18-150mM_peak_6936_lociStitched	ENSG00000221406	MIR320B2	41275.12
10_S18-150mM_peak_72984_lociStitched		LOC389602	39571.35
4_S18-150mM_peak_58798_lociStitched	ENSG00000231160	KLF3-AS1	38593.06
29_S18-150mM_peak_10432_lociStitched	ENSG00000107554	DNMBP	45137.4
15_S18-150mM_peak_42919_lociStitched	ENSG00000138061	CYP1B1	42092.34
10_S18-150mM_peak_79276_lociStitched	ENSG00000044574	HSPA5	39944.74
4_S18-150mM_peak_76750_lociStitched	ENSG00000160948	VPS28	38702.63
11_S18-150mM_peak_7367_lociStitched	ENSG00000168264	IRF2BP2	38922.96
20_S18-150mM_peak_65148_lociStitched	ENSG00000145945	FAM50B	46762.67
5_S18-150mM_peak_44049_lociStitched	ENSG00000034510	TMSB10	40784.25
13_S18-150mM_peak_2853_lociStitched	ENSG00000142949	PTPRF	40609.37
14_S18-150mM_peak_22162_lociStitched		LINC00431	41170.55
12_S18-150mM_peak_33681_lociStitched	ENSG00000108819	PPP1R9B	40510.1
13_S18-150mM_peak_3282_lociStitched	ENSG00000265822	MIR4422	41351.83
14_S18-150mM_peak_11097_lociStitched	ENSG00000138162	TACC2	42348.63
8_S18-150mM_peak_2722_lociStitched	ENSG00000127124	HIVEP3	41275.39
13_S18-150mM_peak_19755_lociStitched	ENSG00000196668	LINC00173	42511.22
S18-150mM_peak_40729	ENSG00000104881	PPP1R13L	38638.63
S18-150mM_peak_19856	ENSG00000089159	PXN	39266.57
7_S18-150mM_peak_52647_lociStitched	ENSG00000100403	ZC3H7B	39697.09
14_S18-150mM_peak_7467_lociStitched		LINC01348	42825.9
25_S18-150mM_peak_33022_lociStitched	ENSG00000175906	ARL4D	42264.36
S18-150mM_peak_64904	ENSG00000161011	SQSTM1	38366.31
S18-150mM_peak_65053	ENSG00000266750	MIR4645	39550.32
14_S18-150mM_peak_57225_lociStitched	ENSG00000163584	RPL22L1	47146.45
33_S18-150mM_peak_27656_lociStitched	ENSG00000262185	LOC102724927	51267.14
18_S18-150mM_peak_1704_lociStitched	ENSG00000158062	UBXN11	41794.75
15_S18-150mM_peak_37884_lociStitched	ENSG00000142002	DPP9	41397.32
8_S18-150mM_peak_76227_lociStitched	ENSG00000153317	ASAP1	42610.08
S18-150mM_peak_39349	ENSG00000130511	SSBP4	39659.76
4_S18-150mM_peak_94_lociStitched	ENSG00000107404	DVL1	39110.74
11_S18-150mM_peak_23726_lociStitched	ENSG00000119681	LTBP2	41556.72
20_S18-150mM_peak_44119_lociStitched	ENSG00000152292	SH2D6	44034.54
15_S18-150mM_peak_11138_lociStitched	ENSG00000187908	DMBT1	41290.94
S18-150mM_peak_53233	ENSG00000182257	PRR34	38972.74
25_S18-150mM_peak_796_lociStitched	ENSG00000283789	MIR7846	44127.26
18_S18-150mM_peak_39621_lociStitched	ENSG00000131941	RHPN2	43383.52
22_S18-150mM_peak_44521_lociStitched	ENSG00000265965	MIR4266	42005.06

19_S18-150mM_peak_48266_lociStitched	ENSG00000149639	SOGA1	44237.46
15_S18-150mM_peak_50262_lociStitched	ENSG00000166979	EVA1C	43739.33
1_S18-150mM_peak_66457_lociStitched	ENSG00000204256	BRD2	38815.67
10_S18-150mM_peak_51091_lociStitched	ENSG00000237664	LINC00316	41378.01
4_S18-150mM_peak_51338_lociStitched	ENSG00000249923	LOC284865	39883.49
22_S18-150mM_peak_37684_lociStitched	ENSG00000095932	SMIM24	42854.4
S18-150mM_peak_12666	ENSG00000205177	C11orf91	39696.71
22_S18-150mM_peak_44652_lociStitched	ENSG00000115008	IL1A	46021.68
17_S18-150mM_peak_21596_lociStitched	ENSG00000136153	LMO7	43339.94
19_S18-150mM_peak_19186_lociStitched	ENSG00000059758	CDK17	44543.75
17_S18-150mM_peak_56275_lociStitched	ENSG00000163884	KLF15	45312.25
13_S18-150mM_peak_61191_lociStitched	ENSG00000038382	TRIO	53933.16
S18-150mM_peak_19963	ENSG00000139725	RHOF	39856.93
9_S18-150mM_peak_39240_lociStitched	ENSG00000105643	ARRDC2	41696.66
6_S18-150mM_peak_41280_lociStitched	ENSG00000087086	FTL	41811.41
21_S18-150mM_peak_19132_lociStitched	ENSG00000257150	PGAM1P5	43249.41
16_S18-150mM_peak_440_lociStitched	ENSG00000171735	CAMTA1	42538.06
7_S18-150mM_peak_54916_lociStitched	ENSG00000213689	TREX1	41610.26
5_S18-150mM_peak_39784_lociStitched	ENSG00000126247	CAPNS1	40739.52
23_S18-150mM_peak_79354_lociStitched	ENSG00000136859	ANGPTL2	46105.73
6_S18-150mM_peak_46269_lociStitched	ENSG00000144579	CTDSP1	40582.63
8_S18-150mM_peak_6438_lociStitched	ENSG00000182795	C1orf116	42478.85
S18-150mM_peak_34828	ENSG00000092929	UNC13D	40201.01
12_S18-150mM_peak_58875_lociStitched	ENSG00000249241	LINC02265	42885.4
10_S18-150mM_peak_39841_lociStitched	ENSG00000167644	C19orf33	42190.24
7_S18-150mM_peak_22343_lociStitched	ENSG00000185989	RASA3	40916.89
22_S18-150mM_peak_29727_lociStitched	ENSG00000157350	ST3GAL2	43345.18
S18-150mM_peak_32680	ENSG00000131746	TNS4	41440.64
7_S18-150mM_peak_22195_lociStitched	ENSG00000232684	ATP11A-AS1	43442.37
5_S18-150mM_peak_29144_lociStitched	ENSG00000125148	MT2A	41340.03
2_S18-150mM_peak_5003_lociStitched	ENSG00000169242	EFNA1	42033.97
18_S18-150mM_peak_3477_lociStitched	ENSG00000184588	PDE4B	45363.47
20_S18-150mM_peak_41654_lociStitched	ENSG00000231274	SBK3	44607.26
17_S18-150mM_peak_72516_lociStitched	ENSG00000128567	PODXL	44815.41
34_S18-150mM_peak_32007_lociStitched	ENSG00000168961	LGALS9	49281.64
26_S18-150mM_peak_63985_lociStitched	ENSG00000254333	NDST1-AS1	46911.57
7_S18-150mM_peak_52488_lociStitched	ENSG00000100307	CBX7	41789.29
27_S18-150mM_peak_48763_lociStitched		LINC01754	48380.93
12_S18-150mM_peak_25966_lociStitched	ENSG00000259473	LINC02205	44241.04
24_S18-150mM_peak_76590_lociStitched		SNORD149	47084.6
13_S18-150mM_peak_76620_lociStitched	ENSG00000204839	MROH6	42585.5
S18-150mM_peak_49059	ENSG00000203999	LINC01270	42469.55
8_S18-150mM_peak_13026_lociStitched	ENSG00000110497	AMBRA1	44341.18
21_S18-150mM_peak_6104_lociStitched	ENSG00000163435	ELF3	49512.7

S18-150mM_peak_56654	ENSG00000277723	MIR6827	41789.03
20_S18-150mM_peak_1021_lociStitched	ENSG00000185519	FAM131C	45379.86
S18-150mM_peak_76457	ENSG00000105339	DENND3	41598.37
25_S18-150mM_peak_55926_lociStitched	ENSG00000144824	PHLDB2	46554.4
S18-150mM_peak_9327	ENSG00000168209	DDIT4	42071.75
S18-150mM_peak_307	ENSG00000207776	MIR551A	41573.47
11_S18-150mM_peak_77728_lociStitched	ENSG00000119125	GDA	45750.57
S18-150mM_peak_27554	ENSG00000006327	TNFRSF12A	41356.94
13_S18-150mM_peak_59263_lociStitched	ENSG00000081041	CXCL2	43085.17
7_S18-150mM_peak_67038_lociStitched	ENSG00000124688	MAD2L1BP	42796.22
10_S18-150mM_peak_52142_lociStitched	ENSG00000133466	C1QTNF6	45500.37
14_S18-150mM_peak_77790_lociStitched	ENSG00000135046	ANXA1	48722.23
13_S18-150mM_peak_66937_lociStitched	ENSG00000048544	MRPS10	46321.72
12_S18-150mM_peak_34140_lociStitched	ENSG00000173838	10-Mar	45449.74
S18-150mM_peak_80371	ENSG00000186350	RXRA	42049.28
S18-150mM_peak_20446	ENSG00000265345	MIR5188	42167.91
5_S18-150mM_peak_37767_lociStitched	ENSG00000178951	ZBTB7A	42873.38
13_S18-150mM_peak_67428_lociStitched	ENSG00000112245	PTP4A1	49745.66
15_S18-150mM_peak_75646_lociStitched	ENSG00000147689	FAM83A	48110.35
18_S18-150mM_peak_38868_lociStitched	ENSG00000141867	BRD4	48733.58
19_S18-150mM_peak_12289_lociStitched	ENSG00000187079	TEAD1	46389.66
15_S18-150mM_peak_8771_lociStitched	ENSG00000231131	LINC01468	47040.46
S18-150mM_peak_66436	ENSG00000204304	PBX2	43230.66
13_S18-150mM_peak_47781_lociStitched	ENSG00000100994	PYGB	43005.81
13_S18-150mM_peak_22253_lociStitched		LOC101928841	46270.6
12_S18-150mM_peak_32818_lociStitched	ENSG00000173801	JUP	44271.93
18_S18-150mM_peak_47061_lociStitched	ENSG00000065802	ASB1	45662.63
10_S18-150mM_peak_25037_lociStitched		EHD4-AS1	45397.79
28_S18-150mM_peak_60454_lociStitched	ENSG00000129116	PALLD	52614.29
18_S18-150mM_peak_1436_lociStitched	ENSG00000179546	HTR1D	46761.82
7_S18-150mM_peak_26098_lociStitched	ENSG00000067225	PKM	43224.55
34_S18-150mM_peak_74085_lociStitched	ENSG00000251191	LINC00589	51511.46
24_S18-150mM_peak_20104_lociStitched	ENSG00000150967	ABCB9	46064.02
23_S18-150mM_peak_52935_lociStitched	ENSG00000100271	TTLL1	48731.08
22_S18-150mM_peak_42279_lociStitched	ENSG00000068697	LAPTM4A	48919.68
14_S18-150mM_peak_42636_lociStitched	ENSG00000229951	FLJ31356	44383.26
11_S18-150mM_peak_5283_lociStitched	ENSG00000158710	TAGLN2	44199.77
5_S18-150mM_peak_31928_lociStitched	ENSG00000034152	MAP2K3	44256.23
23_S18-150mM_peak_77005_lociStitched		MIR31HG	45198.18
20_S18-150mM_peak_36575_lociStitched	ENSG00000175387	SMAD2	45462.08
S18-150mM_peak_2969	ENSG00000188396	TCTEX1D4	43246.23
16_S18-150mM_peak_12146_lociStitched	ENSG00000247271	ZBED5-AS1	45975.51
19_S18-150mM_peak_32892_lociStitched	ENSG00000177469	CAVIN1	46637.7
14_S18-150mM_peak_65497_lociStitched	ENSG00000137210	TMEM14B	49296.87

11_S18-150mM_peak_43661_lociStitched	ENSG00000234572	LINC01800	46257.77
17_S18-150mM_peak_70969_lociStitched	ENSG00000146648	EGFR	48888.75
9_S18-150mM_peak_27233_lociStitched	ENSG00000103275	UBE2I	44956.7
3_S18-150mM_peak_15314_lociStitched	ENSG00000137693	YAP1	45149.24
26_S18-150mM_peak_43103_lociStitched	ENSG00000152518	ZFP36L2	47916.64
8_S18-150mM_peak_65626_lociStitched	ENSG00000226673	LINC01108	46610.52
15_S18-150mM_peak_63856_lociStitched	ENSG00000207714	MIR584	48717.6
3_S18-150mM_peak_15531_lociStitched	ENSG00000137713	PPP2R1B	45945.76
17_S18-150mM_peak_36177_lociStitched	ENSG00000168454	TXNDC2	49221.01
S18-150mM_peak_11838	ENSG00000181649	PHLDA2	44285.64
5_S18-150mM_peak_10492_lociStitched	ENSG00000107821	KAZALD1	46786.59
S18-150mM_peak_66203	ENSG00000228022	HCG20	44538.94
12_S18-150mM_peak_33389_lociStitched	ENSG00000159314	ARHGAP27	46634
9_S18-150mM_peak_33535_lociStitched	ENSG00000082641	NFE2L1	47673.25
16_S18-150mM_peak_26066_lociStitched		THSD4-AS1	47577.53
S18-150mM_peak_79719	ENSG00000119383	PTPA	44851.6
20_S18-150mM_peak_21848_lociStitched	ENSG00000102572	STK24	48818.49
21_S18-150mM_peak_11795_lociStitched	ENSG00000229414	KCNQ1-AS1	47519.99
32_S18-150mM_peak_15902_lociStitched	ENSG00000255248	MIR100HG	50586.5
36_S18-150mM_peak_49286_lociStitched	ENSG00000266761	MIR3194	58226.63
20_S18-150mM_peak_38121_lociStitched	ENSG00000125726	CD70	48545.17
13_S18-150mM_peak_57750_lociStitched	ENSG00000180611	MB21D2	48604.16
5_S18-150mM_peak_65527_lociStitched	ENSG00000111863	ADTRP	45568.53
24_S18-150mM_peak_37646_lociStitched	ENSG00000141905	NFIC	47233.02
9_S18-150mM_peak_994_lociStitched	ENSG00000116809	ZBTB17	46187.22
18_S18-150mM_peak_29273_lociStitched	ENSG00000205336	ADGRG1	49753.21
S18-150mM_peak_32691	ENSG00000131746	TNS4	45377.66
S18-150mM_peak_80826	ENSG00000203993	ARRDC1-AS1	45627.39
24_S18-150mM_peak_51907_lociStitched	ENSG00000241878	PISD	47552.69
15_S18-150mM_peak_57926_lociStitched	ENSG00000061938	TNK2	49292.23
3_S18-150mM_peak_4876_lociStitched	ENSG00000188643	S100A16	46530
17_S18-150mM_peak_70701_lociStitched	ENSG00000105953	OGDH	47765.87
21_S18-150mM_peak_72428_lociStitched	ENSG00000128585	MKLN1	48997.66
26_S18-150mM_peak_28277_lociStitched	ENSG00000103222	ABCC1	53827.23
13_S18-150mM_peak_35311_lociStitched	ENSG00000141570	CBX8	48730.25
16_S18-150mM_peak_60890_lociStitched	ENSG00000049656	CLPTM1L	50752.64
16_S18-150mM_peak_79239_lociStitched	ENSG00000185585	OLFML2A	52973.48
11_S18-150mM_peak_1354_lociStitched	ENSG00000076864	RAP1GAP	48317.85
18_S18-150mM_peak_48242_lociStitched	ENSG00000080845	DLGAP4	52166.05
S18-150mM_peak_47873	ENSG00000281376	ABALON	46106.19
S18-150mM_peak_9325	ENSG00000168209	DDIT4	46441.95
14_S18-150mM_peak_48203_lociStitched	ENSG00000088367	EPB41L1	51396.68
S18-150mM_peak_73317		LOC102723313	46806.67
22_S18-150mM_peak_33860_lociStitched	ENSG00000121060	TRIM25	51539.5

6_S18-150mM_peak_50897_lociStitched	ENSG00000160209	PDXK	47135.53
21_S18-150mM_peak_12185_lociStitched	ENSG00000050165	DKK3	51188.75
16_S18-150mM_peak_22006_lociStitched	ENSG00000236242	MYO16-AS1	48889.67
14_S18-150mM_peak_9165_lociStitched	ENSG00000237512	UNC5B-AS1	51276.51
16_S18-150mM_peak_10823_lociStitched	ENSG00000138166	DUSP5	51556.37
14_S18-150mM_peak_51295_lociStitched	ENSG00000185838	GNB1L	48448.12
19_S18-150mM_peak_57800_lociStitched	ENSG00000214145	LINC00887	50137.84
17_S18-150mM_peak_69522_lociStitched	ENSG00000164828	SUN1	49564.71
S18-150mM_peak_10699	ENSG00000148841	ITPRIP	46781.04
S18-150mM_peak_19581	ENSG00000111252	SH2B3	46704.64
10_S18-150mM_peak_72350_lociStitched		LOC105375504	49127.86
22_S18-150mM_peak_74016_lociStitched	ENSG00000186918	ZNF395	51505.05
S18-150mM_peak_12239	ENSG00000275373	MIR6124	46736.05
16_S18-150mM_peak_50569_lociStitched		LOC400867	50923.85
6_S18-150mM_peak_44552_lociStitched	ENSG00000144063	MALL	47483.21
20_S18-150mM_peak_1495_lociStitched	ENSG00000117318	ID3	50213.76
20_S18-150mM_peak_78245_lociStitched		LOC101928119	50182.88
11_S18-150mM_peak_30570_lociStitched		LOC100287036	48432.34
10_S18-150mM_peak_56901_lociStitched	ENSG00000169908	TM4SF1	52647.58
15_S18-150mM_peak_1729_lociStitched	ENSG00000176092	CRYBG2	49068.31
17_S18-150mM_peak_38955_lociStitched	ENSG00000127528	KLF2	51246.74
14_S18-150mM_peak_16569_lociStitched	ENSG00000111640	GAPDH	49569.2
S18-150mM_peak_17507	ENSG00000061273	HDAC7	47736.57
3_S18-150mM_peak_17123_lociStitched	ENSG00000235884	LINC00941	47153.96
S18-150mM_peak_11947	ENSG00000166333	ILK	48001.22
11_S18-150mM_peak_11515_lociStitched	ENSG00000184363	PKP3	48216.46
13_S18-150mM_peak_64033_lociStitched	ENSG00000171992	SYNPO	51026.61
9_S18-150mM_peak_33004_lociStitched	ENSG00000188825	LINC00910	48681.82
13_S18-150mM_peak_18479_lociStitched	ENSG00000196935	SRGAP1	54232.61
S18-150mM_peak_53576	ENSG00000235831	BHLHE40-AS1	48885.32
22_S18-150mM_peak_2774_lociStitched	ENSG00000117395	EBNA1BP2	52184.81
S18-150mM_peak_79626	ENSG00000119333	WDR34	48158.11
8_S18-150mM_peak_20029_lociStitched	ENSG00000130779	CLIP1	49903.58
24_S18-150mM_peak_53307_lociStitched	ENSG00000075275	CELSR1	52398.37
S18-150mM_peak_9914	ENSG00000173269	MMRN2	48090
15_S18-150mM_peak_38048_lociStitched	ENSG00000087903	RFX2	51872.32
35_S18-150mM_peak_61741_lociStitched	ENSG00000134363	FST	55970
15_S18-150mM_peak_53779_lociStitched	ENSG00000197548	ATG7	53363.71
2_S18-150mM_peak_22470_lociStitched	ENSG00000129474	AJUBA	47817.83
S18-150mM_peak_35234	ENSG00000178404	CEP295NL	48075.59
10_S18-150mM_peak_42103_lociStitched	ENSG00000115756	HPCAL1	50378.4
25_S18-150mM_peak_80445_lociStitched	ENSG00000130558	OLFM1	53437.2
18_S18-150mM_peak_22886_lociStitched	ENSG00000214900	LINC01588	53092.6
21_S18-150mM_peak_12818_lociStitched		SNORD164	53783.3

S18-150mM_peak_17953	ENSG00000111077	TNS2	49076.56
24_S18-150mM_peak_53031_lociStitched	ENSG00000138944	SHISAL1	52951.61
6_S18-150mM_peak_56912_lociStitched	ENSG00000018408	WWTR1	51392.78
12_S18-150mM_peak_73708_lociStitched	ENSG00000008853	RHOBTB2	51616.08
14_S18-150mM_peak_63736_lociStitched	ENSG00000230789	ARHGAP26-IT1	53165.15
S18-150mM_peak_40191	ENSG00000142039	CCDC97	48709.17
S18-150mM_peak_5084	ENSG00000116584	ARHGEF2	48922.93
14_S18-150mM_peak_75261_lociStitched	ENSG00000248599	FLJ42969	58350.85
14_S18-150mM_peak_26312_lociStitched	ENSG00000173548	SNX33	50650.2
19_S18-150mM_peak_67080_lociStitched		LINC01512	52857.44
S18-150mM_peak_51729	ENSG00000232530	LIF-AS1	48796.22
11_S18-150mM_peak_18785_lociStitched	ENSG00000139289	PHLDA1	51342.75
S18-150mM_peak_31815	ENSG00000177427	MIEF2	48870.47
23_S18-150mM_peak_44816_lociStitched	ENSG00000136717	BIN1	52287.12
13_S18-150mM_peak_63275_lociStitched	ENSG00000249082	C5orf66-AS1	55097.66
21_S18-150mM_peak_40429_lociStitched	ENSG00000124466	LYPD3	56257.74
8_S18-150mM_peak_12422_lociStitched	ENSG00000134333	LDHA	51222.93
S18-150mM_peak_13990	ENSG00000162241	SLC25A45	49650.94
16_S18-150mM_peak_6281_lociStitched	ENSG00000143850	PLEKHA6	55760.48
S18-150mM_peak_56645	ENSG00000240006	LINC02004	49879.91
29_S18-150mM_peak_39077_lociStitched		SNORA118	56826.95
18_S18-150mM_peak_611_lociStitched	ENSG00000230679	ENO1-AS1	51839.09
14_S18-150mM_peak_37373_lociStitched	ENSG00000129911	KLF16	51028.82
23_S18-150mM_peak_56194_lociStitched	ENSG00000082781	ITGB5	58149.11
20_S18-150mM_peak_73004_lociStitched	ENSG00000105993	DNAJB6	52164.93
16_S18-150mM_peak_69082_lociStitched	ENSG00000078269	SYNJ2	53959.99
16_S18-150mM_peak_69486_lociStitched	ENSG00000223855	HRAT92	51193.34
S18-150mM_peak_4970	ENSG00000160691	SHC1	49498.83
19_S18-150mM_peak_43275_lociStitched	ENSG00000239332	LINC01119	53270.61
22_S18-150mM_peak_69941_lociStitched	ENSG00000136238	RAC1	55943.7
6_S18-150mM_peak_46785_lociStitched	ENSG00000077044	DGKD	51907.47
28_S18-150mM_peak_37970_lociStitched	ENSG00000105426	PTPRS	55203.4
16_S18-150mM_peak_24179_lociStitched	ENSG00000205476	CCDC85C	52929.28
S18-150mM_peak_16544	ENSG00000111319	SCNN1A	50495.72
16_S18-150mM_peak_48922_lociStitched	ENSG00000197818	SLC9A8	56134.17
S18-150mM_peak_64557	ENSG00000037241	RPL26L1	51433.83
S18-150mM_peak_4986	ENSG00000160685	ZBTB7B	50370.36
33_S18-150mM_peak_35462_lociStitched	ENSG00000262833	LOC101928855	57882.61
12_S18-150mM_peak_50655_lociStitched	ENSG00000183421	RIPK4	53853.11
S18-150mM_peak_55184	ENSG00000114353	GNAI2	51106.69
10_S18-150mM_peak_63474_lociStitched	ENSG00000171604	CXXC5	54806.84
14_S18-150mM_peak_53892_lociStitched	ENSG00000144711	IQSEC1	54564.81
19_S18-150mM_peak_27089_lociStitched	ENSG00000103126	AXIN1	56982.67
19_S18-150mM_peak_74689_lociStitched	ENSG00000198363	ASPH	57689.96

S18-150mM_peak_49068	ENSG00000233077	LINC01271	51553.99
20_S18-150mM_peak_62594_lociStitched	ENSG00000175426	PCSK1	64171.17
8_S18-150mM_peak_30153_lociStitched	ENSG00000140950	TLDC1	53665.45
25_S18-150mM_peak_74326_lociStitched	ENSG00000169499	PLEKHA2	55407.76
9_S18-150mM_peak_26982_lociStitched	ENSG00000183475	ASB7	52904.39
22_S18-150mM_peak_59012_lociStitched	ENSG00000064042	LIMCH1	60407.08
13_S18-150mM_peak_56744_lociStitched	ENSG00000175110	MRPS22	56206.11
26_S18-150mM_peak_62466_lociStitched	ENSG00000248323	LUCAT1	60730.33
6_S18-150mM_peak_55129_lociStitched	ENSG00000164078	MST1R	53133.91
16_S18-150mM_peak_6994_lociStitched	ENSG00000143815	LBR	53472.47
S18-150mM_peak_52382		SNORA92	51740.68
S18-150mM_peak_66218	ENSG00000214894	LINC00243	52855.85
S18-150mM_peak_4718	ENSG00000143420	ENSA	52465.36
13_S18-150mM_peak_75930_lociStitched	ENSG00000136997	MYC	56051.37
S18-150mM_peak_53239	ENSG00000197182	MIRLET7BHG	51595.17
20_S18-150mM_peak_5438_lociStitched	ENSG00000233693	LOC100505795	58916.29
19_S18-150mM_peak_42065_lociStitched	ENSG00000115756	HPCAL1	55221.18
10_S18-150mM_peak_26810_lociStitched		ASB9P1	54222.91
S18-150mM_peak_17478	ENSG00000079337	RAPGEF3	52593.57
14_S18-150mM_peak_81230_lociStitched	ENSG00000188158	NHS	62476.22
19_S18-150mM_peak_80769_lociStitched	ENSG00000198435	NRARP	55087.65
S18-150mM_peak_14369	ENSG00000175482	POLD4	52909.2
21_S18-150mM_peak_29405_lociStitched	ENSG00000179776	CDH5	56606.12
26_S18-150mM_peak_51550_lociStitched	ENSG00000206069	TMEM211	55572.92
17_S18-150mM_peak_26670_lociStitched	ENSG00000185033	SEMA4B	56264.23
13_S18-150mM_peak_18014_lociStitched	ENSG00000185591	SP1	56375.71
11_S18-150mM_peak_77404_lociStitched	ENSG00000198853	RUSC2	55142.16
S18-150mM_peak_12109	ENSG00000148926	ADM	52835.6
S18-150mM_peak_66202	ENSG00000228022	HCG20	54768.17
S18-150mM_peak_79509	ENSG00000167106	FAM102A	54027.43
13_S18-150mM_peak_32384_lociStitched	ENSG00000277268	LHX1-DT	55806.18
11_S18-150mM_peak_12970_lociStitched	ENSG00000157613	CREB3L1	56565.94
29_S18-150mM_peak_13182_lociStitched	ENSG00000149177	PTPRJ	64347.16
16_S18-150mM_peak_63590_lociStitched	ENSG00000156453	PCDH1	59332.28
10_S18-150mM_peak_6538_lociStitched	ENSG00000196878	LAMB3	55920.39
16_S18-150mM_peak_15617_lociStitched	ENSG00000166741	NNMT	58683.96
27_S18-150mM_peak_4023_lociStitched	ENSG00000117525	F3	61735.55
25_S18-150mM_peak_48895_lociStitched	ENSG00000158470	B4GALT5	62490.5
9_S18-150mM_peak_358_lociStitched	ENSG00000097021	ACOT7	57698.45
25_S18-150mM_peak_20592_lociStitched	ENSG00000177169	ULK1	59237.05
29_S18-150mM_peak_30318_lociStitched	ENSG00000168367	LINC00917	59396.76
22_S18-150mM_peak_70192_lociStitched	ENSG00000232759	LOC100506178	56780.88
6_S18-150mM_peak_63749_lociStitched	ENSG00000113580	NR3C1	54594.36
19_S18-150mM_peak_65202_lociStitched			67639.69

21_S18-150mM_peak_64317_lociStitched	ENSG00000113645	WWC1	59338.79
21_S18-150mM_peak_49530_lociStitched	ENSG00000087510	TFAP2C	61319.19
18_S18-150mM_peak_8765_lociStitched	ENSG00000107984	DKK1	62358.43
18_S18-150mM_peak_70845_lociStitched	ENSG00000136205	TNS3	57070.21
19_S18-150mM_peak_81094_lociStitched	ENSG00000205542	TMSB4X	59697.08
27_S18-150mM_peak_52084_lociStitched	ENSG00000100345	MYH9	58731.11
S18-150mM_peak_14126	ENSG00000175592	FOSL1	54941.1
S18-150mM_peak_66318	ENSG00000213719	CLIC1	55079.2
24_S18-150mM_peak_52170_lociStitched	ENSG00000237862	LOC100506271	58464.34
10_S18-150mM_peak_920_lociStitched	ENSG00000142634	EFHD2	56940.08
17_S18-150mM_peak_63075_lociStitched	ENSG00000125347	IRF1	64557.1
6_S18-150mM_peak_65074_lociStitched		LINC01011	57678.52
24_S18-150mM_peak_78502_lociStitched	ENSG00000136826	KLF4	62704.71
12_S18-150mM_peak_73768_lociStitched	ENSG00000147454	SLC25A37	59338.15
13_S18-150mM_peak_37823_lociStitched	ENSG00000141985	SH3GL1	59482.82
12_S18-150mM_peak_33925_lociStitched	ENSG00000180891	CUEDC1	60010.33
S18-150mM_peak_65054	ENSG00000266750	MIR4645	55729.58
10_S18-150mM_peak_74700_lociStitched	ENSG00000264408	MIR4470	60696.51
13_S18-150mM_peak_71178_lociStitched	ENSG00000189143	CLDN4	58829.84
S18-150mM_peak_66193	ENSG00000137331	IER3	57363.67
15_S18-150mM_peak_53334_lociStitched	ENSG00000054611	TBC1D22A	60160.98
30_S18-150mM_peak_50187_lociStitched	ENSG00000215533	LINC00189	66630.08
9_S18-150mM_peak_61650_lociStitched		FLJ32255	60258.12
14_S18-150mM_peak_73744_lociStitched	ENSG00000253837	LOC100507156	60079.86
7_S18-150mM_peak_38909_lociStitched	ENSG00000167460	TPM4	59504.9
25_S18-150mM_peak_867_lociStitched	ENSG00000276830	MIR6730	64603.84
5_S18-150mM_peak_33208_lociStitched	ENSG00000180340	FZD2	59768.83
17_S18-150mM_peak_23450_lociStitched	ENSG00000185650	ZFP36L1	60321.56
S18-150mM_peak_55189	ENSG00000232352	SEMA3B-AS1	57971.68
S18-150mM_peak_69846	ENSG00000075618	FSCN1	58330
18_S18-150mM_peak_56329_lociStitched	ENSG00000114554	PLXNA1	63807.56
27_S18-150mM_peak_52835_lociStitched	ENSG00000128274	A4GALT	64430.98
25_S18-150mM_peak_73972_lociStitched	ENSG00000265847	MIR4287	62927.06
20_S18-150mM_peak_23463_lociStitched	ENSG00000072110	ACTN1	62661.7
20_S18-150mM_peak_7452_lociStitched	ENSG00000238005	LOC101927851	63700.6
4_S18-150mM_peak_24601_lociStitched	ENSG00000185567	AHNAK2	58760.31
16_S18-150mM_peak_57277_lociStitched	ENSG00000075651	PLD1	64103.44
S18-150mM_peak_53237		LOC642648	58460.47
26_S18-150mM_peak_75503_lociStitched		SNORD168	70555.72
24_S18-150mM_peak_28008_lociStitched	ENSG00000189067	LITAF	64988.52
20_S18-150mM_peak_79687_lociStitched	ENSG00000171097	KYAT1	64340.76
11_S18-150mM_peak_50047_lociStitched	ENSG00000171703	TCEA2	60413.22
27_S18-150mM_peak_656_lociStitched	ENSG00000171621	SPSB1	64017.42
S18-150mM_peak_4868	ENSG00000196754	S100A2	58421.39

S18-150mM_peak_14057	ENSG00000168056	LTBP3	59005.95
18_S18-150mM_peak_21813_lociStitched	ENSG00000139793	MBNL2	65311.5
24_S18-150mM_peak_16022_lociStitched	ENSG00000023171	LOC100128242	65027.9
22_S18-150mM_peak_69758_lociStitched	ENSG00000164916	FOXK1	66080.66
5_S18-150mM_peak_15211_lociStitched	ENSG00000255929	LOC105369438	63454.31
10_S18-150mM_peak_67693_lociStitched	ENSG00000135318	NT5E	64245.89
26_S18-150mM_peak_57298_lociStitched	ENSG00000075420	FNDC3B	68542.51
S18-150mM_peak_19964	ENSG00000212694	LINC01089	59545.88
15_S18-150mM_peak_2386_lociStitched	ENSG00000054116	TRAPPC3	63850.22
28_S18-150mM_peak_20425_lociStitched	ENSG00000073060	SCARB1	65373.31
20_S18-150mM_peak_67537_lociStitched	ENSG00000156508	EEF1A1	66268.36
33_S18-150mM_peak_22746_lociStitched	ENSG00000100906	NFKBIA	67003.3
S18-150mM_peak_33332	ENSG00000267288	LOC105371795	60105.57
S18-150mM_peak_49964	ENSG00000243509	TNFRSF6B	60638.74
21_S18-150mM_peak_9237_lociStitched	ENSG00000122863	CHST3	66528.04
26_S18-150mM_peak_34569_lociStitched	ENSG00000264026	LINC02003	70535.79
26_S18-150mM_peak_45946_lociStitched	ENSG00000237166	LINC01792	67002.96
10_S18-150mM_peak_9206_lociStitched	ENSG00000107738	VSIR	63282.43
S18-150mM_peak_11824	ENSG00000110628	SLC22A18	61639.5
38_S18-150mM_peak_30047_lociStitched	ENSG00000153815	CMIP	68677.26
S18-150mM_peak_16102		GSEC	61461.4
S18-150mM_peak_17857	ENSG00000135480	KRT7	60704.86
31_S18-150mM_peak_50688_lociStitched	ENSG00000160179	ABCG1	69693.71
17_S18-150mM_peak_9997_lociStitched	ENSG00000119922	IFIT2	65815.64
31_S18-150mM_peak_3722_lociStitched	ENSG00000142867	BCL10	68666.1
27_S18-150mM_peak_71800_lociStitched	ENSG00000106366	SERPINE1	64452.43
31_S18-150mM_peak_63328_lociStitched	ENSG00000120708	TGFBI	71549.93
29_S18-150mM_peak_5751_lociStitched	ENSG00000162783	IER5	68768
10_S18-150mM_peak_6427_lociStitched	ENSG00000162892	IL24	62642.5
17_S18-150mM_peak_11063_lociStitched	ENSG00000151929	BAG3	63905.48
S18-150mM_peak_38749	ENSG00000207980	MIR23A	61155.14
25_S18-150mM_peak_13958_lociStitched	ENSG00000149798	CDC42EP2	70310.91
12_S18-150mM_peak_69155_lociStitched	ENSG00000243775	OSTCP1	65209.3
14_S18-150mM_peak_61228_lociStitched	ENSG00000145555	MYO10	72552.54
4_S18-150mM_peak_14148_lociStitched	ENSG00000175315	CST6	63703.96
S18-150mM_peak_5179	ENSG00000143321	HDGF	61829.18
S18-150mM_peak_42508	ENSG00000213699	SLC35F6	63483.7
14_S18-150mM_peak_37619_lociStitched	ENSG00000267551	LOC100996351	65481.79
9_S18-150mM_peak_40416_lociStitched		PRG1	67881.28
4_S18-150mM_peak_61139_lociStitched	ENSG00000039139	DNAH5	66380.61
20_S18-150mM_peak_27825_lociStitched	ENSG00000118898	PPL	68882.72
30_S18-150mM_peak_34427_lociStitched	ENSG00000154217	PITPNC1	70471.74
13_S18-150mM_peak_73354_lociStitched	ENSG00000154319	FAM167A	66123.78
17_S18-150mM_peak_32620_lociStitched	ENSG00000126368	NR1D1	68807.22

S18-150mM_peak_14375	ENSG00000175505	CLCF1	64018.39
6_S18-150mM_peak_29907_lociStitched	ENSG00000050820	BCAR1	64354.22
S18-150mM_peak_63192	ENSG00000251169	LINC01843	63932.45
S18-150mM_peak_64545	ENSG00000113719	ERGIC1	64087.45
42_S18-150mM_peak_42784_lociStitched	ENSG00000150938	CRIM1	74115.72
22_S18-150mM_peak_54856_lociStitched	ENSG00000160799	CCDC12	71654.05
25_S18-150mM_peak_26919_lociStitched	ENSG00000264480	MIR4714	69032.36
S18-150mM_peak_55497	ENSG00000136068	FLNB	64040.8
S18-150mM_peak_49773	ENSG00000101187	SLCO4A1	63913.16
20_S18-150mM_peak_5221_lociStitched		KIRREL1-IT1	67520.83
23_S18-150mM_peak_12201_lociStitched	ENSG00000254486	LINC02547	72684.13
32_S18-150mM_peak_73597_lociStitched	ENSG00000168490	PHYHIP	69256.1
6_S18-150mM_peak_66581_lociStitched	ENSG00000137309	HMGA1	66117.51
14_S18-150mM_peak_76480_lociStitched	ENSG00000022567	SLC45A4	68963.58
27_S18-150mM_peak_33370_lociStitched	ENSG00000006062	MAP3K14	70186.41
31_S18-150mM_peak_49181_lociStitched	ENSG00000196396	PTPN1	82293.41
25_S18-150mM_peak_17167_lociStitched	ENSG00000255867	DENND5B-AS1	72045.1
25_S18-150mM_peak_28202_lociStitched	ENSG00000284184	MIR6506	72139.12
11_S18-150mM_peak_45090_lociStitched	ENSG00000115919	KYNU	66991.62
24_S18-150mM_peak_68199_lociStitched	ENSG00000111912	NCOA7	73515.93
23_S18-150mM_peak_969_lociStitched	ENSG00000162458	FBLIM1	70060.18
7_S18-150mM_peak_58402_lociStitched	ENSG00000196526	AFAP1	66417.07
15_S18-150mM_peak_14742_lociStitched	ENSG00000186635	ARAP1	68745.31
S18-150mM_peak_49782	ENSG00000101187	SLCO4A1	67254.48
21_S18-150mM_peak_25490_lociStitched	ENSG00000182718	ANXA2	72099.67
21_S18-150mM_peak_55093_lociStitched	ENSG00000173402	DAG1	71900.35
10_S18-150mM_peak_30533_lociStitched	ENSG00000103335	PIEZO1	67188.62
30_S18-150mM_peak_42357_lociStitched	ENSG00000143878	RHOB	72845.3
S18-150mM_peak_35754	ENSG00000176155	CCDC57	66350.22
13_S18-150mM_peak_64047_lociStitched	ENSG00000145901	TNIP1	73001.13
19_S18-150mM_peak_34057_lociStitched	ENSG00000284190	MIR21	70030.12
34_S18-150mM_peak_80388_lociStitched	ENSG00000130635	COL5A1	76702.52
19_S18-150mM_peak_15112_lociStitched	ENSG00000150687	PRSS23	74678.37
13_S18-150mM_peak_8671_lociStitched	ENSG00000204175	GPRIN2	76490.44
34_S18-150mM_peak_23044_lociStitched	ENSG00000131981	LGALS3	73658.46
19_S18-150mM_peak_29854_lociStitched	ENSG00000140836	ZFHX3	74930.27
26_S18-150mM_peak_79819_lociStitched	ENSG00000233901	LINC01503	73325.36
36_S18-150mM_peak_20149_lociStitched	ENSG00000090975	PITPNM2	80690.51
21_S18-150mM_peak_32351_lociStitched	ENSG00000277268	LHX1-DT	77685.16
S18-150mM_peak_38750	ENSG00000187556	NANOS3	68023.38
S18-150mM_peak_52421	ENSG00000213923	CSNK1E	69004.4
39_S18-150mM_peak_73204_lociStitched	ENSG00000173295	FAM86B3P	83931.06
33_S18-150mM_peak_35574_lociStitched	ENSG00000185332	TMEM105	74103.54
21_S18-150mM_peak_26179_lociStitched	ENSG00000277391	MIR6881	74027.84

20_S18-150mM_peak_73386_lociStitched	ENSG00000164733	CTSB	76104.3
21_S18-150mM_peak_61166_lociStitched	ENSG00000038382	TRIO	85872.48
36_S18-150mM_peak_80265_lociStitched	ENSG00000160293	VAV2	76865.06
25_S18-150mM_peak_72490_lociStitched	ENSG00000128567	PODXL	76991.46
14_S18-150mM_peak_37696_lociStitched	ENSG00000161091	MFSD12	72530.21
S18-150mM_peak_51738	ENSG00000128342	LIF	71248.68
31_S18-150mM_peak_30675_lociStitched	ENSG00000262003	LOC101927727	77276.71
S18-150mM_peak_49962	ENSG00000243509	TNFRSF6B	71652.59
S18-150mM_peak_55316	ENSG00000164086	DUSP7	72176.01
12_S18-150mM_peak_77206_lociStitched	ENSG00000122711	SPINK4	75633.41
23_S18-150mM_peak_75740_lociStitched	ENSG00000173334	TRIB1	81151.25
14_S18-150mM_peak_30098_lociStitched	ENSG00000140945	CDH13	77516.72
29_S18-150mM_peak_28358_lociStitched	ENSG00000205730	ITPRIPL2	79416.73
22_S18-150mM_peak_8003_lociStitched	ENSG00000225778	PROSER2-AS1	73951.5
22_S18-150mM_peak_51358_lociStitched	ENSG00000099917	MED15	79660.8
37_S18-150mM_peak_68934_lociStitched	ENSG00000120254	MTHFD1L	83996.46
15_S18-150mM_peak_901_lociStitched	ENSG00000233485	LOC101927417	76584.77
21_S18-150mM_peak_17188_lociStitched	ENSG00000151743	AMN1	79362.59
S18-150mM_peak_48720	ENSG00000267882	LOC100131496	74101.14
20_S18-150mM_peak_17681_lociStitched	ENSG00000050405	LIMA1	84226
8_S18-150mM_peak_14181_lociStitched	ENSG00000174791	RIN1	76695.97
S18-150mM_peak_37279	ENSG00000167470	MIDN	74067.49
S18-150mM_peak_13866	ENSG00000171219	CDC42BPG	75367.33
14_S18-150mM_peak_9068_lociStitched	ENSG00000156510	HKDC1	79873.68
51_S18-150mM_peak_72875_lociStitched	ENSG00000106617	PRKAG2	84378.29
34_S18-150mM_peak_7243_lociStitched	ENSG00000143641	GALNT2	84303.17
48_S18-150mM_peak_35148_lociStitched	ENSG00000187775	DNAH17	88107.71
S18-150mM_peak_19847	ENSG00000089159	PXN	76027.62
14_S18-150mM_peak_17395_lociStitched		LOC100288798	83666.29
39_S18-150mM_peak_53191_lociStitched	ENSG00000188064	WNT7B	83286.72
22_S18-150mM_peak_52286_lociStitched	ENSG00000100106	TRIOBP	78483.79
42_S18-150mM_peak_39382_lociStitched	ENSG00000105656	ELL	84881.74
28_S18-150mM_peak_25443_lociStitched	ENSG00000171989	LDHAL6B	83647.51
17_S18-150mM_peak_61072_lociStitched	ENSG00000164236	ANKRD33B	89991.57
20_S18-150mM_peak_56038_lociStitched	ENSG00000082701	GSK3B	84777.55
22_S18-150mM_peak_2106_lociStitched	ENSG00000084636	COL16A1	82476.1
11_S18-150mM_peak_54064_lociStitched	ENSG00000169814	BTD	81817.97
23_S18-150mM_peak_10570_lociStitched	ENSG00000171206	TRIM8	82968.85
19_S18-150mM_peak_13385_lociStitched	ENSG00000134780	DAGLA	84739.6
28_S18-150mM_peak_9750_lociStitched	ENSG00000108179	PPIF	86327.04
9_S18-150mM_peak_48607_lociStitched	ENSG00000124145	SDC4	85411.75
46_S18-150mM_peak_23384_lociStitched		LOC100996664	91191.25
S18-150mM_peak_34719	ENSG00000170190	SLC16A5	79935.47
36_S18-150mM_peak_6240_lociStitched	ENSG00000143842	SOX13	87968.62

42_S18-150mM_peak_17365_lociStitched		LOC100288798	93982.05
10_S18-150mM_peak_38507_lociStitched	ENSG00000161888	SPC24	82516.2
14_S18-150mM_peak_40077_lociStitched	ENSG00000090006	LTBP4	86516.61
19_S18-150mM_peak_74766_lociStitched	ENSG00000169085	VXN	84346.86
S18-150mM_peak_3916	ENSG00000137936	BCAR3	81230.54
S18-150mM_peak_69831	ENSG00000075624	ACTB	81076.18
32_S18-150mM_peak_2255_lociStitched	ENSG00000162522	KIAA1522	86137.24
21_S18-150mM_peak_24427_lociStitched	ENSG00000259230	LINC02323	85784.79
16_S18-150mM_peak_11455_lociStitched	ENSG00000151651	ADAM8	83059.95
27_S18-150mM_peak_44914_lociStitched	ENSG00000178171	AMER3	85448.32
24_S18-150mM_peak_76355_lociStitched	ENSG00000123908	AGO2	94864.48
15_S18-150mM_peak_11748_lociStitched	ENSG00000265258	MIR4686	84366.53
39_S18-150mM_peak_25685_lociStitched	ENSG00000137834	SMAD6	90528.28
9_S18-150mM_peak_61536_lociStitched	ENSG00000164318	EGFLAM	89828.35
21_S18-150mM_peak_38495_lociStitched	ENSG00000130164	LDLR	89900.1
8_S18-150mM_peak_29767_lociStitched	ENSG00000132613	MTSS1L	85856.05
17_S18-150mM_peak_29329_lociStitched		LOC388282	87364.11
34_S18-150mM_peak_27917_lociStitched	ENSG00000182831	C16orf72	96080.94
S18-150mM_peak_16531	ENSG00000067182	TNFRSF1A	84840.41
S18-150mM_peak_38692	ENSG00000160888	IER2	84809.24
S18-150mM_peak_39283	ENSG00000267959	MIR3188	86043.12
S18-150mM_peak_80341	ENSG00000186350	RXRA	85043.94
S18-150mM_peak_48736	ENSG00000101040	ZMYND8	84957.58
S18-150mM_peak_14291	ENSG00000173237	C11orf86	86540.6
27_S18-150mM_peak_11023_lociStitched	ENSG00000198873	GRK5	92255.63
S18-150mM_peak_38631	ENSG00000263800	MIR5684	86120.38
45_S18-150mM_peak_34272_lociStitched	ENSG00000108854	SMURF2	94227.61
11_S18-150mM_peak_5142_lociStitched	ENSG00000116604	MEF2D	90723.5
S18-150mM_peak_49740	ENSG00000130702	LAMA5	85850.16
22_S18-150mM_peak_32513_lociStitched	ENSG00000002834	LASP1	90865.78
14_S18-150mM_peak_60816_lociStitched	ENSG00000071539	TRIP13	95138.64
26_S18-150mM_peak_9801_lociStitched	ENSG00000122359	ANXA11	94406
26_S18-150mM_peak_35498_lociStitched	ENSG00000262833	LOC101928855	93515.66
24_S18-150mM_peak_50379_lociStitched	ENSG00000159216	RUNX1	98050.03
19_S18-150mM_peak_49017_lociStitched	ENSG00000240849	TMEM189	92971.84
13_S18-150mM_peak_63657_lociStitched	ENSG00000231185	SPRY4-AS1	94170.02
S18-150mM_peak_33312	ENSG00000161714	PLCD3	87695.21
29_S18-150mM_peak_79937_lociStitched	ENSG00000148344	PTGES	95436.51
17_S18-150mM_peak_48410_lociStitched	ENSG00000198900	TOP1	93704.49
36_S18-150mM_peak_31714_lociStitched	ENSG00000237328	RAI1-AS1	98524.1
36_S18-150mM_peak_36150_lociStitched	ENSG00000168461	RAB31	96029.33
31_S18-150mM_peak_24244_lociStitched	ENSG00000176473	WDR25	95433.2
15_S18-150mM_peak_80199_lociStitched	ENSG00000160271	RALGDS	93725.85
21_S18-150mM_peak_48175_lociStitched	ENSG00000171222	SCAND1	98347.87

16_S18-150mM_peak_6358_lociStitched	ENSG00000163545	NUAK2	94628.22
S18-150mM_peak_40618	ENSG00000069399	BCL3	91194.12
S18-150mM_peak_20219	ENSG00000275967	MIR6880	91760.96
S18-150mM_peak_39322	ENSG00000130513	GDF15	93004.92
28_S18-150mM_peak_47291_lociStitched	ENSG00000101255	TRIB3	94518.33
S18-150mM_peak_66162	ENSG00000146112	PPP1R18	92246.46
S18-150mM_peak_14009	ENSG00000245532	NEAT1	91202.97
16_S18-150mM_peak_2144_lociStitched	ENSG00000184007	PTP4A2	95117.07
41_S18-150mM_peak_2829_lociStitched	ENSG00000178922	HYI	102737.1
31_S18-150mM_peak_18768_lociStitched	ENSG00000139289	PHLDA1	102937.3
S18-150mM_peak_49739	ENSG00000228812	LAMA5-AS1	93508.48
26_S18-150mM_peak_9432_lociStitched	ENSG00000122861	PLAU	101693.4
S18-150mM_peak_65448	ENSG00000137203	TFAP2A	94337.76
25_S18-150mM_peak_42395_lociStitched	ENSG00000231948	HS1BP3-IT1	102915
17_S18-150mM_peak_24491_lociStitched	ENSG00000185215	TNFAIP2	98990.75
21_S18-150mM_peak_17750_lociStitched	ENSG00000123268	ATF1	105818.5
26_S18-150mM_peak_71250_lociStitched	ENSG00000006704	GTF2IRD1	102173
25_S18-150mM_peak_48483_lociStitched	ENSG00000124191	TOX2	108037.5
10_S18-150mM_peak_30857_lociStitched	ENSG00000070444	MNT	100869.2
24_S18-150mM_peak_49443_lociStitched	ENSG00000235415	LOC105372672	106617.3
S18-150mM_peak_14129	ENSG00000175573	C11orf68	99210.75
S18-150mM_peak_17874	ENSG00000170442	KRT86	100441.1
21_S18-150mM_peak_53727_lociStitched	ENSG00000134070	IRAK2	105714.6
25_S18-150mM_peak_79798_lociStitched	ENSG00000233901	LINC01503	104952.2
32_S18-150mM_peak_34492_lociStitched	ENSG00000108946	PRKAR1A	113802.3
19_S18-150mM_peak_9841_lociStitched	ENSG00000108219	TSPAN14	109148.2
53_S18-150mM_peak_43169_lociStitched	ENSG00000116016	EPAS1	120761.3
40_S18-150mM_peak_46865_lociStitched	ENSG00000130147	SH3BP4	111985.7
48_S18-150mM_peak_54119_lociStitched	ENSG00000131386	GALNT15	119136.5
34_S18-150mM_peak_46668_lociStitched	ENSG00000177673	TEX44	110647.2
33_S18-150mM_peak_2588_lociStitched	ENSG00000179862	CITED4	112972.7
31_S18-150mM_peak_8997_lociStitched	ENSG00000138347	MYPN	117150.2
27_S18-150mM_peak_14533_lociStitched	ENSG00000172927	MYEOV	117889.9
12_S18-150mM_peak_34805_lociStitched	ENSG00000132470	ITGB4	113378.3
S18-150mM_peak_16840	ENSG00000234498	RPL13AP20	110229.9
S18-150mM_peak_47877	ENSG00000281376	ABALON	110189.3
30_S18-150mM_peak_32133_lociStitched	ENSG00000196535	MYO18A	117585.3
34_S18-150mM_peak_77203_lociStitched	ENSG00000233554	B4GALT1-AS1	121027.5
34_S18-150mM_peak_1860_lociStitched	ENSG00000126705	AHDC1	118008.9
S18-150mM_peak_14443	ENSG00000006534	ALDH3B1	110394
57_S18-150mM_peak_31585_lociStitched	ENSG00000133030	MPRIP	124891.3
25_S18-150mM_peak_13474_lociStitched	ENSG00000167996	FTH1	116628.8
42_S18-150mM_peak_41927_lociStitched	ENSG00000151693	ASAP2	122365.2
S18-150mM_peak_48111	ENSG00000125998	FAM83C	112599.7

6_S18-150mM_peak_76526_lociStitched	ENSG00000167653	PSCA	114884.8
14_S18-150mM_peak_31727_lociStitched	ENSG00000072310	SREBF1	114880.5
54_S18-150mM_peak_50843_lociStitched		LINC00322	122588.4
32_S18-150mM_peak_75065_lociStitched	ENSG00000284288	MIR378D2	129745.9
27_S18-150mM_peak_23813_lociStitched	ENSG00000258602	LINC01629	119383.8
S18-150mM_peak_33344	ENSG00000184922	FMNL1	114469.1
31_S18-150mM_peak_75367_lociStitched		GASAL1	127614.5
25_S18-150mM_peak_1332_lociStitched	ENSG00000231105	LOC100506801	123326.9
18_S18-150mM_peak_2748_lociStitched	ENSG00000227533	SLC2A1-AS1	121296
12_S18-150mM_peak_24563_lociStitched	ENSG00000203485	INF2	117704.3
33_S18-150mM_peak_43049_lociStitched	ENSG00000231826	LINC01819	128961.7
45_S18-150mM_peak_54738_lociStitched	ENSG00000163814	CDCP1	139434.8
88_S18-150mM_peak_23531_lociStitched		SNORD169	146892.4
40_S18-150mM_peak_37538_lociStitched	ENSG00000099860	GADD45B	133920
21_S18-150mM_peak_32452_lociStitched	ENSG00000275832	ARHGAP23	133181.9
13_S18-150mM_peak_69797_lociStitched	ENSG00000182095	TNRC18	131332.8
28_S18-150mM_peak_35523_lociStitched	ENSG00000175866	BAIAP2	136635.3
S18-150mM_peak_37262	ENSG00000064932	SBNO2	133748.5
S18-150mM_peak_13535	ENSG00000124942	AHNAK	136588.5
26_S18-150mM_peak_19301_lociStitched	ENSG00000198431	TXNRD1	150022.1
25_S18-150mM_peak_31508_lociStitched	ENSG00000170425	ADORA2B	145657
26_S18-150mM_peak_34986_lociStitched	ENSG00000184640	9-Sep	145702.4
22_S18-150mM_peak_33745_lociStitched	ENSG00000108846	ABCC3	149434.8
8_S18-150mM_peak_5102_lociStitched	ENSG00000160789	LMNA	145891.1
25_S18-150mM_peak_54013_lociStitched	ENSG00000131389	SLC6A6	154694.8
35_S18-150mM_peak_10141_lociStitched	ENSG00000138119	MYOF	161231.2
17_S18-150mM_peak_48556_lociStitched	ENSG00000064205	WISP2	158593.5
S18-150mM_peak_55160	ENSG00000001617	SEMA3F	152455.8
12_S18-150mM_peak_53403_lociStitched	ENSG00000198355	PIM3	154762.7
56_S18-150mM_peak_10790_lociStitched	ENSG00000119953	SMNDC1	166163
S18-150mM_peak_9338	ENSG00000148719	DNAJB12	155093.2
44_S18-150mM_peak_21136_lociStitched	ENSG00000276319	MIR8079	167432.5
35_S18-150mM_peak_67135_lociStitched	ENSG00000237686	LOC101929705	167270.3
18_S18-150mM_peak_40174_lociStitched	ENSG00000167601	AXL	170692.3
37_S18-150mM_peak_34649_lociStitched	ENSG00000179604	CDC42EP4	176055.2
25_S18-150mM_peak_37419_lociStitched	ENSG00000099875	MKNK2	170886.8
S18-150mM_peak_17849	ENSG00000167767	KRT80	170597.8
53_S18-150mM_peak_48353_lociStitched	ENSG00000198959	TGM2	194094.3
68_S18-150mM_peak_24024_lociStitched	ENSG00000258730	ITPK1-AS1	185726.1
44_S18-150mM_peak_17912_lociStitched	ENSG00000170421	KRT8	200009.4
5_S18-150mM_peak_75860_lociStitched		CCAT1	186954.2
67_S18-150mM_peak_9683_lociStitched	ENSG00000108175	ZMIZ1	197492.4
20_S18-150mM_peak_33646_lociStitched	ENSG00000246640	PICART1	188619.1
46_S18-150mM_peak_43336_lociStitched	ENSG00000180398	MCFD2	198357.1

74_S18-150mM_peak_25796_lociStitched	ENSG00000166949	SMAD3	215219.2
41_S18-150mM_peak_39910_lociStitched	ENSG00000130402	ACTN4	221813.8
S18-150mM_peak_17834	ENSG00000167767	KRT80	216359
S18-150mM_peak_76680	ENSG00000178209	PLEC	217998.8
S18-150mM_peak_15730	ENSG00000186174	BCL9L	221337.8
70_S18-150mM_peak_77546_lociStitched	ENSG00000107338	SHB	248675.7
77_S18-150mM_peak_78733_lociStitched	ENSG00000266315	MIR4668	255016.9
24_S18-150mM_peak_79854_lociStitched		LINC00963	237297.4
15_S18-150mM_peak_14032_lociStitched	ENSG00000251562	MALAT1	237507
24_S18-150mM_peak_1044_lociStitched	ENSG00000142627	EPHA2	284740.1
32_S18-150mM_peak_79396_lociStitched	ENSG00000136830	FAM129B	292567.2

Supplementary Data 2, H3K27ac ChIP-Seq data of S26 cells

PeakID	Nearest Ensembl	Gene Name	tag
S26_peak_24786	ENSG00000266559	MIR4530	24381.95
7_S26_peak_6055_lociStitched	ENSG00000231131	LINC01468	26038.71
5_S26_peak_30399_lociStitched	ENSG00000124116	WFDC3	25089.93
2_S26_peak_10166_lociStitched	ENSG00000272575	LINC02098	24347.57
2_S26_peak_6695_lociStitched	ENSG00000234942	GRID1-AS1	24645.35
6_S26_peak_9755_lociStitched	ENSG00000023445	BIRC3	26346.61
S26_peak_25937		LOC101929882	24589.87
8_S26_peak_23499_lociStitched	ENSG00000104885	DOT1L	27009.51
3_S26_peak_49826_lociStitched	ENSG00000252153	MIR2278	26061.64
1_S26_peak_35040_lociStitched	ENSG00000177311	ZBTB38	24913.66
9_S26_peak_52041_lociStitched	ENSG00000180772	AGTR2	25180.46
11_S26_peak_40956_lociStitched	ENSG00000168994	PXDC1	30340.48
3_S26_peak_39662_lociStitched	ENSG00000145779	TNFAIP8	28478.88
8_S26_peak_6577_lociStitched	ENSG00000224596	ZMIZ1-AS1	27013.02
4_S26_peak_16968_lociStitched	ENSG00000137868	STRA6	25724.51
11_S26_peak_32773_lociStitched	ENSG00000238120	LINC01589	29549.14
4_S26_peak_50661_lociStitched	ENSG00000188483	IER5L	26146.95
3_S26_peak_26101_lociStitched	ENSG00000143867	OSR1	25307.09
10_S26_peak_30313_lociStitched	ENSG00000124191	TOX2	28815.43
7_S26_peak_12025_lociStitched	ENSG00000271614	ATP2B1-AS1	26985.97
7_S26_peak_18788_lociStitched	ENSG00000205336	ADGRG1	27359.23
4_S26_peak_13283_lociStitched		LINC00423	26636.35
11_S26_peak_5175_lociStitched	ENSG00000143669	LYST	27713.14
5_S26_peak_48619_lociStitched	ENSG00000169398	PTK2	28755
5_S26_peak_11291_lociStitched	ENSG00000170423	KRT78	26445.69
5_S26_peak_25832_lociStitched	ENSG00000182551	ADI1	25764.63
5_S26_peak_37490_lociStitched	ENSG00000151458	ANKRD50	25161.85
3_S26_peak_20722_lociStitched	ENSG00000131759	RARA	26786.48
4_S26_peak_16617_lociStitched	ENSG00000140455	USP3	26420.19
S26_peak_20788	ENSG00000171401	KRT13	25521.88

5_S26_peak_36101_lociStitched	ENSG00000087266	SH3BP2	26253.08
5_S26_peak_2184_lociStitched	ENSG00000116745	RPE65	25846.38
6_S26_peak_2550_lociStitched	ENSG00000117525	F3	28070.18
14_S26_peak_14834_lociStitched	ENSG00000165527	ARF6	28055.84
10_S26_peak_51230_lociStitched	ENSG00000177138	FAM9B	27183.89
5_S26_peak_30199_lociStitched	ENSG00000088367	EPB41L1	26421.46
11_S26_peak_33555_lociStitched	ENSG00000163513	TGFBR2	28709.67
4_S26_peak_6430_lociStitched	ENSG00000227540	DNAJC9-AS1	25994.43
8_S26_peak_43809_lociStitched	ENSG00000078269	SYNJ2	31635.2
7_S26_peak_6251_lociStitched	ENSG00000099282	TSPAN15	28553.23
S26_peak_40567	ENSG00000120129	DUSP1	25946.49
2_S26_peak_30791_lociStitched		SUMO1P1	26889.01
5_S26_peak_16326_lociStitched	ENSG00000284386	MIR147B	26583.34
5_S26_peak_22730_lociStitched	ENSG00000212710	CTAGE1	25452.52
9_S26_peak_49028_lociStitched	ENSG00000147883	CDKN2B	28016.83
3_S26_peak_17375_lociStitched	ENSG00000140564	FURIN	27092.91
1_S26_peak_41995_lociStitched	ENSG00000096395	MLN	26253.47
3_S26_peak_5985_lociStitched		LOC107001062	27058.39
9_S26_peak_866_lociStitched	ENSG00000117318	ID3	27808.36
5_S26_peak_51443_lociStitched	ENSG00000264090	MIR4666B	25762.81
10_S26_peak_50069_lociStitched	ENSG00000148156	ACTL7B	29136.73
7_S26_peak_50898_lociStitched	ENSG00000186350	RXRA	27411.78
3_S26_peak_41514_lociStitched	ENSG00000187837	HIST1H1C	26297.82
3_S26_peak_29821_lociStitched	ENSG00000125863	MKKS	26293.32
3_S26_peak_42170_lociStitched	ENSG00000112578	BYSL	26818.81
8_S26_peak_31625_lociStitched	ENSG00000183255	PTTG1IP	28916.12
6_S26_peak_35897_lociStitched	ENSG00000276489	MIR6829	27493.74
5_S26_peak_35153_lociStitched	ENSG00000196428	TSC22D2	27332.49
8_S26_peak_41382_lociStitched	ENSG00000124788	ATXN1	28637.76
4_S26_peak_41792_lociStitched	ENSG00000206344	HCG27	26511.14
1_S26_peak_9256_lociStitched	ENSG00000110721	CHKA	26248.61
10_S26_peak_21238_lociStitched	ENSG00000277478	MIR6165	31609.11
7_S26_peak_48237_lociStitched	ENSG00000173334	TRIB1	30081.02
5_S26_peak_44733_lociStitched	ENSG00000164542	KIAA0895	26848
3_S26_peak_8908_lociStitched	ENSG00000110076	NRXN2	26973.17
9_S26_peak_37664_lociStitched	ENSG00000151612	ZNF827	26713.78
2_S26_peak_30719_lociStitched	ENSG00000101096	NFATC2	26859.74
5_S26_peak_45038_lociStitched	ENSG00000146648	EGFR	29577.97
7_S26_peak_10367_lociStitched	ENSG00000008323	PLEKHG6	29190.29
2_S26_peak_15841_lociStitched	ENSG00000283071	LBHD2	26879.55
6_S26_peak_218_lociStitched	ENSG00000207776	MIR551A	28523.82
3_S26_peak_19355_lociStitched	ENSG00000103264	FBXO31	26379.44
13_S26_peak_25092_lociStitched	ENSG00000284258	MIR8085	28797.57
8_S26_peak_39138_lociStitched	ENSG00000049860	HEXB	29398.73

9_S26_peak_24915_lociStitched	ENSG00000160570	DEDD2	28360.89
10_S26_peak_46768_lociStitched	ENSG00000168453	HR	30134.27
5_S26_peak_39124_lociStitched	ENSG00000249343	LINC01333	29048.9
7_S26_peak_29957_lociStitched	ENSG00000204684	LOC284788	27658.02
7_S26_peak_26228_lociStitched	ENSG00000205639	MFSD2B	27213.95
8_S26_peak_26973_lociStitched	ENSG00000233723	LINC01122	30656.66
10_S26_peak_6645_lociStitched	ENSG00000189129	PLAC9	28731.5
3_S26_peak_39863_lociStitched	ENSG00000113575	PPP2CA	27402.3
11_S26_peak_438_lociStitched	ENSG00000171621	SPSB1	31545.62
3_S26_peak_25896_lociStitched	ENSG00000151694	ADAM17	27607.1
S26_peak_20794	ENSG00000171346	KRT15	27318.2
9_S26_peak_40850_lociStitched	ENSG00000164379	FOXQ1	29407.79
1_S26_peak_19604_lociStitched	ENSG00000197879	MYO1C	26869.03
7_S26_peak_42106_lociStitched	ENSG00000137193	PIM1	28060.66
8_S26_peak_47649_lociStitched	ENSG00000272138	LINC01607	28893.89
2_S26_peak_45507_lociStitched	ENSG00000164713	BRI3	26914.45
9_S26_peak_50054_lociStitched	ENSG00000136826	KLF4	28647.89
4_S26_peak_51154_lociStitched	ENSG00000187609	EXD3	27350.43
10_S26_peak_32103_lociStitched	ENSG00000182541	LIMK2	29405.48
5_S26_peak_26773_lociStitched	ENSG00000239332	LINC01119	28276.81
6_S26_peak_17264_lociStitched	ENSG00000172183	ISG20	28550.55
10_S26_peak_49715_lociStitched	ENSG00000229694	LINC00484	30553.56
7_S26_peak_8019_lociStitched	ENSG00000166483	WEE1	28658.15
6_S26_peak_33950_lociStitched	ENSG00000173402	DAG1	28119.6
12_S26_peak_46949_lociStitched	ENSG00000147459	DOCK5	29106.36
4_S26_peak_22539_lociStitched		DLGAP1-AS2	27069.37
4_S26_peak_14110_lociStitched		PCCA-AS1	28201.84
9_S26_peak_44456_lociStitched	ENSG00000236039	LOC101927630	30615.88
1_S26_peak_44429_lociStitched		KCCAT333	27285.66
12_S26_peak_2319_lociStitched	ENSG00000142867	BCL10	30810.42
10_S26_peak_28409_lociStitched	ENSG00000265396	MIR3128	28664.93
S26_peak_3334	ENSG00000160685	ZBTB7B	27729.51
7_S26_peak_26357_lociStitched	ENSG00000243147	MRPL33	28705.21
5_S26_peak_1566_lociStitched	ENSG00000127124	HIVEP3	28896.78
2_S26_peak_27168_lociStitched	ENSG00000244617	ASPRV1	27916.47
2_S26_peak_8065_lociStitched	ENSG00000247271	ZBED5-AS1	27581.17
9_S26_peak_17858_lociStitched	ENSG00000172460	PRSS30P	30906.86
6_S26_peak_42469_lociStitched	ENSG00000137269	LRRC1	28009.95
4_S26_peak_14012_lociStitched		GPC6-AS2	28226.57
9_S26_peak_38550_lociStitched	ENSG00000168685	IL7R	31300.01
2_S26_peak_20564_lociStitched	ENSG00000277268	LHX1-DT	29574.43
6_S26_peak_15393_lociStitched	ENSG00000119681	LTBP2	29233.12
8_S26_peak_23653_lociStitched	ENSG00000105298	CACTIN	29263.23
4_S26_peak_23307_lociStitched	ENSG00000011304	PTBP1	29393.88

6_S26_peak_19483_lociStitched		LOC100287036	29218.87
2_S26_peak_51994_lociStitched	ENSG00000197565	COL4A6	27760.75
22_S26_peak_49334_lociStitched	ENSG00000122696	SLC25A51	37438.47
9_S26_peak_46605_lociStitched	ENSG00000171060	C8orf74	33354.25
5_S26_peak_44431_lociStitched		KCCAT333	28035.06
9_S26_peak_21343_lociStitched	ENSG00000008294	SPAG9	29494.61
12_S26_peak_24841_lociStitched	ENSG00000090006	LTBP4	30999.9
3_S26_peak_29553_lociStitched	ENSG00000063660	GPC1	28721.56
11_S26_peak_48353_lociStitched	ENSG00000283710	MIR1204	30502.31
4_S26_peak_34397_lociStitched	ENSG00000265328	MIR548AB	27945.14
8_S26_peak_9578_lociStitched	ENSG00000150687	PRSS23	32430.97
14_S26_peak_11670_lociStitched		LOC100507065	33691.32
7_S26_peak_11031_lociStitched	ENSG00000139209	SLC38A4	31270
9_S26_peak_6326_lociStitched	ENSG00000197746	PSAP	31194.75
1_S26_peak_13403_lociStitched	ENSG00000120688	WBP4	28182.73
11_S26_peak_30352_lociStitched	ENSG00000064205	WISP2	30700.45
4_S26_peak_4428_lociStitched	ENSG00000196352	CD55	32562.36
3_S26_peak_21093_lociStitched	ENSG00000267288	LOC105371795	28832.96
5_S26_peak_39463_lociStitched	ENSG00000153113	CAST	31939.62
2_S26_peak_24612_lociStitched	ENSG00000089356	FXYD3	29726.82
5_S26_peak_33260_lociStitched	ENSG00000163520	FBLN2	29680.7
5_S26_peak_39913_lociStitched	ENSG00000249082	C5orf66-AS1	31447.56
8_S26_peak_34688_lociStitched	ENSG00000239523	MYLK-AS1	33567.29
12_S26_peak_52126_lociStitched	ENSG00000102034	ELF4	30818.23
1_S26_peak_19736_lociStitched	ENSG00000004660	CAMKK1	28850.17
3_S26_peak_3548_lociStitched	ENSG00000158793	NIT1	28983.31
16_S26_peak_13068_lociStitched	ENSG00000177169	ULK1	32052.06
8_S26_peak_22202_lociStitched	ENSG00000171246	NPTX1	29987.59
5_S26_peak_23439_lociStitched	ENSG00000205922	ONECUT3	28843.04
11_S26_peak_23609_lociStitched	ENSG00000095932	SMIM24	30981.55
5_S26_peak_18813_lociStitched		LOC388282	30589.59
14_S26_peak_4804_lociStitched	ENSG00000143815	LBR	32930.12
2_S26_peak_39406_lociStitched	ENSG00000236882	LINC01554	29098.31
9_S26_peak_22952_lociStitched	ENSG00000101665	SMAD7	31872.6
S26_peak_14365	ENSG00000139842	CUL4A	29002.37
2_S26_peak_25443_lociStitched	ENSG00000087074	PPP1R15A	28786.22
9_S26_peak_11764_lociStitched	ENSG00000153233	PTPRR	31292.54
6_S26_peak_31423_lociStitched		LINC00111	31153.88
6_S26_peak_27396_lociStitched	ENSG00000176407	KCMF1	30456.54
14_S26_peak_24565_lociStitched	ENSG00000131941	RHPN2	30578.97
6_S26_peak_45008_lociStitched		LOC100996654	30167
2_S26_peak_19788_lociStitched	ENSG00000108515	ENO3	29810.89
4_S26_peak_8899_lociStitched	ENSG00000162302	RPS6KA4	29336.38
6_S26_peak_36616_lociStitched	ENSG00000174343	CHRNA9	31761.7

5_S26_peak_22549_lociStitched		DLGAP1-AS2	30169.89
12_S26_peak_8511_lociStitched	ENSG00000110497	AMBRA1	31948.55
15_S26_peak_25353_lociStitched	ENSG00000063169	BICRA	33588.2
9_S26_peak_35681_lociStitched	ENSG00000230115	TPRG1-AS2	34278.02
4_S26_peak_13168_lociStitched	ENSG00000102699	PARP4	30913.65
1_S26_peak_7963_lociStitched	ENSG00000166337	TAF10	30204.83
2_S26_peak_31803_lociStitched	ENSG00000236499	LINC00896	29415.82
6_S26_peak_4888_lociStitched	ENSG00000143776	CDC42BPA	32186.88
12_S26_peak_21105_lociStitched	ENSG00000006062	MAP3K14	32974.52
6_S26_peak_11815_lociStitched	ENSG00000111615	KRR1	31830.46
S26_peak_41180	ENSG00000285278	TFAP2A-AS2	30047.91
12_S26_peak_47091_lociStitched	ENSG00000251191	LINC00589	31404.58
4_S26_peak_16851_lociStitched	ENSG00000259473	LINC02205	31833.56
2_S26_peak_33675_lociStitched	ENSG00000144655	CSRNP1	31341.47
7_S26_peak_29942_lociStitched	ENSG00000173418	NAA20	31031.25
1_S26_peak_35467_lociStitched	ENSG00000114346	ECT2	29631.36
8_S26_peak_41160_lociStitched	ENSG00000285278	TFAP2A-AS2	32751.89
13_S26_peak_7370_lociStitched		SNORA87	31678.11
2_S26_peak_39340_lociStitched	ENSG00000248323	LUCAT1	30083.96
5_S26_peak_15374_lociStitched	ENSG00000156030	ELMSAN1	32381.94
6_S26_peak_31480_lociStitched	ENSG00000160179	ABCG1	32387.97
14_S26_peak_28331_lociStitched	ENSG00000091409	ITGA6	33491.66
4_S26_peak_42974_lociStitched	ENSG00000203801	LINC00222	31104.45
6_S26_peak_29166_lociStitched		LOC646736	30850.29
11_S26_peak_29510_lociStitched	ENSG00000065802	ASB1	31113.3
6_S26_peak_692_lociStitched	ENSG00000142619	PADI3	32290.93
7_S26_peak_6948_lociStitched	ENSG00000173124	ACSM6	33208.36
1_S26_peak_41265_lociStitched	ENSG00000095951	HIVEP1	30043.38
5_S26_peak_28978_lociStitched	ENSG00000135926	TMBIM1	32515.9
13_S26_peak_25980_lociStitched	ENSG00000115756	HPCAL1	33559.02
13_S26_peak_19047_lociStitched	ENSG00000132613	MTSS1L	32349.5
3_S26_peak_32567_lociStitched	ENSG00000100403	ZC3H7B	31169.6
13_S26_peak_21751_lociStitched	ENSG00000260785	CASC17	34327.06
S26_peak_34973	ENSG00000114019	AMOTL2	30509.44
6_S26_peak_11296_lociStitched	ENSG00000170421	KRT8	32234.75
2_S26_peak_484_lociStitched	ENSG00000175262	C1orf127	30712.09
11_S26_peak_41132_lociStitched		HULC	35118.62
2_S26_peak_26894_lociStitched	ENSG00000115306	SPTBN1	32286.48
3_S26_peak_42281_lociStitched	ENSG00000172432	GTPBP2	30529.21
8_S26_peak_43243_lociStitched	ENSG00000146376	ARHGAP18	32672.18
15_S26_peak_25912_lociStitched	ENSG00000115750	TAF1B	36255.1
10_S26_peak_25362_lociStitched	ENSG00000105373	NOP53	32843.98
4_S26_peak_43655_lociStitched	ENSG00000120254	MTHFD1L	31199.7
5_S26_peak_1331_lociStitched	ENSG00000004455	AK2	33183.91

12_S26_peak_32659_lociStitched	ENSG00000128274	A4GALT	35989.75
3_S26_peak_41763_lociStitched	ENSG00000204580	DDR1	32777.41
12_S26_peak_46350_lociStitched	ENSG00000106617	PRKAG2	35084.84
18_S26_peak_19561_lociStitched	ENSG00000262003	LOC101927727	34993.71
8_S26_peak_1943_lociStitched	ENSG00000265822	MIR4422	34112.88
6_S26_peak_6681_lociStitched		LOC102723703	33286.44
5_S26_peak_38579_lociStitched	ENSG00000197603	CPLANE1	32039.24
6_S26_peak_4114_lociStitched	ENSG00000116833	NR5A2	35742.38
5_S26_peak_19096_lociStitched	ENSG00000140836	ZFHX3	32126.38
4_S26_peak_31585_lociStitched	ENSG00000160209	PDXK	33582.28
9_S26_peak_48981_lociStitched	ENSG00000099810	MTAP	31623.26
5_S26_peak_20485_lociStitched		LOC646030	33509.61
17_S26_peak_20359_lociStitched	ENSG00000168961	LGALS9	38972.27
4_S26_peak_4696_lociStitched	ENSG00000234754	C1orf140	33124.6
8_S26_peak_43083_lociStitched	ENSG00000047936	ROS1	32383.95
4_S26_peak_25268_lociStitched	ENSG00000105281	SLC1A5	31723.69
2_S26_peak_38311_lociStitched	ENSG00000164236	ANKRD33B	32672.07
11_S26_peak_46644_lociStitched	ENSG00000164733	CTSB	36664.6
3_S26_peak_47190_lociStitched		LINC01605	31876.04
12_S26_peak_565_lociStitched	ENSG00000142634	EFHD2	33064.76
14_S26_peak_25239_lociStitched		IGFL2-AS1	33975.29
12_S26_peak_38189_lociStitched	ENSG00000113504	SLC12A7	37434.02
10_S26_peak_36196_lociStitched	ENSG00000132405	TBC1D14	34570.73
2_S26_peak_30853_lociStitched	ENSG00000124225	PMEPA1	32664.95
12_S26_peak_36950_lociStitched	ENSG00000174808	BTC	33234.08
3_S26_peak_20968_lociStitched	ENSG00000005102	MEOX1	32969.78
7_S26_peak_47325_lociStitched	ENSG00000164808	SPIDR	36512.01
7_S26_peak_48591_lociStitched	ENSG00000123908	AGO2	35772.61
13_S26_peak_25176_lociStitched	ENSG00000284544	MIR330	34479
5_S26_peak_31266_lociStitched	ENSG00000159216	RUNX1	35024.22
2_S26_peak_7741_lociStitched	ENSG00000184363	PKP3	32376.84
18_S26_peak_38738_lociStitched	ENSG00000134363	FST	34065.06
7_S26_peak_3509_lociStitched	ENSG00000158710	TAGLN2	32998.67
S26_peak_11258	ENSG00000135480	KRT7	32388.75
12_S26_peak_17040_lociStitched	ENSG00000138621	PPCDC	39491.57
10_S26_peak_5402_lociStitched	ENSG00000230573	LOC105376365	33428.64
2_S26_peak_50922_lociStitched	ENSG00000186350	RXRA	34125.22
12_S26_peak_51590_lociStitched	ENSG00000207725	MIR222	33014.67
5_S26_peak_35804_lociStitched	ENSG00000114315	HES1	33303.51
6_S26_peak_32796_lociStitched	ENSG00000280424	LOC730668	32778.77
11_S26_peak_3950_lociStitched	ENSG00000162704	ARPC5	34274.99
4_S26_peak_48347_lociStitched	ENSG00000136997	MYC	33058.02
6_S26_peak_13540_lociStitched	ENSG00000180332	KCTD4	37512.46
12_S26_peak_21630_lociStitched	ENSG00000108854	SMURF2	38163.61

4_S26_peak_14864_lociStitched	ENSG00000214900	LINC01588	33514.21
S26_peak_25134	ENSG00000012061	ERCC1	32295.84
5_S26_peak_24822_lociStitched	ENSG00000197019	SERTAD1	33871.13
9_S26_peak_34201_lociStitched	ENSG00000136068	FLNB	33372.42
6_S26_peak_22787_lociStitched		LINC01543	32875.91
3_S26_peak_42880_lociStitched	ENSG00000112246	SIM1	34405.83
9_S26_peak_39117_lociStitched	ENSG00000248942	LINC01335	35085.82
5_S26_peak_29560_lociStitched	ENSG00000063660	GPC1	34388.48
4_S26_peak_4310_lociStitched	ENSG00000143850	PLEKHA6	33498.98
1_S26_peak_7859_lociStitched	ENSG00000149043	SYT8	33035.43
9_S26_peak_24204_lociStitched	ENSG00000132002	DNAJB1	33489.42
1_S26_peak_10823_lociStitched	ENSG00000235884	LINC00941	32886.99
4_S26_peak_28769_lociStitched	ENSG00000226312	CFLAR-AS1	33151.13
1_S26_peak_10601_lociStitched	ENSG00000111305	GSG1	32919.94
2_S26_peak_8336_lociStitched	ENSG00000205177	C11orf91	33097.85
7_S26_peak_33715_lociStitched	ENSG00000182606	TRAK1	35859.23
12_S26_peak_15036_lociStitched	ENSG00000100592	DAAM1	34115.81
6_S26_peak_35110_lociStitched	ENSG00000169908	TM4SF1	36839.12
3_S26_peak_44196_lociStitched	ENSG00000264357	MIR4648	33415.2
2_S26_peak_43598_lociStitched	ENSG00000055208	TAB2	33409.93
5_S26_peak_38339_lociStitched	ENSG00000039139	DNAH5	36758.48
6_S26_peak_29261_lociStitched	ENSG00000226125	LINC01907	33778.73
S26_peak_10588	ENSG00000013588	GPRC5A	33467.49
19_S26_peak_47105_lociStitched	ENSG00000253490	LINC02099	39441.08
11_S26_peak_34585_lociStitched		SNORD155	35295.01
2_S26_peak_36493_lociStitched	ENSG00000248685	LINC02484	33361.29
3_S26_peak_29409_lociStitched	ENSG00000130147	SH3BP4	33660.71
13_S26_peak_50874_lociStitched	ENSG00000160293	VAV2	36024.59
4_S26_peak_8006_lociStitched	ENSG00000205339	IPO7	33944.36
7_S26_peak_1960_lociStitched	ENSG00000184292	TACSTD2	35631.67
8_S26_peak_27595_lociStitched	ENSG00000170485	NPAS2	35253.84
4_S26_peak_18726_lociStitched	ENSG00000125148	MT2A	33913.65
9_S26_peak_46886_lociStitched	ENSG00000253837	LOC100507156	34793.64
9_S26_peak_307_lociStitched	ENSG00000171735	CAMTA1	35580.82
9_S26_peak_50684_lociStitched		LINC00963	36516.82
17_S26_peak_15251_lociStitched		LOC100996664	37023.93
7_S26_peak_10044_lociStitched	ENSG00000137699	TRIM29	35460.66
2_S26_peak_11054_lociStitched	ENSG00000079337	RAPGEF3	34829.12
8_S26_peak_561_lociStitched	ENSG00000233485	LOC101927417	35658.97
15_S26_peak_3736_lociStitched	ENSG00000120337	TNFSF18	42151.27
3_S26_peak_26963_lociStitched	ENSG00000028116	VRK2	35239.09
4_S26_peak_30590_lociStitched	ENSG00000233077	LINC01271	34989.85
S26_peak_34957	ENSG00000240006	LINC02004	34986.13
5_S26_peak_20101_lociStitched	ENSG00000221355	MIR1288	36233.52

11_S26_peak_5386_lociStitched	ENSG00000067082	KLF6	35552.17
S26_peak_10852	ENSG00000151743	AMN1	34800.62
9_S26_peak_40340_lociStitched	ENSG00000145901	TNIP1	37139.26
11_S26_peak_44320_lociStitched	ENSG00000136238	RAC1	37179.79
S26_peak_49176	ENSG00000275651	MIR6851	35344.31
3_S26_peak_23747_lociStitched	ENSG00000105355	PLIN3	34814.52
10_S26_peak_6230_lociStitched	ENSG00000156510	HKDC1	38247.75
5_S26_peak_8053_lociStitched	ENSG00000283813	MIR4485	35467.95
10_S26_peak_32693_lociStitched	ENSG00000100290	BIK	38368.86
4_S26_peak_22298_lociStitched	ENSG00000207736	MIR657	35588.36
10_S26_peak_46568_lociStitched	ENSG00000253958	CLDN23	39670.02
S26_peak_30654	ENSG00000234693	LOC100506175	34843.84
13_S26_peak_27176_lociStitched	ENSG00000244617	ASPRV1	41086.6
3_S26_peak_24244_lociStitched	ENSG00000214049	UCA1	34784.56
S26_peak_34013	ENSG00000012171	SEMA3B	35610
7_S26_peak_43167_lociStitched	ENSG00000111912	NCOA7	36835.17
13_S26_peak_40259_lociStitched	ENSG00000207714	MIR584	38589.61
1_S26_peak_9736_lociStitched	ENSG00000137693	YAP1	34648.63
10_S26_peak_8991_lociStitched	ENSG00000162241	SLC25A45	38142.54
4_S26_peak_3806_lociStitched	ENSG00000075391	RASAL2	35612.8
9_S26_peak_43271_lociStitched	ENSG00000112319	EYA4	37104.72
S26_peak_12735	ENSG00000139725	RHOF	35592.45
9_S26_peak_2786_lociStitched	ENSG00000197323	TRIM33	37035
2_S26_peak_8042_lociStitched	ENSG00000148926	ADM	35508.25
4_S26_peak_27608_lociStitched	ENSG00000204634	TBC1D8	37253.99
3_S26_peak_856_lociStitched	ENSG00000117318	ID3	35495.18
9_S26_peak_32318_lociStitched	ENSG00000166897	ELFN2	37172.93
6_S26_peak_24970_lociStitched		PRG1	36054.81
16_S26_peak_43369_lociStitched	ENSG00000118503	TNFAIP3	40468.38
10_S26_peak_47941_lociStitched	ENSG00000248599	FLJ42969	39663.51
3_S26_peak_5037_lociStitched	ENSG00000135778	NTPCR	36184.31
S26_peak_12737	ENSG00000212694	LINC01089	35644.84
2_S26_peak_10383_lociStitched	ENSG00000111319	SCNN1A	37007.12
2_S26_peak_17669_lociStitched	ENSG00000102854	MSLN	35729.89
1_S26_peak_35287_lociStitched		IQCJ-SCHIP1-AS1	35826.78
6_S26_peak_34744_lociStitched	ENSG00000144908	ALDH1L1	38551.25
13_S26_peak_23851_lociStitched	ENSG00000125726	CD70	40021.64
4_S26_peak_27701_lociStitched	ENSG00000144063	MALL	36849.09
2_S26_peak_4104_lociStitched	ENSG00000231718	LOC400800	36963.56
10_S26_peak_32363_lociStitched	ENSG00000100106	TRIOBP	37139.17
11_S26_peak_23363_lociStitched	ENSG00000167468	GPX4	40365.56
5_S26_peak_40916_lociStitched		LINC01011	38950.47
4_S26_peak_11345_lociStitched	ENSG00000185591	SP1	38769.59
9_S26_peak_979_lociStitched	ENSG00000142669	SH3BGRL3	39008.19

6_S26_peak_15198_lociStitched	ENSG00000182185	RAD51B	38880.08
8_S26_peak_33652_lociStitched	ENSG00000172939	OXSR1	38366.7
2_S26_peak_24694_lociStitched	ENSG00000167644	C19orf33	37019.78
4_S26_peak_42039_lociStitched	ENSG00000007866	TEAD3	37473.28
7_S26_peak_51579_lociStitched	ENSG00000229563	LINC01204	37525.03
5_S26_peak_30761_lociStitched	ENSG00000171940	ZNF217	38310.43
2_S26_peak_5102_lociStitched	ENSG00000227630	LINC01132	37774.89
9_S26_peak_31438_lociStitched	ENSG00000232401	LINC00112	41014.19
6_S26_peak_44093_lociStitched	ENSG00000105963	ADAP1	41360.75
8_S26_peak_3819_lociStitched	ENSG00000213057	C1orf220	39278.5
3_S26_peak_40758_lociStitched	ENSG00000196923	PDLIM7	38690.98
10_S26_peak_26782_lociStitched	ENSG00000180398	MCFD2	42695.78
7_S26_peak_17343_lociStitched	ENSG00000185033	SEMA4B	41406.49
5_S26_peak_34785_lociStitched	ENSG00000114554	PLXNA1	38782.33
7_S26_peak_29036_lociStitched	ENSG00000135924	DNAJB2	38894.66
8_S26_peak_35779_lociStitched	ENSG00000180611	MB21D2	39183.09
6_S26_peak_7467_lociStitched	ENSG00000265719	MIR4681	38939.51
12_S26_peak_32851_lociStitched	ENSG00000075275	CELSR1	41402.07
6_S26_peak_3173_lociStitched	ENSG00000159445	THEM4	38509.13
4_S26_peak_26394_lociStitched	ENSG00000229951	FLJ31356	39925.69
9_S26_peak_30448_lociStitched		LINC01754	39260.16
8_S26_peak_24481_lociStitched	ENSG00000105717	PBX4	42064.22
8_S26_peak_3402_lociStitched	ENSG00000116584	ARHGEF2	41091.63
4_S26_peak_11144_lociStitched	ENSG00000050405	LIMA1	39696.01
4_S26_peak_6123_lociStitched	ENSG00000150347	ARID5B	38372.49
6_S26_peak_8486_lociStitched	ENSG00000157613	CREB3L1	39440.3
5_S26_peak_42728_lociStitched	ENSG00000146242	TPBG	39288.14
8_S26_peak_47361_lociStitched	ENSG00000253455	LOC101929217	39688.98
12_S26_peak_45905_lociStitched	ENSG00000105976	MET	45518.84
9_S26_peak_20644_lociStitched	ENSG00000002834	LASP1	42811.44
17_S26_peak_5860_lociStitched	ENSG00000150093	ITGB1	42489.74
3_S26_peak_30572_lociStitched	ENSG00000277449	CEBPB-AS1	40581.89
S26_peak_49174	ENSG00000165272	AQP3	39798.89
6_S26_peak_33227_lociStitched	ENSG00000088726	TMEM40	40299.47
3_S26_peak_2053_lociStitched	ENSG00000079739	PGM1	40115.47
5_S26_peak_12317_lociStitched	ENSG00000264295	MIR3922	39536.66
15_S26_peak_2160_lociStitched	ENSG00000116717	GADD45A	44764.61
4_S26_peak_1227_lociStitched	ENSG00000142910	TINAGL1	40353.2
8_S26_peak_29380_lociStitched	ENSG00000072080	SPP2	40255.83
12_S26_peak_26646_lociStitched		LINC02580	42540.71
8_S26_peak_34625_lociStitched	ENSG00000082701	GSK3B	45439.07
8_S26_peak_12167_lociStitched	ENSG00000257150	PGAM1P5	44614.85
3_S26_peak_3926_lociStitched	ENSG00000224468	LAMC1-AS1	39967.31
21_S26_peak_20173_lociStitched	ENSG00000108557	RAI1	49306.53

9_S26_peak_19226_lociStitched	ENSG00000140945	CDH13	42865.79
6_S26_peak_34815_lociStitched	ENSG00000074416	MGLL	44516.68
28_S26_peak_12908_lociStitched	ENSG00000196498	NCOR2	48730.31
6_S26_peak_12122_lociStitched	ENSG00000258274	LOC101928731	41684.87
7_S26_peak_11057_lociStitched	ENSG00000061273	HDAC7	43172.32
5_S26_peak_7012_lociStitched	ENSG00000155252	PI4K2A	40175.35
5_S26_peak_35126_lociStitched	ENSG00000018408	WWTR1	41593.41
4_S26_peak_4127_lociStitched	ENSG00000162702	ZNF281	42201.21
5_S26_peak_28325_lociStitched	ENSG00000236651	DLX2-DT	40500.63
14_S26_peak_31896_lociStitched	ENSG00000206069	TMEM211	45097.09
6_S26_peak_31197_lociStitched	ENSG00000159128	IFNGR2	40756.62
2_S26_peak_11465_lociStitched	ENSG00000076067	RBMS2	41084.96
3_S26_peak_14128_lociStitched	ENSG00000198542	ITGBL1	40714.45
18_S26_peak_19461_lociStitched	ENSG00000103335	PIEZO1	42306.92
S26_peak_10856	ENSG00000151743	AMN1	40169.83
7_S26_peak_50656_lociStitched	ENSG00000119383	PTPA	41372.64
S26_peak_33889	ENSG00000114270	COL7A1	40253.11
10_S26_peak_44935_lociStitched	ENSG00000136205	TNS3	43472.97
13_S26_peak_26522_lociStitched	ENSG00000150938	CRIM1	46668.69
4_S26_peak_33362_lociStitched	ENSG00000169814	BTD	42828.08
1_S26_peak_39664_lociStitched	ENSG00000145779	TNFAIP8	40671.77
5_S26_peak_43964_lociStitched	ENSG00000112541	PDE10A	40990.82
8_S26_peak_25870_lociStitched	ENSG00000151693	ASAP2	43381.07
9_S26_peak_22319_lociStitched	ENSG00000185332	TMEM105	42671.31
S26_peak_25128	ENSG00000104881	PPP1R13L	40489.34
9_S26_peak_13774_lociStitched	ENSG00000102554	KLF5	48315.98
S26_peak_41950	ENSG00000197251	LINC00336	40398.13
5_S26_peak_47776_lociStitched	ENSG00000284288	MIR378D2	43018.49
1_S26_peak_17893_lociStitched	ENSG00000006327	TNFRSF12A	40317.97
S26_peak_10988		LOC100288798	40677.19
5_S26_peak_44969_lociStitched	ENSG00000183696	UPP1	42373.83
5_S26_peak_35173_lociStitched	ENSG00000138271	GPR87	42922.6
5_S26_peak_8697_lociStitched	ENSG00000134780	DAGLA	42231.1
5_S26_peak_13555_lociStitched		TPT1-AS1	44513.42
5_S26_peak_4582_lociStitched	ENSG00000117691	NENF	42753.87
7_S26_peak_20561_lociStitched	ENSG00000277268	LHX1-DT	42293.14
6_S26_peak_21569_lociStitched	ENSG00000173838	10-Mar	43118.83
8_S26_peak_4255_lociStitched	ENSG00000188770	OPTC	43651.24
19_S26_peak_26177_lociStitched	ENSG00000143878	RHOB	47136.09
14_S26_peak_38361_lociStitched	ENSG00000038382	TRIO	50648.76
8_S26_peak_44276_lociStitched	ENSG00000075618	FSCN1	43744.08
6_S26_peak_359_lociStitched	ENSG00000116285	ERRFI1	42805.66
22_S26_peak_6619_lociStitched	ENSG00000108179	PPIF	49427.06
9_S26_peak_40135_lociStitched	ENSG00000231185	SPRY4-AS1	44805.55

10_S26_peak_24385_lociStitched	ENSG00000267959	MIR3188	44133.81
9_S26_peak_21696_lociStitched	ENSG00000207688	MIR548AA2	44748.59
11_S26_peak_30019_lociStitched	ENSG00000230613	HM13-AS1	46231.86
9_S26_peak_42212_lociStitched	ENSG00000048544	MRPS10	45781.48
10_S26_peak_28746_lociStitched	ENSG00000082153	BZW1	45686.4
4_S26_peak_3125_lociStitched	ENSG00000143398	PIP5K1A	42264.15
12_S26_peak_47466_lociStitched	ENSG00000035681	NSMAF	45569.44
22_S26_peak_196_lociStitched	ENSG00000130762	ARHGEF16	45399.89
9_S26_peak_23755_lociStitched	ENSG00000263409	MIR4747	45178.84
4_S26_peak_47919_lociStitched	ENSG00000164924	YWHAZ	42856.72
18_S26_peak_17404_lociStitched	ENSG00000176463	SLCO3A1	46175.91
4_S26_peak_36556_lociStitched	ENSG00000231160	KLF3-AS1	42905.48
6_S26_peak_1402_lociStitched	ENSG00000196182	STK40	43682.5
16_S26_peak_49842_lociStitched		LOC101928119	50213.84
7_S26_peak_17756_lociStitched	ENSG00000265820	MIR3177	43849.64
5_S26_peak_19244_lociStitched	ENSG00000140950	TLDC1	43011.96
8_S26_peak_9384_lociStitched	ENSG00000186635	ARAP1	45516.45
9_S26_peak_50738_lociStitched	ENSG00000148344	PTGES	49342.32
11_S26_peak_3226_lociStitched	ENSG00000197956	S100A6	45919.23
10_S26_peak_4590_lociStitched	ENSG00000162772	ATF3	49103.74
5_S26_peak_40203_lociStitched	ENSG00000266478	MIR5197	43874.77
2_S26_peak_51175_lociStitched	ENSG00000203993	ARRDC1-AS1	43636.29
13_S26_peak_5129_lociStitched	ENSG00000238005	LOC101927851	49852.05
6_S26_peak_43239_lociStitched	ENSG00000146376	ARHGAP18	44408.92
9_S26_peak_36408_lociStitched	ENSG00000109819	PPARGC1A	44943.98
2_S26_peak_45873_lociStitched	ENSG00000237870	LOC102724434	43940.85
1_S26_peak_31252_lociStitched	ENSG00000159216	RUNX1	43029.13
12_S26_peak_41369_lociStitched	ENSG00000124788	ATXN1	46871.01
7_S26_peak_1508_lociStitched	ENSG00000179862	CITED4	44645.01
2_S26_peak_43414_lociStitched	ENSG00000164442	CITED2	43796.56
S26_peak_11239	ENSG00000167767	KRT80	44317.42
4_S26_peak_20322_lociStitched	ENSG00000034152	MAP2K3	44025.35
3_S26_peak_38364_lociStitched	ENSG00000038382	TRIO	44396.98
S26_peak_10578	ENSG00000234498	RPL13AP20	43339.01
11_S26_peak_20068_lociStitched	ENSG00000221926	TRIM16	44957.74
22_S26_peak_21774_lociStitched	ENSG00000264026	LINC02003	50129.19
8_S26_peak_26352_lociStitched	ENSG00000205334	LINC01460	47612.7
8_S26_peak_21_lociStitched	ENSG00000188157	AGRN	45409.94
8_S26_peak_30827_lociStitched	ENSG00000087510	TFAP2C	45923.81
8_S26_peak_12937_lociStitched	ENSG00000196498	NCOR2	46773.05
8_S26_peak_16935_lociStitched	ENSG00000067225	PKM	45807.58
S26_peak_9161	ENSG00000173237	C11orf86	45099.39
7_S26_peak_9024_lociStitched	ENSG00000168056	LTBP3	46293.52
7_S26_peak_46834_lociStitched	ENSG00000120913	PDLIM2	46555.86

6_S26_peak_48253_lociStitched	ENSG00000173334	TRIB1	47968.48
6_S26_peak_27063_lociStitched	ENSG00000234572	LINC01800	45206.62
8_S26_peak_12412_lociStitched	ENSG00000207622	MIR619	45847.27
S26_peak_6727		AGAP11	44411.95
4_S26_peak_41389_lociStitched		LOC101928433	46997.76
8_S26_peak_45020_lociStitched	ENSG00000146648	EGFR	48903.9
6_S26_peak_29699_lociStitched	ENSG00000275491	LINC01730	45437.15
8_S26_peak_35317_lociStitched	ENSG00000179674	ARL14	46306.27
8_S26_peak_17093_lociStitched	ENSG00000173548	SNX33	47498.12
8_S26_peak_44078_lociStitched	ENSG00000164828	SUN1	48217.28
7_S26_peak_44858_lociStitched	ENSG00000105953	OGDH	45700.08
S26_peak_41725	ENSG00000146112	PPP1R18	45950.34
17_S26_peak_39945_lociStitched	ENSG00000120708	TGFBI	56936.51
6_S26_peak_50609_lociStitched	ENSG00000119333	WDR34	46397.37
7_S26_peak_36403_lociStitched	ENSG00000109819	PPARGC1A	46310.4
5_S26_peak_14427_lociStitched	ENSG00000185989	RASA3	45641.18
S26_peak_41756	ENSG00000228022	HCG20	45654.93
6_S26_peak_4925_lociStitched	ENSG00000198835	GJC2	47452.05
7_S26_peak_49257_lociStitched	ENSG00000198853	RUSC2	47116.88
S26_peak_40620	ENSG00000037241	RPL26L1	45832.86
6_S26_peak_6665_lociStitched	ENSG00000122359	ANXA11	47599.95
5_S26_peak_15266_lociStitched	ENSG00000185650	ZFP36L1	46371.28
1_S26_peak_41908_lociStitched	ENSG00000204256	BRD2	45471.04
5_S26_peak_46857_lociStitched	ENSG00000008853	RHOBTB2	47059.6
4_S26_peak_11176_lociStitched	ENSG00000123268	ATF1	48203.39
12_S26_peak_46107_lociStitched	ENSG00000128585	MKLN1	49944.15
13_S26_peak_1028_lociStitched	ENSG00000175793	SFN	48469.34
S26_peak_23393	ENSG00000099622	CIRBP	45941.7
2_S26_peak_48722_lociStitched	ENSG00000204839	MROH6	46765.14
4_S26_peak_40795_lociStitched	ENSG00000161011	SQSTM1	46729.65
10_S26_peak_20834_lociStitched	ENSG00000173801	JUP	48487.61
9_S26_peak_32274_lociStitched	ENSG00000100348	TXN2	47730.16
3_S26_peak_33272_lociStitched	ENSG00000154764	WNT7A	46997.72
9_S26_peak_36236_lociStitched	ENSG00000196526	AFAP1	48524.8
6_S26_peak_11863_lociStitched	ENSG00000139289	PHLDA1	47884.11
11_S26_peak_46545_lociStitched	ENSG00000173295	FAM86B3P	49899.14
8_S26_peak_42631_lociStitched	ENSG00000156508	EEF1A1	49272.04
8_S26_peak_26143_lociStitched	ENSG00000115884	SDC1	49872.5
9_S26_peak_15844_lociStitched	ENSG00000185215	TNFAIP2	49812.79
6_S26_peak_7015_lociStitched	ENSG00000119986	AVPI1	48453.66
12_S26_peak_15803_lociStitched	ENSG00000266015	MIR4309	49829.35
6_S26_peak_23681_lociStitched	ENSG00000178951	ZBTB7A	48505.3
5_S26_peak_22416_lociStitched	ENSG00000176155	CCDC57	48125.66
19_S26_peak_15298_lociStitched		SNORD169	53004.19

11_S26_peak_47029_lociStitched	ENSG00000186918	ZNF395	51367.73
11_S26_peak_32497_lociStitched	ENSG00000209480	SNORD83B	50533.61
10_S26_peak_22430_lociStitched	ENSG00000265692	LINC01970	50756.54
9_S26_peak_23885_lociStitched		LOC100128573	52053.62
7_S26_peak_6314_lociStitched	ENSG00000107738	VSIR	50622.16
2_S26_peak_14522_lociStitched	ENSG00000129474	AJUBA	48101.79
11_S26_peak_23628_lociStitched	ENSG00000161091	MFSD12	50207.69
6_S26_peak_606_lociStitched	ENSG00000233954	UQCRHL	48889.02
8_S26_peak_48654_lociStitched	ENSG00000105339	DENND3	52574.38
4_S26_peak_41759_lociStitched	ENSG00000214894	LINC00243	51293.29
1_S26_peak_30447_lociStitched	ENSG00000101040	ZMYND8	48708.52
7_S26_peak_30772_lociStitched	ENSG00000235415	LOC105372672	49648.7
19_S26_peak_25029_lociStitched	ENSG00000105771	SMG9	53546.3
2_S26_peak_83_lociStitched	ENSG00000284372	MIR6808	48714.24
8_S26_peak_40044_lociStitched	ENSG00000113068	PFDN1	51366.21
8_S26_peak_21124_lociStitched	ENSG00000159314	ARHGAP27	50430.1
S26_peak_3323	ENSG00000160691	SHC1	48510.06
11_S26_peak_18261_lociStitched	ENSG00000205730	ITPRIPL2	52689.53
5_S26_peak_50161_lociStitched	ENSG00000165181	C9orf84	50767.79
7_S26_peak_31386_lociStitched		LOC400867	49924.95
8_S26_peak_48645_lociStitched	ENSG00000105339	DENND3	52235.13
10_S26_peak_48972_lociStitched		MIR31HG	51710.75
6_S26_peak_51354_lociStitched	ENSG00000265465	MIR4768	50119.11
6_S26_peak_47498_lociStitched	ENSG00000264408	MIR4470	52626.37
13_S26_peak_14321_lociStitched		LINC00431	54130.29
9_S26_peak_48294_lociStitched	ENSG00000168672	FAM84B	53724.87
8_S26_peak_30001_lociStitched	ENSG00000100994	PYGB	51332.89
4_S26_peak_7057_lociStitched	ENSG00000023839	ABCC2	51686.83
12_S26_peak_50539_lociStitched	ENSG00000167106	FAM102A	53907.25
8_S26_peak_25970_lociStitched	ENSG00000115756	HPCAL1	52284.32
11_S26_peak_11310_lociStitched	ENSG00000170421	KRT8	54773.86
4_S26_peak_7829_lociStitched	ENSG00000215182	MUC5AC	50200.1
8_S26_peak_21395_lociStitched	ENSG00000121060	TRIM25	52148
10_S26_peak_13804_lociStitched		LINC00392	57042.66
8_S26_peak_21921_lociStitched	ENSG00000092929	UNC13D	52458.41
6_S26_peak_21497_lociStitched	ENSG00000284190	MIR21	51801.44
13_S26_peak_32128_lociStitched	ENSG00000241878	PISD	55001.14
6_S26_peak_12154_lociStitched	ENSG00000180263	FGD6	52287.65
2_S26_peak_41825_lociStitched	ENSG00000213719	CLIC1	51457.01
1_S26_peak_15924_lociStitched	ENSG00000185567	AHNAK2	50901.63
14_S26_peak_25455_lociStitched	ENSG00000087086	FTL	54345.27
8_S26_peak_6203_lociStitched	ENSG00000138347	MYPN	53925.5
10_S26_peak_15902_lociStitched	ENSG00000203485	INF2	51936.24
7_S26_peak_42188_lociStitched	ENSG00000048544	MRPS10	53515.59

3_S26_peak_27390_lociStitched	ENSG00000034510	TMSB10	51314.48
9_S26_peak_1652_lociStitched	ENSG00000178922	HYI	52678.82
16_S26_peak_30251_lociStitched	ENSG00000198959	TGM2	65793.46
6_S26_peak_36916_lociStitched	ENSG00000081041	CXCL2	52211.47
4_S26_peak_48663_lociStitched	ENSG00000022567	SLC45A4	53492.38
3_S26_peak_48634_lociStitched	ENSG00000105339	DENND3	52673.57
17_S26_peak_21508_lociStitched	ENSG00000284190	MIR21	54499.9
12_S26_peak_37573_lociStitched	ENSG00000151012	SLC7A11	53483.39
S26_peak_51088	ENSG00000266507	MIR4479	52528.26
5_S26_peak_46422_lociStitched		LOC389602	53150.78
12_S26_peak_18208_lociStitched	ENSG00000284184	MIR6506	57813.42
3_S26_peak_48801_lociStitched	ENSG00000160948	VPS28	52805.77
9_S26_peak_46062_lociStitched		LOC105375504	54370.51
6_S26_peak_30968_lociStitched	ENSG00000101187	SLCO4A1	55244.91
12_S26_peak_4227_lociStitched	ENSG00000077157	PPP1R12B	56694.12
12_S26_peak_46123_lociStitched	ENSG00000128567	PODXL	58206.75
S26_peak_41751	ENSG00000228022	HCG20	53079.24
11_S26_peak_15663_lociStitched	ENSG00000205476	CCDC85C	56102.59
19_S26_peak_32328_lociStitched	ENSG00000100065	CARD10	56633.54
19_S26_peak_20618_lociStitched	ENSG00000275832	ARHGAP23	56054.38
5_S26_peak_21098_lociStitched	ENSG00000184922	FMNL1	53607.47
7_S26_peak_17422_lociStitched		ASB9P1	56248.61
18_S26_peak_2109_lociStitched	ENSG00000184588	PDE4B	58265.9
17_S26_peak_6283_lociStitched	ENSG00000237512	UNC5B-AS1	56563.91
12_S26_peak_51147_lociStitched	ENSG00000198435	NRARP	57980.99
10_S26_peak_45889_lociStitched	ENSG00000105971	CAV2	58820.86
6_S26_peak_39441_lociStitched	ENSG00000175426	PCSK1	57273.28
14_S26_peak_17738_lociStitched	ENSG00000138834	MAPK8IP3	55829.95
15_S26_peak_33791_lociStitched	ENSG00000163814	CDCP1	55855.78
4_S26_peak_30959_lociStitched	ENSG00000101187	SLCO4A1	53824.39
5_S26_peak_44951_lociStitched	ENSG00000146666	LINC00525	55017.12
3_S26_peak_50322_lociStitched	ENSG00000235865	GSN-AS1	54970.31
8_S26_peak_18116_lociStitched	ENSG00000189067	LITAF	58322.95
10_S26_peak_26438_lociStitched	ENSG00000115295	CLIP4	57095.6
2_S26_peak_29014_lociStitched	ENSG00000135925	WNT10A	53975.23
11_S26_peak_22117_lociStitched	ENSG00000178404	CEP295NL	57385.71
22_S26_peak_43678_lociStitched	ENSG00000120254	MTHFD1L	63644.33
19_S26_peak_3909_lociStitched	ENSG00000162783	IER5	65800.22
9_S26_peak_20224_lociStitched	ENSG00000177731	FLII	58836.49
6_S26_peak_35713_lociStitched	ENSG00000216058	MIR944	56718.61
9_S26_peak_20700_lociStitched	ENSG00000126368	NR1D1	56529.66
9_S26_peak_30389_lociStitched	ENSG00000124145	SDC4	58426.57
10_S26_peak_3089_lociStitched	ENSG00000143420	ENSA	58406.92
17_S26_peak_23538_lociStitched	ENSG00000099860	GADD45B	58008.7

6_S26_peak_47924_lociStitched	ENSG00000248599	FLJ42969	59233.85
6_S26_peak_31645_lociStitched	ENSG00000275874	PICSAR	57612.47
6_S26_peak_45899_lociStitched	ENSG00000105974	CAV1	57938.44
7_S26_peak_2557_lociStitched	ENSG00000263526	MIR378G	57807.71
8_S26_peak_4315_lociStitched	ENSG00000158615	PPP1R15B	57819.07
22_S26_peak_30619_lociStitched	ENSG00000196396	PTPN1	63519.04
6_S26_peak_23708_lociStitched	ENSG00000141985	SH3GL1	59209.98
14_S26_peak_9564_lociStitched	ENSG00000150687	PRSS23	59178.26
2_S26_peak_19138_lociStitched	ENSG00000050820	BCAR1	56903.35
13_S26_peak_30559_lociStitched	ENSG00000240849	TMEM189	62764.87
10_S26_peak_11602_lociStitched	ENSG00000196935	SRGAP1	57835.72
26_S26_peak_31363_lociStitched	ENSG00000157557	ETS2	63033.8
S26_peak_32032	ENSG00000128342	LIF	57546.71
8_S26_peak_32413_lociStitched		SNORA92	59249.35
7_S26_peak_21802_lociStitched	ENSG00000179604	CDC42EP4	60157.28
3_S26_peak_5096_lociStitched	ENSG00000228044	LOC101927787	57392.49
4_S26_peak_16905_lociStitched		THSD4-AS1	57425.7
11_S26_peak_10369_lociStitched	ENSG00000067182	TNFRSF1A	61571.37
10_S26_peak_7863_lociStitched	ENSG00000265258	MIR4686	61210.13
S26_peak_41744	ENSG00000137331	IER3	58153.39
S26_peak_12987	ENSG00000265345	MIR5188	57676.12
11_S26_peak_7081_lociStitched	ENSG00000235823	OLMALINC	61115.15
8_S26_peak_26284_lociStitched	ENSG00000213699	SLC35F6	61036.58
18_S26_peak_46802_lociStitched	ENSG00000168487	BMP1	64466.1
7_S26_peak_1732_lociStitched	ENSG00000188396	TCTEX1D4	58925.81
18_S26_peak_17964_lociStitched	ENSG00000126603	GLIS2	61836.72
12_S26_peak_44063_lociStitched	ENSG00000223855	HRAT92	61514.37
15_S26_peak_40485_lociStitched	ENSG00000113645	WWC1	66168.47
7_S26_peak_24059_lociStitched	ENSG00000161888	SPC24	59435.15
10_S26_peak_992_lociStitched	ENSG00000176092	CRYBG2	60649.2
13_S26_peak_16584_lociStitched	ENSG00000182718	ANXA2	62117.7
4_S26_peak_49127_lociStitched	ENSG00000233554	B4GALT1-AS1	60368.62
7_S26_peak_1352_lociStitched	ENSG00000188910	GJB3	60865.63
18_S26_peak_2820_lociStitched	ENSG00000134198	TSPAN2	61396.61
12_S26_peak_5141_lociStitched		LINC01348	62323.64
4_S26_peak_48684_lociStitched	ENSG00000167653	PSCA	62668.98
9_S26_peak_13995_lociStitched	ENSG00000231674	LINC00410	60695.73
10_S26_peak_15439_lociStitched	ENSG00000258602	LINC01629	60543.26
7_S26_peak_7913_lociStitched	ENSG00000181649	PHLDA2	64113.12
21_S26_peak_12953_lociStitched	ENSG00000196498	NCOR2	69232.68
18_S26_peak_20143_lociStitched	ENSG00000133030	MPRIP	64053.73
13_S26_peak_31817_lociStitched	ENSG00000099917	MED15	65013.1
17_S26_peak_50835_lociStitched	ENSG00000160271	RALGDS	66186.07
6_S26_peak_22538_lociStitched		DLGAP1-AS2	60536

13_S26_peak_45175_lociStitched	ENSG00000189143	CLDN4	64036.21
16_S26_peak_16476_lociStitched	ENSG00000069869	NEDD4	68663.95
15_S26_peak_19376_lociStitched		LOC102724467	63235.75
19_S26_peak_5046_lociStitched	ENSG00000265744	MIR4427	66195.73
30_S26_peak_356_lociStitched	ENSG00000116285	ERRFI1	70444.32
20_S26_peak_42321_lociStitched	ENSG00000237686	LOC101929705	64684.82
S26_peak_9244	ENSG00000006534	ALDH3B1	61404.86
29_S26_peak_4479_lociStitched		LINC01698	73418.2
5_S26_peak_33970_lociStitched	ENSG00000164078	MST1R	63056.26
14_S26_peak_45025_lociStitched	ENSG00000146648	EGFR	66260.82
22_S26_peak_4991_lociStitched	ENSG00000143641	GALNT2	67047.43
7_S26_peak_30357_lociStitched	ENSG00000064205	WISP2	64132.57
12_S26_peak_7307_lociStitched	ENSG00000138166	DUSP5	64403.22
7_S26_peak_8731_lociStitched	ENSG00000167996	FTH1	62940.86
5_S26_peak_13797_lociStitched		LINC00392	66371.14
6_S26_peak_6195_lociStitched	ENSG00000138347	MYPN	64750.38
9_S26_peak_19675_lociStitched	ENSG00000070444	MNT	63903.22
8_S26_peak_45203_lociStitched	ENSG00000006704	GTF2IRD1	65723.65
6_S26_peak_47500_lociStitched	ENSG00000264408	MIR4470	63525.1
10_S26_peak_42007_lociStitched	ENSG00000137309	HMGA1	65200.56
16_S26_peak_15238_lociStitched		LOC100996664	67014.21
18_S26_peak_1074_lociStitched	ENSG00000126705	AHDC1	66445.17
S26_peak_49130	ENSG00000122711	SPINK4	64018.07
7_S26_peak_30285_lociStitched		LOC100128988	65408.92
13_S26_peak_26799_lociStitched	ENSG00000180398	MCFD2	67325.44
12_S26_peak_38176_lociStitched	ENSG00000071539	TRIP13	69149.08
3_S26_peak_34074_lociStitched	ENSG00000164086	DUSP7	65522.15
5_S26_peak_17567_lociStitched	ENSG00000183475	ASB7	66187.84
20_S26_peak_22060_lociStitched	ENSG00000184557	SOCS3	71673.56
14_S26_peak_30666_lociStitched	ENSG00000124171	PARD6B	67178.41
6_S26_peak_20426_lociStitched	ENSG00000196535	MYO18A	68166.95
7_S26_peak_40173_lociStitched	ENSG00000230789	ARHGAP26-IT1	68113.59
2_S26_peak_44250_lociStitched	ENSG00000182095	TNRC18	65327.5
13_S26_peak_27942_lociStitched	ENSG00000178171	AMER3	68486.4
14_S26_peak_35227_lociStitched	ENSG00000181467	RAP2B	70866.2
9_S26_peak_26189_lociStitched	ENSG00000231948	HS1BP3-IT1	69810.19
13_S26_peak_41063_lociStitched	ENSG00000216863	LY86-AS1	73799.5
16_S26_peak_8082_lociStitched	ENSG00000254486	LINC02547	74274.6
13_S26_peak_35403_lociStitched	ENSG00000075651	PLD1	74176.32
1_S26_peak_40192_lociStitched	ENSG00000113580	NR3C1	68383.84
11_S26_peak_39028_lociStitched	ENSG00000197822	OCLN	72579.17
25_S26_peak_34515_lociStitched	ENSG00000144827	ABHD10	72807.14
10_S26_peak_1314_lociStitched	ENSG00000162522	KIAA1522	71400.63
5_S26_peak_49464_lociStitched	ENSG00000119125	GDA	69994.23

S26_peak_30943	ENSG00000130702	LAMA5	69454.39
12_S26_peak_35730_lociStitched	ENSG00000216058	MIR944	76753.31
12_S26_peak_22291_lociStitched	ENSG00000175866	BAIAP2	72881.05
22_S26_peak_50198_lociStitched	ENSG00000266315	MIR4668	80022.37
14_S26_peak_20192_lociStitched	ENSG00000072310	SREBF1	73700.17
9_S26_peak_3198_lociStitched	ENSG00000163191	S100A11	71664.8
7_S26_peak_3181_lociStitched	ENSG00000197747	S100A10	73260.71
10_S26_peak_50672_lociStitched	ENSG00000233901	LINC01503	73772.56
9_S26_peak_9177_lociStitched	ENSG00000173156	RHOD	73729.57
14_S26_peak_7226_lociStitched	ENSG00000065618	COL17A1	75990.27
14_S26_peak_7713_lociStitched	ENSG00000151651	ADAM8	73845.88
18_S26_peak_35444_lociStitched	ENSG00000075420	FNDC3B	80903.62
10_S26_peak_5524_lociStitched	ENSG00000225778	PROSER2-AS1	73916.43
20_S26_peak_32951_lociStitched	ENSG00000196576	PLXNB2	75449.02
8_S26_peak_1248_lociStitched	ENSG00000184007	PTP4A2	76234.47
9_S26_peak_40900_lociStitched	ENSG00000266750	MIR4645	79982.93
6_S26_peak_24872_lociStitched	ENSG00000167601	AXL	75769.98
11_S26_peak_24402_lociStitched	ENSG00000130513	GDF15	76948.36
30_S26_peak_33318_lociStitched	ENSG00000131389	SLC6A6	83560.81
19_S26_peak_12882_lociStitched	ENSG00000275967	MIR6880	81458.7
7_S26_peak_15593_lociStitched	ENSG00000258730	ITPK1-AS1	77265.9
17_S26_peak_10575_lociStitched	ENSG00000213782	DDX47	82016.92
12_S26_peak_47567_lociStitched	ENSG00000169085	VXN	78258.14
18_S26_peak_14981_lociStitched	ENSG00000131981	LGALS3	80464.37
4_S26_peak_6028_lociStitched	ENSG00000107984	DKK1	78156.72
5_S26_peak_31039_lociStitched	ENSG00000243509	TNFRSF6B	78193.27
3_S26_peak_3447_lociStitched	ENSG00000116604	MEF2D	77795.7
7_S26_peak_9293_lociStitched	ENSG00000172927	MYEOV	82672.82
7_S26_peak_24147_lociStitched	ENSG00000160888	IER2	80701.41
6_S26_peak_30188_lociStitched	ENSG00000171222	SCAND1	79876.74
7_S26_peak_50402_lociStitched	ENSG00000185585	OLFML2A	82797.66
20_S26_peak_22967_lociStitched	ENSG00000264269	LOC100129878	82768.02
3_S26_peak_41257_lociStitched	ENSG00000111863	ADTRP	79408.4
7_S26_peak_9657_lociStitched	ENSG00000255929	LOC105369438	82144.34
26_S26_peak_388_lociStitched	ENSG00000227634	LINC01714	86606.74
13_S26_peak_18031_lociStitched	ENSG00000118898	PPL	85346.83
3_S26_peak_24119_lociStitched	ENSG00000263800	MIR5684	81599.52
9_S26_peak_50037_lociStitched	ENSG00000136826	KLF4	82895.08
17_S26_peak_16975_lociStitched	ENSG00000277391	MIR6881	87508.02
16_S26_peak_30505_lociStitched	ENSG00000158470	B4GALT5	90027.42
11_S26_peak_6043_lociStitched	ENSG00000231131	LINC01468	86516.91
10_S26_peak_30439_lociStitched	ENSG00000267882	LOC100131496	87299.09
37_S26_peak_32250_lociStitched	ENSG00000100345	MYH9	94588.7
16_S26_peak_8734_lociStitched	ENSG00000167996	FTH1	88001.34

7_S26_peak_6460_lociStitched	ENSG00000122861	PLAU	88132.73
9_S26_peak_10839_lociStitched	ENSG00000255867	DENND5B-AS1	90803.45
13_S26_peak_4738_lociStitched	ENSG00000162909	CAPN2	90598.82
5_S26_peak_39881_lociStitched	ENSG00000251169	LINC01843	88979.91
6_S26_peak_24046_lociStitched	ENSG00000130164	LDLR	87464.8
20_S26_peak_32916_lociStitched	ENSG00000198355	PIM3	90575
8_S26_peak_41340_lociStitched	ENSG00000226673	LINC01108	90324.54
21_S26_peak_32427_lociStitched	ENSG00000213923	CSNK1E	92979.31
8_S26_peak_28055_lociStitched	ENSG00000115919	KYNU	88554.67
19_S26_peak_20758_lociStitched	ENSG00000131746	TNS4	94879.25
9_S26_peak_8992_lociStitched	ENSG00000245532	NEAT1	90180.93
25_S26_peak_11003_lociStitched		LOC100288798	101057.2
S26_peak_30942	ENSG00000228812	LAMA5-AS1	90224.64
8_S26_peak_13793_lociStitched		LINC00392	96099.68
5_S26_peak_3243_lociStitched	ENSG00000196754	S100A2	91269.98
3_S26_peak_3464_lociStitched	ENSG00000143314	MRPL24	91620.32
7_S26_peak_43863_lociStitched	ENSG00000243775	OSTCP1	95731.19
13_S26_peak_14237_lociStitched	ENSG00000236242	MYO16-AS1	96638.66
10_S26_peak_8758_lociStitched	ENSG00000124942	AHNAK	94608.32
16_S26_peak_32073_lociStitched	ENSG00000184792	OSBP2	98173.98
24_S26_peak_31271_lociStitched	ENSG00000159216	RUNX1	104389.2
10_S26_peak_48465_lociStitched	ENSG00000221261	MIR1208	100316.2
21_S26_peak_8942_lociStitched	ENSG00000110047	EHD1	100528.6
13_S26_peak_4414_lociStitched	ENSG00000182795	C1orf116	105585.6
10_S26_peak_31239_lociStitched	ENSG00000234380	LINC01426	103297.7
6_S26_peak_4502_lociStitched	ENSG00000196878	LAMB3	101439.1
5_S26_peak_6593_lociStitched	ENSG00000108175	ZMIZ1	102923
9_S26_peak_5095_lociStitched	ENSG00000168264	IRF2BP2	106591.6
14_S26_peak_2508_lociStitched	ENSG00000230439	MIG7	109196.2
10_S26_peak_49509_lociStitched	ENSG00000135046	ANXA1	107273.8
21_S26_peak_16522_lociStitched	ENSG00000171989	LDHAL6B	110298.9
17_S26_peak_21850_lociStitched	ENSG00000125449	ARMC7	110615.5
16_S26_peak_22014_lociStitched	ENSG00000184640	9-Sep	111388.5
25_S26_peak_11850_lociStitched	ENSG00000139289	PHLDA1	111208.8
5_S26_peak_21903_lociStitched	ENSG00000108469	RECQL5	110100.7
13_S26_peak_9214_lociStitched	ENSG00000175482	POLD4	111994.2
11_S26_peak_21073_lociStitched	ENSG00000277249	MIR6784	114230.1
26_S26_peak_31542_lociStitched		LINC00322	120582.3
10_S26_peak_14049_lociStitched	ENSG00000139793	MBNL2	112709.9
12_S26_peak_50907_lociStitched	ENSG00000186350	RXRA	114197.3
18_S26_peak_23469_lociStitched	ENSG00000099875	MKNK2	113619.5
21_S26_peak_17519_lociStitched	ENSG00000264480	MIR4714	119112.1
23_S26_peak_29627_lociStitched	ENSG00000125826	RBCK1	117528.7
S26_peak_23373	ENSG00000064932	SBNO2	113026.7

5_S26_peak_32809_lociStitched		LOC642648	114196.2
10_S26_peak_8110_lociStitched	ENSG00000275373	MIR6124	115800.4
16_S26_peak_4359_lociStitched	ENSG00000163545	NUAK2	122922.1
34_S26_peak_22266_lociStitched	ENSG00000262833	LOC101928855	122059.2
22_S26_peak_12281_lociStitched	ENSG00000198431	TXNRD1	122545.9
2_S26_peak_11272_lociStitched	ENSG00000205426	KRT81	114266.6
14_S26_peak_21310_lociStitched	ENSG00000108846	ABCC3	121922.4
S26_peak_30056	ENSG00000281376	ABALON	116063.3
18_S26_peak_13854_lociStitched	ENSG00000136153	LMO7	121816.8
18_S26_peak_50482_lociStitched	ENSG00000136830	FAM129B	125180.3
10_S26_peak_9068_lociStitched	ENSG00000175592	FOSL1	120827.4
17_S26_peak_6924_lociStitched	ENSG00000138119	MYOF	125605.3
11_S26_peak_46078_lociStitched	ENSG00000231721	LINC-PINT	123987.4
32_S26_peak_33404_lociStitched	ENSG00000131386	GALNT15	134698.6
6_S26_peak_11022_lociStitched		LOC100288798	126791.5
S26_peak_41181	ENSG00000137203	TFAP2A	126622.8
17_S26_peak_44272_lociStitched	ENSG00000075624	ACTB	137310.7
10_S26_peak_33028_lociStitched	ENSG00000235831	BHLHE40-AS1	133903.6
18_S26_peak_9004_lociStitched	ENSG00000251562	MALAT1	133648.8
16_S26_peak_12681_lociStitched	ENSG00000089159	PXN	135007.8
9_S26_peak_48006_lociStitched		GASAL1	138642.5
27_S26_peak_2492_lociStitched	ENSG00000137936	BCAR3	143286
14_S26_peak_20073_lociStitched	ENSG00000170425	ADORA2B	139402.3
7_S26_peak_30160_lociStitched	ENSG00000125998	FAM83C	143347.1
35_S26_peak_49379_lociStitched	ENSG00000107338	SHB	152461.2
3_S26_peak_9991_lociStitched	ENSG00000186174	BCL9L	146169.3
17_S26_peak_1591_lociStitched	ENSG00000227533	SLC2A1-AS1	152272.2
59_S26_peak_48420_lociStitched	ENSG00000280055	TMEM75	182832.4
12_S26_peak_3417_lociStitched	ENSG00000160789	LMNA	159661.3
11_S26_peak_13755_lociStitched	ENSG00000102554	KLF5	169479.8
29_S26_peak_24736_lociStitched	ENSG00000130402	ACTN4	165580.7
11_S26_peak_21272_lociStitched	ENSG00000246640	PICART1	164468.2
43_S26_peak_16744_lociStitched	ENSG00000166949	SMAD3	183973.8
5_S26_peak_24169_lociStitched	ENSG00000187556	NANOS3	166330.1
5_S26_peak_13498_lociStitched	ENSG00000151778	SERP2	197619.4
6_S26_peak_48759_lociStitched	ENSG00000178209	PLEC	202909.4
21_S26_peak_50713_lociStitched		LINC00963	212218.3
37_S26_peak_6383_lociStitched	ENSG00000168209	DDIT4	260266.5
S26_peak_11225	ENSG00000167767	KRT80	259094.9
4_S26_peak_48321_lociStitched		CCAT1	267960.6
14_S26_peak_635_lociStitched	ENSG00000142627	EPHA2	271916.8

Supplementary Data 3, GRO-Seq data of seRNA LOC100506178

PeakID (cmd= annotatePeaks.pl Enhancer.bed hg38)	Chr	Start	End	Gene Name
4996	chr2	27647720	27668382	SLC4A1AP
5231	chr2	9204314	9210844	ASAP2
4461	chr19	49612577	49617930	PRR12
7292	chr6	122695770	122698238	PKIB
6624	chr4	76305590	76307527	STBD1
9307	chrX	47190821	47193363	UBA1
4917	chr2	226426069	226427717	MIR5702
5449	chr20	58388334	58414009	VAPB
5829	chr3	115144294	115148065	ZBTB20
2062	chr12	51165931	51174721	TFCP2
8689	chr8	78804377	78806552	IL7
5108	chr2	55441058	55443049	CCDC88A
9012	chr9	36571865	36577051	MELK
1947	chr12	19742999	19760130	LINC02398
6601	chr4	6782762	6818586	KIAA0232
6165	chr3	37238741	37270398	LOC152048
3344	chr16	56419234	56426238	AMFR
6052	chr3	183369609	183373272	MCF2L2
6920	chr5	177002017	177007650	UIMC1
6113	chr3	196318129	196327627	TM4SF19-AS1
2784	chr14	77299279	77304636	POMT2
3281	chr16	30949074	30968572	SETD1A
1765	chr12	101390531	101411881	ARL1
7994	chr7	116352997	116356728	LOC102724434
9224	chrX	13109886	13112113	FAM9C
2604	chr14	28652669	28655697	FOXG1-AS1
4108	chr18	63360981	63370670	KDSR
2478	chr13	72738312	72784631	PIBF1
2943	chr15	56018635	56051419	NEDD4
6639	chr4	79972117	80075042	ANTXR2
908	chr1	93515811	93517062	FNBP1L
3884	chr17	75011438	75023110	MRPL58
9341	chrX	55158351	55162401	FAM104B
8989	chr9	33437075	33447964	AQP3
2322	chr13	109752494	109785628	IRS2
6071	chr3	186785680	186788523	SNORA63
5921	chr3	138159668	138176365	DBR1
834	chr1	67413028	67432675	SERBP1
6755	chr5	132482662	132492065	IRF1

8059	chr7	140175770	140178749	KDM7A-DT
6894	chr5	172730460	172732630	LOC101928093
8476	chr8	129983671	130000925	MIR5194
4358	chr19	42736880	42741269	PSG3
2139	chr12	6166925	6168355	CD9
4595	chr2	101295416	101310051	RNF149
700	chr1	37691113	37697386	CDCA8
4876	chr2	209473356	209475230	MAP2
4734	chr2	169797479	169825138	SNORD3K
5876	chr3	126406232	126479405	ZXDC
8856	chr9	127704623	127716817	CFAP157
3868	chr17	7213717	7243954	MIR324
4387	chr19	44641429	44668130	MIR4531
4269	chr19	29955872	30039916	URI1
7052	chr5	57734074	57737454	LOC101928505
7223	chr6	10031998	10034618	TFAP2A-AS2
3144	chr16	11626883	11629741	SNN
6026	chr3	170863589	170871347	RPL22L1
9250	chrX	154246837	154249339	OPN1MW2
2920	chr15	49618989	49657120	DTWD1
1695	chr11	832280	870306	TSPAN4
9127	chr9	92763998	92766648	BICD2
8076	chr7	149231923	149235787	ZNF212
228	chr1	15601995	15605416	DDI2
7949	chr7	101121429	101123730	SERPINE1
6185	chr3	44508089	44512160	ZNF852
456	chr1	211255613	211259131	RCOR3
3319	chr16	46965060	46977651	DNAJA2
5253	chr2	98589985	98622426	COA5
309	chr1	169108736	169110651	ATP1B1
1482	chr11	33076785	33162914	CSTF3
7327	chr6	133703529	133747437	LINC01312
7945	chr7	100704656	100711582	POP7
5982	chr3	153159915	153194445	RAP2B
1052	chr10	13347293	13351803	SEPHS1
7848	chr6_GL000253v2_alt	1316422	1323375	ZNRD1ASP
1586	chr11	64565752	64567851	SLC22A11
7889	chr6_GL000255v2_alt	103407	104304	LINC01623
428	chr1	205811939	205816166	SLC41A1
7279	chr6	116680269	116684677	KPNA5
5726	chr22	42613070	42616425	POLDIP3
7383	chr6	145785134	145833509	FBXO30
2003	chr12	40689782	40691887	CNTN1
3730	chr17	47076836	47091214	RPRML

8969	chr9	21918338	21921354	CDKN2A-DT
8151	chr7	24693528	24711858	GSDME
7973	chr7	106344465	106348046	NAMPT
6839	chr5	144151322	144171552	YIPF5
6971	chr5	27327040	27329352	PURPL
6745	chr5	131169060	131178743	LYRM7
4954	chr2	237692831	237696643	LRRFIP1
3496	chr16_KI270853v1_alt	1556794	1558854	FOPNL
2661	chr14	49882204	49884957	ARF6
8850	chr9	127412654	127419777	ZNF79
8124	chr7	17404642	17410711	KCCAT333
8074	chr7	149089729	149094101	ZNF786
5598	chr21	6553227	6555714	CRYAA
4790	chr2	190536883	190543055	NEMP2
264	chr1	161096885	161134984	NIT1
3625	chr17	3630522	3675596	TAX1BP3
3442	chr16	81445641	81456647	CMIP
6733	chr5	121959794	121996576	SRFBP1
4302	chr19	36532912	36539248	ZNF260
38	chr1	109591183	109615417	MIR197
3593	chr17	2974967	2990781	LOC101927911
5224	chr2	87566201	87698872	MIR4435-2
4986	chr2	27085054	27087377	KHK
7049	chr5	56908584	56912909	SETD9
8445	chr8	123166475	123168408	FAM83A
627	chr1	26350297	26353372	CRYBG2
2561	chr14	104969436	104971883	AHNAK2
1847	chr12	118386094	118389398	SUDS3
5607	chr22	16824481	16826795	HSFY1P1
3170	chr16	15588595	15592070	MIR6506
7900	chr6_GL000255v2_alt	2034921	2038613	HCG20
8964	chr9	21574743	21581951	MIR31HG
4689	chr2	148644258	148647738	EPC2
6868	chr5	157870435	157872118	CLINT1
8942	chr9	137198906	137221096	NDOR1
7990	chr7	113741277	113744339	PPP1R3A
5189	chr2	74458556	74467806	MOGS
8616	chr8	42186146	42187851	PLAT
4599	chr2	102735621	102738172	MFSD9
6218	chr3	48613176	48615234	UQCRC1
4323	chr19	39406140	39412427	MIR4530
2644	chr14	42191317	42194074	LRFN5
9187	chrX	109724401	109733421	ACSL4
1365	chr11	112158836	112165180	IL18

7124	chr5	75714974	75718722	LOC441087
4471	chr19	51393507	51396649	C19orf84
3676	chr17	42958940	42965619	AARSD1
7943	chr7	100426784	100437689	MEPCE
2317	chr13	106347590	106349604	LINC00460
1961	chr12	26298451	26300266	SSPN
6934	chr5	179633643	179635170	C5orf60
7940	chr7	100151332	100161255	MIR4658
4099	chr18	54827186	54833462	RAB27B
6676	chr4	98910781	98931218	EIF4E
1267	chr10	88989748	89000642	FAS-AS1
6012	chr3	160748335	160763125	PPM1L
8166	chr7	27652531	27663723	HIBADH
778	chr1	52402643	52416878	PRPF38A
717	chr1	40683134	40704246	NFYC-AS1
3448	chr16	844462	847482	PRR25
4343	chr19	41254380	41302722	HNRNPUL1
3205	chr16	2214839	2218744	PGP
4802	chr2	196765651	196779574	GTF3C3
1476	chr11	31439890	31518582	IMMP1L
1971	chr12	2798043	2810596	ITFG2
7694	chr6	57217646	57223474	RAB23
4487	chr19	55337988	55343923	KMT5C
4043	chr18	22347157	22348195	CTAGE1
5233	chr2	9385497	9391926	ITGB1BP1
9208	chrX	123730428	123733893	THOC2
524	chr1	226109729	226111955	LINC01703
757	chr1	46267403	46299921	LRRC41
6016	chr3	161415081	161417286	LINC02067
791	chr1	56687503	56690445	LOC101929935
4419	chr19	46734770	46749739	FKRP
9261	chrX	1589850	1596904	AKAP17A
4820	chr2	200524336	200527399	SGO2
662	chr1	31529635	31532206	LINC01226
5534	chr21	36294786	36298161	MORC3
8640	chr8	54036457	54051424	TCEA1
4111	chr18	63505294	63507660	SERPINB5
4815	chr2	199974361	199984669	TYW5
623	chr1	25870400	25877218	PAQR7
1959	chr12	26112324	26116344	BHLHE41
8210	chr7	43911626	43928979	URGCP
8404	chr8	103019611	103068399	ATP6V1C1
4843	chr2	202248459	202251446	SUMO1
4596	chr2	101735959	101742093	MAP4K4

958	chr10	103366784	103369018	TAF5
2215	chr12	75721074	75723906	KRR1
2151	chr12	64087844	64140077	C12orf66
3961	chr17	81529113	81532984	FSCN2
7296	chr6	12471899	12473887	LINC02530
4982	chr2	26342726	26345832	ADGRF3
6816	chr5	14139315	14318841	TRIO
2827	chr15	100712489	100714625	ASB7
295	chr1	167216507	167225048	POU2F1
1091	chr10	29162530	29165631	LYZL1
3429	chr16	74397415	74398490	LOC105376772
1298	chr10	95393606	95395155	SORBS1
3561	chr17	2330660	2337707	TSR1
6336	chr4	110192101	110199652	ELOVL6
7646	chr6	43031678	43040462	RRP36
4537	chr19	7390201	7391958	ARHGEF18
2405	chr13	37701151	37703369	POSTN
5977	chr3	152262722	152297602	MBNL1-AS1
5375	chr20	45327298	45350193	SDC4
7675	chr6	52418821	52422993	EFHC1
8655	chr8	62862954	62865173	UG0898H09
136	chr1	147241224	147250335	CHD1L
546	chr1	230065453	230113584	GALNT2
1569	chr11	62876897	62888106	SLC3A2
8367	chr7	96686480	96710736	SEM1
4106	chr18	62525331	62528296	ZCCHC2
7073	chr5	64714591	64716007	SHISAL2B
143	chr1	148397605	148413895	RNVU1-3
6394	chr4	139264384	139268010	MGARP
5333	chr20	36091064	36095178	EPB41L1
3681	chr17	43232723	43234160	RNU2-1
5320	chr20	35399348	35413114	UQCC1
6248	chr3	5125736	5135988	ARL8B
4015	chr17_KI270857v1_alt	41743	93418	TAF15
1402	chr11	12376675	12389333	PARVA
6300	chr3	98799293	98836711	ST3GAL6
4496	chr19	55652857	55671733	U2AF2
2731	chr14	67693520	67696663	RDH11
1978	chr12	29341194	29383030	ERGIC2
2366	chr13	21148847	21155162	SAP18
6708	chr5	110899569	110900803	SLC25A46
8628	chr8	47954580	47969850	MCM4
2282	chr12	95652912	95656120	PGAM1P5
3491	chr16	9088923	9152124	C16orf72

1748	chr11	9663481	9666755	SWAP70
4786	chr2	189437106	189455320	WDR75
4206	chr19	16515303	16522563	C19orf44
2674	chr14	52551754	52554215	GPR137C
1580	chr11	64165046	64172046	MACROD1
4214	chr19	17417988	17421106	MVB12A
528	chr1	226700480	226705049	ITPKB-IT1
217	chr1	155197090	155199087	MIR92B
5740	chr22	44010807	44029565	PARVB
367	chr1	184936306	184955110	FAM129A
5892	chr3	129209219	129213872	CNBP
2638	chr14	39098624	39106195	SEC23A-AS1
5962	chr3	150300364	150301633	LINC01214
3323	chr16	4767214	4768471	ZNF500
5232	chr2	9233599	9249750	ASAP2
6414	chr4	147545463	147547503	EDNRA
2437	chr13	45463819	45475433	COG3
2664	chr14	50059473	50064429	LINC01599
8011	chr7	12646319	12648405	ARL4A
9329	chrX	52893507	52900059	FAM156B
7243	chr6	10721515	10730211	TMEM14C
5563	chr21	41994117	42013219	ZNF295-AS1
1641	chr11	67345215	67359861	POLD4
7168	chr5	8906036	8908359	LINC02199
4645	chr2	118012290	118015201	CCDC93
1811	chr12	110431450	110432539	ARPC3
8092	chr7	151726211	151728772	PRKAG2
5277	chr20	16728978	16745116	SNRPB2
8668	chr8	66506013	66508250	VXN
4578	chr1_GL383519v1_alt	2789	13781	MTX1
4407	chr19	45851671	45863265	SYMPK
5280	chr20	18505273	18513353	SEC23B
5039	chr2	37217843	37220962	CEBPZ
8889	chr9	129606100	129608863	NTMT1
7498	chr6	26100866	26105550	HIST1H4C
9231	chrX	135334843	135349911	ZNF75D
3653	chr17	408780	411367	C17orf97
5953	chr3	149223470	149226063	CP
851	chr1	77574804	77684729	ZZZ3
385	chr1	193081522	193099351	GLRX2
2311	chr13	102762739	102775097	TEX30
517	chr1	225699353	225701720	ENAH
8953	chr9	14990953	14996010	LOC389705
2324	chr13	110137344	110140062	LOC105370361

7058	chr5	60698601	60701087	DEPDC1B
6770	chr5	134427522	134486242	LOC102546229
1991	chr12	31740336	31760629	AMN1
6304	chr3	98979093	98982529	DCBLD2
2078	chr12	53003947	53033860	EIF4B
3557	chr17	20907911	20911092	CCDC144NL
3609	chr17	32006859	32009531	LRRC37B
3318	chr16	4615351	4618007	UBALD1
4303	chr19	36560906	36578411	ZNF529-AS1
8552	chr8	22243200	22261831	POLR3D
8051	chr7	139039775	139110954	ZC3HAV1
3292	chr16	31439283	31461180	ZNF843
2904	chr15	44545164	44553773	EIF3J
3810	chr17	60519503	60530016	APPBP2
9383	chrX	9162446	9167604	FAM9B
1848	chr12	11883702	11887405	BCL2L14
3376	chr16	67310888	67313170	KCTD19
5230	chr2	91597734	91600352	LOC654342
5623	chr22	20392520	20395805	ZNF74
5171	chr2	70276507	70296501	SNRPG
9024	chr9	38021870	38047526	SHB
3786	chr17	55458031	55460054	MMD
1921	chr12	131949202	131952620	EP400
1891	chr12	123581940	123596000	TMED2
3756	chr17	49400955	49415940	PHB
225	chr1	155889484	155894504	RIT1
9285	chrX	30282634	30284790	NR0B1
1185	chr10	68899234	68918045	DDX50
1128	chr10	43420003	43421944	SNORD3J
2079	chr12	53066655	53080416	SPRYD3
5131	chr2	64441651	64463358	LGALSL
6832	chr5	143281918	143399178	NR3C1
33	chr1	109212056	109246868	SARS
2011	chr12	4514328	4547189	C12orf4
1222	chr10	73785649	73808585	ZSWIM8-AS1
2758	chr14	73057096	73071208	RBM25
2915	chr15	48318931	48348417	DUT
1329	chr11	102842001	102844734	MMP3
7096	chr5	70047173	70050766	SMN2
3861	chr17	7004435	7018371	ALOX12-AS1
8887	chr9	129459429	129461954	LINC00963
6935	chr5	179657297	179661233	LOC105377763
9280	chrX	23898104	23902490	APOO
6705	chr5	109410055	109412097	PJA2

3549	chr17	19234528	19241684	EPN2
876	chr1	85324356	85330233	BCL10
376	chr1	1899168	1911188	CALML6
7988	chr7	112938643	112941655	BMT2
1694	chr11	83181388	83196106	PCF11-AS1
7667	chr6	47134203	47136505	ADGRF1
4395	chr19	45412035	45426906	ERCC1
1060	chr10	17060935	17064590	CUBN
2419	chr13	41249254	41264746	MTRF1
2337	chr13	112657159	112659428	ATP11AUN
9288	chrX	315420	317014	GTPBP6
156	chr1	149799125	149813705	HIST2H2BF
2431	chr13	44708487	44711943	LINC00407
5084	chr2	47369453	47373270	EPCAM
8310	chr7	75449415	75487303	POM121C
802	chr1	59426413	59428507	FGGY
7283	chr6	117563487	117605066	GOPC
2998	chr15	67234085	67257933	AAGAB
8331	chr7	7967152	7973734	LOC100505921
5796	chr3	10190975	10193679	IRAK2
1752	chr11	9831166	9833192	LOC101928008
2302	chr13	100672239	100676198	TMTC4
3646	chr17	39925020	39928554	ORMDL3
531	chr1	227288264	227317785	CDC42BPA
6618	chr4	75458122	75518629	RCHY1
6488	chr4	187813069	187817080	LINC02492
947	chr10	101726489	101729255	FBXW4
2948	chr15	56918284	56941855	TCF12
1143	chr10	51688579	51701911	CSTF2T
4254	chr19	23956666	23959209	LOC100505851
1484	chr11	3349468	3379937	ZNF195
1681	chr11	767623	797295	LOC171391
3971	chr17	81936033	81939689	PYCR1
1393	chr11	12176985	12211779	MIR6124
5049	chr2	38526896	38529494	LOC101929596
419	chr1	204415971	204418683	PPP1R15B
5178	chr2	73111996	73115561	RAB11FIP5
1293	chr10	93655538	93703975	FRA10AC1
907	chr1	93448469	93455232	FNBP1L
4989	chr2	27353332	27359277	GTF3C2
4366	chr19	43175867	43207303	PSG5
8150	chr7	24571906	24585198	MPP6
835	chr1	67681892	67692630	GADD45A
8066	chr7	144833430	144837126	TPK1

1882	chr12	122713543	122715687	HCAR3	
6077	chr3	189022352	189023724	TPRG1-AS1	
1572	chr11	63613950	63615817	PLA2G16	
5975	chr3	151765666	151783872	AADACP1	
5788	chr3	100708061	100763520	TFG	
2355	chr13	19631832	19641948	MPHOSPH8	
8811	chr9	115372300	115374573		1-Dec
7431	chr6	156686087	156687578	ARID1B	
6034	chr3	172702939	172712795	NCEH1	
2481	chr13	73193771	73196507	KLF5	
6009	chr3	16057290	16063190	GALNT15	
2023	chr12	46546784	46617946	LOC100288798	
5764	chr22	49854001	49857976	ZBED4	
2651	chr14	45251045	45256316	MIS18BP1	
2395	chr13	33242175	33286948	STARD13-AS	
2219	chr12	75942571	75956524	PHLDA1	
2027	chr12	47650077	47707997	RPAP3	
5922	chr3	138595303	138646630	FAIM	
2407	chr13	39653904	39680702	MIR4305	
3293	chr16	31484114	31513634	C16orf58	
927	chr1	95071100	95074768	ALG14	
8952	chr9	14311908	14317932	NFIB	
62	chr1	114568414	114583668	BCAS2	
6028	chr3	171095620	171098026	MIR569	
5819	chr3	112556085	112567065	SLC35A5	
2521	chr13	97222853	97266288	MBNL2	
4080	chr18	46085800	46099735	ATP5F1A	
8669	chr8	66517866	66518874	VXN	
973	chr10	106512248	106515320	LOC105378470	
9103	chr9	83915959	83922036	KIF27	
6076	chr3	188947041	188949974	TPRG1-AS1	
4430	chr19	47314170	47318024	C5AR1	
3761	chr17	50045904	50086971	ITGA3	
8333	chr7	80769941	80921064	SEMA3C	
1487	chr11	34049840	34075930	CAPRIN1	
3123	chr15_GL383554v1_alt	286292	292659	HERC2P11	
4433	chr19	47597337	47602299	BICRA	
1729	chr11	93760736	93767472	MED17	
4633	chr2	112581757	112585846	CHCHD5	
412	chr1	203304103	203312588	BTG2	
4444	chr19	4827282	4833525	TICAM1	
593	chr1	241479092	241521167	FH	
1145	chr10	52333479	52337446	DKK1	
648	chr1	28284480	28290070	SESN2	

1319	chr10	99975367	99978110	DNMBP
8394	chr8	100950329	100957077	YWHAZ
3126	chr15_KI270849v1_alt	29685	38273	B2M
1627	chr11	66545082	66546936	ZDHHC24
1257	chr10	86744959	86770150	BMPR1A
9238	chrX	141172839	141178022	LDOC1
1367	chr11	114295481	114300507	NNMT
1935	chr12	1586932	1593123	FBXL14
4233	chr19	18570577	18581285	UBA52
1195	chr10	70403734	70437256	EIF4EBP2
1184	chr10	68719418	68746717	CCAR1
2715	chr14	64382335	64390842	MTHFD1
664	chr1	31923168	31935801	PTP4A2
2966	chr15	63189204	63192795	RAB8B
2048	chr12	49835004	49844764	BCDIN3D
5055	chr2	39386584	39440753	MAP4K3
5423	chr20	51330937	51351208	MIR3194
9239	chrX	141403920	141407930	SPANXA2-OT1
6446	chr4	168599905	168602693	PALLD
7041	chr5	553309	555096	MIR4456
677	chr1	32956302	32965781	RNF19B
3276	chr16	30748468	30778047	RNF40
63	chr1	114665120	114672013	DENND2C
6649	chr4	83033706	83039142	COPS4
6178	chr3	42566453	42619124	NKTR
7023	chr5	44874243	44878232	MRPS30
1920	chr12	131928415	131936871	PUS1
7778	chr6	9478492	9483067	HULC
3088	chr15	89688223	89691716	PEX11A
3773	chr17	5108665	5112874	LOC101928000
85	chr1	11978636	11980380	MFN2
9084	chr9	72585300	72586906	TMC1
2684	chr14	54414077	54418137	CDKN3
6586	chr4	56085535	56087992	
5481	chr20	64065165	64069090	TCEA2
6576	chr4	53364020	53367395	SCFD2
5618	chr22	19840752	19859074	RTL10
7406	chr6	149850561	149865569	RAET1E-AS1
8691	chr8	79789153	79796514	LINC01607
3328	chr16	4875739	4902767	UBN1
4835	chr2	201415277	201421157	TRAK2
8798	chr9	113200699	113203726	FKBP15
1348	chr11	10802226	10810995	EIF4G2
8742	chr8	99495291	99497099	MIR599

4615	chr2	10935552	10937835	KCNF1
6216	chr3	48462836	48474082	TREX1
7408	chr6	149941122	149943233	ULBP2
5409	chr20	50307201	50311568	LINC01271
2236	chr12	8025472	8028227	FOXJ2
6187	chr3	44623967	44656531	ZNF35
2318	chr13	106375538	106382185	LINC00460
1519	chr11	47405884	47413148	SLC39A13
922	chr1	94525736	94541586	F3
2181	chr12	68744426	68751455	SLC35E3
2906	chr15	44636188	44664221	SPG11
8757	chr9	100425336	100441731	MSANTD3
3727	chr17	46769747	46773047	WNT3
684	chr1	34839744	34860601	SMIM12
5539	chr21	36987273	36992459	HLCS
278	chr1	162561522	162620687	UAP1
1274	chr10	89699195	89703180	KIF20B
3551	chr17	19376652	19377505	MAPK7
4657	chr2	127418662	127423806	MIR4783
4975	chr2	24791004	24796317	PTRHD1
6665	chr4	88590428	88596111	HERC3
7661	chr6	43768459	43789491	VEGFA
5228	chr2	88688871	88692718	RPIA
3419	chr16	71893597	71900616	IST1
5505	chr21	31352746	31355912	TIAM1
669	chr1	32194235	32227909	DCDC2B
2976	chr15	64243842	64248240	PPIB
7269	chr6	112216031	112218102	LOC101927640
3817	chr17	62422329	62433881	METTL2A
3477	chr16	88799792	88807381	CDT1
636	chr1	26887653	26891295	GPN2
579	chr1	23553193	23563231	ID3
5578	chr21	43767662	43770738	CSTB
3540	chr17	1857171	1858672	RPA1
2291	chr12	98502519	98505396	LINC02453
8421	chr8	11283361	11288219	MTMR9
8438	chr8	121639900	121643682	HAS2
2941	chr15	55626808	55628976	PYGO1
4605	chr2	105036258	105043518	LOC102724691
2783	chr14	77093834	77104183	CIPC
2957	chr15	59664216	59689396	BNIP2
8514	chr8	144305354	144327075	MIR6848
3271	chr16	30555122	30560144	ZNF764
8097	chr7	151874614	151879268	PRKAG2-AS1

4235	chr19	18682457	18687226	CRTC1
2946	chr15	56709966	56736241	ZNF280D
8122	chr7	17295675	17338469	AHR
4217	chr19	17793970	17801681	B3GNT3
1767	chr12	101696836	101705983	CHPT1
626	chr1	26303389	26307638	UBXN11
5838	chr3	119419929	119465278	TMEM39A
9020	chr9	37766529	37786828	EXOSC3
3067	chr15	82210297	82262650	EFL1
4359	chr19	42759292	42769797	PSG8
5183	chr2	73925140	73954648	DGUOK
999	chr10	11896367	11899754	PROSER2-AS1
2383	chr13	29800892	29851232	UBL3
4946	chr2	234257242	234259359	LINC01891
7894	chr6_GL000255v2_alt	184418	191177	TRIM27
5800	chr3	104278623	104281671	MIR548AB
6162	chr3	36646646	36649485	DCLK3
2119	chr12	56708647	56727131	NACA
4219	chr19	17999715	18002729	ARRDC2
4112	chr18	657167	659176	TYMSOS
9323	chrX	49070752	49075011	PRAF2
4040	chr18	21097877	21112811	ROCK1
1800	chr12	109468886	109484981	KCTD10
6474	chr4	183653237	183663132	RWDD4
2526	chr13	99214798	99221006	UBAC2
3330	chr16	4922230	4939254	PPL
8673	chr8	67052625	67066730	COPS5
4528	chr19	6414731	6424898	MIR3940
9336	chrX	53579110	53685896	HUWE1
496	chr1	221986301	221988927	LINC02257
1531	chr11	57656792	57658881	CLP1
6259	chr3	52283938	52290524	GLYCTK
6927	chr5	178144715	178156448	NHP2
6714	chr5	112859046	112923083	SRP19
3975	chr17	82210652	82260896	SLC16A3
2631	chr14	35396689	35407956	NFKBIA
1264	chr10	87860338	87987631	PTEN
6858	chr5	153550971	153553011	GRIA1
867	chr1	8200584	8204476	LINC01714
9044	chr9	5782136	5797355	ERMP1
8471	chr8	127793826	128118563	MIR1205
9275	chrX	21931791	21933728	SMS
5634	chr22	23964877	23976538	DDT
5152	chr2	67289027	67291768	LINC01829

8420	chr8	11173405	11202634	XKR6
8104	chr7	154926603	154929565	PAXIP1-AS2
450	chr1	209826659	209860764	UTP25
1300	chr10	96127750	96176299	ZNF518A
5129	chr2	63585246	63606657	MDH1
3631	chr17	37399028	37409590	ACACA
8543	chr8	19311812	19317209	SH2D4A
5583	chr21	44778055	44807434	UBE2G2
1691	chr11	820443	822871	PNPLA2
4270	chr19	3175885	3177097	S1PR4
746	chr1	44984000	44987397	EIF2B3
9309	chrX	47654346	47664574	UXT
2220	chr12	75971472	75981199	PHLDA1
8875	chr9	128761579	128772687	ZER1
3485	chr16	89654829	89658745	SPATA33
4061	chr18	3248226	3282395	MYL12B
7591	chr6	34222551	34226042	HMGA1
6896	chr5	172764007	172774567	DUSP1
6080	chr3	189587331	189589833	TP63
5724	chr22	42078158	42087645	SMDT1
269	chr1	161312677	161317682	SDHC
2710	chr14	61761169	61769350	SNAPC1
2858	chr15	36578108	36584452	C15orf41
8570	chr8	26571113	26573471	DPYSL2
5949	chr3	146496363	146546742	PLSCR1
8184	chr7	32479977	32492179	LSM5
6347	chr4	118828988	118835769	SEC24D
1664	chr11	71787286	71792866	FAM86C1
4623	chr2	111119856	111124346	BCL2L11
1594	chr11	64910516	64917177	ATG2A
3841	chr17	65121292	65124845	RGS9
8447	chr8	123256411	123272894	ZHX1
748	chr1	45335960	45351790	MUTYH
4933	chr2	231665407	231668098	PTMA
4223	chr19	18223292	18224975	PDE4C
630	chr1	26442425	26447787	DHDDS
2944	chr15	56116198	56135196	RFX7
6630	chr4	78058122	78059872	FRAS1
7313	chr6	1312588	1317229	FOXQ1
9367	chrX	7371888	7374317	STS
3649	chr17	40090373	40104078	NR1D1
2125	chr12	57227967	57244531	NDUFA4L2
8916	chr9	133347217	133353563	SNORD36A
6656	chr4	84636355	84638471	CDS1

8304	chr7	74655872	74660322	GTF2I
991	chr10	11453142	11458804	USP6NL
2622	chr14	34424604	34426887	SPTSSA
5150	chr2	66432398	66437367	MEIS1
8818	chr9	120764428	120796926	FBXW2
2822	chr14_GL000225v1_random	48759	51041	
656	chr1	29155127	29182732	SRSF4
7572	chr6	3151107	3164048	TUBB2A
8512	chr8	144134016	144140012	HGH1
7529	chr6	27790258	27792488	HIST1H2BL
4463	chr19	49764926	49768975	AP2A1
967	chr10	104297184	104342393	LOC101927472
382	chr1	192806626	192814286	RGS2
3098	chr15	90927548	90932772	UNC45A
7618	chr6	37167991	37177831	PIM1
325	chr1	172933279	172934981	TNFSF18
8414	chr8	108185806	108250885	EIF3E
3048	chr15	75757857	75760702	MIR4313
5663	chr22	30839892	30870149	OSBP2
3983	chr17	82495572	82498824	FOXK2
5201	chr2	82992069	82995192	DHFRP3
3229	chr16	2742826	2753243	SRRM2-AS1
5693	chr22	38200912	38207470	MAFF
8108	chr7	155292911	155297106	INSIG1
3800	chr17	58657634	58659941	TEX14
7916	chr6_GL000256v2_alt	1273352	1275936	HCG9
1146	chr10	52439471	52456763	LINC01468
9072	chr9	69172935	69185208	TJP2
7046	chr5	55988591	56000732	IL6ST
8027	chr7	130034311	130052318	ZC3HC1
7953	chr7	101320553	101323829	IFT22
8106	chr7	154980438	155010249	PAXIP1
3503	chr16_KI270854v1_alt	22416	29178	RAB11FIP3
4282	chr19	34403744	34405671	PDCD2L
7167	chr5	88881328	88885715	MEF2C
333	chr1	1746220	1747834	SLC35E2A
7078	chr5	65532884	65600024	PPWD1
5569	chr21	43175921	43178257	CRYAA2
3081	chr15	88540535	88549002	DET1
5795	chr3	101723718	101730320	CEP97
3347	chr16	56605580	56608717	MT2A
671	chr1	32381906	32395987	BSDC1
3555	chr17	2039666	2047796	OVCA2

8213	chr7	44080271	44084107	POLM
4929	chr2	231461206	231465801	NCL
9016	chr9	37450749	37455172	ZBTB5
1057	chr10	15846603	15862264	MINDY3
1341	chr11	105243185	105246115	CARD18
311	chr1	169478273	169488335	SLC19A2
7106	chr5	72441437	72442824	ZNF366
6771	chr5	134502445	134504778	LINC01843
2359	chr13	19956575	19958196	ZMYM2
2818	chr14	99511738	99516556	CCNK
94	chr1	120951216	120962699	RNVU1-4
9040	chr9	4791887	4798085	RCL1
4480	chr19	53865713	53870863	MYADM
806	chr1	6205451	6207045	RNF207
8626	chr8	47512597	47515232	SPIDR
3301	chr16	3281197	3324319	ZNF75A
4554	chr19	9414624	9437493	ZNF266
5307	chr20	33394352	33402342	CDK5RAP1
4829	chr2	200959723	200971115	ORC2
2201	chr12	71754168	71805759	RAB21
1537	chr11	59543774	59550172	OR4D9
8403	chr8	102804021	102810203	GASAL1
821	chr1	65253621	65266169	DNAJC6
4405	chr19	45710205	45717428	FBXO46
1382	chr11	118885192	118921635	MIR4492
7314	chr6	131697634	131715930	CTAGE9
1069	chr10	1958426	1991391	LINC00700
6555	chr4	41709080	41711011	PHOX2B
9164	chrX	100819519	100821754	CSTF2
5492	chr21	25732496	25776570	ATP5PF
3299	chr16	3218919	3236038	ZNF200
8766	chr9	106915525	106921789	ZNF462
877	chr1	85442214	85466664	DDAH1
4334	chr19	40402856	40404600	PRX
2020	chr12	46428239	46496347	LOC100288798
680	chr1	33319348	33349278	MIR3605
7622	chr6	3796530	3799318	PXDC1
5043	chr2	37560593	37564447	CDC42EP3
1964	chr12	27020504	27036977	MED21
8722	chr8	94240615	94243620	GEM
709	chr1	39105232	39107583	MACF1
5143	chr2	65224756	65238138	ACTR2
7450	chr6	160100298	160104616	LOC729603
6278	chr3	68635695	68637571	LOC105377146

3822	chr17	62966092	63169644	TANC2
2299	chr12_KI270835v1_alt	180699	216386	FLJ13224
7777	chr6	90501281	90588852	MAP3K7
5463	chr20	62345418	62350911	LAMA5-AS1
6706	chr5	109688697	109696309	MAN2A1
3920	chr17	7832702	7835399	KDM6B
5385	chr20	45963793	45973572	ZNF335
685	chr1	35025291	35033749	ZMYM6
2866	chr15	40337598	40340605	CCDC9B
786	chr1	54562671	54567643	ACOT11
1308	chr10	97493419	97499035	MMS19
1574	chr11	63680314	63696788	RTN3
2842	chr15	26081124	26083603	LINC00929
6670	chr4	94453358	94475568	PDLIM5
4368	chr19	43263159	43278137	PSG9
4991	chr2	27396637	27410740	PPM1G
4641	chr2	113615489	113646884	RABL2A
4188	chr19	14079094	14085788	C19orf67
4286	chr19	3540163	3552985	C19orf71
3044	chr15	75451137	75453719	SIN3A
6273	chr3	57754901	57756160	SLMAP
3331	chr16	50244898	50261832	ADCY7
6445	chr4	168588252	168590753	PALLD
8534	chr8	17466094	17468967	SLC7A2
279	chr1	163285390	163323494	RGS5
1889	chr12	123383243	123389939	KMT5A
4489	chr19	55374995	55377325	TMEM190
2463	chr13	51451028	51469633	INTS6-AS1
4631	chr2	112271647	112285776	ZC3H6
1506	chr11	45044638	45047383	PRDM11
8379	chr7	99451943	99461608	ATP5MF
4500	chr19	5672541	5681773	HSD11B1L
5511	chr21	32735048	32773454	PAXBP1
1899	chr12	124859189	124865028	SCARB1
1053	chr10	13864670	13867479	FRMD4A
4891	chr2	218215520	218234264	ARPC2
8416	chr8	108441927	108450823	EMC2
635	chr1	26861599	26867133	SFN
6713	chr5	112706617	112711301	APC
3364	chr16	58620800	58631105	CNOT1
8049	chr7	135743743	135747892	FAM180A
1019	chr10	122979139	122983851	PSTK
6917	chr5	176341201	176353119	KIAA1191
3635	chr17	38349877	38356392	SOCS7

8747	chr8_KI270816v1_alt	103561	108766	TSTA3
2970	chr15	63573896	63578465	FBXL22
1177	chr10	68059658	68073798	HERC4
575	chr1	235316277	235340338	ARID4B
1962	chr12	26823194	26833075	ITPR2
3032	chr15	73051212	73055344	NEO1
153	chr1	149390373	149413441	NBPF19
4913	chr2	224558140	224587567	CUL3
7896	chr6_GL000255v2_alt	1938618	1945707	PPP1R18
8678	chr8	683359	688889	ERICH1
2296	chr12_GL383550v2_alt	164394	169108	LOC100506844
8827	chr9	121735745	121737947	DAB2IP
5908	chr3	133650230	133671471	TOPBP1
7523	chr6	27139285	27149488	HIST1H2BK
6719	chr5	115215346	115265056	PGGT1B
3489	chr16	90021797	90023784	GAS8
3147	chr16	11777979	11808092	ZC3H7A
3697	chr17	44203230	44222814	MIR6782
9311	chrX	48473516	48480131	FTSJ1
8318	chr7	76379068	76381367	ZP3
8258	chr7	6439982	6447868	DAGLB
7213	chr5_KI270791v1_alt	184804	195646	CLPTM1L
1026	chr10	125002880	125008098	CTBP2
4450	chr19	48613084	48620932	SPHK2
1909	chr12	12871263	12887137	RPL13AP20
1194	chr10	70221025	70234852	PPA1
3667	chr17	42543484	42558771	HSD17B1
6886	chr5	169976416	169978690	FAM196B
1709	chr11	88171164	88177156	RAB38
3754	chr17	4918713	4957239	RNF167
3736	chr17	47489733	47494291	MRPL45P2
8335	chr7	84057619	84062101	SEMA3A
4002	chr17_GL000258v2_alt	592443	597545	KANSL1
2839	chr15	23034967	23040432	TUBGCP5
513	chr1	225403605	225427243	LBR
2341	chr13	113161627	113254914	CUL4A
7088	chr5	69093823	69111075	SLC30A5
8749	chr8_KI270816v1_alt	61022	65190	MROH6
104	chr1	144410117	144414587	RNVU1-15
1288	chr10	92592045	92598844	KIF11
3462	chr16	87400231	87409890	MAP1LC3B
4261	chr19	2776449	2784979	SGTA
6646	chr4	82494668	82498094	TMEM150C
4198	chr19	1566538	1568681	MEX3D

5262	chr2	99632501	99633642	REV1
2650	chr14	45127663	45139285	FKBP3
1579	chr11	64091151	64103932	FLRT1
6078	chr3	189275084	189278565	TPRG1-AS2
5384	chr20	45941829	45959023	PCIF1
125	chr1	146037621	146040476	RNVU1-6
6704	chr5	109014718	109026059	FER
6879	chr5	163502937	163505109	MAT2B
9327	chrX	49170918	49178213	PLP2
4679	chr2	13651918	13654136	LOC100506474
5797	chr3	10197726	10201560	IRAK2
1349	chr11	108171725	108173307	NPAT
8222	chr7	45742063	45772207	SEPT7P2
3329	chr16	4914955	4917947	PPL
7845	chr6_GL000252v2_alt	4514401	4521846	B3GALT4
3085	chr15	89302807	89325950	MIR6766
20	chr1	108107026	108108793	SLC25A24
6546	chr4	40050618	40059500	N4BP2
8594	chr8	33511623	33514669	TTI2
1980	chr12	30417489	30419644	IPO8
2647	chr14	44948104	44965944	KLHL28
4647	chr2	118185686	118187255	LOC100506797
4252	chr19	2227578	2255665	SF3A2
6828	chr5	142470563	142473548	SPRY4-AS1
2018	chr12	46355735	46374734	SLC38A2
7636	chr6	41939880	41942968	CCND3
2376	chr13	27048101	27173852	USP12-AS1
4687	chr2	144329345	144334182	GTDC1
6390	chr4	13905754	13907188	LINC01085
9116	chr9	89307013	89369210	SECISBP2
283	chr1	164770963	164772705	LOC100505795
2283	chr12	95785773	95792075	NTN4
476	chr1	216600345	216602446	ESRRG
8893	chr9	129802285	129805770	TOR1B
7250	chr6	108290521	108298397	AFG1L
621	chr1	25332062	25345859	TMEM50A
1533	chr11	57739999	57753022	SELENOH
3428	chr16	74380873	74396081	NPIPB15
9109	chr9	86097529	86101205	GOLM1
9076	chr9	70417464	70422081	KLF9
3027	chr15	72364935	72378658	HEXA-AS1
93	chr1	120940828	120947488	RNVU1-4
8192	chr7	35686695	35701212	HERPUD2
7731	chr6	7539228	7579169	DSP

6110	chr3	195913644	195916123	TNK2
5882	chr3	127775449	127824602	MGLL
6200	chr3	47280054	47284428	KLHL18
7920	chr6_GL000256v2_alt	1904200	1916829	PPP1R10
4915	chr2	226180373	226187279	LOC646736
1233	chr10	77345341	77352235	KCNMA1
7851	chr6_GL000253v2_alt	1864322	1879284	GNL1
4970	chr2	241233108	241243599	HDLBP
7018	chr5	43446507	43486698	TMEM267
6280	chr3	71059995	71066242	FOXP1
9017	chr9	37470601	37473057	ZBTB5
8202	chr7	40898308	40900929	LINC01450
1126	chr10	42781761	42795432	BMS1
3740	chr17	47896361	47926391	SP2
4380	chr19	44024365	44032470	ZNF222
8834	chr9	124865659	124876188	ARPC5L
4697	chr2	150616779	150626676	LOC101929282
4659	chr2	127637117	127639486	GPR17
4708	chr2	156331590	156335806	NR4A2
4699	chr2	150910615	150912835	LOC101929282
1018	chr10	122111533	122118104	TACC2
4999	chr2	27751698	27787055	MRPL33
1063	chr10	17228583	17240921	VIM-AS1
1125	chr10	42634370	42640574	ZNF33B
9380	chrX	80707154	80710835	BRWD3
4301	chr19	36525267	36529456	ZNF260
9384	chrX	96684763	96687078	DIAPH2
2727	chr14	67587895	67590209	PIGH
6818	chr5	141609781	141639715	DIAPH1
4227	chr19	18318949	18324907	LSM4
597	chr1	24320107	24324922	GRHL3
5147	chr2	65423667	65438444	SPRED2
3061	chr15	79877169	79898869	MTHFS
4831	chr2	201147688	201155693	CFLAR-AS1
2869	chr15	40693398	40696369	RAD51-AS1
5034	chr2	36592080	36598866	FEZ2
3270	chr16	30536057	30538343	ZNF747
516	chr1	225654585	225656339	ENAH
3570	chr17	2697089	2713379	CLUH
3325	chr16	4831511	4854811	GLYR1
6963	chr5	2148150	2151155	LOC100506858
1276	chr10	90917429	90921752	ANKRD1
2680	chr14	52785995	52793319	GNPNAT1
8187	chr7	32884367	32894196	KBTBD2

4853	chr2	20498874	20503616	RHOB
3687	chr17	43387545	43389973	LINC00910
6470	chr4	182815223	182824150	DCTD
2095	chr12	54287260	54289538	NFE2
8139	chr7	22716861	22719050	IL6
4247	chr19	19774021	19777919	LINC00663
883	chr1	87333304	87358669	LMO4
4665	chr2	130371747	130375996	PTPN18
1229	chr10	75208631	75235154	VDAC2
6943	chr5	180327480	180332333	GFPT2
3463	chr16	87516372	87518665	LOC101928737
2144	chr12	6279119	6281158	PLEKHG6
2342	chr13	113293011	113301447	LAMP1
8470	chr8	127732135	127744854	MYC
8439	chr8	121697013	121700434	HAS2
5761	chr22	47122872	47129346	TBC1D22A-AS1
2989	chr15	65691703	65733346	MIR4511
5157	chr2	68463553	68483708	APLF
4483	chr19	55113939	55119588	PPP1R12C
714	chr1	40159762	40177173	RLF
1287	chr10	92565326	92573605	IDE
3891	chr17	75203641	75211222	NUP85
7153	chr5	82264485	82278129	RPS23
3582	chr17	28752753	28758995	TRAF4
4875	chr2	208024086	208026808	PLEKHM3
2348	chr13	114061574	114066811	RASA3
7008	chr5	40409501	40412798	PTGER4
4309	chr19	37931811	37938559	SIPA1L3
23	chr1	108658466	108664526	HENMT1
7746	chr6	81450488	81451367	TENT5A
1693	chr11	83049525	83101307	RAB30-AS1
8716	chr8	93710974	93742329	RBM12B-AS1
3383	chr16	67935412	67945547	PSMB10
5400	chr20	49654728	49716074	B4GALT5
8045	chr7	135147151	135149826	TMEM140
5279	chr20	18287037	18290825	ZNF133
8871	chr9	128503815	128512712	GLE1
6073	chr3	187137671	187140194	RPL39L
2766	chr14	73785025	73789348	ELMSAN1
3162	chr16	1490826	1498258	TELO2
2388	chr13	30718662	30720261	ALOX5AP
4987	chr2	27210880	27229360	CAD
9218	chrX	130098224	130110976	ELF4
6693	chr5	104183189	104192335	NUDT12

5342	chr20	37487916	37531359	BLCAP
8099	chr7	152095402	152147881	GALNT11
3829	chr17	64073495	64084364	ERN1
3853	chr17	68266628	68269740	ARSG
7007	chr5	40382276	40386123	PTGER4
3577	chr17	28596840	28601164	SPAG5
5850	chr3	121770910	121836355	IQCB1
3379	chr16	67562505	67567181	CTCF
5300	chr20	31709561	31724913	ABALON
5199	chr2	81480125	81523685	LINC01815
5295	chr20	2830779	2841808	VPS16
147	chr1	148612032	148681500	NBPF14
7672	chr6	49549027	49565350	C6orf141
1645	chr11	67461115	67470449	TMEM134
4731	chr2	167139597	167141987	XIRP2-AS1
4757	chr2	176104024	176108238	HOXD11
8191	chr7	34851569	34853592	NPSR1-AS1
5476	chr20	63743222	63745485	SLC2A4RG
4045	chr18	22550986	22553169	CTAGE1
6626	chr4	77048646	77075382	CCNI
1377	chr11	118399181	118414892	ATP5MG
7738	chr6	7883388	7911532	TXNDC5
1607	chr11	65564048	65577701	SSSCA1
8558	chr8	23010718	23071492	LOC286059
7629	chr6	3976375	4031137	PRPF4B
3112	chr15	97688664	97693240	LOC101927310
6240	chr3	49939317	49945065	RBM6
2292	chr12	98508690	98520698	TMPO
4250	chr19	21142267	21155313	ZNF431
415	chr1	203861357	203882060	SNRPE
2438	chr13	45985867	46057875	ZC3H13
4785	chr2	189203763	189205355	COL5A2
3063	chr15	80693019	80704493	ABHD17C
2992	chr15	66331082	66334316	SCARNA14
2979	chr15	64458867	64472060	ZNF609
3502	chr16_KI270854v1_alt	118579	134116	LINC00235
7476	chr6	18211096	18214926	DEK
2006	chr12	42430922	42454110	PRICKLE1
3642	chr17	39460062	39470584	CDK12
2418	chr13	41184505	41197235	KBTBD7
5148	chr2	65576165	65578492	SPRED2
1027	chr10	125082125	125160912	CTBP2
3194	chr16	20888995	20917260	LYRM1
2938	chr15	55331715	55342052	PIGBOS1

9101	chr9	81687241	81689076	TLE1
3413	chr16	71276866	71290494	CMTR2
4710	chr2	156433539	156440682	GPD2
8908	chr9	132346636	132356492	SETX
3537	chr17	1827880	1832009	RPA1
3090	chr15	89996313	89999514	ZNF710
8360	chr7	93591019	93673209	CALCR
5310	chr20	34362100	34385287	ITCH
6147	chr3	30537863	30540170	LINC01985
1787	chr12	106286978	106289916	TCP11L2
8674	chr8	67164961	67169760	CSPP1
379	chr1	192245772	192248313	RGS21
6331	chr4	109079260	109080543	COL25A1
7829	chr6_GL000252v2_alt	1993218	1999956	FLOT1
7036	chr5	5345295	5348261	ICE1
556	chr1	231827360	231828271	DISC2
651	chr1	28436597	28442660	PHACTR4
32	chr1	109195704	109199240	KIAA1324
1272	chr10	89413502	89425250	IFIT5
8302	chr7	74251850	74259249	RFC2
2740	chr14	68545524	68550134	LOC100996664
643	chr1	27828854	27833746	PPP1R8
6380	chr4	1288413	1323711	MAEA
3026	chr15	72235606	72274615	PARP6
4115	chr18	67627907	67630274	DSEL
9034	chr9	40500617	40505236	LOC102724580
9353	chrX	69164033	69166096	PJA1
2007	chr12	4319257	4322045	TIGAR
8906	chr9	131371826	131374670	PRRC2B
5648	chr22	29602004	29614205	NF2
6672	chr4	94756899	94778231	BMPR1B
9193	chrX	118343934	118358159	WDR44
6970	chr5	27314901	27317288	PURPL
29	chr1	108992154	108993198	CLCC1
1809	chr12	110340910	110355445	ANAPC7
6122	chr3	196866452	196869258	SENP5
2498	chr13	76990748	77028111	CLN5
8309	chr7	75407740	75448077	NSUN5P1
5644	chr22	28739672	28745693	HSCB
5989	chr3	155931059	155935295	GMPS
3644	chr17	39635068	39638060	STARD3
3465	chr16	87689210	87691105	KLHDC4
4534	chr19	7068572	7071827	ZNF557
9361	chrX	71419177	71444854	TAF1

2845	chr15	30622243	30641823	ARHGAP11B
1733	chr11	94188189	94190802	PANX1
9369	chrX	74172933	74174700	MIR421
8181	chr7	30547518	30551621	LOC401320
6695	chr5	1044648	1068571	MIR4635
2911	chr15	45277159	45280681	LOC101928414
3708	chr17	45056410	45069241	NMT1
4248	chr19	2052848	2063368	MKNK2
1498	chr11	3841205	3842283	RHOG
3052	chr15	76899402	76906646	SCAPER
8446	chr8	123180917	123184066	FAM83A
7587	chr6	33588397	33592515	LINC00336
6333	chr4	109550394	109557410	MCUB
1438	chr11	14442746	14447234	COPB1
4459	chr19	4928565	4938841	MIR4747
3858	chr17	68773838	68776151	LINC01482
1661	chr11	70370832	70391307	CTTN
4881	chr2	210556571	210639343	CPS1
2124	chr12	57117849	57120018	STAT6
7003	chr5	38919175	38952737	OSMR
3097	chr15	90920016	90926599	UNC45A
5069	chr2	45608609	45612224	SRBD1
3553	chr17	19640211	19659045	ALDH3A2
730	chr1	43469957	43478224	HYI
5791	chr3	101559874	101565247	TRMT10C
493	chr1	221828441	221830959	LINC01655
1904	chr12	12610233	12614920	CREBL2
8833	chr9	124817212	124820297	OLFML2A
5247	chr2	96832773	96835891	ANKRD23
1552	chr11	61915743	61918788	RAB3IL1
1874	chr12	122076983	122086798	MLXIP
9204	chrX	119928407	119946168	NKAP
3638	chr17	3884185	3892566	CAMKK1
1831	chr12	113297909	113300989	MIR6762
1470	chr11	2897018	2899956	SLC22A18
7453	chr6	161241591	161275739	AGPAT4
5483	chr21	10014465	10015130	LINC01667
1553	chr11	61948481	61952811	BEST1
8713	chr8	9074241	9076516	SNORD3I
1593	chr11	64873578	64882771	EHD1
4044	chr18	22467195	22469352	CTAGE1
2304	chr13	101411372	101417903	NALCN
4220	chr19	18020124	18025073	ARRDC2
7193	chr5	96399382	96408435	PCSK1

346	chr1	179228070	179230845	ABL2
1622	chr11	66019921	66022301	CATSPER1
4173	chr19	12936729	12942914	MIR6515
5387	chr20	46676794	46691286	SLC13A3
3900	chr17	75774463	75784685	H3F3B
2886	chr15	42203992	42208704	VPS39
439	chr1	207319472	207379495	CD55
7159	chr5	84380312	84383274	EDIL3
2121	chr12	57075692	57080368	NEMP1
8750	chr8_KI270821v1_alt	310073	312959	MIR7160
9081	chr9	72345469	72367524	ZFAND5
4129	chr18	8703768	8705975	GACAT2
1879	chr12	122497851	122502839	ZCCHC8
4666	chr2	130797159	130798586	AMER3
6257	chr3	52196949	52212587	ALAS1
9241	chrX	149914267	149941614	HSFX4
4557	chr19	9618212	9623665	ZNF561
5678	chr22	35647697	35651109	APOL6
4866	chr2	207332926	207337800	MIR1302-4
7644	chr6	43003122	43015775	MEA1
3107	chr15	94229712	94237630	MCTP2
1902	chr12	124937588	124941382	MIR5188
5138	chr2	64863582	64865644	LINC01800
6824	chr5	142023661	142026361	GNPDA1
4739	chr2	171763866	171792516	DYNC1I2
8079	chr7	149567767	149585342	ZNF767P
2750	chr14	69995464	70003849	SNORD169
2483	chr13	73325350	73326797	LINC00392
2900	chr15	44281652	44290969	CASC4
1974	chr12	2873894	2906037	TULP3
614	chr1	24745512	24749870	CLIC4
6277	chr3	63910746	63921730	SCAANT1
381	chr1	192738324	192740195	MIR4426
7117	chr5	74622570	74642707	HEXB
8123	chr7	17369571	17373757	KCCAT333
3176	chr16	1780408	1791872	NUBP2
6188	chr3	44746535	44765533	KIAA1143
3699	chr17	44344459	44354132	GRN
3887	chr17	75086255	75095090	SLC16A5
3815	chr17	62037492	62065841	MED13
5113	chr2	57196890	57198631	VRK2
3031	chr15	72768647	72784465	ADPGK-AS1
6100	chr3	194496147	194501458	LINC00884
4959	chr2	238643322	238645865	LINC01107

4618	chr2	110204254	110213139	NPHP1
3732	chr17	4730438	4736285	MED11
9382	chrX	8272786	8275391	VCX2
2231	chr12	78703029	78706202	SYT1
4412	chr19	46068667	46070135	LOC400706
6400	chr4	139665047	139668030	MGST2
3612	chr17	32338992	32342684	C17orf75
7501	chr6	26182356	26183458	HIST1H2BE
4610	chr2	105402152	105405483	FHL2
1243	chr10	79148095	79163957	ZMIZ1
5746	chr22	45125373	45190686	NUP50-DT
8268	chr7	66113449	66129943	CRCP
2036	chr12	48998437	49062594	PRKAG1
1690	chr11	803157	814840	RPLP2
9063	chr9	65871030	65874675	LOC101929583
3500	chr16_KI270853v1_alt	812320	828650	PDXDC1
3825	chr17	63739934	63744078	STRADA
7544	chr6	2834107	2843275	SERPINB1
4124	chr18	76563519	76567861	LINC00908
6481	chr4	1854500	1859906	LETM1
3954	chr17	81290911	81296499	SLC38A10
235	chr1	156327875	156339787	TSACC
2199	chr12	7090071	7113503	C1RL-AS1
4614	chr2	108718080	108737848	RANBP2
5664	chr22	30883361	30920121	LOC107985544
4085	chr18	48001759	48005364	SMAD2
4488	chr19	55352617	55354129	COX6B2
6800	chr5	139516876	139519591	TMEM173
7903	chr6_GL000255v2_alt	249824	252241	HCG15
6393	chr4	13919775	13922437	LINC01085
7509	chr6	26519304	26522439	HCG11
6703	chr5	108918596	108951414	FER
5133	chr2	64626722	64659170	SERTAD2
1786	chr12	106239377	106247723	CKAP4
8308	chr7	75355352	75376935	STAG3L1
1781	chr12	105105859	105124375	WASHC4
4498	chr19	56308033	56317218	ZSCAN5A
1167	chr10	63132123	63138654	NRBF2
7014	chr5	43006494	43010101	LOC648987
3576	chr17	28566738	28572724	PIGS
9028	chr9	38597004	38621339	ANKRD18A
2831	chr15	101490253	101493802	PCSK6
722	chr1	42656855	42660881	PPIH
4629	chr2	111859468	111885653	ANAPC1

606	chr1	244854240	244867044	HNRNPU
7878	chr6_GL000254v2_alt	22648	25506	LINC01623
5785	chr3	100334038	100341869	NIT2
4806	chr2	197425397	197436610	SF3B1
8699	chr8	8225789	8246776	FAM86B3P
3469	chr16	87805813	87807806	LOC102724467
860	chr1	8044885	8049806	ERRFI1
8366	chr7	96297430	96323217	SLC25A13
3172	chr16	15886752	15889812	FOPNL
1236	chr10	77856211	77874723	DLG5
336	chr1	174987456	175025590	CACYBP
3435	chr16	75231946	75267164	BCAR1
5485	chr21	14366991	14385804	HSPA13
3272	chr16	30600764	30611348	ZNF689
836	chr1	67802743	67833310	GNG12-AS1
4033	chr18	12878492	12892641	PTPN2
2204	chr12	7214295	7218545	PEX5
8103	chr7	1531325	1550229	LOC100128653
3134	chr15_KI270905v1_alt	4826404	4832320	LOC101928042
1527	chr11	5677093	5686306	TRIM5
3498	chr16_KI270853v1_alt	573347	614214	RRN3
3200	chr16	21788979	21793046	RRN3P1
804	chr1	61056491	61058386	NFIA
6320	chr4	102497469	102570791	LOC105377621
318	chr1	171540127	171556108	PRRC2C
1251	chr10	82349753	82352801	NRG3-AS1
2711	chr14	63530920	63547065	PPP2R5E
6221	chr3	48843794	48849351	PRKAR2A
8317	chr7	7634658	7643306	UMAD1
3533	chr17	17838492	17841998	SREBF1
9301	chrX	45739078	45776614	MIR222
7351	chr6	137863729	137885734	LOC100130476
753	chr1	45670828	45691691	GPBP1L1
8087	chr7	151230773	151246013	MIR671
6810	chr5	140685553	140700047	HARS
1646	chr11	67481960	67483560	AIP
4753	chr2	174390606	174400579	CIR1
4824	chr2	200780181	200783530	BZW1
6806	chr5	140298929	140304510	PFDN1
3758	chr17	49669469	49679073	SPOP
4167	chr19	12665551	12671071	WDR83OS
1855	chr12	120316262	120318702	PLA2G1B
2042	chr12	49364593	49374335	SPATS2
1048	chr10	133259669	133264975	MIR202HG

1998	chr12	3449013	3456233	PRMT8
6962	chr5	21150979	21154592	GUSBP1
6198	chr3	46975595	46978013	CCDC12
1810	chr12	110393628	110405829	ANAPC7
6941	chr5	179854246	179861301	MRNIP
1900	chr12	124914862	124919036	MIR5188
3622	chr17	35573266	35580686	PEX12
4669	chr2	131102312	131108172	PLEKHB2
1999	chr12	357493	392335	CCDC77
4113	chr18	66509617	66514038	CDH19
2113	chr12	56292940	56302669	CS
8976	chr9	26893774	26950808	IFT74
2539	chr14	100978537	101006793	SNORD114-31
5662	chr22	30828885	30839371	OSBP2
3715	chr17	45228011	45231372	FMNL1
3871	chr17	72465199	72466831	LINC02003
300	chr1	167932629	167952163	MPC2
1734	chr11	94490348	94506103	ANKRD49
5510	chr21	32607005	32614947	CFAP298
4819	chr2	20047517	20053957	LAPTM4A
2387	chr13	30603008	30666921	USPL1
1930	chr12	14306518	14308931	ATF7IP
8945	chr9	137297192	137302686	NRARP
1296	chr10	94544115	94562426	HELLS
2823	chr14_GL000225v1_random	73868	75938	
5636	chr22	24481381	24483430	ADORA2A-AS1
5328	chr20	35794180	35806557	PHF20
9360	chrX	71364563	71374569	TAF1
3345	chr16	56439160	56483821	OGFOD1
955	chr10	102644693	102648809	TRIM8
4576	chr1_GL383518v1_alt	43274	48297	INTS3
9232	chrX	135520317	135521357	INTS6L-AS1
7172	chr5	91161832	91163716	LUCAT1
2772	chr14	74762545	74769511	YLPM1
3100	chr15	91015339	91023942	VPS33B
7704	chr6	6819543	6821626	LY86-AS1
3115	chr15	98644754	98660188	IRAIN
3184	chr16	19101893	19116228	ITPRIPL2
7801	chr6_GL000251v2_alt	1688875	1694774	TRIM26
6531	chr4	38665848	38706836	KLF3-AS1
5126	chr2	62506077	62507218	TMEM17
1371	chr11	117087337	117100471	SIK3
555	chr1	231516399	231560879	TSNAX

1538	chr11	59556184	59557745	MIR3162
1223	chr10	73964931	73967418	VCL
2442	chr13	47983216	48002379	SUCLA2
2799	chr14	85532281	85571470	FLRT2
5396	chr20	49128161	49135372	STAU1
9328	chrX	49878331	49880707	USP27X-AS1
1988	chr12	31658469	31662339	ETFBKMT
972	chr10	106507109	106509907	LOC105378470
4326	chr19	3950837	3970206	MIR637
505	chr1	223987166	224025160	LOC100287497
8605	chr8	38526271	38529602	C8orf86
2099	chr12	55810482	55833881	ORMDL2
8727	chr8	94716468	94723555	DPY19L4
7910	chr6_GL000255v2_alt	3395934	3403090	AGPAT1
5624	chr22	20508410	20512699	MED15
3018	chr15	71112246	71135206	CT62
6084	chr3	189913889	189916476	MIR944
5812	chr3	11135935	11139182	HRH1
9077	chr9	71906646	71928165	C9orf85
8756	chr9	100411044	100412635	MSANTD3
7368	chr6	142374689	142418545	ADGRG6
5322	chr20	35540826	35546078	ERGIC3
6088	chr3	190230678	190232930	CLDN1
8881	chr9	129110702	129112526	PTPA
6545	chr4	39967507	39979191	PDS5A
9053	chr9	62892342	62908733	LOC403323
4060	chr18	32088801	32097689	RNF138
6313	chr3_KI270935v1_alt	4792	9437	LOC105374297
2525	chr13	99189406	99203364	UBAC2
921	chr1	94322274	94327025	ARHGAP29
5997	chr3	157087066	157090136	LINC00881
7683	chr6	53732937	53736387	LRRC1
8714	chr8	91057557	91095485	OTUD6B
4442	chr19	48252688	48256641	CARD8-AS1
8214	chr7	44199526	44202981	YKT6
7620	chr6	37254148	37272214	TMEM217
768	chr1	51760445	51768264	OSBPL9
5519	chr21	34374086	34381953	SMIM11B
6227	chr3	49102534	49105607	QARS
5093	chr2	52933119	52935466	MIR4431
1682	chr11	76781845	76785246	TSKU
4648	chr2	119360500	119371711	C2orf76
5939	chr3	142556405	142581365	ATR
6120	chr3	196739342	196753349	PAK2

7853	chr6_GL000253v2_alt	2028597	2037627	TUBB
5005	chr2	28809942	28822682	SPDYA
2962	chr15	62383549	62391815	MIR6085
7787	chr6_GL000250v2_alt	1315837	1329769	PPP1R11
1255	chr10	86514971	86521861	WAPL
3766	chr17	50354772	50374677	MRPL27
3711	chr17	45124925	45133988	PLCD3
288	chr1	165761748	165771990	TMCO1
7601	chr6	35480655	35487148	TEAD3
1549	chr11	61398855	61401333	TMEM216
4324	chr19	39429652	39437653	RPS16
2880	chr15	41537832	41545288	RPAP1
5221	chr2	8679176	8681715	ID2-AS1
2600	chr14	24298217	24320975	LTB4R2
1602	chr11	6540103	6553163	DNHD1
6764	chr5	133984385	134009437	VDAC1
1225	chr10	74144318	74154337	AP3M1
6402	chr4	140147545	140153316	MAML3
7138	chr5	79233196	79247965	JMY
4095	chr18	5236002	5248752	LINC00667
9167	chrX	101096679	101103054	CENPI
6423	chr4	153153163	153154319	TRIM2
5685	chr22	37430187	37433677	ELFN2
8528	chr8	144992163	145004522	TMED10P1
7685	chr6	54714539	54802777	FAM83B
77	chr1	117927509	117944770	WDR3
6595	chr4	6691907	6698010	S100P
6268	chr3	57552268	57559656	PDE12
5954	chr3	149366083	149368094	TM4SF1-AS1
3268	chr16	30442071	30446973	SEPHS2
3221	chr16	2531419	2541325	PDPK1
3894	chr17	75280843	75291088	SLC25A19
6651	chr4	83183435	83185870	PLAC8
9096	chr9	78019402	78034641	GNAQ
5504	chr21	30769358	30771819	KRTAP21-1
1560	chr11	62587355	62593587	TUT1
846	chr1	75372316	75376851	SLC44A5
5878	chr3	126910428	126913568	PLXNA1
2210	chr12	7495656	7500642	CD163
3959	chr17	8139919	8158354	PER1
1601	chr11	65384801	65388217	FRMD8
3606	chr17	31347072	31351992	EVI2A
3132	chr15_KI270905v1_alt	3500180	3503613	LINC02352
4293	chr19	35757269	35760617	PROSER3

7992	chr7	116210767	116264879	TES
4967	chr2	24063683	24080105	SF3B6
3691	chr17	4364158	4366798	UBE2G1
2912	chr15	45400465	45411829	SPATA5L1
1346	chr11	10749045	10757462	CTR9
7134	chr5	78677361	78679869	LHFPL2
3238	chr16	28552279	28556501	SGF29
5559	chr21	41677248	41680180	LINC00111
2053	chr12	50244154	50250306	MIR1293
7481	chr6	20400346	20423601	E2F3
765	chr1	51510632	51519196	EPS15
3003	chr15	67839507	67842130	RNU6-8
1417	chr11	128204293	128210854	LINC02098
4522	chr19	58557323	58565233	MZF1-AS1
2276	chr12	945629	950701	RAD52
5883	chr3	128072068	128081444	RUVBL1-AS1
3643	chr17	39617170	39618895	PPP1R1B
807	chr1	62437349	62450163	USP1
183	chr1	151604997	151607590	SNX27
84	chr1	119711865	119748547	PHGDH
7944	chr7	100675987	100682114	GNB2
6944	chr5	180439830	180447920	CNOT6
7229	chr6	10308648	10311919	TFAP2A-AS2
2532	chr14	100278393	100286946	MIR6764
942	chr10	101031842	101035005	SFXN3
6404	chr4	141220085	141233642	ZNF330
8829	chr9	122898192	122917673	RC3H2
361	chr1	183158864	183161885	LAMC1-AS1
1515	chr11	47165258	47178050	ARFGAP2
7897	chr6_GL000255v2_alt	1973382	1982839	TUBB
4902	chr2	219273399	219276172	DNAJB2
1771	chr12	103836842	103843444	NT5DC3
7653	chr6	4350544	4353917	
7403	chr6	149745388	149747707	NUP43
5345	chr20	3794599	3800693	CDC25B
7295	chr6	12322975	12326073	EDN1
1525	chr11	47904533	47907040	NUP160
1817	chr12	11149786	11183521	SMIM10L1
3585	chr17	2892888	2895428	LOC101927911
2178	chr12	6850421	6853437	CDCA3
3985	chr17	82874976	82880294	ZNF750
1200	chr10	71867179	71869957	PSAP
8120	chr7	16643770	16649600	BZW2
3522	chr17	16339779	16353745	CENPV

3967	chr17	81865271	81902816	ANAPC11	
7378	chr6	144263314	144266229	UTRN	
2782	chr14	77031214	77035113	IRF2BPL	
4056	chr18	29161847	29166572	CDH2	
491	chr1	221436961	221442082	C1orf140	
8461	chr8	126434123	126435734	LOC101927657	
6774	chr5	134757816	134765143	DDX46	
1953	chr12	22624136	22684583	ETNK1	
2242	chr12	85626897	85628636	RASSF9	
3886	chr17	75043987	75050485	KCTD2	
5430	chr20	51983373	51986840	ZFP64	
8556	chr8	22982341	22987905	RHOBTB2	
7110	chr5	73436892	73451574	FOXD1	
7775	chr6	89816285	89821283	MDN1	
542	chr1	229259493	229285354	LOC105373159	
7804	chr6_GL000251v2_alt	2035045	2048675	GNL1	
492	chr1	221692158	221748998	DUSP10	
9174	chrX	103623141	103629531	TCEAL1	
2351	chr13	114150121	114152790	RASA3	
3679	chr17	43121086	43127709	BRCA1	
7218	chr5_KI270897v1_alt	471934	499705	SMN1	
5241	chr2	96204208	96214900	STARD7	
783	chr1	54198673	54229108	MRPL37	
6438	chr4	164340490	164351452		1-Mar
1098	chr10	3093970	3110304	PFKP	
1837	chr12	116526674	116530430	LINC00173	
7542	chr6	28320017	28321328	ZSCAN31	
3968	chr17	81914650	81918164	SIRT7	
6534	chr4	39029966	39031111	TMEM156	
1826	chr12	112115621	112117615	NAA25	
8084	chr7	151072372	151083174	FASTK	
4593	chr2	10119526	10128911	RRM2	
4125	chr18	76810251	76828821	ZNF236-DT	
6610	chr4	74036391	74041488	CXCL3	
3844	chr17	67390020	67395935	PITPNC1	
7179	chr5	94567879	94570619	KIAA0825	
3407	chr16	70298330	70308164	DDX19B	
6039	chr3	179345825	179353140	MFN1	
8599	chr8	37848002	37850671	BRF2	
7847	chr6_GL000253v2_alt	1236476	1240104	HCG9	
1950	chr12	21486648	21513950	RECQL	
1965	chr12	27242872	27251134	STK38L	
7717	chr6	7266630	7268612	SSR1	
7951	chr7	101215612	101223091	PLOD3	

7042	chr5	55533346	55536214	RNF138P1
9099	chr9	79102903	79106307	LOC101927450
7561	chr6	30057450	30062725	ZNRD1ASP
1081	chr10	24710720	24719847	ARHGAP21
7789	chr6_GL000250v2_alt	1648674	1659675	HCG17
7195	chr5	96508526	96512097	PCSK1
4262	chr19	27789240	27797169	LOC101927151
5257	chr2	99149542	99204446	MITD1
1613	chr11	6584171	6620570	RRP8
1590	chr11	64802510	64804432	MAP4K2
5064	chr2	43575236	43597929	THADA
6050	chr3	183251705	183320180	B3GNT5
7233	chr6	105598866	105603527	PREP
8324	chr7	76950349	76951976	DTX2P1-UPK3BP1-PMS2P1 1
7554	chr6	28916120	28925702	TRIM27
1362	chr11	112016150	112027655	DLAT
1336	chr11	104937509	104969253	CASP4
9108	chr9	86038170	86044933	GOLM1
281	chr1	16365395	16371884	SZRD1
8779	chr9	109115493	109119076	TMEM245
3004	chr15	68270827	68307489	FEM1B
5653	chr22	30195183	30198322	LIF-AS1
483	chr1	220035600	220047598	EPRS
7027	chr5	5145538	5165078	ADAMTS16
3193	chr16	2087148	2091710	MIR1225
4749	chr2	173352510	173358140	CDCA7
1576	chr11	6385538	6397173	SMPD1
1409	chr11	125568973	125577209	EI24
4098	chr18	54268353	54276037	POLI
3417	chr16	71800692	71811628	AP1G1
8981	chr9	31302978	31306549	LINC01243
5694	chr22	38234397	38241909	SNORA92
1970	chr12	27963960	27968292	PTHLH
7600	chr6	34882298	34895848	ANKS1A
8602	chr8	38259039	38265087	PLPP5
405	chr1	201712787	201717284	MIR5191
3073	chr15	83377874	83381469	SH3GL3
8458	chr8	125431543	125441913	TRIB1
292	chr1	166488916	166491333	LINC01675
3236	chr16	28490733	28493315	CLN3
569	chr1	234720907	234726516	LINC01132
1015	chr10	121945326	121949041	ATE1
5779	chr22_KI270879v1_alt	22790	26885	SMARCB1

1770	chr12	10212870	10220024	GABARAPL1
7659	chr6	43635029	43645867	RSPH9
5769	chr22	50318066	50330206	DENND6B
4039	chr18	14826275	14830250	MIR3156-2
9279	chrX	23883198	23885014	APOO
185	chr1	151753647	151765879	OAZ3
7116	chr5	74314994	74320193	LINC01333
5078	chr2	46696079	46771968	SOCS5
6432	chr4	158209443	158212905	TMEM144
349	chr1	179879120	179917585	TOR1AIP1
3273	chr16	30631809	30637122	PRR14
992	chr10	11459317	11467968	USP6NL
4458	chr19	49115636	49119550	C19orf73
1894	chr12	123711615	123723112	ATP6V0A2
4709	chr2	156400153	156401495	GPD2
1004	chr10	119314962	119318697	MIR4681
5371	chr20	44938505	44962425	TOMM34
4905	chr2	219683727	219685760	SLC4A3
5169	chr2	70247861	70249680	TIA1
9027	chr9	38585160	38595616	ANKRD18A
6750	chr5	132093252	132095892	CSF2
7750	chr6	82360777	82365813	TPBG
1139	chr10	49489229	49540128	PGBD3
3458	chr16	86930723	86933113	LINC02181
6353	chr4	120060349	120072561	MAD2L1
6947	chr5	181101762	181102358	OR2V1
4715	chr2	159283759	159288778	WDSUB1
8113	chr7	156627186	156647031	LINC01006
3824	chr17	63600453	63603111	TACO1
1631	chr11	66716013	66728888	SPTBN2
7516	chr6	2683038	2686236	LINC01600
9111	chr9	86312083	86356632	TUT7
78	chr1	11801545	11806056	MTHFR
7108	chr5	72953152	72965897	FCHO2
4704	chr2	152199921	152201962	STAM2
1901	chr12	124926934	124930729	MIR5188
8365	chr7	95433871	95435985	PON2
8589	chr8	30740938	30746912	UBXN8
4195	chr19	14776272	14777062	ADGRE2
842	chr1	71049304	71083239	MIR186
756	chr1	46186537	46200106	POMGNT1
1963	chr12	26918023	26951900	INTS13
5052	chr2	38874346	38877340	DHX57
4204	chr19	16323074	16330383	KLF2

6845	chr5	14860919	14877859	ANKH
4402	chr19	45580305	45586240	OPA3
938	chr10	100277964	100287487	BLOC1S2
4516	chr19	58372317	58374916	ZNF837
2356	chr13	19649248	19679270	MPHOSPH8
3058	chr15	78858997	78861780	MORF4L1
8205	chr7	41681569	41704605	INHBA-AS1
8974	chr9	22238180	22256682	DMRTA1
263	chr1	161044104	161049413	USF1
8395	chr8	101000950	101005733	FLJ42969
9214	chrX	129602920	129605197	APLN
3544	chr17	18991956	19007595	FAM83G
5215	chr2	86191776	86197624	IMMT
5205	chr2	84937651	84947474	KCMF1
7899	chr6_GL000255v2_alt	2006568	2008731	IER3
1040	chr10	132307325	132309072	STK32C
4718	chr2	160207317	160209161	ITGB6
6265	chr3	56555633	56560690	CCDC66
6062	chr3	184361564	184373829	POLR2H
65	chr1	114765481	114782474	SIKE1
5891	chr3	129170296	129189001	CNBP
6958	chr5	1945404	1968977	CTD-2194D22.4
515	chr1	225498468	225511805	LBR
3400	chr16	69423603	69426631	CYB5B
2045	chr12	49582207	49585375	MCRS1
7764	chr6	87212362	87239296	ZNF292
982	chr10	110871266	110889394	PDCD4
1888	chr12	123362453	123366892	MIR8072
2475	chr13	66939376	66941476	PCDH9-AS3
5757	chr22	46530039	46540393	CELSR1
5595	chr21	6091867	6102682	LINC00319
642	chr1	27658964	27660796	LINC02574
8274	chr7	66674885	66690640	RABGEF1
5445	chr20	56785525	56790216	TFAP2C
7126	chr5	76812732	76823934	F2RL1
5659	chr22	30667080	30669296	DUSP18
2375	chr13	26199065	26223920	RNF6
414	chr1	203794270	203842578	ZBED6
7652	chr6	43480788	43509066	TJAP1
2633	chr14	36289081	36319759	MBIP
2288	chr12	96402973	96417672	CDK17
2814	chr14	96498397	96584603	PAPOLA
8778	chr9	109106082	109112617	TMEM245
6767	chr5	134363558	134369487	CDKL3

8905	chr9	131124303	131130941	NUP214
9007	chr9	35657218	35659927	CCDC107
4780	chr2	186477200	186528268	ZC3H15
2022	chr12	46515438	46532318	LOC100288798
6692	chr5	104079338	104173296	NUDT12
7420	chr6	151450352	151460588	ARMT1
8380	chr7	99495401	99501876	ZNF394
6848	chr5	149140445	149142336	ABLIM3
9342	chrX	56992658	56996476	SPIN3
4919	chr2	226826767	226851825	RHBDD1
4351	chr19	42187490	42193816	DEDD2
3411	chr16	70520242	70528639	SF3B3
7344	chr6	136249118	136291897	BCLAF1
2571	chr14	20439169	20459850	OSGEP
2967	chr15	63275873	63278233	APH1B
3405	chr16	69761504	69764751	WWP2
750	chr1	45520386	45523650	PRDX1
2207	chr12	7421558	7433064	CD163L1
3267	chr16	3043416	3045579	MMP25
979	chr10	110409223	110412043	DUSP5
4587	chr1_KI270765v1_alt	70862	74522	RNVU1-11
785	chr1	54487427	54489674	ACOT11
9333	chrX	53418697	53423517	SMC1A
628	chr1	26357954	26359502	CRYBG2
1368	chr11	114438512	114458216	REXO2
1059	chr10	17022124	17024466	CUBN
4674	chr2	135124397	135129437	SNORA40B
7270	chr6	112330081	112331990	RFPL4B
2010	chr12	43834840	43838137	TMEM117
3326	chr16	48354021	48375858	SIAH1
5475	chr20	63679578	63707773	TNFRSF6B
5875	chr3	126282725	126286676	KLF15
2479	chr13	73054836	73080782	KLF5
4926	chr2	230414794	230443701	SP100
2306	chr13	101638736	101641533	ITGBL1
7000	chr5	38657767	38661827	LINC01265
1396	chr11	122882838	122886650	JHY
1343	chr11	106823589	106827201	GUCY1A2
1703	chr11	86301047	86307272	HIKESHI
2492	chr13	74289236	74291723	LINC00402
7527	chr6	2764100	2798299	MYLK4
4335	chr19	40419838	40426920	SERTAD1
3002	chr15	67538948	67550985	MAP2K5
4525	chr19	5922530	5982438	RANBP3

3175	chr16	171445	177825	HBA2
3613	chr17	32347655	32398784	ZNF207
5736	chr22	43173806	43186780	TTLL12
8522	chr8	144533677	144536874	C8orf82
3818	chr17	62479416	62486129	TLK2
1404	chr11	124673320	124676028	SIAE
5626	chr22	20915755	20923615	CRKL
3587	chr17	29152398	29157044	MYO18A
8743	chr8	99574412	99587673	MIR875
6449	chr4	169183043	169227102	SH3RF1
7478	chr6	1878360	1919128	GMDS
7275	chr6	116246264	116257446	TSPYL4
8409	chr8	105877935	105901461	ZFPM2-AS1
2319	chr13	108203945	108217111	LIG4
8484	chr8	135456113	135458133	KHDRBS3
7605	chr6	36028037	36030475	MAPK14
3464	chr16	87612	90885	MPG
7493	chr6	25961854	25964142	TRIM38
1760	chr11_KI270831v1_alt	57348	58837	KCNQ1-AS1
3178	chr16	18398990	18401932	PKD1P5-LOC105376752
4182	chr19	1382392	1384728	NDUFS7
3029	chr15	72684228	72691564	BBS4
8903	chr9	130912765	130914494	QRFP
5167	chr2	70144048	70144587	LINC01816
3099	chr15	90959685	90977596	PRC1-AS1
1518	chr11	47268174	47277499	LOC101928943
4542	chr19	781856	783806	PTBP1
4181	chr19	13793480	13802508	ZSWIM4
645	chr1	28245159	28248181	ATP5IF1
3856	chr17	68460115	68462399	WIPI1
3668	chr17	42561110	42581514	MLX
1421	chr11	12896234	12901557	LINC00958
3006	chr15	68584854	68591224	CORO2B
1757	chr11_KI270831v1_alt	129319	131934	SLC22A18
7800	chr6_GL000251v2_alt	1535442	1542343	ZNRD1ASP
2490	chr13	74112606	74141360	KLF12
6519	chr4	34180219	34184357	LINC02484
7265	chr6	111594810	111607038	TRAF3IP2
4789	chr2	190342008	190358557	INPP1
5422	chr20	5105228	5114209	TMEM230
8398	chr8	101157584	101160825	ZNF706
7473	chr6	17615945	17620240	FAM8A1
102	chr1	143798735	143801672	LOC388692
7140	chr5	79466986	79516774	HOMER1

1735	chr11	94705679	94721361	LOC105369438
3558	chr17	21125328	21128406	DHRS7B
4019	chr17_KI270861v1_alt	139133	149449	MIR22
3478	chr16	89092956	89095779	ACSF3
6731	chr5	119321344	119404260	TNFAIP8
4292	chr19	35744456	35756680	LIN37
5847	chr3	120619600	120624524	NDUFB4
7956	chr7	101881630	101886487	CUX1
395	chr1	199741219	199744273	NR5A2
6044	chr3	180432113	180434128	LINC02053
4584	chr1_KI270763v1_alt	855974	869752	AKT3
8208	chr7	43608041	43649243	STK17A
7657	chr6	4357412	4361622	
5372	chr20	44965372	44984223	STK4
26	chr1	108815122	108817527	AKNAD1
2046	chr12	49649066	49650834	PRPF40B
2753	chr14	71317137	71345786	SIPA1L1
8717	chr8	93754137	93760220	TMEM67
8248	chr7	5689294	5693379	RNF216-IT1
4283	chr19	3491971	3518944	FZR1
1197	chr10	71772141	71774385	VSIR
5683	chr22	36507577	36531480	EIF3D
4732	chr2	168242772	168248983	STK39
1892	chr12	123600504	123628168	SNORA9B
3526	chr17	17009310	17012249	LINC02090
6475	chr4	184267310	184269285	ENPP6
2393	chr13	32584702	32614492	PDS5B
5852	chr3	122679396	122695029	PARP14
4396	chr19	45441353	45446009	MIR6088
3224	chr16	2628207	2649647	FLJ42627
596	chr1	24235737	24237225	LOC284632
8614	chr8	41826590	41829274	ANK1
6681	chr4	99816895	99895598	LAMTOR3
4415	chr19	46187561	46204062	IGFL2-AS1
2432	chr13	44986580	44994363	GPALPP1
4822	chr2	200602906	200654777	AOX1
1616	chr11	65901133	65905243	FOSL1
3053	chr15	76930310	76935443	RCN2
8433	chr8	119414706	119416928	NOV
3857	chr17	6867457	6870026	ALOX12P2
3762	chr17	50148480	50153584	PPP1R9B
1479	chr11	32890084	32931789	QSER1
6563	chr4	47458248	47464779	COMMD8
4682	chr2	142666916	142669940	KYNU

5456	chr20	59939641	59944385	FAM217B
4067	chr18	3592470	3612690	DLGAP1-AS2
5912	chr3	134426990	134428331	MIR4788
7146	chr5	80966676	80969197	RASGRF2-AS1
2898	chr15	43865446	43901776	PIN4P1
7318	chr6	132796483	132799879	SLC18B1
122	chr1	145978226	145981120	POLR3GL
6553	chr4	41157186	41161370	APBB2
4150	chr19	10956643	10962945	SMARCA4
6033	chr3	172620604	172627268	LINC02068
5125	chr2	61853409	61855537	FAM161A
4130	chr18	9112232	9166275	ANKRD12
2362	chr13	20613079	20618007	IFT88
1556	chr11	62042273	62047709	FTH1
4116	chr18	68277547	68278708	TMX3
3340	chr16	53129763	53130680	CHD9
5284	chr20	22375341	22378414	LOC284788
2193	chr12	69688253	69689905	BEST3
2183	chr12	69006665	69010602	CPM
2384	chr13	30393738	30396917	LINC00426
5574	chr21	43436067	43449805	LINC00319
7948	chr7	101098632	101101756	TRIM56
1353	chr11	108663871	108669519	DDX10
16	chr1	10636618	10639435	CASZ1
3338	chr16	53054835	53060417	CHD9
580	chr1	235907996	235912663	LYST
2812	chr14	95125724	95159617	MIR3173
61	chr1	114435579	114516420	TRIM33
7112	chr5	73549478	73576068	ANKRA2
5159	chr2	69371792	69386752	GFPT1
87	chr1	120311830	120345281	NBPF8
3701	chr17	44684575	44692825	CCDC43
1174	chr10	67790363	67792443	DNAJC12
4972	chr2	241571442	241580348	BOK-AS1
5353	chr20	405690	441075	RBCK1
4635	chr2	112771930	112779697	IL1A
2416	chr13	41058663	41061469	WBP4
7446	chr6	158949195	159005807	RSPH3
8533	chr8	1744551	1747109	LOC101927752
3196	chr16	2132070	2133671	MIR4516
5914	chr3	135511943	135515743	PPP2R3A
202	chr1	153801646	153806919	LOC343052
1819	chr12	111682387	111693510	ACAD10
7507	chr6	26350671	26352637	BTN3A2

8914	chr9	133140870	133150304	RALGDS
3764	chr17	50289341	50291205	TMEM92-AS1
9332	chrX	53211751	53226755	KDM5C
3131	chr15_KI270905v1_alt	3273453	3279432	MTMR10
4653	chr2	12574533	12590410	TRIB2
7716	chr6	72622172	72638852	KCNQ5-IT1
5132	chr2	64522777	64524930	LOC101927402
7235	chr6	106133790	106136148	PRDM1
8013	chr7	126697243	126702623	MIR592
7055	chr5	59032591	59037717	PDE4D
291	chr1	16640682	16647138	MST1P2
298	chr1	167714388	167715860	MPZL1
1780	chr12	104956250	104960090	SLC41A2
3772	chr17	50859253	50868952	TOB1
1201	chr10	71886396	71891019	PSAP
56	chr1	113557741	113562072	MAGI3
7534	chr6	27884962	27903768	HIST1H2BO
789	chr1	55442132	55445502	MIR4422
2810	chr14	93180715	93188270	MOAP1
4622	chr2	111031825	111035196	ACOXL-AS1
7474	chr6	17976842	17990581	KIF13A
4133	chr18	9580517	9618122	PPP4R1
8314	chr7	76165362	76168159	SRRM3
7728	chr6	7504774	7506989	DSP
1265	chr10	88150974	88154803	PTEN
7709	chr6	7057272	7059860	RREB1
1254	chr10	85641036	85644231	GRID1-AS1
240	chr1	156646677	156661645	BCAN
4590	chr2	10037035	10044722	LOC101929882
4062	chr18	3446294	3465685	TGIF1
6079	chr3	189322983	189326145	TPRG1-AS2
1451	chr11	18553046	18554043	UEVLD
3305	chr16	3711997	3722022	TRAP1
6068	chr3	185908357	185939049	TRA2B
5617	chr22	19413466	19453188	MRPL40
6322	chr4	103018004	103020971	SLC9B1
1704	chr11	86522479	86525526	ME3
9219	chrX	130113495	130116507	ELF4
4138	chr18_GL383567v1_alt	15072	17559	SKA1
6794	chr5	138552933	138575022	SNORD63
8096	chr7	1518227	1521790	MAFK
3484	chr16	89559133	89559601	RPL13
2357	chr13	19780457	19783948	PSPC1
3452	chr16	85025949	85031137	KIAA0513

4694	chr2	150463689	150481125	RND3
179	chr1	151253243	151274574	PSMD4
3977	chr17	82286728	82290500	LINC01970
6258	chr3	52250808	52280124	MIRLET7G
3461	chr16	87386666	87390976	FBXO31
8701	chr8	85207866	85221177	C8orf59
4860	chr2	20646658	20652301	HS1BP3
8694	chr8	80483667	80497755	ZBTB10
8733	chr8	96247861	96262439	MTERF3
1824	chr12	112007106	112021945	ERP29
9271	chrX	20136727	20143800	EIF1AX-AS1
7316	chr6	1324005	1326484	FOXQ1
7379	chr6	144285138	144291768	UTRN
7363	chr6	140050671	140052628	LOC103352541
8434	chr8	119827681	119838848	TAF2
1021	chr10	124071889	124094687	CHST15
8086	chr7	1511637	1514487	INTS1
1240	chr10	78032431	78047217	RPS24
322	chr1	17208473	17209679	PADI1
2244	chr12	86711020	86714143	MGAT4C
9151	chr9	97801243	97803578	PTCSC2
162	chr1	150291502	150311231	MRPS21
4456	chr19	49084088	49088106	SNRNP70
3777	chr17	51177337	51187792	NME2
2867	chr15	40568368	40577105	RPUSD2
814	chr1	63589689	63596404	PGM1
5465	chr20	62621008	62624703	SLCO4A1
6323	chr4	10431591	10457370	ZNF518B
9344	chrX	57894070	57911772	ZXDA
6306	chr3	99636659	99638558	COL8A1
5621	chr22	20114163	20122336	RANBP1
2762	chr14	73564032	73569756	ACOT2
6543	chr4	39634353	39641181	SMIM14
7090	chr5	69233679	69256276	CDK7
4185	chr19	13861276	13863125	NANOS3
6880	chr5	163508177	163510876	MAT2B
5347	chr20	3803592	3812575	LINC01730
410	chr1	203006053	203009932	TMEM183A
4797	chr2	191676049	191726292	LOC105747689
2601	chr14	24434152	24448272	SDR39U1
8740	chr8	98819129	98827214	STK3
3355	chr16	57299286	57301149	PLLP
3093	chr15	90387096	90410971	IQGAP1
8121	chr7	17233552	17235472	AHR

725	chr1	42931804	42960517	SLC2A1-AS1
9236	chrX	1367259	1402479	SLC25A6
2024	chr12	47072642	47102322	AMIGO2
3964	chr17	8174306	8178439	TMEM107
8078	chr7	149540251	149546987	ZNF746
8299	chr7	74001244	74012925	ELN
666	chr1	31943258	31947275	PTP4A2
9065	chr9	6679026	6683928	GLDC
7349	chr6	137148101	137149741	IL22RA2
2714	chr14	64334762	64339887	ESR2
6500	chr4	23939007	23947562	PPARGC1A
4553	chr19	9322839	9341145	ZNF559-ZNF177
8843	chr9	125702391	125709009	MAPKAP1
9146	chr9	96415622	96421726	ZNF367
4196	chr19	15124439	15126221	ILVBL
3387	chr16	68236170	68239341	ESRP2
7650	chr6	43426715	43429882	ABCC10
3940	chr17	80258276	80265241	RNF213
1044	chr10	132903359	132906175	CFAP46
4942	chr2	23379696	23382271	KLHL29
7676	chr6	5242850	5269522	LYRM4
5204	chr2	84904196	84909877	TMSB10
4619	chr2	110353273	110357289	LINC01123
7938	chr7	100106763	100109187	MCM7
4398	chr19	45454525	45457364	FOSB
6779	chr5	135327618	135332444	H2AFY
1660	chr11	702878	707465	EPS8L2
8376	chr7	99323116	99329463	ARPC1A
7009	chr5	40676805	40678844	PTGER4
2287	chr12	96196977	96228471	ELK3
2558	chr14	104687819	104691891	INF2
9045	chr9	5876501	5881094	MLANA
8094	chr7	151755980	151756899	PRKAG2
1636	chr11	67031167	67032863	SYT12
5057	chr2	39994879	39998557	SLC8A1-AS1
3406	chr16	70112186	70120307	PDPR
6597	chr4	67430208	67447082	LOC101927237
9037	chr9	42723595	42727868	XLOC_007697
357	chr1	181086923	181097891	IER5
3862	chr17	7033527	7038921	SLC16A13
461	chr1	212015045	212054532	DTL
4232	chr19	18517837	18522905	ELL
3994	chr17	9801147	9803985	DHRS7C
5403	chr20	49907156	49917550	SPATA2

9372	chrX	75152160	75157833	ABCB7
4012	chr17_KI270857v1_alt	2798652	2806357	PSMB3
6133	chr3	19939863	19999711	RAB5A
8340	chr7	87150978	87212921	DMTF1
7415	chr6	150863526	150870978	MTHFD1L
5699	chr22	38754396	38758272	SUN2
1075	chr10	22434763	22444415	LOC100499489
5500	chr21	29056794	29074981	CCT8
3396	chr16	69131345	69144363	UTP4
2792	chr14	81371373	81403272	STON2
8212	chr7	44002065	44016778	SPDYE1
4600	chr2	10281771	10284678	HPCAL1
2477	chr13	72715975	72730808	BORA
7263	chr6	111329125	111344124	MFSD4B
8965	chr9	21615158	21617784	MIR31HG
2396	chr13	33493857	33496349	LINC02344
6343	chr4	114014442	114017465	ARSJ
2136	chr12	59053987	59057509	LRIG3
4306	chr19	37076371	37079410	ZNF420
887	chr1	88773407	88778802	PKN2-AS1
6105	chr3	194729290	194731424	LINC01968
3876	chr17	7322718	7330984	NEURL4
6637	chr4	7968839	7976749	MIR95
4404	chr19	45639777	45644161	EML2-AS1
2697	chr14	58242339	58249083	PSMA3
8817	chr9	120574979	120581232	CDK5RAP2
4017	chr17_KI270857v1_alt	835856	840424	MRM1
120	chr1	145955547	145959703	LIX1L
4201	chr19	16072784	16087559	TPM4
5713	chr22	41090057	41105703	EP300
165	chr1	150486122	150491989	TARS2
6330	chr4	108619082	108649724	RPL34
4532	chr19	679031	685797	FSTL3
6783	chr5	136011027	136012942	TGFBI
5564	chr21	42513658	42519949	SLC37A1
1603	chr11	65413025	65422692	NEAT1
8735	chr8	97642502	97651136	MTDH
1687	chr11	78126469	78141885	ALG8
2557	chr14	104682165	104683418	MIR4710
8196	chr7	37665114	37668405	GPR141
9129	chr9	93093775	93097304	CARD19
4466	chr19	49870482	49880824	AKT1S1
9326	chrX	49166330	49168994	PLP2
6847	chr5	149133411	149136139	ABLIM3

7282	chr6	117447924	117449872	ROS1
8251	chr7	6007558	6026598	SNORA80D
4475	chr19	52098160	52101889	ZNF841
4869	chr2	207515170	207550159	CREB1
8826	chr9	121181388	121208537	GSN
477	chr1	217583555	217632398	GPATCH2
7324	chr6	133521397	133544378	LINC01312
532	chr1	227354924	227358238	CDC42BPA
9286	chrX	30883824	30889344	TAB3
1156	chr10	5975514	5978672	IL15RA
9302	chrX	46445637	46460975	KRBOX4
7061	chr5	61151499	61167435	SMIM15-AS1
655	chr1	28736950	28755113	YTHDF2
3946	chr17	80760501	80771118	LOC101928855
3440	chr16	803163	809354	PRR25
4997	chr2	27705206	27708274	LINC01460
2370	chr13	24483660	24512256	PARP4
6027	chr3	170902615	170918898	EIF5A2
2870	chr15	40736271	40756682	RMDN3
4118	chr18	70197228	70206872	RTTN
3117	chr15	99015020	99018329	LUNAR1
8708	chr8	87653210	87735595	DCAF4L2
6443	chr4	168469309	168482074	DDX60L
6823	chr5	141968304	141987843	RNF14
4110	chr18	63474874	63502101	SERPINB5
5164	chr2	69911297	69949072	MXD1
1436	chr11	13939289	13941978	LOC101928132
4884	chr2	215135924	215138466	ABCA12
1597	chr11	65114022	65138412	MIR6751
7947	chr7	101084463	101088202	TRIM56
7196	chr5	96661220	96681944	CAST
3357	chr16	57619590	57621831	ADGRG1
5107	chr2	55266988	55296690	PRORSD1P
257	chr1	160021906	160034866	PIGM
471	chr1	214346525	214355437	SMYD2
2776	chr14	75100798	75129593	NEK9
8641	chr8	54133854	54140380	MRPL15
9112	chr9	87724779	87734329	CTSL
849	chr1	77123052	77130130	MIR7156
4205	chr19	16496155	16497236	C19orf44
7094	chr5	69838531	69840845	LOC653080
7593	chr6	34392155	34394552	NUDT3
9145	chr9	96378792	96384779	SLC35D2
1074	chr10	22338537	22342832	SPAG6

400	chr1	200636256	200641837	KIF14
5759	chr22	46752911	46756572	TBC1D22A
8452	chr8	124361916	124372226	TMEM65
8610	chr8	405022	407756	FBXO25
634	chr1	26785636	26791168	PIGV
121	chr1	145962316	145966310	ANKRD34A
5376	chr20	45361775	45364769	SYS1-DBNDD2
8316	chr7	76315383	76360588	YWHAG
5619	chr22	19940120	19957424	COMT
679	chr1	33047503	33051379	AK2
438	chr1	207309135	207311977	CD55
2840	chr15	23300358	23306721	GOLGA6L22
5540	chr21	37065949	37085699	TTC3
6735	chr5	122844481	122857163	SNX24
2919	chr15	49364422	49367071	FGF7
392	chr1	19797152	19801150	TMCO4
3943	chr17	80500130	80503791	NPTX1
4958	chr2	238289210	238291052	PER2
6841	chr5	146079442	146183848	PLAC8L1
7843	chr6_GL000252v2_alt	4442101	4448897	RXRB
5393	chr20	47499903	47511824	NCOA3
3388	chr16	68262971	68265912	SLC7A6
5549	chr21	38909340	38915095	LOC400867
8215	chr7	44433268	44492021	NUDCD3
2586	chr14	22886432	22888481	REM2
7573	chr6	31662213	31666978	GPANK1
8145	chr7	23297579	23317852	MALSU1
5172	chr2	70299876	70302487	FAM136A
7640	chr6	42780122	42782858	LOC401261
1423	chr11	129637018	129641139	LINC01395
5874	chr3	126265275	126269185	ALDH1L1
8003	chr7	118309774	118313319	ANKRD7
7817	chr6_GL000251v2_alt	3433510	3437663	SKIV2L
1644	chr11	67427297	67450367	PTPRCAP
486	chr1	220246130	220274089	AURKAPS1
4035	chr18	12990276	13000637	CEP192
649	chr1	28328177	28334111	MED18
910	chr1	93689595	93717523	BCAR3
632	chr1	26618384	26624337	MIR1976
5367	chr20	44474168	44483044	TTPAL
5026	chr2	3377614	3415670	TRAPPC12
3928	chr17	79024837	79028095	C1QTNF1
7979	chr7	107558702	107570420	COG5
369	chr1	185283624	185319686	IVNS1ABP

2239	chr12	8232827	8243471	LINC02449	
5019	chr2	32153588	32159259	SLC30A6	
5965	chr3	150369880	150379533	TSC22D2	
6246	chr3	50319176	50338415	TUSC2	
1585	chr11	64371962	64374075	MIR1237	
7882	chr6_GL000254v2_alt	3297854	3306669	NELFE	
1006	chr10	119590167	119603677	TIAL1	
8064	chr7	144175766	144187430	CTAGE8	
8485	chr8	140463432	140467021	TRAPPC9	
6040	chr3	179403255	179409457	GNB4	
446	chr1	20942375	21023690	MIR1256	
371	chr1	186558672	186560876	PDC	
176	chr1	151067936	151074585	GABPB2	
4399	chr19	45476921	45479513	FOSB	
1763	chr12	100575798	100585832	GAS2L3	
1292	chr10	93495669	93498266	CEP55	
4911	chr2	224085022	224086375	SERPINE2	
8080	chr7	149595702	149608780	ZNF767P	
2059	chr12	50756436	50778140	ATF1	
2730	chr14	67663775	67675891	VTI1B	
6418	chr4	151318254	151326450	PRSS48	
1738	chr11	94876121	94925441	CWC15	
6181	chr3	43285643	43325084	SNRK	
7170	chr5	90465500	90478943	MBLAC2	
951	chr10	102132633	102162338	NOLC1	
3880	chr17	74747636	74749798	MIR3615	
6108	chr3	195632178	195638885	LOC105374297	
7315	chr6	131949519	131952618	CTGF	
469	chr1	21344382	21346550	ECE1	
4084	chr18	47156323	47157388	HDHD2	
4014	chr17_KI270857v1_alt	2857527	2861975	CWC25	
1856	chr12	120437320	120440049	COX6A1	
9115	chr9	89177823	89182485	SHC3	
695	chr1	36451245	36466293	MRPS15	
686	chr1	35078370	35082583	ZMYM1	
723	chr1	42814978	42826441	SVBP	
5718	chr22	41464768	41474575	ACO2	
3918	chr17	77254236	77256386		9-Sep
9005	chr9	35488702	35498753	RUSC2	
1790	chr12	10699549	10724322	YBX3	
1275	chr10	90855773	90860584	HTR7	
6282	chr3	75428516	75436706	FAM86DP	
2597	chr14	24159146	24174680	REC8	
8759	chr9	101532605	101536883	RNF20	

7980	chr7	107740236	107770532	LOC101927974
6729	chr5	119069296	119070974	LOC105379143
7030	chr5	53035558	53047595	ITGA2
7580	chr6	3273171	3287983	PSMG4
1715	chr11	8943473	8950897	ASCL3
4288	chr19	35527868	35555805	TMEM147
8142	chr7	22853902	22855893	SNHG26
1058	chr10	16813418	16819070	RSU1
873	chr1	85197563	85203456	SYDE2
625	chr1	26122775	26126872	PDIK1L
2517	chr13	94564462	94596632	TGDS
6972	chr5	29191425	29193572	LINC02109
2051	chr12	50112049	50115729	COX14
3190	chr16	2014653	2018941	NPW
5036	chr2	36962439	36967622	STRN
3206	chr16	22194666	22196767	EEF2K
5344	chr20	3780369	3788493	CENPB
2350	chr13	114130886	114132794	RASA3
3796	chr17	5830868	5836797	LOC339166
5702	chr22	39254620	39256216	PDGFB
2511	chr13	85235306	85237534	LINC00375
7412	chr6	150291210	150294636	IYD
1678	chr11	75561812	75567607	SERPINH1
7369	chr6	142775807	142803253	LOC153910
3619	chr17	35076242	35083582	RFFL
3157	chr16	14070204	14099810	MKL2
1390	chr11	120335126	120351371	ARHGEF12
2212	chr12	75380835	75392802	CAPS2
6937	chr5	179697383	179705258	CANX
6865	chr5	157131432	157144454	MED7
6431	chr4	156965346	156971965	PDGFC
7744	chr6	80437398	80441385	BCKDHB
7063	chr5	613241	626825	CEP72
1471	chr11	29292688	29296006	LINC02546
5209	chr2	85345403	85358058	RETSAT
874	chr1	85248070	85276666	C1orf52
3138	chr16	10470790	10475374	LINC01290
8386	chr7_KI270809v1_alt	109405	118287	POMZP3
6397	chr4	139430769	139458277	RAB33B
1256	chr10	86709446	86714726	LDB3
3958	chr17	81364681	81366623	LOC100130370
9042	chr9	5434416	5439326	PLGRKT
5955	chr3	149369875	149384162	TM4SF1-AS1
6571	chr4	48757564	48781839	FRYL

1055	chr10	14916436	14920832	DCLRE1C
7950	chr7	101136840	101140927	SERPINE1
1917	chr12	131736706	131777782	SFSWAP
854	chr1	7781361	7783886	PER3
4255	chr19	2450776	2457445	LMNB2
9263	chrX	16717964	16742163	SYAP1
963	chr10	104026809	104084442	MIR936
331	chr1	174237741	174250914	RABGAP1L
7788	chr6_GL000250v2_alt	160437	163289	HCG14
3445	chr16	828551	829841	PRR25
561	chr1	233607058	233671097	MIR4427
8275	chr7	66730729	66741742	RABGEF1
8105	chr7	154937223	154959652	PAXIP1-AS2
2994	chr15	66385546	66394483	MAP2K1
6297	chr3	97760055	97766396	ARL6
2662	chr14	49906275	49909584	ARF6
8455	chr8	125272920	125302660	TRIB1
7357	chr6	138771795	138780027	GVQW2
144	chr1	148416812	148463246	LINC01138
3996	chr17_GL000258v2_alt	1511680	1514956	LRRC37A
19	chr1	1077880	1081406	RNF223
1169	chr10	63500279	63598569	JMJD1C
8867	chr9	128320897	128335591	TRUB2
4121	chr18	74147337	74154189	TIMM21
939	chr10	100369721	100379298	OLMALINC
212	chr1	154954362	154965272	LOC101928120
5630	chr22	21847455	21869767	MAPK1
5203	chr2	84880812	84882433	TRABD2A
7946	chr7	100864993	100889812	SRRT
8085	chr7	151085449	151092679	AGAP3
6288	chr3	8736788	8743404	CAV3
2176	chr12	67267166	67281444	CAND1
7753	chr6	83392510	83393710	ME1
451	chr1	209917837	209920967	SYT14
6014	chr3	161219226	161248794	NMD3
2381	chr13	28645560	28685098	POMP
3235	chr16	28424332	28427104	EIF3C
5765	chr22	49916727	49918901	ALG12
7459	chr6	165564158	165576691	PDE10A
7044	chr5	55931671	55945178	IL6ST
224	chr1	155743916	155749299	MSTO2P
6581	chr4	55351270	55353847	SRD5A3
1915	chr12	13099206	13103178	GSG1
7828	chr6_GL000252v2_alt	1853080	1872274	PPP1R10

6372	chr4	127831321	127847881	PLK4
1290	chr10	93417799	93439198	MYOF
6172	chr3	40447394	40455325	ENTPD3-AS1
4845	chr2	202373416	202380846	BMPR2
6196	chr3	45812529	45843717	LZTFL1
377	chr1	19009149	19012439	IFFO2
2347	chr13	113920031	113928633	LINC00565
1258	chr10	86962292	86984442	AGAP11
3792	chr17	57083187	57091560	AKAP1
3909	chr17	7622709	7628655	SAT2
157	chr1	149830222	149863629	HIST2H2BC
760	chr1	47331832	47340222	CMPK1
2064	chr12	51269588	51275642	SMAGP
2718	chr14	64913564	64941163	CHURC1-FNTB
6526	chr4	37660929	37668331	RELL1
64	chr1	114708115	114758942	NRAS
3562	chr17	2341955	2347242	SGSM2
8991	chr9	33505975	33512870	SUGT1P1
2251	chr12	89225240	89226571	DUSP6
540	chr1	228485103	228491444	RNF187
4923	chr2	229900380	229923813	TRIP12
1912	chr12	12926700	12940751	GPRC5D-AS1
2456	chr13	49506641	49533937	PHF11
127	chr1	146156560	146228914	NOTCH2NL
1221	chr10	73772480	73784804	CHCHD1
9322	chrX	49040801	49042860	TFE3
4713	chr2	159097710	159103219	MIR6888
7154	chr5	82356473	82359350	ATP6AP1L
4634	chr2	112626594	112630543	FLJ42351
913	chr1	93803988	93806351	MIG7
6424	chr4	153307332	153309923	ANXA2P1
2029	chr12	47810047	47822057	HDAC7
954	chr10	102460465	102462432	MFSD13A
7610	chr6	36592366	36616042	SRSF3
285	chr1	16513017	16516926	RNVU1-18
4812	chr2	19973834	19992004	WDR35
2623	chr14	34544675	34547884	RNVU1-18
4608	chr2	105331473	105359141	C2orf49
7359	chr6	139025213	139035891	ABRACL
8430	chr8	11839588	11869643	CTSB
6003	chr3	158570326	158572232	MLF1
7103	chr5	72192410	72200654	MAP1B
2542	chr14	101808178	101818596	PPP2R5C
6662	chr4	88155012	88160856	ABCG2

4767	chr2	180014681	180017540	CWC22
8180	chr7	30499182	30506960	GGCT
5391	chr20	47346542	47361710	LOC101927377
2514	chr13	94232626	94234936	GPC6-AS1
3430	chr16	74598413	74608450	GLG1
3296	chr16	3186492	3188682	OR1F1
582	chr1	236197686	236205749	GPR137B
9237	chrX	13732414	13750428	OFD1
8391	chr8	100703900	100726276	PABPC1
4284	chr19	34998924	35003413	GRAMD1A
4948	chr2	23702077	23714272	ATAD2B
9138	chr9	94553114	94560559	PCAT7
5382	chr20	45853258	45861689	ACOT8
2897	chr15	43822921	43825967	MFAP1
3726	chr17	46721027	46725858	WNT3
559	chr1	23324008	23345905	HNRNPR
2609	chr14	30621082	30685447	SCFD1
4627	chr2	11154105	11155728	PQLC3
7097	chr5	70414008	70458842	GTF2H2
6136	chr3	23738435	23740697	UBE2E1
8503	chr8	143822124	143834687	PUF60
8158	chr7	25856217	25859149	MIR148A
8891	chr9	129747466	129753975	PTGES
9357	chrX	71117874	71141035	MED12
5833	chr3	11627400	11643580	VGLL4
7698	chr6	63569764	63587109	PTP4A1
7113	chr5	73624345	73649840	ARHGEF28
2232	chr12	78887678	78889262	SYT1
4379	chr19	43921019	43936386	ZNF45
3028	chr15	72457367	72610403	MIR630
880	chr1	86362242	86397803	ODF2L
258	chr1	160204971	160223691	LOC729867
4882	chr2	210754896	210756984	CPS1-IT1
2590	chr14	23059767	23102446	ACIN1
3145	chr16	11638155	11642393	SNN
7301	chr6	125917752	125935737	NCOA7
2795	chr14	83556213	83558832	LINC02301
1050	chr10	133373014	133374553	ECHS1
8065	chr7	144276797	144301744	ARHGEF34P
208	chr1	154173345	154184259	TPM3
7824	chr6_GL000251v2_alt	66336	78644	ZBED9
8642	chr8	55768742	55796528	TMEM68
1101	chr10	31313312	31321155	ZEB1
8765	chr9	106847932	106906694	ZNF462

1821	chr12	111908199	111914658	ADAM1A
7273	chr6	114016553	114020004	HS3ST5
2932	chr15	52565872	52569911	ARPP19
3017	chr15	70826004	70859291	LRRC49
6573	chr4	49143550	49145805	CWH43
2368	chr13	21601589	21605112	MICU2
5661	chr22	30801782	30812333	OSBP2
6978	chr5	32174518	32177195	GOLPH3
9183	chrX	108281353	108314716	COL4A6
8862	chr9	128123952	128129255	PTGES2
6486	chr4	186829322	186841956	FAT1
3633	chr17	37604004	37611024	SYNRG
2208	chr12	74337061	74339017	LOC100507377
8262	chr7	65404642	65409485	ZNF92
9305	chrX	47128349	47133011	NDUFB11
7238	chr6	106545990	106565105	CRYBG1
6249	chr3	51339580	51341364	MANF
5304	chr20	32276742	32284281	KIF3B
4783	chr2	187461460	187555679	TFPI
734	chr1	43943960	43965933	IPO13
4571	chr19_GL949752v1_alt	175044	184893	RPS9
3981	chr17	82456339	82460601	NARF
9290	chrX	38795302	38810615	MID1IP1-AS1
5835	chr3	117733477	117736223	LINC02024
3109	chr15	96322536	96336630	NR2F2
8932	chr9	136727288	136734492	SNHG7
4725	chr2	163344930	163351064	FIGN
698	chr1	37531365	37546015	SNIP1
1756	chr11_KI270831v1_alt	116811	119300	CDKN1C
8566	chr8	25414917	25437742	GNRH1
5999	chr3	15721367	15739208	ANKRD28
2780	chr14	76151015	76215682	GPATCH2L
211	chr1	154625152	154628972	ADAR
4808	chr2	197513613	197519360	MOB4
6075	chr3	188880635	188903241	TPRG1-AS1
9256	chrX	154761788	154767798	DKC1
5907	chr3	133572134	133596228	CDV3
6138	chr3	25627301	25665976	TOP2B
7968	chr7	104262165	104268566	ORC5
5060	chr2	423985	426717	LINC01874
1150	chr10	5449100	5465026	NET1
2130	chr12	57755586	57773565	CYP27B1
6451	chr4	169618283	169624172	CLCN3
8866	chr9	128295218	128296800	SWI5

4760	chr2	177164344	177166710	HNRNPA3
7240	chr6	106746101	106747491	MIR587
8035	chr7	131093652	131116045	LINC-PINT
1928	chr12	133080067	133090209	ZNF140
6085	chr3	189935144	189938008	MIR944
8805	chr9	114550732	114554627	ATP6V1G1
8133	chr7	1937345	1941285	MAD1L1
1728	chr11	93750209	93753218	C11orf54
1449	chr11	18506354	18538857	TSG101
6807	chr5	140339720	140345702	HBEGF
5276	chr20	1387060	1394455	MIR6869
8968	chr9	21863179	21870943	MTAP
4066	chr18	35581132	35584328	MIR3975
1852	chr12	120201442	120203102	PXN-AS1
7071	chr5	63261136	63264470	IPO11-LRRC70
6453	chr4	169712229	169734622	HPF1
5986	chr3	155068506	155072236	MME
6604	chr4	7054662	7068916	GRPEL1
8635	chr8	51880149	51905630	PCMTD1
3274	chr16	30650214	30653616	PRR14
8526	chr8	144875845	144879863	ZNF250
90	chr1	120552325	120559995	LOC100996724
3244	chr16	28924388	28926652	RABEP2
5141	chr2	65098513	65133046	RAB1A
1244	chr10	79309143	79322752	PPIF
7799	chr6_GL000251v2_alt	1454525	1457510	HCG9
1117	chr10	3760305	3767963	KLF6
6010	chr3	16065896	16076584	GALNT15
1863	chr12	120903602	120905670	XLOC_009911
3481	chr16	89470817	89477635	ANKRD11
6554	chr4	41215031	41218341	APBB2
8877	chr9	128817254	128830163	ENDOG
1676	chr11	74748091	74757514	RNF169
4443	chr19	48258699	48260946	CARD8
3752	chr17	48943585	48947014	SNF8
2687	chr14	54559029	54763377	SAMD4A
4054	chr18	2652566	2678639	SMCHD1
2087	chr12	53440052	53479173	PCBP2-OT1
7306	chr6	127052411	127056273	RSPO3
5392	chr20	47390483	47394283	LINC01754
6966	chr5	23630651	23632884	PRDM9
2510	chr13	80472778	80474929	SPRY2
1008	chr10	119686546	119688316	BAG3
9212	chrX	126471776	126473891	DCAF12L1

7187	chr5	95833977	95837104	GLRX
5470	chr20	62793793	62795943	MRGBP
8845	chr9	126788549	126789462	ZBTB43
9197	chrX	119571258	119587925	UBE2A
4912	chr2	224146508	224148181	SERPINE2
740	chr1	44738658	44742246	KIF2C
7603	chr6	35828501	35835414	LHFPL5
2903	chr15	44534016	44536858	EIF3J-DT
8390	chr8	10055148	10066103	MSRA
315	chr1	1707817	1730972	CDK11A
9089	chr9	73142807	73145067	ANXA1
2454	chr13	49435906	49453682	SETDB2
6146	chr3	30347206	30348988	LINC01985
3623	chr17	35585420	35609842	AP2B1
4	chr1	1003674	1017305	ISG15
5555	chr21	39841916	39843520	PCP4
8879	chr9	128993549	129011816	SH3GLB2
924	chr1	94647117	94649459	MIR378G
3149	chr16	11862682	11878786	RSL1D1
500	chr1	222814177	222870355	DISP1
5116	chr2	58198698	58242904	FANCL
8985	chr9	33021051	33028577	DNAJA1
5099	chr2	54329522	54331352	C2orf73
6889	chr5	171386362	171403726	NPM1
7560	chr6	2998508	3021975	NQO2
3479	chr16	89215823	89220503	ZNF778
3538	chr17	18309475	18329797	SMCR8
9376	chrX	77889603	77897573	MAGT1
5213	chr2	85592090	85615059	USP39
2073	chr12	52272977	52282576	KRT86
8748	chr8_KI270816v1_alt	160180	167286	ZNF707
5820	chr3	112695135	112697238	LINC02042
5904	chr3	132634604	132692515	UBA5
6804	chr5	140105279	140114486	MALINC1
3586	chr17	28931795	28953665	PHF12
1282	chr10	91796630	91873984	TNKS2
9150	chr9	97703850	97704737	XPA
6368	chr4	125197830	125200114	FAT4
2105	chr12	56114732	56119438	RPL41
4772	chr2	181790469	181792773	SSFA2
935	chr10	100152498	100190214	ERLIN1
7169	chr5	892967	927455	TRIP13
1455	chr11	19714373	19723563	LOC100126784
7656	chr6	43573865	43577658	XPO5

7702	chr6	6778468	6793273	LY86-AS1
2186	chr12	69356898	69365645	YEATS4
1958	chr12	25958956	25969148	RASSF8-AS1
7922	chr6_GL000256v2_alt	203798	207227	HCG14
1114	chr10	35912592	35915666	PCAT5
6426	chr4	154532065	154551287	PLRG1
1825	chr12	112095008	112110934	NAA25
1468	chr11	28100124	28175053	METTL15
6111	chr3	195980048	195991706	SDHAP1
8955	chr9	15552094	15553827	CCDC171
3925	chr17	78781898	78839985	CYTH1
9122	chr9	92227212	92298408	MIR3651
3834	chr17	64678806	64683992	SMURF2
1179	chr10	68085276	68210389	MYPN
7503	chr6	26214570	26218924	HIST1H2BG
2077	chr12	52940830	52955333	KRT18
8585	chr8	29638797	29670849	LINC00589
923	chr1	94542169	94545434	F3
2816	chr14	98972290	98974007	C14orf177
1354	chr11	108927841	108949952	DDX10
862	chr1	8076601	8079367	ERRFI1
6213	chr3	48418846	48430690	PLXNB1
7784	chr6	99404616	99437012	LOC101927365
6448	chr4	168994435	169011548	CBR4
899	chr1	9234345	9241948	H6PD
6820	chr5	141820396	141822548	PCDH1
1147	chr10	52487149	52491547	LINC01468
990	chr10	114025892	114027783	ADRB1
1986	chr12	31480354	31526290	DENND5B-AS1
5368	chr20	44514277	44523532	SERINC3
2587	chr14	22914346	22930425	PRMT5-AS1
1710	chr11	88309478	88348624	CTSC
1783	chr12	105253023	105254808	APPL2
4907	chr2	220511307	220513564	MIR4268
5994	chr3	156525788	156557129	SSR3
2704	chr14	60163091	60167218	DHRS7
9080	chr9	72304475	72306589	LINC01504
6514	chr4	2841682	2846722	ADD1
9310	chrX	47835411	47847760	ZNF81
4491	chr19	55405001	55409073	UBE2S
130	chr1	146374500	146378070	RNU1-4
1849	chr12	119178399	119198216	HSPB8
8463	chr8	126549413	126560628	FAM84B
969	chr10	104808431	104811275	SORCS3-AS1

7528	chr6	27692947	27695311	LINC01012
2614	chr14	31242438	31245133	HECTD1
7543	chr6	28323915	28338553	ZSCAN31
7965	chr7	103717402	103720692	RELN
5287	chr20	24979443	24994541	APMAP
8176	chr7	29656892	29689879	MIR550A3
558	chr1	233043773	233115171	NTPCR
5173	chr2	70547447	70555962	TGFA
5509	chr21	32462885	32464597	EVA1C
7693	chr6	56942438	56955194	DST
391	chr1	197191433	197207639	ZBTB41
2453	chr13	49263362	49278496	CDADC1
3047	chr15	75645657	75651084	SNX33
9178	chrX	10616959	10620233	MID1
8786	chr9	111702592	111704541	GNG10
1818	chr12	111589139	111601725	ATXN2-AS
6070	chr3	186562301	186568862	TBCCD1
132	chr1	146938414	146958472	NBPF12
8600	chr8	38104033	38115974	ASH2L
8972	chr9	21996063	22023181	CDKN2B
2504	chr13	78612939	78660053	RNF219
9262	chrX	16184048	16186801	MAGEB17
6294	chr3	9354476	9394956	THUMPD3
7660	chr6	43663436	43690044	MRPS18A
8297	chr7	73827052	73830026	CLDN4
1620	chr11	65978902	65984372	SART1
4688	chr2	144409748	144519787	ZEB2-AS1
9195	chrX	118973676	119017483	LONRF3
6422	chr4	152778136	152781151	TIGD4
4333	chr19	4034709	4043293	ZBTB7A
9	chr1	102878840	102880689	COL11A1
2462	chr13	50908623	50937441	RNASEH2B-AS1
735	chr1	44027196	44032898	SLC6A9
1753	chr11	98329783	98333012	CNTN5
3450	chr16	84724993	84748759	USP10
3036	chr15	74394722	74399105	MIR6881
317	chr1	171483830	171527240	PRRC2C
1951	chr12	21644686	21658666	LDHB
2109	chr12	56220323	56223874	RNF41
6701	chr5	108744605	108796656	FER
7908	chr6_GL000255v2_alt	3060693	3063699	LSM2
5888	chr3	128786499	128793447	RAB7A
1400	chr11	123197338	123199615	CLMP
6728	chr5	118986581	118989663	DTWD2

3779	chr17	53105757	53106518	C17orf112
7808	chr6_GL000251v2_alt	2259810	2263695	HCG20
8960	chr9	19370973	19384784	RPS6
5045	chr2	37635671	37642903	CDC42EP3
8413	chr8	10690598	10691849	C8orf74
4325	chr19	39439755	39456207	SUPT5H
1908	chr12	12802797	12805634	DDX47
4175	chr19	13114271	13118129	TRMT1
1176	chr10	67883096	67912258	SIRT1
8143	chr7	23103165	23114927	KLHL7
9090	chr9	73147789	73175225	ANXA1
5041	chr2	37304951	37325822	PRKD3
6159	chr3	33440528	33442894	UBP1
6878	chr5	163448039	163462587	NUDCD2
5249	chr2	9752043	9755429	TAF1B
1164	chr10	62267882	62269638	RTKN2
1231	chr10	75399475	75405095	ZNF503
766	chr1	51557068	51563699	EPS15
5140	chr2	65057072	65069940	CEP68
1808	chr12	110280077	110294954	ATP2A2
3165	chr16	15027788	15030554	MIR1972-1
728	chr1	43359212	43369434	MIR6734
6687	chr5	100776370	100779619	MIR548P
8923	chr9	134370583	134382675	RXRA
1846	chr12	118145020	118153991	PEBP1
182	chr1	151422421	151462263	POGZ
2021	chr12	4646496	4673449	NDUFA9
5264	chr20	10446419	10448796	SLX4IP
1897	chr12	124631521	124637528	NCOR2
8332	chr7	80721612	80724631	CD36
4706	chr2	15584686	15611934	DDX1
2577	chr14	21417664	21438593	CHD8
9315	chrX	48596612	48601028	WDR13
5467	chr20	62703295	62705633	NTSR1
1096	chr10	29598345	29602486	MIR938
721	chr1	42454770	42465778	CCDC30
1002	chr10	119160893	119167216	SFXN4
3911	chr17	76352594	76354768	PRPSAP1
4382	chr19	44093232	44098518	ZNF224
4308	chr19	37770695	37784440	ZNF573
8966	chr9	21678101	21690375	MTAP
2520	chr13	96034435	96054779	UGGT2
6180	chr3	42798624	42804532	HIGD1A
4714	chr2	159238843	159242584	WDSUB1

6668	chr4	928255	937375	GAK
6097	chr3	194127403	194143249	HES1
7176	chr5	92551940	92553710	NR2F1-AS1
5643	chr22	28708355	28713879	CHEK2
5451	chr20	58990251	59000085	CTSZ
658	chr1	31056842	31066400	PUM1
1297	chr10	95288021	95290797	PDLIM1
8440	chr8	121809853	121813465	HAS2
6954	chr5	18697206	18747646	LINC02223
1653	chr11	68111012	68130925	CHKA
6365	chr4	124625434	124641231	LINC02516
7372	chr6	143336058	143339774	ADAT2
5741	chr22	44346269	44366132	SHISAL1
4794	chr2	190874065	190972798	GLS
5561	chr21	41757539	41769602	RIPK4
221	chr1	155305791	155322340	FDPS
4007	chr17_KI270857v1_alt	1731822	1735897	DUSP14
9097	chr9	78232649	78248674	CEP78
4424	chr19	47035228	47036840	NPAS1
41	chr1	110405030	110410436	LAMTOR5-AS1
3438	chr16	77189963	77194206	MON1B
2670	chr14	51396972	51399802	LINC02310
8559	chr8	23183551	23224499	TNFRSF10A
5407	chr20	50190254	50208611	CEBPB-AS1
5097	chr2	53965119	53969877	PSME4
5319	chr20	35294308	35321969	FAM83C
5887	chr3	128764172	128775342	RAB7A
5695	chr22	38267206	38319945	TMEM184B
8037	chr7	131233922	131238083	MKLN1-AS
3177	chr16	1820891	1834922	FAHD1
7823	chr6_GL000251v2_alt	4708864	4712226	RGL2
6436	chr4	163491493	163503115	TMA16
3987	chr17	8434803	8497422	NDEL1
2469	chr13	52597086	52601236	HNRNPA1L2
3648	chr17	40051488	40055931	MED24
4346	chr19	41435924	41442552	DMAC2
8220	chr7	44799033	44805876	PPIA
853	chr1	77809745	77819504	MIGA1
6416	chr4	14996772	15002209	CPEB2-DT
141	chr1	148361146	148364401	RNVU1-1
6524	chr4	37438252	37440180	C4orf19
2809	chr14	92791886	92802852	GOLGA5
6310	chr3_KI270924v1_alt	155236	162148	LOC105374297
1326	chr11	102342593	102369965	BIRC2

5042	chr2	37534907	37537228	CDC42EP3
7549	chr6	2857567	2859228	MIR4645
8373	chr7	98300954	98302032	BRI3
6515	chr4	30715655	30723394	PCDH7
1650	chr11	67799055	67805548	FAM86C2P
9216	chrX	12973055	12980224	TMSB4X
9272	chrX	20515235	20517471	RPS6KA3
6022	chr3	169762163	169763917	TERC
4027	chr18	11848569	11859904	CHMP1B
5781	chr22_KI270928v1_alt	496	7690	SMDT1
7807	chr6_GL000251v2_alt	2227890	2233644	IER3
4852	chr2	20445287	20452594	RHOB
6128	chr3	197733985	197738579	RUBCN
4271	chr19	32344299	32382570	ZNF507
8954	chr9	15421922	15426887	SNAPC3
3782	chr17	5475338	5493162	DERL2
5696	chr22	38399467	38401183	TPTEP2
9130	chr9	93119318	93120498	NINJ1
4601	chr2	10297027	10299786	HPCAL1
320	chr1	171780488	171785920	METTL13
2372	chr13	25274814	25288497	MTMR6
1877	chr12	122379684	122407614	CLIP1-AS1
7387	chr6	146913008	146916710	KATNBL1P6
1751	chr11	9814921	9826947	LOC101928008
6883	chr5	16506733	16510712	RETREG1
1207	chr10	72609578	72627765	MICU1
5161	chr2	69633375	69647242	ANXA4
51	chr1	112697830	112698369	RHOC
6360	chr4	122825408	122880872	FGF2
7825	chr6_GL000252v2_alt	1195721	1199688	HLA-A
4053	chr18	26224801	26229113	TAF4B
2554	chr14	103411993	103425736	MARK3
8718	chr8	93872967	93875140	MIR378D2
1334	chr11	10453682	10458026	AMPD3
7855	chr6_GL000253v2_alt	2058425	2064152	MUC22
4583	chr1_KI270763v1_alt	279211	281149	SDCCAG8
6711	chr5	111502289	111514056	STARD4-AS1
310	chr1	169354794	169367545	BLZF1
1372	chr11	117135627	117138929	PAFAH1B2
8888	chr9	129476716	129516080	LINC00963
6463	chr4	176635298	176636774	VEGFC
5611	chr22	17636681	17656472	BCL2L13
1670	chr11	73305752	73310986	ARHGEF17
2882	chr15	41933731	41941780	EHD4-AS1

2768	chr14	73883334	73895574	ZNF410
8467	chr8	127389314	127392528	CCAT2
1785	chr12	10594440	10614898	KLRA1P
6718	chr5	115169085	115170366	TRIM36
3362	chr16	58390255	58396608	GINS3
5274	chr20	13634687	13640102	TASP1
234	chr1	156269706	156284772	SMG5
7515	chr6	26626616	26661038	ZNF322
2991	chr15	65868608	65875133	RAB11A
497	chr1	222556983	222593103	TAF1A-AS1
6623	chr4	76132617	76154055	NUP54
6835	chr5	143506552	143507998	NR3C1
6406	chr4	143176274	143196272	USP38
4345	chr19	41362211	41365583	TMEM91
8591	chr8	33470227	33472855	FUT10
8885	chr9	129405287	129407352	LINC01503
8820	chr9	120839863	120858511	CUTALP
7743	chr6	7985932	7990237	PIP5K1P1
4828	chr2	200893405	200905509	NIF3L1
9018	chr9	37485129	37487567	POLR1E
3010	chr15	69402647	69416520	KIF23
5981	chr3	153137539	153139863	RAP2B
5197	chr2	78493123	78542287	LOC105374820
6457	chr4	174283889	174294980	FBXO8
6344	chr4	116394641	116397586	MIR1973
7487	chr6	23174083	23176402	LOC105374972
6296	chr3	9748727	9751959	OGG1
2708	chr14	61693830	61746985	HIF1A
6644	chr4	8199053	8204509	SH3TC1
5639	chr22	26588935	26591023	TPST2
6061	chr3	184345722	184355058	CLCN2
8883	chr9	129174069	129178301	IER5L
8783	chr9	111595958	111600540	PTGR1
8814	chr9	115689564	115692870	LOC101928775
5517	chr21	33898360	33916286	ATP5PO
9029	chr9	38681591	38683875	FAM201A
1913	chr12	12964313	12968803	GPRC5D-AS1
7925	chr6_GL000256v2_alt	227731	234150	TRIM27
199	chr1	153657643	153672451	ILF2
8899	chr9	130680753	130683647	EXOSC2
1009	chr10	119723911	119727137	INPP5F
9086	chr9	73117732	73119699	ANXA1
2732	chr14	67737142	67740030	RDH12
4314	chr19	38373603	38378770	PSMD8

8422	chr8	115621605	115671296	TRPS1
4936	chr2	232696241	232705849	GIGYF2
3892	chr17	75227831	75233791	NUP85
5880	chr3	12730156	12761066	TMEM40
2540	chr14	101321898	101330276	LINC00524
7711	chr6	7106618	7117866	RREB1
8762	chr9	105242430	105247006	SLC44A1
4010	chr17_KI270857v1_alt	2384635	2391166	SOCS7
975	chr10	110207049	110210287	MXI1
6995	chr5	37000794	37013145	NIPBL
5568	chr21	42974288	43005741	PKNOX1
2896	chr15	43791459	43806841	SERINC4
4730	chr2	167133800	167135933	XIRP2-AS1
4977	chr2	25277779	25280092	DNMT3A
1529	chr11	57323131	57326574	TNKS1BP1
8679	chr8	68988551	68990722	LINC01592
4470	chr19	51089744	51100037	KLK14
6829	chr5	142828400	142829687	ARHGAP26-AS1
2755	chr14	72893438	72895507	DPF3
5832	chr3	115789733	115795980	SNORD155
4038	chr18	14189424	14190374	ANKRD20A5P
4568	chr19_GL949749v2_alt	174445	181217	RPS9
297	chr1	167446735	167448876	CD247
726	chr1	42966355	42968801	SLC2A1
6032	chr3	172038654	172387422	FNDC3B
2739	chr14	68463410	68466086	LOC100996664
9284	chrX	29660324	29665569	MIR4666B
2433	chr13	45119858	45127437	GTF2F2
1815	chr12	110612594	110621556	TCTN1
1591	chr11	64808963	64811180	MEN1
915	chr1	93844407	93848782	MIR760
9128	chr9	92854581	92879825	ZNF484
2451	chr13	49186817	49217994	MLNR
8520	chr8	144506712	144511934	MFSD3
1408	chr11	125096479	125098146	TMEM218
3320	chr16	47454479	47484191	PHKB
2695	chr14	57213479	57273897	AP5M1
197	chr1	153621528	153628092	S100A13
8375	chr7	99108414	99145886	SMURF1
4950	chr2	237458689	237461153	MLPH
7752	chr6	82407681	82421383	TPBG
459	chr1	21172818	21178131	EIF4G3
4296	chr19	36113476	36116535	TBCB
4649	chr2	119758244	119761969	PTPN4

114	chr1	145286154	145427373	NBPF20
1740	chr11	95059108	95074920	SRSF8
1624	chr11	66266711	66271589	RAB1B
3168	chr16	15501320	15503565	C16orf45
8289	chr7	72987412	73007852	STAG3L3
1159	chr10	61627828	61630492	CABCOCO1
5752	chr22	46065650	46112862	MIR3619
3618	chr17	35049581	35065681	RFFL
8250	chr7	5895580	5904639	CCZ1
8973	chr9	22101609	22103181	CDKN2B
5784	chr3	10020528	10030109	CIDECP
11	chr1	10428394	10434715	CENPS-CORT
3938	chr17	80036164	80037348	CCDC40
7879	chr6_GL000254v2_alt	2989185	2996700	BAG6
7469	chr6	170528423	170557455	PSMB1
5179	chr2	73213858	73220417	SMYD5
1204	chr10	72214038	72226812	ASCC1
14	chr1	10527180	10563704	PEX14
801	chr1	58901625	58905624	LINC01358
7032	chr5	53365026	53368351	FST
5592	chr21	46634287	46641839	PRMT2
1799	chr12	109051707	109059166	USP30-AS1
1472	chr11	2964315	2992365	NAP1L4
1881	chr12	122698411	122700742	HCAR2
1443	chr11	17082503	17088359	RPS13
1379	chr11	118556293	118560018	IFT46
7724	chr6	73579256	73582730	EEF1A1
5573	chr21	43386906	43399126	LINC01679
2184	chr12	69024158	69026479	CPM
2559	chr14	104809403	104817304	LINC00638
5266	chr20	11001054	11003227	C20orf187
9343	chrX	57589674	57605424	ZXDB
3554	chr17	1987598	1989930	LOC105371485
387	chr1	19335281	19344692	PQLC2
5884	chr3	128642897	128651749	RPN1
6314	chr3_KI270937v1_alt	159293	162619	LOC105374297
2245	chr12	8697732	8706591	RIMKLB
7625	chr6	3825054	3827564	FAM50B
586	chr1	236793851	236811704	MTR
9014	chr9	37117245	37161712	ZCCHC7
8359	chr7	93230168	93234043	HEPACAM2
2196	chr12	6998571	7000469	LPCAT3
1492	chr11	35028394	35033809	MIR1343
9098	chr9	78295432	78305644	PSAT1

4439	chr19	4811784	4813633	TICAM1
5515	chr21	33501878	33590377	SON
932	chr1	9941305	9952969	NMNAT1
6786	chr5	13663312	13667316	DNAH5
6348	chr4	119024506	119026886	MYOZ2
4745	chr2	172553865	172559496	PDK1
3309	chr16	4177910	4185824	SRL
1065	chr10	17638156	17651118	STAM
4171	chr19	12777014	12780508	HOOK2
731	chr1	43522287	43642758	PTPRF
8732	chr8	96231884	96236179	UQCRB
3151	chr16	11911024	11917627	GSPT1
35	chr1	109396934	109431051	PSMA5
9229	chrX	134806547	134808037	FAM122C
196	chr1	153562682	153573838	S100A2
1514	chr11	47146307	47164065	ARFGAP2
1707	chr11	86802134	86822014	PRSS23
9073	chr9	69702108	69761484	PTAR1
7035	chr5	53427342	53429207	FST
6560	chr4	4288216	4291793	LYAR
2485	chr13	73364803	73366968	LINC00392
824	chr1	6578850	6583930	ZBTB48
149	chr1	148951087	148961094	PDE4DIP
7012	chr5	41902761	41930564	FBXO4
7242	chr6	107113781	107118191	BEND3
8172	chr7	2861731	2865218	GNA12
45	chr1	111197982	111205466	DENND2D
3899	chr17	75661860	75696104	RECQL5
3780	chr17	5364665	5423908	RPAIN
2122	chr12	57083671	57099457	NAB2
4141	chr19	10184990	10195900	DNMT1
3232	chr16	28181759	28213912	XPO6
308	chr1	16903473	16906645	RNU1-4
4031	chr18	12435011	12440215	LOC105371998
5844	chr3	120176470	120178786	GPR156
7737	chr6	7877895	7882713	TXNDC5
3947	chr17	80790423	80802668	LOC101928855
2786	chr14	77614990	77618577	SPTLC2
80	chr1	119000278	119002822	LOC105378933
1883	chr12	122750658	122778703	CCDC62
1168	chr10	63189868	63270763	JMJD1C
780	chr1	52701806	52704781	COA7
2237	chr12	8079408	8093786	NECAP1
6964	chr5	21679678	21682552	SNORA105B

8957	chr9	19049270	19056568	RRAGA
8816	chr9	118575315	118580726	LINC02578
5947	chr3	146155535	146166364	PLOD2
2235	chr12	80150145	80151183	OTOGL
2089	chr12	53622421	53626927	ATF7
409	chr1	202800090	202811345	KDM5B
3248	chr16	29274607	29297427	SNX29P2
9149	chr9	97624207	97698647	TSTD2
9163	chrX	10016719	10019109	WWC3
7870	chr6_GL000254v2_alt	1398987	1406667	ZNRD1ASP
8457	chr8	125409261	125411439	TRIB1
3659	chr17	41783099	41785267	JUP
6933	chr5	179605809	179625465	HNRNPH1
5437	chr20	53642212	53643908	LOC105372672
5087	chr2	47842063	47911125	FBXO11
8347	chr7	90401921	90422226	CLDN12
886	chr1	88682252	88723568	PKN2-AS1
6473	chr4	183505374	183508573	ING2
8624	chr8	43240459	43242637	POTEA
6561	chr4	44674885	44726513	GUF1
5963	chr3	150345798	150349558	LINC01214
2410	chr13	40162963	40165118	LINC00332
1152	chr10	5544622	5548933	CALML3-AS1
1106	chr10	32510542	32517781	CCDC7
4145	chr19	10379414	10381810	TYK2
3849	chr17	68019157	68021302	KPNA2
2775	chr14	75057927	75070435	ACYP1
5424	chr20	51352121	51367303	MIR3194
3195	chr16	21299544	21305086	CRYM
2374	chr13	25900932	25902966	SHISA2
2883	chr15	41967177	41973901	EHD4
7670	chr6	49455109	49465128	MUT
37	chr1	109545274	109572749	GNAI3
9268	chrX	17376571	17542270	MIR4768
3317	chr16	4523508	4539463	CDIP1
6739	chr5	124654866	124656874	ZNF608
4411	chr19	46042875	46045517	IGFL4
7708	chr6	7050211	7051742	RREB1
763	chr1	50952316	50961221	FAF1
8608	chr8	390907	393032	FAM87A
489	chr1	220528632	220538188	MARK1
2117	chr12	56521809	56533528	RBMS2
8383	chr7	99615988	99622883	ZSCAN25
8344	chr7	90177926	90181067	STEAP1

5238	chr2	95397542	95404262	FAHD2A
1505	chr11	447781	450033	PTDSS2
8604	chr8	38406564	38416622	LETM2
6183	chr3	43765323	43768874	ANO10
2945	chr15	56364252	56366355	TEX9
6395	chr4	139298733	139311245	NDUFC1
670	chr1	32289816	32293257	HDAC1
5684	chr22	37253976	37255718	RAC2
1182	chr10	68515267	68528838	SLC25A16
7615	chr6	37101854	37103653	PIM1
4032	chr18	12585000	12608237	SPIRE1
5153	chr2	67372638	67416300	ETAA1
1437	chr11	14310795	14358895	RRAS2
5503	chr21	30716743	30719541	KRTAP21-3
2446	chr13	48297685	48337761	RB1
6376	chr4	128162198	128176099	LARP1B
7462	chr6	166364312	166387526	MPC1
5837	chr3	119233850	119241721	B4GALT4
3220	chr16	25110425	25115989	LCMT1
9158	chr9	99996529	100002911	STX17
8560	chr8	23352821	23355038	LOC100507156
7230	chr6	10312553	10317396	TFAP2A-AS2
9188	chrX	11110443	11115833	HCCS
4863	chr2	207069878	207172588	MIR2355
7818	chr6_GL000251v2_alt	3607752	3618166	AGPAT1
6732	chr5	120454913	120458874	PRR16
4479	chr19	52686731	52692077	ZNF83
8352	chr7	92125011	92136786	CYP51A1
5139	chr2	64986592	64990398	SLC1A4
507	chr1	224180697	224191787	DEGS1
6210	chr3	48038376	48040612	MAP4
1461	chr11	2395709	2407027	TSSC4
2325	chr13	110318331	110322098	COL4A2
5686	chr22	37454465	37456071	ELFN2
17	chr1	1067916	1070677	RNF223
3476	chr16	8864854	8869089	CARHSP1
9378	chrX	78102326	78141029	PGK1
7630	chr6	4058152	4066232	C6orf201
1457	chr11	20362127	20370682	HTATIP2
5478	chr20	63895056	63914869	DNAJC5
8480	chr8	132774792	132855921	PHF20L1
4493	chr19	55597697	55600443	FIZ1
987	chr10	112949657	112968964	TCF7L2
7037	chr5	53477448	53490477	FST

9318	chrX	48889708	48899289	PQBP1
3677	chr17	42979200	42988046	RUNDC1
536	chr1	228107362	228118431	MRPL55
4586	chr1_KI270765v1_alt	62031	65020	RNVU1-11
4375	chr19	43650959	43673321	PLAUR
48	chr1	112393376	112400645	CTTNBP2NL
81	chr1	119131723	119171486	WARS2
5082	chr2	47163601	47178132	CALM2
7228	chr6	102295395	102297648	GRIK2
3710	chr17	45100790	45124106	MIR6784
6991	chr5	36233848	36270393	NADK2
3230	chr16	27548015	27555877	KIAA0556
1049	chr10	133292391	133309090	ZNF511
7457	chr6	163586524	163587500	LOC102724152
4478	chr19	52655709	52672081	ZNF83
326	chr1	173475372	173493079	PRDX6
2071	chr12	52165448	52194756	KRT80
3813	chr17	61858882	61864149	BRIP1
2741	chr14	68686705	68688948	LOC100996664
3662	chr17	42376696	42390268	STAT3
9085	chr9	73049399	73052084	ALDH1A1
3092	chr15	90262079	90278457	CIB1
2486	chr13	73367524	73370118	LINC00392
7595	chr6	34694746	34698464	C6orf106
4162	chr19	1182791	1186924	SBNO2
3904	chr17	75892103	75907171	TRIM65
1087	chr10	27097598	27101467	ANKRD26
5959	chr3	149745802	149753792	COMMD2
8397	chr8	101077575	101083042	FLJ42969
6008	chr3	160435307	160442429	TRIM59-IFT80
9154	chr9	98055790	98061387	NANS
762	chr1	50539540	50605232	DMRTA2
3601	chr17	30884479	30912122	TEFM
190	chr1	152028645	152038332	S100A11
4406	chr19	45766504	45768838	DM1-AS
7954	chr7	101790999	101793707	CUX1
8648	chr8	58828838	58831566	NSMAF
2665	chr14	50099360	50117128	LINC01599
6717	chr5	113161706	113164614	MCC
2640	chr14	39155618	39172437	TRAPPC6B
5379	chr20	455933	462950	TBC1D20
3547	chr17	19160352	19163886	LOC388436
9351	chrX	68495665	68518274	YIPF6
6102	chr3	194581362	194591814	TMEM44-AS1

3843	chr17	66949551	66953346	CACNG4
2150	chr12	63904625	64042742	SRGAP1
3282	chr16	30994485	30999556	HSD3B7
9177	chrX	10579107	10580913	MID1
5572	chr21	43360201	43363330	LINC01679
4369	chr19	43315234	43353549	PRG1
8769	chr9	107546849	107549730	KLF4
2217	chr12	75861783	75864778	PHLDA1
9071	chr9	69119489	69123315	TJP2
8459	chr8	125605008	125608004	TRIB1
904	chr1	93170171	93173872	TMED5
2423	chr13	41950561	41964462	VWA8
2043	chr12	49506581	49520321	KCNH3
9013	chr9	37078386	37095823	EBLN3P
2531	chr14	100238443	100242492	YY1
368	chr1	185226677	185227619	SWT1
7208	chr5_GL339449v2_alt	483040	487425	SMN2
1210	chr10	72690643	72696208	MCU
6583	chr4	55394202	55549800	CLOCK
8622	chr8	43056372	43065611	FNTA
2494	chr13	75546741	75642276	LMO7
8157	chr7	2552403	2555507	BRAT1
1655	chr11	69293111	69306424	MYEOV
118	chr1	145915572	145931512	LIX1L-AS1
3548	chr17	19213596	19218234	LOC388436
151	chr1	149161107	149164145	NBPF9
833	chr1	67029961	67055903	SLC35D1
1238	chr10	77948764	77950415	DLG5-AS1
4231	chr19	18414757	18417693	SSBP4
8432	chr8	119206051	119236052	MAL2
7466	chr6	169162401	169163780	LINC01615
2455	chr13	49496101	49501236	PHF11
5218	chr2	86606813	86625798	RNF103
2749	chr14	69765122	69790022	LOC100289511
1558	chr11	62551218	62557040	MIR3654
9377	chrX	77898884	77906096	COX7B
6712	chr5	112159011	112170382	SNORA13
5383	chr20	45882822	45891034	SPATA25
3877	chr17	7341118	7357273	KCTD11
6777	chr5	135031117	135040667	PITX1
8336	chr7	84235075	84238718	SEMA3A
8062	chr7	141736784	141774555	TAS2R3
3799	chr17	58630152	58633091	TEX14
2272	chr12	93675076	93691544	CRADD

2583	chr14	22763661	22779917	OXA1L
1170	chr10	63628022	63630481	JMJD1C
5355	chr20	41007536	41011231	TOP1
5427	chr20	51525105	51535226	NFATC2
1181	chr10	68403950	68408423	RUFY2
1161	chr10	61836580	61838353	ARID5B
2735	chr14	67867028	67868955	RAD51B
425	chr1	20494095	20511779	MUL1
5960	chr3	149811293	149825460	RNF13
8231	chr7	5044180	5063547	RBAK
4898	chr2	219157231	219161545	NHEJ1
4620	chr2	110367535	110372947	LINC01123
3566	chr17	2403672	2408408	MNT
2249	chr12	88297698	88301179	TMTC3
6640	chr4	80076488	80078955	ANTXR2
8725	chr8	94470370	94476538	RAD54B
1955	chr12	25383463	25387730	KRAS
3936	chr17	79833742	79840655	CBX4
3133	chr15_KI270905v1_alt	4746385	4752703	LOC100288203
8179	chr7	30287442	30299570	MIR550B1
9248	chrX	154171888	154174161	OPN1MW
4804	chr2	196914543	196928369	PGAP1
1721	chr11	92999793	93001738	MTNR1B
6686	chr5	100532813	100556289	FAM174A
1213	chr10	73220287	73257052	DNAJC9
1890	chr12	12356227	12359221	BORCS5
7793	chr6_GL000250v2_alt	22692	24507	LINC01623
1797	chr12	108836023	108839838	MIR619
5025	chr2	33592908	33602170	FAM98A
8617	chr8	42337330	42339514	POLB
7863	chr6_GL000253v2_alt	2953248	2959355	BAG6
8249	chr7	5770180	5782079	RNF216
6174	chr3	41219930	41248992	CTNNB1
4036	chr18	13692829	13712247	FAM210A
2308	chr13	102189874	102193805	FGF14
9365	chrX	72707197	72715403	PHKA1
1626	chr11	66532679	66534217	ZDHHC24
5192	chr2	74834378	74838062	HK2
4637	chr2	11317747	11347056	ROCK2
1563	chr11	62664058	62666530	CSKMT
4932	chr2	231642036	231644203	TEX44
6219	chr3	48647742	48671988	CELSR3
9120	chr9	92083076	92117214	SPTLC1
7607	chr6	36422458	36425112	KCTD20

3422	chr16	72787985	72796414	LINC01572
8548	chr8	20077929	20081219	SLC18A1
3608	chr17	31934764	31951584	SUZ12
2281	chr12	95472421	95482576	METAP2
585	chr1	236582280	236605220	HEATR1
1621	chr11	66014305	66016412	CST6
2833	chr15	20501265	20509613	HERC2P3
4980	chr2	26216537	26254069	HADHA
3693	chr17	44064041	44067845	LSM12
6965	chr5	21684551	21686905	SNORA105B
6411	chr4	145733924	145735703	C4orf51
1324	chr11	102267370	102268558	BIRC3
5860	chr3	123730325	123735553	MYLK-AS2
548	chr1	230867821	230869609	C1orf198
8707	chr8	87627898	87644280	DCAF4L2
4906	chr2	220227833	220229815	MIR4268
4591	chr2	100561431	100564620	PDCL3
598	chr1	243253194	243262124	SDCCAG8
6528	chr4	37824757	37836868	PGM2
8711	chr8	89929764	89986775	NBN
3937	chr17	79992016	79995382	TBC1D16
6065	chr3	185584970	185588688	SENP2
6168	chr3	39050921	39061564	WDR48
4518	chr19	58401266	58410502	ZNF584
3460	chr16	87382733	87385882	FBXO31
7492	chr6	25274996	25280596	CARMIL1
5728	chr22	42734102	42746252	A4GALT
6765	chr5	134165770	134179521	SKP1
3972	chr17	81975898	81978325	ASPSCR1
7346	chr6	136605762	136611737	LOC101928461
9225	chrX	131829709	131832029	FIRRE
1764	chr12	101381995	101385372	ARL1
1295	chr10	94402736	94418791	TBC1D12
2546	chr14	102137414	102148170	WDR20
7852	chr6_GL000253v2_alt	1907530	1926673	PPP1R10
9289	chrX	3815384	3822095	LOC389906
4567	chr19_GL949748v2_alt	174723	177254	RPS9
302	chr1	168075301	168083933	GPR161
5460	chr20	62133021	62143135	PSMA7
8040	chr7	133036056	133054853	MIR3654
7148	chr5	81295989	81319862	ZCCHC9
407	chr1	201952949	201965108	TIMM17A
1919	chr12	131894690	131899246	ULK1
1130	chr10	43451608	43456352	ZNF487

1816	chr12	111435012	111454752	SH2B3
6536	chr4	39181291	39189312	WDR19
7730	chr6	75262566	75288524	TMEM30A
4355	chr19	42323723	42329032	MEGF8
2131	chr12	57863334	57866315	CTDSP2
511	chr1	224928252	224952220	DNAH14
7754	chr6	83423853	83433845	ME1
9046	chr9	6005558	6016055	KIAA2026
1116	chr10	3739827	3742395	KLF6
5464	chr20	62387873	62392749	RPS21
4677	chr2	135518245	135536626	ZRANB3
8426	chr8	117520069	117523169	MED30
7396	chr6	149312605	149409658	SUMO4
43	chr1	11097205	11100587	LOC105376736
4295	chr19	36051581	36056042	THAP8
4091	chr18	50966932	50975162	ELAC1
7133	chr5	78636861	78648725	LHFPL2
4157	chr19	11522486	11524131	ECSIT
7832	chr6_GL000252v2_alt	2035780	2040041	HCG20
6098	chr3	194332244	194338047	CPN2
4427	chr19	47130872	47140770	SAE1
4092	chr18	51022776	51063953	SMAD4
6787	chr5	137745094	137757122	HNRNPA0
6302	chr3	98921685	98925481	DCBLD2
2070	chr12	52131433	52150833	OR7E47P
6721	chr5	115505183	115546358	FEM1C
6910	chr5	173310917	173331358	STC2
5455	chr20	5940655	5956915	TRMT6
4716	chr2	159501328	159517541	LOC643072
424	chr1	20485076	20487169	CAMK2N1
8684	chr8	73862428	73879855	UBE2W
8840	chr9	125260514	125274131	GAPVD1
6428	chr4	15679028	15695997	FAM200B
3588	chr17	29173833	29178219	MYO18A
8228	chr7	47781619	47787853	C7orf69
1985	chr12	31295663	31326142	FLJ13224
1357	chr11	110182291	110184547	ZC3H12C
5896	chr3	130094255	130112498	FAM86HP
2667	chr14	50635295	50670681	SAV1
1600	chr11	65381269	65383717	SLC25A45
2312	chr13	102797781	102825368	BIVM-ERCC5
6467	chr4	17811691	17815522	NCAPG
1062	chr10	17198940	17201524	TRDMT1
6047	chr3	18097805	18100930	LOC339862

3510	chr17	1266491	1270007	BHLHA9
9240	chrX	14877798	14879167	MOSPD2
7483	chr6	21022110	21030556	LINC00581
7	chr1	1019037	1033644	AGRN
1141	chr10	50598114	50629616	SGMS1
9282	chrX	2490190	2501198	ZBED1
4119	chr18	73323563	73327869	LINC02582
5062	chr2	43158439	43160194	LINC02580
9068	chr9	67721143	67728965	FAM27B
7028	chr5	5271233	5273321	ADAMTS16
3979	chr17	82417034	82422853	OGFOD3
1803	chr12	109948589	109949716	GIT2
3597	chr17	30599664	30615199	SH3GL1P2
3602	chr17	30970082	30977590	DPRXP4
6429	chr4	156905395	156907720	PDGFC
2629	chr14	35118937	35130974	KIAA0391
8790	chr9	111959195	111961112	MIR4668
4507	chr19	57361916	57383799	TRAPPC2B
547	chr1	23017804	23022857	KDM1A
7733	chr6	75747117	75758608	MYO6
2523	chr13	98522489	98572220	STK24
5732	chr22	43088200	43090413	TTLL1
5050	chr2	38537410	38603780	HNRNPLL
3369	chr16	66928894	66942601	CES2
9087	chr9	73130891	73135521	ANXA1
9092	chr9	75084689	75088376	NMRK1
8281	chr7	6728282	6737146	PMS2CL
1070	chr10	2108895	2113341	MIR6072
1995	chr12	32115438	32220555	BICD1
8107	chr7	1552224	1557161	TMEM184A
7967	chr7	104159032	104210142	ORC5
8984	chr9	32568998	32575036	NDUFB6
6391	chr4	139157497	139159177	ELF2
8372	chr7	98280634	98300107	BRI3
5650	chr22	29764995	29772196	UQCR10
8261	chr7	65064382	65071682	SNORA22C
1218	chr10	73622916	73632766	USP54
4609	chr2	105360211	105398337	FHL2
576	chr1	235365633	235369812	TBCE
5386	chr20	46364013	46365804	SLC35C2
3156	chr16	14049601	14051645	MKL2
8345	chr7	90345261	90365302	GTPBP10
615	chr1	248730557	248732141	LYPD8
287	chr1	165627610	165639149	MGST3

7671	chr6	49540049	49542766	C6orf141
8926	chr9	135908006	135924393	CAMSAP1
7891	chr6_GL000255v2_alt	1313158	1317749	ZNRD1ASP
4109	chr18	63414416	63424439	VPS4B
4759	chr2	176269091	176351056	MTX2
3651	chr17	40137347	40157278	CASC3
40	chr1	110336900	110339153	RBM15-AS1
9140	chr9	95081136	95088730	MIR23B
1316	chr10	99660067	99687421	ENTPD7
4103	chr18	58222691	58242828	NEDD4L
123	chr1	145989533	145998977	TXNIP
5895	chr3	129626353	129629205	PLXND1
5805	chr3	107430336	107433470	LINC01990
2472	chr13	56732865	56736673	PRR20E
6811	chr5	140710317	140712000	VTRNA1-1
2899	chr15	43902612	43905457	PIN4P1
2424	chr13	42045955	42053438	DGKH
5395	chr20	49034322	49041615	CSE1L-AS1
3525	chr17	1665062	1685763	PRPF8
7513	chr6	26571030	26572460	LOC105374988
6142	chr3	28240664	28312996	CMC1
5278	chr20	17963653	17978869	SNX5
1403	chr11	124621075	124641663	
3060	chr15	79311091	79319259	TMED3
8223	chr7	4657220	4659372	FOXK1
6478	chr4	184534000	184538532	LINC02427
881	chr1	86772343	86775927	SH3GLB1
4527	chr19	631488	634978	POLRMT
3524	chr17	16534339	16536854	ZNF287
7253	chr6	109007750	109010907	SESN1
3000	chr15	67464392	67465309	IQCH-AS1
6743	chr5	130549099	130553471	HINT1
7264	chr6	111464301	111483335	TRAF3IP2-AS1
7443	chr6	158706395	158708649	SYTL3
2630	chr14	35291785	35298980	PSMA6
2389	chr13	30869175	30871023	LINC00545
5612	chr22	18073391	18087288	PEX26
752	chr1	45597979	45622381	CCDC17
4352	chr19	42212601	42223861	DEDD2
3279	chr16	30893077	30898037	CTF1
2939	chr15	55351307	55358481	MIR628
4049	chr18	23453791	23456238	RIOK3
4197	chr19	15427175	15433690	WIZ
925	chr1	94712921	94714806	MIR378G

7272	chr6	113857339	113860474	MARCKS
7582	chr6	33197303	33206913	RXRB
7684	chr6	53983360	53985658	MLIP-IT1
906	chr1	93346083	93383088	DR1
5413	chr20	50482442	50485150	PTPN1
1725	chr11	93178142	93202560	SLC36A4
4632	chr2	112480587	112488774	TTL
613	chr1	247311967	247369661	ZNF496
2096	chr12	54324968	54335974	COPZ1
1700	chr11	86115629	86117397	PICALM
2902	chr15	44515304	44532020	EIF3J-DT
5950	chr3	148901628	148904772	CPA3
6089	chr3	190343429	190346935	CLDN1
650	chr1	28351294	28353644	PHACTR4
5553	chr21	39436169	39450021	LCA5L
5388	chr20	4685473	4687231	PRNP
6518	chr4	31460494	31462654	LINC02501
2881	chr15	41618652	41626589	MGA
4656	chr2	127368983	127389359	MAP3K2
2398	chr13	34155201	34158347	LINC02343
909	chr1	93553123	93684355	BCAR3
2394	chr13	32823975	32826325	LINC00423
6063	chr3	184705619	184712606	MAGEF1
8339	chr7	84700540	84702813	LOC101927378
7538	chr6	28140673	28163915	ZNF192P1
2025	chr12	47277402	47279517	LOC105369747
370	chr1	186306895	186382564	TPR
4317	chr19	38798788	38800559	LGALS7B
3065	chr15	81993357	81995837	MEX3B
934	chr1	99971244	99974043	SLC35A3
8007	chr7	12208936	12231858	TMEM106B
1314	chr10	99415212	99444454	GOT1
1577	chr11	63937955	63940118	NAA40
590	chr1	23957891	23967097	PNRC2
6433	chr4	158277763	158279124	TMEM144
7065	chr5	61455636	61558646	C5orf64
2441	chr13	47214456	47216781	HTR2A
3863	chr17	71070837	71073867	CASC17
950	chr10	102064437	102075378	HPS6
6439	chr4	165325745	165339591	MSMO1
3791	chr17	56956577	56962056	COIL
4426	chr19	47105404	47117946	ZC3H4
8846	chr9	126802878	126832543	ZBTB43
1583	chr11	64303192	64305504	ESRRA

5352	chr20	396720	404110	RBCK1
7576	chr6	31805131	31807767	LSM2
1483	chr11	33256121	33266028	HIPK3
3398	chr16	69335796	69350688	COG8
8831	chr9	124331878	124414265	LOC100129034
3280	chr16	30913815	30924885	FBXL19
4016	chr17_KI270857v1_alt	777509	797683	GGNBP2
6998	chr5	38458346	38469521	EGFLAM
6605	chr4	70685592	70699658	UTP3
9252	chrX	154376860	154385535	EMD
6969	chr5	27289507	27291656	PURPL
5259	chr2	99463088	99489574	REV1
5389	chr20	47307861	47320713	LOC100131496
5160	chr2	69429769	69438670	NFU1
1203	chr10	71964211	71968046	CHST3
3913	chr17	76550414	76560445	SNHG16
2093	chr12	53993971	54005905	HOXC-AS1
978	chr10	110397440	110401411	SMNDC1
4925	chr2	230326197	230337938	SP140L
3682	chr17	43272016	43276915	RNU2-1
683	chr1	34777619	34789364	GJB3
5220	chr2	86717606	86736120	RNF103-CHMP3
2811	chr14	94079585	94083900	IFI27L1
4161	chr19	11735448	11740359	ZNF823
3127	chr15_KI270850v1_alt	321670	331702	RPS17
8941	chr9	137183006	137193188	SSNA1
5149	chr2	66247304	66249585	MIR4778
6877	chr5	1628295	1636371	LOC728613
5770	chr22	50523927	50538903	ODF3B
5351	chr20	38959632	38972074	DHX35
8487	chr8	140991552	141000938	PTK2
3042	chr15	75149110	75151581	C15orf39
1375	chr11	118238095	118253017	MPZL3
2227	chr12	77018420	77064615	E2F7
8797	chr9	113150068	113166005	SLC31A2
8997	chr9	34116193	34128463	DCAF12
6689	chr5	102744010	102907694	PAM
8516	chr8	144372871	144376504	ADCK5
4222	chr19	18192346	18193856	MPV17L2
8569	chr8	26447431	26458306	PNMA2
2106	chr12	56126585	56136056	ESYT1
8947	chr9	137424247	137431472	NOXA1
4953	chr2	237589094	237593462	RAB17
6684	chr4_KI270786v1_alt	141724	145052	FRG1-DT

8054	chr7	139229826	139239879	UBN2
8411	chr8	106454924	106456679	OXR1
89	chr1	120413964	120442687	PFN1P2
8928	chr9	136351029	136355489	GPSM1
848	chr1	75721351	75734326	ACADM
8338	chr7	84672807	84676727	LOC101927378
8443	chr8	123057559	123058952	TBC1D31
2582	chr14	22569120	22590336	DAD1
9019	chr9	37544971	37593509	FBXO10
8415	chr8	10827965	10841708	PINX1
2655	chr14	49615552	49626661	MGAT2
3696	chr17	44137011	44139589	C17orf53
6680	chr4	99562527	99565996	MTTP
66	chr1	115166490	115171600	TSPAN2
8091	chr7	151685611	151690337	PRKAG2
7623	chr6	3803112	3804721	FAM50B
383	chr1	192825472	192828438	RGS2
7923	chr6_GL000256v2_alt	2038256	2045263	FLOT1
4582	chr1_KI270713v1_random	20757	24711	RNU1-3
8692	chr8	80028509	80031208	MRPS28
2363	chr13	20890811	20905881	XPO4
2875	chr15	40969997	41006987	CHAC1
6994	chr5	36859122	36897669	NIPBL
2923	chr15	50658476	50688860	TRPM7
755	chr1	45801646	45805360	MAST2
5046	chr2	37664063	37673360	CDC42EP3
3611	chr17	32227789	32232050	RHBDL3
1994	chr12	32107175	32114741	BICD1
9070	chr9	69034876	69040760	FXN
8909	chr9	132394890	132408091	TTF1
2656	chr14	49628684	49649224	DNAAF2
5397	chr20	49218448	49232565	DDX27
5429	chr20	51764036	51768137	ATP9A
1523	chr11	47756532	47769472	FNBP4
8524	chr8	144785591	144801263	RPL8
1022	chr10	124151812	124153824	CHST15
2725	chr14	67412596	67415738	PLEK2
4846	chr2	202868443	202872623	ICA1L
9242	chrX	150687339	150698493	MTMR1
3859	chr17	69172342	69177105	ABCA6
9196	chrX	119465336	119473864	SLC25A5-AS1
2050	chr12	50084189	50097823	SMARCD1
9319	chrX	48903077	48914047	SLC35A2
8350	chr7	91939924	91948947	AKAP9

6334	chr4	109814656	109834154	RRH
7525	chr6	27285153	27287825	POM121L2
6911	chr5	173453614	173456903	MIR8056
9292	chrX	40174267	40178921	BCOR
2951	chr15	58769968	58774187	MINDY2
3803	chr17	59685703	59724063	VMP1
850	chr1	77212172	77221326	PIGK
8915	chr9	133325983	133337382	SURF6
6599	chr4	6762857	6764331	KIAA0232
9114	chr9	89151911	89155618	SHC3
7329	chr6	133883848	133892830	TARID
273	chr1	161747036	161762940	DUSP12
1422	chr11	12911901	12956570	LINC00958
499	chr1	222642672	222726770	AIDA
6754	chr5	132475056	132480951	IRF1
3234	chr16	2834504	2838011	ZG16B
4676	chr2	135206350	135210309	SNORA40B
839	chr1	70203053	70237482	SRSF11
4841	chr2	202198009	202207165	SUMO1
7389	chr6	14741085	14745908	LINC01108
640	chr1	27365403	27367461	MAP3K6
1193	chr10	70152386	70172259	SAR1A
8303	chr7	74450864	74465825	GTF2IRD1
7449	chr6	159781173	159795871	TCP1
1439	chr11	14490633	14521872	COPB1
6625	chr4	76957917	76965328	11-Sep
5032	chr2	36514502	36563453	FEZ2
5349	chr20	38446244	38453534	SNORA60
1665	chr11	72076648	72104961	NUMA1
6252	chr3	51670705	51675905	TEX264
4117	chr18	701810	712550	ENOSF1
5815	chr3	11192639	11266262	HRH1
8100	chr7	152422719	152437993	KMT2C
2702	chr14	60089930	60124277	PCNX4
3373	chr16	67207246	67212599	MIR328
5285	chr20	23348429	23359987	NXT1
1943	chr12	18721376	18726423	PLCZ1
8396	chr8	101024375	101027901	FLJ42969
8919	chr9	133990499	133992017	VAV2
2636	chr14	37578837	37582097	FOXA1
4376	chr19	43700325	43702602	IRGC
7425	chr6	153088921	153091915	RGS17
6356	chr4	122109268	122110934	KIAA1109
4981	chr2	26272510	26278580	HADHB

9186	chrX	109536199	109538274	NXT2
8910	chr9	132666268	132698689	DDX31
5000	chr2	27796721	27798821	MRPL33
148	chr1	148748729	148752605	NUDT4B
5823	chr3	112988048	112993970	GTPBP8
3982	chr17	8248268	8252040	PFAS
4854	chr2	20509489	20511109	RHOB
1507	chr11	45912645	45918961	PEX16
4005	chr17_KI270857v1_alt	1155567	1159920	LHX1-DT
5558	chr21	41640719	41643070	LINC00111
5286	chr20	23362241	23369520	GZF1
974	chr10	107545293	107548671	SORCS1
2599	chr14	24270084	24273422	RABGGTA
9234	chrX	13651051	13655053	TCEANC
9083	chr9	72556734	72558820	TMC1
1960	chr12	26121902	26129006	BHLHE41
3259	chr16	30059717	30064034	ALDOA
609	chr1	246138275	246143354	SMYD3
5528	chr21	35216937	35219199	RUNX1
8165	chr7	27644366	27648010	HIBADH
1232	chr10	75407839	75409899	LOC101929234
6318	chr4	101410953	101413555	FLJ20021
3152	chr16	12610666	12615894	MIR4718
6420	chr4	151669803	151671390	GATB
4678	chr2	135968533	135987667	DARS-AS1
3678	chr17	42997255	43007748	RPL27
3394	chr16	690806	693732	WDR24
1510	chr11	46614146	46620254	HARBI1
6399	chr4	139605507	139609539	SETD7
4566	chr19_GL949747v2_alt	173921	180681	RPS9
2747	chr14	69139235	69155710	DCAF5
3707	chr17	45032781	45052621	DCAKD
7666	chr6	4686464	4690058	CDYL
888	chr1	88885486	88892294	GTF2B
8530	chr8	17153436	17157593	ZDHHC2
194	chr1	15351482	15355225	LOC101927417
9170	chrX	101405774	101412224	HNRNPH2
8770	chr9	107710476	107713566	KLF4
8760	chr9	104747898	104750648	NIPSNAP3A
5496	chr21	28880716	28886233	N6AMT1
353	chr1	180521258	180522801	ACBD6
4602	chr2	10448888	10452916	LOC101929715
2864	chr15	40027714	40051968	SRP14-AS1
8496	chr8	142732126	142734528	THEM6

5373	chr20	45035194	45045935	KCNS1
8550	chr8	21917937	21919576	XPO7
1105	chr10	32444878	32448966	CCDC7
6993	chr5	36688602	36691804	SLC1A3
6663	chr4	88280892	88285898	PPM1K
3529	chr17	17189700	17202571	PLD6
8152	chr7	24810230	24822443	GSDME
4921	chr2	227942172	227944172	DAW1
7933	chr6_GL000256v2_alt	4857480	4868751	PHF1
1248	chr10	80077120	80083697	TMEM254
4179	chr19	1352265	1361780	MUM1
7761	chr6	85488522	85498376	NT5E
5637	chr22	26480461	26487008	HPS4
2309	chr13	102592426	102690044	TPP2
259	chr1	160248423	160267690	LOC100287049
5037	chr2	37079644	37087432	HEATR5B
6145	chr3	30341021	30344119	LINC01985
4312	chr19	38302410	38305846	C19orf33
8240	chr7	5512086	5515509	FBXL18
4000	chr17_GL000258v2_alt	385956	394181	ARL17B
6529	chr4	38159617	38161939	TBC1D1
4238	chr19	18935876	18943399	HOMER3
629	chr1	26429099	26433821	DHDDS
5946	chr3	14471787	14481286	GRIP2
3039	chr15	74887696	74907034	MPI
7557	chr6	29790693	29792518	HLA-V
8491	chr8	141203400	141205202	SLC45A4
1067	chr10	18680895	18686689	ARL5B
5606	chr22	16608341	16610387	TPTEP1
8700	chr8	85106008	85110408	LRRCC1
4535	chr19	708637	709624	PALM
4058	chr18	31682007	31685100	B4GALT6
2723	chr14	67324743	67339026	ATP6V1D
3991	chr17	899943	921010	LOC101927727
3590	chr17	29387665	29411180	TAOK1
6683	chr4_GL000257v2_alt	31875	44995	YTHDC1
7612	chr6	36884382	36900180	C6orf89
1547	chr11	61305561	61335959	TKFC
7812	chr6_GL000251v2_alt	3138223	3148709	GPANK1
1121	chr10	3808911	3812129	LOC105376365
3212	chr16	22605027	22609357	LOC653786
7197	chr5	96701100	96732411	CAST
427	chr1	205207826	205212305	DSTYK
3657	chr17	41686859	41695394	EIF1

2297	chr12_GL383552v1_alt	131543	134077	
1363	chr11	112072599	112079095	NKAPD1
7416	chr6	151003996	151016814	MTHFD1L
1938	chr12	16346406	16372884	MGST1
2556	chr14	103625389	103659142	KLC1
5910	chr3	134176861	134252845	RYK
6956	chr5	1935355	1937834	CTD-2194D22.4
1356	chr11	110173553	110179339	ZC3H12C
6000	chr3	15742834	15798915	ANKRD28
6346	chr4	118683332	118688302	METTL14
7209	chr5_GL339449v2_alt	506962	510922	SERF1B
3199	chr16	21768567	21774792	RRN3P1
8378	chr7	99436585	99440948	PTCD1
5434	chr20	53596892	53597862	LOC105372672
4672	chr2	132651195	132673172	LYPD1
7874	chr6_GL000254v2_alt	1941315	1960405	PPP1R10
3022	chr15	71856559	71858007	NR2E3
1211	chr10	73041499	73045182	P4HA1
892	chr1	89522819	89526263	LRRC8B
4978	chr2	25339727	25342888	DNMT3A
1812	chr12	110464196	110504217	GPN3
3978	chr17	82295879	82299984	LINC01970
5973	chr3	150751467	150768198	SIAH2
3670	chr17	42658091	42663958	TUBG2
710	chr1	39756559	39767489	OXCT2
6908	chr5	173084836	173086894	CREBRF
352	chr1	180490481	180504023	ACBD6
2031	chr12	48343885	48354531	ZNF641
8346	chr7	903993	907488	ADAP1
782	chr1	54050635	54056342	TMEM59
4844	chr2	202262087	202276227	NOP58
7712	chr6	71382140	71421417	MIR30A
6544	chr4	39697054	39713185	UBE2K
1551	chr11	61780485	61805900	FEN1
8813	chr9	115670499	115674869	LOC101928775
3255	chr16	29907477	29928755	KCTD13
1608	chr11	6559703	6579606	RRP8
422	chr1	204514917	204532167	MDM4
3750	chr17	48890400	48901856	ATP5MC1
475	chr1	21625757	21628307	RAP1GAP
3767	chr17	50376341	50383936	EME1
3528	chr17	1708797	1719627	MIR22
3948	chr17	80825533	80831025	LOC101928855
1425	chr11	129880222	129896038	NFRKB

8698	chr8	81828783	81843078	SNX16
4452	chr19	486590	491439	MADCAM1
3645	chr17	39738290	39742303	GRB7
5123	chr2	61597812	61599878	XPO1
6208	chr3	47777343	47781712	SMARCC1
1772	chr12	103857996	103860313	NT5DC3
5489	chr21	16585711	16595642	MIR125B2
2468	chr13	52445639	52454687	VPS36
7156	chr5	82934118	82971019	LINC01338
5210	chr2	85411725	85414634	CAPG
7641	chr6	42877982	42891479	RPL7L1
8245	chr7	5574868	5576129	FSCN1
1078	chr10	23511765	23513675	OTUD1
5438	chr20	53751800	53754959	SUMO1P1
3155	chr16	13956960	13959906	ERCC4
7955	chr7	101814055	101817628	CUX1
9124	chr9	92384265	92388623	OGN
9337	chrX	54041643	54045966	PHF8
2385	chr13	30420194	30423097	LINC01058
4321	chr19	3932363	3938690	NMRK2
4818	chr2	200404939	200461138	KCTD18
2461	chr13	50100859	50130899	DLEU2
2594	chr14	24112965	24116502	DCAF11
2334	chr13	110943353	110949325	LINC00431
2216	chr12	7580315	7586647	CD163
6161	chr3	36165292	36168118	STAC
1778	chr12	104285679	104297509	TXNRD1
200	chr1	153726541	153731891	INTS3
7252	chr6	108734795	108739123	LINC00222
2769	chr14	74473350	74484665	MIR4709
1475	chr11	308103	312548	IFITM2
911	chr1	93740434	93777341	MIG7
431	chr1	206685070	206728098	MAPKAPK2
4078	chr18	45792615	45794474	SIGLEC15
2849	chr15	34036491	34042697	AVEN
2738	chr14	68400179	68403404	LOC100996664
1127	chr10	43133301	43141598	CSGALNACT2
8895	chr9	129828036	129840864	C9orf78
6220	chr3	4865678	4869350	BHLHE40-AS1
3454	chr16	85677174	85681919	GINS2
2975	chr15	64160409	64164314	PPIB
117	chr1	145858550	145860566	PIAS3
7810	chr6_GL000251v2_alt	2677463	2695766	HCG27
8236	chr7	54829554	54832912	LOC100996654

3757	chr17	4962218	4983719	MIR6865
4243	chr19	19403644	19406766	GATAD2A
7654	chr6	43509771	43535364	POLR1C
3179	chr16	1869846	1871296	MEIOB
1359	chr11	110360625	110361753	LOC105369486
6191	chr3	44973825	45001292	EXOSC7
3071	chr15	82984424	82988965	RAMMET
2326	chr13	110347243	110349820	MIR8073
6253	chr3	5187617	5202895	EDEM1
6698	chr5	10681344	10762118	DAP
3050	chr15	76291654	76314236	ETFA
4417	chr19	46344569	46351967	PPP5C
338	chr1	176199025	176209062	COP1
8131	chr7	18893847	18898179	TWIST1
8802	chr9	113409071	113413908	POLE3
34	chr1	109273768	109279107	PSRC1
9003	chr9	34980029	35006282	DNAJB5
6015	chr3	161265866	161299597	NMD3
4473	chr19	52043673	52050210	ZNF432
4400	chr19	45506110	45513680	VASP
2820	chr14_GL000194v1_random	59396	116231	MAFIP
6938	chr5	179731603	179732894	MAML1
1830	chr12	113180401	113186447	DDX54
3475	chr16	88568116	88574788	ZC3H18
2884	chr15	42033680	42036592	PLA2G4E
128	chr1	146297336	146302245	NUDT4B
137	chr1	147927362	147949496	GPR89B
7178	chr5	94088963	94113713	FAM172A
1996	chr12	32398351	32402694	FGD4
912	chr1	93778207	93782952	MIG7
5631	chr22	23750599	23753975	ZNF70
177	chr1	151153861	151169304	LYSMD1
3513	chr17	1487167	1490105	MYO1C
2722	chr14	65410387	65414328	FUT8-AS1
5729	chr22	42848160	42859933	ARFGAP3
5642	chr22	27902578	27935235	PITPNB
4520	chr19	58516870	58521243	ZBTB45
1190	chr10	69438040	69440135	TSPAN15
294	chr1	166837245	166866517	POGK
3043	chr15	75334899	75337074	COMMD4
8873	chr9	128682148	128685854	SET
5361	chr20	43590760	43593114	IFT52
7309	chr6	129488114	129492873	ARHGAP18

3353	chr16	57164572	57200962	FAM192A
2717	chr14	64702535	64711329	PLEKHG3
2	chr1	100130971	100157064	TRMT13
2403	chr13	36838896	36852272	RFXAP
8260	chr7	6463818	6484851	KDELR2
4123	chr18	75204833	75209364	ZADH2
4101	chr18	57615396	57623005	NARS
7205	chr5_GL339449v2_alt	283375	291573	LOC101928924
5707	chr22	40495801	40500870	LOC101927257
4691	chr2	149420205	149424416	LYPD6
7839	chr6_GL000252v2_alt	3052987	3055452	LSM2
5165	chr2	70072570	70086005	PCBP1-AS1
5924	chr3	138832855	138836521	PIK3CB
8058	chr7	13990160	13992906	ETV1
817	chr1	64935348	64950251	JAK1
8227	chr7	47675056	47677291	C7orf65
5323	chr20	35549032	35562401	ERGIC3
1395	chr11	122407257	122424072	MIR100HG
7448	chr6	159710512	159738555	SOD2
6477	chr4	184469499	184481808	IRF2
8029	chr7	130293610	130295005	CPA4
4924	chr2	230218910	230221027	SP110
6535	chr4	39044846	39049984	KLHL5
5656	chr22	30324127	30327994	TBC1D10A
6671	chr4	94487388	94657073	PDLIM5
6785	chr5	136523419	136547578	TRPC7
8577	chr8	28385715	28389019	ZNF395
4174	chr19	12946007	12956283	RAD23A
2585	chr14	22870184	22873943	LRP10
5655	chr22	30236832	30259204	LIF
4331	chr19	40276141	40286319	MIR641
562	chr1	23366410	23375371	ZNF436
8305	chr7	74795883	74799724	NCF1
9061	chr9	6565233	6568561	GLDC
5979	chr3	15254359	15266890	SH3BP5-AS1
3535	chr17	18180372	18188964	ALKBH5
5100	chr2	54554114	54668373	SPTBN1
1413	chr11	126211126	126214497	FAM118B
6121	chr3	196784359	196802752	PAK2
794	chr1	58629255	58645156	TACSTD2
8799	chr9	113213062	113225886	FKBP15
8704	chr8	86440285	86445626	RMDN1
1454	chr11	195749	220450	RIC8A
3990	chr17	8914677	8917123	PIK3R5

855	chr1	77887462	77891824	NEXN-AS1
9338	chrX	54162540	54188537	FAM120C
4530	chr19	6599565	6603031	CD70
8680	chr8	70394628	70403377	NCOA2
7518	chr6	26957143	26958566	LINC00240
2533	chr14	100345101	100379161	WARS
6006	chr3	159748949	159769710	IQCJ-SCHIP1-AS1
2964	chr15	63133989	63139295	LACTB
3575	chr17	28391515	28395044	SLC46A1
637	chr1	26898892	26902238	GPATCH3
3033	chr15	73560999	73636112	NPTN-IT1
8209	chr7	43715054	43731112	COA1
5599	chr21	7095784	7099269	LOC102723360
7328	chr6	133800639	133818599	LINC01312
751	chr1	45549371	45558465	AKR1A1
7109	chr5	73369258	73372527	LINC02230
4256	chr19	2503079	2505021	GADD45B
3101	chr15	91560542	91562353	CRAT37
2506	chr13	79480308	79528086	NDFIP2-AS1
1452	chr11	18583223	18593445	UEVLD
4280	chr19	34170528	34218762	LSM14A
8631	chr8	48625771	48659353	LOC101929217
4226	chr19	18303805	18307401	LSM4
1667	chr11	72791919	72794914	STARD10
2678	chr14	52766742	52767719	GNPNAT1
2699	chr14	58402575	58431793	TIMM9
5665	chr22	31158606	31172467	RNF185
2509	chr13	80341445	80343384	SPRY2
6955	chr5	1874798	1896088	IRX4
5390	chr20	47329079	47334944	LOC100131496
3173	chr16	15898792	15901064	FOPNL
4524	chr19	5902027	5905280	NDUFA11
4026	chr18	107389	110648	ROCK1P1
8257	chr7	6392361	6420105	RAC1
3313	chr16	4289551	4300156	GLIS2
2170	chr12	65813003	65886698	RPSAP52
5703	chr22	39288826	39292633	SNORD83B
1638	chr11	67076930	67080656	RHOD
8671	chr8	66605764	66616736	MYBL1
1490	chr11	34233539	34235290	ABTB2
5410	chr20	50371365	50373238	LINC01271
5735	chr22	43152706	43162455	TSPO
8320	chr7	76548312	76592280	LOC100133091
3114	chr15	98547808	98549501	FAM169B

6587	chr4	56449826	56504197	SRP72
4673	chr2	135051240	135056167	RAB3GAP1
5669	chr22	31659090	31663405	PISD
1313	chr10	987756	1004837	GTPBP4
2625	chr14	34871736	34877142	BAZ1A
8272	chr7	66627595	66630416	KCTD7
6415	chr4	147670517	147685575	PRMT9
3840	chr17	65098483	65101288	LOC100507002
7356	chr6	138682751	138695914	FLJ46906
1458	chr11	2176072	2177238	MIR4686
1828	chr12	112140093	112143230	TRAFD1
3915	chr17	76678881	76680955	LOC105274304
4490	chr19	55397566	55401792	SNORD157
5305	chr20	323144	328030	SOX12
7099	chr5	70899887	70904053	SERF1B
2190	chr12	69584458	69608784	CCT2
8551	chr8	22088146	22100706	FAM160B2
7964	chr7	103346076	103373399	PSMC2
7614	chr6	37055207	37057565	FGD2
6598	chr4	67534357	67546732	CENPC
8554	chr8	22438996	22443174	PPP3CC
3008	chr15	69042866	69046965	NOX5
7592	chr6	34233254	34251879	MIR6835
8116	chr7	157334751	157342412	DNAJB6
9191	chrX	116283768	116286376	AGTR2
6685	chr5	100046298	100050658	LOC100133050
1652	chr11	68028497	68034977	NDUFS8
2069	chr12	52050313	52054724	NR4A1
2127	chr12	57511126	57550724	MBD6
812	chr1	63433142	63448337	ALG6
6408	chr4	143808655	143812743	LOC105377458
8868	chr9	128338666	128346881	SLC27A4
7495	chr6	26025242	26028623	HIST1H4B
3223	chr16	2598194	2609779	LOC652276
1543	chr11	60125454	60128425	MS4A2
618	chr1	248869869	248875546	ZNF692
397	chr1	200335893	200337348	LINC00862
7782	chr6	96887300	96901058	NDUFAF4
7996	chr7	116440856	116444557	CAV2
2960	chr15	60411221	60480609	ICE2
6758	chr5	132554412	132574168	RAD50
1618	chr11	65959258	65970662	SART1
2746	chr14	69077607	69085370	DCAF5
567	chr1	234642619	234659507	LOC101927787

675	chr1	32680283	32691029	SYNC
8796	chr9	112484503	112486832	KIAA1958
1155	chr10	58263215	58298612	CISD1
8739	chr8	98288431	98296019	NIPAL2
8009	chr7	123742806	123754564	WASL
334	chr1	174814264	174818791	RABGAP1L
4432	chr19	47482056	47486483	KPTN
2316	chr13	106154541	106158672	LINC00460
7216	chr5_KI270897v1_alt	272556	301391	SMN2
4410	chr19	45994200	46004805	CCDC61
3425	chr16	736161	741917	NARFL
7162	chr5	86617150	86620319	COX7C
1151	chr10	55144772	55147227	PCDH15
8216	chr7	44558690	44583574	DDX56
7276	chr6	116258187	116281347	DSE
8493	chr8	142659149	142673820	JRK
4414	chr19	46180050	46182250	IGFL2-AS1
305	chr1	168245481	168251796	ANKRD36BP1
8387	chr7_KI270809v1_alt	35146	90533	LOC100133091
8188	chr7	33032640	33041637	NT5C3A
5340	chr20	37074147	37097250	RBL1
6303	chr3	98969299	98973324	DCBLD2
7375	chr6	143677493	143689676	PHACTR2
7692	chr6	56824910	56842405	DST
5974	chr3	151292161	151317762	GPR87
3921	chr17	7838014	7886104	NAA38
8242	chr7	5546998	5550965	ACTB
5957	chr3	14939646	14968609	FGD5-AS1
3784	chr17	55264871	55266751	HLF
4782	chr2	186590342	186604030	ITGAV
8110	chr7	155823992	155826007	SHH
6763	chr5	133047730	133063907	HSPA4
1559	chr11	62567549	62575723	EEF1G
5600	chr21	7657077	7660942	LOC102723360
4089	chr18	50228254	50242622	CFAP53
8925	chr9	135471515	135481979	PPP1R26
8573	chr8	27361466	27366238	PTK2B
5302	chr20	3208058	3216995	ITPA
6979	chr5	32296770	32315122	MTMR12
6996	chr5	37248102	37273348	CPLANE1
3532	chr17	17693138	17702400	RAI1
3283	chr16	31029731	31057149	STX4
1554	chr11	61961384	61976640	FTH1
7367	chr6	142297262	142308897	ADGRG6

3664	chr17	42456575	42469807	ATP6V0A1
5085	chr2	47781204	47784832	MSH6
2035	chr12	48949624	48959361	ARF3
4090	chr18	50307237	50309752	CXXC1
8482	chr8	133570525	133571287	ST3GAL1
5881	chr3	127589353	127595624	TPRA1
8102	chr7	152674679	152678137	XRCC2
1840	chr12	116815080	116820117	HRK
1871	chr12	121711776	121714412	TMEM120B
4517	chr19	58377203	58390321	ZNF837
4055	chr18	26548132	26550603	KCTD1
3249	chr16	29391604	29426480	NPIPB11
3226	chr16	27202186	27205398	KDM8
4041	chr18	21739470	21745746	MIB1
9100	chr9	79164475	79166485	LOC101927450
1673	chr11	73782307	73790428	MRPL48
6494	chr4	189939335	189944485	FRG1
5016	chr2	31378573	31385893	XDH
2868	chr15	40592768	40596271	KNL1
6262	chr3	52768919	52771802	NEK4
3509	chr17	11992738	11998536	ZNF18
404	chr1	201524266	201528725	RPS10P7
9079	chr9	72147937	72153957	GDA
2535	chr14	100585746	100588442	BEGAIN
5900	chr3	131008968	131034486	NEK11
5275	chr20	13768392	13825448	NDUFAF5
2358	chr13	19863760	19866370	ZMYM5
2574	chr14	20986102	20999246	METTL17
8265	chr7	6575659	6587757	ZDHHC4
7569	chr6	30747587	30753773	IER3
9228	chrX	134372018	134379708	PHF6
243	chr1	15684198	15687102	PLEKHM2
705	chr1	38872644	38876656	LOC105378663
142	chr1	148387391	148390989	MIR6077
6048	chr3	182793533	182797548	ATP11B
402	chr1	201169222	201172000	TMEM9
3113	chr15	97959198	97960793	ARRDC4
754	chr1	45749860	45751725	IPP
5420	chr20	50884133	50968515	ADNP-AS1
1391	chr11	12062861	12068851	LINC02547
5444	chr20	56467558	56515598	RTF2
9386	chrY	1367182	1397492	SLC25A6
1973	chr12	28129888	28131787	CCDC91
1768	chr12	101874585	101879744	DRAM1

1805	chr12	110045611	110050156	C12orf76
4931	chr2	231612093	231616079	TEX44
654	chr1	28638692	28649850	LINC01715
8330	chr7	77695125	77702266	APTR
6361	chr4	122881611	122907334	NUDT6
8823	chr9	121145255	121146747	CNTRL
7866	chr6_GL000253v2_alt	3470889	3486911	AGPAT1
3883	chr17	74981259	74987146	CDR2L
1374	chr11	117285675	117294556	BACE1-AS
5827	chr3	114051674	114091642	QTRT2
1643	chr11	67390380	67402983	RAD9A
7714	chr6	72552645	72555230	KCNQ5
8683	chr8	73419391	73422251	STAU2-AS1
1844	chr12	118045125	118068012	WSB2
4365	chr19	43032871	43034557	PSG11
4832	chr2	201257042	201268302	CASP8
6821	chr5	141847421	141850947	PCDH1
7262	chr6	110956399	110960138	GTF3C6
3712	chr17	45140654	45162713	HEXIM1
6566	chr4	48015850	48024513	NIPAL1
2729	chr14	67625938	67628261	ARG2
2551	chr14	103256179	103258516	LOC105378183
3135	chr15_KI270905v1_alt	566269	572391	HERC2P11
5291	chr20	25621569	25627783	ZNF337-AS1
380	chr1	19249834	19266946	MRTO4
6298	chr3	97964118	97973196	RIOX2
7480	chr6	20209955	20213711	MBOAT1
8218	chr7	44732953	44737183	ZMIZ2
2241	chr12	82685979	82687825	TMTC2
9091	chr9	73437004	73438847	ANXA1
1632	chr11	66738745	66752563	C11orf80
1555	chr11	62024307	62028269	FTH1
2942	chr15	55987206	56002569	NEDD4
7768	chr6	87662870	87703425	AKIRIN2
189	chr1	151986530	151996121	S100A10
3610	chr17	32139244	32144682	RHOT1
5737	chr22	43292628	43294231	LOC101927447
6780	chr5	135362944	135403233	H2AFY
9354	chrX	70131942	70151827	IGBP1
6987	chr5	34890495	34981902	DNAJC21
3869	chr17	72361105	72363594	LINC02003
8371	chr7	98119111	98124772	LMTK2
1545	chr11	60878694	60906839	PRPF19
7939	chr7	100115521	100121805	TAF6

2452	chr13	49245855	49254899	CDADC1
2163	chr12	6557892	6571536	NOP2
6588	chr4	56640713	56642546	HOPX
5586	chr21	44988431	45026068	PICSAR
5793	chr3	101674263	101681688	ZBTB11
9025	chr9	38048738	38068879	SHB
7594	chr6	34412672	34426935	RPS10
416	chr1	203887885	203890231	SNRPE
3550	chr17	19359425	19361347	B9D1
6860	chr5	154413673	154416001	SAP30L-AS1
3865	chr17	71422943	71425734	CASC17
1037	chr10	130717598	130718877	MIR378C
2847	chr15	32534041	32538233	LINC02256
1992	chr12	31967019	32005293	KIAA1551
266	chr1	16117220	16161104	EPHA2
6558	chr4	41987518	42022617	SLC30A9
2259	chr12	90090331	90092087	LINC02399
7904	chr6_GL000255v2_alt	2654730	2661318	MICA
2026	chr12	47421367	47428091	LINC02156
4604	chr2	10492952	10494684	LOC101929715
5252	chr2	9818757	9821067	TAF1B
742	chr1	44775777	44777867	SNORD46
7877	chr6_GL000254v2_alt	206663	207233	LINC01556
1684	chr11	77626484	77638914	CLNS1A
2567	chr14	16091680	16095970	OR11H12
8296	chr7	73821040	73824042	CLDN4
4579	chr1_GL383519v1_alt	66257	79699	SCAMP3
6748	chr5	131606289	131637495	RAPGEF6
4246	chr19	19648846	19665413	ATP13A1
1429	chr11	130521680	130524489	ADAMTS15
307	chr1	16893649	16897251	RNVU1-18
2378	chr13	27422992	27452563	MTIF3
1969	chr12	27821377	27822783	KLHL42
4102	chr18	57818618	57821054	ATP8B1
6377	chr4	128247724	128249114	PGRMC2
2148	chr12	6380964	6386544	LTBR
9265	chrX	16830894	16872502	RBBP7
1845	chr12	118134271	118142917	PEBP1
6091	chr3	190584682	190588134	IL1RAP
374	chr1	187690512	187692469	LINC01037
7713	chr6	71477821	71480039	LINC01626
4022	chr17_KI270908v1_alt	1108298	1128233	NSFP1
8517	chr8	144398251	144409188	MIR6849
3578	chr17	28630373	28647591	KIAA0100

3986	chr17	83046288	83049379	B3GNTL1
6940	chr5	179821389	179835663	SQSTM1
25	chr1	108746291	108748681	STXBP3
3579	chr17	28709639	28719735	PROCA1
3848	chr17	67823929	67828507	BPTF
2537	chr14	100869275	100873106	MIR493
7200	chr5	98308336	98311070	LINC01846
7433	chr6	157301610	157324870	TMEM242
964	chr10	104117041	104129249	SFR1
2230	chr12	78697581	78700407	SYT1
2927	chr15	51596671	51604504	DMXL2
2447	chr13	48481473	48489985	LPAR6
9032	chr9	39870310	39877144	LOC102724238
7362	chr6	14000221	14002652	RNF182
1854	chr12	120290664	120293675	RNU4-2
4515	chr19	58358336	58365145	LOC105372483
5909	chr3	133804898	133819367	SRPRB
2671	chr14	51552663	51560769	FRMD6-AS2
4541	chr19	7624687	7644923	PCP2
2987	chr15	65551460	65572600	HACD3
5162	chr2	69740905	69757488	ANXA4
6286	chr3	87049087	87050539	LINC00506
5751	chr22	46055677	46061614	PRR34
2913	chr15	45454260	45460336	MIR147B
3594	chr17	29912805	29932789	EFCAB5
2934	chr15	54801500	54804589	LOC105370829
7760	chr6	85471787	85487428	NT5E
4607	chr2	10523084	10535940	LOC101929715
7101	chr5	71035855	71068893	GTF2H2C
3521	chr17	16209155	16227735	PIGL
8568	chr8	26150686	26159857	EBF2
5581	chr21	44424474	44431449	TRPM2-AS
70	chr1	115974598	115997991	LOC101928977
4943	chr2	23383279	23401543	KLHL29
7706	chr6	70394705	70400584	EVADR
5216	chr2	86198786	86203104	MRPL35
5411	chr20	50414733	50418779	PTPN1
6099	chr3	194482398	194486475	LINC00884
4268	chr19	29811162	29812807	CCNE1
733	chr1	43706699	43714184	ST3GAL3
5652	chr22	30037672	30039546	MIR6818
393	chr1	199224844	199227670	LOC400800
5998	chr3	157134426	157162124	CCNL1
8435	chr8	119854438	119857392	DSCC1

9312	chrX	48521016	48523164	EBP
5944	chr3	143442429	143446904	SLC9A9-AS1
8313	chr7	75914500	75917804	MIR4651
3286	chr16	31107425	31123834	KAT8
3472	chr16	88117733	88120036	LOC400553
314	chr1	170707564	170709017	PRRX1
7883	chr6_GL000254v2_alt	3513912	3521456	AGPAT1
6	chr1	101235227	101236758	LOC101928370
4945	chr2	234248990	234251339	SPP2
2986	chr15	65529679	65536513	HACD3
3980	chr17	82445389	82453087	CYBC1
4585	chr1_KI270765v1_alt	33380	37126	RNVU1-11
1093	chr10	29452339	29459454	SVIL-AS1
335	chr1	174847863	174853480	RABGAP1L
865	chr1	818027	835638	LINC00115
776	chr1	52357495	52369076	CC2D1B
4241	chr19	19318390	19340184	MAU2
3999	chr17_GL000258v2_alt	373737	380415	NSFP1
8478	chr8	130399781	130449039	ASAP1
1333	chr11	103108317	103110174	DYNC2H1
1957	chr12	25614086	25616009	LMNTD1
2189	chr12	69568820	69574098	MIR3913-2
591	chr1	23971843	23982169	SRSF10
6851	chr5	149937579	149940384	PDE6A
3889	chr17	75151246	75154277	JPT1
6173	chr3	40522990	40527479	ZNF621
7689	chr6	56334782	56336969	COL21A1
3753	chr17	48993609	49009327	IGF2BP1
9105	chr9	85211494	85214749	AGTPBP1
5433	chr20	53572785	53580818	ZNF217
6797	chr5	139293486	139321620	MATR3
8194	chr7	36152123	36153483	EEPD1
8983	chr9	32510278	32555070	DDX58
7084	chr5	66848881	66851510	MAST4
2300	chr12_KI270904v1_alt	239328	379027	TAS2R45
9133	chr9	94026135	94045691	PTPDC1
6606	chr4	72109926	72114782	NPFFR2
5077	chr2	46689478	46692061	SOCS5
7548	chr6	2852755	2855076	MIR4645
1277	chr10	91157840	91218794	PCGF5
6802	chr5	139665844	139669412	CXXC5
351	chr1	180152684	180164402	QSOX1
7249	chr6	108240795	108262701	SNX3
9317	chrX	48736362	48738514	GLOD5

2807	chr14	92111949	92123982	NDUFB1
3341	chr16	53434833	53439885	RBL2
2448	chr13	48527526	48534160	RCBTB2
2751	chr14	70552737	70603109	MED6
4289	chr19	3562173	3564110	MFSD12
7673	chr6	5003299	5005259	RPP40
4392	chr19	45077666	45097900	GEMIN7-AS1
5877	chr3	12662274	12665535	RAF1
5792	chr3	101575914	101582307	PCNP
1504	chr11	44064979	44068168	ACCS
9339	chrX	54528159	54547086	GNL3L
232	chr1	156190203	156204582	SLC25A44
4825	chr2	200785995	200788734	BZW1
9056	chr9	63837715	63840638	MIR4477A
8580	chr8	28890417	28915250	HMBOX1
8979	chr9	29331532	29334430	LINGO2
1867	chr12	121331840	121353605	ANAPC5
1412	chr11	125890288	125892603	HYLS1
54	chr1	112945360	112959827	SLC16A1
8462	chr8	126505058	126508156	FAM84B
3443	chr16	82144715	82171843	MPHOSPH6
3262	chr16	30190372	30202222	SLX1A-SULT1A3
4698	chr2	150680418	150683929	LOC101929282
3332	chr16	5070668	5074199	ALG1
5190	chr2	74468563	74476532	MRPL53
4046	chr18	22930058	22937501	RBBP8
9082	chr9	72476277	72479186	TMC1
7837	chr6_GL000252v2_alt	2898292	2919306	C6orf47
3993	chr17	9641367	9646633	USP43
6135	chr3	23667883	23669469	UBE2E1
1448	chr11	18406730	18410639	LDHC
6055	chr3	184132303	184138852	EIF2B5
4104	chr18	59895921	59906931	PMAIP1
747	chr1	45199484	45208123	ZSWIM5
1411	chr11	125621309	125630910	CHEK1
3	chr1	100265027	100276141	RTCA-AS1
5023	chr2	33069215	33074840	LTBP1
2930	chr15	52017090	52027034	MAPK6
4968	chr2	241071048	241103991	MTERF4
3186	chr16	1960059	1967908	RPS2
159	chr1	149920165	149929856	SF3B4
6837	chr5	143628929	143631090	MIR5197
6403	chr4	140255460	140272137	SCOC
5059	chr2	42279350	42289899	COX7A2L

4667	chr2	130833060	130837837	AMER3
1137	chr10	4769080	4771979	AKR1E2
8454	chr8	124518476	124542502	TATDN1
7470	chr6	170610855	170626877	PDCD2
8077	chr7	1494798	1506581	INTS1
9064	chr9	66266789	66271201	LOC642929
3343	chr16	54430850	54432299	LINC02140
1392	chr11	12084869	12107670	MICAL2
7634	chr6	41783030	41788705	TOMM6
5314	chr20	34875587	34878455	ACSS2
5787	chr3	100398190	100403552	LNP1
2427	chr13	44139638	44145645	SMIM2-AS1
9179	chrX	107131263	107132560	RBM41
3382	chr16	67892025	67900143	PSKH1
8672	chr8	66625256	66632213	MYBL1
241	chr1	156759669	156761329	PRCC
5088	chr2	48106598	48107721	FBXO11
3969	chr17	8191861	8193716	BORCS6
5897	chr3	130739374	130749549	PIK3R4
8595	chr8	37141114	37144896	LINC01605
4617	chr2	109986056	109999013	LINC01123
7226	chr6	101082546	101085605	ASCC3
6114	chr3	196331639	196346169	TM4SF19
2057	chr12	50531775	50543390	DIP2B
6982	chr5	32805712	32807394	NPR3
4350	chr19	42112925	42114895	POU2F2
7436	chr6	158054426	158059753	SYNJ2
4063	chr18	35239551	35256423	ZNF397
5940	chr3	142595021	142599218	PLS1
2458	chr13	49788022	49794031	KPNA3
2009	chr12	43790305	43810519	TWF1
3905	chr17	75935189	75942691	FBF1
5462	chr20	62303667	62310813	ADRM1
6843	chr5	14657626	14722075	LOC100130744
793	chr1	58571367	58578784	TACSTD2
2961	chr15	60485141	60487465	RORA-AS1
6093	chr3	191328236	191409772	UTS2B
5168	chr2	70189265	70192209	C2orf42
5649	chr22	29620555	29623020	NF2
3694	chr17	44069335	44088133	G6PC3
5255	chr2	99133587	99146407	TSGA10
430	chr1	206633299	206640229	DYRK3
5562	chr21	41864572	41882579	PRDM15
2138	chr12	6092636	6094451	VWF

9184	chrX	108391939	108397975	COL4A6
3334	chr16	515164	533102	LINC00235
9004	chr9	3514963	3537201	RFX3
2058	chr12	50752520	50754602	ATF1
5688	chr22	37742364	37759457	TRIOBP
3358	chr16	57753339	57802561	KIFC3
9200	chrX	119788423	119793589	RPL39
2261	chr12	91777195	91781064	LINC01619
9057	chr9	63852416	63859401	FRG1JP
7325	chr6	133563038	133571403	LINC01312
2339	chr13	113022118	113023360	MCF2L
1956	chr12	25457265	25459808	LMNTD1
2313	chr13	105917521	105920941	LINC00343
2681	chr14	52951275	52952987	FERMT2
8633	chr8	48918319	48925592	SNAI2
3215	chr16	23456732	23467110	COG7
1051	chr10	133392814	133415241	MTG1
8865	chr9	128291300	128293811	SWI5
745	chr1	44818780	44822392	BTBD19
5609	chr22	17475778	17478590	SLC25A18
5588	chr21	45286915	45299599	LOC642852
7519	chr6	26959130	26963887	LINC00240
6164	chr3	37159998	37187910	LRRFIP2
98	chr1	1302176	1310733	ACAP3
9147	chr9	96766986	96780135	ZNF510
1513	chr11	47124368	47126991	ARFGAP2
3494	chr16_KI270853v1_alt	1246591	1251038	MIR6506
2329	chr13	110615618	110632958	NAXD
3706	chr17	44980812	44982913	C1QL1
5547	chr21	38806929	38827743	ETS2
857	chr1	78832094	78833365	ADGRL4
601	chr1	244046934	244060389	ZBTB18
1516	chr11	47184609	47187653	PACSIN3
8878	chr9	128940010	128952356	NUP188
2349	chr13	114113544	114118389	RASA3
2566	chr14	105396985	105415801	TEX22
4574	chr19_KI270938v1_alt	174039	177287	RPS9
7794	chr6_GL000250v2_alt	249513	253007	HCG15
1679	chr11	76234727	76236551	WNT11
5841	chr3	119817346	119996816	NR1I2
3837	chr17	64962763	64967269	AMZ2P1
2436	chr13	45388591	45400456	SLC25A30-AS1
1073	chr10	22318932	22334654	BMI1
1241	chr10	7815399	7819546	TAF3

9123	chr9	92299278	92334443	CENPP
2996	chr15	66841210	66847508	LINC02206
8773	chr9	108056878	108057800	KLF4
8450	chr8	124270745	124274387	LOC101927588
6867	chr5	157724012	157734329	THG1L
8269	chr7	6634490	6640431	ZNF316
2002	chr12	40325146	40327372	MUC19
5066	chr2	44165478	44175927	PPM1B
4949	chr2	237085069	237114651	COPS8
484	chr1	220092943	220096955	IARS2
8662	chr8	6598591	6609170	MIR8055
1508	chr11	46241329	46250648	CREB3L1
1086	chr10	26745279	26751552	PDSS1
3775	chr17	51151623	51161581	NME1
1565	chr11	62703923	62730112	BSCL2
1648	chr11	67580825	67591211	GSTP1
4029	chr18	12305897	12311603	TUBB6
905	chr1	93250890	93253417	CCDC18
7013	chr5	42983945	43001517	FLJ32255
236	chr1	156456620	156458945	C1orf61
4363	chr19	42929959	42936128	PSG7
1235	chr10	7781922	7800831	ATP5F1C
5223	chr2	87453427	87493940	CYTOR
5406	chr20	50165347	50168230	LINC01273
5926	chr3	139346744	139406532	COPB2
8685	chr8	73955430	73974291	ELOC
75	chr1	11736022	11738980	AGTRAP
432	chr1	20696121	20702496	KIF17
2550	chr14	103121861	103125526	LINC00677
7189	chr5	95872702	95964304	LOC101929710
522	chr1	225989259	226000980	SDE2
9189	chrX	11191696	11193952	ARHGAP6
6140	chr3	25787618	25797579	OXSM
3695	chr17	44118027	44125105	HDAC5
4738	chr2	171552952	171558174	CYBRD1
610	chr1	24638519	24676378	SRRM1
7414	chr6	150856398	150859006	MTHFD1L
3520	chr17	15979859	16059524	ZSWIM7
5625	chr22	20856307	20860405	PI4KA
1705	chr11	86741352	86744734	PRSS23
7585	chr6	33297460	33300839	RGL2
3573	chr17	28318321	28321041	TMEM97
1876	chr12	122357274	122367802	CLIP1-AS1
4391	chr19	44999961	45004767	RELB

7616	chr6	3712903	3756487	PXDC1
8177	chr7	29961771	29989388	SCRN1
268	chr1	161200893	161207893	NDUFS2
8295	chr7	73736902	73740162	ABHD11
3368	chr16	66550728	66555137	CKLF-CMTM1
2401	chr13	35926135	35928984	DCLK1
749	chr1	45499208	45502037	MMACHC
830	chr1	66892581	66930094	WDR78
3539	chr17	18410732	18413360	LINC02076
5272	chr20	13028833	13031011	SPTLC3
1304	chr10	97317106	97320057	FRAT1
272	chr1	16171387	16185461	EPHA2
6405	chr4	142403848	142406635	INPP4B
7645	chr6	43025724	43028661	RRP36
4149	chr19	10926229	10933637	YIPF2
8794	chr9	112325453	112335428	PTBP3
1944	chr12	19030441	19031928	PLEKHA5
7803	chr6_GL000251v2_alt	1995392	1996866	LINC02569
2386	chr13	30455279	30465416	HMGB1
1163	chr10	62048333	62083814	ARID5B
7805	chr6_GL000251v2_alt	2199030	2202009	TUBB
6153	chr3	32549895	32576872	DYNC1LI1
1407	chr11	125085157	125089553	SLC37A2
5289	chr20	25236143	25242177	PYGB
1702	chr11	86243035	86254721	EED
3142	chr16	11344500	11370833	RMI2
4940	chr2	233423510	233436507	DGKD
2545	chr14	102073338	102091205	HSP90AA1
704	chr1	38010739	38032109	UTP11
2449	chr13	48943831	48952268	FNDC3A
4213	chr19	17399665	17408289	BST2
5502	chr21	29195311	29198157	LINC00189
9117	chr9	90996022	90999922	LOC100129316
7352	chr6	138092300	138106744	PERP
3083	chr15	89200736	89205982	RLBP1
2174	chr12	67064798	67070014	LOC102724421
2691	chr14	55189403	55192740	DLGAP5
7862	chr6_GL000253v2_alt	2578626	2582587	HLA-C
7715	chr6	7260740	7262707	SSR1
3965	chr17	81826298	81837690	MCRIP1
7188	chr5	95858195	95863548	LINC01554
4990	chr2	27360731	27365245	GTF3C2
1361	chr11	111749344	111767469	PPP2R1B
2645	chr14	42214093	42216079	LRFN5

8364	chr7	95263476	95266032	PON1
7224	chr6	100499406	100531665	SIM1
7426	chr6	154291687	154294229	IPCEF1
7144	chr5	80253632	80260919	SERINC5
9273	chrX	21838915	21841173	MBTPS2
9062	chr9	65675400	65681475	CBWD5
9243	chrX	152823426	152833825	CETN2
5870	chr3	125913850	125925711	FAM86JP
3617	chr17	34978254	34986853	LIG3
76	chr1	117776472	117778788	GDAP2
1029	chr10	125809624	125847741	BCCIP
4127	chr18	79964533	79973151	HSBP1L1
8625	chr8	47259648	47263707	SPIDR
6137	chr3	23912774	23923289	RPL15
3881	chr17	74763589	74777633	NAT9
1160	chr10	6162969	6185697	MIR3155B
5610	chr22	17618377	17631642	ATP6V1E1
7422	chr6	15247353	15262003	JARID2
1216	chr10	73522253	73526573	PPP3CB
2192	chr12	6963465	6985242	EMG1
767	chr1	51727811	51731717	OSBPL9
8246	chr7	56032765	56034589	PSPH
9235	chrX	13667435	13670347	TCEANC
1773	chr12	103940516	103958683	C12orf73
8207	chr7	42904049	42942016	PSMA2
712	chr1	40037760	40043292	CAP1
5226	chr2	88032059	88056978	KRCC1
3514	chr17	1514340	1522390	PITPNA-AS1
1762	chr12	100200473	100206531	ACTR6
8436	chr8	121206055	121207746	SNTB1
5710	chr22	40650143	40656724	MKL1
7680	chr6	53546226	53549294	GCLC
6799	chr5	139402434	139405182	SPATA24
457	chr1	211378071	211389838	LINC00467
6885	chr5	169582910	169591719	SPDL1
4158	chr19	11527238	11530387	ECSIT
8656	chr8	63165909	63180514	YTHDF3
8927	chr9	136101308	136118529	TMEM250
6454	chr4	1706898	1717168	SLBP
8374	chr7	98418148	98425879	BAIAP2L1
8325	chr7	77083870	77086023	SPDYE18
3953	chr17	8125385	8126474	HES7
9132	chr9	93985592	93988020	BARX1-DT
2677	chr14	52729054	52732067	STYX

8781	chr9	110237829	110258386	TXN
5102	chr2	54903187	54906755	RTN4
1466	chr11	27717886	27719331	BDNF
5362	chr20	43649888	43651942	MYBL2
4564	chr19_GL383574v1_alt	18164	40488	LSM14A
7157	chr5	83039427	83083436	SCARNA18
6409	chr4	145175636	145184543	OTUD4
1039	chr10	131966820	131980571	BNIP3
5235	chr2	95158896	95172735	ZNF2
9293	chrX	40580023	40584614	ATP6AP2
1575	chr11	63811352	63820897	SPINDOC
1226	chr10	74175069	74184066	ADK
1318	chr10	99778611	99787405	ABCC2
2534	chr14	100409525	100411896	WDR25
1945	chr12	19437735	19493749	AEBP2
5526	chr21	35044107	35049448	RUNX1
7902	chr6_GL000255v2_alt	2460660	2469963	HCG27
4939	chr2	233246814	233248860	ATG16L1
9022	chr9	37864285	37955631	SLC25A51
5810	chr3	108862898	108864716	TRAT1
6224	chr3	49005802	49010551	WDR6
4807	chr2	197483131	197508690	HSPD1
7578	chr6	31897830	31900366	C2
9125	chr9	92587873	92624108	CENPP
6485	chr4	1868239	1903104	NSD2
233	chr1	156211840	156231044	PMF1-BGLAP
9059	chr9	65145346	65150657	LOC101929583
4880	chr2	210546817	210551098	CPS1
3072	chr15	83199696	83208462	HDGFL3
4538	chr19	7459329	7463017	LOC100128573
1573	chr11	63665931	63672538	ATL3
4338	chr19	40662041	40667782	NUMBL
1038	chr10	131947378	131953038	BNIP3
3973	chr17	82097392	82109932	FASN
289	chr1	165827348	165850532	UCK2
9094	chr9	76379179	76396817	RFK
3603	chr17	31038513	31039969	LOC646030
7989	chr7	113108177	113119881	SMIM30
8028	chr7	130193273	130206547	TMEM209
4193	chr19	14517591	14520099	DNAJB1
1526	chr11	47980496	47986186	PTPRJ
5180	chr2	73232756	73256948	CCT7
4867	chr2	207393695	207397190	MIR1302-4
7434	chr6	157762281	157764532	SNX9

3895	chr17	75387372	75397494	GRB2
9008	chr9	35709301	35782972	GBA2
2187	chr12	6936565	6941397	C12orf57
8634	chr8	51186407	51188551	PXDNL
2402	chr13	36288614	36348811	CCDC169
879	chr1	85646743	85710669	ZNHIT6
4236	chr19	18830892	18845676	UPF1
4814	chr2	199921830	199939039	C2orf69
3086	chr15	89573816	89579239	TICRR
2267	chr12	93227541	93231473	LINC02412
1172	chr10	63706282	63708529	JMJD1C
3737	chr17	47501415	47504241	MRPL45P2
4202	chr19	16110652	16113466	RAB8A
8112	chr7	1564896	1570063	PSMG3
6190	chr3	4492535	4494277	ITPR1
7147	chr5	81223603	81227469	CKMT2
426	chr1	205103234	205123394	RBBP5
4865	chr2	207309026	207313128	MIR1302-4
660	chr1	31430851	31468809	SERINC2
7203	chr5_GL339449v2_alt	114135	116683	CCDC125
8912	chr9	132874331	132881646	SPACA9
3719	chr17	46264025	46267966	LRRC37A
271	chr1	161398218	161400876	CFAP126
3108	chr15	96273111	96276039	NR2F2
5976	chr3	15204358	15221387	CAPN7
9297	chrX	44873431	44892795	KDM6A
8596	chr8	37736364	37753610	LOC728024
3447	chr16	83953569	83957895	OSGIN1
5531	chr21	35524112	35526892	LOC100506403
42	chr1	110949294	110966056	LRIF1
6043	chr3	179652475	179655921	USP13
8441	chr8	12184035	12196595	FAM86B1
7427	chr6	154729528	154799260	SCAF8
9006	chr9	35645535	35648104	LOC101926948
8950	chr9	137585287	137592609	ZMYND19
4244	chr19	1940918	1943774	CSNK1G2
1792	chr12	107728202	107760729	LOC101929162
4386	chr19	44586933	44589806	IGSF23
6533	chr4	38965058	38968177	TMEM156
3519	chr17	15965865	15967353	ADORA2B
6968	chr5	27241055	27241667	CDH9
4072	chr18	3651458	3655928	DLGAP1-AS2
3785	chr17	55432299	55435372	MMD
8934	chr9	136806895	136870316	AJM1

9157	chr9	99809454	99822036	LOC101928438
5029	chr2	3571915	3590446	RPS7
6197	chr3	46681923	46693569	ALS2CL
5985	chr3	154235747	154325457	DHX36
3546	chr17	19111328	19113728	SNORD3D
3660	chr17	41792011	41795969	JUP
2162	chr12	65535891	65538452	LOC105369187
7234	chr6	106096892	106101959	PRDM1
6589	chr4	56908113	56915454	REST
1481	chr11	33071820	33074366	LINC00294
4836	chr2	201639913	201644868	TMEM237
9363	chrX	72180101	72194651	PIN4
2981	chr15	64681877	64704381	OAZ2
5309	chr20	33809141	33846113	CHMP4B
4081	chr18	46320647	46322800	RNF165
8980	chr9	31270535	31276748	LINC01243
364	chr1	183604665	183634570	ARPC5
3084	chr15	89242755	89245559	FANCI
1659	chr11	70268780	70290175	MIR548K
2094	chr12	54015510	54034871	HOXC6
7184	chr5	95729241	95744916	SPATA9
5969	chr3	150553817	150562091	EIF2A
8841	chr9	125643375	125650687	MAPKAP1
5331	chr20	36079457	36081526	EPB41L1
4266	chr19	29599270	29614865	POP4
3087	chr15	89621860	89626231	KIF7
4834	chr2	201287060	201293666	CASP8
5913	chr3	134478894	134494119	CEP63
348	chr1	179837929	179876914	TOR1AIP2
7399	chr6	149588341	149609713	RPS18P9
6245	chr3	50297651	50301069	NAA80
1005	chr10	119540843	119544447	RGS10
5089	chr2	48113392	48115402	FOXN2
4086	chr18	48945448	48954294	SMAD7
1870	chr12	121625652	121629403	ORAI1
52	chr1	112901261	112905763	AKR7A2P1
681	chr1	34754242	34756793	GJB5
7785	chr6	99452273	99516910	USP45
761	chr1	50420191	50424819	DMRTA2
7127	chr5	77029634	77042314	AGGF1
7089	chr5	69166067	69176950	CCNB1
403	chr1	201500414	201506818	CSRP1
7317	chr6	13276698	13331064	LOC100130357
8782	chr9	111478267	111487102	ECPAS

2909	chr15	45161436	45167973	SHF
8224	chr7	4680286	4688716	FOXK1
1486	chr11	33710482	33775016	CD59
5966	chr3	150387069	150392473	TSC22D2
1430	chr11	13213758	13217944	ARNTL
7358	chr6	1388043	1399284	MIR6720
7155	chr5	82405009	82407073	ATP6AP1L
5775	chr22_KI270734v1_random	129096	131042	DGCR6
9173	chrX	102768525	102777275	LINC00630
4562	chr19	9824504	9829924	UBL5
7608	chr6	36440170	36458523	PXT1
2918	chr15	49168917	49187419	GALK2
7546	chr6	28398667	28401665	ZSCAN12
6207	chr3	47509778	47515961	ELP6
5118	chr2	61045257	61047920	KIAA1841
3171	chr16	15671555	15675206	NDE1
5638	chr22	26505865	26517000	TFIP11
2728	chr14	67596601	67602910	PIGH
7609	chr6	36538144	36548886	STK38
1522	chr11	47636279	47644451	MTCH2
2796	chr14	85087487	85089574	SNORD3P3
4239	chr19	19031062	19035106	SUGP2
7192	chr5	96093338	96095853	MIR583
6134	chr3	20183242	20188869	SGO1
820	chr1	65062810	65069763	JAK1
800	chr1	58881584	58884879	LINC01135
1340	chr11	105150837	105154305	CARD18
8327	chr7	77119280	77123423	FAM185BP
9366	chrX	73561230	73565148	CHIC1
3748	chr17	48610101	48611680	HOXB7
6608	chr4	73225362	73276514	ANKRD17
1989	chr12	3167989	3173791	TSPAN9
5691	chr22	37903803	37913201	MICALL1
5872	chr3	126206934	126210645	ALDH1L1
6007	chr3	160395228	160429656	MIR16-2
3263	chr16	30231819	30249368	NPIPB12
689	chr1	3541496	3544363	MIR551A
4212	chr19	17335517	17339778	GTPBP3
7520	chr6	27014571	27021637	LOC100270746
9143	chr9	95505547	95507542	PTCH1
5468	chr20	62738879	62742608	NTSR1
6023	chr3	169771009	169793114	MYNN
6760	chr5	132864964	132880577	LEAP2
919	chr1	94243897	94248713	ARHGAP29

989	chr10	113852262	113872123	NHLRC2
4153	chr19	11140944	11144361	SPC24
1370	chr11	116761441	116776559	BUD13
1535	chr11	58568654	58639060	CNTF
5030	chr2	3604181	3607931	COLEC11
3890	chr17	75169727	75184838	SUMO2
2243	chr12	86660075	86661865	MGAT4C
71	chr1	116376776	116419434	ATP1A1
3769	chr17	50632737	50674939	ABCC3
238	chr1	156569875	156572284	IQGAP3
7386	chr6	146544336	146557851	RAB32
5525	chr21	35011389	35016575	RUNX1-IT1
3976	chr17	8221376	8222492	LINC00324
8539	chr8	18078968	18085820	ASAH1
5472	chr20	62872323	62880288	SNORA117
8138	chr7	22577171	22579698	LOC100506178
5432	chr20	53080536	53083653	TSHZ2
3365	chr16	622723	630169	WFIKKN1
453	chr1	210327922	210329883	HHAT
690	chr1	35634541	35642803	PSMB2
8456	chr8	125309254	125326034	TRIB1
6833	chr5	143402278	143413439	NR3C1
388	chr1	19595394	19633316	RPS14P3
2592	chr14	23555401	23570070	LOC102724814
8111	chr7	155949758	155983761	LOC389602
603	chr1	244433110	244453349	ADSS
1714	chr11	8904667	8907531	ST5
1012	chr10	12161735	12171284	CDC123
8639	chr8	53951930	54024881	TCEA1
7129	chr5	78261614	78295798	AP3B1
8830	chr9	124305414	124308520	NEK6
6532	chr4	38851397	38859227	TLR6
7638	chr6	42223526	42244007	MRPS10
2721	chr14	65355399	65358631	MIR4708
5109	chr2	55519807	55529151	CFAP36
7186	chr5	95821448	95825227	GLRX
4773	chr2	181874828	181878162	SSFA2
6936	chr5	179668706	179670664	CBY3
2397	chr13	33816453	33868875	RFC3
5512	chr21	33323387	33355315	IFNAR1
1836	chr12	11647831	11654178	ETV6
3290	chr16	31178066	31185241	FUS
5325	chr20	35698816	35706027	NFS1
2373	chr13	25318203	25350410	NUP58

6990	chr5	36135895	36151903	MIR580
2074	chr12	52872271	52875131	KRT78
8442	chr8	123015596	123043276	DERL1
5767	chr22	49970213	49972357	MIR6821
3666	chr17	42534710	42540392	NAGLU
6407	chr4	143512246	143529384	SMARCA5-AS1
1977	chr12	28778941	28781357	FAR2
775	chr1	52331773	52336281	CC2D1B
8081	chr7	150366747	150383173	REPIN1
1629	chr11	66612339	66654971	RBM4
5001	chr2	27879094	27893658	BABAM2
6603	chr4	6906972	6911331	TBC1D14
7198	chr5	96932872	96939455	LNPEP
6375	chr4	128081329	128084170	LARP1B
2536	chr14	100673143	100694756	LINC00523
3219	chr16	24728526	24834882	TNRC6A
617	chr1	248835730	248860956	ZNF672
4272	chr19	32690752	32710885	NUDT19
3049	chr15	75841987	75851962	UBE2Q2
1503	chr11	43642710	43653280	HSD17B12
1007	chr10	119650446	119661065	BAG3
5466	chr20	62640651	62645779	SLCO4A1
2203	chr12	7187923	7196565	PEX5
2663	chr14	49999357	50000350	LINC01588
458	chr1	211416349	211427557	LINC00467
3423	chr16	73039134	73056552	ZFHX3
5474	chr20	63270983	63276029	ARFGAP1
8277	chr7	66844124	66846665	GTF2IRD1P1
2637	chr14	38142833	38144961	SSTR1
1351	chr11	108465959	108468837	C11orf65
6149	chr3	31211794	31214174	MIR466
8541	chr8	19138262	19142158	LOC100128993
5782	chr2_GL383521v1_alt	5257	6708	LOC100288911
7163	chr5	87119465	87122770	LOC101929380
8288	chr7	72942731	72957596	NSUN5P2
1239	chr10	78023994	78030527	POLR3A
2621	chr14	34058992	34061520	EGLN3
5454	chr20	5918463	5920623	CHGB
1542	chr11	59753994	59815988	MRPL16
4203	chr19	16184325	16187303	FAM32A
9330	chrX	52993284	52996795	FAM156A
818	chr1	64964973	64967146	JAK1
3873	chr17	7305843	7318474	GPS2
8956	chr9	18489391	18492088	ADAMTSL1

6884	chr5	167642873	167644626	TENM2
3536	chr17	18241160	18259771	FLII
8384	chr7	99904709	99927905	TRIM4
9136	chr9	94374524	94389748	MFSD14B
8427	chr8	117529195	117535546	MED30
1339	chr11	105131916	105134608	CARD18
8095	chr7	151795670	151797697	LOC644090
4501	chr19	5689194	5700407	RPL36
2476	chr13	68887560	68890765	LINC00550
7819	chr6_GL000251v2_alt	385671	415494	HCG14
2560	chr14	104857507	104867088	CEP170B
5771	chr22	50543633	50547661	KLHDC7B
9058	chr9	64640309	64646059	LOC101929583
2808	chr14	92510749	92514994	RIN3
2705	chr14	60246063	60311989	PPM1A
1020	chr10	12346588	12351392	CAMK1D
1261	chr10	87634061	87636848	PAPSS2
8270	chr7	66491462	66494523	GS1-124K5.4
3903	chr17	75872555	75879132	TRIM47
5733	chr22	43109242	43112336	BIK
7299	chr6	125266664	125304270	HDDC2
5324	chr20	35633527	35665826	CPNE1
5613	chr22	18148776	18152300	USP18
2255	chr12	89591333	89607119	ATP2B1
4658	chr2	127438004	127528759	IWS1
563	chr1	234356256	234360641	LOC101927765
4733	chr2	169583246	169587322	PPIG
6499	chr4	20698933	20715191	PACRGL
2152	chr12	64146527	64182939	C12orf66
8362	chr7	94653218	94658653	SGCE
8938	chr9	136990967	136994721	PAXX
552	chr1	231228723	231253031	GNPAT
6702	chr5	108800735	108834382	FER
1933	chr12	14795607	14811658	C12orf60
365	chr1	184049579	184082927	TSEN15
9035	chr9	41353641	41359896	LOC107984035
5121	chr2	61474335	61489363	USP34
1428	chr11	130306335	130315764	ZBTB44
2575	chr14	21307570	21310748	RPGRIP1
2505	chr13	79373649	79429929	RBM26
1077	chr10	2312255	2317753	LINC00701
343	chr1	179025383	179043092	FAM20B
6067	chr3	185798009	185822377	IGF2BP2
512	chr1	225392455	225400227	LBR

7814	chr6_GL000251v2_alt	3282640	3285283	LSM2	
5763	chr22	49770148	49774722	BRD1	
3011	chr15	69451448	69460623	RPLP1	
1286	chr10	92288916	92305495		5-Mar
4094	chr18	51185871	51198951	MEX3C	
9190	chrX	116270853	116273994	AGTR2	
1730	chr11	93781515	93810379	MED17	
2983	chr15	65168118	65185599	CLPX	
3166	chr16	15061849	15097768	RRN3	
5546	chr21	37560939	37566038	DYRK1A	
9220	chrX	130158079	130168554	AIFM1	
2692	chr14	55365902	55378999	ATG14	
825	chr1	6598728	6605230	KLHL21	
5760	chr22	46759469	46766617	TBC1D22A	
6476	chr4	184379770	184385406	LINC02362	
1227	chr10	74825149	74828806	KAT6B	
3589	chr17	29292153	29296312	NUFIP2	
7404	chr6	149748466	149758484	PCMT1	
10	chr1	103522913	103566520	AMY2B	
1283	chr10	91922252	91959165	BTAF1	
4377	chr19	43751371	43758637	SMG9	
3312	chr16	4269119	4279492	TFAP4	
4265	chr19	2944134	2947171	ZNF77	
7679	chr6	53324200	53350192	RPS16P5	
7978	chr7	107399434	107422324	GPR22	
738	chr1	44720272	44721782	RNU5F-1	
929	chr1	96721179	96726855	PTBP2	
4774	chr2	181891770	181929659	SSFA2	
4561	chr19	982296	985676	WDR18	
1456	chr11	19977884	19979879	NAV2	
6607	chr4	72207461	72211429	NPFFR2	
4434	chr19	47605165	47615298	BICRA	
8424	chr8	116679814	116757696	EIF3H	
6912	chr5	173595395	173618622	BOD1	
4529	chr19	6529268	6533541	TNFSF9	
8623	chr8	43138986	43154763	HGSNAT	
6493	chr4	188940647	188942926	LINC02508	
6327	chr4	106308021	106321253	AIMP1	
4761	chr2	177263901	177267054	NFE2L2	
7444	chr6	158834868	158836226	EZR	
1911	chr12	12888930	12907970	GPRC5A	
8697	chr8	81684685	81748036	ZFAND1	
9257	chrX	155025725	155030780	FUNDC2	
2634	chr14	36662141	36664361	PAX9	

1568	chr11	62840554	62843646	WDR74
6505	chr4	2534780	2547267	RNF4
4536	chr19	7096110	7101412	ZNF557
5674	chr22	32531102	32536958	FBXO7
2264	chr12	9281764	9305035	LOC642846
1003	chr10	119175647	119179765	PRDX3
3451	chr16	84998734	85013608	ZDHHC7
3342	chr16	53700413	53708509	FTO
2668	chr14	50895681	50896878	ABHD12B
5899	chr3	130885595	130910657	ATP2C1
792	chr1	58529835	58547882	OMA1
3217	chr16	23632968	23644963	PALB2
2055	chr12	50399585	50418144	LARP4
207	chr1	15416351	15420697	EFHD2
3412	chr16	70791249	70806938	VAC14
2925	chr15	50906551	50913088	AP4E1
4743	chr2	172247009	172255262	DLX2-DT
1619	chr11	65973207	65975427	SART1
6106	chr3	195320866	195354318	XXYLT1
6642	chr4	81163639	81167374	LOC101928942
9015	chr9	37421079	37425115	GRHPR
3094	chr15	90529692	90532662	CRTC3
4071	chr18	3620523	3626865	DLGAP1-AS2
8564	chr8	25174626	25176504	DOCK5
5567	chr21	42954184	42956985	MIR5692B
72	chr1	116561040	116571913	CD58
518	chr1	225777276	225786056	SRP9
3790	chr17	56876366	56917312	MIR3614
521	chr1	225911844	225926011	MIR6741
4826	chr2	200809882	200832879	BZW1
6870	chr5	159239349	159241717	LINC01932
6447	chr4	168629537	168636114	PALLD
4630	chr2	112252697	112256178	ZC3H8
5033	chr2	3658562	3663335	ALLC
2377	chr13	27227190	27254714	LINC00412
1562	chr11	62650299	62654482	INTS5
7160	chr5	8455375	8469623	MIR4458
1242	chr10	78971441	78975195	ZMIZ1-AS1
5747	chr22	45308895	45310320	FAM118A
5889	chr3	128871236	128901197	ACAD9
7398	chr6	149565568	149569212	GINM1
7841	chr6_GL000252v2_alt	3081434	3084258	C6orf48
5928	chr3	140939808	140976100	SLC25A36
3291	chr16	3130155	3153880	CASP16P

1202	chr10	71954646	71957528	CHST3
4077	chr18	40910613	40913631	KC6
4362	chr19	42873159	42879772	PSG1
8137	chr7	22564439	22565368	LOC100506178
8824	chr9	121150954	121155426	CNTRL
5330	chr20	35947378	35958400	SCAND1
5198	chr2	80488265	80490738	CTNNA2
5687	chr22	37674843	37677930	LGALS1
2627	chr14	35044826	35054647	FAM177A1
5381	chr20	45790247	45798310	DNTTIP1
3571	chr17	27242505	27246339	MIR4522
3275	chr16	30696337	30705992	SRCAP
7477	chr6	18226358	18266409	DEK
9148	chr9	96981466	97013656	MFSD14C
6710	chr5	111092077	111134222	WDR36
1532	chr11	57702422	57716361	MED19
7643	chr6	42966690	42979918	PEX6
2049	chr12	50020123	50026588	RACGAP1
1306	chr10	97398226	97402685	RRP12
8632	chr8	48788212	48790325	EFCAB1
4327	chr19	39835477	39847443	FBL
1335	chr11	10486543	10497111	MIR4485
3391	chr16	68635946	68637325	CDH3
1614	chr11	65848824	65870099	MUS81
6846	chr5	148983279	148985672	MIR584
7077	chr5	65481913	65484541	ADAMTS6
4245	chr19	19514635	19519190	NDUFA13
7596	chr6	34717966	34720892	LOC101929243
7678	chr6	52573368	52578889	TRAM2
8611	chr8	41489266	41497024	GOLGA7
6465	chr4	176838304	176840268	LINC02509
6778	chr5	135139397	135143434	C5orf66-AS1
7347	chr6	136790067	136792157	MAP3K5
3122	chr15	99716811	99724252	LYSMD4
7120	chr5	75234244	75238288	ANKRD31
2686	chr14	54467631	54490101	GMFB
9359	chrX	71282453	71290481	NONO
605	chr1	244833719	244851185	COX20
4481	chr19	54199307	54206122	RPS9
1031	chr10	126197932	126200676	ADAM12
7038	chr5	5418920	5446633	ICE1
7067	chr5	62304987	62345857	KIF2A
7613	chr6	36982593	36989841	MTCH1
4514	chr19	58324326	58331382	LOC105372480

2172	chr12	66112681	66134677	LLPH-DT
7735	chr6	7696999	7699474	BMP6
7075	chr5	65033434	65037227	CWC27
2305	chr13	101454416	101455552	ITGBL1
8990	chr9	33451958	33477560	MIR6851
7876	chr6_GL000254v2_alt	2063132	2071780	TUBB
3278	chr16	30868470	30877475	MIR4519
1331	chr11	102995345	102998128	MMP13
7268	chr6	112064820	112087360	FAM229B
145	chr1	148520274	148524278	PPIAL4G
931	chr1	98322556	98325669	LINC01776
2430	chr13	44559571	44580110	TSC22D1-AS1
5350	chr20	38470692	38478747	RALGAPB
6461	chr4	176269879	176272072	ASB5
2252	chr12	89345856	89373407	DUSP6
7822	chr6_GL000251v2_alt	4677632	4689489	RPS18
1066	chr10	18649008	18653271	NSUN6
1548	chr11	61359687	61365559	CYB561A3
4159	chr19	11531155	11533246	ECSIT
6861	chr5	154418595	154425669	SAP30L-AS1
5146	chr2	65362653	65368030	SPRED2
5412	chr20	50437587	50442697	PTPN1
2707	chr14	61177499	61179912	TMEM30B
8807	chr9	114731975	114735972	TEX48
5348	chr20	3819396	3822532	AP5S1
3795	chr17	57977582	58010216	VEZF1
1936	chr12	15879680	15916839	DERA
1841	chr12	116909830	116923817	FBXW8
965	chr10	104188045	104205278	MIR609
889	chr1	8889816	8890937	ENO1-AS1
2056	chr12	50504308	50512349	DIP2B
3159	chr16	1440080	1481060	CLCN7
5096	chr2	53818313	53830829	MIR3682
7190	chr5	96066543	96069659	MIR583
6283	chr3	81750960	81762909	GBE1
5942	chr3	142975569	142977360	PAQR9-AS1
5916	chr3	136137310	136198936	MSL2
3482	chr16	89490818	89493594	ANKRD11
8217	chr7	44637034	44641480	OGDH
7521	chr6	27091290	27092887	HIST1H2BJ
5811	chr3	110900449	110901417	NECTIN3-AS1
7864	chr6_GL000253v2_alt	2962138	2976646	GPANK1
6678	chr4	99061782	99090137	ADH5
8468	chr8	12743710	12755085	LONRF1

4670	chr2	131460353	131464043	TUBA3D
2804	chr14	90845943	90852958	TTC7B
6057	chr3	184184714	184195018	ABCF3
8130	chr7	18865745	18868803	TWIST1
8586	chr8	30056369	30060960	SARAF
8504	chr8	143858186	143880130	EPPK1
672	chr1	32538000	32546630	ZBTB8A
3118	chr15	99129352	99132387	SYNM
2616	chr14	31560168	31569268	NUBPL
8154	chr7	24980672	24983292	OSBPL3
3721	chr17	46355984	46363125	ARL17B
1539	chr11	59559311	59562582	MIR3162
619	chr1	248904248	248910074	PGBD2
5263	chr20	10402979	10437024	MKKS
6582	chr4	55378474	55379603	SRD5A3-AS1
7617	chr6	37135641	37141226	PIM1
4626	chr2	111532455	111533975	MIR4435-2HG
171	chr1	150925821	150931987	SETDB1
902	chr1	92960769	92964040	FAM69A
2333	chr13	110915633	110920188	ANKRD10
7993	chr7	116314041	116318083	LOC102724434
8001	chr7	116771986	116795686	CAPZA2
9033	chr9	40038978	40113116	ANKRD20A2
1262	chr10	87741647	87748576	ATAD1
5269	chr20	11847301	11853906	LINC00687
3021	chr15	71847136	71849863	NR2E3
6726	chr5	116545270	116548058	SEMA6A-AS2
7861	chr6_GL000253v2_alt	2506317	2511543	MICB
5579	chr21	43787313	43791528	RRP1
7180	chr5	94610743	94625071	KIAA0825
3403	chr16	69708234	69714439	SNORD13H
6781	chr5	13565993	13571399	DNAH5
9379	chrX	7920433	7930359	PNPLA4
6725	chr5	116051560	116056860	ARL14EPL
8490	chr8	141153144	141155895	DENND3
6199	chr3	4710433	4713918	EGOT
319	chr1	171736508	171747516	VAMP4
5552	chr21	39379440	39383129	WRB
9321	chrX	48991712	49003138	GRIPAP1
389	chr1	19642340	19644105	NBL1
8531	chr8	17220310	17283692	CNOT7
884	chr1	8810546	8819424	RERE
8890	chr9	129631473	129642378	ASB6
2977	chr15	64355379	64357474	CSNK1G1

2652	chr14	47269114	47272469	MDGA2
8093	chr7	151754426	151755359	PRKAG2
6329	chr4	107989047	108009448	HADH
6255	chr3	51978878	51996683	ACY1
539	chr1	228450089	228463385	HIST3H2A
901	chr1	92842007	92844603	SNORA66
4920	chr2	227797879	227819767	CCL20
4654	chr2	127106066	127108425	BIN1
2949	chr15	57705122	57722598	POLR2M
7111	chr5	73496099	73512113	BTF3
9291	chrX	40106938	40110749	BCOR
5672	chr22	32410678	32412407	RTCB
1711	chr11	88408313	88408936	CTSC
8536	chr8	1754699	1757142	LOC101927752
2371	chr13	24911849	24924831	CENPJ
8197	chr7	38176766	38181994	STARD3NL
5879	chr3	126982077	126984446	PLXNA1
3111	chr15	97502890	97505658	LINC02254
2797	chr14	85415611	85417442	LINC00911
8615	chr8	41994880	42054456	LOC105379393
8565	chr8	25182735	25195309	DOCK5
7869	chr6_GL000254v2_alt	1052543	1055164	HLA-V
8645	chr8	58409970	58447138	UBXN2B
3798	chr17	58399387	58404068	RNF43
3128	chr15_KI270851v1_alt	137050	145905	HERC2P2
7010	chr5	40751654	40799691	TTC33
195	chr1	153528337	153536171	S100A6
523	chr1	226082026	226085086	LINC01703
2206	chr12	73862928	73864758	LOC100507377
3161	chr16	14745039	14751813	NPIPA3
92	chr1	120911430	120912998	LINC00623
8721	chr8	93990685	93992812	PDP1
1838	chr12	116551140	116553559	MAP1LC3B2
3898	chr17	7556900	7591698	SNORA48
7813	chr6_GL000251v2_alt	3176146	3181486	MIR4646
6657	chr4	84965827	84970088	WDFY3-AS2
5839	chr3	119467239	119472780	POGLUT1
2016	chr12	45990362	45993691	SCAF11
2968	chr15	63475525	63477445	USP3
6081	chr3	189823977	189827327	MIR944
4963	chr2	240255303	240256698	OTOS
6301	chr3	9889140	9899887	JAGN1
9299	chrX	45477816	45481830	LINC01204
3486	chr16	89716879	89725590	VPS9D1

7669	chr6	48824799	48829213	MUT	
5576	chr21	43710951	43713212	PDXK	
3490	chr16	9041858	9071157	C16orf72	
706	chr1	3891563	3901725	C1orf174	
2724	chr14	67362653	67392267	EIF2S1	
3661	chr17	42014323	42018900	DNAJC7	
3225	chr16	2650371	2705892	KCTD5	
4264	chr19	29200424	29214375	UQCRFS1	
470	chr1	214273737	214283546	SMYD2	
6271	chr3	57690305	57694443	DENND6A	
7871	chr6_GL000254v2_alt	160274	164064	HCG14	
6694	chr5	10441045	10442369	ROPN1L-AS1	
3089	chr15	89869272	89895077	MIR5009	
134	chr1	147081831	147086466	RNVU1-8	
4209	chr19	17074479	17076145	HAUS8	
4225	chr19	18290151	18295368	MIR3188	
8234	chr7	5420547	5434305	LOC100129484	
962	chr10	103969654	103974965	SLK	
572	chr1	234996632	234998706	LOC101927851	
8311	chr7	7565561	7603268	MIOS	
8225	chr7	46931548	46934459	LOC730338	
538	chr1	228388578	228408788	MIR6742	
6932	chr5	179545356	179551902	RUFY1	
8020	chr7	128737551	128767131	CALU	
5918	chr3	136326134	136334276	PCCB	
6269	chr3	57565060	57581962	PDE12	
6204	chr3	47408305	47432763	PTPN23	
8159	chr7	26861886	26866431	SKAP2	
3565	chr17	2397699	2400359	MNT	
3051	chr15	76333451	76337710	ISL2	
3821	chr17	62790276	62792641		10-Mar
1990	chr12	31730883	31736014	AMN1	
3284	chr16	3103828	3108377	ZNF205	
514	chr1	225444288	225445868	LBR	
7382	chr6	145728083	145738834	LOC100507557	
5817	chr3	112001396	112005280	TAGLN3	
7424	chr6	152967862	153014756	FBXO5	
4895	chr2	218568844	218588211	CNOT9	
7353	chr6	138159310	138163678	ARFGEF3	
1196	chr10	71264339	71270785	UNC5B-AS1	
4052	chr18	2514595	2516641	METTL4	
6456	chr4	173369746	173372719	SAP30	
8232	chr7	516866	526595	HRAT92	
8896	chr9	130038241	130069831	GPR107	

8203	chr7	41106270	41109420	LINC01449
3095	chr15	90659270	90666823	LINC01585
2890	chr15	42541758	42554766	LRRC57
1089	chr10	27241681	27246306	ACBD5
7763	chr6	87150977	87182346	ZNF292
3222	chr16	2551911	2572398	PDPK1
9152	chr9	97909468	97923725	TRMO
6650	chr4	83112400	83116074	PLAC8
2713	chr14	63720105	63729644	SGPP1
7136	chr5	78984980	78987206	ARSB
7237	chr6	106524488	106529259	CRYBG1
8647	chr8	58656458	58663258	NSMAF
342	chr1	178723768	178755361	RALGPS2
1723	chr11	9310226	9316929	TMEM41B
1102	chr10	31919349	31932367	ARHGAP12
5282	chr20	19974051	19981649	NAA20
7831	chr6_GL000252v2_alt	2023805	2026781	HCG20
8098	chr7	152023871	152050309	GALNT11
1761	chr11_KI270832v1_alt	29388	34422	RNH1
2602	chr14	26840272	26843468	LINC02294
8300	chr7	74171764	74201978	MIR590
2004	chr12	42228795	42238411	YAF2
6798	chr5	139389653	139393724	MZB1
6214	chr3	48434364	48443814	CCDC51
1183	chr10	68632141	68633110	TET1
3794	chr17	57847251	57853842	MRPS23
8046	chr7	135157338	135173519	CYREN
6383	chr4	1336947	1345456	UVSSA
2548	chr14	102527263	102529080	MIR4309
7854	chr6_GL000253v2_alt	2050696	2054952	FLOT1
4879	chr2	210445838	210477866	LANCL1
6530	chr4	38317576	38319832	LINC02513
5577	chr21	43718393	43743978	PDXK
8283	chr7	6819076	6829537	CCZ1B
1083	chr10	25042479	25061937	ENKUR
2530	chr14	100211234	100215720	YY1
1025	chr10	124914716	124920136	ZRANB1
1672	chr11	73778148	73781221	MRPL48
4028	chr18	11906558	11910522	MPPE1
3302	chr16	3441644	3445474	ZNF597
8115	chr7	157196999	157224182	UBE3C
3552	chr17	19507147	19508638	SLC47A1
3432	chr16	74698976	74702302	MLKL
2361	chr13	20562774	20582638	IFT88

3949	chr17	80854983	80859937	LOC101928855
8847	chr9	126858963	126861583	ZBTB34
4877	chr2	210001252	210064237	LOC101928020
5596	chr21	6139397	6151365	SIK1
7104	chr5	72308241	72333263	PTCD2
6822	chr5	141919265	141925075	DELE1
1107	chr10	32978730	32984653	ITGB1
5024	chr2	33476045	33477806	RASGRP3
6645	chr4	82419019	82442697	ENOPH1
7531	chr6	27820566	27826508	HIST1H4J
6479	chr4	184643434	184654316	CASP3
3832	chr17	64518614	64554753	DDX5
6096	chr3	194070541	194073422	LINC02028
8936	chr9	136900320	136911374	MIR4479
3040	chr15	74935643	74939746	COX5A
1719	chr11	925510	936018	AP2A2
3459	chr16	86951264	86953904	LINC02181
5521	chr21	34769869	34773360	LINC01426
4383	chr19	44211545	44229436	ZNF227
8821	chr9	121072799	121078006	C5
2142	chr12	62465832	62474252	MON2
976	chr10	110277845	110306709	SMNDC1
3207	chr16	2222367	2238078	DNASE1L2
3684	chr17	43289206	43293518	RNU2-1
7411	chr6	150269083	150273807	IYD
8931	chr9	136558974	136570846	NALT1
4717	chr2	159601786	159618882	LOC643072
5181	chr2	73735315	73738728	TPRKB
8774	chr9	108442666	108444500	ACTL7B
3351	chr16	56930070	56951595	HERPUD1
6859	chr5	154028478	154062582	MFAP3
9259	chrX	15672157	15692141	CA5BP1
897	chr1	9162333	9164613	MIR34A
468	chr1	212856161	212866212	FLVCR1
4539	chr19	7486176	7490031	PEX11G
296	chr1	16737984	16741784	RNU1-2
2613	chr14	31228163	31231165	HECTD1
2835	chr15	22493335	22503362	HERC2P2
6124	chr3	197455922	197458332	LINC02012
4613	chr2	108446689	108450629	GCC2
1131	chr10	4352589	4355932	LINC00703
6345	chr4	117398908	117401876	LINC01378
560	chr1	233327549	233332318	MAP3K21
6421	chr4	152671941	152682072	TMEM154

481	chr1	2189235	2191287	FAAP20
4070	chr18	36185918	36235198	LOC101927809
2409	chr13	40114146	40117219	LINC00332
7320	chr6	133243784	133367418	EYA4
4979	chr2	25870087	25880084	ASXL2
1399	chr11	123110530	123112356	HSPA8
3106	chr15	92882670	93031693	MIR3175
2482	chr13	73215343	73217586	KLF5
8863	chr9	128146635	128152706	LCN2
209	chr1	154207638	154253481	UBAP2L
5214	chr2	86079228	86111988	POLR1A
6487	chr4	186919109	186921725	FAT1
2017	chr12	46188988	46275978	SLC38A1
7517	chr6	2687867	2689871	LINC01600
8315	chr7	76290792	76293076	HSPB1
8326	chr7	77102361	77106559	FAM185BP
1416	chr11	12672711	12749553	TEAD1
7892	chr6_GL000255v2_alt	160152	163375	HCG14
6148	chr3	30606548	30691743	TGFBR2
237	chr1	156469595	156504814	MEF2D
7834	chr6_GL000252v2_alt	2456111	2458293	HCG27
2307	chr13	101648910	101651582	ITGBL1
3295	chr16	3183480	3184951	OR1F1
3842	chr17	6633992	6647104	TXNDC17
3640	chr17	39198559	39206644	RPL19
566	chr1	234598119	234615533	IRF2BP2
1294	chr10	94308856	94364632	NOC3L
6211	chr3	48168607	48182293	CDC25A
3690	chr17	43543533	43549034	ETV4
4075	chr18	40809800	40812495	KC6
3598	chr17	30781388	30792125	CRLF3
6503	chr4	2456713	2483843	RNF4
215	chr1	155001646	155008929	ZBTB7B
8126	chr7	17597711	17602030	LOC101927630
67	chr1	115208897	115228318	NGF
7563	chr6	30555317	30560516	GNL1
9010	chr9	36188001	36196287	CLTA
1166	chr10	6274168	6276899	LOC399715
1082	chr10	24847026	24848985	PRTFDC1
246	chr1	15798602	15803389	UQCRHL
8307	chr7	74869911	74893277	STAG3L2
8579	chr8	28759315	28764541	EXTL3
4199	chr19	15825188	15840096	UCA1
533	chr1	227562503	227565232	ZNF678

6233	chr3	49672521	49717965	RNF123
7721	chr6	73446764	73453187	CGAS
5727	chr22	42675160	42681635	CYB5R3
8961	chr9	201081	203980	DOCK8
8170	chr7	2838391	2844392	GNA12
4859	chr2	206271910	206300150	ZDBF2
5182	chr2	73827912	73846583	STAMBP
3300	chr16	326773	328743	AXIN1
1024	chr10	124800519	124811020	ABRAXAS2
8646	chr8	58553488	58598698	SDCBP
4934	chr2	231700377	231715705	PTMA
6364	chr4	124205410	124208723	LINC02516
7720	chr6	73388360	73390382	DDX43
3266	chr16	30399813	30401463	ZNF48
9126	chr9	92659699	92677245	IPPK
6241	chr3	50087662	50126307	RBM5-AS1
1270	chr10	89326107	89332006	IFIT3
6992	chr5	36606241	36622708	SLC1A3
8312	chr7	75842191	75844415	CCL24
8944	chr9	137275639	137295704	TOR4A
5862	chr3	124728945	124743569	MIR544B
140	chr1	148333557	148336181	MIR5087
2958	chr15	60367535	60394220	ANXA2
6396	chr4	139423347	139428268	RAB33B
5317	chr20	35139233	35149378	EDEM2
6287	chr3	87225507	87234719	CHMP2B
4114	chr18	665870	674317	TYMSOS
7811	chr6_GL000251v2_alt	3015657	3023559	DDX39B-AS1
1465	chr11	27503709	27519715	MIR8087
6177	chr3	42019905	42023766	ULK4
6339	chr4	112296212	112302292	ALPK1
3116	chr15	98852699	98868263	MIR4714
2034	chr12	48842640	48853511	DDX23
9026	chr9	38168424	38171827	SHB
8815	chr9	117703323	117710222	TLR4
479	chr1	218284740	218351745	TGFB2
5061	chr2	43072599	43076821	LINC02580
7091	chr5	69327557	69333234	CCDC125
1635	chr11	66969746	66972622	C11orf86
6643	chr4	8190677	8192443	SH3TC1
7512	chr6	26568342	26569596	LOC105374988
9348	chrX	65532429	65536057	LAS1L
9254	chrX	154443768	154461674	MIR6858
8163	chr7	27168389	27175148	MIR196B

6045	chr3	180600513	180623298	TTC14
7749	chr6	82142404	82145856	LINC02542
7388	chr6	14731427	14734920	LINC01108
1596	chr11	65039938	65042981	SAC3D1
2314	chr13	106095344	106098191	LINC00460
6674	chr4	98256774	98264804	RAP1GDS1
2290	chr12	9848620	9871308	CLEC2B
3030	chr15	72730112	72749169	ADPGK-AS1
5828	chr3	114623336	114626282	ZBTB20
8553	chr8	22351786	22354769	SLC39A14
3377	chr16	67467826	67482644	ATP6V0D1
2973	chr15	64084723	64097326	FAM96A
5673	chr22	32473281	32508622	FBXO7
1376	chr11	118358359	118367145	UBE4A
3213	chr16	23128454	23146639	USP31
498	chr1	222617199	222619266	MIA3
5978	chr3	152298127	152462126	TMEM14EP
7281	chr6	117437652	117443617	ROS1
6715	chr5	112975162	112987917	DCP2
4847	chr2	203236114	203242086	CYP20A1
7998	chr7	116510556	116513451	CAV2
9213	chrX	12955332	12958323	TLR8-AS1
6945	chr5	180832569	180863124	ZFP62
693	chr1	36334769	36389274	STK40
7019	chr5	43511498	43516692	C5orf34
8204	chr7	41186245	41189447	LINC01449
2690	chr14	55099929	55104592	LGALS3
2901	chr15	44426188	44429907	CTDSPL2
2380	chr13	28128519	28141853	PAN3
5548	chr21	38896026	38899624	LOC101928398
3935	chr17	79799916	79801664	CBX8
8527	chr8	144893381	144905628	ZNF250
4210	chr19	17133800	17136772	SNORA118
5495	chr21	26844861	26845848	ADAMTS1
529	chr1	226937707	226941057	COQ8A
841	chr1	70411199	70414587	CTH
8406	chr8	103371599	103419650	DCAF13
5806	chr3	107524088	107539402	BBX
5484	chr21	10325420	10344564	BAGE
1688	chr11	78565371	78576080	NARS2
2068	chr12	52031136	52034958	NR4A1
9389	chrY	314966	328009	LINC00685
633	chr1	26687516	26704987	ARID1A
810	chr1	628973	635060	

6756	chr5	132494576	132498965	IRF1
7723	chr6	73521727	73524970	EEF1A1
1489	chr11	34147351	34166682	NAT10
2426	chr13	42995307	42997640	EPSTI1
6574	chr4	52659186	52671321	USP46-AS1
4228	chr19	18363775	18367022	GDF15
9192	chrX	116430223	116464306	SLC6A14
2959	chr15	60405259	60410269	ANXA2
5864	chr3	12483276	12521505	TSEN2
3245	chr16	28950147	28964199	MIR4517
2081	chr12	53173287	53202604	CSAD
9107	chr9	86015594	86029290	GOLM1
344	chr1	179110781	179134939	ABL2
9335	chrX	53439026	53443570	HSD17B10
3421	chr16	72091380	72105033	TXNL4B
6776	chr5	134923286	134970649	
3130	chr15_KI270905v1_alt	2907187	2950665	LOC100288637
5593	chr21	5600961	5605957	LOC102723360
903	chr1	93077936	93090583	MTF2
5972	chr3	15062100	15099765	MRPS25
3620	chr17	35243411	35262323	SLFN5
7729	chr6	75241822	75245275	COX7A2
646	chr1	28248964	28250639	SESN2
4776	chr2	182714317	182746015	DNAJC10
5680	chr22	36408131	36411208	MYH9
1582	chr11	64284333	64287703	GPR137
5524	chr21	34971214	34991565	RUNX1-IT1
1820	chr12	111836799	111843947	MAPKAPK5-AS1
1663	chr11	71298423	71301632	SHANK2
1246	chr10	79657909	79663908	LOC105378385
3897	chr17	75524772	75527023	LLGL2
5777	chr22_KI270879v1_alt	148159	152014	MIF-AS1
9161	chrUn_GL000218v1	87281	98179	LOC100233156
4258	chr19	2524858	2525961	GADD45B
6112	chr3	196280110	196291300	PCYT1A
673	chr1	32599614	32606544	ZBTB8OS
3600	chr17	30830161	30833838	ATAD5
2028	chr12	47751735	47789686	SLC48A1
4723	chr2	161302306	161415596	PSMD14
8306	chr7	74844534	74852640	GTF2IRD2
7930	chr6_GL000256v2_alt	3481605	3498006	AGPAT1
4735	chr2	170977785	170998595	GORASP2
3468	chr16	87789108	87791444	LOC102724467
8929	chr9	136386249	136441149	SDCCAG3

5083	chr2	47268704	47270705	BCYRN1
1747	chr11	96368230	96398475	CCDC82
4768	chr2	180741252	180743687	SCHLAP1
1611	chr11	65711048	65721519	KAT5
6235	chr3	4980841	4988737	BHLHE40-AS1
8719	chr8	93879678	93881355	MIR378D2
5341	chr20	37287382	37311634	MANBAL
5404	chr20	49949922	49963230	RNF114
3675	chr17	42830170	42837651	PSME3
6697	chr5	10595157	10604278	ANKRD33B
421	chr1	204506018	204507585	MDM4
2497	chr13	75759364	75781603	LMO7
5366	chr20	44191688	44215444	OSER1
2164	chr12	65601477	65623578	LINC02454
2756	chr14	72940408	72942751	DCAF4
2484	chr13	73333863	73336879	LINC00392
691	chr1	35807060	35811300	AGO4
8949	chr9	137551060	137553686	MRPL41
8156	chr7	25114920	25127859	CYCS
7977	chr7	107166188	107176140	HBP1
1940	chr12	1688556	1721694	ADIPOR2
7394	chr6	149060584	149064942	LOC105378047
6871	chr5	159257869	159290432	UBLCP1
7741	chr6	79231032	79237926	HMGN3
2519	chr13	95662851	95690593	DNAJC3-DT
7355	chr6	138453515	138456366	MIR3145
9113	chr9	88383684	88388950	SPIN1
6808	chr5	140560200	140572627	APBB3
4397	chr19	45447637	45452115	MIR6088
2465	chr13	51764046	51776580	DHRS12
3399	chr16	69397733	69399657	TERF2
2547	chr14	102358945	102368373	CINP
4120	chr18	73646200	73648152	LINC02582
7118	chr5	74715939	74784797	NSA2
3076	chr15	84732772	84735811	ZNF592
5869	chr3	12578375	12592940	MKRN2
1010	chr10	119891944	119895662	SEC23IP
4941	chr2	233555817	233567904	USP40
3363	chr16	58510447	58522039	SETD6
8618	chr8	42541958	42549554	SLC20A2
7783	chr6	97380996	97383427	LOC101927314
1338	chr11	10510469	10541132	RNF141
1352	chr11	108493436	108499819	KDELC2
8388	chr8	100148257	100158361	POLR2K

8286	chr7	72877979	72923493	POM121
6664	chr4	88516906	88527085	PYURF
8900	chr9	130689474	130691197	EXOSC2
1592	chr11	64847528	64856823	CDC42BPG
3567	chr17	2471612	2486851	METTL16
6352	chr4	119451708	119508604	LOC645513
4093	chr18	51131484	51136173	MEX3C
2041	chr12	49321519	49323037	TROAP
7409	chr6	149962591	149964417	ULBP1
7482	chr6	20876497	20901482	CDKAL1
3506	chr16_KI270855v1_alt	172901	178133	NMRAL1
994	chr10	11607006	11613596	USP6NL
7599	chr6	34790825	34802048	UHRF1BP1
1524	chr11	47831774	47849927	NUP160
1491	chr11	34437119	34440700	CAT
7402	chr6	149692335	149719440	LATS1
420	chr1	204493748	204495937	PIK3C2B
2443	chr13	48021632	48053877	NUDT15
161	chr1	150233369	150237271	ANP32E
795	chr1	58682420	58701503	MYSM1
977	chr10	110356449	110358181	SMNDC1
7963	chr7	103342069	103345480	DNAJC2
8117	chr7	157563678	157565991	MIR153-2
494	chr1	221838937	221841209	LINC01655
7765	chr6	87321215	87324607	SMIM8
7909	chr6_GL000255v2_alt	3089534	3097531	SNORD52
6738	chr5	123509567	123512874	CSNK1G3
1875	chr12	122265307	122267527	VPS33A
7303	chr6	125979751	126038728	TRMT11
6690	chr5	103114440	103122234	GIN1
2848	chr15	32611604	32617764	LOC101928042
205	chr1	153970943	153979063	JTB
3014	chr15	70059678	70099238	MIR629
2271	chr12	93590101	93592688	SOCS2
8502	chr8	143683117	143686390	ZNF707
8836	chr9	124950943	124953596	GOLGA1
1727	chr11	93660740	93673150	CEP295
7559	chr6	29886683	29889970	HLA-H
3998	chr17_GL000258v2_alt	1755215	1758932	WNT3
5186	chr2	74205142	74212588	MTHFD2
1180	chr10	68329238	68350082	PBLD
1997	chr12	32747217	32757287	YARS2
5901	chr3	131479413	131503377	MRPL3
8657	chr8	6403973	6408785	LOC100287015

1982	chr12	30775674	30817206	LINC00941
5155	chr2	68167396	68184856	PNO1
2323	chr13	110011373	110013431	LINC00396
9370	chrX	74280518	74293407	MIR545
891	chr1	89010039	89024158	GBP3
8753	chr9	100003518	100005792	ERP44
4446	chr19	48331538	48333800	EMP3
3713	chr17	45169561	45173428	LOC105371795
6973	chr5	29262027	29263862	LINC02109
1381	chr11	118633737	118635405	MIR6716
7244	chr6	10732881	10744945	TMEM14B
1355	chr11	10898162	10901504	ZBED5-AS1
6324	chr4	105145810	105152170	TET2
4842	chr2	202230752	202239522	SUMO1
5267	chr20	11451570	11456594	LOC339593
916	chr1	93877234	93880752	DNTTIP2
6311	chr3_KI270934v1_alt	152008	160896	LOC105374297
6522	chr4	36092316	36129734	ARAP2
1758	chr11_KI270831v1_alt	195580	223092	NAP1L4
3434	chr16	75147846	75150948	ZFP1
6074	chr3	187738484	187747351	BCL6
8282	chr7	67300469	67305123	STAG3L4
9350	chrX	68432600	68435388	OPHN1
6517	chr4	31088645	31091855	LINC02497
4650	chr2	120210028	120224806	TMEM185B
2838	chr15	22861900	22872114	NIPA2
7256	chr6	109685069	109692730	AK9
5202	chr2	84453454	84460874	SUCLG1
7567	chr6	30719117	30727216	TUBB
2133	chr12	58413210	58416115	LOC100506869
8019	chr7	128527012	128571893	METTL2B
6565	chr4	47872319	47916474	LOC101927157
2563	chr14	105017488	105025114	CDCA4
7260	chr6	110873381	110906563	AMD1
1269	chr10	89283304	89286902	IFIT2
6666	chr4	89052532	89057906	FAM13A
1895	chr12	12438796	12440833	BORCS5
1224	chr10	74106082	74109165	AP3M1
7539	chr6	28211491	28220699	TOB2P1
7277	chr6	116361565	116395463	LOC100287467
5028	chr2	3535017	3564213	RNASEH1
3121	chr15	99702478	99711039	LYSMD4
8736	chr8	97774669	97779895	LAPTM4B
6980	chr5	32409276	32447519	ZFR

8171	chr7	28410110	28445573	CREB5
5408	chr20	50284844	50287553	LINC01270
8738	chr8	98114601	98121571	POP1
551	chr1	231022399	231024960	MIR1182
8852	chr9	127544425	127562985	FAM129B
8532	chr8	17348235	17349654	MTMR7
5591	chr21	46435273	46470331	DIP2A
8498	chr8	143275652	143282393	MINCR
3160	chr16	14620197	14644242	BFAR
6766	chr5	134197900	134225718	PPP2CA
8004	chr7	12064518	12069595	TMEM106B
5200	chr2	81643892	81721489	LINC01815
5854	chr3	122781163	122828787	DIRC2
1864	chr12	120925412	120926920	XLOC_009911
7093	chr5	69559065	69580982	GTF2H2C
9194	chrX	118726838	118731634	IL13RA1
2019	chr12	46383519	46420718	LOC100288798
3831	chr17	64495879	64506147	MIR3064
3256	chr16	29943119	29945022	KCTD13
2581	chr14	22332807	22335037	LOC105370401
1219	chr10	73696269	73733253	BMS1P4
6387	chr4	138172394	138251844	SLC7A11
1186	chr10	68951851	68959419	DDX21
2985	chr15	65302842	65305908	RNU5B-1
7491	chr6	24774345	24791312	GMNN
7004	chr5	39075527	39079373	RICTOR
6468	chr4	17835131	17846234	NCAPG
577	chr1	235480851	235512794	B3GALNT2
3569	chr17	2591350	2602094	PAFAH1B1
7821	chr6_GL000251v2_alt	4609086	4618467	RXRB
4998	chr2	27715126	27718456	LINC01460
7564	chr6	30596225	30597970	MIR877
9155	chr9	98222240	98224133	MIR6854
8119	chr7	16421196	16422629	ISPD
2005	chr12	42316360	42350023	PPHLN1
411	chr1	203025622	203026947	PPFIA4
4139	chr18_GL383567v1_alt	81779	84251	SKA1
9131	chr9	93447742	93507169	FAM120AOS
9009	chr9	35810952	35816452	HINT2
9387	chrY	1590249	1596506	AKAP17A
7024	chr5	50438629	50442137	EMB
5660	chr22	30692578	30696333	OSBP2
1658	chr11	70202075	70212617	FADD
4064	chr18	35284538	35302509	ZNF271P

1496	chr11	35661297	35703511	TRIM44
7726	chr6	73695169	73708398	LOC101928489
3809	chr17	60392339	60396409	USP32
1076	chr10	22815953	22818817	PIP4K2A
5446	chr20	57350736	57377870	MTRNR2L3
5711	chr22	40948417	40961529	RBX1
2471	chr13	54571177	54573620	MIR1297
1033	chr10	126387865	126389378	ADAM12
1473	chr11	2994173	3059411	CARS-AS1
8494	chr8	142696856	142709287	LY6K
4420	chr19	46776131	46785718	SLC1A5
3324	chr16	4800082	4803523	ROGDI
7927	chr6_GL000256v2_alt	294843	297525	HCG15
7211	chr5_GL339449v2_alt	100	4357	MRPS36
2921	chr15	50306388	50373353	GABPB1-AS1
5294	chr20	2663258	2665227	IDH3B
4727	chr2	164573405	164574554	GRB14
6483	chr4	186190000	186198459	CYP4V2
1927	chr12	133046938	133066393	ZNF84
4712	chr2	158967245	158972906	TANC1
6030	chr3	171959665	171964096	TMEM212-AS1
7838	chr6_GL000252v2_alt	2946875	2953011	ABHD16A
1410	chr11	125590660	125599870	STT3A
6967	chr5	24222493	24226885	C5orf17
2743	chr14	68784622	68796096	ZFP36L1
406	chr1	201810638	201812940	MIR1231
1099	chr10	30983558	30987429	ZNF438
4519	chr19	58467925	58469744	ZNF324
3397	chr16	69317713	69330567	PDF
8670	chr8	66533736	66537379	VXN
3686	chr17	43359314	43364930	LINC00910
1541	chr11	59705598	59708230	OR10V1
9324	chrX	49078619	49081261	WDR45
6179	chr3	42629203	42632849	ZBTB47
1158	chr10	60943238	60945714	RHOBTB1
5401	chr20	49774167	49777508	SLC9A8
5571	chr21	43305218	43309604	LINC00322
5933	chr3	141736672	141757859	RNF7
526	chr1	226305268	226310225	LIN9
226	chr1	155926407	155937066	KHDC4
4361	chr19	42869545	42871889	PSG1
6613	chr4	74132764	74138062	MTHFD2L
9316	chrX	48674276	48679621	WAS
9088	chr9	73136137	73138458	ANXA1

7342	chr6	136020050	136022780	LOC644135
1132	chr10	45948290	46031979	SNORA74C-1
3261	chr16	3018138	3029759	THOC6
5142	chr2	65204192	65206384	ACTR2
7767	chr6	87578539	87610387	ORC3
803	chr1	6020967	6026988	KCNAB2
6527	chr4	37811945	37813598	PGM2
3882	chr17	7482676	7487314	POLR2A
2878	chr15	41395822	41397535	NDUFAF1
2404	chr13	36979525	37013988	ALG5
1952	chr12	22534242	22547245	C2CD5
1398	chr11	123082550	123090165	HSPA8
5453	chr20	59062062	59065135	SLMO2-ATP5E
3819	chr17	62515923	62517993	TLK2
4422	chr19	46858889	46871888	AP2S1
2993	chr15	66350869	66357774	TIPIN
7868	chr6_GL000253v2_alt	4616799	4627071	RXRB
8201	chr7	40141226	40143927	SUGCT
350	chr1	179953812	179965673	CEP350
3614	chr17	32444925	32461726	PSMD11
7131	chr5	78510708	78530558	LHFPL2
7959	chr7	102621872	102671656	SPDYE2
6510	chr4	26583357	26587791	TBC1D19
7772	chr6	89146294	89150754	PM20D2
473	chr1	214900787	214903844	KCNK2
1834	chr12	114921606	114937971	TBX3
8466	chr8	127338043	127341405	CCAT2
5780	chr22_KI270879v1_alt	265663	274016	GSTT1-AS1
946	chr10	101621504	101624910	MIR3158-2
3349	chr16	56813987	56819636	NUP93
7856	chr6_GL000253v2_alt	206217	206985	LINC01556
8627	chr8	47659322	47660541	SPIDR
7468	chr6	17032487	17034053	STMND1
1249	chr10	80205565	80223342	LINC00857
460	chr1	211927661	211930181	LOC102723727
7820	chr6_GL000251v2_alt	427316	427954	LINC01556
7374	chr6	143481853	143518441	FUCA2
6974	chr5	30504391	30506734	LOC105374704
720	chr1	42323845	42341729	FOXJ3
7259	chr6	110812003	110816546	AMD1
6036	chr3	173394530	173396668	NLGN1
7011	chr5	40833360	40837929	RPL37
7626	chr6	38635606	38647489	BTBD9
5923	chr3	138822563	138826506	PIK3CB

4147	chr19	10571921	10577725	CDKN2D
2012	chr12	45211210	45223013	PLEKHA8P1
1668	chr11	72811966	72815563	ATG16L2
6184	chr3	44463965	44477902	ZNF445
1418	chr11	12838192	12855634	LINC00958
667	chr1	31953894	31957443	PTP4A2
8488	chr8	141074158	141077686	DENND3
39	chr1	11011243	11016495	TARDBP
3069	chr15	82746452	82755296	SNHG21
6129	chr3	197750389	197758792	FYTTD1
5124	chr2	61755496	61767074	FAM161A
3020	chr15	71779866	71786789	NR2E3
6083	chr3	189907813	189910696	MIR944
6101	chr3	194569987	194579967	TMEM44-AS1
1628	chr11	66590671	66597826	CCS
9217	chrX	129904542	129915390	UTP14A
8705	chr8	86495421	86510321	RMDN1
1701	chr11	86175127	86178524	EED
5575	chr21	43653141	43667985	RRP1B
230	chr1	156121960	156143408	LMNA
6961	chr5	20678020	20680495	LINC02146
8061	chr7	140918635	140926445	BRAF
771	chr1	52026405	52034632	KTI12
2146	chr12	6310564	6337482	PLEKHG6
7081	chr5	660140	667078	TPPP
9258	chrX	155215284	155221382	VBP1
1036	chr10	130134587	130231602	GLRX3
4870	chr2	207611492	207613777	METTL21A
2632	chr14	35825421	35828206	BRMS1L
6358	chr4	122763035	122768045	BBS12
638	chr1	26993169	26994491	TRNP1
359	chr1	181134159	181137743	LINC01732
6751	chr5	132289923	132299506	SLC22A4
3136	chr15_KI270905v1_alt	863969	867359	HERC2P2
7917	chr6_GL000256v2_alt	1355337	1370295	ZNRD1
9198	chrX	119602845	119607443	NKRF
7496	chr6	26032175	26034887	HIST1H2AB
1925	chr12	132755447	132769318	ANKLE2
3395	chr16	69115132	69117369	HAS3
6942	chr5	180066513	180073034	RNF130
3437	chr16	758811	782399	MIR662
2097	chr12	54355434	54359117	LOC102724050
6838	chr5	143920132	143924980	HMHB1
9303	chrX	46542523	46553782	ZNF674-AS1

8693	chr8	80161901	80173127	TPD52
4128	chr18	807042	815782	YES1
6895	chr5	172756584	172759793	LOC101928093
7886	chr6_GL000254v2_alt	4635038	4643188	HSD17B8
2877	chr15	41330765	41335318	NUSAP1
3183	chr16	18945259	18947283	SMG1
5250	chr2	97588789	97591111	ANKRD36B
8859	chr9	127948916	127969686	FAM102A
5103	chr2	54966937	54972682	RTN4
1419	chr11	128462429	128508851	ETS1
9203	chrX	119868231	119881746	RNF113A
4294	chr19	36013141	36015960	LOC101927572
4813	chr2	199909918	199913130	C2orf69
7236	chr6	106269605	106327129	ATG5
6223	chr3	48918085	48932297	ARIH2OS
3264	chr16	30347212	30358167	CD2BP2
449	chr1	209805503	209806808	IRF6
4588	chr1_KI270765v1_alt	77802	80329	RNVU1-11
6359	chr4	122781040	122784758	FGF2
1360	chr11	111727641	111732648	PPP2R1B
1079	chr10	2389178	2391287	LINC00701
7880	chr6_GL000254v2_alt	3042920	3046849	ABHD16A
6537	chr4	39276160	39289113	RFC1
9075	chr9	70256955	70279344	SMC5
7942	chr7	100331722	100344723	PMS2P1
7751	chr6	82395256	82402499	TPBG
8812	chr9	115614435	115617051	LOC101928775
9043	chr9	5507805	5511438	PDCD1LG2
4817	chr2	200305257	200307571	SPATS2L
3714	chr17	45175592	45177598	LOC105371795
4374	chr19	43594239	43626909	SRRM5
6464	chr4	176740529	176782471	VEGFC
6673	chr4	98142049	98145207	STPG2
573	chr1	235060197	235069985	LINC01348
7060	chr5	60931029	60983399	NDUFAF2
6017	chr3	16254906	16277438	OXNAD1
6201	chr3	47355029	47356512	PTPN23
6025	chr3	170354944	170358174	SKIL
7439	chr6	158229031	158238895	GTF2H5
727	chr1	43205629	43208784	EBNA1BP2
1014	chr10	121920800	121931673	ATE1
7177	chr5	93259665	93262078	NR2F1-AS1
4896	chr2	218658767	218671849	BCS1L
8400	chr8	102323763	102419356	UBR5

3408	chr16	70341928	70352353	DDX19A
2972	chr15	63824143	63836000	HERC1
7092	chr5	694102	703519	TPPP
840	chr1	70296772	70356732	ANKRD13C
7050	chr5	57171125	57173664	GPBP1
3916	chr17	76707074	76750882	METTL23
7107	chr5	72814466	72865742	TNPO1
2247	chr12	87827019	87829057	MKRN9P
983	chr10	110911311	110976547	RPL13AP6
2570	chr14	20410627	20415137	TEP1
4300	chr19	3651071	3657784	CACTIN
2075	chr12	52894191	52905263	KRT8
1675	chr11	74592065	74600398	POLD3
5144	chr2	65271743	65281105	ACTR2
3781	chr17	5446181	5468706	DHX33
9169	chrX	101394886	101400040	RPL36A-HNRNPH2
8598	chr8	37779206	37782444	ADGRA2
4468	chr19	50475760	50481791	FAM71E1
7919	chr6_GL000256v2_alt	1855134	1869043	GNL1
8088	chr7	151348767	151357050	NUB1
5902	chr3	132510838	132521535	ACKR4
9066	chr9	66899912	66904973	ZNF658
4190	chr19	14414792	14421186	DDX39A
3616	chr17	34958837	34974833	ZNF830
1718	chr11	90186722	90226366	CHORDC1
1279	chr10	91597320	91599713	HECTD2-AS1
3960	chr17	81513424	81524391	ACTG1
5499	chr21	29011865	29021946	RWDD2B
5002	chr2	28389332	28432111	FLJ31356
3776	chr17	51165863	51171335	NME2
1234	chr10	77354792	77357509	KCNMA1-AS3
1215	chr10	73490912	73502507	PPP3CB
8319	chr7	76510177	76512387	UPK3B
1311	chr10	97710417	97720684	MARVELD1
3164	chr16	14972922	14980601	PDXDC1
2188	chr12	6942529	6945676	C12orf57
537	chr1	228138200	228158132	GJC2
5425	chr20	51411908	51413466	MIR3194
6018	chr3	165460906	165471634	LINC01322
3378	chr16	67527476	67533971	RIPOR1
384	chr1	192980466	193074269	SCARNA18B
1085	chr10	26486227	26488008	APBB1IP
508	chr1	224249570	224267360	MIR320B2
8937	chr9	136938124	136945904	FBXW5

8729	chr8	95099948	95101219	MIR3150BHG
777	chr1	52382673	52395610	ORC1
3914	chr17	76669978	76673415	LOC105274304
480	chr1	218584184	218587198	C1orf143
1898	chr12	124824074	124829768	SCARB1
5984	chr3	15422100	15437341	EAF1
1016	chr10	121971453	121978186	NSMCE4A
5582	chr21	44516468	44534154	TSPEAR-AS2
6655	chr4	84581918	84585350	CDS1
3074	chr15	84080291	84081725	EFL1P1
8263	chr7	65437229	65440095	ZNF92
2391	chr13	32313722	32323108	BRCA2
5106	chr2	55147651	55240490	RPS27A
3501	chr16_KI270853v1_alt	858768	875433	MIR3180-2
4606	chr2	10513555	10516933	LOC101929715
9346	chrX	63921394	63923673	MIR1468
9373	chrX	75326605	75328415	UPRT
506	chr1	224112910	224157582	FBXO28
8301	chr7	74218919	74222238	LAT2
4435	chr19	47647303	47648717	BICRA
4872	chr2	207710150	207715875	CCNYL1
784	chr1	5423745	5425601	
6119	chr3	196710749	196719828	PIGX
4775	chr2	181953085	181955981	PPP1R1C
2580	chr14	21497568	21514007	METTL3
4803	chr2	196795088	196802942	GTF3C3
665	chr1	31939097	31939843	PTP4A2
1906	chr12	127229285	127232730	LINC02376
1071	chr10	21997937	22004610	DNAJC1
2937	chr15	55303672	55321718	PIGB
2225	chr12	76855504	76858329	CSRP2
8911	chr9	13278524	13280554	MPDZ
3747	chr17	4831744	4844929	MINK1
594	chr1	241531032	241532950	KMO
6628	chr4	778332	782984	LOC100129917
467	chr1	212779713	212794013	NSL1
5120	chr2	61175920	61195510	AHSA2P
1949	chr12	21436305	21466362	PYROXD1
2624	chr14	34704494	34715886	CFL2
6293	chr3	93469889	93471147	PROS1
2885	chr15	42088660	42093776	PLA2G4D
1133	chr10	4661613	4664489	LINC00705
251	chr1	159075758	159078298	AIM2
4830	chr2	201056448	201075693	FAM126B

3692	chr17	43769740	43780107	DUSP3
1939	chr12	1659455	1661524	MIR3649
6442	chr4	167902265	167903913	ANXA10
844	chr1	74197339	74203242	FPGT
1088	chr10	27149748	27158830	YME1L1
3805	chr17	59836214	59847522	MIR21
5374	chr20	45059812	45097626	KCNS1
2180	chr12	68685132	68692680	NUP107
858	chr1	7999486	8026236	ERRFI1
7690	chr6	56530212	56544033	DST
4068	chr18	35972517	35986262	C18orf21
1835	chr12	115312466	115339258	TBX3
5527	chr21	35197038	35199551	RUNX1
5778	chr22_KI270879v1_alt	201587	212522	DDT
5927	chr3	14018151	14020303	TPRXL
4006	chr17_KI270857v1_alt	1634029	1648742	ACACA
4901	chr2	219243519	219252986	STK16
8745	chr8_KI270815v1_alt	122561	131259	ZNF251
6893	chr5	172417818	172421782	SH3PXD2B
3242	chr16	28812509	28848351	ATXN2L
856	chr1	77923461	78011954	DNAJB4
5364	chr20	43974829	43977479	TOX2
8519	chr8	144477148	144490779	PPP1R16A
7068	chr5	62399200	62405841	DIMT1
6903	chr5	172903166	172907407	RPL26L1
8854	chr9	127611188	127617555	STXBP1
222	chr1	155612430	155618400	MSTO2P
2639	chr14	39113194	39115944	GEMIN2
5431	chr20	52191874	52193111	ZFP64
3942	chr17	80450590	80456816	NPTX1
8920	chr9	134065100	134068058	BRD3
2660	chr14	49866776	49869727	RN7SL2
5851	chr3	122358555	122514876	LOC102723582
9345	chrX	63745445	63757146	ARHGEF9
6615	chr4	74443558	74458984	AREG
815	chr1	63730823	63733017	ROR1
1589	chr11	64777791	64781212	SF1
8410	chr8	106057235	106062637	ZFPM2-AS1
8055	chr7	139337270	139343012	LUC7L2
413	chr1	203511196	203532817	OPTC
1209	chr10	72687617	72689050	MCU
719	chr1	41379826	41386109	LOC101929901
4183	chr19	13827424	13840770	LOC284454
3265	chr16	30376232	30379443	ZNF48

2100	chr12	55919716	55942721	DGKA
4416	chr19	46287962	46290266	HIF3A
4724	chr2	163209221	163212012	KCNH7
2132	chr12	57932094	57937564	LOC100506844
8247	chr7	56095369	56106542	CHCHD2
8787	chr9	111742121	111745369	C9orf84
5971	chr3	150576268	150615578	SELENOT
4683	chr2	142866428	142870150	KYNU
9159	chr9_GL383542v1_alt	18127	20122	
6285	chr3	86792949	86795501	VGLL3
8021	chr7	128863178	128870329	LOC100130705
5893	chr3	129238104	129241276	COPG1
375	chr1	189656747	189659518	BRINP3
8810	chr9	115088013	115118265	TNC
3327	chr16	48585389	48613896	N4BP1
5585	chr21	44851714	44879432	PTTG1IP
223	chr1	155650905	155691022	SCARNA26A
2789	chr14	77799567	77802172	ADCK1
6341	chr4	113663579	113764267	CAMK2D
3167	chr16	15151273	15154479	MIR3180-4
7986	chr7	10972725	10982255	PHF14
5701	chr22	39243331	39245496	PDGFB
1637	chr11	67053511	67063473	RHOD
6326	chr4	105694292	105729092	GSTCD
1474	chr11	30322270	30349823	ARL14EP
5288	chr20	2503820	2511120	ZNF343
8271	chr7	66561380	66594098	GS1-124K5.11
8658	chr8	6532909	6593844	ANGPT2
2085	chr12	53370458	53372903	SP1
2806	chr14	92023799	92042703	TRIP11
565	chr1	234474644	234480019	TARBP1
3243	chr16	28862735	28880086	ATP2A1
5011	chr2	29118222	29125935	CLIP4
2588	chr14	22954162	22958333	MIR4707
7624	chr6	38205560	38207920	BTBD9-AS1
4339	chr19	40714028	40723303	ITPKC
587	chr1	237626027	237627057	MIR4428
3912	chr17	76383000	76390906	SPHK1
8041	chr7	133253137	133261614	EXOC4
4259	chr19	2538767	2542534	GADD45B
8695	chr8	81049813	81052038	PAG1
3493	chr16_KI270853v1_alt	1233386	1237330	MIR6506
299	chr1	167720105	167726378	MPZL1
711	chr1	39797809	39802142	LOC101929536

8828	chr9	122824348	122829242	PDCL
1722	chr11	93054017	93058365	MTNR1B
2709	chr14	61747761	61755597	LOC105370526
5773	chr22_GL383582v2_alt	511	3975	SMDT1
4328	chr19	39966457	39976251	PSMC4
3729	chr17	47060787	47073099	RPRML
2922	chr15	50422600	50439678	USP8
6109	chr3	195892261	195897255	TNK2
7102	chr5	71111197	71119588	NAIP
6090	chr3	190384551	190388142	CLDN16
8063	chr7	143374043	143396392	MIR6892
5813	chr3	111673205	111678525	PLCXD2
5491	chr21	17587736	17616174	BTG3-AS1
1932	chr12	14774276	14780710	H2AFJ
4356	chr19	42395338	42404327	LIPE-AS1
6768	chr5	134371430	134390098	UBE2B
3556	chr17	20531021	20533558	KRT16P3
181	chr1	151305146	151313607	PI4KB
509	chr1	224324691	224331681	NVL
1934	chr12	14835348	14837613	ART4
6206	chr3	4747584	4752345	EGOT
6976	chr5	31853286	31858421	MIR4279
4565	chr19_GL949746v1_alt	352815	370048	LENG8
1206	chr10	72311071	72327490	DNAJB12
2084	chr12	53319405	53328278	AAAS
1685	chr11	77813645	77825112	RSF1
7231	chr6	10353477	10372219	TFAP2A-AS2
1478	chr11	32582911	32612163	EIF3M
7524	chr6	27188154	27189152	MIR3143
3615	chr17	32485758	32487973	CDK5R1
2083	chr12	53294627	53304348	C12orf10
3096	chr15	90865235	90886968	FURIN
1284	chr10	92006198	92041523	BTAF1
4307	chr19	3742856	3764570	APBA3
1414	chr11	126301447	126305493	DCPS
6042	chr3	179573196	179623818	MRPL47
4746	chr2	173047821	173050757	MAP3K20
3214	chr16	232384	238926	FAM234A
8575	chr8	27872066	27873641	MIR4287
2503	chr13	78021346	78023205	LINC00446
5719	chr22	41609888	41626304	DESI1
7417	chr6	151066283	151094494	MTHFD1L
6844	chr5	148382270	148430598	FBXO38
7083	chr5	66581342	66583976	MAST4

5750	chr22	46005208	46017657	LOC730668
6369	chr4	126849356	126851880	INTU
2367	chr13	21525587	21531687	ZDHHC20
9000	chr9	34325615	34329495	KIF24
2218	chr12	75931882	75935421	PHLDA1
6001	chr3	157498542	157500749	VEPH1
5031	chr2	36403693	36437993	CRIM1
8052	chr7	139132644	139136801	TTC26
113	chr1	145278888	145282801	RNVU1-14
4390	chr19	44953427	44959667	CLPTM1
6915	chr5	174410334	174420528	LINC01411
1485	chr11	33682960	33703344	C11orf91
4492	chr19	55548387	55551258	SBK3
306	chr1	1687545	1694370	SLC35E2B
4499	chr19	56671969	56674338	ZNF835
9168	chrX	101336463	101349549	TIMM8A
3124	chr15_GL383554v1_alt	4964	12872	HERC2P11
823	chr1	65423349	65442009	LEPROT
7294	chr6	12287080	12300933	EDN1
7707	chr6	70406050	70431112	LOC105377849
3564	chr17	2365853	2371362	SGSM2
7532	chr6	27829103	27856481	HIST1H2BN
5306	chr20	32353914	32458925	ASXL1
5490	chr21	17504900	17515553	CXADR
5047	chr2	37923009	37928072	RMDN2
1806	chr12	110124039	110136935	IFT81
7997	chr7	116497087	116506020	CAV2
3064	chr15	80981715	80991172	MESD
7307	chr6	127281606	127352169	ECHDC1
1501	chr11	413162	427686	SIGIRR
4340	chr19	40750535	40757921	SNRPA
1931	chr12	14759736	14772605	HIST4H4
9308	chrX	47231035	47242981	USP11
1136	chr10	47550051	47556470	BMS1P2
3104	chr15	92818703	92821888	ASB9P1
1981	chr12	30686616	30695838	IPO8
3855	chr17	68380307	68385468	PRKAR1A
2080	chr12	53097475	53100096	IGFBP6
6521	chr4	36002417	36008077	ARAP2
4329	chr19	40052163	40092086	ZNF780B
4668	chr2	131087167	131095487	FAM168B
8993	chr9	33766280	33771680	PRSS3
8161	chr7	27106157	27108516	HOXA2
2331	chr13	110667498	110707026	CARS2

4050	chr18	23502182	23504377	RMC1
4048	chr18	23106186	23108139	CABLES1
8290	chr7	73305900	73310284	NSUN5
5298	chr20	31603730	31608411	ID1
3584	chr17	28893105	28900345	FLOT2
5265	chr20	10510729	10512666	SLX4IP
4628	chr2	11176400	11205073	PQLC3
7001	chr5	38806997	38862859	OSMR-AS1
5715	chr22	41278577	41290569	RANGAP1
8696	chr8	81521083	81522423	FABP12
4744	chr2	172425709	172484736	ITGA6
6842	chr5	146447544	146510458	TCERG1
1587	chr11	64657182	64661415	NRXN2
2701	chr14	59478518	59487427	L3HYPDH
2553	chr14	103360222	103362771	SNORA28
4429	chr19	47254809	47261838	CCDC9
7931	chr6_GL000256v2_alt	4369302	4371691	BRD2
8650	chr8	60644515	60654725	CHD7
7221	chr6	100227133	100230207	MCHR2
2785	chr14	77318832	77324301	GSTZ1
5067	chr2	44328259	44363386	PREPL
5856	chr3	123200750	123228865	SEC22A
8047	chr7	135557007	135569957	NUP205
2263	chr12	92759155	92885292	PLEKHG7
7533	chr6	27870738	27874438	HIST1H3I
8132	chr7	19112975	19118006	TWIST1
8513	chr8	144288462	144302011	HSF1
7651	chr6	43454297	43458817	DLK2
3311	chr16	4252518	4254273	LINC01569
1967	chr12	27709578	27750812	MRPS35
7687	chr6	54873054	54927265	FAM83B
6373	chr4	127872259	127884944	PLK4
167	chr1	150559153	150582983	ADAMTSL4-AS1
4547	chr19	8383588	8392587	MIR4999
8515	chr8	144353953	144363183	SLC52A2
896	chr1	91498489	91535464	CDC7
2995	chr15	66503707	66509681	ZWILCH
7047	chr5	56122816	56124782	ANKRD55
6072	chr3	186801632	186808779	RFC4
1724	chr11	93142233	93151223	SLC36A4
9255	chrX	154484790	154492056	UBL4A
6923	chr5	177344847	177353149	LMAN2
8992	chr9	33522303	33565507	ANKRD18B
4890	chr2	217221907	217224871	DIRC3-AS1

504	chr1	223887227	223889415	TP53BP2
3277	chr16	30813684	30816477	ZNF629
525	chr1	226135072	226191800	ACBD3-AS1
6398	chr4	139544856	139559877	SETD7
676	chr1	32768522	32777618	KIAA1522
3431	chr16	74694691	74696723	MLKL
7842	chr6_GL000252v2_alt	3145297	3148274	C2
8328	chr7	77413310	77418350	GSAP
124	chr1	146034681	146036183	HJV
779	chr1	52689616	52699556	COA7
7405	chr6	149811680	149825370	RAET1E-AS1
7266	chr6	11179524	11233742	NEDD9
2963	chr15	62894917	62896897	MIR190A
8776	chr9	108879913	108944953	ELP1
2211	chr12	750351	766571	WNK1
57	chr1	113662787	113665489	PHTF1
7935	chr6_KI270801v1_alt	646122	870455	PTPRK
7151	chr5	82009269	82013090	ATG10
4837	chr2	201764717	201782118	ALS2
1896	chr12	124429694	124430738	MIR6880
3595	chr17	30115373	30120121	MIR3184
6720	chr5	115414091	115416594	CCDC112
8621	chr8	42892923	42903883	HOOK3
2421	chr13	41309695	41323327	MTRF1
3929	chr17	79035324	79037691	C1QTNF1
3237	chr16	2853470	2862632	PRSS22
5533	chr21	36054392	36062527	SETD4
6788	chr5	137885398	137891668	PKD2L2
8082	chr7	151027062	151043939	ABCB8
7500	chr6	26156421	26173912	HIST1H2BD
5859	chr3	123634738	123641962	MYLK
2576	chr14	21347181	21384744	SUPT16H
7051	chr5	57365868	57368056	ACTBL2
6913	chr5	173762812	173765825	LINC01484
2682	chr14	53147550	53154563	DDHD1
8389	chr8	100209643	100267524	RNF19A
3392	chr16	68843661	68846098	TANGO6
6669	chr4	94206908	94212594	SMARCAD1
3066	chr15	82092659	82095669	LINC01583
2090	chr12	53937746	53940877	HOXC13-AS
261	chr1	16074874	16077050	FAM131C
3371	chr16	67109107	67110737	C16orf70
5316	chr20	35086852	35094489	TRPC4AP
126	chr1	146050714	146054374	LINC01719

6591	chr4	61373072	61376723	ADGRL3
4004	chr17_KI270857v1_alt	1086521	1088856	LHX1-DT
8060	chr7	140694971	140704580	NDUFB2-AS1
8153	chr7	24901840	24908759	OSBPL3
7467	chr6	169448786	169454299	THBS2
4701	chr2	151408753	151428460	RIF1
604	chr1	244598784	244601768	DESI2
4337	chr19	40568321	40570954	SHKBP1
4505	chr19	57319068	57325495	ZNF543
3125	chr15_KI270849v1_alt	102922	109593	TRIM69
7125	chr5	76789620	76792148	F2RL1
6852	chr5	149998103	150046658	TIGD6
2294	chr12	98642788	98644940	IKBIP
1717	chr11	8981164	8985149	TMEM9B
4423	chr19	46892863	46897374	ARHGAP35
6950	chr5	181187347	181189014	LINC01962
2737	chr14	68191254	68193797	RAD51B
8014	chr7	127375159	127395444	ZNF800
1862	chr12	120685817	120691142	MLEC
5523	chr21	34941449	34958833	RUNX1
3070	chr15	82808171	82846590	WHAMM
7790	chr6_GL000250v2_alt	181796	191180	TRIM27
3919	chr17	78185749	78188553	TK1
3495	chr16_KI270853v1_alt	1542877	1547784	FOPNL
1499	chr11	390789	401132	PKP3
7985	chr7	109642796	109648562	EIF3IP1
4652	chr2	121754050	121762159	TSN
1566	chr11	62752641	62754788	ZBTB3
945	chr10	101609857	101616781	MIR3158-2
3294	chr16	31699574	31712956	CLUHP3
4215	chr19	1746369	1750584	ONECUT3
5543	chr21	37340802	37342839	DYRK1A
611	chr1	246552933	246665595	CNST
6575	chr4	52710310	52719155	SNORA26
3885	chr17	75033404	75035185	KCTD2
3216	chr16	23575444	23583132	NDUFAB1
8951	chr9	137608322	137625211	ARRDC1-AS1
436	chr1	207239278	207242465	CD55
8349	chr7	91556929	91559876	FZD1
952	chr10	102394430	102408956	NFKB2
5196	chr2	75646157	75651346	MRPL19
5334	chr20	361948	365049	NRSN2
6539	chr4	39444020	39461991	RPL9
1442	chr11	17076164	17081633	RPS13

1388	chr11	119083732	119086183	HMBS
8475	chr8	129853828	129941286	FAM49B
2907	chr15	44706019	44714950	B2M
433	chr1	207025016	207034903	C1orf116
4184	chr19	13845264	13853206	MIR23A
1937	chr12	1604953	1606878	WNT5B
110	chr1	144566386	144570076	RNVU1-11
1832	chr12	114672536	114684064	TBX3
4702	chr2	152095940	152100079	CACNB4
993	chr10	114778452	114781470	ABLIM1
6592	chr4	6639780	6645349	MRFAP1
3019	chr15	71747385	71765068	NR2E3
8031	chr7	130372839	130374701	CPA1
7312	chr6	131134427	131137065	AKAP7
8712	chr8	9001923	9029902	ERI1
5079	chr2	46855064	46858123	LINC01119
918	chr1	94217281	94239369	ARHGAP29
7890	chr6_GL000255v2_alt	1202114	1204913	HLA-A
7039	chr5	55159434	55162579	GPX8
8125	chr7	1740582	1743925	ELFN1-AS1
6244	chr3	50270868	50292682	LSMEM2
5020	chr2	32163890	32177459	SLC30A6
6195	chr3	45688192	45701027	SACM1L
674	chr1	32647403	32679522	RBBP4
9314	chrX	48572686	48583430	RBM3
5537	chr21	36908246	36911227	HLCS
6899	chr5	172832156	172835232	ERGIC1
2360	chr13	20524534	20527332	CRYL1
6881	chr5	163648572	163651593	MAT2B
4357	chr19	4246221	4249510	YJU2
3733	chr17	47306522	47307976	THCAT158
1606	chr11	65557073	65560107	LTBP3
5380	chr20	45778007	45780312	WFDC3
7795	chr6_GL000250v2_alt	3137093	3140493	LSM2
4756	chr2	176095843	176097947	HOXD12
1743	chr11	95765446	95814186	FAM76B
3404	chr16	69724447	69729188	NQO1
5477	chr20	63861507	63871659	TPD52L2
1669	chr11	72822827	72824739	ATG16L2
1228	chr10	75110051	75117432	SAMD8
5040	chr2	37229142	37233708	CEBPZ
1520	chr11	47413970	47427632	PSMC3
3639	chr17	38866810	38868315	LASP1
787	chr1	55063137	55073455	PCSK9

2399	chr13	34940470	34977419	NBEA
6192	chr3	45075169	45146110	CDCP1
5920	chr3	136852844	136865298	NCK1-DT
2745	chr14	68961794	68983135	ACTN1
870	chr1	84477623	84511849	SPATA1
599	chr1	243266884	243269106	SDCCAG8
4510	chr19	5785321	5792307	DUS3L
7407	chr6	149877718	149881052	RAET1E-AS1
4851	chr2	20436055	20440166	RHOB
8549	chr8	2126810	2130049	MIR7160
8644	chr8	56964040	56994505	IMPAD1
1497	chr11	36773887	36775911	RAG2
7435	chr6	157822645	157827046	SNX9
6498	chr4	20192192	20195510	SLIT2
9375	chrX	77769934	77786803	ATRX
8654	chr8	61754863	61763833	MIR4470
9334	chrX	53433023	53435851	HSD17B10
9060	chr9	65381562	65385247	FOXD4L5
5988	chr3	155868751	155904653	GMPS
9368	chrX	73916529	73918328	JPX
8025	chr7	129642548	129651438	NRF1
204	chr1	153956136	153969744	SLC39A1
6167	chr3	38167646	38269416	SLC22A13
3605	chr17	31092742	31103574	NF1
7602	chr6	35650360	35652066	MIR5690
1386	chr11	119045614	119059990	HYOU1
5906	chr3	13281912	13283641	NUP210
4472	chr19	5172520	5174467	PTPRS
3304	chr16	368699	372315	MRPL28
4221	chr19	18151284	18153035	PIK3R2
6144	chr3	29282907	29294452	RBMS3
6319	chr4	102342787	102346085	SLC39A8
8500	chr8	143571026	143575250	MROH6
4290	chr19	35647400	35652564	COX6B1
4207	chr19	16538955	16546563	CHERP
4695	chr2	150493815	150501287	RND3
3515	chr17	1523755	1526262	PITPNA-AS1
8663	chr8	66112068	66115163	TRIM55
5603	chr21_GL383581v2_alt	25839	30800	IFNGR2
832	chr1	6695771	6703101	DNAJC11
1191	chr10	70127497	70133767	AIFM2
9039	chr9	4678094	4682199	CDC37L1
4624	chr2	111196375	111384521	MIR4435-2
1441	chr11	16605551	16608475	SOX6

2683	chr14	53390125	53393102	LOC101927620
6095	chr3	192956647	192958938	MB21D2
8016	chr7	128408744	128410684	IMPDH1
1886	chr12	123231813	123254730	C12orf65
4765	chr2	178425696	178446444	PRKRA
4764	chr2	178413738	178418614	LOC101927027
6742	chr5	126869250	126872023	LMNB1
1744	chr11	95838829	95864144	CEP57
5110	chr2	55599038	55624216	PPP4R3B
7181	chr5	95505342	95556688	TTC37
8709	chr8	89703338	89793879	LOC101929709
6104	chr3	194667728	194673869	LSG1
4533	chr19	6826829	6830177	VAV1
5992	chr3	15635122	15637244	BTD
2365	chr13	21138035	21142210	SAP18
3704	chr17	44888849	44900396	EFTUD2
3188	chr16	1982631	1989584	GFER
6229	chr3	49352208	49360627	GPX1
3833	chr17	64566852	64664469	SMURF2
5482	chr20	64253496	64262070	PCMTD2
774	chr1	52142491	52148054	ZFYVE9
6985	chr5	34005645	34009885	AMACR
4509	chr19	57804721	57857204	ZNF587B
1323	chr11	102255298	102257550	BIRC3
1983	chr12	30850451	30853577	LINC00941
6918	chr5	176446208	176452551	FAF2
6803	chr5	14009518	14013434	DNAH5
7145	chr5	80405846	80436297	ZFYVE16
2805	chr14	91507184	91512744	PPP4R3A
3254	chr16	29820192	29831600	MVP
1453	chr11	18627937	18637339	SPTY2D1
7128	chr5	77063597	77079659	SNORA47
2108	chr12	56186353	56191073	SMARCC2
3847	chr17	67716488	67743565	SNORA38B
5756	chr22	46450596	46456255	CELSR1
8971	chr9	21959601	21976156	CDKN2A-DT
861	chr1	8060282	8062417	ERRFI1
4625	chr2	111456438	111497173	MIR4435-2HG
544	chr1	229512264	229514939	LOC101927478
8775	chr9	108477205	108479628	ACTL7B
4477	chr19	52268154	52272131	ZNF766
8571	chr8	27272009	27275694	STMN4
1987	chr12	31555793	31591200	DENND5B-AS1
3583	chr17	28854737	28857178	ERAL1

1742	chr11	9571406	9584098	WEE1
7755	chr6	8415240	8437768	SLC35B3
898	chr1	92287289	92323062	GLMN
9162	chrUn_GL000220v1	129459	142146	MIR6724-2
871	chr1	84566899	84575819	CTBS
1444	chr11	17201150	17213423	PIK3C2A
759	chr1	47311789	47315672	STIL
5938	chr3	142435586	142451166	XRN1
7776	chr6	89828349	89849371	CASP8AP2
2988	chr15	65604277	65613971	INTS14
7247	chr6	107946146	107959703	SEC63
15	chr1	10599050	10600257	PEX14
7428	chr6	155222914	155225170	TIAM2
2406	chr13	38327139	38369258	UFM1
3354	chr16	57243618	57259956	ARL2BP
8606	chr8	38786148	38788054	TACC1
1642	chr11	67360607	67379187	CLCF1
5542	chr21	37337439	37340057	DYRK1A
8876	chr9	128786106	128788524	TBC1D13
3655	chr17	41505538	41507519	KRT13
724	chr1	42922933	42928981	SLC2A1-AS1
448	chr1	209776142	209786033	C1orf74
2748	chr14	69187482	69192991	EXD2
2982	chr15	65131251	65135536	PDCD7
5929	chr3	14108017	14113919	CHCHD4
3765	chr17	50347069	50349696	XYLT2
4922	chr2	229453640	229456212	PID1
3933	chr17	79768962	79770628	CBX2
3700	chr17	44488100	44505048	GPATCH8
2285	chr12	95937596	95943948	CCDC38
6130	chr3	197890215	197900034	IQCG
5245	chr2	96332910	96337294	NCAPH
2610	chr14	31017091	31026217	AP4S1
5358	chr20	4134783	4137715	SMOX
5958	chr3	149565845	149577561	WWTR1
2198	chr12	70741419	70753672	PTPRR
8109	chr7	155297718	155314096	INSIG1
8772	chr9	107750968	107760072	KLF4
4675	chr2	135181367	135184294	SNORA40B
8039	chr7	131469415	131534317	PODXL
3137	chr16	10455142	10465864	LINC01290
6789	chr5	138025100	138035526	LOC100130172
7792	chr6_GL000250v2_alt	2010637	2013762	PPP1R18
8521	chr8	144518178	144530244	LRRC24

1829	chr12	112379330	112384070	HECTD4	
6437	chr4	163569804	163571794		1-Mar
7293	chr6	122787452	122795111	SMPDL3A	
8321	chr7	76600732	76603460	POMZP3	
7748	chr6	81936637	81939835	LINC02542	
7928	chr6_GL000256v2_alt	2996467	3004342	MIR4646	
4474	chr19	52068939	52097206	ZNF841	
4960	chr2	23919920	23929106	ATAD2B	
2999	chr15	67429688	67431197	IQCH-AS1	
1072	chr10	22228533	22230375	EBLN1	
3492	chr16_KI270853v1_alt	1159312	1161790	C16orf45	
7336	chr6	13460176	13487850	GFOD1	
378	chr1	19171769	19214898	UBR4	
8193	chr7	35798141	35807508		7-Sep
2513	chr13	93962600	93965078	GPC6-AS2	
1869	chr12	121434384	121441391	MIR7107	
829	chr1	66512158	66566531	SGIP1	
2978	chr15	64386460	64392938	TRIP4	
894	chr1	89993439	90027277	ZNF326	
6815	chr5	141309137	141322602	TAF7	
417	chr1	204316511	204320593	PLEKHA6	
4304	chr19	36686336	36688897	ZNF567	
3449	chr16	84481150	84505950	TLDC1	
4816	chr2	200301206	200304425	SPATS2L	
4655	chr2	12715618	12718566	TRIB2	
5377	chr20	45413982	45451195	MIR6812	
1647	chr11	67502663	67509742	PITPNM1	
2460	chr13	49987745	50085931	MIR16-1	
7798	chr6_GL000250v2_alt	3503043	3514475	AGPAT1	
9175	chrX	103672655	103690001	MORF4L2	
3720	chr17	46320120	46347827	ARL17B	
1814	chr12	110573645	110584211	PPTC7	
9362	chrX	71531040	71537743	OGT	
3545	chr17	19088293	19092097	LOC79999	
4811	chr2	199439201	199463513	SATB2	
9078	chr9	72139732	72140805	GDA	
4621	chr2	110677359	110679921	BUB1	
8643	chr8	56318686	56321122	SDR16C5	
8405	chr8	103295841	103324377	SNORD173	
7005	chr5	39136603	39139666	RICTOR	
3139	chr16	10741545	10747837	NUBP1	
5698	chr22	38695931	38697831	JOSD1	
2675	chr14	52682812	52696804	ERO1A	
213	chr1	154966035	154980233	SHC1	

59	chr1	113901436	113906678	DCLRE1B
4791	chr2	190584437	190611376	NAB1
9320	chrX	48950732	48959787	OTUD5
4192	chr19	14504553	14507316	GIPC1
3258	chr16	29993460	30009176	INO80E
1544	chr11	60840699	60855786	CCDC86
2641	chr14	39173449	39180588	PNN
3770	chr17	50689916	50699542	ANKRD40
3542	chr17	18780335	18805740	TVP23B
105	chr1	144417672	144420606	RNVU1-15
5114	chr2	58044940	58087800	VRK2
5700	chr22	38923051	38925344	APOBEC3A
8186	chr7	32753256	32762062	LINC00997
7031	chr5	53329986	53333739	FST
8473	chr8	128321999	128325763	MIR1208
1273	chr10	89643050	89646555	PANK1
8754	chr9	100089647	100100636	ERP44
8609	chr8	40171489	40174637	TCIM
7442	chr6	158630005	158644818	DYNLT1
5970	chr3	150563233	150571890	EIF2A
1140	chr10	49920626	49971271	TIMM23B
3922	chr17	78531578	78534341	DNAH17
172	chr1	150975022	150979329	CERS2
1432	chr11	13276107	13290408	ARNTL
4938	chr2	232987966	232989702	LOC101928881
3497	chr16_KI270853v1_alt	199497	217292	BFAR
837	chr1	68160457	68162043	MIR1262
1948	chr12	20807425	20844040	SLCO1B3
3663	chr17	42405897	42424131	CAVIN1
5622	chr22	20205248	20220480	LINC00896
2265	chr12	92894621	92901117	EEA1
7210	chr5_GL339449v2_alt	955028	999483	GTF2H2C
1042	chr10	132446994	132448439	C10orf91
3416	chr16	71720263	71725482	PHLPP2
2364	chr13	21019882	21062962	LATS2
7340	chr6	135476999	135502011	AHI1
3630	chr17	3714275	3726969	HASPIN
472	chr1	214534409	214553077	PTPN14
8182	chr7	30590410	30637628	GARS
4937	chr2	232879059	232901730	SNORC
8667	chr8	66454840	66457779	ADHFE1
6491	chr4	188157395	188174116	TRIML1
12	chr1	10441977	10443101	CORT
1011	chr10	12038236	12042694	UPF2

4546	chr19	8362839	8364845	ANGPTL4
644	chr1	28234582	28241050	ATP5IF1
3997	chr17_GL000258v2_alt	1574691	1581820	NSFP1
875	chr1	85296751	85299574	BCL10
3944	chr17	80544052	80548462	RPTOR
1001	chr10	119074359	119085403	EIF3A
9172	chrX	102598291	102600690	ARMCX5-GPRASP2
3480	chr16	8942395	8964751	USP7
6194	chr3	45387426	45390979	LARS2
466	chr1	212604673	212608712	ATF3
7485	chr6	2240309	2253839	GMDS-DT
5919	chr3	136748064	136754967	STAG1
6154	chr3	32684255	32688976	CNOT10
6614	chr4	74372916	74378870	EREG
4692	chr2	149559717	149589688	LOC101929231
2129	chr12	57743362	57753058	MIR6759
5003	chr2	28580662	28586877	PLB1
1708	chr11	8686418	8690502	SNORA3B
6727	chr5	117403636	117410328	LINC00992
2159	chr12	6489214	6496147	MRPL51
7488	chr6	24646774	24674924	TDP2
7705	chr6	69790578	69797509	LMBRD1
4900	chr2	219233913	219240757	GLB1L
3702	chr17	44707598	44711467	DBF4B
1217	chr10	73588430	73593170	USP54
4189	chr19	14113176	14121451	PRKACA
8894	chr9	129817762	129821211	TOR1A
3046	chr15	75636509	75641638	IMP3
7421	chr6	151762843	151766761	ESR1
4187	chr19	1406186	1432990	DAZAP1
3604	chr17	31050950	31053234	LOC646030
4018	chr17_KI270860v1_alt	53671	58194	B3GNTL1
8547	chr8	19816972	19833859	INTS10
6160	chr3	33710961	33719575	CLASP2
6317	chr4	101207792	101350518	MIR8066
9355	chrX	70251712	70254950	P2RY4
3992	chr17	930916	934639	LOC101927727
7137	chr5	79130934	79135065	BHMT
4788	chr2	190317047	190320939	HIBCH
5855	chr3	123065430	123072525	PDIA5
3628	chr17	36919483	36923737	LHX1-DT
2826	chr14_KI270847v1_alt	440955	452295	BTBD7
4661	chr2	127882637	127890856	AMMECR1L
583	chr1	236266328	236268565	ERO1B

3148	chr16	11842594	11853389	RSL1D1
4966	chr2	240578531	240588476	CAPN10-DT
8464	chr8	127087201	127088645	PCAT2
1511	chr11	46696410	46703904	ARHGAP1
4686	chr2	144126340	144133201	GTDC1
7183	chr5	95711597	95712443	RHOBTB3
1755	chr11_KI270830v1_alt	14013	17557	DUSP8
1366	chr11	112224856	112251839	PTS
8144	chr7	23179187	23209491	NUPL2
6653	chr4	83439174	83460724	HELQ
5522	chr21	34792446	34886485	RUNX1
6988	chr5	35853537	35863271	IL7R
1570	chr11	6314509	6321476	CAVIN3
2258	chr12	89945694	89949467	LINC02399
3828	chr17	63839682	63844936	SMARCD2
6925	chr5	177533762	177538528	FAM193B
3826	chr17	63770216	63789509	DDX42
4152	chr19	11131588	11137641	MIR6886
7289	chr6	121446911	121449764	GJA1
4454	chr19	48961660	48969187	FTL
7912	chr6_GL000255v2_alt	4391973	4401765	RXRB
82	chr1	11922307	11927593	KIAA2013
5166	chr2	70107669	70110689	LINC01816
8495	chr8	142724210	142727180	LOC100288181
2969	chr15	63500529	63567643	USP3
1394	chr11	121828373	121831268	MIR125B1
4464	chr19	49844096	49851465	PTOV1
2790	chr14	80209575	80212406	DIO2-AS1
4984	chr2	27031345	27049890	TMEM214
6862	chr5	154748717	154764493	LARP1
3768	chr17	50477523	50485180	RSAD1
253	chr1	15969210	15977121	ZBTB17
4983	chr2	26763589	26778087	SLC35F6
6363	chr4	123397201	123399020	SPRY1
2103	chr12	56040034	56048034	RPS26
3034	chr15	73926632	73927876	LOXL1-AS1
88	chr1	120353763	120356830	NBPF8
5871	chr3	126072040	126086645	SLC41A3
3192	chr16	20792171	20814259	REXO5
3988	chr17	8629728	8633142	MYH10
2522	chr13	98494089	98510535	STK24
8542	chr8	19208283	19210695	LOC100128993
2917	chr15	49114265	49158930	COPS2
7305	chr6	126720727	126723577	MIR588

1041	chr10	132318920	132335723	STK32C
797	chr1	58784338	58787949	LINC01135
5506	chr21	31663673	31733343	SCAF4
2716	chr14	64496162	64519734	ZBTB25
2112	chr12	56268239	56272688	COQ10A
6203	chr3	4739581	4743395	EGOT
7429	chr6	155305581	155316745	TFB1M
6891	chr5	17212250	17287152	BASP1
1327	chr11	102391526	102422139	LOC101928424
8423	chr8	115968554	115970966	TRPS1
6707	chr5	110733462	110761301	SLC25A46
8134	chr7	1956175	1967693	MAD1L1
3348	chr16	56729062	56731459	NUP93
5315	chr20	34939438	34956753	GSS
4848	chr2	203326875	203332258	ABI2
5772	chr22	50763751	50791618	RABL2B
592	chr1	2401067	2408071	PEX10
1309	chr10	97639331	97660588	PI4K2A
6513	chr4	27462639	27465679	LINC02261
8788	chr9	111794463	111797573	C9orf84
8469	chr8	127477489	127480622	CASC8
4540	chr19	750235	754416	MISP
4281	chr19	34346764	34350999	GPI
4001	chr17_GL000258v2_alt	429092	431583	LRRC37A4P
4318	chr19	38850505	38853492	HNRNPL
2779	chr14	75658240	75663842	ERG28
4059	chr18	31937545	31946086	TRAPPC8
7718	chr6	7305858	7314618	SSR1
6455	chr4	173167562	173170076	GALNT7
7082	chr5	66142430	66170501	SREK1
9180	chrX	107676207	107678093	TSC22D3
8746	chr8_KI270815v1_alt	55822	61460	ZNF251
5329	chr20	35870532	35887988	SCAND1
5755	chr22	46376617	46378750	TRMU
55	chr1	113455885	113460393	MAGI3
5170	chr2	70257185	70259012	PCYOX1
3385	chr16	679528	686346	JMJD8
3103	chr15	92809636	92812773	ASB9P1
6551	chr4	40995575	41010651	APBB2
3917	chr17	7678229	7691584	WRAP53
8237	chr7	54966178	54969235	EGFR
9358	chrX	71180874	71183331	GJB1
5075	chr2	46540099	46563997	RHOQ
6234	chr3	4975803	4979462	BHLHE40

4935	chr2	232534429	232562447	MIR5001
2800	chr14	88823460	88836981	TTC8
718	chr1	41013607	41061668	SLFNL1
2459	chr13	49927444	49943150	SPRYD7
8860	chr9	128006107	128007465	FAM102A
6759	chr5	132822194	132832507	SHROOM1
2179	chr12	68609002	68656288	SNORA70G
3812	chr17	61825229	61834390	BRIP1
2223	chr12	76507753	76562734	OSBPL8
868	chr1	8209958	8213746	LINC01714
6230	chr3	49404959	49414467	RHOA
5501	chr21	29181071	29183741	LINC00189
4299	chr19	36488529	36490724	LOC728752
501	chr1	223699963	223705154	CAPN2
3945	chr17	80617986	80622832	RPTOR
1802	chr12	109899544	109902046	TCHP
1918	chr12	13179160	13181623	EMP1
6512	chr4	2736431	2748683	TNIP2
2689	chr14	55063067	55078890	MAPK1IP1L
96	chr1	121115266	121119264	HIST2H2BA
4151	chr19	11087659	11101549	LDLR
5176	chr2	71066034	71082259	NAGK
5584	chr21	44815536	44820713	SUMO3
7887	chr6_GL000254v2_alt	4704736	4714258	RPS18
4878	chr2	210160232	210171531	KANSL1L
5195	chr2	74954688	74971582	POLE4
3015	chr15	70745003	70765421	UACA
2014	chr12	45712211	45753900	ARID2
5370	chr20	44883130	44914581	PABPC1L
3728	chr17	4694878	4707108	LOC101559451
3488	chr16	89970912	89993279	CENPBD1
1853	chr12	120239659	120264067	PXN
1907	chr12	12758368	12762182	MIR613
7911	chr6_GL000255v2_alt	4164463	4179635	BRD2
7393	chr6	149023498	149026074	LOC105378047
6620	chr4	75722868	75732666	USO1
4598	chr2	102707878	102711506	MFSD9
1434	chr11	13414104	13465395	BTBD10
4868	chr2	207488029	207489105	CREB1
8651	chr8	60677057	60760743	CHD7
7285	chr6	118930348	118939077	MCM9
4154	chr19	11373050	11376259	SWSAP1
115	chr1	145605380	145654530	GPR89A
7199	chr5	97836830	97839542	LINC02234

6564	chr4	47524632	47527393	ATP10D
5130	chr2	63988485	64020413	VPS54
4965	chr2	24044058	24048552	WDCP
267	chr1	1611925	1617154	MIB2
980	chr10	110483874	110516411	DUSP5
5667	chr22	31397564	31405742	DRG1
4644	chr2	114048469	114052151	LOC100499194
7682	chr6	53643775	53653662	KLHL31
581	chr1	235915443	235923991	LYST
2626	chr14	34980175	34987046	SRP54
9294	chrX	40733629	40736847	MED14OS
5270	chr20	11890726	11936377	BTBD3
7586	chr6	33423662	33426706	SYNGAP1
8206	chr7	42659424	42662789	LINC01448
6011	chr3	160675258	160681190	ARL14
2088	chr12	53495286	53510114	TARBP2
7464	chr6	166979487	167039923	FGFR1OP
4685	chr2	144081948	144085508	GTDC1
1337	chr11	105028463	105048625	CASP1
5441	chr20	55537004	55541333	LINC01441
5402	chr20	49811063	49828672	SLC9A8
2777	chr14	75164560	75178494	TMED10
7510	chr6	26531726	26533525	HMGN4
3439	chr16	787065	789733	RPUSD1
2344	chr13	113445816	113455879	ADPRHL1
7826	chr6_GL000252v2_alt	1311734	1318509	ZNRD1ASP
4393	chr19	45374673	45377543	ERCC2
2128	chr12	57589974	57597197	PIP4K2C
3735	chr17	47431904	47441928	MRPL45P2
5365	chr20	44186512	44189091	JPH2
4122	chr18	75069544	75072537	ZADH2
3908	chr17	76096316	76105059	MIR6868
5508	chr21	32377759	32395955	URB1
3830	chr17	64144034	64148201	SNORD104
7674	chr6	52253885	52286099	MCM3
5804	chr3	107237379	107242024	LINC00882
530	chr1	226981421	226989951	COQ8A
5017	chr2	31996388	32012960	MEMO1
2145	chr12	62933707	62936535	PPM1H
487	chr1	22024857	22028088	LINC01635
7182	chr5	95646133	95648487	RFESD
8901	chr9	130692452	130696323	EXOSC2
2161	chr12	6534437	6540117	GAPDH
4873	chr2	207744647	207751947	MIR4775

3952	chr17	8119036	8120574	ALOXE3
5207	chr2	84971045	84988215	KCMF1
5335	chr20	36571522	36577656	TGIF2
2470	chr13	52616443	52642238	HNRNPA1L2
847	chr1	7547882	7551191	CAMTA1
7463	chr6	166584564	166586865	RPS6KA2
2222	chr12	76291788	76293390	LINC02407
6826	chr5	142341093	142347781	SPRY4-AS1
5080	chr2	46905958	46918555	MCFD2
4168	chr19	12680872	12682508	DHPS
7410	chr6	150025121	150026330	RAET1L
8822	chr9	121121354	121124917	CNTRL
5261	chr2	99502995	99506035	REV1
1640	chr11	67315901	67319233	LOC100130987
2773	chr14	74881191	74909300	DLST
372	chr1	186677126	186685863	PACERR
5840	chr3	119495366	119528362	TIMMDC1
996	chr10	117371812	117377315	PDZD8
6242	chr3	5015332	5028418	BHLHE40-AS1
6557	chr4	41932088	41953753	TMEM33
2353	chr13	114312119	114332341	CHAMP1
7057	chr5	59789065	59792602	PDE4D
8083	chr7	151061384	151063536	SLC4A2
5450	chr20	58649947	58675638	STX16
1307	chr10	97425286	97472795	ZDHHC16
8238	chr7	54984421	55003281	EGFR
8428	chr8	117539704	117547645	MED30
1674	chr11	73862278	73882174	PAAF1
1795	chr12	108689129	108730843	CORO1C
9185	chrX	108414747	108531451	COL4A5
3361	chr16	58248895	58255934	CCDC113
2579	chr14	21475534	21479280	TOX4
5941	chr3	142887979	142888675	PCOLCE2
3984	chr17	8287132	8291011	RANGRF
2327	chr13	110498924	110504751	COL4A2-AS1
2160	chr12	65167220	65245750	LEMD3
4575	chr19_KI270938v1_alt	428741	446233	LENG8
4721	chr2	161159074	161232504	TANK
5748	chr22	45600532	45613491	LINC01589
5604	chr21_KI270872v1_alt	1507	5133	FTCD-AS1
3507	chr16_KI270855v1_alt	43206	46955	PAM16
4886	chr2	216108049	216225464	LINC01963
3722	chr17	46371119	46377653	NSFP1
9118	chr9	91191352	91198779	LINC00484

9144	chr9	95864505	96015746	LINC00476
9230	chrX	13486439	13488130	EGFL6
9106	chr9	85739923	85744419	AGTPBP1
2565	chr14	105313231	105318878	BRF1
5398	chr20	49239185	49254764	DDX27
5995	chr3	156672661	156728856	TIPARP
5865	chr3	124836058	124839420	ITGB5
6611	chr4	74094308	74099157	CXCL2
1129	chr10	43435825	43438039	ZNF487
5018	chr2	32034929	32042501	DPY30
8068	chr7	14766710	14768726	DGKB
3456	chr16	86386440	86389969	LINC00917
930	chr1	98025519	98028968	MIR2682
5440	chr20	53827364	53830739	SUMO1P1
6594	chr4	667519	685609	MYL5
7227	chr6	101300897	101304203	GRIK2
5917	chr3	136249957	136251815	PCCB
4857	chr2	206076206	206088447	INO80D
5789	chr3	10113955	10123535	BRK1
5053	chr2	39112702	39126887	SOS1
2793	chr14	81431664	81438421	STON2
4720	chr2	160490783	160495513	RBMS1
4034	chr18	12944248	12956032	SEH1L
8789	chr9	111866056	111868473	UGCG
5290	chr20	25383876	25392498	ABHD12
1109	chr10	3466666	3469678	LOC101927880
5885	chr3	128679386	128682509	RPN1
158	chr1	149879216	149889423	HIST2H2BE
5311	chr20	34675255	34677990	PIGU
7621	chr6	37432588	37438467	CMTR1
8555	chr8	22597772	22599156	C8orf58
5647	chr22	29256655	29303925	RHBDD3
3896	chr17	75455039	75457482	TMEM94
1266	chr10	88879126	88899145	STAMBPL1
2787	chr14	77703079	77737963	SLIRP
1851	chr12	120193985	120196049	GCN1
2507	chr13	80149693	80154288	LINC01080
8044	chr7	134985395	135002863	AGBL3
9211	chrX	123845263	123848103	XIAP
4549	chr19	8829447	8833504	ZNF558
2865	chr15	40323702	40329627	INAFM2
7952	chr7	101243447	101245061	FIS1
8010	chr7	12422933	12426000	VWDE
6370	chr4	127632064	127635992	INTU

4747	chr2	173071698	173125857	MAP3K20
2767	chr14	73850747	73860125	PTGR2
6596	chr4	6701327	6711122	MRFAP1L1
8175	chr7	29630960	29633359	MIR550A3
8127	chr7	17695318	17698301	LOC101927630
8857	chr9	127801511	127805472	FPGS
8329	chr7	77534676	77576983	PTPN12
1321	chr11	100732572	100734600	LOC100128386
6452	chr4	169658395	169671951	CLCN3
7343	chr6	13610774	13633121	NOL7
6413	chr4	145937539	145938883	ZNF827
3974	chr17	82204410	82207864	CCDC57
5480	chr20	63977191	63980395	SAMD10
7400	chr6	149617721	149619888	RPS18P9
1097	chr10	30433225	30436175	MAP3K8
2091	chr12	53971123	53979256	HOTAIR
191	chr1	152043135	152050975	S100A11
46	chr1	111420648	111461111	ATP5PB
5094	chr2	53403053	53406640	CHAC2
770	chr1	51897213	51899123	NRDC
5301	chr20	31736886	31741807	TPX2
7665	chr6	44386272	44391356	CDC5L
2248	chr12	88124163	88189162	TMTC3
5814	chr3	111851179	111974669	PHLDB2
5497	chr21	28987609	28994276	LTN1
6773	chr5	134647087	134652907	SEC24A
6542	chr4	39546228	39553253	UGDH-AS1
3771	chr17	50718062	50736479	LUC7L3
7288	chr6	121329723	121336333	TBC1D32
2266	chr12	92920786	92931440	EEA1
8870	chr9	128455350	128459273	ODF2
7681	chr6	53614782	53617530	LINC01564
401	chr1	200737991	200752729	CAMSAP2
9287	chrX	308861	310827	GTPBP6
2270	chr12	93554614	93585331	SOCS2
2491	chr13	74242745	74245676	LINC00402
3297	chr16	31876400	31900990	ZNF267
7413	chr6	150296150	150299876	IYD
7232	chr6	10392833	10421592	TFAP2A-AS2
2120	chr12	569202	573147	LOC105369595
7085	chr5	6711202	6755294	TENT4A
5191	chr2	74524262	74539367	AUP1
7423	chr6	152488686	152492745	SYNE1-AS1
8369	chr7	97141030	97148601	SDHAF3

7452	chr6	160989516	160999605	MAP3K4
7397	chr6	149531145	149546857	PPIL4
5551	chr21	39181356	39185860	PSMG1
1746	chr11	9611916	9616289	WEE1
5447	chr20	57663451	57719979	PMEPA1
3035	chr15	73988952	73992869	STOML1
6276	chr3	61560181	61565095	PTPRG
7541	chr6	28266139	28269219	ZSCAN26
2197	chr12	70227637	70366332	CNOT2
4728	chr2	164833207	164843025	LOC101929633
5532	chr21	36040972	36051991	SETD4
324	chr1	172530755	172562911	SUCO
3516	chr17	15642060	15652662	CDRT1
3789	chr17	56871163	56873018	MTVR2
7455	chr6	163391274	163393164	CAHM
2761	chr14	73560419	73563398	HEATR4
5550	chr21	39019189	39022475	LINC01700
1263	chr10	87844786	87847457	KLLN
490	chr1	221034160	221038007	HLX-AS1
2669	chr14	50942757	50945888	PYGL
3950	chr17	80889176	80901273	LOC400627
8763	chr9	105556573	105569090	FKTN
8511	chr8	144071649	144110798	CYC1
6382	chr4	131608365	131611759	SNHG27
7175	chr5	92018603	92020980	ARRDC3
4690	chr2	149410117	149412785	LYPD6
1028	chr10	125716733	125759933	EDRF1-AS1
2813	chr14	96359245	96362937	ATG2B
6087	chr3	190052386	190055236	P3H2-AS1
7858	chr6_GL000253v2_alt	2090076	2093769	HCG20
8864	chr9	128192020	128195340	CIZ1
1189	chr10	69316694	69320772	HK1
928	chr1	964669	967464	PLEKHN1
8483	chr8	133673658	133675792	LOC105375773
229	chr1	156047637	156055960	UBQLN4
4126	chr18	79673827	79681342	LOC284241
3783	chr17	54900545	54930856	TOM1L1
5136	chr2	64829669	64833000	LINC01800
3624	chr17	35807522	35858954	TAF15
2149	chr12	63841408	63884201	SRGAP1
6155	chr3	33033752	33037062	TMPPE
2489	chr13	73496090	73500282	LINC00392
4371	chr19	43458242	43466490	LYPD3
5346	chr20	37978737	38101097	RPRD1B

2254	chr12	89568480	89586628	POC1B-GALNT4
7905	chr6_GL000255v2_alt	2905063	2909822	BAG6
7490	chr6	24742107	24745016	C6orf62
5952	chr3	149057296	149087584	HLTF-AS1
5816	chr3	111976866	111986933	ABHD10
4020	chr17_KI270861v1_alt	95033	116330	PRPF8
169	chr1	150683449	150698269	GOLPH3L
6266	chr3	56575060	56580213	CCDC66
7033	chr5	53397105	53398771	FST
8023	chr7	129050158	129058181	TNPO3
5293	chr20	2648543	2653831	NOP56
7932	chr6_GL000256v2_alt	4717940	4724184	RPS18
4502	chr19	5702259	5721310	LONP1
2802	chr14	90395314	90409848	CALM1
3939	chr17	80133435	80152076	EIF4A3
1278	chr10	91405652	91427237	HECTD2
2065	chr12	5138101	5141346	KCNA5
5104	chr2	55043088	55053609	RTN4
9156	chr9	99211913	99238836	SEC61B
701	chr1	37854906	37867208	MTF1
1798	chr12	108849902	108855561	SSH1
1536	chr11	58850196	58851665	GLYATL2
3931	chr17	7909215	7918463	RNF227
550	chr1	230971320	230999768	ARV1
543	chr1	229502468	229510318	NUP133
1873	chr12	121887058	121896658	PSMD9
3306	chr16	380473	384609	LOC100134368
3120	chr15	99564132	99588315	MEF2A
4418	chr19	46711951	46715760	PRKD2
6289	chr3	88119468	88121778	C3orf38
155	chr1	149636594	149638241	LINC00869
1124	chr10	3849186	3854300	LOC105376365
1271	chr10	89391105	89395359	IFIT1
8576	chr8	28090856	28181361	ELP3
1094	chr10	2948118	2953056	PFKP
5544	chr21	37363359	37366399	DYRK1A
5762	chr22	47327487	47332168	LOC339685
8557	chr8	229825	243153	ZNF596
3963	chr17	81661685	81715258	HGS
1023	chr10	12447623	12449626	CAMK1D
8229	chr7	48046362	48050180	C7orf57
1530	chr11	57521589	57531639	TIMM10
7335	chr6	134589209	134591020	LOC101928304
8581	chr8	29047899	29067820	HMBOX1

3511	chr17	13345433	13348569	LINC02093
519	chr1	225789131	225796306	SRP9
6175	chr3	41880072	41881997	ULK4
885	chr1	8861361	8881696	MIR6728
6118	chr3	196625671	196633399	LINC01063
6634	chr4	78776385	78796452	BMP2K
2914	chr15	45581639	45598954	BLOC1S6
9074	chr9	70216103	70217828	MAMDC2-AS1
8676	chr8	67314605	67347735	ARFGEF1
8361	chr7	94579987	94581726	CASD1
6863	chr5	154856548	154868047	CNOT8
3716	chr17	45258373	45270045	SPATA32
8839	chr9	125237243	125244552	HSPA5
8407	chr8	104745117	104746855	LRP12
6166	chr3	38129028	38140498	ACAA1
3360	chr16	58123273	58130278	CFAP20
7212	chr5_GL949742v1_alt	163723	166143	LINC02109
8755	chr9	100338086	100353715	TEX10
3009	chr15	69159457	69162731	GLCE
5242	chr2	96263432	96268434	TMEM127
6350	chr4	119207919	119255769	LOC101929762
2060	chr12	51016440	51026156	SLC11A2
150	chr1	149076513	149107519	NBPF9
4131	chr18	9422193	9425005	RALBP1
4681	chr2	138485832	138508676	SPOPL
3110	chr15	96355661	96358557	NR2F2
4460	chr19	49486662	49489253	RPL13A
7251	chr6	108559549	108574372	FOXO3
8401	chr8	102653929	102659396	KLF10
4742	chr2	172101151	172103952	DLX2
3734	chr17	47318901	47327486	THCAT158
5858	chr3	123609430	123614881	MYLK
6335	chr4	110158433	110164381	ELOVL6
3169	chr16	15575364	15579420	MIR6506
1502	chr11	43358286	43372539	TTC17
339	chr1	1773032	1781510	NADK
329	chr1	173836060	173840644	DARS2
9050	chr9	61851419	61859937	FAM27C
7207	chr5_GL339449v2_alt	341609	386007	GTF2H2C
6092	chr3	191140845	191142911	OSTN
741	chr1	44772388	44773985	RPS8
3288	chr16	31141162	31143391	PRSS8
7373	chr6	143429755	143474129	PEX3
58	chr1	113807216	113813361	RSBN1

8408	chr8	105089039	105090234	ZFPM2
6744	chr5	131153912	131166290	HINT1
7662	chr6	43797638	43800976	VEGFA
5283	chr20	2099285	2110136	STK35
7770	chr6	89033905	89036698	PNRC1
4438	chr19	48098519	48111995	PLA2G4C
6975	chr5	31525187	31545547	C5orf22
6922	chr5	177300087	177309343	PRELID1
2101	chr12	55971636	55985096	RAB5B
9055	chr9	63335769	63340349	AQP7P1
6809	chr5	140659385	140678708	WDR55
1154	chr10	5807812	5816149	GDI2
5177	chr2	71128468	71132747	MPHOSPH10
5354	chr20	41003792	41006694	TOP1
7026	chr5	51381564	51385254	LOC642366
4521	chr19	58543298	58555703	MIR6807
7105	chr5	72387186	72388804	PTCD2
872	chr1	84603131	84605203	LINC01461
6700	chr5	108725328	108730546	LINC01023
7095	chr5	70024469	70028497	SERF1B
218	chr1	155221402	155229429	GBAP1
1345	chr11	107447851	107459268	CWF19L2
2893	chr15	43092556	43108032	UBR1
53	chr1	112922249	112937057	AKR7A2P1
1092	chr10	29408724	29431830	SVIL-AS1
936	chr10	100210352	100234647	CHUK
1557	chr11	62122497	62125651	INCENP
8819	chr9	120834078	120835134	PSMD5
805	chr1	61715905	61727096	TM2D1
2952	chr15	58922283	58935607	SLTM
1941	chr12	1757190	1769927	LRTM2
5211	chr2	85559754	85563409	GGCX
6857	chr5	150697474	150702890	RBM22
4240	chr19	19201175	19204631	NR2C2AP
1885	chr12	122973546	122987504	ARL6IP4
4497	chr19	5602420	5659004	SAFB
7981	chr7	107889461	107955816	DLD
549	chr1	2309139	2312354	LOC100129534
8429	chr8	117859971	118108860	SNORD168
2175	chr12	6722358	6730980	COPS7A
3287	chr16	31137722	31139302	PRSS8
5111	chr2	55779247	55782091	PNPT1
1604	chr11	65456234	65458402	MIR612
3091	chr15	90227607	90230006	CIB1

8907	chr9	131967796	132080641	MED27
1649	chr11	67629561	67630686	NUDT8
7606	chr6	36194820	36212587	BRPF3
2887	chr15	42268075	42277318	TMEM87A
2268	chr12	93395425	93446081	UBE2N
3669	chr17	42603538	42616336	RETREG3
4253	chr19	2323552	2333445	LSM7
4719	chr2	160400829	160408252	MIR4785
2598	chr14	24212536	24245084	NEDD8
703	chr1	37986307	37990911	SF3A3
3888	chr17	75119907	75128386	NT5C
5071	chr2	45962078	45966915	PRKCE
8636	chr8	52675834	52721728	RB1CC1
8486	chr8	140622302	140647470	AGO2
6158	chr3	33299451	33366672	FBXL2
1769	chr12	102110559	102124817	NUP37
8898	chr9	130575310	130585584	FUBP3
8497	chr8	143217736	143220891	GPIHBP1
6593	chr4	6672674	6684528	LOC93622
6489	chr4	188002038	188003977	ZFP42
7337	chr6	134908142	134909912	ALDH8A1
355	chr1	180953044	180979431	KIAA1614-AS1
2143	chr12	62600155	62636796	MIRLET7I
6850	chr5	149547741	149555405	CSNK1A1
1651	chr11	68009012	68019760	ALDH3B1
252	chr1	15957108	15961660	ZBTB17
7929	chr6_GL000256v2_alt	3243985	3263599	MIR1236
502	chr1	223705726	223708753	CAPN2
7833	chr6_GL000252v2_alt	2163413	2168665	GTF2H4
1884	chr12	122945483	122949497	ABCB9
960	chr10	103582789	103589883	NEURL1-AS1
4082	chr18	46916968	46918618	KATNAL2
9102	chr9	83622252	83711146	LOC105376114
5015	chr2	30664153	30666609	CAPN13
1446	chr11	18317599	18337865	GTF2H1
7166	chr5	88229863	88304545	TMEM161B
1142	chr10	50739270	50742433	ASAH2B
7255	chr6	109368997	109385131	CD164
5174	chr2	70595064	70598125	TGFA
2390	chr13	31044108	31047260	TEX26
1712	chr11	88534713	88537218	GRM5-AS1
6813	chr5	140725145	140727114	VTRNA1-3
7780	chr6	9519219	9521872	HULC
5633	chr22	23911701	23915290	MIF-AS1

6019	chr3	167660274	167733853	PDCD10
7584	chr6	33288641	33295283	MIR6834
8808	chr9	114882812	114884624	TNFSF8
7975	chr7	106655051	106661707	CCDC71L
7961	chr7	103144971	103150965	NAPEPLD
330	chr1	174157339	174170102	RABGAP1L
6005	chr3	158790809	158816456	MFSD1
5717	chr22	41430379	41446764	TOB2
6904	chr5	172919576	172938005	RPL26L1
109	chr1	144559565	144563099	RNVU1-11
6869	chr5	159148719	159211779	RNF145
7699	chr6	63634562	63667014	PHF3
3574	chr17	28323518	28359668	TNFAIP1
6791	chr5	138208848	138214238	CDC23
1571	chr11	63566595	63569300	HRASLS2
1534	chr11	57760259	57798174	CTNND1
1993	chr12	32050043	32053197	BICD1
2512	chr13	90811458	90849595	LINC00410
3181	chr16	18793796	18804042	ARL6IP1
3474	chr16	8844773	8862247	LOC100130283
981	chr10	110772765	110776809	PDCD4-AS1
5556	chr21	41235153	41275615	FAM3B
2246	chr12	87817565	87822812	MKRN9P
5193	chr2	74850317	74853484	HK2
1654	chr11	68898971	68904874	MRPL21
5723	chr22	42067213	42072302	NAGA
1716	chr11	8958369	8968606	TMEM9B-AS1
7781	chr6	96508787	96553239	UFL1
8728	chr8	95024144	95027723	NDUFAF6
2836	chr15	22662773	22666798	WHAMMP3
7809	chr6_GL000251v2_alt	248468	249615	LINC00533
527	chr1	226407153	226409660	PARP1
2605	chr14	28675801	28678023	FOXG1-AS1
6450	chr4	169591226	169613702	NEK1
6060	chr3	184313835	184321318	EIF4G1
7771	chr6	89079028	89081996	PNRC1
5931	chr3	141365726	141453517	ZBTB38
4947	chr2	235025353	235050386	SH3BP4
2894	chr15	43324464	43333079	ADAL
2440	chr13	46767186	46798574	ESD
6834	chr5	143456371	143458438	NR3C1
8917	chr9	133392312	133419982	ADAMTS13
5709	chr22	40632650	40638338	MKL1
2861	chr15	39580383	39595414	THBS1

8221	chr7	45105784	45114410	TBRG4
3054	chr15	78147307	78161746	IDH3A
7589	chr6	33769047	33793697	LEMD2
8267	chr7	65956247	65965772	GUSB
6825	chr5	142325814	142333303	SPRY4-AS1
9176	chrX	104148089	104164150	SLC25A53
6309	chr3_KI270895v1_alt	151223	159834	LOC105374297
8706	chr8	87550887	87593748	DCAF4L2
2167	chr12	65655491	65658325	LOC100507065
6237	chr3	49893841	49902651	MST1R
4778	chr2	183123197	183125999	NUP35
4503	chr19	571258	574476	BSG
8510	chr8	144059855	144062086	OPLAH
2153	chr12	64449134	64454353	TBK1
8597	chr8	37760487	37769824	PLPBP
8620	chr8	42851889	42857342	THAP1
3629	chr17	36947564	37021812	AATF
6205	chr3	47472996	47476721	SCAP
2033	chr12	48702944	48719617	CCNT1
8399	chr8	101162896	101215292	ZNF706
2873	chr15	40845494	40847852	SPINT1-AS1
7377	chr6	144215819	144217461	STX11
7536	chr6	2809123	2811312	SERPINB1
390	chr1	197145360	197147974	ASPM
4164	chr19	11924444	11929068	ZNF700
5246	chr2	96591949	96592936	KANSL3
5857	chr3	123569799	123603141	MYLK-AS1
1178	chr10	68082355	68084756	HERC4
103	chr1	143900562	143905828	HIST2H3PS2
1328	chr11	102599199	102601064	MMP20
4431	chr19	47347925	47363320	DHX34
6316	chr4	10075339	10119873	MIR3138
2111	chr12	56260997	56263096	ANKRD52
1861	chr12	120572500	120582497	POP5
7297	chr6	12482638	12487790	LINC02530
3932	chr17	7923165	7938028	KCNAB3
8090	chr7	151620948	151633069	PRKAG2
9209	chrX	123763875	123766708	THOC2
2280	chr12	95213995	95235131	VEZT
6688	chr5	10266763	10269016	CCT5
8183	chr7	32161953	32164673	PDE1C
3441	chr16	81313687	81335716	GAN
3075	chr15	84709455	84719440	SEC11A
116	chr1	145812404	145827576	POLR3C

6388	chr4	139013472	139026919	NOCT
2524	chr13	99082063	99090627	DOCK9
7185	chr5	95812839	95816042	GLRX
203	chr1	153917582	153925491	GATAD2B
2464	chr13	51524014	51525811	MIR4703
5065	chr2	43901925	43997197	LRPPRC
4993	chr2	27489093	27490714	IFT172
1034	chr10	126904809	126906496	DOCK1
6540	chr4	3946528	3957952	FAM86EP
4097	chr18	53795030	53797205	LINC01917
8363	chr7	94822261	94825020	PPP1R9A
2854	chr15	34436049	34439102	GOLGA8A
7345	chr6	136416867	136550213	MAP7
5418	chr20	50734571	50749720	PARD6B
8761	chr9	104989811	104992248	ABCA1
5127	chr2	62577194	62580274	TMEM17
4504	chr19	57275963	57290477	ZNF460
5518	chr21	33946574	33951292	LINC00649
6325	chr4	105450707	105474848	PPA2
6270	chr3	57590310	57603438	ARF4
5566	chr21	42891777	42925279	NDUFV3
1791	chr12	107683643	107705858	PWP1
1245	chr10	79326569	79360029	PPIF
948	chr10	101805389	101819387	OGA
8252	chr7	6042719	6060432	EIF2AK1
2480	chr13	73088681	73092004	KLF5
6281	chr3	71502711	71505905	MIR1284
7655	chr6	43538099	43544381	POLR1C
8804	chr9	114503831	114506805	WHRN
6151	chr3	31529860	31535912	STT3B
8593	chr8	33497106	33505039	TTI2
363	chr1	183560422	183567854	NCF2
4186	chr19	14001722	14008769	RFX1
2194	chr12	69738703	69753423	RAB3IP
7333	chr6	134420902	134424101	LINC01010
8902	chr9	130833574	130873031	ABL1
2990	chr15	65756542	65795121	DENND4A
3163	chr16	14910703	14915044	PKD1P3-NPIPA1
6484	chr4	186710130	186729521	FAT1
8356	chr7	92824579	92832839	CDK6
8071	chr7	148986352	148988558	GHET1
4883	chr2	214806699	214808651	BARD1
8584	chr8	29553863	29557530	LINC00589
743	chr1	44799348	44804846	PLK3

7898	chr6_GL000255v2_alt	2003549	2005989	IER3
4781	chr2	186543632	186545691	ITGAV
7152	chr5	82257940	82260315	RPS23
1013	chr10	12190502	12199735	CDC123
6215	chr3	48445360	48451264	ATRIP
1198	chr10	71817663	71851977	PSAP
7066	chr5	6157762	6161252	LINC02145
3864	chr17	71411185	71414856	CASC17
557	chr1	232949273	232952283	NTPCR
2871	chr15	40763611	40765012	GCHFR
443	chr1	20786399	20789264	HP1BP3
8652	chr8	61514625	61579969	ASPH
2420	chr13	41283465	41285849	MTRF1
7441	chr6	158558603	158588593	MIR7161
9226	chrX	132022719	132025312	STK26
5219	chr2	86702942	86705690	RMND5A
3346	chr16	56515747	56521215	BBS2
6652	chr4	83280256	83286109	COQ2
2980	chr15	64528857	64530491	ZNF609
2859	chr15	37097707	37111472	MEIS2
9069	chr9	68409247	68416368	PGM5-AS1
418	chr1	204377462	204412845	PPP1R15B
7390	chr6	14795748	14798393	JARID2
4840	chr2	20219326	20226678	SDC1
2794	chr14	81480606	81530761	SEL1L
808	chr1	62681145	62692247	DOCK7
7806	chr6_GL000251v2_alt	2216574	2223845	FLOT1
5536	chr21	36697830	36701019	SIM2
4297	chr19	36135783	36143787	CAPNS1
5848	chr3	121536798	121547466	POLQ
6549	chr4	40622180	40631974	RBM47
7918	chr6_GL000256v2_alt	1816529	1818332	LINC02569
6914	chr5	174158908	174161016	NSG2
47	chr1	111737334	111740634	INKA2
5676	chr22	32694582	32696729	TIMP3
6193	chr3	45166420	45168898	CDCP1
5098	chr2	54111693	54124332	ACYP2
220	chr1	155260146	155274676	SCAMP3
3751	chr17	48906353	48923612	LOC105371814
5677	chr22	35397182	35409145	MCM5
3724	chr17	46577354	46581736	ARL17B
2140	chr12	6197954	6223003	CD9
7773	chr6	89660326	89663893	LYRM2
5786	chr3	100353720	100357805	NIT2

3572	chr17	27332140	27355065	WSB1
7331	chr6	134166093	134176546	SGK1
7937	chr7	100087502	100100574	MIR106B
1118	chr10	3772531	3788095	KLF6
2352	chr13	114244684	114303454	MIR4502
8837	chr9	125162976	125192267	PPP6C
9349	chrX	65668070	65702195	MSN
4904	chr2	219570605	219571567	OBSL1
1929	chr12	133128932	133140110	ZNF10
7506	chr6	2628366	2635145	LINC02521
941	chr10	100974751	100998047	MRPL43
6864	chr5	154939624	154946830	MRPL22
2910	chr15	45198119	45199281	SHF
332	chr1	17430523	17444549	RCC2
3763	chr17	50197780	50200462	COL1A1
6440	chr4	165873670	165875738	TLL1
4594	chr2	101250716	101258959	CNOT11
5915	chr3	13600148	13602369	SNORA93
2572	chr14	20612682	20615714	RNASE12
7371	chr6	143058706	143071516	AIG1
2564	chr14	105091433	105092542	LINC02298
5313	chr20	34816747	34828764	NCOA6
5054	chr2	39227358	39230494	CDKL4
3665	chr17	42517894	42526586	MIR5010
4897	chr2	218891480	218897555	WNT10A
4160	chr19	11553904	11561486	ELOF1
1122	chr10	3823857	3826526	LOC105376365
7130	chr5	78351567	78368497	SCAMP1
6021	chr3	169452017	169456534	LOC105374205
8567	chr8	25453600	25458051	KCTD9
8287	chr7	72924306	72936745	NSUN5P2
5845	chr3	120342100	120351231	LRRC58
4332	chr19	40346121	40351512	C19orf47
8418	chr8	109538138	109545288	EBAG9
3854	chr17	68376873	68379219	PRKAR1A
1373	chr11	117143214	117147414	PAFAH1B2
4952	chr2	237522071	237523577	MIR6811
7906	chr6_GL000255v2_alt	2954522	2960557	MIR4646
356	chr1	181018101	181022987	STX6
3808	chr17	60081166	60084185	LOC105371849
8904	chr9	130961773	130964046	FIBCD1
4899	chr2	219176624	219184891	RETREG2
7384	chr6	145848220	145852563	LOC100507557
6501	chr4	24311134	24378276	PPARGC1A

8160	chr7	27094409	27104382	HOTAIRM1
5809	chr3	108747469	108750186	RETNLB
2273	chr12	93783923	93793741	LOC101928731
1467	chr11	27933654	27936348	MIR610
8243	chr7	5553937	5558283	ACTB
6679	chr4	992126	994252	SLC26A1
8162	chr7	27110570	27117369	HOXA3
231	chr1	15616057	15625922	DDI2
4874	chr2	207768672	207770546	FZD5
7913	chr6_GL000255v2_alt	4462630	4472873	RPS18
2257	chr12	89755772	89758199	ATP2B1-AS1
198	chr1	153632507	153636377	S100A13
6267	chr3	57226167	57233862	APPL1
1517	chr11	47232282	47261215	NR1H3
1035	chr10	130106981	130112052	C10orf143
6459	chr4	17576629	17580513	LAP3
210	chr1	154403360	154438446	IL6R-AS1
6675	chr4	98649572	98656175	TSPAN5
7742	chr6	79537933	79555263	LCA5
4413	chr19	46130825	46135072	IGFL3
2950	chr15	58744616	58751707	ADAM10
5824	chr3	113511768	113516928	SPICE1
3924	chr17	78733994	78737711	CYTH1
2892	chr15	42919016	42920785	TTBK2
9171	chrX	101928888	101932763	ZMAT1
574	chr1	235128429	235163032	RBM34
1120	chr10	3803679	3808179	KLF6
6819	chr5	141680162	141682195	ARAP3
6366	chr4	124903166	124905178	ANKRD50
2688	chr14	55018541	55034661	WDHD1
7064	chr5	61329847	61375335	ZSWIM6
1056	chr10	14959673	14962324	MEIG1
5117	chr2	60935507	60947751	REL
7766	chr6	87486920	87489786	SLC35A1
3801	chr17	58691409	58696266	RAD51C
7995	chr7	116423295	116428071	CAV2
3866	chr17	71437926	71442405	CASC17
6315	chr4	100347366	100349828	SNORA101A
4370	chr19	43438284	43439682	TEX101
6056	chr3	184154788	184159083	DVL3
8959	chr9	19229859	19234514	DENND4C
3970	chr17	81922503	81928174	MAFG
4148	chr19	10648961	10676995	ILF3
1459	chr11	2177846	2182249	MIR4686

4305	chr19	3669631	3672062	PIP5K1C
3180	chr16	18778691	18791174	RPS15A
8164	chr7	27179721	27188498	HOXA11
2154	chr12	6450357	6455443	TAPBPL
7245	chr6	107451240	107460787	PDSS2
22	chr1	108638428	108639430	HENMT1
8948	chr9	137524965	137526305	PNPLA7
616	chr1	248818255	248830024	SH3BP5L
1173	chr10	6721495	6724113	LINP1
1312	chr10	97735571	97767487	ZFYVE27
3218	chr16	2469167	2475278	NTN3
8481	chr8	133290062	133299246	NDRG1
1750	chr11	97965496	97968463	CNTN5
2166	chr12	65647528	65651025	LOC100507065
3823	chr17	63548206	63566286	DCAF7
6568	chr4	48327230	48335446	SLAIN2
5681	chr22	36416651	36418234	MYH9
6984	chr5	33440973	33494992	TARS
6274	chr3	57804317	57809129	SLMAP
3470	chr16	87855910	87870904	SLC7A5
4134	chr18	9657086	9661403	PPP4R1-AS1
2965	chr15	63143910	63158845	RPS27L
1546	chr11	61155164	61162805	VPS37C
4320	chr19	39111565	39113125	PAK4
8254	chr7	6157679	6166912	USP42
1822	chr12	111929278	111941043	ADAM1A
878	chr1	85579913	85586502	CYR61
2013	chr12	45231309	45293958	ANO6
7364	chr6	140812731	140898274	MIR4465
178	chr1	151183339	151215806	PIP5K1A
6790	chr5	138153569	138180071	BRD8
1149	chr10	5315634	5318568	LINC02561
2214	chr12	75659070	75662966	KRR1
6559	chr4	4232360	4250313	TMEM128
437	chr1	20727257	20734864	SH2D5
7740	chr6	78976977	79080649	PHIP
7121	chr5	75335844	75367981	HMGCR
1462	chr11	268871	270824	NLRP6
2115	chr12	56446261	56450931	TIMELESS
8118	chr7	158814109	158832957	ESYT2
8720	chr8	93914262	93916832	MIR378D2
5930	chr3	141306847	141313184	ZBTB38
1110	chr10	35194577	35197598	CREM
5570	chr21	43210059	43212541	CRYAA2

74	chr1	117146490	117149131	TRIM45
6272	chr3	57750008	57753860	SLMAP
3473	chr16	88236859	88249821	LINC02182
4693	chr2	150164838	150167403	LINC01817
4838	chr2	20183421	20187693	SDC1
5830	chr3	115238754	115249090	ZBTB20
44	chr1	111130809	111159102	DRAM2
7827	chr6_GL000252v2_alt	1811574	1824638	GNL1
7545	chr6	28347103	28362294	ZSCAN31
5743	chr22	44726685	44729145	ARHGAP8
3314	chr16	4341348	4347572	PAM16
303	chr1	168177480	168185737	TIPRL
2984	chr15	65294966	65297218	RNU5A-1
4003	chr17_GL383566v1_alt	45043	47010	LOC105371907
4194	chr19	14688228	14694233	ZNF333
7361	chr6	139977051	139979348	LOC100507477
1625	chr11	66342016	66346425	BRMS1
3872	chr17	7259558	7264307	CLDN7
3102	chr15	91851924	91857246	SLCO3A1
9121	chr9	92146185	92149171	LINC00475
8851	chr9	127421897	127441895	ZNF79
7458	chr6	16491523	16774482	ATXN1
8377	chr7	99378879	99412586	PDAP1
5070	chr2	45645851	45651198	PRKCE
8043	chr7	134641614	134648098	BPGM
3835	chr17	64747712	64751710	MIR6080
6931	chr5	178906664	178908494	ZFP2
2488	chr13	73464940	73467532	LINC00392
8505	chr8	143896876	143942492	PLEC
3836	chr17	64914607	64920685	LRRC37A3
4799	chr2	195561385	195563156	SLC39A10
6981	chr5	32584240	32600823	SUB1
799	chr1	58847593	58850586	LINC01135
9134	chr9	94138899	94141110	MIRLET7A1
4403	chr19	45610440	45615303	GPR4
3901	chr17	75792939	75815546	UNK
4316	chr19	38655578	38729119	ACTN4
8141	chr7	22807405	22825177	TOMM7
1268	chr10	89276924	89279806	IFIT2
8075	chr7	149223223	149228658	ZNF212
7164	chr5	87266062	87300699	RASA1
239	chr1	156586243	156604718	NAXE
6250	chr3	51376788	51400577	RBM15B
5873	chr3	126215956	126217954	ALDH1L1

3755	chr17	49191038	49193694	GNGT2
175	chr1	151045380	151054113	C1orf56
3154	chr16	1374461	1380149	UNKL
8922	chr9	134322318	134336064	RXRA
4779	chr2	183607256	183608504	NUP35
4025	chr17_KI270910v1_alt	40078	45833	TIMM22
8198	chr7	39564459	39577501	YAE1D1
4962	chr2	24014909	24017144	MFSD2B
3966	chr17	8185796	8188628	MIR4521
7043	chr5	55710989	55713181	SLC38A9
6035	chr3	172747622	172767528	ECT2
5712	chr22	41017790	41023987	SNORD140
2260	chr12	9062779	9068619	A2M-AS1
6312	chr3_KI270935v1_alt	27816	33978	SDHAP2
769	chr1	51862744	51880513	NRDC
3335	chr16	51637895	51639511	LINC01571
6385	chr4	13615076	13638594	BOD1L1
3512	chr17	14050190	14072542	COX10
7668	chr6	47477331	47490426	CD2AP
9270	chrX	19708713	19711198	SH3KBP1
1662	chr11	70394420	70401407	CTTN
3228	chr16	27312713	27347475	IL4R
5158	chr2	6899253	6909094	RNF144A
1680	chr11	76439039	76463584	EMSY
6716	chr5	113028887	113038743	DCP2
270	chr1	161388074	161391490	CFAP126
5488	chr21	16536114	16539915	MIR99A
8880	chr9	129080931	129086523	DOLPP1
9374	chrX	76144018	76146989	PBDC1
9244	chrX	152939933	152942747	ZNF185
7598	chr6	34755259	34763513	SNRPC
6103	chr3	194634553	194635763	TMEM44
4260	chr19	27710998	27755856	LOC101927151
3079	chr15	85378895	85434455	AKAP13
6909	chr5	173143746	173145879	BNIP1
2286	chr12	96028021	96036884	LTA4H
5692	chr22	38178724	38181733	PLA2G6
6037	chr3	177187979	177197138	TBL1XR1
7530	chr6	27812876	27814745	HIST1H2AJ
3420	chr16	72008084	72009699	DHODH
8050	chr7	135913208	135978446	MTPN
7619	chr6	37238587	37244133	TMEM217
5798	chr3	10204902	10206772	IRAK2
8278	chr7	66988964	66996554	SBDS

9202	chrX	119841540	119854163	UPF3B
4511	chr19	58179565	58185265	ZNF274
6049	chr3	182973697	182981664	DCUN1D1
2408	chr13	40108017	40110683	LINC00332
4274	chr19	32976253	32981122	FAAP24
3315	chr16	4423560	4433068	DNAJA3
8637	chr8	53820864	53845742	ATP6V1H
4447	chr19	48362639	48364224	TMEM143
301	chr1	168055005	168058588	MIR1255B2
3409	chr16	70367779	70382308	DDX19A
3926	chr17	78861001	78864799	USP36
8412	chr8	10652040	10653504	RP1L1
2940	chr15	55497242	55500242	DNAAF4-CCPG1
6997	chr5	37368317	37374928	NUP155
277	chr1	162496667	162519074	UHMK1
5184	chr2	73975307	74003362	TET3
7475	chr6	18144972	18159189	TPMT
7484	chr6	21592328	21603641	SOX4
6186	chr3	4456740	4468541	SUMF1
1389	chr11	119168120	119172782	NLRX1
7627	chr6	38672196	38677702	GLO1
1839	chr12	116737223	116739637	RNFT2
6222	chr3	48898048	48899990	SLC25A20
4311	chr19	38262489	38268741	SPINT2
7447	chr6	159690254	159694705	SOD2
5867	chr3	12555976	12567029	MKRN2
7703	chr6	6801316	6805221	LY86-AS1
1113	chr10	35634791	35645551	MIR4683
5243	chr2	96270386	96276256	CIAO1
5419	chr20	50761122	50762533	PARD6B
3760	chr17	50022261	50030297	PICART1
7354	chr6	138402808	138425142	HEBP2
8451	chr8	12427103	12438949	FAM86B2
5514	chr21	33402834	33418132	IFNGR2
5754	chr22	46295391	46298348	GTSE1
2508	chr13	80328192	80339829	SPRY2
7816	chr6_GL000251v2_alt	3373911	3384776	ZBTB12
8603	chr8	38378233	38389112	NSD3
8034	chr7	130846453	130941833	MIR29B1
1380	chr11	118622216	118624285	PHLDB1
7206	chr5_GL339449v2_alt	294035	298793	LOC647859
5807	chr3	108584917	108591536	DZIP3
4008	chr17_KI270857v1_alt	2286677	2293291	LOC440434
7079	chr5	65616094	65645173	TRAPPC13

1823	chr12	111942489	111944509	ADAM1A
4401	chr19	45527106	45533502	VASP
1259	chr10	87235967	87358283	LINC00863
8499	chr8	143289002	143295196	ZNF696
3453	chr16	85557148	85559179	GSE1
5090	chr2	48306763	48317518	FOXN2
6435	chr4	162421013	162422022	FSTL5
4616	chr2	109793041	109795144	RGPD8
6307	chr3	9984332	10011604	EMC3-AS1
2202	chr12	71838162	71858524	MRS2P2
8393	chr8	100910135	100948998	YWHAZ
7392	chr6	1485033	1487051	MIR6720
2860	chr15	38254290	38265864	SPRED1
3143	chr16	11549677	11589764	LITAF
7284	chr6	117674379	117722546	NUS1
7633	chr6	41636320	41656151	MDFI
2905	chr15	44559641	44584177	EIF3J
8523	chr8	144683608	144686931	ARHGAP39
1387	chr11	119066527	119070642	VPS11
3374	chr16	67261263	67264832	SLC9A5
4858	chr2	206210001	206213484	GPR1
5651	chr22	29816800	29839451	ASCC2
7758	chr6	85399250	85406197	LINC02535
4069	chr18	36033532	36068505	RPRD1A
2417	chr13	41123470	41141387	KBTBD6
5081	chr2	46939071	46944560	MCFD2
4457	chr19	4908955	4917836	UHRF1
5268	chr20	11790868	11793522	LINC00687
4572	chr19_GL949752v1_alt	350926	368886	LENG8
4612	chr2	10685033	10693033	NOL10
3820	chr17	62703784	62706710	LOC105371855
5758	chr22	46694349	46696072	CERK
3744	chr17	48096818	48102695	CBX1
4680	chr2	138384016	138397157	SPOPL
4909	chr2	223748363	223761452	AP1S3
5520	chr21	34613101	34616903	RCAN1
7002	chr5	38871452	38903204	OSMR
8687	chr8	75300914	75324616	CASC9
4267	chr19	29711319	29718233	C19orf12
7914	chr6_GL000255v2_alt	4481428	4488069	MIR6834
3956	chr17	81335935	81347348	TMEM105
6299	chr3	9822924	9845381	RPUSD3
8537	chr8	17803845	17806842	MTUS1
4636	chr2	112792053	112795473	IL1A

5227	chr2	88626763	88631157	LOC101928403
9306	chrX	47141362	47147870	NDUFB11
28	chr1	10899054	10903135	C1orf127
578	chr1	235531094	235533200	
2620	chr14	32506805	32508902	AKAP6
7258	chr6	110120738	110141970	WASF1
4047	chr18	2301215	2304829	METTL4
133	chr1	1469767	1477797	ATAD3B
8279	chr7	67225124	67227225	PMS2P4
3793	chr17	57254755	57261064	MSI2
4088	chr18	49799903	49824435	ACAA2
2801	chr14	90378873	90384724	CALM1
4469	chr19	50509905	50521482	ASPDH
3012	chr15	69461229	69463834	RPLP1
4850	chr2	203519742	203537119	RAPH1
4330	chr19	40184363	40194362	MAP3K10
6590	chr4	56957281	56984644	NOA1
7086	chr5	6773682	6775844	LINC02236
2052	chr12	50195082	50224045	LIMA1
2269	chr12	93492057	93501235	MRPL42
2825	chr14_KI270847v1_alt	319749	325648	GON7
5818	chr3	112073699	112076471	C3orf52
3146	chr16	11743145	11746479	TXNDC11
6290	chr3	88148515	88152547	CGGBP1
5187	chr2	74213530	74226730	MTHFD2
5801	chr3	104362336	104450406	MIR548AB
7551	chr6	2876045	2877839	SERPINB9P1
622	chr1	25829255	25839463	LOC646471
5580	chr21	43865468	43880308	AGPAT3
7846	chr6_GL000252v2_alt	4541750	4545740	RGL2
9253	chrX	154396855	154402967	SNORA70
260	chr1	160304252	160364766	NCSTN
9049	chr9	6182290	6185469	IL33
607	chr1	244971618	244978462	LOC101928068
6793	chr5	138534591	138551255	ETF1
1431	chr11	13260222	13263364	ARNTL
2568	chr14	19131710	19141560	BMS1P22
2250	chr12	88924284	88927171	LINC02458
8518	chr8	144443797	144445625	TONSL
659	chr1	31287771	31304983	SNRNP40
8114	chr7	156886202	156896968	LMBR1
6872	chr5	1596179	1599930	SDHAP3
3427	chr16	74358067	74369996	LOC283922
3197	chr16	21492013	21523336	MIR3680-1

5479	chr20	63925898	63938567	MIR1914
2646	chr14	44895977	44900716	C14orf28
3231	chr16	2769852	2782821	ELOB
8544	chr8	19379341	19386666	SH2D4A
7936	chr7	100048815	100055222	ZSCAN21
6471	chr4	182975060	182976790	DCTD
8958	chr9	19087541	19104034	HAUS6
2233	chr12	79626404	79694135	PAWR
1305	chr10	97324943	97336055	FRAT2
2754	chr14	71371309	71373446	SNORD56B
2123	chr12	57101923	57112539	STAT6
6444	chr4	168485773	168490215	DDX60L
7934	chr6_GL000256v2_alt	4900100	4907505	ZBTB9
5048	chr2	38371524	38377160	ATL2
772	chr1	52045920	52073226	BTF3L4
6117	chr3	196566317	196578723	FBXO45
822	chr1	65411416	65419502	LEPROT
7691	chr6	56672941	56694673	DST
926	chr1	94734405	94736738	MIR378G
6667	chr4	9153338	9156882	FAM90A26
455	chr1	210438302	210441119	HHAT
5905	chr3	132713267	132724056	NPHP3-AS1
3709	chr17	45092628	45094569	MIR6784
8425	chr8	116847633	116876405	RAD21-AS1
8017	chr7	128454846	128456534	HILPDA
663	chr1	31573815	31589771	TINAGL1
2679	chr14	52768561	52773526	GNPNAT1
864	chr1	8137576	8138673	LINC01714
4180	chr19	13730192	13734500	CCDC130
8529	chr8	16711047	16713715	FGF20
9388	chrY	2472568	2501623	ZBED1
1302	chr10	96522602	96591641	TM9SF3
7759	chr6	85462674	85470924	NT5E
1162	chr10	61897727	61917299	ARID5B
247	chr1	15828792	15838138	SPEN
2346	chr13	113854210	113863890	GAS6
4560	chr19	9767448	9770740	ZNF846
9251	chrX	154346773	154370935	FLNA
5058	chr2	40625039	40627136	SLC8A1
4449	chr19	48444118	48479209	KCNJ14
2474	chr13	65945642	65947766	MIR548X2
7291	chr6	122445512	122473188	SERINC1
4508	chr19	57388241	57395960	ZNF548
69	chr1	115544826	115547114	VANGL1

6059	chr3	184299383	184312989	PSMD2
5705	chr22	39524581	39534530	RPS19BP1
668	chr1	32177844	32183762	TXLNA
8489	chr8	141122931	141130892	DENND3
6441	chr4	167030582	167033789	SPOCK3
474	chr1	215566322	215591703	KCTD3
5194	chr2	74912364	74920033	LINC01291
1480	chr11	33014313	33017503	DEPDC7
3380	chr16	67653782	67654432	ACD
3995	chr17	9836766	9840108	GLP2R
7298	chr6	124958549	125021959	RNF217
4893	chr2	218289471	218293045	TMBIM1
8284	chr7	7180124	7243032	C1GALT1
6176	chr3	41960641	41962255	ULK4
8173	chr7	29144910	29148936	CHN2
5991	chr3	15630425	15632436	BTD
4973	chr2	24355547	24360285	ITSN2
249	chr1	15885475	15888726	
7884	chr6_GL000254v2_alt	3711219	3713896	C6orf10
1623	chr11	66050889	66075029	PACS1
2277	chr12	94566698	94570213	MIR7844
8448	chr8	123767625	123823230	FAM91A1
282	chr1	164557567	164563410	PBX1
8963	chr9	21569473	21572312	MIR31HG
4137	chr18	9912582	9954039	VAPA
6898	chr5	172804971	172809604	ERGIC1
6723	chr5	115824002	115840668	AP3S1
3725	chr17	46588723	46595750	NSFP1
1469	chr11	2825161	2827339	KCNQ1-AS1
1103	chr10	31939369	31941019	ARHGAP12
2924	chr15	50879052	50882072	AP4E1
5439	chr20	53772775	53775851	SUMO1P1
4800	chr2	195611666	195615166	SLC39A10
6600	chr4	67686851	67713394	UBA6
7401	chr6	149640807	149649367	KATNA1
6392	chr4	139171036	139178938	ELF2
7558	chr6	2987533	2993207	LINC01011
8382	chr7	99555916	99581206	ZNF655
2428	chr13	44286938	44315677	SERP2
5640	chr22	26671951	26679408	MIATNB
8726	chr8	94545962	94561663	VIRMA
7786	chr6	99520674	99570866	CCNC
2863	chr15	39933102	39935741	EIF2AK4
5188	chr2	74439560	74442099	RTKN

7648	chr6	43225423	43228175	DNPH1
9249	chrX	154209028	154211398	OPN1MW2
8255	chr7	6345383	6350278	FAM220A
99	chr1	1336322	1346145	MIR6808
5336	chr20	36604430	36611822	RAB5IF
5112	chr2	57180597	57182989	VRK2
4344	chr19	41351858	41354882	TGFB1
6225	chr3	49022198	49031020	IMPDH2
5229	chr2	9070299	9072376	MBOAT2
9047	chr9	6105213	6119674	RANBP6
5646	chr22	28791618	28805053	XBP1
6507	chr4	25913600	25918682	SMIM20
7020	chr5	43598701	43604148	NNT
7132	chr5	7856198	7919090	MTRR
8758	chr9	101380846	101424216	ZNF189
6170	chr3	39404708	39407892	RPSA
6749	chr5	131778714	131801124	FNIP1
5725	chr22	42251704	42271995	OGFRP1
3745	chr17	48106984	48109407	SNX11
1364	chr11	112119745	112122352	SDHD
3951	chr17	81109555	81112752	MIR657
3517	chr17	15698971	15727337	ZNF286A
6020	chr3	168089662	168097642	GOLIM4
3023	chr15	72040231	72046227	SENP8
3487	chr16	89815539	89819694	FANCA
7849	chr6_GL000253v2_alt	1825639	1827346	LINC02569
9277	chrX	23705404	23745146	ACOT9
2191	chr12	6960766	6962477	MIR200CHG
131	chr1	146385612	146388940	RNU1-3
554	chr1	231388698	231427389	EGLN1
4969	chr2	24120074	24127053	FAM228B
9269	chrX	18674514	18676784	RS1
2330	chr13	110633495	110650422	NAXD
5394	chr20	48956585	48960761	ARFGEF2
6677	chr4	98993918	99012245	MIR3684
4100	chr18	56633436	56640529	TXNL1
2974	chr15	64152072	64154701	SNX22
6952	chr5	181223878	181230163	TRIM41
8800	chr9	113263872	113285749	CDC26
6762	chr5	133013012	133027399	ZCCHC10
2473	chr13	60394639	60404567	TDRD3
8053	chr7	13918448	13921818	ETV1
5589	chr21	46142726	46144873	FTCD-AS1
6058	chr3	184238457	184251441	MIR1224

7302	chr6	125956371	125962237	HINT3
1260	chr10	87501703	87515118	MINPP1
5768	chr22	50299103	50308188	PLXNB2
8872	chr9	128645196	128658548	WDR34
1397	chr11	123062414	123064876	HSPA8
8073	chr7	149062751	149066265	ZNF786
5794	chr3	101702685	101710522	PDCL3P4
8000	chr7	116669633	116699303	MET
828	chr1	66477093	66481618	SGIP1
713	chr1	40068428	40079512	PPT1
4961	chr2	239398093	239401180	HDAC4
7688	chr6	54979628	54982438	HCRTR2
7261	chr6	11091618	11117487	ERVFRD-1
1108	chr10	33330368	33335896	NRP1
8235	chr7	54732117	54761963	SEC61G
6379	chr4	128809466	128819806	JADE1
1916	chr12	131424680	131427436	LINC02370
5516	chr21	33654628	33682468	ITSN1
9247	chrX	153969811	153972709	HCFC1
3559	chr17	21285226	21307958	MAP2K3
2444	chr13	48154897	48156972	MED4
6342	chr4	113970689	113985225	ARSJ
788	chr1	55208572	55219208	USP24
8832	chr9	124704623	124709220	MIR181B2
6691	chr5	103258074	103269398	C5orf30
2803	chr14	90825332	90829661	TTC7B
3688	chr17	43467869	43470458	DHX8
454	chr1	210371685	210374738	HHAT
5822	chr3	11287256	11295245	ATG7
8259	chr7	64562143	64564041	ZNF680
3444	chr16	82652284	82702130	MIR8058
8070	chr7	148939513	148942644	RNY5
5044	chr2	37584792	37588143	CDC42EP3
201	chr1	153782201	153784416	SLC27A3
6616	chr4	74631475	74634283	BTC
7098	chr5	70712980	70716004	LOC653080
6156	chr3	33094814	33098808	TMPPE
2515	chr13	94237278	94240722	GPC6-AS1
1291	chr10	93460981	93480516	MYOF
503	chr1	223710657	223769730	CAPN2
6251	chr3	51498081	51501412	DCAF1
4995	chr2	27623050	27637010	CCDC121
6617	chr4	74658837	74662153	BTC
863	chr1	8089046	8100069	ERRFI1

4795	chr2	191168208	191170728	STAT4
6569	chr4	48338097	48358159	SLAIN2
163	chr1	150319995	150355093	PRPF3
6957	chr5	194023	287498	SDHA
2889	chr15	42494621	42496514	SNAP23
399	chr1	200414794	200418047	ZNF281
4177	chr19	13163218	13165788	IER2
6261	chr3	52707180	52713735	GLT8D1
4849	chr2	20335762	20350653	PUM2
1726	chr11	9362794	9366016	IPO7
3656	chr17	41618110	41626043	KRT17
6263	chr3	53888877	53894107	SELENOK
4143	chr19	10288445	10290584	ICAM5
8381	chr7	99505753	99508882	ZKSCAN5
2200	chr12	71684370	71687253	TMEM19
4132	chr18	9538867	9541346	RALBP1
3375	chr16	67278247	67279384	PLEKHG4
5993	chr3	15645397	15647801	BTD
6746	chr5	131260804	131269905	CDC42SE2
3652	chr17	40316905	40320788	RARA
1745	chr11	95870228	95928330	MTMR2
7360	chr6	139375753	139377331	CITED2
2619	chr14	32483907	32485622	AKAP6
4208	chr19	16615882	16661459	MED26
7114	chr5	74033649	74036338	ARHGEF28
7087	chr5	68214452	68216032	PIK3R1
3802	chr17	59092303	59113434	TRIM37
3518	chr17	15945695	15961309	ADORA2B
312	chr1	169789936	169801746	C1orf112
7972	chr7	106125506	106126500	SYPL1
4512	chr19	58228141	58240720	ZNF544
6256	chr3	52044823	52058651	DUSP7
8437	chr8	121599191	121601030	HAS2-AS1
2578	chr14	21442309	21457439	CHD8
2719	chr14	64985976	64991270	FNTB
8751	chr8_KI270926v1_alt	157482	159615	LOC101927752
7570	chr6	30767130	30770600	HCG20
6143	chr3	28317132	28360064	AZI2
6053	chr3	183445887	183451286	LINC00888
4787	chr2	189781210	189784325	PMS1
8002	chr7	116950541	116990056	ST7-AS1
9139	chr9	94725431	94731894	C9orf3
602	chr1	24408089	24414360	NIPAL3
5903	chr3	132522989	132548920	ACKR4

6202	chr3	47370299	47389801	PTPN23
4544	chr19	7942720	7945793	TIMM44
7015	chr5	43014653	43021131	LOC648987
8449	chr8	124199093	124202116	FER1L6-AS2
5251	chr2	97987313	97998457	TMEM131
188	chr1	151943389	151948266	THEM4
4861	chr2	206614309	206616419	FAM237A
8089	chr7	151455686	151519337	RHEB
8508	chr8	143976260	143977821	PLEC
2648	chr14	45084256	45093615	PRPF39
3253	chr16	29814712	29819569	PAGR1
6131	chr3	197946565	197953459	IQCG
8032	chr7	130484806	130492188	MEST
4696	chr2	150532374	150534378	LINC01920
275	chr1	16205760	16208057	ARHGEF19
639	chr1	27144745	27156999	SLC9A1
3366	chr16	635780	637794	LOC100287175
7069	chr5	62411726	62415682	IPO11
2659	chr14	49861476	49866194	RN7SL2
9352	chrX	68827307	68830487	EFNB1
920	chr1	9427790	9430401	LOC100506022
4737	chr2	171429889	171439152	DCAF17
1237	chr10	77918198	77928495	DLG5
6247	chr3	50347843	50352391	CYB561D2
6658	chr4	86924683	86938126	AFF1-AS1
9295	chrX	41009589	41011891	USP9X
4353	chr19	42242534	42257458	ERF
6082	chr3	189833187	189837543	MIR944
5128	chr2	62715264	62729351	EHBP1
819	chr1	64999926	65004599	LINC01359
8858	chr9	12791996	12825511	LURAP1L-AS1
2562	chr14	104984240	104991983	CLBA1
8943	chr9	137254297	137258107	NELFB
192	chr1	153098578	153102017	SPRR2E
5486	chr21	15729964	15874172	USP25
2279	chr12	95196820	95206065	FGD6
7969	chr7	104975536	105087368	KMT2E
3211	chr16	22584314	22588668	LOC653786
5961	chr3	149960528	149980960	PFN2
8006	chr7	121529683	121531858	FAM3C
4373	chr19	43523502	43528068	ETHE1
534	chr1	227720570	227738466	SNAP47
7962	chr7	103295196	103322044	PMPCB
6417	chr4	151098622	151106665	SNORD73B

758	chr1	46338193	46346529	NSUN4
2603	chr14	28264585	28266061	FOXG1-AS1
2712	chr14	63640685	63643603	WDR89
219	chr1	15525562	15538806	DNAJC16
8849	chr9	127165749	127168429	ANGPTL2
6552	chr4	41149247	41156569	APBB2
1893	chr12	123670463	123672750	TCTN2
2643	chr14	39428292	39434812	FBXO33
6239	chr3	49928538	49931998	MON1A
2720	chr14	65081371	65105424	LOC100506321
4169	chr19	12707554	12724215	TNPO2
5134	chr2	64742228	64753109	SERTAD2
8351	chr7	92117518	92120552	CYP51A1
6381	chr4	129087645	129094596	SCLT1
7556	chr6	2975923	2977879	SERPINB6
6466	chr4	177440473	177443942	AGA
1683	chr11	77298138	77301864	GDPD4
7611	chr6	36677734	36690661	CDKN1A
4348	chr19	42073773	42075949	ZNF574
6236	chr3	49856042	49857500	TRAIP
8853	chr9	127578001	127580243	FAM129B
441	chr1	207392444	207396336	CR2
5739	chr22	43952470	43957773	SAMM50
2888	chr15	42487081	42493635	ZNF106
2781	chr14	76952336	76955367	LINC01629
2369	chr13	24159491	24163095	SPATA13
5119	chr2	61137514	61148092	LOC339803
1046	chr10	13301214	13302855	PHYH
9110	chr9	86252134	86284059	C9orf153
4762	chr2	177330578	177339926	MIR6512
3153	chr16	1305354	1309759	UBE2I
4011	chr17_KI270857v1_alt	2735576	2739522	MIR4734
5007	chr2	28831015	28847265	SPDYA
187	chr1	1519331	1533216	ATAD3A
6737	chr5	123370784	123425236	CEP120
7958	chr7	1024989	1029706	C7orf50
4551	chr19	9017628	9019590	MUC16
160	chr1	149984926	150011829	OTUD7B
8057	chr7	139749278	139779501	HIPK2
5745	chr22	45104786	45106891	NUP50-DT
6621	chr4	759284	771065	LOC100129917
3424	chr16	73067781	73071407	ZFHX3
8848	chr9	127157331	127161590	ANGPTL2
2030	chr12	48118547	48142177	MIR6505

1656	chr11	69380839	69384845	LOC102724265
274	chr1	161764024	161831896	ATF6
5925	chr3	138914249	138915937	LINC01391
2044	chr12	49551735	49569575	MCRS1
6308	chr3_KI270782v1_alt	102169	104833	LINC02012
1966	chr12	27521786	27530621	PPFIBP1
5	chr1	100887377	101038733	DPH5
3910	chr17	763107	798202	MRM3
6243	chr3	50245776	50248158	GNAI2
5185	chr2	74131362	74135675	BOLA3
4569	chr19_GL949750v2_alt	174326	177137	RPS9
8185	chr7	32493419	32524556	AVL9
4729	chr2	165724501	165727058	GALNT3
3119	chr15	99247179	99252781	TTC23
4273	chr19	32962903	32973694	CEP89
940	chr10	100522675	100529727	NDUFB8
5730	chr22	42952553	42960902	PACSIN2
8962	chr9	21559775	21565580	MIR31HG
1135	chr10	47461562	47463216	ANXA8
6139	chr3	25768729	25786137	NGLY1
111	chr1	144776154	144778220	LOC645166
3322	chr16	47598432	47602623	PHKB
6753	chr5	132407867	132412469	C5orf56
6362	chr4	122917343	122927069	NUDT6
5943	chr3	142996845	143046925	U2SURP
1281	chr10	91616153	91618358	HECTD2-AS1
6946	chr5	181097036	181098281	OR2V1
1713	chr11	8869178	8872070	ST5
8780	chr9	110069146	110070967	AKAP2
4476	chr19	52186640	52225252	MIR6801
1739	chr11	94955529	94976438	CWC15
7700	chr6	63807605	63810342	PHF3
256	chr1	159910892	159928167	TAGLN2
2971	chr15	63584052	63602732	FBXL22
5356	chr20	41328706	41331032	LPIN3
1794	chr12	108527778	108571951	SART3
8649	chr8	58848117	58853774	NSMAF
7907	chr6_GL000255v2_alt	2979935	2992254	DDAH2
6525	chr4	3744628	3749480	ADRA2C
8855	chr9	127686296	127687453	MIR3911
595	chr1	241845640	241852876	EXO1
4441	chr19	48198847	48200284	ZSWIM9
2693	chr14	55410118	55413367	ATG14
5679	chr22	36309785	36390549	MYH9

7734	chr6	7588985	7616837	SNRNP48
843	chr1	73181202	73186265	LINC01360
3298	chr16	3193323	3194274	OR1F1
4096	chr18	5293641	5297808	ZBTB14
100	chr1	1396454	1412380	MRPL20
2658	chr14	49790813	49800073	KLHDC2
3189	chr16	1994542	2011212	ZNF598
2763	chr14	73644091	73647316	DNAL1
3989	chr17	879754	881423	NXN
2649	chr14	45108719	45121293	SNORD127
4065	chr18	35495521	35499785	INO80C
1720	chr11	9263384	9265026	DENND5A
7021	chr5	437333	483134	PP7080
4216	chr19	17503758	17525836	LOC100507551
5459	chr20	62120903	62123299	LSM14B
5135	chr2	64766110	64768077	LINC01800
6504	chr4	25147825	25172585	SEPSECS
4146	chr19	10399903	10404526	MIR1181
146	chr1	148527690	148530536	PPIAL4G
398	chr1	200384227	200412005	ZNF281
4603	chr2	104855158	104856261	POU3F3
3455	chr16	85795686	85810361	COX4I1
3174	chr16	1611081	1617028	CRAMP1
6340	chr4	113431582	113435704	ANK2
1567	chr11	62769624	62772327	TAF6L
4871	chr2	207615171	207626515	METTL21A
8724	chr8	94370442	94374310	FSBP
4660	chr2	127801859	127812571	WDR33
3007	chr15	68802988	68825514	ANP32A
7239	chr6	106628237	106635732	RTN4IP1
8825	chr9	121167046	121169479	GSN
4229	chr19	18372881	18381792	GDF15
6855	chr5	150438941	150450971	RPS14
1926	chr12	132820943	132826489	GOLGA3
1405	chr11	124772857	124802922	MSANTD2
3816	chr17	62177014	62180672	TBC1D3P2
5557	chr21	41419084	41423695	MX1
3581	chr17	28727168	28730264	NEK8
2205	chr12	7345744	7370823	ACSM4
7815	chr6_GL000251v2_alt	3310454	3319070	SNORD52
6013	chr3	16098880	16105113	GALNT15
4671	chr2	132438230	132493005	GPR39
2862	chr15	39640582	39643096	THBS1
5013	chr2	30445748	30449528	LCLAT1

809	chr1	62782887	62788804	ATG4C
2298	chr12_GL877876v1_alt	358632	408187	SMIM10L1
7141	chr5	79610543	79630117	TENT2
2611	chr14	31107610	31114373	AP4S1
2817	chr14	99473128	99510655	CCNK
5458	chr20	62062933	62071105	TAF4
7568	chr6	30737367	30745037	FLOT1
6141	chr3	27532474	27535550	SLC4A7
6907	chr5	17303983	17306691	LINC02111
8921	chr9	134134007	134154608	WDR5
8884	chr9	129332763	129337079	LINC01503
8341	chr7	87217090	87221245	TMEM243
5217	chr2	86549535	86565427	CHMP3
3738	chr17	47642419	47666873	KPNB1
2596	chr14	24142354	24148159	PSME2
9142	chr9	95420949	95428231	LOC100507346
7054	chr5	58446734	58459943	PLK2
91	chr1	120849386	120852940	RNVU1-19
4172	chr19	12799171	12803683	PRDX2
4057	chr18	31100669	31103979	DSC2
736	chr1	44154333	44155822	KLF18
9054	chr9	63120232	63125060	LOC101929583
6520	chr4	35019847	35024666	LINC02484
5513	chr21	33379001	33384349	IFNGR2
7479	chr6	19800853	19806156	LOC100506885
452	chr1	210229939	210233374	SERTAD4
6046	chr3	180966740	180989613	DNAJC19
7322	chr6	133450206	133471266	EYA4
1332	chr11	103044610	103095061	DCUN1D5
8562	chr8	23796742	23798353	STC1
5417	chr20	50727947	50730662	PARD6B
6722	chr5	115616655	115628337	TMED7-TICAM2
6462	chr4	176317288	176326281	SPCS3
4349	chr19	42084318	42088092	ZNF574
7639	chr6	42504141	42505988	TRERF1
7581	chr6	32964786	32971598	BRD2
7438	chr6	158164639	158170290	SERAC1
5115	chr2	58145973	58170997	FANCL
9182	chrX	108149359	108158089	ATG4A
8474	chr8	129684560	129686933	CCDC26
1789	chr12	106355616	106367013	POLR3B
4384	chr19	4443051	4456467	MIR4746
9266	chrX	16952701	16955232	REPS2
6916	chr5	174750458	174752265	MIR4634

1737	chr11	94794423	94870434	AMOTL1
4573	chr19_GL949753v2_alt	174431	181231	RPS9
2552	chr14	103329100	103350068	SNORA28
8148	chr7	24051635	24055523	NPY
1677	chr11	74924050	74976722	SPCS2
8861	chr9	128065018	128070162	NAIF1
882	chr1	87044158	87048876	LINC01140
2063	chr12	51238050	51248264	DAZAP2
7725	chr6	73584888	73586937	EEF1A1
3246	chr16	28972218	28977486	SPNS1
5560	chr21	41725539	41728451	LINC00112
1858	chr12	120484490	120500469	NRAV
6929	chr5	1785346	1833483	NDUFS6
6209	chr3	47799492	47804631	DHX30
7366	chr6	141324988	141328398	MIR4465
9222	chrX	130400263	130403692	RBMX2
1775	chr12	104218181	104220926	TXNRD1
5776	chr22_KI270876v1_alt	153274	161412	RBFOX2
3723	chr17	46538140	46553816	LRRC37A2
8128	chr7	17765513	17947162	SNX13
4642	chr2	113715313	113759650	MIR4782
943	chr10	101352392	101357515	BTRC
7502	chr6	26189064	26207504	HIST1H2AD
5273	chr20	1322406	1357285	SDCBP2
8465	chr8	127194271	127227258	CCAT1
4646	chr2	118111947	118118038	INSIG2
6939	chr5	179803041	179809407	SQSTM1
3233	chr16	28290411	28293132	SBK1
6171	chr3	40308357	40319459	EIF1B-AS1
3025	chr15	72197169	72233290	PKM
5051	chr2	38736651	38752934	SRSF7
811	chr1	63365963	63373227	ALG6
4545	chr19	8309249	8328106	NDUFA7
4107	chr18	63237319	63239163	BCL2
8146	chr7	23455734	23479983	IGF2BP3
2345	chr13	113478896	113503399	TMCO3
5338	chr20	36859035	36862891	SOGA1
1426	chr11	129986281	130008977	LINC00167
8630	chr8	48547341	48549332	LOC101929268
5697	chr22	38476438	38508531	DDX17
2947	chr15	56885381	56889588	TCF12
5802	chr3	105363952	105365348	ALCAM
2528	chr13	99535795	99538963	TM9SF2
6866	chr5	157390046	157391846	FNDC9

739	chr1	44729348	44732255	RNU5D-1
5254	chr2	98867851	98871059	KIAA1211L
2195	chr12	69778598	69798096	RAB3IP
4918	chr2	226776284	226795724	IRS1
869	chr1	8349482	8349958	SLC45A1
2612	chr14	31191994	31210306	HECTD1
8659	chr8	65624081	65635925	ARMC1
7280	chr6	11735951	11773988	ADTRP
6548	chr4	40332231	40334973	CHRNA9
2320	chr13	108915244	108917738	MYO16-AS1
4364	chr19	43020165	43026284	PSG11
6831	chr5	143240063	143248014	ARHGAP26-IT1
6412	chr4	145802897	145815534	C4orf51
2926	chr15	51023976	51026065	MIR4713HG
3874	chr17	73190793	73211836	COG1
2156	chr12	64624436	64626656	MIR548Z
7574	chr6	31701175	31704262	ABHD16A
985	chr10	112387904	112391004	ACSL5
2061	chr12	51044849	51066285	LETMD1
8298	chr7	73849805	73851543	METTL27
5076	chr2	46612316	46619273	PIGF
6516	chr4	3074528	3078732	HTT
2445	chr13	48232252	48255421	ITM2B
8686	chr8	73975052	73978288	TMEM70
9385	chrY	11326256	11326753	GYG2P1
2157	chr12	64678869	64680850	MIR548Z
5721	chr22	41675294	41683241	SNU13
7577	chr6	31833404	31841668	SNORD52
3333	chr16	5081860	5087881	ALG1
8977	chr9	26954744	26961284	IFT74
7873	chr6_GL000254v2_alt	1884872	1912890	PRR3
5886	chr3	128725113	128738360	RAB7A
4663	chr2	130179697	130183588	SMPD4
6169	chr3	39381508	39385794	SLC25A38
4083	chr18	47135291	47152012	HDHD2
7865	chr6_GL000253v2_alt	3005797	3010214	ABHD16A
8792	chr9	112062942	112067839	SUSD1
6905	chr5	172956785	172962069	RPL26L1
4051	chr18	251007	270996	THOC1
6292	chr3	911516	914748	CNTN6
1782	chr12	105168814	105208366	APPL2
6458	chr4	175417809	175421002	GPM6A
4313	chr19	38315123	38318648	YIF1B
7575	chr6	31727633	31737344	DDAH2

5448	chr20	58309491	58325305	RAB22A
360	chr1	183020896	183036663	LAMC1
8607	chr8	38993546	39024268	SNORD38D
1689	chr11	7985834	8007954	EIF3F
716	chr1	40529483	40533363	ZNF684
7257	chr6	11008143	11009601	ELOVL2-AS1
3860	chr17	69298343	69328462	ABCA5
6126	chr3	197647768	197658379	MIR922
9356	chrX	70287557	70292242	PDZD11
3381	chr16	67659210	67661681	ACD
1435	chr11	134213849	134225374	NCAPD3
4726	chr2	163731314	163737831	FIGN
5766	chr22	49932939	49936966	CRELD2
5790	chr3	10140546	10149657	VHL
2392	chr13	32537146	32539776	N4BP2L2
1801	chr12	109565010	109575526	MMAB
7850	chr6_GL000253v2_alt	184274	191368	TRIM27
3875	chr17	73221997	73234026	FAM104A
1406	chr11	124951391	124958120	CCDC15
4662	chr2	128089641	128108671	UGGT1
1247	chr10	79693912	79838938	LOC642361
1779	chr12	104670505	104673659	MIR3922
7062	chr5	61300514	61303444	ZSWIM6
5657	chr22	30394552	30398794	SEC14L2
1692	chr11	82899079	82901364	PRCP
9283	chrX	24994207	24997806	ARX
1450	chr11	18543192	18549929	TSG101
5321	chr20	35453067	35459918	CEP250
6836	chr5	143593619	143597536	MIR5197
7732	chr6	75600144	75617828	SENP6
1460	chr11	234937	255563	PSMD13
245	chr1	157118846	157141283	CYCSP52
4163	chr19	1190658	1193710	STK11
30	chr1	109073802	109077753	TAF13
2654	chr14	49595604	49603976	RPS29
8572	chr8	27307999	27312900	TRIM35
6038	chr3	17736734	17742606	TBC1D5
7339	chr6	135180275	135181777	MYB
2110	chr12	56245741	56259798	ANKRD52
7158	chr5	83472079	83476448	VCAN
423	chr1	204562291	204579767	MDM4
8844	chr9	125744294	125777280	PBX3
2457	chr13	49574154	49587249	RCBTB1
8967	chr9	21801227	21809771	MTAP

7220	chr6	100201981	100204307	MCHR2
3742	chr17	48023255	48026111	MIR152
6562	chr4	46432290	46434172	GABRA2
8660	chr8	65643186	65657554	MTFR1
1731	chr11	9383055	9388926	IPO7
2657	chr14	49766737	49775949	KLHDC2
2819	chr14	99970998	99972485	EVL
6854	chr5	1502305	1525756	MIR6075
2104	chr12	56079016	56080210	ERBB3
9160	chrUn_GL000195v1	31005	34465	LOC389831
3705	chr17	44947003	44949267	KIF18B
2850	chr15	34097319	34108551	PGBD4
1617	chr11	65910938	65939247	DRAP1
852	chr1	776337	799987	LOC100288069
31	chr1	109087763	109094333	TMEM167B
7330	chr6	133948039	133963571	TBPL1
1880	chr12	122505429	122529591	RSRC2
8509	chr8	143980611	143987209	PARP10
5457	chr20	6122245	6124586	FERMT1
4042	chr18	22169295	22177348	GATA6
1424	chr11	129834488	129837608	TMEM45B
1330	chr11	10289347	10297724	SBF2
5494	chr21	26388034	26391970	CYYR1-AS1
4144	chr19	10329174	10335638	RAVER1
5934	chr3	141876226	141889869	ATP1B3
5529	chr21	35254901	35258263	RUNX1
624	chr1	25891052	25893689	STMN1
7727	chr6	7385374	7409277	RIOK1
4155	chr19	1138130	1180201	SBNO2
3367	chr16	66381657	66383779	CDH5
4927	chr2	230512666	230535592	SP100
5021	chr2	32275936	32293933	YIPF4
6002	chr3	158109432	158122716	RSRC1
6757	chr5	132512629	132514844	IRF1
5666	chr22	31346349	31348585	LINC01521
4385	chr19	44498388	44502513	ZNF180
1972	chr12	2811468	2816370	ITFG2-AS1
6638	chr4	79850512	79853103	PCAT4
1595	chr11	65012446	65019725	ARL2-SNX15
7472	chr6	17530624	17532255	LOC101928491
293	chr1	16665681	16669289	RNU1-3
8619	chr8	42840982	42844565	THAP1
7583	chr6	33270784	33278323	RPS18
1427	chr11	130068349	130091085	APLP2

7991	chr7	1156630	1170556	LOC101927021
4550	chr19	891818	894358	MED16
995	chr10	116999531	117007748	SHTN1
4700	chr2	151285747	151291387	NMI
2936	chr15	55289343	55290964	RAB27A
1000	chr10	119029059	119037304	NANOS1
6275	chr3	57976912	57978763	FLNB
2757	chr14	73023446	73028435	ZFYVE1
5369	chr20	44665542	44671760	LINC01260
4218	chr19	17996196	17998736	ARRDC2
4888	chr2	217065880	217067558	LINC01921
652	chr1	28504247	28507122	RCC1
4805	chr2	197158863	197160551	LOC101927596
2412	chr13	40658735	40668501	FOXO1
316	chr1	171433923	171436138	PRRC2C
5951	chr3	148990779	149026769	GYG1
8337	chr7	84528179	84530745	LOC101927378
4191	chr19	14484318	14490336	GIPC1
2759	chr14	73291251	73293783	LOC101928123
3731	chr17	47094164	47101322	CDC27
2830	chr15	100915310	100920087	LRRK1
4495	chr19	55644377	55646383	ZNF581
7395	chr6	149232244	149236322	TAB2
284	chr1	1650279	1663959	CDK11B
4465	chr19	49857966	49869034	PNKP
9036	chr9	42564410	42570741	LOC103908605
9223	chrX	13100672	13103435	FAM9C
6511	chr4	26858938	26862045	LOC105374546
5091	chr2	48438962	48453198	PPP1R21
2015	chr12	45928380	45989630	SCAF11
3934	chr17	79791496	79798496	CBX8
6926	chr5	178129492	178133719	RMND5B
959	chr10	103389076	103399955	MIR1307
570	chr1	234770325	234773779	PP2672
5602	chr21	8105825	8110002	LOC100507412
1512	chr11	46842614	46846856	CKAP5
2852	chr15	34229382	34235731	EMC4
8737	chr8	98042993	98052462	RPL30
2589	chr14	22978361	22988171	AJUBA
3941	chr17	80410907	80432470	MIR4730
6355	chr4	121799659	121806562	EXOSC9
6189	chr3	44861267	44867190	TMEM42
9165	chrX	100910555	100911750	XKRX
8838	chr9	125198354	125201714	LOC105376271

2293	chr12	98592650	98599081	SLC25A3
7555	chr6	28940487	28941538	LINC01556
1581	chr11	64266821	64276691	GPR137
661	chr1	31482277	31484687	LINC01225
1112	chr10	35325276	35344718	CCNY
6632	chr4	78550514	78621216	ANXA3
1850	chr12	120110089	120125208	RAB35
8987	chr9	33142997	33169805	B4GALT1-AS1
5530	chr21	35510694	35512870	LOC100506403
7885	chr6_GL000254v2_alt	4260833	4270788	BRD2
3850	chr17	68033911	68039862	KPNA2
495	chr1	221886201	221888503	LINC01655
6584	chr4	55906453	55910018	CEP135
5206	chr2	84948513	84959588	KCMF1
5936	chr3	141956208	141961357	TFDP2
7267	chr6	111972473	111975205	LINC02527
7796	chr6_GL000250v2_alt	3153268	3156658	HSPA1B
3902	chr17	75841620	75857577	UNC13D
7241	chr6	107028013	107049870	C6orf203
8444	chr8	123155997	123158701	FAM83A
7022	chr5	44809251	44855716	MRPS30
184	chr1	151610642	151642183	SNX27
2653	chr14	49573040	49587525	RPS29
7334	chr6	134435426	134437644	LINC01010
8771	chr9	107738105	107739894	KLF4
732	chr1	43648711	43656671	KDM4A
7901	chr6_GL000255v2_alt	206227	207522	LINC01556
2549	chr14	102582791	102585152	RCOR1
4462	chr19	49639256	49653350	SCAF1
8933	chr9	136792852	136799734	CCDC183
1859	chr12	120522847	120530499	COQ5
2874	chr15	40947784	40954644	CHAC1
6374	chr4	128059434	128063609	LARP1B
6264	chr3	54634793	54636042	ESRG
5627	chr22	20977960	20983623	LZTR1
164	chr1	150362579	150367547	RPRD2
1910	chr12	128788815	128825659	SLC15A4
7287	chr6	12007470	12104790	HIVEP1
9093	chr9	75888990	75891351	PCSK5
5834	chr3	11711753	11721684	VGLL4
7941	chr7	100207982	100219759	PVRIG
8996	chr9	33903046	33950380	UBAP2
5296	chr20	296174	305569	ZCCHC3
657	chr1	30617114	30619491	MATN1-AS1

6086	chr3	190011014	190013454	P3H2-AS1
7505	chr6	26248978	26251457	HIST1H3F
7278	chr6	116452145	116454036	CALHM6
5753	chr22	46247159	46258251	CDPF1
6212	chr3	48295597	48304716	NME6
3637	chr17	38823028	38826859	CWC25
3370	chr16	67027906	67046529	CBFB
2422	chr13	41690781	41697833	MIR5006
737	chr1	44630170	44655225	SNORD145
6107	chr3	195533329	195544933	PPP1R2
6627	chr4	77811984	77821532	CNOT6L
781	chr1	53236437	53240695	MAGOH
7465	chr6	167825868	167827619	AFDN-DT
2573	chr14	20681257	20682966	ANG
2496	chr13	75714324	75727705	LMO7
6004	chr3	158765947	158786033	MFSD1
827	chr1	66293480	66296019	PDE4B
3607	chr17	31546189	31551371	MIR193A
1061	chr10	17073693	17076373	CUBN
2466	chr13	51794679	51808358	DHRS12
708	chr1	39025448	39028313	NDUFS5
1741	chr11	9564374	9567878	WEE1
957	chr10	103096875	103100114	NT5C2
7350	chr6	137198766	137219048	IFNGR1
9276	chrX	21942252	21995807	SMS
7722	chr6	73458777	73478831	MTO1
7143	chr5	79970990	79993541	MTX3
2487	chr13	73419719	73423391	LINC00392
5122	chr2	61531998	61538182	XPO1
7647	chr6	43180466	43189297	CUL9
3310	chr16	423473	425560	RAB11FIP3
9067	chr9	6756436	6760608	KDM4C
4944	chr2	234240852	234243251	SPP2
24	chr1	108687966	108699223	PRPF38B
3717	chr17	45305361	45319189	MAP3K14
8690	chr8	79762300	79787304	LINC01607
227	chr1	155949106	155985372	ARHGEF2
7522	chr6	27123169	27136101	HIST1H2BJ
7332	chr6	134287486	134290070	SGK1
4956	chr2	238055445	238057478	SCLY
2502	chr13	77927989	77930252	EDNRB
8940	chr9	137125304	137133948	GRIN1
2303	chr13	101256548	101259542	NALCN
8869	chr9	128369585	128398522	MIR219B

7966	chr7	103733937	103736716	RELN
6747	chr5	131305308	131312209	CDC42SE2
2876	chr15	41229670	41236913	CHP1
2843	chr15	28587133	28594581	HERC2P11
6612	chr4	74107206	74112163	CXCL2
8030	chr7	130356446	130358088	LOC105375504
2846	chr15	30989027	30994100	MTMR10
327	chr1	173713039	173782694	KLHL20
8230	chr7	48083560	48113624	UPP1
9030	chr9	39460296	39464652	ZNF658B
6066	chr3	185622873	185677533	IGF2BP2-AS1
5137	chr2	64837633	64840004	LINC01800
49	chr1	112604323	112623666	ST7L
5175	chr2	70971485	70978842	ANKRD53
7100	chr5	70923089	70927779	SMN1
1550	chr11	61696047	61697636	DAGLA
5635	chr22	24269434	24274300	SPECC1L
6709	chr5	111074585	111081582	TSLP
5436	chr20	53621263	53624540	LOC105372672
2224	chr12	76762183	76776680	ZDHHC17
8561	chr8	23438212	23458395	ENTPD4
7540	chr6	28246973	28253647	ZKSCAN4
9201	chrX	119804334	119808137	RPL39
4224	chr19	18269056	18280958	IQCN
1942	chr12	18688893	18692608	PLCZ1
7547	chr6	2845785	2847910	SERPINB1
2415	chr13	40919914	40922523	TPTE2P5
3257	chr16	29961078	29963558	TMEM219
1887	chr12	123271120	123273398	CDK2AP1
8723	chr8	94245914	94260702	GEM
3787	chr17	56698671	56700471	NOG
7663	chr6	43999765	44010861	LOC101929705
4425	chr19	4706235	4725261	DPP9
7308	chr6	128449877	128523771	PTPRK
1358	chr11	110285488	110298385	RDX
3955	chr17	81328328	81331585	TMEM105
7984	chr7	108556055	108581901	THAP5
7881	chr6_GL000254v2_alt	3239597	3250051	ZBTB12
1905	chr12	12715839	12730684	APOLD1
4736	chr2	171123537	171162911	TLK1
7637	chr6	42208735	42219163	MRPS10
8200	chr7	39946982	39966248	CDK13
8294	chr7	73716718	73718891	STX1A
1095	chr10	29491244	29496690	MIR604

6041	chr3	179413752	179419814	GNB4
2555	chr14	103551870	103564471	BAG5
1310	chr10	97681319	97691354	AVPI1
5836	chr3	118912417	118914748	IGSF11-AS1
4903	chr2	219277340	219288781	DNAJB2
2685	chr14	54428015	54443032	CNIH1
5212	chr2	85576744	85591572	VAMP5
3467	chr16	87777676	87779443	LOC102724467
4445	chr19	48320960	48327072	EMP3
2544	chr14	101971419	101982900	DYNC1H1
7248	chr6	108046603	108077001	OSTM1
3626	chr17	36530492	36556860	GGNBP2
5749	chr22	45655693	45695017	ATXN10
1175	chr10	67848341	67853011	DNAJC12
6930	chr5	178590410	178629130	CLK4
4755	chr2	175998288	176003660	LNPK
1793	chr12	108514304	108519237	FICD
3308	chr16	4135114	4137982	ADCY9
429	chr1	20633790	20635666	MIR6084
845	chr1	74732871	74750472	CRYZ
4951	chr2	237464536	237467042	MLPH
8629	chr8	48513638	48516601	LOC101929268
7588	chr6	33612989	33615428	ITPR3
1188	chr10	69177845	69226565	HKDC1
8178	chr7	30017136	30026577	FKBP14
8291	chr7	73507014	73523071	BAZ1B
5092	chr2	51811506	51814641	LINC01867
4798	chr2	19537329	19540059	OSR1
9264	chrX	16785752	16800094	TXLNG
1320	chr11	100685941	100726413	LOC100128386
5734	chr22	43142927	43144874	MCAT
1975	chr12	28758219	28761355	FAR2
7059	chr5	60837067	60847425	ELOVL7
5339	chr20	36949357	36953195	SAMHD1
4021	chr17_KI270908v1_alt	1095730	1101403	ARL17B
8385	chr7_KI270805v1_alt	43618	45287	
7222	chr6	100296503	100298412	SIM1
2262	chr12	92130822	92147991	LINC01619
4563	chr19_GL383574v1_alt	108431	117451	LSM14A
3689	chr17	43535042	43536179	ETV4
101	chr1	143362442	143364853	LOC645166
4577	chr1_GL383519v1_alt	24251	33958	GBAP1
1171	chr10	63699281	63701728	JMJD1C
2591	chr14	23305567	23306866	BCL2L2-PABPN1

5714	chr22	41204245	41207714	L3MBTL2	
1639	chr11	67302220	67303946	SSH3	
510	chr1	224435018	224444839	CNIH3	
1954	chr12	25194234	25216558	ETFRF1	
3505	chr16_KI270855v1_alt	157683	167210	NMRAL1	
5022	chr2	32355054	32368913	BIRC6	
6659	chr4	87217832	87221638	KLHL8	
7664	chr6	44056880	44060574	LOC101929705	
2666	chr14	50224065	50231832	SOS2	
4766	chr2	179958295	180008489	CWC22	
3906	chr17	75972175	75983527	TEN1-CDK3	
2047	chr12	49740678	49757222	TMBIM6	
8592	chr8	33483659	33488405	MAK16	
3436	chr16	75637753	75666620	TERF2IP	
2935	chr15	55172534	55198208	RSL24D1	
7048	chr5	56542894	56545044	LINC01948	
4640	chr2	113473323	113476639	FOXD4L1	
2382	chr13	29514378	29596524	SLC7A1	
3685	chr17	43299114	43307176	RNU2-1	
1205	chr10	72293640	72300380	DDIT4	
6953	chr5	181244509	181267527	TRIM52	
4170	chr19	12772189	12776337	HOOK2	
5853	chr3	122738644	122746361	HSPBAP1	
9051	chr9	62368143	62380544	FGF7P6	
6609	chr4	73703962	73705742	CXCL8	
5541	chr21	37216677	37271324	VPS26C	
2171	chr12	6597027	6604992	CHD4	
2516	chr13	94548085	94551413	DCT	
5826	chr3	113708156	113760906	NAA50	
2284	chr12	95804597	95807422	LINC02410	
7215	chr5_KI270897v1_alt	249801	253540	SERF1B	
7915	chr6_GL000255v2_alt	4492572	4494535	RGL2	
3739	chr17	4789028	4791160	GLTPD2	
5616	chr22	19168001	19178836	LINC01311	
27	chr1	108946086	108964686	CLCC1	
4251	chr19	2163153	2168165	DOT1L	
8785	chr9	111660312	111669198	GNG10	
9199	chrX	119691926	119693357		6-Sep
7460	chr6	165577760	165583923	PDE10A	
2584	chr14	22828949	22837909	MMP14	
8675	chr8	67172220	67204442	CSPP1	
6699	chr5	107592099	107674299	EFNA5	
6127	chr3	197707949	197713826	RUBCN	
4581	chr1_KI270712v1_random	139562	142125		

9137	chr9	94452396	94468954	MFSD14B
1463	chr11	27362260	27364377	CCDC34
541	chr1	228646726	228654129	RNA5S7
8590	chr8	32332999	32335503	NRG1-IT3
3239	chr16	2865390	2870067	PRSS22
2038	chr12	49125442	49134732	TUBA1B
7686	chr6	54844624	54851820	FAM83B
8918	chr9	133458754	133462324	CACFD1
8731	chr8	95268540	95270891	C8orf37
8665	chr8	6620131	6627441	MIR8055
6332	chr4	109427614	109439815	SEC24B
2234	chr12	79858497	79946793	PPP1R12A
2955	chr15	59252600	59258394	LDHAL6B
1317	chr10	99701395	99767461	COX15
6784	chr5	136131758	136153393	SMAD5-AS1
478	chr1	21781308	21784302	USP48
8370	chr7	97951753	97975415	MIR5692A1
7173	chr5	91278780	91313922	LUCAT1
2821	chr14_GL000225v1_random	100527	102830	
9340	chrX	54637738	54645919	GNL3L
2856	chr15	34972890	34989742	ZNF770
6150	chr3	3125599	3131883	TRNT1
6775	chr5	134844573	134861223	C5orf24
1111	chr10	35305996	35308116	CCNY
6490	chr4	188105894	188114380	TRIML2
8253	chr7	6078376	6082176	EIF2AK1
1054	chr10	14836135	14856329	HSPA14
3654	chr17	4132442	4145878	
2168	chr12	65669295	65670056	LOC100507065
440	chr1	207389169	207391551	CR2
5487	chr21	16193342	16197744	MIR99AHG
5244	chr2	96293429	96308348	SNRNP200
1134	chr10	46785188	46794941	GLUD1P2
1280	chr10	91612209	91615044	HECTD2-AS1
304	chr1	168224278	168239735	SFT2D2
9364	chrX	72617540	72624675	HDAC8
5945	chr3	143969803	143975552	C3orf58
7987	chr7	112787146	112791352	TMEM168
2182	chr12	68806441	68858137	MDM2
1813	chr12	110524386	110538279	RAD9B
8882	chr9	129169792	129171946	IER5L
8358	chr7	93113024	93119396	SAMD9
953	chr10	102448852	102450764	RPARP-AS1

6924	chr5	177488540	177499625	PDLIM7
2082	chr12	53220176	53222526	RARG
2956	chr15	59371886	59375390	MYO1E
8189	chr7	33053396	33065470	NT5C3A
5271	chr20	12341258	12347557	BTBD3
6876	chr5	160397829	160420851	ZBED8
1440	chr11	14883823	14893417	CYP2R1
6888	chr5	171308232	171310522	TLX3
6752	chr5	132367304	132375123	MIR3936HG
6029	chr3	171808500	171813395	PLD1
242	chr1	156765839	156775084	PRCC
2928	chr15	51616987	51623082	DMXL2
18	chr1	107056214	107064355	PRMT6
6795	chr5	138750867	138777620	LOC105379194
4087	chr18	48992052	48999161	LOC100129878
5452	chr20	59016116	59034382	TUBB1
8419	chr8	109642976	109646701	SYBU
826	chr1	66270479	66273081	PDE4B
7859	chr6_GL000253v2_alt	2128569	2130247	LINC00243
5803	chr3	105366622	105523584	ALCAM
5473	chr20	62882660	62952676	DIDO1
866	chr1	8181289	8184131	LINC01714
1199	chr10	71859997	71861452	PSAP
7844	chr6_GL000252v2_alt	4449715	4458777	RING1
4440	chr19	48169040	48172667	ZSWIM9
1976	chr12	28769316	28771680	FAR2
5716	chr22	41379321	41386345	TEF
248	chr1	15843665	15854033	FLJ37453
6378	chr4	128552258	128554914	LOC100507487
2569	chr14	20341982	20364015	PARP2
4833	chr2	201268832	201282841	CASP8
3698	chr17	44314740	44329211	SLC25A39
5654	chr22	30203828	30206189	LIF-AS1
8342	chr7	874413	878291	GET4
465	chr1	21259916	21263645	ECE1
3923	chr17	78591197	78594314	DNAH17
4651	chr2	121644744	121652377	CLASP1
4796	chr2	191244316	191267866	MYO1B
280	chr1	16344517	16353975	FBXO42
1415	chr11	126352116	126354691	GSEC
8734	chr8	96337853	96344558	LOC102724804
3337	chr16	52812282	52814451	CASC16
7514	chr6	26595148	26606178	ABT1
933	chr1	99848684	99852794	AGL

620	chr1	25229665	25244177	SYF2
7045	chr5	5596647	5601054	ICE1
9181	chrX	108080915	108095688	PSMD10
545	chr1	229555986	229560329	ABCB10
5038	chr2	37147229	37158831	EIF2AK2
3247	chr16	29263472	29271614	SNX29P2
4531	chr19	6760328	6768300	SH2D3A
8048	chr7	135660550	135719846	STMP1
2642	chr14	39263485	39274859	MIA2
5332	chr20	36084251	36087006	EPB41L1
6064	chr3	185279385	185300504	MAP3K13
2829	chr15	100850334	100852910	ALDH1A3
3672	chr17	42743277	42745893	EZH1
7365	chr6	141320909	141323078	MIR4465
7631	chr6	41053146	41072746	OARD1
8886	chr9	129411887	129417089	LINC00963
1860	chr12	120533531	120551959	RNF10
6585	chr4	55946593	55956952	CEP135
112	chr1	145159561	145218988	FAM231D
6472	chr4	183437741	183457726	CDKN2AIP
7034	chr5	53405854	53407752	FST
7604	chr6	35915706	35920721	SRPK1
5068	chr2	45168483	45171044	LINC01121
6840	chr5	145833231	145836844	PRELID2
6977	chr5	32092104	32112690	GOLPH3
1736	chr11	94746430	94753195	LOC105369438
5105	chr2	55095323	55102901	RTN4
8219	chr7	44747424	44752628	ZMIZ2
2931	chr15	52178274	52180636	GNB5
1045	chr10	132997724	133012362	LINC01168
5428	chr20	51560688	51563902	NFATC2
4455	chr19	49023392	49027010	CGB3
8653	chr8	61720225	61722810	MIR4470
8939	chr9	137102725	137118589	DPP7
9135	chr9	94167907	94205606	MIRLET7D
7552	chr6	288589	299199	DUSP22
2791	chr14	81203514	81220928	GTF2A1
3390	chr16	68308993	68311952	PRMT7
328	chr1	173812430	173834789	CENPL
4360	chr19	42848429	42855579	PSG10P
8540	chr8	19009544	19013957	PSD3
3201	chr16	21818239	21819634	RRN3P1
1561	chr11	62618481	62623229	B3GAT3
2066	chr12	51390491	51395957	GALNT6

7960	chr7	102990712	102992709	NFE4
6986	chr5	34608170	34610243	RAI14
9052	chr9	62528762	62538964	LOC103908604
6782	chr5	13590186	13593189	DNAH5
1842	chr12	117140040	117144584	TESC-AS1
2467	chr13	52347264	52350225	LINC02333
3285	chr16	31070378	31086684	ZNF646
8431	chr8	118618327	118623797	SAMD12-AS1
900	chr1	92783205	92785876	EVI5
6874	chr5	160023232	160058709	TTC1
4928	chr2	230713290	230825450	CAB39
5378	chr20	4554851	4557055	PRNP
2321	chr13	109218822	109281923	MYO16-AS1
3414	chr16	71472909	71475840	ZNF23
4589	chr2	100012478	100015129	AFF3
4263	chr19	290374	295214	PLPP2
5222	chr2	87050818	87079037	LOC285074
3227	chr16	27255446	27271803	FLJ21408
5237	chr2	95345663	95347129	KCNIP3
1521	chr11	47569629	47575127	KBTBD4
7566	chr6	30678430	30684048	PPP1R18
1633	chr11	6675539	6684801	MRPL17
7999	chr7	116522991	116558917	CAV1
6538	chr4	39360070	39367683	RFC1
5326	chr20	35736803	35745200	RBM39
7056	chr5	5962366	5964614	LINC02145
1865	chr12	121209029	121221459	P2RX4
4200	chr19	1584646	1607104	MBD3
6805	chr5	140172569	140179780	CYSTM1
4784	chr2	188292843	188309017	MIR561
7658	chr6	43601123	43632148	GTPBP2
7370	chr6	142926862	142948994	HIVEP2
7201	chr5	98770856	98774543	RGMB-AS1
5239	chr2	9564566	9588729	ADAM17
7983	chr7	108510112	108526132	PNPLA8
6648	chr4	83011143	83014770	LIN54
5248	chr2	97094290	97096044	FAHD2B
6238	chr3	4992350	4996838	BHLHE40-AS1
8842	chr9	125663812	125676362	MAPKAP1
588	chr1	23772098	23783586	ELOA-AS1
798	chr1	58813853	58818277	LINC01135
1495	chr11	353921	356401	B4GALNT4
1212	chr10	73072651	73096703	P4HA1
4711	chr2	157864279	157880130	ACVR1

386	chr1	193120341	193129565	CDC73
6389	chr4	139037805	139052201	NOCT
5312	chr20	3469878	3474723	ATRN
5843	chr3	120144472	120151288	GSK3B
2335	chr13	110963256	110997687	LINC00431
3673	chr17	42771914	42799720	WNK4
1984	chr12	31071200	31091304	DDX11-AS1
3827	chr17	63812520	63837135	PSMC5
6572	chr4	490404	546141	PIGG
4914	chr2	226113734	226120604	LOC646736
3418	chr16	71844974	71848998	ATXN1L
968	chr10	1045359	1049166	IDI1
3350	chr16	568638	581953	PIGQ
354	chr1	180700491	180704306	XPR1
5565	chr21	42871783	42882189	WDR4
3627	chr17	36850512	36852583	LHX1-DT
3907	chr17	76070174	76074087	SRP68
4664	chr2	130341664	130344577	CCDC115
462	chr1	212280909	212298979	PPP2R5A
119	chr1	145935073	145937260	RBM8A
8323	chr7	76725224	76728916	POMZP3
5987	chr3	155839076	155857738	SLC33A1
7150	chr5	81970434	81982676	ATG10
7380	chr6	144414342	144421639	SNORA98
3851	chr17	68101272	68108718	LINC00674
8982	chr9	32474840	32486112	DDX58
8008	chr7	122237021	122240442	FEZF1-AS1
358	chr1	181098863	181101030	IER5
396	chr1	199757671	199760639	NR5A2
7957	chr7	102471094	102475669	MIR4467
2815	chr14	97105070	97109090	LINC02304
7119	chr5	74842782	74867326	LOC441086
5890	chr3	129160620	129164267	ISY1
6427	chr4	15634146	15657203	FBXL5
7504	chr6	26222415	26231665	HIST1H3E
5256	chr2	9913936	9918221	GRHL1
7526	chr6	27460985	27476994	ZNF184
1253	chr10	84268907	84270246	LINC00858
535	chr1	228068273	228102107	ARF1
5868	chr3	125581804	125597692	OSBPL11
7418	chr6	151362082	151366577	ZBTB2
8682	chr8	73354827	73358028	RDH10-AS1
1344	chr11	107268632	107281549	CWF19L2
1923	chr12	132442369	132448673	FBRSL1

5545	chr21	37367009	37538339	DYRK1A
3260	chr16	30074272	30089453	PPP4C
4638	chr2	113234658	113276163	PAX8-AS1
1301	chr10	96266136	96269420	BLNK
8710	chr8	89898887	89903225	OSGIN2
7321	chr6	133428700	133435967	EYA4
1123	chr10	38390912	38406060	SEPT7P9
715	chr1	40251737	40268966	ZMPSTE24
6661	chr4	87420173	87425538	NUDT9
1445	chr11	17352628	17359564	NCR3LG1
5303	chr20	32109076	32122178	TM9SF4
1214	chr10	73405110	73415537	ANXA7
8241	chr7	55363478	55378860	LANCL2
3129	chr15_KI270905v1_alt	2054509	2109166	TJP1
914	chr1	93812423	93814168	MIR760
5240	chr2	96008208	96012292	FAHD2CP
5799	chr3	10298094	10320882	SEC13
4910	chr2	223956005	223971744	MRPL44
5095	chr2	53775818	53817503	ASB3
5983	chr3	153199295	153204804	RAP2B
3839	chr17	65003389	65059660	GNA13
7769	chr6	88950936	88964643	RNGTT
2340	chr13	113153219	113155227	PROZ
8038	chr7	131326773	131412053	MKLN1
8913	chr9	133026333	133052556	GTF3C5
8768	chr9	107280950	107353143	RAD23B
9023	chr9	37981736	38011208	SHB
6495	chr4	190176798	190180281	DUX4
3303	chr16	3605951	3614026	SLX4
108	chr1	144550736	144553645	RNVU1-11
2607	chr14	29235825	29237884	LINC02282
8835	chr9	124936820	124948636	GOLGA1
7445	chr6	158849086	158855594	OSTCP1
1047	chr10	133142729	133145262	KNDC1
3386	chr16	68017742	68029568	DDX28
6024	chr3	170158026	170182407	PHC3
895	chr1	90891491	91023478	ZNF644
6260	chr3	52535615	52550945	SMIM4
988	chr10	113072545	113097421	SNORA87
3683	chr17	43283058	43287290	RNU2-1
7974	chr7	106423350	106426987	NAMPT
4754	chr2	175153760	175169595	MIR933
7080	chr5	6591168	6637794	SRD5A1
4285	chr19	35299964	35323086	CD22

394	chr1	199271841	199274718	LOC400800
2000	chr12	3871365	3874371	PARP11
2137	chr12	59594491	59599228	SLC16A7
8190	chr7	33127292	33130877	BBS9
6351	chr4	119292231	119302146	C4orf3
9141	chr9	95310646	95319193	FANCC
2857	chr15	35535260	35549988	DPH6-DT
4771	chr2	181784482	181785937	NEUROD1
337	chr1	175191685	175194182	KIAA0040
8292	chr7	73548352	73559004	BCL7B
4342	chr19	41222563	41229583	AXL
5258	chr2	99329998	99339232	TXNDC9
1153	chr10	56349943	56364465	ZWINT
7016	chr5	43058753	43078831	LOC648987
7571	chr6	30780096	30783528	HCG20
6736	chr5	123035943	123039010	PPIC
8970	chr9	21932853	21934466	CDKN2A-DT
9001	chr9	34635535	34639633	SIGMAR1
3204	chr16	2204209	2207310	MLST8
4436	chr19	47728414	47734576	NOP53
2828	chr15	100720876	100723318	ASB7
3838	chr17	64971158	64975423	AMZ2P1
5704	chr22	39518004	39520607	ATF4
3013	chr15	70032201	70035379	MIR629
8638	chr8	53877185	53947075	RGS20
2135	chr12	58908863	58920123	LRIG3
6949	chr5	181183632	181184736	LINC01962
9221	chrX	130259313	130270458	ZNF280C
4823	chr2	200764455	200766759	LINC01792
7497	chr6	26053278	26058880	HIST1H1C
5415	chr20	5060091	5062758	SLC23A2
3674	chr17	42817185	42825508	BECN1
3045	chr15	75574677	75579446	PTPN9
2518	chr13	94600301	94626995	GPR180
7437	chr6	158089104	158091103	SYNJ2
1754	chr11	9900803	9907281	LOC101928008
1686	chr11	78066877	78081288	NDUFC2
2672	chr14	52079218	52082454	NID2
9371	chrX	74572411	74615814	RLIM
1322	chr11	102108378	102243409	YAP1
1634	chr11	66829411	66832300	RCE1
8995	chr9	33814060	33830399	UBE2R2
2274	chr12	9427033	9450514	DDX12P
1401	chr11	123429621	123432601	LOC100128242

1804	chr12	109991384	109997318	GIT2
7762	chr6	85588237	85604539	SNX14
2824	chr14_KI270847v1_alt	1179439	1197610	DDX24
7710	chr6	70666596	70674319	SMAP1
4513	chr19	58276407	58287214	ZNF8-ERVK3-1
21	chr1	108167030	108200110	SLC25A24
4810	chr2	19901941	19913003	TTC32
5935	chr3	141939819	141942510	TFDP2
265	chr1	161152846	161167007	USP21
8801	chr9	113338562	113341297	WDR31
7381	chr6	144924151	144933233	SNORA98
7757	chr6	84758574	84764248	TBX18
6578	chr4	54046270	54066329	CHIC2
7970	chr7	105244587	105270186	SRPK2
1612	chr11	65768022	65781509	AP5B1
6357	chr4	122151109	122155630	KIAA1109
8417	chr8	109316437	109347608	NUDCD1
2173	chr12	66135600	66155352	LLPH
8140	chr7	22724439	22734141	LOC541472
3016	chr15	70794111	70796484	UACA
7304	chr6	126715028	126719016	MIR588
341	chr1	178125446	178162344	RASAL2-AS1
2354	chr13	19600484	19617982	LINC00350
2778	chr14	75535237	75538009	BATF
8280	chr7	67243355	67244836	PMS2P4
3356	chr16	57471699	57475179	DOK4
1315	chr10	99614867	99623917	SLC25A28
3471	chr16	8785643	8826327	PMM2
4592	chr2	101000310	101035276	RPL31
6054	chr3	184017382	184019349	ABCC5
6480	chr4	185423125	185427191	UFSP2
95	chr1	121000328	121003012	LOC100996721
2134	chr12	58423883	58426571	LOC100506869
1696	chr11	83276551	83287064	CCDC90B
3269	chr16	3052418	3060208	MMP25-AS1
2834	chr15	22237313	22240733	MIR1268A
631	chr1	26496053	26503424	HMGN2
8507	chr8	143959456	143974092	PLEC
1488	chr11	34104314	34121941	NAT10
3187	chr16	1969496	1973472	TBL3
1342	chr11	105360656	105363110	CARD18
8545	chr8	19658150	19667399	CSGALNACT1
6295	chr3	96958760	96963507	EPHA6
3241	chr16	28802427	28805188	ATXN2L

8453	chr8	124460404	124483845	RNF139-AS1
8136	chr7	2105291	2111380	SNORA114
4178	chr19	13166321	13169408	IER2
2541	chr14	101729144	101734469	LINC00239
3879	chr17	7393835	7397167	PLSCR3
5706	chr22	40344010	40353909	ADSL
4892	chr2	218280968	218288887	TMBIM1
6434	chr4	158711259	158724226	PPID
8501	chr8	143631613	143639655	ZNF623
8322	chr7	76622509	76630821	POMZP3
8155	chr7	25027453	25031123	OSBPL3
647	chr1	28257379	28281105	SESN2
6570	chr4	48669321	48701149	FRYL
4559	chr19	9689838	9692072	ZNF812P
584	chr1	236520649	236533349	LGALS8-AS1
7857	chr6_GL000253v2_alt	2077372	2081146	HCG20
1528	chr11	56931950	56934875	OR5AK2
9205	chrX	120546212	120551154	CUL4B
5006	chr2	28824520	28829924	SPDYA
4639	chr2	113410294	113415853	CBWD2
7376	chr6	144001217	144008769	PLAGL1
7489	chr6	24706355	24712374	C6orf62
5614	chr22	18903832	18906114	DGCR6
4611	chr2	106190526	106193912	UXS1
4105	chr18	62184327	62190536	KIAA1468
3563	chr17	2356272	2361675	SGSM2
5426	chr20	51486950	51488993	MIR3194
3852	chr17	68244161	68258769	AMZ2
3560	chr17	2302489	2308592	SRR
2700	chr14	59186908	59196887	DAAM1
5956	chr3	149384666	149390386	TM4SF1
998	chr10	118749830	118755765	CACUL1
790	chr1	56643714	56649799	LOC101929935
1766	chr12	10167066	10174107	TMEM52B
463	chr1	212431898	212436996	NENF
8924	chr9	134641722	134643149	COL5A1
4570	chr19_GL949752v1_alt	109679	112534	CNOT3
7017	chr5	43298745	43317595	HMGCS1
6508	chr4	26195731	26198134	RBPJ
6740	chr5	126422098	126427990	GRAMD2B
7499	chr6	26121429	26144809	HIST1H2AC
2107	chr12	56149246	56168800	MYL6
5911	chr3	134350914	134376063	MIR6827
8199	chr7	39614703	39643430	RALA

961	chr10	103916108	103919212	STN1
4135	chr18	9742122	9754649	RAB31
7696	chr6	6031684	6033592	NRN1
5498	chr21	29000815	29009455	LTN1
3658	chr17	41736632	41741861	HAP1
4009	chr17_KI270857v1_alt	2320402	2322282	MRPL45
5208	chr2	85311008	85330931	TGOLN2
3198	chr16	21597554	21605996	METTL9
5894	chr3	129247290	129287691	MIR6826
8535	chr8	17494160	17496399	SLC7A2
4451	chr19	4865868	4869208	PLIN3
373	chr1	1867976	1892097	GNB1
2841	chr15	25371754	25441570	UBE3A
5675	chr22	32557075	32559648	FBXO7
6928	chr5	178203728	178234086	PHYKPL
7494	chr6	26019326	26023574	HIST1H4A
3927	chr17	78978062	78980534	LGALS3BP
9278	chrX	23783371	23788423	SAT1
4276	chr19	33173712	33178246	LRP3
6817	chr5	141410678	141419276	PCDHGA10
2788	chr14	77756318	77762178	C14orf178
7142	chr5	79651066	79696879	CMYA5
7432	chr6	156766791	156813203	MIR4466
8226	chr7	4737921	4740361	AP5Z1
1064	chr10	17346570	17355773	ST8SIA6-AS1
4319	chr19	38927988	38938521	MRPS12
6734	chr5	122062897	122074219	LOX
4839	chr2	202032290	202037357	FZD7
1509	chr11	46585144	46595905	AMBRA1
8015	chr7	127649041	127852621	SND1
2832	chr15	101634877	101656187	TM2D3
2765	chr14	73754133	73762798	MIR4505
7774	chr6	89713132	89722417	LYRM2
6384	chr4	13446336	13485839	RAB28
4448	chr19	48392092	48403444	GRIN2D
5842	chr3	120094203	120110642	GSK3B
1698	chr11	85626894	85665950	TMEM126A
5308	chr20	33487142	33491825	CBFA2T2
7139	chr5	79323169	79332954	JMY
1252	chr10	8365721	8367062	LINC00708
3457	chr16	86662251	86666046	LINC02189
7225	chr6	100868669	100885065	ASCC3
5535	chr21	36428266	36435393	CLDN14
6367	chr4	124939068	124946686	ANKRD50

6892	chr5	172272534	172285446	UBTD2
5668	chr22	31425311	31451948	DRG1
7310	chr6	129499901	129505464	ARHGAP18
6410	chr4	145613878	145623028	MMAA
3210	chr16	2254185	2269357	RNPS1
1500	chr11	4091764	4137804	RRM1
3741	chr17	47935612	47951297	PNPO
4176	chr19	13149639	13157471	IER2
2253	chr12	8942171	8951758	M6PR
3068	chr15	82532552	82542517	RPS17
5861	chr3	123947348	123962051	CCDC14
5035	chr2	36649069	36651156	VIT
8477	chr8	130005685	130018239	MIR5194
3508	chr17	10715645	10718626	MAGOH2P
5964	chr3	150364234	150366104	LINC01214
1615	chr11	65888038	65893959	CCDC85B
4279	chr19	33819545	33822074	KCTD15
1208	chr10	72675283	72677808	MCU
5004	chr2	28745571	28765098	PPP1CB
8293	chr7	73678441	73687551	DNAJC30
1477	chr11	32086953	32109554	RCN1
8791	chr9	112011562	112013244	MIR4668
5014	chr2	30605733	30608202	LCLAT1
216	chr1	155125815	155139869	SLC50A1
7976	chr7	106716744	106719562	CCDC71L
7701	chr6	6683967	6687974	LY86-AS1
8	chr1	10208706	10214799	KIF1B
3077	chr15	84747086	84755228	ZNF592
4275	chr19	33080310	33088596	GPATCH1
3778	chr17	51236606	51265865	MBTD1
7451	chr6	1604519	1622791	FOXC1
4751	chr2	174241404	174250566	OLA1
444	chr1	209374452	209377363	LINC01698
8353	chr7	92447623	92462579	GATAD1
1	chr1	100032755	100053005	MFSD14A
6812	chr5	140718028	140719858	VTRNA1-2
1827	chr12	112129476	112134103	TRAFD1
2295	chr12	988221	995081	ERC1
445	chr1	209403541	209406003	MIR205HG
707	chr1	38990179	39000369	AKIRIN1
6631	chr4	78179899	78181341	SNORD161
6635	chr4	7938658	7940549	AFAP1
5416	chr20	50645010	50647540	LOC100506175
5260	chr2	9950051	9969929	GRHL1

6157	chr3	33112832	33115280	CRTAP
5632	chr22	23786340	23790547	SMARCB1
1788	chr12	106300295	106313380	TCP11L2
8588	chr8	30553720	30556521	SMIM18
3057	chr15	78537862	78548276	PSMA4
2229	chr12	77938373	77978497	NAV3
3632	chr17	37486912	37494122	DUSP14
3466	chr16	87750972	87769470	KLHDC4
4526	chr19	6076399	6077967	RFX2
1605	chr11	65470058	65479603	MALAT1
4916	chr2	226409894	226413229	MIR5702
3001	chr15	67515965	67532721	IQCH-AS1
6031	chr3	171966097	171968856	TMEM212-AS1
3389	chr16	68288047	68306658	PRMT7
4237	chr19	18912185	18930091	DDX49
7779	chr6	9502483	9505004	HULC
2411	chr13	40584254	40619706	FOXO1
5783	chr2_KI270767v1_alt	113308	115585	LINC01880
3534	chr17	18037222	18040073	ATPAF2
5337	chr20	36767791	36776122	DSN1
138	chr1	148149235	148151926	NBPF11
6741	chr5	126775106	126779630	LMNB1
7836	chr6_GL000252v2_alt	2866848	2887947	MIR6832
4707	chr2	155964282	155966535	LINC01876
5682	chr22	36453686	36463991	TXN2
3634	chr17	38285364	38287591	MRPL45
1599	chr11	65260577	65267742	POLA2
8072	chr7	149018218	149030286	PDIA4
8793	chr9	112171456	112178999	SUSD1
3252	chr16	29794373	29813005	MAZ
3591	chr17	29566345	29569575	TP53I13
7040	chr5	55300392	55320646	MTREX
8233	chr7	530619	540311	HRAT92
3893	chr17	75251922	75267896	GGA3
5072	chr2	46066177	46071956	EPAS1
9038	chr9	4296728	4299217	GLIS3
4748	chr2	173150646	173185048	MAP3K20
6814	chr5	140976391	140978471	PCDHAC2
4703	chr2	152170934	152177740	STAM2
1464	chr11	27471559	27473896	LGR4
890	chr1	88983631	88997768	KYAT3
4211	chr19	17266951	17269476	BABAM1
1706	chr11	8679003	8684224	RPL27A
6321	chr4	102780752	102830542	UBE2D3

2733	chr14	67810022	67817851	ZFYVE26
2635	chr14	37196180	37200057	MIPOL1
7311	chr6	129747169	129750942	ARHGAP18
6873	chr5	159902041	159903268	ADRA1B
2606	chr14	28762498	28773841	FOXG1
5359	chr20	41609513	41619149	CHD6
83	chr1	119631439	119650542	ZNF697
3307	chr16	3851858	3883764	CREBBP
5863	chr3	124752628	124779867	MIR544B
3870	chr17	72420601	72424352	LINC02003
5645	chr22	28772392	28781524	CCDC117
2752	chr14	71117038	71127272	SIPA1L1
2618	chr14	32073494	32077015	ARHGAP5-AS1
2501	chr13	77536819	77538583	SCEL
7074	chr5	64745269	64783481	SREK1IP1
1420	chr11	128907965	128909697	C11orf45
1776	chr12	104255120	104257740	TXNRD1
4389	chr19	44691135	44693609	CEACAM16
6772	chr5	134510327	134513576	LINC01843
5849	chr3	121715444	121753626	GOLGB1
6132	chr3	197955935	197961866	IQCG
7875	chr6_GL000254v2_alt	2029947	2036531	PPP1R18
3182	chr16	18904705	18928521	SMG1
1165	chr10	62384118	62385575	LOC283045
250	chr1	159013321	159018208	IFI16
1784	chr12	105561677	105563428	CASC18
8211	chr7	43952137	43956224	UBE2D4
4777	chr2	183077604	183079986	DUSP19
9207	chrX	120624785	120631715	C1GALT1C1
729	chr1	43370009	43462761	SZT2
4354	chr19	42267443	42281922	CIC
1104	chr10	32323641	32348905	EPC1
7553	chr6	28894785	28898200	HCG14
4024	chr17_KI270908v1_alt	968407	970168	KANSL1
3082	chr15	88621390	88664062	ISG20
5825	chr3	113693908	113698468	USF3
5225	chr2	87810057	87825584	RGPD2
1350	chr11	10841780	10861890	ZBED5
3746	chr17	48238405	48241868	THRA1/BTR
4278	chr19	33793173	33803288	KCTD15
2413	chr13	40743265	40772241	MRPS31
9381	chrX	80807233	80813627	BRWD3
7926	chr6_GL000256v2_alt	2947679	2966630	APOM
9390	chrY	9141749	9142295	RBMY1A3P

2694	chr14	55577066	55611581	KTN1
5720	chr22	41665860	41673832	SNU13
313	chr1	170529300	170558375	LOC101928650
5866	chr3	125329484	125381234	ZNF148
6849	chr5	149343150	149361428	GRPEL2-AS1
4310	chr19	37998447	38001423	SIPA1L3
2499	chr13	77286722	77328133	MYCBP2
170	chr1	150871351	150878245	ARNT
6115	chr3	196423474	196434203	UBXN7-AS1
4257	chr19	2506388	2508106	GADD45B
8688	chr8	75406315	75409540	HNF4G
5163	chr2	69827865	69831367	GMCL1
5594	chr21	6034433	6041857	LINC01669
8930	chr9	136452364	136489343	SEC16A
276	chr1	162067684	162070536	NOS1AP
8764	chr9	105694370	105703996	TMEM38B
2696	chr14	58198476	58205598	ACTR10
1807	chr12	110238493	110242240	ATP2A2
4315	chr19	38617925	38642915	EIF3K
3056	chr15	78436462	78480733	IREB2
7135	chr5	78699330	78701220	LHFPL2
3005	chr15	68577815	68581678	CORO2B
8998	chr9	34223480	34228487	UBAP1
3580	chr17	28722033	28724825	SNORD42A
3352	chr16	56982161	57002238	NLRC5
5086	chr2	47794330	47817530	MSH6
4770	chr2	180977932	180984125	UBE2E3
1759	chr11_KI270831v1_alt	224719	290443	CARS-AS1
8730	chr8	95132963	95161069	PLEKHF2
3191	chr16	2033701	2035138	SLC9A3R2
166	chr1	1504868	1518052	ATAD3A
8357	chr7	92902952	92905854	CDK6
6338	chr4	112145606	112194293	FAM241A
4242	chr19	19384698	19392194	GATAD2A
9347	chrX	64204297	64208392	AMER1
1378	chr11	11840359	11965482	USP47
3962	chr17	81623527	81638615	NPLOC4
3080	chr15	87667537	87671401	LINC00052
970	chr10	106435397	106438064	LOC105378470
5597	chr21	6213798	6220638	LOC102723360
7290	chr6	122397922	122400107	HSF2
2158	chr12	64756648	64762633	TBC1D30
6284	chr3	8627549	8631677	SSUH2
5601	chr21	7743547	7752370	SMIM11B

2435	chr13	45360879	45382115	TPT1-AS1
4234	chr19	18677036	18679427	CRTC1
3321	chr16	47564474	47571639	PHKB
9095	chr9	7787999	7801046	DMAC1
7835	chr6_GL000252v2_alt	2462281	2471731	HCG27
608	chr1	24501581	24503343	RCAN3AS
5073	chr2	46223866	46226787	EPAS1
2673	chr14	52110452	52113333	NID2
3703	chr17	44825982	44832049	GJC1
2008	chr12	43755084	43776124	IRAK4
4381	chr19	44071240	44072728	ZNF284
8752	chr8_KI270926v1_alt	167147	170250	LOC101927752
3531	chr17	17588775	17594291	PEMT
7174	chr5	91365826	91392286	ARRDC3
4347	chr19	41882300	41895714	ARHGEF1
8492	chr8	141391086	141397807	PTP4A3
7895	chr6_GL000255v2_alt	1852364	1871159	PPP1R10
612	chr1	247185978	247188892	MIR3916
3055	chr15	78271829	78302753	WDR61
7565	chr6	30606473	30616094	PPP1R10
8479	chr8	131903351	131961853	EFR3A
1017	chr10	122062384	122064594	TACC2
3024	chr15	72111459	72123739	MYO9A
3402	chr16	69559863	69602702	NFAT5
464	chr1	212553648	212562411	ATF3
4988	chr2	27311094	27327158	MPV17
6856	chr5	150640548	150642093	SYNPO
4388	chr19	44685242	44690044	CEACAM16
2240	chr12	82649508	82652074	TMTC2
5846	chr3	120595054	120605281	NDUFB4
6724	chr5	115852485	115934200	ATG12
2726	chr14	67531877	67533803	PLEKHH1
1043	chr10	132901	135919	ZMYND11
4486	chr19	55213387	55224893	PTPRH
7053	chr5	58258844	58260961	LINC02101
5507	chr21	32260747	32281615	MIS18A
2228	chr12	77121130	77124265	E2F7
2879	chr15	41401169	41403596	NDUFAF1
4394	chr19	45401156	45410428	CD3EAP
6960	chr5	19927779	19931244	CDH18
1080	chr10	24464839	24499506	KIAA1217
6226	chr3	49090553	49094752	QRICH1
2037	chr12	49068268	49069512	RHEBL1
7115	chr5	74240374	74243105	LINC01335

2126	chr12	57460253	57462736	GLI1
2336	chr13	112582534	112595520	TUBGCP3
6231	chr3	49428667	49430520	NICN1
7326	chr6	133607526	133611365	LINC01312
9227	chrX	13223304	13225333	LINC02154
6492	chr4	188738278	188742117	LINC02508
6305	chr3	9902022	9904938	IL17RE
1138	chr10	47705057	47711250	FAM35DP
193	chr1	15345227	15347558	LOC101927417
2997	chr15	67106002	67168521	SMAD3
435	chr1	207231731	207234071	CD55
7217	chr5_KI270897v1_alt	448606	450969	SERF1B
5898	chr3	130819543	130824701	ATP2C1
600	chr1	243843876	243852957	AKT3
553	chr1	231325001	231369087	SPRTN
4740	chr2	171888704	171895351	SLC25A12
254	chr1	159852767	159855299	SNHG28
4964	chr2	240431333	240440034	GPC1
3105	chr15	92831081	92833027	ASB9P1
2908	chr15	44779854	44785738	TRIM69
4484	chr19	55165954	55168566	DNAAF3
1588	chr11	6472894	6475340	TRIM3
6419	chr4	151407733	151412497	FAM160A1
1609	chr11	65614503	65640902	MIR4690
3250	chr16	29450864	29458826	BOLA2-SMG1P6
4287	chr19	35503670	35510843	DMKN
5008	chr2	28866100	28871658	TRMT61B
694	chr1	36396694	36400512	LSM10
4378	chr19	43764781	43766320	SMG9
4955	chr2	237965657	237971406	UBE2F
4985	chr2	27050938	27060162	AGBL5
5990	chr3	15597337	15603578	BTD
9281	chrX	24145451	24150496	ZFX
2954	chr15	59111789	59117286	CCNB2
1732	chr11	94126793	94148955	PANX1
8574	chr8	27763590	27774235	CCDC25
1192	chr10	70145397	70147748	TYSND1
2054	chr12	50277909	50291768	LIMA1
1796	chr12	108732894	108735219	CORO1C
9245	chrX	153687538	153700276	SLC6A8
2226	chr12	76878126	76880345	CSRP2
6469	chr4	182134170	182161542	LOC90768
5948	chr3	14644370	14666834	CCDC174
687	chr1	35170687	35194355	SFPQ

1157	chr10	59897204	59909093	CCDC6
9153	chr9	97972977	98010898	ANP32B
97	chr1	1226417	1240078	B3GALT6
7430	chr6	15662406	15663567	DTNBP1
186	chr1	151772201	151774765	OAZ3
7677	chr6	52512264	52513766	TRAM2
2953	chr15	59083420	59087095	CCNB2
2169	chr12	6580132	6583081	SCARNA11
3504	chr16_KI270855v1_alt	125921	135142	DNAJA3
13	chr1	10465140	10473446	DFFA
6547	chr4	40237130	40240311	RHOH
6337	chr4	112082418	112084720	FAM241A
9210	chrX	123823744	123825916	XIAP
6580	chr4	55345352	55349400	SRD5A3
5363	chr20	43654719	43659819	MYBL2
7893	chr6_GL000255v2_alt	1770623	1772937	LINC02569
7867	chr6_GL000253v2_alt	4389926	4391237	BRD2
773	chr1	52086952	52092874	BTF3L4
4864	chr2	207238255	207241783	MYOSLID
4076	chr18	40866462	40869202	KC6
8784	chr9	111628431	111640544	DNAJC25
1578	chr11	63984961	63989847	OTUB1
5996	chr3	156811795	156831051	LINC00886
3062	chr15	80059933	80136259	ZFAND6
2770	chr14	74487155	74501106	ISCA2
2837	chr15	22838788	22846901	NIPA2
3680	chr17	43152507	43192197	NBR1
4971	chr2	241260677	241338676	2-Sep
2116	chr12	56467326	56486486	SPRYD4
859	chr1	8027186	8030108	ERRFI1
5145	chr2	65338317	65341237	SPRED2
8024	chr7	129438264	129441425	STRIP2
8666	chr8	66431132	66439094	ADHFE1
5461	chr20	62179460	62200562	MTG2
4885	chr2	215360822	215381829	ATIC
174	chr1	151035655	151039514	BNIPL
7286	chr6	1189431	1191817	LINC01622
4821	chr2	200564946	200572638	AOX1
5689	chr22	37775843	37786255	H1F0
744	chr1	44806443	44813343	BTBD19
7642	chr6	42927821	42931004	CNPY3
2379	chr13	27612669	27653802	POLR1D
5360	chr20	43456685	43475648	SRSF6
2608	chr14	30557911	30565964	G2E3

6761	chr5	132937823	132966557	AFF4
5156	chr2	68251783	68258241	PPP3R1
2450	chr13	48971656	49018810	FNDC3A
2844	chr15	30469852	30475885	LOC100288203
1187	chr10	69122349	69153636	VPS26A
2039	chr12	49186137	49189605	TUBA1A
5605	chr22	15784945	15871500	BMS1P22
2238	chr12	81167035	81169335	MIR4699
139	chr1	148262084	148264939	LOC101927468
5101	chr2	54720646	54725204	EML6
7149	chr5	81739230	81753431	SSBP2
2147	chr12	63779677	63803891	RXYLT1
3339	chr16	53098586	53103297	CHD9
2072	chr12	52231530	52251455	KRT7-AS
6853	chr5	150057033	150063315	CSF1R
3599	chr17	30823068	30825739	CRLF3
8276	chr7	6679091	6708370	ZNF12
3596	chr17	30288831	30294114	BLMH
6633	chr4	78642501	78648090	LINC01094
488	chr1	220299958	220302938	RAB3GAP2
8661	chr8	65715389	65723426	MTFR1
7635	chr6	41888737	41898998	USP49
3804	chr17	59781625	59791755	MIR21
6619	chr4	75663683	75675315	G3BP2
9041	chr9	4982501	4991483	JAK2
4976	chr2	24915962	24921434	ADCY3
5343	chr20	37693453	37696947	CTNNBL1
937	chr10	100259320	100268642	CWF19L1
1833	chr12	114773510	114775146	TBX3
2760	chr14	73490325	73501403	RIOX1
8546	chr8	19725697	19733351	CSGALNACT1
4887	chr2	216497768	216514362	RPL37A
8795	chr9	112367959	112383946	HSDL2
7597	chr6	34742987	34745218	SNRPC
3814	chr17	61924006	61929161	INTS2
8167	chr7	27677459	27679429	HIBADH
5808	chr3	1086343	1088562	CNTN6
6577	chr4	53376749	53404726	FIP1L1
5738	chr22	43806486	43821049	EFCAB6
4994	chr2	27580911	27594273	ZNF512
1385	chr11	119023272	119028173	SLC37A4
2102	chr12	56006720	56009015	IKZF4
3078	chr15	85342161	85346463	MIR7706
8018	chr7	128475375	128479235	METTL2B

7840	chr6_GL000252v2_alt	3068672	3071309	HSPA1B
8256	chr7	6372650	6376540	RAC1
7747	chr6	81741022	81754083	TENT5A
2495	chr13	75703521	75709394	LMO7
5980	chr3	15268555	15270514	SH3BP5-AS1
323	chr1	17231426	17235718	PADI3
3867	chr17	71897454	71902977	ROCR
4249	chr19	2084320	2099462	MOB3A
8578	chr8	28629820	28631837	EXTL3-AS1
6602	chr4	68338317	68351174	YTHDC1
568	chr1	234683505	234685089	LOC101927787
340	chr1	178094386	178119061	RASAL2-AS1
2275	chr12	94458249	94465006	CEP83-DT
1289	chr10	92639254	92641076	KIF11
5399	chr20	49344993	49346518	SNORD12
1968	chr12	27778676	27804732	KLHL42
2933	chr15	53501633	53541213	WDR72
4758	chr2	176177096	176179932	HAGLROS
8777	chr9	108999836	109030556	CTNNAL1
154	chr1	149557667	149560961	PPIAL4C
520	chr1	225880115	225883827	TMEM63A
4485	chr19	55206095	55210736	PTPRH
3636	chr17	38463869	38505583	ARHGAP23
6906	chr5	172996169	173017800	SNORA74B
571	chr1	234978746	234981939	LOC101927851
5615	chr22	19103875	19121580	DGCR2
5932	chr3	14137975	14150750	TMEM43
8525	chr8	144827829	144846933	ZNF7
4889	chr2	217103086	217107309	DIRC3-AS1
8036	chr7	131163366	131179936	MKLN1
321	chr1	171840831	171841801	DNM3
2872	chr15	40805435	40810416	DNAJC17
7632	chr6	4132698	4135929	ECI2
9104	chr9	83963648	83984579	MIR7-1
8892	chr9	129776375	129778865	PTGES
5821	chr3	11271196	11284662	ATG7
4548	chr19	8511280	8514614	ZNF414
6550	chr4	40958964	40993813	APBB2
2891	chr15	42830511	42833655	TTBK2
8354	chr7	92615274	92634228	FAM133B
4894	chr2	218395108	218400757	CTDSP1
4705	chr2	152506259	152508823	FMNL2
3499	chr16_KI270853v1_alt	689150	696468	PDXDC1
7076	chr5	65196521	65200197	ADAMTS6

5742	chr22	44485407	44499714	RTL6
4166	chr19	12608523	12623031	ZNF791
4552	chr19	9138860	9173191	ZNF317
9300	chrX	45516157	45519102	LINC01204
1843	chr12	118021473	118024484	RFC5
4494	chr19	55604134	55626003	ZNF865
8744	chr8	99946237	99950238	COX6C
2256	chr12	89709522	89714361	ATP2B1-AS1
3845	chr17	67420566	67426518	PITPNC1
7006	chr5	39416408	39426541	DAB2
8975	chr9	25674557	25684221	TUSC1
7860	chr6_GL000253v2_alt	22958	24434	LINC01623
5442	chr20	5606589	5609525	GPCPD1
7219	chr5_KI270897v1_alt	573506	617448	GTF2H2C
1493	chr11	35076407	35078584	LOC100507144
7191	chr5	96077916	96081221	MIR583
3240	chr16	28688243	28690683	EIF3C
7888	chr6_GL000254v2_alt	4734629	4736510	RGL2
4992	chr2	27430746	27447061	KRTCAP3
2278	chr12	94617079	94620357	TMCC3
2086	chr12	53421030	53421969	AMHR2
8285	chr7	72829470	72836489	SBDSP1
8681	chr8	70656832	70671333	LACTB2
4291	chr19	35715235	35720328	KMT2B
8988	chr9	33288624	33345074	NFX1
6900	chr5	172857053	172860760	ERGIC1
3208	chr16	22296969	22299582	POLR3E
3037	chr15	74506922	74508156	ARID3B
3650	chr17	40110104	40128534	MSL1
5493	chr21	25853924	25856253	ATP5PF
107	chr1	144522085	144525548	RNVU1-11
8803	chr9	113564311	113566448	RGS3
8343	chr7	87873797	87881543	SLC25A40
6349	chr4	119101872	119104756	MYOZ2
2851	chr15	34224273	34226528	EMC4
347	chr1	179364876	179367187	AXDND1
6482	chr4	185826848	185828057	SORBS2
1540	chr11	59657454	59673423	PATL1
366	chr1	184733761	184756314	EDEM3
4079	chr18	45802507	45804340	SIGLEC15
68	chr1	115277896	115302088	NGF
36	chr1	109482892	109486851	ATXN7L2
4722	chr2	161241320	161242335	LINC01806
6641	chr4	81151214	81155878	PRKG2

816	chr1	6489411	6497634	PLEKHG5
8005	chr7	120949198	120979877	ING3
3415	chr16	71482208	71486758	ZNF19
8677	chr8	67585347	67585968	CPA6
2165	chr12	65629693	65633391	LOC100507065
6629	chr4	77861632	77867775	MRPL1
8897	chr9	130170069	130173305	NCS1
2141	chr12	62257160	62293970	MIR6125
1068	chr10	1953469	1956088	LINC00700
893	chr1	89634785	89642435	LRRC8C
7579	chr6	31942154	31964381	MIR1236
4298	chr19	3625830	3628412	CACTIN
7194	chr5	96491022	96494173	PCSK1
4908	chr2	223718977	223726556	AP1S3
4409	chr19	45898665	45904067	MYPOP
3393	chr16	68921049	68923051	TANGO6
949	chr10	102057344	102059040	ARMH3
5435	chr20	53608831	53613236	LOC105372672
6902	chr5	172884392	172893033	ERGIC1
1979	chr12	2957682	2961497	TEAD4
106	chr1	144423337	144461567	NBPF15
7440	chr6	158241560	158252590	TULP4
4555	chr19	9537073	9541842	ZNF426-DT
2500	chr13	77329318	77331718	MYCBP2
813	chr1	63516719	63525457	ITGB3BP
7341	chr6	13569212	13578539	SIRT5
1494	chr11	35136328	35228850	SNORD164
8147	chr7	23517311	23533083	TRA2A
3384	chr16	67952451	67969817	SLC12A4
79	chr1	118961978	118964763	TBX15
6875	chr5	160164602	160166591	FABP6
1749	chr11	9756304	9773115	LOC440028
4165	chr19	1247300	1277728	CIRBP
2543	chr14	101946526	101952788	DYNC1H1
8012	chr7	12658827	12661523	ARL4A
6509	chr4	26316610	26334535	RBPJ
4367	chr19	43214862	43216317	PSG4
764	chr1	51235006	51241594	RNF11
7921	chr6_GL000256v2_alt	1979098	1984410	PPP1R18
3359	chr16	57806755	57811566	LOC388282
7391	chr6	148341479	148347222	SASH1
2414	chr13	40786231	40794491	SLC25A15
6460	chr4	17613229	17621454	MED28
6792	chr5	138352636	138358251	KDM3B

6622	chr4	75961906	75970304	LOC101928809
8978	chr9	27523162	27529950	IFNK
1946	chr12	19710866	19714491	AEBP2
7246	chr6	10745879	10802277	TMEM14B
5690	chr22	37841059	37868170	EIF3L
5554	chr21	39452383	39454922	SH3BGR
7214	chr5_KI270793v1_alt	71703	73155	MIR4456
6523	chr4	36229524	36245940	ARAP2
2118	chr12	56666679	56689580	PTGES3
2774	chr14	74999279	75019180	EIF2B2
3038	chr15	74534713	74562044	ARID3B
6921	chr5	177133243	177141518	NSD1
3671	chr17	42672622	42673648	PLEKHH3
3059	chr15	78872714	78933949	MORF4L1
1699	chr11	85959164	86071242	PICALM
60	chr1	113926949	113939052	HIPK1-AS1
9246	chrX	153925935	153929182	ARHGAP4
7971	chr7	106105974	106114684	SYPL1
3209	chr16	22430750	22447196	RRN3P3
5774	chr22_KB663609v1_alt	503	3705	SMDT1
4580	chr1_GL383520v2_alt	78517	80555	ATP6V1G3
4801	chr2	195657263	195680197	SLC39A10
9325	chrX	49154949	49157537	MAGIX
173	chr1	151007409	151012058	PRUNE1
4684	chr2	142871775	143008420	KYNU
5357	chr20	41338825	41342866	LPIN3
3811	chr17	60599154	60609099	PPM1D
4336	chr19	40431165	40434937	SERTAD1
5405	chr20	50114770	50155468	TMEM189
5629	chr22	21665011	21668566	PPIL2
5063	chr2	43218385	43231132	ZFP36L2
6506	chr4	25858470	25862891	SEL1L3
2593	chr14	24034356	24037585	DHRS4L1
1872	chr12	121775228	121804139	RHOF
7508	chr6	26439582	26445202	BTN3A3
3185	chr16	19494885	19521852	GDE1
5151	chr2	670044	680268	TMEM18
944	chr10	101584635	101592942	DPCD
3592	chr17	29572228	29579221	TP53I13
1630	chr11	66674770	66679348	RBM4B
4763	chr2	177617816	177620702	TTC30A
6827	chr5	142360171	142362073	SPRY4-AS1
696	chr1	37477805	37486813	MIR6732
9002	chr9	34649846	34656370	IL11RA

9260	chrX	15789395	15791591	ZRSR2
6541	chr4	39521932	39530784	UGDH
7756	chr6	84617169	84619724	TBX18-AS1
1100	chr10	31030105	31034337	ZNF438
971	chr10	106441461	106443391	LOC105378470
2209	chr12	74536724	74553201	ATXN7L3B
8583	chr8	29331542	29342689	DUSP4
6647	chr4	82890525	82893028	SEC31A
4752	chr2	174263236	174265409	OLA1
8994	chr9	33774917	33777835	PRSS3
6051	chr3	183323751	183324862	B3GNT5
3141	chr16	10943308	10948037	CLEC16A
5831	chr3	11564899	11574268	VGLL4
1671	chr11	73754413	73762035	RAB6A
3806	chr17	59875450	59880358	TUBD1
3203	chr16	22005935	22093534	MOSMO
3401	chr16	69528741	69532184	MIR1538
7791	chr6_GL000250v2_alt	1845138	1846859	LINC02569
8135	chr7	20327506	20353679	LOC101927811
7511	chr6	26536001	26549040	HMGN4
206	chr1	153984862	153987593	RAB13
7736	chr6	7725079	7727243	BMP6
6796	chr5	138932689	138945452	CTNNA1
1144	chr10	52316741	52321881	DKK1
1115	chr10	3667381	3669455	KLF6
984	chr10	112250256	112252963	TECTB
8460	chr8	125654511	125656686	TRIB1
8402	chr8	102789204	102792208	GASAL1
4930	chr2	231509871	231515937	LINC00471
682	chr1	34761444	34772622	GJB4
5608	chr22	17036364	17061873	CECR7
6228	chr3	49338366	49341984	USP4
8702	chr8	85244922	85247400	CA13
1230	chr10	75294300	75295211	ZNF503-AS1
6254	chr3	51942313	51943583	PARP3
4428	chr19	47226655	47242957	BBC3
1303	chr10	96827991	96846774	LCOR
702	chr1	3792288	3797522	LRRC47
2853	chr15	34337248	34338848	SLC12A6
2439	chr13	46553926	46564732	LRCH1
7471	chr6	17392040	17397424	CAP2
1598	chr11	65180327	65183541	LOC728975
699	chr1	37579843	37596802	GNL2
5471	chr20	62848594	62858216	TCFL5

4437	chr19	47777337	47780213	SELENOW
3749	chr17	48874045	48875893	ATP5MC1
6425	chr4	153464969	153471331	TMEM131L
8026	chr7	129957971	129959742	UBE2H
3426	chr16	74292770	74309594	PSMD7
1325	chr11	102305788	102307800	BIRC3
4792	chr2	190632698	190649024	NAB1
1774	chr12	104108340	104139436	
3410	chr16	70452995	70458811	FUK
1030	chr10	125865779	125897904	DHX32
7719	chr6	73307399	73311173	KHDC1
7348	chr6	136820983	136825963	PEX7
6682	chr4	99938534	99953222	DNAJB14
6401	chr4	140095074	140097092	LOC101927516
1878	chr12	122414439	122424427	CLIP1
1866	chr12	121242957	121244277	CAMKK2
4023	chr17_KI270908v1_alt	1288755	1292465	WNT3
4037	chr18	13749313	13753401	RNMT
4073	chr18	3664301	3667676	DLGAP1-AS2
7072	chr5	6362189	6385900	MED10
2771	chr14	74707208	74718131	AREL1
7649	chr6	43365369	43372431	ZNF318
9166	chrX	101050657	101052974	TRMT2B
1922	chr12	132135848	132138916	DDX51
8195	chr7	36390625	36421700	KIAA0895
5538	chr21	36973790	36978176	HLCS
86	chr1	120145072	120159409	SEC22B
2040	chr12	49262563	49276816	TUBA1C
6152	chr3	31641421	31670510	OSBPL10-AS1
6354	chr4	121668358	121692024	ANXA5
2092	chr12	53984142	53991006	HOXC-AS3
2434	chr13	45219095	45240954	KCTD4
6386	chr4	13700892	13703390	LINC01182
4643	chr2	113885740	113905316	LOC101060091
3797	chr17	58350556	58355339	SUPT4H1
2001	chr12	38900753	38906478	CPNE8
485	chr1	220143055	220154493	MIR194-1
7456	chr6	163409412	163477718	QKI
3316	chr16	4470931	4480099	NMRAL1
5708	chr22	40502372	40513644	LOC101927257
8703	chr8	8635018	8636100	CLDN23
1564	chr11	62669177	62680643	LBHD1
3788	chr17	56786224	56788515	C17orf67
8806	chr9	114586530	114597747	ATP6V1G1

5744	chr22	45070446	45075284	PHF21B
4156	chr19	11504725	11506912	ZNF653
8174	chr7	29610279	29626005	PRR15
2343	chr13	113392901	113412719	LOC101928841
2098	chr12	55720891	55739793	CD63
4230	chr19	18384130	18391372	MIR3189
2706	chr14	61076060	61078825	TRMT5
8986	chr9	33031669	33044094	DNAJA1
5641	chr22	27717080	27719350	MN1
255	chr1	159888903	159891071	MIR4259
2929	chr15	51828527	51876837	TMOD3
9233	chrX	136247789	136252155	MAP7D3
1857	chr12	120442306	120448641	TRIAP1
9011	chr9	36214723	36223780	CLTA
5421	chr20	51032513	51038321	KCNG1
7123	chr5	75566588	75596165	ANKDD1B
2676	chr14	52705531	52727204	PSMC6
2895	chr15	43509160	43511256	TP53BP1
4136	chr18	9827031	9832594	TXNDC2
3846	chr17	67430287	67435665	MIR548AA2
5318	chr20	35261632	35287426	MMP24OS
8069	chr7	148696071	148822502	CUL1
3251	chr16	2967909	2972510	PAQR4
3336	chr16	51879868	51882961	LINC01571
5967	chr3	150395470	150411145	TSC22D2
8601	chr8	38168276	38177440	BAG4
7982	chr7	107986060	107986762	LAMB1
4957	chr2	238191777	238204940	ILKAP
3140	chr16	1080923	1092179	SSTR5
6497	chr4	2004908	2010921	NELFA
5937	chr3	142223884	142226897	GK5
697	chr1	37510728	37516665	MEAF6
2529	chr13	99605764	99608346	CLYBL
4793	chr2	190681726	190702801	NAB1
168	chr1	150600831	150605901	MCL1
1384	chr11	119018939	119022545	
3957	chr17	81349382	81350815	TMEM105
831	chr1	66943366	66998149	MIER1
678	chr1	32975247	32982529	RNF19B
8101	chr7	152461317	152467381	LINC01003
6959	chr5	19530363	19534016	CDH18
838	chr1	68382063	68497400	RPE65
2155	chr12	64608260	64610570	RASSF3
7550	chr6	28582946	28588770	ZBED9

290	chr1	16609766	16615357	NBPF1
2338	chr13	112716843	112718207	ATP11A
262	chr1	161017026	161022428	F11R
1299	chr10	96047620	96067694	MIR3157
6430	chr4	156909042	156912864	PDGFC
7872	chr6_GL000254v2_alt	1859695	1861471	LINC02569
1090	chr10	28518750	28619579	WAC
7628	chr6	3907711	3914563	FAM50B
3523	chr17	1648859	1650950	RILP
6371	chr4	127781179	127796734	HSPA4L
6094	chr3	192864440	192918800	MB21D2
2744	chr14	68798391	68801713	ZFP36L1
482	chr1	219173197	219244762	LYPLAL1
4372	chr19	43468611	43476800	LYPD3
8612	chr8	41576458	41581873	LOC102723729
5722	chr22	41684661	41691396	SNU13
7419	chr6	151380868	151416497	ZBTB2
4030	chr18	12317501	12332070	TUBB6
3541	chr17	18676706	18683729	ZNF286B
5469	chr20	62772109	62778059	LINC00659
5671	chr22	32049708	32052095	SLC5A1
362	chr1	183466167	183513785	SMG7
2328	chr13	110560233	110563157	RAB20
6830	chr5	143007862	143012666	ARHGAP26-AS1
3289	chr16	3114404	3117136	ZNF205-AS1
688	chr1	35265914	35294432	ZMYM4
7271	chr6	11337239	11344112	NEDD9
8129	chr7	18507667	18622914	HDAC9
3878	chr17	7379741	7382994	TNK1
5299	chr20	31653054	31700628	COX4I2
2527	chr13	99496861	99529361	TM9SF2
9119	chr9	91398316	91420320	NFIL3
1777	chr12	104271130	104274736	TXNRD1
7590	chr6	33839239	33841732	MLN
4506	chr19	57349986	57354055	ZNF304
8999	chr9	34242564	34262146	UBAP1
4769	chr2	180872573	180873761	UBE2E3
7338	chr6	135011409	135055638	HBS1L
8334	chr7	811937	815121	SUN1
6897	chr5	172776919	172779805	DUSP1
2067	chr12	51882676	51887181	ANKRD33
8266	chr7	65769652	65772021	LOC441242
8067	chr7	145623492	145626609	CNTNAP2
4809	chr2	197703864	197717501	MARS2

6901	chr5	172869348	172873376	ERGIC1
5443	chr20	56388452	56430562	CASS4
3530	chr17	17273899	17282470	COPS3
4556	chr19	9548538	9585432	ZNF121
4453	chr19	48873520	48885098	PPP1R15A
6328	chr4	107813577	107846933	SGMS2
1697	chr11	83552677	83555649	DLG2
7695	chr6	57316393	57323766	PRIM2
1666	chr11	72567267	72571293	LINC01537
1903	chr12	12531834	12564825	DUSP16
4597	chr2	102257342	102260097	IL1RL1
5027	chr2	3510052	3521508	ADI1
7537	chr6	28101830	28106945	ZSCAN12P1
9298	chrX	45445299	45447675	LINC01204
1447	chr11	18391762	18402494	LDHA
2538	chr14	100971705	100975674	SNORD114-16
1250	chr10	80453397	80457339	TSPAN14
3930	chr17	79073345	79076407	ENGASE
9313	chrX	48537592	48542122	TBC1D25
2855	chr15	34933639	34943551	AQR
2400	chr13	35890865	35893488	DCLK1
5056	chr2	39480782	39487401	LOC728730
1433	chr11	1335708	1337264	TOLLIP-AS1
4558	chr19	9653837	9679904	ZNF562
4142	chr19	1027186	1029482	CNN2
8741	chr8	98992904	99021869	VPS13B
8809	chr9	114893847	114897090	TNFSF8
1148	chr10	5290508	5292710	LINC02561
1868	chr12	121397608	121406094	RNF34
9304	chrX	46837249	46841327	RP2
3718	chr17	4544263	4557228	MYBBP1A
2185	chr12	69238631	69294825	MIR1279
4341	chr19	4119111	4125094	MAP2K2
7323	chr6	133484067	133507554	EYA4
5281	chr20	18523561	18539451	SEC23B
6983	chr5	33311239	33314689	LINC02160
345	chr1	179135897	179145944	ABL2
8935	chr9	136874747	136884012	TRAF2
5010	chr2	29106478	29109563	CLIP4
8264	chr7	65750953	65766442	SNORA15B-1
6502	chr4	24528246	24585985	DHX15
6279	chr3	69198810	69201318	LMOD3
8042	chr7	133703845	133710835	LOC101928861
8563	chr8	24155208	24157704	ADAM28

5154	chr2	68051337	68064532	C1D
5327	chr20	35771380	35787934	PHF20
2032	chr12	48668263	48682853	KANSL2
6919	chr5	176552330	176554995	CDHR2
5234	chr2	94576633	94579220	LOC100133920
9031	chr9	39801231	39815211	GLIDR
5236	chr2	9528319	9558545	ADAM17
6769	chr5	134393795	134401066	CDKN2AIPNL
6556	chr4	41912408	41914504	TMEM33
1347	chr11	108007814	108012627	CUL5
8946	chr9	137307626	137309231	NRARP
2798	chr14	85526979	85530143	FLRT2
2177	chr12	6749378	6755555	MLF2
7122	chr5	75489249	75542680	COL4A3BP
5628	chr22	20993747	21005205	THAP7-AS1
6696	chr5	10560167	10575148	ANKRD33B
5414	chr20	50509168	50530543	PTPN1
6801	chr5	139560296	139571536	UBE2D2
447	chr1	209605511	209659285	MIR4260
3483	chr16	89504765	89509632	SPG7
7486	chr6	22827724	22829366	LOC105374972
966	chr10	104253886	104271936	MIR4482
4467	chr19	49926396	49930633	ATF5
3433	chr16	74741912	74745227	FA2H
4074	chr18	36826917	36831977	TPGS2
7274	chr6	115958972	115960825	TPI1P3
7254	chr6	109091673	109105971	CEP57L1
3446	chr16	83926421	83928821	OSGIN1
4013	chr17_KI270857v1_alt	2828437	2836187	PIP4K2B
5658	chr22	30417715	30430057	MTFP1
9331	chrX	53080790	53090705	TSPYL2
3743	chr17	48054671	48076423	NFE2L1
2425	chr13	42719754	42722239	FAM216B
2493	chr13	75523612	75538436	COMMD6
8538	chr8	17921010	17929088	PCM1
8355	chr7	92764240	92767726	CDK6
2742	chr14	68693958	68696624	LOC100996664
692	chr1	36222829	36237455	THRAP3
8033	chr7	130666598	130670511	COPG2
5292	chr20	25682870	25701267	ZNF337
2289	chr12	96906307	96913781	NEDD1
6291	chr3	8831371	8873145	OXTR
6654	chr4	83534576	83549829	GPAT3
6882	chr5	16437401	16488745	ZNF622

4741	chr2	171998384	172001826	METAP1D
8582	chr8	29124917	29127098	KIF13B
6116	chr3	196491376	196505203	RNF168
3543	chr17	18855549	18857561	PRPSAP2
9296	chrX	41331166	41354060	DDX3X
7171	chr5	90508835	90516606	LYSMD3
5670	chr22	31737128	31759464	PRR14L
7830	chr6_GL000252v2_alt	2003820	2009981	IER3
2315	chr13	106150620	106152212	LINC00460
7204	chr5_GL339449v2_alt	16627	33113	CDK7
3621	chr17	35566082	35569313	LINC02001
6890	chr5	171449800	171452090	FGF18
796	chr1	58777844	58781543	JUN
434	chr1	207036653	207057390	YOD1
9267	chrX	16970301	16972060	REPS2
3774	chr17	51114203	51123681	SPAG9
3647	chr17	39980580	39987719	PSMD3
3759	chr17	49786933	49799844	KAT7
4421	chr19	46848746	46851568	AP2S1
8506	chr8	143943383	143951431	MIR661
6579	chr4	55174807	55191251	KDR
2076	chr12	52907439	52909163	KRT8
6163	chr3	36678152	36680832	DCLK3
8715	chr8	92964853	92967124	TRIQK
6948	chr5	181173487	181174417	LINC01962
7029	chr5	52986729	53017348	ITGA2
214	chr1	154982448	154985337	FLAD1
7562	chr6	30515249	30517077	LINC02569
653	chr1	28576634	28581824	SNORD99
5620	chr22	20019882	20023957	TANGO2
4543	chr19	7920320	7921481	SNAPC2
2429	chr13	44430741	44439064	TSC22D1
5012	chr2	30145764	30147919	YPEL5
2703	chr14	60135643	60142736	DHRS7
6989	chr5	35874833	35887334	IL7R
4277	chr19	3358748	3361755	NFIC
5587	chr21	45072457	45105385	ADARB1
6232	chr3	49468761	49472864	DAG1
2734	chr14	67819868	67822808	RAD51B
7165	chr5	87302206	87406814	LOC644285
5590	chr21	46287327	46288937	YBEY
3158	chr16	1413261	1422699	C16orf91
589	chr1	23819794	23826512	HMGCL
7797	chr6_GL000250v2_alt	3166034	3170963	SNORD48

6999	chr5	38544805	38560439	LIFR
73	chr1	116989139	116991239	CD101
8239	chr7	55005822	55205218	EGFR
956	chr10	102853416	102855636	BORCS7-ASMT
7924	chr6_GL000256v2_alt	2048962	2052151	IER3
7202	chr5	98915290	98956007	LOC100289230
6636	chr4	7960366	7961694	AFAP1
4140	chr19	10095265	10097377	ANGPTL6
8767	chr9	107211826	107214348	RAD23B
6217	chr3	48555458	48597139	MIR711
4855	chr2	205681575	205684206	NRP2
5009	chr2	28894496	28913093	WDR43
7161	chr5	85662848	85673429	NBPF22P
4750	chr2	173939844	173968404	SP3
3807	chr17	59891557	59901304	RPS6KB1
7802	chr6_GL000251v2_alt	1788457	1809400	HCG17
9021	chr9	37797935	37809828	DCAF10
5297	chr20	31543470	31574699	HM13-AS1
5731	chr22	43010585	43016409	PACSIN2
8022	chr7	128947231	128950297	IRF5
7319	chr6	132812112	132814339	RPS12
7385	chr6	145964629	145965405	SHPRH
1285	chr10	92234774	92241995	CPEB3
408	chr1	202336095	202342896	UBE2T
7300	chr6	125780028	125825101	NCOA7
9215	chrX	129676215	129679595	APLN
2332	chr13	110714092	110717284	ING1
9274	chrX	21925421	21928128	SMS
2114	chr12	56311652	56320609	CNPY2
8664	chr8	66122865	66125159	TRIM55
9048	chr9	6149450	6155742	IL33
7745	chr6	8086899	8103148	EEF1E1
5968	chr3	150412347	150478405	TSC22D2
1610	chr11	65651593	65664650	RELA
4523	chr19	58574563	58576783	CENPBD1P1
3202	chr16	21944445	21982936	UQCRC2
3568	chr17	2507292	2513129	METTL16
2628	chr14	35078180	35086797	LOC101927178
6496	chr4	1977612	1983731	MIR943
8368	chr7	97116344	97121097	SDHAF3
6660	chr4	87389634	87392509	HSD17B11
135	chr1	147148202	147176897	PRKAB2
8472	chr8	128126229	128269218	MIR1208
3150	chr16	11891634	11900109	GSPT1

4408	chr19	45878935	45888186	IRF2BP1
129	chr1	146367144	146371452	RNU1-4
8392	chr8	100788434	100790969	PABPC1
2764	chr14	73697397	73702146	PNMA1
4856	chr2	20577406	20580706	HS1BP3-IT1
8056	chr7	139349461	139393197	LUC7L2
3372	chr16	67182026	67184828	KIAA0895L
917	chr1	93907168	93910205	GCLM
7739	chr6	78861029	78868618	IRAK1BP1
244	chr1	1568897	1577585	SSU72
2615	chr14	31417311	31421914	HEATR5A
6951	chr5	181191052	181200431	LINC01962
7535	chr6	28073824	28100502	ZSCAN12P1
1657	chr11	69944922	69950621	LOC101928443
9206	chrX	120602694	120610333	MCTS1
180	chr1	151279399	151295560	ZNF687
4322	chr19	39383428	39394873	PAF1
3527	chr17	17043814	17082468	MPRIP
2617	chr14	31936993	31938692	LINC02313
442	chr1	207747022	207757182	CD46
6730	chr5	119273782	119296036	TNFAIP8
8613	chr8	41791324	41794086	ANK1
2301	chr13	100391455	100403370	PCCA-AS1
2916	chr15	48876690	48883075	EID1
4974	chr2	24490081	24492118	NCOA1
4827	chr2	200855418	200866199	CLK1
8587	chr8	30077786	30089444	SARAF
997	chr10	11791909	11795350	PROSER2
8169	chr7	2833019	2836084	GNA12
6182	chr3	43683017	43718282	ANO10
7697	chr6	63526805	63531503	PTP4A1
1584	chr11	64357379	64371033	MIR1237
6069	chr3	186071637	186110663	ETV5
1924	chr12	132685747	132688422	POLE
4862	chr2	206764032	206805129	MIR3130-1
6567	chr4	48268371	48270773	TEC
8348	chr7	90596599	90597966	CDK14
6125	chr3	197625049	197629416	LOC220729
8273	chr7	66647660	66663757	LOC100996437
3041	chr15	74953196	74957441	RPP25
50	chr1	112673548	112680459	MOV10
8244	chr7	55568508	55572518	VOPP1
564	chr1	234370520	234389919	COA6
1220	chr10	73740726	73746357	SEC24C

2310	chr13	102729009	102731075	CCDC168
5074	chr2	46294600	46384515	EPAS1
6123	chr3	197163737	197302525	DLG1
286	chr1	165597223	165599879	LOC400794
8168	chr7	27738208	27739718	TAX1BP1
7025	chr5	50666283	50733521	PARP8
1369	chr11	116470244	116473436	LOC101929011
6887	chr5	17118153	17119817	BASP1
1032	chr10	126376366	126378980	ADAM12
2736	chr14	68136691	68143111	RAD51B
1119	chr10	37847825	37859617	ZNF248
7070	chr5	63254851	63257814	IPO11-LRRC70
4482	chr19	54446386	54463632	LENG8
641	chr1	27486922	27491900	WASF2
986	chr10	112439667	112452195	ZDHHC6
7461	chr6	165883256	165988126	LINC00602
1084	chr10	25067633	25070136	ENKUR
2213	chr12	75485922	75512776	KRR1
2698	chr14	58254732	58298852	LINC00216
3641	chr17	39446676	39454220	MED1
2595	chr14	24135563	24138301	PSME1
8149	chr7	24074672	24076887	NPY
1914	chr12	12996664	13001643	HEBP1
7454	chr6	161287857	161312128	AGPAT4
2221	chr12	76019559	76086079	PHLDA1
1383	chr11	118998150	119013851	RPL23AP64
8874	chr9	128688928	128701735	SET
152	chr1	149174219	149194690	NBPF9

Supplementary Data 4, RNA-Seq data of S18 and S26 cells

log2FC	Pvalue	FDR	Style	S18-1	S18-2	S18-3	S26-1	S26-2	S26-3	Symbol
-2.5735	2.29E-05	0.000151	down	3	5	13	48	58	23	BDKRB2
-5.79318	0.0001417	0.000768	down	0	0	0	5	21	8	ZNF709
-1.34045	3.93E-07	3.79E-06	down	259	212	236	380	1001	571	AP5Z1
-4.71725	0.0020658	0.008085	down	0	0	1	14	4	10	ZNF385B
-3.08009	0.0040155	0.014135	down	1	1	3	22	18	3	TNFSF18
-1.80164	2.82E-12	5.98E-11	down	156	155	196	375	701	789	NREP
-1.97872	8.09E-09	1.05E-07	down	25	38	24	82	157	129	SLC37A2
-3.63292	0.0021734	0.00843	down	3	0	0	7	15	18	
-1.32739	0.0059176	0.019645	down	10	28	13	41	54	41	HTR7P1
-2.35828	8.36E-14	2.18E-12	down	44	48	43	159	401	196	MYO15B
-3.42984	1.97E-08	2.37E-07	down	5	4	3	54	40	39	CCDC110
-8.7205	1.55E-12	3.43E-11	down	1	0	0	200	160	106	ABCA12
-1.37288	5.69E-07	5.32E-06	down	97	128	100	200	450	264	PAM16

-2.37961	0.0071015	0.022938	down	2	9	4	55	11	10	CSF3R
-5.51186	0.000312	0.001542	down	0	0	0	9	8	9	
-4.5886	1.69E-16	5.97E-15	down	10	2	1	80	154	104	KLHL30
-1.65208	2.39E-07	2.39E-06	down	38	39	51	149	114	147	MGAT4A
-2.70478	0.0025113	0.009476	down	3	2	2	23	17	7	SPANXB1
-2.3911	9.95E-27	8.71E-25	down	524	464	392	1884	3649	2301	HR
-1.17084	0.0098284	0.030249	down	21	24	16	37	81	32	SMIM2-AS1
-1.75674	0.0075001	0.024034	down	8	6	5	29	26	12	KLLN
-1.80931	3.08E-17	1.16E-15	down	147	169	157	411	769	593	ALOX5
-2.6487	0.0062424	0.020526	down	4	2	0	12	20	9	
-2.05575	1.24E-12	2.77E-11	down	67	47	41	166	283	240	FAM83H-AS1
-1.02796	8.57E-06	6.17E-05	down	308	245	227	409	766	537	STK11IP
-1.13646	2.56E-06	2.08E-05	down	127	152	133	213	422	336	ACYP1
-2.59754	0.0099242	0.030494	down	1	4	1	4	19	17	
-5.37441	0.0009095	0.003948	down	0	0	0	7	15	3	
-5.1734	0.0013066	0.005426	down	0	0	0	7	9	5	LOC102724601
-1.52739	0.0053113	0.017933	down	11	10	11	16	56	29	
-2.54634	7.67E-06	5.61E-05	down	7	8	12	79	56	25	DNAJC15
-1.56498	4.97E-06	3.79E-05	down	21	21	32	70	87	71	CALML4
-1.36325	4.63E-13	1.08E-11	down	438	450	659	1057	1717	1407	MTRF1
-2.07458	9.33E-11	1.60E-09	down	156	108	275	932	709	633	PLS1
-3.21252	1.06E-24	8.04E-23	down	85	85	104	1183	811	581	EGR1
-1.15566	3.64E-05	0.000228	down	224	271	242	307	854	625	MGST2
-2.38097	5.10E-13	1.19E-11	down	39	28	64	268	255	174	GPR75
-1.32855	2.45E-05	0.00016	down	40	85	91	137	241	195	
-1.86282	8.36E-10	1.26E-08	down	47	29	47	165	166	132	HECW1
-8.5947	4.05E-12	8.39E-11	down	0	0	0	52	107	70	AOC1
-4.81307	9.49E-19	4.12E-17	down	2	6	3	100	144	84	AKR1B15
-1.60611	1.11E-20	5.79E-19	down	940	1122	1072	2789	4422	2945	ALS2CL
-1.21003	3.97E-07	3.83E-06	down	231	269	462	721	888	687	
-2.39111	0.0006003	0.002743	down	3	3	9	37	26	16	
-1.12763	0.0012413	0.005203	down	47	53	81	164	102	131	ZNF611
-9.1862	7.15E-13	1.64E-11	down	0	0	0	173	97	54	CAPN8
-3.40308	3.88E-05	0.000242	down	1	4	2	16	50	16	ZC3H12D
-3.14536	4.92E-06	3.76E-05	down	4	8	2	16	78	45	IBA57-AS1
-3.51048	0.0019019	0.007534	down	1	1	1	9	20	8	LINC01132
-1.3048	0.0131672	0.038487	down	9	10	10	22	27	26	C1orf220
-3.87978	3.41E-26	2.85E-24	down	12	6	14	137	202	156	FAM134B
-5.04199	1.18E-34	1.55E-32	down	15	20	10	235	823	579	SOX15
-5.90195	7.92E-05	0.000458	down	0	0	0	13	17	5	
-1.20867	3.97E-05	0.000247	down	304	312	190	403	940	694	C16orf74
-1.35883	1.25E-12	2.80E-11	down	278	276	316	556	1030	791	COX20
-5.36304	0.000124	0.000682	down	0	1	0	19	18	9	
-1.48535	1.59E-07	1.65E-06	down	76	58	56	128	256	188	LCMT2

-3.22028	5.92E-77	3.63E-74	down	569	565	753	4568	7363	6576	EPCAM
-3.59738	0.0012949	0.005388	down	2	0	1	11	14	13	IGSF11
-3.18103	1.33E-09	1.94E-08	down	9	10	8	32	136	101	CRAT
-5.17195	5.15E-37	7.95E-35	down	5	5	11	210	275	302	NPNT
-2.00997	2.17E-06	1.79E-05	down	8	14	20	51	67	58	SHOX2
-1.55269	0.0018898	0.007491	down	26	13	9	35	77	42	
-2.36742	1.81E-06	1.52E-05	down	22	29	30	223	152	50	ZNF114
-2.07714	9.04E-07	8.12E-06	down	16	10	12	46	65	58	
-1.1921	2.47E-08	2.93E-07	down	380	306	527	934	969	942	GEN1
-5.52892	0.0004379	0.002073	down	0	0	0	7	5	14	
-1.03613	0.0023328	0.008932	down	37	39	35	80	105	56	ZNF503-AS2
-2.49158	0.0127225	0.037394	down	0	2	4	7	13	15	
-2.00266	0.0140313	0.040588	down	5	5	1	8	20	20	
-1.11424	6.91E-10	1.05E-08	down	534	577	494	995	1487	1210	ATP8B3
-1.43896	1.21E-05	8.44E-05	down	35	35	42	70	163	94	RAET1G
-4.27291	0.006199	0.020408	down	1	0	0	4	8	10	
-1.96132	0.0177786	0.049301	down	3	2	4	9	16	12	C6orf99
-4.13219	0.0115813	0.03464	down	0	0	1	4	14	3	ZFR2
-5.31477	0.0001158	0.000641	down	0	1	0	14	18	13	ACE2
-1.03814	4.05E-12	8.39E-11	down	520	545	601	1014	1428	1160	PTK2B
-3.75379	0.007302	0.023501	down	0	2	0	11	14	4	ZNF608
-3.38781	7.56E-94	6.53E-91	down	224	258	244	2231	3210	2591	TJP3
-8.42438	1.10E-10	1.86E-09	down	0	0	0	23	71	108	DPP6
-4.93225	4.12E-05	0.000255	down	0	0	2	17	22	24	INPP5D
-5.91841	7.65E-46	1.82E-43	down	12	3	3	333	462	356	MYH14
-1.08733	0.0018656	0.00741	down	96	129	73	117	341	239	ETFB
-4.62656	0.0023015	0.008831	down	0	0	1	11	13	4	TNFRSF8
-1.59699	4.52E-05	0.000277	down	24	28	18	48	111	71	VAMP5
-4.8126	1.01E-29	1.08E-27	down	14	11	5	159	436	327	PRSS36
-1.94492	5.01E-06	3.82E-05	down	13	18	13	38	81	63	
-1.26482	1.07E-09	1.59E-08	down	496	451	694	970	1621	1546	IFIT3
-1.55874	2.81E-11	5.17E-10	down	136	127	237	420	616	497	
-4.22338	9.29E-06	6.65E-05	down	2	2	0	35	29	14	LINC00987
-3.62416	0.0054566	0.018339	down	0	3	0	7	32	4	
-1.58748	5.17E-23	3.44E-21	down	320	288	345	894	1189	925	SLC45A4
-1.18349	0.000136	0.00074	down	36	51	52	74	151	112	LOC105378405
-3.47669	0.0047648	0.016325	down	0	0	3	13	15	6	C1orf234
-1.21203	6.10E-05	0.000363	down	62	52	40	110	133	133	ARHGEF35
-2.11055	5.06E-05	0.000307	down	9	7	12	53	38	32	SPRY3
-5.73358	2.65E-21	1.48E-19	down	4	1	3	99	228	132	KLK10
-2.18958	2.38E-07	2.38E-06	down	11	12	14	45	84	51	LGALS9
-4.7042	0.0074041	0.023782	down	0	0	0	6	6	3	LOC101928738
-1.16369	0.0027827	0.010344	down	93	69	37	108	256	129	CPLX1
-6.56953	2.89E-07	2.84E-06	down	0	0	1	30	51	28	

-4.49916	0.0132765	0.038777	down	0	0	0	5	5	3	
-1.07858	9.88E-05	0.000557	down	80	117	74	175	257	179	ASIC3
-1.02742	9.98E-07	8.87E-06	down	119	124	176	265	342	283	
-1.38309	5.53E-07	5.17E-06	down	233	190	162	320	749	587	COG8
-1.74661	0.0010633	0.004534	down	8	14	10	19	59	39	
-2.31109	0.0151447	0.043204	down	2	1	3	9	12	10	
-2.58577	4.49E-06	3.45E-05	down	8	12	2	36	55	51	RNF223
-6.23295	9.45E-06	6.75E-05	down	0	0	0	13	13	17	LDLRAD1
-1.07771	0.0044647	0.015449	down	32	34	22	60	90	49	LOC440600
-6.33569	5.73E-06	4.30E-05	down	0	0	0	10	23	15	LTC4S
-1.84783	9.68E-09	1.23E-07	down	40	21	53	125	164	137	COL4A4
-3.6741	1.57E-08	1.93E-07	down	5	3	5	84	59	26	PXDN
-1.36363	0.0028249	0.010482	down	23	20	13	28	74	55	NRL
-1.98559	0.0004247	0.002021	down	18	12	4	26	68	54	CAPS
-1.65943	0.0073624	0.023657	down	6	5	9	18	19	28	
-3.69375	3.93E-11	7.09E-10	down	6	10	10	167	138	41	FAM83E
-7.12873	1.18E-26	1.02E-24	down	3	0	3	166	434	305	CKMT1B
-3.63139	0.010952	0.03307	down	1	0	1	2	14	11	
-5.06598	1.14E-08	1.43E-07	down	1	3	0	23	79	46	MMP12
-7.61258	6.86E-09	8.99E-08	down	1	0	0	33	168	38	PRSS56
-9.94458	7.98E-21	4.28E-19	down	2	0	0	394	1118	640	TNNI2
-1.81437	1.35E-12	3.00E-11	down	55	46	64	148	257	209	FIRRE
-8.98308	3.68E-12	7.66E-11	down	0	0	0	28	160	120	TNNT3
-1.32241	0.0008623	0.003764	down	28	16	51	75	104	67	ERV3-1
-4.36061	9.98E-49	2.92E-46	down	15	19	22	322	417	461	EPB41L4A
-1.26404	4.64E-05	0.000284	down	73	67	47	152	211	117	PNPLA7
-1.83858	1.18E-17	4.65E-16	down	107	111	103	303	502	416	SLC25A20
-5.7128	8.12E-07	7.39E-06	down	1	0	1	17	53	43	FOXJ1
-2.16783	1.01E-15	3.22E-14	down	445	311	231	1298	2086	1398	LSR
-1.64154	4.56E-10	7.11E-09	down	63	57	51	174	192	192	H19
-6.28507	3.73E-08	4.28E-07	down	1	0	1	68	68	26	PSAPL1
-1.21629	0.0149797	0.042823	down	8	19	14	26	35	39	MAST1
-2.86356	0.0015277	0.006216	down	2	3	1	8	19	20	LOC150051
-1.97229	0.0022409	0.008653	down	6	13	2	29	33	26	
-2.4946	0.0083196	0.026252	down	2	3	1	7	19	11	
-5.46156	0.000632	0.00287	down	0	0	0	3	16	8	UNC5B-AS1
-1.36665	0.0004738	0.002221	down	30	34	36	42	126	110	DGCR5
-1.09398	0.0079079	0.025114	down	17	18	25	30	61	45	
-3.55668	0.0019147	0.007574	down	0	2	1	14	9	13	
-3.21719	1.29E-20	6.59E-19	down	34	21	60	225	435	456	B4GALNT3
-1.64166	1.53E-11	2.93E-10	down	69	58	72	161	280	218	STON2
-1.60745	4.56E-05	0.000279	down	15	17	18	42	66	53	NPAS1
-1.63708	0.0140606	0.040654	down	12	3	5	17	32	18	TFCP2L1
-1.46681	4.97E-09	6.65E-08	down	99	93	75	195	341	255	ZNF691

-1.32801	3.14E-08	3.65E-07	down	121	105	98	290	321	242	ERMAP
-1.74928	1.26E-13	3.20E-12	down	390	238	277	916	1414	915	ZC3H4
-1.04386	0.0100895	0.030898	down	20	25	32	54	43	65	ZNF713
-1.82482	0.0019122	0.007567	down	10	7	4	24	31	24	SMPDL3B
-1.6487	0.0001041	0.000584	down	37	19	16	64	120	61	TPRG1
-2.35095	8.49E-09	1.10E-07	down	25	35	18	77	238	126	CHRNA10
-1.70226	1.94E-06	1.62E-05	down	39	17	34	77	138	96	TP53I3
-1.8074	3.42E-22	2.05E-20	down	428	423	373	1183	1931	1461	MST1R
-5.4483	0.0010535	0.004501	down	0	0	0	6	19	2	GRM2
-1.13764	0.000443	0.002094	down	103	122	75	133	347	243	PBX4
-1.0983	0.0016522	0.006656	down	30	29	31	46	80	78	PITX2
-3.40585	3.56E-47	9.06E-45	down	76	77	107	899	862	1065	PRSS12
-1.07609	4.86E-07	4.61E-06	down	1253	1271	1069	1855	3760	2577	ATP13A1
-1.37762	0.0087681	0.027453	down	14	18	11	21	72	31	CCER2
-1.52277	3.01E-05	0.000192	down	20	24	22	48	87	67	
-5.91188	2.19E-10	3.57E-09	down	2	0	1	65	82	43	
-4.10549	0.0168626	0.047117	down	1	0	0	13	3	2	
-1.99343	0.0012342	0.005176	down	9	3	6	19	32	25	KLRG1
-1.76527	0.0002572	0.001303	down	9	17	12	32	72	36	ADAT3
-1.01657	0.0019231	0.007604	down	101	87	63	168	264	116	TAPBPL
-2.66684	1.56E-25	1.25E-23	down	110	112	80	704	787	531	TMEM139
-1.73373	0.0001342	0.000732	down	18	39	20	44	113	120	TMEM52
-1.96195	1.77E-15	5.62E-14	down	65	56	86	209	322	314	TMEM117
-5.83521	8.91E-05	0.000508	down	0	0	0	13	14	6	PIK3C2G
-2.2067	2.61E-13	6.30E-12	down	39	43	54	254	229	163	GCA
-1.27401	8.23E-08	8.96E-07	down	240	206	392	696	725	653	IFIH1
-2.54861	1.96E-14	5.54E-13	down	22	35	41	232	185	168	HPGD
-6.60933	3.89E-07	3.75E-06	down	1	0	0	48	38	22	ITGB6
-1.95775	1.73E-13	4.28E-12	down	171	158	172	393	1064	661	REEP6
-2.34935	4.50E-24	3.23E-22	down	169	127	135	814	788	680	C6orf132
-2.2999	8.59E-20	4.06E-18	down	344	355	214	1249	1937	1640	DUSP9
-1.68534	5.81E-21	3.16E-19	down	771	748	684	1916	3122	2517	SLC6A8
-1.90262	1.25E-12	2.79E-11	down	73	62	54	207	347	204	
-1.27285	0.0012316	0.005169	down	21	19	23	36	77	50	
-1.23291	0.0001693	0.000897	down	29	34	62	87	114	103	LOC100506282
-2.16981	0.0004855	0.002269	down	2	9	7	24	39	23	
-2.77518	0.0120894	0.035925	down	1	1	3	17	3	13	
-3.00809	0.0018542	0.007374	down	1	3	1	10	24	10	
-4.76293	8.44E-21	4.49E-19	down	4	3	11	177	241	93	GCNT3
-2.5712	1.89E-05	0.000127	down	3	4	9	27	46	27	
-1.13656	0.000187	0.000981	down	635	489	1028	2050	1392	1284	SERINC5
-1.58408	0.0008511	0.003723	down	17	7	21	36	69	38	POLN
-7.32683	8.50E-09	1.10E-07	down	0	0	1	81	49	45	LUM
-1.34643	1.34E-12	2.98E-11	down	512	506	754	1535	1723	1398	F2RL1

-1.67926	0.0005566	0.002563	down	6	16	24	50	65	38	DISP2
-4.46726	1.03E-30	1.18E-28	down	8	9	9	139	247	224	SCGB3A2
-3.94059	0.0003086	0.001528	down	0	1	2	14	13	20	SPINK1
-1.71535	3.16E-18	1.31E-16	down	251	206	333	719	1163	849	DUSP6
-2.26812	0.0007601	0.003375	down	12	9	1	23	66	30	LOXL3
-1.20475	3.57E-09	4.88E-08	down	468	517	479	821	1634	1173	HTRA2
-1.62238	5.20E-06	3.94E-05	down	19	29	30	57	102	95	HYKK
-3.10341	9.09E-05	0.000517	down	2	2	3	13	28	23	
-1.7793	4.80E-09	6.47E-08	down	159	182	281	884	538	710	GALNT3
-1.29987	0.0017089	0.006865	down	18	14	21	39	62	37	C15orf62
-4.57094	7.68E-13	1.75E-11	down	3	3	2	66	87	48	LPPR3
-1.83061	0.0007258	0.003248	down	9	9	8	39	28	28	BAALC-AS1
-1.52651	0.0007781	0.003449	down	10	24	15	46	65	39	MIR210HG
-1.35922	0.0083764	0.026414	down	13	36	14	31	94	54	ADAP1
-1.26484	0.0025099	0.009474	down	23	15	24	48	75	35	ANKRD34A
-1.01353	0.0008546	0.003737	down	39	48	69	79	148	106	
-2.27794	0.0040425	0.014217	down	1	4	5	10	20	21	
-2.23177	7.84E-07	7.15E-06	down	12	10	13	48	88	40	
-4.71014	0.0123203	0.03646	down	0	0	0	10	2	2	
-2.50756	0.0015158	0.006172	down	9	0	3	18	32	23	LINC01521
-1.83728	0.0001584	0.000846	down	6	13	19	34	69	41	PPM1N
-1.90448	0.0075247	0.024099	down	6	1	9	14	29	20	GDF9
-3.04049	0.0131511	0.038446	down	1	0	2	10	8	7	
-2.64575	0.0003891	0.001869	down	4	2	3	15	27	18	
-1.08969	0.002402	0.009139	down	43	36	54	118	75	93	ARRDC4
-1.00652	5.12E-07	4.82E-06	down	234	228	201	406	586	423	TERT
-1.09972	9.25E-12	1.82E-10	down	925	772	957	1566	2450	1997	EMP2
-1.43038	3.16E-06	2.51E-05	down	45	71	65	110	254	162	PRR19
-1.32728	9.56E-05	0.00054	down	25	31	47	90	86	89	JAKMIP3
-3.49727	9.93E-07	8.83E-06	down	3	5	1	20	59	33	
-1.89695	0.0024797	0.009371	down	5	4	10	29	24	19	
-1.41613	8.89E-06	6.38E-05	down	530	373	688	1979	1336	951	FN1
-1.41765	9.38E-05	0.000531	down	23	18	31	54	90	59	
-1.94262	0.0130594	0.038213	down	4	9	2	7	31	26	
-2.35051	0.007437	0.023871	down	2	3	2	14	12	11	
-2.14896	0.0002244	0.001154	down	6	4	15	25	63	30	
-1.21637	0.0106545	0.032305	down	15	15	22	27	74	30	
-1.64215	8.44E-06	6.09E-05	down	22	21	26	47	103	80	
-1.32914	6.04E-07	5.63E-06	down	197	190	117	364	557	438	JMJD4
-1.75176	4.03E-10	6.34E-09	down	43	66	67	196	271	158	RAET1E
-2.88448	0.0010616	0.004528	down	3	2	1	11	17	19	
-6.05532	3.84E-06	2.99E-05	down	1	0	0	18	32	26	FLG-AS1
-3.9109	0.0160535	0.045284	down	1	0	0	4	6	7	
-1.97519	5.31E-12	1.08E-10	down	49	35	41	138	240	147	TCONS_00029157

-1.16316	1.16E-06	1.01E-05	down	99	109	99	180	335	222	GALT
-1.06963	1.26E-07	1.33E-06	down	363	417	410	604	1098	958	MFGE8
-1.06453	1.52E-11	2.91E-10	down	646	562	654	1222	1643	1234	CHMP3
-1.24374	7.03E-09	9.21E-08	down	116	109	153	247	351	338	IFT88
-1.92108	0.001805	0.0072	down	2	14	8	24	42	31	
-1.17252	1.82E-05	0.000122	down	50	62	83	113	180	168	
-4.47748	0.0138428	0.040141	down	0	0	0	3	5	5	
-2.12078	4.29E-16	1.43E-14	down	45	48	41	160	259	201	LOC100506100
-4.12143	0.0017206	0.006903	down	0	2	0	16	11	9	LAMB4
-1.24874	1.09E-07	1.16E-06	down	163	176	149	334	585	328	RELL2
-1.02961	9.69E-14	2.50E-12	down	1309	1232	1436	2443	3410	2693	KIAA0141
-1.91536	0.0005257	0.00244	down	6	13	6	33	41	26	SP8
-1.26832	2.28E-05	0.00015	down	47	61	46	90	168	139	DUSP28
-1.25547	2.27E-08	2.72E-07	down	290	259	248	558	964	523	SH3TC1
-10.7239	4.48E-24	3.22E-22	down	2	0	0	683	1917	1093	SYT8
-3.00364	1.01E-26	8.83E-25	down	70	65	138	788	709	720	RALGAPA2
-1.38784	4.67E-06	3.58E-05	down	755	641	1255	3029	1910	1968	RB1
-2.92006	2.40E-07	2.40E-06	down	4	6	8	51	65	27	SSUH2
-3.37206	8.31E-46	1.95E-43	down	39	32	49	396	517	387	GALNT6
-4.40345	2.96E-05	0.00019	down	3	0	0	21	19	26	SH3GL3
-1.35377	4.86E-09	6.53E-08	down	527	409	655	1562	1342	1244	BRCA2
-1.90377	9.86E-11	1.68E-09	down	36	39	54	181	185	133	CD24
-1.21915	1.19E-12	2.67E-11	down	258	289	295	566	848	659	PROCR
-1.51145	1.28E-05	8.95E-05	down	50	33	67	97	243	120	ID2
-1.84608	1.47E-05	0.000101	down	36	27	14	67	158	80	
-1.54641	0.0149784	0.042823	down	6	6	6	19	21	15	
-1.05662	8.77E-09	1.13E-07	down	287	233	261	497	685	532	ZBED6CL
-1.44647	0.0014985	0.006117	down	45	36	25	39	173	110	
-2.56715	0.004989	0.016998	down	1	2	3	11	14	12	TMSB4XP4
-1.84105	8.00E-09	1.04E-07	down	43	63	55	235	229	136	
-3.42981	0.0015482	0.006282	down	0	1	3	12	8	23	SCIN
-3.42539	0.0138736	0.040218	down	0	1	1	7	6	9	
-9.23158	8.17E-14	2.13E-12	down	0	0	0	70	187	104	CKMT1A
-2.92929	6.52E-09	8.57E-08	down	7	8	7	38	95	49	TMEM198
-2.23831	2.21E-26	1.87E-24	down	95	138	122	500	688	577	TINCR
-1.46222	1.15E-13	2.92E-12	down	163	152	195	473	506	477	CHMP4C
-6.22426	1.27E-05	8.84E-05	down	0	0	0	8	24	13	SLC17A9
-1.27204	9.89E-13	2.24E-11	down	1115	964	967	2080	3402	2373	ZNF335
-2.4359	0.0040072	0.014113	down	3	0	6	16	20	14	MYLK3
-3.3492	3.42E-15	1.06E-13	down	9	5	19	91	164	99	AKR1B10
-1.17366	1.15E-07	1.23E-06	down	168	137	140	265	461	346	VPS33A
-1.90629	2.19E-21	1.24E-19	down	469	446	415	1243	2364	1749	ZNF76
-2.78165	0.0121394	0.036026	down	0	5	0	11	14	12	SERPINI1
-6.01144	4.52E-06	3.48E-05	down	0	1	0	20	28	25	NFASC

-2.6949	0.0095421	0.029464	down	1	1	3	7	8	18	CTAGE6	
-5.37001	0.0001094	0.000611	down	1	0	0	12	25	11		
-2.65915	0.0054323	0.01827	down	2	0	4	10	14	15	LOC100130172	
-1.0978	0.0104657	0.03185	down	19	15	24	47	38	42	MNS1	
-1.6791	0.0075643	0.024186	down	10	6	5	13	28	31	MCOLN3	
-1.10628	4.05E-08	4.60E-07	down	649	621	883	1691	1599	1464	ATP6V1A	
-2.24786	6.26E-42	1.23E-39	down	675	735	679	2828	4441	3291	SLC41A3	
-1.21222	1.87E-08	2.27E-07	down	509	480	685	1479	1358	1138	SPPL2A	
-1.54131	2.40E-07	2.40E-06	down	36	34	47	88	165	109	KIAA1407	
-1.34761	1.51E-08	1.86E-07	down	137	137	165	248	536	411	OAS1	
-1.37782	4.17E-08	4.73E-07	down	61	65	75	185	204	155	TASP1	
-1.61265	3.25E-06	2.58E-05	down	541	556	293	737	2270	1703	ISG15	
-2.32516	1.26E-18	5.37E-17	down	37	46	38	200	237	199	MMP28	
-1.78451	5.07E-14	1.37E-12	down	474	370	639	1966	1817	1439	TET3	
-1.17947	0.0083916	0.026453	down	40	16	19	42	88	54	C17orf97	
-2.00246	0.001588	0.006426	down	12	8	4	16	34	53	VMAC	
-1.43702	0.007551	0.024152	down	10	8	10	21	39	21	C3orf67	
-1.38737	9.21E-12	1.81E-10	down	703	658	1027	2105	2098	2196	RBM47	
-1.1452	6.63E-12	1.33E-10	down	408	407	560	906	1225	1045	TRMT6	
-1.73084	0.0057775	0.019244	down	7	7	5	23	30	14	SHC3	
-3.67142	0.0007453	0.003319	down	4	2	2	83	12	2	NTRK2	
-1.36989	2.82E-11	5.19E-10	down	317	339	316	609	1128	948	SH3BP1	
-1.66808	0.0001349	0.000735	down	26	66	32	107	243	85	MFNG	
-7.35207	3.80E-08	4.36E-07	down	0	0	0	46	25	20	RIMS1	
-1.28413	0.0001549	0.000829	down	25	24	33	54	84	72	NEK11	
-3.47505	1.29E-06	1.12E-05	down	3	6	1	17	56	49	PRODH	
-1.62002	1.42E-09	2.07E-08	down	47	80	81	197	238	231	IL17RE	
-1.9092	6.99E-11	1.23E-09	down	106	90	67	222	503	350	YPEL3	
-3.24312	1.57E-05	0.000107	down	1	5	2	27	33	20		
-1.0616	0.0135174	0.039335	down	36	31	102	140	99	110		
-4.24316	0.0093455	0.028941	down	0	0	1	12	3	5	IFNL3	
-1.32989	8.85E-07	7.98E-06	down	228	223	176	359	872	494	MRPL57	
-2.08754	5.70E-08	6.37E-07	down	18	18	22	57	136	73	CARD9	
-1.2065	2.90E-06	2.32E-05	down	557	626	421	852	1874	1307	RHOD	
-1.48464	2.33E-06	1.91E-05	down	305	285	136	604	789	780	CCL5	
-1.90176	0.0020723	0.008104	down	5	7	5	18	25	24	ZNF429	
-1.04214	1.07E-11	2.08E-10	down	1609	1540	1720	2705	4400	3529	MRRF	
-5.01363	9.26E-10	1.38E-08	down	2	3	0	24	83	70	LOC339988	
-1.88234	2.07E-22	1.27E-20	down	174	217	218	657	1039	688	GSN	
-1.14877	2.94E-05	0.000188	down	411	388	235	587	1095	806	ARRDC1	
-1.06867	8.64E-06	6.22E-05	down	155	144	138	214	438	333	C9orf3	
-1.15458	0.004878	0.016668	down	25	38	17	52	85	55	CYP2D6	
-2.81189	3.51E-20	1.72E-18	down	22	27	31	139	282	180		3-S
-1.95556	6.79E-25	5.22E-23	down	211	184	245	857	978	748	DEPDC5	

-2.13856	8.91E-15	2.62E-13	down	39	40	44	151	195	220	NTN1
-3.67402	0.0067057	0.021841	down	1	0	1	5	11	11	TRPC3
-2.51422	2.97E-23	2.01E-21	down	1183	1318	1086	4102	####	7828	SLPI
-3.45757	1.23E-22	7.74E-21	down	16	12	18	137	264	140	PI3
-2.79065	8.55E-11	1.48E-09	down	30	19	19	229	162	92	TNNC1
-5.10324	0.0003792	0.001827	down	1	0	0	11	22	7	SLC6A20
-1.27664	2.39E-06	1.96E-05	down	99	62	81	205	244	166	ERBB3
-5.31291	9.10E-10	1.36E-08	down	1	2	1	35	102	38	VIPR1
-5.14265	0.0015127	0.006162	down	0	0	0	8	6	6	C2orf78
-8.68962	6.82E-139	########	down	9	17	13	3326	8491	5653	FXYD3
-2.63607	1.33E-06	1.15E-05	down	7	6	7	53	37	37	KLF15
-2.22677	0.0013157	0.005458	down	6	5	2	15	31	20	TMEM253
-1.14017	2.85E-07	2.81E-06	down	126	104	174	284	353	288	KIAA1919
-5.99778	3.33E-05	0.000211	down	0	0	0	9	19	10	MAB21L3
-1.17835	1.81E-06	1.52E-05	down	80	98	84	159	281	194	SLC22A4
-1.85757	0.0090724	0.028243	down	3	3	8	15	17	20	
-1.24735	2.82E-08	3.32E-07	down	264	210	294	636	810	466	MMRN2
-1.13801	5.27E-07	4.95E-06	down	2001	2292	1592	3328	6204	4469	SNCG
-3.79063	6.61E-15	1.98E-13	down	16	4	7	70	181	153	IL20RA
-6.27804	1.82E-05	0.000122	down	0	0	0	16	25	5	ADCY5
-1.56821	1.65E-05	0.000112	down	68	35	70	208	128	180	LRRC8B
-2.12278	0.0078962	0.025093	down	5	4	1	14	15	17	
-2.53385	4.20E-17	1.56E-15	down	21	27	25	118	181	148	SULT1A2
-2.02657	7.50E-14	1.98E-12	down	59	52	53	156	321	240	BIK
-2.25628	0.0070669	0.022849	down	2	2	4	12	17	11	APOBEC3H
-1.59568	0.0102427	0.031291	down	10	3	8	17	28	22	NEURL2
-5.91249	1.91E-07	1.95E-06	down	1	1	0	24	47	57	PCK1
-2.61801	0.0048265	0.016504	down	5	3	0	18	29	7	CRB2
-1.04006	2.79E-05	0.00018	down	365	299	308	448	1073	652	RHBDD3
-1.20271	3.63E-09	4.95E-08	down	154	130	174	308	484	323	MOCS3
-1.16686	8.70E-10	1.30E-08	down	460	393	405	768	1327	925	RTKN
-4.15968	0.0083748	0.026413	down	0	0	1	5	6	9	SLC51A
-5.62968	0.0003104	0.001535	down	0	0	0	14	10	4	
-1.29906	3.91E-06	3.04E-05	down	120	135	227	466	430	313	INO80D
-1.09957	0.0020053	0.007887	down	190	213	117	196	651	394	ZMAT5
-4.83971	0.0162365	0.045674	down	0	0	0	7	10	0	C17orf99
-4.0504	1.41E-13	3.51E-12	down	7	2	4	53	108	70	
-2.96467	0.0028781	0.010654	down	2	3	0	8	15	19	
-1.39705	8.16E-05	0.00047	down	34	56	33	83	173	96	
-1.36645	0.0086817	0.027236	down	13	8	13	23	28	40	LOC100506351
-5.01018	0.0034926	0.012565	down	0	0	0	7	10	2	LINC00336
-1.87661	6.04E-21	3.28E-19	down	133	161	202	541	815	562	ZNF165
-3.67963	3.08E-18	1.28E-16	down	8	11	7	120	108	117	PAIP2B
-10.1633	1.88E-16	6.58E-15	down	0	0	0	291	168	180	POF1B

-1.39288	0.0047109	0.01617	down	13	10	13	21	39	40	MLC1
-1.35955	2.40E-09	3.34E-08	down	272	331	341	535	1150	911	ARV1
-1.1043	0.0057457	0.019154	down	36	48	97	166	102	118	ATP8A1
-1.48013	5.11E-06	3.89E-05	down	67	59	51	121	287	132	C5AR1
-1.38912	0.0012546	0.00525	down	15	25	19	32	67	66	
-5.54101	0.0002706	0.001364	down	0	0	0	9	11	7	
-1.56935	3.65E-05	0.000229	down	20	16	25	60	81	49	OGDHL
-1.22419	4.13E-05	0.000256	down	35	44	45	78	136	94	
-1.58116	9.33E-07	8.34E-06	down	28	25	46	93	108	105	ZNF860
-6.80343	5.17E-188	########	down	24	42	37	3432	4405	4205	COL17A1
-1.23939	0.0002906	0.00145	down	23	27	43	71	88	69	STARD4-AS1
-5.1492	1.05E-28	1.05E-26	down	5	2	9	147	285	172	GRM4
-1.61434	0.0057721	0.019229	down	8	6	7	20	29	19	RNU6-45P
-1.03565	7.80E-09	1.01E-07	down	258	238	286	434	712	552	GMIP
-1.51533	9.07E-06	6.50E-05	down	57	28	33	104	129	122	ZNF268
-3.98559	0.0067672	0.022011	down	0	1	1	19	12	1	
-1.46063	1.33E-09	1.94E-08	down	123	122	139	235	478	414	PIGH
-3.92965	1.84E-10	3.04E-09	down	4	3	5	28	85	83	SLC28A3
-1.40991	0.0027937	0.010376	down	45	12	77	119	155	91	C5orf56
-1.78493	0.0033308	0.012058	down	10	2	12	29	31	25	TTC30A
-1.28265	0.00017	0.0009	down	26	24	30	53	88	65	SSPO
-2.17118	7.80E-14	2.05E-12	down	79	67	136	482	504	317	
-3.17639	2.98E-06	2.39E-05	down	1	8	3	41	50	24	
-1.11798	3.61E-05	0.000227	down	100	122	103	262	307	174	HHLA3
-2.52061	2.11E-05	0.00014	down	10	4	4	32	53	26	CCDC154
-1.70484	1.12E-05	7.91E-05	down	26	50	25	117	156	79	
-1.42646	3.46E-08	3.99E-07	down	96	123	122	224	489	276	UBAP1L
-2.26788	0.0008432	0.003697	down	4	9	4	12	51	28	OTUB2
-3.35224	1.16E-16	4.14E-15	down	28	27	33	464	236	198	PCSK5
-3.48219	3.51E-08	4.04E-07	down	2	6	3	44	56	30	GAS7
-1.06454	7.23E-07	6.64E-06	down	384	302	378	837	806	659	DTX4
-4.02938	5.78E-14	1.55E-12	down	4	5	4	61	107	59	MOCS1
-2.86894	1.63E-12	3.59E-11	down	16	13	15	68	185	97	UNC5CL
-1.08013	0.0021032	0.008203	down	36	42	30	54	117	76	SLC25A10
-1.64943	0.0009726	0.004193	down	10	14	9	23	47	41	LINC01176
-1.50354	3.03E-10	4.86E-09	down	78	74	95	176	331	238	LYSMD4
-1.42057	0.0039219	0.013875	down	19	18	12	29	83	33	DND1
-1.88385	0.0003717	0.001796	down	9	8	8	25	48	26	
-1.64707	3.90E-05	0.000243	down	20	21	26	41	116	70	ARHGAP4
-2.0648	6.31E-18	2.54E-16	down	89	92	72	338	477	311	C9orf172
-2.70325	2.62E-06	2.12E-05	down	3	11	8	24	67	63	DUSP2
-2.77122	0.0003172	0.001564	down	1	5	4	33	19	17	GRHL3
-2.51024	6.64E-22	3.93E-20	down	623	732	467	2387	5274	3665	ADIRF
-1.0381	1.09E-07	1.17E-06	down	1223	1273	1151	1841	3452	2739	SLC9A3R1

-3.04876	3.87E-22	2.32E-20	down	23	17	26	156	270	155	PALMD
-1.2745	2.47E-15	7.78E-14	down	1237	1109	1436	3098	3529	2885	EIF4EBP2
-1.06003	4.57E-05	0.00028	down	350	347	295	493	1173	597	CDKN1A
-1.48975	2.35E-11	4.36E-10	down	133	160	119	347	499	384	TSPAN15
-1.9075	0.0061971	0.020405	down	6	2	7	12	29	19	ARHGEF37
-1.56003	1.99E-15	6.30E-14	down	1348	1532	1323	3191	6057	4095	S100A10
-3.98114	0.0003269	0.001606	down	0	2	1	19	23	8	COL21A1
-2.16645	1.36E-20	6.96E-19	down	201	169	170	711	1246	650	EME2
-3.38904	1.40E-22	8.74E-21	down	35	31	34	451	430	207	CALHM3
-2.1513	0.0004375	0.002071	down	7	7	3	26	31	23	
-6.52159	1.78E-06	1.50E-05	down	0	0	0	14	24	16	RIPPLY3
-1.73664	0.0027178	0.010138	down	7	5	11	23	39	19	LOC102723830
-1.09665	0.0001446	0.000782	down	174	183	119	230	525	356	SMDT1
-2.61553	0.0117259	0.035012	down	1	1	3	5	18	10	CHST6
-1.21226	0.0108779	0.032883	down	19	16	13	37	30	48	
-4.3954	1.04E-08	1.31E-07	down	0	4	4	91	48	29	TSPAN2
-2.60778	9.50E-09	1.21E-07	down	9	6	21	73	95	59	XDH
-5.84515	6.61E-05	0.000389	down	0	0	0	12	12	9	SULT1E1
-1.63166	0.0032909	0.01195	down	5	12	9	25	28	31	CATSPERG
-4.15502	0.0014274	0.005855	down	1	1	0	11	22	6	MIR662
-2.12552	8.59E-22	5.04E-20	down	99	110	171	486	736	516	MUC13
-1.5808	1.87E-06	1.57E-05	down	189	208	137	280	929	563	RHPN1
-2.87256	1.98E-16	6.85E-15	down	31	43	16	227	263	210	FOLR1
-2.85541	0.0101844	0.031138	down	0	5	0	8	19	13	ZNF461
-1.0518	1.86E-10	3.07E-09	down	277	260	308	535	711	592	SORT1
-1.29878	5.50E-06	4.15E-05	down	54	70	53	116	215	137	VPS9D1
-3.52372	3.94E-50	1.23E-47	down	86	83	140	965	1268	1444	VAV3
-1.26556	2.09E-08	2.52E-07	down	383	241	371	699	1146	695	SLC9A8
-2.01908	0.0002181	0.001125	down	5	12	11	24	40	55	GDPGP1
-2.9208	1.94E-09	2.76E-08	down	10	8	4	50	70	57	FAM212B
-4.59409	7.11E-07	6.55E-06	down	0	0	4	25	52	25	APOC3
-1.30478	7.59E-07	6.95E-06	down	55	56	65	110	181	168	MTG1
-2.3148	2.67E-23	1.83E-21	down	192	123	208	937	965	784	KIF13B
-3.44021	2.68E-57	1.04E-54	down	105	137	100	1025	1584	1342	RAB17
-5.70308	0.0007037	0.003157	down	0	0	0	18	10	1	ODAM
-1.16869	0.0001955	0.001022	down	35	45	71	85	153	119	HYPK
-1.4087	0.0001603	0.000855	down	27	21	35	88	83	56	SAMD12-AS1
-2.15621	2.97E-20	1.48E-18	down	246	199	179	732	1401	872	GPR153
-1.10086	3.96E-13	9.37E-12	down	4829	4864	4924	8781	####	####	JUP
-1.0954	1.29E-05	8.98E-05	down	317	329	368	443	1169	738	FKBP11
-2.62721	1.25E-08	1.56E-07	down	7	9	11	41	83	54	CD101
-1.48588	0.0026132	0.009808	down	36	17	13	37	111	57	TIGD3
-1.39809	0.012505	0.036858	down	6	10	17	17	35	39	
-1.87155	0.0138911	0.040252	down	7	2	6	7	33	20	GPRASP2

-5.69335	2.48E-05	0.000162	down	0	1	0	19	27	13	SLCO4A1-AS1
-1.11931	5.28E-06	4.00E-05	down	101	98	124	258	229	234	PLCG2
-4.62632	0.0102588	0.03132	down	0	0	0	3	9	3	COL4A2-AS1
-1.8377	7.81E-06	5.69E-05	down	21	13	16	68	61	56	RSAD2
-2.72284	4.23E-06	3.27E-05	down	3	3	13	42	54	33	DNM3
-3.43551	2.14E-07	2.16E-06	down	0	12	3	56	66	50	C19orf71
-2.39568	0.0002877	0.001439	down	3	10	4	15	48	35	LOC105376382
-1.79467	1.79E-05	0.000121	down	20	9	18	50	68	53	LINC01012
-6.89517	4.62E-08	5.22E-07	down	1	0	0	39	61	36	
-1.32425	4.47E-10	6.98E-09	down	249	197	185	465	657	553	XXYLT1
-1.56347	1.12E-06	9.88E-06	down	65	91	55	132	309	237	MDK
-1.21641	0.001506	0.006141	down	33	41	64	56	181	109	CCDC159
-1.70233	0.0051499	0.017462	down	10	10	6	12	40	40	RIBC1
-1.01181	3.82E-06	2.98E-05	down	183	159	217	270	515	412	
-1.48312	8.56E-13	1.95E-11	down	397	437	456	1361	1387	999	RIPK4
-3.49168	0.0004899	0.002287	down	1	1	2	14	25	9	TREX2
-1.02389	5.09E-06	3.87E-05	down	460	546	411	719	1377	1011	COMMD9
-2.37786	0.0062252	0.02048	down	6	0	4	22	23	9	LOC101060542
-1.4309	1.73E-06	1.46E-05	down	512	457	939	2161	1503	1472	PEG10
-1.56485	7.59E-11	1.32E-09	down	60	63	80	160	269	206	STAG3L5P
-1.27964	0.000188	0.000985	down	54	27	36	78	114	108	
-2.65409	0.0087053	0.027291	down	1	1	4	4	24	13	SMG7-AS1
-2.01005	0.0031374	0.011473	down	6	5	5	11	41	19	NRG2
-1.13393	2.09E-05	0.000139	down	811	572	1153	2215	1864	1560	PRR14L
-4.20748	0.0114676	0.034343	down	0	1	0	5	2	13	SLCO1B1
-1.73985	0.0018577	0.007386	down	17	14	17	92	42	26	SOX5
-1.46681	0.0040463	0.014228	down	19	18	10	54	60	24	CD22
-1.31917	0.0068162	0.022159	down	13	40	17	56	91	42	
-2.26709	4.15E-13	9.73E-12	down	53	30	38	147	221	246	CD82
-1.94183	0.0133265	0.038887	down	2	3	6	17	14	12	HCG25
-1.21154	2.48E-08	2.95E-07	down	180	201	229	345	700	467	SFXN5
-1.26818	2.43E-05	0.000159	down	109	55	103	241	239	184	TRANK1
-3.96697	3.89E-05	0.000243	down	1	1	2	29	23	12	ANKRD53
-1.29675	4.98E-07	4.70E-06	down	77	53	93	162	209	199	ZNF514
-1.61684	1.68E-05	0.000114	down	25	21	25	50	118	67	KCNIP2
-2.08548	5.91E-08	6.58E-07	down	23	17	30	124	111	70	SCARA5
-3.33307	3.15E-06	2.51E-05	down	7	3	0	25	45	39	SLC2A9
-1.20477	1.47E-05	0.000101	down	193	173	124	278	592	361	SLC22A18
-1.2538	6.92E-12	1.38E-10	down	2180	2027	1766	4276	6301	4599	MISP
-1.35766	7.46E-06	5.48E-05	down	382	343	196	576	1238	775	MFSD10
-1.99655	3.56E-06	2.80E-05	down	16	12	13	37	70	67	
-2.42426	0.0079162	0.025133	down	3	5	0	18	19	9	MIR4746
-1.0224	1.44E-06	1.24E-05	down	895	849	718	1242	2332	1807	TRMT2A
-1.30098	2.31E-10	3.74E-09	down	194	156	166	423	504	407	ARVCF

-3.84122	0.0039661	0.013985	down	1	0	1	11	7	11	CCR3
-1.19834	1.52E-05	0.000104	down	53	75	86	139	243	140	BSCL2
-3.22944	6.95E-48	1.89E-45	down	95	76	92	613	1028	970	KREMEN1
-4.50031	0.0045813	0.015771	down	1	0	0	10	14	2	SLC35F3
-3.29721	1.86E-43	3.98E-41	down	75	126	102	773	1270	1120	F11R
-1.38243	5.25E-08	5.90E-07	down	179	132	236	544	498	416	MOB3B
-2.94287	0.0017077	0.006862	down	2	1	3	20	22	6	
-7.68729	5.83E-10	9.02E-09	down	1	0	0	64	70	96	DSG3
-4.81237	8.12E-14	2.12E-12	down	0	4	4	86	71	73	DSC2
-5.87968	1.50E-21	8.64E-20	down	1	1	5	119	168	144	DSC3
-2.93992	0.0074909	0.02402	down	1	3	0	7	12	14	HGFAC
-1.29698	5.76E-07	5.38E-06	down	74	73	135	200	289	232	PPARGC1A
-2.37739	1.63E-28	1.61E-26	down	856	674	621	3657	5046	3193	SUSD2
-1.8568	1.13E-08	1.42E-07	down	28	25	25	74	130	97	POLR2J3
-5.94682	4.35E-05	0.000268	down	0	0	0	14	11	10	SCN1A
-5.00578	0.0039195	0.013869	down	0	0	0	10	6	2	FOXI1
-5.84331	0.0001601	0.000854	down	0	0	0	3	13	18	GALNT13
-3.0189	0.0022392	0.008648	down	2	1	2	5	22	17	MPZ
-1.52288	0.0007806	0.003457	down	30	13	29	95	61	53	ERCC5
-4.74246	0.0001279	0.000699	down	0	2	0	12	33	14	SLC5A11
-1.24931	8.42E-05	0.000484	down	145	112	73	185	392	278	DMTN
-2.51682	0.0001686	0.000894	down	4	5	3	23	32	18	LYG1
-1.30203	9.00E-05	0.000512	down	26	25	34	58	86	76	EDA
-2.11537	5.56E-05	0.000334	down	12	6	7	31	54	31	FGF17
-1.10791	7.92E-13	1.80E-11	down	766	790	782	1472	2173	1692	ZNF276
-5.56702	3.81E-05	0.000239	down	0	0	1	15	23	16	NPM2
-2.30261	4.31E-07	4.12E-06	down	13	11	9	38	85	53	
-1.87658	0.0135432	0.039404	down	3	6	6	29	19	8	IL23A
-2.76101	1.43E-05	9.83E-05	down	4	5	6	48	36	20	PRDM6
-1.26562	0.0115569	0.034583	down	12	17	12	19	52	36	
-3.00657	8.43E-06	6.08E-05	down	6	1	4	22	35	36	
-1.06235	8.44E-06	6.09E-05	down	120	108	137	287	278	223	BTBD9
-1.05084	0.002165	0.008404	down	40	26	33	65	93	59	LOC101927550
-1.03143	0.017532	0.048762	down	16	15	23	31	55	31	CNTF
-1.08853	0.003856	0.013678	down	26	18	30	40	69	57	
-1.29553	1.12E-11	2.18E-10	down	342	352	526	888	1139	1080	LMBRD1
-1.0915	0.0002116	0.001093	down	64	78	49	111	176	148	GLYCTK
-1.5128	9.03E-07	8.11E-06	down	37	32	51	86	169	109	GNG4
-6.54049	8.61E-09	1.11E-07	down	2	0	0	33	124	50	GALR2
-2.956	0.0052081	0.017634	down	1	1	3	21	14	4	
-2.59694	0.008909	0.027811	down	3	1	2	19	6	11	SLC44A3
-2.48976	0.0044704	0.015462	down	4	0	4	10	18	19	ASIC2
-3.57067	2.24E-58	8.88E-56	down	57	51	85	659	879	848	SPINK5
-2.15054	0.0030733	0.011263	down	4	4	4	12	32	14	

-2.38652	0.0003231	0.00159	down	2	6	5	19	31	22	
-3.44622	0.0126816	0.037285	down	1	1	0	9	9	5	DTX2P1-UPK3BP1-PMS2F
-1.78562	0.0015967	0.006454	down	10	6	9	16	39	37	
-3.33223	1.42E-05	9.78E-05	down	5	1	2	25	21	37	UGT1A6
-1.45658	0.0002765	0.001391	down	25	17	19	38	77	64	XCL1
-5.19121	1.47E-26	1.27E-24	down	9	16	17	822	519	206	C1orf116
-3.61229	1.90E-06	1.59E-05	down	2	2	3	34	23	30	UGT1A1
-1.81723	8.92E-35	1.19E-32	down	987	967	1145	3066	4452	3967	CREG1
-7.03985	1.52E-07	1.58E-06	down	0	0	0	30	32	14	GPA33
-1.70974	4.47E-10	6.99E-09	down	108	119	81	287	522	282	KPTN
-1.23011	9.23E-05	0.000524	down	31	29	37	69	89	80	HSD17B1
-1.25238	9.99E-07	8.88E-06	down	257	250	174	436	818	505	NAGLU
-1.46066	1.60E-07	1.65E-06	down	184	181	337	697	554	695	LRRCC1
-1.34112	6.48E-07	6.00E-06	down	427	454	388	615	1667	1223	COL1A1
-1.3269	8.27E-08	9.00E-07	down	987	1058	745	1656	3599	2373	COQ4
-5.21373	0.001139	0.004813	down	0	0	0	8	6	7	LINC00483
-1.0058	0.0006725	0.00303	down	550	542	349	604	1583	1001	ARPC4
-2.80261	1.67E-06	1.42E-05	down	3	6	6	26	41	43	HCAR2
-4.12093	0.0148386	0.04247	down	0	0	1	1	13	7	PRRX2
-1.42529	1.17E-06	1.02E-05	down	65	52	81	211	196	140	GRID1
-2.15323	0.0003525	0.001713	down	5	3	14	37	34	28	LINC00893
-1.68257	3.45E-08	3.99E-07	down	35	29	34	110	120	98	
-4.71386	0.0084159	0.026525	down	0	0	0	7	6	2	
-3.97271	0.0034582	0.01246	down	2	0	0	6	21	8	CA14
-4.64324	0.0089828	0.028016	down	0	0	0	3	8	4	DCN
-2.16165	1.92E-07	1.96E-06	down	18	23	11	88	97	61	TNFRSF14
-1.21402	4.44E-12	9.15E-11	down	1242	1424	1318	2597	4355	2924	UFC1
-5.27851	1.61E-08	1.98E-07	down	0	0	4	19	71	74	PVRL4
-1.31704	4.57E-06	3.51E-05	down	72	58	53	117	179	187	GAREML
-1.18218	5.63E-06	4.24E-05	down	62	49	59	117	154	134	MREG
-2.14169	8.30E-07	7.53E-06	down	8	12	16	54	62	49	SLCO1A2
-1.73536	5.43E-05	0.000327	down	16	16	13	48	74	38	NUDT18
-1.25391	4.23E-05	0.000261	down	31	40	40	87	111	80	MAP2K6
-4.42669	1.22E-13	3.10E-12	down	6	1	3	82	79	62	
-2.83968	0.0016066	0.006493	down	0	5	3	7	28	27	
-1.27853	0.0055404	0.018582	down	19	11	13	34	44	32	MIR4664
-1.76669	0.0003748	0.001808	down	10	11	11	22	54	41	KATNAL2
-2.84286	0.0086415	0.027137	down	1	2	1	5	14	12	
-4.1279	0.0021515	0.008362	down	0	2	0	16	16	5	LOC102724548
-1.79541	4.65E-08	5.25E-07	down	30	24	25	79	129	84	CGN
-2.35035	1.22E-28	1.21E-26	down	68	74	90	377	501	363	ECM1
-1.21628	7.97E-08	8.70E-07	down	84	103	92	204	262	216	LYPLAL1
-3.22086	0.0112241	0.03374	down	0	0	4	23	7	5	HMCN1
-1.56856	0.0014965	0.00611	down	11	9	15	41	32	33	TTC9

-1.92765	8.45E-12	1.67E-10	down	59	49	107	277	311	252	PIGM
-1.31592	2.29E-06	1.88E-05	down	116	73	77	182	322	207	TSPEAR-AS2
-1.24295	9.46E-07	8.45E-06	down	124	161	265	405	493	443	C11orf54
-2.25126	0.0001659	0.000882	down	4	6	11	45	32	24	FILIP1
-2.90662	6.26E-07	5.82E-06	down	1	8	8	40	58	36	ODF3L1
-1.22561	9.78E-07	8.71E-06	down	109	77	87	168	287	225	ZNF320
-3.89961	0.0177779	0.049301	down	0	0	1	6	8	3	
-1.16026	0.0134619	0.03921	down	11	16	15	34	37	27	
-1.5746	7.88E-06	5.74E-05	down	75	134	88	153	480	338	FN3K
-1.73559	0.0124578	0.036775	down	2	11	6	27	19	19	LOC101929610
-2.92604	0.0072556	0.023372	down	3	0	1	9	12	11	
-2.31349	0.0097959	0.030164	down	2	3	2	7	14	16	
-1.16287	2.43E-12	5.21E-11	down	728	731	940	1439	2422	1822	SMYD2
-1.68417	9.14E-11	1.57E-09	down	105	98	81	231	466	290	
-3.72773	0.0066741	0.021761	down	2	0	0	6	15	8	
-3.05702	0.0003074	0.001525	down	2	2	2	15	17	20	OAZ3
-2.31619	3.22E-15	1.01E-13	down	50	50	41	215	362	178	SEMA6C
-1.68196	1.20E-05	8.41E-05	down	72	46	141	364	258	205	MAST4
-1.42709	1.86E-13	4.58E-12	down	597	472	772	1655	1841	1615	PTPRU
-1.04504	0.0010603	0.004526	down	623	615	987	2180	1411	1007	ANKRD13C
-2.61162	2.73E-14	7.62E-13	down	15	18	21	83	149	118	ANXA9
-1.8245	0.0065855	0.02152	down	5	4	7	12	32	17	HHIPL2
-1.48379	6.77E-05	0.000397	down	83	85	109	388	249	145	DUSP10
-2.55932	0.0020577	0.00806	down	5	2	1	13	24	14	ALG1L2
-5.42817	7.99E-05	0.000462	down	1	0	0	16	22	11	
-1.85579	0.0063386	0.020804	down	5	5	4	15	24	15	
-3.26868	0.0072185	0.02326	down	2	1	0	8	6	16	LINC01337
-2.16109	1.36E-06	1.17E-05	down	20	15	8	57	98	53	TMEM191A
-1.82638	1.17E-05	8.24E-05	down	14	12	16	39	69	50	
-1.43936	4.16E-07	4.00E-06	down	54	47	74	174	191	127	EFR3B
-1.042	6.79E-05	0.000399	down	196	213	217	258	669	469	SLC27A3
-1.22761	3.23E-14	8.97E-13	down	616	638	818	1396	1905	1770	MED28
-2.72997	0.0114287	0.034238	down	1	2	1	7	10	11	ACTA1
-1.12394	1.90E-10	3.14E-09	down	259	274	328	510	824	649	C1orf131
-1.393	0.0024202	0.009192	down	12	19	21	25	65	56	
-1.61177	7.10E-17	2.57E-15	down	171	139	203	480	676	490	ZNF641
-1.5287	5.50E-07	5.15E-06	down	42	79	70	129	260	200	PROCA1
-1.45767	7.21E-13	1.65E-11	down	537	634	518	1209	2205	1571	VAMP8
-1.18595	1.25E-07	1.33E-06	down	277	264	233	423	796	669	DHRS13
-1.02699	0.0038791	0.013749	down	40	47	51	50	141	112	CORO6
-5.96847	1.03E-23	7.25E-22	down	4	2	2	197	185	137	DAPP1
-5.95926	4.87E-05	0.000296	down	0	0	0	12	8	15	ZNF610
-1.09158	0.0001537	0.000824	down	56	45	91	124	174	128	ZNF701
-1.61318	1.36E-14	3.91E-13	down	100	102	110	320	388	292	LINC00958

-4.31205	3.55E-08	4.10E-07	down	5	0	1	40	58	29	FAM109B
-1.25728	3.20E-08	3.71E-07	down	129	123	150	332	423	254	
-3.16574	0.0022138	0.008567	down	2	2	0	13	14	11	
-1.38908	3.17E-06	2.52E-05	down	83	40	78	142	235	179	MR1
-2.3892	0.0060504	0.020002	down	11	3	4	68	10	13	KDR
-1.69644	3.74E-08	4.29E-07	down	48	48	32	118	200	128	RILP
-4.81066	8.68E-70	4.23E-67	down	101	78	80	1481	3871	2567	SLC43A2
-2.29617	1.94E-06	1.62E-05	down	12	18	7	37	84	76	GPT
-1.29108	3.00E-08	3.50E-07	down	316	294	229	684	923	580	WDR81
-2.38089	1.75E-06	1.48E-05	down	13	10	5	36	73	49	SERPINF2
-4.35735	3.80E-15	1.17E-13	down	6	5	2	52	132	105	TMC4
-1.28524	1.63E-09	2.35E-08	down	493	503	623	1528	1418	1115	ABCG2
-1.82947	1.19E-12	2.68E-11	down	254	292	199	661	1398	827	PSCA
-9.36125	1.09E-14	3.19E-13	down	0	1	0	198	367	194	LY6D
-2.28587	9.89E-05	0.000557	down	3	15	9	24	75	45	LOC101929125
-3.02068	0.0172727	0.048126	down	1	1	1	10	14	2	
-2.26866	0.0119718	0.035656	down	1	3	3	8	17	11	
-3.03903	0.0034077	0.012306	down	1	1	6	46	8	7	HNF1B
-5.26719	8.34E-06	6.04E-05	down	0	1	1	26	41	15	PLEKHB1
-1.5701	1.98E-05	0.000132	down	25	29	38	52	138	103	
-1.25214	0.0113668	0.034079	down	14	20	11	22	58	37	
-1.91035	0.0146544	0.042077	down	3	6	5	14	37	7	
-1.25133	0.0148181	0.042438	down	9	10	13	20	31	29	
-1.89769	1.28E-15	4.08E-14	down	56	56	70	221	287	203	ITPKB
-1.04549	0.00145	0.005941	down	50	28	48	67	109	98	SRR
-3.87355	0.0001372	0.000746	down	0	0	4	23	21	15	TRPV3
-1.63419	1.12E-10	1.88E-09	down	1259	1202	1460	5275	4048	3093	PPL
-2.17941	0.0135674	0.039463	down	4	4	2	4	18	27	KLK1
-8.04144	3.22E-10	5.11E-09	down	0	0	0	25	72	60	KLK11
-7.0121	1.77E-14	5.06E-13	down	0	3	0	71	178	168	KLK6
-1.88343	7.30E-09	9.51E-08	down	30	25	22	84	121	96	WNT9A
-1.26815	2.40E-13	5.80E-12	down	221	229	282	502	746	607	MFSD1
-1.99318	7.94E-33	9.92E-31	down	495	504	496	1935	2532	1810	PSEN2
-1.0899	0.000115	0.000637	down	56	48	83	100	177	142	SGK3
-2.17431	0.0012426	0.005208	down	4	9	3	24	19	32	
-6.18624	1.97E-24	1.44E-22	down	2	2	3	137	229	174	
-2.37509	9.46E-06	6.76E-05	down	5	9	7	27	43	45	
-1.74204	0.0002601	0.001315	down	15	11	8	35	50	36	RBFADN
-1.03793	0.0003314	0.001625	down	44	52	44	92	112	98	
-1.65069	0.0077507	0.024703	down	6	7	7	12	28	27	
-3.94041	0.0175154	0.048723	down	1	0	0	2	9	7	
-5.9873	5.37E-05	0.000324	down	0	0	0	17	13	6	ADGRF4
-2.56294	0.0132094	0.038592	down	0	3	2	9	12	10	PLA2R1
-1.6411	1.11E-11	2.16E-10	down	317	243	394	1119	1219	743	PTPRR

-2.37725	1.80E-07	1.84E-06	down	19	21	9	56	153	73	PRR22
-4.31073	0.0058995	0.019591	down	0	0	1	10	8	4	SOST
-5.31486	4.08E-35	5.57E-33	down	10	9	5	185	521	339	ATP6V1C2
-1.40825	2.69E-05	0.000174	down	37	23	40	71	130	81	MEIS1
-2.06707	1.68E-06	1.43E-05	down	21	14	21	105	86	50	GDAP1
-3.48414	8.81E-37	1.33E-34	down	23	41	50	409	561	367	NIPAL2
-2.37301	3.85E-07	3.72E-06	down	7	7	15	45	66	46	
-7.78935	8.34E-108	########	down	21	40	86	12656	####	9346	CALB1
-2.49637	0.00332	0.012027	down	1	4	6	5	33	29	EYA1
-2.32187	2.77E-08	3.25E-07	down	14	10	12	54	84	53	
-2.76982	0.006904	0.022395	down	1	3	1	6	23	9	
-7.06339	8.60E-27	7.62E-25	down	3	0	3	169	370	317	MAOB
-5.30296	1.23E-53	4.34E-51	down	22	12	13	754	613	543	ST6GALNAC1
-1.84904	0.000732	0.003271	down	6	7	10	21	34	32	MYO16-AS1
-1.10664	8.61E-07	7.79E-06	down	121	148	212	329	421	327	
-4.82342	0.006252	0.020554	down	0	0	0	5	10	2	
-4.2514	0.0063973	0.02098	down	1	0	0	4	10	8	
-1.19349	1.10E-08	1.39E-07	down	958	910	838	1981	2986	1646	KRT17
-2.27118	3.00E-08	3.50E-07	down	14	20	12	52	112	76	
-2.75364	0.0105054	0.031945	down	2	1	1	10	9	9	LRRN4CL
-1.98215	0.0024184	0.009189	down	3	11	5	16	42	24	LOC102724362
-4.14149	3.04E-63	1.37E-60	down	32	39	56	732	771	806	ESRP1
-1.57468	7.41E-05	0.000432	down	14	27	19	54	82	54	HIC1
-1.19001	0.0154152	0.043844	down	13	21	38	27	97	53	
-1.21081	0.0002103	0.001088	down	54	34	78	132	167	100	
-2.967	0.0086806	0.027236	down	3	0	1	13	15	5	LOC101927239
-1.43139	6.17E-05	0.000366	down	565	285	719	1982	1330	921	EPPK1
-3.17	0.0021621	0.008395	down	3	0	2	17	26	5	
-3.14069	3.04E-07	2.99E-06	down	4	2	6	32	47	32	LINC00479
-4.72387	0.0013776	0.00568	down	0	0	1	12	8	9	
-4.16692	0.0124972	0.036841	down	0	0	1	2	16	4	LOC105274304
-1.22304	0.0063037	0.020709	down	23	15	13	30	51	46	
-4.54578	0.0124434	0.036744	down	0	0	0	6	3	4	
-2.3377	0.0108982	0.032939	down	0	5	3	8	17	18	
-2.13884	3.82E-06	2.98E-05	down	15	8	12	53	73	37	
-1.93907	1.14E-10	1.93E-09	down	340	397	293	689	2015	1616	IRX3
-4.38124	0.0005498	0.002535	down	1	0	1	11	25	9	RPRM
-1.3794	2.75E-08	3.23E-07	down	209	194	210	640	615	400	ADPRHL1
-2.50925	2.55E-06	2.07E-05	down	7	6	20	85	56	46	FOXP2
-3.04086	0.0053489	0.018041	down	0	2	2	11	18	6	
-1.51922	9.09E-07	8.15E-06	down	41	48	53	134	202	96	
-3.09612	3.38E-13	8.06E-12	down	21	5	12	88	145	112	ST6GALNAC2
-3.56853	0.0116127	0.034728	down	0	2	0	4	12	10	KCNH2
-2.07134	0.0129708	0.037983	down	4	4	1	13	14	13	LINC00896

-5.75435	0.0001013	0.000569	down	0	0	0	9	10	12	LOC100288748
-1.30478	3.61E-05	0.000227	down	40	40	36	68	135	104	
-2.66341	7.64E-09	9.92E-08	down	20	4	13	68	106	74	LINC01134
-1.75975	0.0007678	0.003406	down	5	13	17	48	41	32	FAM157A
-1.83317	1.00E-21	5.83E-20	down	284	231	302	970	1256	830	GBA
-1.00036	0.0004221	0.002011	down	196	141	235	476	342	340	SAMD12
-1.18743	4.94E-09	6.62E-08	down	373	416	403	654	1280	975	UBXN8
-4.17505	1.71E-99	2.32E-96	down	726	493	880	11202	####	####	CPS1
-3.19599	3.98E-13	9.39E-12	down	13	9	10	55	148	114	PECAM1
-1.18659	0.0017125	0.006875	down	25	22	35	40	80	77	IRF5
-3.62098	0.0128419	0.037652	down	0	2	0	5	19	4	TMEM249
-4.41607	0.0045928	0.015802	down	1	0	0	10	4	9	
-1.88651	4.62E-12	9.47E-11	down	39	42	55	175	205	144	
-1.56607	4.39E-21	2.43E-19	down	608	576	591	1482	2399	1715	MKNK1
-1.8302	6.04E-06	4.51E-05	down	26	17	17	57	117	57	C7orf31
-7.53109	1.05E-85	7.97E-83	down	13	5	9	1887	2194	1152	BTBD16
-1.46243	2.06E-11	3.85E-10	down	109	120	171	297	492	372	C2orf48
-1.11835	1.58E-07	1.64E-06	down	164	197	275	395	537	505	CCDC138
-1.62317	4.95E-16	1.64E-14	down	141	113	142	348	543	400	FBXO41
-2.8936	0.000985	0.004242	down	2	1	4	18	29	8	CYP2C8
-3.05794	4.65E-15	1.42E-13	down	41	35	49	135	567	433	ESPN
-2.59167	1.69E-06	1.43E-05	down	5	6	7	31	51	33	DNAJC28
-1.17883	1.18E-11	2.28E-10	down	931	1039	1011	1756	2999	2431	ADCK3
-1.77985	0.0006013	0.002747	down	7	14	8	32	46	28	NOSTRIN
-2.96468	5.61E-09	7.47E-08	down	5	7	7	38	58	60	SGPP2
-2.39475	0.005475	0.018397	down	0	8	14	70	15	24	PTPRD
-1.58486	7.34E-05	0.000428	down	16	20	16	47	73	46	PLCD1
-7.89527	4.75E-97	5.01E-94	down	9	8	31	4242	4913	2565	SLC12A3
-2.67011	3.16E-13	7.55E-12	down	26	32	28	117	338	147	
-1.1673	4.64E-09	6.25E-08	down	225	282	252	472	685	643	PIGB
-4.98919	0.002884	0.010674	down	0	0	0	8	6	4	FGL1
-1.01404	4.30E-05	0.000265	down	203	209	188	263	580	463	HOXD9
-1.78793	3.23E-21	1.80E-19	down	958	940	924	3389	4193	2686	GYS1
-4.27938	4.52E-25	3.51E-23	down	6	8	9	137	216	121	
-1.46439	0.0157268	0.04455	down	7	10	5	22	24	18	
-1.86382	5.38E-20	2.59E-18	down	120	105	113	377	545	380	SVBP
-2.49521	3.59E-17	1.34E-15	down	41	56	57	315	400	199	CALHM2
-2.12412	1.21E-07	1.29E-06	down	39	43	50	224	303	83	BTBD19
-3.05528	6.54E-07	6.05E-06	down	4	4	6	29	70	27	DUSP13
-1.32423	7.69E-06	5.62E-05	down	40	49	74	137	138	144	FAM185A
-3.11599	5.87E-18	2.37E-16	down	13	24	16	173	185	123	GPR87
-1.02488	3.33E-08	3.86E-07	down	212	184	229	398	481	448	LYSMD1
-2.17096	0.000337	0.001649	down	7	7	5	39	27	22	SLIT1
-3.73587	0.0007259	0.003248	down	3	1	0	34	9	10	ANGPTL5

-1.69769	1.67E-05	0.000113	down	125	142	60	206	672	316	AMH
-2.09529	4.07E-13	9.55E-12	down	99	71	168	533	512	424	MYO5C
-2.86819	0.0057049	0.019045	down	0	1	4	7	16	15	
-6.25644	7.78E-06	5.67E-05	down	0	0	0	11	20	14	RRS1-AS1
-2.31841	0.0007481	0.00333	down	2	10	3	19	37	25	
-3.23163	7.87E-05	0.000456	down	2	2	3	12	42	18	
-3.25031	0.0160233	0.045221	down	2	1	0	10	21	1	
-1.52535	2.59E-08	3.07E-07	down	94	90	94	171	425	271	MAMDC4
-2.41872	4.39E-08	4.97E-07	down	11	22	13	48	107	109	SSTR5-AS1
-4.44765	0.0163538	0.045942	down	0	0	0	2	6	5	
-4.98228	2.84E-19	1.29E-17	down	6	2	2	105	134	94	ALPPL2
-4.69945	1.54E-60	6.38E-58	down	106	76	54	2210	2709	1604	ALPP
-3.39189	1.23E-33	1.58E-31	down	36	21	42	268	490	344	EWSAT1
-2.91753	4.33E-38	7.34E-36	down	50	62	56	362	572	414	HYAL1
-1.20347	0.0002945	0.001468	down	135	111	174	457	292	224	NIPAL1
-6.3782	2.87E-41	5.40E-39	down	16	4	8	362	1503	729	ALPI
-1.02042	0.0007451	0.003319	down	50	55	53	83	169	93	CYB561D2
-4.53913	0.0142583	0.041113	down	0	0	0	7	4	2	CIDEC
-1.76661	1.80E-22	1.11E-20	down	229	219	216	651	1011	739	CLN6
-1.06233	0.000844	0.003699	down	32	36	47	81	82	85	UBR5-AS1
-1.11928	1.31E-06	1.13E-05	down	115	79	119	206	269	236	SESN1
-1.48571	0.0004784	0.002241	down	20	28	49	45	122	120	CROT
-1.49128	0.0139924	0.040482	down	4	9	10	15	33	21	SLC15A2
-1.27002	0.0018781	0.007452	down	28	21	23	47	99	42	J3QL48
-2.5917	1.36E-08	1.68E-07	down	17	11	11	61	142	54	
-1.35282	1.27E-07	1.34E-06	down	174	150	133	266	575	421	TMEM79
-8.2809	5.22E-40	9.63E-38	down	2	3	1	501	872	616	C1orf106
-1.38648	0.0002646	0.001336	down	49	52	24	76	165	116	RNF208
-1.82768	0.0004078	0.001949	down	8	8	14	22	44	46	
-7.93332	4.25E-19	1.90E-17	down	1	2	0	153	397	247	VIL1
-1.00165	0.0167102	0.046792	down	20	22	48	54	54	75	
-2.80491	0.002074	0.008109	down	2	3	1	8	24	14	
-2.92231	7.48E-06	5.49E-05	down	5	3	5	23	28	51	CTSH
-1.96907	0.0001607	0.000857	down	9	5	14	41	37	34	
-2.00149	0.0009707	0.004188	down	2	7	14	25	36	34	
-1.3918	0.0113511	0.034042	down	15	18	9	16	63	43	
-1.18841	0.0005115	0.002379	down	68	77	54	87	253	158	
-1.04204	4.83E-11	8.62E-10	down	374	361	399	728	948	776	VSIG10
-2.05436	0.0003306	0.001622	down	13	10	4	21	58	44	
-5.6461	0.0002433	0.001239	down	0	0	0	14	8	6	
-1.59009	1.51E-08	1.86E-07	down	92	78	125	362	297	246	IQCH-AS1
-2.08145	9.25E-05	0.000525	down	8	14	6	25	61	43	TBXA2R
-3.79805	9.11E-36	1.30E-33	down	25	27	47	296	685	487	CYP4F12
-3.76941	1.05E-10	1.79E-09	down	2	5	7	42	115	50	

-5.40599	0.0001122	0.000624	down	0	1	0	21	17	9	GRIN1
-2.77671	3.04E-12	6.42E-11	down	19	23	22	84	274	125	SLC25A45
-1.04316	0.0061258	0.020195	down	42	17	39	60	81	70	TCEANC
-3.33167	0.0049603	0.016919	down	2	1	0	5	15	13	USH1C
-4.61605	0.0111128	0.033465	down	0	0	0	2	9	4	LINC00494
-1.19544	0.000151	0.000812	down	61	71	60	89	234	155	C2orf76
-2.8287	6.73E-07	6.21E-06	down	5	7	4	38	54	29	THBS4
-1.66292	1.13E-13	2.88E-12	down	121	102	139	406	394	382	KIAA0319
-2.72066	0.0148339	0.04247	down	2	2	4	42	4	3	TNFRSF19
-1.75001	0.000169	0.000896	down	11	16	11	30	66	42	
-1.13138	0.0020548	0.00805	down	39	25	24	52	88	66	
-1.6256	0.0006545	0.002959	down	32	19	22	29	128	91	
-2.18756	4.36E-12	9.00E-11	down	21	37	53	143	224	163	RASGRF2
-4.86139	3.92E-25	3.09E-23	down	8	6	7	102	325	237	
-1.65933	0.0049081	0.016765	down	11	17	5	18	61	37	
-7.26687	6.42E-09	8.45E-08	down	0	1	0	39	66	70	FUT2
-3.37944	2.95E-10	4.74E-09	down	3	13	4	72	96	54	C8G
-2.42558	0.0009957	0.004285	down	5	3	2	17	23	17	
-3.8939	2.07E-11	3.86E-10	down	12	2	3	61	152	65	
-1.55455	0.0146178	0.041984	down	4	11	9	11	35	30	
-7.15026	1.97E-24	1.44E-22	down	0	1	4	181	348	227	TTC22
-1.58775	1.14E-05	8.02E-05	down	40	49	29	72	177	137	
-1.42478	0.000704	0.003158	down	16	19	32	47	100	45	LINC01024
-3.11582	0.0002106	0.001089	down	2	2	2	17	24	14	
-1.12332	3.14E-05	0.0002	down	1586	1710	1077	2125	4778	3501	DPP7
-1.70668	0.0134112	0.039086	down	6	9	2	20	22	17	FGF19
-2.56401	4.18E-06	3.24E-05	down	9	10	4	39	80	30	A2M-AS1
-4.54657	0.0133176	0.038869	down	0	0	0	4	8	2	
-2.70637	0.0006209	0.002826	down	0	3	7	24	22	20	LOC101928737
-2.43723	0.0026375	0.009881	down	1	4	4	9	23	20	
-1.81216	0.0004815	0.002254	down	13	5	11	36	40	30	KLHL32
-1.83663	8.55E-21	4.53E-19	down	211	159	208	693	778	678	KIAA0040
-5.76581	1.75E-05	0.000118	down	0	1	0	13	24	25	
-2.16871	1.35E-08	1.67E-07	down	17	11	31	83	104	87	RNF144B
-3.85997	0.0004932	0.002301	down	1	2	0	8	25	15	
-1.31648	1.82E-09	2.60E-08	down	158	147	151	356	386	440	SGCE
-1.47008	0.0022657	0.008729	down	17	18	61	108	101	58	ZNF117
-1.85553	0.0148367	0.04247	down	2	6	5	8	26	17	GAS5-AS1
-2.10763	6.76E-06	5.01E-05	down	9	12	9	37	62	39	
-2.74934	1.15E-09	1.69E-08	down	16	23	5	90	114	112	ZSWIM5
-3.19104	1.65E-10	2.74E-09	down	5	6	8	60	77	45	PRCAT47
-2.31804	2.72E-11	5.02E-10	down	38	38	32	98	278	210	
-3.43761	0.0001086	0.000607	down	1	3	1	16	20	21	LOC729652
-3.50021	3.42E-08	3.95E-07	down	3	2	5	38	41	38	CCDC30

-2.12076	0.0022543	0.008694	down	1	12	6	32	36	19	SLC25A34
-1.94514	1.58E-24	1.18E-22	down	214	159	209	699	960	700	FBXL19-AS1
-1.07137	0.006872	0.022318	down	25	28	19	37	74	52	AKR7A3
-4.96699	0.0006653	0.003	down	0	1	0	7	22	8	SPATA12
-2.26893	0.0002388	0.001219	down	9	7	2	23	42	29	
-1.17603	5.91E-08	6.58E-07	down	114	97	106	209	287	258	PARS2
-3.6599	1.29E-27	1.20E-25	down	22	18	29	171	432	332	ACOT11
-1.38077	0.0006337	0.002877	down	18	15	26	41	77	45	NUDT17
-2.69602	4.27E-05	0.000263	down	1	5	8	20	38	37	
-1.34302	2.48E-07	2.48E-06	down	74	79	76	169	293	160	PKI55
-1.8263	3.50E-35	4.85E-33	down	1286	1249	1271	4074	5768	4430	RXRA
-2.95477	2.13E-29	2.25E-27	down	34	28	35	213	294	282	
-1.22251	6.11E-09	8.08E-08	down	143	129	175	364	396	322	ZNF75D
-2.20758	2.59E-41	4.93E-39	down	421	452	446	1687	2689	2095	
-1.62217	4.82E-07	4.57E-06	down	44	45	40	133	199	92	FHDC1
-1.40526	1.60E-06	1.36E-05	down	883	621	1466	3142	2442	2308	SYNE2
-2.73629	4.05E-16	1.35E-14	down	62	79	146	686	926	378	FGFBP1
-1.00541	6.10E-05	0.000363	down	233	215	431	589	644	572	PDE4D
-4.62804	4.54E-52	1.51E-49	down	91	89	137	3579	2324	1967	CDH1
-1.05579	3.31E-06	2.62E-05	down	642	720	563	939	1861	1512	NRM
-4.54261	2.99E-11	5.46E-10	down	2	1	4	45	56	68	SULT2B1
-4.65202	0.0090793	0.028246	down	0	0	0	6	3	5	DLX6
-1.45217	0.0008501	0.003721	down	36	22	22	36	115	88	RAB11B-AS1
-3.79868	1.51E-06	1.30E-05	down	2	1	4	31	57	16	
-2.77141	1.11E-12	2.50E-11	down	12	9	16	67	109	90	SYNC
-1.28657	0.0039578	0.013966	down	16	17	27	64	43	41	HMGN3-AS1
-6.42749	5.00E-06	3.81E-05	down	0	0	0	8	28	16	CLCNKA
-1.69998	5.95E-08	6.62E-07	down	117	98	62	208	456	318	
-4.71901	0.0012675	0.005291	down	0	1	0	7	12	11	SMC2-AS1
-1.37475	1.26E-12	2.81E-11	down	517	478	423	1129	1662	1127	MEGF6
-1.0403	1.75E-09	2.51E-08	down	370	437	479	867	1010	879	NFIA
-2.05837	0.0042783	0.014906	down	5	5	2	16	20	17	
-3.95884	4.27E-06	3.30E-05	down	1	3	1	18	46	21	
-1.94905	0.0004346	0.002059	down	4	10	9	30	37	26	MAGIX
-5.49169	6.85E-05	0.000401	down	1	0	0	21	18	11	MYT1L
-4.01616	3.82E-06	2.98E-05	down	1	2	2	39	24	19	PCLO
-2.15296	0.0149923	0.042846	down	1	4	3	9	11	17	
-1.61809	0.0005405	0.002498	down	16	10	13	29	45	52	
-1.33576	0.0002275	0.001169	down	21	24	24	55	78	51	
-1.19937	1.89E-07	1.93E-06	down	140	136	144	235	482	319	TTC28-AS1
-2.91703	1.90E-35	2.67E-33	down	47	67	65	387	513	516	GBP2
-1.06759	0.0038863	0.013767	down	38	26	61	102	99	67	ZNF556
-2.12575	3.13E-06	2.50E-05	down	10	15	10	35	66	62	EPHX4
-2.98291	7.13E-09	9.32E-08	down	4	5	13	42	88	54	MYH7B

-5.25187	0.0012335	0.005174	down	0	0	0	3	12	8	TMEM47
-2.68328	1.21E-78	8.18E-76	down	792	755	815	4498	6219	5263	TRIM29
-4.16388	0.0089022	0.027795	down	1	0	0	5	12	4	
-1.53989	1.39E-05	9.61E-05	down	172	154	302	882	493	432	SORL1
-3.1909	9.25E-05	0.000525	down	2	1	6	34	41	10	MMP13
-3.08526	0.0102909	0.031398	down	2	1	0	7	9	11	ATP2C2
-4.4179	2.64E-05	0.000171	down	0	0	3	17	30	20	VANGL2
-1.28702	1.39E-09	2.03E-08	down	108	105	101	234	315	259	C1orf74
-7.82439	6.40E-10	9.81E-09	down	0	0	0	32	50	50	TDRD5
-4.65604	0.0108362	0.032778	down	0	0	0	8	4	2	MPPED1
-1.9843	0.0067364	0.02193	down	3	2	10	24	22	14	FSCN2
-1.57572	0.0011655	0.004917	down	14	16	10	25	65	40	
-1.44271	1.19E-11	2.31E-10	down	139	137	176	303	543	455	KIAA0895L
-1.15146	0.016189	0.045567	down	19	12	15	37	48	23	FRMD3
-3.98618	0.0179853	0.049761	down	0	1	0	4	13	2	FOXE3
-2.25561	1.02E-10	1.73E-09	down	13	24	26	89	134	94	SYCP2
-2.99744	3.95E-18	1.62E-16	down	51	61	31	226	518	496	TP73
-3.00875	0.0011901	0.005013	down	5	4	7	102	11	7	PTPRT
-1.36197	7.10E-10	1.08E-08	down	238	208	177	483	737	491	CCDC120
-1.26868	9.19E-07	8.23E-06	down	744	1230	818	1596	3113	2543	EBPL
-2.12353	2.32E-06	1.90E-05	down	16	10	24	82	102	43	SPTBN5
-1.06722	9.91E-09	1.25E-07	down	226	241	224	443	628	462	TMEM62
-5.98721	5.28E-14	1.42E-12	down	1	3	0	78	112	79	PAK6
-1.01438	3.40E-06	2.69E-05	down	538	455	645	1260	1090	1035	CNST
-5.87012	6.70E-05	0.000394	down	0	0	0	7	18	10	DUOX1
-1.03832	9.07E-05	0.000516	down	60	59	87	134	190	120	
-1.21833	7.86E-05	0.000456	down	33	41	51	71	132	105	LOC102724023
-1.14033	2.59E-08	3.07E-07	down	295	349	266	605	865	663	SMAD6
-2.429	1.80E-11	3.40E-10	down	15	12	23	77	114	91	PDZD7
-1.98541	0.0029069	0.010746	down	7	2	8	14	36	22	
-1.02746	2.41E-05	0.000158	down	63	72	89	129	193	158	
-4.60146	1.66E-20	8.38E-19	down	4	6	4	125	135	95	SRGAP3
-4.51488	0.0035425	0.012718	down	1	0	0	13	8	4	
-1.26507	0.0111696	0.033594	down	13	29	12	28	69	45	
-1.84147	0.0002012	0.001047	down	9	8	11	26	44	36	
-8.25562	6.27E-10	9.62E-09	down	0	0	0	14	78	91	TNFRSF18
-5.88675	7.30E-05	0.000426	down	0	0	0	15	8	10	ZNF836
-2.95732	9.82E-09	1.25E-07	down	5	11	7	48	103	43	
-2.63051	0.0095351	0.029456	down	0	3	3	15	19	5	DPEP1
-2.45327	1.42E-19	6.64E-18	down	284	396	276	1271	2963	1504	S100A4
-1.83198	0.0044462	0.0154	down	4	12	12	11	58	40	
-1.13665	2.90E-10	4.66E-09	down	975	914	1452	2237	2926	2467	MPHOSPH8
-1.1488	4.48E-13	1.05E-11	down	520	551	702	1227	1583	1295	SERPINB5
-2.50849	0.0020237	0.007945	down	6	0	4	22	23	14	LOC101928100

-2.35761	8.22E-07	7.46E-06	down	11	6	13	47	78	38	IGSF1
-5.05272	2.05E-05	0.000137	down	0	0	2	19	27	23	CXorf57
-1.24289	6.29E-06	4.69E-05	down	161	155	106	238	476	367	GCHFR
-1.09153	1.14E-11	2.22E-10	down	460	494	508	994	1302	982	BPNT1
-3.55961	2.77E-05	0.000179	down	3	3	0	23	39	15	ZACN
-1.66213	0.0027466	0.010221	down	12	12	15	66	35	23	MMP19
-1.44432	8.44E-08	9.16E-07	down	186	166	287	700	547	512	DBT
-4.38046	5.83E-42	1.15E-39	down	237	216	207	2592	9181	3580	NR4A1
-4.28965	3.02E-17	1.14E-15	down	8	3	4	76	106	126	SLC44A5
-1.45132	3.74E-11	6.76E-10	down	158	178	225	370	651	598	IFI44
-7.64313	1.91E-12	4.17E-11	down	0	0	2	75	189	163	CLCA2
-1.3003	0.0003098	0.001533	down	39	33	87	144	139	114	TTLL7
-2.11956	0.0007912	0.003496	down	3	6	7	18	35	21	
-2.75404	3.60E-06	2.83E-05	down	10	4	3	33	60	31	
-2.18241	1.58E-07	1.64E-06	down	10	16	26	81	70	89	IFI44L
-5.48674	0.0004458	0.002105	down	0	0	0	4	14	9	CLEC12A
-1.61426	0.0001278	0.000699	down	13	15	19	54	54	41	
-2.50982	0.004683	0.016083	down	6	2	1	7	36	15	
-1.87118	7.41E-05	0.000432	down	13	7	13	34	45	47	STARD5
-1.2884	1.03E-08	1.30E-07	down	212	174	165	352	637	455	ALG14
-2.36565	4.88E-08	5.49E-07	down	10	13	11	63	74	47	
-2.1544	0.0088388	0.027625	down	1	3	6	13	24	10	HGD
-1.59735	0.0002014	0.001048	down	14	14	29	67	55	53	ARNT2
-4.47848	0.0037442	0.013326	down	1	0	0	12	5	7	CLDN16
-6.03123	1.49E-05	0.000103	down	0	0	1	7	50	22	LINC01451
-1.04086	0.0013665	0.005639	down	43	42	91	121	135	115	
-3.5393	5.30E-10	8.23E-09	down	3	7	4	53	83	38	CGB7
-2.40589	3.10E-20	1.53E-18	down	48	51	73	327	374	246	
-1.79488	1.54E-07	1.60E-06	down	29	39	27	74	157	124	LINC00852
-4.64965	0.0095299	0.02945	down	0	0	0	2	7	6	STX17-AS1
-1.8531	0.0067818	0.022055	down	9	6	2	14	31	22	
-5.49923	0.0003525	0.001713	down	0	0	0	10	10	6	HRG
-1.65782	0.0165691	0.046457	down	8	6	4	11	37	15	HAR1B
-2.94841	7.74E-06	5.65E-05	down	6	3	2	27	36	27	
-2.46682	0.0170726	0.047639	down	1	2	2	5	16	9	
-6.49598	2.13E-06	1.76E-05	down	0	0	0	15	24	14	LINC01508
-1.57613	0.0020366	0.007988	down	9	13	22	21	66	53	
-1.1438	0.0013492	0.005573	down	56	30	31	70	108	97	
-1.17327	2.93E-07	2.88E-06	down	202	220	164	363	619	437	LPAR2
-1.42253	5.10E-14	1.38E-12	down	522	474	702	1159	1996	1644	LRP1
-1.01086	1.19E-06	1.04E-05	down	172	143	189	352	377	325	KIAA1841
-1.68891	7.08E-08	7.80E-07	down	46	56	44	138	252	118	LINC00176
-1.03506	1.36E-07	1.42E-06	down	138	132	159	247	369	310	ZNF124
-1.1341	0.0007235	0.003239	down	50	43	32	68	134	94	PRTN3

-2.79835	0.0002132	0.001101	down	3	7	1	28	41	14	NOXO1
-6.24507	1.53E-06	1.31E-05	down	0	0	1	24	28	33	C2orf54
-2.91456	1.98E-17	7.63E-16	down	34	32	21	146	362	210	
-1.6715	8.93E-05	0.000509	down	15	31	22	82	101	46	
-3.23704	0.0013985	0.005756	down	3	1	1	4	26	22	
-1.64165	0.0001396	0.000758	down	13	29	18	41	92	69	ZNF774
-1.18535	0.0046225	0.015898	down	19	28	16	49	49	52	SBK3
-1.0889	3.61E-07	3.50E-06	down	468	479	459	689	1440	1089	PNKP
-2.22786	9.71E-05	0.000548	down	16	12	3	38	86	37	
-1.18592	4.30E-10	6.72E-09	down	700	696	667	1184	2215	1634	ABCA7
-4.13644	0.0084538	0.026627	down	0	0	1	5	8	7	SNCAIP
-1.50733	1.51E-20	7.64E-19	down	2021	2195	2020	5235	7938	5682	CNN2
-1.56067	1.62E-06	1.38E-05	down	186	191	112	277	745	563	CEBPA
-2.80463	0.0051643	0.017505	down	2	1	2	5	20	13	HMSD
-1.20266	4.97E-07	4.70E-06	down	73	96	121	216	254	223	SMPDL3A
-1.18598	2.64E-08	3.12E-07	down	643	473	883	1481	1699	1507	XRCC2
-1.02468	0.0003134	0.001548	down	589	557	1067	1867	1488	1181	MYO6
-1.61096	0.0001344	0.000733	down	21	15	23	34	94	66	
-1.07227	0.0020873	0.008154	down	38	33	82	85	131	117	
-3.6309	8.86E-11	1.52E-09	down	11	3	2	54	98	62	
-5.25871	9.69E-06	6.91E-05	down	2	0	0	23	44	16	
-1.83456	0.013501	0.039305	down	3	2	13	25	27	13	
-1.07452	0.0001119	0.000622	down	105	126	81	164	305	239	
-2.65868	0.0032711	0.011889	down	3	1	2	12	17	11	LPP-AS2
-2.31146	5.73E-07	5.35E-06	down	9	16	11	37	96	61	
-2.76252	1.56E-14	4.51E-13	down	19	11	17	102	141	93	TMEM40
-3.0981	5.37E-14	1.44E-12	down	12	7	11	71	122	80	HCP5
-1.40024	0.0001169	0.000646	down	23	34	47	72	148	73	TIAF1
-4.57402	0.002072	0.008104	down	0	0	1	8	11	8	LINC01465
-1.54199	4.28E-12	8.85E-11	down	465	422	687	1702	1706	1296	MYH10
-2.28653	4.79E-28	4.58E-26	down	1317	1060	1876	6823	7182	7264	MAL2
-3.15887	0.0001167	0.000646	down	4	10	10	163	24	15	NOTCH3
-1.1276	1.15E-10	1.94E-09	down	475	455	548	841	1494	1099	EBAG9
-1.28284	5.90E-09	7.82E-08	down	778	660	1174	2225	2181	2085	LPGAT1
-2.19274	1.20E-11	2.31E-10	down	21	19	33	105	125	116	CHRM3
-2.32112	8.64E-15	2.55E-13	down	74	52	106	286	638	317	CFAP44
-1.46657	0.0001025	0.000576	down	31	16	24	66	82	58	TTC30B
-2.23378	1.65E-05	0.000112	down	8	16	9	28	62	76	HSH2D
-1.36756	9.13E-06	6.54E-05	down	103	93	208	403	359	292	BAZ2B
-1.23509	0.0002068	0.001072	down	39	54	46	70	135	143	
-1.79525	0.0077596	0.024723	down	3	5	8	14	29	16	
-1.52838	0.0022586	0.008706	down	7	15	16	25	42	48	
-1.18621	5.23E-05	0.000316	down	36	55	50	91	131	116	LOC102724200
-5.15918	1.97E-07	2.01E-06	down	1	2	0	26	67	25	

-2.82296	3.95E-07	3.80E-06	down	19	13	11	164	114	35	
-2.01156	0.0016138	0.006515	down	4	4	8	18	31	19	
-1.05688	6.30E-05	0.000373	down	59	57	94	130	189	138	
-3.50979	6.12E-06	4.56E-05	down	3	2	2	15	46	26	
-1.82367	6.19E-12	1.25E-10	down	40	43	43	126	194	152	SLC4A11
-4.38138	3.25E-06	2.58E-05	down	3	1	0	36	19	30	
-4.75191	0.0061039	0.020138	down	0	0	0	4	8	4	
-1.40599	0.0029774	0.010956	down	16	20	9	32	59	38	LINC00689
-1.29426	2.52E-16	8.63E-15	down	1034	908	1045	2431	2904	2329	MAVS
-1.86285	2.47E-08	2.93E-07	down	31	31	26	73	153	118	EBF4
-7.0618	8.09E-08	8.81E-07	down	0	0	0	19	37	23	LGR6
-2.15455	0.0165737	0.046457	down	2	1	5	15	12	9	GHRL
-1.20363	2.10E-12	4.54E-11	down	582	525	700	1408	1667	1260	PIK3C2B
-1.04494	1.79E-06	1.51E-05	down	207	171	162	309	509	372	RBPMS
-1.08755	0.0170499	0.047593	down	12	15	25	27	55	35	ZNF596
-1.12206	4.27E-06	3.30E-05	down	165	158	277	456	446	432	ZNF431
-1.75417	1.52E-18	6.48E-17	down	119	149	141	444	567	439	GM2A
-6.78148	1.10E-07	1.17E-06	down	0	0	1	27	65	36	
-1.3831	0.001486	0.006073	down	27	22	16	38	99	49	S100A2
-4.56895	0.017202	0.047971	down	0	0	0	1	11	3	STOML3
-2.5766	4.18E-10	6.55E-09	down	24	12	10	70	128	97	TP53TG1
-4.07662	0.0108327	0.032772	down	1	0	0	4	6	9	UNC5C
-1.01139	0.000531	0.00246	down	225	204	368	672	465	475	RAP2C
-1.15015	1.80E-09	2.58E-08	down	480	463	666	1214	1250	1211	PLA2G12A
-2.57282	1.84E-05	0.000123	down	6	5	4	21	38	36	RGMA
-2.47538	3.45E-05	0.000218	down	3	7	8	22	57	29	
-1.66919	9.45E-06	6.75E-05	down	16	21	22	64	81	52	
-2.49804	0.0027434	0.010215	down	2	2	4	8	21	19	
-1.05418	1.35E-08	1.67E-07	down	338	302	366	703	735	727	ARHGEF5
-1.95094	0.0005647	0.002596	down	12	4	8	21	43	35	
-4.91477	0.0049798	0.016976	down	0	0	0	9	6	2	CALCRL
-1.76943	0.0059041	0.019603	down	4	8	7	22	34	13	MIR3654
-2.15018	0.0076375	0.024387	down	0	15	7	37	54	14	COLQ
-1.23101	8.81E-11	1.52E-09	down	424	407	402	738	1263	1081	CPT2
-2.93213	1.99E-26	1.69E-24	down	37	43	45	339	424	240	KIAA1211L
-1.35726	0.0013589	0.005608	down	17	15	18	31	64	42	
-3.65576	0.0008921	0.003882	down	1	1	1	13	18	9	SH3PXD2A-AS1
-2.55138	0.0030482	0.011186	down	2	0	7	17	23	14	GPR83
-1.76923	2.75E-06	2.22E-05	down	23	18	18	70	87	55	
-1.17078	0.0073504	0.023637	down	14	22	26	29	75	46	
-2.37627	8.86E-06	6.37E-05	down	11	5	8	28	70	37	
-1.53281	0.0162412	0.04568	down	5	4	21	19	41	30	
-3.88887	6.38E-05	0.000377	down	3	1	0	12	33	20	
-4.39552	0.0042313	0.014773	down	0	1	0	10	6	7	

-3.75859	0.0053622	0.018073	down	0	2	0	8	12	9	
-1.843	0.0031101	0.011389	down	3	10	6	20	28	24	
-1.25546	1.15E-06	1.01E-05	down	93	116	78	217	299	213	KCNJ14
-3.86196	7.55E-06	5.53E-05	down	3	0	2	26	31	19	LOC102723854
-2.20736	0.0114178	0.034216	down	5	2	1	13	14	12	CLEC18A
-2.96747	0.0042446	0.01481	down	0	3	2	5	24	14	
-2.36566	1.35E-06	1.17E-05	down	18	10	24	136	75	56	RNF43
-1.9359	0.000127	0.000696	down	12	21	64	161	100	102	CACNB4
-4.86243	0.0071791	0.023145	down	0	0	0	1	11	6	IFNL1
-1.555	6.15E-10	9.47E-09	down	229	231	212	417	867	832	RAPGEFL1
-3.88899	0.0177186	0.049189	down	0	1	0	3	7	7	ELN
-4.37689	1.64E-91	1.35E-88	down	103	119	120	1874	3640	2124	ANXA8
-1.02494	0.0141743	0.040896	down	33	29	16	40	78	54	
-1.21335	0.0001603	0.000855	down	39	49	44	80	164	86	
-2.47648	0.0079533	0.025234	down	2	3	1	8	16	12	
-1.64983	5.67E-07	5.30E-06	down	28	30	26	70	127	85	
-6.66656	7.33E-07	6.72E-06	down	0	0	0	17	23	19	
-1.27555	0.0004875	0.002277	down	20	30	27	48	90	61	
-2.58347	5.68E-08	6.36E-07	down	12	9	8	38	96	55	
-1.30979	2.26E-12	4.87E-11	down	332	384	381	946	1084	814	MYD88
-1.43277	6.44E-08	7.12E-07	down	84	75	124	175	331	299	FAM81A
-1.40508	0.0029223	0.010791	down	17	21	10	36	42	56	TEX19
-1.67871	5.52E-06	4.17E-05	down	19	15	25	60	76	61	TSHZ2
-4.06904	0.0162877	0.045784	down	0	0	1	1	11	8	LOC101929231
-3.29241	0.0047217	0.016195	down	1	1	1	7	18	7	
-3.32754	6.40E-17	2.32E-15	down	10	15	26	97	261	189	SCARF1
-2.4836	0.0007142	0.003201	down	1	5	11	13	61	29	
-2.24763	0.0052853	0.017858	down	2	3	5	7	25	19	
-2.67006	0.0020381	0.007992	down	0	2	12	32	47	12	
-1.26028	1.69E-06	1.43E-05	down	97	68	83	144	273	217	PXN-AS1
-1.12961	9.83E-05	0.000554	down	37	54	50	98	126	100	LINC00346
-2.71821	0.0177291	0.049209	down	1	0	5	25	6	6	KLRC4-KLRK1
-1.41101	0.0023154	0.008878	down	11	10	18	32	42	34	
-1.41245	6.27E-07	5.82E-06	down	40	38	48	102	152	100	PGBD4
-1.04968	0.0001246	0.000685	down	54	51	86	121	170	122	TRAF3IP2-AS1
-2.57568	9.18E-05	0.000522	down	4	2	7	20	39	23	
-2.70731	1.58E-06	1.35E-05	down	9	3	6	27	52	46	IPCEF1
-5.06784	4.70E-130	########	down	127	157	134	4872	5993	3906	ALDH3A1
-4.46042	1.41E-17	5.51E-16	down	4	5	4	81	149	77	STYK1
-1.74831	0.0005498	0.002535	down	9	11	13	24	64	32	GVQW2
-3.34559	8.50E-06	6.12E-05	down	3	0	5	22	36	27	
-3.51348	6.32E-57	2.40E-54	down	147	101	139	1584	1774	1249	LIMK2
-2.04657	0.0017922	0.007154	down	3	9	7	36	30	15	CDRT1
-1.96952	0.0001409	0.000764	down	9	11	8	23	59	37	

-3.08621	1.55E-12	3.43E-11	down	10	7	8	62	83	78	OFCC1
-4.46694	4.67E-26	3.81E-24	down	12	5	5	131	234	156	
-2.48464	0.0004707	0.002209	down	6	3	3	12	29	31	LOC100506688
-3.70609	3.01E-11	5.49E-10	down	3	9	3	42	91	78	SYNE4
-1.76482	0.0020502	0.008034	down	7	8	6	19	32	25	SERPINF1
-1.27907	0.0001902	0.000996	down	30	36	54	111	118	72	CARD6
-2.95287	6.93E-08	7.64E-07	down	4	3	11	37	58	50	KLRC2
-6.99627	1.16E-07	1.23E-06	down	0	0	0	21	34	20	RAB19
-1.0725	0.0002939	0.001465	down	54	69	68	87	196	148	
-2.15554	0.0106612	0.03232	down	5	2	4	13	35	6	FBXW10
-1.15804	2.48E-07	2.48E-06	down	127	190	163	347	436	343	SCYL3
-1.7774	0.0089198	0.027841	down	7	4	6	27	15	17	
-3.297	2.18E-13	5.32E-12	down	10	6	16	60	165	112	MUC5AC
-5.54502	0.0003081	0.001527	down	0	0	0	6	15	7	
-1.70174	3.41E-12	7.15E-11	down	97	79	84	213	411	283	SFXN2
-1.24385	0.0103796	0.031633	down	20	23	10	26	63	48	FOXD3-AS1
-2.35948	4.42E-42	8.93E-40	down	4084	3598	3806	20706	####	####	ITGB4
-10.5261	1.69E-33	2.14E-31	down	0	0	3	934	2049	1734	
-1.47825	2.18E-06	1.80E-05	down	66	54	38	113	205	156	BORCS8
-5.22107	6.62E-23	4.34E-21	down	7	4	0	130	167	137	ETV7
-1.21404	0.002202	0.008532	down	19	32	28	38	91	68	
-6.10233	1.93E-05	0.000129	down	0	0	0	15	12	12	
-1.2943	0.0043569	0.015144	down	61	21	84	187	126	93	
-1.01728	0.0003072	0.001524	down	119	76	73	165	212	196	HOMEZ
-3.41021	6.18E-73	3.36E-70	down	156	186	203	1607	2756	1804	LINC00842
-5.38895	1.84E-83	1.34E-80	down	21	15	16	646	985	680	AATK
-1.6105	4.09E-09	5.53E-08	down	121	88	98	201	438	368	DDN
-1.83478	0.0024532	0.009292	down	4	10	6	20	26	29	
-1.40389	0.0012195	0.005122	down	22	12	20	32	59	60	
-2.37548	2.55E-05	0.000166	down	6	5	10	21	59	37	LOC101927503
-1.33245	2.44E-09	3.39E-08	down	128	100	162	330	389	301	DOPEY1
-4.9349	4.11E-07	3.96E-06	down	1	0	2	28	39	29	SOX2
-1.83623	0.0141768	0.040897	down	7	5	3	9	37	14	TMIE
-1.1878	0.0155004	0.04406	down	10	11	26	35	35	39	LINC00598
-5.58859	0.0002386	0.001219	down	0	0	0	6	10	12	AATBC
-1.13928	0.0007989	0.003525	down	1327	967	2248	4649	2678	2554	CD109
-3.33227	1.54E-09	2.22E-08	down	5	3	10	47	104	43	PTGS2
-1.10849	2.13E-09	3.01E-08	down	196	216	233	458	525	467	BCKDHB
-1.20586	2.40E-08	2.86E-07	down	133	103	167	284	365	319	ANKEF1
-2.52643	0.0003284	0.001613	down	4	5	4	10	42	30	
-1.31918	3.99E-05	0.000249	down	27	40	44	70	129	95	ALKBH6
-2.09853	1.02E-06	9.05E-06	down	23	51	15	99	187	130	MYL5
-4.53581	0.0003124	0.001543	down	0	2	0	11	28	12	
-5.28662	0.0008262	0.003632	down	0	0	0	5	10	8	EPB41L4A-AS2

-1.84858	4.94E-05	0.0003	down	12	18	12	40	84	40	ANKHD1-EIF4EBP3
-2.18015	2.23E-14	6.31E-13	down	77	42	67	250	418	230	C6orf223
-7.3333	1.57E-08	1.92E-07	down	0	0	0	33	36	24	ANKRD20A11P
-4.41953	0.0170514	0.047593	down	0	0	0	5	3	4	MIR3124
-1.00527	1.79E-10	2.97E-09	down	977	939	1075	1613	2685	2074	UTP14A
-2.29744	0.0121549	0.036066	down	0	3	5	13	19	9	C20orf202
-1.08775	2.89E-09	4.00E-08	down	502	451	431	930	1274	912	CCDC97
-1.19688	9.95E-14	2.56E-12	down	342	369	441	819	1081	866	GATS
-4.66597	0.0018188	0.007252	down	0	0	1	10	14	5	RHBG
-1.08166	1.18E-08	1.47E-07	down	682	635	915	1671	1735	1464	NPTN
-1.78661	0.0022949	0.008811	down	4	10	8	18	36	27	RIMS3
-2.59902	0.0004095	0.001956	down	8	4	1	16	50	22	KCNQ4
-4.59044	3.05E-07	2.99E-06	down	0	3	1	37	28	34	PADI2
-7.56684	2.58E-08	3.06E-07	down	0	0	0	61	25	18	SYT11
-3.39245	8.55E-17	3.07E-15	down	37	33	17	394	407	167	SPOCK2
-1.6642	4.24E-06	3.28E-05	down	19	30	33	57	123	97	
-2.37524	0.0005134	0.002388	down	4	4	7	38	27	14	TNFSF15
-2.29681	0.0088113	0.027564	down	0	5	4	14	23	10	ATG9B
-1.32646	1.21E-06	1.05E-05	down	62	55	64	122	231	133	
-3.93158	7.16E-08	7.87E-07	down	1	4	2	25	42	46	STAB1
-1.00868	0.0147406	0.04226	down	59	42	51	155	93	62	FRY
-1.69566	3.54E-06	2.79E-05	down	51	60	27	103	228	158	TRPM2
-1.46619	2.72E-14	7.61E-13	down	305	323	340	671	1273	916	LPIN3
-6.7783	1.51E-06	1.30E-05	down	0	0	0	8	44	16	GCGR
-1.02662	1.68E-05	0.000114	down	266	220	369	630	725	450	KDM5B
-1.0746	2.82E-07	2.78E-06	down	458	413	417	644	1275	992	TOE1
-5.17742	0.0005435	0.002511	down	0	0	1	23	5	10	KANK4
-1.36598	2.01E-10	3.30E-09	down	551	475	685	1667	1470	1375	INADL
-1.01917	0.0034146	0.012326	down	58	41	32	95	93	90	ZSWIM3
-1.39504	0.0029213	0.010789	down	21	21	9	40	47	55	AMN
-1.86255	1.13E-20	5.84E-19	down	255	339	271	904	1388	1056	SPINT1
-3.77115	0.0049977	0.017022	down	0	1	1	5	12	12	LRTM2
-2.20895	1.73E-05	0.000117	down	7	6	10	30	41	40	DEPDC4
-2.49341	0.004565	0.015726	down	9	1	2	6	43	27	ADGRB1
-2.19511	0.0001744	0.00092	down	5	9	7	17	43	43	AQP1
-2.18916	0.0099649	0.030591	down	2	1	7	8	23	17	
-4.99792	0.0037931	0.013482	down	0	0	0	4	3	11	CAMK2B
-1.03382	4.51E-06	3.47E-05	down	377	331	355	502	1118	731	CROCC
-1.23657	1.75E-06	1.48E-05	down	145	161	108	258	471	324	LMTK3
-1.92812	9.69E-05	0.000547	down	10	7	29	62	68	48	FRRS1
-1.99965	1.85E-22	1.14E-20	down	600	585	484	1722	2974	2454	CDK18
-1.53536	1.60E-08	1.96E-07	down	64	81	65	145	267	238	P2RX5
-1.34886	0.0001625	0.000865	down	37	25	51	111	86	95	GATSL2
-2.58722	6.55E-12	1.32E-10	down	9	20	27	98	156	100	MTUS2

-1.76171	6.74E-11	1.19E-09	down	402	315	274	771	1863	1047	ID3
-1.04902	8.72E-07	7.87E-06	down	241	191	206	349	624	439	CASP9
-1.08818	1.97E-07	2.01E-06	down	108	100	125	225	292	225	IBA57
-3.33848	0.0040635	0.014283	down	1	1	1	5	17	11	C16orf71
-1.96225	7.92E-11	1.37E-09	down	58	59	35	157	252	225	CLDN7
-2.09227	7.54E-14	1.99E-12	down	210	196	177	1040	995	558	NEURL1
-1.14962	0.0004432	0.002094	down	63	65	114	223	197	127	SH3PXD2A
-2.73342	0.0152112	0.043374	down	0	2	2	10	7	10	LOC255187
-2.22156	4.62E-21	2.54E-19	down	154	171	164	532	1198	738	YJEFN3
-4.94318	0.0012965	0.005393	down	1	0	0	2	16	18	
-1.96259	0.0010827	0.004604	down	8	2	12	25	36	28	NDUFB2-AS1
-1.5033	0.0018644	0.007408	down	26	30	35	27	159	99	FZD10-AS1
-7.082	7.05E-08	7.76E-07	down	0	0	0	25	34	20	
-1.1206	0.00034	0.001662	down	48	52	44	81	165	92	GET4
-1.04905	0.0161425	0.045466	down	39	18	25	72	51	51	
-4.04222	6.00E-56	2.19E-53	down	31	49	52	530	1015	771	TSPAN1
-2.1932	2.18E-09	3.06E-08	down	36	33	27	108	266	108	ARTN
-2.90749	3.11E-16	1.05E-14	down	18	14	12	99	144	107	CCDC149
-2.11256	0.0113378	0.034019	down	3	4	2	7	18	17	
-4.05395	0.0015003	0.006122	down	1	1	0	10	12	13	
-2.44024	6.72E-21	3.62E-19	down	68	47	100	392	439	371	PPFIBP2
-1.4103	0.0120615	0.035867	down	9	6	10	20	30	20	
-4.22821	1.17E-10	1.96E-09	down	6	3	0	60	77	43	CYB5R2
-3.68259	0.0033067	0.011996	down	1	2	0	26	5	7	BTK
-4.63901	2.28E-10	3.70E-09	down	4	1	1	40	78	43	
-5.5428	0.0005386	0.002491	down	0	0	0	4	20	5	CT62
-1.60825	9.50E-06	6.78E-05	down	47	18	56	104	163	119	KIAA1456
-2.43867	0.0001062	0.000594	down	19	25	32	286	56	50	FBN3
-4.19834	0.0078972	0.025093	down	0	0	1	9	5	6	RIC3
-7.90208	1.93E-96	1.93E-93	down	2	10	8	1343	1859	1814	IRF6
-1.52265	2.62E-18	1.10E-16	down	746	592	666	1657	2574	1885	FGFR3
-3.3463	7.17E-05	0.000419	down	2	1	3	22	29	13	TTC34
-3.60108	9.46E-14	2.45E-12	down	11	3	9	57	125	115	ATAD3C
-1.40627	1.57E-06	1.34E-05	down	1308	1560	2348	6030	4972	3026	F3
-1.78707	0.0116747	0.034892	down	2	6	18	37	40	14	
-2.10035	0.0136852	0.039744	down	2	2	6	21	14	8	PKD1L2
-5.25895	0.0024228	0.009197	down	0	0	0	1	16	7	
-1.50747	0.0152918	0.043571	down	11	6	6	12	34	25	
-2.86933	0.0013804	0.005689	down	1	2	3	9	17	20	
-5.24978	0.0015151	0.00617	down	0	0	0	3	6	13	LINC00939
-5.44129	0.0006276	0.002852	down	0	0	0	3	12	11	
-6.1075	2.90E-05	0.000186	down	0	0	0	16	18	6	ANTXRL
-4.69915	0.0021119	0.008228	down	1	0	0	3	12	15	
-1.04763	1.09E-12	2.46E-11	down	765	687	828	1400	1990	1573	PHLDB1

-5.7997	8.58E-05	0.000491	down	0	0	0	12	12	8	
-2.25567	0.0011356	0.0048	down	3	6	5	16	42	15	NAIP
-1.34659	7.45E-17	2.69E-15	down	1591	1721	1634	3473	5574	4336	BLCAP
-3.02728	0.0161436	0.045466	down	0	1	2	11	10	4	
-2.90056	0.0054341	0.018273	down	0	2	4	25	6	12	SMPD3
-1.16459	3.19E-11	5.80E-10	down	388	334	452	751	1200	832	SLC25A13
-4.00644	0.0002588	0.001309	down	3	0	0	17	20	14	PADI3
-3.39777	8.44E-13	1.92E-11	down	13	21	13	249	181	80	PADI1
-1.25318	8.23E-05	0.000474	down	29	34	34	70	87	85	IQCD
-3.0633	0.0004795	0.002246	down	1	2	3	10	28	16	
-3.98182	0.0004038	0.001933	down	1	1	1	5	21	25	
-4.67583	0.0078267	0.024891	down	0	0	0	3	6	6	
-1.28546	1.11E-05	7.83E-05	down	98	87	55	179	258	189	ZNF446
-1.25382	0.0001078	0.000602	down	133	104	63	248	323	195	ZNF324
-1.07646	1.10E-05	7.79E-05	down	349	322	566	985	888	781	RABGAP1L
-5.06794	0.0020584	0.008061	down	0	0	0	8	6	5	FGD3
-6.19177	1.91E-06	1.59E-05	down	1	0	0	24	28	30	
-1.49011	2.30E-12	4.95E-11	down	152	134	130	326	540	381	FA2H
-2.55246	6.48E-12	1.30E-10	down	17	11	17	72	104	101	PDK4
-5.08529	0.0044057	0.015288	down	0	0	0	5	15	1	
-1.22663	0.0035641	0.012785	down	33	18	18	57	52	59	IL22RA1
-1.33062	3.30E-08	3.82E-07	down	386	382	316	621	1388	951	CNKSR1
-1.33185	3.58E-07	3.48E-06	down	234	233	169	367	786	584	ARHGAP33
-1.51412	3.94E-06	3.06E-05	down	183	116	149	589	438	283	HIVEP3
-6.04175	2.29E-24	1.67E-22	down	1	8	1	119	356	248	SYTL1
-3.62702	1.18E-05	8.28E-05	down	2	1	3	13	41	26	MMP10
-1.73123	0.00028	0.001406	down	9	10	12	32	43	33	TMPRSS5
-1.68223	7.98E-15	2.36E-13	down	311	322	472	1258	1474	948	PLEKHA7
-2.05405	1.69E-10	2.81E-09	down	31	38	31	92	202	153	MLYCD
-1.38624	2.81E-05	0.000181	down	39	25	32	87	96	79	UPI00024673AA
-1.09619	2.50E-07	2.49E-06	down	193	198	263	411	679	392	
-2.6644	0.0001617	0.000861	down	4	2	4	18	29	20	LOC101928255
-1.72183	5.87E-06	4.40E-05	down	26	21	23	50	125	75	NOP14-AS1
-1.81397	2.19E-07	2.21E-06	down	60	39	28	107	205	170	
-1.3929	0.0002597	0.001314	down	102	108	65	110	404	287	PI4KAP1
-1.97321	1.10E-10	1.86E-09	down	37	45	30	155	158	147	KIAA0101
-4.89752	0.0051348	0.017424	down	0	0	0	5	11	2	
-2.66928	2.04E-06	1.70E-05	down	5	7	5	41	36	35	LOC101928595
-1.40933	0.0157238	0.044548	down	11	5	11	28	20	25	HOXC13-AS
-1.28905	0.0025505	0.009605	down	21	16	25	30	62	68	PSTPIP2
-4.9416	6.97E-11	1.22E-09	down	1	3	1	48	64	50	ARL11
-2.49691	0.0070898	0.022912	down	0	3	4	16	12	12	MASP1
-2.44751	2.37E-29	2.49E-27	down	91	80	121	551	594	502	PRKACB
-1.16798	0.0003218	0.001584	down	315	340	209	349	1056	739	RHBDL1

-10.0575	1.05E-16	3.74E-15	down	0	0	1	283	637	322	GPX2
-4.45113	0.016794	0.046996	down	0	0	0	4	7	2	
-4.96576	0.002794	0.010376	down	0	0	0	5	6	7	
-4.29009	0.0011588	0.00489	down	0	1	1	4	24	15	
-4.65765	0.0023619	0.00902	down	0	1	0	3	17	10	
-2.64455	6.55E-05	0.000386	down	2	6	4	25	32	22	SMIM1
-6.99764	5.42E-36	7.86E-34	down	3	3	3	236	506	485	SEPP1
-3.48758	5.16E-15	1.57E-13	down	7	5	12	93	88	95	PDE3B
-1.17914	3.21E-11	5.85E-10	down	815	850	752	1543	2482	1817	MBD6
-3.22816	3.79E-05	0.000237	down	1	5	2	24	42	15	
-1.89463	3.00E-09	4.14E-08	down	91	85	124	489	427	227	PTPRB
-4.10059	0.0103303	0.031493	down	0	1	0	5	11	4	
-1.26123	1.18E-13	2.99E-12	down	5559	4861	4765	11756	####	####	PIEZO1
-4.61978	1.28E-22	8.06E-21	down	1	9	10	106	241	178	MUC5B
-1.90666	5.99E-09	7.94E-08	down	39	46	92	146	302	249	SNAI2
-7.74313	1.25E-09	1.83E-08	down	0	0	0	27	59	41	ANKRD65
-2.48858	0.0092339	0.028656	down	3	3	2	29	6	9	EPHA3
-1.75085	2.49E-07	2.48E-06	down	111	141	64	240	567	364	
-2.70487	0.0002775	0.001394	down	4	3	2	21	19	21	
-1.6753	0.0068674	0.022307	down	8	5	7	15	36	18	
-1.61793	0.0031163	0.01141	down	7	9	10	17	37	31	
-3.97021	0.013618	0.039567	down	0	1	0	4	8	6	
-2.70606	1.13E-12	2.55E-11	down	13	12	20	73	149	91	
-1.07784	0.0011142	0.004719	down	37	38	29	60	97	77	
-7.0357	6.11E-94	5.53E-91	down	21	4	19	1474	2915	1776	CCDC187
-1.41668	0.0161551	0.045478	down	13	9	7	13	44	28	LOC105370681
-3.22726	0.0026933	0.010064	down	0	2	2	11	8	19	
-4.69693	0.0014128	0.005806	down	0	1	0	8	9	12	
-2.02117	1.53E-05	0.000105	down	11	7	14	44	45	45	
-1.1887	3.92E-09	5.32E-08	down	310	271	309	501	970	700	SPRYD4
-4.85955	3.65E-08	4.20E-07	down	3	1	0	24	65	38	
-3.65367	1.27E-07	1.34E-06	down	2	3	7	17	88	59	PYCARD
-1.57106	1.29E-07	1.36E-06	down	58	68	55	115	254	210	HSD11B2
-1.81353	0.0026182	0.009819	down	8	3	9	22	27	24	
-1.23338	0.0002826	0.001417	down	35	44	60	68	133	143	REEP1
-2.96362	1.80E-08	2.18E-07	down	9	10	2	50	74	52	
-3.03477	4.23E-11	7.61E-10	down	4	10	13	76	72	79	LINC00562
-4.39149	0.0039552	0.013959	down	0	0	1	7	11	6	
-1.97025	1.44E-06	1.24E-05	down	18	24	23	105	110	51	
-5.15329	0.0024843	0.009385	down	0	0	0	11	7	2	BFSP2-AS1
-1.04352	0.0045035	0.015548	down	41	34	31	48	85	99	
-2.69355	4.30E-06	3.32E-05	down	6	7	3	26	53	33	
-5.03866	0.0045108	0.01557	down	0	0	0	9	9	1	
-5.58262	3.05E-61	1.29E-58	down	6	14	19	606	867	493	

-1.38893	3.27E-12	6.89E-11	down	242	220	245	467	878	634	PRR15
-1.42828	6.89E-06	5.09E-05	down	314	203	422	1116	695	712	KIAA2018
-2.97043	5.03E-06	3.83E-05	down	2	5	4	23	36	32	IGSF10
-1.64582	0.0002715	0.001368	down	16	12	17	29	77	46	CTAGE4
-8.25513	1.73E-11	3.28E-10	down	0	0	1	108	160	81	NTF4
-2.94626	0.0002866	0.001434	down	1	2	4	16	21	19	PLEKHH2
-2.38455	2.12E-07	2.14E-06	down	11	9	8	49	61	44	WFIKKN1
-1.02789	4.94E-05	0.0003	down	168	186	148	237	514	357	UQCR11
-3.04772	6.30E-17	2.30E-15	down	11	15	19	110	184	101	
-2.714	3.95E-15	1.22E-13	down	37	14	24	146	223	153	ADGRE2
-1.55841	4.68E-05	0.000286	down	20	21	18	47	85	54	
-5.76816	1.63E-05	0.000111	down	0	1	0	14	24	24	
-3.76927	0.0077814	0.024763	down	1	0	1	2	17	11	KCNJ18
-1.95275	0.0009203	0.00399	down	8	9	6	20	56	22	
-3.39524	1.10E-18	4.75E-17	down	14	25	16	112	252	257	ENTPD2
-2.67199	0.0091802	0.028513	down	2	0	3	8	12	13	
-1.1482	2.34E-05	0.000154	down	908	964	573	1298	2668	1940	PHPT1
-2.32858	2.06E-06	1.71E-05	down	7	10	13	29	67	64	
-1.91428	2.08E-08	2.51E-07	down	20	25	28	102	101	82	
-1.02726	1.31E-05	9.12E-05	down	408	312	324	581	1113	604	
-4.77926	0.0003181	0.001568	down	0	2	0	35	13	7	NEURL1-AS1
-1.44842	0.0011408	0.004819	down	22	18	16	37	91	39	SCX
-3.44906	0.002583	0.009708	down	2	0	1	12	12	10	LINC01372
-1.12199	4.78E-10	7.43E-09	down	13000	####	####	22338	####	####	
-3.07773	3.17E-10	5.05E-09	down	7	21	12	56	207	111	
-1.3288	2.99E-06	2.39E-05	down	83	116	88	158	355	266	CCDC78
-2.20959	0.0003358	0.001644	down	7	8	3	20	46	25	SSTR5
-5.44787	1.95E-99	2.47E-96	down	34	25	54	1691	1700	1664	MYO1D
-2.33204	7.64E-06	5.59E-05	down	7	6	9	28	55	35	
-1.83334	3.89E-16	1.31E-14	down	79	83	104	250	448	309	IL11RA
-3.28757	4.93E-09	6.61E-08	down	6	5	7	28	103	61	RASGRP2
-5.81299	0.0001795	0.000944	down	0	0	0	13	17	3	AP3B2
-2.61783	0.0135701	0.039463	down	0	2	3	14	10	7	
-3.0403	4.74E-09	6.39E-08	down	7	6	5	39	75	45	
-1.33063	2.01E-07	2.04E-06	down	80	65	71	144	263	174	
-1.95847	8.49E-27	7.57E-25	down	140	167	168	564	745	630	OPN3
-1.52318	1.35E-05	9.33E-05	down	40	38	29	72	169	94	SARM1
-1.13365	0.0070332	0.022748	down	25	25	21	31	88	51	
-1.69572	6.02E-09	7.98E-08	down	36	49	39	105	180	143	CYP2R1
-1.24533	0.0013001	0.005403	down	34	35	25	48	127	69	NDUFA13
-1.64815	1.25E-08	1.55E-07	down	167	135	116	274	708	451	AMDHD2
-8.13616	1.81E-10	2.99E-09	down	0	0	0	27	85	57	CCDC64B
-4.67609	0.0014878	0.006079	down	0	0	1	7	11	11	NTN3
-1.15955	3.73E-08	4.28E-07	down	250	181	227	407	693	466	ZNF75A

-4.92409	0.006666	0.021738	down	0	0	0	10	6	1	HTR3E
-5.57572	7.97E-09	1.03E-07	down	2	0	1	65	46	35	ANKRD22
-1.23926	8.42E-11	1.46E-09	down	291	289	327	553	1031	703	ARHGEF39
-7.94543	4.09E-94	3.89E-91	down	4	5	11	1701	2069	1371	KRT5
-5.46448	0.0003823	0.00184	down	0	0	0	7	12	7	
-2.16618	7.07E-10	1.07E-08	down	18	15	32	94	114	94	METTL25
-3.05573	0.0120861	0.035924	down	1	2	0	11	8	7	TBX15
-3.48156	7.33E-09	9.54E-08	down	6	3	3	39	70	35	
-4.6805	0.0023712	0.009044	down	0	1	0	4	21	6	
-1.48939	0.0054408	0.018292	down	9	11	9	19	44	25	
-4.00028	0.0153578	0.04372	down	0	0	1	4	12	3	
-2.02168	0.0018893	0.00749	down	11	2	6	20	41	22	
-4.63758	0.0031253	0.011436	down	0	1	0	6	21	3	
-1.79695	1.03E-06	9.13E-06	down	18	15	29	60	95	71	LIPT2
-2.39014	1.28E-07	1.35E-06	down	9	13	9	39	76	59	UCP3
-2.4239	0.0001117	0.000622	down	5	6	3	22	33	25	UPI0004620B7C
-1.73687	0.0109673	0.0331	down	10	11	1	20	34	26	
-4.85812	5.02E-05	0.000304	down	0	2	0	17	27	18	
-2.04024	1.37E-05	9.49E-05	down	32	10	11	60	93	80	TEX9
-3.28511	0.0057575	0.019184	down	1	2	0	9	17	6	
-1.0133	1.44E-06	1.24E-05	down	1912	1867	3125	4764	4895	4601	KLF5
-3.02939	2.57E-21	1.44E-19	down	48	50	43	209	582	460	COL9A3
-2.40593	0.0008567	0.003744	down	5	6	2	34	21	16	
-1.15109	1.32E-05	9.18E-05	down	207	208	400	660	648	535	LINC00641
-1.23707	0.0017539	0.007024	down	23	20	38	77	61	56	F8
-1.66706	8.70E-10	1.30E-08	down	47	36	49	115	178	149	NAPEPLD
-1.20866	2.84E-07	2.80E-06	down	77	80	79	169	204	198	UBOX5
-1.80244	0.0007804	0.003457	down	9	7	13	23	59	27	PDF
-2.5587	0.0045018	0.015547	down	3	4	7	60	11	7	ROBO2
-1.13492	0.0049435	0.016868	down	41	31	26	39	102	92	
-1.05235	8.86E-07	7.98E-06	down	312	228	358	468	795	700	ZNF37BP
-2.29958	6.35E-07	5.90E-06	down	14	8	9	40	59	62	
-9.0174	5.50E-18	2.23E-16	down	2	0	0	297	423	371	VILL
-1.24702	2.66E-11	4.90E-10	down	267	263	248	508	805	650	ROGDI
-4.05629	0.002064	0.00808	down	2	0	0	10	18	8	DPYSL4
-4.80397	5.04E-103	########	down	37	56	73	1436	1905	1500	AKR1C2
-5.12328	3.31E-15	1.03E-13	down	5	1	1	64	104	91	KDF1
-1.02328	1.60E-11	3.04E-10	down	898	799	1044	1766	2275	1787	SUPT20H
-3.53534	2.67E-36	3.96E-34	down	41	40	30	434	601	335	MEOX1
-2.80568	4.75E-05	0.00029	down	2	3	7	17	48	25	
-1.86562	6.49E-10	9.93E-09	down	54	27	55	145	223	154	TTLL11
-1.07132	4.89E-09	6.57E-08	down	207	186	246	377	580	458	SLC35E3
-1.23555	0.0015684	0.006353	down	17	28	26	50	83	45	FAM43A
-3.28528	8.45E-32	1.03E-29	down	101	120	148	668	1854	1358	ANO9

-1.34404	6.73E-05	0.000396	down	63	66	66	84	236	214	INPP5J
-4.69673	0.0120311	0.035805	down	0	0	0	7	1	6	
-1.75886	0.0085533	0.0269	down	6	7	4	19	31	12	LINC00239
-1.97987	0.0067496	0.021969	down	4	2	6	15	18	16	
-8.37654	2.23E-19	1.03E-17	down	1	3	2	1132	513	339	SCEL
-1.3085	3.10E-11	5.65E-10	down	145	127	144	314	448	326	SAYSD1
-1.75012	4.69E-19	2.10E-17	down	265	263	363	757	1398	1024	PHF11
-1.063	0.0017106	0.006869	down	34	36	52	71	81	111	PDK3
-2.06187	0.0019589	0.007732	down	10	7	13	76	18	27	PZP
-2.2713	1.75E-17	6.83E-16	down	86	110	80	301	619	515	ELMO3
-2.74713	0.000753	0.003349	down	4	1	3	10	29	19	
-1.15218	7.07E-10	1.07E-08	down	577	578	559	1230	1735	1077	PLEKHG3
-3.98702	2.23E-13	5.44E-12	down	4	3	6	54	106	60	PRSS22
-1.18965	0.0001251	0.000687	down	61	52	56	153	155	94	HSPA2
-1.21992	1.95E-09	2.76E-08	down	3572	3388	2789	5950	####	7931	MSLN
-1.10311	2.86E-08	3.36E-07	down	178	166	248	388	484	449	CLN5
-1.29648	2.96E-05	0.000189	down	76	52	105	226	213	147	CREG2
-1.45405	0.0014552	0.005958	down	11	15	15	42	44	31	CARD8-AS1
-1.25543	0.0069199	0.022435	down	13	11	19	36	44	27	PTOV1-AS1
-2.15212	1.12E-09	1.65E-08	down	27	30	34	176	130	107	ZDHHC21
-2.96307	3.61E-20	1.77E-18	down	48	58	72	606	526	292	
-5.21602	2.97E-21	1.66E-19	down	2	5	5	161	223	88	A2M
-4.273	0.0012813	0.005341	down	0	0	2	15	19	6	
-1.00083	2.43E-06	1.98E-05	down	443	452	354	684	1166	834	BDH1
-1.98114	0.0003301	0.00162	down	13	9	8	20	74	37	UPI0001AE6402
-2.2375	0.0106039	0.032178	down	3	2	3	18	9	11	CCDC116
-1.92109	0.0010813	0.004601	down	8	11	4	24	48	23	
-2.94756	7.05E-06	5.20E-05	down	3	7	3	25	61	24	
-2.44866	0.0086211	0.027095	down	1	4	2	10	25	7	NACAD
-3.85162	6.32E-29	6.45E-27	down	12	10	16	147	258	177	MYO1G
-6.60424	1.22E-28	1.21E-26	down	2	3	2	217	274	223	CCL28
-2.89277	4.70E-19	2.10E-17	down	29	13	37	173	227	209	RAPGEF5
-4.04111	2.69E-28	2.61E-26	down	34	23	11	295	602	328	DOK7
-1.15838	0.0004483	0.002116	down	76	87	51	104	243	174	ORAI3
-1.35363	2.08E-05	0.000138	down	29	35	36	90	102	75	PARD6A
-2.29444	1.05E-10	1.78E-09	down	20	28	19	126	117	99	DENND2C
-1.28648	0.0006016	0.002747	down	23	31	27	58	104	50	TMEM141
-2.83385	0.0119237	0.035529	down	2	1	1	3	14	14	LOC101559451
-1.51422	6.11E-08	6.79E-07	down	178	222	147	342	810	553	PLLP
-3.06406	0.0004611	0.002169	down	0	2	10	52	34	12	
-6.5285	3.57E-07	3.47E-06	down	1	0	0	33	46	26	PLXDC1
-1.17173	5.74E-07	5.36E-06	down	880	895	635	1360	2525	1976	GAS2L1
-1.00744	5.46E-06	4.12E-05	down	812	888	753	1155	2488	1697	CENPT
-3.93453	3.72E-06	2.91E-05	down	2	1	2	15	38	29	

-5.23091	0.0020114	0.007908	down	0	0	0	12	7	2	SLC6A14
-1.8173	1.41E-08	1.75E-07	down	215	196	482	1263	1035	854	ZBTB37
-2.79729	2.67E-06	2.16E-05	down	5	4	8	50	49	23	KIAA0825
-2.42073	1.47E-09	2.13E-08	down	14	12	14	75	93	57	PRC1-AS1
-1.58064	0.0074587	0.023933	down	17	8	4	28	32	32	NOTUM
-2.6408	0.0060315	0.01996	down	4	0	2	14	15	10	
-5.93598	0.000124	0.000682	down	0	0	0	11	23	3	ADAMTS7
-1.05542	2.02E-06	1.68E-05	down	159	124	211	322	442	309	FIG4
-1.961	1.59E-05	0.000108	down	16	10	38	71	129	61	SCHIP1
-1.22593	8.27E-12	1.64E-10	down	2450	2673	3678	6309	7406	7565	PERP
-1.16046	0.0010202	0.004378	down	200	197	383	834	476	415	ARFGEF3
-2.3905	1.52E-27	1.40E-25	down	96	101	156	619	676	613	EPM2A
-1.87141	3.82E-14	1.05E-12	down	127	164	215	472	665	788	METTL7A
-1.59664	1.32E-09	1.93E-08	down	56	40	58	150	181	155	IFNLR1
-1.15584	1.68E-07	1.73E-06	down	196	193	167	375	442	482	C19orf68
-4.53625	0.0128334	0.037645	down	0	0	0	3	8	3	
-2.39257	1.20E-06	1.05E-05	down	5	15	10	36	76	57	STAC3
-5.22087	1.06E-22	6.77E-21	down	6	1	4	137	184	111	
-2.63869	2.19E-08	2.62E-07	down	9	8	11	38	95	55	
-1.12115	1.21E-05	8.46E-05	down	94	74	68	158	212	173	ACOX3
-3.43324	0.0009314	0.004035	down	0	1	3	15	21	9	GRIN2C
-3.7744	1.63E-47	4.31E-45	down	27	25	29	300	506	371	MDFI
-1.26914	6.04E-06	4.51E-05	down	184	215	141	284	629	500	FDXR
-2.55661	9.00E-06	6.46E-05	down	4	11	5	31	66	31	CYGB
-1.77869	4.37E-21	2.42E-19	down	276	204	320	840	1097	927	BRCC3
-1.5909	2.15E-12	4.64E-11	down	129	168	126	352	539	464	TMEM143
-1.52079	0.0004543	0.002141	down	36	26	33	38	163	99	MLXIPL
-1.52362	4.84E-07	4.59E-06	down	86	97	59	182	369	207	LMNTD2
-2.53542	0.0002007	0.001045	down	4	3	5	15	26	32	
-2.26194	2.89E-23	1.96E-21	down	121	111	140	409	836	659	OLFML2A
-3.92633	0.0035789	0.012829	down	0	0	2	8	10	13	
-4.07662	0.0108327	0.032772	down	1	0	0	4	6	9	FOXN3-AS1
-4.72204	9.56E-05	0.00054	down	1	0	1	12	24	20	
-2.25894	0.0104743	0.031866	down	3	2	2	11	12	12	
-1.20854	3.01E-08	3.50E-07	down	254	229	189	434	659	561	GLTSCR1
-1.28353	9.04E-12	1.78E-10	down	422	488	505	876	1628	1175	RPL21
-2.65944	2.90E-18	1.21E-16	down	60	85	77	262	745	518	ZNF385A
-2.8847	1.49E-29	1.59E-27	down	195	137	112	965	1549	1011	SPHK2
-2.17103	0.0001527	0.00082	down	6	12	6	19	51	47	FAM212A
-1.25022	3.39E-12	7.13E-11	down	520	533	566	984	1801	1327	MPP3
-2.25861	2.62E-29	2.72E-27	down	469	384	579	2475	2441	2132	PBX1
-3.48787	3.25E-17	1.22E-15	down	41	37	12	233	599	294	JOSD2
-2.52603	2.73E-09	3.77E-08	down	10	11	10	58	69	60	PMEL
-3.69523	0.0016574	0.006674	down	3	0	0	7	17	18	SYN3

-1.94584	0.0001479	0.000797	down	5	11	11	34	44	31	
-2.84523	6.99E-31	8.09E-29	down	38	55	54	332	471	312	DKFZp434J0226
-3.86501	0.0038618	0.013696	down	1	0	1	7	8	15	ACPP
-2.49346	7.73E-11	1.34E-09	down	52	42	29	224	401	131	DOK3
-3.11158	7.08E-14	1.87E-12	down	22	24	19	280	156	133	TLL1
-1.33882	7.66E-13	1.75E-11	down	508	401	581	1294	1463	1156	SH3BP2
-1.27166	4.13E-08	4.69E-07	down	179	127	246	423	534	425	LPCAT2
-1.43745	9.05E-11	1.55E-09	down	641	620	874	1312	2347	2425	IFIT1
-3.62103	6.09E-05	0.000362	down	2	0	3	19	32	14	AQP5
-1.67982	1.55E-16	5.50E-15	down	526	548	897	1770	2766	2071	FAM46A
-2.15739	4.91E-14	1.33E-12	down	230	229	162	573	1504	965	ADAMTSL5
-2.6826	0.0071192	0.022987	down	3	5	3	53	3	11	PAX7
-1.46433	0.000152	0.000816	down	22	15	21	49	59	59	
-4.46054	1.33E-13	3.35E-12	down	4	1	6	45	133	83	KCNQ1
-2.25716	0.0092752	0.028761	down	2	5	1	10	16	15	
-1.0491	2.36E-10	3.82E-09	down	496	481	664	972	1390	1193	ANAPC4
-1.1714	0.0012606	0.005267	down	39	22	26	62	83	62	
-1.86227	5.26E-27	4.73E-25	down	1480	1526	1478	4213	7279	5897	FBLN1
-1.32842	1.49E-06	1.28E-05	down	105	90	177	339	304	306	TXNDC16
-1.37682	4.91E-06	3.75E-05	down	102	59	157	276	325	248	STX17
-5.04148	0.005296	0.017884	down	0	0	0	1	16	4	KIF12
-2.07861	0.0033742	0.012203	down	2	5	6	12	24	22	ANGPTL2
-4.83929	7.72E-70	3.86E-67	down	16	36	41	716	1152	936	CDC42BPG
-7.00021	1.09E-07	1.17E-06	down	0	0	0	18	31	26	NPTX1
-1.2601	2.18E-16	7.52E-15	down	2610	2461	2491	5831	7080	6091	LAMP1
-2.52486	0.0081174	0.025699	down	2	3	1	6	20	12	ATP6V0C
-1.66929	4.42E-18	1.82E-16	down	186	203	229	676	704	657	RALGPS1
-3.82425	1.02E-62	4.42E-60	down	70	81	53	877	1216	976	PRSS16
-2.14716	0.0004683	0.002199	down	6	4	8	31	35	17	FAM71F2
-1.56669	0.0023054	0.008843	down	10	16	8	28	53	28	SYCE1L
-2.24759	0.0013702	0.00565	down	7	4	2	14	31	22	CTAGE8
-2.55498	2.49E-06	2.02E-05	down	4	6	11	48	42	36	SEMA5A
-2.65778	5.21E-18	2.12E-16	down	21	25	22	144	172	134	
-1.7351	0.0027858	0.010349	down	8	5	9	25	34	18	
-5.28378	0.0001507	0.00081	down	0	0	1	17	13	13	
-3.83238	0.0044196	0.015328	down	1	0	1	6	18	7	
-1.84194	1.37E-13	3.44E-12	down	165	252	268	916	996	640	
-1.68109	8.80E-11	1.52E-09	down	59	51	52	137	228	187	FAXC
-2.74719	0.0003906	0.001875	down	3	2	3	16	26	15	CYP39A1
-1.89601	0.0046548	0.015998	down	9	6	5	9	42	31	KCNK3
-3.7681	3.83E-26	3.19E-24	down	31	43	73	328	1049	771	FAM213A
-3.29151	1.00E-11	1.96E-10	down	6	6	7	57	87	53	LDB3
-4.53713	4.28E-76	2.54E-73	down	39	42	68	1210	1416	962	KRT15
-1.57143	6.31E-18	2.54E-16	down	1029	1009	973	2790	4179	2561	KRT19

-3.75875	2.87E-05	0.000184	down	1	2	2	10	40	24	PLK5
-1.54447	1.69E-19	7.82E-18	down	218	230	239	626	789	686	C9orf156
-2.13236	3.05E-08	3.55E-07	down	13	17	16	64	90	59	
-5.75809	1.51E-30	1.71E-28	down	2	4	6	150	290	249	CTSV
-3.58435	0.0020774	0.008121	down	3	0	0	11	13	14	TSC22D1-AS1
-1.46171	0.0001577	0.000843	down	15	27	25	45	83	68	
-1.71328	0.000101	0.000568	down	21	19	12	35	78	71	
-5.66223	2.51E-05	0.000163	down	1	0	0	19	22	16	
-1.4735	1.06E-06	9.41E-06	down	39	45	45	82	169	132	ZNF653
-1.02325	1.24E-08	1.55E-07	down	704	656	662	1079	1895	1417	CXCL16
-2.1413	8.69E-08	9.41E-07	down	15	20	11	57	86	73	VIM-AS1
-4.65983	0.0015619	0.006334	down	0	1	0	7	13	9	KLHDC8B
-1.25413	0.0136224	0.039574	down	18	21	9	29	67	30	PSENEN
-1.8073	0.0034806	0.012533	down	3	13	23	64	32	38	FGF11
-1.27257	0.0007963	0.003515	down	17	21	24	44	60	53	TNFSF13
-6.47244	2.62E-06	2.12E-05	down	0	0	0	19	19	13	CALHM1
-1.69983	1.04E-08	1.31E-07	down	118	138	214	596	401	535	TRIQK
-3.99143	7.27E-15	2.17E-13	down	5	5	4	70	102	64	NAT1
-2.76743	1.09E-21	6.31E-20	down	27	23	38	164	228	233	MCC
-3.26624	1.73E-24	1.29E-22	down	23	23	15	151	265	212	
-9.23873	4.56E-149	########	down	3	9	16	5969	7001	4593	KRT13
-1.6691	3.16E-05	0.000201	down	45	26	48	180	105	95	TMEM170B
-1.39691	1.54E-13	3.81E-12	down	473	450	642	1040	1794	1512	PDE7A
-2.97159	8.78E-13	1.99E-11	down	27	24	66	426	303	185	
-1.41125	0.0023685	0.009037	down	21	21	35	98	75	35	CFH
-3.44301	5.69E-05	0.000342	down	0	3	3	13	34	23	CFI
-5.8222	0.0001155	0.000639	down	0	0	0	5	11	17	FGR
-1.97493	3.42E-05	0.000216	down	12	12	7	36	48	45	EXOC3L4
-7.96258	2.18E-10	3.56E-09	down	1	0	0	125	89	60	KRT6A
-1.1497	0.0012506	0.005238	down	31	39	31	51	90	97	EEPD1
-2.24792	0.0020252	0.007949	down	5	5	2	17	32	13	
-3.48522	7.52E-08	8.23E-07	down	5	4	8	99	72	22	
-1.78317	3.59E-06	2.82E-05	down	31	21	29	52	150	100	LOC101929719
-4.78188	0.0025789	0.009694	down	1	0	0	20	6	3	
-2.18327	4.48E-05	0.000275	down	6	8	8	25	51	31	
-1.30875	1.81E-13	4.46E-12	down	710	702	665	1364	2268	1851	ADGRG1
-1.1326	6.95E-11	1.22E-09	down	342	380	432	786	930	918	ETFDH
-3.23946	0.0019289	0.00762	down	1	1	2	9	24	8	LOC101928445
-2.35067	0.0044033	0.015283	down	4	2	2	14	17	12	
-1.02943	0.0003082	0.001527	down	78	69	53	120	190	127	
-1.86282	3.04E-09	4.19E-08	down	48	42	27	127	190	138	SLC25A42
-4.13163	0.0086465	0.027144	down	0	1	0	6	9	5	PODNL1
-1.26812	0.000835	0.003666	down	132	118	106	113	404	416	MAPK15
-1.61461	0.0075675	0.024192	down	5	8	16	16	31	45	

-1.75789	1.46E-12	3.22E-11	down	92	63	113	247	425	286	LOC100133091
-4.91779	0.0034858	0.01255	down	0	0	0	4	9	5	
-7.29435	3.99E-51	1.28E-48	down	1	5	5	592	656	547	TP63
-2.2895	7.59E-06	5.56E-05	down	11	9	9	34	87	34	LRRC24
-1.22756	0.0024946	0.009418	down	32	16	24	58	77	43	FAM161B
-1.86181	2.89E-17	1.09E-15	down	89	84	94	332	401	284	
-6.05777	1.93E-12	4.21E-11	down	1	0	3	130	85	53	
-5.33929	2.16E-75	1.21E-72	down	192	171	356	12070	9712	7522	MUC16
-1.25344	2.80E-13	6.74E-12	down	602	485	589	1294	1503	1371	DHTKD1
-3.19806	0.0010642	0.004537	down	0	4	1	13	14	21	CA9
-2.22138	2.01E-30	2.27E-28	down	456	542	474	1750	3129	2464	LLGL2
-1.11579	0.0076612	0.024454	down	31	24	25	30	83	74	ACVR2B-AS1
-1.99341	0.0001206	0.000665	down	9	7	16	27	74	36	SERF1B
-2.9358	1.25E-07	1.32E-06	down	4	11	5	49	82	34	
-2.54595	0.0034194	0.012335	down	3	5	0	13	17	20	LOC100507103
-3.42248	0.0044438	0.015397	down	2	0	1	16	12	5	LINC01204
-10.4915	5.26E-18	2.13E-16	down	0	0	0	303	329	198	HHLA1
-1.9277	0.0124532	0.036768	down	2	3	7	18	13	15	DENND1C
-1.31224	3.55E-09	4.86E-08	down	280	408	420	670	1207	1042	FRAT2
-1.50028	5.97E-12	1.21E-10	down	102	84	103	223	362	281	TMEM102
-4.5067	0.0028436	0.010541	down	0	0	1	10	7	8	EGR2
-1.10084	2.10E-09	2.96E-08	down	2495	2688	2791	4147	8017	6135	BAG1
-1.36526	1.38E-07	1.45E-06	down	229	257	166	412	778	624	SLC12A9
-1.83947	8.46E-05	0.000485	down	14	12	8	34	55	41	C17orf107
-2.0955	2.11E-17	8.09E-16	down	44	55	58	211	294	202	PLAU
-4.26456	6.06E-09	8.02E-08	down	6	1	1	25	91	54	EPHA1
-5.80014	1.98E-05	0.000132	down	0	0	1	17	36	12	PTGDS
-1.41301	5.32E-06	4.03E-05	down	51	32	62	124	127	146	FPGT
-3.163	0.009513	0.029412	down	1	1	1	15	7	5	ZNF540
-1.95875	3.63E-08	4.17E-07	down	21	26	46	126	162	88	AMY2B
-2.26437	2.51E-07	2.49E-06	down	11	7	16	49	71	51	COL28A1
-2.3951	0.0024257	0.009201	down	1	3	6	12	29	15	
-1.13745	2.24E-13	5.44E-12	down	849	765	815	1615	2286	1747	C22orf29
-4.08879	0.0131057	0.038343	down	0	0	1	9	8	2	
-5.46406	2.46E-06	2.01E-05	down	0	0	2	32	28	30	C4orf50
-5.41464	0.0004642	0.002183	down	0	0	0	7	11	7	
-2.9408	0.0044972	0.015543	down	2	3	6	67	7	4	CNTNAP5
-1.77729	6.42E-05	0.000379	down	18	13	17	63	76	34	CCDC183-AS1
-1.06017	6.75E-10	1.03E-08	down	420	415	390	780	1074	847	ZNF341
-5.86295	1.85E-28	1.81E-26	down	5	3	3	138	339	216	C10orf91
-2.59356	4.20E-07	4.03E-06	down	11	9	5	47	80	36	
-1.83173	7.37E-28	6.94E-26	down	2289	2224	2041	6631	####	7949	DDR1
-5.72397	0.000481	0.002252	down	0	0	0	19	8	2	HAPLN1
-2.29988	1.36E-23	9.41E-22	down	287	267	386	1800	1498	1419	COBL

-1.41175	3.35E-07	3.27E-06	down	216	215	175	323	836	599	ABHD11
-1.0537	1.73E-12	3.81E-11	down	1889	1758	1818	3370	4868	3769	PPP1R10
-3.3398	8.66E-17	3.10E-15	down	76	89	161	1721	964	576	SYNE1
-1.59668	1.55E-05	0.000106	down	45	48	66	73	237	207	CPVL
-1.31185	1.42E-08	1.75E-07	down	134	127	116	243	378	371	LFNG
-1.45969	9.42E-05	0.000533	down	22	22	18	50	68	62	MAPK8IP1
-1.28833	2.20E-05	0.000145	down	55	49	81	176	173	116	TSPAN12
-4.62877	0.0001342	0.000732	down	1	0	1	14	21	17	
-1.11997	0.000239	0.00122	down	73	67	60	112	241	119	PILRB
-1.00673	3.61E-05	0.000227	down	91	100	137	161	292	243	FAM103A1
-1.6693	1.88E-06	1.57E-05	down	18	29	44	86	130	87	LINC01125
-1.69847	2.70E-05	0.000175	down	65	78	24	176	266	148	ACYP2
-1.99241	0.0094544	0.029249	down	2	15	2	18	43	23	
-2.02664	3.09E-18	1.29E-16	down	58	51	62	216	296	221	AK9
-1.23626	9.72E-07	8.66E-06	down	462	469	321	708	1410	1078	LRRC45
-4.67883	0.0079186	0.025137	down	0	0	0	5	7	3	
-5.00053	0.0025638	0.009649	down	0	0	0	4	9	6	
-2.73267	0.0157364	0.044564	down	1	3	0	8	14	7	
-4.46246	0.0119899	0.035705	down	1	0	0	12	13	0	ACTA2-AS1
-3.2946	1.46E-05	0.0001	down	3	2	2	21	26	25	TMEM125
-2.94586	7.96E-20	3.77E-18	down	31	18	25	132	264	213	HLA-F
-1.74571	1.98E-06	1.65E-05	down	98	92	44	171	446	253	TH
-3.41971	0.0009297	0.004029	down	5	0	0	25	14	16	
-1.07359	6.81E-12	1.36E-10	down	690	801	876	1576	2049	1599	TTLL3
-1.364	0.0001008	0.000567	down	45	27	31	73	136	76	CASC10
-2.59383	3.96E-15	1.22E-13	down	52	65	64	475	439	218	CCRL2
-1.57996	5.28E-16	1.73E-14	down	4758	4113	4049	9633	####	####	SREBF1
-4.83578	2.24E-06	1.84E-05	down	0	0	3	21	47	23	TRIM10
-3.82011	4.12E-05	0.000255	down	3	0	2	14	49	15	TRIM15
-5.75371	6.42E-15	1.93E-13	down	2	1	2	107	95	76	TRIM31
-6.86674	2.39E-07	2.39E-06	down	0	0	0	22	23	22	RNF39
-2.25082	5.20E-16	1.71E-14	down	37	48	63	195	248	288	TNFSF10
-2.7536	4.93E-05	0.0003	down	11	1	4	44	45	24	SLC10A5
-4.68975	1.30E-38	2.26E-36	down	7	9	12	193	320	251	NRG4
-1.84739	1.18E-10	1.98E-09	down	42	34	66	173	198	157	KCNS3
-2.64519	6.58E-09	8.63E-08	down	18	6	10	51	96	80	GPR37
-4.25967	0.0065483	0.021413	down	0	0	1	7	11	4	TDRD6
-1.0038	1.81E-09	2.58E-08	down	641	670	776	1101	1782	1539	CHCHD7
-1.72647	2.41E-11	4.46E-10	down	99	139	176	312	663	484	
-4.46986	7.66E-13	1.75E-11	down	4	3	2	72	56	77	
-1.88972	0.015352	0.04371	down	5	2	5	16	23	8	WEE2-AS1
-1.65283	1.14E-18	4.87E-17	down	200	201	286	654	942	670	GOLGA2P5
-5.3799	6.68E-06	4.95E-05	down	1	1	0	26	52	13	GOLGA7B
-2.61168	1.14E-32	1.42E-30	down	124	109	124	810	776	672	RAB11FIP4

-4.19152	0.0102781	0.031368	down	0	1	0	2	14	6	SPDYC
-4.33421	0.0058176	0.019364	down	0	1	0	7	4	11	MAMDC2-AS1
-1.47891	7.16E-18	2.86E-16	down	911	757	1135	2153	3303	2748	DHFR
-3.14041	0.000389	0.001869	down	2	0	4	17	24	14	C1QTNF2
-3.14092	1.47E-09	2.12E-08	down	4	9	5	43	77	50	CYP3A5
-4.70124	0.0089998	0.028055	down	0	0	0	2	10	4	LOC105371083
-5.29656	0.0021333	0.008303	down	0	0	0	15	3	3	HYAL4
-1.12073	2.02E-07	2.05E-06	down	624	565	975	1649	1771	1415	PCDH7
-3.77735	0.0056549	0.018911	down	0	1	1	4	17	9	
-4.05299	0.0023253	0.008909	down	0	0	2	7	17	11	
-1.19494	0.0162924	0.04579	down	15	19	13	26	66	26	
-3.00603	0.0003935	0.001887	down	1	3	3	15	35	11	LINC01484
-1.93612	0.0137828	0.039997	down	2	3	7	8	25	16	CALML6
-4.51834	0.0031806	0.011603	down	0	0	1	6	16	5	
-1.75563	5.28E-13	1.23E-11	down	626	784	893	3132	2983	1907	UCA1
-2.49713	0.0157179	0.044538	down	1	2	2	10	15	5	ITGAM
-1.17539	0.0067235	0.021892	down	19	17	15	42	47	32	
-1.83926	0.011624	0.034751	down	5	3	7	14	34	10	
-1.9233	0.0037854	0.013462	down	4	8	7	26	40	11	C1orf101
-4.98128	0.002713	0.010122	down	0	0	0	7	6	5	
-1.10872	1.86E-05	0.000125	down	69	71	75	120	229	147	GLRX2
-1.53165	0.0014756	0.006037	down	16	10	11	26	54	35	
-1.46014	4.04E-09	5.47E-08	down	563	730	531	1151	2604	1708	MROH6
-1.90595	4.29E-07	4.11E-06	down	16	21	20	50	100	78	MRPL53
-3.73043	0.0001385	0.000752	down	3	2	0	36	25	8	
-2.98688	0.004221	0.014744	down	1	2	1	10	10	13	XK
-6.26883	1.13E-05	7.99E-05	down	0	0	0	13	25	8	
-3.98654	0.0004582	0.002157	down	3	0	0	21	20	9	TAS1R3
-2.36179	0.0039759	0.014014	down	2	3	5	27	12	12	PDGFD
-4.54592	0.0023901	0.009102	down	1	0	0	10	9	7	
-3.55807	9.70E-06	6.91E-05	down	3	1	2	22	34	19	PAXBP1-AS1
-4.15303	8.04E-05	0.000464	down	1	0	2	19	20	16	LINC00426
-2.74495	0.0106412	0.03228	down	1	1	2	8	11	9	
-1.63602	6.45E-06	4.80E-05	down	27	20	22	71	98	58	
-3.9109	0.0161951	0.045578	down	0	0	1	4	6	7	
-1.46365	1.39E-10	2.32E-09	down	220	130	202	467	655	480	C12orf4
-7.5855	1.79E-09	2.56E-08	down	1	0	0	90	82	42	SLC7A7
-1.16366	1.05E-05	7.44E-05	down	50	53	59	121	141	117	FBXO48
-1.53388	6.70E-12	1.34E-10	down	449	538	446	962	1972	1536	SPIRE2
-4.06886	0.0129122	0.037835	down	0	1	0	8	9	2	
-1.8647	0.0164202	0.046095	down	5	3	4	8	15	23	MBLAC1
-1.41583	1.55E-07	1.61E-06	down	516	446	1065	1725	2057	1743	SLC7A2
-1.36266	2.53E-08	3.01E-07	down	5741	4451	9665	17362	####	####	DSP
-4.06491	0.0127511	0.037453	down	0	0	1	8	3	7	VWA1

-9.56111	8.48E-35	1.14E-32	down	0	0	4	596	1359	1257	AGR2
-2.35794	1.41E-12	3.12E-11	down	27	24	34	176	156	116	TSPAN13
-6.02625	5.12E-06	3.89E-05	down	0	0	1	28	22	22	RARRES2
-2.12616	1.73E-05	0.000117	down	16	15	10	36	117	46	
-2.11582	0.0001148	0.000636	down	8	11	5	37	49	25	
-5.37458	3.95E-31	4.64E-29	down	4	4	6	187	256	168	TPRXL
-2.03643	3.52E-15	1.09E-13	down	564	548	393	1386	3136	2210	ACAP3
-2.71754	0.0170419	0.04758	down	1	0	3	9	11	7	LRGUK
-2.85603	0.000764	0.00339	down	5	1	1	15	22	17	
-1.09753	0.0002573	0.001303	down	92	105	69	127	271	218	KREMEN2
-5.45894	0.0003961	0.001897	down	0	0	0	6	12	8	ANO1
-3.63587	0.0038714	0.013727	down	1	2	2	52	5	1	PGR
-3.5849	0.0123758	0.036567	down	1	1	0	10	14	2	
-2.27099	6.20E-05	0.000368	down	8	3	13	47	36	34	
-2.84151	4.75E-05	0.000289	down	2	3	5	18	35	23	SENP3-EIF4A1
-1.51921	2.00E-12	4.34E-11	down	241	303	226	601	992	768	TMEM120A
-9.32026	1.18E-14	3.43E-13	down	0	0	1	169	304	258	CRMP1
-3.54644	3.60E-36	5.31E-34	down	26	22	29	222	420	316	ATP6V1B1
-1.68976	0.0081345	0.025749	down	8	3	11	31	24	17	PIK3AP1
-1.02474	0.0042169	0.014733	down	195	154	279	629	394	255	PARD3B
-1.3657	2.33E-13	5.63E-12	down	241	231	214	548	714	602	CORO2A
-6.5148	2.00E-44	4.42E-42	down	2	3	8	379	536	334	SLC47A2
-6.61199	8.39E-38	1.37E-35	down	3	5	2	342	353	322	SYK
-1.53652	2.37E-07	2.37E-06	down	85	72	97	285	327	158	ARL14
-1.48019	0.0023506	0.008987	down	45	23	11	77	105	56	KIAA1875
-1.24028	2.57E-07	2.55E-06	down	112	103	126	185	374	300	NMRK1
-5.71745	0.0001329	0.000725	down	0	0	0	6	13	12	APOD
-2.4868	0.0127563	0.037453	down	0	0	12	21	30	16	ALDH1A1
-4.16071	4.19E-24	3.03E-22	down	6	9	12	153	241	118	
-4.49985	0.003511	0.012619	down	1	0	0	12	9	4	
-1.19339	3.79E-12	7.89E-11	down	716	771	812	1367	2421	1815	
-1.42089	0.0054156	0.018217	down	10	8	16	32	28	33	
-1.24949	8.42E-12	1.67E-10	down	236	222	258	511	779	513	CLUHP3
-4.50646	0.0126601	0.03724	down	0	0	0	4	4	5	
-1.95326	3.88E-06	3.02E-05	down	12	22	14	64	59	70	PIPOX
-1.36192	8.35E-10	1.26E-08	down	456	415	429	1303	1120	1023	ANKS6
-2.59557	0.0145751	0.041874	down	3	1	1	16	10	5	FBP1
-1.50876	7.68E-08	8.39E-07	down	403	381	694	1730	1232	1256	ZDHHC20
-7.30307	5.71E-08	6.37E-07	down	0	0	0	42	17	28	LOC100506457
-4.93586	0.008203	0.025931	down	0	0	0	3	1	13	
-6.01859	0.0003266	0.001605	down	0	1	2	26	6	158	
-2.77304	0.0009952	0.004283	down	2	3	2	12	28	12	CDIPT-AS1
-1.7886	4.50E-21	2.48E-19	down	358	326	296	1040	1499	1060	B3GNT3
-2.18812	8.09E-09	1.05E-07	down	24	30	16	101	163	80	KIAA1324

-1.11676	0.0004774	0.002238	down	33	50	74	113	121	116	OXTR
-4.36359	0.0047512	0.016284	down	0	0	1	4	12	8	CLIC2
-2.19047	0.0001135	0.00063	down	9	3	13	40	54	25	AKAP5
-2.19643	2.65E-07	2.63E-06	down	10	10	27	62	95	67	KCNK2
-1.72201	0.0095552	0.029499	down	6	9	4	11	36	22	
-2.57927	2.80E-07	2.76E-06	down	5	12	26	47	113	109	RAET1L
-6.67905	5.79E-06	4.34E-05	down	0	0	0	36	14	6	ABHD12B
-1.55323	0.0002594	0.001312	down	26	35	94	185	141	126	CAPS2
-1.94317	1.93E-06	1.61E-05	down	35	18	23	63	173	84	
-4.72159	0.0151501	0.043213	down	0	0	0	11	2	1	TNFSF8
-2.60768	0.0024189	0.009189	down	2	3	2	8	21	17	
-2.62126	1.10E-46	2.68E-44	down	811	1022	901	4487	7485	5957	AQP3
-1.21932	8.80E-07	7.94E-06	down	76	87	92	145	253	231	
-1.12887	0.0029611	0.010903	down	69	47	43	61	194	127	C15orf61
-1.16508	2.84E-06	2.29E-05	down	75	60	74	132	214	151	LOC100129550
-2.28584	8.66E-05	0.000496	down	4	19	5	36	65	47	
-2.24219	6.81E-10	1.04E-08	down	17	15	18	71	87	89	C2orf15
-1.84877	0.0062221	0.020473	down	6	7	6	33	28	10	LINC01057
-2.78753	0.0031471	0.0115	down	4	0	2	9	18	17	
-2.72794	2.01E-29	2.13E-27	down	51	60	56	319	536	326	GJB5
-5.2035	0.0003519	0.001711	down	1	0	0	5	14	23	FHIT
-2.00016	0.0035553	0.012756	down	3	9	3	17	25	22	
-2.65625	0.0064189	0.021044	down	2	2	2	4	25	13	
-4.48295	3.91E-08	4.46E-07	down	0	2	3	28	38	50	
-4.64219	0.0092531	0.028706	down	0	0	0	5	3	6	
-3.72869	0.0065424	0.021401	down	1	0	1	10	14	4	
-3.68615	3.00E-05	0.000192	down	1	3	1	17	37	16	
-4.17636	1.30E-09	1.91E-08	down	4	1	3	27	75	54	EVPLL
-1.7041	3.15E-09	4.33E-08	down	111	111	218	536	443	467	SLITRK5
-1.39898	2.54E-11	4.70E-10	down	2302	1791	1598	4285	6754	5021	FAM83H
-1.11172	9.32E-05	0.000528	down	70	66	72	179	162	124	HECTD2
-4.55149	1.59E-26	1.36E-24	down	4	7	8	138	165	161	DDX26B
-6.88206	2.45E-189	########	down	57	50	62	4571	8819	7823	S100A14
-2.37154	0.0071422	0.023054	down	4	5	1	27	7	18	
-2.61644	4.56E-06	3.50E-05	down	7	5	6	31	62	26	
-2.0157	0.0033076	0.011997	down	4	15	3	16	39	42	ALG1L
-1.24861	7.73E-15	2.29E-13	down	594	570	680	1331	1972	1327	ATP6V0A1
-2.10217	0.006832	0.022207	down	2	3	8	24	11	20	
-1.22784	2.73E-06	2.20E-05	down	53	51	73	119	188	130	
-2.00927	9.62E-11	1.65E-09	down	25	48	40	122	185	173	BARX2
-2.71415	0.0066933	0.021812	down	4	1	1	20	16	5	ISPD
-3.81325	0.0061127	0.020155	down	1	1	0	3	21	8	
-2.68125	5.58E-08	6.25E-07	down	7	7	10	33	79	53	ZMYND15
-2.9572	0.0015914	0.006434	down	5	2	7	83	9	9	ZNF750

-1.93551	4.00E-05	0.000249	down	13	17	8	48	71	37	SLC7A8
-1.11098	2.06E-09	2.91E-08	down	899	843	761	1488	2243	2010	TBCD
-4.6057	4.69E-20	2.27E-18	down	5	7	2	93	171	103	GJB4
-1.35409	1.95E-16	6.76E-15	down	547	541	736	1507	1828	1514	SKA3
-1.77378	9.17E-19	4.00E-17	down	293	308	421	1243	1232	1119	MICU2
-1.53083	0.0005466	0.002523	down	13	16	43	53	99	65	HLA-F-AS1
-2.01085	0.0012539	0.005248	down	10	3	14	15	60	42	RHOU
-2.00249	0.0141725	0.040896	down	1	3	7	14	22	10	
-1.49325	1.03E-05	7.29E-05	down	32	31	55	74	135	139	
-1.99828	0.002941	0.010849	down	5	6	3	19	22	18	ABHD14A
-2.79401	5.12E-05	0.00031	down	8	4	4	11	59	52	
-4.61312	0.0003386	0.001656	down	0	1	1	5	31	18	
-7.24981	2.64E-08	3.12E-07	down	0	0	1	84	58	25	DRGX
-3.52913	0.0008778	0.003826	down	0	0	4	12	17	18	
-1.75671	1.48E-06	1.27E-05	down	44	51	32	86	248	139	ADM5
-2.19225	1.46E-08	1.80E-07	down	17	13	15	63	95	60	PRKAR2A-AS1
-1.67915	8.95E-07	8.05E-06	down	149	125	73	231	616	381	COMTD1
-4.30425	3.86E-19	1.73E-17	down	4	10	3	92	128	135	FAAH2
-2.18581	7.06E-06	5.20E-05	down	9	8	10	43	54	32	SMARCA1
-3.01796	7.60E-05	0.000441	down	2	4	2	14	33	23	SYP
-1.63534	2.19E-09	3.08E-08	down	73	65	123	294	304	234	SWT1
-1.48734	7.99E-12	1.59E-10	down	237	242	397	660	973	923	C1orf21
-2.27029	9.53E-27	8.38E-25	down	165	161	137	690	1032	660	FBXO2
-1.32896	2.13E-08	2.56E-07	down	91	104	107	184	332	290	
-4.2079	0.0011909	0.005015	down	0	2	0	7	17	16	
-3.28491	0.0051487	0.017461	down	1	0	2	9	10	11	
-1.18599	0.0023574	0.009009	down	17	22	24	41	68	43	
-1.1884	1.37E-06	1.19E-05	down	131	112	119	194	348	335	NCF2
-4.97229	1.97E-10	3.23E-09	down	1	3	1	63	64	37	
-1.36781	3.66E-13	8.70E-12	down	3743	4306	3648	7910	####	####	
-2.83717	0.000113	0.000628	down	7	12	16	181	31	24	CACNA1C
-1.68471	0.0012833	0.005347	down	14	9	10	19	49	46	STOX1
-2.0697	0.0001195	0.00066	down	16	10	4	31	56	49	SRP14-AS1
-3.9349	0.0156008	0.044272	down	0	0	1	7	6	4	ENKUR
-1.02022	7.39E-09	9.61E-08	down	610	656	612	1020	1688	1349	FUNDC2
-1.50905	2.90E-11	5.31E-10	down	131	110	127	374	359	351	ENOX2
-1.04776	1.07E-06	9.48E-06	down	124	109	139	247	337	225	TGM1
-2.51053	0.0001195	0.00066	down	9	3	3	21	47	25	SLC22A1
-3.07215	0.0045458	0.015671	down	3	1	0	12	9	14	
-1.11524	4.57E-10	7.12E-09	down	341	351	463	829	1024	756	PHTF1
-2.2341	5.74E-12	1.16E-10	down	329	356	394	2501	1590	1042	CTH
-4.23391	0.0128564	0.037683	down	0	0	1	7	1	12	CFHR3
-1.80872	6.35E-07	5.89E-06	down	25	23	50	83	124	148	PLA2G4A
-1.84108	0.007998	0.025359	down	1	6	12	18	26	26	

-1.14718	4.64E-05	0.000284	down	60	43	50	96	148	115	LOC105369779
-4.45113	0.016794	0.046996	down	0	0	0	4	7	2	
-1.07326	5.71E-14	1.53E-12	down	2E+05	####	####	3E+05	####	####	
-1.70543	3.47E-12	7.26E-11	down	377	307	252	745	1472	1084	ZNF219
-4.38571	0.0039542	0.013958	down	0	1	0	6	11	7	
-1.4742	9.09E-06	6.51E-05	down	32	25	47	72	140	94	
-1.17887	0.0003114	0.001539	down	64	94	64	104	272	173	
-4.27081	0.0009428	0.004079	down	1	1	0	6	25	12	F10
-2.34657	0.0021368	0.008313	down	5	1	4	15	19	19	
-5.2931	0.001626	0.006559	down	0	0	0	6	16	2	
-1.28961	0.0041219	0.014451	down	15	16	15	26	58	37	GYLTL1B
-2.37372	6.30E-06	4.69E-05	down	6	7	12	42	67	28	RAPSN
-2.9722	7.61E-19	3.34E-17	down	31	32	69	396	294	349	TC2N
-1.81194	2.57E-13	6.20E-12	down	129	79	125	352	555	332	
-2.30229	0.0002084	0.001079	down	4	10	4	21	50	26	
-1.33681	0.005482	0.018414	down	16	18	14	23	72	38	
-3.72107	0.0060568	0.020019	down	0	1	1	9	14	5	
-1.70636	0.0032312	0.011769	down	9	11	5	17	43	29	
-2.83204	1.14E-05	8.01E-05	down	3	4	5	34	30	24	
-1.21847	0.000947	0.004095	down	33	38	21	75	83	68	
-2.38265	9.77E-06	6.96E-05	down	6	10	7	41	61	26	
-1.09278	0.0002052	0.001065	down	45	45	49	72	132	111	
-2.91437	3.02E-10	4.84E-09	down	13	4	9	61	82	63	
-1.06999	0.0080648	0.02555	down	41	51	29	45	136	98	TPGS1
-1.65491	0.0014192	0.005831	down	9	11	9	20	44	34	
-1.7749	2.47E-07	2.46E-06	down	46	40	36	91	242	124	PCSK1
-3.875	0.0038128	0.013543	down	0	1	1	8	7	15	FLJ46284
-5.53014	0.0003423	0.001671	down	0	0	0	9	6	11	LPL
-1.65122	6.64E-07	6.14E-06	down	32	19	38	90	112	89	RFESD
-2.31336	6.46E-23	4.25E-21	down	58	63	90	336	374	374	C4orf33
-1.01116	2.05E-07	2.08E-06	down	910	1049	873	1484	2494	2116	IRF3
-4.2063	8.75E-21	4.62E-19	down	3	5	12	101	144	139	PRRG2
-1.66779	1.46E-19	6.78E-18	down	201	196	203	515	832	676	PRKCZ
-1.22752	4.76E-08	5.36E-07	down	105	89	117	241	312	211	
-4.84911	0.0007823	0.003462	down	0	1	0	9	15	9	LOC105369635
-2.04393	1.73E-16	6.08E-15	down	228	293	234	750	1685	953	PTK6
-1.11739	3.48E-10	5.51E-09	down	299	256	326	521	808	687	ORC5
-6.68608	6.77E-07	6.25E-06	down	0	0	0	18	25	17	DEFB1
-3.69355	0.0086977	0.027273	down	1	1	0	7	19	3	
-4.13147	4.97E-24	3.54E-22	down	6	15	8	129	265	155	GPER1
-2.36834	2.78E-19	1.26E-17	down	234	198	146	783	1562	904	TMEM184A
-2.15623	2.59E-12	5.54E-11	down	66	80	51	188	456	314	
-1.06529	0.0016189	0.006533	down	29	32	59	71	96	93	LINC00910
-1.37549	0.0022998	0.008828	down	16	16	11	30	49	40	

-2.3806	0.0009381	0.004063	down	5	5	4	8	40	32	ENTPD8
-1.3785	2.10E-05	0.000139	down	38	26	48	72	135	101	
-1.58655	0.0055867	0.018716	down	4	12	14	20	33	41	
-3.98204	2.70E-12	5.77E-11	down	2	2	11	90	71	77	ZNF595
-2.34406	0.0146941	0.042171	down	1	2	3	12	9	10	LINC00571
-1.47651	0.0176887	0.04914	down	4	8	9	21	25	15	
-3.75222	3.66E-13	8.69E-12	down	4	4	8	74	99	53	PCDH15
-1.64434	1.02E-17	4.03E-16	down	195	219	294	614	987	731	LPAR1
-1.62684	8.08E-05	0.000466	down	20	25	12	56	78	54	MB
-4.71882	4.56E-45	1.04E-42	down	14	12	23	288	524	545	HMGN5
-1.01489	2.02E-13	4.97E-12	down	4564	4411	5018	8912	####	9734	TFDP1
-3.07491	0.0013321	0.005517	down	1	2	2	8	26	12	NUDT7
-1.02399	7.14E-08	7.85E-07	down	512	465	538	1120	1172	910	CDS2
-6.7395	2.93E-14	8.18E-13	down	1	1	1	92	142	105	MARVELD3
-2.07566	6.81E-05	0.0004	down	11	4	14	34	41	51	NKD1
-1.56947	2.24E-05	0.000148	down	35	34	34	128	128	63	MYL9
-2.28794	0.0019674	0.007763	down	1	5	10	30	39	12	TP53INP1
-2.32179	0.0041512	0.01453	down	1	8	2	12	22	25	
-1.70204	7.06E-12	1.41E-10	down	369	368	262	962	1651	903	GJB3
-1.27309	0.0002092	0.001083	down	22	32	31	57	82	77	LOC102723582
-2.24367	0.000297	0.001479	down	6	13	2	26	46	36	LOC257396
-2.70573	2.62E-52	8.89E-50	down	136	157	187	994	1258	1020	KIAA1161
-1.98171	0.010107	0.030937	down	5	1	6	14	24	12	C9orf24
-1.52461	5.12E-07	4.82E-06	down	37	34	46	84	167	108	
-1.78494	5.72E-08	6.39E-07	down	56	67	37	139	295	168	
-2.13982	9.19E-10	1.37E-08	down	32	25	17	90	152	108	SLC16A13
-5.43029	0.000125	0.000687	down	0	0	1	22	19	7	C9orf152
-1.14461	0.0040478	0.01423	down	19	46	37	54	119	70	
-1.09611	0.0056656	0.018941	down	66	61	105	65	245	222	CHRNA9
-1.23702	2.20E-12	4.76E-11	down	526	609	616	1089	1740	1540	
-1.44396	4.44E-07	4.24E-06	down	116	80	78	170	352	281	TNK1
-1.52751	2.45E-08	2.91E-07	down	48	47	43	116	181	126	GRK4
-1.13539	0.0061466	0.02026	down	41	15	45	79	69	78	STX18-AS1
-1.15778	3.65E-11	6.61E-10	down	256	255	343	563	819	619	ATP5S
-6.85154	5.14E-07	4.83E-06	down	0	0	0	11	39	20	TLDC2
-2.40883	9.60E-10	1.43E-08	down	13	11	23	61	101	99	PELI2
-4.10072	0.0112097	0.033709	down	1	0	0	9	7	3	LINC01619
-1.86147	0.0031493	0.011504	down	6	3	9	17	25	26	
-1.72161	0.0083596	0.026369	down	5	5	8	12	34	18	
-4.90391	0.0039358	0.013912	down	0	0	0	4	10	4	
-1.51379	6.83E-06	5.05E-05	down	32	36	32	64	146	98	
-5.08808	0.0022238	0.008598	down	0	0	0	7	10	3	
-1.6265	0.0024226	0.009197	down	8	6	12	23	34	27	
-1.38872	0.00039	0.001873	down	41	50	31	65	197	92	

-4.1019	0.0001276	0.000699	down	1	0	2	11	21	22	LINGO2
-1.79662	2.07E-10	3.38E-09	down	46	46	36	150	177	141	OSER1-AS1
-1.1389	0.0022141	0.008567	down	30	29	22	45	88	59	
-1.13514	2.48E-11	4.59E-10	down	521	433	551	964	1497	1038	WWP2
-2.14519	7.69E-20	3.65E-18	down	113	112	110	341	683	563	N6AMT2
-1.30685	3.87E-13	9.18E-12	down	759	855	777	1568	2700	2051	SAP18
-1.56102	8.19E-06	5.94E-05	down	27	20	44	69	109	102	
-1.13053	0.007036	0.022753	down	26	18	20	30	71	50	CFL1P1
-5.58738	5.51E-126	########	down	60	40	36	1951	2797	2191	ST14
-2.61392	0.0003099	0.001534	down	2	5	3	23	24	17	
-6.05861	1.13E-05	7.94E-05	down	1	0	0	7	42	30	LOC200772
-1.64027	0.00016	0.000854	down	26	32	12	55	116	68	
-1.14457	0.0014811	0.006057	down	52	34	81	76	197	121	
-1.06815	0.0112253	0.03374	down	40	59	75	187	101	76	ITGBL1
-1.02592	6.25E-08	6.92E-07	down	632	697	1018	1483	1790	1670	GTF2IP4
-1.29546	4.42E-05	0.000272	down	30	50	40	98	123	89	
-2.87967	0.0081944	0.025913	down	2	0	2	7	13	11	
-1.55637	1.88E-12	4.10E-11	down	105	90	138	317	418	289	LOC283335
-1.64761	2.62E-15	8.23E-14	down	149	121	143	448	494	407	ZNF480
-2.35289	5.32E-06	4.03E-05	down	11	6	20	87	51	50	ZNF808
-2.11706	0.0022163	0.008572	down	6	6	3	11	27	32	
-3.05827	0.0113043	0.033934	down	0	2	1	9	9	8	LOC100506286
-5.11541	1.86E-09	2.64E-08	down	1	0	3	46	44	52	SH3BGRL2
-3.62964	1.05E-23	7.32E-22	down	11	17	20	126	303	209	LINC00926
-1.66736	1.64E-07	1.69E-06	down	36	27	37	87	160	92	FEZ1
-2.31139	0.000201	0.001046	down	6	5	6	25	49	17	PLCH2
-2.57437	5.48E-39	9.74E-37	down	637	524	492	3244	4217	2962	RHOBTB2
-1.31162	6.16E-05	0.000366	down	58	32	68	147	131	123	RYR2
-3.00305	0.0139756	0.040452	down	2	0	1	7	8	10	
-2.07974	0.0026372	0.009881	down	5	2	9	18	39	15	
-2.56577	5.72E-30	6.29E-28	down	66	59	85	322	540	450	MAGEA1
-1.16802	0.0013034	0.005416	down	29	23	42	47	101	76	DIABLO
-1.63844	2.27E-08	2.72E-07	down	157	149	305	753	608	554	SYNJ1
-1.0602	2.96E-05	0.000189	down	79	71	69	124	211	152	C21orf59
-7.49328	2.62E-07	2.59E-06	down	7	0	1	986	268	168	TG
-2.59844	1.78E-39	3.22E-37	down	156	177	152	885	1347	896	
-1.2696	0.000426	0.002026	down	32	65	50	80	196	109	CNBD2
-4.9277	0.0060906	0.020107	down	0	0	0	7	10	1	KIAA1755
-1.79867	0.0111405	0.033527	down	4	8	3	14	17	24	SPATA25
-1.32588	1.82E-16	6.39E-15	down	298	309	351	704	1034	795	PANK2
-1.66417	6.15E-05	0.000366	down	28	30	15	48	114	90	SDCBP2
-2.06335	1.48E-11	2.83E-10	down	35	40	61	221	207	155	FOSB
-5.41276	0.0005112	0.002379	down	0	0	0	5	10	10	FMO4
-1.32457	8.48E-10	1.27E-08	down	1184	1375	942	2528	3914	2954	CD70

-1.84063	2.04E-20	1.02E-18	down	284	302	279	854	1521	949	SH2D3A
-1.06338	1.66E-08	2.03E-07	down	686	673	599	1090	1819	1461	ANKRD13B
-1.8593	2.68E-16	9.13E-15	down	19796	####	####	45113	####	####	CYTB
-4.39205	6.39E-11	1.13E-09	down	6	3	0	42	113	54	MAPK8IP2
-1.13463	1.17E-05	8.25E-05	down	77	86	93	131	276	195	SMOC1
-4.4587	0.004499	0.015546	down	0	0	1	3	8	14	PCP2
-2.84975	4.69E-15	1.43E-13	down	24	9	21	103	176	133	PPP1R14C
-1.02945	5.39E-09	7.19E-08	down	46541	####	####	72215	####	####	ND2
-2.11218	1.16E-10	1.95E-09	down	35	30	33	91	223	144	EPS8L3
-2.3471	0.0031004	0.011356	down	3	5	3	22	31	7	CILP2
-4.8074	8.09E-16	2.61E-14	down	1	6	4	57	181	99	MUC2
-1.54324	2.66E-06	2.15E-05	down	59	45	105	235	188	191	RASSF9
-4.34198	1.28E-08	1.59E-07	down	0	4	2	32	65	34	TFF1
-3.21813	0.0036648	0.013068	down	2	1	1	21	13	4	TFF2
-1.71234	1.35E-21	7.80E-20	down	466	477	457	1563	1892	1368	TMPRSS3
-2.16397	0.0001366	0.000743	down	8	11	5	33	61	23	SCAMP5
-2.07225	6.44E-18	2.58E-16	down	140	109	104	384	728	486	ZNF362
-3.35935	2.03E-06	1.69E-05	down	3	3	2	23	35	29	
-1.20169	3.57E-10	5.65E-09	down	284	330	390	671	1092	685	
-1.56461	0.0001765	0.00093	down	10	20	27	49	64	62	TRPV4
-1.78952	0.0041107	0.014425	down	12	4	8	16	52	23	PRSS8
-1.43984	0.0001511	0.000812	down	26	35	19	80	81	67	PIH1D2
-2.72509	6.69E-28	6.33E-26	down	91	88	152	531	1082	710	CCDC64
-2.11375	5.54E-38	9.15E-36	down	299	258	304	1161	1572	1189	PLXNA2
-5.848	6.29E-05	0.000372	down	0	0	0	11	11	11	TGM3
-3.62063	0.0095609	0.029512	down	1	0	1	5	17	5	GNRH2
-1.37078	9.78E-09	1.24E-07	down	104	95	90	197	360	244	RNFT2
-1.49879	2.42E-05	0.000159	down	21	20	38	68	84	79	PARP11
-2.58219	4.85E-05	0.000295	down	1	6	11	43	36	30	PDZK1
-1.34822	0.0143294	0.041281	down	10	7	15	33	33	18	BFSP1
-1.03732	6.91E-05	0.000405	down	422	439	337	525	1272	884	ITPA
-1.06355	3.86E-06	3.00E-05	down	157	191	166	265	520	369	ZNF133
-1.99952	1.86E-13	4.59E-12	down	99	76	95	280	597	290	BMP2
-4.03926	0.0006065	0.002767	down	3	0	0	28	13	9	FLRT3
-1.392	1.46E-09	2.11E-08	down	141	127	105	285	436	323	AP5S1
-1.7441	0.0076905	0.024536	down	6	4	6	19	22	15	
-5.50566	1.51E-20	7.63E-19	down	0	4	4	101	156	126	OVOL2
-2.19176	4.22E-09	5.71E-08	down	24	41	24	112	229	102	CNIH2
-1.54551	6.05E-12	1.22E-10	down	3443	4163	3099	7419	####	####	ND3
-8.22528	1.91E-11	3.60E-10	down	0	0	1	103	156	83	DUSP27
-5.51292	4.62E-16	1.53E-14	down	3	2	2	51	159	136	CES1
-1.46878	2.65E-09	3.68E-08	down	119	81	94	262	290	297	LRRC3
-1.17247	0.0006677	0.00301	down	63	88	54	93	254	160	NDUFA11
-1.33885	3.96E-12	8.23E-11	down	611	552	637	1105	2191	1580	ITFG2

-1.64269	0.0031897	0.011633	down	20	17	8	19	84	54	GJC2
-4.65202	0.0090793	0.028246	down	0	0	0	6	3	5	
-1.04092	0.000118	0.000652	down	963	1160	673	1383	2889	2005	SNRPB
-2.44791	2.90E-07	2.85E-06	down	13	6	23	96	81	55	PCDH9
-1.14286	2.40E-06	1.96E-05	down	912	817	564	1406	2330	1720	CENPB
-3.79406	2.28E-10	3.70E-09	down	9	1	4	49	112	50	
-4.5918	0.0101695	0.031098	down	0	0	0	5	6	3	
-1.01044	1.22E-05	8.51E-05	down	69181	####	####	96712	####	####	ND1
-2.56142	1.88E-08	2.28E-07	down	23	18	20	172	144	56	SFMBT2
-1.77904	2.19E-11	4.06E-10	down	382	402	697	2033	1685	1422	STS
-1.22031	5.62E-11	9.97E-10	down	18948	####	####	34855	####	####	ATP6
-1.50439	2.57E-07	2.55E-06	down	73	68	57	160	298	149	KCNJ12
-5.92055	5.98E-25	4.62E-23	down	4	2	3	132	303	155	
-2.35925	0.0158385	0.044793	down	1	2	3	5	16	12	
-6.20058	3.25E-14	9.01E-13	down	2	1	1	57	163	100	KCP
-3.52273	1.34E-09	1.96E-08	down	9	0	10	84	62	74	LCA5
-2.45737	0.0161463	0.045467	down	5	6	1	51	11	3	EPHA7
-1.08165	9.97E-11	1.70E-09	down	1876	1510	1878	3320	5084	3403	L1CAM
-1.25737	5.58E-08	6.25E-07	down	122	97	169	299	341	318	CEP162
-2.25262	6.81E-05	0.0004	down	4	5	15	36	36	44	BHLHB9
-2.28186	2.26E-48	6.50E-46	down	650	742	716	3009	4185	3615	SCNN1A
-1.39618	7.08E-09	9.26E-08	down	65734	####	####	1E+05	####	####	COX3
-1.25113	4.49E-07	4.28E-06	down	80	64	79	154	196	205	ARMCX5
-5.79591	0.0005167	0.002399	down	0	0	0	19	11	1	CCSER1
-1.99948	0.017591	0.048912	down	2	3	4	7	15	16	GPRASP1
-1.1302	0.003061	0.011222	down	24	22	24	47	75	41	DOC2A
-1.19269	3.92E-10	6.17E-09	down	369	284	447	763	1096	779	FOXO1
-1.80537	1.38E-13	3.46E-12	down	243	280	196	640	1269	816	ABCD1
-1.33401	0.0023022	0.008832	down	16	19	16	27	61	50	
-4.67894	0.0023892	0.009102	down	1	0	0	9	18	3	MMP25
-1.64856	0.000189	0.00099	down	16	12	21	32	83	50	OAS2
-1.60296	1.74E-14	4.99E-13	down	214	239	361	738	918	902	ZDHHC23
-1.70742	2.71E-28	2.62E-26	down	453	456	487	1430	1934	1439	NAGA
-1.841	0.001103	0.004677	down	6	17	7	44	43	26	MFAP3L
-7.52892	6.19E-09	8.18E-08	down	0	0	0	20	52	38	RNF157-AS1
-1.5137	0.0004991	0.002327	down	11	17	22	32	64	55	
-2.10983	4.57E-17	1.69E-15	down	241	252	206	659	1566	1058	PLA2G6
-1.99409	2.94E-05	0.000188	down	16	11	13	68	66	32	ACTRT3
-1.04083	5.71E-06	4.30E-05	down	121	143	126	244	379	232	TACSTD2
-7.35149	2.59E-08	3.06E-07	down	0	0	0	29	51	17	LOC101927292
-1.33023	1.75E-06	1.48E-05	down	49	60	75	115	229	149	CNPY2
-2.37013	1.46E-06	1.26E-05	down	13	5	19	79	71	45	
-4.40704	0.0037437	0.013326	down	0	0	1	8	10	6	
-1.06547	1.42E-08	1.76E-07	down	222	212	209	381	587	460	CYB5R1

-2.9048	2.49E-19	1.14E-17	down	21	28	39	256	206	209	PRRG4
-2.55886	5.90E-05	0.000353	down	6	1	10	30	50	25	EHF
-1.80538	0.0063899	0.02096	down	4	9	6	12	41	20	BLOC1S1
-5.14746	0.0015427	0.006264	down	0	0	0	7	5	8	GLS2
-1.11894	2.36E-08	2.82E-07	down	154	145	144	304	375	331	TBC1D30
-1.76656	9.57E-07	8.53E-06	down	62	110	50	161	405	265	LINC01503
-2.45015	2.61E-05	0.000169	down	5	10	4	36	50	25	AVIL
-1.176	0.0001083	0.000605	down	93	72	118	124	281	273	FZD10
-2.18101	7.45E-08	8.16E-07	down	12	21	21	68	134	61	
-1.08142	0.0005094	0.002372	down	103	93	67	125	298	185	LOC105371795
-1.0406	0.009295	0.028814	down	36	33	26	36	103	74	
-1.12553	6.52E-08	7.20E-07	down	324	358	323	642	766	886	FOXO4
-4.05415	0.0156956	0.044488	down	1	0	0	4	14	2	FOXD4L1
-4.37997	8.38E-06	6.06E-05	down	4	0	0	16	55	22	STON1
-2.49359	2.51E-05	0.000164	down	9	7	3	29	61	27	
-2.00351	5.98E-37	9.17E-35	down	439	367	479	1586	2074	1735	CDH3
-1.80688	0.0003606	0.001749	down	22	22	57	190	88	66	PAG1
-3.96471	0.0143885	0.04142	down	0	0	1	5	9	4	
-1.26384	6.54E-06	4.85E-05	down	65	66	52	113	210	149	MPND
-3.55738	0.0126243	0.037163	down	0	0	2	9	10	5	FAIM2
-4.45784	0.003827	0.01359	down	1	0	0	4	15	7	GAST
-4.6208	2.94E-11	5.40E-10	down	3	1	3	68	47	60	PLEKHG6
-4.70824	4.36E-11	7.83E-10	down	5	0	2	36	102	61	PKIB
-4.46512	8.58E-08	9.31E-07	down	1	0	4	34	31	47	SLITRK6
-1.66384	6.64E-05	0.000391	down	14	12	25	54	69	45	PDE4B
-1.22413	0.006884	0.022349	down	21	16	17	24	56	55	
-1.92904	0.0027599	0.010267	down	9	4	5	13	34	27	
-6.02991	5.02E-06	3.83E-05	down	1	0	0	22	36	17	
-2.67607	0.0104238	0.031737	down	0	4	1	9	13	12	
-1.22024	0.0005149	0.002394	down	45	56	32	90	163	83	CCDC17
-2.87369	9.86E-08	1.06E-06	down	7	4	12	74	46	49	MBNL3
-3.18231	0.0002054	0.001066	down	1	1	4	15	25	17	NELL2
-1.40347	1.78E-05	0.000121	down	97	125	63	170	335	311	PTPN6
-2.42912	0.0041485	0.014523	down	0	3	6	13	19	18	MFSD7
-3.23337	6.45E-09	8.49E-08	down	1	7	9	41	76	52	COL4A3
-1.54141	3.89E-10	6.12E-09	down	99	92	82	251	374	222	CCDC142
-1.99395	1.32E-07	1.39E-06	down	36	28	31	89	235	91	KCNAB3
-1.14485	6.34E-09	8.37E-08	down	749	624	900	1829	1827	1524	MECP2
-1.23982	7.94E-19	3.48E-17	down	1592	1550	1933	3475	4996	4123	UPF3A
-1.65619	1.05E-10	1.78E-09	down	102	103	75	230	373	339	PRADC1
-4.65275	5.71E-06	4.30E-05	down	1	2	0	13	37	32	ROR2
-2.60924	0.0017193	0.0069	down	7	0	3	17	35	14	
-2.76364	0.0039136	0.013856	down	2	4	0	8	22	15	
-1.81703	4.56E-06	3.51E-05	down	30	14	18	73	96	62	CAMSAP3

-1.54466	1.01E-15	3.22E-14	down	312	291	288	914	988	813	MAN2B2
-1.20427	0.0047983	0.016417	down	16	29	17	35	64	54	EAF2
-1.75772	6.69E-13	1.55E-11	down	75	73	77	190	372	253	NEK8
-2.62623	1.40E-13	3.49E-12	down	29	33	47	116	368	244	SPON2
-1.04451	1.91E-08	2.31E-07	down	373	428	490	679	1163	977	SCPEP1
-1.66516	1.77E-16	6.24E-15	down	191	172	203	449	763	689	SNN
-1.05479	2.20E-07	2.21E-06	down	179	164	170	282	477	375	TCTA
-1.03637	8.89E-08	9.62E-07	down	466	409	590	1055	1065	975	CSNK1G1
-1.18889	2.10E-06	1.73E-05	down	314	369	271	549	1163	656	PAQR6
-2.02667	0.0158751	0.044883	down	2	6	2	19	12	11	APOBR
-2.02041	0.0041293	0.014466	down	3	5	5	11	23	22	AFAP1L2
-3.2767	1.01E-39	1.85E-37	down	405	347	320	4263	3683	2817	MUC4
-2.47651	0.001882	0.007465	down	6	5	1	10	48	18	FOXD4
-1.07125	0.0032371	0.011784	down	62	83	49	77	228	144	TM4SF19
-3.69193	0.0082795	0.026134	down	0	0	2	5	12	10	ZNF670
-2.87252	0.0080561	0.025526	down	2	3	0	4	27	11	ZDHHC1
-5.27159	0.0009582	0.004138	down	0	0	0	6	12	5	TPPP3
-3.49556	3.71E-16	1.25E-14	down	15	11	13	198	111	135	FRK
-3.10483	2.86E-16	9.69E-15	down	25	26	11	174	268	134	UPK3B
-1.61085	0.0067544	0.021977	down	3	10	11	22	33	22	
-1.4906	9.17E-14	2.38E-12	down	204	181	189	419	716	582	
-1.02396	1.38E-06	1.19E-05	down	135	133	165	247	420	268	RGS12
-1.81182	6.92E-07	6.38E-06	down	20	26	32	93	133	63	ADRA1B
-2.51436	0.0052289	0.017692	down	5	7	7	85	8	9	EPHA5
-1.22745	0.0067544	0.021977	down	21	21	18	60	59	28	CORIN
-2.40188	7.57E-11	1.32E-09	down	19	14	19	104	97	83	LYPD5
-4.21903	6.00E-06	4.48E-05	down	1	3	0	29	33	17	CXCL11
-2.48498	8.48E-12	1.68E-10	down	29	17	31	177	164	103	SH3TC2
-4.37037	0.0004336	0.002057	down	1	0	1	14	16	13	CXCL10
-6.61943	1.07E-06	9.49E-06	down	0	0	0	15	27	16	ABCC11
-3.59319	2.54E-05	0.000165	down	1	4	1	29	35	13	SLC26A1
-2.17141	1.48E-06	1.27E-05	down	16	15	10	41	105	56	MAFB
-2.49942	4.00E-06	3.10E-05	down	10	6	5	45	50	30	GABARAP
-1.57794	0.0001225	0.000674	down	38	25	60	171	115	82	RNASEL
-1.23374	1.58E-07	1.64E-06	down	123	104	111	233	388	228	
-5.26057	0.0011358	0.0048	down	0	0	0	6	13	4	KIAA1614
-3.09435	2.73E-16	9.30E-15	down	26	25	70	421	296	310	MAN1A1
-2.01373	4.70E-07	4.47E-06	down	13	22	17	61	70	88	MAK
-1.67995	3.21E-09	4.41E-08	down	58	39	78	148	264	180	RSPH4A
-1.38987	0.0037746	0.013427	down	16	7	18	29	48	36	
-1.03909	0.0012001	0.00505	down	43	42	76	76	141	130	NME2
-2.0549	0.0001836	0.000964	down	10	6	8	25	32	47	LOC101927787
-2.07023	0.002942	0.010851	down	2	5	7	22	25	14	
-1.6424	0.0018369	0.007311	down	13	9	7	34	35	26	

-1.1656	2.40E-09	3.34E-08	down	364	341	430	618	1112	968	IL10RB
-1.02333	4.90E-07	4.63E-06	down	139	174	165	283	392	348	SCD5
-3.67704	2.18E-09	3.06E-08	down	3	7	3	78	49	43	SORCS2
-1.35703	9.92E-10	1.47E-08	down	449	365	706	1135	1725	1206	FOS
-1.411	0.0050392	0.017145	down	11	9	21	37	53	24	OR2A20P
-4.72711	0.0071759	0.023139	down	0	0	0	3	9	4	EDAR
-3.66449	8.39E-12	1.66E-10	down	8	4	3	48	96	61	LOC102724788
-2.63001	3.46E-12	7.26E-11	down	14	10	24	87	107	113	TSGA10
-1.13936	1.70E-08	2.07E-07	down	294	337	264	591	788	706	DNASE1L1
-1.36312	1.14E-06	1.00E-05	down	164	179	154	252	686	457	DNAJB2
-3.06788	2.23E-09	3.12E-08	down	17	8	4	49	129	89	WNT10A
-1.67807	0.0034298	0.012369	down	6	14	7	36	31	23	LOC100287808
-1.81827	0.0001668	0.000886	down	15	15	8	55	48	37	CYP27A1
-5.39053	0.0008235	0.003623	down	0	0	0	8	14	3	CHRND
-6.23015	2.69E-06	2.17E-05	down	0	0	1	13	45	30	SMIM22
-1.10822	0.0023	0.008828	down	28	33	26	44	81	75	LIN37
-2.6638	1.21E-08	1.51E-07	down	9	8	9	48	84	44	
-1.93104	2.07E-07	2.09E-06	down	20	19	17	53	98	77	HOXA10-AS
-2.55096	0.0095264	0.029448	down	1	2	3	17	14	5	
-4.70117	0.006967	0.022568	down	0	0	0	4	5	6	
-2.9227	0.0005568	0.002564	down	4	2	1	22	23	11	
-1.52389	2.55E-05	0.000166	down	19	21	32	55	99	65	
-1.28903	0.0003135	0.001548	down	26	20	31	63	81	53	
-6.12573	1.63E-05	0.000111	down	0	0	0	9	17	15	FAT2
-1.12137	0.0126704	0.037258	down	12	22	16	30	51	35	CLCNKB
-1.58763	0.0045319	0.015629	down	12	12	5	20	45	30	LCN12
-2.56644	0.0123493	0.036517	down	0	1	5	7	17	13	
-1.53157	1.16E-06	1.01E-05	down	58	57	91	249	214	145	KIAA1024
-1.76487	0.0073628	0.023657	down	3	8	6	14	23	24	
-1.02824	0.0032348	0.011778	down	31	26	56	72	83	82	
-7.24995	2.58E-06	2.10E-05	down	1	2	0	350	62	34	KRT4
-1.87227	0.0123275	0.036476	down	5	2	5	11	17	18	MZB1
-1.34475	0.0062188	0.020466	down	12	11	12	21	43	31	SLC23A1
-6.77841	8.46E-07	7.67E-06	down	0	0	0	21	34	10	CYP4F29P
-1.74461	1.76E-14	5.05E-13	down	115	124	163	496	509	387	LHX4
-2.688	0.0076487	0.024419	down	1	2	2	8	20	7	
-2.70731	5.29E-12	1.08E-10	down	11	13	18	72	143	78	SPIN3
-3.01977	0.0002352	0.001204	down	3	2	2	12	34	16	
-5.20727	0.0001824	0.000958	down	0	1	0	11	17	14	
-1.75374	9.82E-07	8.74E-06	down	28	24	19	66	117	74	PSMB8-AS1
-1.92481	0.0003095	0.001532	down	7	7	13	39	29	36	OR2A7
-3.63605	7.22E-08	7.93E-07	down	5	2	2	26	59	36	RALY-AS1
-6.0395	2.98E-11	5.45E-10	down	1	1	1	62	74	70	PTAFR
-1.76449	0.004689	0.0161	down	10	6	4	22	34	17	MZF1-AS1

-2.38429	4.28E-05	0.000264	down	8	4	5	28	40	26	
-3.16948	2.39E-10	3.87E-09	down	7	13	4	61	117	57	
-3.23129	0.0001577	0.000843	down	0	4	8	60	12	35	RASSF6
-2.31612	2.00E-05	0.000134	down	22	17	4	55	131	53	CLIC3
-2.24553	2.18E-17	8.33E-16	down	36	42	41	165	260	174	TMEM14A
-6.38062	2.08E-24	1.52E-22	down	5	3	2	445	257	142	
-1.18811	0.0006984	0.003136	down	46	47	32	61	136	111	APOM
-2.74748	8.33E-05	0.000479	down	5	5	1	19	36	25	LAT2
-2.66066	0.0044194	0.015328	down	2	4	1	9	33	8	
-1.69926	2.06E-10	3.37E-09	down	56	71	63	229	217	194	HSPA1L
-3.01247	2.48E-36	3.71E-34	down	54	55	65	454	653	377	VWA7
-5.68936	8.20E-14	2.14E-12	down	4	0	1	53	104	117	C1orf210
-2.29961	0.015899	0.044931	down	1	3	2	10	12	9	
-2.22091	1.38E-06	1.19E-05	down	10	15	22	36	106	92	
-2.79973	0.00011	0.000613	down	3	6	1	19	32	24	ACY1
-10.2431	2.80E-17	1.06E-15	down	0	0	0	195	340	181	
1.150981	1.32E-07	1.39E-06	up	364	350	371	178	164	164	DLX1
1.126043	0.0012901	0.005373	up	1630	1619	2757	1336	588	776	TMED7
4.092989	1.18E-41	2.29E-39	up	2095	1813	3038	188	138	86	ETS1
1.486279	3.57E-18	1.47E-16	up	734	716	725	232	320	267	BCL7A
2.962611	1.32E-07	1.39E-06	up	38	35	57	6	3	8	PCDHB14
2.922198	0.0080786	0.025583	up	11	12	5	1	2	1	PCDHB12
1.649629	0.0087625	0.027445	up	19	25	24	12	5	5	ST7-AS1
1.046099	2.44E-08	2.90E-07	up	768	738	1124	360	523	447	IRF2
2.422524	0.0001685	0.000894	up	24	34	26	7	2	7	
3.003864	0.0015111	0.006159	up	14	13	11	2	2	1	LINC00882
5.334107	0.0015375	0.006249	up	14	3	4	0	0	0	
1.065158	0.0001279	0.0007	up	391	390	589	280	201	181	SDPR
1.761868	0.0139844	0.040471	up	22	12	15	7	5	3	
1.228305	4.24E-10	6.64E-09	up	520	430	488	189	226	227	RHBDD1
1.473178	4.18E-14	1.14E-12	up	2761	2512	3916	1145	1170	1084	MAP4K4
1.179332	4.87E-09	6.54E-08	up	368	308	370	147	170	165	NCK2
1.731156	6.68E-14	1.77E-12	up	316	340	470	122	117	110	
2.579953	7.31E-36	1.05E-33	up	4559	4262	6704	922	878	855	RAB31
4.771385	8.65E-10	1.30E-08	up	40	45	47	2	0	3	LINC01537
1.186457	7.29E-10	1.10E-08	up	1000	1331	1202	417	677	555	GMPPA
2.213085	1.86E-12	4.08E-11	up	315	362	330	38	84	109	TAMM41
1.017314	4.71E-07	4.47E-06	up	1285	1441	1364	492	834	819	COPS7B
1.426533	1.54E-06	1.32E-05	up	342	387	326	69	196	161	IFT43
1.613061	0.0030122	0.011073	up	21	22	42	8	13	8	CCDC176
1.350186	2.79E-12	5.94E-11	up	7189	7686	####	3579	3460	3151	ARCN1
1.550265	2.71E-14	7.60E-13	up	1165	1035	1352	459	431	360	MTCL1
1.263676	3.87E-14	1.07E-12	up	6830	7428	9981	3247	3794	3415	COPB1
2.798122	8.62E-27	7.62E-25	up	285	236	334	46	41	40	STARD8

2.021509	8.24E-23	5.29E-21	up	1112	1581	1694	362	449	319	SAT1
1.58271	5.72E-27	5.13E-25	up	1844	1781	2258	603	800	653	FHL1
2.023298	6.19E-21	3.35E-19	up	502	449	413	99	143	114	POMGNT2
7.014269	7.30E-11	1.28E-09	up	71	80	95	0	1	1	RBMS3
4.624966	0.0002368	0.001211	up	12	14	20	0	2	0	GAMT
1.053482	0.0021425	0.008332	up	71	81	70	23	46	45	TGFB3
1.353545	2.14E-10	3.49E-09	up	264	228	325	97	128	109	DNAL1
1.437837	4.63E-06	3.55E-05	up	108	80	115	36	53	29	ENOX1
3.751792	6.81E-52	2.23E-49	up	752	915	757	40	78	74	TNNT1
1.095714	0.0041477	0.014523	up	515	388	862	418	194	197	PTPRG
4.389015	4.52E-42	9.03E-40	up	246	246	269	12	12	14	VASH1
1.322313	7.44E-11	1.30E-09	up	4870	4741	5141	1328	2636	2335	TGFBI
1.182914	2.61E-06	2.11E-05	up	3500	3385	6568	2073	1899	2024	RBM25
2.683652	9.28E-10	1.38E-08	up	63	49	81	10	9	12	ZNF30
2.657242	2.62E-05	0.00017	up	1260	1253	2201	410	225	104	IL7R
2.542159	0.0001345	0.000733	up	24	27	27	7	6	1	ROBO4
4.821821	1.78E-31	2.14E-29	up	260	284	197	11	14	3	ROBO3
1.028375	1.27E-09	1.86E-08	up	1002	1005	966	421	595	524	FAM64A
1.20162	1.63E-10	2.70E-09	up	1324	1336	1439	430	790	677	DOCK6
1.402242	7.57E-08	8.28E-07	up	503	466	920	240	237	250	MOSPD2
2.594901	4.02E-16	1.34E-14	up	910	904	1428	256	166	117	UBASH3B
1.222984	4.46E-14	1.21E-12	up	1164	997	1202	409	598	511	USP40
2.666793	9.49E-32	1.15E-29	up	439	346	534	64	78	74	LRIG1
2.202642	3.35E-08	3.87E-07	up	109	64	134	21	17	30	ZFP30
1.240777	0.0006802	0.003061	up	67	49	93	33	32	26	IL17RD
4.443477	0.0024116	0.009169	up	23	18	1	2	0	0	USP6
1.018542	1.54E-08	1.90E-07	up	2225	2279	1992	1051	1349	990	PVRL2
1.059345	0.0020213	0.007937	up	78	104	90	28	49	61	APOE
1.867131	6.23E-14	1.66E-12	up	297	299	241	70	86	86	PRX
1.691373	8.15E-07	7.41E-06	up	2891	2430	4898	1540	878	713	PHLDB2
1.077352	3.21E-07	3.14E-06	up	1142	1264	996	435	640	636	NUMBL
1.919306	2.82E-16	9.60E-15	up	529	556	591	178	144	134	SOCS2
2.343789	2.37E-18	1.00E-16	up	319	292	248	41	83	58	PKIG
2.521999	1.42E-06	1.23E-05	up	29	59	51	10	7	8	SH2D3C
2.329178	0.0006257	0.002846	up	19	23	19	2	6	5	CNTD2
1.472072	1.27E-13	3.20E-12	up	1765	2002	2831	749	829	871	ARL1
4.955208	4.48E-05	0.000275	up	18	16	24	0	2	0	GLT8D2
2.074671	1.52E-07	1.58E-06	up	72	87	161	32	25	20	
2.983274	1.11E-56	4.12E-54	up	652	660	736	74	110	89	TSPAN5
1.348327	0.0078928	0.025088	up	35	41	128	16	31	35	NDUFV2
4.045732	1.32E-17	5.18E-16	up	99	108	179	4	5	15	METTL10
1.491991	0.012788	0.037529	up	24	18	22	9	11	4	
1.328123	1.01E-06	9.01E-06	up	348	400	608	218	192	141	ABCA5
3.400475	3.78E-16	1.27E-14	up	88	98	142	8	19	6	ABCA10

1.054444	7.40E-07	6.78E-06	up	1408	1337	1215	454	831	754	GTPBP3
1.063944	1.72E-09	2.46E-08	up	1758	2005	1862	927	1038	850	PITPNC1
1.84348	0.0002552	0.001293	up	40	59	29	9	13	16	FAM229B
1.37619	8.32E-06	6.02E-05	up	90	115	97	29	53	42	APLP1
1.114684	3.89E-14	1.07E-12	up	3955	3893	3966	1732	2226	1782	PLIN3
1.001945	5.07E-05	0.000307	up	1035	1010	959	301	711	606	ICAM5
1.775361	3.07E-06	2.45E-05	up	92	71	138	38	33	19	TRPC1
1.553726	0.0064236	0.021052	up	23	17	47	11	9	10	
1.594431	1.96E-12	4.28E-11	up	1147	962	1616	430	392	436	KDELC2
2.574295	2.92E-34	3.78E-32	up	2352	2570	2267	281	571	446	TGFB1
1.349012	8.70E-10	1.30E-08	up	1222	1144	1942	535	568	629	MXI1
3.962946	0.0149327	0.042714	up	6	5	5	0	1	0	PPP1R3C
1.669628	1.43E-08	1.77E-07	up	1076	901	1582	436	267	415	OGFRL1
1.416099	1.11E-05	7.84E-05	up	79	68	83	24	38	29	
1.420832	1.84E-05	0.000123	up	69	92	103	24	50	31	CFAP45
7.967918	7.42E-10	1.12E-08	up	41	62	27	0	0	0	LOC101927727
3.499306	6.91E-76	3.98E-73	up	1602	1457	1619	144	143	143	FAM167A
1.889056	5.04E-19	2.24E-17	up	849	792	790	159	327	220	SLC44A2
1.250539	2.09E-12	4.53E-11	up	2084	2458	2202	860	1059	1055	SNHG5
4.431634	1.06E-24	8.04E-23	up	211	197	162	3	8	17	CACNG6
1.574379	0.0109173	0.032986	up	36	32	13	6	16	8	CLEC11A
1.116821	1.28E-06	1.12E-05	up	843	758	1374	449	464	489	CEP120
4.541641	0.0035656	0.012788	up	3	12	9	1	0	0	STXBP6
1.832042	3.25E-19	1.47E-17	up	1276	1054	1167	241	409	392	MEGF8
1.040489	4.75E-08	5.35E-07	up	1320	1457	1196	549	871	643	PXDC1
1.574526	9.62E-09	1.23E-07	up	193	156	289	59	101	65	ZNF354B
2.087167	2.06E-17	7.93E-16	up	790	801	828	118	233	254	CDKN2AIPNL
1.600164	1.45E-09	2.10E-08	up	597	676	422	152	273	180	RABAC1
5.043025	0.0038509	0.013665	up	10	5	2	0	0	0	
1.409237	0.0003311	0.001624	up	106	64	58	32	30	28	
1.27196	0.0003075	0.001525	up	84	70	70	30	47	22	HIST1H4E
2.043888	5.84E-08	6.51E-07	up	81	62	111	23	18	22	
1.051683	6.15E-05	0.000366	up	141	121	130	53	73	73	
3.505602	0.0001583	0.000846	up	21	11	23	3	2	0	GBP5
1.771377	1.83E-06	1.54E-05	up	91	64	97	14	31	33	UNC13A
1.007711	0.0002169	0.001119	up	227	205	325	151	119	113	SH3RF1
1.526806	0.0001112	0.000619	up	138	98	59	29	44	37	KLHDC7B
2.603594	2.98E-08	3.48E-07	up	69	67	99	19	17	4	OLFM1
3.156654	6.89E-23	4.50E-21	up	245	228	227	19	48	18	CXCL2
1.900463	1.58E-19	7.33E-18	up	1099	1114	920	236	403	262	GDF15
4.988583	0.0005498	0.002535	up	12	7	14	0	1	0	LINC00632
1.534224	1.85E-09	2.63E-08	up	367	430	335	88	179	153	FOXD2-AS1
3.167119	0.0078983	0.025093	up	7	6	28	0	5	0	SORBS2
1.418032	1.17E-09	1.73E-08	up	635	618	489	168	269	258	SIPA1

3.470394	1.01E-38	1.78E-36	up	387	389	358	33	51	25	IL11
1.065844	2.41E-05	0.000158	up	298	193	296	136	142	112	ERN1
9.481248	7.63E-15	2.27E-13	up	107	107	168	0	0	0	TMPRSS15
1.255896	1.39E-08	1.72E-07	up	257	334	301	103	162	131	ARG2
1.486735	2.55E-06	2.07E-05	up	97	96	116	28	60	30	CYP2E1
1.322383	2.28E-07	2.30E-06	up	2239	2035	2949	1235	931	764	SEC14L1
5.10326	2.97E-14	8.28E-13	up	64	98	65	1	2	4	TMEM59L
1.915005	4.03E-23	2.69E-21	up	904	1021	1362	290	361	257	IL1A
2.667586	1.13E-08	1.42E-07	up	49	53	91	9	17	6	CCL20
1.258882	1.67E-07	1.72E-06	up	317	372	270	128	154	141	JAK3
1.111432	0.0081984	0.025921	up	34	48	42	18	28	15	PDE4C
2.63097	0.0003084	0.001528	up	27	13	45	7	6	1	
5.24703	0.0013113	0.005444	up	10	4	6	0	0	0	GPR65
4.941874	0.0060656	0.020041	up	11	2	3	0	0	0	PTPRC
3.660841	9.30E-31	1.07E-28	up	178	170	222	16	17	14	THBD
2.360301	8.93E-50	2.74E-47	up	9001	8624	8812	1588	1913	1889	WARS
4.960878	7.60E-43	1.57E-40	up	521	498	939	24	11	28	ADAMTS1
1.45056	4.07E-13	9.55E-12	up	818	797	971	234	372	389	MRPL39
2.399028	4.01E-13	9.45E-12	up	136	114	190	16	38	34	CFAP69
1.835628	1.19E-05	8.34E-05	up	39	62	73	12	19	20	C1orf204
1.352387	2.27E-09	3.18E-08	up	218	193	282	81	106	96	
1.131095	2.09E-09	2.95E-08	up	1362	1633	2012	762	801	796	PGRMC2
1.678641	1.46E-08	1.80E-07	up	540	489	871	247	157	190	KCTD12
1.545737	1.65E-07	1.70E-06	up	128	183	202	69	67	46	SCN1B
1.523326	2.01E-12	4.37E-11	up	686	855	698	277	315	227	WNT7B
1.019263	4.10E-05	0.000255	up	255	254	278	82	180	153	C11orf95
1.1146	7.44E-06	5.47E-05	up	1181	1161	1901	770	615	598	CDK6
4.532487	5.76E-43	1.20E-40	up	363	393	286	11	19	18	GPSM3
1.303929	5.74E-05	0.000344	up	318	357	727	233	201	139	STK17B
2.341339	0.0128419	0.037652	up	15	6	12	1	4	2	
2.209198	0.0002709	0.001366	up	27	22	33	2	10	7	S1PR3
1.119979	1.16E-06	1.01E-05	up	306	275	282	130	131	152	RAD52
2.890133	5.01E-16	1.65E-14	up	334	286	504	73	55	26	CLIP4
1.438687	3.38E-09	4.64E-08	up	258	263	377	111	105	124	FBXO8
6.762412	3.05E-13	7.30E-12	up	84	65	167	0	1	2	FAM198B
1.805441	4.44E-16	1.47E-14	up	1603	1424	1710	541	500	359	AMIGO2
1.002515	0.0003047	0.001513	up	122	169	201	58	119	84	PLCL2
1.378212	2.62E-10	4.23E-09	up	1683	1784	1460	457	817	754	MAPK12
1.59903	6.32E-07	5.87E-06	up	500	396	1071	236	209	207	NHS
1.621274	1.34E-10	2.25E-09	up	4723	4658	8100	2217	1771	1746	NAMPT
1.896548	9.91E-14	2.55E-12	up	228	225	293	71	61	74	ZC3H6
5.495262	6.59E-05	0.000388	up	18	16	12	0	1	0	PON3
3.681129	1.01E-46	2.50E-44	up	639	645	848	32	89	57	DFNA5
1.039937	3.98E-08	4.52E-07	up	536	497	691	286	316	267	ZNF134

1.472774	5.46E-16	1.78E-14	up	573	594	580	193	274	199	HYI
1.108659	1.52E-09	2.19E-08	up	542	508	650	208	329	293	SLC27A2
1.670216	6.59E-07	6.09E-06	up	3512	3777	5248	1958	1262	738	PHLDA1
1.349737	0.0030498	0.01119	up	124	65	67	47	21	34	INHBE
4.062709	0.0117576	0.035096	up	7	6	4	1	0	0	EGFLAM
5.516441	5.49E-05	0.00033	up	18	14	15	0	1	0	DUOXA1
2.428698	1.72E-13	4.27E-12	up	246	248	414	77	57	37	GLIPR1
1.554494	5.29E-05	0.000319	up	1308	1243	3186	941	495	470	RICTOR
1.042346	0.003634	0.012985	up	991	731	1733	757	366	521	CFAP97
4.292482	6.00E-05	0.000358	up	27	11	19	1	0	2	PSTPIP1
2.289826	0.0013869	0.005712	up	17	17	24	6	2	4	F2RL2
2.080636	1.30E-11	2.51E-10	up	2305	2144	3249	861	577	394	EFEMP1
1.25395	2.04E-05	0.000136	up	2364	2554	4719	1711	1227	1105	PRRC1
1.962544	1.37E-16	4.86E-15	up	640	643	941	187	170	226	POC1B
1.179355	1.44E-09	2.10E-08	up	658	816	728	270	450	317	DDAH2
1.303229	8.08E-14	2.11E-12	up	1897	1797	2439	767	880	926	ANKRD40
1.943517	5.86E-26	4.76E-24	up	1810	2139	2903	525	707	623	WDR75
2.622724	0.0043526	0.015137	up	6	13	23	3	3	1	EVA1A
5.068158	0.0102396	0.031287	up	8	9	0	0	0	0	ESM1
1.18612	6.37E-09	8.39E-08	up	375	384	383	174	180	169	HOXA3
1.3759	1.59E-14	4.57E-13	up	560	661	701	214	297	267	HABP4
1.805622	8.13E-07	7.40E-06	up	75	70	96	21	19	31	RAB6B
1.527586	3.43E-06	2.71E-05	up	184	178	125	36	69	76	DOK1
1.210975	1.40E-05	9.69E-05	up	170	122	200	51	86	86	TTC26
2.220699	2.14E-06	1.76E-05	up	179	131	110	52	27	13	TRAF1
5.130262	0.0026803	0.010022	up	2	6	11	0	0	0	
1.246643	0.0045274	0.01562	up	77	63	52	14	28	44	SLC35A1
4.661623	0.0152155	0.04338	up	9	3	1	0	0	0	EBF1
1.560791	0.0022162	0.008572	up	58	68	96	45	15	14	SNX18
1.513717	0.0109806	0.03312	up	25	30	18	4	15	9	CYP11A1
2.022895	8.78E-24	6.18E-22	up	525	561	762	145	165	161	FAM92A1
1.802764	3.55E-23	2.38E-21	up	758	787	1044	222	279	271	TCHP
1.434661	4.24E-16	1.41E-14	up	589	556	750	201	297	238	FOCAD
1.478628	8.29E-12	1.65E-10	up	1883	1974	2015	457	999	807	MAN2C1
1.525986	0.0024054	0.009149	up	35	35	26	8	18	10	SDSL
1.040913	0.0016407	0.006614	up	1089	1106	2147	959	541	585	GLS
4.970234	0.0041687	0.014586	up	21	10	0	1	0	0	
1.692058	1.10E-05	7.75E-05	up	121	87	275	44	54	54	
2.841981	3.00E-18	1.25E-16	up	297	272	628	63	57	49	ITGA1
4.98998	0.0053914	0.018151	up	1	8	8	0	0	0	HBE1
6.352315	5.87E-06	4.40E-05	up	18	12	13	0	0	0	
2.480796	2.96E-05	0.000189	up	29	27	28	3	7	6	ACVRL1
1.192169	4.77E-06	3.65E-05	up	3748	3096	2105	1055	1885	1305	H2AFX
1.659384	0.0001076	0.000602	up	56	43	55	11	16	24	LIPE-AS1

1.613674	3.81E-23	2.55E-21	up	4509	4534	4853	1206	1856	1734	SMARCD1
2.105953	7.69E-23	4.97E-21	up	517	533	500	96	139	145	DUSP12
1.799896	0.0008238	0.003623	up	26	48	31	6	11	15	
3.483009	0.0032661	0.01188	up	8	15	8	1	2	0	STEAP1
1.211784	4.54E-08	5.14E-07	up	808	876	1442	451	508	429	FAM72B
1.239078	0.0001242	0.000682	up	152	109	197	82	60	55	WDR7
1.957152	2.30E-06	1.89E-05	up	60	50	76	21	14	14	ZNF704
1.923433	4.59E-36	6.70E-34	up	1326	1260	1585	329	451	373	GALNT10
3.93343	0.0166548	0.046678	up	4	4	8	1	0	0	MYOZ3
1.546693	1.89E-11	3.57E-10	up	1938	1884	1584	423	809	753	C16orf58
1.122561	0.009628	0.02969	up	31	31	45	16	18	17	ITGAX
1.806528	0.0001135	0.00063	up	56	38	61	22	12	11	NFATC2
2.640716	6.19E-20	2.96E-18	up	158	191	216	20	42	34	MAP3K12
1.76163	2.70E-19	1.23E-17	up	1038	1181	1060	329	417	275	FHL2
1.370689	0.0001877	0.000984	up	49	61	85	23	26	29	
1.478407	0.0004931	0.002301	up	105	103	55	25	57	22	SOX17
1.324644	1.14E-15	3.65E-14	up	16179	####	####	5657	8556	7773	CTSB
1.417599	0.0005807	0.002661	up	134	90	128	69	31	32	DLC1
1.146214	2.93E-05	0.000188	up	10049	9304	####	6603	4592	5317	ITGB1
4.740169	3.56E-07	3.46E-06	up	22	50	26	0	3	1	ARSD
2.803886	0.0008083	0.003562	up	15	13	20	3	1	3	ARHGAP44
2.267488	1.71E-12	3.77E-11	up	231	205	406	69	48	59	MPP7
2.249022	3.14E-38	5.43E-36	up	1674	1678	2054	389	402	386	PTPRJ
1.39685	1.30E-07	1.37E-06	up	420	349	745	190	209	189	SLC25A30
1.106662	0.0008267	0.003633	up	97	84	128	60	42	44	LINC00630
3.877015	0.0049518	0.016893	up	7	6	16	0	0	2	SERPING1
1.7802	2.86E-23	1.95E-21	up	13592	####	####	2969	5023	4686	SSRP1
1.205335	0.0001943	0.001016	up	90	118	85	36	46	52	BIRC7
1.420978	8.17E-07	7.42E-06	up	194	175	220	41	110	85	CTSF
2.498915	2.19E-15	6.92E-14	up	423	408	564	117	83	51	MSRB3
1.317594	4.23E-07	4.06E-06	up	313	304	338	75	183	153	SIRPA
1.723625	7.99E-22	4.70E-20	up	1317	1524	1908	483	562	449	CDR2
1.535681	4.85E-05	0.000295	up	75	92	181	46	36	39	ATP8B1
3.540462	0.0027097	0.010114	up	9	6	20	1	0	2	
1.232539	0.013356	0.038949	up	38	36	53	25	24	7	DPPA2
1.502336	1.90E-13	4.67E-12	up	3062	2950	4692	1014	1416	1491	IFI16
1.908398	2.15E-09	3.02E-08	up	141	113	100	30	40	30	ADORA1
1.350911	2.74E-06	2.21E-05	up	127	119	149	35	61	67	SEC14L2
1.053824	0.0003951	0.001893	up	84	86	115	49	56	38	C4orf36
7.674015	1.26E-78	8.26E-76	up	1089	896	1114	4	8	4	PTX3
1.089654	0.0025703	0.009668	up	72	120	156	43	49	76	RGPD1
2.459292	5.67E-22	3.37E-20	up	295	241	298	54	48	55	ZKSCAN4
1.493435	0.0001205	0.000664	up	82	74	52	25	35	19	
1.021996	0.0008452	0.003702	up	1344	1148	1851	1003	622	517	APBB2

1.317372	1.60E-08	1.96E-07	up	6134	6961	4444	2108	3252	2209	LGALS1
1.34478	3.04E-05	0.000194	up	212	209	393	121	81	119	PLSCR4
1.07391	9.41E-05	0.000533	up	719	619	1052	472	325	345	FGF2
1.193055	3.33E-10	5.28E-09	up	541	579	727	266	285	285	COPS4
3.271475	1.50E-09	2.16E-08	up	37	46	66	6	6	4	PLA2G3
2.534189	3.47E-11	6.29E-10	up	99	64	104	13	16	19	ARHGAP24
1.746645	3.98E-12	8.26E-11	up	727	698	1160	302	250	230	HERC3
1.062465	2.31E-07	2.32E-06	up	431	456	441	160	300	220	MOK
1.735672	2.99E-11	5.46E-10	up	328	387	432	140	135	82	SEMA7A
1.408216	0.000109	0.000608	up	93	78	59	30	37	25	SLC35E4
1.648908	9.55E-21	5.01E-19	up	1887	1592	1687	540	620	564	PPM1F
1.218991	2.15E-06	1.78E-05	up	394	419	413	104	260	204	HEMK1
1.044367	0.0001032	0.000579	up	1325	1268	2116	883	611	798	LARP1B
1.152522	2.79E-07	2.76E-06	up	391	384	313	137	208	176	GCAT
4.141755	5.57E-13	1.29E-11	up	70	60	54	4	4	3	CXCL3
5.924254	8.78E-05	0.000502	up	9	5	19	0	0	0	CPA3
1.62075	1.08E-11	2.11E-10	up	4099	3959	5704	1763	1302	1462	TCEA1
1.394439	2.29E-10	3.72E-09	up	2592	2240	3894	1202	1178	1014	THSD4
1.163105	2.79E-07	2.76E-06	up	375	435	383	137	206	220	MRPS33
2.192159	2.87E-08	3.36E-07	up	85	57	66	13	18	17	ZNF70
1.019986	0.0012587	0.00526	up	173	166	106	65	109	63	SERTAD1
2.964183	1.90E-14	5.39E-13	up	78	84	113	8	15	14	BNC2
1.70258	6.58E-17	2.39E-15	up	1024	1131	900	265	381	350	TOX2
1.218678	9.13E-07	8.19E-06	up	384	364	597	187	178	225	ZMYM1
1.483422	2.87E-11	5.27E-10	up	985	1186	1806	482	515	463	UGCG
2.487793	2.06E-13	5.05E-12	up	152	126	129	31	21	23	ZSCAN26
1.428814	1.94E-11	3.64E-10	up	264	270	323	82	137	117	LEF1
1.103503	6.46E-06	4.80E-05	up	2919	2993	3517	1858	1554	1088	ANXA3
1.571059	0.0014344	0.005882	up	28	32	30	9	13	10	SLC12A5
2.686557	0.0103175	0.031464	up	8	13	9	2	3	0	ARHGAP40
2.980098	5.15E-46	1.24E-43	up	613	756	789	75	105	107	KDELR3
3.624963	2.67E-34	3.47E-32	up	651	529	923	75	47	50	FRAS1
1.041822	5.19E-06	3.94E-05	up	1481	1200	2343	811	873	814	11-S
1.013557	0.0003929	0.001884	up	541	494	855	390	260	289	CNOT6L
1.778624	0.0017818	0.007117	up	26	29	53	6	22	6	AADAC
1.005453	1.04E-07	1.11E-06	up	882	720	939	417	557	357	PREX1
1.37261	3.34E-07	3.26E-06	up	348	402	490	105	171	225	NAAA
1.024061	0.0003913	0.001877	up	585	624	1195	439	338	409	KLHL24
1.149114	1.45E-05	0.0001	up	159	132	237	76	96	75	ARHGEF26
4.325446	5.39E-35	7.31E-33	up	233	198	333	12	20	8	TTC28
1.974369	3.92E-09	5.32E-08	up	294	257	247	96	74	40	RGS3
1.011679	2.74E-11	5.06E-10	up	3756	3554	4305	1932	2199	1864	CDCP1
1.039474	6.23E-10	9.57E-09	up	1000	1133	1409	549	683	564	NDC80
1.118537	8.90E-10	1.33E-08	up	1986	1797	1893	919	925	872	SUN2

1.668974	4.86E-16	1.61E-14	up	427	335	491	122	154	135	FYCO1
2.825575	1.21E-26	1.05E-24	up	18351	####	####	3649	2610	2308	FBN2
1.568356	6.15E-05	0.000366	up	94	78	94	44	23	24	PEAR1
1.168905	0.0003014	0.001497	up	91	108	148	59	43	55	ZMYM6
1.481528	6.86E-15	2.05E-13	up	715	924	841	288	362	284	HIST1H1C
2.463534	3.49E-20	1.72E-18	up	481	434	479	108	95	59	ICAM1
4.693761	1.05E-47	2.81E-45	up	309	271	335	14	14	9	ABLIM3
1.726769	2.15E-06	1.78E-05	up	64	71	129	23	35	25	CTSO
3.638499	6.35E-17	2.31E-15	up	106	78	105	10	6	8	MILR1
1.46313	2.61E-06	2.11E-05	up	716	588	1230	399	269	250	ZBTB21
1.519045	4.35E-07	4.16E-06	up	120	139	124	43	41	55	COMMD1
1.146586	3.78E-08	4.33E-07	up	1027	1059	852	370	629	426	PEPD
1.953014	0.0028291	0.010494	up	19	21	21	2	9	6	ASTN2
1.721805	6.06E-12	1.22E-10	up	388	352	284	84	125	121	BCAS4
2.792358	5.40E-24	3.81E-22	up	220	252	246	34	51	25	SNAI1
1.58414	4.24E-17	1.57E-15	up	1773	2005	2006	472	847	734	PDIA5
1.030617	2.02E-10	3.31E-09	up	1891	1759	2417	969	1133	981	TTL
3.820803	2.08E-21	1.18E-19	up	151	115	157	8	19	5	KIAA1644
1.605	2.64E-12	5.63E-11	up	4469	4435	7323	1974	2090	1418	ARHGAP11B
1.437453	0.0153616	0.043724	up	33	23	21	13	13	4	DNER
2.815385	0.0041279	0.014464	up	13	17	8	0	4	2	ACR
1.209071	0.0001013	0.000569	up	302	343	297	190	126	101	PDGFB
1.799242	2.95E-13	7.08E-12	up	374	283	404	118	111	86	NEK4
1.335989	4.18E-08	4.74E-07	up	465	584	504	138	315	212	
3.310117	7.93E-32	9.73E-30	up	485	483	692	74	49	47	PPP1R3B
2.233187	0.0156417	0.044363	up	12	9	11	2	1	4	SERPINA1
2.779066	4.75E-12	9.72E-11	up	211	153	258	47	17	26	MYLK
2.926703	4.98E-18	2.03E-16	up	215	204	237	43	24	21	P3H2
2.574819	0.0176711	0.049106	up	11	10	8	1	0	4	IL2RB
3.865168	1.44E-07	1.51E-06	up	48	39	24	2	1	5	KCTD17
1.734447	0.0036012	0.012887	up	53	49	26	23	7	9	SAA1
1.586882	1.86E-16	6.51E-15	up	3452	3331	2755	893	1362	1130	RNF26
5.938924	6.07E-70	3.12E-67	up	1008	880	1696	22	12	25	PTPRM
1.233852	0.000141	0.000764	up	148	199	110	51	93	66	STMN3
6.091559	2.08E-05	0.000139	up	13	11	12	0	0	0	SMIM3
5.334107	0.0015375	0.006249	up	14	3	4	0	0	0	ZNF486
6.225366	3.48E-05	0.00022	up	25	6	8	0	0	0	PCDHB11
2.299621	0.0126327	0.037182	up	13	19	9	0	3	6	
2.882967	3.91E-26	3.23E-24	up	933	1123	1191	190	170	95	COL13A1
1.617961	0.0010484	0.004484	up	51	37	34	6	20	17	ZNF517
2.297031	3.87E-42	7.92E-40	up	5778	5746	6909	1256	1628	1053	ADARB1
1.266538	4.98E-20	2.41E-18	up	20841	####	####	8167	####	9858	VIM
2.252103	0.0004567	0.002152	up	20	17	43	7	5	5	
1.341123	2.78E-05	0.000179	up	111	85	168	51	48	48	CDKL1

1.019752	0.000909	0.003948	up	441	549	505	346	238	169	PROSER2
1.161113	2.14E-12	4.62E-11	up	3232	3509	4178	1666	1966	1458	GOLGA3
3.194822	3.48E-20	1.72E-18	up	640	529	1199	119	84	56	ZEB1
1.627064	0.0003506	0.001706	up	55	66	74	7	39	23	LAG3
1.301773	0.0036577	0.01305	up	67	91	65	14	30	52	MST1
3.246266	1.10E-11	2.15E-10	up	66	52	63	6	7	7	SCN4A
1.764043	0.0180602	0.049953	up	19	18	13	9	3	3	TNFRSF10C
1.760559	6.98E-20	3.32E-18	up	771	760	944	195	274	295	VTI1B
1.463621	1.99E-06	1.65E-05	up	727	494	954	343	211	235	MYO5A
1.137781	0.0004086	0.001953	up	1174	842	2024	790	523	513	NIN
1.032594	8.17E-07	7.42E-06	up	653	670	1061	352	426	425	CEP83
1.479998	4.57E-17	1.69E-15	up	2420	2256	3290	963	1110	886	SIPA1L1
1.605628	9.98E-06	7.09E-05	up	80	65	115	34	25	28	
2.497322	3.93E-13	9.32E-12	up	118	138	108	25	27	16	SOCS2-AS1
2.471031	5.73E-11	1.01E-09	up	79	87	98	20	13	16	
1.12389	2.45E-05	0.00016	up	221	350	239	118	143	131	HOMER3
2.492157	2.97E-35	4.15E-33	up	1101	964	985	136	249	194	EFNB1
2.313364	0.0052301	0.017693	up	274	192	337	105	37	15	MFAP5
2.353123	5.04E-16	1.66E-14	up	162	189	251	46	38	37	HIST1H2BJ
1.19559	2.81E-10	4.52E-09	up	441	410	492	155	268	199	MED20
11.26285	5.35E-21	2.92E-19	up	382	403	522	0	0	0	VWA5A
1.180282	0.0002053	0.001066	up	134	135	202	89	77	47	ZNF615
1.189648	5.29E-07	4.97E-06	up	3939	4161	2750	1256	2019	1826	PYCR1
5.163399	0.0014946	0.006104	up	7	5	7	0	0	0	ADGRG2
1.185677	5.05E-10	7.84E-09	up	375	336	368	142	197	161	AARS2
1.160671	7.14E-07	6.57E-06	up	490	492	622	151	329	284	DHRS7
1.586065	1.20E-09	1.76E-08	up	3398	3266	6516	1643	1563	1249	HIF1A
2.082688	0.0158215	0.044758	up	9	15	12	1	3	5	RGPD6
1.266041	7.34E-11	1.28E-09	up	1170	1303	1334	381	751	562	LGMN
1.096455	0.0009817	0.00423	up	64	62	74	25	35	38	
4.032505	4.35E-54	1.56E-51	up	374	374	403	24	30	20	IRAK2
1.06964	0.0005985	0.002736	up	90	100	101	30	72	47	CAMK1
2.508556	1.11E-08	1.39E-07	up	60	80	64	16	9	12	HTR7
1.335329	0.0100804	0.03088	up	122	99	138	90	33	17	ANKRD1
1.059529	1.02E-07	1.10E-06	up	603	607	560	222	356	324	TMED1
1.369618	1.11E-07	1.18E-06	up	4298	4343	4067	2138	1705	1222	LOXL2
3.368771	4.56E-63	2.01E-60	up	698	846	929	70	95	86	PMP22
1.769108	2.60E-22	1.57E-20	up	8624	8146	7702	2025	2713	2825	AARS
1.35141	1.16E-08	1.45E-07	up	820	847	1137	441	348	330	NRP1
2.371096	6.10E-32	7.53E-30	up	1155	1116	1696	244	267	279	FAM114A1
1.44261	5.91E-10	9.13E-09	up	2008	2080	3269	1025	875	849	AGFG1
1.911443	1.08E-18	4.67E-17	up	2829	2618	3148	907	846	606	PARVA
1.799608	2.79E-22	1.68E-20	up	3645	3608	3173	876	1138	1143	TMEM109
1.302004	9.85E-09	1.25E-07	up	672	829	930	385	360	272	ALDOC

1.434988	5.93E-10	9.15E-09	up	219	255	241	69	111	100	HOXC6
1.064474	0.0007356	0.003286	up	375	350	695	247	167	264	CEP57L1
1.099489	2.76E-06	2.22E-05	up	310	354	404	175	152	185	TXNRD3
2.504984	4.01E-05	0.00025	up	29	37	23	3	9	5	C2CD4C
4.734885	0.0077313	0.024645	up	7	3	4	0	0	0	
2.983625	6.65E-09	8.72E-08	up	60	55	40	4	9	8	LOC100506691
1.265718	3.61E-09	4.93E-08	up	1379	1231	2021	694	739	550	TNC
1.238474	1.32E-07	1.39E-06	up	532	500	745	208	394	200	SLC9A7
5.430071	0.0005417	0.002503	up	5	8	10	0	0	0	
1.364444	1.44E-08	1.77E-07	up	1133	992	2001	535	609	499	POLQ
1.909199	0.0013435	0.005554	up	47	50	23	3	15	17	DLL3
1.438667	1.35E-10	2.25E-09	up	409	517	696	193	211	213	KATNBL1
1.399827	2.28E-08	2.73E-07	up	169	188	193	52	84	84	DPH6
2.491	5.25E-15	1.60E-13	up	2865	2501	4730	834	486	463	HEG1
5.900665	1.56E-05	0.000107	up	11	17	35	0	1	0	PTPN22
1.563109	0.0022736	0.008749	up	24	31	46	11	18	7	STX1B
1.117577	2.02E-08	2.43E-07	up	1058	1083	987	375	574	578	PSRC1
1.005302	7.10E-11	1.25E-09	up	1218	1108	1219	538	719	601	CEP164
1.26577	2.02E-07	2.05E-06	up	657	575	1005	350	310	287	ADAM12
1.285505	1.00E-14	2.94E-13	up	1333	1258	1321	439	718	549	ITPRIP
1.064032	0.0001821	0.000957	up	224	156	289	113	96	117	ZNF181
1.300807	0.0018309	0.007294	up	384	455	839	365	135	160	SGTB
6.124415	1.73E-05	0.000117	up	11	12	14	0	0	0	MAGEB2
1.683275	0.0032988	0.011969	up	89	98	101	61	18	9	DHRS2
1.537149	2.85E-10	4.58E-09	up	1312	1242	951	323	439	516	PCK2
1.989607	1.61E-14	4.63E-13	up	444	397	728	121	127	156	RUNX2
1.317699	7.10E-06	5.23E-05	up	566	556	1083	362	277	250	TRIP11
2.871102	0.0003797	0.001828	up	20	42	44	12	1	1	CCBE1
1.065866	1.12E-06	9.88E-06	up	213	183	240	98	124	96	
3.143727	0.0134254	0.039121	up	16	5	5	2	0	1	
5.514943	0.0003646	0.001764	up	7	10	7	0	0	0	
1.350428	1.62E-08	1.99E-07	up	151	173	191	64	84	64	ZNF823
4.430474	1.20E-20	6.18E-19	up	147	101	132	3	13	3	ZNF311
2.891994	3.89E-27	3.52E-25	up	226	233	255	37	33	30	FST
3.751501	0.0060879	0.020101	up	6	9	12	2	0	0	SAA2
3.713447	0.0064258	0.021056	up	8	9	9	2	0	0	
4.144014	5.38E-06	4.07E-05	up	25	19	24	2	1	1	GALNT18
1.165672	1.10E-09	1.62E-08	up	13591	####	####	7724	7291	6331	CD44
1.01882	0.0011222	0.004752	up	140	147	92	52	79	69	FHL3
2.371213	3.25E-17	1.22E-15	up	167	184	182	24	51	35	MYRF
6.694328	1.24E-06	1.08E-05	up	15	26	13	0	0	0	KCNG1
6.366606	1.00E-08	1.27E-07	up	60	58	39	2	0	0	LOC100506178
1.426107	2.79E-10	4.49E-09	up	205	253	304	88	112	96	HIST1H2BD
1.161425	7.66E-11	1.33E-09	up	1107	1215	1068	475	649	481	HIST1H2BK

4.579365	9.68E-09	1.23E-07	up	41	32	40	0	2	3	CPE
1.431568	8.42E-06	6.08E-05	up	113	84	112	42	34	42	MEX3B
1.499976	4.46E-05	0.000274	up	78	87	161	46	45	27	GAB1
1.26846	3.73E-11	6.75E-10	up	1112	1183	1595	484	564	622	ZNF330
1.305898	6.14E-15	1.86E-13	up	1780	1840	2452	769	909	869	ARFGAP3
3.125606	1.48E-05	0.000102	up	27	23	23	1	5	3	LYRM9
4.726708	0.0018706	0.007425	up	8	4	16	1	0	0	
1.230681	0.0173341	0.048261	up	33	46	58	29	24	7	ANXA10
1.108943	7.81E-05	0.000453	up	250	224	366	150	109	135	MBTPS2
1.616351	0.0015639	0.006339	up	43	39	30	6	14	19	
4.574398	0.0141055	0.040753	up	3	2	8	0	0	0	
2.226606	4.48E-05	0.000275	up	33	55	37	13	11	4	GPR1
2.393543	0.0002382	0.001218	up	24	37	20	8	4	4	HOXB8
1.080924	3.83E-08	4.38E-07	up	1412	1349	2024	751	770	802	GTF3C3
1.780975	1.24E-20	6.40E-19	up	1092	1210	1030	266	425	342	UPP1
4.383176	3.62E-06	2.84E-05	up	23	22	32	0	4	0	NAALADL1
2.27345	2.94E-12	6.24E-11	up	120	124	120	29	22	27	FAM101B
1.048337	5.70E-08	6.37E-07	up	987	930	1411	523	570	565	WDR11
5.555379	0.000284	0.001422	up	7	8	10	0	0	0	CPZ
2.729313	1.04E-28	1.05E-26	up	305	327	300	36	59	54	HCN2
1.645001	4.90E-18	2.00E-16	up	1514	1598	1442	516	551	456	GADD45B
1.181732	0.000292	0.001457	up	68	60	81	25	40	32	NPHP1
1.325888	3.70E-07	3.58E-06	up	354	343	245	100	173	131	MRPL54
2.507591	9.32E-14	2.41E-12	up	139	100	208	22	35	25	ZSCAN12
1.015761	1.78E-06	1.50E-05	up	11609	####	8484	4711	7118	5046	SLC3A2
1.455005	1.87E-06	1.57E-05	up	474	386	295	84	216	160	VASN
1.120435	7.57E-06	5.54E-05	up	2632	2762	1742	866	1356	1295	RNF187
2.804242	3.70E-27	3.36E-25	up	300	259	370	47	41	49	FHOD3
1.134225	0.0023878	0.009098	up	432	441	705	364	147	194	RNF138
1.701172	4.53E-09	6.11E-08	up	2890	2567	3805	1285	739	824	KLHL5
1.655673	4.64E-14	1.26E-12	up	1164	957	1697	421	435	387	PPFIBP1
3.983905	0.0154695	0.043985	up	6	7	3	1	0	0	CRYAB
2.121332	2.72E-07	2.69E-06	up	53	57	55	10	15	15	DERL3
1.920231	0.0168486	0.047096	up	14	7	23	2	4	6	CKLF
1.533146	0.0024474	0.009275	up	43	53	34	14	28	7	GGT5
1.472734	1.77E-08	2.16E-07	up	297	379	538	142	135	170	TRIM52
2.795529	3.26E-38	5.58E-36	up	472	560	497	65	93	75	FAHD2B
3.004834	6.10E-29	6.27E-27	up	202	224	266	23	40	28	LHFP
1.162611	1.17E-06	1.02E-05	up	328	345	500	130	196	220	PXK
5.357837	0.0006182	0.002815	up	32	6	3	1	0	0	AFF3
1.876498	0.0010421	0.00446	up	51	54	23	5	23	11	PIGZ
1.541774	0.0043516	0.015136	up	56	41	73	35	15	8	CD274
3.279313	4.93E-06	3.77E-05	up	32	36	24	1	2	7	GGACT
1.183825	1.30E-06	1.13E-05	up	1802	1895	3641	1075	1226	1006	CAPRIN2

1.478302	1.31E-14	3.81E-13	up	745	805	837	211	392	310	ASIC1
1.462398	3.53E-10	5.59E-09	up	536	516	501	140	207	247	COQ5
1.029941	2.56E-12	5.50E-11	up	4508	4558	5354	2264	2635	2451	STT3A
1.583101	3.78E-10	5.96E-09	up	1523	1199	2171	649	567	446	ARHGAP32
1.195492	2.68E-17	1.02E-15	up	76861	####	####	35197	####	####	HSPA8
1.014481	1.08E-13	2.76E-12	up	2977	2992	3310	1403	1874	1549	ZPR1
1.033573	5.37E-08	6.03E-07	up	745	617	672	279	391	377	CSRNP2
1.237838	2.43E-05	0.000159	up	779	666	1374	486	350	361	WDFY1
3.34036	0.0050872	0.017277	up	6	10	13	0	1	2	
4.74659	2.38E-06	1.94E-05	up	20	24	32	0	2	1	C14orf132
1.686239	9.05E-18	3.59E-16	up	1871	1513	2268	629	658	527	KIRREL
1.772339	1.72E-07	1.77E-06	up	158	152	272	66	41	64	ZNF697
4.644136	6.64E-28	6.31E-26	up	157	151	170	4	5	11	IFT46
1.37156	2.69E-10	4.34E-09	up	680	454	779	212	303	258	ZNF618
1.682181	1.10E-08	1.38E-07	up	1260	1112	2341	604	454	414	TMTC1
2.875462	9.80E-11	1.68E-09	up	51	78	78	7	15	8	CCL2
2.440781	0.001976	0.007792	up	10	24	24	2	2	7	LRRIQ1
1.987509	9.50E-21	5.00E-19	up	577	580	602	128	151	184	C17orf75
1.25016	0.0015898	0.006431	up	88	59	70	24	55	20	KRBA1
1.048623	4.42E-11	7.92E-10	up	2692	2841	3714	1447	1759	1444	RNF216
1.012408	3.47E-09	4.75E-08	up	607	623	634	282	359	329	ANO8
4.525076	9.63E-06	6.87E-05	up	16	21	31	2	0	1	NAT16
7.253464	3.84E-08	4.39E-07	up	21	36	23	0	0	0	GPRC5B
1.029803	8.54E-07	7.73E-06	up	910	971	1559	458	710	591	WDR19
2.243717	3.58E-05	0.000225	up	56	49	36	3	20	10	DPF1
5.185637	0.0025455	0.009588	up	26	10	0	1	0	0	SLC34A2
2.005171	6.75E-13	1.56E-11	up	190	139	211	34	53	54	SYT7
2.367514	0.0004402	0.002083	up	17	22	29	4	8	2	ITGA10
1.569295	3.97E-13	9.37E-12	up	731	572	866	254	241	256	TUBE1
1.866126	5.14E-30	5.75E-28	up	1088	1119	1252	315	362	313	CNTNAP1
1.269066	1.06E-11	2.07E-10	up	1640	1549	2150	777	773	730	SMURF2
2.262662	7.14E-23	4.64E-21	up	2167	2073	2897	595	547	385	MICAL2
1.071447	7.52E-14	1.98E-12	up	6266	6780	7544	2787	4302	3272	LRRC59
1.890522	1.93E-08	2.33E-07	up	195	237	138	38	85	45	TMEM92
1.117274	6.89E-13	1.59E-11	up	3953	4263	5603	1937	2559	2150	GOLGA2
4.899106	1.50E-41	2.88E-39	up	258	298	256	6	14	9	CERCAM
2.607471	3.41E-48	9.68E-46	up	3546	3551	3382	445	777	614	SEMA3F
1.465381	1.86E-13	4.59E-12	up	864	737	944	333	315	303	TBC1D16
2.022243	0.0050092	0.017055	up	21	16	13	3	4	6	FTCDNL1
1.676681	3.98E-16	1.33E-14	up	2753	3063	2764	628	1274	984	PLAUR
1.169348	0.0003104	0.001535	up	173	156	287	119	84	73	B4GALT6
1.170154	1.10E-08	1.39E-07	up	1614	1669	1273	647	854	649	QPCTL
1.289089	5.86E-10	9.06E-09	up	527	499	807	231	283	262	CREB1
1.245748	4.15E-09	5.61E-08	up	2053	1926	1626	613	959	943	MEN1

1.368417	4.98E-06	3.80E-05	up	1008	859	1771	596	417	396	FMNL2
2.433869	1.02E-09	1.51E-08	up	90	71	156	24	22	14	SLC16A6
1.230867	8.93E-11	1.54E-09	up	860	813	844	306	391	427	DPH1
1.280947	2.74E-13	6.61E-12	up	2201	2371	3024	893	1155	1205	THAP9-AS1
1.156136	1.23E-06	1.07E-05	up	1265	1104	1453	700	526	520	C16orf72
1.750964	3.83E-05	0.00024	up	48	42	45	14	15	13	ZNF280B
2.186832	0.0025727	0.009675	up	22	22	14	3	9	2	
2.074593	6.06E-05	0.000361	up	41	39	27	7	10	10	IGF2
3.942465	0.0001169	0.000646	up	23	26	8	2	2	0	ADGRG3
3.564424	1.09E-27	1.02E-25	up	271	300	248	18	42	15	GALNT9
1.065062	2.27E-11	4.21E-10	up	7019	6926	9129	3712	4223	3505	MCL1
1.135223	2.01E-09	2.85E-08	up	959	1038	1003	344	587	521	TARS2
3.451244	5.81E-48	1.62E-45	up	601	631	625	36	77	68	GNA15
1.900452	0.0059793	0.019811	up	21	18	13	3	7	5	RGS7
3.782794	0.0016571	0.006674	up	20	6	14	0	0	3	TMEM92-AS1
1.026658	6.92E-13	1.59E-11	up	5637	6223	6283	2649	3744	3006	SRPR
5.843611	0.0001024	0.000575	up	15	8	7	0	0	0	
1.144951	9.41E-06	6.73E-05	up	1112	1157	881	299	687	559	PPP2R3B
2.421788	0.0001565	0.000837	up	46	24	34	4	3	13	TMEM159
7.63864	2.33E-09	3.25E-08	up	28	35	43	0	0	0	LINC00668
7.312003	2.71E-08	3.20E-07	up	35	28	20	0	0	0	GACAT2
1.229694	7.14E-09	9.33E-08	up	701	513	650	283	316	231	
5.08136	4.02E-24	2.92E-22	up	146	150	117	3	6	4	PWP2
2.00714	0.0100374	0.030783	up	12	14	27	8	2	3	VASH2
1.270972	2.40E-06	1.96E-05	up	224	222	406	107	121	134	DYRK3
2.790121	3.56E-09	4.87E-08	up	72	59	167	18	11	14	SYT14
1.500685	0.0096113	0.029644	up	20	20	37	6	8	14	FAM63A
2.480881	0.0003634	0.00176	up	25	21	19	5	2	5	NKAIN1
2.432527	1.22E-37	1.97E-35	up	980	988	1310	180	285	176	SGK1
4.928249	0.0042473	0.014817	up	3	8	5	0	0	0	TCF21
1.654134	3.83E-07	3.70E-06	up	1155	1095	1738	575	495	223	TNFAIP3
1.541445	3.50E-23	2.36E-21	up	1274	1319	1364	403	586	448	RAB32
1.969063	0.0002097	0.001085	up	46	70	27	7	19	14	
1.231601	3.17E-19	1.43E-17	up	23433	####	####	10101	####	####	TPM4
2.596451	0.0117032	0.034955	up	13	7	9	1	1	3	LINC00923
1.450398	0.0008785	0.003828	up	40	69	50	12	32	19	EFNA3
1.596247	4.97E-09	6.65E-08	up	665	639	1161	323	250	249	CDK17
1.495142	1.92E-10	3.16E-09	up	287	268	270	78	109	121	SNAPIN
1.15796	2.05E-09	2.90E-08	up	1402	1469	1447	542	971	563	MVD
4.218714	4.50E-98	5.02E-95	up	1226	1242	1302	60	72	80	TUBA1A
1.767904	3.66E-06	2.87E-05	up	49	72	90	21	21	22	
3.729911	7.52E-05	0.000437	up	12	19	35	1	0	4	LVCAT1
1.590945	7.72E-10	1.17E-08	up	19615	####	####	8275	####	####	MALAT1
6.456839	4.83E-09	6.49E-08	up	46	54	66	0	2	0	

7.325203	8.25E-18	3.29E-16	up	2712	2966	4051	42	8	8	SLC2A3
2.000113	9.38E-13	2.13E-11	up	468	452	751	153	193	89	DAB2
1.327662	3.46E-13	8.23E-12	up	1398	1571	2115	624	745	729	CWC27
1.502131	2.95E-08	3.45E-07	up	3600	2854	5152	1733	1289	1109	TJP1
1.150556	0.0001731	0.000915	up	385	353	779	268	213	205	LYST
1.50901	3.29E-13	7.86E-12	up	1715	1598	2412	733	685	641	NFYA
3.355711	1.14E-23	7.90E-22	up	210	195	246	19	38	11	SCCPDH
2.924586	4.31E-39	7.72E-37	up	12376	####	####	1977	1656	1358	AXL
1.145142	1.24E-07	1.31E-06	up	5848	5551	8755	3224	2880	3171	ZNF146
2.236124	1.27E-07	1.34E-06	up	61	72	59	9	14	20	DNAAF3
1.905728	5.17E-12	1.06E-10	up	443	397	350	63	152	128	SEMA6B
1.385644	2.19E-09	3.07E-08	up	249	275	240	79	135	98	FOXD1
1.182475	3.35E-12	7.06E-11	up	2078	2123	1918	774	1142	945	DEAF1
1.33561	0.0003004	0.001494	up	155	95	252	84	63	53	ENTPD1-AS1
1.566428	0.0060971	0.020125	up	39	24	18	10	11	8	RGPD3
2.561372	2.70E-44	5.89E-42	up	1153	1273	1636	189	274	257	BMP6
2.862591	7.73E-08	8.44E-07	up	42	34	66	6	5	9	CLGN
2.226369	1.28E-17	5.03E-16	up	1740	1669	2405	524	353	376	BCAT1
1.332287	6.21E-11	1.10E-09	up	1279	1138	1793	567	568	580	GCLC
1.492936	1.46E-11	2.81E-10	up	1529	1692	1235	507	725	460	BCL3
1.428117	8.49E-09	1.10E-07	up	155	188	192	55	93	63	PDGFRL
2.486265	0.000862	0.003764	up	13	21	19	2	4	4	LOC105374366
3.071505	0.0003627	0.001757	up	10	22	52	6	4	0	
1.356402	4.16E-07	4.00E-06	up	1787	1626	3249	977	795	847	RPS6KA3
1.361881	1.65E-07	1.70E-06	up	157	133	162	47	85	55	TPST2
1.038463	3.42E-06	2.70E-05	up	985	932	1521	616	586	509	RBMS1
1.238192	3.18E-07	3.11E-06	up	747	753	642	198	410	368	ICT1
1.024967	2.59E-08	3.07E-07	up	585	587	787	302	349	347	VPS54
1.247393	1.89E-16	6.60E-15	up	3420	3480	3812	1295	1754	1686	SRP68
1.085619	3.75E-07	3.63E-06	up	522	542	586	180	384	270	PLAT
1.607062	9.52E-13	2.16E-11	up	2075	2059	3445	907	886	749	ELL2
2.072408	6.18E-05	0.000367	up	26	33	41	7	11	7	CPQ
1.494063	0.0006912	0.003107	up	58	63	42	25	24	12	WFDC21P
2.320574	6.04E-31	7.04E-29	up	855	856	1147	208	216	168	WIPI1
2.05226	4.70E-14	1.28E-12	up	369	321	304	49	108	100	ABCA3
2.226554	8.27E-10	1.25E-08	up	1925	1340	3178	676	386	298	ZBTB38
1.492165	1.79E-08	2.18E-07	up	571	567	440	141	198	254	RAB3IL1
1.027107	0.0022812	0.008771	up	158	177	332	134	88	105	ZC2HC1A
3.732059	1.30E-14	3.76E-13	up	77	57	110	7	7	5	IL7
3.369677	1.78E-23	1.23E-21	up	209	224	211	29	15	20	PTPRH
1.566175	5.73E-11	1.01E-09	up	9289	8783	####	4249	3565	3549	CALU
1.615186	1.96E-09	2.78E-08	up	319	315	626	112	180	136	STRIP2
1.338302	3.71E-07	3.59E-06	up	4292	4387	8224	2567	2497	1741	SQLE
3.583585	9.62E-21	5.04E-19	up	400	317	688	60	35	22	DOCK4

1.890419	2.34E-05	0.000154	up	58	54	128	29	20	16	U2AF1L5
4.53715	0.0129035	0.037815	up	18	5	0	1	0	0	RUNX1T1
1.064565	0.0020023	0.007877	up	1057	979	940	736	392	307	JUN
1.679054	9.05E-09	1.16E-07	up	295	307	482	138	92	111	OSBPL6
1.984032	5.73E-05	0.000343	up	28	50	52	14	12	8	GAD1
2.04953	4.70E-11	8.40E-10	up	577	561	386	74	146	175	R3HCC1
1.43612	1.76E-11	3.33E-10	up	1255	1215	1935	568	551	546	ERI1
2.516011	0.0127332	0.03742	up	8	9	17	4	2	0	
1.079908	0.0001698	0.000899	up	924	1355	1216	672	719	339	DUSP5
7.300982	2.73E-08	3.22E-07	up	37	21	25	0	0	0	LBX1
1.245807	0.0002406	0.001227	up	493	522	955	382	205	234	TWSG1
1.650326	6.24E-10	9.58E-09	up	946	978	1306	439	287	312	CNKSR3
3.32164	2.64E-26	2.22E-24	up	174	191	208	23	24	13	LURAP1L
1.004011	0.0035913	0.012856	up	4718	3532	7537	3822	2091	1858	TEAD1
4.088628	3.47E-18	1.43E-16	up	94	88	155	4	13	4	TNS1
2.280278	1.28E-27	1.19E-25	up	2129	2188	1812	317	542	489	AAAS
1.13323	1.71E-07	1.75E-06	up	434	524	448	228	257	188	RAPGEF3
3.909224	0.0050032	0.017038	up	12	9	6	0	2	0	
3.50997	0.0111691	0.033594	up	9	8	5	1	0	1	ZNF620
4.735235	3.94E-08	4.49E-07	up	38	26	67	0	0	5	ZNF619
1.304488	6.85E-14	1.81E-12	up	3063	3777	4547	1418	1728	1645	AP3S1
1.145127	0.0001748	0.000922	up	188	175	133	52	120	72	BOLA3
5.536085	1.49E-43	3.21E-41	up	250	318	411	4	7	11	WNT5A
1.890688	1.79E-14	5.09E-13	up	1096	1082	1451	407	352	244	CDC42EP3
1.540801	7.92E-09	1.03E-07	up	161	154	219	69	63	57	LYPD6
2.774119	4.39E-15	1.35E-13	up	97	90	106	12	17	16	FBXO36
1.111139	0.0003333	0.001634	up	547	497	1129	405	324	280	TTBK2
5.737397	5.82E-10	9.01E-09	up	52	42	59	1	1	1	PID1
1.424967	9.81E-09	1.25E-07	up	930	893	623	284	431	264	RELB
1.056562	1.82E-09	2.59E-08	up	1745	1692	1509	725	995	799	ERCC2
1.919066	0.001174	0.00495	up	31	19	27	7	5	9	DLL4
6.63599	1.04E-09	1.54E-08	up	53	69	69	2	0	0	NOVA2
1.306101	3.24E-15	1.01E-13	up	10325	####	####	4562	6400	4782	FDFT1
3.734589	1.14E-34	1.50E-32	up	736	632	1265	84	58	57	OPHN1
1.089675	2.78E-09	3.84E-08	up	2826	2946	2498	1299	1512	1275	BET1L
3.085475	9.14E-06	6.54E-05	up	24	23	35	5	4	1	COL6A3
1.321972	5.13E-05	0.000311	up	105	90	189	50	52	55	GCNT1
2.011094	1.87E-21	1.07E-19	up	568	559	751	167	192	126	C17orf51
3.785539	0.0010228	0.004386	up	10	7	25	2	0	1	SEMA3D
3.165217	4.49E-12	9.23E-11	up	182	194	347	48	17	14	AOX1
1.32372	2.64E-05	0.000171	up	390	352	674	249	165	152	PAQR3
2.647332	7.90E-37	1.20E-34	up	1143	1102	1608	222	245	170	ANTXR2
1.185781	0.0001929	0.001009	up	364	282	755	223	209	189	DYNC2H1
3.302352	3.16E-12	6.68E-11	up	69	50	97	5	11	7	HS3ST3A1

4.388527	6.04E-35	8.13E-33	up	3016	2721	4433	252	147	86	MYPN
1.493418	0.0001274	0.000698	up	60	74	133	39	30	27	CACNA2D1
6.522433	2.63E-06	2.13E-05	up	10	18	21	0	0	0	ZNF365
1.037897	0.0025884	0.009726	up	823	855	1506	746	373	409	ACAP2
4.365403	2.71E-05	0.000175	up	26	16	18	2	0	1	ANKFN1
1.016792	9.20E-05	0.000523	up	479	556	348	180	298	256	SNAPC2
2.160137	7.56E-22	4.46E-20	up	491	430	428	76	144	103	IL27RA
3.757265	1.71E-18	7.25E-17	up	116	102	210	13	7	12	MID2
1.24648	1.83E-07	1.87E-06	up	446	551	382	160	263	200	WDR54
2.618579	4.42E-26	3.62E-24	up	406	453	471	47	115	71	IGFBP7
4.819295	3.96E-35	5.45E-33	up	245	215	395	15	10	6	IGFN1
2.729893	0.0051942	0.017594	up	13	20	11	0	1	6	PCDHB13
1.069379	0.0003731	0.001801	up	3960	3524	8043	3007	2362	2037	SSFA2
1.543816	7.73E-06	5.65E-05	up	1048	982	1941	605	291	442	GNB4
2.089356	1.87E-06	1.57E-05	up	53	38	70	14	13	12	HECW2
1.178296	1.84E-08	2.23E-07	up	350	344	486	179	210	153	GBE1
1.418214	3.23E-06	2.56E-05	up	2091	1770	4059	1215	886	856	BBX
3.165301	0.0095293	0.02945	up	9	6	10	0	2	1	
3.218165	0.0002293	0.001176	up	11	26	16	2	2	2	
1.627733	6.00E-05	0.000358	up	67	64	54	12	34	19	LINC00847
2.232718	1.10E-11	2.14E-10	up	176	154	232	19	62	47	
1.105308	9.82E-09	1.25E-07	up	1195	1456	1157	517	737	624	STX5
1.096185	1.84E-08	2.23E-07	up	597	654	700	238	355	367	SLC22A23
1.171991	3.76E-11	6.78E-10	up	707	793	751	279	429	351	FOXF2
1.71992	0.0098894	0.030407	up	16	14	32	6	4	9	
1.671631	0.0127358	0.037422	up	19	14	31	9	3	8	IKZF2
7.152257	4.13E-08	4.69E-07	up	80	36	28	0	1	0	PCDHB15
1.909459	2.10E-09	2.96E-08	up	124	132	117	21	52	34	FRS3
5.922488	0.0009578	0.004137	up	28	2	1	0	0	0	PCDHB6
5.71319	0.0002827	0.001417	up	36	15	1	0	1	0	PCDHB7
2.213262	4.54E-12	9.33E-11	up	267	261	170	33	72	58	STYXL1
1.45606	1.49E-07	1.55E-06	up	1374	1315	2835	683	630	718	CEP152
1.419484	5.53E-18	2.23E-16	up	1655	1728	2071	538	871	743	PNMA1
1.215873	1.02E-06	9.01E-06	up	430	468	379	124	240	225	FBXO17
3.909228	1.27E-22	7.96E-21	up	176	171	196	10	25	4	C8orf4
1.11587	1.83E-09	2.61E-08	up	1759	1982	1661	688	1114	859	ZFPL1
2.770515	5.60E-30	6.18E-28	up	855	812	1106	89	153	184	AGK
2.288249	0.0009713	0.00419	up	24	17	20	3	3	7	
1.161224	8.59E-07	7.77E-06	up	433	454	331	154	244	185	CA5BP1
1.682947	2.64E-12	5.63E-11	up	272	287	343	63	119	115	BACE1
1.432951	8.07E-07	7.35E-06	up	475	442	877	268	227	177	ARRDC3
1.885705	0.0001713	0.000906	up	36	35	60	5	21	12	NPR3
4.167195	2.06E-45	4.78E-43	up	314	287	381	14	18	25	PGBD1
1.442148	1.47E-11	2.82E-10	up	6285	6869	6488	1616	3175	2939	FLOT1

1.744685	3.44E-10	5.46E-09	up	857	858	1487	380	266	313	POLR3G
1.496147	3.65E-08	4.20E-07	up	259	310	277	60	146	117	ZSCAN16-AS1
1.183486	6.84E-10	1.04E-08	up	1404	1538	1948	779	773	667	FOXC1
2.355172	4.77E-19	2.12E-17	up	168	188	229	36	41	42	PPAP2B
2.443033	7.78E-16	2.51E-14	up	393	377	744	110	81	89	KDM7A
5.264767	2.39E-05	0.000157	up	31	32	8	1	1	0	ITM2A
1.140567	0.0013376	0.005533	up	47	64	66	27	32	25	LOC101930370
1.148573	2.48E-07	2.47E-06	up	486	413	729	229	268	260	ZNF197
1.418745	0.0120298	0.035805	up	25	17	62	9	16	15	MIR1208
1.38075	0.001545	0.006272	up	42	42	43	9	22	21	LOC101927793
1.367396	0.0010855	0.004611	up	45	74	51	24	31	15	
1.572441	1.56E-06	1.33E-05	up	98	117	120	23	44	52	
3.100469	3.74E-11	6.76E-10	up	57	56	121	9	10	9	
1.651391	1.68E-09	2.41E-08	up	201	158	220	73	63	54	LINC00941
1.827608	0.0004665	0.002192	up	22	41	41	6	13	12	CYP4X1
1.302935	1.06E-07	1.13E-06	up	275	243	356	114	110	140	ANKRD42
1.514548	1.43E-18	6.12E-17	up	1402	1420	1916	550	621	547	RAD1
1.02611	9.95E-11	1.70E-09	up	4256	4742	5695	1952	3087	2567	BRIX1
2.653593	2.50E-14	7.03E-13	up	6608	6028	####	1859	922	830	LIMCH1
1.630006	2.01E-10	3.30E-09	up	542	509	905	241	215	187	RAB30
1.05304	3.48E-08	4.02E-07	up	612	480	579	251	302	289	CCDC90B
2.777212	4.71E-43	9.94E-41	up	1459	1142	1363	143	252	219	SDC3
1.087125	6.51E-05	0.000384	up	269	250	446	173	145	143	
1.247867	1.70E-09	2.45E-08	up	351	388	383	124	191	184	CAMK2N1
10.78584	3.11E-19	1.41E-17	up	274	269	399	0	0	0	CYP4F8
1.31307	7.45E-09	9.68E-08	up	3943	4206	7476	2078	2534	1869	INSIG1
1.15617	0.0018596	0.007391	up	143	177	285	131	72	67	CCPG1
2.735758	2.07E-06	1.71E-05	up	28	50	34	5	9	4	LOC100996419
2.774793	0.0077269	0.024635	up	14	12	16	0	7	0	PNMA6A
1.725778	0.0007293	0.003261	up	29	26	34	10	10	8	SULF1
2.655458	2.84E-11	5.23E-10	up	143	114	167	36	18	14	DNAH5
1.265554	2.30E-10	3.73E-09	up	1290	1379	1124	425	721	544	SPATA20
3.127095	1.28E-10	2.14E-09	up	56	44	85	7	9	6	NEXN
1.478054	4.57E-13	1.07E-11	up	702	719	1067	309	338	275	TYW3
1.114747	0.0018316	0.007295	up	1466	1442	2541	1259	564	644	SEC24A
1.267049	6.87E-05	0.000402	up	238	222	409	158	116	90	TTC33
1.058505	4.17E-05	0.000258	up	248	271	235	85	142	157	SPA17
1.117659	0.0033916	0.012255	up	74	64	68	23	28	48	KTN1-AS1
3.572686	2.55E-21	1.44E-19	up	211	240	252	31	22	8	PDE2A
1.153353	0.0002127	0.001099	up	87	99	88	29	53	49	
1.076778	3.70E-08	4.25E-07	up	1157	1203	1450	529	607	736	KLF11
1.097106	5.22E-11	9.27E-10	up	3845	3839	5612	1838	2495	2142	UBE2H
1.769643	6.72E-17	2.43E-15	up	709	834	1091	197	303	308	HDAC8
3.26926	3.97E-105	########	up	4064	3782	4923	383	550	457	SH3KBP1

2.812736	3.49E-08	4.03E-07	up	43	38	80	6	12	6	ST3GAL6
4.747167	0.007549	0.024149	up	7	4	3	0	0	0	NLRP3
1.972499	4.95E-38	8.33E-36	up	1925	1841	2007	442	576	526	ZNF496
1.219886	4.74E-05	0.000289	up	239	297	328	65	202	135	CASP1
1.801284	0.0001202	0.000663	up	197	131	201	91	29	29	DDR2
1.264322	5.92E-16	1.92E-14	up	2999	3332	3592	1372	1560	1372	PEA15
1.073919	4.22E-07	4.05E-06	up	415	482	414	205	221	225	BNIP1
1.808292	5.14E-11	9.15E-10	up	1324	1237	902	207	445	417	TMEM132A
1.203848	9.86E-12	1.93E-10	up	2868	2638	2537	938	1547	1238	DBN1
1.096767	7.11E-06	5.24E-05	up	715	775	1153	462	370	421	RBM15
2.889007	0.0011284	0.004773	up	15	11	17	3	2	1	SLFN12
1.554924	0.0001045	0.000585	up	45	58	55	12	23	22	SPIN2B
1.195833	7.61E-06	5.57E-05	up	247	266	210	93	163	84	
1.978799	2.94E-10	4.71E-09	up	528	485	826	213	125	128	IL1RAP
2.015969	1.81E-14	5.14E-13	up	233	268	241	41	91	65	UBE2D4
1.544345	0.0123639	0.036544	up	29	36	16	4	11	15	CHST7
1.462922	1.97E-12	4.29E-11	up	6407	6551	5142	1699	2823	2502	PKN1
2.186353	0.0120476	0.035843	up	12	11	12	4	3	1	SLC16A2
1.347345	3.13E-06	2.50E-05	up	304	248	469	137	111	158	ALKBH8
1.678376	1.82E-09	2.60E-08	up	457	469	514	79	210	194	DNMT3B
1.291022	2.19E-10	3.56E-09	up	446	496	461	149	265	198	OSBPL7
1.070261	0.0065133	0.021313	up	40	50	84	28	36	22	SEMA6D
1.328008	0.0001273	0.000698	up	353	363	209	66	184	154	C19orf60
3.184783	2.20E-22	1.34E-20	up	131	169	171	17	17	20	PLCB2
1.601509	2.23E-09	3.12E-08	up	791	575	1002	312	227	250	HIPK2
1.782413	3.09E-16	1.05E-14	up	421	327	478	110	127	133	LRRC49
1.587755	0.0001051	0.000588	up	52	45	45	15	21	14	PKDCC
1.082869	0.0001693	0.000897	up	162	214	203	54	135	105	NDUFAF1
6.249112	2.45E-06	2.00E-05	up	22	16	42	0	1	0	ITGA11
2.2257	5.86E-08	6.53E-07	up	587	500	745	218	118	55	THBS1
7.054165	1.29E-07	1.35E-06	up	16	31	23	0	0	0	IL24
1.292803	1.86E-11	3.50E-10	up	431	364	453	139	220	179	MAPT
3.207099	4.68E-07	4.46E-06	up	45	38	29	5	7	1	FCMR
1.410778	9.29E-19	4.04E-17	up	1136	1300	1321	420	581	487	POFUT2
2.854449	2.35E-28	2.29E-26	up	662	751	1124	132	103	121	PAQR5
2.530005	2.33E-22	1.42E-20	up	281	209	328	38	55	55	TRABD2A
1.053249	2.19E-06	1.80E-05	up	531	605	447	212	334	269	RNF216P1
1.632721	2.63E-18	1.10E-16	up	4441	4639	6764	1530	1836	1913	PAIP1
3.623135	0.0013427	0.005551	up	9	8	19	1	1	1	NEGR1
3.737164	0.0001839	0.000965	up	20	9	24	2	0	2	
1.091197	3.89E-08	4.44E-07	up	483	439	441	189	240	243	ZNF250
2.842221	3.38E-27	3.09E-25	up	301	273	265	28	56	41	PLEKHG4
2.649805	6.43E-09	8.46E-08	up	277	186	324	75	31	18	FAT4
2.091393	3.78E-06	2.95E-05	up	53	80	87	26	11	15	SEMA4A

5.472366	0.0005687	0.002614	up	9	10	4	0	0	0	
2.581866	2.04E-13	5.01E-12	up	160	156	321	26	34	49	DOCK11
5.011118	0.0053689	0.018087	up	1	10	6	0	0	0	NXF3
2.717171	4.45E-18	1.82E-16	up	1511	1476	2806	397	260	222	ARHGAP29
1.799449	0.0088171	0.027574	up	31	18	98	22	5	13	PWAR5
4.770119	0.0069392	0.022482	up	19	8	0	1	0	0	NEUROD1
1.688874	6.36E-15	1.92E-13	up	833	719	1128	279	270	303	SUCLG2
2.14587	3.58E-10	5.66E-09	up	150	222	253	53	32	58	
1.197982	0.0010897	0.004626	up	156	198	141	32	118	87	
1.146044	1.34E-09	1.95E-08	up	14428	####	####	6631	9604	9071	CD55
4.581012	6.36E-30	6.91E-28	up	154	186	200	6	14	4	NTNG2
1.313451	3.90E-09	5.29E-08	up	628	663	1074	326	370	285	BCL6
2.188853	1.92E-21	1.09E-19	up	1980	1626	2861	510	458	476	RAI14
2.604806	0.0006367	0.002887	up	12	20	25	2	6	2	LOC100506801
3.307763	7.57E-25	5.78E-23	up	214	213	353	33	30	18	CSGALNACT1
5.546178	8.77E-05	0.000501	up	19	21	7	1	0	0	SARDH
1.21763	2.29E-13	5.56E-12	up	2585	2468	2472	1059	1197	1126	GTPBP2
1.074455	4.47E-05	0.000275	up	652	516	827	395	298	268	TSC22D2
2.853296	6.20E-12	1.25E-10	up	75	90	97	5	21	13	NFE2
1.084969	0.0001608	0.000857	up	1056	1026	2078	780	602	585	ZNF24
1.285914	9.75E-09	1.24E-07	up	597	594	449	202	291	224	ZNF777
1.342109	0.0018135	0.007232	up	48	59	56	10	38	22	C20orf96
2.14744	4.12E-05	0.000255	up	33	72	74	9	28	7	
5.558022	0.0004811	0.002252	up	3	12	10	0	0	0	
2.14021	1.40E-11	2.70E-10	up	402	508	970	162	174	100	
1.473078	3.53E-07	3.44E-06	up	314	354	267	70	185	113	CEBPD
2.063317	1.71E-16	6.06E-15	up	296	302	331	90	78	62	GLI2
3.655802	0.0101541	0.031061	up	6	8	9	0	2	0	IL13RA2
1.488549	3.97E-13	9.37E-12	up	413	500	562	142	204	205	PPAPDC1B
1.272156	3.98E-11	7.16E-10	up	1129	1243	1746	580	642	539	RAB12
1.474503	2.44E-06	1.99E-05	up	97	78	132	37	46	32	TPK1
6.807057	4.94E-100	7.22E-97	up	2478	2262	4690	37	25	23	MME
2.607186	1.49E-20	7.58E-19	up	247	374	450	56	56	69	
2.411756	1.38E-05	9.56E-05	up	28	33	42	9	7	4	FAR2
1.236004	0.0024389	0.009247	up	38	79	63	22	27	31	
8.509607	2.78E-12	5.91E-11	up	100	122	156	0	0	1	
4.750776	0.0074956	0.024027	up	4	7	3	0	0	0	ARHGAP28
1.442785	2.05E-07	2.07E-06	up	126	105	166	44	64	45	ADAMTS6
3.490282	1.65E-11	3.14E-10	up	56	110	87	13	5	5	SLC12A8
1.148599	5.05E-07	4.76E-06	up	2285	2455	1825	731	1462	1015	FADS3
1.524912	3.43E-09	4.70E-08	up	589	592	1059	279	244	266	LRP12
1.465113	7.11E-07	6.55E-06	up	368	463	303	86	218	145	TMEM134
4.518985	1.18E-44	2.64E-42	up	848	1197	1593	23	76	70	MMP1
1.118905	0.0090459	0.028179	up	139	102	287	119	59	60	VGLL3

1.09413	8.65E-10	1.30E-08	up	700	601	845	309	380	357	ZFP62
1.061186	0.000347	0.001692	up	155	119	171	87	65	66	EBF3
1.099857	3.27E-05	0.000208	up	351	462	308	127	227	207	RPL41
1.589171	8.18E-21	4.38E-19	up	656	661	778	227	277	224	WRB
3.164301	1.58E-11	3.02E-10	up	84	77	65	3	11	13	TNFRSF9
5.249456	0.0010588	0.004521	up	7	7	6	0	0	0	SIGLEC1
6.349142	7.76E-06	5.66E-05	up	12	9	23	0	0	0	
1.267506	2.02E-06	1.68E-05	up	1242	1160	887	285	673	528	SMOX
1.329568	6.26E-08	6.93E-07	up	845	1340	1731	535	617	459	
5.064164	0.0112337	0.03376	up	12	5	0	0	0	0	PCDHA3
3.181374	7.10E-09	9.28E-08	up	64	47	55	3	14	3	TMCC2
1.263147	5.58E-13	1.29E-11	up	4766	5693	5251	1738	2971	2274	SEC13
2.138594	1.04E-07	1.12E-06	up	67	54	69	9	22	15	
1.033637	5.37E-06	4.06E-05	up	455	474	723	261	264	302	DSTYK
4.91214	1.05E-63	4.87E-61	up	413	502	491	15	17	17	TMEM171
1.047922	5.40E-12	1.10E-10	up	1596	1770	2068	823	1080	850	TCEAL4
3.860495	1.42E-13	3.55E-12	up	52	71	87	4	4	7	DNAJC12
1.553537	2.01E-20	1.01E-18	up	3378	3240	4305	1282	1412	1162	ZMIZ1
1.291082	3.93E-06	3.06E-05	up	670	602	906	372	356	188	COL27A1
1.402152	0.0003606	0.001749	up	73	86	57	21	47	21	
2.083073	1.36E-10	2.27E-09	up	153	89	161	31	37	31	MAML3
1.299129	8.17E-10	1.23E-08	up	7576	7732	5810	2291	3974	2970	UBE2S
2.045524	3.17E-14	8.81E-13	up	2040	1691	2958	684	502	448	CREB3L2
1.822914	1.89E-09	2.69E-08	up	150	202	223	62	44	60	FAM230C
1.620383	1.88E-24	1.39E-22	up	1052	1011	1281	327	428	382	NFE2L3
1.041443	3.52E-07	3.43E-06	up	1067	1064	844	469	569	488	NECAP2
2.814676	1.68E-13	4.17E-12	up	200	150	224	40	32	12	NRG1
5.118967	0.0026142	0.009809	up	4	3	12	0	0	0	
1.842518	2.59E-14	7.28E-13	up	2345	2465	1758	434	887	664	DYNLL1
1.707888	3.96E-05	0.000247	up	58	60	66	9	33	19	LRRC20
1.003624	1.27E-07	1.34E-06	up	1129	1271	1165	451	786	659	MUS81
1.003821	2.28E-06	1.87E-05	up	199	228	254	101	135	120	FUT10
3.756752	0.0177772	0.049301	up	26	8	0	0	3	0	RGPD4
1.170744	1.27E-05	8.83E-05	up	608	538	1103	349	316	346	C9orf72
1.04978	6.63E-07	6.13E-06	up	271	323	367	160	171	151	LINC-PINT
1.508686	0.0003836	0.001844	up	41	44	88	20	27	16	SCN8A
4.574398	0.0141055	0.040753	up	3	2	8	0	0	0	VLDLR
1.409938	8.13E-15	2.41E-13	up	1961	2046	2279	862	892	702	MGLL
2.85679	3.53E-15	1.09E-13	up	99	83	138	14	18	14	CA12
1.794042	0.0017561	0.007031	up	39	29	44	3	11	20	LOC653160
5.240834	4.75E-12	9.72E-11	up	68	46	69	2	0	3	CES3
1.469198	4.87E-17	1.79E-15	up	894	879	835	294	387	313	ST3GAL2
1.431907	1.30E-10	2.18E-09	up	277	291	381	121	120	122	C1S
1.292493	3.58E-17	1.34E-15	up	3514	3874	4302	1426	2146	1475	CASP4

1.523224	6.80E-08	7.50E-07	up	283	345	307	62	147	140	DHRS4
1.490711	0.0005592	0.002573	up	66	61	44	23	30	12	HRASLS2
1.110987	0.0019859	0.007823	up	88	90	55	35	46	34	RARRES3
1.200998	0.0001738	0.000918	up	601	536	408	114	349	274	MACROD1
3.506601	0.0001196	0.00066	up	11	20	24	1	1	3	DAW1
1.04545	1.83E-06	1.54E-05	up	1148	1254	1796	737	658	680	ZCCHC7
1.951248	3.16E-10	5.04E-09	up	979	1017	1472	426	260	213	NTN4
3.241116	1.13E-17	4.44E-16	up	196	133	120	19	18	13	LAMC3
1.387971	4.05E-10	6.37E-09	up	672	690	583	180	332	284	BCS1L
1.013916	0.0001498	0.000806	up	488	535	464	313	266	184	MYEOV
1.260902	1.09E-08	1.38E-07	up	646	579	708	316	280	235	NUAK1
1.59371	0.0002284	0.001173	up	59	48	37	14	22	15	MRGPRF
1.001268	6.87E-05	0.000402	up	328	251	387	117	178	209	
4.223692	3.74E-06	2.92E-05	up	22	24	25	2	2	0	GXYLT2
1.515583	1.14E-14	3.34E-13	up	717	683	701	204	360	222	SH3RF3
1.582209	5.10E-14	1.38E-12	up	743	729	667	185	352	231	C1QTNF6
1.841228	0.0056996	0.019031	up	17	16	18	4	6	5	ZNF19
1.336193	4.40E-20	2.14E-18	up	2137	2294	2415	798	1118	940	MORC2
1.41625	3.84E-06	2.99E-05	up	481	328	781	237	213	154	PDZD2
1.561392	3.40E-09	4.67E-08	up	473	444	845	221	222	167	ZNF532
1.499122	0.0013994	0.005758	up	57	78	76	12	55	16	HOXB6
1.226562	6.44E-05	0.00038	up	5419	4886	####	3654	2420	2854	LMAN1
1.191458	6.67E-10	1.02E-08	up	317	350	415	138	207	154	LOC101927056
1.031669	2.71E-09	3.76E-08	up	1705	1970	1957	719	1209	999	ADI1
3.687657	7.89E-06	5.75E-05	up	28	25	22	2	0	4	SPNS3
2.122269	7.68E-06	5.61E-05	up	41	44	45	9	8	14	LCA5L
2.490695	2.83E-07	2.80E-06	up	123	89	159	40	13	12	EPGN
1.714867	6.18E-14	1.65E-12	up	259	263	261	82	100	69	TSC22D3
1.385986	2.22E-11	4.13E-10	up	340	365	450	111	195	161	AFAP1L1
5.085269	0.0024284	0.009208	up	3	8	7	0	0	0	TNIP3
1.028407	4.32E-06	3.33E-05	up	389	408	337	145	259	192	MKS1
2.326512	0.0069685	0.02257	up	15	9	14	1	3	4	CHRNE
2.898499	2.71E-05	0.000175	up	20	24	41	4	7	1	ICAM2
1.222114	5.51E-16	1.80E-14	up	1346	1313	1462	513	714	631	MXRA7
1.042984	0.0003956	0.001895	up	561	470	410	136	289	325	EEFSEC
1.904258	3.53E-15	1.09E-13	up	655	683	1132	241	254	183	DDX60L
1.434079	5.84E-14	1.56E-12	up	1057	1010	1177	296	492	480	RAP1GAP2
1.211378	0.0003197	0.001575	up	1725	1598	3401	1330	755	779	PRKAA1
1.314115	5.69E-05	0.000341	up	76	61	85	25	39	30	PTPRE
1.748832	1.57E-06	1.34E-05	up	84	61	73	18	23	27	SHROOM2
1.412205	1.81E-12	3.97E-11	up	1356	1276	1253	367	587	593	NLE1
1.172899	7.80E-06	5.68E-05	up	1531	1437	2976	939	883	847	ZNF217
1.847349	5.86E-06	4.40E-05	up	59	57	62	22	15	14	SCG2
5.970694	4.08E-05	0.000254	up	13	10	10	0	0	0	

1.64614	3.15E-08	3.66E-07	up	164	208	215	48	58	89	MEX3A
2.654774	3.53E-05	0.000222	up	31	24	29	2	4	8	YY2
2.288065	4.47E-11	8.01E-10	up	101	114	147	21	42	16	PPP2R2B
1.631557	0.0032444	0.011808	up	35	24	27	4	15	11	
1.132488	0.0001436	0.000777	up	1292	1163	2332	920	624	635	RSF1
2.551331	4.27E-14	1.17E-12	up	315	283	349	32	46	90	COQ3
1.52114	3.54E-16	1.19E-14	up	703	737	849	200	345	299	POPDC3
1.163474	1.15E-06	1.00E-05	up	309	457	541	150	268	199	GSDMB
1.056678	1.73E-06	1.46E-05	up	1180	1144	1768	688	619	698	USP13
3.397785	5.52E-08	6.19E-07	up	34	35	42	2	3	6	RASSF4
1.577965	2.43E-08	2.89E-07	up	220	133	204	51	89	58	DHRS4-AS1
1.566752	6.03E-13	1.40E-11	up	936	1029	774	248	405	336	MPV17L2
2.232396	0.0001539	0.000825	up	57	59	40	21	10	3	
1.939794	2.84E-15	8.91E-14	up	380	409	322	101	100	102	UBE2L6
1.195667	2.72E-06	2.20E-05	up	1107	1115	2044	667	583	634	VPS13D
1.278044	1.27E-10	2.13E-09	up	1707	1772	1473	544	848	781	ERAL1
3.740596	0.0066975	0.021818	up	9	6	11	0	0	2	
3.297869	2.61E-29	2.72E-27	up	3052	3074	3296	456	301	217	HKDC1
2.41287	7.67E-16	2.48E-14	up	150	172	279	31	51	36	MATN2
1.982894	5.93E-23	3.93E-21	up	639	695	794	168	256	146	DLG4
1.423441	4.34E-11	7.81E-10	up	586	767	594	200	313	260	VPS4A
1.435255	3.60E-12	7.51E-11	up	1117	1277	1192	316	568	528	NSMCE4A
1.657685	2.91E-08	3.42E-07	up	130	119	103	34	45	39	HEIH
1.500992	3.20E-06	2.54E-05	up	181	144	312	85	66	76	FANCB
1.260908	1.76E-07	1.81E-06	up	202	171	208	82	106	67	GPR176
1.517233	1.43E-09	2.07E-08	up	366	432	506	94	202	188	NES
1.328595	0.0141814	0.040904	up	34	32	83	27	10	21	ADGRL2
2.865879	7.82E-10	1.18E-08	up	92	83	64	4	21	11	SMCO4
1.270011	1.23E-05	8.57E-05	up	289	301	303	151	162	75	ABTB2
1.055627	7.60E-05	0.000442	up	1929	2031	1345	641	1398	758	PHLDA2
1.035666	5.17E-08	5.82E-07	up	2352	2212	2396	807	1561	1261	HTRA1
1.036606	6.49E-05	0.000383	up	118	133	176	71	79	66	TCP11L2
1.025624	0.0010373	0.004444	up	395	456	735	345	214	219	SPRED1
1.278798	0.0005791	0.002656	up	63	42	71	20	30	26	SENP8
1.049559	9.27E-08	1.00E-06	up	788	730	710	276	460	409	ALKBH3
3.372053	0.0004285	0.002036	up	12	12	28	2	0	3	
3.48161	2.68E-47	6.97E-45	up	759	790	619	53	77	76	COL6A2
1.329812	3.04E-10	4.86E-09	up	1024	900	1372	381	442	530	TBC1D31
1.569068	1.68E-09	2.41E-08	up	963	1165	1009	205	506	431	ATHL1
1.29221	8.10E-05	0.000467	up	358	370	642	252	142	162	CTBS
1.168317	7.40E-11	1.29E-09	up	2593	2531	3480	1310	1343	1290	UAP1
1.760943	0.0002478	0.001261	up	50	41	71	25	12	11	GBP1
1.451114	8.82E-09	1.13E-07	up	384	407	554	196	189	122	GBP3
1.140326	2.81E-06	2.26E-05	up	733	849	631	232	487	367	CUEDC2

4.627355	0.0096973	0.02988	up	4	5	4	0	0	0	ADAMTS15
2.008847	0.0058487	0.019447	up	18	20	12	4	3	6	
1.520697	1.89E-11	3.56E-10	up	1466	1741	2590	727	689	647	IKBIP
1.623512	1.14E-09	1.68E-08	up	261	286	214	75	121	69	GPR3
2.048971	1.89E-11	3.57E-10	up	480	406	818	173	120	120	FBN1
1.830704	4.20E-26	3.45E-24	up	1067	1101	1032	277	360	311	LARP6
1.406248	3.08E-09	4.23E-08	up	282	223	239	94	119	83	ZNF319
1.295493	6.92E-06	5.12E-05	up	272	294	508	173	135	134	FAM72D
1.648485	2.78E-25	2.21E-23	up	4444	4045	4480	1216	1564	1555	ECE1
1.369016	4.60E-08	5.20E-07	up	369	383	315	95	179	168	GEMIN7
3.918218	1.10E-05	7.77E-05	up	21	28	20	0	4	1	ADAMTS10
2.538286	7.06E-06	5.21E-05	up	40	66	31	11	10	4	ALOX5AP
1.36746	2.46E-19	1.13E-17	up	2096	2273	2434	747	1170	876	DKK1
2.479093	5.68E-29	5.86E-27	up	825	728	1251	131	208	186	CLMP
1.417816	6.80E-11	1.20E-09	up	210	209	255	77	107	81	ZNF329
2.176119	2.82E-05	0.000181	up	33	28	49	10	8	7	PNMA2
2.611834	1.99E-06	1.66E-05	up	45	46	29	4	8	9	
1.188522	3.15E-06	2.51E-05	up	3066	2607	4816	1777	1391	1472	BUB1B
1.121929	1.71E-09	2.46E-08	up	9773	####	####	5265	5580	5665	EIF4A2
2.571076	1.43E-11	2.75E-10	up	79	68	107	10	20	15	CACNG8
1.398474	1.11E-13	2.84E-12	up	3925	3651	5402	1728	1935	1431	TRIM44
1.275965	3.08E-13	7.36E-12	up	3671	4197	3545	1382	1918	1688	ILK
1.475698	0.0048986	0.016736	up	29	36	28	5	16	15	C11orf74
4.977901	0.0131281	0.038388	up	10	6	0	0	0	0	PCDHA10
1.772324	0.0001673	0.000888	up	34	38	66	16	11	14	LINC01021
4.784066	1.78E-23	1.23E-21	up	134	146	150	9	1	6	SFTA1P
6.890526	3.29E-07	3.22E-06	up	13	25	25	0	0	0	ZNF718
1.069103	0.0033937	0.01226	up	46	58	64	26	26	31	LOC283140
1.120087	2.40E-07	2.40E-06	up	487	488	553	163	291	290	SLC47A1
1.581694	9.50E-20	4.48E-18	up	1189	1138	1292	419	438	399	CUL7
6.147231	1.84E-05	0.000123	up	9	11	18	0	0	0	HSD11B1
1.302299	1.02E-14	2.97E-13	up	904	911	922	331	430	405	HCCS
1.843494	6.64E-18	2.66E-16	up	410	338	403	83	135	121	ST5
2.750624	1.12E-19	5.25E-18	up	241	185	356	37	36	46	PRTG
9.322853	1.91E-14	5.41E-13	up	106	118	114	0	0	0	GAGE2C
2.877987	1.05E-11	2.06E-10	up	74	59	83	6	13	12	CNN3
2.666212	1.06E-06	9.42E-06	up	37	59	44	5	5	13	
2.627398	5.68E-45	1.28E-42	up	1152	1257	1599	192	235	247	RIOK2
3.336419	3.42E-21	1.90E-19	up	167	233	222	25	13	25	RCN3
1.049014	0.0013049	0.00542	up	115	80	187	60	65	64	CCDC68
1.138848	8.65E-05	0.000496	up	1541	1951	1206	430	1124	788	SH3BGRL3
3.180821	8.68E-07	7.84E-06	up	43	29	36	1	9	3	BEAN1
1.755405	5.99E-12	1.21E-10	up	198	181	220	41	86	62	TK2
3.091003	1.67E-24	1.24E-22	up	228	187	264	34	25	23	PRDM16

1.828351	2.27E-09	3.17E-08	up	218	131	195	45	49	65	CCDC113
5.95201	3.01E-91	2.39E-88	up	756	808	897	7	18	17	FYN
2.276218	5.13E-21	2.81E-19	up	267	228	283	57	57	53	LINC01234
1.16619	0.000125	0.000687	up	280	265	496	195	144	128	AP1S3
2.784301	0.0113715	0.034087	up	10	5	12	2	1	1	
5.378125	0.0006719	0.003028	up	5	9	8	0	0	0	GPR162
4.999815	4.74E-99	5.63E-96	up	2957	3445	3236	121	123	71	NNMT
1.096898	9.77E-10	1.45E-08	up	609	728	874	298	439	349	HSDL1
1.294553	1.07E-13	2.75E-12	up	11910	####	####	4569	6036	4786	COTL1
1.264503	1.74E-12	3.82E-11	up	1628	1829	1653	571	964	735	NGFRAP1
2.371401	0.0003137	0.001548	up	29	30	17	2	8	6	NECAB2
1.084303	0.0157721	0.044652	up	52	27	47	23	25	14	ZNF613
2.662926	3.93E-14	1.08E-12	up	471	494	383	29	98	104	NUPR1
3.10567	1.33E-06	1.15E-05	up	25	34	31	3	4	4	
3.931317	0.000394	0.001888	up	20	11	12	0	1	2	
1.118357	9.76E-05	0.000551	up	2193	2176	4534	1593	1251	1265	ACSL4
1.008536	2.24E-07	2.25E-06	up	883	781	1200	490	546	436	FAM111A
1.485818	7.16E-09	9.34E-08	up	510	487	401	114	205	214	KIF7
2.517648	5.74E-15	1.74E-13	up	900	743	1213	241	151	109	NAV2
1.429456	3.14E-08	3.65E-07	up	270	298	417	119	108	147	C18orf54
1.775563	8.31E-17	2.98E-15	up	1446	1293	1942	488	436	473	MGAT5
2.238265	5.37E-30	5.96E-28	up	505	469	667	106	140	117	ZBTB39
1.455959	1.91E-16	6.64E-15	up	1216	1267	1256	388	516	528	GPKOW
1.839964	4.86E-11	8.67E-10	up	1001	852	1573	402	326	241	SLFN5
1.549947	4.37E-13	1.02E-11	up	600	538	917	208	306	219	ARL14EP
1.062782	0.0002768	0.001391	up	1231	1088	2101	891	599	624	YES1
3.202495	3.22E-31	3.80E-29	up	4879	6321	8083	914	711	503	CYR61
2.169551	4.11E-22	2.46E-20	up	271	267	282	58	77	57	C16orf45
2.315324	3.64E-19	1.64E-17	up	680	632	700	76	185	172	SAAL1
3.44542	0.0023578	0.009009	up	11	11	9	1	1	1	YPEL4
3.701558	8.13E-16	2.61E-14	up	124	131	119	1	14	16	FOXF1
1.165089	1.49E-13	3.72E-12	up	1984	2239	2682	990	1229	994	TMEM39A
2.61113	6.22E-26	5.03E-24	up	293	305	269	39	64	48	SPHK1
1.296104	4.97E-06	3.79E-05	up	323	314	219	88	182	109	LMF1
1.837407	1.92E-17	7.40E-16	up	651	691	988	210	215	245	MNAT1
4.067307	3.33E-11	6.05E-10	up	59	74	44	6	2	3	
2.567852	0.0019024	0.007534	up	17	11	17	3	4	1	
1.28004	0.0031542	0.011515	up	31	43	39	14	19	16	NYAP1
1.531403	4.47E-12	9.20E-11	up	523	539	430	155	197	193	
1.111805	0.0002017	0.001049	up	86	100	94	33	53	51	
2.592411	1.64E-37	2.59E-35	up	777	914	845	110	172	163	EVA1C
1.018317	5.88E-09	7.81E-08	up	8478	7580	7066	3546	4419	4055	MARS
1.11349	0.000373	0.001801	up	1113	920	2423	737	617	705	ATAD5
1.023722	4.72E-08	5.33E-07	up	578	460	550	219	336	271	CPPED1

1.275483	4.51E-10	7.04E-09	up	15061	####	####	6596	5862	5637	PTPRF
1.378962	8.97E-07	8.06E-06	up	350	258	305	78	128	164	CLUAP1
1.695198	0.0061871	0.020375	up	18	20	38	5	6	13	AZIN2
4.149757	0.0099552	0.03057	up	5	9	4	0	0	1	MEFV
3.467252	0.0102445	0.031292	up	2	5	25	0	2	1	CD74
2.403864	2.58E-10	4.17E-09	up	628	587	1092	229	109	91	SAMD4A
4.702504	0.0082343	0.026017	up	3	4	7	0	0	0	
1.246153	0.0028529	0.010571	up	61	80	77	12	51	37	
9.218227	6.00E-14	1.60E-12	up	84	89	146	0	0	0	LOC105376603
9.402469	1.32E-14	3.82E-13	up	92	122	146	0	0	0	
1.924935	1.31E-05	9.13E-05	up	310	270	438	151	41	68	SPOCK1
1.61645	1.12E-13	2.86E-12	up	537	561	607	200	174	199	NMT2
1.001809	0.000441	0.002087	up	880	751	1463	627	441	480	JMY
3.053814	0.0012586	0.00526	up	12	16	11	1	2	2	QPRT
1.00568	3.45E-06	2.72E-05	up	670	670	991	421	390	377	CWF19L2
2.13916	3.26E-10	5.18E-09	up	86	80	105	18	30	17	DMRTA1
1.615748	1.08E-06	9.55E-06	up	159	252	138	45	88	61	TMEM158
2.902396	1.72E-27	1.58E-25	up	686	578	742	113	73	87	LAMP3
1.126779	8.91E-09	1.14E-07	up	2219	2105	3308	1150	1221	1219	CCDC50
6.61073	2.27E-13	5.50E-12	up	75	85	120	1	2	0	SPEF2
1.9398	2.58E-09	3.58E-08	up	183	187	136	43	69	30	WDR25
1.53015	1.68E-08	2.05E-07	up	1045	1098	738	217	481	386	WDR24
1.020934	0.0006609	0.002984	up	106	111	132	71	58	48	
1.736674	3.16E-14	8.79E-13	up	1191	1330	1069	369	501	277	PLA2G16
1.304609	0.0042307	0.014773	up	34	34	33	11	15	17	IQCH
1.075392	7.64E-07	6.99E-06	up	1200	1129	1676	664	589	686	ARNTL2
2.735071	0.000121	0.000667	up	22	13	55	3	7	4	
1.705233	0.0022045	0.00854	up	26	39	23	5	12	12	ATP2A1-AS1
2.546929	4.81E-09	6.47E-08	up	52	58	102	8	17	13	GNRHR2
1.85495	0.016151	0.045474	up	10	13	24	7	3	3	RNF152
3.120297	0.0001086	0.000607	up	15	17	26	1	3	3	LURAP1L-AS1
3.005748	5.85E-20	2.80E-18	up	537	568	1162	115	73	93	LOX
2.601852	1.11E-06	9.78E-06	up	27	42	61	8	7	7	CNBD1
3.392781	3.58E-47	9.06E-45	up	1874	1760	3154	229	255	181	DIO2
1.622218	2.49E-09	3.45E-08	up	414	436	275	103	148	139	TMEM261
3.446282	5.84E-05	0.000349	up	15	18	41	0	2	5	IL33
3.969278	1.50E-33	1.92E-31	up	218	242	207	11	16	18	CORO2B
2.374019	4.25E-20	2.08E-18	up	952	1036	1397	214	173	277	LACTB
1.367581	9.27E-07	8.30E-06	up	3455	3132	6070	2027	1548	1354	MACF1
4.149737	0.0098824	0.030391	up	5	9	4	1	0	0	IGDCC4
1.2074	1.11E-07	1.18E-06	up	465	465	478	143	241	261	ACSF3
1.462331	3.35E-06	2.65E-05	up	105	131	199	59	50	52	ANKRD46
1.806276	1.29E-09	1.89E-08	up	115	156	217	46	50	48	DNAJB5
4.270934	0.0001442	0.00078	up	9	17	33	3	0	0	LINC01583

1.049097	0.0024419	0.009256	up	66	130	106	35	63	57	PPP1R32
4.10168	2.15E-10	3.51E-09	up	57	36	53	3	5	1	FOXL1
1.446722	8.38E-15	2.48E-13	up	461	474	619	164	226	206	ZSCAN9
1.435518	8.75E-14	2.27E-12	up	804	1085	1261	348	478	393	PIM1
4.633936	1.45E-20	7.40E-19	up	112	132	106	6	8	1	FOXC2
4.147792	1.56E-49	4.71E-47	up	450	459	524	32	38	15	BDNF
1.067749	5.46E-05	0.000329	up	1265	1256	767	422	757	522	FLYWCH2
1.091313	0.0001032	0.000579	up	2825	2373	5677	1853	1814	1498	HMGCR
1.606755	5.76E-09	7.66E-08	up	175	183	144	49	62	63	TMEM8B
5.057331	3.07E-143	########	up	1990	2021	1958	51	79	60	SPARC
1.158413	0.0003448	0.001681	up	88	104	90	27	64	45	PPP2R5B
1.674354	0.0136085	0.039546	up	20	34	17	13	7	3	
1.0849	3.82E-08	4.37E-07	up	11530	####	9982	3984	6522	6132	PHGDH
1.166038	0.0060877	0.020101	up	83	64	39	23	29	36	FLRT1
1.193887	6.90E-11	1.21E-09	up	2927	3322	4468	1590	1757	1493	DGKD
4.782859	0.0077725	0.024752	up	4	2	9	0	0	0	HIST1H3E
1.008914	0.0002435	0.00124	up	1080	1244	661	420	670	511	C11orf68
3.51377	1.79E-17	6.94E-16	up	210	224	467	41	19	18	MCTP1
1.58946	0.0024017	0.009139	up	44	31	53	13	7	23	LOC100507291
1.09972	0.0002221	0.001144	up	377	381	706	283	206	197	FAM160B1
1.855559	0.000857	0.003744	up	21	30	32	9	10	5	PRKCG
1.139222	4.15E-05	0.000257	up	149	151	131	72	81	53	WNK4
5.210181	1.55E-07	1.61E-06	up	216	149	296	11	7	0	NAV3
1.018749	9.63E-12	1.89E-10	up	16245	####	####	7379	####	7747	SQSTM1
1.599382	0.0043028	0.014986	up	26	43	24	5	13	15	
1.640217	9.91E-06	7.04E-05	up	89	85	79	37	27	20	CA13
1.375518	2.93E-08	3.43E-07	up	692	821	776	182	380	380	MRPL48
2.467186	8.11E-23	5.22E-21	up	246	207	307	42	51	50	NEK3
4.838223	0.0052845	0.017858	up	7	4	4	0	0	0	
1.87682	1.39E-07	1.46E-06	up	112	81	124	24	24	41	RNF144A
1.177569	4.44E-06	3.42E-05	up	21684	####	####	12926	9434	7781	FLNB
1.456266	7.03E-13	1.62E-11	up	6395	6991	9807	3047	2871	2731	SOD2
3.741498	1.13E-06	9.92E-06	up	25	21	47	4	1	2	ALDH1L2
1.717186	1.06E-25	8.55E-24	up	5923	6072	6635	1482	2336	2168	CKAP4
1.221374	5.07E-13	1.18E-11	up	641	612	701	242	332	306	PPARD
1.238835	2.96E-08	3.46E-07	up	2932	2591	4325	1546	1379	1323	DNAJC10
1.007886	5.75E-05	0.000345	up	202	197	221	97	154	77	ADSSL1
1.575383	0.0150104	0.042885	up	16	15	44	4	10	12	
1.080952	1.97E-06	1.64E-05	up	4005	3586	5800	2421	2059	1954	SCRN1
1.600463	1.79E-08	2.18E-07	up	120	113	154	35	46	52	FLRT2
1.001836	1.84E-08	2.23E-07	up	654	738	905	377	466	354	SPRY2
2.205811	6.61E-06	4.90E-05	up	48	61	60	18	6	13	LCP1
1.206345	1.01E-10	1.72E-09	up	408	456	455	162	248	195	RPS27L
1.594911	1.10E-13	2.82E-12	up	865	821	1188	315	300	360	NBAS

1.085129	0.0039374	0.013914	up	67	78	100	42	26	49	CCDC122
1.653342	8.13E-06	5.90E-05	up	66	51	69	20	25	17	THSD1
1.61821	1.84E-06	1.55E-05	up	71	63	83	23	29	22	BACH2
1.153587	2.29E-08	2.74E-07	up	304	320	453	152	194	158	SIX1
1.280803	0.0003636	0.00176	up	1229	974	2205	865	423	490	WWC2
9.578631	1.83E-15	5.81E-14	up	249	215	330	0	1	0	FLI1
5.507056	0.0003474	0.001693	up	8	8	8	0	0	0	EVI2A
2.031691	5.18E-24	3.67E-22	up	1228	1024	1122	202	383	297	ZNF629
4.542318	5.37E-17	1.97E-15	up	72	88	179	6	7	2	TGFB2
1.035958	4.44E-06	3.42E-05	up	1570	1830	1475	545	1121	897	EXOSC5
1.80652	6.40E-21	3.45E-19	up	653	706	739	160	233	238	LYRM1
2.889961	1.05E-05	7.44E-05	up	24	43	23	4	6	3	
2.569903	2.00E-15	6.32E-14	up	188	172	209	26	25	48	HOXD8
1.042566	4.41E-06	3.40E-05	up	664	526	929	329	345	381	ZNF445
1.042162	9.93E-09	1.26E-07	up	3224	4085	3375	1526	2267	1723	IFITM2
2.253875	3.55E-37	5.52E-35	up	1021	1020	1380	235	281	230	WBP5
1.317592	2.14E-11	3.98E-10	up	461	419	413	155	207	185	ZNF74
1.105845	1.12E-06	9.87E-06	up	1331	1254	1756	754	598	699	NOMO2
2.265271	0.0079087	0.025114	up	16	10	11	3	2	3	
1.107916	1.34E-07	1.41E-06	up	1091	1275	1216	420	627	704	MAPKAPK5-AS1
1.664346	2.81E-12	5.97E-11	up	426	441	588	181	152	137	C5orf28
1.862654	1.72E-10	2.85E-09	up	135	150	156	30	65	35	FBXO4
6.38708	7.72E-83	5.43E-80	up	759	722	808	7	16	6	IL6
1.844018	7.78E-14	2.04E-12	up	220	185	270	66	70	59	ALDH5A1
3.111452	2.90E-13	6.97E-12	up	101	93	168	20	17	6	SOBP
1.134209	0.0025425	0.009578	up	82	68	84	50	33	26	BEND6
5.514211	0.0003512	0.001709	up	8	9	7	0	0	0	
1.276269	7.93E-06	5.77E-05	up	259	337	529	107	178	198	RGPD2
2.155089	0.0021248	0.008274	up	23	14	34	5	2	9	
1.108709	1.70E-06	1.44E-05	up	1340	1791	1188	539	876	731	UBALD2
5.833609	8.11E-05	0.000468	up	12	9	9	0	0	0	IL3RA
2.771888	0.0002233	0.00115	up	65	35	88	22	2	2	MCF2L2
1.034521	5.43E-08	6.10E-07	up	10172	####	####	3943	6803	6018	GAS5
3.591163	0.0137823	0.039997	up	4	13	6	0	0	2	SLC25A21-AS1
1.000573	3.85E-08	4.40E-07	up	1794	1670	2475	925	1061	1080	POLD3
3.298317	1.87E-22	1.15E-20	up	185	171	227	11	20	31	TTC5
2.402674	0.0143232	0.041269	up	11	8	18	5	2	0	ARL14EPL
1.148107	2.20E-06	1.81E-05	up	302	353	343	101	230	154	MAPK11
1.671566	2.87E-17	1.09E-15	up	1124	1284	1220	295	444	463	RSAD1
2.09312	6.84E-29	6.95E-27	up	1264	1387	1425	246	381	381	PRPSAP1
2.775239	0.0015026	0.00613	up	14	15	15	1	5	1	HSD17B14
1.173884	3.39E-06	2.68E-05	up	555	457	864	310	286	251	PDE10A
1.54297	0.0003443	0.001679	up	111	106	54	17	41	43	FAM131C
1.069978	0.0006935	0.003115	up	2518	2220	4104	1933	1094	1149	QKI

1.191986	2.28E-09	3.19E-08	up	469	470	641	229	241	244	NLK
3.48576	0.0176965	0.049148	up	3	8	11	0	0	2	SHISA8
1.781714	9.02E-06	6.47E-05	up	46	62	76	20	16	19	SLC6A16
1.530006	0.001066	0.004544	up	31	37	33	10	15	12	
1.123655	0.0041042	0.014413	up	51	70	49	22	26	34	GUCA1B
2.503416	1.85E-40	3.45E-38	up	1012	1071	1053	195	210	173	FGFR1
1.005052	3.13E-10	4.99E-09	up	1529	1376	1894	742	920	827	EXOC2
1.14732	4.77E-14	1.29E-12	up	1915	2000	2194	808	1241	872	ITGA5
1.450201	6.71E-10	1.03E-08	up	973	1048	956	231	503	437	MRPL40
1.299756	0.0001029	0.000578	up	117	83	82	28	56	39	NDUFA4L2
1.448653	1.63E-07	1.68E-06	up	171	146	269	74	80	67	LMLN
1.301752	0.0018917	0.007497	up	98	114	55	20	57	42	FAM171A2
1.486074	1.84E-06	1.55E-05	up	1364	1275	2717	791	515	593	BRWD1
4.123759	9.34E-08	1.01E-06	up	43	25	32	3	2	1	PTK7
2.285983	1.00E-13	2.58E-12	up	306	307	570	91	65	88	PYGO1
1.693011	0.0009421	0.004077	up	23	29	38	9	11	9	PKIA
1.343012	0.0009241	0.004005	up	787	580	1650	598	284	274	MYBL1
1.360097	7.60E-12	1.51E-10	up	1127	1146	1550	496	487	547	FEZ2
4.5438	0.0026396	0.009887	up	10	6	8	0	0	1	PRAME
5.964487	5.10E-05	0.000309	up	15	7	11	0	0	0	RASGEF1C
1.161139	1.93E-09	2.74E-08	up	1708	1549	2029	877	842	717	TNFRSF21
1.08635	0.014292	0.041192	up	59	41	40	13	39	20	TRIM7
1.542852	3.91E-06	3.04E-05	up	100	116	100	23	42	50	MMP2
1.255718	1.34E-08	1.67E-07	up	1414	1216	1986	714	647	612	FYTTD1
2.804363	0.0030596	0.011221	up	20	10	11	4	1	1	PCDHB18P
1.767541	0.0022824	0.008774	up	45	26	31	7	6	18	PPP1R3G
2.133117	4.60E-06	3.53E-05	up	34	55	69	15	12	10	LAMA4
2.318838	6.55E-15	1.97E-13	up	504	421	457	48	117	131	FMNL3
2.877011	3.80E-28	3.65E-26	up	339	302	434	59	53	39	PRKCE
1.830425	6.50E-16	2.11E-14	up	410	409	387	85	135	139	MORN4
1.416204	7.08E-05	0.000414	up	87	82	158	27	45	55	LOC155060
1.378176	3.00E-07	2.95E-06	up	6020	5102	9940	3294	2669	2212	TRIO
1.351651	3.03E-20	1.51E-18	up	5289	6036	6178	2152	2814	2242	PPP1R18
1.037207	5.01E-07	4.73E-06	up	634	540	542	230	322	330	NAT8L
1.578101	8.22E-21	4.39E-19	up	1557	1432	1846	472	601	615	STXBP1
2.248824	3.26E-12	6.88E-11	up	126	145	215	42	37	26	TLR4
4.520233	1.67E-35	2.37E-33	up	222	179	286	12	10	9	EVI2B
1.397221	9.41E-08	1.01E-06	up	1156	1632	1012	345	645	565	IFITM1
1.055484	0.0009075	0.003942	up	270	288	504	225	151	136	TBX18
2.524183	5.32E-09	7.11E-08	up	79	69	52	9	14	14	RDM1
1.965844	9.85E-10	1.46E-08	up	105	123	118	23	48	24	TMEM231
1.3368	0.0001138	0.000632	up	74	82	73	33	27	34	
1.172641	0.0001001	0.000564	up	102	147	184	66	60	71	
2.179784	9.53E-05	0.000539	up	21	37	37	5	8	9	LOC100507033

1.478554	0.0147163	0.042203	up	23	15	31	9	13	4	
1.246801	2.53E-18	1.07E-16	up	2697	2910	3198	1162	1527	1206	TPBG
1.082598	0.0020337	0.007979	up	384	346	772	319	181	200	ZNF267
1.640004	0.0003134	0.001548	up	50	58	38	9	26	16	LMX1B
2.525543	1.21E-19	5.69E-18	up	7330	7118	####	1889	1881	1604	HMGCS1
1.370435	1.82E-05	0.000122	up	119	115	151	27	79	54	ADCY10P1
6.307916	1.15E-06	1.00E-05	up	28	22	32	1	0	0	RSPO3
1.251205	9.94E-09	1.26E-07	up	249	268	255	87	140	117	RTN4RL1
1.666022	9.07E-09	1.16E-07	up	338	286	601	147	140	106	DSEL
1.196895	4.90E-07	4.63E-06	up	255	275	373	121	128	157	LRRC8C
1.335712	2.30E-06	1.89E-05	up	261	222	194	63	104	118	NT5M
4.50847	0.014113	0.040768	up	3	5	4	0	0	0	PTGFR
2.252431	0.0148179	0.042438	up	11	14	10	0	4	4	
4.627355	0.0096973	0.02988	up	4	5	4	0	0	0	PCDHGA3
1.677479	4.88E-24	3.48E-22	up	4162	3881	4417	1366	1460	1225	PVR
1.923215	5.44E-05	0.000327	up	34	36	48	12	9	11	VIT
7.424105	0.0013166	0.00546	up	213	68	1	0	2	0	MUC12
1.737415	1.09E-06	9.60E-06	up	114	71	112	37	32	23	PDE7B
1.404991	1.94E-06	1.62E-05	up	696	653	1386	411	331	296	FAM126A
5.292668	0.0011936	0.005025	up	3	8	10	0	0	0	WIPF3
1.03675	1.28E-11	2.48E-10	up	1878	1756	1868	835	1044	934	GNA12
9.441804	1.13E-252	########	up	14576	####	####	21	24	18	KRT81
3.902895	0.0035145	0.012627	up	11	11	6	1	1	0	
4.313081	0.006402	0.020992	up	5	4	12	1	0	0	ERVV-2
5.037448	0.0031696	0.011565	up	5	9	3	0	0	0	C15orf59
5.79791	2.21E-12	4.76E-11	up	77	78	59	4	0	0	MOV10L1
3.820937	8.46E-21	4.49E-19	up	365	317	592	48	32	11	CREB5
1.108782	1.43E-11	2.75E-10	up	2102	2250	2943	1088	1281	1141	MCU
1.066413	5.05E-05	0.000307	up	167	145	200	72	79	102	LOC344887
2.218873	1.51E-22	9.38E-21	up	470	451	553	78	166	95	IGFBP3
4.320851	9.55E-10	1.42E-08	up	51	39	46	3	0	4	GLIPR2
1.546894	8.23E-09	1.06E-07	up	144	199	205	58	64	73	TWIST1
1.481135	0.011118	0.033476	up	46	27	29	3	20	17	RPH3AL
1.75597	7.24E-23	4.70E-21	up	3932	4440	3756	1100	1593	1126	TMSB4X
1.200781	0.0001452	0.000785	up	4873	4096	6743	3265	1769	1758	EGFR
2.75201	0.0009432	0.00408	up	206	152	327	67	17	13	INHBA
1.600803	2.27E-13	5.50E-12	up	588	641	604	143	300	207	PIR
1.554459	1.41E-06	1.21E-05	up	322	257	296	141	81	80	PIK3CD
2.380512	8.61E-10	1.29E-08	up	73	67	68	12	15	15	
1.400856	2.81E-05	0.000181	up	188	141	211	89	49	68	BCL2
5.327874	6.61E-15	1.98E-13	up	64	76	90	1	2	3	
1.37686	1.00E-07	1.08E-06	up	190	271	332	77	143	103	MTMR11
1.697425	2.46E-09	3.42E-08	up	972	877	2061	420	428	374	
3.943948	0.0005522	0.002546	up	12	10	25	3	0	0	DPF3

1.547167	2.08E-07	2.11E-06	up	550	576	1174	311	262	220	MPDZ
2.32627	1.20E-21	6.96E-20	up	1161	982	1647	294	241	233	CALD1
1.645104	5.16E-20	2.49E-18	up	1633	1393	1924	534	562	540	ACO1
1.939009	0.000736	0.003287	up	34	38	24	8	5	13	BBOX1-AS1
4.664993	0.014627	0.042004	up	1	3	10	0	0	0	
1.112388	2.14E-11	3.98E-10	up	6335	6417	7964	2455	4093	3570	HMGN1
1.805183	1.64E-11	3.12E-10	up	199	213	175	44	81	55	TMEM51
1.344743	4.46E-07	4.25E-06	up	229	218	171	64	113	84	HIST3H2A
2.259392	0.0005595	0.002574	up	41	43	57	21	6	2	CRLF2
3.512249	0.0023121	0.008867	up	14	8	11	2	1	0	SENCR
2.821148	1.56E-26	1.34E-24	up	240	285	398	33	57	47	TMEM136
11.82459	4.91E-30	5.52E-28	up	1943	2152	2849	1	1	0	SRGN
1.675868	1.12E-13	2.85E-12	up	371	440	370	100	146	145	
1.06662	1.88E-07	1.92E-06	up	286	286	401	134	185	166	MLLT3
1.004153	4.14E-06	3.21E-05	up	491	417	633	282	284	226	RNASEH1
8.133932	1.36E-10	2.26E-09	up	37	40	74	0	0	0	KLF17
3.419183	5.16E-72	2.73E-69	up	1568	1619	1633	135	158	178	HOXB9
1.020932	0.001842	0.00733	up	172	287	227	132	157	68	TRIML2
1.403019	5.14E-15	1.57E-13	up	4160	4327	6148	1454	2363	1996	ACSS2
8.794001	5.45E-17	1.99E-15	up	254	233	369	2	0	0	
1.647653	5.92E-30	6.47E-28	up	7296	8283	8846	2373	3070	2725	HLA-E
1.236268	6.50E-06	4.83E-05	up	630	570	1076	377	290	306	PLEKHA8
1.194259	4.95E-05	0.000301	up	694	686	1327	446	316	421	ANKRD18A
2.761709	3.93E-25	3.09E-23	up	2070	1846	3669	431	416	292	AKAP12
1.513607	8.46E-11	1.46E-09	up	317	383	325	104	129	144	ULBP2
1.251706	0.0015029	0.00613	up	43	45	59	18	20	26	HOXA13
1.412001	7.74E-11	1.34E-09	up	241	245	330	101	119	99	DEPDC7
1.775022	2.20E-13	5.37E-12	up	3192	3021	4531	1278	982	921	OSMR
1.480551	6.32E-15	1.91E-13	up	528	515	631	198	210	214	CTPS2
1.03979	9.46E-07	8.45E-06	up	3192	2529	4084	1767	1739	1392	ANTXR1
1.001794	0.0113415	0.034022	up	77	39	111	36	39	41	RGPD8
1.78929	5.42E-19	2.40E-17	up	543	624	854	185	226	196	TMEM133
5.528286	0.0004352	0.002062	up	5	6	14	0	0	0	
5.873968	6.19E-15	1.88E-13	up	86	113	73	1	4	0	C5orf60
2.981574	0.0028482	0.010556	up	16	9	12	0	2	3	GGN
1.17739	0.0032305	0.011769	up	90	107	54	23	55	43	F12
7.090221	8.72E-08	9.45E-07	up	18	21	34	0	0	0	FAR2P1
2.426015	0.0039691	0.013993	up	16	11	24	4	6	0	SH3BGRL
1.142917	0.0010409	0.004459	up	82	81	124	24	55	58	HOTAIR
1.114982	3.38E-07	3.30E-06	up	346	321	465	175	175	188	COMMD10
1.154005	1.54E-08	1.89E-07	up	2462	2860	4346	1407	1629	1441	ATG12
2.602146	4.29E-07	4.11E-06	up	45	54	37	4	10	10	TSLP
1.144894	6.13E-10	9.46E-09	up	479	501	620	236	263	252	ZNF227
1.011876	5.46E-11	9.69E-10	up	832	835	973	408	523	440	ZCCHC17

1.404249	1.26E-05	8.81E-05	up	86	78	134	40	41	35	GJA3
1.079592	6.10E-06	4.55E-05	up	1274	1258	2108	813	698	716	RASA1
1.065462	0.0005298	0.002457	up	195	172	310	137	108	83	TMTC2
1.789293	7.12E-06	5.24E-05	up	211	297	241	24	112	101	MINCR
1.147192	3.00E-05	0.000192	up	1645	1572	1167	402	1036	729	GADD45GIP1
1.827588	4.21E-05	0.00026	up	66	49	43	18	19	10	LOC100288181
4.819232	3.93E-08	4.48E-07	up	46	16	77	3	2	0	FAM196B
1.205391	9.05E-11	1.55E-09	up	3229	3272	2750	1112	1767	1404	MARVELD1
6.892205	8.26E-07	7.50E-06	up	13	11	41	0	0	0	
2.646962	3.49E-12	7.31E-11	up	80	99	144	10	21	23	RHOBTB1
1.773458	5.95E-06	4.45E-05	up	132	155	163	69	30	33	LOC100268168
10.08352	3.80E-17	1.41E-15	up	336	361	424	0	1	0	TMEM156
1.902953	1.90E-17	7.35E-16	up	1057	1073	1562	379	347	285	ARHGAP26
1.034656	0.0007573	0.003365	up	409	414	816	319	220	260	CSGALNACT2
4.058228	4.51E-25	3.51E-23	up	220	233	158	7	23	10	TMEM54
6.373679	1.29E-05	8.98E-05	up	15	5	25	0	0	0	
4.421771	2.31E-05	0.000152	up	23	20	20	3	0	0	MTSS1
2.150839	1.40E-16	4.99E-15	up	297	255	428	81	73	72	REPS2
1.004212	1.70E-08	2.08E-07	up	764	694	983	389	452	421	LDLRAD3
2.918879	0.0042024	0.014687	up	12	7	19	2	0	3	PCDHGA10
3.757277	0.0053586	0.018066	up	6	8	12	1	1	0	
1.943527	2.80E-05	0.00018	up	43	41	39	7	14	13	PPM1J
1.630152	3.18E-18	1.32E-16	up	574	554	716	155	256	217	SH3BP5
1.358089	4.50E-05	0.000276	up	963	927	1766	660	358	391	HSPA13
2.948469	6.51E-53	2.25E-50	up	741	716	773	99	109	94	SERPINE1
1.114222	6.14E-08	6.81E-07	up	324	363	500	171	214	185	TPST1
3.056841	5.95E-51	1.88E-48	up	6772	8567	####	1079	1335	990	TM4SF1
6.553734	1.39E-21	8.00E-20	up	198	210	119	3	3	0	OSCAR
1.506993	0.0004736	0.002221	up	43	51	71	10	23	28	PAQR8
1.79947	2.10E-14	5.93E-13	up	1771	1433	2643	580	536	593	TANC2
5.638736	0.0002304	0.001181	up	11	9	6	0	0	0	MYZAP
1.069598	1.11E-07	1.18E-06	up	1846	1772	1620	637	1005	1007	ZNF768
1.145021	3.96E-05	0.000247	up	195	195	179	53	118	105	TMEM42
4.831694	7.28E-15	2.17E-13	up	87	54	189	4	7	1	PGM5P2
1.045445	3.01E-05	0.000192	up	243	185	312	108	122	140	ZNF35
1.53254	1.49E-09	2.16E-08	up	515	528	593	239	176	163	IFFO2
2.653275	0.0012094	0.005084	up	12	19	26	6	1	2	
4.885993	7.32E-12	1.46E-10	up	68	45	57	2	1	3	S1PR1
1.619478	3.27E-11	5.94E-10	up	231	184	227	64	74	80	TIGD2
1.960028	2.69E-17	1.02E-15	up	564	588	440	133	170	131	FZD2
1.062028	5.79E-05	0.000347	up	116	136	190	69	84	67	SNAPC1
1.183882	0.0100415	0.03079	up	46	64	34	16	20	31	
7.140805	1.33E-08	1.65E-07	up	58	40	47	1	0	0	ANO2
3.157068	0.0023479	0.00898	up	9	9	17	2	1	1	PCDHGB2

2.093623	1.59E-37	2.53E-35	up	1329	1222	1568	288	428	301	WWC3
1.382734	1.78E-05	0.000121	up	1028	977	2256	674	465	484	SCML1
4.01169	1.44E-07	1.50E-06	up	31	27	35	3	2	1	PCDHGA1
1.268901	0.0013406	0.005544	up	52	49	42	16	25	22	CD177
3.221067	3.05E-20	1.51E-18	up	232	172	150	13	30	21	UQCC3
2.017089	6.19E-23	4.09E-21	up	1693	1386	1802	290	488	494	MEST
3.816027	6.34E-105	########	up	2662	2985	3468	224	246	203	GFPT2
5.066101	0.0104205	0.031732	up	10	7	0	0	0	0	PCDHA2
1.01233	4.00E-05	0.000249	up	446	356	586	260	217	224	ZNF184
1.414351	2.08E-22	1.27E-20	up	1804	1841	2073	629	868	757	WDR70
2.280614	5.81E-19	2.56E-17	up	492	380	331	76	97	89	FJX1
2.479927	2.32E-16	7.96E-15	up	831	966	1201	246	195	108	LINC00707
1.608945	1.08E-05	7.67E-05	up	79	69	129	26	27	40	
1.037458	6.36E-10	9.75E-09	up	1303	1294	1366	659	780	584	STK10
1.106445	7.49E-07	6.86E-06	up	777	765	1193	452	412	432	SIRT1
2.732265	7.17E-06	5.27E-05	up	23	34	47	8	5	3	SLC1A1
1.29167	2.02E-16	6.98E-15	up	4166	4449	5913	1791	2353	2042	BZW1
1.571788	1.63E-12	3.59E-11	up	383	316	434	94	155	152	STRADB
1.593486	9.99E-15	2.94E-13	up	6320	5565	8397	2491	2267	2114	EPAS1
2.057321	2.35E-16	8.06E-15	up	495	376	664	94	135	154	FKTN
2.04667	6.90E-10	1.05E-08	up	282	219	254	88	52	46	HTR1D
1.957501	5.17E-16	1.70E-14	up	509	468	689	170	145	124	HIST1H2AC
6.64199	2.70E-05	0.000174	up	39	10	2	0	0	0	CIITA
1.337392	1.03E-05	7.29E-05	up	96	88	103	30	57	34	HIST1H2BF
1.264013	6.83E-06	5.05E-05	up	802	578	1299	426	364	337	ARID2
1.163835	1.44E-05	9.89E-05	up	211	216	380	127	124	117	FAM72C
1.891465	2.17E-09	3.05E-08	up	177	161	237	66	62	32	EVC
1.5393	2.13E-16	7.34E-15	up	774	829	946	310	310	288	GID4
1.858745	9.32E-09	1.19E-07	up	319	434	342	50	133	142	STARD10
1.305145	0.0077017	0.024567	up	22	33	36	10	18	11	KHDRBS3
4.84299	5.62E-12	1.14E-10	up	49	50	66	2	2	2	KRT34
2.329308	1.51E-11	2.88E-10	up	197	151	151	45	27	30	MB21D2
4.747167	0.007549	0.024149	up	7	4	3	0	0	0	ZNF594
2.531122	1.64E-12	3.62E-11	up	877	664	1472	255	144	117	MAP1B
3.653596	0.0077768	0.02476	up	9	4	12	1	0	1	PRUNE2
5.464163	2.82E-12	5.98E-11	up	58	42	115	2	2	1	ABCA1
1.359144	4.52E-07	4.31E-06	up	184	162	292	76	87	93	CNTNAP3
1.791261	1.66E-11	3.14E-10	up	208	202	227	66	85	43	LETM2
1.189223	9.00E-05	0.000512	up	182	156	339	108	110	85	ZNF567
1.552074	4.06E-10	6.37E-09	up	767	881	859	171	386	359	ALKBH2
4.542395	0.0044261	0.015347	up	2	13	9	0	1	0	C10orf105
9.298313	3.89E-14	1.07E-12	up	99	137	94	0	0	0	
1.32562	0.0147123	0.042198	up	27	16	48	12	12	13	
1.209497	6.14E-05	0.000365	up	1495	1372	3035	1011	732	802	RALGPS2

1.619415	0.000152	0.000816	up	175	144	322	111	53	41	JAK2
2.308068	1.87E-21	1.07E-19	up	567	475	883	112	141	148	PRKAB2
2.708441	0.0029179	0.010781	up	9	11	18	2	2	2	ELMO1
1.225998	3.97E-07	3.83E-06	up	208	277	228	92	138	94	PCED1B
1.730747	1.08E-22	6.89E-21	up	2809	3018	2683	821	952	915	QSOX1
1.209009	1.13E-06	9.90E-06	up	895	874	1184	518	369	407	CHSY1
1.355299	9.29E-07	8.31E-06	up	633	441	804	304	245	197	SVEP1
1.417705	0.015553	0.044176	up	39	25	43	24	10	6	PIPSL
1.414677	0.0002241	0.001153	up	350	335	349	203	132	60	CDH5
1.240153	1.42E-17	5.54E-16	up	3645	4128	4528	1522	2151	1804	PDHA1
2.202731	0.0008424	0.003694	up	25	12	40	6	5	6	UBE2Q2P1
2.682581	4.35E-15	1.34E-13	up	139	146	193	26	17	33	LHX6
1.043903	5.02E-09	6.71E-08	up	948	1108	1421	514	647	593	PTGR1
1.545038	4.89E-07	4.63E-06	up	363	314	603	177	115	147	PIGA
1.33691	0.0021189	0.008252	up	49	38	48	24	17	14	ECM2
1.55157	1.39E-07	1.46E-06	up	195	225	247	99	66	67	TLE4
1.575861	0.0004233	0.002015	up	57	53	35	17	15	19	
1.422067	2.41E-08	2.87E-07	up	230	171	246	62	120	75	LINC00667
2.055716	1.12E-10	1.89E-09	up	182	146	259	31	80	40	PXYLP1
1.547808	1.66E-09	2.39E-08	up	2107	1878	3343	1003	901	647	ERRFI1
2.02575	8.84E-22	5.17E-20	up	485	468	553	87	162	143	ARSG
2.316893	5.70E-16	1.85E-14	up	519	416	784	143	121	87	ARSJ
6.931782	2.84E-07	2.80E-06	up	30	17	17	0	0	0	MAP3K15
1.985459	1.36E-06	1.18E-05	up	298	396	196	46	159	49	CITED4
1.336162	3.72E-09	5.06E-08	up	723	766	633	200	405	301	ENG
1.517478	0.0013335	0.00552	up	38	33	56	16	10	19	ZNF792
1.442159	1.66E-07	1.71E-06	up	173	185	317	86	97	73	MAOA
6.748207	9.46E-07	8.45E-06	up	16	27	13	0	0	0	INSL3
2.830054	0.0176828	0.049131	up	9	10	10	4	0	0	
1.570054	8.31E-06	6.02E-05	up	137	155	116	23	74	53	TRIM52-AS1
1.187801	0.0001568	0.000838	up	92	76	115	28	54	49	
1.020327	2.02E-10	3.31E-09	up	1175	1311	1619	595	853	676	TFPI
1.85129	9.80E-21	5.10E-19	up	386	351	424	90	143	107	OTUD1
2.000273	2.03E-07	2.05E-06	up	211	192	270	90	43	35	GATA6
1.93919	2.57E-15	8.08E-14	up	197	183	255	44	71	59	PSTK
1.601025	0.006895	0.022373	up	14	26	34	8	7	10	
1.459857	0.0060857	0.020101	up	31	48	26	8	24	10	
1.029035	0.0158165	0.044751	up	36	46	41	13	33	19	
1.208648	1.14E-13	2.90E-12	up	8437	7468	8626	3669	3995	3383	NFE2L1
1.853172	9.20E-09	1.17E-07	up	78	89	103	24	28	26	C10orf10
1.810999	9.67E-21	5.05E-19	up	689	685	930	196	234	251	ZCCHC24
2.773936	1.78E-14	5.08E-13	up	98	128	99	17	18	15	SH2D5
3.499357	1.22E-08	1.52E-07	up	37	36	44	2	6	3	TTYH2
1.03376	6.97E-12	1.39E-10	up	3688	3526	3712	1488	2299	1869	INPPL1

1.560318	2.03E-11	3.80E-10	up	1127	1113	1524	515	427	361	ARHGEF28
1.685954	3.04E-10	4.86E-09	up	167	205	246	43	84	76	CCDC40
1.050913	1.53E-05	0.000105	up	637	615	1151	383	400	401	DDIAS
2.325513	0.008476	0.026683	up	15	15	8	4	3	1	ASGR1
1.633584	0.0122928	0.036401	up	14	22	19	3	9	7	
1.71328	9.91E-09	1.25E-07	up	112	91	117	33	38	31	TMCC3
1.126361	0.0004267	0.002029	up	3702	3852	7427	3049	1793	1947	TRAM1
6.185117	1.60E-05	0.000109	up	13	8	18	0	0	0	
2.071301	0.0022455	0.008667	up	37	23	16	4	4	11	DBNDD1
1.250896	8.46E-08	9.18E-07	up	421	388	388	116	254	172	CACNB1
1.27823	3.05E-10	4.87E-09	up	1720	1664	2297	873	799	729	PHKA1
1.327783	2.36E-19	1.08E-17	up	6925	7134	7265	2419	3477	3070	TNFRSF1A
2.945241	2.23E-19	1.03E-17	up	992	1050	948	61	147	206	P3H4
1.539435	1.78E-07	1.83E-06	up	163	128	207	37	70	73	PRSS53
1.017056	4.14E-05	0.000256	up	585	531	892	380	314	313	PMAIP1
10.62774	2.01E-18	8.51E-17	up	239	208	403	0	0	0	SCML2
2.35443	1.20E-10	2.02E-09	up	165	133	316	51	39	31	DNAJC6
1.166525	0.0002199	0.001134	up	94	109	93	34	70	38	FBXO6
1.4221	0.0001783	0.000938	up	1210	1312	2810	958	428	550	SLC4A7
1.468159	0.0025654	0.009653	up	39	24	45	8	16	17	ZMIZ1-AS1
1.162153	1.37E-13	3.44E-12	up	3503	3488	4590	1686	1949	1734	BMS1
1.079078	4.30E-06	3.32E-05	up	281	242	430	142	177	148	KIAA1462
3.731853	0.0050117	0.017057	up	8	8	10	1	0	1	
1.182992	0.0019362	0.007647	up	97	55	110	45	51	24	GPR158
1.609431	1.04E-09	1.54E-08	up	1869	2013	3282	956	727	684	TMEM263
1.004074	0.0013332	0.00552	up	132	146	100	44	81	77	C9orf9
1.106417	1.47E-06	1.27E-05	up	176	217	224	81	113	107	ZDHHC14
5.769897	8.07E-68	3.83E-65	up	774	829	753	11	9	25	FKBP10
1.594413	1.78E-22	1.10E-20	up	5653	5942	6713	1555	2687	2192	SMS
1.799869	3.58E-10	5.66E-09	up	1068	1261	925	173	441	400	TAZ
1.680226	1.97E-11	3.69E-10	up	215	238	256	49	105	82	WLS
1.427504	4.65E-17	1.71E-15	up	1578	1482	1512	567	702	519	GADD45A
1.106299	2.08E-05	0.000139	up	339	397	623	193	198	254	CD58
1.428777	2.21E-17	8.45E-16	up	1202	1293	1419	395	583	552	METTL22
2.583256	0.0001671	0.000887	up	22	19	22	4	4	3	
2.503723	1.09E-09	1.61E-08	up	966	789	2099	351	155	153	ARHGAP42
1.946556	1.11E-08	1.39E-07	up	270	270	590	119	79	94	TRAF6
1.013062	5.90E-06	4.42E-05	up	353	260	388	165	173	175	DIP2C
1.061105	2.03E-10	3.32E-09	up	3143	2965	2819	1226	1763	1541	PACS1
3.691045	8.87E-07	7.99E-06	up	40	16	61	5	1	3	KLHL4
1.843528	4.98E-10	7.75E-09	up	139	160	129	29	51	47	LRFN3
1.021774	3.76E-05	0.000236	up	232	238	222	81	173	113	PDCD2L
1.638762	7.66E-17	2.76E-15	up	1663	1658	2040	566	547	659	TUBGCP3
3.387687	1.85E-06	1.56E-05	up	52	47	30	1	12	1	LOC113230

3.236881	0.0118302	0.035285	up	12	4	13	3	0	0	CACNA1A
1.175736	4.61E-06	3.54E-05	up	515	448	769	301	245	232	JADE3
1.028613	7.07E-06	5.21E-05	up	640	542	731	206	399	388	KIF21B
4.480877	0.0165725	0.046457	up	2	4	6	0	0	0	
2.238976	4.38E-25	3.43E-23	up	544	570	640	85	181	131	TTC7B
2.298895	6.41E-10	9.81E-09	up	70	95	90	11	23	21	
2.565111	0.0012703	0.0053	up	11	16	31	5	4	1	AGBL2
1.088307	0.0061499	0.020267	up	40	45	67	19	24	31	SMCO2
1.616618	0.0040078	0.014113	up	31	19	27	8	7	11	MID1
1.631864	2.33E-07	2.34E-06	up	226	209	255	98	54	73	LHX1
1.551819	0.0015495	0.006286	up	32	36	53	20	13	9	LOC101928820
1.138492	1.54E-06	1.32E-05	up	284	232	360	138	132	139	MAGI1
4.685166	0.001776	0.007099	up	6	7	14	0	0	1	ACTN3
1.094716	3.95E-14	1.08E-12	up	2325	2147	2645	1061	1328	1095	EXT2
5.754879	1.79E-05	0.000121	up	17	16	23	0	1	0	CHST1
2.186216	4.76E-11	8.50E-10	up	166	174	270	58	34	43	SYTL4
2.916124	0.0105395	0.032018	up	7	7	14	0	3	1	SNORA60
1.46297	5.39E-19	2.39E-17	up	1690	2049	1954	640	840	693	MAGED2
1.22046	7.20E-09	9.39E-08	up	349	363	326	133	203	138	PORCN
2.310502	1.16E-12	2.60E-11	up	178	204	219	33	31	61	NRIP3
1.247894	0.0001802	0.000948	up	1179	1136	2340	885	503	547	ANKRD50
1.74313	1.64E-05	0.000112	up	1075	1037	1966	664	250	268	NEK7
2.642981	1.62E-15	5.14E-14	up	455	389	607	112	56	64	NALCN
1.019173	0.004702	0.016142	up	108	90	65	29	67	45	DLX2
1.056382	3.18E-05	0.000202	up	573	552	400	183	307	295	ZNRD1
3.586894	0.0027642	0.010281	up	35	12	4	0	5	0	PCDHB16
1.834912	0.0010761	0.004582	up	30	33	23	10	6	9	
4.624345	0.0023286	0.008919	up	12	8	5	1	0	0	DOC2B
4.157398	0.001651	0.006652	up	9	8	18	0	0	2	SULT1C2
1.086275	0.0085149	0.026792	up	42	36	66	26	27	17	ZXDA
2.144628	3.58E-06	2.81E-05	up	75	76	40	9	22	16	CTF1
2.4961	1.20E-14	3.49E-13	up	113	107	151	22	21	25	CAPN5
1.581017	8.72E-18	3.47E-16	up	447	472	583	143	208	176	PAK1
1.599279	3.74E-10	5.90E-09	up	681	650	1141	311	261	255	SOWAHC
1.164625	0.0001599	0.000854	up	459	541	416	107	308	272	CTU2
3.472653	2.70E-77	1.71E-74	up	4927	4901	4470	347	615	419	SERPINH1
6.674412	1.56E-07	1.63E-06	up	36	34	35	0	0	1	ENDOD1
1.579227	0.0043676	0.015178	up	28	32	23	13	10	6	REM2
3.045965	5.20E-10	8.08E-09	up	79	50	61	6	6	12	SNPH
1.949905	4.56E-20	2.21E-18	up	760	684	1118	227	244	212	RNF24
3.752645	3.64E-09	4.97E-08	up	53	29	59	4	6	1	ISM1
2.249824	5.68E-12	1.15E-10	up	414	337	686	135	85	82	FRMD6
1.857952	2.00E-11	3.75E-10	up	2076	2130	3226	898	688	490	RND3
5.146479	0.0093169	0.028867	up	13	5	0	0	0	0	DCSTAMP

3.482438	5.71E-19	2.52E-17	up	158	211	262	21	9	27	GEM
1.945654	2.49E-14	7.00E-13	up	1958	1727	3507	660	608	623	ZAK
1.212099	0.0002026	0.001052	up	1731	1572	3172	1283	758	727	PDP1
1.065063	5.34E-09	7.13E-08	up	3482	3241	4754	1872	1948	1826	SEL1L3
1.621753	0.0039244	0.013882	up	33	18	47	13	7	12	CCDC171
1.941182	1.54E-10	2.57E-09	up	176	186	156	28	57	59	SNHG11
1.005957	0.0002652	0.001339	up	2778	2581	5601	1932	1686	1873	ZKSCAN8
5.892804	2.15E-37	3.38E-35	up	249	254	276	1	10	3	CSF2RA
1.260632	3.67E-09	5.00E-08	up	266	315	345	108	182	120	
1.15079	3.81E-05	0.000239	up	186	236	359	80	151	138	
1.126542	0.0176928	0.049145	up	42	53	33	9	31	24	MIR503HG
2.15912	2.67E-23	1.83E-21	up	506	577	493	93	163	121	DEF8
2.091416	0.0002757	0.001388	up	23	32	27	8	6	6	BMP4
1.076136	0.0002538	0.001288	up	2563	2211	5094	1849	1503	1342	ATM
2.300168	2.34E-09	3.27E-08	up	3989	3633	5211	1449	663	461	CDH13
6.7469	1.23E-07	1.31E-06	up	27	41	43	1	0	0	RTN1
1.245926	3.67E-09	5.00E-08	up	2520	2307	2861	1012	988	1336	POFUT1
4.105607	1.26E-20	6.48E-19	up	121	117	146	13	7	3	NLRP1
1.781529	2.24E-15	7.06E-14	up	37280	####	####	14350	####	####	TXNRD1
1.193421	0.0157318	0.044558	up	23	25	50	13	15	16	
1.353905	1.69E-06	1.43E-05	up	28506	####	####	17048	####	####	ASPH
1.137952	1.30E-05	9.07E-05	up	2912	2787	4382	1894	1310	1403	GFPT1
1.004038	2.00E-07	2.03E-06	up	3236	2899	4548	1342	2363	1907	FADS1
1.916231	1.86E-16	6.51E-15	up	672	638	541	118	216	191	TKFC
4.925249	2.14E-08	2.57E-07	up	51	38	26	2	1	1	GDF1
2.000408	5.08E-38	8.48E-36	up	15043	####	####	3662	4109	3638	HYOU1
1.194991	8.76E-09	1.13E-07	up	592	636	502	231	311	259	MIF4GD
2.443935	3.48E-05	0.00022	up	33	39	28	10	5	4	HS3ST3B1
1.078064	1.33E-13	3.34E-12	up	5980	6528	7900	2902	3826	3375	VAPA
1.46971	0.0009172	0.003979	up	83	46	139	42	36	20	ZFHX4
1.846852	4.95E-13	1.15E-11	up	331	477	451	137	129	97	IL1B
1.814016	3.19E-17	1.20E-15	up	555	428	731	147	194	166	LPIN2
1.431496	2.35E-12	5.06E-11	up	1049	1458	1700	417	703	528	REXO2
1.448483	1.55E-05	0.000106	up	659	742	1198	457	252	236	RBM7
1.164981	1.31E-08	1.63E-07	up	432	437	406	189	246	166	IL20RB
3.641634	0.0025689	0.009664	up	7	10	20	0	0	3	
1.32931	0.0025573	0.009626	up	55	40	50	27	16	16	NKAPP1
2.586668	1.29E-24	9.72E-23	up	1388	1930	1703	171	424	309	TPM2
1.131214	6.93E-11	1.22E-09	up	2611	2518	3554	1272	1421	1402	RALBP1
1.572244	4.47E-12	9.20E-11	up	939	1016	1619	432	440	360	MPZL2
1.830911	9.03E-08	9.76E-07	up	97	140	104	39	34	27	LYPD1
1.141481	0.0009003	0.003914	up	108	72	185	49	62	59	CCDC15
1.489883	1.10E-18	4.75E-17	up	3729	3558	3308	1077	1636	1298	EI24
2.020865	1.90E-18	8.07E-17	up	696	840	684	129	240	216	COMMD7

4.773473	0.0105179	0.031972	up	1	4	10	0	0	0	LAMA1
2.589882	8.44E-08	9.16E-07	up	169	150	165	51	18	11	SLCO2A1
7.001799	2.51E-07	2.49E-06	up	15	34	18	0	0	0	PTGIR
1.824438	7.48E-25	5.73E-23	up	861	892	1220	259	330	285	STK39
1.223536	6.83E-10	1.04E-08	up	586	573	528	196	298	272	B4GAT1
1.101177	4.68E-11	8.38E-10	up	4969	5529	5488	1955	3403	2588	MYL12A
1.001876	1.43E-11	2.75E-10	up	4104	3913	4762	1764	2616	2333	CALM1
1.490193	5.10E-09	6.81E-08	up	156	177	161	56	77	53	CCDC28B
1.177846	5.89E-05	0.000352	up	90	88	119	36	49	52	ZBTB8A
1.225267	1.91E-10	3.16E-09	up	3140	3089	4152	1613	1508	1435	DENND5A
1.699411	2.35E-08	2.81E-07	up	1922	1840	2432	906	493	508	PAPSS2
1.673402	2.10E-06	1.74E-05	up	67	73	76	20	34	18	CCNJL
1.079816	1.52E-13	3.79E-12	up	7412	6864	8244	2986	4305	3910	PSAT1
2.187961	0.0001539	0.000825	up	22	24	37	6	8	5	FAM189A2
6.067799	6.52E-48	1.79E-45	up	326	337	435	9	5	3	ADAM19
1.085741	3.43E-14	9.47E-13	up	7956	8603	####	3810	5118	4409	PRSS23
1.370677	6.15E-10	9.47E-09	up	389	445	483	119	251	175	C9orf40
1.373929	9.90E-12	1.94E-10	up	917	889	821	256	477	354	TNS2
1.711812	9.81E-05	0.000553	up	39	39	73	11	19	18	DLG2
1.548828	1.59E-11	3.04E-10	up	408	517	749	162	252	185	GPCPD1
1.580315	5.61E-20	2.69E-18	up	1381	1140	1499	381	524	503	SH3PXD2B
1.118088	6.94E-05	0.000406	up	1714	1508	3244	1116	890	983	KIAA1551
1.051015	2.53E-06	2.06E-05	up	253	225	349	109	161	147	POLI
1.237703	2.40E-05	0.000158	up	767	702	1573	412	383	505	CEP290
1.031526	2.83E-05	0.000182	up	6620	6266	####	4324	3291	3838	SRP72
1.094826	3.10E-05	0.000198	up	1003	936	638	291	581	437	RIN1
1.332778	0.0001741	0.000919	up	551	434	901	354	172	211	FAM169A
5.500288	2.62E-59	1.06E-56	up	447	417	535	16	9	7	ALPK2
7.317607	0.003118	0.011414	up	62	18	1	0	0	0	ZNF521
2.188928	3.91E-26	3.23E-24	up	554	552	824	119	167	155	FAN1
2.616029	5.54E-47	1.39E-44	up	5852	6492	5599	835	1369	927	CSRP1
1.19172	0.0071243	0.023	up	46	60	125	39	46	19	STC1
2.686562	0.0010554	0.004508	up	14	14	20	1	5	2	STAG3
5.532249	1.87E-17	7.26E-16	up	110	108	83	1	5	1	WNT5B
1.432148	5.15E-11	9.17E-10	up	1488	1321	2068	665	590	589	ELK3
2.436937	3.21E-25	2.54E-23	up	575	562	819	138	140	95	TBX3
1.141799	2.77E-05	0.000179	up	144	144	156	68	61	79	CBS
2.330277	1.24E-19	5.80E-18	up	178	215	237	38	56	38	CCDC80
1.275801	1.56E-06	1.33E-05	up	2776	2405	4454	1556	1140	1301	ARHGAP11A
1.90827	5.63E-16	1.84E-14	up	516	528	870	180	196	149	MID1
1.0138	5.87E-08	6.54E-07	up	496	539	698	241	338	318	ZNF277
1.25064	7.26E-08	7.96E-07	up	1164	1242	1117	318	677	596	DTD1
1.642858	2.15E-05	0.000143	up	88	122	71	18	40	39	SELM
1.260526	1.27E-14	3.69E-13	up	1697	1540	1589	571	857	706	DENND4B

1.169305	5.47E-12	1.11E-10	up	1440	1573	1697	568	829	806	NKAP
1.411316	6.64E-14	1.76E-12	up	1042	1137	1378	404	461	521	TSEN15
1.083197	2.81E-07	2.77E-06	up	809	788	1234	419	454	500	RSRC1
1.633957	1.92E-20	9.66E-19	up	1482	1432	1506	369	663	488	CMSS1
2.069057	1.28E-12	2.84E-11	up	182	217	199	47	73	32	ITGB2
1.516048	8.26E-23	5.29E-21	up	1323	1221	1379	410	591	447	RUSC2
4.028599	0.0020943	0.008175	up	8	9	14	0	1	1	STKLD1
1.246683	4.57E-12	9.38E-11	up	2454	2210	2559	1100	1102	953	TBL1X
1.366315	1.81E-10	2.99E-09	up	2698	3113	2508	776	1430	1253	OAF
2.42091	9.79E-12	1.92E-10	up	138	127	146	11	35	36	GPR173
1.28875	1.71E-14	4.90E-13	up	778	730	856	319	366	324	SIM2
1.124328	7.20E-08	7.90E-07	up	740	771	1134	374	413	462	RINT1
1.318898	2.28E-16	7.86E-15	up	3934	3583	3991	1563	1715	1527	UBE2Z
2.222489	6.67E-16	2.16E-14	up	1691	1649	2923	558	413	379	SEC24D
2.60892	7.63E-30	8.24E-28	up	567	599	500	71	111	108	SEZ6L2
1.502831	0.0007201	0.003225	up	56	46	33	14	22	15	ASPHD1
1.248702	7.86E-07	7.17E-06	up	473	338	666	194	208	235	DCLRE1A
1.09946	0.0004429	0.002094	up	3923	3941	6289	3107	2122	1404	NT5E
1.435265	7.33E-08	8.04E-07	up	335	438	751	171	233	179	HMGA2
1.197233	0.0001056	0.000591	up	79	72	94	29	42	41	
1.629828	1.73E-08	2.11E-07	up	148	163	128	36	67	49	CCDC22
1.454678	0.0001315	0.000718	up	50	60	89	20	24	31	PRKD1
1.060577	0.0084236	0.026545	up	89	121	68	22	69	55	C9orf116
2.193126	2.02E-31	2.42E-29	up	2771	2706	2480	448	776	635	KLC2
2.097987	3.39E-14	9.38E-13	up	725	863	554	153	261	128	IL32
1.393053	8.86E-09	1.14E-07	up	12843	####	####	5984	6003	3396	TGM2
2.153481	4.08E-10	6.41E-09	up	400	532	541	44	180	137	
2.457	5.39E-07	5.05E-06	up	95	74	174	35	18	9	MAML2
1.027943	1.51E-06	1.29E-05	up	982	1031	1359	608	524	558	PRPS2
1.334253	4.21E-10	6.59E-09	up	754	722	847	345	379	237	CSF1
2.044383	3.86E-12	8.03E-11	up	128	111	152	23	42	35	MOSPD1
2.41708	5.07E-08	5.71E-07	up	98	109	117	35	14	12	PSG4
3.322178	3.11E-33	3.92E-31	up	312	441	437	44	53	28	
1.229557	5.62E-06	4.24E-05	up	1854	1968	2643	1076	679	1016	TPD52
2.724514	5.38E-14	1.44E-12	up	131	109	115	24	20	12	PRR5L
1.90135	2.06E-08	2.48E-07	up	547	379	843	218	141	114	ITPR1
2.321577	0.0001903	0.000996	up	21	32	24	6	4	6	B4GALNT1
2.153075	8.00E-14	2.10E-12	up	138	187	169	33	53	32	C1R
1.690095	6.22E-14	1.65E-12	up	1083	839	1038	359	328	261	NFIX
5.065969	2.73E-08	3.22E-07	up	33	67	24	1	2	1	PRPH
3.377288	1.32E-12	2.93E-11	up	64	72	62	6	6	8	BRSK1
1.796148	1.28E-13	3.23E-12	up	216	268	283	72	101	60	COX6B2
1.177054	7.29E-10	1.10E-08	up	8436	8828	####	4559	5059	4378	COPB2
1.782875	0.0001098	0.000612	up	45	70	36	12	19	16	TBX2-AS1

1.641259	7.73E-06	5.64E-05	up	282	342	171	46	117	115	PTP4A3
5.979941	6.74E-05	0.000396	up	6	18	9	0	0	0	
3.57739	8.94E-05	0.000509	up	22	18	15	0	3	2	TMEM255B
1.459179	6.20E-08	6.87E-07	up	128	140	194	52	59	63	HIST1H2BN
1.026213	0.0015862	0.00642	up	343	276	459	242	131	155	ZFP1
1.129821	2.23E-05	0.000147	up	248	207	174	82	109	113	HDDC3
2.046734	2.42E-25	1.93E-23	up	630	741	683	169	199	154	SOCS3
1.676638	6.41E-15	1.93E-13	up	1327	1557	1220	322	599	459	TMEM173
1.654002	2.61E-16	8.90E-15	up	1904	1680	2168	421	884	649	MCAM
1.731429	0.0155209	0.044098	up	32	20	23	1	7	16	
1.114091	0.0001261	0.000692	up	169	144	295	80	133	81	TMEM154
1.096218	2.51E-06	2.04E-05	up	749	635	1180	418	427	382	GNPTAB
1.543131	2.41E-07	2.41E-06	up	290	222	319	126	99	67	IRS1
2.040945	2.38E-14	6.71E-13	up	255	274	231	42	79	76	DYSF
1.639165	4.18E-05	0.000258	up	354	315	426	35	199	151	HIST2H2BE
1.583274	4.68E-22	2.79E-20	up	1315	1401	1330	400	562	465	ASMTL
1.659369	1.07E-12	2.42E-11	up	1778	2088	3292	822	830	667	MSMO1
1.043559	0.0001287	0.000703	up	143	107	121	59	71	59	ALX4
1.222394	0.0139744	0.040452	up	37	26	25	13	17	10	
1.918225	8.43E-15	2.49E-13	up	381	370	335	65	126	116	DGAT2
2.214612	8.96E-09	1.15E-07	up	87	96	77	10	25	25	TBX2
1.062198	7.94E-11	1.37E-09	up	2265	2266	2464	991	1230	1280	ATL3
6.297268	2.01E-11	3.76E-10	up	258	220	358	0	2	9	ARMC4
1.315875	1.10E-08	1.39E-07	up	230	238	329	80	139	118	ZNF354A
1.763162	5.09E-14	1.38E-12	up	206	189	225	52	82	59	EPDR1
2.327904	1.81E-05	0.000122	up	30	42	41	11	5	7	CPT1C
1.77794	1.01E-10	1.73E-09	up	214	266	300	45	110	88	MN1
1.611407	1.34E-07	1.41E-06	up	1567	1826	2971	859	500	711	GOLT1B
1.23836	5.36E-09	7.15E-08	up	2514	2988	2433	813	1603	1207	MVP
1.005141	1.45E-07	1.52E-06	up	1943	2230	3179	1207	1401	1178	PGM3
1.159131	2.96E-09	4.08E-08	up	3483	3260	2904	1122	2028	1490	ARHGEF1
1.443836	0.0073355	0.023601	up	29	17	49	9	13	14	CDKL5
2.871751	0.0073493	0.023637	up	11	8	9	1	1	2	FAM131B
1.12706	8.56E-06	6.17E-05	up	247	173	290	113	113	109	DUBR
2.324277	8.43E-16	2.71E-14	up	294	302	449	90	65	57	ADRB2
1.054646	0.0004342	0.002058	up	1642	1489	2577	1202	697	835	NMD3
1.374899	0.000248	0.001262	up	194	194	481	146	108	80	CHIC1
1.110432	0.0011521	0.004864	up	76	82	146	42	44	58	RBM43
1.040507	8.56E-12	1.69E-10	up	11061	####	####	5366	6351	6240	CRTAP
1.360748	3.96E-11	7.13E-10	up	386	390	522	131	204	195	ASF1A
1.907902	5.46E-12	1.11E-10	up	1281	951	1579	424	276	322	FAM129A
4.044028	0.0117228	0.035008	up	6	5	6	0	0	1	NPL
2.119869	6.87E-15	2.05E-13	up	150	164	205	31	46	48	SMIM8
3.978826	3.53E-49	1.05E-46	up	985	919	1442	92	69	55	GCNT2

2.355841	0.0002112	0.001092	up	25	39	30	4	3	12	CFB
1.454075	9.32E-14	2.41E-12	up	1338	1242	1383	374	734	445	GPATCH4
1.458517	1.13E-05	7.94E-05	up	1704	2067	2070	1061	688	407	PLAC8
6.520066	4.97E-07	4.70E-06	up	29	21	46	1	0	0	PRR16
1.250808	2.00E-11	3.76E-10	up	1792	2070	1837	814	1011	700	LY6K
1.528627	7.22E-11	1.26E-09	up	471	603	601	128	279	215	APOO
2.969655	4.96E-17	1.82E-15	up	196	127	286	28	22	29	ANK2
1.1335	9.11E-06	6.53E-05	up	284	301	487	177	159	162	CAMK2D
1.161382	6.21E-10	9.56E-09	up	691	704	681	273	344	357	PYURF
1.850104	2.54E-07	2.52E-06	up	67	73	83	24	20	20	SNCA
1.023292	0.0006547	0.002959	up	715	644	1349	549	399	385	SLC30A1
1.895372	9.99E-18	3.96E-16	up	240	252	316	65	85	77	DCLK2
2.728792	1.08E-37	1.75E-35	up	1213	1335	1714	237	208	214	GNE
1.161433	2.26E-06	1.86E-05	up	253	254	288	76	163	140	ARMC9
4.174086	0.010742	0.032524	up	7	2	10	1	0	0	PCDHGA12
1.471608	0.0030318	0.011133	up	48	37	27	9	15	19	LOC102723996
1.814341	0.0001287	0.000703	up	67	87	104	6	36	37	
6.084788	2.85E-05	0.000183	up	7	15	14	0	0	0	
1.56663	9.12E-19	3.98E-17	up	816	906	835	252	360	301	CPSF4
2.126815	7.13E-13	1.64E-11	up	114	114	158	27	33	32	ASAH2B
1.073253	6.82E-05	0.0004	up	474	524	925	338	286	300	RUFY2
1.494484	2.75E-12	5.87E-11	up	1967	1902	1836	465	1016	706	FMNL1
1.617175	5.00E-06	3.81E-05	up	102	124	133	53	45	23	CFAP57
1.089395	2.08E-10	3.40E-09	up	4418	4440	6354	2332	2673	2387	CDK1
2.323221	3.75E-17	1.40E-15	up	243	200	190	36	60	39	ORAI2
6.927275	2.53E-07	2.51E-06	up	16	28	20	0	0	0	RPLP0P2
1.032635	9.74E-05	0.000549	up	708	647	1176	492	439	327	PRDM10
1.05078	1.51E-05	0.000104	up	3319	3357	5081	2198	1652	1882	CCNA2
3.398508	0.0001643	0.000874	up	17	32	12	4	1	1	KISS1
1.013298	0.0002539	0.001289	up	205	181	319	131	109	115	PDGFC
1.746203	2.14E-09	3.02E-08	up	323	301	553	125	96	133	ERO1B
1.888064	0.0140736	0.040679	up	11	15	18	6	2	4	
1.070841	3.13E-08	3.64E-07	up	1882	1948	2919	1026	1135	1146	RDH11
1.231132	7.50E-05	0.000436	up	164	171	193	47	136	62	COL11A2
3.09536	1.05E-18	4.52E-17	up	215	163	278	35	30	14	CXCL8
4.380645	1.26E-24	9.52E-23	up	114	123	159	4	9	7	SDC2
4.113325	8.73E-29	8.83E-27	up	592	534	428	49	25	18	KRT86
1.180774	0.0047808	0.016365	up	35	53	50	20	18	25	
3.42771	1.22E-22	7.74E-21	up	189	210	320	32	21	15	ADAMTS16
2.474198	2.78E-31	3.30E-29	up	4513	4689	7318	1003	1292	798	GRAMD1B
1.658038	0.0019413	0.007666	up	36	29	56	9	8	22	
1.816878	1.00E-07	1.07E-06	up	152	258	197	77	59	42	CARD16
4.314495	0.0068414	0.022234	up	3	6	12	0	0	1	ASIC4
1.266464	0.000161	0.000858	up	4244	3615	6751	2805	1322	1846	CLIC4

1.615433	3.54E-12	7.38E-11	up	434	449	402	108	216	128	SPEG
1.344308	3.51E-12	7.34E-11	up	4070	4592	3827	1267	2179	1814	C6orf48
1.621777	7.56E-09	9.82E-08	up	495	488	893	239	182	193	METTL15
1.335303	2.54E-05	0.000165	up	1101	1357	1546	630	322	635	SDHD
2.933186	0.0101467	0.031043	up	6	18	5	2	1	1	KRT83
1.75555	2.02E-11	3.78E-10	up	157	150	180	51	60	40	PFKFB3
1.867508	1.27E-12	2.84E-11	up	314	334	239	66	100	93	SMAGP
2.091814	2.59E-10	4.19E-09	up	384	246	570	119	103	64	ZEB2
1.570781	0.0040001	0.014093	up	24	24	38	5	17	9	
1.99824	2.17E-13	5.30E-12	up	1105	1021	1427	394	266	239	CDH2

Supplementary Data 5, The 5' and 3' RACE data of seRNA LOC100506178 in S18 cells

5-RACE sequence data 1528bp

GACTGAAGGAGTAGAAAAAAGAAGAGGCCCTGTGAAGGGAGACCCGAGGGAGAGGTGTTGCAGGGAAAAAGGTCGT
GCATCAGGAAGTGTCATTTAAAAAGGACCTGAGTTGGGCTGAGTTATCAGCAAATACACGCCATGGCTATGCTCCC
CACCCCCCCACCGTACACTAACAGGGCAGCAGCTCTGATTGTCTAACGAGCAGCAGAGCCGCCCAGCAGCAACCAC
ATTAGGAAGGAATCGGAAACCAACGCTCCGCTGGCTGTCACCCCTTACCCACTTGCTGTCAGACCACAGCCTGAGG
CAGCCAAACCCATTTCCTTGGGGATCCAGCGCAAACCAGAGAGTGTGGACAGGCCGATGGAAGAATGTTGGACCAG
CTTTAAGAATGGGCTGTTCTAAGTCATGAATATTGTAGGAAGCAGCTCTAAGGACCACAGGGCTGGAGGGTGCACA
GGAAGGGGCCAGTGCATACAGGTGTCCAAACAGTGTCATCCGGTGTCCTGACATGCGGCCCCAATGGACTGATCCA
GGGTGGAAAGGAAGAGTGAGAAGAAATCAGACAGAAGAATTCAGGAGATGTGGCAACAGAGTGGATGTAGCAGAAG
GGACATAGAGAGTGCCTAAAGATGAGTCTTTGGTCAGCATGTGATGAAAAGAATGAAGACACTGGGGAAGTGGGGA
GCAGAAGCGGATGTGGGCCCAGGAGGAAGAAGATTCCAGAGAGCAGAGATGGCAAAAAGATGCATGGAAGAGAGAC
ACAGCAGGGTCGGGTTCTGGCCTCAGGAGCCAAGGGGGAAGTGCCTGAGGAGACCCCCAAATGAGGCCTGTGTCAA
TGAGGTCTTTAGGGGACTGGTTTCCATGTATGCCAGGCCATACTCCCTGGATTCCCAGCATTGAGAAGACCAAACA
CTTCTACACATTTATTACTGAAGCCCCCTGCAAGCTGAAGGCGGAGGAAGTGGCCAATCTCCTAAGCCGTGTGTGG
CTGAGGCAGTTGTCAGAGCTGTGATGAGGGAAATGCTCATTATTCTTCTATCCCTCACTTGGCCAAATAAAGAACT
TCTTAGAATTCTTCCCCTGTGACAAAACGGGGCTGTGATTTGGAAAGCCTCAGGCAAGTTTGCTAGGGCCGCGGTA
ACCAAGCACCACAGACTGGGTGGCTTCAAAACTGGACGGCTTCAACAGAAAGTTATTGTTGCTCAGTTCTGGAAGC
CAGAAGTCCAAAATCAAGGTACTTGCAGGGTCAGTTCGATCTGAGGGCTGCGAGGGAAGGATCTGTTGTAGACCTC
TCTCCTAGGTTTGCAGGCATTGGTCCTGATGTTCACAGGGCATCCTTCCTGTGCCTTCACATCATCTTCTCTCTGA
ATGTATCTCTGGGTCCAAATTTCCCTATTTTAAAATAAGGAAATCAGTCCTATTGGGTTAGCACCCCGCCCCTCAC
TTTAACTTGATTACCTCTGTAAAGACCCTACCTCCAAATAAGATCACATTCTGCGTACTGAGGGCTAGGACCTCAA
CATCTGAATTCAGGGTGGGTGGATG

*The box represents the 5-RACE splice sequence，# Green represents R2 primer sequence

Intermediate fragment of Loc100506178 sequencing data 1243bp

CTGGAAGCCAGAAGTCCAAAATCAAGGTACTTGCAGGGTCAGTTCGATCTGAGGGCTGCGAGGGAAGGATCTGTTG
TAGACCTCTCTCCTAGGTTTGCAGGCATTGGTCCTGATGTTCACAGGGCATCCTTCCTGTGCCTTCACATCATCTT
CTCTCTGAATGTATCTCTGGGTCCAAATTTCCCTATTTTAAAATAAGGAAATCAGTCCTATTGGGTTAGCACCCCG
CCCCTCACTTTAACTTGATTACCTCTGTAAAGACCCTACCTCCAAATAAGATCACATTCTGCGTACTGAGGGCTAG
GACCTCAACATCTGAATTCAGGGTGGGTGGATGCACAATTCATTCCATAACATTAAGAAGGAAGGGATAAGTGCCC

AAGGAATCACATGCTCGCTTGGGGAGAGTTCCAAGCGTGCAGAGCTGGTGGTCATAGCCTAGCCTCAGGCCTTCGG
AAATCCCCTGAATCTGGTTTTCCCAGTTCTCATAATCTCATTTCACCAAAATACGGCATCTCAATACATTCCATGC
ATTTCTGGTGACAAGCTCATTCTGGTGCACGCTAAGCAATGTGCTAACTACGTCTTAACTAGGGTGGTTTAACTGG
TTTGGTTAACATAGATCTCTGAGTCATTTCACGCATTCCTTAAGCCTAAACAAATCTCATCCAGGTGTTTAAGACA
TTGCACAATATTAGACCATCTGAAGCTCCATCTCCTTGGGGCAATGATGATGATGGTGATGAAAGTGGCTGGTGAC
TGTTCTCAGTCTCATCGCCATAATGGAACCCTACGTTGTGTTCCTCCACACTGGTGTGAGGGACACTACTAAGTGC
GGCAGAGTTTGCACTCAGGATAGAGAAATATGGACTCACGCACAGATTCCAGCTGAGTCCCTCCGATGTGCTAGGT
ACTATGTCCAGCCCCCCAGGATTCACCTGTAAGCAGGACAGTCTTTTGGATGACAGAGGGAATTCTCATATATCCA
CAGGCAGATGGAATTTACAAGTGGAAAGACCTAGAAGTTCAGAGACAGGGGGACTGAACTTTTTGCTTTCAGTCAG
AATCTTTGCTTACGGTTGTGCAAATGCTTGCATTGGTGGGTTTCCATGCCATATAACGTGCTACATATTTTTACGT
TACCCTTGCACATAAGCATACTGTTGGGAGGGAAAATGAAGAGGGTGGAGGTGGTAACAAACTCAAGCTAAGGAAA
TGGAATTTGGATGGGTACAAAGTCCAG

3-RACE sequence data 378bp

CGGTTGTGCAAATGCTTGCATTGGTGGGTTTCCATGCCATATAACGTGCTACATATTTTTACGTTACCCTTGCACA
TAAGCATACTGTTGGGAGGGAAAATGAAGAGGGTGGAGGTGGTAACAAACTCAAGCTAAGGAAATGGAATTTGGAT
GGGTACAAAGTCCAGAGAATTAGCTTGAGGAAAATGAACCAGGATTCACACAGGAGTTTTCAAACATCTCAATATC
AGGACCCCTTTACCCTCTTAAAACTTACTTCGTGAGGACCTTAAAGAGCTTTTGTTTGTGTGACTTATATGTATTA
ATATTTACTATATTAGAAATTAAAAGTAAGGAATGGTTGTAAAAAAAAAAAAAAAAAAACAAAAAAAAAAAAAAACA
CTGTCATGCCGTTACGTAGCG

The box represents the 3-RACE adaptor sequence; Yellow represents F2 primer sequence

Split joint sequence：2645bp

AAAGAAGAGGCCCTGTGAAGGGAGACCCGAGGGAGAGGTGTTGCAGGGAAAAAGGTCGTGCATCAGGAAGTGTCAT
TTAAAAAGGACCTGAGTTGGGCTGAGTTATCAGCAAATACACGCCATGGCTATGCTCCCCACCCCCCCACCGTACA
CTAACAGGGCAGCAGCTCTGATTGTCTAACGAGCAGCAGAGCCGCCCAGCAGCAACCACATTAGGAAGGAATCGGA
AACCAACGCTCCGCTGGCTGTCACCCCTTACCCACTTGCTGTCAGACCACAGCCTGAGGCAGCCAAACCCATTTCC
TTGGGGATCCAGCGCAAACCAGAGAGTGTGGACAGGCCGATGGAAGAATGTTGGACCAGCTTTAAGAATGGGCTGT
TCTAAGTCATGAATATTGTAGGAAGCAGCTCTAAGGACCACAGGGCTGGAGGGTGCACAGGAAGGGGCCAGTGCAT
ACAGGTGTCCAAACAGTGTCATCCGGTGTCCTGACATGCGGCCCCAATGGACTGATCCAGGGTGGAAAGGAAGAGT
GAGAAGAAATCAGACAGAAGAATTCAGGAGATGTGGCAACAGAGTGGATGTAGCAGAAGGGACATAGAGAGTGCCT
AAAGATGAGTCTTTGGTCAGCATGTGATGAAAAGAATGAAGACACTGGGGAAGTGGGGAGCAGAAGCGGATGTGGG
CCCAGGAGGAAGAAGATTCCAGAGAGCAGAGATGGCAAAAAGATGCATGGAAGAGAGACACAGCAGGGTCGGGTTC
TGGCCTCAGGAGCCAAGGGGGAAGTGCCTGAGGAGACCCCCAAATGAGGCCTGTGTCAATGAGGTCTTTAGGGGAC
TGGTTTCCATGTATGCCAGGCCATACTCCCTGGATTCCCAGCATTGAGAAGACCAAACACTTCTACACATTTATTA
CTGAAGCCCCCTGCAAGCTGAAGGCGGAGGAAGTGGCCAATCTCCTAAGCCGTGTGTGGCTGAGGCAGTTGTCAGA
GCTGTGATGAGGGAAATGCTCATTATTCTTCTATCCCTCACTTGGCCAAATAAAGAACTTCTTAGAATTCTTCCCC
TGTGACAAAACGGGGCTGTGATTTGGAAAGCCTCAGGCAAGTTTGCTAGGGCCGCGGTAACCAAGCACCACAGACT
GGGTGGCTTCAAAACTGGACGGCTTCAACAGAAAGTTATTGTTGCTCAGTTCTGGAAGCCAGAAGTCCAAAATCAA
GGTACTTGCAGGGTCAGTTCGATCTGAGGGCTGCGAGGGAAGGATCTGTTGTAGACCTCTCTCCTAGGTTTGCAGG
CATTGGTCCTGATGTTCACAGGGCATCCTTCCTGTGCCTTCACATCATCTTCTCTCTGAATGTATCTCTGGGTCCA
AATTTCCCTATTTTAAAATAAGGAAATCAGTCCTATTGGGTTAGCACCCCGCCCCTCACTTTAACTTGATTACCTC
TGTAAAGACCCTACCTCCAAATAAGATCACATTCTGCGTACTGAGGGCTAGGACCTCAACATCTGAATTCAGGGTG
GGTGGATGCACAATTCATTCCATAACATTAAGAAGGAAGGGATAAGTGCCCAAGGAATCACATGCTCGCTTGGGGA
GAGTTCCAAGCGTGCAGAGCTGGTGGTCATAGCCTAGCCTCAGGCCTTCGGAAATCCCCTGAATCTGGTTTTCCCA

GTTCTCATAATCTCATTTCACCAAAATACGGCATCTCAATACATTCCATGCATTTCTGGTGACAAGCTCATTCTGG
TGCACGCTAAGCAATGTGCTAACTACGTCTTAACTAGGGTGGTTTAACTGGTTTGGTTAACATAGATCTCTGAGTC
ATTTCACGCATTCCTTAAGCCTAAACAAATCTCATCCAGGTGTTTAAGACATTGCACAATATTAGACCATCTGAAG
CTCCATCTCCTTGGGGCAATGATGATGATGGTGATGAAAGTGGCTGGTGACTGTTCTCAGTCTCATCGCCATAATG
GAACCCTACGTTGTGTTCCTCCACACTGGTGTGAGGGACACTACTAAGTGCGGCAGAGTTTGCACTCAGGATAGAG
AAATATGGACTCACGCACAGATTCCAGCTGAGTCCCTCCGATGTGCTAGGTACTATGTCCAGCCCCCCAGGATTCA
CCTGTAAGCAGGACAGTCTTTTGGATGACAGAGGGAATTCTCATATATCCACAGGCAGATGGAATTTACAAGTGGA
AAGACCTAGAAGTTCAGAGACAGGGGGACTGAACTTTTTGCTTTCAGTCAGAATCTTTGCTTACGGTTGTGCAAAT
GCTTGCATTGGTGGGTTTCCATGCCATATAACGTGCTACATATTTTTACGTTACCCTTGCACATAAGCATACTGTT
GGGAGGGAAAATGAAGAGGGTGGAGGTGGTAACAAACTCAAGCTAAGGAAATGGAATTTGGATGGGTACAAAGTCC
AGAGAATTAGCTTGAGGAAAATGAACCAGGATTCACACAGGAGTTTTCAAACATCTCAATATCAGGACCCCTTTAC
CCTCTTAAAACTTACTTCGTGAGGACCTTAAAGAGCTTTTGTTTGTGTGACTTATATGTATTAATATTTACTATAT
TAGAAATTAAAAGTAAGGAATGGTTGTAAAAAAAAAAAAAAAAACAAAAAAAAAAAAAAA

Supplementary Data 6, RNA pull-down-MS data of seRNA LOC100506178 in S18 cells

Prot hit number	prot_acc	Prot score	Prot mass	Prot Matches sig	Prot Sequence sig	prot_cover	prot_pi	emPAI
2	sp|Q12906|ILF3_HUMAN	1140	95678	38	18	20.6	8.86	1.32
11	sp|Q08211|DHX9_HUMAN	841	1E+05	34	19	14.2	6.41	0.65
15	sp|P22626|ROA2_HUMAN	805	37464	29	10	27.8	8.97	2.27
16	sp|P31943|HNRH1_HUMAN	656	49484	20	6	15.8	5.89	0.91
19	sp|P55795|HNRH2_HUMAN	540	49517	17	5	13.4	5.89	0.67
23	sp|P09651|ROA1_HUMAN	482	38837	17	5	11.3	9.17	0.77
26	sp|P61978|HNRPK_HUMAN	472	51230	23	11	24	5.39	1.11
32	sp|P50990|TCPQ_HUMAN	420	60153	13	13	26.3	5.42	1
42	sp|P16989|YBOX3_HUMAN	337	40066	13	7	20.2	9.77	1.21
45	sp|P11387|TOP1_HUMAN	321	91125	10	8	10.5	9.33	0.37
49	sp|Q96SI9|STRBP_HUMAN	304	74290	9	5	6.3	8.91	0.3
50	sp|P51991|ROA3_HUMAN	302	39799	12	6	15.9	9.1	0.75
52	sp|Q7KZF4|SND1_HUMAN	297	1E+05	10	9	10.8	6.74	0.37
55	sp|Q9Y6M1|IF2B2_HUMAN	289	66195	9	5	12	8.48	0.4
59	sp|P19525|E2AK2_HUMAN	283	62512	11	7	11.1	8.58	0.59
60	sp|P31942|HNRH3_HUMAN	282	36960	10	5	14.7	6.37	0.67
61	sp|Q9Y2W1|TR150_HUMAN	264	1E+05	12	7	6	10.16	0.27
62	sp|O00571|DDX3X_HUMAN	264	73597	12	7	10.4	6.73	0.36
63	sp|P41091|IF2G_HUMAN	251	51647	10	7	19.3	8.66	0.64
66	sp|P42704|LPPRC_HUMAN	228	2E+05	10	10	7.7	5.81	0.23
67	sp|Q07955|SRSF1_HUMAN	226	27842	11	4	17.3	10.37	0.76
70	sp|P62750|RL23A_HUMAN	222	17684	11	5	27.6	10.44	2.41
71	sp|Q12905|ILF2_HUMAN	218	43263	9	5	15.1	5.19	0.44
73	sp|Q14444|CAPR1_HUMAN	217	78489	8	5	7.9	5.14	0.28
76	sp|Q9Y3I0|RTCB_HUMAN	213	55688	8	6	12.9	6.77	0.58
77	sp|Q92499|DDX1_HUMAN	213	83349	11	8	10	6.81	0.42

78	sp|Q99832|TCPH_HUMAN	211	59842	10	7	12.5	7.55	0.53
79	sp|P26640|SYVC_HUMAN	211	1E+05	5	5	5.4	7.53	0.12
83	sp|P16401|H15_HUMAN	194	22566	6	3	10.6	10.91	0.52
86	sp|Q7L2E3|DHX30_HUMAN	189	1E+05	6	5	4.5	8.99	0.15
87	sp|O60506|HNRPQ_HUMAN	187	69788	12	6	8.2	8.68	0.51
88	sp|P52597|HNRPF_HUMAN	187	45985	10	4	9.6	5.38	0.52
92	sp|P49368|TCPG_HUMAN	184	61066	8	5	10.3	6.1	0.37
93	sp|Q9NZI8|IF2B1_HUMAN	178	63783	8	5	9.5	9.26	0.42
94	sp|Q96KR1|ZFR_HUMAN	175	1E+05	6	4	5.5	9.17	0.12
95	sp|Q01844|EWS_HUMAN	175	68721	6	4	8.1	9.37	0.26
96	sp|O43390|HNRPR_HUMAN	173	71184	10	4	6.3	8.23	0.37
97	sp|Q92945|FUBP2_HUMAN	170	73355	8	6	8.7	6.85	0.3
98	sp|P29692|EF1D_HUMAN	167	31217	5	3	17.8	4.9	0.5
99	sp|O00425|IF2B3_HUMAN	165	64008	8	5	10.7	8.99	0.42
101	sp|Q14103|HNRPD_HUMAN	160	38581	5	2	6.8	7.62	0.18
102	sp|Q92804|RBP56_HUMAN	160	62021	6	4	10	8.04	0.23
106	sp|P18621|RL17_HUMAN	150	21611	6	3	17.4	10.18	0.78
107	sp|O43143|DHX15_HUMAN	149	91673	4	3	4.7	7.12	0.11
109	sp|Q9NZB2|F120A_HUMAN	145	1E+05	6	6	6.7	9.07	0.17
115	sp|P14866|HNRPL_HUMAN	131	64720	6	4	9.7	8.46	0.28
117	sp|O75533|SF3B1_HUMAN	129	1E+05	3	3	2.5	6.65	0.07
119	sp|Q9UHD8|SEPT9_HUMAN	125	65646	5	4	8.4	9.06	0.22
122	sp|P38159|RBMX_HUMAN	124	42306	4	3	7.4	10.06	0.35
123	sp|P78371|TCPB_HUMAN	122	57794	5	4	8.2	6.01	0.25
126	sp|P78563|RED1_HUMAN	116	81112	5	4	5.4	9.11	0.22
127	sp|P17987|TCPA_HUMAN	116	60819	8	7	14.9	5.8	0.45
128	sp|P15880|RS2_HUMAN	115	31590	5	3	9.9	10.25	0.35
129	sp|Q96I24|FUBP3_HUMAN	115	61944	6	5	8.7	8.6	0.3
130	sp|P39023|RL3_HUMAN	114	46365	4	3	7.9	10.19	0.32
131	sp|Q7Z417|NUFP2_HUMAN	114	76132	6	6	9.9	8.7	0.29
132	sp|P50991|TCPD_HUMAN	113	58401	6	6	11.1	7.96	0.39
133	sp|O14979|HNRDL_HUMAN	112	46580	4	2	4.3	9.59	0.15
137	sp|O15042|SR140_HUMAN	111	1E+05	5	3	3.1	8.59	0.08
138	sp|P40227|TCPZ_HUMAN	110	58444	9	7	10.9	6.23	0.47
139	sp|P47897|SYQ_HUMAN	110	88655	4	4	6.2	6.71	0.16
142	sp|Q8NE71|ABCF1_HUMAN	108	96323	5	5	5.8	6.4	0.18
143	sp|Q9BUJ2|HNRL1_HUMAN	104	96250	4	4	4.9	6.49	0.14
145	sp|Q96HS1|PGAM5_HUMAN	102	32213	5	5	17.3	8.88	0.63
146	sp|Q8WWM7|ATX2L_HUMAN	100	1E+05	8	6	7.5	8.7	0.19
148	sp|O60841|IF2P_HUMAN	99	1E+05	4	4	3.8	5.39	0.1
149	sp|P51116|FXR2_HUMAN	98	74520	6	4	5.6	5.95	0.19
155	sp|P26038|MOES_HUMAN	97	67892	8	8	11.4	6.08	0.46
159	sp|Q9UN86|G3BP2_HUMAN	96	54145	5	3	6.8	5.41	0.34
160	sp|P05198|IF2A_HUMAN	94	36374	3	3	10.2	5.02	0.3

161	sp|P62899|RL31_HUMAN	94	14454	2	1	7.2	10.54	0.24
165	sp|Q9UQ80|PA2G4_HUMAN	92	44101	4	3	7.9	6.13	0.34
168	sp|O76021|RL1D1_HUMAN	89	55167	3	2	4.3	10.13	0.19
169	sp|Q9Y5M8|SRPRB_HUMAN	89	29912	3	3	14	9.17	0.37
170	sp|Q9P258|RCC2_HUMAN	88	56790	4	4	5.9	9.02	0.25
172	sp|Q9UMS4|PRP19_HUMAN	87	55603	4	4	7.7	6.14	0.26
173	sp|Q08J23|NSUN2_HUMAN	86	87214	3	3	4.3	6.33	0.12
175	sp|Q07157|ZO1_HUMAN	84	2E+05	3	3	1.9	6.24	0.05
176	sp|Q6UN15|FIP1_HUMAN	84	66601	2	2	4.5	5.42	0.1
177	sp|P61247|RS3A_HUMAN	83	30154	6	4	11.7	9.75	0.69
178	sp|P48643|TCPE_HUMAN	82	60089	5	4	8.3	5.45	0.31
180	sp|Q13243|SRSF5_HUMAN	82	31359	2	2	7.7	11.59	0.22
181	sp|Q9UKV8|AGO2_HUMAN	81	98400	4	4	5.5	9.32	0.14
182	sp|P62316|SMD2_HUMAN	80	13632	3	3	22.9	9.92	0.95
183	sp|E9PAV3|NACAM_HUMAN	80	2E+05	1	1	0.7	9.6	0.02
184	sp|Q01130|SRSF2_HUMAN	80	25461	3	2	11.3	11.86	0.28
185	sp|Q13247|SRSF6_HUMAN	79	39677	3	3	7.3	11.42	0.27
188	sp|P39019|RS19_HUMAN	78	16051	4	3	17.2	10.31	0.77
190	sp|Q8N684|CPSF7_HUMAN	77	52189	2	2	4.2	7.82	0.13
191	sp|Q16629|SRSF7_HUMAN	77	27578	3	2	8.8	11.83	0.26
192	sp|Q96AE4|FUBP1_HUMAN	76	67690	6	6	9.9	7.18	0.33
194	sp|P62841|RS15_HUMAN	75	17029	4	1	8.3	10.39	0.44
195	sp|Q96T37|RBM15_HUMAN	74	1E+05	4	4	4.7	10.09	0.13
196	sp|Q15427|SF3B4_HUMAN	74	44414	1	1	3.3	8.54	0.07
198	sp|P51114|FXR1_HUMAN	73	70020	5	4	6.3	5.84	0.2
199	sp|Q9BUQ8|DDX23_HUMAN	73	95866	4	4	5	9.58	0.14
200	sp|P47914|RL29_HUMAN	73	17798	2	1	9.4	11.66	0.19
202	sp|Q07065|CKAP4_HUMAN	72	66097	2	2	3.8	5.63	0.1
204	sp|O43684|BUB3_HUMAN	72	37587	3	3	11.3	6.36	0.29
205	sp|Q8WXF1|PSPC1_HUMAN	72	58820	2	2	4.2	6.26	0.11
207	sp|P14868|SYDC_HUMAN	71	57499	5	5	9	6.11	0.32
208	sp|P41252|SYIC_HUMAN	70	1E+05	4	4	3.2	5.82	0.09
209	sp|O75569|PRKRA_HUMAN	70	34839	2	2	8	8.69	0.2
210	sp|P60842|IF4A1_HUMAN	68	46353	2	2	5.2	5.32	0.15
213	sp|Q92522|H1X_HUMAN	68	22474	2	2	10.3	10.76	0.32
214	sp|P63104|1433Z_HUMAN	68	27899	1	1	5.7	4.73	0.12
215	sp|P26196|DDX6_HUMAN	68	54781	3	3	8.3	8.85	0.19
216	sp|O75534|CSDE1_HUMAN	67	89684	5	5	5.5	5.88	0.2
219	sp|Q86UK7|ZN598_HUMAN	66	1E+05	1	1	1.4	8.75	0.03
221	sp|Q1KMD3|HNRL2_HUMAN	62	85622	3	3	3.7	4.85	0.12
222	sp|Q86V81|THOC4_HUMAN	62	26872	2	1	4.3	11.15	0.26
223	sp|P02545|LMNA_HUMAN	61	74380	2	2	3.8	6.57	0.09
224	sp|Q6P158|DHX57_HUMAN	61	2E+05	2	2	1.4	7.83	0.04
225	sp|Q14258|TRI25_HUMAN	61	72581	2	2	3.7	8.44	0.09

227	sp|Q5T9A4|ATD3B_HUMAN	61	73098	3	3	4.2	9.3	0.14
231	sp|Q15029|U5S1_HUMAN	60	1E+05	3	2	2.6	4.84	0.09
232	sp|Q9NYF8|BCLF1_HUMAN	59	1E+05	6	6	7.5	9.99	0.2
233	sp|P55769|NH2L1_HUMAN	59	14393	1	1	9.4	8.72	0.24
234	sp|B5ME19|EIFCL_HUMAN	59	1E+05	1	1	1.5	5.45	0.03
235	sp|P62495|ERF1_HUMAN	59	49228	1	1	2.5	5.51	0.07
240	sp|Q9UG63|ABCF2_HUMAN	57	71815	2	2	3.4	6.95	0.09
241	sp|Q8IUD2|RB6I2_HUMAN	57	1E+05	1	1	1.1	5.72	0.03
242	sp|Q8NAB2|KBTB3_HUMAN	57	71220	6	1	1.1	5.18	0.05
243	sp|Q9BY77|PDIP3_HUMAN	57	46289	2	2	6.4	10	0.15
244	sp|B2RPK0|HGB1A_HUMAN	56	24394	2	2	7.1	5.92	0.29
247	sp|O75342|LX12B_HUMAN	55	81216	1	1	1.7	7.57	0.04
248	sp|O75477|ERLN1_HUMAN	55	39318	1	1	3.4	7.67	0.08
251	sp|P27816|MAP4_HUMAN	54	1E+05	2	2	2.4	5.32	0.05
253	sp|Q05193|DYN1_HUMAN	54	97746	2	2	2.2	6.73	0.07
254	sp|P20042|IF2B_HUMAN	54	38706	1	1	3	5.6	0.09
256	sp|Q92900|RENT1_HUMAN	54	1E+05	2	2	1.8	6.18	0.05
257	sp|P12081|SYHC_HUMAN	53	57944	1	1	1.6	5.72	0.06
258	sp|Q9HCY8|S10AE_HUMAN	53	11826	1	1	14.4	5.16	0.29
259	sp|P08865|RSSA_HUMAN	53	32947	1	1	4.4	4.79	0.1
260	sp|P50416|CPT1A_HUMAN	52	88995	2	2	2.7	8.85	0.07
264	sp|Q01085|TIAR_HUMAN	51	41906	1	1	2.9	7.62	0.08
265	sp|P62917|RL8_HUMAN	51	28235	2	2	10.5	11.03	0.25
266	sp|P14678|RSMB_HUMAN	51	24765	4	2	9.2	11.2	0.46
267	sp|Q9Y285|SYFA_HUMAN	51	57585	2	2	3.5	7.31	0.12
269	sp|O60832|DKC1_HUMAN	50	58094	1	1	2.3	9.46	0.06
270	sp|Q15366|PCBP2_HUMAN	49	38955	2	2	6.8	6.33	0.18
272	sp|P62826|RAN_HUMAN	49	24579	1	1	5.1	7.01	0.14
274	sp|P06702|S10A9_HUMAN	48	13291	2	1	11.4	5.71	0.58
275	sp|P23490|LORI_HUMAN	48	26828	2	1	4.2	8.5	0.26
276	sp|O43670|ZN207_HUMAN	48	51002	1	1	2.7	9.19	0.06
278	sp|P0DN76|U2AF5_HUMAN	48	28368	1	1	3.3	9.09	0.12
279	sp|Q15459|SF3A1_HUMAN	47	88888	2	2	2.6	5.15	0.08
280	sp|Q15393|SF3B3_HUMAN	47	1E+05	1	1	1.2	5.13	0.02
281	sp|P07814|SYEP_HUMAN	47	2E+05	3	3	2.2	7.02	0.06
283	sp|P23284|PPIB_HUMAN	47	23785	2	2	9.3	9.42	0.3
284	sp|Q99959|PKP2_HUMAN	47	97868	1	1	1.2	9.39	0.03
285	sp|O75494|SRS10_HUMAN	46	31339	1	1	4.2	11.26	0.11
286	sp|O94906|PRP6_HUMAN	46	1E+05	3	3	3.2	8.49	0.09
287	sp|P46063|RECQ1_HUMAN	46	74436	2	2	3.1	8.12	0.09
288	sp|Q13409|DC1I2_HUMAN	46	71811	2	2	5.5	5.08	0.09
289	sp|P62314|SMD1_HUMAN	46	13273	1	1	9.2	11.56	0.26
290	sp|Q9H0A0|NAT10_HUMAN	46	1E+05	2	2	2	8.5	0.06
292	sp|Q13435|SF3B2_HUMAN	45	1E+05	3	3	3	5.52	0.1

293	sp|P78344|IF4G2_HUMAN	45	1E+05	2	2	3.5	6.7	0.06
294	sp|Q8IX12|CCAR1_HUMAN	45	1E+05	1	1	1	5.57	0.02
296	sp|P23588|IF4B_HUMAN	45	69167	2	1	2.6	5.55	0.1
298	sp|Q06787|FMR1_HUMAN	44	71473	3	2	2.5	6.99	0.09
299	sp|P42766|RL35_HUMAN	44	14543	1	1	8.1	11.04	0.23
302	sp|A6NHQ2|FBLL1_HUMAN	44	34839	1	1	3.3	10.35	0.1
304	sp|P34897|GLYM_HUMAN	44	56414	1	1	1.8	8.76	0.06
305	sp|Q6P2E9|EDC4_HUMAN	43	2E+05	1	1	0.7	5.55	0.02
307	sp|Q52LJ0|FA98B_HUMAN	43	45918	1	1	3	8.88	0.07
308	sp|P14618|KPYM_HUMAN	43	58470	1	1	2.4	7.96	0.06
309	sp|O95786|DDX58_HUMAN	42	1E+05	1	1	1.1	6.03	0.03
310	sp|Q8NFW8|NEUA_HUMAN	42	49033	1	1	2.8	8.16	0.07
311	sp|P62280|RS11_HUMAN	42	18590	3	3	15.8	10.31	0.65
313	sp|P05976|MYL1_HUMAN	42	21189	1	1	5.7	4.97	0.16
314	sp|Q5JNZ5|RS26L_HUMAN	42	13336	1	1	7.8	10.55	0.26
315	sp|Q5SSJ5|HP1B3_HUMAN	41	61454	2	2	3.1	9.69	0.11
317	sp|P62910|RL32_HUMAN	41	15964	1	1	9.6	11.32	0.21
318	sp|P48634|PRC2A_HUMAN	41	2E+05	1	1	0.5	9.48	0.01
319	sp|P99999|CYC_HUMAN	41	11855	1	1	7.6	9.59	0.29
320	sp|Q9BQG0|MBB1A_HUMAN	41	1E+05	1	1	0.9	9.34	0.02
322	sp|Q969S3|ZN622_HUMAN	40	54808	1	1	3.1	5.8	0.06
323	sp|P01859|IGHG2_HUMAN	40	36505	1	1	4.3	7.66	0.09
324	sp|Q16666|IF16_HUMAN	40	88656	1	1	1.3	9.31	0.04
325	sp|Q12849|GRSF1_HUMAN	40	53606	1	1	2.3	5.83	0.06
328	sp|Q12797|ASPH_HUMAN	40	86266	1	1	1.2	4.92	0.04
329	sp|O95347|SMC2_HUMAN	40	1E+05	1	1	0.9	8.54	0.02
331	sp|Q9BYE4|SPR2G_HUMAN	40	8779	1	1	17.8	8.3	0.4
332	sp|Q5VZF2|MBNL2_HUMAN	39	41518	1	1	2.4	8.77	0.08
333	sp|O60678|ANM3_HUMAN	39	60549	1	1	1.9	5.18	0.05
334	sp|P43243|MATR3_HUMAN	38	95078	2	1	1.3	5.87	0.03
335	sp|P17096|HMGA1_HUMAN	38	11669	1	1	15	10.32	0.3
336	sp|Q14204|DYHC1_HUMAN	38	5E+05	3	3	0.7	6.01	0.02
338	sp|P08708|RS17_HUMAN	38	15597	1	1	8.9	9.85	0.22
339	sp|P26368|U2AF2_HUMAN	38	53809	1	1	2.1	9.19	0.06
340	sp|Q9H0L4|CSTFT_HUMAN	38	64624	1	1	1.8	6.79	0.05
341	sp|Q8IYB8|SUV3_HUMAN	37	88791	1	1	1.3	8.2	0.04
342	sp|Q14694|UBP10_HUMAN	37	87707	2	2	3.5	5.19	0.08
344	sp|O00567|NOP56_HUMAN	37	66408	1	1	1.3	9.24	0.05
345	sp|P62847|RS24_HUMAN	37	15413	2	1	8.3	10.79	0.49
346	sp|P17275|JUNB_HUMAN	37	36028	1	1	4	9.27	0.09
347	sp|Q86UE4|LYRIC_HUMAN	37	63856	2	2	4.1	9.33	0.11
348	sp|P62306|RUXF_HUMAN	37	9776	1	1	9.3	4.7	0.36
349	sp|Q00577|PURA_HUMAN	36	35003	1	1	2.8	6.07	0.09
350	sp|O94992|HEXI1_HUMAN	36	40884	1	1	4.7	4.84	0.08

352	sp|Q01469|FABP5_HUMAN	36	15497	1	1	8.1	6.6	0.22
353	sp|P49748|ACADV_HUMAN	36	70745	1	1	2	8.92	0.05
354	sp|Q6EKJ0|GTD2B_HUMAN	36	1E+05	1	1	0.9	5.62	0.03
355	sp|Q6NVV1|R13P3_HUMAN	36	12184	1	1	10.8	10.76	0.28
356	sp|P04040|CATA_HUMAN	35	59947	1	1	2.5	6.9	0.05
357	sp|P62995|TRA2B_HUMAN	35	33760	2	1	2.4	11.25	0.1
359	sp|Q14152|EIF3A_HUMAN	35	2E+05	2	1	0.7	6.38	0.04
361	sp|P54136|SYRC_HUMAN	35	76129	2	2	3	6.26	0.09
362	sp|P49589|SYCC_HUMAN	35	86103	1	1	1.5	6.31	0.04
363	sp|Q9UKN8|TF3C4_HUMAN	35	93235	1	1	1.1	6.21	0.04
365	sp|P16615|AT2A2_HUMAN	35	1E+05	2	2	2.5	5.23	0.06
366	sp|P42285|MTREX_HUMAN	35	1E+05	1	1	1	6.12	0.03
367	sp|P26583|HMGB2_HUMAN	35	24190	1	1	6.2	7.62	0.14
368	sp|Q8NBJ5|GT251_HUMAN	35	71933	1	1	2.3	6.85	0.05
369	sp|Q15424|SAFB1_HUMAN	34	1E+05	2	2	2	5.32	0.06
370	sp|P39748|FEN1_HUMAN	34	42908	1	1	2.9	8.8	0.08
371	sp|O43290|SNUT1_HUMAN	34	90371	1	1	2	5.89	0.04
372	sp|P32322|P5CR1_HUMAN	34	33568	1	1	3.4	7.18	0.1
373	sp|Q00341|VIGLN_HUMAN	34	1E+05	2	2	1.8	6.43	0.05
374	sp|Q7Z2W4|ZCCHV_HUMAN	33	1E+05	2	2	3.1	8.72	0.06
375	sp|P13489|RINI_HUMAN	33	51766	1	1	2	4.71	0.06
377	sp|Q6PI48|SYDM_HUMAN	33	74086	2	2	2.3	8.19	0.09
378	sp|Q9NUQ6|SPS2L_HUMAN	33	62204	1	1	1.6	9.67	0.05
379	sp|Q7L576|CYFP1_HUMAN	33	1E+05	1	1	0.9	6.46	0.02
380	sp|Q00325|MPCP_HUMAN	32	40525	2	2	5.5	9.45	0.17
381	sp|O75390|CISY_HUMAN	32	51908	1	1	2.4	8.45	0.06
382	sp|Q13347|EIF3I_HUMAN	32	36878	1	1	3.1	5.38	0.09
383	sp|P25398|RS12_HUMAN	32	14905	1	1	7.6	6.81	0.23
384	sp|Q9P2J5|SYLC_HUMAN	31	1E+05	1	1	0.9	6.95	0.02
386	sp|O60716|CTND1_HUMAN	31	1E+05	1	1	1.1	5.86	0.03
388	sp|P36873|PP1G_HUMAN	31	37701	1	1	3.1	6.13	0.09
389	sp|P57088|TMM33_HUMAN	31	28302	1	1	4	9.75	0.12
390	sp|Q6PKG0|LARP1_HUMAN	31	1E+05	1	1	1.1	8.91	0.03
391	sp|Q6DN72|FCRL6_HUMAN	31	48515	2	1	1.8	7.53	0.07
392	sp|Q8N766|EMC1_HUMAN	31	1E+05	1	1	0.7	7.38	0.03
394	sp|P35249|RFC4_HUMAN	31	40170	1	1	2.8	8.26	0.08
395	sp|O43776|SYNC_HUMAN	30	63758	1	1	2.2	5.9	0.05
396	sp|Q8TEQ6|GEMI5_HUMAN	30	2E+05	1	1	0.7	6.17	0.02
397	sp|Q63ZY6|NSN5C_HUMAN	30	34952	1	1	4.4	9.01	0.09
398	sp|P61353|RL27_HUMAN	30	15788	1	1	6.6	10.56	0.22
399	sp|Q07666|KHDR1_HUMAN	30	48311	1	1	2.3	8.73	0.07
400	sp|Q01813|PFKAP_HUMAN	30	86454	1	1	2.4	7.5	0.04
401	sp|P98175|RBM10_HUMAN	30	1E+05	1	1	1.2	5.69	0.03
403	sp|Q9Y230|RUVB2_HUMAN	30	51296	1	1	2.6	5.49	0.06

404	sp|P08621|RU17_HUMAN	30	51583	1	1	3	9.94	0.06
406	sp|Q8NC51|PAIRB_HUMAN	30	44995	2	1	2	8.66	0.15
408	sp|Q6WCQ1|MPRIP_HUMAN	29	1E+05	1	1	1.2	5.89	0.03
409	sp|P26639|SYTC_HUMAN	29	84294	1	1	1.4	6.23	0.04
410	sp|Q92879|CELF1_HUMAN	29	52429	1	1	1.6	8.7	0.06
411	sp|Q9Y224|RTRAF_HUMAN	29	28165	1	1	3.7	6.19	0.12
413	sp|P50579|MAP2_HUMAN	29	53713	1	1	2.1	5.57	0.06
415	sp|P24534|EF1B_HUMAN	29	24919	1	1	5.8	4.5	0.13
416	sp|Q7Z6B7|SRGP1_HUMAN	29	1E+05	1	1	1.1	6.36	0.03
417	sp|Q99700|ATX2_HUMAN	29	1E+05	1	1	1.4	9.6	0.02
419	sp|Q9Y2X3|NOP58_HUMAN	29	60054	1	1	2.5	9.03	0.05
420	sp|P30101|PDIA3_HUMAN	29	57146	1	1	1.6	5.98	0.06
421	sp|P62829|RL23_HUMAN	29	14970	2	1	5.7	10.51	0.23
422	sp|P04083|ANXA1_HUMAN	28	38918	1	1	3.2	6.57	0.08
424	sp|Q13242|SRSF9_HUMAN	28	25640	1	1	5	8.74	0.13
425	sp|Q9H2U1|DHX36_HUMAN	28	1E+05	2	2	2.3	7.58	0.06
426	sp|Q8TB72|PUM2_HUMAN	28	1E+05	1	1	0.6	6.61	0.03
428	sp|P78310|CXAR_HUMAN	28	40575	1	1	1.6	7.49	0.08
429	sp|Q6NZI2|CAVN1_HUMAN	28	43450	1	1	2.8	5.51	0.08
430	sp|Q08945|SSRP1_HUMAN	28	81367	1	1	1.6	6.45	0.04
432	sp|P46778|RL21_HUMAN	27	18610	1	1	4.4	10.49	0.18
433	sp|Q86XP3|DDX42_HUMAN	27	1E+05	1	1	0.9	6.54	0.03
437	sp|P54577|SYYC_HUMAN	27	59448	1	1	1.7	6.61	0.06
438	sp|Q8N1G4|LRC47_HUMAN	27	64004	1	1	2.1	8.55	0.05
439	sp|Q5T8P6|RBM26_HUMAN	27	1E+05	1	1	1.2	9.21	0.03
441	sp|P22528|SPR1B_HUMAN	27	10337	1	1	19.1	8.85	0.34
443	sp|Q13064|MKRN3_HUMAN	27	56749	1	1	1.8	5.51	0.06
445	sp|P61221|ABCE1_HUMAN	27	68240	1	1	2	8.63	0.05
447	sp|P49790|NU153_HUMAN	27	2E+05	1	1	0.9	8.97	0.02
448	sp|Q01082|SPTB2_HUMAN	26	3E+05	1	1	0.3	5.39	0.01
449	sp|P62258|1433E_HUMAN	26	29326	1	1	4.3	4.63	0.11
450	sp|Q16630|CPSF6_HUMAN	26	59344	1	1	2.5	6.66	0.06
451	sp|Q13751|LAMB3_HUMAN	26	1E+05	1	1	0.9	7.14	0.02
453	sp|O00442|RTCA_HUMAN	26	39825	1	1	2.7	8.01	0.08
456	sp|Q9UBU9|NXF1_HUMAN	26	70652	1	1	1.6	8.74	0.05
457	sp|Q96C36|P5CR2_HUMAN	26	33958	1	1	2.5	7.66	0.1
459	sp|Q8TF40|FNIP1_HUMAN	26	1E+05	1	1	1.1	5.33	0.02
460	sp|Q96RM1|SPR2F_HUMAN	26	8541	1	1	18.1	8.73	0.41
461	sp|O00443|P3C2A_HUMAN	25	2E+05	1	1	0.7	8.25	0.02
462	sp|Q92974|ARHG2_HUMAN	25	1E+05	1	1	1	6.89	0.03
463	sp|Q5D862|FILA2_HUMAN	25	2E+05	2	2	1.5	8.45	0.03
464	sp|P23381|SYWC_HUMAN	25	53474	1	1	3	5.83	0.06
465	sp|P61981|1433G_HUMAN	25	28456	1	1	5.7	4.8	0.12
466	sp|Q8N884|CGAS_HUMAN	25	59462	1	1	1.3	9.54	0.06

467	sp|P45379|TNNT2_HUMAN	25	35902	1	1	2.3	4.94	0.09
468	sp|O15347|HMGB3_HUMAN	25	23137	1	1	6.5	8.48	0.15
471	sp|O00148|DX39A_HUMAN	25	49611	1	1	1.9	5.46	0.07
472	sp|P27348|1433T_HUMAN	24	28032	1	1	5.7	4.68	0.12
473	sp|P06730|IF4E_HUMAN	24	25310	1	1	6.5	5.79	0.13
474	sp|Q15717|ELAV1_HUMAN	24	36240	1	1	2.5	9.23	0.09
475	sp|P47813|IF1AX_HUMAN	24	16564	1	1	6.9	5.07	0.2
477	sp|P60660|MYL6_HUMAN	24	17090	1	1	5.3	4.56	0.2
478	sp|Q8IXB1|DJC10_HUMAN	24	92333	1	1	1.3	6.78	0.04
479	sp|Q9BY44|EIF2A_HUMAN	24	65519	1	1	1.5	9	0.05
480	sp|Q9Y383|LC7L2_HUMAN	23	46942	1	1	3.8	10.02	0.07
481	sp|Q7Z7H5|TMED4_HUMAN	23	26097	1	1	4	8.41	0.13
482	sp|Q96QR8|PURB_HUMAN	23	33392	1	1	1.9	5.35	0.1
483	sp|Q9Y3B2|EXOS1_HUMAN	23	21780	1	1	7.2	8.51	0.15
484	sp|Q9NRH3|TBG2_HUMAN	23	51402	1	1	2	5.5	0.06
485	sp|Q13428|TCOF_HUMAN	23	2E+05	1	1	0.9	9.06	0.02
486	sp|P07910|HNRPC_HUMAN	23	33707	1	1	2.9	4.95	0.1
487	sp|Q969T4|UB2E3_HUMAN	22	23184	1	1	3.4	6.73	0.14
488	sp|P62081|RS7_HUMAN	22	22113	1	1	4.1	10.09	0.15
489	sp|P57103|NAC3_HUMAN	21	1E+05	1	1	1.9	5.01	0.03
491	sp|P53396|ACLY_HUMAN	21	1E+05	1	1	0.6	6.95	0.03
493	sp|Q9Y2Z9|COQ6_HUMAN	20	51123	1	1	1.9	6.81	0.06
494	sp|P22735|TGM1_HUMAN	18	90529	1	1	1.1	5.68	0.04
495	sp|Q9Y490|TLN1_HUMAN	18	3E+05	1	1	0.5	5.77	0.01
496	sp|O75688|PPM1B_HUMAN	17	53180	1	1	2.9	4.95	0.06
497	sp|Q16363|LAMA4_HUMAN	17	2E+05	1	1	1	5.89	0.02
498	sp|P23760|PAX3_HUMAN	16	53334	1	1	2.1	8.92	0.06
499	sp|P30419|NMT1_HUMAN	16	57112	1	1	1.4	7.66	0.06
500	sp|Q9Y219|JAG2_HUMAN	15	1E+05	1	1	1.1	5.53	0.02
501	sp|Q6ZU15|SEP14_HUMAN	15	50449	1	1	2.1	5.87	0.07
503	sp|Q9P0V9|SEP10_HUMAN	13	53016	1	1	2	6.35	0.06
504	sp|Q6ZVH7|ESPNL_HUMAN	13	1E+05	1	1	0.7	6.01	0.03
