# Supplementary material for: A novel oncogenic seRNA promotes nasopharyngeal carcinoma metastasis
Source: Cell Death Dis. 2022 Apr 23;13(4):401. doi: 10.1038/s41419-022-04846-1 (PMC9035166; doi:10.1038/s41419-022-04846-1)
Supplement: Supplementary file 3 — Supplementary Material 3 [file 41419_2022_4846_MOESM3_ESM.pdf]

Supplementary Data 7, RNA-Seq data of S18-shseRNA#3 and shscramble cells

shseRNA#3	shseRNA#3	shseRNA#3	shscrambl	shscrambl	shscrambl	log2FoldC	pvalue	gene
14375.37	14723.27	14647.28	1088.153	1247.804	1304.373	3.587209	0	FN1
10996.31	11097.19	10070.86	1721.958	1607.767	1778.306	2.654605	0	OASL
9850.489	9259.383	9030.557	323.6451	413.4858	450.6593	4.56692	0	NR4A1
1093.7	1195.814	1130.645	8463.528	8666.411	8432.407	-2.90253	0	GLO1
7218.24	7405.723	6719.078	1019.69	884.6917	821.9772	2.968416	0	ISG15
7281.148	6436.787	6994.667	1033.175	915.1259	911.8975	2.856286	0	-
5825.278	5675.905	5531.855	205.3902	208.8418	283.5134	4.610304	0	KRT17
3250.545	3749.921	3631.934	360.9888	379.9032	416.807	3.199225	8.87E-295	LRP1
17992.58	18061.05	18633.28	55149.34	57004.37	55352.81	-1.61494	8.03E-285	EIF4G2
2353.657	2409.461	2259.464	258.2937	234.0288	242.2558	3.25671	3.36E-283	RSAD2
608.4099	665.772	675.2842	5945.942	5316.546	4787.991	-3.04186	5.30E-279	MMP1
2015.751	2259.86	1904.484	54.97818	52.47282	41.25754	5.375776	6.01E-278	IFI6
8966.183	9372.326	9120.899	2588.124	2606.85	2612.978	1.814232	1.41E-262	CLU
4276.843	4532.6	4397.56	943.965	955.0053	933.0552	2.221266	1.33E-260	COL6A1
5639.25	5913.68	6190.714	20383.42	21483.42	21246.58	-1.83066	2.51E-256	DDX21
9290.608	8749.156	9002.268	1788.347	1985.571	2087.208	2.206125	2.88E-251	BHLHE40
3226.28	3318.953	2960.3	460.5719	454.4146	502.4957	2.745449	4.00E-248	AQP3
709.0627	750.975	753.7632	4174.192	3956.45	3730.105	-2.42193	2.51E-245	POLE3
15460.98	14818.38	15132.75	4181.454	4063.495	4492.84	1.834025	4.15E-243	CITED2
1055.057	1134.388	1144.333	4903.431	5398.403	5151.903	-2.21299	1.87E-239	DCBLD2
24215.97	23484.32	24722.7	8305.854	8280.211	8329.792	1.539413	9.37E-238	CYR61
5400.2	5669.96	5105.696	1134.833	1195.331	1225.032	2.185808	1.13E-233	L1CAM
2577.429	2619.496	2639.996	409.743	447.0684	385.0704	2.657648	8.68E-233	CSPG4
2618.769	2607.607	2639.084	8250.876	8418.739	8329.792	-1.66825	1.38E-231	CORO1C
2390.503	2254.906	2258.552	347.5036	345.2711	359.6811	2.713852	1.16E-229	EEF1A2
26710.72	27833.63	28667.64	77684.17	77013.3	78386.15	-1.48599	6.04E-224	HSP90AA1
5433.451	5688.784	5482.578	17495.51	17363.26	16470.22	-1.62829	1.27E-223	CSE1L
6432.789	6448.676	5927.9	1577.77	1578.382	1481.04	2.020014	9.54E-223	SREBF1
20029.9	21026.31	20357.08	58741.59	55668.41	57412.51	-1.48433	8.86E-220	NPM1
3957.81	4299.778	3816.268	759.3213	638.0695	701.3782	2.523972	2.14E-217	SNCG
2847.934	3058.39	2609.882	360.9888	300.1445	379.781	3.032224	3.24E-215	FOSB
1481.932	1443.497	1534.903	5513.378	5683.856	5313.76	-1.88797	1.12E-210	HACD2
4640.811	4959.605	4894.898	14430.22	14489.84	15085.45	-1.60222	4.22E-209	SUB1
3885.915	3839.087	3931.249	1006.204	939.2634	966.9075	2.000811	6.58E-208	OTUD1
864.5352	933.2697	980.9872	3931.458	4097.078	3959.666	-2.10929	2.47E-204	UTP20
3519.252	3455.674	3427.524	683.5966	573.0032	540.5796	2.53278	1.23E-201	-
35104.44	34542.87	35436.91	8974.928	9741.054	10616.94	1.84099	1.06E-199	SCD
10698.85	11092.24	10914.05	27692.41	27699.35	28045.61	-1.35122	1.73E-199	SET
2156.845	2118.185	2034.978	371.362	356.8152	367.0863	2.526318	3.07E-198	SIK1
5190.806	4900.161	4651.248	1063.257	878.395	908.7238	2.370569	7.94E-197	UCA1
3305.364	3730.107	3268.741	530.0726	434.4749	392.4756	2.924115	3.81E-196	HELZ2
8749.6	8515.343	7967.441	2589.161	2458.876	2506.131	1.739894	8.31E-192	JUNB
3043.847	3029.659	2895.509	534.2219	499.5412	407.286	2.637413	6.32E-189	SLC25A1
3582.16	3736.051	3804.405	10417.85	10957.37	10741.77	-1.5299	1.07E-183	HSPH1
4125.864	4164.047	3784.329	938.7783	921.4227	818.8035	2.171929	1.25E-183	MKNK2
3698.09	3700.385	3464.938	788.3663	632.8222	680.2205	2.369784	3.52E-182	JUND
2838.048	2733.43	2942.049	8258.137	8395.651	8356.239	-1.55445	6.73E-182	NUP153
1723.678	1848.706	1849.731	5968.763	5778.307	5737.972	-1.68945	6.09E-179	BRIX1
3219.989	3424.961	3238.627	838.1579	861.6037	843.1349	1.958404	1.36E-178	PC
3018.684	3004.891	3290.642	9836.945	10773.72	10157.82	-1.72382	2.73E-178	ATP13A3
2292.546	2142.954	2187.373	429.4522	371.5075	363.9127	2.507089	1.96E-177	SIK1B
4732.477	4761.459	4729.727	12184.41	12685.83	12222.81	-1.38287	6.22E-176	TIMP2

1676.947	1691.18	1763.039	5285.166	5571.564	5385.696	-1.66234	6.89E-175	ABCE1
2350.961	2595.718	2567.905	448.124	370.4581	452.7751	2.56316	9.12E-175	ATF3
448.444	443.848	475.4366	2105.768	2197.562	2218.386	-2.25308	1.93E-174	C5orf22
3530.036	3628.061	3545.242	869.2776	909.8787	980.66	1.955515	6.07E-174	LAMB2
20266.25	21625.7	20562.4	7818.312	7543.492	7262.385	1.464872	5.14E-172	COL5A1
6231.484	6635.924	6454.439	16406.32	16650.67	16381.36	-1.35552	3.39E-169	SERBP1
1866.569	1849.697	1801.366	5663.79	5912.637	5508.411	-1.63063	9.36E-169	TFDP1
3476.115	3501.248	3537.942	966.7861	1022.17	1018.744	1.805835	2.05E-168	RRBP1
2833.555	3106.936	2940.224	9940.677	9435.662	8993.086	-1.67587	3.47E-167	COLGALT1
14258.54	15017.52	14923.78	35828.14	35517.8	36805.96	-1.29098	4.71E-167	CCT5
1269.842	1437.552	1266.614	149.3747	124.8853	129.0621	3.299773	1.50E-166	C11orf86
4324.474	4700.033	4675.887	13956.16	14369.16	13115.67	-1.59698	1.79E-166	IPO5
4723.49	5165.678	5041.818	1420.097	1500.723	1488.445	1.759621	6.49E-166	AHNAK2
12679.55	11826.37	13285.76	3745.777	3195.595	3407.45	1.868614	9.82E-166	EGR1
2359.049	2349.026	2439.236	6656.509	6908.571	7102.644	-1.53181	2.54E-163	SRSF2
1156.608	1154.203	1123.344	3746.815	3675.196	3679.326	-1.69278	2.40E-162	NETO2
2707.739	2861.235	2934.749	8230.13	8397.75	8877.777	-1.58472	3.45E-162	NOLC1
2110.113	2279.675	2078.78	327.7944	268.6608	222.156	2.981365	4.25E-162	PCSK9
2680.778	2691.819	2695.662	7173.096	7080.682	7084.66	-1.40314	1.85E-161	CTNNAL1
2644.831	2649.218	2571.555	7268.53	7894.011	7795.56	-1.5454	2.75E-160	ICE1
1526.866	1460.339	1620.682	4949.073	5163.325	4911.763	-1.70473	4.54E-159	LBR
2249.409	2226.175	2348.894	6263.363	6433.167	6430.887	-1.48671	6.88E-158	PSMD12
2686.17	2686.866	2660.985	8359.795	7590.718	7715.16	-1.55864	1.99E-157	MTATP6P1
2753.572	2557.08	2442.886	589.2001	586.6461	605.1106	2.122325	3.17E-157	TNFAIP3
123.1199	107.9898	121.3686	1065.332	1080.94	1047.307	-3.17753	4.57E-157	NRG1
3706.178	4069.928	3944.937	12874.23	12042.51	11483.35	-1.63503	4.81E-157	ODC1
691.089	728.1882	697.1853	2621.318	2840.878	2707.129	-1.94898	1.57E-155	DDAH1
15108.7	14764.88	15008.65	6208.385	5840.225	6044.759	1.31069	1.80E-155	BASP1
2665.501	2760.18	2747.677	7628.482	7232.853	7361.826	-1.44318	2.25E-155	EBNA1BP2
16475.6	16361.94	16712.37	6067.309	5334.387	5383.58	1.56165	2.28E-154	DUSP1
5168.339	5467.851	5533.68	1430.47	1290.831	1129.822	2.069734	1.38E-151	COL7A1
1744.348	1666.412	1559.541	280.0775	309.5896	286.687	2.503833	3.57E-150	ADAMTSL4
3036.658	2976.159	3078.018	7937.604	8586.652	8483.185	-1.45976	4.66E-150	SRSF1
1835.115	1897.252	1630.72	300.824	244.5233	245.4295	2.761108	8.86E-150	PALM
1647.29	1720.902	1866.157	5452.176	5492.855	5685.078	-1.66768	1.08E-149	EIF3J
9832.516	10648.39	10527.13	26854.25	26798.92	25526.78	-1.35257	1.57E-149	HSPD1
1956.438	2075.584	1929.123	448.124	421.8815	400.9387	2.229272	1.87E-149	CKB
2426.45	2393.609	2350.719	464.7212	418.7331	334.2919	2.557446	3.77E-148	MAFK
2625.06	2765.134	2400.909	598.536	574.0526	612.5158	2.125626	9.70E-148	CD70
3697.191	3724.162	3739.614	9022.645	9426.217	9389.793	-1.31863	1.36E-147	RBMX
4533.867	4517.739	4655.811	12434.4	11440.12	11846.2	-1.38178	1.85E-147	RBM3
6021.191	6055.355	5812.919	1703.286	1674.932	1944.394	1.748976	2.03E-147	ASS1
2105.62	2110.26	2066.917	452.2733	423.9804	501.4378	2.18929	6.99E-147	IFITM1
682.1021	624.1613	709.961	2573.601	2563.822	2596.051	-1.93856	5.01E-146	KIF18A
1740.753	1906.169	1945.549	5598.438	5723.735	5581.405	-1.59601	6.13E-146	NDC1
3896.699	3886.642	4044.405	9635.704	9881.681	9658.496	-1.30254	6.33E-146	TOMM20
2558.557	2637.329	2456.574	686.7086	693.6906	674.9311	1.896383	1.14E-145	GRB10
1518.778	1534.644	1596.956	4703.228	5106.655	4687.491	-1.64032	1.67E-145	SKP2
4824.143	5151.807	5094.746	12930.25	12394.08	12581.43	-1.3308	2.95E-145	CCT2
5355.266	5624.387	4986.153	1263.461	968.6482	997.5862	2.305247	3.73E-145	LYPD3
1779.397	1834.836	1766.689	329.8691	323.2326	251.7768	2.571514	3.24E-143	GSN
1640.101	1761.522	1732.013	4872.311	4972.324	5137.093	-1.54542	3.69E-143	OLA1
1475.641	1997.316	1691.861	192.9423	168.9625	206.2877	3.183903	2.21E-142	EPPK1
4041.388	4197.732	3889.272	1289.394	1190.083	1154.153	1.738663	2.82E-141	LTBP3

1606.849	1642.634	1726.537	4690.78	4973.374	4907.532	-1.55005	3.25E-140	WDR43
3196.624	2855.29	3294.292	661.8128	702.0863	559.6215	2.280667	5.13E-140	H19
3468.026	3669.672	3693.987	9013.309	9420.97	9169.753	-1.34973	3.26E-139	SUPT16H
5525.117	5533.239	5259.004	1590.218	1585.729	1818.505	1.708084	2.30E-138	NDRG1
5750.687	5915.662	6477.253	16113.79	16790.25	17279.5	-1.46771	4.96E-138	SYNCRIP
19884.31	23643.82	22444.99	64963.46	65058.95	63211.84	-1.55046	5.26E-138	HSPA8
1625.722	1716.939	1880.758	5702.171	6438.415	5931.565	-1.79068	7.21E-138	IL6ST
2011.258	2199.425	1880.758	372.3994	373.6065	428.4437	2.374732	7.32E-138	S100P
2096.633	2258.869	2228.438	8014.366	7683.07	6760.947	-1.77043	7.36E-138	RPL7P1
7673.874	7800.034	7381.586	3008.24	2955.269	2853.118	1.374151	1.89E-137	ITGA5
1159.304	1183.925	1062.204	114.1057	70.31358	111.078	3.526268	2.98E-137	RHOV
732.4285	757.9101	711.7861	2499.951	2696.053	2632.02	-1.83011	3.06E-137	AVL9
576.0573	531.0324	583.117	2174.231	2194.413	2120.003	-1.93992	1.17E-136	TTL
2647.527	2734.421	2757.715	7405.457	7050.248	6920.688	-1.39307	2.41E-136	UBA2
2040.914	1891.308	1817.792	428.4149	369.4086	387.1862	2.27856	4.59E-136	LTBP4
1878.252	1864.558	1922.735	5028.947	5084.616	4933.979	-1.40915	7.84E-136	MTRR
2779.634	2632.375	3024.178	8641.947	9916.313	9735.722	-1.74559	1.13E-135	LRRC58
3894.003	3768.745	4123.796	9999.805	10584.82	10240.33	-1.38679	1.16E-135	TMPO
3824.805	3879.707	3842.732	8960.406	9407.327	9240.631	-1.25759	2.70E-134	GSPT1
1661.669	1618.856	1644.408	4531.032	4615.509	4392.341	-1.45881	4.93E-134	DNAJC10
2046.306	2157.815	2085.168	449.1614	340.0239	345.9286	2.469508	5.75E-134	-
1695.819	1567.338	1683.648	4651.361	4914.604	4986.873	-1.5563	7.34E-134	CNOT7
2380.617	2624.45	2780.528	7695.908	8214.095	7933.085	-1.6148	1.23E-133	ATAD2
1935.768	1985.427	2090.643	5365.04	5552.674	5420.606	-1.44235	3.97E-133	TFAM
956.2011	955.0658	965.4739	2917.993	3046.572	2950.443	-1.63175	4.97E-133	STAG1
5796.52	6272.325	6271.018	14670.88	14762.7	14921.48	-1.27422	4.65E-132	XRCC5
15509.51	16994.03	16450.47	5285.166	4960.78	4290.784	1.751638	3.44E-131	LAMA5
852.8523	919.3995	993.7628	3162.801	3135.776	3222.32	-1.78339	5.27E-131	WDR75
6828.211	7010.421	6464.477	2603.684	2533.388	2491.321	1.412133	6.78E-131	-
3725.949	3275.361	3320.756	894.1734	778.6966	722.5359	2.107301	2.03E-130	CEBPB
2893.767	2904.827	2853.532	6917.915	6860.296	6875.199	-1.2553	6.71E-130	METAP2
1561.016	1580.218	1590.568	4188.715	4315.364	4419.846	-1.44959	2.16E-129	ZHX1
1174.582	1189.869	1124.257	193.9796	154.2701	161.8565	2.773229	2.74E-129	WISP2
6692.509	6631.961	7342.347	17420.82	18685.57	18910.76	-1.41249	7.61E-129	DEK
848.3589	904.5385	745.5503	56.0155	43.02771	53.95217	4.028888	2.29E-128	FBXO2
1615.836	1618.856	1816.88	5128.531	5779.356	5604.678	-1.70855	4.46E-128	QSER1
785.4509	735.1233	746.4628	45.64226	31.48369	44.4312	4.221019	6.98E-128	-
1081.118	1101.694	1022.964	141.0761	101.7973	138.583	3.070817	2.12E-127	SEZ6L2
6073.315	6238.641	6392.386	14406.36	15345.15	14447.54	-1.24066	1.92E-126	TNPO1
646.1547	631.0964	677.1093	2329.83	2344.485	2202.518	-1.81471	2.78E-126	RPF2
2168.528	2358.933	2212.012	5971.875	5787.752	5898.771	-1.39002	2.71E-125	CCNA2
1995.081	1900.224	2059.617	5159.65	5294.507	5279.907	-1.40142	3.55E-125	ECT2
961.5932	861.937	793.0027	80.91128	69.26412	75.10988	3.537697	3.36E-124	SLC25A29
2302.432	2266.795	2271.328	5513.378	5736.328	5725.277	-1.31115	3.50E-124	MYO10
7246.998	8066.541	8122.574	2322.569	2597.404	2639.425	1.632396	5.77E-124	CPS1
537.4138	551.8378	523.8015	1897.266	1944.643	1986.709	-1.85369	6.31E-123	PUM3
2122.695	2053.788	2171.86	5392.011	5288.211	5314.818	-1.33296	6.72E-123	RARS
6002.319	6111.827	6695.352	15365.88	16102.86	15737.11	-1.32746	2.58E-122	NUCKS1
10750.07	10277.86	11326.52	24788.93	25960.4	26027.16	-1.24662	4.30E-122	NAP1L1
2206.272	2074.593	2341.594	6013.368	6513.976	6251.046	-1.50329	1.18E-121	CPNE3
5687.779	5725.441	5457.939	1766.563	1855.439	2056.53	1.571048	3.91E-121	IFIT3
1197.947	1253.276	1247.451	3419.02	3467.404	3386.292	-1.47395	1.25E-120	SLC25A32
4567.119	4743.626	4323.644	1470.926	1221.567	1301.199	1.771244	1.40E-120	HSPB1
833.9799	767.8174	763.8012	73.65001	78.70923	75.10988	3.378741	1.73E-120	PRR15

14396.94	15515.86	15827.2	36075.02	34951.09	37186.8	-1.24238	2.15E-120	NCL
1774.903	1865.549	1950.111	5120.232	5732.131	5618.431	-1.55887	4.24E-120	SACS
10422.05	10142.13	10511.62	4053.863	3805.329	4299.247	1.353891	6.93E-120	SGK1
1056.854	1095.75	1027.527	184.6437	172.1108	169.2617	2.595495	8.09E-120	GDPD5
837.5747	771.7804	716.3488	50.82888	52.47282	30.67868	4.116281	2.65E-119	HES4
1560.118	1625.792	1507.526	338.1677	362.0624	377.6652	2.122445	4.35E-119	HERC5
5898.071	5909.717	5815.657	12661.58	13411	13422.45	-1.16418	8.27E-119	EIF2S3
5099.14	5072.549	5431.475	12727.97	13195.86	12152.99	-1.28701	8.84E-119	EIF1AX
9405.64	9449.604	10029.8	21893.76	23988.47	23683.94	-1.26803	4.79E-118	IPO7
462.8229	541.9305	489.1248	1881.706	2014.956	2020.562	-1.98707	1.06E-117	NRP1
1175.48	1296.868	1308.591	3663.829	3740.262	3917.351	-1.58255	1.10E-117	BCCIP
5805.507	6030.587	6156.949	13254.93	13413.1	13068.06	-1.14305	1.28E-117	MCM4
3191.231	3202.046	3346.307	7473.92	7854.131	7763.823	-1.24538	3.59E-117	ZFR
8653.441	8805.628	8967.592	18619.97	18420.06	18170.24	-1.06295	5.81E-117	CCT6A
5518.826	5727.423	5783.718	12295.4	12365.74	12278.88	-1.11716	5.82E-117	NSUN2
836.676	808.4375	773.8392	82.98593	96.54998	84.63085	3.19499	7.68E-117	AL645608.1
2873.097	2820.615	2921.973	6627.464	6597.932	6669.969	-1.20728	1.05E-116	ACTR3
1500.804	1537.616	1599.694	4105.729	4241.903	4044.297	-1.41779	1.10E-116	NUP107
724.3403	738.0955	658.8584	24.89578	15.74185	23.27348	5.052049	1.29E-116	-
4237.301	4845.671	4852.008	12194.78	12568.29	12287.34	-1.41095	2.97E-116	DHX9
1483.729	1437.552	1510.264	4290.373	4984.918	5050.346	-1.6925	3.23E-116	KIF20B
2699.651	2898.882	2862.657	6771.652	7057.594	7192.565	-1.31313	3.84E-116	USP7
6460.648	6961.875	6479.078	2705.341	2604.751	2607.688	1.329555	8.21E-116	GALNT2
2194.589	2230.138	2190.111	5705.283	6303.035	6558.891	-1.48901	1.06E-115	AHCTF1
415.1926	371.5246	376.8816	1540.426	1601.47	1651.36	-2.04157	1.82E-115	H3F3AP4
2689.765	2629.403	2622.658	783.1797	834.3178	882.2767	1.667877	4.86E-115	MAP7
2820.074	2913.743	2871.783	6723.935	6651.454	6510.228	-1.20851	2.20E-114	EIF3M
10159.64	10499.78	10680.44	22030.69	23396.58	23418.42	-1.13538	6.45E-114	CSDE1
2645.729	2577.885	2541.441	863.0537	857.4058	798.7037	1.623984	2.12E-113	GABARAPL1
6608.932	6458.583	6629.648	14497.64	15781.72	15996.29	-1.2322	6.01E-113	TCP1
3728.645	3395.239	3638.322	1248.938	1129.215	1199.642	1.58897	9.80E-113	DUSP5
488.8848	456.7275	483.6495	1856.81	1911.06	1706.37	-1.93683	1.87E-112	NIFK
2033.725	1896.261	1803.191	503.1022	515.2831	504.6115	1.912517	1.88E-112	KLF4
1392.962	1594.088	1498.401	4424.187	4162.144	4387.052	-1.53279	3.50E-112	DNTTIP2
6423.802	6234.678	6308.432	13406.38	13087.77	13187.6	-1.06494	9.22E-112	EIF3E
7866.192	8025.921	8499.455	18715.4	18329.8	17730.16	-1.16714	1.76E-111	HMGB1
637.1679	794.5672	695.3602	23.85846	29.38478	40.19966	4.509894	2.58E-111	MUC16
1066.739	1094.759	1107.831	2963.635	3014.039	2984.295	-1.4549	2.84E-111	NLN
1528.664	1614.893	1626.157	4069.423	4344.749	4353.2	-1.42063	2.93E-111	GFM1
1024.501	894.6312	939.9226	129.6655	133.281	99.44125	2.979692	9.15E-111	SLC9A3-AS1
481.6953	519.1437	535.6646	1969.879	1831.301	1831.2	-1.87445	9.29E-111	POP1
1684.136	1672.356	1784.94	4484.352	4759.285	5020.725	-1.47199	1.99E-110	CCAR1
1486.425	1389.997	1476.5	247.9205	323.2326	294.0922	2.331326	2.23E-110	DDX60
1217.719	1250.304	1252.013	283.1895	274.9576	261.2978	2.182363	2.66E-110	RNF44
6538.834	7367.084	6738.241	2423.189	2149.287	2039.604	1.642343	2.71E-110	BST2
414.2939	379.4504	413.3834	1691.876	1906.862	1617.507	-2.11066	3.28E-110	TMEM64
484.3914	494.3754	518.3263	1727.145	1739.999	1765.611	-1.80534	6.75E-110	DNAJC2
808.8168	871.8443	845.0178	101.6578	104.9456	71.93623	3.179726	1.83E-109	VTN
1866.569	1879.419	2030.415	4886.834	5010.105	4841.943	-1.35126	2.41E-109	DENR
1243.78	1132.407	1242.888	3349.52	3419.129	3357.729	-1.48382	2.46E-109	RFC3
2066.077	2080.538	2072.392	645.2156	659.0586	668.5837	1.656434	6.01E-109	P4HA2
3837.386	3605.274	4079.082	9869.102	11164.12	11118.38	-1.48033	1.20E-108	IMPAD1
4764.829	4682.2	4684.1	1788.347	1634.004	1850.242	1.422335	1.35E-108	IFIT1
1801.864	1821.956	1833.305	4360.911	4590.322	4638.829	-1.31634	1.40E-108	TOPBP1

1437.896	1520.774	1489.275	3768.599	3975.341	3836.951	-1.38079	1.97E-108	DHX33
3203.813	3302.11	3453.987	8210.421	8686.35	7874.901	-1.3145	4.35E-108	ARPP19
984.9591	1072.963	972.7743	180.4944	147.9733	148.104	2.66806	7.83E-108	OAS1
481.6953	520.1344	557.5657	2076.723	1853.34	1982.478	-1.92299	1.32E-107	ANXA3
546.4007	596.4208	600.4554	2110.955	2105.209	1924.294	-1.81703	1.71E-107	UTP15
639.8639	640.013	710.8735	2135.85	2172.375	2183.476	-1.70493	1.86E-107	NOC3L
546.4007	634.0686	629.6569	2104.731	2126.199	2264.933	-1.84415	1.86E-107	FAM35A
1736.26	1496.996	1610.644	4528.957	5030.044	4845.116	-1.5717	3.21E-107	COPS2
1626.62	1639.662	1619.77	453.3106	394.5956	387.1862	1.983722	3.51E-107	FGFR3
1865.671	1903.197	2003.039	4914.842	5146.534	5508.411	-1.43156	7.44E-107	SMC3
2915.335	2926.623	3015.053	6539.291	6857.148	6800.089	-1.18916	7.91E-107	GDI2
954.4038	911.4736	978.2495	2696.005	2787.356	2670.103	-1.51901	1.02E-106	BAZ1A
2041.813	1958.678	2118.02	5556.945	5603.047	5079.967	-1.40809	1.61E-106	SPCS3
2247.612	2360.915	2333.381	6203.198	5841.274	5589.868	-1.34516	3.13E-106	EMP1
5223.159	5731.386	5185.088	2075.686	2040.143	2105.192	1.37521	3.65E-106	EHBP1L1
7073.552	7216.493	7489.267	15161.53	15045.01	15066.41	-1.05569	4.35E-106	TPM3
5284.269	5157.752	5358.471	11180.28	12029.92	11657.9	-1.14185	9.48E-106	NAA50
1054.158	1028.38	1087.755	2905.545	2936.379	2834.076	-1.45216	2.87E-105	NCEH1
869.9274	908.5014	808.5159	107.8817	77.65977	114.2517	3.109041	3.95E-105	OAS2
3938.938	3823.235	4167.599	8968.704	9152.309	9425.761	-1.20715	6.35E-105	SRP72
3061.821	3271.398	3036.954	991.6819	906.7303	796.5879	1.797433	8.05E-105	AKR1C1
5769.559	5539.184	5497.178	2427.338	2356.029	2294.554	1.247574	9.25E-105	ZFP36L2
1525.069	1500.959	1300.378	231.3233	174.2098	136.4673	2.99584	1.41E-104	CITED4
1298.6	1381.081	1289.428	301.8613	280.2048	238.0243	2.274316	5.82E-104	DMPK
1379.482	1386.034	1401.671	3430.431	3521.975	3575.654	-1.33706	5.88E-104	THAP12
2400.388	2511.506	2335.206	649.3649	538.3711	472.8749	2.125214	1.76E-103	TNFSF9
1183.569	1187.888	1190.873	3316.325	3226.029	3060.463	-1.43069	1.82E-103	TDG
5721.929	5879.995	5692.463	2436.674	2317.2	2513.536	1.250749	1.98E-103	WARS
2616.972	2502.589	2732.163	6388.879	6629.416	7103.702	-1.35745	3.17E-103	EIF5B
2626.857	2529.339	2776.878	6372.282	7034.506	6818.073	-1.34995	4.58E-103	FAR1
1147.621	1363.247	1139.77	185.681	183.6549	227.4454	2.612772	5.41E-103	COX6A1
660.5337	660.8184	617.7938	18.67183	9.445107	16.92617	5.427111	5.93E-103	ALPI
2278.167	2351.998	2390.871	5417.944	5624.037	5858.571	-1.26735	8.09E-103	USP1
3437.471	3687.505	3356.345	1014.503	887.8401	753.2146	1.980372	8.61E-103	PLD3
1907.909	1849.697	1884.408	4463.606	4525.256	4372.241	-1.24362	1.18E-102	SELENOT
1224.908	1194.823	1095.968	237.5472	252.919	217.9244	2.310994	1.51E-102	CEBPD
993.0472	1020.454	1098.706	2875.463	3052.869	3098.547	-1.53617	1.91E-102	MRPL19
1416.328	1309.748	1410.796	3644.12	4076.088	3886.672	-1.48793	2.15E-102	IKBIP
819.601	843.1131	812.1661	2349.539	2322.447	2389.764	-1.51292	2.21E-102	POLR1B
1131.445	1257.239	1205.474	3208.444	3329.925	3269.925	-1.44886	4.11E-102	MYO1B
864.5352	921.3809	878.782	158.7106	136.4293	140.6988	2.611538	5.03E-102	ALPP
4617.445	4815.949	4779.917	9850.43	10159.79	9947.299	-1.07576	5.80E-102	ETF1
3194.826	3430.906	3087.144	933.5917	1065.198	873.8136	1.757337	1.47E-101	MCAM
1386.671	1477.182	1514.827	3997.847	4584.025	4134.217	-1.53821	2.04E-101	CDK6
1818.939	1780.346	1861.594	4331.866	4332.156	4267.511	-1.24352	2.57E-101	EFEMP1
641.6613	622.1798	555.7406	37.34367	39.87934	44.4312	3.903247	4.45E-101	CDK18
1866.569	1818.984	1776.727	4349.5	4359.442	4580.645	-1.28266	4.52E-101	COPS3
1235.692	1228.508	1183.572	3126.495	3408.634	3356.671	-1.43924	5.45E-101	TTK
656.9389	641.0037	587.6798	32.15705	52.47282	45.48908	3.858211	1.49E-100	DBP
890.5971	863.9185	912.5462	2672.147	2485.113	2515.652	-1.52426	1.89E-100	NUDCD1
1791.08	1836.817	1870.72	4513.397	5097.209	5029.188	-1.41276	2.21E-100	CHD1
4082.727	4187.825	4025.241	1638.972	1589.926	1463.056	1.389727	2.22E-100	SREBF2
2424.653	2544.2	2394.521	5696.984	5723.735	5533.8	-1.20349	2.31E-100	MCFD2
658.7363	602.3652	688.0598	2062.2	2085.27	2077.687	-1.67438	3.02E-100	CIP2A

5939.411	6354.556	6187.063	13195.8	12783.43	12940.06	-1.07457	3.84E-100	CCT8
945.4169	955.0658	900.6831	178.4198	152.1712	143.8725	2.561021	5.25E-100	PARP10
2604.39	2637.329	2841.669	6444.895	7132.105	7081.487	-1.35361	5.36E-100	PRPF40A
815.1075	924.3531	935.3599	2673.184	2692.905	2660.582	-1.58586	8.96E-100	GEMIN5
2239.524	2110.26	2191.023	738.5748	703.1358	734.1727	1.588029	1.00E-99	TBX3
2525.306	2580.857	2990.414	7236.373	7777.521	7759.591	-1.49184	2.24E-99	DDX46
1243.78	1216.619	1356.956	3566.32	4111.77	4101.423	-1.62533	2.33E-99	HEATR1
787.2483	828.2521	852.3182	2384.808	2354.98	2333.696	-1.51936	3.38E-99	PSMG1
287.5793	267.4977	268.2886	1293.543	1168.045	1164.732	-2.13844	6.13E-99	FST
754.8956	810.4189	882.4322	2495.802	2769.515	2659.525	-1.69496	6.94E-99	GNPNAT1
2374.327	2458.997	2495.814	6278.923	6198.089	5658.63	-1.3072	9.85E-99	POFUT1
3963.202	3863.855	3440.299	1309.103	1195.331	1165.79	1.618054	1.40E-98	HPCAL1
638.0665	568.6803	577.6418	47.71691	45.12662	39.14177	3.756657	1.42E-98	SPEG
14955.02	15343.47	15344.46	35517.98	34633.11	31566.25	-1.15612	1.82E-98	TXNRD1
4490.73	4570.248	4190.412	1693.95	1598.322	1758.206	1.391586	2.67E-98	LGALS3
4145.635	4198.723	3995.127	1543.538	1656.042	1459.882	1.404949	2.69E-98	JUP
11035.86	11010.01	11203.33	4720.862	4426.607	3987.171	1.339887	2.95E-98	FASN
2736.497	2843.401	3005.015	7112.932	6759.548	6640.348	-1.25668	3.24E-98	PA2G4
1135.938	1110.611	1073.154	243.7712	223.5342	254.9504	2.200549	4.33E-98	EML2
4208.543	4184.853	4044.405	1571.546	1435.656	1646.07	1.418425	4.59E-98	CTSA
2043.61	2001.279	2060.529	698.1191	650.6629	670.6995	1.596121	7.06E-98	OLR1
1617.634	1721.893	1872.545	4698.041	5263.024	5339.149	-1.55361	7.64E-98	RIF1
6275.519	6582.425	6133.223	2662.811	2772.664	2550.562	1.249652	9.26E-98	LDLR
942.7209	914.4458	857.7934	167.0092	134.3304	134.3515	2.638979	1.83E-97	UBE2L6
2309.621	2534.293	2446.536	5813.164	5628.234	5681.904	-1.2322	2.08E-97	EI24
697.3798	664.7813	539.3148	43.56761	33.5826	33.85234	4.097414	2.23E-97	BIRC7
12163.71	13062.8	12753.75	5107.784	4952.384	4301.363	1.402953	2.79E-97	GANAB
4412.545	4496.933	4489.727	9041.317	9366.398	9458.556	-1.0564	3.06E-97	IARS
1068.537	1019.463	1149.808	2925.254	3071.759	3063.637	-1.4841	3.41E-97	NOL8
4000.947	4288.88	4408.511	9323.469	9742.103	9792.848	-1.18443	5.70E-97	LRPPRC
650.6481	581.5598	626.9193	1919.05	1946.742	1958.146	-1.6465	1.22E-96	HMGA2
2411.173	2508.534	2227.525	790.441	768.202	783.8933	1.609089	1.71E-96	KRT19
1384.874	1512.848	1479.237	3838.099	4447.596	4264.337	-1.51995	1.91E-96	SETD7
1928.579	1568.329	1793.153	341.2796	398.7934	417.8648	2.192408	2.01E-96	IL6
3388.044	3393.258	3387.372	7606.698	7243.348	7161.886	-1.11418	2.02E-96	PSME3
1366.002	1506.904	1502.964	3717.77	3723.471	3762.899	-1.35674	2.17E-96	NOL11
956.2011	990.7322	904.3333	158.7106	186.8032	179.8406	2.440323	2.32E-96	ISG20
576.9559	594.4393	594.0676	1793.533	1838.648	1943.336	-1.65918	2.52E-96	ZNF131
1342.636	1340.461	1372.47	3490.596	3340.42	3299.545	-1.32071	2.85E-96	DCAF13
1403.746	1532.663	1422.66	3813.204	3953.302	3647.59	-1.3892	3.82E-96	NAT10
949.9103	959.0287	1014.751	2673.184	2714.944	2938.806	-1.50984	5.42E-96	SBDS
823.1957	780.697	756.5008	100.6204	95.50053	126.9463	2.869771	7.96E-96	MUC13
886.1037	813.3911	831.3296	2495.802	2841.928	2545.273	-1.63871	7.97E-96	MMS22L
3714.266	3978.78	3774.291	1130.683	892.0379	807.1668	2.018388	9.01E-96	PTMS
2294.344	2388.655	2434.673	5434.541	5511.745	5268.271	-1.1879	1.73E-95	SSR3
2648.426	2565.996	2562.43	5920.009	5902.142	5612.084	-1.1646	1.76E-95	CACYBP
2027.434	2322.276	1993.913	607.8719	565.657	616.7473	1.824752	1.76E-95	SERF2
1968.121	2039.918	1807.754	523.8487	467.0081	396.7071	2.066911	1.16E-94	IGSF8
975.0735	845.0945	871.4816	150.412	107.0445	114.2517	2.85583	1.35E-94	FOXD1
2265.586	2193.481	2147.221	575.7149	428.1782	401.9966	2.23194	1.44E-94	SOCS3
1581.686	1617.866	1545.853	3939.757	3753.905	4084.497	-1.31167	1.98E-94	SRPRA
789.9444	880.7609	733.6872	104.7697	62.96738	80.39931	3.275243	3.59E-94	FIBCD1
1239.287	1071.972	1210.036	3204.294	3322.579	3308.009	-1.48101	6.27E-94	ZMPSTE24
9260.952	9329.725	8942.04	19685.3	18205.97	19208.03	-1.05236	7.34E-94	RPL23

1029.893	1099.713	1158.934	2948.075	2942.676	2952.559	-1.42727	1.11E-93	FARSB
9323.86	10076.74	10257.02	22698.73	21584.17	21054.04	-1.13955	1.63E-93	PAICS
1259.058	1296.868	1210.036	317.4212	287.551	254.9504	2.13018	1.89E-93	FBXL19
584.1454	561.7451	490.9499	23.85846	23.08804	20.09983	4.60892	2.02E-93	CA9
3424.89	3624.098	3438.474	1423.209	1391.579	1323.415	1.341361	2.32E-93	GATA2
3007.001	3266.444	3123.646	1109.937	1144.957	991.2389	1.533227	3.94E-93	PLXND1
3258.633	3114.862	3286.079	6849.451	7266.436	7057.155	-1.13201	4.28E-93	TWF1
2447.12	2475.84	2478.476	5381.638	5852.818	5800.387	-1.20261	6.00E-93	NUP50
1554.726	1541.579	1446.386	343.3543	226.6826	263.4135	2.445861	1.24E-92	MEGF6
1041.576	1135.379	1212.774	3093.301	3336.222	3337.629	-1.52688	1.35E-92	OXCT1
4006.339	3915.374	3836.344	1579.845	1461.893	1358.325	1.417931	1.64E-92	FAM83A
6396.842	6593.323	6466.303	15078.54	14249.52	13454.19	-1.13683	3.55E-92	TUBA1C
2475.878	2574.913	2561.517	5433.504	5502.3	5492.542	-1.1099	8.20E-92	AMD1
5818.987	6014.735	5551.931	1902.452	1509.118	1396.409	1.854192	8.75E-92	AES
562.577	527.0695	541.1399	1668.017	1793.521	1711.659	-1.665	8.76E-92	UTP23
3972.189	3955.994	3839.082	1601.628	1409.42	1392.178	1.417993	1.50E-91	SEMA3B
661.4324	615.2447	600.4554	65.35142	65.06629	78.28354	3.169675	1.92E-91	PLCG2
6429.194	6713.201	6437.101	13441.65	13466.62	12830.04	-1.02129	2.01E-91	CAV1
392.7255	394.3114	420.6838	1370.305	1484.981	1408.046	-1.81945	3.38E-91	BRIP1
2800.303	2613.551	2792.391	6187.638	6923.264	6674.201	-1.26937	8.24E-91	CAV2
5920.539	6329.788	5692.463	2551.817	2549.129	2592.878	1.221491	9.48E-91	JADE2
2643.932	2647.236	2665.548	5599.476	5631.383	5544.379	-1.07608	1.13E-90	GOT2
6437.283	6350.593	6431.626	12733.15	13903.2	13308.2	-1.05539	1.41E-90	NORAD
9127.946	7773.285	9841.811	725.0896	1008.528	883.3345	3.353291	1.67E-90	CTGF
663.2297	778.7155	695.3602	2214.687	2150.336	2188.765	-1.61764	2.44E-90	NUP88
1498.108	1454.395	1635.283	3831.875	4038.308	4200.864	-1.39534	3.06E-90	ZC3H15
1754.234	1659.476	1634.37	4495.763	4275.485	4025.255	-1.34184	3.24E-90	ATP1B3
1456.769	1135.379	1240.15	258.2937	258.1663	250.7189	2.321033	3.93E-90	ZC3H12A
434.9637	434.9314	345.855	1586.069	1712.713	1587.886	-2.00769	3.95E-90	GPAT3
9070.431	9687.379	9660.214	19280.74	21116.11	20406.61	-1.0974	4.03E-90	FBN2
2281.762	2305.434	2198.324	733.3882	787.0923	660.1207	1.637554	4.30E-90	BMP1
756.693	754.9379	747.3754	118.255	135.3799	123.7726	2.581631	4.39E-90	PBXIP1
650.6481	638.0315	698.0979	1989.588	2284.666	2093.556	-1.67999	4.44E-90	GUF1
3606.424	3623.108	4146.61	11353.51	11561.86	9752.648	-1.5218	6.35E-90	THBS1
2602.593	2556.089	2643.646	5487.445	5510.695	5556.016	-1.0851	1.07E-89	CKAP2
2478.574	2493.673	2632.696	5728.104	6269.452	5806.734	-1.22714	1.16E-89	PAK2
4930.187	4890.254	5001.666	2104.731	2023.352	2238.486	1.219238	1.20E-89	RPL23A
3063.618	3618.154	3667.523	1087.116	1099.83	962.676	1.716038	1.37E-89	HSPG2
4618.344	4969.513	4946.913	10044.41	10217.51	10538.66	-1.08356	1.66E-89	CALU
901.3813	949.1214	965.4739	2431.488	2583.762	2559.025	-1.42763	1.74E-89	CEBPZ
762.9838	853.0204	780.227	135.8895	125.9348	143.8725	2.561988	5.16E-89	ADGRG1
1910.605	2069.64	1843.343	619.2825	528.926	542.6954	1.783649	5.89E-89	BLVRB
1042.475	1032.343	1061.291	2588.124	2749.576	2771.66	-1.37045	1.18E-88	NCAPG
822.297	812.4004	874.2193	2366.136	2763.219	2781.181	-1.65643	1.40E-88	MIS18BP1
936.4301	852.0297	789.3525	2786.253	3369.804	3586.232	-1.9179	1.64E-88	CENPE
476.3032	461.6812	497.3377	1522.792	1546.899	1508.545	-1.67296	2.69E-88	MAN2A1
2806.594	2982.104	2736.726	1093.34	1040.011	987.0073	1.449747	2.80E-88	LRP10
4143.838	4410.74	4447.75	9883.624	9376.892	9138.016	-1.12716	3.12E-88	RSL1D1
4552.74	4497.924	4221.439	1878.594	1682.279	1715.891	1.330557	4.69E-88	HMG20B
1130.546	1274.082	1304.941	3248.899	3375.052	3453.997	-1.4422	6.11E-88	PNPT1
3135.513	3214.926	3256.877	6872.272	7565.531	7697.176	-1.20413	1.28E-87	ITGA6
1484.628	1569.32	1514.827	3881.667	3686.74	3566.133	-1.28547	1.50E-87	MRTO4
2241.321	2250.944	2585.243	6032.04	6560.152	5999.27	-1.39317	2.60E-87	TMED7
3282.897	3493.322	3092.619	1015.54	855.3069	738.4042	1.918879	3.20E-87	MSLN

569.7665	554.81	519.2388	42.53029	48.27499	57.12583	3.474938	3.89E-87	MEIS3
1737.159	1832.855	1702.811	462.6466	348.4195	322.6551	2.216894	3.94E-87	CSRNP1
580.5507	590.4764	590.4174	1735.443	1947.791	2017.388	-1.69436	4.02E-87	EEA1
3246.051	3278.333	3158.322	1326.738	1219.468	1329.762	1.320787	5.79E-87	CTSB
722.543	700.4476	799.3905	2132.738	2313.002	2197.229	-1.5792	6.44E-87	CHML
1757.828	1763.503	1826.005	3958.429	4218.815	4138.449	-1.20352	6.60E-87	MRPS35
2697.853	2951.391	3012.315	6489.5	6977.835	6804.321	-1.2269	7.81E-87	KIF11
1611.343	1770.438	1738.401	4137.886	4060.347	3972.361	-1.24949	2.01E-86	DDX18
1525.968	1590.125	1649.884	3659.68	3839.961	3796.752	-1.24506	2.44E-86	VPS26A
1034.387	1034.324	1082.28	2586.049	2741.18	2697.608	-1.34852	2.71E-86	KLHL5
28431.71	30888.06	30645.13	60287.2	59948.09	58036.67	-0.98667	3.16E-86	HSP90AB1
2565.746	2432.247	2679.236	5861.919	6398.535	6650.927	-1.30034	8.60E-86	KIF23
1983.398	2124.13	1740.226	551.8564	549.9151	569.1425	1.806975	1.14E-85	COX8A
1236.591	1219.591	1221.899	261.4057	183.6549	162.9144	2.596062	1.19E-85	PABPN1
2027.434	2138.991	2122.583	5303.838	5059.429	4718.17	-1.26206	1.74E-85	MCMBP
690.1903	617.2261	695.3602	1942.908	2067.429	1971.899	-1.57778	2.01E-85	NCBP3
14872.34	15753.63	14748.57	6638.874	6240.067	5549.668	1.299857	2.11E-85	LGALS3BP
3077.997	3126.751	3245.927	6536.179	6964.192	6677.374	-1.09425	2.50E-85	RRM2
1393.861	1374.146	1577.792	3658.642	3924.967	3898.309	-1.40135	7.39E-85	TCERG1
3215.496	3309.045	3358.17	6877.459	7504.662	7060.329	-1.11752	8.23E-85	TFRC
2393.199	2438.192	2632.696	5481.221	5706.944	5606.794	-1.16991	9.39E-85	ANP32E
1461.262	1507.894	1578.705	3520.678	3651.059	3542.859	-1.23632	1.04E-84	DIS3
816.9049	800.5116	768.3639	132.7775	160.5668	128.0042	2.501348	1.40E-84	EVI5L
3592.045	3850.976	3747.827	1586.069	1592.025	1482.098	1.263659	3.06E-84	PTPRS
1892.631	1761.522	1727.45	585.0508	574.0526	512.0167	1.687161	3.77E-84	KIAA1522
4647.102	5264.751	5058.244	11587.95	11465.31	10785.14	-1.17678	7.50E-84	RRM1
1228.503	1179.962	1210.036	3549.723	3276.403	3027.669	-1.44517	1.11E-83	GLRX3
1162	1084.852	1090.493	2782.103	2723.339	2729.345	-1.30273	3.78E-83	TAF9B
8740.613	8766.989	8820.672	4405.516	4466.486	4595.455	0.967161	4.27E-83	PCDH7
549.0967	568.6803	550.2654	1656.607	1769.383	1615.391	-1.59598	5.37E-83	MRPL35
2794.911	2765.134	3003.19	6061.085	6222.227	6141.026	-1.10523	7.95E-83	HDAC2
1514.285	1567.338	1632.545	3770.673	3781.191	4174.417	-1.31466	9.42E-83	GNL3
929.2406	984.7878	969.1241	199.1662	228.7815	229.5612	2.132701	1.11E-82	APOL1
1911.504	1923.011	2093.381	4449.083	4642.795	4529.866	-1.20018	1.11E-82	NBN
502.3651	554.81	547.5277	1606.815	1616.163	1622.797	-1.59503	1.58E-82	RBL1
1963.627	2056.76	1892.621	709.5297	638.0695	634.7314	1.576333	1.79E-82	RBM15B
535.6164	494.3754	541.1399	42.53029	32.53315	53.95217	3.607183	2.35E-82	GBP1
657.8376	570.6617	572.1665	40.45564	54.57173	70.87834	3.441925	2.43E-82	IFI44L
1468.452	1573.283	1540.378	3689.762	3575.498	3473.039	-1.229	2.73E-82	NUS1
4206.746	4035.252	4024.329	8331.787	8639.125	8103.404	-1.03144	6.02E-82	CS
825.8918	729.1789	761.0635	150.412	133.281	129.0621	2.488353	6.09E-82	TMC6
669.5205	637.0408	638.7824	106.8444	90.25325	102.6149	2.698353	6.57E-82	TCIM
633.5731	658.8369	582.2045	67.42607	67.16521	92.03605	3.048761	6.70E-82	ALDOC
1715.59	1724.865	1830.568	3942.869	3975.341	3891.961	-1.16377	8.99E-82	ZNF146
1567.307	1685.235	1652.621	4098.468	3758.103	4214.617	-1.29944	2.21E-81	SNRPD1
1973.513	1868.521	2008.514	4515.472	5190.611	4778.47	-1.30761	3.39E-81	NEK7
3512.961	3628.061	3655.66	7783.043	8089.21	7316.337	-1.10289	3.97E-81	NCKAP1
2092.139	2177.629	2045.016	4506.136	4767.68	4613.439	-1.13718	4.58E-81	OPA1
1995.98	1836.817	2115.282	4915.879	5487.607	5797.213	-1.44519	4.65E-81	PRPF4B
822.297	800.5116	912.5462	2267.591	2324.546	2365.432	-1.45591	6.74E-81	RAD18
516.744	617.2261	598.6303	1820.504	1843.895	1775.132	-1.65151	6.82E-81	FAM111B
1188.062	1284.98	1222.812	358.9142	355.7657	331.1182	1.820889	7.60E-81	SLC44A2
292.9714	320.0065	313.0034	1135.87	1126.067	1086.449	-1.85515	8.16E-81	AOX1
582.3481	626.1427	626.0067	86.0979	71.36303	92.03605	2.87823	8.39E-81	FOXO4

984.0604	981.8156	1015.664	2455.346	2514.497	2404.574	-1.30638	1.03E-80	TOR1AIP1
5988.839	6527.934	6059.307	2776.917	2785.257	2912.359	1.132121	1.12E-80	PRKCSH
1184.467	1278.045	1229.2	2904.508	2961.566	2926.112	-1.25234	1.17E-80	DLGAP5
1465.756	1495.015	1549.503	3363.005	3442.217	3414.855	-1.1801	1.28E-80	ECHDC1
1201.542	1253.276	1198.173	358.9142	301.194	303.6132	1.921905	2.13E-80	KDM4B
2598.998	2637.329	2525.015	996.8685	931.9172	833.6139	1.490123	2.39E-80	NUCB1
713.5561	689.5496	741.9001	86.0979	102.8467	128.0042	2.759766	3.27E-80	TMEM45A
795.3365	819.3355	856.8809	2159.709	2111.506	2205.692	-1.38988	4.33E-80	RIOK1
1852.19	1954.715	2072.392	4442.859	4617.608	4456.872	-1.20114	4.49E-80	TOMM70
1887.239	1976.511	1972.012	4380.62	4151.649	4434.657	-1.15199	5.09E-80	SSB
1073.03	1081.88	1166.234	2686.67	2727.537	2774.834	-1.30184	5.36E-80	PPIG
3764.593	3764.782	4028.892	7750.886	8110.199	7930.969	-1.04147	5.90E-80	GNG12
817.8036	962.9917	918.0215	2453.272	2516.596	2468.047	-1.46335	7.80E-80	FAM98B
3757.403	3849.985	3944.937	7891.962	7931.791	7525.799	-1.01524	8.17E-80	PPP2CA
913.9629	935.2512	921.6717	208.5022	174.2098	144.9303	2.391921	9.13E-80	PLEKHH3
1114.37	1141.323	1088.668	290.4508	292.7983	322.6551	1.884358	9.88E-80	PHF21A
1359.711	1450.432	1319.542	378.6233	290.6994	280.3397	2.11986	1.58E-79	FLYWCH2
1036.184	1049.185	1064.029	2847.455	2662.471	2548.447	-1.35549	1.71E-79	CPA4
1455.87	1297.859	1356.044	3288.318	3305.787	3468.807	-1.2914	1.73E-79	PITPNB
1142.229	1141.323	1023.877	287.3388	231.9299	240.14	2.122264	1.84E-79	CCDC71L
1714.692	1727.837	1912.697	4087.057	4277.584	4319.347	-1.24381	3.52E-79	ALCAM
4383.787	4277.982	4689.575	9473.881	10411.66	10793.61	-1.20015	3.59E-79	BCLAF1
882.5089	860.9463	781.1396	171.1585	171.0614	184.0721	2.262188	3.95E-79	LZTS2
2848.832	2936.53	3046.079	6052.786	6510.827	6255.278	-1.09146	4.88E-79	NUP155
3063.618	3106.936	2869.045	1219.893	1269.842	1250.421	1.273076	5.19E-79	INPPL1
1435.2	1506.904	1168.972	278.0029	201.4956	178.7827	2.641888	6.72E-79	PSCA
968.7827	990.7322	964.5614	255.1817	208.8418	212.635	2.110982	6.79E-79	CRIP2
709.9614	715.3086	699.0104	1896.229	2012.857	2169.724	-1.51684	8.01E-79	TPP2
695.5824	788.6228	745.5503	2012.409	2181.82	2082.977	-1.49395	9.98E-79	PGM2
1561.016	1455.386	1538.553	3531.051	3530.371	3381.003	-1.1966	1.55E-78	FAM208B
1595.166	1639.662	1665.397	3532.089	3653.158	3613.737	-1.14003	1.88E-78	HSPA13
3868.84	4221.51	3876.496	1638.972	1486.03	1376.309	1.410336	3.28E-78	PRDX5
854.6497	798.5301	815.8163	2239.583	2091.566	2145.392	-1.39094	3.93E-78	FBXO5
2078.659	2114.222	2112.544	4508.211	4441.299	4316.174	-1.07311	4.14E-78	NOP58
541.0085	536.9768	523.8015	69.50072	55.62119	68.76257	3.046285	5.21E-78	TMPRSS3
1014.616	1078.907	987.375	262.443	204.644	205.2298	2.195394	7.88E-78	SIPA1
645.256	603.3559	592.2425	73.65001	91.3027	97.32548	2.812079	1.03E-77	SNAI1
1994.183	2109.269	1969.275	4343.276	4358.392	4326.752	-1.10156	1.26E-77	DLD
238.1516	286.3216	229.9616	1001.018	1114.523	1081.159	-2.08517	1.57E-77	KBTBD6
603.0178	702.4291	661.596	1873.407	2072.676	2146.45	-1.63176	1.73E-77	MPHOSPH10
4514.096	4566.285	4695.05	8902.316	9618.267	9128.495	-1.00512	2.56E-77	XPOT
223.7726	243.7201	254.6004	1070.519	947.6591	962.676	-2.04594	3.52E-77	MRPL1
421.4834	468.6163	430.7218	23.85846	12.59348	17.98406	4.598912	3.95E-77	TLE2
1035.285	1173.027	1186.31	2934.59	3381.348	3139.805	-1.47827	4.19E-77	ANAPC1
1633.81	1651.551	1738.401	3726.068	4113.869	3872.919	-1.22117	4.57E-77	LYPLA1
2340.176	2174.657	2388.133	4909.655	4996.462	5072.562	-1.11726	6.68E-77	NUDT21
3215.496	3424.961	3612.77	7175.171	7366.134	7322.685	-1.09253	8.19E-77	POLR2B
1124.255	1158.166	1060.379	272.8162	298.0456	317.3657	1.912136	1.12E-76	SLC12A3
1709.299	1899.234	1677.26	601.648	545.7173	570.2004	1.621302	1.17E-76	ALDH3B1
24211.48	24227.36	25025.67	42967	45279.84	44600.46	-0.85464	1.42E-76	YWHAZ
1576.294	1187.888	1197.261	252.0698	216.188	275.0503	2.414458	1.60E-76	ZFP36
674.014	717.2901	603.1931	112.031	83.95651	87.80451	2.81208	1.73E-76	ARSA
3382.651	3684.533	3226.763	1352.671	1165.946	1164.732	1.482381	1.74E-76	MAF1
641.6613	553.8193	530.1894	46.67959	40.9288	70.87834	3.446104	1.92E-76	NR4A2

1011.021	1004.602	977.337	260.3684	273.9081	272.9345	1.890629	2.89E-76	CALCOCO1
715.3535	781.6877	754.6757	1940.833	2120.951	2112.598	-1.45573	3.01E-76	ADAM12
11673.92	12798.28	12502.8	24729.81	23520.42	24168.46	-0.96989	3.12E-76	HNRNPC
2151.453	2098.371	2012.164	803.9262	738.8173	800.8195	1.417899	3.69E-76	TMEM173
539.2112	570.6617	611.406	1716.771	1741.048	1620.681	-1.56102	5.48E-76	CLSPN
5659.021	6023.652	5728.965	11532.97	11510.44	12484.11	-1.02899	6.42E-76	STIP1
940.9235	874.8165	916.1964	2285.225	2250.034	2416.211	-1.34692	7.83E-76	RPAP3
11803.33	12393.07	12733.67	22958.06	24202.56	24477.36	-0.95594	8.76E-76	HSPA9
597.6257	590.4764	558.4783	1665.943	1589.926	1662.996	-1.49391	9.28E-76	LTV1
750.4022	723.2345	723.6492	1880.669	1883.774	1941.22	-1.37645	1.02E-75	EXOSC9
2494.75	2670.023	2483.038	1055.996	978.0933	963.7339	1.350807	1.45E-75	SMOX
414.2939	387.3763	420.6838	1269.685	1308.672	1269.463	-1.65365	1.52E-75	LAMTOR3
1026.299	1026.399	991.9377	2430.45	2393.81	2397.169	-1.24612	3.35E-75	YWHAH
583.2467	526.0788	613.2311	1663.868	1687.526	1749.743	-1.56526	3.61E-75	TWISTNB
7643.318	7769.322	7751.168	14433.33	15968.53	15679.98	-0.99233	4.35E-75	DDX3X
3196.624	3348.675	3240.452	6384.73	6374.398	6379.051	-0.96787	4.70E-75	CDC42
6596.35	6777.599	6938.089	12993.52	12452.85	13100.86	-0.92431	4.88E-75	TARS
1399.253	1396.932	1467.374	3159.689	3299.491	3157.789	-1.1734	5.15E-75	MRPL42
1831.521	1955.705	2123.495	4536.218	4934.544	4933.979	-1.28514	5.78E-75	SUZ12
2938.701	2686.866	2929.273	5897.188	6049.066	6097.653	-1.07636	6.22E-75	PRELID3B
752.1996	914.4458	753.7632	146.2627	107.0445	98.38337	2.781469	6.80E-75	-
1327.358	1316.683	1360.606	2992.68	2939.527	3037.19	-1.16321	6.88E-75	MAPK6
5225.855	5288.528	4845.62	2315.307	2146.138	2022.677	1.244038	8.67E-75	SHC1
1816.243	1818.984	1719.237	556.0057	416.6342	408.3439	1.954601	9.68E-75	HR
4349.637	4710.932	4747.065	9581.763	9173.298	9253.326	-1.02052	9.87E-75	RPL7L1
549.0967	595.43	600.4554	1627.562	1698.02	1617.507	-1.50261	1.21E-74	ZW10
3581.261	3228.796	3236.801	1303.916	1161.748	1057.886	1.51159	1.58E-74	MIDN
1822.534	1764.494	1924.56	4401.366	4082.385	4073.918	-1.18782	1.59E-74	EIF2A
4106.093	4148.196	4011.553	7940.716	7679.922	8050.51	-0.94854	1.84E-74	PSMA4
2697.853	2777.022	2607.145	1127.571	1146.006	1162.616	1.2338	1.87E-74	MINK1
7302.717	7703.933	7751.168	14199.93	15288.48	15063.23	-0.96916	3.12E-74	SLC38A1
640.7626	681.6237	708.1359	2048.715	1942.544	1811.1	-1.51503	3.13E-74	ORC1
3588.45	3576.543	3583.569	6777.876	6945.302	6781.047	-0.93177	4.34E-74	CAPZA1
603.0178	662.7998	686.2348	1817.392	1825.005	1780.422	-1.47442	5.09E-74	TGS1
837.5747	888.6868	915.2839	2324.643	2640.432	2315.712	-1.46286	5.79E-74	PM20D2
1876.455	1955.705	1699.161	620.3198	526.8271	507.7851	1.740393	6.67E-74	B4GALNT4
617.3968	693.5125	679.8469	1783.16	1932.049	1872.458	-1.48951	6.99E-74	PUS7
1286.019	1243.369	1381.595	3027.949	3227.078	3083.737	-1.25534	7.21E-74	GOLT1B
10157.84	10953.53	9964.092	3700.135	3073.858	2567.489	1.733932	8.86E-74	AGRN
1099.092	1152.222	1169.884	2746.834	2740.131	2600.283	-1.24133	1.17E-73	TNFRSF21
1101.788	1136.37	1169.884	2677.334	3075.957	2961.022	-1.35444	1.34E-73	SMC2
2550.469	2512.497	2577.943	5238.487	5012.204	5321.165	-1.02704	1.69E-73	REEP5
2212.563	2405.498	2334.293	5491.594	5371.118	4913.879	-1.18248	2.04E-73	NUP188
6864.158	7166.957	6916.188	3601.589	3424.376	3585.175	0.9811	2.07E-73	CTSZ
534.7177	462.6719	503.7255	61.20212	58.76956	51.8364	3.127198	2.14E-73	TNS1
23519.49	25678.79	23783.69	12112.83	11638.47	10714.27	1.082327	2.36E-73	PSAP
1978.006	1947.779	2081.518	4410.702	5063.627	4642.002	-1.23242	5.58E-73	GDA
681.2034	686.5774	658.8584	112.031	129.0831	138.583	2.416592	6.02E-73	COL1A1
3699.887	3662.737	3658.398	1582.957	1407.321	1293.794	1.363051	8.34E-73	EPN1
507.7572	443.848	393.3074	23.85846	17.84076	31.73657	4.19596	9.56E-73	ZNF385A
415.1926	424.0334	381.4443	1341.26	1283.485	1243.016	-1.66439	1.57E-72	NDUFAF4
824.9931	781.6877	885.1698	2142.074	2163.979	2115.771	-1.36519	1.71E-72	NUP54
879.8129	1008.565	1009.276	2539.369	2895.45	2622.499	-1.47585	1.71E-72	GNB4
87517.57	88118.69	86536.76	156272.9	155351	147739	-0.80912	1.79E-72	ACTB

2343.771	2262.832	2372.62	4690.78	4905.159	5056.694	-1.06982	1.81E-72	DNM1L
260.6187	239.7572	290.1897	1095.414	1051.555	967.9654	-1.97708	1.83E-72	C1orf109
4580.599	4566.285	4613.834	1835.026	1834.45	2128.466	1.247054	1.94E-72	IFIT2
554.4888	543.912	483.6495	68.46339	51.42336	69.82045	3.059796	3.06E-72	ZNF467
2597.2	2414.414	2258.552	731.3135	576.1515	492.9747	2.013327	3.38E-72	PPDPF
8550.99	9114.736	9154.664	16458.18	17237.32	16920.88	-0.91633	4.10E-72	KPNB1
386.4347	374.4968	369.5812	1179.438	1191.133	1160.501	-1.64298	4.32E-72	MEST
442.1532	509.2363	507.3757	1526.941	1483.931	1441.898	-1.61075	5.55E-72	MAK16
5045.219	4793.162	5130.335	9391.933	9572.091	9538.955	-0.929	5.56E-72	TMEM123
1011.021	954.0751	965.4739	281.1148	251.8695	249.661	1.904673	5.64E-72	ABCD1
1680.541	1807.095	1772.165	3782.084	4126.462	4226.253	-1.20626	8.87E-72	ZNF664
497.8716	429.9778	505.5506	1586.069	1519.613	1436.609	-1.66281	1.04E-71	FAM13B
1097.295	1104.666	1031.177	306.0106	270.7597	234.8506	1.993495	1.09E-71	NECAB3
1091.903	1078.907	1115.131	2531.071	2646.729	2487.089	-1.22183	1.20E-71	ABCG2
465.519	455.7368	498.2502	1368.231	1579.432	1536.05	-1.65884	1.36E-71	RMND5A
3796.047	3740.014	3385.546	1456.403	1284.535	1177.427	1.47866	1.53E-71	OAF
6096.681	6423.907	6333.983	11645	11782.25	11431.51	-0.8867	1.54E-71	HSPA4
3031.265	2944.456	3228.589	6422.074	7035.555	7332.206	-1.17532	1.89E-71	U2SURP
3061.821	2893.929	3296.117	6545.515	7051.297	7192.565	-1.16778	2.26E-71	UBE2K
1943.856	1769.448	2034.978	4294.522	4513.712	4664.218	-1.22835	2.53E-71	RBM25
665.9258	679.6423	724.5617	1831.914	1835.499	1768.785	-1.3928	3.01E-71	RIOK2
1871.063	1869.512	1745.701	671.1487	711.5314	679.1626	1.411826	4.05E-71	PNPLA2
1615.836	1678.3	1746.613	3657.605	3865.148	3620.085	-1.14445	6.27E-71	TMX1
2747.281	2786.93	2942.049	5587.028	5716.389	5860.687	-1.01783	6.95E-71	NUSAP1
2829.96	2745.319	2511.327	874.4642	705.2347	605.1106	1.887714	7.12E-71	MFSD12
1311.182	1362.257	1281.215	2895.172	3097.995	2985.353	-1.1832	7.59E-71	NAE1
5766.863	6085.077	6269.193	11456.21	11627.98	11413.53	-0.92888	7.83E-71	EIF5
718.0495	715.3086	718.1739	156.6359	152.1712	166.0881	2.179823	9.98E-71	SDC2
1445.985	1426.654	1426.31	3071.517	3105.341	3270.983	-1.13592	1.38E-70	UBE2R2
1845.001	1929.946	1891.708	3887.891	4211.468	4043.239	-1.09966	1.54E-70	IREB2
2359.049	2594.728	2630.871	5346.369	5405.75	5325.396	-1.0841	2.27E-70	DDX1
1463.06	1389.007	1536.728	449.1614	510.0358	474.9907	1.613941	2.47E-70	GADD45B
2962.067	2841.42	2780.528	1041.473	805.9825	774.3723	1.710854	2.74E-70	CSNK1E
765.6799	671.7164	743.7252	139.0014	114.3907	86.74663	2.680249	2.81E-70	PER1
493.3782	509.2363	522.889	1412.835	1483.931	1414.393	-1.49887	3.49E-70	POLR3G
1310.283	1293.896	1426.31	3006.165	3123.182	3126.052	-1.19906	4.45E-70	NCBP1
1881.847	1873.475	1821.442	4013.407	3859.9	4256.932	-1.12112	6.56E-70	RTRAF
826.7905	822.3077	801.2156	2042.491	1943.593	2037.488	-1.29771	6.75E-70	MRPS22
562.577	581.5598	606.8432	1842.288	1763.087	1575.192	-1.56506	7.24E-70	PNO1
1031.691	1017.482	1120.607	2488.541	2549.129	2493.437	-1.24818	1.02E-69	NIP7
2423.754	2445.127	2503.114	4766.504	4984.918	4957.252	-0.99651	1.20E-69	TTC37
2112.809	2189.518	2098.856	900.3974	887.8401	860.0611	1.273067	1.21E-69	DHCR7
1348.028	1435.571	1417.184	3737.479	3411.783	3201.162	-1.30123	1.30E-69	JPT2
5116.215	5321.223	5181.437	9530.934	9758.895	10138.78	-0.914	1.42E-69	C1QBP
1059.55	1175.008	1098.706	2701.192	2584.811	2601.341	-1.24321	1.68E-69	PSMC2
2982.736	3110.899	2988.589	1313.252	1213.172	1102.317	1.323315	3.03E-69	SEMA3F
4328.068	4557.368	4169.424	1983.364	1702.218	1792.058	1.252758	3.12E-69	CD99
4714.503	4784.246	4984.327	2432.525	2367.574	2306.191	1.027173	3.90E-69	SERINC1
2406.679	2511.506	2679.236	5358.816	5194.809	5402.622	-1.07054	4.00E-69	STT3A
1951.046	1918.057	1815.967	678.41	637.02	536.348	1.617975	4.99E-69	EPS8L2
1158.405	1240.397	1106.006	312.2346	305.3918	363.9127	1.836157	5.31E-69	H2AFJ
2070.571	2172.676	2143.571	893.1361	885.7412	835.7297	1.288292	6.18E-69	PPP1R9B
612.0047	632.0871	666.1587	1737.518	1636.102	1763.495	-1.42719	6.55E-69	PFDN4
1268.045	1339.47	1169.884	391.0712	334.7766	316.3078	1.857212	6.58E-69	BCL3

2980.04	2955.354	2862.657	5779.97	5611.443	5635.357	-0.95259	6.67E-69	MRPL3
4920.302	5062.641	4995.278	9601.472	9422.019	9027.996	-0.90529	7.94E-69	CAPN2
1286.917	1358.294	1575.055	3544.537	3642.663	3607.39	-1.35478	8.37E-69	WDR36
618.2955	598.4022	706.3108	1751.003	1864.884	1881.979	-1.51487	9.19E-69	ZNF770
2084.95	2181.592	2133.533	4674.183	4344.749	4363.778	-1.06441	1.61E-68	DKC1
893.2932	840.1409	912.5462	2114.067	2117.803	2167.608	-1.27362	1.78E-68	ZWILCH
1676.947	1768.457	1741.138	3553.872	3807.428	3759.726	-1.10062	1.87E-68	KIF3B
737.8206	763.8545	744.6377	1811.168	1958.286	1978.246	-1.35561	1.88E-68	KIDINS220
1434.302	1425.664	1503.876	3120.271	3419.129	3258.288	-1.16669	2.00E-68	PPAT
342.3991	373.506	354.9805	1097.489	1271.941	1228.205	-1.74886	2.04E-68	BLM
425.9768	415.1168	366.8436	34.2317	24.1375	26.44714	3.83093	2.05E-68	TINCR
1224.908	1133.398	1233.762	2738.536	3041.324	3104.894	-1.30604	2.06E-68	ATF1
4811.561	4971.494	5194.213	9427.202	9779.884	9405.662	-0.93389	2.12E-68	G3BP1
1142.229	1232.471	1248.363	2895.172	2706.548	2931.401	-1.23611	3.08E-68	SLIRP
6195.536	6032.568	6316.645	11084.85	11120.04	11394.49	-0.85733	3.34E-68	RBM39
32.35267	54.49027	47.4524	466.7959	414.5353	477.1064	-3.34468	3.43E-68	HSPE1P2
1604.153	1799.17	1847.906	4055.937	3923.917	4001.982	-1.19039	3.98E-68	MAD2L1
2546.874	2579.867	2659.16	6068.346	7396.568	7950.011	-1.45964	4.62E-68	SMC4
828.5878	757.9101	792.0901	2048.715	2034.896	1920.063	-1.33524	5.83E-68	NSMAF
2659.21	2878.077	2940.224	5730.179	5976.654	6188.631	-1.07799	5.89E-68	GMPS
1626.62	1682.263	1730.188	3534.163	3936.511	3859.167	-1.16891	6.04E-68	RFC1
3332.325	3369.48	3526.079	6451.119	6833.01	6694.301	-0.96588	7.89E-68	PDCD6IP
478.1006	487.4402	495.5126	1372.38	1476.585	1345.631	-1.52158	9.12E-68	TTC27
2457.904	2364.878	2460.225	4707.377	4970.225	4910.705	-1.00201	9.25E-68	TMEM33
2374.327	2405.498	2406.384	4624.391	4907.258	4959.368	-1.01186	1.23E-67	BAZ1B
928.3419	958.038	968.2115	2164.895	2284.666	2325.233	-1.24696	1.41E-67	MCUR1
4090.815	4263.121	4239.69	8674.104	9831.307	10515.38	-1.20442	1.63E-67	CENPF
375.6504	408.1817	392.3949	1231.304	1164.897	1177.427	-1.60393	2.54E-67	YEATS4
1579.889	1656.504	1639.846	3385.826	3355.112	3441.302	-1.0624	2.56E-67	GTPBP4
13372.44	14382.46	12825.84	6544.478	5621.938	5715.756	1.182182	3.40E-67	LGALS1
1738.057	1786.29	1872.545	3687.687	3854.653	3840.125	-1.07658	4.37E-67	XPO7
3769.985	3792.523	3872.846	7146.126	7877.219	7597.735	-0.98413	4.37E-67	PDS5A
1785.688	1756.568	2024.028	4133.737	4400.37	4475.914	-1.22456	4.90E-67	KPNA3
1405.544	1349.377	1454.599	3070.479	3333.073	3483.618	-1.23158	5.31E-67	CAMSAP2
1944.755	1853.66	1794.978	719.903	651.7124	597.7054	1.505846	5.62E-67	TCIRG1
4847.508	5182.52	5220.677	9823.46	10932.19	10255.14	-1.02396	5.67E-67	MTDH
13944	14460.73	13238.31	7121.23	6297.788	6795.858	1.042598	6.68E-67	IFITM3
2075.064	1989.39	2180.073	4270.663	4523.157	4644.118	-1.10536	9.17E-67	TAF1D
407.1044	462.6719	436.1971	1260.349	1388.431	1322.357	-1.60531	9.46E-67	SLC25A13
1726.374	1759.54	1761.214	3522.753	3795.884	3773.478	-1.07996	1.03E-66	SETD2
3964.999	4045.159	3964.101	7725.99	7300.018	7423.184	-0.90681	1.37E-66	RANBP1
1368.698	1527.709	1516.652	3495.782	3239.672	3402.16	-1.20024	1.39E-66	PWP1
1941.16	1962.64	2066.005	3983.325	4187.331	4168.07	-1.04735	1.99E-66	DLAT
1552.928	1558.422	1572.317	3193.921	3278.502	3221.262	-1.04939	2.12E-66	MCCC2
1416.328	1438.543	1554.979	3167.988	3276.403	3361.961	-1.15285	2.38E-66	TXLNG
1510.69	1510.867	1661.747	3467.775	3367.705	3575.654	-1.15233	3.10E-66	LSM3
381.9412	409.1724	401.5203	20.74648	27.28586	39.14177	3.775967	5.00E-66	GBP2
2587.315	2589.774	2490.339	1146.243	1105.078	1166.848	1.165481	5.35E-66	TIMP3
598.5244	579.5783	619.6189	1554.949	1553.195	1649.244	-1.40358	5.50E-66	RASAL2
592.2336	594.4393	592.2425	1593.33	1507.019	1673.575	-1.42418	6.86E-66	NOL10
3509.366	3311.027	3563.493	6778.913	7462.684	6972.524	-1.03048	6.99E-66	CD164
11975.88	12592.21	12339.45	23241.25	21925.24	21764.94	-0.85882	7.89E-66	ANXA1
967.884	1034.324	1012.926	2401.405	2349.733	2258.586	-1.21752	8.81E-66	PRMT3
948.113	1038.287	985.5499	2310.121	2377.019	2273.396	-1.22833	1.02E-65	DHX36

1768.613	1921.03	1965.625	3981.25	3982.687	4016.792	-1.08324	1.24E-65	EIF4E
4124.965	4275.009	4479.689	8058.971	8247.677	8138.314	-0.92445	1.30E-65	DHX15
479.8979	485.4588	485.4746	1347.484	1296.079	1364.673	-1.46615	1.30E-65	ERCC6L
718.9482	741.0677	760.151	1921.124	1862.785	1776.19	-1.32457	1.31E-65	ALDH1A3
1451.377	1509.876	1429.96	547.7071	497.4423	538.4638	1.471174	1.41E-65	MELTF
436.761	349.7285	378.7067	1348.521	1415.717	1231.379	-1.77653	2.12E-65	FASTKD3
1318.371	1379.099	1453.686	3110.935	3560.805	3256.172	-1.25799	3.10E-65	RC3H2
1169.19	1179.962	1115.131	355.8022	373.6065	304.6711	1.74389	3.88E-65	MCF2L
636.2692	647.9388	663.4211	1789.384	1613.014	1833.316	-1.42668	4.62E-65	CMSS1
722.543	758.9008	749.2004	1848.512	1772.532	1815.332	-1.28549	1.09E-64	MAP2K4
2170.325	2187.537	2312.392	4385.806	4721.504	4721.344	-1.05175	1.17E-64	LARP4
1666.163	1633.717	1728.363	3943.906	3553.459	3589.406	-1.1406	1.35E-64	PSMA3
414.2939	337.8397	463.5735	2119.253	1690.674	1543.455	-2.13747	1.70E-64	RPSAP47
1175.48	1105.657	1121.519	2482.317	2739.081	2684.914	-1.21603	1.71E-64	OTUD4
2265.586	2111.25	2223.875	4369.209	4747.741	4743.559	-1.06999	1.82E-64	KPNA4
1190.758	1063.056	1245.626	2802.85	3257.512	3062.579	-1.38175	2.16E-64	OSBPL8
1828.825	2055.769	1866.157	728.2015	647.5146	596.6475	1.543301	2.38E-64	MAGED2
789.9444	610.291	610.4934	107.8817	117.5391	95.20971	2.649027	2.70E-64	PGGHG
688.3929	711.3457	717.2613	130.7028	146.9239	173.4933	2.230971	3.24E-64	TRANK1
7500.427	7662.323	7554.058	13994.54	13119.25	13484.87	-0.83772	3.74E-64	KPNA2
2365.34	2540.237	2362.582	1047.697	956.0547	1044.133	1.253542	5.18E-64	ROMO1
615.5994	684.5959	707.2233	2214.687	1983.472	1806.869	-1.58123	6.10E-64	PHF5A
851.9536	833.2058	896.1204	1999.961	2030.698	2035.372	-1.23234	7.13E-64	DIMT1
19897.79	20549.77	19674.5	39804.2	35634.29	37164.58	-0.90531	7.79E-64	RPL6
944.5182	896.6126	857.7934	258.2937	231.9299	207.3456	1.951631	8.20E-64	NR1D1
854.6497	905.5292	972.7743	2155.56	2365.475	2363.317	-1.33286	8.63E-64	KRR1
1433.403	1355.322	1439.998	3030.024	3412.832	3467.749	-1.22842	9.37E-64	SENP6
422.3821	493.3846	425.2465	58.09015	50.3739	55.01006	3.035346	1.02E-63	UNC5B
706.3666	729.1789	825.8543	2031.081	2028.599	1904.194	-1.39887	1.06E-63	MESD
134.8028	137.7118	171.5587	657.6635	789.1912	812.4562	-2.34608	1.57E-63	EIF2S3B
446.6466	430.9685	385.0945	46.67959	51.42336	41.25754	3.179269	1.90E-63	KIFC2
1084.713	1096.741	1138.858	2393.107	2498.756	2447.947	-1.14436	2.47E-63	ATP1B1
2403.084	2473.858	2564.255	4801.773	4842.192	4785.875	-0.95547	2.53E-63	EIF2S1
5983.447	6506.138	6300.219	3266.534	3047.621	3050.942	1.004428	2.74E-63	NCOR2
2390.503	2308.406	2344.331	1075.705	1000.132	1042.017	1.175736	3.53E-63	SPOCK1
1596.964	1809.077	1699.161	3607.813	3752.856	3688.847	-1.11442	4.09E-63	SF3A3
919.355	1101.694	1066.767	2573.601	2987.802	3013.916	-1.47411	4.67E-63	KNL1
1109.876	1162.129	1034.827	2569.452	2470.42	2553.736	-1.1998	6.42E-63	LSM5
507.7572	477.5329	469.0488	80.91128	54.57173	58.18371	2.907831	7.10E-63	MDGA1
2903.652	3010.835	3369.121	6499.873	6755.35	6499.65	-1.08939	7.55E-63	CDK1
2245.815	2420.359	2361.67	4896.17	4628.102	4632.481	-1.0106	7.78E-63	NOP56
1036.184	1004.602	1074.067	2287.3	2520.794	2468.047	-1.22368	9.21E-63	POLA1
773.768	739.0862	845.9303	1990.625	1949.89	1930.641	-1.31506	1.03E-62	PTPRR
1037.981	1094.759	1084.105	2356.8	2593.207	2655.293	-1.24152	1.13E-62	IDE
423.2808	375.4875	477.2617	1370.305	1318.117	1328.704	-1.65321	1.20E-62	RRP15
758.4904	746.0213	683.4971	175.3078	184.7043	170.3196	2.044514	1.34E-62	IFI35
511.3519	550.8471	574.9041	1508.269	1466.091	1436.609	-1.43023	1.59E-62	UTP11
2185.603	2323.267	2372.62	4508.211	4563.036	4674.797	-0.99834	1.64E-62	KIF4A
4183.38	4112.529	4062.656	1851.624	2086.319	1906.31	1.080468	1.82E-62	ITGB4
1488.223	1428.636	1616.119	3391.013	3730.817	3370.424	-1.21047	2.10E-62	GMFB
1093.7	918.4087	933.5348	263.4803	199.3967	197.8246	2.156324	2.38E-62	NPDC1
1440.593	1443.497	1328.667	484.4304	528.926	494.0326	1.482633	2.63E-62	SYNPO
2658.311	2753.245	2859.007	5211.516	5412.046	5395.217	-0.95372	4.38E-62	NUP205
2051.698	2023.075	1938.248	653.5142	482.7499	428.4437	1.941788	4.53E-62	FBRS

1144.026	1126.462	1124.257	350.6156	396.6945	331.1182	1.654349	5.06E-62	SULT1A1
1624.823	1653.532	1774.902	3473.999	3801.131	3661.342	-1.11376	5.97E-62	11-Sep
1507.994	1664.43	1671.785	3400.349	3704.581	3604.216	-1.14476	6.56E-62	PRKAR2A
487.9861	427.0056	523.8015	1373.417	1517.514	1573.076	-1.63226	8.18E-62	XRCC2
368.461	339.8211	374.1439	1048.735	1154.402	1134.053	-1.62348	9.70E-62	BRCC3
2828.163	2863.216	3021.441	5602.588	6286.244	6100.827	-1.04589	1.06E-61	7-Sep
2310.52	2288.591	2233.001	1049.772	996.9835	1022.975	1.154168	1.23E-61	TLE3
865.4339	815.3726	872.3942	246.8831	243.4739	243.3137	1.799357	1.35E-61	LINC00205
934.6327	922.3717	939.9226	2102.656	2073.726	2073.456	-1.15987	1.40E-61	ARMC1
1755.132	1842.762	1784.028	4044.527	3888.236	3601.043	-1.09992	1.46E-61	RRP1B
2954.877	2639.311	2646.384	591.2748	824.8727	874.8715	1.847103	1.49E-61	-
3099.566	3301.12	2973.988	6594.27	6289.392	6092.364	-1.01761	1.56E-61	RPS26
10094.03	10111.41	10249.72	17056.72	17859.65	17207.57	-0.77525	1.69E-61	RHOA
918.4564	927.3253	932.6222	2046.641	2223.798	2273.396	-1.23584	1.79E-61	ZNF644
1459.465	1569.32	1596.956	587.1255	525.7776	487.6853	1.530705	1.97E-61	TTYH3
1241.084	1330.553	1439.085	3007.203	3168.309	3020.264	-1.19716	2.17E-61	WDR3
1151.216	1193.832	1207.299	2489.578	2677.163	2666.93	-1.14099	2.45E-61	VEZT
3978.48	4148.196	4061.743	7249.858	7326.255	7290.948	-0.84336	2.60E-61	LRRC59
235.4555	256.5996	233.6118	913.8826	804.933	898.1449	-1.85151	3.06E-61	SKA3
4575.207	4420.647	4761.666	8290.295	8627.581	8734.962	-0.89875	3.14E-61	TMED2
12758.63	13285.72	13505.68	22758.89	22483.55	22370.05	-0.77364	3.22E-61	HNRNPK
3068.112	3093.066	3088.056	5979.136	5673.361	5662.862	-0.90469	3.66E-61	KARS
1724.577	1858.614	1862.507	3693.911	4087.632	4101.423	-1.12586	3.98E-61	AKAP12
1408.24	1452.413	1319.542	501.0276	517.382	459.1224	1.500059	4.04E-61	SRF
584.1454	556.7915	521.0639	103.7324	70.31358	95.20971	2.625576	4.11E-61	DUSP2
3279.303	3387.313	3325.318	6155.481	6046.967	6020.427	-0.86703	4.38E-61	DIAPH1
852.8523	784.6599	784.7897	1905.564	2137.743	2005.751	-1.31994	4.42E-61	SNRNP48
1334.548	1311.729	1381.595	2827.746	3152.567	3220.204	-1.19148	4.64E-61	NAA25
2814.682	2844.392	2843.494	6024.779	5853.867	5347.612	-1.0187	4.67E-61	GLUD1
521.2375	440.8758	465.3986	7.261269	2.098913	7.4052	6.411905	4.94E-61	WNT6
2432.741	2461.969	2391.784	4728.123	4520.008	4652.581	-0.93196	5.30E-61	ARPC3
780.0588	781.6877	746.4628	206.4275	208.8418	213.6929	1.875712	6.48E-61	ABCA7
1844.102	1896.261	2031.328	3961.541	4025.715	3867.63	-1.03838	7.25E-61	VTA1
686.5956	770.7896	656.1207	140.0388	171.0614	143.8725	2.215376	7.50E-61	BCL6
920.2537	911.4736	932.6222	2268.628	2103.111	2064.993	-1.21935	9.56E-61	MRRF
3054.631	3311.027	3239.539	7394.046	6966.291	6334.619	-1.10757	1.60E-60	PPA1
2529.799	2665.07	2656.422	4896.17	5209.501	5103.24	-0.95403	1.89E-60	MSH6
1497.21	1587.153	1596.956	610.9839	566.7064	512.0167	1.46979	2.01E-60	DAB2IP
1106.282	1129.435	1091.405	393.1458	341.0733	334.2919	1.638278	2.01E-60	FAM102A
1587.977	1481.145	1607.906	3232.302	3629.02	3561.901	-1.15575	2.18E-60	TMED5
622.7889	652.8925	544.7901	125.5162	115.4402	119.5411	2.335725	2.37E-60	GPRC5C
1008.325	1112.592	1127.907	321.5705	286.5016	230.6191	1.953065	2.60E-60	KIF26A
749.5035	642.9852	770.189	1866.146	1961.434	2110.482	-1.45614	2.72E-60	PHF20L1
296.5661	327.9324	313.0034	958.4875	967.5988	1004.991	-1.64535	3.03E-60	MPP7
563.4757	571.6525	660.6835	1658.681	1883.774	1643.954	-1.52978	3.21E-60	MTMR6
944.5182	949.1214	897.9455	261.4057	288.6005	227.4454	1.843972	4.67E-60	ACAP3
528.4269	574.6247	553.003	110.9937	76.61031	97.32548	2.53839	5.23E-60	-
2813.784	2758.198	2683.798	1019.69	809.1308	698.2045	1.707694	5.24E-60	DDIT4
1058.651	969.9268	1103.268	2415.928	2834.582	2672.219	-1.33842	5.47E-60	PAWR
1163.797	1147.268	1242.888	2653.475	2527.091	2582.299	-1.12694	5.65E-60	USP16
45134.67	41157.99	40424.88	5450.101	4313.266	3429.665	3.263751	5.73E-60	FTH1
6785.973	7338.353	6568.508	2902.433	2534.437	2142.218	1.448868	7.39E-60	GRN
932.8353	918.4087	946.3104	2086.059	2418.997	2278.686	-1.27773	7.99E-60	LTN1
2758.964	2888.975	3030.566	5789.306	6765.845	6536.676	-1.13743	8.10E-60	KIF5B

973.2762	955.0658	844.1053	252.0698	243.4739	192.5352	2.009988	8.38E-60	GAA
501.4664	501.3105	549.3528	90.2472	96.54998	98.38337	2.444682	8.62E-60	DHX58
457.4308	554.81	546.6152	1436.694	1573.135	1569.902	-1.55563	8.65E-60	PTPN14
1764.119	1738.735	1783.115	3449.103	3613.278	3450.823	-0.99186	9.85E-60	PUM2
1071.233	1044.232	1096.881	2329.83	2542.833	2676.451	-1.23241	1.03E-59	AQR
956.2011	959.0287	939.9226	310.1599	250.8201	256.0083	1.804767	1.11E-59	FKBP2
483.4927	529.051	604.1056	1550.8	1514.366	1590.002	-1.52582	1.33E-59	CCDC59
1897.125	2026.047	2046.841	4412.777	3955.401	4253.758	-1.0803	1.37E-59	NSA2
1134.141	1130.425	1189.96	2463.645	2455.728	2446.89	-1.0923	1.50E-59	RAD1
390.9281	410.1631	364.1059	44.60494	48.27499	42.31543	3.107012	1.96E-59	NR4A3
874.4208	803.4838	855.9684	256.2191	225.6331	220.0402	1.852032	2.05E-59	IL17RC
612.9034	586.5134	617.7938	133.8148	141.6766	138.583	2.134076	2.90E-59	PLXNA3
2022.941	2090.445	2131.708	3973.989	4239.804	4285.495	-1.00107	2.94E-59	UBP1
10335.78	10927.78	10376.56	20376.16	18883.92	18690.72	-0.87315	4.04E-59	CNBP
4899.632	5058.678	4929.575	2186.679	1835.499	1635.491	1.395686	4.09E-59	FKBP8
706.3666	737.1047	761.0635	1906.602	1709.564	1899.963	-1.32329	5.11E-59	UTP14A
1994.183	1953.724	1849.731	841.2699	828.0211	796.5879	1.233267	5.56E-59	ABCA2
897.7866	873.8258	752.8506	229.2486	211.9902	194.651	1.988699	5.87E-59	SPPL2B
2109.214	1922.02	2130.795	4240.581	4092.88	4297.132	-1.03503	6.24E-59	NOL7
351.3859	332.886	340.3797	17.63451	7.346194	24.33137	4.378299	6.37E-59	RASD1
2351.859	2394.6	2443.799	4447.009	4715.207	4756.254	-0.95287	7.26E-59	WAPL
1669.757	1642.634	1623.42	3747.852	3546.113	3296.372	-1.10142	7.46E-59	AMIGO2
363.9675	388.367	362.2808	1133.795	1053.654	1071.638	-1.54863	8.01E-59	GABPB1
454.7348	455.7368	519.2388	1322.588	1342.255	1296.968	-1.47003	8.31E-59	ZNHIT6
551.7928	462.6719	525.6266	1402.462	1522.761	1423.914	-1.49649	8.37E-59	RNF138
3934.444	4007.512	3892.01	2083.984	1987.67	1938.047	0.97744	8.63E-59	PIEZO1
1505.298	1464.302	1419.009	573.6402	582.4483	527.885	1.381806	9.88E-59	TPCN1
1300.398	1447.46	1322.279	3080.853	3103.242	2860.523	-1.15255	1.07E-58	WDR82
909.4695	973.8897	974.5994	2101.619	2118.852	2167.608	-1.16068	1.62E-58	CHUK
292.9714	254.6182	297.4901	888.9868	1003.28	1013.454	-1.78027	1.83E-58	GPATCH11
476.3032	471.5885	504.6381	1259.312	1472.387	1409.104	-1.51101	2.20E-58	C4orf46
247.1385	247.683	256.4255	928.4051	821.7243	829.3824	-1.77971	2.52E-58	E2F8
2258.396	2368.841	2466.612	4713.601	4947.137	5364.538	-1.08276	2.57E-58	BMS1
600.3218	646.9481	627.8318	96.47114	81.85759	136.4673	2.57501	2.82E-58	CDH3
1012.818	919.3995	1077.717	2406.592	2866.065	2586.53	-1.38405	3.34E-58	SLC4A7
4479.946	4638.608	4642.123	8012.292	8083.962	7982.805	-0.8073	3.46E-58	CALM1
7719.707	7987.283	8092.46	13492.48	13794.05	14276.17	-0.80438	3.57E-58	TPX2
1097.295	1116.555	1116.044	302.8986	382.0021	358.6232	1.674257	3.80E-58	NAV2
824.0944	831.2243	901.5957	1963.655	2247.935	2275.512	-1.34294	4.05E-58	CFAP97
132.1067	106.9991	141.4447	595.4241	607.6352	589.2423	-2.2328	4.17E-58	AC245041.1
1062.246	1050.176	1135.207	2724.013	2488.261	2390.822	-1.22706	4.35E-58	TOR1A
510.4532	611.2818	527.4517	1656.607	1499.673	1510.661	-1.50202	4.64E-58	FASTKD2
281.2885	291.2753	241.8247	911.8079	914.0765	898.1449	-1.74291	4.67E-58	CLCN4
737.8206	712.3364	697.1853	1654.532	1699.07	1720.122	-1.24035	4.98E-58	TMEM9B
35072.09	34587.45	32120.71	15747.62	13260.93	11808.12	1.318202	5.03E-58	FTL
4486.237	4559.349	4258.853	1840.213	1478.684	1321.299	1.51945	5.06E-58	PIM3
1420.821	1421.701	1412.622	2851.604	3203.99	3107.01	-1.10652	5.54E-58	NET1
984.0604	922.3717	957.261	2158.672	2094.715	2299.843	-1.19393	6.36E-58	LRRC8D
2118.201	2205.37	2137.183	952.2636	948.7085	838.9033	1.237336	6.54E-58	PLCB3
856.4471	940.2048	866.0064	251.0324	201.4956	171.3775	2.09255	6.58E-58	PLPPR2
872.6234	865.8999	984.6374	2154.522	2557.525	2385.532	-1.38171	6.91E-58	ACER3
961.5932	852.0297	882.4322	193.9796	255.0179	245.4295	1.957666	7.71E-58	SLC12A8
1726.374	1652.541	1551.329	665.9621	634.9211	590.3002	1.382229	8.00E-58	BCAM
3273.91	3452.702	3661.135	6772.689	7198.221	6658.332	-0.98984	8.60E-58	EPRS

534.7177	545.8934	525.6266	1397.276	1332.81	1354.094	-1.34664	8.92E-58	CENPU
407.1044	479.5144	469.0488	1252.05	1463.992	1470.461	-1.62746	9.00E-58	MOB4
1002.034	976.8619	985.5499	2197.053	2102.061	2278.686	-1.14969	9.71E-58	UCHL5
420.5847	379.4504	369.5812	49.79156	49.32445	45.48908	3.015845	9.73E-58	GPR153
1639.202	1651.551	1801.366	3495.782	3894.532	3990.345	-1.16008	1.09E-57	RANBP2
7667.583	7762.387	7818.696	12932.32	12964.98	12868.12	-0.73763	1.10E-57	TGFBI
327.1214	339.8211	299.3152	17.63451	9.445107	11.63674	4.639131	1.13E-57	CLIC3
1142.229	1065.037	1133.382	2442.898	2388.563	2362.259	-1.10619	1.14E-57	CDCA7
2354.555	2645.255	2712.087	5248.86	5345.931	5271.444	-1.04101	1.18E-57	PSMD1
1853.988	1817.994	1711.937	698.1191	552.014	537.4059	1.590356	1.66E-57	HIP1R
2231.436	2346.054	2281.366	939.8157	732.5205	882.2767	1.424681	1.68E-57	FBLN1
1018.21	999.6488	885.1698	303.936	290.6994	272.9345	1.742197	1.78E-57	SECTM1
918.4564	1026.399	922.5842	263.4803	308.5402	263.4135	1.778779	1.80E-57	SPINK6
937.3287	904.5385	977.337	2094.357	2437.887	2368.606	-1.29113	1.88E-57	SMC5
1467.553	1603.005	1618.857	3187.697	3270.106	3293.198	-1.05639	1.89E-57	TSR1
4936.478	5287.538	5485.315	9871.176	11109.54	10415.94	-0.99903	1.89E-57	MET
1256.362	1335.507	1279.39	458.4973	341.0733	379.781	1.714347	1.90E-57	TRIM41
313.6412	337.8397	303.8779	10.37324	10.49456	13.75251	4.78708	2.06E-57	CHRD
1050.563	1020.454	1072.242	2241.657	2533.388	2542.099	-1.21874	2.37E-57	SMIM15
456.5321	416.1075	439.8473	1259.312	1302.375	1157.327	-1.50198	2.61E-57	DSCC1
626.3836	678.6515	823.1167	2007.222	2129.347	2004.693	-1.52877	2.62E-57	SEH1L
1239.287	1245.35	1362.432	2943.926	2918.538	2691.261	-1.15267	2.71E-57	MSH2
594.9297	624.1613	633.3071	102.6951	131.182	144.9303	2.290499	2.74E-57	MAP2K6
1416.328	1421.701	1531.253	2967.784	3043.423	2996.99	-1.04369	2.97E-57	PRR11
512.2506	474.5607	507.3757	94.3965	91.3027	98.38337	2.395404	3.01E-57	WHRN
1334.548	1330.553	1269.352	511.4008	432.376	449.6014	1.4973	3.60E-57	KCNN4
1553.827	1743.689	1730.188	4131.662	3918.67	3546.033	-1.20603	4.24E-57	FH
800.7286	854.0111	771.1016	238.5846	221.4353	234.8506	1.803267	4.28E-57	SOX12
3380.854	3763.792	3401.06	1613.039	1410.469	1340.341	1.272658	4.80E-57	AKT1
4682.15	4819.912	5021.742	8938.622	9437.761	8683.126	-0.89773	4.90E-57	IL7R
618.2955	587.5042	610.4934	1483.374	1510.168	1450.361	-1.29045	5.76E-57	KCTD12
566.1717	661.8091	668.8964	1627.562	1875.378	1746.569	-1.4691	6.02E-57	HSPA4L
1209.63	1262.193	1249.276	2610.945	2632.037	2510.363	-1.05931	6.61E-57	ATXN10
1088.308	1038.287	1162.584	2698.08	2560.673	2392.937	-1.21771	7.01E-57	DYNC1LI1
1010.122	964.9731	940.8352	2225.06	2280.469	2089.324	-1.17729	7.81E-57	HIGD1A
1105.383	1007.575	948.1355	311.1972	332.6777	297.2659	1.701652	8.22E-57	ULK1
974.1748	946.1492	1022.964	2121.328	2356.029	2274.454	-1.1975	8.50E-57	DEPDC1
3241.558	3127.741	3276.953	1569.471	1623.509	1640.781	0.996994	9.79E-57	BNIP3
5015.563	4823.875	4922.274	8986.339	9810.318	10409.6	-0.98433	1.29E-56	KTN1
2789.519	2657.144	2562.43	1199.147	1071.495	978.5442	1.301418	1.31E-56	PDLIM7
2359.948	2375.776	2503.114	4522.733	4946.088	4773.18	-0.97624	1.34E-56	PAFAH1B2
1616.735	1616.875	1585.093	3122.346	3148.369	3126.052	-0.96356	1.70E-56	FAM136A
902.28	999.6488	899.7706	236.5099	215.1386	288.8028	1.919793	1.82E-56	C1RL
1067.638	1122.5	1135.207	2409.704	2302.507	2393.995	-1.09576	2.74E-56	FAM98A
11556.19	12935.99	12490.02	22552.46	22283.11	21970.17	-0.85323	2.88E-56	XRCC6
2468.688	2366.859	2724.863	5185.583	6025.978	5903.002	-1.17845	2.88E-56	SCAF11
1030.792	1049.185	1024.789	2213.65	2177.622	2336.869	-1.11583	2.93E-56	THUMPD1
2894.665	3035.603	3093.532	5517.527	5896.895	5627.952	-0.91739	3.31E-56	MAGT1
1229.401	1329.563	1243.8	489.617	447.0684	466.5276	1.437917	3.32E-56	OSBPL5
376.5491	407.1909	383.2694	48.75423	47.22554	58.18371	2.920443	3.59E-56	SNPH
1407.341	1474.209	1419.922	3012.389	2851.373	3136.631	-1.06538	3.60E-56	TMCO1
532.0217	502.3012	516.5012	1460.552	1316.018	1324.473	-1.40264	4.30E-56	ORC6
3540.82	3569.608	3346.307	1737.518	1607.767	1512.777	1.105797	5.08E-56	SRSF9
1586.18	1718.92	1627.07	3280.019	3312.084	3512.18	-1.035	5.52E-56	DNAJC7

4305.601	4446.406	4174.899	10738.38	8744.07	9219.474	-1.15087	5.62E-56	RPL34
1155.709	1157.175	1063.116	328.8318	286.5016	211.5771	2.028879	5.87E-56	SCAND1
652.4455	641.9945	627.8318	1487.523	1650.795	1639.723	-1.31352	6.06E-56	ARID2
5095.546	5472.805	5607.597	9713.503	9969.835	10346.12	-0.89259	7.49E-56	SF3B1
2759.862	2605.626	2557.867	1232.341	1169.094	1063.175	1.193357	7.90E-56	ADM
470.9111	513.1993	500.9879	1247.901	1335.958	1301.199	-1.3879	1.26E-55	CWC22
2036.421	2173.666	1968.362	916.9945	809.1308	809.2825	1.284639	1.49E-55	CEP170B
638.9652	643.9759	566.6912	148.3374	107.0445	116.3674	2.314057	1.69E-55	MAP3K10
422.3821	396.2929	355.893	1155.579	1109.275	1132.996	-1.53248	1.70E-55	MTERF3
1942.059	1903.197	2003.951	3687.687	3705.63	3807.331	-0.93709	1.94E-55	CCNB2
1139.533	1145.286	1133.382	363.0634	426.0793	343.8128	1.593077	2.51E-55	TNS2
4319.081	4452.35	4391.172	7588.026	7666.279	8028.294	-0.82286	2.56E-55	EIF2S2
692.8864	738.0955	733.6872	197.0916	202.5451	207.3456	1.834329	2.75E-55	PGPEP1
3435.674	3732.088	3628.284	1411.798	1641.35	1678.865	1.189985	2.99E-55	IDH1
1149.418	1200.767	1249.276	2515.511	2777.911	2587.588	-1.13065	3.08E-55	OGFRL1
549.9954	594.4393	473.6115	93.35917	102.8467	103.6728	2.431523	3.10E-55	PFKFB4
1866.569	1884.373	1856.119	3516.529	3567.102	3514.296	-0.91853	3.35E-55	CLNS1A
2276.37	2157.815	2506.764	5120.232	6219.078	6581.107	-1.36818	4.47E-55	ASPM
479.8979	420.0704	369.5812	57.05283	53.52227	58.18371	2.911477	4.55E-55	TAF10
2119.1	2067.658	2317.867	4228.133	4490.624	4512.94	-1.02421	5.96E-55	RAP1B
768.3759	743.0491	729.1244	1661.793	1752.592	1765.611	-1.20894	6.11E-55	TXNL1
583.2467	659.8276	676.1967	1567.397	1620.361	1669.344	-1.33998	6.44E-55	DDX10
388.232	387.3763	393.3074	1033.175	1085.138	1122.417	-1.47101	7.15E-55	SEMA3A
558.0836	585.5227	534.7521	71.57537	104.9456	115.3095	2.524636	7.26E-55	METTL7A
654.2429	607.3188	680.7595	1536.277	1737.9	1735.99	-1.36639	9.91E-55	ARHGAP18
1994.183	2060.723	1972.925	887.9495	904.6314	793.4143	1.220675	1.02E-54	ITPRIP
612.9034	613.2632	612.3185	163.8972	135.3799	141.7567	2.059191	1.04E-54	PWWP2B
15448.4	15843.79	13332.3	7261.269	6419.524	7007.435	1.108975	1.14E-54	S100A6
1050.563	1029.371	1045.778	2190.829	2265.776	2152.797	-1.08021	1.39E-54	TMEM248
1206.934	1196.804	1298.553	360.9888	443.92	434.791	1.578826	1.59E-54	STARD4
3273.012	3172.324	3135.509	5651.342	6099.44	6041.585	-0.89295	1.99E-54	ZNF207
1001.135	1078.907	1034.827	357.8768	361.013	376.6073	1.50739	2.43E-54	C1S
516.744	535.9861	542.0525	112.031	124.8853	108.9622	2.20491	2.48E-54	PITPNM3
3236.166	3304.092	3391.022	5936.606	6593.734	6495.418	-0.93788	2.48E-54	SMG1
998.4393	1048.195	1086.843	2331.905	2463.074	2214.155	-1.16159	2.59E-54	AASDHPPT
5584.43	6077.151	6166.075	10901.24	11224.99	10614.82	-0.87708	3.36E-54	PARP1
1757.828	1794.216	1846.994	4237.469	3712.977	3677.211	-1.10685	3.36E-54	RSL24D1
997.5407	1016.491	1072.242	2147.261	2264.727	2253.296	-1.11071	3.44E-54	GTF2H1
4139.344	4149.186	3939.462	2124.44	2201.759	2104.135	0.927161	3.61E-54	SLC6A6
275.8964	278.3957	296.5775	867.203	945.5602	854.7716	-1.64831	3.70E-54	SASS6
4497.021	4692.108	4588.282	7987.396	8211.996	7836.817	-0.80301	3.88E-54	NUP98
3230.774	3395.239	3530.641	6215.646	6459.404	6171.705	-0.89194	4.02E-54	PLS3
1563.712	1561.394	1645.321	3162.801	3662.603	3524.875	-1.11734	4.03E-54	PIK3C2A
2872.198	2690.829	2633.608	1314.29	1268.793	1179.543	1.123283	4.20E-54	TGFB1
8552.788	8651.073	8824.322	15100.33	14944.26	14211.64	-0.76582	4.29E-54	PTGES3
3288.289	3087.121	3319.843	5816.276	6134.072	6236.236	-0.90728	4.42E-54	HMGN1
1955.539	1912.113	1862.507	730.2762	824.8727	846.3085	1.25486	4.46E-54	DDX58
1393.861	1300.831	1391.633	2726.088	2871.313	2781.181	-1.03549	4.55E-54	CUL3
652.4455	722.2438	698.0979	1637.935	1628.756	1780.422	-1.2844	4.68E-54	VRK1
277.6938	254.6182	303.8779	853.7178	1001.181	970.0812	-1.75515	5.56E-54	PYROXD1
3146.297	3289.231	3213.075	1609.927	1409.42	1557.208	1.075921	5.63E-54	EDF1
9574.593	10081.69	10367.44	17220.62	18334	18918.17	-0.85945	6.57E-54	ITGB1
744.1114	776.734	837.7174	1883.781	1956.187	1780.422	-1.25277	6.61E-54	PPTC7
1681.44	1595.079	1745.701	3268.608	3333.073	3359.845	-0.98768	6.63E-54	RCN2

1733.564	1772.42	1977.488	3778.972	4347.898	4340.505	-1.18486	7.80E-54	MED13
3546.212	3310.036	3585.394	6335.976	6874.989	6523.981	-0.91818	1.04E-53	CMPK1
1921.389	1991.372	2056.879	3957.392	4049.852	3736.452	-0.97624	1.04E-53	METAP1
997.5407	1096.741	1032.09	352.6902	373.6065	367.0863	1.515404	1.25E-53	C3
1467.553	1412.784	1416.272	2870.276	3312.084	3302.719	-1.14229	1.37E-53	WWTR1
982.263	1007.575	1070.417	2213.65	2178.671	2166.55	-1.09976	1.49E-53	CDC5L
1424.416	1335.507	1570.492	3071.517	3280.601	3165.194	-1.13557	1.61E-53	SPDL1
422.3821	387.3763	365.931	1200.184	1148.105	1063.175	-1.53666	1.79E-53	ISOC1
598.5244	602.3652	646.9953	1461.59	1556.344	1471.519	-1.28047	2.05E-53	SLC35B4
951.7077	945.1585	963.6488	1993.737	2239.54	2207.807	-1.17089	2.13E-53	BCAT1
399.9149	452.7646	491.8624	1274.871	1505.97	1307.547	-1.60469	2.51E-53	FBXO45
2086.747	2243.018	2294.141	4178.342	4300.672	4267.511	-0.94449	2.51E-53	ZFP91
1493.615	1481.145	1546.766	3032.098	3104.292	2896.491	-0.99828	2.53E-53	SNX6
1109.876	1140.333	1164.409	2358.875	2594.256	2386.59	-1.10404	2.64E-53	HIPK1
2306.925	2397.572	2448.362	4360.911	4733.048	4722.402	-0.94982	2.85E-53	CDC27
8273.297	9039.44	8480.292	4610.906	4560.937	4125.754	0.955682	3.39E-53	PLOD1
473.6071	466.6349	438.0222	92.32185	65.06629	63.47314	2.640574	3.77E-53	OPLAH
4865.482	4998.244	5016.267	8348.385	8907.785	8862.966	-0.81176	3.99E-53	PAPOLA
1104.484	1269.128	1151.633	401.4444	429.2276	370.26	1.553026	4.10E-53	DMBT1
626.3836	604.3466	630.5694	1452.254	1454.546	1552.976	-1.2603	4.22E-53	PPIL4
4442.201	4696.071	4560.906	8890.905	8622.333	7989.153	-0.89668	4.23E-53	CCND1
934.6327	1011.538	942.6602	2037.305	2129.347	2107.308	-1.1194	4.24E-53	ZC3H14
742.314	787.6321	716.3488	229.2486	187.8527	192.5352	1.880856	5.12E-53	TNNT1
1205.137	1086.833	1151.633	2370.286	2526.041	2491.321	-1.10065	5.34E-53	PRPF38A
2043.61	2361.906	2159.084	868.2403	824.8727	958.4444	1.307636	5.55E-53	MYO18A
615.5994	567.6895	537.4897	100.6204	138.5282	107.9043	2.310166	5.56E-53	TP53INP2
2655.615	2959.317	2958.475	5433.504	5528.536	5759.13	-0.96393	6.04E-53	NASP
1148.52	1172.036	1219.162	2467.794	2427.393	2378.127	-1.03899	6.16E-53	PTCD3
566.1717	590.4764	646.0827	1454.328	1569.987	1497.966	-1.32677	6.17E-53	ZMYND11
629.0797	659.8276	626.0067	177.3824	166.8636	165.0302	1.910356	6.22E-53	GALNS
829.4865	837.1687	875.1318	278.0029	280.2048	260.2399	1.63482	6.59E-53	SH3BP2
366.6636	414.1261	429.8093	1092.302	1217.369	1164.732	-1.5215	6.99E-53	DDIAS
2439.931	2377.757	2522.278	4436.635	4690.02	4791.164	-0.9229	7.71E-53	GARS
6652.068	6688.433	6937.176	11297.5	12151.66	12130.78	-0.81113	8.16E-53	CRIM1
1095.497	1147.268	1158.021	2304.934	2386.464	2332.638	-1.04659	8.48E-53	HTATSF1
5158.454	5487.666	5706.152	9710.391	9821.862	9683.885	-0.83733	8.53E-53	DNAJA1
1085.612	1174.018	1178.097	2429.413	2389.612	2600.283	-1.11007	9.34E-53	BUB1B
3477.013	3352.638	3731.401	6454.231	7025.061	6916.457	-0.94932	9.59E-53	CBX3
858.2444	748.9935	803.9532	1903.49	2211.205	1946.51	-1.32919	1.08E-52	ARHGAP29
1898.922	1986.418	1993.001	3858.846	3728.718	3637.011	-0.93334	1.26E-52	NARS
944.5182	986.7692	949.0481	347.5036	317.9853	332.1761	1.529318	1.28E-52	GLCCI1
3304.466	3503.229	3385.546	6504.022	6340.815	5982.343	-0.88539	1.37E-52	EFTUD2
599.4231	693.5125	737.3373	1706.398	1750.493	1718.006	-1.35027	1.48E-52	SDAD1
1512.487	1559.412	1471.937	505.1769	622.3276	496.1484	1.484561	1.50E-52	CABIN1
1780.296	1917.067	1815.054	3580.843	3630.07	3466.691	-0.95419	1.52E-52	PRPF4
396.3202	382.4226	416.1211	1327.775	1116.622	1118.185	-1.57576	1.57E-52	TIMM13
649.7495	652.8925	772.0141	1694.988	1955.137	1944.394	-1.43072	1.64E-52	PUS7L
1191.657	1334.516	1249.276	473.0198	472.2554	411.5175	1.475913	1.68E-52	NIPSNAP1
1917.794	1832.855	1860.682	705.3804	587.6956	481.338	1.660717	1.71E-52	CBX6
289.3767	286.3216	269.2011	15.55986	14.69239	12.69463	4.297353	1.91E-52	LINC01843
9444.284	9315.855	9197.553	16796.35	15991.62	15386.95	-0.78505	1.97E-52	H3F3B
2315.013	2394.6	2078.78	969.8981	792.3395	803.9931	1.403078	1.99E-52	IER5
1506.197	1467.274	1460.074	512.4381	514.2336	622.0368	1.427414	2.15E-52	NDUFA1
1770.41	1751.614	1677.26	3899.301	3610.13	3384.176	-1.06719	2.32E-52	ERH

1166.493	1153.212	1089.58	420.1163	334.7766	327.9446	1.654223	2.33E-52	C17orf62
97.05801	114.9249	115.8934	490.6543	500.5907	535.2902	-2.22029	2.83E-52	HSP90AA2P
1569.104	1600.032	1680.91	3482.297	3264.859	3147.21	-1.02862	3.13E-52	ERLIN1
514.9467	489.4217	484.562	1218.856	1407.321	1318.126	-1.4052	3.68E-52	C12orf29
1425.315	1425.664	1363.344	2916.955	2893.351	2711.361	-1.01597	3.85E-52	VAMP3
625.485	687.5681	652.4705	160.7852	154.2701	187.2458	1.968242	3.89E-52	ADPRHL1
4734.274	4766.413	4898.548	8174.114	8491.151	8098.115	-0.7822	3.93E-52	PPP1CC
16322.82	17000.96	16959.67	27318.97	28931.41	29812.28	-0.77532	4.16E-52	HSP90B1
338.8044	273.4421	279.2391	923.2185	1110.325	1014.512	-1.77233	4.29E-52	ESF1
875.3195	881.7516	876.9569	1873.407	1852.29	1950.741	-1.10774	5.29E-52	SLC35F2
567.9691	568.6803	574.9041	149.3747	113.3413	116.3674	2.174128	5.35E-52	MPG
4620.141	4934.837	5140.373	9104.594	9186.941	10099.63	-0.95011	6.48E-52	EIF3A
319.0333	296.2289	272.8513	19.70916	7.346194	10.57886	4.557685	6.94E-52	C6orf223
714.4548	761.873	752.8506	1627.562	1747.345	1721.18	-1.19317	6.95E-52	IL1RAP
3589.349	3612.21	3580.831	6662.733	6627.317	6154.779	-0.85076	7.00E-52	IARS2
930.1393	941.1956	1073.154	2182.53	2323.496	2226.849	-1.19294	7.06E-52	ATF6
330.7162	313.0714	284.7144	32.15705	26.23641	22.2156	3.524689	9.70E-52	MYL9
326.2228	343.7841	354.9805	4.149297	3.148369	10.57886	5.845568	9.95E-52	EGR3
5995.13	6198.021	6333.071	12127.36	13544.28	15519.18	-1.15275	1.03E-51	MKI67
820.4997	884.7238	891.5577	1992.7	1957.236	1852.358	-1.16025	1.05E-51	COA7
476.3032	462.6719	460.8358	90.2472	83.95651	102.6149	2.338584	1.09E-51	RTN4RL1
285.7819	288.3031	319.3912	888.9868	877.3455	978.5442	-1.61884	1.21E-51	TIMM21
777.3628	813.3911	824.0292	1989.588	1911.06	1743.396	-1.22507	1.30E-51	MTREX
2345.569	2305.434	2271.328	1136.907	1148.105	1094.912	1.034246	1.33E-51	FADS1
968.7827	980.8249	1013.839	2019.67	2116.753	2129.524	-1.08018	1.38E-51	USP15
434.065	389.3577	350.4177	53.94086	59.81901	45.48908	2.881652	1.40E-51	ALDOA
679.4061	688.5589	727.2993	1559.098	1617.212	1677.807	-1.21193	1.42E-51	PCF11
902.28	875.8072	876.9569	309.1226	283.3532	304.6711	1.565365	1.67E-51	FZD4
3558.794	3843.05	3741.439	1894.154	1833.4	1975.073	0.966355	1.95E-51	ZNFX1
382.8399	395.3021	438.0222	53.94086	70.31358	44.4312	2.849689	2.05E-51	N4BP3
1128.749	1190.86	1145.246	385.8846	262.3641	268.703	1.917256	2.13E-51	CDKN1A
1397.456	1510.867	1310.416	3400.349	3189.298	2941.98	-1.1764	2.26E-51	SRPRB
845.6628	935.2512	808.5159	239.6219	282.3038	243.3137	1.758254	2.27E-51	SLC12A4
541.0085	546.8842	615.9687	1453.291	1502.821	1374.193	-1.34539	2.53E-51	OTUD6B
1906.111	1879.419	2055.967	3682.501	3790.636	3786.173	-0.94649	2.86E-51	BUB1
1044.272	1082.87	1063.116	2269.665	2151.386	2379.185	-1.09208	2.87E-51	SNX5
2856.022	3149.538	2992.239	1357.857	1478.684	1475.751	1.060979	3.20E-51	AFAP1
1993.284	1980.474	2158.172	3902.413	4412.964	4336.273	-1.04474	3.60E-51	NUP58
192.3186	201.1186	211.7107	727.1642	661.1575	699.2624	-1.7866	3.89E-51	LRRC8C
1660.77	1627.773	1758.477	3324.624	3875.642	3580.943	-1.09481	4.01E-51	EDIL3
3314.351	3433.878	3595.432	6121.25	6686.086	6522.923	-0.9021	4.75E-51	API5
690.1903	772.7711	797.5654	221.9874	204.644	222.156	1.800588	5.20E-51	OBSCN
456.5321	461.6812	419.7713	80.91128	92.35216	77.22565	2.416988	5.69E-51	PTK2B
553.5901	594.4393	537.4897	142.1134	136.4293	125.8884	2.058582	6.36E-51	IDH2
4738.767	5144.872	4977.027	2735.424	2551.228	2551.62	0.922708	8.06E-51	IFITM2
2244.916	2571.941	2608.97	4878.535	5270.37	5231.245	-1.05066	8.45E-51	COL12A1
525.7309	538.9583	513.7635	1236.49	1383.183	1363.615	-1.33565	9.82E-51	SGO2
2752.673	2772.069	2598.019	1193.96	951.8569	893.9134	1.417759	9.99E-51	SLC2A4RG
3411.409	3757.847	3570.793	7134.715	6451.008	6845.578	-0.92801	1.00E-50	ILF2
363.9675	393.3207	333.9919	56.0155	41.97825	44.4312	2.936545	1.10E-50	C1QL1
6134.426	6274.307	6928.051	11770.52	12104.43	11596.54	-0.87527	1.49E-50	XPO1
962.4919	1075.935	982.8123	344.3916	285.4521	251.7768	1.776122	1.69E-50	DNPH1
7772.729	8085.365	7683.639	4177.304	3878.791	3451.881	1.032472	1.74E-50	SCARB1
2555.861	2823.587	2548.742	1110.974	915.1259	806.1089	1.484678	2.14E-50	GIPC1

2180.21	2285.619	2281.366	4041.415	4155.847	4104.596	-0.86665	2.35E-50	CEP55
385.536	386.3855	393.3074	1123.422	1091.435	989.1231	-1.45929	2.49E-50	TP53RK
2013.954	1613.903	1820.53	643.141	711.5314	598.7633	1.480176	2.79E-50	MAPK8IP3
2072.368	2080.538	2270.415	4065.273	4340.551	4089.786	-0.9599	3.34E-50	USP14
650.6481	552.8286	567.6037	141.0761	137.4788	106.8465	2.2001	3.51E-50	AC020916.1
671.3179	743.0491	727.2993	1897.266	1725.306	1630.202	-1.2949	3.97E-50	ZNF639
1269.842	1322.627	1231.025	526.9607	505.838	467.5855	1.34917	4.18E-50	ZER1
838.4734	843.1131	902.5082	1922.162	2060.083	1844.953	-1.17301	4.41E-50	ZDHHC2
898.6853	924.3531	909.8086	1908.676	1918.406	1888.326	-1.06464	4.87E-50	STIL
1105.383	1102.685	1157.109	2247.881	2543.882	2468.047	-1.10908	4.88E-50	MGA
2504.636	2184.564	2275.89	982.346	809.1308	743.6936	1.45801	5.22E-50	IER2
442.1532	537.9676	459.9233	89.20988	99.69835	83.57297	2.401033	6.27E-50	MRPL12
698.2785	745.0306	777.4894	1670.092	1708.515	1867.168	-1.24017	6.99E-50	KIF2A
716.2522	700.4476	655.2082	175.3078	208.8418	196.7667	1.834758	7.41E-50	MARK4
758.4904	776.734	792.0901	1706.398	1668.636	1685.212	-1.12061	7.42E-50	DNAJC9
1073.929	1071.972	1141.595	2246.844	2614.196	2472.279	-1.15729	7.66E-50	LATS1
2040.914	2107.287	2103.419	3950.13	3854.653	3732.221	-0.8841	8.98E-50	LAS1L
681.2034	674.6886	708.1359	1512.419	1656.042	1720.122	-1.24369	9.99E-50	APC
411.5979	417.0982	430.7218	1063.257	1068.347	1108.664	-1.36328	1.03E-49	STRN3
1097.295	1010.547	1080.455	2160.746	2316.15	2240.602	-1.07463	1.06E-49	GLS
7610.067	8474.723	8051.395	15123.15	14491.94	13952.45	-0.85219	1.06E-49	CCT3
469.1137	379.4504	350.4177	46.67959	32.53315	57.12583	3.137345	1.13E-49	MX2
461.0255	427.0056	425.2465	1088.153	1152.303	1177.427	-1.37944	1.16E-49	TICRR
11262.32	11528.16	11053.67	18044.25	18592.17	18065.51	-0.69273	1.20E-49	CD44
977.7696	951.1029	1045.778	2185.642	2051.687	2127.408	-1.09706	1.20E-49	POLR2K
3134.614	3471.526	3225.851	1662.831	1567.888	1662.996	1.006304	1.27E-49	CSNK1D
1123.357	1132.407	1167.147	2282.113	2356.029	2256.47	-1.01021	1.30E-49	PSMD6
15460.98	16562.07	16364.69	26356.33	26578.53	26197.48	-0.70967	1.31E-49	HSPA5
483.4927	557.7822	521.0639	1271.759	1444.052	1356.209	-1.38287	1.70E-49	SYNJ2
2689.765	2872.133	2837.106	1452.254	1332.81	1376.309	1.012958	1.71E-49	TNKS1BP1
416.0913	432.95	469.0488	1129.646	1125.017	1211.279	-1.39469	2.04E-49	NPAT
2039.117	2002.27	1921.822	770.7318	921.4227	863.2347	1.22267	2.14E-49	MOV10
443.9505	436.9129	500.0753	1188.773	1172.243	1254.652	-1.38806	2.22E-49	RBBP8
7659.495	8058.616	7639.837	4523.771	4603.965	4388.11	0.789157	2.24E-49	LOXL2
2369.833	2454.044	2481.213	4357.799	4778.175	4855.695	-0.93762	2.35E-49	ANKRD17
614.7007	579.5783	588.5923	1357.857	1416.766	1506.429	-1.26335	2.47E-49	CCP110
596.727	511.2178	634.2196	1463.664	1717.96	1609.044	-1.45823	2.84E-49	CENPK
14671.04	14937.27	14405.45	28133.27	25775.7	24704.8	-0.83686	2.86E-49	RPL5
3966.797	4083.798	3954.975	6702.151	6986.231	6801.147	-0.77128	2.95E-49	VCL
1623.026	1602.014	1710.112	3302.84	3865.148	3394.755	-1.09767	2.96E-49	DHFR
372.9544	378.4597	387.8321	974.0474	1019.022	1073.754	-1.42859	3.02E-49	TERF1
720.7456	717.2901	638.7824	201.2409	156.369	144.9303	2.046298	3.27E-49	PHLDA3
1206.934	1306.776	1208.211	2468.831	2581.663	2719.824	-1.06232	3.71E-49	UTP6
1428.91	1507.894	1616.119	3130.644	3171.457	2976.89	-1.02722	3.93E-49	PSMD5
259.72	301.1826	268.2886	819.4861	830.12	861.1189	-1.5997	4.18E-49	SRFBP1
952.6064	910.4829	1125.169	2312.196	2672.965	2498.726	-1.32397	4.26E-49	PPP1R12A
24566.46	26432.73	24227.19	13377.33	12439.21	14339.64	0.905593	5.38E-49	TMSB10
3781.668	4015.438	4150.26	7025.796	7779.62	7643.224	-0.90997	5.72E-49	STAG2
381.0426	399.2651	343.1174	62.23945	52.47282	58.18371	2.699312	5.77E-49	KREMEN1
6011.306	6545.767	6498.242	11640.85	11436.98	10821.11	-0.83116	5.91E-49	SRSF3
428.6729	495.3661	450.7978	1273.834	1259.348	1139.343	-1.41861	7.08E-49	ABHD5
613.802	676.6701	652.4705	1478.187	1698.02	1769.843	-1.34842	7.26E-49	PBRM1
276.7951	316.0436	246.3875	7.261269	16.7913	12.69463	4.514599	7.58E-49	LMTK3
1064.043	1078.907	1094.143	2152.448	2405.354	2258.586	-1.07429	8.01E-49	TWSG1

869.9274	800.5116	833.1547	255.1817	227.732	286.687	1.702259	8.88E-49	MNT
1702.11	1724.865	1877.108	3376.49	3469.503	3408.508	-0.95096	9.17E-49	CCNC
3788.857	4320.583	4135.659	7573.504	7937.038	7480.31	-0.90909	9.41E-49	CAND1
781.8562	721.253	762.8886	1701.212	1717.96	1621.739	-1.15304	1.00E-48	VKORC1L1
22509.37	21640.56	22188.56	36181.87	39375.6	41015.29	-0.81329	1.10E-48	PABPC1
549.9954	580.5691	657.9458	1523.829	1438.805	1505.371	-1.32076	1.19E-48	MED21
1596.964	1748.642	1735.663	3208.444	3448.514	3495.254	-0.99877	1.21E-48	MDN1
729.7324	653.8832	682.5846	1546.65	1572.086	1655.591	-1.20764	1.33E-48	POLQ
1197.049	1325.6	1294.903	2518.623	2604.751	2632.02	-1.02294	1.36E-48	ELOA
1065.841	993.7044	1094.143	2178.381	2177.622	2169.724	-1.04858	1.42E-48	FYTTD1
1326.459	1470.247	1391.633	2795.589	3184.051	2920.822	-1.08788	1.45E-48	IPO8
1373.191	1495.015	1503.876	2885.836	2921.686	3167.31	-1.03777	1.49E-48	CTR9
1309.384	1317.674	1229.2	527.998	439.7222	423.1543	1.4708	1.56E-48	CARD10
301.9583	258.5811	303.8779	930.4798	869.9993	842.077	-1.61074	1.70E-48	RPP40
757.5917	797.5394	730.9495	255.1817	213.0396	221.0981	1.729056	1.74E-48	ARL4D
644.3573	641.9945	651.558	157.6733	199.3967	167.1459	1.886461	2.17E-48	MMP2
485.2901	473.57	550.2654	1234.416	1363.244	1413.335	-1.40963	2.22E-48	BRCA2
539.2112	504.2827	587.6798	1344.372	1465.041	1331.878	-1.34343	2.26E-48	MIGA1
354.082	374.4968	427.9842	1038.361	1148.105	1091.738	-1.50287	2.26E-48	ESCO2
743.2127	723.2345	667.9838	1767.6	1683.328	1572.018	-1.23483	2.36E-48	CDCA8
417.8887	450.7831	460.8358	1162.84	1223.666	1091.738	-1.38783	2.81E-48	ATAD5
6759.012	6732.025	6404.249	3817.353	3861.999	3626.432	0.815309	2.82E-48	RHOB
379.2452	417.0982	382.3569	1067.407	1016.923	1024.033	-1.39987	2.88E-48	RNF34
999.338	1049.185	1022.052	2364.062	2065.33	2242.718	-1.11991	2.94E-48	CDC123
2732.902	2881.049	2607.145	1269.685	1242.556	1048.365	1.206913	3.04E-48	MVP
661.4324	629.1149	592.2425	180.4944	154.2701	135.4094	2.001078	3.47E-48	ZNF865
972.3775	962.9917	998.3256	1949.132	2114.655	2105.192	-1.07213	4.64E-48	DNAJB11
371.157	342.7933	353.1554	44.60494	54.57173	27.50503	3.073693	4.78E-48	SLC29A4
504.1624	570.6617	597.7178	1357.857	1388.431	1401.699	-1.31078	6.46E-48	MMGT1
620.0928	704.4106	646.0827	193.9796	164.7646	148.104	1.958036	6.51E-48	MVD
1371.394	1545.542	1571.405	3038.322	3408.634	3549.206	-1.15539	6.82E-48	PCM1
4432.316	4285.907	4354.671	7442.801	7337.799	7162.944	-0.74715	7.03E-48	SRP9
969.6814	1034.324	979.1621	318.4585	267.6114	202.0562	1.919608	7.74E-48	GPSM1
292.0727	358.645	326.6915	8.298593	4.197825	3.173657	5.957912	9.92E-48	METRNL
351.3859	362.608	437.1096	1055.996	1271.941	1255.71	-1.63794	9.97E-48	CEP135
2319.507	2325.248	2400.909	4087.057	4273.386	4270.684	-0.8421	1.01E-47	CFL2
675.8113	717.2901	722.7366	1551.837	1565.789	1691.559	-1.18476	1.01E-47	ORC3
993.9459	1024.417	1016.576	2000.998	2122.001	2074.514	-1.03014	1.09E-47	DCAF1
2026.535	1931.928	1930.035	3525.865	3620.624	3669.805	-0.87706	1.10E-47	USP10
207.5963	210.0352	226.3115	668.0367	749.3118	696.0888	-1.71431	1.13E-47	FAM169A
680.3048	715.3086	655.2082	1536.277	1547.948	1506.429	-1.16303	1.18E-47	GPATCH4
1005.629	1047.204	1017.489	2039.379	2311.952	2241.66	-1.1027	1.21E-47	HAUS6
1064.942	1109.62	1051.253	2193.941	2120.951	2180.302	-1.01003	1.25E-47	MRPS10
459.2282	467.6256	483.6495	81.94861	99.69835	111.078	2.269296	1.30E-47	NREP
628.181	523.1066	661.596	1521.755	1563.69	1621.739	-1.37529	1.36E-47	TBK1
910.3682	975.8712	939.0101	1925.274	1998.165	1945.452	-1.05511	1.38E-47	ELK3
3800.54	4048.132	4003.34	2006.185	1843.895	1627.028	1.113447	1.61E-47	COL4A2
1164.696	1243.369	1070.417	428.4149	361.013	320.5394	1.64728	1.64E-47	MAFF
772.8693	766.8267	827.6794	1685.652	1706.416	1721.18	-1.11068	1.86E-47	CHAMP1
423.2808	590.4764	542.965	81.94861	102.8467	92.03605	2.490955	1.95E-47	RAB3D
4444.897	4719.848	4769.879	7798.603	8123.842	8026.179	-0.78135	2.07E-47	UBQLN1
1793.776	1811.058	1818.705	3295.579	3513.58	3710.005	-0.95568	2.72E-47	PSME4
540.1099	560.7544	499.1628	1337.111	1307.623	1248.305	-1.28341	2.79E-47	TET3
1118.863	1215.628	1200.911	2500.988	2354.98	2683.856	-1.09292	3.21E-47	DPM1

412.4965	458.709	447.1476	1428.395	1179.589	1166.848	-1.51833	3.51E-47	EIF5AL1
5047.915	5124.067	5002.578	10597.3	9278.244	9079.833	-0.93224	3.79E-47	DKK1
871.7247	870.8536	855.0558	1763.451	1873.28	1812.158	-1.06879	3.80E-47	PITRM1
8886.2	9293.068	9205.766	16513.16	15513.06	14959.56	-0.7789	4.13E-47	RPS3A
1643.695	1681.273	1750.264	3120.271	3465.305	3374.655	-0.97266	4.23E-47	PKN2
3388.044	3311.027	3495.052	1532.128	1731.603	1766.669	1.019187	4.65E-47	AFDN
803.4246	795.5579	808.5159	1654.532	1713.762	1734.933	-1.08374	5.04E-47	ATG3
1055.955	1181.943	1085.93	409.743	330.5787	298.3238	1.677416	5.10E-47	TMEM161A
1152.115	1235.443	1142.508	442.9374	370.4581	316.3078	1.643118	5.19E-47	YIF1B
677.6087	774.7526	805.7783	1724.033	1737.9	1819.563	-1.2262	6.15E-47	LPGAT1
509.5546	441.8666	540.2274	1243.752	1445.101	1373.136	-1.44411	7.74E-47	THAP5
1119.762	1220.582	1378.857	3113.01	3160.963	2697.608	-1.27042	8.35E-47	SFT2D2
5123.405	5019.049	5200.601	8306.892	8757.713	8515.98	-0.73738	8.64E-47	SSR1
678.5074	690.5403	713.6111	1562.21	1491.277	1522.297	-1.1356	1.14E-46	JKAMP
1185.366	1389.007	1231.937	3171.1	2714.944	2817.15	-1.19381	1.17E-46	NME1
638.0665	587.5042	660.6835	1421.134	1605.668	1672.517	-1.31621	1.17E-46	CDC7
730.6311	773.7618	745.5503	1577.77	1634.004	1658.765	-1.11454	1.23E-46	LEPROTL1
4794.486	4815.949	4834.67	7770.595	8016.797	7863.264	-0.7113	1.26E-46	MAPRE1
1297.702	1299.841	1262.051	511.4008	517.382	573.374	1.268546	1.54E-46	PCDH1
3223.584	3086.131	3185.699	5779.97	6254.76	6932.325	-0.99806	1.60E-46	PNN
902.28	894.6312	776.5768	290.4508	241.375	226.3875	1.762482	1.60E-46	FADS3
322.628	312.0806	340.3797	851.6431	962.3515	949.9813	-1.50255	1.93E-46	GCFC2
3999.149	3752.893	3889.272	2186.679	2107.308	2078.745	0.869372	2.06E-46	FOSL2
309.1477	286.3216	316.6535	832.9713	939.2634	864.2926	-1.53049	2.13E-46	C12orf4
820.4997	763.8545	761.9761	251.0324	259.2157	272.9345	1.583345	2.24E-46	MN1
1138.634	1218.601	1214.599	2450.16	2330.843	2337.927	-0.9953	2.41E-46	SUCLA2
2265.586	2163.759	2309.654	3952.205	4123.314	4122.58	-0.85579	2.42E-46	CLINT1
714.4548	710.355	635.1322	211.6141	166.8636	155.5092	1.947014	2.53E-46	ANKRD9
769.2746	827.2614	883.3447	1767.6	1924.703	1866.11	-1.16449	2.79E-46	GALNT1
1422.619	1421.701	1581.443	2886.873	3191.397	3312.24	-1.08505	2.83E-46	SBNO1
481.6953	416.1075	417.9462	1127.571	1203.726	1116.069	-1.38882	3.10E-46	KLHDC10
763.8825	802.4931	719.999	248.9578	250.8201	214.7508	1.677449	3.13E-46	CARD19
308.2491	272.4513	268.2886	28.00775	28.33532	14.8104	3.575374	3.49E-46	ADSSL1
486.1887	459.6997	515.5886	1198.109	1176.441	1181.658	-1.28225	3.56E-46	WDR76
1246.476	1154.203	1164.409	2382.734	2308.804	2386.59	-0.98911	4.34E-46	RRAS2
370.2583	443.848	397.8702	76.76199	73.46194	74.052	2.433223	4.38E-46	FAM78A
733.3272	717.2901	826.7669	1733.369	1789.323	1670.401	-1.18878	4.40E-46	CARNMT1
1968.121	2135.028	1934.598	881.7255	830.12	688.6836	1.330236	4.86E-46	OGFR
2199.083	2410.451	2318.78	4204.275	4301.722	4139.507	-0.86836	5.21E-46	EIF3L
580.5507	663.7906	558.4783	157.6733	159.5174	161.8565	1.91141	5.39E-46	CHRNA9
812.4115	765.836	816.7289	1684.614	1793.521	1683.096	-1.10726	5.69E-46	SEC24B
1203.34	1320.646	1206.386	483.393	441.8211	520.4798	1.367299	5.96E-46	RAB13
1215.921	1238.415	1203.648	2437.712	2389.612	2299.843	-0.96246	6.20E-46	EIF2B1
798.9312	838.1594	807.6034	1711.585	1757.839	1678.865	-1.07478	6.28E-46	MPP5
292.0727	310.0992	266.4635	31.11972	12.59348	11.63674	3.968195	6.95E-46	TMEM141
904.0774	919.3995	788.4399	288.3761	269.7103	222.156	1.742506	7.21E-46	UBALD1
1504.399	1480.154	1368.819	540.4459	387.2494	359.6811	1.75724	7.27E-46	METRN
660.5337	663.7906	659.7709	1404.537	1496.525	1533.934	-1.16043	9.80E-46	DBR1
1643.695	1548.514	1744.788	3115.084	3241.771	3224.436	-0.95617	9.88E-46	CHRAC1
668.6218	620.1983	678.0218	1432.545	1480.783	1524.413	-1.17328	1.05E-45	ARMC8
2228.739	2351.998	2418.247	4240.581	4357.343	4133.159	-0.86324	1.11E-45	NRAS
1240.186	1279.035	1239.238	2572.564	2365.475	2456.411	-0.97652	1.34E-45	ESD
2180.21	2060.723	2339.769	4128.55	4175.787	4082.381	-0.91215	1.40E-45	PCNP
4460.175	4813.968	4581.895	2547.668	2292.013	2195.113	0.977724	1.40E-45	TNRC18

715.3535	805.4653	707.2233	1610.964	1784.076	1764.553	-1.21224	1.42E-45	BMPR1A
1299.499	1295.878	1314.067	2415.928	2534.437	2567.489	-0.94327	1.43E-45	DARS
515.8454	552.8286	538.4023	1232.341	1246.754	1248.305	-1.21419	1.52E-45	PLK4
425.9768	461.6812	445.3226	1073.63	1096.682	1184.832	-1.33221	1.83E-45	CISD2
1073.03	1103.676	1040.303	2185.642	2695.004	2609.804	-1.21944	1.93E-45	CLOCK
265.1122	277.405	262.8133	28.00775	26.23641	27.50503	3.299995	2.16E-45	SP8
395.4215	408.1817	419.7713	1030.063	1295.029	1132.996	-1.49911	2.26E-45	RBM7
873.5221	762.8638	797.5654	1730.257	1882.725	1953.915	-1.19288	2.59E-45	LIMS1
767.4772	872.835	834.9798	1791.459	2044.341	1842.837	-1.19851	2.72E-45	WDR12
357.6767	423.0426	409.7333	78.83663	51.42336	51.8364	2.707186	2.85E-45	CGA
529.3256	555.8008	576.7292	1298.73	1256.199	1291.678	-1.21093	2.89E-45	CGAS
5705.753	5933.495	6170.638	9806.862	10511.35	10645.5	-0.7979	3.14E-45	SON
464.6203	428.987	442.5849	90.2472	102.8467	103.6728	2.171354	3.19E-45	ENO2
4092.613	3989.678	4300.83	7071.439	7963.275	7905.58	-0.88938	3.88E-45	ENO3
951.7077	995.6858	926.2344	364.1008	366.2603	318.4236	1.453718	4.03E-45	ENO4
6842.59	7273.956	7485.617	11886.7	12741.45	12638.56	-0.78674	4.17E-45	ENO5
2971.952	3163.408	2924.711	5714.619	5885.351	5264.039	-0.89659	4.20E-45	ENO6
362.1702	282.3587	336.7296	933.5917	1055.753	964.7917	-1.58824	4.64E-45	ENO7
1205.137	1258.23	1319.542	2463.645	2637.284	2459.584	-0.99903	4.84E-45	ENO8
1128.749	1073.954	1115.131	2130.664	2272.073	2190.881	-0.99051	5.09E-45	ENO9
1229.401	1076.926	1038.478	321.5705	379.9032	410.4596	1.589387	5.10E-45	ENO10
912.1656	885.7146	851.4056	1780.048	1851.241	1856.589	-1.05058	5.13E-45	ENO11
395.4215	408.1817	427.9842	1095.414	1068.347	991.2389	-1.35721	5.25E-45	ENO12
284.8832	310.0992	305.703	800.8142	957.1042	903.4344	-1.5635	5.81E-45	ENO13
612.0047	581.5598	587.6798	170.1212	156.369	181.9563	1.809002	5.85E-45	ENO14
312.7425	306.1362	310.2657	49.79156	37.78043	39.14177	2.873637	5.90E-45	ENO15
231.8608	290.2845	251.8628	12.44789	22.03858	14.8104	3.972735	6.36E-45	ENO16
354.9807	359.6358	347.6801	916.9945	899.3841	972.1969	-1.3925	6.43E-45	ENO17
490.6822	511.2178	506.4632	1173.214	1408.37	1265.231	-1.35084	6.82E-45	ENO18
754.8956	696.4847	760.151	1587.106	1652.894	1575.192	-1.12194	7.02E-45	ENO19
404.4084	410.1631	390.5698	79.87396	80.80814	87.80451	2.278054	7.28E-45	ENO20
1386.671	1426.654	1283.04	597.4987	549.9151	509.9009	1.305082	7.66E-45	ENO21
1364.204	1502.941	1354.219	584.0135	441.8211	469.7012	1.496505	7.87E-45	ENO22
442.1532	566.6988	560.3034	1378.604	1390.53	1402.756	-1.41172	8.42E-45	ENO23
621.8902	581.5598	602.2805	1431.507	1328.612	1351.978	-1.1869	8.62E-45	ENO24
1037.981	954.0751	1065.854	314.3092	378.8537	380.8388	1.510177	9.16E-45	ENO25
844.7642	744.0399	781.1396	272.8162	259.2157	230.6191	1.635879	9.91E-45	ENO26
686.5956	702.4291	725.4742	1581.919	1491.277	1514.892	-1.11763	1.07E-44	ENO27
3251.443	3223.843	3038.779	1656.607	1374.788	1447.188	1.086872	1.09E-44	ENO28
2660.108	2591.755	2541.441	1311.178	1099.83	1259.942	1.086052	1.10E-44	ENO29
1094.599	1063.056	904.3333	336.093	237.1771	226.3875	1.936403	1.18E-44	ENO30
515.8454	527.0695	589.5049	1343.335	1289.782	1272.636	-1.25839	1.19E-44	ENO31
657.8376	745.0306	752.8506	1705.361	1705.367	1560.381	-1.20593	1.20E-44	ENO32
2038.218	2198.435	2124.408	3753.039	3900.829	4036.892	-0.87826	1.22E-44	ENO33
1668.859	1819.975	1635.283	786.2917	790.2406	774.3723	1.12371	1.36E-44	ENO34
1162	1187.888	1092.318	2218.836	2287.815	2303.017	-0.98451	1.42E-44	ENO35
591.3349	608.3096	521.0639	161.8226	124.8853	120.599	2.077921	1.43E-44	ENO36
1611.343	1636.69	1619.77	716.791	792.3395	698.2045	1.140898	1.47E-44	ENO37
9708.497	9272.262	9536.108	5712.544	5566.316	5927.333	0.72896	1.51E-44	ENO38
298.3635	299.2011	325.779	926.3305	842.7134	831.4981	-1.49367	1.58E-44	ENO39
925.6458	917.418	915.2839	1815.317	1986.621	1891.5	-1.04543	1.61E-44	ENO40
434.9637	414.1261	423.4214	1045.623	1020.072	1048.365	-1.29075	1.72E-44	ENO41
1181.771	1097.731	1098.706	434.6388	355.7657	309.9605	1.61797	1.83E-44	ENO42
416.99	406.2002	429.8093	1023.839	1227.864	1091.738	-1.41559	1.99E-44	ENO43

6239.572	6234.678	6513.755	10064.12	10336.1	10451.91	-0.70023	1.99E-44	EN044
3397.929	3377.406	3261.44	1568.434	1213.172	1134.053	1.357772	2.19E-44	EN045
1893.53	1867.53	1812.317	927.3678	878.395	823.0351	1.083997	2.32E-44	EN046
1508.893	1527.709	1524.865	703.3058	653.8113	723.5938	1.132412	2.35E-44	EN047
625.485	605.3374	624.1816	1329.85	1441.953	1385.83	-1.16404	2.40E-44	EN048
2800.303	2919.688	3101.745	5133.717	5312.348	5287.313	-0.83469	2.49E-44	EN049
3113.046	3050.464	3038.779	1714.697	1638.201	1567.787	0.903084	2.56E-44	EN050
2430.944	2337.137	2358.019	1269.685	1177.49	1162.616	0.981193	2.62E-44	EN051
2457.006	2593.737	2711.175	4550.741	4675.328	4776.354	-0.85123	2.70E-44	EN052
257.9227	259.5718	272.8513	803.9262	758.7569	730.999	-1.53704	2.80E-44	EN053
470.9111	416.1075	430.7218	1201.221	1053.654	1153.095	-1.37015	3.14E-44	EN054
1735.361	1689.198	1809.579	3144.129	3281.65	3410.623	-0.90993	3.20E-44	EN055
825.8918	911.4736	901.5957	1810.131	1834.45	1881.979	-1.06683	3.28E-44	EN056
604.8152	600.3837	599.5429	164.9345	183.6549	135.4094	1.898429	3.40E-44	EN057
2172.122	2201.407	2206.537	4123.363	3859.9	3829.546	-0.84425	3.52E-44	EN058
1621.228	1564.366	1598.781	775.9185	732.5205	702.4361	1.113702	3.62E-44	EN059
863.6366	856.9833	803.9532	295.6374	290.6994	318.4236	1.480466	4.03E-44	EN060
891.4958	840.1409	878.782	1818.429	2166.078	2269.165	-1.26006	4.53E-44	EN061
1227.604	1254.267	1230.112	2487.503	2329.793	2347.448	-0.9489	4.67E-44	EN062
374.7518	334.8675	319.3912	911.8079	940.3129	929.8815	-1.43433	4.70E-44	EN063
235.4555	219.9425	229.0491	676.3353	674.8004	714.0728	-1.5926	5.85E-44	EN064
1716.489	1709.013	1539.465	759.3213	691.5917	641.0787	1.246603	6.30E-44	EN065
6071.518	6355.547	6168.812	10710.37	10409.56	9908.157	-0.7387	6.38E-44	EN066
1301.296	1326.59	1135.207	418.0416	279.1554	254.9504	1.982025	6.94E-44	EN067
1088.308	1017.482	1088.668	2061.163	2234.293	2166.55	-1.01596	6.96E-44	EN068
528.4269	572.6432	531.1019	158.7106	144.825	137.5251	1.887061	7.13E-44	EN069
2606.187	2567.978	2692.924	4464.643	4679.526	4857.811	-0.83159	7.63E-44	EN070
1753.335	1764.494	1836.956	802.8889	878.395	751.0988	1.13844	8.04E-44	EN071
560.7796	593.4486	579.4668	163.8972	176.3087	143.8725	1.840117	9.03E-44	EN072
1042.475	1032.343	1007.451	420.1163	408.2385	427.3858	1.295461	9.25E-44	EN073
1452.275	1536.626	1466.462	692.9325	686.3445	636.8472	1.14363	1.02E-43	EN074
968.7827	1064.046	992.8503	411.8177	368.3592	376.6073	1.386617	1.03E-43	EN075
352.2846	388.367	348.5927	942.9276	955.0053	925.65	-1.37519	1.08E-43	EN076
8604.013	8595.592	8602.573	13225.88	13362.73	13352.63	-0.63038	1.11E-43	EN077
1670.656	1708.022	1639.846	828.822	743.0151	746.8673	1.113689	1.21E-43	EN078
584.1454	490.4124	596.8052	1398.313	1327.562	1403.814	-1.30376	1.69E-43	EN079
2568.443	2824.577	2526.84	1148.318	990.6868	807.1668	1.426271	1.70E-43	EN080
2004.967	1869.512	1962.887	3683.538	3469.503	3509.007	-0.86883	1.70E-43	EN081
1803.661	2001.279	1987.526	4101.58	3959.599	3572.48	-1.00634	1.74E-43	EN082
732.4285	781.6877	785.7023	1605.778	1623.509	1761.38	-1.11792	1.92E-43	EN083
514.9467	502.3012	543.8775	1216.781	1257.249	1172.137	-1.22343	2.04E-43	EN084
337.9057	343.7841	325.779	63.27677	48.27499	41.25754	2.719634	2.05E-43	EN085
1340.838	1333.526	1397.108	2570.489	2521.844	2517.768	-0.90225	2.30E-43	EN086
960.6946	1147.268	1109.656	2231.284	2365.475	2380.243	-1.11721	2.33E-43	EN087
564.3744	532.0232	531.1019	1276.946	1232.062	1213.395	-1.19331	2.54E-43	EN088
1378.583	1377.118	1236.5	577.7895	490.0961	474.9907	1.37118	2.55E-43	EN089
832.1826	843.1131	807.6034	1655.569	1714.812	1766.669	-1.04905	2.93E-43	EN090
1633.81	1657.495	1638.933	596.4614	566.7064	746.8673	1.368245	2.95E-43	EN091
35445.05	37810.3	33912.96	19140.7	16429.24	14702.5	1.092007	3.09E-43	EN092
212.0897	249.6645	275.589	790.441	770.301	752.1567	-1.64986	3.13E-43	EN093
6264.735	6992.588	6675.276	12229.01	11342.52	11396.6	-0.81106	3.29E-43	EN094
4531.171	4678.237	5072.844	8126.397	8419.788	8654.563	-0.8192	3.31E-43	EN095
311.8438	262.544	239.9997	24.89578	19.93967	29.6208	3.452042	3.71E-43	EN096
4598.573	4849.634	4853.833	8111.875	7703.01	8032.526	-0.7377	3.93E-43	EN097

453.8361	468.6163	491.8624	1084.004	1192.182	1258.884	-1.32154	4.32E-43	EN098
1361.508	1434.58	1398.021	2548.705	2686.608	2629.904	-0.90735	4.59E-43	EN099
788.147	773.7618	782.9647	1652.457	1589.926	1598.465	-1.04565	5.16E-43	EN0100
3188.535	3341.74	3422.961	5596.364	6166.605	5952.723	-0.83183	5.20E-43	EN0101
993.0472	924.3531	861.4436	281.1148	286.5016	190.4194	1.873761	5.48E-43	EN0102
5981.649	6486.324	6135.048	10047.52	10390.67	10462.49	-0.73224	5.64E-43	EN0103
771.072	845.0945	755.5883	267.6296	198.3472	185.13	1.864087	5.72E-43	EN0104
4050.375	3884.661	3951.325	6854.638	7183.529	6515.518	-0.79001	6.04E-43	EN0105
167.1555	190.2206	174.2963	604.76	616.0309	561.7373	-1.74668	6.11E-43	EN0106
3759.201	3741.995	3608.208	2077.76	1825.005	1799.464	0.962052	6.18E-43	EN0107
196.8121	224.8962	229.9616	692.9325	647.5146	738.4042	-1.67427	6.20E-43	EN0108
948.113	847.076	997.413	1943.945	2080.022	2007.867	-1.11021	7.93E-43	EN0109
6029.28	6088.049	6790.256	11025.72	12396.18	11540.47	-0.88678	8.00E-43	EN0110
594.9297	656.8554	576.7292	182.569	131.182	126.9463	2.051726	8.14E-43	EN0111
165.3581	198.1464	178.8591	600.6107	598.1901	599.8212	-1.7313	8.33E-43	EN0112
1362.407	1296.868	1303.116	2955.336	2527.091	2666.93	-1.04021	8.35E-43	EN0113
1452.275	1484.117	1463.724	624.4691	677.9488	699.2624	1.13641	8.75E-43	EN0114
691.089	641.9945	706.3108	1431.507	1548.998	1525.471	-1.14305	9.23E-43	EN0115
974.1748	1084.852	1042.128	2162.821	2055.885	2060.761	-1.01839	9.23E-43	EN0116
2578.328	2582.839	2559.692	4826.669	4485.376	4394.457	-0.82809	9.71E-43	EN0117
810.6141	901.5663	874.2193	2176.306	1893.219	1838.605	-1.19227	1.16E-42	EN0118
895.0905	878.7794	951.7857	1816.355	2027.55	1896.789	-1.07433	1.27E-42	EN0119
3721.456	3919.336	3849.12	6243.654	6697.63	6558.891	-0.76322	1.39E-42	EN0120
1605.052	1593.097	1626.157	2937.702	3449.563	3419.087	-1.02328	1.39E-42	EN0121
714.4548	673.6979	571.2539	146.2627	194.1494	162.9144	1.961002	1.44E-42	EN0122
5324.71	5486.675	5305.544	9476.993	8803.889	8746.599	-0.74595	1.54E-42	EN0123
3009.697	2866.188	3103.57	5078.739	5164.375	5163.54	-0.77862	1.58E-42	EN0124
3044.746	3054.427	3027.828	5233.3	5083.567	4986.873	-0.74572	1.63E-42	EN0125
2124.492	2219.24	2245.776	3833.95	4332.156	4290.784	-0.91874	1.69E-42	EN0126
819.601	959.0287	861.4436	280.0775	325.3315	303.6132	1.537787	1.82E-42	EN0127
1600.558	1710.004	1626.157	794.5903	691.5917	665.4101	1.197737	1.93E-42	EN0128
1304.891	1073.954	1090.493	446.0494	389.3483	414.6912	1.472922	2.04E-42	EN0129
625.485	671.7164	636.9573	1403.5	1390.53	1385.83	-1.11229	2.13E-42	EN0130
8833.178	8858.136	8601.661	13975.87	13678.61	13480.64	-0.64573	2.17E-42	EN0131
689.2916	752.9565	724.5617	240.6592	198.3472	165.0302	1.842014	2.24E-42	EN0132
2714.03	2799.809	2721.213	4811.109	4617.608	4597.571	-0.76843	2.25E-42	EN0133
1989.689	1980.474	2063.267	3651.381	3471.602	3553.438	-0.8233	2.32E-42	EN0134
977.7696	1006.584	1012.014	1937.721	2055.885	1939.104	-0.98558	2.52E-42	EN0135
1521.474	1646.597	1692.773	2984.382	3325.727	3238.188	-0.97416	2.54E-42	EN0136
3910.18	3919.336	4121.971	6751.943	7452.19	7839.991	-0.88313	2.56E-42	EN0137
253.4292	269.4792	271.9388	35.26902	28.33532	26.44714	3.140771	2.67E-42	EN0138
227.3674	247.683	218.0985	7.261269	8.395651	8.463085	4.845059	2.83E-42	EN0139
734.2259	817.354	706.3108	1623.412	1691.724	1627.028	-1.13101	2.84E-42	EN0140
969.6814	990.7322	1045.778	1949.132	2011.808	1990.941	-0.98534	2.91E-42	EN0141
260.6187	232.8221	214.4484	6.223945	14.69239	7.4052	4.644596	3.06E-42	EN0142
395.4215	425.0241	459.0107	1127.571	1035.813	1061.059	-1.33368	3.07E-42	EN0143
1402.848	1359.285	1409.884	2806.999	2574.316	2587.588	-0.9335	3.38E-42	EN0144
663.2297	671.7164	761.0635	1510.344	1605.668	1569.902	-1.16038	3.41E-42	EN0145
408.0031	422.0519	402.4329	100.6204	80.80814	88.8624	2.18845	3.63E-42	EN0146
529.3256	481.4958	521.9764	1189.811	1203.726	1156.269	-1.21084	4.10E-42	EN0147
665.9258	631.0964	691.71	213.6888	217.2375	221.0981	1.609244	4.39E-42	EN0148
1549.333	1430.617	1481.063	713.679	644.3662	647.426	1.153534	4.47E-42	EN0149
2187.4	2301.471	2420.985	4104.692	4366.788	4116.233	-0.86533	4.84E-42	EN0150
300.1609	287.3123	360.4558	860.979	931.9172	958.4444	-1.53627	4.90E-42	EN0151

249.8345	297.2197	262.8133	26.97043	35.68152	23.27348	3.235626	5.02E-42	EN0152
248.0371	227.8684	206.2354	8.298593	12.59348	12.69463	4.345739	6.04E-42	EN0153
265.1122	280.3772	249.1251	730.2762	845.8618	759.5619	-1.55626	6.82E-42	EN0154
863.6366	797.5394	914.3713	1772.787	1953.038	1833.316	-1.10934	7.22E-42	EN0155
591.3349	580.5691	607.7558	1287.319	1279.287	1333.994	-1.13183	7.36E-42	EN0156
1247.375	1327.581	1318.629	2436.674	2612.097	2434.195	-0.94274	7.42E-42	EN0157
3727.747	3762.801	3714.976	2070.499	1950.939	1744.453	0.958457	7.81E-42	EN0158
2090.342	1989.39	2156.347	1006.204	1080.94	977.4864	1.025113	8.80E-42	EN0159
230.9621	242.7294	211.7107	695.0072	656.9597	701.3782	-1.58383	8.88E-42	EN0160
2164.034	2151.87	2217.487	3901.376	3980.588	3686.732	-0.82428	9.06E-42	EN0161
584.1454	602.3652	625.0942	1288.357	1339.106	1402.756	-1.15356	1.04E-41	EN0162
387.3334	340.8119	376.8816	914.9199	1024.269	955.2708	-1.38815	1.06E-41	EN0163
454.7348	448.8017	400.6078	1160.766	1032.665	1087.506	-1.33133	1.08E-41	EN0164
1626.62	1577.246	1515.739	765.5452	732.5205	687.6257	1.110459	1.09E-41	EN0165
361.2715	377.469	384.182	61.20212	49.32445	22.2156	3.078503	1.14E-41	EN0166
2604.39	2573.922	2412.772	4319.418	4501.118	4606.034	-0.82275	1.17E-41	EN0167
603.0178	594.4393	575.8167	1280.058	1282.436	1302.257	-1.12397	1.17E-41	EN0168
2475.878	2553.117	2671.023	4387.881	4691.07	4496.014	-0.81801	1.18E-41	EN0169
2984.534	3139.63	3273.303	5606.737	6178.15	6753.542	-0.98017	1.30E-41	EN0170
586.8415	570.6617	534.7521	1263.461	1309.722	1238.784	-1.17165	1.49E-41	EN0171
945.4169	968.9361	901.5957	388.9966	357.8646	349.1023	1.361043	1.65E-41	EN0172
245.3411	208.0538	238.1746	5.186621	14.69239	8.463085	4.611149	1.86E-41	EN0173
511.3519	471.5885	509.2008	1166.99	1116.622	1229.263	-1.23458	2.00E-41	EN0174
627.2823	672.7071	634.2196	1356.82	1469.239	1399.583	-1.12791	2.08E-41	EN0175
1145.824	1167.083	1168.972	2321.531	2798.9	2386.59	-1.1084	2.19E-41	EN0176
4418.836	4465.23	4543.568	7069.364	7207.666	7307.874	-0.6848	2.43E-41	EN0177
212.0897	230.8406	221.7487	11.41057	6.296738	10.57886	4.553618	2.46E-41	EN0178
1115.268	1048.195	1042.128	2036.267	2311.952	2300.901	-1.0523	2.58E-41	EN0179
774.6667	886.7053	784.7897	1772.787	1810.312	1688.386	-1.10857	2.65E-41	EN0180
18619.86	18514.8	17299.14	11108.7	9885.879	9786.5	0.822422	2.66E-41	EN0181
634.4718	709.3642	679.8469	1549.762	1470.288	1427.088	-1.13658	2.81E-41	EN0182
280.3898	264.5255	187.072	11.41057	10.49456	13.75251	4.359891	2.99E-41	EN0183
4711.807	4766.413	4991.628	7750.886	7822.648	8048.394	-0.70704	3.04E-41	EN0184
848.3589	817.354	849.5805	1652.457	1886.923	1797.348	-1.08493	3.09E-41	EN0185
2625.958	2567.978	2497.639	1277.983	1071.495	974.3127	1.210253	3.29E-41	EN0186
578.7533	634.0686	688.0598	1477.15	1443.002	1412.277	-1.1887	3.37E-41	EN0187
742.314	709.3642	686.2348	234.4353	246.6222	257.0662	1.534519	3.68E-41	EN0188
884.3063	892.6497	947.223	1782.123	2063.231	2031.14	-1.10896	3.74E-41	EN0189
272.3016	255.6089	344.9425	877.5762	996.9835	878.0451	-1.65581	3.90E-41	EN0190
1117.066	1064.046	1074.067	2126.514	2029.649	2110.482	-0.94474	4.24E-41	EN0191
601.2205	533.0139	569.4288	123.4416	122.7864	63.47314	2.45901	4.34E-41	EN0192
181.5344	200.1279	219.9236	662.8501	645.4157	609.3421	-1.67275	4.37E-41	EN0193
279.4911	267.4977	292.0148	757.2466	751.4107	836.7876	-1.48245	4.45E-41	EN0194
371.157	396.2929	323.9539	73.65001	55.62119	46.54697	2.632465	4.45E-41	EN0195
1286.917	1344.424	1266.614	605.7973	545.7173	580.7792	1.169745	4.53E-41	EN0196
764.7812	758.9008	753.7632	1553.912	1536.404	1674.633	-1.06498	4.54E-41	EN0197
704.5693	661.8091	722.7366	247.9205	195.1989	213.6929	1.669324	4.61E-41	EN0198
452.0387	521.1251	489.1248	123.4416	133.281	106.8465	2.007184	4.63E-41	EN0199
901.3813	993.7044	962.7363	1974.028	1853.34	1960.262	-1.01854	4.68E-41	EN0200
7335.968	7623.684	7395.275	11641.89	11745.52	11493.93	-0.64194	5.05E-41	EN0201
1454.971	1539.598	1531.253	3037.285	3016.138	2707.129	-0.95307	5.23E-41	EN0202
471.8098	479.5144	520.1513	1163.878	1168.045	1103.375	-1.22303	5.35E-41	EN0203
1674.251	1641.643	1570.492	777.9931	623.3771	690.7993	1.223598	5.45E-41	EN0204
523.0348	528.0603	556.6532	1185.661	1341.205	1220.8	-1.22081	5.83E-41	EN0205

1849.494	2040.908	1847.906	936.7037	828.0211	777.546	1.174041	5.86E-41	EN0206
1858.481	2003.26	2030.415	981.3086	968.6482	880.1609	1.057687	6.18E-41	EN0207
3817.615	3921.318	3748.74	7104.633	6409.03	6484.839	-0.79993	6.25E-41	EN0208
435.8624	415.1168	384.182	101.6578	85.00596	86.74663	2.175169	6.42E-41	EN0209
1843.204	1864.558	1814.142	885.8748	886.7906	744.7515	1.132999	6.60E-41	EN0210
594.9297	584.532	612.3185	1295.618	1351.7	1267.347	-1.12727	6.68E-41	EN0211
1137.736	1102.685	1088.668	429.4522	459.6619	496.1484	1.265247	7.07E-41	EN0212
286.6806	283.3494	287.4521	46.67959	39.87934	30.67868	2.869584	7.12E-41	EN0213
257.024	243.7201	315.741	771.7692	919.3238	908.7238	-1.66977	7.30E-41	EN0214
693.785	828.2521	819.4665	1683.577	1765.186	1804.753	-1.16648	8.03E-41	EN0215
726.1377	822.3077	717.2613	261.4057	187.8527	211.5771	1.776772	8.08E-41	EN0216
875.3195	841.1316	875.1318	1691.876	1720.059	1748.685	-0.99339	8.91E-41	EN0217
1549.333	1687.217	1831.48	3240.601	3508.333	3318.587	-0.99027	9.71E-41	EN0218
852.8523	858.9648	740.9875	299.7867	283.3532	277.166	1.511131	1.01E-40	EN0219
1336.345	1356.312	1361.519	2425.264	2669.817	2716.65	-0.94621	1.04E-40	EN0220
973.2762	933.2697	1032.09	1916.975	2232.194	2141.161	-1.09758	1.04E-40	EN0221
1886.34	1804.123	1935.511	3269.646	3496.789	3373.597	-0.84961	1.08E-40	EN0222
761.1864	732.1511	698.0979	182.569	219.3364	256.0083	1.736474	1.13E-40	EN0223
233.6582	274.4328	280.1517	810.1502	719.9271	795.53	-1.56175	1.32E-40	EN0224
1020.906	1045.222	1105.093	2000.998	2095.764	2154.913	-0.97911	1.39E-40	EN0225
1220.415	1093.768	1147.983	2277.964	2701.301	2834.076	-1.17384	1.55E-40	EN0226
5980.751	6062.29	6534.743	10200.01	11209.24	11488.64	-0.82436	1.71E-40	EN0227
1139.533	1101.694	1010.189	423.2282	330.5787	294.0922	1.633091	1.73E-40	EN0228
998.4393	1019.463	970.0366	1933.572	1903.714	1924.294	-0.94754	1.74E-40	EN0229
2643.033	2916.716	2934.749	4890.983	5293.458	5351.844	-0.87119	1.77E-40	EN0230
3516.555	3618.154	3383.721	1928.386	1870.131	1664.054	0.94507	1.94E-40	EN0231
1170.088	1119.527	1226.462	2194.978	2273.122	2347.448	-0.95446	1.95E-40	EN0232
459.2282	436.9129	420.6838	110.9937	80.80814	66.6468	2.348077	1.97E-40	EN0233
674.9126	743.0491	693.5351	1476.112	1695.921	1539.224	-1.15839	2.13E-40	EN0234
368.461	384.4041	356.8056	85.06058	66.11575	57.12583	2.412174	2.23E-40	EN0235
3990.163	4140.27	4036.192	6432.447	6763.746	6836.057	-0.71948	2.23E-40	EN0236
1598.761	1698.115	1521.215	613.0586	659.0586	758.504	1.246534	2.26E-40	EN0237
1224.009	1142.314	1171.709	531.11	522.6293	486.6274	1.199781	2.28E-40	EN0238
701.8732	770.7896	786.6148	1760.339	1604.619	1575.192	-1.12909	3.01E-40	EN0239
16392.92	16021.13	16115.57	24628.15	25058.92	26588.9	-0.65234	3.78E-40	EN0240
2734.699	2809.716	2826.156	1548.725	1589.926	1543.455	0.838116	3.91E-40	EN0241
788.147	819.3355	810.341	1587.106	1623.509	1640.781	-1.00488	4.21E-40	EN0242
219.2792	168.4245	219.9236	663.8874	643.3167	696.0888	-1.71862	4.25E-40	EN0243
2001.372	2018.121	1832.393	3758.225	3549.261	3479.386	-0.88251	4.39E-40	EN0244
1206.036	1188.879	1145.246	538.3712	525.7776	488.7432	1.188697	4.50E-40	EN0245
1341.737	1366.22	1502.051	2729.2	2606.85	2692.319	-0.93116	4.83E-40	EN0246
3384.449	3471.526	3370.033	5485.37	5552.674	5543.321	-0.69742	5.39E-40	EN0247
2508.231	2532.311	2502.202	4171.08	4312.216	4159.606	-0.74521	5.39E-40	EN0248
904.0774	859.9555	856.8809	1698.1	1855.439	1945.452	-1.06882	5.73E-40	EN0249
571.5638	663.7906	503.7255	158.7106	108.094	128.0042	2.138034	5.82E-40	EN0250
2412.071	2392.618	2463.875	1351.633	1236.26	1282.157	0.909309	5.86E-40	EN0251
1759.626	1746.661	1769.427	3453.252	3143.122	3112.3	-0.87992	6.48E-40	EN0252
989.4525	1144.296	1055.816	2645.177	2395.909	2152.797	-1.17402	7.14E-40	EN0253
25739.25	26461.47	25321.33	45258.45	41520.69	40491.63	-0.71525	7.18E-40	EN0254
1416.328	1494.024	1550.416	2680.446	2764.268	2846.77	-0.8944	7.37E-40	EN0255
1064.942	1025.408	1137.033	2047.678	2189.166	2123.177	-0.97826	7.56E-40	EN0256
4005.44	3724.162	3752.39	2060.126	2093.665	2231.081	0.846823	7.76E-40	EN0257
772.8693	708.3735	830.4171	1682.54	1706.416	1601.639	-1.10958	8.48E-40	EN0258
2276.37	2588.783	2567.905	4525.845	4676.377	4432.541	-0.87554	8.79E-40	EN0259

1034.387	1000.639	1097.793	2070.499	2148.237	1983.536	-0.98502	8.86E-40	EN0260
2368.036	2431.257	2265.852	1038.361	828.0211	686.5678	1.468235	9.57E-40	EN0261
920.2537	1042.25	907.0709	2178.381	2021.253	1931.699	-1.09615	9.58E-40	EN0262
590.4362	626.1427	675.2842	1344.372	1552.146	1469.403	-1.20646	1.01E-39	EN0263
341.5004	281.3679	300.2277	840.2326	935.0656	821.9772	-1.49119	1.01E-39	EN0264
1454.073	1455.386	1489.275	2675.259	2965.764	2674.335	-0.91864	1.07E-39	EN0265
1770.41	1812.049	1810.492	3106.786	3104.292	3248.767	-0.8108	1.09E-39	EN0266
715.3535	655.8647	668.8964	234.4353	243.4739	215.8087	1.55639	1.23E-39	EN0267
8901.478	8679.805	9421.127	14289.14	15667.33	15073.81	-0.73774	1.29E-39	EN0268
2814.682	2606.616	2770.49	4814.221	4567.234	4698.07	-0.78109	1.33E-39	EN0269
317.2359	320.0065	295.665	57.05283	38.82988	28.56291	2.904291	1.49E-39	EN0270
704.5693	636.0501	564.8661	154.5613	92.35216	70.87834	2.582864	1.51E-39	EN0271
1769.511	1732.791	1782.203	3097.45	3022.434	3082.679	-0.80014	1.57E-39	EN0272
1311.182	1261.202	1477.412	2686.67	3218.683	3365.134	-1.19446	1.57E-39	EN0273
1587.977	1425.664	1479.237	692.9325	639.1189	559.6215	1.24799	1.62E-39	EN0274
525.7309	581.5598	547.5277	169.0838	164.7646	170.3196	1.714277	1.65E-39	EN0275
1904.314	1940.844	1961.062	3424.207	3398.14	3780.883	-0.86882	1.91E-39	EN0276
1362.407	1442.506	1346.918	2525.884	2517.646	2537.868	-0.86908	1.99E-39	EN0277
1578.99	1777.374	1770.34	3853.659	3445.365	3240.304	-1.03999	2.11E-39	EN0278
735.1246	710.355	745.5503	269.7043	251.8695	210.5192	1.581273	2.37E-39	EN0279
2802.999	2871.142	2865.395	1413.873	1520.662	1611.16	0.909728	2.82E-39	EN0280
576.0573	574.6247	657.0333	1296.655	1422.013	1461.998	-1.20913	3.16E-39	EN0281
265.1122	278.3957	247.3	30.0824	36.73097	38.08388	2.914692	3.27E-39	EN0282
940.9235	979.8341	845.9303	372.3994	317.9853	330.0603	1.438446	3.31E-39	EN0283
2309.621	2441.164	2188.286	1197.072	1076.742	998.6441	1.084014	3.61E-39	EN0284
984.9591	1012.528	1093.23	2000.998	2300.408	2113.656	-1.05342	3.64E-39	EN0285
794.4378	793.5765	887.9075	1653.495	1797.719	1743.396	-1.06873	3.71E-39	EN0286
2541.482	2700.736	2498.552	1156.616	872.0982	765.9092	1.469499	4.00E-39	EN0287
469.1137	484.468	479.0868	1036.287	1154.402	1130.88	-1.21329	4.05E-39	EN0288
587.7402	591.4671	614.1436	1283.17	1397.876	1261	-1.13621	4.05E-39	EN0289
426.8755	478.5236	395.1325	108.919	98.6489	101.557	2.072014	4.26E-39	EN0290
618.2955	589.4856	559.3908	1276.946	1268.793	1285.331	-1.11621	4.43E-39	EN0291
1987.892	2091.436	2115.282	3609.888	3656.306	3499.486	-0.7975	4.60E-39	EN0292
4008.136	4060.02	4253.378	6764.391	7032.407	6587.454	-0.72625	4.62E-39	EN0293
920.2537	921.3809	863.2687	378.6233	338.9744	318.4236	1.384136	4.66E-39	EN0294
758.4904	751.9657	756.5008	268.667	294.8972	239.0822	1.497797	4.88E-39	EN0295
680.3048	700.4476	686.2348	215.7634	245.5728	253.8926	1.531231	4.93E-39	EN0296
736.9219	778.7155	751.9381	1518.643	1509.118	1564.613	-1.01844	5.22E-39	EN0297
624.5863	664.7813	628.7443	215.7634	216.188	225.3296	1.544879	6.28E-39	EN0298
17071.43	17862.9	17958	26670.64	27492.61	28529.06	-0.64471	6.56E-39	EN0299
226.4687	236.785	231.7867	26.97043	13.64293	22.2156	3.466512	7.49E-39	EN0300
258.8214	240.7479	241.8247	30.0824	25.18695	33.85234	3.056906	7.69E-39	EN0301
1065.841	1125.472	1105.093	2052.864	2204.908	2060.761	-0.93895	8.20E-39	EN0302
608.4099	614.254	521.0639	173.2331	181.5559	158.6829	1.763395	8.22E-39	EN0303
1921.389	2111.25	2020.377	987.5326	886.7906	769.0829	1.194862	8.87E-39	EN0304
11010.69	12415.86	11480.74	25360.5	23272.74	20244.76	-0.98059	9.26E-39	EN0305
713.5561	719.2716	677.1093	1413.873	1529.058	1458.824	-1.06106	1.10E-38	EN0306
807.9181	841.1316	817.6414	1582.957	1785.125	1799.464	-1.06705	1.11E-38	EN0307
596.727	694.5033	575.8167	180.4944	201.4956	161.8565	1.778689	1.21E-38	EN0308
3802.337	3929.244	4061.743	6384.73	7167.787	7045.519	-0.80453	1.30E-38	EN0309
1239.287	1203.74	1276.652	2247.881	2437.887	2550.562	-0.9598	1.32E-38	EN0310
1374.09	1485.108	1487.45	707.4551	608.6847	612.5158	1.171953	1.40E-38	EN0311
916.659	985.7785	991.0252	1840.213	1972.978	1885.152	-0.978	1.64E-38	EN0312
498.7703	443.848	448.9727	112.031	64.01684	56.06794	2.582614	1.66E-38	EN0313

2263.788	2242.027	2349.807	3822.539	4224.062	4029.487	-0.81667	1.74E-38	EN0314
831.2839	833.2058	793.0027	317.4212	331.6282	286.687	1.392775	1.86E-38	EN0315
1002.933	1108.629	950.8732	2030.043	2036.995	2132.698	-1.0182	1.92E-38	EN0316
3679.218	3770.727	3945.85	6103.615	6584.289	6486.955	-0.75069	1.97E-38	EN0317
981.3643	1009.556	1053.078	2088.133	1975.077	1905.252	-0.97146	2.10E-38	EN0318
3788.857	3776.671	3792.542	2269.665	2237.441	2100.961	0.781367	2.15E-38	EN0319
407.1044	430.9685	369.5812	88.17255	48.27499	78.28354	2.490875	2.17E-38	EN0320
603.9165	614.254	612.3185	1392.089	1243.606	1331.878	-1.11616	2.22E-38	EN0321
566.1717	553.8193	584.9421	1187.736	1288.732	1238.784	-1.12343	2.26E-38	EN0322
2390.503	2370.822	2514.065	4297.634	4494.822	4054.876	-0.82029	2.32E-38	EN0323
1064.942	1100.703	1065.854	457.4599	404.0407	336.4076	1.431197	2.38E-38	EN0324
363.9675	318.025	333.0794	72.61269	62.96738	50.77851	2.445017	2.42E-38	EN0325
2560.354	2568.969	2796.954	4901.357	4642.795	4498.13	-0.82499	2.43E-38	EN0326
2235.03	2268.777	2193.761	1155.579	999.0824	904.4923	1.130244	2.58E-38	EN0327
1200.644	1095.75	1252.926	2227.135	2413.75	2458.526	-0.99957	2.60E-38	EN0328
3370.07	3536.914	3168.36	601.648	461.7608	357.5654	2.825705	2.76E-38	EN0329
1056.854	1030.361	978.2495	453.3106	398.7934	425.27	1.262794	2.90E-38	EN0330
603.0178	631.0964	587.6798	213.6888	165.8141	168.2038	1.733175	2.92E-38	EN0331
6661.954	6797.413	6306.607	4023.78	4076.088	3932.161	0.71605	3.10E-38	EN0332
1671.555	1603.005	1782.203	3015.501	3069.66	3034.016	-0.85036	3.16E-38	EN0333
901.3813	815.3726	887.9075	1717.809	2076.874	2033.256	-1.16133	3.16E-38	EN0334
272.3016	281.3679	231.7867	35.26902	27.28586	37.026	2.97925	3.37E-38	EN0335
1915.098	1986.418	1893.533	3602.627	3572.349	3263.577	-0.84925	3.39E-38	EN0336
774.6667	795.5579	746.4628	1526.941	1830.252	1768.785	-1.14592	3.46E-38	EN0337
269.6056	282.3587	254.6004	33.19437	38.82988	44.4312	2.792508	4.10E-38	EN0338
226.4687	228.8591	221.7487	21.78381	17.84076	27.50503	3.334908	4.32E-38	EN0339
1096.396	1120.518	1173.534	2055.976	2219.6	2204.634	-0.93449	4.51E-38	EN0340
580.5507	617.2261	595.8927	1228.192	1373.738	1422.856	-1.16627	4.78E-38	EN0341
446.6466	383.4134	410.6458	88.17255	80.80814	45.48908	2.531515	4.82E-38	EN0342
233.6582	240.7479	238.1746	25.9331	25.18695	32.79446	3.086646	5.03E-38	EN0343
2519.015	2600.672	2671.935	4331.866	4913.555	4671.623	-0.83686	5.31E-38	EN0344
271.403	308.1177	299.3152	828.822	748.2624	774.3723	-1.42081	5.35E-38	EN0345
507.7572	495.3661	487.2997	152.4866	110.1929	103.6728	2.023653	5.39E-38	EN0346
688.3929	635.0593	657.0333	236.5099	237.1771	213.6929	1.526806	5.41E-38	EN0347
1720.982	1840.78	1993.001	876.5389	844.8124	723.5938	1.18373	5.72E-38	EN0348
934.6327	1005.593	1051.253	1906.602	2112.556	2017.388	-1.01299	5.98E-38	EN0349
202.2042	223.9055	198.9351	588.1628	634.9211	627.3262	-1.56694	7.07E-38	EN0350
3582.16	3517.099	3552.542	5686.611	5662.866	5590.926	-0.66932	7.16E-38	EN0351
434.9637	407.1909	495.5126	1053.921	1126.067	1082.217	-1.28522	7.39E-38	EN0352
2501.94	2515.469	2564.255	1431.507	1359.046	1431.319	0.844639	8.52E-38	EN0353
507.7572	523.1066	533.8395	1122.385	1126.067	1195.411	-1.13818	9.00E-38	EN0354
2072.368	1903.197	2042.278	3494.745	3769.647	3515.354	-0.84066	9.56E-38	EN0355
570.6652	626.1427	527.4517	188.793	160.5668	160.7986	1.756111	1.04E-37	EN0356
1496.311	1518.792	1571.405	2715.715	3207.139	3009.685	-0.96161	1.09E-37	EN0357
669.5205	624.1613	595.8927	219.9127	189.9516	165.0302	1.716304	1.11E-37	EN0358
827.6891	878.7794	836.8049	310.1599	220.3858	195.7088	1.80737	1.13E-37	EN0359
1033.488	1032.343	1082.28	1937.721	1983.472	1981.42	-0.90672	1.14E-37	EN0360
744.1114	788.6228	771.1016	291.4881	288.6005	234.8506	1.498766	1.14E-37	EN0361
4537.462	4565.294	4116.496	2257.217	1874.329	1654.533	1.191812	1.15E-37	EN0362
1505.298	1479.163	1337.793	676.3353	651.7124	579.7213	1.179637	1.15E-37	EN0363
8760.384	8699.619	9098.998	13435.42	13763.62	13457.36	-0.61424	1.16E-37	EN0364
2679.88	2556.089	2763.19	1151.43	1377.936	1373.136	1.035722	1.19E-37	EN0365
817.8036	915.4365	946.3104	2160.746	2099.962	1787.827	-1.17494	1.27E-37	EN0366
1519.677	1575.264	1589.656	735.4628	581.3988	542.6954	1.332612	1.29E-37	EN0367

1025.4	1014.51	965.4739	1888.967	2158.732	2260.702	-1.0697	1.38E-37	EN0368
906.7734	946.1492	892.4702	1794.571	1744.196	1748.685	-0.94593	1.40E-37	EN0369
2016.65	1821.956	1865.244	868.2403	903.5819	990.181	1.046587	1.40E-37	EN0370
568.8678	568.6803	634.2196	1239.602	1329.661	1320.241	-1.13403	1.60E-37	EN0371
277.6938	280.3772	294.7524	731.3135	725.1743	765.9092	-1.38163	1.63E-37	EN0372
1059.55	1115.564	1139.77	2070.499	2053.786	2062.877	-0.90048	1.67E-37	EN0373
1170.987	1199.777	1069.504	521.774	476.4532	450.6593	1.247148	1.76E-37	EN0374
3295.479	3298.147	3758.778	5964.614	6431.068	6255.278	-0.84915	1.80E-37	EN0375
1179.974	1248.323	1206.386	2180.455	2223.798	2310.422	-0.88575	1.81E-37	EN0376
431.3689	424.0334	400.6078	104.7697	116.4897	102.6149	1.95528	1.85E-37	EN0377
1795.573	1923.011	1979.313	3354.706	3922.868	3932.161	-0.9763	2.06E-37	EN0378
870.826	915.4365	843.1927	343.3543	296.9961	242.2558	1.574249	2.15E-37	EN0379
990.3512	946.1492	834.0672	252.0698	329.5293	338.5234	1.590622	2.18E-37	EN0380
295.6675	268.4884	249.1251	37.34367	37.78043	45.48908	2.754052	2.68E-37	EN0381
529.3256	483.4773	517.4137	1134.833	1101.929	1141.459	-1.14184	2.95E-37	EN0382
522.1361	571.6525	550.2654	1160.766	1207.924	1194.353	-1.11642	3.15E-37	EN0383
2270.079	2479.803	2326.993	1295.618	1179.589	1141.459	0.968094	3.33E-37	EN0384
1256.362	1163.12	1231.025	509.3262	378.8537	495.0905	1.400135	3.62E-37	EN0385
595.8283	625.152	601.368	181.5317	112.2918	98.38337	2.215004	3.65E-37	EN0386
237.2529	252.6367	301.1403	713.679	819.6254	786.0091	-1.55154	3.83E-37	EN0387
1267.146	1221.573	1356.956	2378.584	2671.916	2440.542	-0.9616	3.94E-37	EN0388
1806.357	1788.272	1690.948	787.329	644.3662	530.0007	1.429622	4.20E-37	EN0389
1762.322	1729.818	1804.104	3071.517	2975.209	3119.705	-0.79124	4.26E-37	EN0390
1017.312	1025.408	1123.344	2002.036	2226.946	2061.819	-0.99036	4.31E-37	EN0391
1428.91	1524.737	1392.546	627.5811	537.3216	431.6174	1.444314	4.52E-37	EN0392
773.768	867.8814	748.2879	306.0106	238.2266	232.7348	1.620172	4.67E-37	EN0393
384.6373	456.7275	392.3949	97.50847	105.9951	90.97817	2.065949	4.85E-37	EN0394
1923.186	2014.159	1996.651	3349.52	3318.381	3400.045	-0.76285	5.00E-37	EN0395
506.8585	519.1437	440.7598	144.1881	133.281	132.2357	1.839448	5.51E-37	EN0396
3386.246	3416.045	3604.558	5746.776	5543.228	5900.886	-0.72405	5.92E-37	EN0397
1020.906	934.2604	905.2458	368.2501	410.3374	348.0444	1.344333	6.09E-37	EN0398
567.0704	619.2076	560.3034	1587.106	1334.908	1286.389	-1.26943	6.18E-37	EN0399
239.949	210.0352	189.8096	8.298593	19.93967	13.75251	3.931248	6.20E-37	EN0400
1091.004	1153.212	1205.474	2149.336	2178.671	2133.755	-0.90557	6.23E-37	EN0401
910.3682	1036.306	947.223	1849.549	1936.247	1990.941	-0.9979	6.50E-37	EN0402
428.6729	399.2651	465.3986	985.4579	1140.759	1223.974	-1.37233	6.64E-37	EN0403
1789.282	1952.733	2127.145	3900.339	4294.375	3658.169	-1.01408	6.93E-37	EN0404
925.6458	975.8712	857.7934	377.586	285.4521	294.0922	1.526883	7.39E-37	EN0405
1057.753	1016.491	957.261	441.9001	354.7162	349.1023	1.403452	7.61E-37	EN0406
727.0364	704.4106	718.1739	1397.276	1564.739	1483.156	-1.04791	7.83E-37	EN0407
505.9598	510.2271	546.6152	165.9719	112.2918	118.4832	1.977219	8.19E-37	EN0408
640.7626	690.5403	673.4591	1422.171	1348.551	1362.557	-1.04433	9.03E-37	EN0409
2072.368	2141.963	1972.012	1117.198	991.7362	980.66	1.001367	9.03E-37	EN0410
527.5283	499.329	433.4595	141.0761	125.9348	131.1778	1.874526	9.31E-37	EN0411
1547.536	1428.636	1342.355	650.4022	513.1842	613.5737	1.280955	1.00E-36	EN0412
740.5167	766.8267	801.2156	1718.846	1663.388	1491.619	-1.07818	1.01E-36	EN0413
2001.372	2067.658	1953.761	3338.109	3493.64	3573.538	-0.78897	1.03E-36	EN0414
495.1756	509.2363	511.9384	1274.871	1194.281	1067.407	-1.22197	1.08E-36	EN0415
1492.716	1577.246	1507.526	772.8065	719.9271	773.3144	1.01415	1.08E-36	EN0416
2417.463	2643.273	2713.912	4409.665	4537.849	4540.445	-0.79497	1.09E-36	EN0417
377.4478	367.5616	316.6535	1054.959	868.9499	938.3446	-1.43119	1.20E-36	EN0418
796.2352	797.5394	793.9152	1571.546	1883.774	1659.823	-1.09914	1.22E-36	EN0419
77.28693	96.10102	110.4181	407.6684	382.0021	435.8489	-2.11119	1.43E-36	EN0420
2515.42	2383.702	2403.647	1175.288	1353.799	1286.389	0.936782	1.57E-36	EN0421

496.0743	476.5422	503.7255	1053.921	1081.989	1100.201	-1.1318	1.65E-36	EN0422
633.5731	721.253	671.634	1424.246	1500.723	1378.425	-1.0873	1.73E-36	EN0423
1505.298	1411.793	1428.135	719.903	634.9211	587.1265	1.161832	1.81E-36	EN0424
983.1617	996.6766	961.8237	1895.191	2035.945	1812.158	-0.9654	1.84E-36	EN0425
985.8578	1086.833	1153.458	424.2656	458.6124	462.296	1.262006	2.01E-36	EN0426
3338.616	3147.556	3016.878	1699.137	1533.256	1349.862	1.052231	2.11E-36	EN0427
236.3542	258.5811	281.0642	799.7769	789.1912	667.5259	-1.54026	2.15E-36	EN0428
1252.767	1357.303	1268.439	2667.998	2416.898	2365.432	-0.94225	2.26E-36	EN0429
1622.127	1630.745	1709.199	2863.015	2851.373	2865.812	-0.78999	2.32E-36	EN0430
358.5754	378.4597	303.8779	68.46339	73.46194	71.93623	2.282711	2.46E-36	EN0431
794.4378	808.4375	738.2499	1515.531	1577.333	1609.044	-1.00632	2.49E-36	EN0432
1305.79	1432.599	1412.622	633.805	500.5907	463.3539	1.376935	2.51E-36	EN0433
639.8639	669.735	694.4477	1357.857	1372.689	1359.383	-1.02927	2.60E-36	EN0434
2054.395	2083.51	2086.081	3480.222	3955.401	4059.107	-0.88503	2.64E-36	EN0435
362.1702	378.4597	379.6192	98.54579	87.10488	83.57297	2.056415	2.69E-36	EN0436
1598.761	1625.792	1527.602	665.9621	590.8439	435.8489	1.488906	2.85E-36	EN0437
771.072	754.9379	703.5731	287.3388	291.7489	265.5293	1.400223	2.99E-36	EN0438
15272.26	15430.65	14989.48	8314.153	7192.974	5997.154	1.087283	3.23E-36	EN0439
1654.48	1593.097	1678.172	2852.641	3028.731	2821.381	-0.82091	3.89E-36	EN0440
354.9807	388.367	334.9045	71.57537	86.05542	55.01006	2.341316	4.21E-36	EN0441
378.3465	358.645	340.3797	89.20988	79.75868	80.39931	2.110976	4.23E-36	EN0442
824.9931	876.798	848.668	369.2874	329.5293	332.1761	1.306298	4.32E-36	EN0443
819.601	785.6506	855.9684	1643.121	1576.283	1617.507	-0.97431	4.45E-36	EN0444
599.4231	446.8202	600.4554	105.8071	118.5886	153.3934	2.125317	4.88E-36	EN0445
1134.141	1077.917	1095.055	1992.7	2085.27	2019.504	-0.88238	4.96E-36	EN0446
1526.866	1647.588	1607.906	2955.336	2912.241	2730.403	-0.8466	5.01E-36	EN0447
204.0016	208.0538	198.0225	20.74648	14.69239	8.463085	3.793668	5.22E-36	EN0448
5674.299	5729.404	5626.76	3562.171	3380.299	3159.905	0.753334	5.52E-36	EN0449
665.9258	665.772	733.6872	1365.119	1495.475	1495.85	-1.07642	5.76E-36	EN0450
629.0797	670.7257	656.1207	1399.35	1289.782	1466.23	-1.08744	6.53E-36	EN0451
2726.611	2697.764	2747.677	4594.309	5443.53	5631.125	-0.93906	6.97E-36	EN0452
575.1586	661.8091	589.5049	1303.916	1386.332	1283.215	-1.1223	7.32E-36	EN0453
764.7812	761.873	811.2536	1494.784	1607.767	1551.918	-0.99318	7.61E-36	EN0454
443.0518	426.0148	459.9233	119.2923	109.1435	72.99411	2.139762	7.99E-36	EN0455
2508.231	2574.913	2693.836	4201.163	4445.497	4472.741	-0.75439	9.17E-36	EN0456
1122.458	1206.712	1106.919	2393.107	2138.792	2149.624	-0.95989	1.03E-35	EN0457
481.6953	455.7368	493.6875	1049.772	1140.759	1306.489	-1.28841	1.06E-35	EN0458
397.2189	372.5153	410.6458	918.0319	1079.891	933.0552	-1.3115	1.09E-35	EN0459
4213.935	4149.186	4290.792	6485.351	6988.33	6945.019	-0.69022	1.09E-35	EN0460
3133.716	3099.01	3161.06	5822.5	5409.947	5064.099	-0.79479	1.11E-35	EN0461
377.4478	430.9685	370.4938	104.7697	81.85759	90.97817	2.085347	1.18E-35	EN0462
2500.142	2465.932	2452.012	1274.871	1389.48	1412.277	0.863797	1.28E-35	EN0463
5414.579	5869.097	5568.357	9263.305	8945.566	8771.988	-0.67919	1.37E-35	EN0464
923.8485	913.4551	856.8809	393.1458	371.5075	342.755	1.282375	1.43E-35	EN0465
467.3163	481.4958	528.3643	1063.257	1122.918	1204.932	-1.19869	1.44E-35	EN0466
1044.272	1093.768	981.8997	478.2064	429.2276	430.5595	1.220987	1.44E-35	EN0467
721.6443	779.7062	772.0141	311.1972	284.4027	263.4135	1.403564	1.54E-35	EN0468
782.7549	846.0853	812.1661	1650.383	1548.998	1597.407	-0.97508	1.58E-35	EN0469
483.4927	452.7646	472.6989	150.412	125.9348	113.1938	1.854451	1.61E-35	EN0470
558.0836	536.9768	560.3034	133.8148	189.9516	159.7407	1.776046	1.66E-35	EN0471
301.9583	311.0899	283.8019	987.5326	753.5097	826.2087	-1.51782	1.70E-35	EN0472
443.0518	441.8666	509.2008	1026.951	1107.176	1177.427	-1.24765	2.04E-35	EN0473
659.635	593.4486	567.6037	209.5395	173.1603	203.114	1.636094	2.05E-35	EN0474
1677.845	1604.986	1593.306	788.3663	581.3988	607.2264	1.302226	2.33E-35	EN0475

954.4038	914.4458	908.896	1767.6	1750.493	1961.32	-0.97989	2.44E-35	EN0476
1289.613	1353.34	1353.306	2423.189	2523.943	2313.596	-0.86166	2.47E-35	EN0477
1055.057	1110.611	1162.584	2053.902	2032.797	2131.64	-0.90183	2.66E-35	EN0478
697.3798	666.7628	609.5809	237.5472	196.2483	166.0881	1.717586	2.68E-35	EN0479
924.7472	1024.417	905.2458	401.4444	405.0901	355.4496	1.295983	2.96E-35	EN0480
4740.565	4788.209	4879.385	7795.491	7772.274	8769.872	-0.75628	3.24E-35	EN0481
880.7116	859.9555	899.7706	1650.383	1653.943	1692.617	-0.92002	3.89E-35	EN0482
612.0047	642.9852	644.2576	1340.223	1244.655	1338.225	-1.04681	4.04E-35	EN0483
2625.958	2918.697	2997.714	5541.386	5664.965	4875.795	-0.91291	4.09E-35	EN0484
3091.477	3072.26	3067.068	5378.526	4932.445	4964.657	-0.72673	4.10E-35	EN0485
251.6319	274.4328	279.2391	698.1191	716.7787	681.2784	-1.38064	4.17E-35	EN0486
1009.224	1028.38	1064.941	1874.445	1970.879	2099.903	-0.9382	4.20E-35	EN0487
494.2769	586.5134	568.5163	1446.03	1432.508	1179.543	-1.29971	4.38E-35	EN0488
1206.036	1182.934	1223.724	2165.933	2116.753	2165.492	-0.83566	4.63E-35	EN0489
424.1795	449.7924	438.9347	106.8444	128.0337	130.1199	1.847005	4.88E-35	EN0490
626.3836	717.2901	656.1207	1495.821	1514.366	1332.936	-1.11972	4.89E-35	EN0491
365.7649	324.9602	330.3417	77.79931	77.65977	77.22565	2.13401	5.40E-35	EN0492
236.3542	275.4235	278.3266	715.7537	704.1852	689.7415	-1.4178	5.76E-35	EN0493
1678.744	1797.188	1680.91	3451.177	3094.847	3014.974	-0.89101	5.84E-35	EN0494
141.0936	147.6191	153.3078	453.3106	493.2445	479.2222	-1.68966	6.07E-35	EN0495
531.123	554.81	541.1399	155.5986	174.2098	192.5352	1.639439	6.49E-35	EN0496
910.3682	879.7702	897.0329	405.5937	362.0624	376.6073	1.231668	6.51E-35	EN0497
11707.17	12359.38	13286.67	20251.68	20194.69	19749.67	-0.68844	6.86E-35	EN0498
6073.315	6782.552	6605.01	10301.67	10559.63	10614.82	-0.69382	7.44E-35	EN0499
11431.28	12214.74	12487.28	18931.17	21736.34	21817.83	-0.7902	7.50E-35	EN0500
274.9977	255.6089	296.5775	723.0149	694.7401	779.6617	-1.40854	7.55E-35	EN0501
2521.711	2914.734	2692.011	1387.94	1148.105	1048.365	1.180879	7.72E-35	EN0502
508.6559	593.4486	489.1248	172.1958	147.9733	140.6988	1.786627	7.84E-35	EN0503
218.3805	191.2113	177.9465	12.44789	18.89021	11.63674	3.773381	8.05E-35	EN0504
2444.424	2546.182	2454.749	1406.612	1318.117	1198.584	0.924016	8.16E-35	EN0505
1380.381	1442.506	1275.74	614.0959	619.1792	678.1047	1.100364	8.48E-35	EN0506
721.6443	719.2716	684.4097	288.3761	259.2157	236.9664	1.437322	8.53E-35	EN0507
1413.632	1443.497	1562.279	2567.377	2748.526	2816.092	-0.87962	8.58E-35	EN0508
2454.309	2491.691	2060.529	1179.438	1057.852	1034.612	1.098297	8.79E-35	EN0509
2065.179	2116.204	2253.077	3582.918	4038.308	3888.788	-0.83893	9.23E-35	EN0510
1270.741	1367.21	1312.241	2586.049	2518.695	2293.496	-0.90563	9.27E-35	EN0511
847.4602	845.0945	860.5311	1736.481	1602.52	1603.755	-0.95308	9.57E-35	EN0512
494.2769	453.7553	493.6875	1023.839	1085.138	1060.001	-1.13548	9.76E-35	EN0513
803.4246	811.4097	830.4171	1594.367	1560.542	1528.645	-0.93763	1.02E-34	EN0514
577.8546	596.4208	549.3528	204.3529	160.5668	142.8146	1.762474	1.03E-34	EN0515
4605.762	4814.958	4523.492	2851.604	2647.778	2841.481	0.74127	1.06E-34	EN0516
808.8168	814.3819	770.189	1508.269	1663.388	1764.553	-1.04441	1.08E-34	EN0517
1046.07	1008.565	1092.318	1907.639	2073.726	2202.518	-0.97419	1.14E-34	EN0518
418.7873	396.2929	422.5089	1034.212	908.8292	947.8656	-1.22358	1.14E-34	EN0519
1418.125	1482.135	1401.671	2483.354	2597.404	2484.973	-0.81477	1.18E-34	EN0520
570.6652	590.4764	626.9193	1318.439	1377.936	1196.469	-1.12247	1.24E-34	EN0521
930.1393	877.7887	954.5233	1718.846	1832.351	1979.304	-1.00099	1.45E-34	EN0522
3913.774	4257.176	3917.561	2401.405	2098.913	2155.971	0.860607	1.62E-34	EN0523
761.1864	776.734	709.961	1662.831	1453.497	1532.876	-1.04878	1.75E-34	EN0524
780.0588	701.4384	690.7975	273.8536	196.2483	189.3615	1.719473	1.78E-34	EN0525
580.5507	580.5691	631.482	1194.997	1383.183	1373.136	-1.13997	1.85E-34	EN0526
7586.701	8225.059	7651.7	4988.492	4650.141	4598.629	0.720597	1.93E-34	EN0527
1018.21	993.7044	949.9606	434.6388	414.5353	459.1224	1.178888	1.98E-34	EN0528
1003.831	1107.639	1095.055	2011.372	2073.726	1949.683	-0.9127	2.04E-34	EN0529

310.0464	319.0158	295.665	25.9331	61.91792	44.4312	2.806407	2.36E-34	EN0530
2013.954	1879.419	1855.206	1012.428	1034.764	935.1709	0.94681	2.54E-34	EN0531
771.9707	858.9648	840.4551	319.4958	328.4798	253.8926	1.453773	2.81E-34	EN0532
959.7959	1085.842	976.4245	436.7135	447.0684	435.8489	1.194881	2.86E-34	EN0533
266.0108	209.0445	215.3609	24.89578	31.48369	27.50503	3.042019	2.89E-34	EN0534
888.7997	1018.473	905.2458	2127.552	1922.604	1815.332	-1.06114	2.90E-34	EN0535
3646.865	3612.21	3589.044	2122.365	2093.665	2261.76	0.74393	3.02E-34	EN0536
810.6141	832.215	875.1318	358.9142	354.7162	352.2759	1.240159	3.12E-34	EN0537
967.884	1006.584	959.0861	1757.227	1842.845	1869.284	-0.89897	3.20E-34	EN0538
713.5561	709.3642	768.3639	1409.724	1454.546	1506.429	-0.99579	3.25E-34	EN0539
899.584	922.3717	938.0975	1674.241	1797.719	1732.817	-0.91517	3.33E-34	EN0540
1012.818	991.7229	1083.192	1859.922	2088.418	2091.44	-0.96762	3.34E-34	EN0541
1495.412	1531.672	1596.043	2628.579	2657.223	2748.387	-0.79725	3.52E-34	EN0542
4256.173	4237.362	4351.933	6471.865	6782.636	6797.973	-0.64246	3.87E-34	EN0543
1288.715	1205.721	1360.606	2403.48	2297.26	2374.953	-0.87567	4.00E-34	EN0544
2307.824	2140.972	2485.776	4001.997	4150.6	4079.207	-0.81839	4.03E-34	EN0545
337.007	346.7563	326.6915	797.7023	821.7243	780.7196	-1.24852	4.17E-34	EN0546
111.437	111.9527	119.5436	385.8846	404.0407	449.6014	-1.8534	4.24E-34	EN0547
408.0031	394.3114	449.8853	924.2558	967.5988	997.5862	-1.20567	4.50E-34	EN0548
273.2003	271.4606	267.376	43.56761	49.32445	55.01006	2.457503	4.85E-34	EN0549
179.7371	174.3689	206.2354	16.59719	8.395651	8.463085	4.063556	5.48E-34	EN0550
206.6976	166.443	184.3343	522.8114	605.5363	627.3262	-1.65325	6.05E-34	EN0551
6157.792	6389.232	6548.432	11834.83	11040.28	10038.28	-0.78549	6.08E-34	EN0552
722.543	740.0769	738.2499	1442.918	1453.497	1384.772	-0.96009	6.64E-34	EN0553
947.2143	962.0009	959.9986	1775.899	1736.85	1746.569	-0.87431	7.28E-34	EN0554
2012.156	2132.056	2101.594	3394.125	3490.492	3479.386	-0.73081	7.41E-34	EN0555
890.5971	980.8249	935.3599	1760.339	2153.484	1968.725	-1.06786	7.99E-34	EN0556
3273.91	3296.166	3188.436	1978.177	1739.999	1779.364	0.827741	8.68E-34	EN0557
1242.882	1234.452	1326.842	2321.531	2414.799	2208.865	-0.86828	9.17E-34	EN0558
564.3744	582.5505	629.6569	1196.035	1321.266	1251.479	-1.08488	9.26E-34	EN0559
3478.811	3293.194	3318.931	1525.904	1734.751	1909.484	0.964989	1.00E-33	EN0560
434.9637	469.6071	428.8967	978.1967	987.5384	969.0233	-1.1388	1.04E-33	EN0561
12047.77	12450.53	12867.81	18358.56	19337.28	19161.48	-0.60562	1.04E-33	EN0562
251.6319	242.7294	231.7867	39.41832	14.69239	19.04194	3.308795	1.09E-33	EN0563
337.9057	310.0992	324.8665	790.441	752.4602	806.1089	-1.27105	1.12E-33	EN0564
552.6914	593.4486	573.9916	176.3451	150.0723	209.4614	1.682581	1.17E-33	EN0565
2732.902	2805.754	2827.981	5027.91	4721.504	4447.351	-0.76293	1.17E-33	EN0566
560.7796	524.0973	500.0753	1153.504	1146.006	1092.796	-1.09766	1.28E-33	EN0567
937.3287	976.8619	1090.493	1875.482	2033.846	2068.166	-0.99218	1.29E-33	EN0568
487.0874	499.329	481.8244	167.0092	144.825	123.7726	1.752291	1.30E-33	EN0569
1427.112	1525.728	1374.295	702.2684	641.2178	546.9269	1.19422	1.34E-33	EN0570
288.478	280.3772	251.8628	738.5748	750.3613	666.468	-1.39327	1.41E-33	EN0571
450.2413	455.7368	541.1399	1072.593	1149.155	1108.664	-1.20206	1.45E-33	EN0572
724.3403	694.5033	648.8204	1656.607	1565.789	1353.036	-1.146	1.57E-33	EN0573
1145.824	1001.63	1000.151	464.7212	421.8815	465.4697	1.2194	1.69E-33	EN0574
894.1919	995.6858	949.0481	1860.96	1741.048	1810.042	-0.9314	1.71E-33	EN0575
1445.985	1530.681	1552.241	2555.967	2748.526	2838.307	-0.84645	1.73E-33	EN0576
2117.303	2290.573	2274.978	4377.508	4192.578	3716.352	-0.87874	1.78E-33	EN0577
388.232	399.2651	355.893	844.3818	910.9281	925.65	-1.22989	1.86E-33	EN0578
274.9977	290.2845	269.2011	54.97818	28.33532	24.33137	2.952213	1.87E-33	EN0579
1416.328	1480.154	1533.99	2581.9	2530.239	2590.762	-0.79802	2.01E-33	EN0580
670.4192	792.5857	704.4857	1492.709	1604.619	1457.766	-1.07241	2.04E-33	EN0581
393.6242	425.0241	426.1591	977.1593	961.302	890.7397	-1.18494	2.13E-33	EN0582
553.5901	584.532	552.0905	198.1289	182.6054	208.4035	1.520323	2.20E-33	EN0583

1949.248	1996.325	2026.765	3236.451	3500.986	3332.34	-0.75369	2.48E-33	EN0584
1129.647	1033.334	1040.303	1912.826	2170.276	2162.318	-0.96287	2.49E-33	EN0585
314.5398	325.9509	275.589	65.35142	59.81901	65.58891	2.263298	2.51E-33	EN0586
1036.184	958.038	1127.907	1939.796	2201.759	2169.724	-1.01482	2.57E-33	EN0587
1780.296	1786.29	1767.602	983.3833	984.3901	929.8815	0.880293	2.64E-33	EN0588
1699.414	1567.338	1708.287	2804.924	2904.895	2953.617	-0.7998	2.72E-33	EN0589
294.7688	273.4421	249.1251	727.1642	689.4928	697.1467	-1.37073	2.83E-33	EN0590
1280.627	1326.59	1456.424	2396.219	2607.899	2678.567	-0.91872	3.04E-33	EN0591
913.9629	859.9555	863.2687	399.3698	370.4581	342.755	1.24503	3.20E-33	EN0592
294.7688	328.9231	362.2808	784.217	851.1091	850.5401	-1.3343	3.24E-33	EN0593
210.2924	205.0816	234.5244	577.7895	617.0803	598.7633	-1.46388	3.27E-33	EN0594
630.8771	490.4124	517.4137	160.7852	172.1108	159.7407	1.734661	3.66E-33	EN0595
10725.81	11456.83	10964.24	19567.05	17915.27	17055.23	-0.71845	3.85E-33	EN0596
471.8098	496.3568	460.8358	979.234	1094.583	1129.822	-1.16507	4.06E-33	EN0597
934.6327	851.0389	905.2458	1719.883	1649.745	1786.769	-0.93772	4.15E-33	EN0598
1687.731	1677.31	1639.846	2774.842	2930.082	2783.297	-0.76214	4.37E-33	EN0599
657.8376	697.4755	595.8927	243.7712	237.1771	233.7927	1.448353	4.52E-33	EN0600
657.8376	554.81	640.6074	1258.274	1474.486	1451.419	-1.17387	5.11E-33	EN0601
797.1338	848.0667	789.3525	256.2191	342.1228	308.9026	1.424133	5.28E-33	EN0602
1348.927	1407.83	1423.572	2377.547	2465.173	2402.458	-0.79353	5.33E-33	EN0603
1516.082	1533.653	1526.69	2590.198	2560.673	2563.257	-0.75334	5.44E-33	EN0604
1223.111	1286.961	1248.363	595.4241	573.0032	466.5276	1.200477	5.57E-33	EN0605
635.3705	642.9852	641.52	1262.423	1282.436	1244.074	-0.98086	5.64E-33	EN0606
918.4564	978.8434	963.6488	1762.414	1800.867	1721.18	-0.88559	6.48E-33	EN0607
1544.84	1283.989	1470.112	2670.072	3278.502	3069.984	-1.06835	6.67E-33	EN0608
747.7062	783.6692	721.8241	319.4958	305.3918	288.8028	1.301734	6.69E-33	EN0609
232.7595	216.9703	216.2735	566.379	617.0803	642.1366	-1.45421	6.70E-33	EN0610
1158.405	1233.462	1142.508	585.0508	553.0635	512.0167	1.09841	6.99E-33	EN0611
683.8995	645.9574	649.7329	257.2564	265.5125	235.9085	1.383698	7.07E-33	EN0612
2495.649	2221.222	2397.259	1152.467	1226.814	1328.704	0.940442	7.22E-33	EN0613
2720.32	2712.625	2417.335	1462.627	1416.766	1438.725	0.862206	7.43E-33	EN0614
906.7734	954.0751	867.8315	420.1163	364.1614	367.0863	1.244349	7.55E-33	EN0615
2315.013	2166.731	2230.263	3621.299	3708.779	3660.284	-0.71118	7.77E-33	EN0616
581.4494	585.5227	614.1436	1185.661	1185.886	1232.437	-1.01667	7.97E-33	EN0617
978.6683	1103.676	1094.143	2086.059	2025.451	1927.468	-0.92735	8.56E-33	EN0618
768.3759	793.5765	714.5237	307.0479	314.8369	309.9605	1.288368	8.75E-33	EN0619
2213.462	2426.303	2281.366	1260.349	1284.535	1130.88	0.912637	9.17E-33	EN0620
2642.135	2863.216	2622.658	1476.112	1197.43	1409.104	0.993171	9.64E-33	EN0621
277.6938	290.2845	299.3152	742.7241	703.1358	692.9151	-1.30241	1.02E-32	EN0622
263.3148	242.7294	246.3875	46.67959	37.78043	45.48908	2.533751	1.06E-32	EN0623
613.802	663.7906	694.4477	1325.7	1350.65	1307.547	-1.01472	1.08E-32	EN0624
2252.105	2338.128	2385.396	3739.554	4251.348	4168.07	-0.80164	1.16E-32	EN0625
873.5221	977.8527	977.337	1894.154	1957.236	1733.875	-0.98191	1.18E-32	EN0626
163.5607	169.4152	187.9845	9.335917	13.64293	9.520971	4.002989	1.23E-32	EN0627
142939.5	138851.1	129208.3	205372.5	207311.7	212945	-0.60617	1.26E-32	EN0628
1853.089	2011.186	1777.64	937.741	729.3722	682.3363	1.263426	1.31E-32	EN0629
1296.803	1271.109	1152.546	548.7445	409.288	364.9706	1.491219	1.33E-32	EN0630
1434.302	1556.44	1432.698	717.8283	651.7124	539.5217	1.211816	1.42E-32	EN0631
576.9559	563.7266	522.889	173.2331	92.35216	101.557	2.178922	1.45E-32	EN0632
1016.413	1092.778	1075.892	1871.333	2137.743	2086.151	-0.93656	1.54E-32	EN0633
190.5213	272.4513	239.9997	31.11972	25.18695	31.73657	2.996123	1.57E-32	EN0634
346.8925	348.7377	313.9159	770.7318	800.7352	825.1508	-1.24762	1.70E-32	EN0635
1362.407	1416.747	1302.203	695.0072	655.9102	588.1844	1.073256	1.70E-32	EN0636
1410.936	1631.736	1327.755	2728.162	2841.928	2954.675	-0.96452	1.72E-32	EN0637

751.3009	724.2252	746.4628	324.6825	270.7597	271.8766	1.357056	1.75E-32	EN0638
685.6969	646.9481	740.075	1339.185	1498.624	1448.245	-1.0475	1.83E-32	EN0639
1865.671	1901.215	1925.473	3160.727	3053.918	3240.304	-0.73207	1.84E-32	EN0640
494.2769	515.1807	398.7827	123.4416	87.10488	60.29948	2.376914	1.93E-32	EN0641
5120.709	5277.63	5380.373	7916.858	8074.517	7929.911	-0.60035	1.99E-32	EN0642
354.082	338.8304	383.2694	66.38874	70.31358	100.4991	2.182891	2.10E-32	EN0643
1791.978	1746.661	1746.613	988.5699	950.8074	891.7976	0.900516	2.18E-32	EN0644
2730.206	2466.923	2750.414	1450.179	1520.662	1384.772	0.867887	2.27E-32	EN0645
751.3009	781.6877	755.5883	1480.262	1463.992	1414.393	-0.92975	2.35E-32	EN0646
341.5004	261.5533	314.8284	978.1967	893.0874	758.504	-1.51711	2.64E-32	EN0647
591.3349	591.4671	649.7329	1329.85	1242.556	1215.511	-1.04737	2.72E-32	EN0648
379.2452	329.9138	360.4558	818.4487	842.7134	834.6718	-1.22134	2.76E-32	EN0649
1052.36	1073.954	1126.995	1919.05	2108.358	2250.123	-0.94817	2.82E-32	EN0650
2119.999	2097.38	2317.867	3595.365	3971.143	4051.702	-0.82988	2.91E-32	EN0651
913.9629	958.038	831.3296	381.7353	378.8537	312.0763	1.33298	2.93E-32	EN0652
1068.537	1002.621	952.6983	1886.893	1890.071	2126.35	-0.96495	3.02E-32	EN0653
756.693	704.4106	773.8392	1400.388	1561.591	1494.792	-0.99518	3.21E-32	EN0654
544.6033	547.8749	590.4174	1224.042	1136.561	1139.343	-1.05625	3.31E-32	EN0655
569.7665	535.9861	531.1019	146.2627	187.8527	192.5352	1.636646	3.33E-32	EN0656
465.519	450.7831	532.927	1071.556	1118.72	1038.844	-1.15524	3.39E-32	EN0657
1529.562	1617.866	1619.77	2784.178	2775.812	2624.614	-0.77998	3.43E-32	EN0658
807.9181	821.317	825.8543	337.1303	324.282	246.4874	1.434746	3.58E-32	EN0659
1085.612	1112.592	1196.348	2012.409	2395.909	2347.448	-0.99277	3.72E-32	EN0660
2902.753	2726.495	2906.46	1399.35	1534.305	1662.996	0.893208	3.90E-32	EN0661
5488.271	5653.118	5655.961	8305.854	9043.165	9014.244	-0.65031	4.37E-32	EN0662
379.2452	383.4134	352.2428	109.9564	94.45107	89.92028	1.920775	4.39E-32	EN0663
1605.951	1713.967	1803.191	3004.091	3283.749	2950.443	-0.85069	4.40E-32	EN0664
1153.912	1096.741	1020.227	509.3262	467.0081	516.2482	1.131898	4.56E-32	EN0665
286.6806	319.0158	329.4292	732.3508	759.8064	786.0091	-1.28507	4.64E-32	EN0666
647.0534	794.5672	745.5503	1458.478	1699.07	1624.912	-1.12944	5.04E-32	EN0667
624.5863	664.7813	628.7443	1232.341	1275.089	1347.746	-1.0075	5.07E-32	EN0668
6467.838	6530.907	6694.439	9601.472	9857.543	9848.916	-0.57358	5.46E-32	EN0669
2730.206	2729.467	2789.654	620.3198	433.4255	385.0704	2.519243	6.06E-32	EN0670
211.191	182.2947	225.3989	28.00775	25.18695	21.15771	3.057144	6.11E-32	EN0671
1736.26	1809.077	1876.195	2954.299	3205.04	3244.535	-0.79456	6.43E-32	EN0672
1460.364	1426.654	1347.831	2375.472	2592.157	2598.167	-0.83716	6.96E-32	EN0673
2027.434	2016.14	2132.621	3519.641	3767.548	3350.324	-0.78429	7.18E-32	EN0674
223.7726	173.3781	158.783	10.37324	11.54402	16.92617	3.841311	7.43E-32	EN0675
988.5538	908.5014	1016.576	441.9001	402.9912	447.4856	1.173197	7.52E-32	EN0676
1268.944	1378.108	1456.424	2430.45	2451.53	2560.083	-0.85897	7.59E-32	EN0677
2385.111	2522.404	2386.308	1368.231	1346.452	1167.906	0.909379	7.65E-32	EN0678
1300.398	1250.304	1284.865	2335.017	2175.523	2216.27	-0.81032	7.79E-32	EN0679
1462.161	1457.367	1546.766	2503.063	2712.845	2866.87	-0.85559	7.83E-32	EN0680
6774.29	7225.41	7012.918	4399.292	4501.118	4619.787	0.636067	7.92E-32	EN0681
1353.42	1383.062	1354.219	2266.553	2493.508	2433.137	-0.81436	8.04E-32	EN0682
1703.907	1898.243	1917.26	3107.823	3515.679	3461.402	-0.86979	8.08E-32	EN0683
389.1307	449.7924	390.5698	117.2176	118.5886	116.3674	1.802983	8.24E-32	EN0684
398.1176	417.0982	375.0565	887.9495	896.2357	868.5241	-1.15682	8.31E-32	EN0685
478.1006	459.6997	505.5506	1071.556	1102.979	978.5442	-1.12693	8.61E-32	EN0686
1806.357	1983.446	1964.712	3155.54	3335.172	3371.482	-0.77745	9.55E-32	EN0687
164.4594	178.3318	227.224	584.0135	622.3276	562.7952	-1.63353	1.01E-31	EN0688
5718.334	6096.966	5556.494	3438.73	3017.187	2729.345	0.919188	1.02E-31	EN0689
185.1292	209.0445	204.4104	596.4614	553.0635	531.0586	-1.49031	1.03E-31	EN0690
2350.961	2395.59	2430.111	3742.665	4050.901	3919.466	-0.70673	1.04E-31	EN0691

464.6203	429.9778	478.1742	942.9276	1119.77	1089.622	-1.19858	1.17E-31	EN0692
1167.392	1084.852	1008.364	502.0649	376.7548	421.0385	1.326608	1.18E-31	EN0693
1349.825	1340.461	1541.291	2514.474	2994.099	2880.623	-0.98708	1.21E-31	EN0694
1086.511	987.76	1152.546	2042.491	2162.93	2489.205	-1.05233	1.26E-31	EN0695
1662.568	1688.208	1724.712	2896.209	2733.834	2910.243	-0.75075	1.29E-31	EN0696
6335.731	6761.747	6581.283	9750.847	10258.44	9995.962	-0.60867	1.33E-31	EN0697
499.669	510.2271	562.1285	1242.714	1147.056	1067.407	-1.1369	1.34E-31	EN0698
1433.403	1350.368	1474.675	2424.227	2771.614	2854.176	-0.9183	1.36E-31	EN0699
801.6273	855.0019	931.7097	1678.39	1720.059	1630.202	-0.95819	1.41E-31	EN0700
345.0951	321.988	302.0528	737.5375	786.0428	805.051	-1.26444	1.48E-31	EN0701
357.6767	333.8767	387.8321	795.6276	924.571	885.4503	-1.27055	1.69E-31	EN0702
7248.795	7312.594	7272.081	11212.44	10622.6	10692.05	-0.57513	2.02E-31	EN0703
436.761	486.4495	466.3111	150.412	154.2701	145.9882	1.62397	2.05E-31	EN0704
1126.053	1191.851	1166.234	2043.529	1999.214	2078.745	-0.81335	2.14E-31	EN0705
1806.357	1772.42	1627.07	914.9199	944.5107	904.4923	0.913335	2.18E-31	EN0706
687.4942	670.7257	696.2728	264.5177	273.9081	206.2877	1.463806	2.27E-31	EN0707
1877.354	1979.483	2080.605	3277.944	3459.008	3684.616	-0.81166	2.36E-31	EN0708
3303.567	3479.451	3144.634	1805.981	1396.826	1330.82	1.130544	2.43E-31	EN0709
465.519	423.0426	467.2237	970.9354	1186.935	1019.802	-1.22813	2.44E-31	EN0710
965.188	875.8072	881.5196	368.2501	426.0793	366.0284	1.230653	2.61E-31	EN0711
5093.748	5276.64	5346.608	7928.268	8096.556	7781.807	-0.59909	2.63E-31	EN0712
1339.041	1448.45	1450.948	2500.988	2549.129	2381.301	-0.81038	2.68E-31	EN0713
1871.961	1891.308	1928.21	3051.808	3438.019	3489.965	-0.81014	2.88E-31	EN0714
417.8887	436.9129	400.6078	120.3296	81.85759	60.29948	2.256433	3.13E-31	EN0715
1705.705	1817.994	1727.45	866.1657	748.2624	605.1106	1.241984	3.18E-31	EN0716
55.71849	51.51807	46.53986	269.7043	313.7874	234.8506	-2.41174	3.24E-31	EN0717
816.0062	856.9833	857.7934	1674.241	1600.421	1517.008	-0.92122	3.47E-31	EN0718
977.7696	1050.176	1093.23	1873.407	1980.324	1889.384	-0.8799	3.61E-31	EN0719
2220.651	2225.184	2175.51	3830.838	3529.322	3552.38	-0.72087	3.75E-31	EN0720
271.403	278.3957	232.6993	52.90353	47.22554	46.54697	2.414723	3.80E-31	EN0721
984.9591	906.5199	973.6868	1740.63	1956.187	2145.392	-1.02742	3.92E-31	EN0722
327.1214	268.4884	262.8133	836.0833	779.7461	698.2045	-1.4298	4.09E-31	EN0723
273.2003	263.5348	302.9653	682.5593	753.5097	680.2205	-1.3329	4.16E-31	EN0724
3330.528	3741.005	3435.737	1827.765	1729.504	1357.267	1.096002	4.30E-31	EN0725
510.4532	520.1344	562.1285	1109.937	1168.045	1062.117	-1.06829	4.35E-31	EN0726
157.2699	187.2484	156.9579	8.298593	4.197825	6.347314	4.731819	4.37E-31	EN0727
2512.724	2393.609	2362.582	1386.902	1196.38	1111.838	0.976006	4.38E-31	EN0728
1509.791	1557.431	1459.161	695.0072	532.0744	439.0226	1.44145	4.46E-31	EN0729
204.0016	179.3225	173.3838	17.63451	14.69239	23.27348	3.324897	4.56E-31	EN0730
1709.299	1775.392	1707.374	2948.075	2798.9	2872.16	-0.73146	4.75E-31	EN0731
81.78036	72.32345	74.82879	306.0106	324.282	299.3816	-2.02061	4.94E-31	EN0732
2359.049	2563.024	2189.198	1208.483	931.9172	863.2347	1.243031	5.33E-31	EN0733
164.4594	156.5357	171.5587	453.3106	518.4314	492.9747	-1.57148	5.42E-31	EN0734
733.3272	771.7804	732.7746	1416.985	1392.629	1409.104	-0.91507	5.48E-31	EN0735
204.9002	203.1001	200.7602	26.97043	31.48369	25.38926	2.860015	5.53E-31	EN0736
918.4564	896.6126	1018.402	428.4149	420.832	424.2122	1.154082	5.61E-31	EN0737
979.567	1051.167	945.3979	480.2811	439.7222	431.6174	1.138216	5.73E-31	EN0738
3246.95	3449.729	3354.52	2040.417	1890.071	1740.222	0.825546	5.90E-31	EN0739
205.7989	195.1742	176.1214	567.4163	622.3276	507.7851	-1.55665	6.05E-31	EN0740
585.9428	599.393	551.1779	1122.385	1192.182	1225.032	-1.02768	6.12E-31	EN0741
484.3914	468.6163	510.1133	993.7565	1042.11	1024.033	-1.06397	6.14E-31	EN0742
4537.462	5071.558	5098.396	7900.261	8009.451	7799.791	-0.68907	6.16E-31	EN0743
738.7193	734.1325	659.7709	266.5923	222.4847	163.9723	1.706659	6.27E-31	EN0744
1484.628	1547.524	1524.865	784.217	639.1189	578.6635	1.186279	6.29E-31	EN0745

1610.444	1516.811	1363.344	674.2607	537.3216	436.9068	1.445434	6.41E-31	EN0746
650.6481	584.532	599.5429	203.3155	218.2869	143.8725	1.697851	6.44E-31	EN0747
443.9505	466.6349	429.8093	105.8071	153.2206	113.1938	1.848364	7.19E-31	EN0748
2366.238	2539.247	2483.951	3969.839	3934.412	3919.466	-0.67838	7.38E-31	EN0749
13047.11	13115.31	13039.37	18560.84	18923.8	18480.2	-0.51361	7.71E-31	EN0750
208.495	186.2576	226.3115	567.4163	552.014	627.3262	-1.49043	7.80E-31	EN0751
420.5847	416.1075	465.3986	118.255	105.9951	61.35737	2.187881	7.91E-31	EN0752
1800.965	1822.947	1888.058	3090.189	2987.802	2974.775	-0.71578	7.92E-31	EN0753
732.4285	748.0028	709.961	1412.835	1346.452	1399.583	-0.92526	7.98E-31	EN0754
303.7556	335.8582	292.9273	754.1346	780.7955	713.0149	-1.27047	8.10E-31	EN0755
541.9072	544.9027	615.0562	1125.497	1248.853	1311.778	-1.1146	8.89E-31	EN0756
1015.514	915.4365	844.1053	348.5409	281.2543	200.9983	1.739499	9.89E-31	EN0757
165.3581	184.2762	162.4332	1.037324	6.296738	10.57886	4.843122	9.91E-31	EN0758
678.5074	724.2252	714.5237	311.1972	267.6114	269.7608	1.318621	9.95E-31	EN0759
1407.341	1450.432	1439.998	776.9558	685.295	640.0208	1.031336	1.06E-30	EN0760
624.5863	647.9388	696.2728	1273.834	1378.986	1284.273	-0.99976	1.08E-30	EN0761
8243.64	9136.532	7450.94	4811.109	4495.871	4679.028	0.828026	1.11E-30	EN0762
4202.252	4180.89	4277.104	6371.245	7015.616	7262.385	-0.70572	1.12E-30	EN0763
2253.903	2273.73	2115.282	1204.333	945.5602	908.7238	1.118668	1.13E-30	EN0764
1007.426	1043.241	1102.356	1842.288	2122.001	2167.608	-0.95951	1.14E-30	EN0765
1233.895	1159.157	1142.508	590.2374	558.3108	598.7633	1.016966	1.14E-30	EN0766
3947.026	4093.705	4056.268	6224.982	6134.072	6018.312	-0.60338	1.15E-30	EN0767
416.99	440.8758	416.1211	127.5909	73.46194	68.76257	2.237962	1.17E-30	EN0768
1050.563	995.6858	1098.706	1896.229	2107.308	1894.673	-0.90684	1.18E-30	EN0769
1101.788	974.8805	1060.379	1876.519	2002.363	1912.657	-0.8839	1.23E-30	EN0770
287.5793	299.2011	287.4521	669.0741	787.0923	885.4503	-1.42155	1.27E-30	EN0771
651.5468	586.5134	622.3565	246.8831	230.8804	243.3137	1.367796	1.33E-30	EN0772
1161.101	1256.248	1302.203	2197.053	2542.833	2290.322	-0.91857	1.38E-30	EN0773
923.8485	841.1316	905.2458	1635.86	1638.201	1645.012	-0.88084	1.45E-30	EN0774
779.1601	812.4004	882.4322	1514.493	1636.102	1688.386	-0.96775	1.46E-30	EN0775
691.9877	673.6979	752.8506	1382.753	1510.168	1350.92	-1.00192	1.64E-30	EN0776
5940.31	6356.538	5707.977	3709.471	3514.629	3195.873	0.788858	1.65E-30	EN0777
562.577	529.051	546.6152	1098.526	1087.237	1099.143	-1.00328	1.75E-30	EN0778
699.1771	699.4569	720.9115	270.7416	157.4185	197.8246	1.759087	1.86E-30	EN0779
2034.623	2010.196	2226.613	3434.58	3701.433	3517.47	-0.76426	1.90E-30	EN0780
931.0379	919.3995	956.3484	1654.532	1686.476	1688.386	-0.84129	1.93E-30	EN0781
30386.35	30040.98	31466.42	20644.82	20064.56	18303.54	0.638933	1.97E-30	EN0782
908.5708	865.8999	865.0938	1595.405	1648.696	1577.308	-0.86895	2.03E-30	EN0783
3165.17	3325.888	3276.953	1597.479	1760.988	1954.973	0.878472	2.05E-30	EN0784
1903.415	1899.234	1930.948	925.2931	1092.484	1030.381	0.911646	2.09E-30	EN0785
1360.61	1426.654	1228.287	620.3198	457.563	418.9227	1.423191	2.14E-30	EN0786
887.9011	835.1872	753.7632	358.9142	296.9961	268.703	1.421201	2.17E-30	EN0787
1010.122	1001.63	906.1584	1796.645	1776.73	1777.248	-0.87498	2.29E-30	EN0788
1024.501	911.4736	1017.489	1763.451	1879.576	1862.937	-0.89788	2.41E-30	EN0789
4457.479	4495.943	4632.085	2684.595	2880.758	2913.417	0.680212	2.44E-30	EN0790
4547.348	4970.503	5096.571	7799.64	8660.114	7879.133	-0.73595	2.56E-30	EN0791
826.7905	871.8443	918.0215	1670.092	1629.806	1580.481	-0.89939	2.75E-30	EN0792
321.7293	356.6636	414.296	843.3445	902.5325	910.8396	-1.28171	3.02E-30	EN0793
573.3612	587.5042	556.6532	1137.945	1139.71	1117.127	-0.9833	3.05E-30	EN0794
295.6675	236.785	275.589	671.1487	680.0477	735.2305	-1.36677	3.13E-30	EN0795
62.90797	54.49027	48.36495	263.4803	353.6668	257.0662	-2.39816	3.36E-30	EN0796
3329.629	3627.071	3808.968	5733.291	6403.783	6239.41	-0.77147	3.37E-30	EN0797
430.4702	405.2095	365.0185	957.4502	916.1754	862.1768	-1.18815	3.40E-30	EN0798
1274.336	1286.961	1314.067	606.8346	422.9309	403.0544	1.435096	3.48E-30	EN0799

371.157	367.5616	351.3303	95.43382	95.50053	113.1938	1.841943	3.69E-30	EN0800
1143.128	1172.036	1203.648	2158.672	1997.115	2039.604	-0.81618	3.79E-30	EN0801
2591.808	2470.886	2443.799	1436.694	1493.376	1319.183	0.820944	3.89E-30	EN0802
177.041	149.6006	156.9579	8.298593	11.54402	12.69463	3.895658	3.98E-30	EN0803
5819.886	6281.242	6337.633	9591.099	9302.381	9387.677	-0.6172	4.02E-30	EN0804
1264.45	1263.184	1409.884	2260.329	2369.672	2455.353	-0.84729	4.07E-30	EN0805
412.4965	383.4134	390.5698	864.091	838.5156	883.3345	-1.12351	4.08E-30	EN0806
177.9397	167.4337	158.783	16.59719	8.395651	4.231543	4.104161	4.14E-30	EN0807
1198.846	1413.775	1322.279	2355.763	2507.151	2367.548	-0.87836	4.18E-30	EN0808
212.9884	230.8406	197.11	607.8719	650.6629	535.2902	-1.4859	4.28E-30	EN0809
702.7719	692.5218	636.9573	1325.7	1279.287	1386.888	-0.97411	4.54E-30	EN0810
278.5924	256.5996	266.4635	60.1648	32.53315	48.66274	2.503146	4.65E-30	EN0811
526.6296	573.6339	565.7787	1078.817	1144.957	1201.758	-1.04026	4.84E-30	EN0812
128.512	123.8415	114.0683	484.4304	463.8597	361.7969	-1.83834	5.27E-30	EN0813
467.3163	459.6997	470.8738	922.1812	1055.753	1020.86	-1.10089	5.46E-30	EN0814
719.8469	657.8462	617.7938	264.5177	253.9684	208.4035	1.45673	5.47E-30	EN0815
658.7363	695.494	650.6455	1297.692	1353.799	1247.247	-0.95996	5.49E-30	EN0816
2902.753	3144.584	2969.425	5378.526	4952.384	4755.196	-0.74279	5.58E-30	EN0817
2531.596	2413.424	2790.566	4263.402	4414.013	4378.589	-0.75485	6.00E-30	EN0818
473.6071	411.1539	418.8587	913.8826	1187.985	1182.716	-1.33249	6.09E-30	EN0819
2925.221	3088.112	3065.243	4714.638	5036.341	4701.244	-0.67088	6.27E-30	EN0820
875.3195	942.1863	881.5196	435.6761	370.4581	384.0125	1.180782	6.37E-30	EN0821
6821.021	6906.394	7234.666	10317.23	10297.27	10665.6	-0.57743	6.39E-30	EN0822
291.174	254.6182	274.6764	637.9543	716.7787	687.6257	-1.31461	6.44E-30	EN0823
1253.666	1209.684	1306.766	645.2156	559.3602	500.3799	1.144752	6.72E-30	EN0824
402.611	418.089	421.5964	919.0692	892.0379	855.8295	-1.10242	7.00E-30	EN0825
1933.072	2026.047	1872.545	1032.138	914.0765	766.9671	1.10357	7.09E-30	EN0826
1311.182	1359.285	1309.504	682.5593	673.751	724.6517	0.935414	7.96E-30	EN0827
2212.563	2291.564	2257.639	3520.678	3854.653	3996.692	-0.75007	8.07E-30	EN0828
2388.705	2527.358	2582.506	4411.74	4278.634	3924.756	-0.7506	8.21E-30	EN0829
2625.06	2667.051	2661.897	4100.542	4626.004	4730.865	-0.75863	8.77E-30	EN0830
883.4076	889.6775	895.2078	1616.151	1706.416	1560.381	-0.87187	8.80E-30	EN0831
3175.954	3409.109	3295.204	5636.819	5511.745	5041.883	-0.71272	8.94E-30	EN0832
1980.702	2103.324	1977.488	3376.49	3279.551	3220.204	-0.7046	9.13E-30	UBE2L3
320.8306	361.6172	312.0908	777.9931	859.5047	748.9831	-1.26392	9.13E-30	POGLUT1
217.4818	171.3967	200.7602	527.998	562.5086	570.2004	-1.49183	9.32E-30	SUV39H2
1701.211	1762.513	1813.229	2816.335	3001.445	2946.212	-0.73191	9.97E-30	CAB39
1316.574	1316.683	1326.842	2181.493	2224.847	2329.464	-0.76625	1.10E-29	PPHLN1
2162.237	2225.184	2085.168	1265.535	1135.512	1065.291	0.900649	1.12E-29	OTUD5
1894.429	1818.984	1815.967	918.0319	817.5265	631.5577	1.22378	1.14E-29	SCAF1
2320.405	2479.803	2323.343	3920.048	3899.78	3705.774	-0.69447	1.19E-29	GRSF1
1422.619	1488.08	1443.648	738.5748	619.1792	526.8271	1.20781	1.22E-29	SCAMP4
243.5437	212.0167	194.3723	37.34367	28.33532	31.73657	2.737801	1.23E-29	ORAI3
1614.937	1687.217	1645.321	2631.691	2842.977	2847.828	-0.75046	1.24E-29	ATP6V1C1
5387.618	5022.021	5563.794	8295.481	9528.014	9839.395	-0.79213	1.28E-29	CASC19
1921.389	2001.279	1858.857	3155.54	3230.227	3081.621	-0.71178	1.31E-29	MAPK1
2274.572	2062.704	2470.263	3919.011	4132.759	4535.156	-0.88635	1.32E-29	FUBP1
6719.47	7341.325	7175.351	11732.14	11509.39	10577.8	-0.67144	1.44E-29	PSMD2
621.8902	623.1705	547.5277	229.2486	226.6826	228.5033	1.388815	1.45E-29	-
2615.174	2757.208	2719.388	4195.976	4278.634	4163.838	-0.64346	1.50E-29	FAM168B
904.9761	907.5107	871.4816	403.5191	358.9141	431.6174	1.168506	1.51E-29	EPB41L1
6806.642	7313.585	7175.351	10651.24	10497.71	10556.64	-0.57429	1.61E-29	EIF3B
2219.753	2343.082	2483.951	3856.771	3829.466	4145.854	-0.74768	1.92E-29	NCAPG2
2423.754	2747.3	2430.111	1346.447	966.5493	1071.638	1.16689	1.93E-29	PPP1R15A

1386.671	1365.229	1317.717	2285.225	2315.101	2248.007	-0.75089	1.95E-29	CDC23
1492.716	1390.988	1523.952	2424.227	2668.767	2703.956	-0.82248	2.00E-29	DNAJA2
485.2901	509.2363	489.1248	1017.615	979.1428	1105.491	-1.06446	2.00E-29	FAM92A
357.6767	331.8953	358.6307	65.35142	101.7973	88.8624	2.034586	2.12E-29	FCHSD1
96.15932	87.18443	124.1063	386.9219	388.2988	360.739	-1.88341	2.20E-29	TAF1A
2159.541	2339.119	2158.172	1229.229	1149.155	975.3706	0.988702	2.32E-29	CAPG
2166.73	2003.26	2014.902	1190.848	1169.094	1077.986	0.847291	2.56E-29	IRF2BP2
286.6806	225.8869	268.2886	49.79156	48.27499	26.44714	2.648246	2.57E-29	-
274.9977	277.405	313.9159	72.61269	39.87934	45.48908	2.454069	2.69E-29	HDAC11
1800.067	1981.464	1899.009	3151.391	3082.253	3117.589	-0.7195	2.73E-29	F2RL1
524.8322	446.8202	521.0639	170.1212	129.0831	102.6149	1.892998	2.85E-29	MXD3
356.7781	348.7377	353.1554	745.8361	800.7352	837.8455	-1.17111	2.91E-29	MAGOHB
733.3272	703.4198	865.0938	1583.994	1630.855	1500.082	-1.0339	3.00E-29	FAM122B
1234.794	1275.072	1294.903	2111.992	2234.293	2382.359	-0.82251	3.01E-29	JMJD1C
694.6837	678.6515	730.037	1355.783	1283.485	1413.335	-0.94582	3.09E-29	SMNDC1
288.478	351.7099	333.0794	88.17255	69.26412	78.28354	2.0448	3.15E-29	VIT
1221.313	1249.313	1152.546	615.1332	498.4918	464.4118	1.198675	3.20E-29	SERINC2
327.1214	320.0065	331.2543	744.7987	731.4711	724.6517	-1.16941	3.29E-29	HAUS3
240.8477	222.9147	225.3989	633.805	544.6678	630.4999	-1.39177	3.33E-29	AC009245.1
401.7123	397.2836	428.8967	874.4642	878.395	862.1768	-1.09034	3.65E-29	MCPH1
730.6311	596.4208	641.52	246.8831	244.5233	260.2399	1.389755	3.92E-29	RHBDF1
386.4347	428.987	448.0602	971.9727	1014.824	870.6399	-1.17773	4.18E-29	TRAPPC8
319.0333	349.7285	344.9425	789.4037	729.3722	789.1827	-1.18755	4.30E-29	ZBED3
4566.22	4631.673	4663.111	6818.332	7514.107	7687.655	-0.66777	4.43E-29	NR3C1
444.8492	460.6905	424.334	150.412	140.6272	155.5092	1.574173	4.62E-29	TMEM139
993.0472	1054.139	1028.44	1804.944	1837.598	1761.38	-0.81345	4.67E-29	POGK
859.1431	905.5292	921.6717	1589.181	1683.328	1602.697	-0.85998	4.75E-29	EPS15
480.7966	452.7646	473.6115	134.8521	146.9239	176.6669	1.618725	4.86E-29	SAMD9L
1081.118	1117.546	998.3256	527.998	520.5303	480.2801	1.063912	4.96E-29	PNPLA6
264.2135	252.6367	256.4255	625.5065	629.6738	609.3421	-1.26943	5.10E-29	LPAR1
1797.371	1934.9	1986.613	3101.599	3296.342	3153.557	-0.74014	5.15E-29	FANCI
567.0704	542.9212	632.3945	1190.848	1189.034	1396.409	-1.1153	5.58E-29	FAM76B
6357.3	6348.612	6492.766	9670.973	10034.9	11169.16	-0.68539	5.60E-29	HNRNPD
700.0758	701.4384	696.2728	1276.946	1301.326	1357.267	-0.90767	5.69E-29	RAP1GDS1
656.9389	681.6237	696.2728	1233.378	1399.975	1357.267	-0.97173	5.76E-29	SOS1
946.3156	966.9546	915.2839	471.9825	413.4858	440.0804	1.093192	5.90E-29	EPN2
967.884	981.8156	1043.04	1766.563	1754.691	1765.611	-0.82085	6.11E-29	HDGFL3
2891.071	3011.826	2896.422	1848.512	1781.977	1704.254	0.721771	6.14E-29	COL4A6
521.2375	517.1622	577.6418	1210.557	1096.682	1071.638	-1.06383	6.18E-29	MRPL32
289.3767	283.3494	338.5546	80.91128	51.42336	58.18371	2.257438	6.30E-29	COL5A3
270.5043	215.9796	253.6878	622.3945	641.2178	636.8472	-1.35858	6.40E-29	ARHGAP19
1255.463	1115.564	1184.485	2135.85	2071.627	2060.761	-0.81748	6.45E-29	HPRT1
223.7726	215.9796	208.9731	540.4459	600.289	545.869	-1.37832	6.56E-29	DNAJC25
1420.821	1244.36	1374.295	2395.181	2530.239	2310.422	-0.84044	6.93E-29	UFM1
681.2034	728.1882	698.0979	1288.357	1463.992	1353.036	-0.96234	6.93E-29	FBN1
487.9861	497.3476	482.7369	1110.974	1025.319	956.3287	-1.0752	6.93E-29	EIF2B3
1470.249	1448.45	1262.964	710.567	592.9428	553.2742	1.170917	6.95E-29	ABHD17A
12316.48	12882.49	13224.62	19338.83	18645.69	18534.16	-0.55676	7.13E-29	DDX5
2334.784	2007.223	1782.203	1003.092	907.7797	803.9931	1.173601	7.23E-29	RPL39P3
185.1292	212.0167	168.8211	26.97043	11.54402	20.09983	3.269469	7.24E-29	HES7
1091.903	1154.203	1184.485	1935.647	2092.616	2018.446	-0.8178	7.49E-29	FZD6
1912.402	1924.002	1781.29	1074.668	1010.626	1065.291	0.834216	7.50E-29	CSTB
957.0998	1006.584	975.5119	1669.055	1808.213	1857.647	-0.86024	7.60E-29	SNHG3
337.007	380.4412	275.589	86.0979	65.06629	59.2416	2.237159	7.68E-29	CDKN2D

453.8361	402.2373	420.6838	905.584	888.8895	992.2968	-1.1253	7.74E-29	RFX7
541.9072	470.5978	492.775	133.8148	147.9733	188.3037	1.679992	7.98E-29	PCSK1
1419.024	1535.635	1502.964	2520.698	2563.822	2428.906	-0.75352	8.03E-29	DHX8
178.8384	171.3967	187.9845	489.617	473.3048	541.6375	-1.48236	8.26E-29	POLE2
787.2483	888.6868	830.4171	374.474	260.2652	288.8028	1.439664	9.99E-29	BAHCC1
451.14	389.3577	436.1971	891.0614	944.5107	923.5342	-1.11067	1.01E-28	USP53
2685.272	2871.142	2933.836	4370.247	4661.685	4580.645	-0.68115	1.02E-28	DDX6
1020.008	1060.083	1096.881	1798.72	1895.318	1959.204	-0.83143	1.03E-28	VPS4B
862.7379	839.1502	914.3713	1625.487	1595.174	1543.455	-0.86441	1.05E-28	ATPAF1
1055.057	1148.259	1137.945	2547.668	2224.847	1986.709	-1.01678	1.07E-28	CDC25A
564.3744	657.8462	542.965	1189.811	1281.386	1215.511	-1.06364	1.07E-28	DNAJC19
1865.671	1783.318	1825.092	2968.822	2911.192	3182.12	-0.72703	1.09E-28	URI1
392.7255	443.848	432.5469	887.9495	925.6205	894.9713	-1.09441	1.14E-28	CRY1
435.8624	475.5514	403.3454	153.524	132.2315	128.0042	1.667026	1.17E-28	ABTB2
2405.78	2494.664	2596.194	4162.782	3931.263	3923.698	-0.68091	1.19E-28	CAPZA2
6146.109	6184.15	6064.782	10094.2	9323.37	9030.112	-0.62904	1.23E-28	SLC25A5
267.8082	235.7943	251.8628	48.75423	55.62119	53.95217	2.255235	1.25E-28	COBL
867.2313	872.835	991.9377	1638.972	1840.746	1770.901	-0.94223	1.25E-28	CUL5
3477.912	3547.812	3595.432	6048.637	5557.921	5382.522	-0.67773	1.26E-28	AK2
161.7634	160.4986	174.2963	19.70916	14.69239	16.92617	3.273719	1.44E-28	FO681492.1
516.744	567.6895	547.5277	220.95	181.5559	171.3775	1.507004	1.46E-28	COQ8B
762.9838	829.2428	868.744	1480.262	1758.889	1710.601	-1.00822	1.47E-28	CLASP2
178.8384	159.5079	177.034	21.78381	14.69239	20.09983	3.187467	1.49E-28	ATP8B4
216.5832	209.0445	240.9122	540.4459	589.7945	616.7473	-1.38927	1.65E-28	ZGRF1
350.4873	352.7007	360.4558	114.1057	99.69835	92.03605	1.797719	1.74E-28	ECM1
686.5956	628.1242	574.9041	256.2191	207.7924	185.13	1.541096	1.77E-28	MPST
169.8515	129.7859	139.6196	462.6466	433.4255	459.1224	-1.62332	1.93E-28	ANXA10
559.8809	572.6432	653.3831	1164.915	1264.595	1212.337	-1.02772	2.02E-28	LNPK
1105.383	1139.342	1203.648	1949.132	2021.253	2144.334	-0.8263	2.04E-28	CTCF
496.0743	483.4773	480.9119	1113.049	1043.16	941.5183	-1.0848	2.06E-28	DUSP12
2219.753	2191.5	2253.077	3391.013	3668.899	3667.69	-0.68668	2.06E-28	CPSF6
590.4362	557.7822	602.2805	1254.125	1112.424	1148.864	-1.00554	2.11E-28	TRIAP1
314.5398	337.8397	252.7753	73.65001	61.91792	47.60486	2.303491	2.12E-28	ZFPM1
319.0333	328.9231	337.6421	774.8811	729.3722	715.1307	-1.17125	2.14E-28	EYA3
743.2127	802.4931	790.265	1381.716	1586.778	1534.992	-0.94728	2.18E-28	DNAJC3
718.0495	792.5857	799.3905	1554.949	1575.234	1384.772	-0.96722	2.31E-28	PRPS2
7643.318	8162.642	7950.103	13580.65	11991.09	12165.69	-0.66778	2.39E-28	RPS7
256.1253	293.2567	228.1366	36.30634	58.76956	45.48908	2.46748	2.46E-28	NDUFA11
2649.324	2565.006	2819.768	4179.379	4653.289	4425.136	-0.72243	2.49E-28	TMOD3
3129.222	2970.215	2730.338	1716.771	1555.294	1408.046	0.915717	2.53E-28	G6PD
719.8469	651.9018	690.7975	1263.461	1393.678	1326.589	-0.94907	2.65E-28	CCDC117
492.4795	529.051	564.8661	1031.1	1193.232	1125.59	-1.07843	2.71E-28	SC5D
2014.852	1987.409	2108.894	3205.332	3636.366	3711.063	-0.78794	2.78E-28	USP24
300.1609	330.9045	371.4063	761.3959	777.6472	853.7137	-1.25513	3.08E-28	MTBP
425.9768	395.3021	417.0336	869.2776	853.208	879.103	-1.07042	3.10E-28	DPH3
613.802	560.7544	584.9421	233.3979	240.3255	199.9404	1.38517	3.29E-28	CPTP
1598.761	1740.716	1718.325	2708.453	3049.72	3029.785	-0.7972	3.44E-28	SETX
1814.446	1853.66	2055.054	970.9354	1080.94	1029.323	0.89347	3.56E-28	FAM102B
546.4007	578.5876	585.8547	1092.302	1277.188	1143.574	-1.03819	3.58E-28	MPP6
239.0503	252.6367	213.5358	30.0824	46.17608	16.92617	2.918786	3.62E-28	RAB26
2330.291	2590.765	2438.323	1453.291	1246.754	1208.105	0.912745	3.72E-28	NOP53
324.4254	373.506	326.6915	729.2389	874.1971	893.9134	-1.28623	3.95E-28	RALGAPA1
1509.791	1327.581	1434.523	765.5452	707.3336	744.7515	0.946231	4.02E-28	AL161431.1
628.181	661.8091	737.3373	1296.655	1347.502	1328.704	-0.97051	4.05E-28	KIF21A

2228.739	2436.21	2184.636	1272.797	1031.616	954.2129	1.071382	4.19E-28	PKP3
926.5445	896.6126	1031.177	1717.809	1849.142	1737.048	-0.8935	4.27E-28	PIK3R4
1034.387	1170.055	1135.207	2183.567	2009.709	1940.162	-0.87752	4.52E-28	HMGB1P6
1238.388	1278.045	1233.762	2376.51	2113.605	2124.234	-0.81888	4.57E-28	DRG1
3178.65	3286.259	3335.356	5240.562	4858.983	5298.949	-0.65194	4.63E-28	HMGB2
167.1555	205.0816	151.4827	18.67183	20.98913	14.8104	3.263625	4.77E-28	RAB17
1351.623	1282.998	1346.918	2152.448	2386.464	2351.68	-0.79098	5.34E-28	FEM1B
2518.116	2642.283	2609.882	3932.496	4277.584	4389.168	-0.69738	5.69E-28	USP47
5980.751	6447.685	6130.485	10635.68	10003.42	9233.226	-0.68682	5.71E-28	SEC61A1
803.4246	857.9741	900.6831	1522.792	1598.322	1578.365	-0.8753	5.75E-28	SRD5A1
1472.945	1403.867	1344.181	735.4628	781.845	706.6676	0.924466	6.25E-28	TSC22D2
576.9559	594.4393	615.0562	1176.326	1177.49	1418.625	-1.07833	6.79E-28	EIF1AXP1
709.0627	722.2438	674.3717	1256.2	1373.738	1409.104	-0.9399	6.94E-28	ARID4B
1099.092	1170.055	1105.093	1941.871	1885.873	1984.594	-0.78493	7.07E-28	ZPR1
257.9227	292.266	302.0528	681.522	736.7184	649.5418	-1.27933	7.10E-28	PRIM1
1537.651	1620.838	1539.465	870.315	886.7906	795.53	0.879741	7.61E-28	TMEM184B
647.0534	680.633	615.0562	287.3388	231.9299	243.3137	1.348523	7.65E-28	IQSEC2
1170.987	1205.721	1305.854	2071.536	2146.138	2212.039	-0.80393	8.33E-28	PPP2R5E
370.2583	361.6172	344.9425	119.2923	87.10488	85.68874	1.881572	8.57E-28	ARMC5
667.7232	673.6979	719.999	1294.581	1543.75	1331.878	-1.01633	8.69E-28	MPHOSPH9
852.8523	777.7248	890.6451	1496.859	1643.449	1670.401	-0.93143	8.87E-28	SMC6
656.0403	680.633	607.7558	264.5177	262.3641	203.114	1.412768	9.83E-28	SMAD7
2358.15	2257.879	2435.586	3743.703	3680.443	4017.85	-0.69807	9.96E-28	SUMO1
562.577	466.6349	452.6229	1012.428	1187.985	1202.816	-1.19877	1.06E-27	FOXN2
464.6203	354.6821	383.2694	103.7324	97.59944	126.9463	1.874241	1.06E-27	CXCL2
540.1099	559.7637	540.2274	1030.063	1075.693	1127.706	-0.97951	1.08E-27	UPF2
738.7193	748.0028	750.113	1615.114	1498.624	1324.473	-0.98867	1.15E-27	TWNK
306.4517	282.3587	283.8019	712.6417	668.5037	650.5997	-1.21888	1.17E-27	EEF1E1
237.2529	312.0806	252.7753	659.7382	746.1635	680.2205	-1.38101	1.18E-27	SIX4
336.1083	410.1631	328.5166	105.8071	71.36303	89.92028	2.007421	1.20E-27	HCFC1R1
1037.981	1199.777	1015.664	471.9825	539.4206	458.0645	1.146116	1.22E-27	CARHSP1
2241.321	2378.748	2483.038	3732.292	3950.154	3791.462	-0.6919	1.29E-27	GAPVD1
599.4231	580.5691	626.0067	213.6888	257.1168	243.3137	1.339023	1.29E-27	-
245.3411	266.507	285.627	612.0212	685.295	647.426	-1.28622	1.34E-27	LIN9
2268.282	2312.369	2079.693	1290.431	1050.506	1062.117	0.968493	1.34E-27	NECTIN2
957.9985	1080.889	959.9986	1981.289	1798.768	1787.827	-0.89342	1.43E-27	CDC45
409.8005	390.3485	407.9082	821.5607	894.1368	1015.57	-1.17639	1.48E-27	TMA16
535.6164	549.8564	525.6266	226.1367	189.9516	188.3037	1.413977	1.50E-27	GRIN2D
150.0804	187.2484	126.8439	6.223945	10.49456	11.63674	4.033907	1.52E-27	C19orf71
720.7456	719.2716	677.1093	321.5705	283.3532	246.4874	1.313698	1.57E-27	FAM160B2
5000.285	5049.762	4677.712	1022.802	683.1961	595.5896	2.677748	1.64E-27	IGFBP6
1761.423	1765.485	1707.374	2825.671	2733.834	2807.629	-0.67682	1.65E-27	SMG7
1314.777	1353.34	1265.702	623.4318	495.3434	390.3598	1.381755	1.66E-27	EGFL7
1925.883	1926.974	1784.94	1101.638	1007.478	1068.465	0.827054	1.69E-27	FAM50A
1921.389	1860.595	2176.423	3330.848	3478.948	3394.755	-0.77592	1.73E-27	TPD52
694.6837	738.0955	772.0141	1335.036	1475.536	1378.425	-0.92606	1.77E-27	FARP2
555.3875	635.0593	604.1056	235.4726	230.8804	249.661	1.325155	1.78E-27	C22orf46
2368.036	2469.895	2406.384	1515.531	1361.145	1382.657	0.766009	1.80E-27	PHGDH
262.4161	292.266	300.2277	636.917	695.7896	690.7993	-1.24345	1.83E-27	MECOM
257.024	292.266	302.9653	671.1487	663.2564	675.9889	-1.23871	1.84E-27	DCLRE1A
395.4215	420.0704	439.8473	860.979	920.3732	865.3505	-1.07627	1.91E-27	FAM204A
2162.237	2291.564	1994.826	1192.923	1001.181	902.3765	1.057997	1.93E-27	TSKU
733.3272	785.6506	773.8392	1387.94	1372.689	1412.277	-0.86428	1.94E-27	ATMIN
907.6721	1114.574	1014.751	2082.947	1975.077	1840.721	-0.95858	1.98E-27	LARS2

612.0047	596.4208	595.8927	1279.021	1146.006	1128.764	-0.97789	2.00E-27	RRS1
1464.857	1431.608	1483.8	2333.979	2456.777	2393.995	-0.71374	2.01E-27	MRS2
284.8832	318.025	296.5775	73.65001	81.85759	51.8364	2.116065	2.02E-27	FGFR4
761.1864	819.3355	817.6414	384.8473	362.0624	345.9286	1.133458	2.04E-27	CELSR2
1590.673	1695.143	1652.621	2611.982	2868.164	2735.692	-0.73456	2.06E-27	CCNT1
2373.428	2433.238	2258.552	1387.94	1175.391	1091.738	0.950564	2.10E-27	CSNK1G2
787.2483	770.7896	701.748	344.3916	329.5293	330.0603	1.170281	2.23E-27	APOL2
1312.081	1224.545	1294.903	2145.186	2241.639	2107.308	-0.76086	2.24E-27	RAB22A
254.3279	294.2475	287.4521	70.53804	62.96738	68.76257	2.046704	2.38E-27	PTK6
4872.672	5182.52	4696.875	3156.577	2697.103	2807.629	0.768079	2.40E-27	GPX4
548.198	598.4022	594.9801	246.8831	224.5837	224.2718	1.323317	2.46E-27	A4GALT
1574.497	1716.939	1679.085	2913.844	2747.477	2669.046	-0.74532	2.52E-27	STC2
431.3689	474.5607	480.9119	976.122	916.1754	964.7917	-1.0432	2.55E-27	PPP4R2
667.7232	631.0964	705.3982	1256.2	1246.754	1359.383	-0.94585	2.56E-27	CCNH
584.1454	652.8925	724.5617	1269.685	1368.491	1367.846	-1.03024	2.60E-27	SCFD1
3513.859	3603.293	3665.698	5281.017	5404.7	5448.111	-0.58137	2.61E-27	CUL4A
1895.327	2111.25	2090.643	1190.848	1017.973	1056.828	0.900467	2.64E-27	POLR2L
1531.36	1731.8	1733.838	2789.365	2805.197	2870.044	-0.7607	2.67E-27	TES
1147.621	1003.612	1040.303	488.5797	540.47	514.1324	1.048821	2.68E-27	-
696.4811	743.0491	820.3791	1379.641	1580.481	1522.297	-0.98795	2.76E-27	MTAP
843.8655	862.9277	782.0521	381.7353	283.3532	351.218	1.291806	3.08E-27	CDR2L
1357.015	1442.506	1345.093	2250.993	2362.326	2299.843	-0.73846	3.22E-27	THSD4
1249.173	1310.739	1312.241	2322.569	2319.299	2082.977	-0.79658	3.23E-27	FBXO21
177.9397	174.3689	161.5207	21.78381	22.03858	23.27348	2.93711	3.25E-27	SLC9A3
1738.057	1657.495	1822.355	2821.522	3162.012	3396.871	-0.84589	3.27E-27	PBX1
3940.735	3942.123	3790.717	2437.712	2203.858	1972.957	0.8194	3.28E-27	PLIN3
863.6366	876.798	857.7934	1609.927	1577.333	1473.635	-0.84326	3.28E-27	REPS1
879.8129	854.0111	902.5082	2432.525	1816.609	1747.627	-1.18562	3.38E-27	RPSAP15
536.5151	535.9861	620.5314	1080.892	1223.666	1225.032	-1.05943	3.56E-27	SLC16A7
549.0967	522.1159	617.7938	1104.75	1170.144	1138.285	-1.01425	3.62E-27	RBM27
289.3767	295.2382	297.4901	56.0155	86.05542	66.6468	2.079782	3.64E-27	IL20RB
390.0294	429.9778	492.775	914.9199	1087.237	1171.079	-1.27322	3.65E-27	NAA16
1141.33	1100.703	1142.508	607.8719	493.2445	501.4378	1.078465	3.69E-27	KRT10
1560.118	1442.506	1456.424	2519.66	2878.659	3259.346	-0.957	3.70E-27	RBM26
1622.127	1563.375	1704.636	2631.691	2878.659	2694.435	-0.74631	3.74E-27	SLC25A46
1153.013	1165.101	1233.762	1967.804	2009.709	2044.893	-0.76164	3.79E-27	SPART
1527.765	1559.412	1710.112	2785.215	2746.427	2601.341	-0.76149	3.91E-27	TOMM34
972.3775	1061.074	1173.534	1959.505	2110.457	1940.162	-0.90624	4.00E-27	TRPM7
691.089	755.9287	627.8318	288.3761	210.9407	189.3615	1.590189	4.03E-27	MIB2
1534.056	1511.857	1571.405	646.2529	710.4819	854.7716	1.062339	4.06E-27	SLFN5
996.642	1058.102	1054.903	1813.243	1744.196	1811.1	-0.78803	4.11E-27	EXOSC10
3761.897	3810.356	3903.873	5943.867	6537.064	5763.361	-0.66881	4.14E-27	CELF1
1596.964	1536.626	1565.017	851.6431	902.5325	900.2607	0.824037	4.29E-27	ZMYM3
938.2274	923.3624	869.6565	376.5487	261.3146	203.114	1.698821	4.40E-27	MCRIP2
2290.749	2351.007	2159.997	4711.526	4169.49	3701.542	-0.88762	4.93E-27	PSMD13
718.9482	728.1882	766.5388	1384.828	1352.749	1314.952	-0.87233	5.04E-27	VTI1B
1369.596	1282.007	1387.07	2677.334	2341.337	2282.917	-0.8541	5.11E-27	OSTC
1043.374	995.6858	977.337	515.5501	512.1347	467.5855	1.012396	5.19E-27	YPEL5
359.4741	370.5338	371.4063	123.4416	108.094	116.3674	1.662352	5.26E-27	MMP15
640.7626	597.4115	538.4023	231.3233	217.2375	242.2558	1.362823	5.29E-27	NME4
1701.211	1609.94	1645.321	913.8826	831.1694	702.4361	1.017899	5.40E-27	RHOT2
276.7951	278.3957	311.1783	642.1036	692.6412	680.2205	-1.21728	5.41E-27	SPATA5
159.966	161.4893	149.6576	17.63451	17.84076	15.86829	3.197351	5.49E-27	SPAG4
24129.7	24670.22	23595.71	34268	34150.36	36835.58	-0.53991	5.55E-27	HNRNPA1

940.9235	1038.287	970.0366	453.3106	332.6777	286.687	1.45847	5.59E-27	LYPLA2
228.2661	248.6738	223.5738	54.97818	31.48369	30.67868	2.578228	5.65E-27	CACNG6
1865.671	1872.484	1826.005	1016.578	799.6857	716.1886	1.135352	5.81E-27	TRIP6
113.2343	133.7488	132.3192	377.586	380.9527	445.3699	-1.66732	5.82E-27	SEC11C
3586.653	3558.71	3216.725	2158.672	2000.264	1911.599	0.771269	5.85E-27	SHISA5
1068.537	1008.565	916.1964	486.505	419.7825	367.0863	1.232766	6.14E-27	FASTK
789.9444	806.456	698.0979	343.3543	249.7706	295.1501	1.368626	6.17E-27	THRA
1098.193	1210.675	1236.5	1979.214	2200.71	2190.881	-0.84574	6.26E-27	MAN1A2
2031.927	2034.964	2178.248	3331.885	3714.026	3349.266	-0.73501	6.35E-27	BTBD1
142.891	152.5728	171.5587	408.7057	492.195	512.0167	-1.59688	6.43E-27	HCFC2
1554.726	1654.523	1510.264	812.2248	674.8004	555.39	1.207926	6.44E-27	CDC42EP1
363.9675	403.228	379.6192	807.0382	849.0102	792.3564	-1.09499	6.62E-27	POLR3C
1013.717	1091.787	1051.253	1770.712	1840.746	1821.679	-0.78371	6.68E-27	VEZF1
3368.272	3200.065	3439.387	1514.493	1742.098	1987.767	0.932503	6.71E-27	FAM46A
1438.795	1464.302	1470.112	798.7396	776.5977	848.4243	0.851471	6.80E-27	MOCOS
550.8941	574.6247	573.9916	1073.63	1131.314	1065.291	-0.94449	7.03E-27	CSNK1G1
14455.35	14131.8	13860.66	25449.71	23885.63	20824.48	-0.72496	7.09E-27	MT-ND4L
936.4301	828.2521	1024.789	1699.137	1855.439	1925.352	-0.97338	7.40E-27	TMEM106B
1169.19	1222.564	1107.831	570.5283	439.7222	377.6652	1.333733	7.41E-27	PKMYT1
634.4718	669.735	636.0447	295.6374	267.6114	262.3556	1.232239	7.46E-27	CUEDC2
939.1261	1003.612	995.5879	508.2888	482.7499	459.1224	1.018426	7.52E-27	STIM1
617.3968	528.0603	594.9801	1145.206	1127.116	1178.485	-0.98646	7.77E-27	NEPRO
2937.802	3243.657	2983.114	4723.974	4856.884	4753.08	-0.64561	8.32E-27	CDK12
1038.88	1024.417	1082.28	1821.541	1814.51	1751.859	-0.77624	8.34E-27	BAG5
476.3032	447.8109	514.6761	947.0769	988.5879	1064.233	-1.0593	8.56E-27	RUFY2
405.3071	436.9129	426.1591	120.3296	123.8358	159.7407	1.651155	8.60E-27	ARRB1
472.7085	432.95	432.5469	157.6733	157.4185	163.9723	1.482371	9.27E-27	ASIC1
1048.766	1111.602	1079.542	1854.736	1861.736	1798.406	-0.76771	9.46E-27	TBL1X
186.9265	201.1186	155.1329	16.59719	27.28586	24.33137	2.993741	9.62E-27	KIAA0513
227.3674	207.063	233.6118	576.7522	549.9151	537.4059	-1.31575	9.72E-27	PCLAF
299.2622	310.0992	355.893	728.2015	735.6689	729.9411	-1.18417	9.83E-27	BORA
786.3496	736.114	740.075	332.981	299.0951	228.5033	1.394247	9.89E-27	NUMBL
2394.098	2442.155	2529.578	3692.874	3992.132	3938.508	-0.65809	1.04E-26	ASAP1
606.6126	615.2447	621.444	213.6888	164.7646	101.557	1.940325	1.11E-26	FAM109A
307.3504	337.8397	333.0794	704.3431	710.4819	762.7356	-1.15492	1.14E-26	RXYLT1
1833.318	1818.984	1865.244	2985.419	2975.209	2818.207	-0.66996	1.14E-26	ETS1
246.2398	260.5626	241.8247	65.35142	46.17608	47.60486	2.232876	1.18E-26	MBOAT7
333.4122	327.9324	309.3532	99.58312	94.45107	81.4572	1.816547	1.19E-26	KLC4
319.932	353.6914	328.5166	101.6578	101.7973	92.03605	1.761228	1.24E-26	HS1BP3
673.1153	663.7906	660.6835	1288.357	1180.638	1357.267	-0.9376	1.26E-26	UPF3B
336.1083	336.8489	307.5281	98.54579	97.59944	88.8624	1.782104	1.35E-26	PCED1A
911.2669	848.0667	931.7097	1551.837	1751.543	1877.747	-0.94459	1.56E-26	ZFC3H1
1552.029	1527.709	1565.017	2436.674	2524.992	2666.93	-0.71566	1.61E-26	SRRM1
275.8964	291.2753	295.665	70.53804	71.36303	83.57297	1.936355	1.63E-26	BTN3A3
2486.662	2638.32	2469.35	1408.686	1575.234	1512.777	0.755932	1.64E-26	NCOA3
3148.993	3179.26	3165.623	1996.849	1983.472	1759.264	0.725943	1.64E-26	ANKRD52
906.7734	956.0566	942.6602	1835.026	1626.657	1632.318	-0.86086	1.74E-26	PLRG1
1178.176	1221.573	1134.295	560.155	400.8923	344.8707	1.435744	1.77E-26	RAB5C
6202.726	6897.477	6984.629	10630.5	10654.08	10028.76	-0.64077	1.79E-26	SNRNP200
1640.101	1639.662	1641.671	2547.668	2629.938	2685.972	-0.67607	1.82E-26	YES1
815.1075	735.1233	850.4931	1432.545	1640.3	1542.397	-0.94222	1.90E-26	KATNAL1
683.8995	736.114	800.303	1479.224	1536.404	1338.225	-0.97165	1.99E-26	GTF2F2
3871.536	3974.817	4094.595	5816.276	6278.897	6462.624	-0.63609	2.02E-26	TOP2B
4318.183	4305.722	4372.921	6246.766	6272.601	6352.603	-0.53805	2.02E-26	UBE2D3

1826.128	1859.604	1926.385	2929.403	3421.228	3498.428	-0.81137	2.33E-26	ROCK2
1016.413	1030.361	898.858	482.3557	476.4532	469.7012	1.043771	2.34E-26	NINJ1
1150.317	1213.647	1246.538	1983.364	2044.341	2051.24	-0.75173	2.41E-26	ASXL2
180.6357	133.7488	164.2583	17.63451	11.54402	17.98406	3.344207	2.44E-26	SCN3A
137.4988	137.7118	159.6956	13.48521	13.64293	9.520971	3.568093	2.44E-26	NTNG2
989.4525	1088.815	1102.356	1802.869	1866.983	1967.667	-0.826	2.49E-26	RBM28
929.2406	948.1307	1034.827	1706.398	1727.405	1689.443	-0.81482	2.51E-26	FEZ2
290.2753	308.1177	253.6878	78.83663	55.62119	64.53103	2.097284	2.60E-26	ENDOG
2662.804	2862.225	2763.19	1591.255	1756.79	1665.112	0.725209	2.69E-26	CDC42BPB
253.4292	256.5996	239.9997	65.35142	36.73097	41.25754	2.386132	2.70E-26	RHEBL1
2248.511	2419.368	2177.335	3692.874	3944.906	3583.059	-0.71333	2.72E-26	GTF3C4
470.0124	413.1353	468.1362	909.7333	915.1259	971.139	-1.04799	2.91E-26	KIAA0586
289.3767	201.1186	187.9845	19.70916	10.49456	37.026	3.337274	2.92E-26	CDKN1C
793.5391	723.2345	708.1359	295.6374	353.6668	287.7449	1.247759	2.97E-26	CPNE7
982.263	922.3717	838.63	441.9001	377.8043	332.1761	1.251582	3.08E-26	VEGFB
1370.495	1298.85	1432.698	2233.359	2591.108	2687.03	-0.8724	3.26E-26	ARHGAP5
436.761	423.0426	363.1934	140.0388	131.182	126.9463	1.618622	3.47E-26	RNASET2
452.9374	418.089	475.4366	894.1734	940.3129	919.3027	-1.03142	3.61E-26	BLZF1
610.2073	510.2271	565.7787	234.4353	191.0011	178.7827	1.480781	3.94E-26	HIST1H2AC
1200.644	1229.499	1215.512	1956.393	2115.704	2034.314	-0.74423	3.99E-26	RNF6
3988.365	4142.251	4189.5	2614.057	2648.828	2334.754	0.69729	4.33E-26	COL4A1
757.5917	771.7804	713.6111	356.8395	280.2048	264.4714	1.314417	4.39E-26	ATG101
598.5244	593.4486	689.8849	1196.035	1313.919	1215.511	-0.98483	4.60E-26	STK38L
509.5546	570.6617	469.0488	1064.295	1156.501	1025.091	-1.06804	4.68E-26	FOXRED2
158.1686	281.3679	178.8591	28.00775	9.445107	14.8104	3.561121	4.70E-26	-
241.7463	234.8035	268.2886	606.8346	631.7727	564.911	-1.27523	4.92E-26	METTL8
2696.056	2779.004	2758.627	4067.348	4163.193	4149.028	-0.58843	5.31E-26	HACD3
1221.313	1348.386	1241.975	2105.768	2132.495	2185.592	-0.75352	5.47E-26	CDH13
706.3666	759.8916	680.7595	320.5332	304.3423	243.3137	1.305554	5.48E-26	ARL2
313.6412	382.4226	310.2657	84.02326	66.11575	33.85234	2.449455	5.74E-26	OSGIN1
895.0905	891.659	868.744	452.2733	355.7657	348.0444	1.199349	5.84E-26	SLC9A3R2
479.8979	467.6256	495.5126	927.3678	951.8569	954.2129	-0.9731	5.88E-26	CNST
797.1338	620.1983	594.9801	267.6296	218.2869	224.2718	1.502789	5.97E-26	NRBP2
1203.34	1303.804	1214.599	664.9248	605.5363	519.4219	1.055602	6.13E-26	BET1L
1684.136	1548.514	1660.834	941.8903	892.0379	803.9931	0.891569	6.14E-26	TAOK2
554.4888	548.8656	536.5772	235.4726	222.4847	213.6929	1.287665	6.18E-26	PFKFB3
1503.5	1495.015	1302.203	751.0227	654.8608	559.6215	1.129312	6.37E-26	R3HDM4
244.4424	218.9518	255.5129	539.4086	695.7896	679.1626	-1.41184	6.44E-26	CCDC18
4457.479	4787.218	4780.83	7096.334	6806.774	7107.934	-0.58321	6.56E-26	HNRNPF
962.4919	1020.454	956.3484	1775.899	1656.042	1678.865	-0.79852	6.98E-26	MRPL20
5031.739	5399.49	5333.833	8358.758	8175.265	7597.735	-0.61433	7.14E-26	CCT4
1419.923	1604.986	1546.766	865.1283	802.8341	755.3304	0.915295	7.25E-26	CELSR1
1334.548	1404.858	1502.051	2319.457	2810.444	2671.161	-0.87907	7.27E-26	LNPEP
6725.761	6371.399	6970.941	9712.466	10809.4	10469.89	-0.62686	7.32E-26	ACTR2
193.2173	187.2484	237.262	540.4459	555.1624	526.8271	-1.39211	7.38E-26	ANKRD1
620.0928	547.8749	515.5886	228.2113	207.7924	175.609	1.46066	7.94E-26	FBXL16
246.2398	298.2104	292.9273	645.2156	685.295	648.4839	-1.2419	8.11E-26	C21orf91
1010.122	1012.528	979.1621	1780.048	1649.745	1873.516	-0.82112	8.16E-26	TLK2
401.7123	430.9685	372.3189	143.1507	96.54998	118.4832	1.749424	8.32E-26	ARHGEF10L
3069.909	3223.843	3091.707	2037.305	2004.462	1960.262	0.644788	8.48E-26	PGM1
12824.24	13664.18	12930.78	9285.088	9025.325	8789.972	0.540503	8.65E-26	SPARC
784.5522	664.7813	616.8812	296.6747	240.3255	251.7768	1.389336	8.86E-26	GGT1
336.1083	342.7933	363.1934	979.234	778.6966	755.3304	-1.27017	8.87E-26	POLR3K
1706.603	1770.438	1906.309	2840.193	3287.947	3242.42	-0.79955	9.72E-26	USP34

307.3504	282.3587	333.0794	716.791	692.6412	674.9311	-1.17458	1.11E-25	NMT2
6287.202	5805.691	6132.311	3850.547	2982.555	3172.599	0.865111	1.15E-25	FOS
620.9915	660.8184	657.0333	294.6001	283.3532	282.4555	1.171811	1.23E-25	GLP2R
539.2112	502.3012	532.927	990.6446	1141.809	1287.447	-1.11858	1.28E-25	ATG2B
1858.481	1920.039	1962.887	3012.389	2944.775	3012.858	-0.64377	1.28E-25	FAM20B
1819.838	1876.447	1773.077	1110.974	1034.764	983.8337	0.805127	1.28E-25	RNF167
505.0611	536.9768	537.4897	220.95	189.9516	158.6829	1.470826	1.32E-25	NPTXR
1481.932	1517.802	1498.401	877.5762	795.4879	861.1189	0.827684	1.38E-25	UBAP1
304.6543	287.3123	310.2657	88.17255	87.10488	65.58891	1.905008	1.38E-25	FAM174B
9297.798	9471.4	9524.245	13908.44	14021.79	13011.99	-0.53315	1.38E-25	BZW1
457.4308	464.6534	489.1248	911.8079	916.1754	985.9494	-0.99551	1.51E-25	PPID
906.7734	913.4551	889.7326	1507.232	1654.993	1586.829	-0.80942	1.58E-25	PIGK
1878.252	1992.362	1800.454	2965.71	3133.677	3038.248	-0.68852	1.67E-25	SYPL1
125.8159	140.684	166.996	394.1832	478.5521	454.8908	-1.61461	1.69E-25	INPP4B
1806.357	1929.946	2060.529	3065.293	3469.503	3325.993	-0.76645	1.70E-25	MED1
1192.555	1328.572	1212.774	2230.247	2104.16	2066.051	-0.77808	1.70E-25	DCTD
3282.897	3163.408	3074.368	2079.835	1929.95	1978.246	0.668975	1.71E-25	ATN1
1457.668	1407.83	1395.283	2263.441	2476.717	2674.335	-0.79907	1.72E-25	SLTM
256.1253	303.164	318.4786	64.3141	78.70923	79.34143	1.981001	1.78E-25	A2M
2173.021	2506.552	2181.898	1229.229	950.8074	831.4981	1.187621	1.78E-25	PKN1
858.2444	772.7711	790.265	1402.462	1550.047	1481.04	-0.87217	1.80E-25	EIF5A2
485.2901	500.3197	451.7104	157.6733	185.7538	188.3037	1.434658	1.86E-25	CXXC5
133.0054	155.545	125.0188	12.44789	8.395651	10.57886	3.716714	1.91E-25	DOK7
416.99	370.5338	422.5089	812.2248	873.1477	851.598	-1.06702	1.92E-25	NKAP
238.1516	245.7016	262.8133	614.0959	601.3385	554.3321	-1.24505	2.04E-25	MYCBP
1210.529	1231.48	1236.5	2005.148	2118.852	1975.073	-0.72953	2.05E-25	CNOT9
143.7896	155.545	156.0454	20.74648	16.7913	13.75251	3.148816	2.06E-25	CLDN7
1880.948	1931.928	1794.978	1134.833	973.8955	970.0812	0.864782	2.09E-25	NXN
1694.022	1599.042	1642.583	2548.705	2764.268	2675.393	-0.6944	2.12E-25	PGRMC2
477.2019	488.431	588.5923	1058.071	1059.951	1113.954	-1.05579	2.38E-25	USP38
779.1601	776.734	747.3754	384.8473	354.7162	345.9286	1.085096	2.47E-25	NFIL3
2013.055	1893.289	2025.853	3039.36	3118.984	3222.32	-0.6608	2.49E-25	ATG12
1079.321	1172.036	1218.249	1942.908	2105.209	1978.246	-0.79669	2.51E-25	ATL2
1516.981	1690.189	1451.861	784.217	774.4988	592.416	1.11445	2.71E-25	CIC
224.6713	234.8035	186.1594	47.71691	36.73097	32.79446	2.459765	2.77E-25	CBX7
279.4911	343.7841	314.8284	663.8874	857.4058	847.3664	-1.33722	2.77E-25	MPHOSPH6
296.5661	238.7665	276.5015	66.38874	67.16521	68.76257	2.005542	2.78E-25	MAST3
453.8361	510.2271	475.4366	986.4953	960.2526	920.3605	-0.99489	2.94E-25	RAD51C
753.0983	828.2521	808.5159	1410.761	1571.036	1416.509	-0.88044	3.04E-25	LACTB
1764.119	1883.382	2010.339	3129.607	3155.715	2993.816	-0.71382	3.07E-25	SMU1
2811.986	3076.223	2932.011	1794.571	1587.827	1408.046	0.880377	3.16E-25	SBF1
450.2413	456.7275	427.9842	850.6058	914.0765	985.9494	-1.04312	3.20E-25	CENPJ
1052.36	1021.445	1102.356	1919.05	1728.455	1885.152	-0.80044	3.20E-25	PDZD8
257.024	305.1455	304.7904	85.06058	61.91792	69.82045	1.998641	3.20E-25	LINC00942
2125.391	2288.591	2295.054	3508.23	3656.306	3422.26	-0.65828	3.22E-25	ATL3
647.9521	675.6793	712.6986	1219.893	1272.991	1411.219	-0.93896	3.23E-25	NOP14
281.2885	273.4421	250.9502	73.65001	51.42336	37.026	2.311846	3.25E-25	TRADD
422.3821	409.1724	450.7978	828.822	887.8401	938.3446	-1.04937	3.28E-25	CCDC91
1544.84	1596.07	1690.948	2633.766	2644.63	2541.041	-0.6945	3.33E-25	BNIP2
2515.42	2546.182	2672.848	1582.957	1656.042	1562.497	0.687847	3.37E-25	SOD2
1118.863	1123.49	1118.782	1916.975	1872.23	1804.753	-0.73499	3.49E-25	FAM96A
2447.12	2530.33	2539.616	3799.718	3727.669	3817.909	-0.59394	3.51E-25	SRSF7
673.1153	695.494	772.0141	1274.871	1415.717	1363.615	-0.92121	3.61E-25	MAPK8
594.031	609.3003	611.406	282.1522	244.5233	245.4295	1.232564	3.62E-25	RNF24

1064.043	1067.019	1089.58	551.8564	469.107	386.1283	1.194286	3.64E-25	VPS51
1296.803	1257.239	1335.968	2086.059	2119.902	2248.007	-0.73013	3.68E-25	NEK2
2199.083	2233.11	2167.297	1346.447	1270.892	1390.062	0.719634	3.69E-25	ADGRE5
1743.449	1830.873	1650.796	2730.237	2883.906	2862.639	-0.69835	3.72E-25	ANKRD33B
174.3449	177.3411	210.7982	459.5346	568.8053	522.5955	-1.46263	3.75E-25	LACTB2
9699.51	9539.76	10110.1	13688.53	14009.19	13873.11	-0.50219	3.85E-25	RAD21
549.0967	579.5783	571.2539	1077.78	1092.484	1032.496	-0.91419	3.94E-25	SIRT1
533.8191	631.0964	555.7406	241.6965	215.1386	181.9563	1.428511	3.96E-25	TPRN
517.6427	540.9398	552.0905	210.5768	235.0782	211.5771	1.29312	4.09E-25	HCN2
111.437	117.8971	93.07971	347.5036	358.9141	329.0024	-1.68469	4.19E-25	TMEM156
3938.938	4083.798	3982.352	2687.707	2666.669	2667.988	0.581441	4.42E-25	DNM2
599.4231	542.9212	624.1816	1146.243	1101.929	1135.111	-0.9367	4.49E-25	NFYB
1251.869	1283.989	1346.006	2071.536	2436.838	2443.716	-0.84061	4.56E-25	ASH1L
3669.332	3716.236	3521.516	2065.312	1703.268	1379.483	1.082993	4.59E-25	GPC1
8786.446	8991.885	8087.897	5704.245	5596.751	5839.529	0.593592	4.65E-25	CD63
183.3318	208.0538	210.7982	490.6543	519.4809	504.6115	-1.3315	4.65E-25	CIPC
529.3256	538.9583	495.5126	1009.316	1048.407	972.1969	-0.95459	4.87E-25	LEMD3
380.1439	397.2836	365.0185	854.7551	767.1526	782.8354	-1.07435	5.01E-25	LANCL2
3348.501	3654.811	3577.181	5820.426	5518.041	5239.708	-0.64808	5.01E-25	SLC7A1
385.536	307.127	297.4901	90.2472	97.59944	82.51508	1.873207	5.22E-25	FOXF1
701.8732	750.975	907.0709	1498.933	1639.251	1767.727	-1.05556	5.53E-25	DIAPH3
945.4169	984.7878	992.8503	525.9233	453.3651	429.5016	1.052657	5.69E-25	MAN2B1
1570.003	1451.423	1550.416	881.7255	839.5651	752.1567	0.88635	5.83E-25	MZT1
969.6814	1083.861	1060.379	2007.222	1983.472	1722.238	-0.87595	6.06E-25	UTP18
2705.043	2666.06	2765.015	4160.707	3996.33	4053.818	-0.58567	6.29E-25	SYAP1
736.9219	746.0213	758.3259	375.5113	344.2217	327.9446	1.096896	6.41E-25	DTX4
502.3651	468.6163	481.8244	173.2331	201.4956	188.3037	1.367999	6.67E-25	BCL2L11
384.6373	322.9787	322.1288	82.98593	111.2424	100.4991	1.805709	6.69E-25	HEY1
2128.985	2117.195	1960.149	1209.52	1013.775	1192.237	0.861532	6.90E-25	NDUFB10
1508.893	1544.551	1322.279	708.4924	600.289	449.6014	1.314851	6.91E-25	ESRRA
139.2962	155.545	135.9694	8.298593	17.84076	13.75251	3.43388	6.92E-25	COX6B2
3834.69	4102.622	3976.876	5800.717	5907.39	5825.776	-0.55763	7.38E-25	CDCP1
514.9467	553.8193	578.5543	1081.929	1041.061	1036.728	-0.93992	7.58E-25	CCZ1
483.4927	524.0973	470.8738	928.4051	989.6373	1066.349	-1.01392	7.81E-25	CHCHD7
669.5205	653.8832	743.7252	1229.229	1316.018	1373.136	-0.92214	7.81E-25	C2CD5
1266.248	1262.193	1401.671	2320.494	2219.6	2135.871	-0.76424	7.90E-25	GMNN
399.0163	400.2558	349.5052	137.9641	89.20379	95.20971	1.83234	8.18E-25	C16orf74
605.7139	607.3188	557.5657	1062.22	1160.699	1146.748	-0.92855	8.19E-25	POLR1E
1358.812	1552.477	1335.055	755.172	632.8222	556.4479	1.126268	8.20E-25	PIN1
2199.982	2088.463	2142.659	3344.333	4038.308	4267.511	-0.85704	8.72E-25	GOLGA4
313.6412	302.1733	291.1022	681.522	628.6244	729.9411	-1.16945	9.05E-25	NUP35
363.0689	395.3021	302.0528	57.05283	99.69835	95.20971	2.073717	9.17E-25	-
9109.074	8982.969	9102.649	6498.836	6454.157	6253.162	0.501764	9.62E-25	TSC22D1
2116.404	2150.88	2084.256	3185.622	3215.534	3318.587	-0.6139	1.02E-24	PARVA
410.6992	424.0334	415.2085	834.0086	828.0211	824.0929	-0.9923	1.05E-24	EMC2
1308.486	1305.785	1288.515	753.0973	611.833	631.5577	0.966812	1.07E-24	LSM7
153.6752	118.8879	124.1063	8.298593	2.098913	6.347314	4.565077	1.08E-24	DEPP1
689.2916	722.2438	768.3639	1296.655	1523.811	1351.978	-0.93662	1.11E-24	CASP3
1557.422	1719.911	1752.089	2674.222	2894.401	2765.313	-0.72882	1.13E-24	ELP1
478.9993	435.9222	480.9119	979.234	879.4444	951.0392	-1.00859	1.13E-24	CCDC34
8697.476	8893.803	8364.399	5662.752	5058.38	4466.393	0.773079	1.21E-24	9-Sep
584.1454	571.6525	641.52	1075.705	1274.04	1293.794	-1.01904	1.25E-24	SLC36A4
345.9938	331.8953	322.1288	783.1797	664.3059	793.4143	-1.16393	1.31E-24	TATDN1
183.3318	204.0908	183.4218	35.26902	35.68152	34.91023	2.430357	1.33E-24	EXD3

4141.142	4237.362	4481.514	6276.848	6315.628	6373.761	-0.56051	1.38E-24	AZIN1
9065.038	9536.788	9184.778	6591.158	6459.404	6292.304	0.522496	1.43E-24	DAG1
459.2282	569.671	454.448	1217.819	1165.946	977.4864	-1.18148	1.44E-24	WDR77
1096.396	1105.657	1227.375	1898.303	2062.182	1964.494	-0.78863	1.44E-24	CEP350
463.7216	453.7553	495.5126	922.1812	902.5325	938.3446	-0.96711	1.49E-24	OTUD6B-AS1
2562.152	2770.087	2849.882	4105.729	4401.42	4432.541	-0.66134	1.49E-24	ADNP
777.3628	880.7609	867.8315	1490.635	1614.064	1482.098	-0.86115	1.49E-24	DPY19L4
25163.19	26720.05	24941.71	37376.86	35456.93	36254.8	-0.50589	1.52E-24	RPS4X
228.2661	193.1928	219.0111	39.41832	37.78043	50.77851	2.324537	1.52E-24	CORO2A
1321.067	1438.543	1334.143	2175.269	2308.804	2256.47	-0.71986	1.52E-24	SWAP70
308.2491	320.0065	339.4672	653.5142	770.301	823.0351	-1.21508	1.55E-24	ZNF33B
1358.812	1366.22	1454.599	2183.567	2446.283	2400.343	-0.75003	1.59E-24	KPNA1
884.3063	950.1122	872.3942	447.0867	421.8815	471.817	1.013217	1.63E-24	PEX19
599.4231	632.0871	593.155	1173.214	1160.699	1075.87	-0.90255	1.68E-24	BTG3
390.9281	394.3114	365.931	139.0014	95.50053	81.4572	1.864222	1.70E-24	PIDD1
2146.959	2245.99	2173.685	3532.089	3540.866	3245.593	-0.65222	1.75E-24	PTPN1
2879.388	2578.876	2984.939	4367.135	4943.989	5071.504	-0.76814	1.78E-24	SRSF10
257.024	283.3494	286.5395	80.91128	58.76956	44.4312	2.165704	1.79E-24	CLIP2
332.5136	363.5987	354.0679	771.7692	722.026	721.478	-1.0775	1.81E-24	MIPEP
629.0797	624.1613	567.6037	274.8909	205.6934	239.0822	1.338823	1.84E-24	MUS81
459.2282	386.3855	375.969	818.4487	965.4998	1032.496	-1.20435	1.92E-24	CEP290
692.8864	780.697	836.8049	1414.91	1569.987	1430.261	-0.93456	1.99E-24	SLC35D1
391.8268	427.0056	389.6572	785.2544	841.664	915.0711	-1.07334	2.08E-24	PAPD5
1148.52	1094.759	1194.523	1896.229	1889.021	1928.526	-0.73257	2.08E-24	CDC73
19811.52	21381.98	19593.28	37461.92	32143.8	30627.91	-0.72158	2.16E-24	RPSA
437.6597	434.9314	375.0565	155.5986	142.7261	138.583	1.513344	2.19E-24	ADGRB2
1064.942	1168.073	1064.029	575.7149	579.2999	591.3581	0.916412	2.27E-24	BTN3A2
517.6427	444.8387	379.6192	149.3747	121.7369	85.68874	1.910664	2.28E-24	ZNF579
271.403	246.6923	232.6993	59.12748	54.57173	68.76257	2.041339	2.51E-24	CEACAM1
1042.475	1164.11	925.3219	324.6825	442.8706	471.817	1.337411	2.51E-24	RPL26
247.1385	228.8591	208.0605	54.97818	51.42336	47.60486	2.150864	2.53E-24	ATP7B
1562.814	1531.672	1627.07	2823.596	2552.278	2487.089	-0.73572	2.53E-24	DR1
1737.159	1725.855	1792.241	2690.819	2755.872	2958.906	-0.67745	2.58E-24	SNHG1
1013.717	1151.231	1071.329	537.3339	420.832	351.218	1.304814	2.63E-24	ARHGAP45
292.0727	282.3587	341.2923	666.9994	682.1466	739.4621	-1.18871	2.67E-24	SPRTN
8177.137	8763.026	8240.292	12717.59	12054.06	11931.89	-0.54373	2.69E-24	EZR
4520.387	4512.785	4431.324	3002.016	2977.308	3119.705	0.565385	2.74E-24	SERPINB1
1087.409	1129.435	1132.47	1782.123	2041.193	1958.146	-0.78763	2.89E-24	PCNX4
1081.118	1040.269	1081.367	141.0761	81.85759	59.2416	3.504144	2.89E-24	SAMD11
1903.415	1848.706	1962.887	2906.582	3125.281	2962.08	-0.65399	2.95E-24	CNIH1
1645.493	1650.56	1619.77	924.2558	935.0656	1012.397	0.775582	2.97E-24	IP6K2
564.3744	607.3188	606.8432	1252.05	1157.55	1058.944	-0.96406	3.03E-24	PLEK2
575.1586	608.3096	585.8547	1189.811	1049.456	1115.012	-0.92323	3.09E-24	BRAP
1079.321	1014.51	1047.603	1734.406	1753.642	1929.583	-0.78588	3.12E-24	MBD4
859.1431	971.9083	891.5577	466.7959	406.1396	362.8548	1.138845	3.16E-24	IRF3
505.9598	523.1066	494.6001	221.9874	172.1108	159.7407	1.459278	3.25E-24	SERTAD1
4222.922	4497.924	4184.024	2756.17	2464.124	2192.997	0.799532	3.42E-24	PTPA
462.8229	475.5514	450.7978	983.3833	851.1091	1001.818	-1.03007	3.44E-24	NKRF
312.7425	348.7377	370.4938	709.5297	753.5097	779.6617	-1.1201	3.50E-24	PARPBP
256.1253	259.5718	208.9731	63.27677	49.32445	45.48908	2.195408	3.52E-24	PPP2R5B
1358.812	1439.534	1356.044	768.6572	675.8499	569.1425	1.044393	3.54E-24	MOGS
433.1663	451.7739	428.8967	939.8157	856.3564	841.0191	-1.00569	3.69E-24	TMEM41A
505.0611	504.2827	531.1019	930.4798	1035.813	1093.854	-0.98998	3.78E-24	KRIT1
164.4594	147.6191	184.3343	432.5642	442.8706	459.1224	-1.42519	3.83E-24	RSBN1

2385.111	2314.35	2444.711	3597.44	3579.696	3586.232	-0.59111	3.85E-24	MRPS27
3406.916	3398.211	3215.813	2156.597	2027.55	1817.448	0.739454	3.87E-24	TNFAIP2
526.6296	539.949	572.1665	988.5699	1066.248	1063.175	-0.92791	4.01E-24	RC3H1
370.2583	396.2929	391.4823	765.5452	816.477	776.4881	-1.02659	4.18E-24	XPO4
281.2885	329.9138	331.2543	699.1565	724.1249	668.5837	-1.15114	4.26E-24	ZCCHC10
476.3032	573.6339	544.7901	1005.167	1095.632	1090.68	-1.00178	4.27E-24	TMEM192
726.1377	659.8276	662.5086	1268.647	1213.172	1243.016	-0.8622	4.31E-24	TMEM38B
4958.047	5347.972	5022.654	3376.49	3407.585	3474.097	0.57931	4.35E-24	CHD3
249.8345	333.8767	282.8893	660.7755	704.1852	693.973	-1.25039	4.43E-24	TEX10
34797.99	35839.74	34152.95	24377.12	21633.49	20124.16	0.664006	4.46E-24	KRT8
202.2042	176.3503	193.4598	41.49297	28.33532	32.79446	2.478518	4.50E-24	RTN4RL2
214.7858	244.7108	248.2126	537.3339	614.9814	556.4479	-1.27236	4.53E-24	GTF2E1
125.8159	154.5542	125.9314	405.5937	365.2108	460.1803	-1.6012	4.54E-24	CCDC66
253.4292	262.544	252.7753	62.23945	32.53315	63.47314	2.28011	4.58E-24	LINC02577
195.9134	235.7943	212.6233	41.49297	50.3739	28.56291	2.418305	4.62E-24	KNDC1
1352.521	1317.674	1339.618	2267.591	2112.556	2124.234	-0.6978	4.66E-24	GORASP2
828.5878	858.9648	895.2078	1455.366	1535.355	1482.098	-0.79229	4.82E-24	DONSON
3122.033	3238.703	3044.254	1933.572	1642.399	1451.419	0.903399	4.86E-24	GNB2
1239.287	1277.054	1225.55	1971.953	2062.182	2029.025	-0.69649	4.94E-24	SS18
416.0913	436.9129	410.6458	841.2699	811.2298	854.7716	-0.98898	4.97E-24	RAVER2
3483.304	3499.266	3272.391	2023.819	1689.625	1417.567	0.998831	5.02E-24	MYDGF
271.403	267.4977	275.589	579.8642	613.932	614.6316	-1.15051	5.10E-24	C1orf131
361.2715	343.7841	300.2277	99.58312	101.7973	110.0201	1.690873	5.12E-24	GMIP
3868.84	3922.309	4146.61	5837.023	6649.355	6149.489	-0.6425	5.17E-24	TMEM30A
1433.403	1487.089	1294.903	734.4255	633.8716	506.7272	1.168317	5.52E-24	MZT2A
746.8075	837.1687	825.8543	371.362	405.0901	338.5234	1.111496	5.61E-24	ATP9A
1442.39	1463.311	1436.348	781.1051	794.4385	878.0451	0.823549	5.61E-24	PDIA5
2858.718	3103.964	3151.022	4539.33	4776.076	4656.813	-0.61659	5.63E-24	PAFAH1B1
556.2862	684.5959	610.4934	1141.057	1387.381	1304.373	-1.05068	5.65E-24	UHRF1BP1L
866.3326	911.4736	864.1813	1446.03	1643.449	1611.16	-0.83147	6.05E-24	ANKRD40
1108.978	982.8063	1091.405	1805.981	1792.471	1835.432	-0.77084	6.16E-24	MFF
245.3411	233.8128	255.5129	69.50072	54.57173	57.12583	2.019324	6.25E-24	ARHGAP33
5864.82	6206.937	5924.25	4146.185	3832.615	3642.3	0.630784	6.57E-24	AMOTL2
2030.13	1980.474	2167.297	3166.951	3589.141	3317.529	-0.70519	6.69E-24	ITGAV
768.3759	847.076	775.6643	409.743	356.8152	348.0444	1.100491	6.78E-24	RCCD1
531.123	541.9305	501.9004	232.3606	199.3967	214.7508	1.284192	6.90E-24	CDC42EP2
1541.245	1539.598	1532.165	942.9276	818.5759	831.4981	0.830928	6.97E-24	POLG
300.1609	242.7294	298.4026	690.8579	649.6135	626.2683	-1.22351	7.26E-24	CENPQ
809.7154	836.178	814.9038	332.981	375.7054	426.3279	1.116624	7.38E-24	EPHX1
553.5901	522.1159	537.4897	962.6368	1059.951	1051.538	-0.92981	7.44E-24	EDRF1
293.8701	286.3216	262.8133	84.02326	48.27499	42.31543	2.269881	7.44E-24	JOSD2
695.5824	663.7906	640.6074	284.2268	162.6657	170.3196	1.695546	7.51E-24	LAGE3
13432.65	14945.19	13500.21	8973.891	7584.421	6779.989	0.843418	8.13E-24	FAM129B
937.3287	1048.195	918.0215	423.2282	406.1396	267.6451	1.403606	8.41E-24	REX1BD
13858.63	14248.71	13947.36	9992.543	9533.261	8811.13	0.569534	8.57E-24	LMNA
337.9057	313.0714	333.9919	115.143	100.7478	96.2676	1.657785	8.58E-24	ZSWIM4
321.7293	283.3494	309.3532	74.68734	102.8467	77.22565	1.844379	9.29E-24	PSG4
1009.224	1069.991	1014.751	530.0726	553.0635	565.9688	0.907589	9.32E-24	DLG3
754.8956	812.4004	755.5883	375.5113	383.0516	379.781	1.028618	9.45E-24	DECR1
268.7069	238.7665	279.2391	563.267	650.6629	632.6156	-1.22969	9.52E-24	ZNF714
453.8361	456.7275	442.5849	892.0988	855.3069	869.582	-0.95175	9.66E-24	LARP1B
3012.393	3159.445	3025.091	5346.369	4871.576	4553.14	-0.68372	9.82E-24	PRMT1
1285.12	1279.035	1213.686	2099.544	1982.423	2201.46	-0.73405	9.97E-24	SNRPA1
328.9188	336.8489	325.779	119.2923	83.95651	92.03605	1.746853	9.99E-24	LSR

1665.264	1742.698	1805.016	3143.092	2939.527	2680.682	-0.74949	1.00E-23	FEN1
503.2638	422.0519	483.6495	977.1593	980.1922	901.3186	-1.01961	1.01E-23	BMI1
513.1493	532.0232	495.5126	211.6141	176.3087	221.0981	1.338811	1.02E-23	ANKZF1
150.9791	142.6654	109.5055	8.298593	14.69239	7.4052	3.729126	1.04E-23	LGALS9
1765.917	1689.198	1763.952	2659.699	3181.952	3041.421	-0.76705	1.07E-23	ITGA2
789.0457	746.0213	817.6414	404.5564	336.8755	325.8288	1.140376	1.10E-23	MAP1S
1114.37	1127.453	1043.04	604.76	555.1624	491.9168	0.991379	1.11E-23	RHBDD2
807.0194	757.9101	734.5997	381.7353	374.6559	348.0444	1.058035	1.11E-23	LDB1
617.3968	552.8286	584.0296	223.0247	260.2652	259.182	1.241127	1.13E-23	CD24
1311.182	1313.711	1358.781	2124.44	2096.814	2291.38	-0.70903	1.13E-23	MAP3K7
2933.309	3160.436	3078.018	1771.75	1914.208	1996.23	0.690689	1.15E-23	MME
1189.859	1174.018	1181.747	660.7755	526.8271	488.7432	1.080436	1.17E-23	ECI1
168.9528	153.5635	156.9579	26.97043	24.1375	21.15771	2.729721	1.17E-23	RAET1G
408.9018	504.2827	496.4251	179.4571	160.5668	124.8305	1.599432	1.26E-23	-
236.3542	270.4699	229.9616	64.3141	54.57173	63.47314	2.013655	1.27E-23	PTPRH
541.9072	622.1798	602.2805	1071.556	1123.968	1146.748	-0.92069	1.30E-23	AP4E1
42275.05	43345.52	42487.24	57320.46	56872.14	57741.52	-0.42451	1.32E-23	LDHA
1036.184	1167.083	1115.131	1933.572	1861.736	1824.853	-0.76069	1.34E-23	NFYA
437.6597	464.6534	426.1591	853.7178	918.2743	851.598	-0.98235	1.39E-23	ATE1
227.3674	196.165	239.9997	51.86621	49.32445	32.79446	2.307847	1.39E-23	PHLDB3
549.9954	477.5329	636.9573	1108.9	1169.094	1209.163	-1.06585	1.41E-23	ZNF800
823.1957	883.7331	892.4702	432.5642	440.7717	447.4856	0.976593	1.53E-23	STX4
1494.514	1398.914	1535.815	2336.054	2523.943	2755.792	-0.78155	1.57E-23	CEP57
414.2939	419.0797	362.2808	149.3747	136.4293	111.078	1.590245	1.62E-23	SH2D2A
1276.133	1277.054	1293.078	724.0522	580.3494	528.9428	1.068688	1.67E-23	RPLP0P6
567.9691	613.2632	551.1779	1038.361	1100.88	1192.237	-0.9439	1.74E-23	MSL2
148.2831	119.8786	106.7679	5.186621	5.247282	9.520971	4.234443	1.77E-23	ITGAX
363.9675	373.506	344.9425	746.8734	730.4216	733.1148	-1.0305	1.77E-23	C3orf58
711.7587	693.5125	689.8849	332.981	332.6777	343.8128	1.053585	1.91E-23	GGA1
3813.122	3842.059	3786.154	5366.078	5527.487	5606.794	-0.52826	1.93E-23	STRAP
852.8523	813.3911	859.6185	1391.052	1501.772	1577.308	-0.82314	1.95E-23	ROCK1
328.0201	342.7933	374.1439	746.8734	776.5977	697.1467	-1.08751	1.95E-23	AF121898.1
1695.819	1621.829	1622.507	3156.577	2953.17	2549.504	-0.80967	2.05E-23	SRM
206.6976	271.4606	229.0491	24.89578	45.12662	55.01006	2.500603	2.06E-23	SPINT1
247.1385	272.4513	225.3989	62.23945	59.81901	66.6468	1.980551	2.08E-23	OCEL1
437.6597	457.7183	376.8816	148.3374	118.5886	79.34143	1.876265	2.12E-23	RFNG
1757.828	1925.983	2016.727	2951.187	3291.095	3252.998	-0.7362	2.13E-23	FBXW2
801.6273	824.2892	910.7211	1438.769	1518.563	1568.844	-0.83518	2.15E-23	MFN1
626.3836	594.4393	681.672	1131.721	1199.529	1286.389	-0.92652	2.15E-23	DNAJC11
156.3712	157.5264	146.9199	26.97043	18.89021	15.86829	2.898439	2.17E-23	HYAL1
158.1686	171.3967	179.7716	456.4226	441.8211	416.807	-1.36883	2.32E-23	TLE4
235.4555	283.3494	260.0757	554.9684	649.6135	652.7155	-1.25486	2.34E-23	RNF219
6149.703	6198.021	6372.31	9373.261	9263.551	8611.189	-0.54158	2.34E-23	COPB1
666.8245	653.8832	582.2045	297.712	240.3255	267.6451	1.239602	2.39E-23	RRAS
494.2769	501.3105	476.3491	887.9495	963.4009	984.8916	-0.9464	2.65E-23	STX6
719.8469	725.216	775.6643	1285.245	1335.958	1285.331	-0.8147	2.80E-23	LYRM2
521.2375	524.0973	514.6761	238.5846	183.6549	185.13	1.360413	2.81E-23	PCBP4
1437.896	1429.627	1359.694	2359.912	2260.529	2185.592	-0.68724	2.84E-23	CPT1A
763.8825	753.9472	796.6528	1342.297	1312.87	1347.746	-0.79015	3.05E-23	STRBP
199.5081	222.9147	188.8971	38.38099	49.32445	30.67868	2.367494	3.05E-23	KCTD17
507.7572	509.2363	529.2768	925.2931	987.5384	1016.628	-0.92166	3.34E-23	AP5M1
1299.499	1422.691	1179.922	711.6044	582.4483	552.2163	1.079066	3.35E-23	CDIPT
913.0642	1008.565	957.261	1931.498	1764.136	1587.886	-0.87649	3.36E-23	RRP12
1015.514	854.0111	971.8617	1614.076	1768.334	1737.048	-0.84855	3.80E-23	ETNK1

1113.471	1105.657	1188.135	1816.355	1926.802	1880.921	-0.72279	4.00E-23	HS2ST1
1094.599	1182.934	1165.322	1950.169	1896.368	1834.374	-0.72288	4.01E-23	URB1
505.9598	480.5051	459.9233	961.5995	921.4227	897.0871	-0.94261	4.49E-23	RNF2
264.2135	283.3494	281.0642	598.536	652.7618	585.0108	-1.14845	4.50E-23	RWDD4
494.2769	511.2178	535.6646	943.965	987.5384	964.7917	-0.91019	4.59E-23	CCDC127
775.5654	725.216	753.7632	1259.312	1443.002	1369.962	-0.85257	4.84E-23	AP1S2
483.4927	452.7646	473.6115	853.7178	944.5107	966.9075	-0.97124	4.89E-23	PEX3
504.1624	441.8666	421.5964	871.3523	966.5493	1055.77	-1.08066	5.20E-23	APIP
1027.197	1027.389	1021.139	1629.636	1716.911	1783.595	-0.73802	5.25E-23	ZNF24
2465.992	2336.146	2546.916	1341.26	1462.942	1563.555	0.750968	5.50E-23	BNIP3L
357.6767	361.6172	310.2657	99.58312	116.4897	117.4253	1.626296	5.56E-23	HIST1H2BD
399.9149	489.4217	406.9956	158.7106	162.6657	137.5251	1.497074	5.57E-23	TSPAN15
130.3094	166.443	142.3572	21.78381	17.84076	12.69463	3.066713	5.75E-23	CRLF2
1862.975	1879.419	1979.313	2852.641	3037.127	3166.252	-0.66232	5.78E-23	PDHA1
456.5321	407.1909	474.524	170.1212	170.0119	176.6669	1.37337	5.81E-23	HES1
434.9637	394.3114	419.7713	870.315	791.2901	832.556	-0.99708	5.84E-23	PRKRA
509.5546	525.0881	513.7635	230.286	211.9902	222.156	1.220362	6.19E-23	TRIB2
282.1872	296.2289	298.4026	71.57537	98.6489	60.29948	1.927064	6.21E-23	MAN1A1
141.0936	153.5635	144.1823	22.82113	16.7913	20.09983	2.876731	6.41E-23	PLEKHN1
1509.791	1464.302	1717.412	2502.026	2877.609	2884.854	-0.81655	6.42E-23	KRAS
2960.269	2670.023	2911.022	4467.755	4866.329	4291.842	-0.67357	6.48E-23	SLC20A1
484.3914	464.6534	457.1857	165.9719	181.5559	205.2298	1.347584	6.61E-23	NR1H3
1880.948	1915.085	1981.138	2879.612	2942.676	3029.785	-0.61561	6.81E-23	UBE2G1
816.9049	848.0667	841.3676	396.2578	426.0793	445.3699	0.983404	6.91E-23	PDE9A
225.57	181.304	218.0985	49.79156	35.68152	24.33137	2.507997	6.97E-23	REEP6
1191.657	1347.396	1315.892	2236.471	2067.429	2139.045	-0.74147	7.18E-23	SHCBP1
415.1926	440.8758	427.9842	164.9345	100.7478	89.92028	1.851271	7.22E-23	ZNF219
516.744	580.5691	604.1056	1099.564	1147.056	1030.381	-0.94605	7.29E-23	TNFRSF10A
186.0279	239.7572	207.148	487.5423	564.6075	549.0427	-1.34083	7.57E-23	CACNA2D1
1125.154	1026.399	1078.63	564.3043	585.5966	599.8212	0.884927	7.61E-23	CYTH2
2317.709	2368.841	2243.951	3500.969	3388.695	3620.085	-0.60083	7.67E-23	PPP2R5C
206.6976	219.9425	253.6878	573.6402	578.2504	506.7272	-1.2856	7.79E-23	MGME1
1196.15	1210.675	1151.633	1921.124	1874.329	1936.989	-0.68806	7.93E-23	SUGT1
1282.424	1030.361	1146.158	452.2733	595.0417	532.1165	1.131547	7.95E-23	SNAI2
274.9977	299.2011	270.1137	80.91128	89.20379	80.39931	1.752409	8.00E-23	APH1B
440.3558	440.8758	396.0451	163.8972	140.6272	101.557	1.652367	8.05E-23	DLX2
386.4347	355.6729	360.4558	817.4114	704.1852	784.9512	-1.06445	8.13E-23	TRMT5
188.7239	188.2391	185.2469	41.49297	22.03858	35.96811	2.497756	8.14E-23	ACBD4
7016.036	7384.918	7432.689	10085.9	10532.34	10925.84	-0.53085	8.59E-23	CKAP5
1088.308	1099.713	1181.747	1795.608	2008.659	1893.615	-0.75762	9.14E-23	USP8
2829.061	2999.937	3190.262	4525.845	4868.428	4591.224	-0.63288	9.40E-23	PRKAR1A
5639.25	5500.545	5294.593	3858.846	3673.097	3634.895	0.557466	9.61E-23	CSRP1
1619.431	1656.504	1565.929	2447.048	2529.19	2574.894	-0.64128	9.67E-23	NUP93
2580.125	2638.32	2745.852	3856.771	4363.639	4244.237	-0.64618	9.83E-23	ADGRG6
846.5615	889.6775	869.6565	388.9966	465.9586	439.0226	1.009969	1.01E-22	ACOT13
290.2753	254.6182	249.1251	594.3867	573.0032	649.5418	-1.19352	1.03E-22	TSEN2
1230.3	1163.12	1333.23	2009.297	2401.156	2221.56	-0.83117	1.03E-22	WASHC4
4027.907	4272.037	3913.911	6431.41	5968.258	5961.186	-0.58832	1.09E-22	PHB2
568.8678	633.0779	618.7063	1078.817	1115.572	1132.996	-0.87045	1.12E-22	DIS3L
1250.97	1151.231	1203.648	665.9621	630.7233	522.5955	0.986956	1.12E-22	RIPOR1
112.3357	100.0639	107.6805	318.4585	317.9853	317.3657	-1.5741	1.15E-22	EVI2B
9853.185	10410.61	10727.89	14569.22	15464.79	14600.94	-0.52632	1.16E-22	CLTC
1785.688	1849.697	1833.305	2726.088	2788.406	2928.228	-0.62659	1.21E-22	PCYOX1
318.1346	319.0158	310.2657	701.2311	665.3553	630.4999	-1.07604	1.22E-22	MTPAP

534.7177	560.7544	567.6037	983.3833	1024.269	1076.928	-0.89133	1.23E-22	TMEM184C
496.973	649.9203	587.6798	240.6592	209.8913	232.7348	1.343149	1.25E-22	SLC22A23
355.8794	407.1909	415.2085	810.1502	845.8618	768.025	-1.04135	1.27E-22	TRMT6
277.6938	249.6645	248.2126	80.91128	53.52227	49.72063	2.073518	1.30E-22	FAM46B
355.8794	342.7933	285.627	102.6951	86.05542	108.9622	1.725068	1.30E-22	POLM
891.4958	888.6868	949.9606	1495.821	1550.047	1548.745	-0.75075	1.34E-22	RAB18
276.7951	250.6552	269.2011	590.2374	573.0032	586.0687	-1.13393	1.35E-22	GPAM
828.5878	745.0306	806.6909	1402.462	1338.057	1485.271	-0.82743	1.40E-22	USP25
1232.996	1276.063	1180.835	1976.102	1986.621	2200.402	-0.74029	1.41E-22	VPS29
171.6489	167.4337	174.2963	34.2317	25.18695	32.79446	2.476918	1.42E-22	CREB3L3
1054.158	1026.399	1068.592	1669.055	1725.306	1793.116	-0.71982	1.51E-22	HMGB3
2366.238	2599.681	2534.141	3832.913	4752.988	5022.841	-0.85964	1.56E-22	KMT2A
1625.722	1603.995	1497.488	947.0769	929.8183	848.4243	0.794385	1.57E-22	STK25
225.57	162.4801	155.1329	9.335917	32.53315	15.86829	3.235575	1.62E-22	PPFIA4
312.7425	301.1826	361.3683	663.8874	726.2238	728.8832	-1.11864	1.67E-22	ZNF518A
798.0325	784.6599	740.075	403.5191	304.3423	314.192	1.18398	1.69E-22	ELOF1
564.3744	513.1993	565.7787	1055.996	972.846	1074.812	-0.91662	1.70E-22	PIAS1
447.5453	470.5978	408.8207	856.8297	854.2575	888.624	-0.97088	1.71E-22	KLHL8
1382.178	1490.061	1545.853	2291.449	2454.678	2425.732	-0.69904	1.77E-22	ZNF638
2243.118	2343.082	2346.156	1516.568	1323.364	1332.936	0.732108	1.82E-22	ZBED1
931.9366	956.0566	982.8123	1618.226	1640.3	1536.05	-0.74002	1.83E-22	MRM2
626.3836	597.4115	629.6569	300.824	282.3038	252.8347	1.148676	1.88E-22	GRAMD2B
221.9753	252.6367	246.3875	41.49297	60.86847	66.6468	2.093512	1.89E-22	F11R
1007.426	998.658	1026.614	1592.293	1810.312	1763.495	-0.76831	1.93E-22	TTF2
700.0758	692.5218	682.5846	332.981	331.6282	350.1602	1.032171	2.06E-22	WFS1
8.986853	14.86098	7.30037	136.9268	109.1435	114.2517	-3.54418	2.10E-22	PABPC1P4
771.072	832.215	838.63	383.8099	434.4749	360.739	1.050175	2.21E-22	ZSWIM8
229.1647	243.7201	250.9502	505.1769	622.3276	592.416	-1.24871	2.21E-22	FAM135A
921.1524	924.3531	943.5728	460.5719	300.1445	280.3397	1.421279	2.21E-22	MEX3D
1090.105	1058.102	1038.478	596.4614	496.3929	447.4856	1.048528	2.23E-22	ARHGEF16
2457.006	2359.924	2602.582	1603.703	1507.019	1525.471	0.678536	2.32E-22	PAIP1
665.0271	711.3457	713.6111	320.5332	321.1336	356.5075	1.066084	2.51E-22	WDR60
726.1377	811.4097	748.2879	1308.066	1330.711	1453.535	-0.84076	2.54E-22	NDC80
478.9993	547.8749	556.6532	1019.69	969.6977	1088.564	-0.95931	2.54E-22	THOC7
2090.342	2332.184	2161.822	3292.467	3487.343	3605.274	-0.65772	2.55E-22	KIAA0100
208.495	238.7665	216.2735	57.05283	51.42336	32.79446	2.230074	2.69E-22	TPPP
1594.268	1638.671	1703.724	2482.317	2731.735	2612.978	-0.6649	2.71E-22	ANKFY1
3823.906	4050.113	3825.394	5737.44	5699.597	5517.932	-0.53547	2.78E-22	PLP2
213.8871	171.3967	205.3229	488.5797	473.3048	497.2063	-1.30288	2.91E-22	KIAA1586
1288.715	1376.127	1457.336	2215.724	2217.501	2231.081	-0.69314	2.99E-22	XRN2
692.8864	748.0028	770.189	1612.002	1372.689	1288.505	-0.95087	3.03E-22	SNRPB2
982.263	909.4921	990.1126	499.9902	496.3929	531.0586	0.916344	3.03E-22	CHMP1B
299.2622	291.2753	295.665	605.7973	628.6244	732.0569	-1.14957	3.30E-22	THEM4
194.116	201.1186	187.072	26.97043	38.82988	47.60486	2.361399	3.53E-22	TSHZ2
275.8964	235.7943	275.589	56.0155	81.85759	46.54697	2.09428	3.55E-22	SLC27A1
87.17247	91.14736	94.90481	300.824	344.2217	605.1106	-2.19378	3.68E-22	KIF15
1002.933	1045.222	1144.333	1721.958	1962.483	2053.356	-0.84568	3.70E-22	EFR3A
766.5785	791.595	730.037	402.4818	324.282	316.3078	1.132836	3.71E-22	SLC16A5
1675.149	1752.605	1641.671	1058.071	998.033	942.5761	0.757166	3.72E-22	ADA
401.7123	408.1817	384.182	758.2839	850.0596	997.5862	-1.12595	3.88E-22	ERCC6L2
402.611	418.089	432.5469	873.4269	800.7352	792.3564	-0.97698	3.89E-22	AMER1
274.9977	335.8582	358.6307	735.4628	831.1694	685.5099	-1.21666	3.97E-22	LCLAT1
2270.978	2433.238	2397.259	3938.72	3940.709	3457.17	-0.675	4.09E-22	CRKL
1457.668	1535.635	1556.804	939.8157	856.3564	834.6718	0.790131	4.17E-22	SMAD6

390.9281	371.5246	364.1059	743.7614	809.1308	724.6517	-1.01536	4.25E-22	FASTKD1
145.587	174.3689	167.9085	397.2951	423.9804	470.7591	-1.40619	4.30E-22	LINC00641
667.7232	612.2725	698.0979	1128.609	1332.81	1358.325	-0.94868	4.39E-22	CEP152
1001.135	1124.481	1070.417	539.4086	559.3602	601.9369	0.909891	4.53E-22	RFX5
160.8647	170.4059	173.3838	411.8177	415.5847	431.6174	-1.3192	4.55E-22	EXOC6
1658.973	1732.791	1524.865	992.7192	922.4721	872.7557	0.818081	4.56E-22	ENG
762.0851	827.2614	782.9647	1396.238	1553.195	1326.589	-0.85043	4.64E-22	TLR4
960.6946	927.3253	887.9075	478.2064	513.1842	449.6014	0.945873	4.68E-22	SUSD2
620.9915	575.6154	690.7975	1113.049	1289.782	1241.958	-0.94869	4.73E-22	PGGT1B
1078.422	1078.907	1150.721	1736.481	2099.962	2058.646	-0.83334	4.81E-22	MDM2
2290.749	2214.286	2327.905	3304.915	3548.212	3671.921	-0.62304	4.82E-22	LRRFIP1
453.8361	387.3763	440.7598	822.598	847.9607	936.2288	-1.02275	4.95E-22	PAXIP1
429.5716	410.1631	400.6078	787.329	799.6857	814.572	-0.95304	5.01E-22	AC005912.1
2076.862	1907.159	2138.096	3070.479	3482.096	3535.454	-0.72018	5.50E-22	ZRANB2
1476.54	1654.523	1679.998	2494.765	2735.933	2865.812	-0.75112	5.58E-22	ANKRD50
388.232	420.0704	418.8587	782.1424	795.4879	810.3404	-0.96086	5.68E-22	CCDC77
2524.407	2592.746	2461.137	1516.568	1607.767	1676.749	0.658479	5.78E-22	RAB5B
2444.424	2671.014	2560.605	4115.065	4111.77	3721.642	-0.63867	5.79E-22	ALDH18A1
567.0704	570.6617	560.3034	1016.578	1009.577	1016.628	-0.84161	5.92E-22	RLF
3506.67	3779.643	4054.443	5715.656	5973.506	5712.583	-0.61776	5.92E-22	LMNB1
543.7046	452.7646	458.0982	945.0023	935.0656	1001.818	-0.98553	6.33E-22	NFE2L3
3368.272	3540.877	3446.687	4979.156	5032.143	4890.605	-0.52521	6.38E-22	NSD2
149.1818	132.7581	116.8059	16.59719	9.445107	16.92617	3.214133	6.38E-22	ARTN
685.6969	685.5867	689.8849	1134.833	1283.485	1286.389	-0.84578	6.60E-22	IDH3A
498.7703	552.8286	538.4023	1080.892	941.3623	1011.339	-0.93259	6.60E-22	EIF1AD
3698.09	3491.34	3964.101	5609.849	5976.654	5518.99	-0.61676	6.74E-22	UHMK1
4399.963	4687.154	4448.663	3155.54	3006.692	2939.864	0.572317	6.81E-22	DHCR24
1175.48	1180.953	1267.527	1877.557	2208.056	2186.65	-0.79123	6.89E-22	ZC3H7A
1422.619	1433.589	1355.131	2442.898	2251.084	2162.318	-0.70335	6.94E-22	CERK
1624.823	1659.476	1639.846	2677.334	2463.074	2515.652	-0.63686	7.16E-22	PBK
1396.557	1348.386	1312.241	2066.35	2173.424	2230.023	-0.67312	7.59E-22	HSD17B12
380.1439	360.6265	366.8436	148.3374	130.1326	115.3095	1.491718	7.65E-22	ABTB1
215.6845	208.0538	192.5473	1.037324	2.098913	1.057886	7.199559	7.92E-22	CACNA1H
105.1462	166.443	145.0948	438.7881	695.7896	431.6174	-1.91233	8.08E-22	PARN
382.8399	382.4226	347.6801	148.3374	99.69835	103.6728	1.661095	8.29E-22	TBX1
1706.603	1675.328	1680.91	2523.81	2571.168	2577.01	-0.59957	8.36E-22	PATL1
1524.17	1460.339	1467.374	2272.777	2322.447	2526.231	-0.67755	8.42E-22	TXNDC12
1166.493	1490.061	1416.272	720.9403	688.4434	634.7314	0.99398	8.69E-22	SPTBN2
10334.88	10028.19	10120.14	14850.33	13660.77	14330.12	-0.49096	9.44E-22	BTF3
778.2615	828.2521	838.63	1524.866	1455.596	1328.704	-0.81779	9.47E-22	DNAAF5
363.0689	401.2465	343.1174	769.6945	761.9053	722.5359	-1.02643	9.67E-22	MASTL
1137.736	1107.639	1137.033	2030.043	1826.054	2365.432	-0.87906	9.81E-22	AC116533.1
1437.896	1538.607	1338.705	842.3072	744.0645	670.6995	0.934489	9.89E-22	CBX4
1058.651	1093.768	1123.344	1703.286	1935.198	1851.3	-0.74491	1.01E-21	CDC42SE2
1026.299	1135.379	1163.496	622.3945	601.3385	567.0267	0.892527	1.06E-21	IGSF3
147.3844	190.2206	166.996	34.2317	22.03858	13.75251	2.846222	1.07E-21	CEBPA
363.0689	348.7377	359.5432	684.6339	769.2515	889.6819	-1.12884	1.14E-21	CEP295
1248.274	1272.1	1192.698	711.6044	529.9755	526.8271	1.069778	1.18E-21	MGLL
787.2483	821.317	772.9266	422.1909	400.8923	404.1123	0.956166	1.18E-21	CC2D1A
7303.615	7053.022	7440.902	9902.296	10252.14	10210.71	-0.47816	1.23E-21	SFPQ
308.2491	266.507	271.0262	574.6776	643.3167	660.1207	-1.14997	1.23E-21	PDP2
176.1423	173.3781	145.0948	4.149297	1.049456	0	6.560841	1.24E-21	MT-TY
381.9412	410.1631	406.9956	732.3508	874.1971	845.2507	-1.03213	1.28E-21	SPAST
898.6853	960.0195	919.8466	508.2888	483.7994	418.9227	0.977122	1.28E-21	TMUB2

483.4927	447.8109	438.0222	837.1206	892.0379	886.5082	-0.93333	1.29E-21	ESCO1
2484.865	2671.014	2351.632	1560.136	1484.981	1299.084	0.78891	1.29E-21	NSMF
1082.916	1053.148	1145.246	1717.809	1939.395	1854.474	-0.7479	1.30E-21	KCTD9
1485.527	1509.876	1471.937	2377.547	2296.21	2243.776	-0.63099	1.32E-21	INTS10
7929.1	8893.803	8264.931	12507.02	12036.21	11945.65	-0.54062	1.37E-21	CCT7
149.1818	144.6469	138.707	22.82113	24.1375	17.98406	2.735024	1.38E-21	BRSK2
665.9258	714.3179	694.4477	1164.915	1266.694	1210.221	-0.81212	1.39E-21	GOLGA7
876.2181	745.0306	780.227	366.1754	387.2494	411.5175	1.044332	1.43E-21	TUBGCP6
612.9034	631.0964	546.6152	279.0402	260.2652	269.7608	1.145705	1.47E-21	PPP1R14BP3
427.7742	453.7553	389.6572	178.4198	132.2315	150.2198	1.462975	1.49E-21	MVK
517.6427	516.1715	626.9193	1066.369	1202.677	1049.423	-0.99824	1.49E-21	INTS2
1714.692	1763.503	1825.092	2684.595	3403.387	3398.987	-0.839	1.50E-21	CCDC88A
1539.448	1534.644	1661.747	2433.562	2769.515	2518.826	-0.70518	1.51E-21	MTMR2
720.7456	789.6135	799.3905	1284.207	1432.508	1558.266	-0.88835	1.52E-21	SPRED1
283.9845	280.3772	333.0794	659.7382	637.02	635.7893	-1.10601	1.54E-21	BRMS1L
803.4246	925.3439	758.3259	420.1163	345.2711	309.9605	1.208797	1.59E-21	DEDD2
1879.151	2040.908	2018.552	2925.254	3232.326	3149.326	-0.64836	1.62E-21	ANKRD28
513.1493	500.3197	511.0259	919.0692	1020.072	916.129	-0.9051	1.64E-21	POLR2M
321.7293	299.2011	291.1022	605.7973	650.6629	668.5837	-1.07732	1.65E-21	ANKRD49
1392.962	1157.175	1256.576	2097.469	2141.94	2250.123	-0.76889	1.71E-21	RSRC2
638.0665	623.1705	734.5997	1163.878	1456.645	1329.762	-0.9845	1.74E-21	TSNAX
2394.098	2526.367	2676.498	3715.695	4175.787	4168.07	-0.66667	1.76E-21	EIF2AK2
1110.775	1163.12	1302.203	1940.833	2025.451	2057.588	-0.75219	1.81E-21	MTR
1883.644	1939.854	2034.978	2896.209	3180.902	3020.264	-0.6349	1.83E-21	YTHDF3
509.5546	484.468	446.2351	203.3155	172.1108	129.0621	1.512812	1.84E-21	KLF2
35712.85	31760.89	31912.65	22174.88	18389.62	16836.25	0.791977	1.86E-21	MT-ND1
611.106	638.0315	559.3908	1171.139	1169.094	1038.844	-0.9024	1.87E-21	LAMC2
838.4734	856.9833	716.3488	354.7649	267.6114	188.3037	1.572224	1.95E-21	KLF16
315.4385	292.266	314.8284	102.6951	97.59944	108.9622	1.577304	2.02E-21	BAMBI
6509.177	6442.731	6567.595	8758.128	9172.248	9226.879	-0.47637	2.03E-21	ANXA5
1282.424	1294.887	1294.903	1985.438	2018.105	2170.781	-0.6731	2.05E-21	NCOA6
1282.424	1349.377	1364.257	2054.939	2189.166	2390.822	-0.73155	2.05E-21	PPFIA1
562.577	606.3281	565.7787	1070.519	999.0824	1150.98	-0.89312	2.07E-21	LEO1
95.26064	129.7859	114.9808	5.186621	2.098913	8.463085	4.433325	2.11E-21	KRT14
1140.432	1291.915	1133.382	615.1332	419.7825	375.5494	1.337436	2.11E-21	NAA60
541.0085	546.8842	512.851	224.062	250.8201	238.0243	1.166911	2.11E-21	RMND5B
334.3109	276.4143	322.1288	95.43382	98.6489	106.8465	1.633295	2.19E-21	TPM2
785.4509	727.1974	733.6872	1353.708	1287.683	1251.479	-0.79297	2.23E-21	NT5C3A
805.222	792.5857	798.4779	265.555	277.0565	408.3439	1.333819	2.26E-21	ABCA1
405.3071	449.7924	447.1476	824.6727	862.6531	817.7456	-0.9444	2.33E-21	MTO1
1578.99	1606.968	1735.663	2553.892	2582.712	2518.826	-0.63726	2.43E-21	SKA2
339.703	375.4875	370.4938	136.9268	124.8853	90.97817	1.620782	2.44E-21	BICRA
206.6976	216.9703	215.3609	467.8332	522.6293	484.5116	-1.20702	2.48E-21	PPP2R3A
323.5267	334.8675	374.1439	700.1938	748.2624	684.452	-1.04645	2.50E-21	AMMECR1
985.8578	1011.538	1007.451	1644.159	1864.884	1617.507	-0.77079	2.55E-21	FBXO28
470.9111	460.6905	492.775	859.9417	908.8292	887.5661	-0.89875	2.55E-21	EMB
1504.399	1365.229	1469.199	2214.687	2550.179	2407.748	-0.72477	2.57E-21	FNDC3A
127.6133	119.8786	118.631	15.55986	13.64293	9.520971	3.239757	2.59E-21	PLEKHG1
421.4834	431.9592	479.0868	863.0537	884.6917	826.2087	-0.94965	2.62E-21	SLC35G1
790.843	824.2892	755.5883	415.967	349.469	301.4974	1.151255	2.63E-21	OBSL1
639.8639	661.8091	644.2576	1212.632	1193.232	1076.928	-0.84009	2.64E-21	ORC4
1007.426	1090.796	1026.614	1734.406	1683.328	1672.517	-0.70445	2.67E-21	PRMT5
478.1006	509.2363	459.9233	883.8002	922.4721	897.0871	-0.90205	2.69E-21	QRSL1
856.4471	830.2336	759.2385	429.4522	407.1891	410.4596	0.971666	2.76E-21	NLRC5

1937.565	2168.713	2091.556	3376.49	3187.199	3117.589	-0.6438	2.77E-21	M6PR
11.68291	13.87025	8.212916	107.8817	125.9348	108.9622	-3.34941	2.84E-21	GALC
2368.934	2431.257	2339.769	1512.419	1327.562	1159.443	0.835889	2.86E-21	BTBD2
934.6327	970.9175	914.3713	518.6621	479.6016	406.2281	1.005142	2.94E-21	PIMREG
1392.962	1360.275	1306.766	790.441	814.3781	801.8773	0.754449	2.94E-21	SF3A2
559.8809	572.6432	548.4403	274.8909	211.9902	202.0562	1.286237	3.01E-21	TWF2
3446.458	3421.989	3306.155	2350.576	2152.435	2076.63	0.628782	3.06E-21	TMEM59
950.809	1006.584	973.6868	494.8036	362.0624	290.9186	1.352034	3.17E-21	PPP1R12C
866.3326	825.2799	930.7971	1474.038	1574.185	1452.477	-0.7788	3.23E-21	AIDA
248.0371	212.0167	302.9653	595.4241	658.0091	607.2264	-1.28435	3.25E-21	C12orf45
390.0294	413.1353	427.0716	782.1424	832.2189	773.3144	-0.95686	3.25E-21	WASF1
470.0124	526.0788	511.9384	1048.735	940.3129	910.8396	-0.94401	3.26E-21	HAUS2
334.3109	368.5524	271.0262	93.35917	97.59944	107.9043	1.703769	3.31E-21	NCF2
812.4115	830.2336	977.337	1496.859	1582.58	1664.054	-0.85608	3.37E-21	TIA1
1747.943	1900.224	1930.035	2840.193	2916.439	2837.249	-0.62371	3.38E-21	NCBP2
612.9034	740.0769	556.6532	258.2937	135.3799	142.8146	1.830624	3.39E-21	MZT2B
320.8306	334.8675	318.4786	108.919	118.5886	116.3674	1.502189	3.47E-21	MAP3K12
1312.081	1554.459	1278.477	768.6572	666.4048	588.1844	1.034044	3.64E-21	TMEM9
356.7781	348.7377	350.4177	681.522	699.9874	704.5519	-0.98208	3.71E-21	NFXL1
7378.206	7600.897	7992.992	10593.15	10827.24	11280.23	-0.50942	3.79E-21	HNRNPA3
2731.105	2714.606	3049.729	4351.575	4245.051	4300.305	-0.60212	4.09E-21	RAB2A
678.5074	656.8554	661.596	342.317	306.4413	264.4714	1.128491	4.15E-21	SNX33
890.5971	884.7238	883.3447	460.5719	491.1456	485.5695	0.887457	4.17E-21	ARHGDIB
1661.669	1701.087	1714.674	3041.434	2786.307	2530.463	-0.71922	4.33E-21	UAP1
3287.391	3401.184	3311.63	5430.392	4850.587	4837.711	-0.59643	4.34E-21	SNU13
900.4827	897.6034	779.3145	435.6761	447.0684	427.3858	0.975964	4.36E-21	LPCAT4
152.7765	124.8323	120.4561	9.335917	12.59348	21.15771	3.210523	4.44E-21	NKAIN4
197.7108	215.9796	216.2735	48.75423	54.57173	55.01006	1.992289	4.46E-21	JPH2
298.3635	265.5162	269.2011	87.13523	69.26412	42.31543	2.06681	4.48E-21	RNF215
3369.171	3496.294	3454.9	4847.416	4875.774	4924.458	-0.50527	4.49E-21	FOXM1
198.6094	217.9611	187.9845	477.1691	498.4918	451.7172	-1.2406	4.50E-21	SCFD2
1388.469	1440.525	1417.184	882.7628	745.114	737.3463	0.843886	4.53E-21	CHPF2
1065.841	1130.425	1033.002	241.6965	141.6766	126.9463	2.661361	4.66E-21	C20orf24
276.7951	333.8767	284.7144	670.1114	682.1466	604.0527	-1.12911	4.70E-21	ARSK
828.5878	863.9185	809.4285	1827.765	1578.382	1385.83	-0.93793	4.76E-21	AAR2
1763.221	2016.14	1911.784	3194.958	2896.5	3009.685	-0.67775	4.76E-21	TIMM23
2105.62	1996.325	2128.058	3054.92	3242.82	3101.721	-0.59308	5.06E-21	PAPD7
215.6845	183.2855	239.0871	51.86621	53.52227	38.08388	2.153173	5.10E-21	RAPGEF3
316.3372	249.6645	251.8628	82.98593	70.31358	79.34143	1.814339	5.39E-21	CTH
245.3411	231.8313	247.3	520.7367	516.3325	560.6794	-1.1404	5.56E-21	C5orf34
127.6133	131.7674	118.631	14.52254	15.74185	16.92617	3.002034	5.82E-21	BBC3
2430.045	2584.82	2690.186	3767.561	3998.429	4277.032	-0.64434	5.88E-21	THOC2
463.7216	471.5885	467.2237	842.3072	927.7194	857.9453	-0.906	5.91E-21	CSGALNACT2
454.7348	492.3939	533.8395	878.6135	1078.841	1109.722	-1.05033	5.94E-21	FER
434.9637	444.8387	439.8473	169.0838	196.2483	180.8985	1.272617	6.01E-21	LRSAM1
751.3009	761.873	824.9418	397.2951	405.0901	400.9387	0.95845	6.12E-21	PPM1H
584.1454	672.7071	591.3299	270.7416	285.4521	295.1501	1.117755	6.51E-21	ST5
1151.216	1123.49	1053.991	554.9684	532.0744	653.7733	0.935337	6.53E-21	PITPNC1
463.7216	503.2919	473.6115	221.9874	199.3967	185.13	1.247389	7.09E-21	SCRN2
715.3535	728.1882	743.7252	390.0339	363.1119	360.739	0.973384	7.34E-21	TEX264
374.7518	427.0056	425.2465	776.9558	820.6749	797.6458	-0.96569	7.46E-21	CRLS1
166.2568	160.4986	141.4447	26.97043	27.28586	29.6208	2.480903	7.61E-21	SOX18
2513.623	2458.997	2512.24	3690.799	3867.247	3547.091	-0.56911	7.71E-21	PSAT1
3759.201	3907.448	4055.355	5561.095	5622.987	6017.254	-0.5533	8.16E-21	KIF20A

329.8175	394.3114	381.4443	724.0522	767.1526	750.0409	-1.02046	8.16E-21	ENTPD7
124.0186	106.0083	117.7185	325.7198	317.9853	330.0603	-1.48401	8.19E-21	WDR89
10953.18	10881.21	10706.9	21620.95	18700.26	15829.14	-0.78704	8.23E-21	RPL29
1190.758	1319.655	1261.139	1995.812	2239.54	2007.867	-0.72751	8.24E-21	ERCC6
830.3852	821.317	916.1964	1408.686	1481.832	1619.623	-0.81223	8.26E-21	RPS6KB1
1199.745	1230.489	1255.664	1915.938	2024.401	1895.731	-0.66303	8.27E-21	REST
1088.308	1123.49	1119.694	1871.333	1748.394	1737.048	-0.68538	8.36E-21	TMEM183A
365.7649	339.8211	370.4938	119.2923	114.3907	148.104	1.4957	8.39E-21	CYP2S1
248.9358	235.7943	235.4369	46.67959	48.27499	78.28354	2.056768	8.48E-21	SEMA6D
244.4424	251.646	219.0111	72.61269	62.96738	46.54697	1.971994	8.50E-21	CLMN
56.61717	64.39759	57.49041	232.3606	224.5837	204.1719	-1.89068	8.50E-21	AADACP1
318.1346	373.506	355.893	768.6572	710.4819	682.3363	-1.04607	8.92E-21	FAM171A1
203.1029	255.6089	199.8476	58.09015	50.3739	33.85234	2.208184	9.01E-21	KRT87P
90.76721	104.0269	89.42953	273.8536	296.9961	290.9186	-1.60192	9.07E-21	CENPP
785.4509	882.7424	824.9418	1852.661	1525.91	1424.972	-0.94668	9.67E-21	DCTPP1
127.6133	158.5171	136.8819	25.9331	16.7913	15.86829	2.849536	9.76E-21	MOSPD3
2066.077	2170.694	2184.636	3172.137	3162.012	3153.557	-0.56331	9.76E-21	BTBD3
867.2313	854.0111	831.3296	1526.941	1423.063	1366.788	-0.7581	9.84E-21	GRPEL1
529.3256	545.8934	510.1133	918.0319	974.9449	1049.423	-0.89243	1.02E-20	C2orf69
1159.304	1193.832	1194.523	1850.586	1851.241	1850.242	-0.64627	1.03E-20	CLPX
609.3086	537.9676	539.3148	985.4579	1117.671	1052.596	-0.90326	1.04E-20	ANGEL2
174.3449	163.4708	150.5701	33.19437	32.53315	27.50503	2.388722	1.07E-20	FZD10-AS1
920.2537	1026.399	919.8466	502.0649	345.2711	324.7709	1.289529	1.07E-20	TMEM115
328.0201	266.507	271.9388	96.47114	83.95651	63.47314	1.828676	1.14E-20	KLHL17
109.6396	142.6654	93.07971	10.37324	8.395651	7.4052	3.719471	1.17E-20	KREMEN2
3371.867	3602.302	3388.284	5148.24	4918.802	4937.152	-0.53423	1.19E-20	SRP14
3134.614	3243.657	3107.22	2181.493	1951.989	1876.689	0.658119	1.21E-20	MAP7D1
283.0859	319.0158	319.3912	661.8128	613.932	638.9629	-1.05576	1.23E-20	PALB2
292.0727	414.1261	255.5129	88.17255	85.00596	80.39931	1.921638	1.23E-20	-
757.5917	762.8638	751.9381	314.3092	382.0021	398.8229	1.053387	1.24E-20	MYLIP
1317.473	1364.238	1398.021	2241.657	2053.786	2142.218	-0.65816	1.24E-20	WTAP
3420.396	3417.035	3324.406	5111.933	4908.307	4708.649	-0.53555	1.25E-20	VDAC2
478.1006	519.1437	427.9842	188.793	189.9516	207.3456	1.281544	1.29E-20	CCS
366.6636	354.6821	389.6572	154.5613	128.0337	113.1938	1.488755	1.30E-20	SLC12A9
2109.214	2115.213	2105.244	3011.352	3276.403	3377.829	-0.61068	1.32E-20	RBBP6
1005.629	1102.685	987.375	592.3121	519.4809	551.1584	0.895979	1.33E-20	MAPK3
667.7232	631.0964	717.2613	346.4663	300.1445	272.9345	1.132569	1.34E-20	TAF1C
2378.82	2263.823	2073.305	1350.596	1347.502	1122.417	0.813709	1.34E-20	TRAF4
177.9397	198.1464	201.6727	479.2438	432.376	455.9487	-1.24371	1.40E-20	LINS1
264.2135	264.5255	251.8628	81.94861	81.85759	80.39931	1.676346	1.40E-20	TAPBPL
1173.683	1231.48	1144.333	686.7086	520.5303	497.2063	1.057852	1.44E-20	PHPT1
363.9675	348.7377	337.6421	719.903	758.7569	654.8312	-1.02229	1.47E-20	TMEM267
103.3488	134.7396	121.3686	308.0853	373.6065	370.26	-1.55109	1.48E-20	AL355032.1
594.031	573.6339	631.482	1048.735	1119.77	1044.133	-0.83602	1.49E-20	CEP97
502.3651	512.2085	563.9536	955.3755	965.4998	960.5602	-0.86801	1.51E-20	PPP3CB
1676.048	1710.994	1727.45	2563.228	2746.427	2525.173	-0.61536	1.54E-20	EMC1
777.3628	828.2521	711.7861	372.3994	274.9576	216.8666	1.422292	1.59E-20	FAM234A
827.6891	887.696	867.8315	1469.888	1501.772	1369.962	-0.74945	1.59E-20	GPD2
1442.39	1425.664	1419.009	2161.784	2187.067	2177.129	-0.60617	1.64E-20	NPTN
612.0047	624.1613	577.6418	292.5254	268.6608	210.5192	1.232308	1.65E-20	CCM2
776.4641	800.5116	772.0141	423.2282	342.1228	302.5553	1.136729	1.71E-20	ZNF687
893.2932	887.696	879.6946	510.3635	441.8211	417.8648	0.957299	1.73E-20	PGP
1675.149	1709.013	1551.329	1011.391	804.933	772.2565	0.930697	1.73E-20	METTL26
508.6559	515.1807	496.4251	239.6219	214.0891	228.5033	1.1558	1.73E-20	ALG5

2697.853	2714.606	2878.171	3951.168	4642.795	4625.076	-0.67297	1.78E-20	TAOK1
282.1872	300.1918	243.6498	94.3965	56.67064	61.35737	1.957872	1.82E-20	TH
1157.507	1097.731	1074.067	1721.958	1814.51	1930.641	-0.71534	1.86E-20	NFX1
3924.559	4064.974	3956.8	2802.85	2698.152	2524.115	0.573825	1.86E-20	BAG1
732.4285	754.9379	694.4477	387.9592	362.0624	354.3917	0.981847	1.88E-20	RNF123
644.3573	575.6154	627.8318	1062.22	1110.325	1236.668	-0.88287	1.94E-20	THAP9-AS1
629.0797	618.2169	626.9193	1103.713	1044.209	1112.896	-0.79882	1.97E-20	ZCCHC8
620.0928	753.9472	741.9001	1347.484	1502.821	1234.553	-0.94963	1.98E-20	ABCC4
305.553	321.988	293.8399	118.255	80.80814	80.39931	1.720065	2.01E-20	RRNAD1
535.6164	570.6617	549.3528	223.0247	243.4739	273.9924	1.160886	2.02E-20	MZF1
389.1307	349.7285	365.0185	684.6339	809.1308	752.1567	-1.024	2.03E-20	IDS
202.2042	197.1557	195.2849	56.0155	45.12662	42.31543	2.050769	2.14E-20	ZNF516
2186.501	1925.983	2074.218	1274.871	1248.853	1306.489	0.692127	2.21E-20	MPZL2
709.0627	718.2808	659.7709	332.981	215.1386	190.4194	1.49808	2.21E-20	AGTRAP
157.2699	145.6376	158.783	19.70916	32.53315	27.50503	2.534672	2.23E-20	TRPM4
548.198	507.2549	533.8395	912.8452	988.5879	997.5862	-0.86659	2.24E-20	TSEN15
3441.965	3682.552	3635.584	2410.741	2163.979	2422.558	0.620708	2.26E-20	UQCRH
1190.758	1086.833	1138.858	1998.924	1814.51	1803.695	-0.71701	2.31E-20	RNF11
3783.465	3712.273	3597.257	2485.429	2609.998	2473.337	0.551521	2.32E-20	ATXN2L
2009.46	2165.741	2095.206	1300.804	1361.145	1344.573	0.646015	2.36E-20	ABL2
1022.704	1024.417	937.185	551.8564	448.1179	386.1283	1.10591	2.37E-20	NDUFAF3
1648.189	1777.374	1641.671	3037.285	2901.747	2514.594	-0.73871	2.41E-20	SLC35A4
307.3504	343.7841	311.1783	629.6558	649.6135	690.7993	-1.03454	2.43E-20	TIGAR
496.973	545.8934	549.3528	228.2113	256.0673	227.4454	1.161387	2.44E-20	TRIM21
949.0117	889.6775	972.7743	1510.344	1530.107	1572.018	-0.71372	2.47E-20	RALA
347.7912	353.6914	323.9539	702.2684	689.4928	652.7155	-0.99594	2.55E-20	TTLL4
549.9954	560.7544	508.2882	264.5177	201.4956	220.0402	1.238205	2.55E-20	PPP1R16A
365.7649	310.0992	302.0528	110.9937	119.638	96.2676	1.581105	2.57E-20	GLI4
1549.333	1621.829	1747.526	2607.833	2815.691	2500.842	-0.68803	2.60E-20	CNOT6
290.2753	273.4421	302.0528	80.91128	93.40161	104.7307	1.634381	2.60E-20	ADIRF-AS1
206.6976	160.4986	175.2089	413.8923	472.2554	494.0326	-1.34558	2.61E-20	SNHG4
2013.055	1961.65	2034.978	1221.968	1322.315	1286.389	0.649869	2.64E-20	RAD23A
361.2715	355.6729	332.1668	135.8895	119.638	86.74663	1.615159	2.71E-20	SIGIRR
163.5607	140.684	113.1557	363.0634	404.0407	406.2281	-1.49075	2.72E-20	ANAPC10
392.7255	378.4597	375.969	117.2176	157.4185	150.2198	1.433559	2.74E-20	CRTC1
566.1717	555.8008	537.4897	989.6072	1114.523	960.5602	-0.88504	2.76E-20	KLHL42
5396.605	5671.942	5334.745	3816.315	3818.972	3469.865	0.56261	2.79E-20	ACADVL
1427.112	1564.366	1458.249	2839.156	2504.003	2290.322	-0.779	2.90E-20	MDH1
1412.733	1211.665	1229.2	705.3804	711.5314	739.4621	0.838037	2.97E-20	TGIF1
845.6628	767.8174	861.4436	1555.986	1357.997	1417.567	-0.80698	2.99E-20	ADAMTS1
271.403	291.2753	251.8628	575.7149	575.1021	573.374	-1.08279	3.03E-20	RIOX2
230.9621	257.5904	215.3609	63.27677	72.41249	56.06794	1.875249	3.04E-20	STK32C
2954.877	3047.492	3150.11	4292.447	4885.219	4922.342	-0.62343	3.04E-20	NF1
149.1818	146.6284	155.1329	351.6529	408.2385	389.3019	-1.34917	3.06E-20	MND1
316.3372	313.0714	248.2126	656.6262	640.1684	624.1525	-1.13079	3.11E-20	PIP4K2A
404.4084	414.1261	401.5203	734.4255	797.5868	791.2985	-0.92939	3.15E-20	CYB5R4
498.7703	470.5978	525.6266	866.1657	1061	1131.938	-1.03238	3.17E-20	TRIP11
2728.409	2758.198	2580.681	1758.264	1603.569	1796.29	0.645151	3.26E-20	GDI1
554.4888	596.4208	557.5657	264.5177	282.3038	253.8926	1.092924	3.33E-20	PJA1
428.6729	443.848	403.3454	192.9423	147.9733	153.3934	1.367292	3.39E-20	TEAD3
6657.461	7499.843	7244.704	10195.86	10331.9	10209.65	-0.52239	3.41E-20	EIF4B
1469.35	1452.413	1345.093	887.9495	805.9825	780.7196	0.785723	3.67E-20	BRI3
175.2436	145.6376	175.2089	398.3325	408.2385	429.5016	-1.31523	3.67E-20	ZC3H8
168.9528	158.5171	161.5207	37.34367	29.38478	31.73657	2.311875	3.86E-20	RASL11A

939.1261	854.0111	791.1776	427.3775	421.8815	465.4697	0.975264	3.89E-20	SSBP3
1413.632	1403.867	1350.568	856.8297	771.3504	846.3085	0.752176	4.00E-20	SPATA20
492.4795	459.6997	568.5163	928.4051	1021.121	991.2389	-0.95061	4.02E-20	MAP4K3
324.4254	336.8489	356.8056	685.6713	704.1852	645.3103	-0.99935	4.02E-20	MSH3
809.7154	819.3355	727.2993	403.5191	392.4967	416.807	0.957974	4.03E-20	EMP2
402.611	417.0982	422.5089	185.681	132.2315	151.2777	1.404186	4.19E-20	ARRDC1
666.8245	662.7998	691.71	1127.571	1226.814	1137.227	-0.78843	4.28E-20	PLEKHA1
1802.763	1807.095	1767.602	1159.728	1021.121	936.2288	0.786513	4.31E-20	NECTIN1
1290.512	1165.101	1258.401	1903.49	2020.203	2131.64	-0.7047	4.37E-20	BLOC1S6
640.7626	628.1242	649.7329	1097.489	1111.374	1079.043	-0.77692	4.46E-20	SLC37A3
238.1516	227.8684	253.6878	483.393	580.3494	634.7314	-1.23802	4.48E-20	ATAD2B
266.9095	300.1918	270.1137	99.58312	87.10488	75.10988	1.676017	4.52E-20	KIAA0895L
832.1826	855.0019	897.0329	1534.202	1428.31	1382.657	-0.74975	4.64E-20	INTS14
167.1555	191.2113	161.5207	43.56761	27.28586	30.67868	2.354571	4.67E-20	SCARA5
672.2166	634.0686	658.8584	1100.601	1118.72	1158.385	-0.78098	4.67E-20	AMMECR1L
178.8384	220.9333	184.3343	32.15705	52.47282	40.19966	2.225926	4.76E-20	-
445.7479	411.1539	470.8738	834.0086	880.4939	811.3983	-0.92702	4.76E-20	TMEM237
965.188	904.5385	965.4739	515.5501	522.6293	426.3279	0.953086	4.78E-20	PIP5K1C
766.5785	742.0584	704.4857	300.824	395.645	346.9865	1.084925	4.83E-20	PLD1
1891.733	2070.63	1953.761	3048.696	2922.736	2944.096	-0.59204	4.84E-20	PVR
2595.403	2614.542	2602.582	3845.361	3683.592	3704.716	-0.52402	4.84E-20	PIP5K1A
1933.072	1819.975	1895.358	2882.724	2919.588	3423.318	-0.70757	4.95E-20	PDCD5
2432.741	2319.304	2659.16	3646.194	3868.296	3922.64	-0.62566	5.02E-20	PHLDB2
196.8121	228.8591	215.3609	42.53029	57.7201	60.29948	1.997581	5.06E-20	-
483.4927	477.5329	440.7598	838.1579	860.5542	911.8975	-0.89722	5.10E-20	CCZ1B
1470.249	1389.997	1408.059	851.6431	840.6145	894.9713	0.722492	5.18E-20	FOXC1
1448.681	1365.229	1559.541	2215.724	2444.184	2501.9	-0.71113	5.21E-20	RABGGTB
474.5058	498.3383	446.2351	215.7634	191.0011	154.4513	1.337521	5.32E-20	PMM1
4578.801	4937.809	4905.848	6665.845	6936.906	6869.91	-0.5055	5.37E-20	SF3B3
573.3612	658.8369	557.5657	283.1895	251.8695	200.9983	1.280901	5.48E-20	ETFB
249.8345	218.9518	219.9236	72.61269	57.7201	46.54697	1.960577	5.59E-20	TRPT1
1334.548	1358.294	1279.39	815.3368	796.5374	759.5619	0.743948	5.61E-20	STAT5B
4187.873	3989.678	4015.203	1213.669	840.6145	716.1886	2.137751	5.65E-20	CYBA
348.6899	308.1177	324.8665	130.7028	107.0445	97.32548	1.550663	5.74E-20	RHOF
645.256	555.8008	698.0979	1106.825	1264.595	1323.415	-0.95916	5.89E-20	MOB1B
1530.461	1548.514	1623.42	2433.562	2329.793	2382.359	-0.60364	6.28E-20	BLMH
397.2189	371.5246	396.0451	759.3213	716.7787	752.1567	-0.9353	6.40E-20	ANAPC4
2146.959	2235.092	2112.544	3416.946	3243.87	3108.068	-0.5892	6.49E-20	MTHFD1L
648.8508	619.2076	623.2691	334.0184	287.551	289.8607	1.053115	6.54E-20	PPARD
1116.167	1086.833	1114.219	1726.107	1706.416	1826.969	-0.66475	6.60E-20	WDR70
1338.142	1430.617	1230.112	2137.925	2155.583	2125.292	-0.6832	6.73E-20	CKS1B
478.9993	419.0797	402.4329	183.6064	164.7646	126.9463	1.451863	6.81E-20	SNAPC2
964.2893	1037.297	1013.839	1793.533	1707.465	1552.976	-0.74544	7.07E-20	METTL16
821.3983	746.0213	857.7934	1378.604	1399.975	1345.631	-0.76532	7.39E-20	UBQLN2
525.7309	479.5144	503.7255	874.4642	972.846	1087.506	-0.95907	7.42E-20	ODR4
1526.866	1554.459	1498.401	882.7628	917.2248	984.8916	0.717662	7.85E-20	PTGR1
2931.511	2739.374	2724.863	1866.146	1664.438	1509.603	0.736139	7.92E-20	FOSL1
221.0766	268.4884	223.5738	73.65001	48.27499	63.47314	1.942285	7.99E-20	DNASE1L1
257.9227	258.5811	267.376	79.87396	83.95651	88.8624	1.633535	8.16E-20	C12orf57
134.8028	128.7952	114.9808	20.74648	14.69239	15.86829	2.882398	8.18E-20	C3orf18
291.174	351.7099	334.9045	123.4416	112.2918	103.6728	1.525518	8.26E-20	ARHGEF19
457.4308	461.6812	515.5886	202.2782	216.188	209.4614	1.192341	8.40E-20	HDAC4
178.8384	136.721	177.034	481.3184	458.6124	380.8388	-1.4209	8.41E-20	LSM11
642.56	680.633	704.4857	1213.669	1156.501	1129.822	-0.78774	8.52E-20	TUBGCP3

458.3295	518.1529	542.965	964.7114	962.3515	909.7817	-0.90118	8.60E-20	ABHD13
2406.679	2514.478	2544.179	3893.077	3673.097	3541.801	-0.57345	8.80E-20	C8orf33
1526.866	1673.347	1666.309	2662.811	2648.828	2397.169	-0.66391	9.01E-20	MIEF1
332.5136	305.1455	286.5395	118.255	86.05542	74.052	1.730506	9.22E-20	PUSL1
1046.07	1115.564	1085.017	1652.457	1817.658	1766.669	-0.68997	9.36E-20	LARP4B
1979.804	2059.732	1936.423	1318.439	1121.869	1104.433	0.753196	9.41E-20	CNP
292.9714	324.9602	321.2163	602.6853	642.2673	695.0309	-1.04708	9.43E-20	ZEB1
416.99	431.9592	464.486	850.6058	799.6857	812.4562	-0.90692	9.44E-20	FECH
131.2081	113.9342	136.8819	20.74648	10.49456	4.231543	3.425277	9.51E-20	BARX2
1546.637	1625.792	1493.838	980.2713	938.214	947.8656	0.702737	9.52E-20	DGKD
696.4811	731.1603	703.5731	358.9142	350.5184	386.1283	0.959872	9.93E-20	IFIH1
157.2699	137.7118	151.4827	369.2874	382.0021	366.0284	-1.32218	1.01E-19	LINC01128
447.5453	507.2549	498.2502	971.9727	893.0874	866.4084	-0.9113	1.01E-19	DEPDC1B
878.9142	953.0844	922.5842	1453.291	1582.58	1501.14	-0.72027	1.05E-19	MED14
368.461	401.2465	399.6952	718.8656	778.6966	746.8673	-0.94099	1.06E-19	ZBTB24
2260.193	2138	2403.647	3313.213	3539.816	3474.097	-0.60213	1.11E-19	FAM199X
775.5654	774.7526	719.0864	409.743	391.4472	388.244	0.931813	1.16E-19	PRKD2
470.9111	431.9592	444.41	208.5022	173.1603	149.1619	1.34349	1.21E-19	NOL3
508.6559	456.7275	536.5772	870.315	1033.715	1030.381	-0.96532	1.21E-19	MYSM1
1318.371	1204.73	1234.675	653.5142	728.3227	756.3883	0.813893	1.24E-19	ARFGAP3
1255.463	1277.054	1167.147	729.2389	624.4265	721.478	0.83398	1.32E-19	HDGFL2
597.6257	693.5125	610.4934	1210.557	1065.198	1210.221	-0.87521	1.34E-19	GAR1
594.031	584.532	584.0296	1108.9	1038.962	978.5442	-0.82684	1.37E-19	OGFOD1
212.9884	189.2298	188.8971	52.90353	18.89021	28.56291	2.55685	1.37E-19	ZNF358
799.8299	867.8814	827.6794	1374.454	1334.908	1460.94	-0.74129	1.42E-19	LRP12
1321.966	1271.109	1276.652	810.1502	695.7896	667.5259	0.832115	1.45E-19	IGFBP7
2024.738	2081.528	2061.442	3025.875	2974.159	2970.543	-0.54058	1.46E-19	YARS
275.8964	290.2845	314.8284	624.4691	614.9814	578.6635	-1.04527	1.48E-19	HUS1
1551.131	1629.754	1658.096	1001.018	819.6254	736.2884	0.919997	1.49E-19	POLRMT
507.7572	457.7183	514.6761	895.2107	902.5325	899.2028	-0.86474	1.56E-19	TBC1D31
258.8214	322.9787	311.1783	631.7304	610.7836	651.6576	-1.08607	1.58E-19	ACADSB
458.3295	402.2373	438.9347	803.9262	814.3781	958.4444	-0.9867	1.63E-19	ITGB3BP
633.5731	649.9203	714.5237	1097.489	1246.754	1225.032	-0.83685	1.66E-19	ZYG11B
541.9072	523.1066	553.9156	924.2558	960.2526	980.66	-0.82318	1.66E-19	CBLL1
148.2831	132.7581	157.8705	402.4818	354.7162	366.0284	-1.35441	1.75E-19	PDSS1
151.8778	143.6562	149.6576	31.11972	28.33532	24.33137	2.409257	1.77E-19	PAGR1
721.6443	789.6135	626.9193	340.2423	237.1771	198.8825	1.460844	1.79E-19	C9orf16
4154.622	4115.501	3841.82	2814.26	2699.202	2502.958	0.595291	1.87E-19	GRINA
671.3179	696.4847	653.3831	307.0479	221.4353	153.3934	1.56689	1.89E-19	TMEM129
674.014	752.9565	710.8735	379.6606	344.2217	362.8548	0.975616	1.90E-19	SWI5
500.5677	517.1622	487.2997	855.7924	992.7857	933.0552	-0.88638	1.94E-19	PKP2
212.9884	169.4152	196.1974	455.3853	518.4314	433.7331	-1.28086	1.99E-19	SPAG1
638.0665	762.8638	700.8355	1205.371	1312.87	1219.742	-0.83152	2.01E-19	DDI2
347.7912	325.9509	337.6421	673.2234	627.5749	715.1307	-0.99461	2.10E-19	COQ2
468.215	481.4958	473.6115	195.0169	188.9021	116.3674	1.507767	2.13E-19	DALRD3
547.2993	581.5598	556.6532	1160.766	959.2031	1014.512	-0.8955	2.17E-19	GPN1
248.0371	225.8869	229.9616	78.83663	48.27499	43.37331	2.044673	2.18E-19	ZNF517
230.9621	188.2391	198.9351	463.6839	467.0081	492.9747	-1.20217	2.28E-19	SLC27A2
3663.94	3558.71	3642.885	4956.335	5148.633	5088.43	-0.48358	2.29E-19	SMARCC1
452.0387	567.6895	498.2502	227.174	216.188	202.0562	1.2328	2.31E-19	ADAM19
1278.829	1305.785	1190.873	666.9994	664.3059	466.5276	1.070054	2.32E-19	PNKD
602.1191	569.671	601.368	971.9727	1107.176	1091.738	-0.83809	2.48E-19	CCNT2
2065.179	2146.917	2060.529	1408.686	1203.726	1193.295	0.720643	2.49E-19	BLCAP
836.676	905.5292	804.8658	457.4599	464.9092	398.8229	0.946424	2.50E-19	XYLT2

298.3635	302.1733	309.3532	616.1705	578.2504	679.1626	-1.04195	2.55E-19	MBIP
186.9265	160.4986	173.3838	394.1832	413.4858	484.5116	-1.30967	2.55E-19	DOPEY1
319.0333	287.3123	331.2543	644.1783	605.5363	656.947	-1.02299	2.61E-19	TBP
657.8376	699.4569	635.1322	340.2423	240.3255	217.9244	1.318432	2.68E-19	AP1M2
502.3651	525.0881	444.41	227.174	195.1989	213.6929	1.209836	2.69E-19	RGS19
1300.398	1307.766	1236.5	1940.833	1989.769	1979.304	-0.62041	2.73E-19	BICD2
643.4587	608.3096	690.7975	1063.257	1255.15	1232.437	-0.86961	2.78E-19	APPL1
1196.15	1131.416	1199.998	1974.028	1914.208	1764.553	-0.68006	2.81E-19	RAE1
789.0457	945.1585	835.8923	453.3106	362.0624	433.7331	1.040273	2.88E-19	MRPL34
232.7595	271.4606	277.4141	537.3339	616.0309	554.3321	-1.12816	2.89E-19	PAPOLG
429.5716	447.8109	444.41	180.4944	201.4956	142.8146	1.332219	2.89E-19	AKR1C3
656.0403	634.0686	623.2691	339.205	312.738	283.5134	1.032185	2.90E-19	MPLKIP
328.0201	341.8026	321.2163	607.8719	707.3336	780.7196	-1.08076	2.96E-19	PMM2
1185.366	1208.693	1029.352	656.6262	494.2939	485.5695	1.064371	3.05E-19	ERF
920.2537	887.696	882.4322	1438.769	1436.706	1437.667	-0.68079	3.06E-19	ABCB7
1047.867	1104.666	1128.82	1656.607	1837.598	1903.136	-0.718	3.07E-19	RSF1
425.0781	378.4597	326.6915	142.1134	135.3799	143.8725	1.423598	3.12E-19	STRA6
268.7069	245.7016	272.8513	532.1473	623.3771	544.8111	-1.11006	3.15E-19	GSTCD
267.8082	321.988	281.9768	91.28452	108.094	86.74663	1.606446	3.22E-19	AC004817.3
1625.722	1718.92	1563.192	2393.107	2719.141	2755.792	-0.68113	3.26E-19	MAL2
529.3256	514.19	442.5849	227.174	184.7043	149.1619	1.404667	3.26E-19	ZBTB45
1605.951	1715.948	1622.507	1038.361	1042.11	925.65	0.71755	3.32E-19	MTCL1
2801.202	2722.532	2928.361	4614.018	4443.398	3958.608	-0.62283	3.35E-19	TCOF1
182.4331	171.3967	135.9694	31.11972	36.73097	24.33137	2.409011	3.40E-19	PSENEN
389.1307	362.608	372.3189	147.3	159.5174	150.2198	1.298686	3.40E-19	CEP131
140.1949	153.5635	156.9579	33.19437	28.33532	24.33137	2.391101	3.42E-19	AKR1B10
9616.831	9750.786	9610.937	15199.91	13016.41	13995.83	-0.5427	3.45E-19	RPS6
1223.111	1227.517	1249.276	1822.579	1988.72	1986.709	-0.64798	3.46E-19	STK24
1642.797	1488.08	1597.868	2323.606	2553.327	2508.247	-0.64273	3.48E-19	TIAL1
702.7719	726.2067	701.748	351.6529	390.3978	367.0863	0.94181	3.53E-19	SLC35E2B
1314.777	1323.618	1357.869	1999.961	2030.698	2033.256	-0.60155	3.57E-19	RBPJ
322.628	329.9138	342.2048	616.1705	664.3059	722.5359	-1.00963	3.58E-19	C1GALT1C1
964.2893	910.4829	992.8503	1518.643	1533.256	1555.092	-0.68351	3.64E-19	CEBPG
335.2096	336.8489	333.0794	633.805	642.2673	690.7993	-0.96849	3.66E-19	C1D
349.5886	369.5431	376.8816	693.9698	678.9983	738.4042	-0.94609	3.90E-19	N4BP2
1120.661	1122.5	1253.839	1839.176	1881.675	2104.135	-0.73587	3.97E-19	SCAF8
781.8562	817.354	854.1433	1287.319	1456.645	1415.451	-0.76162	4.02E-19	NAA35
5578.14	6024.642	5982.653	8156.48	8423.986	8054.742	-0.48645	4.18E-19	RBBP7
292.0727	320.0065	271.0262	100.6204	104.9456	101.557	1.523151	4.18E-19	NAT6
3657.649	3824.226	3972.314	5316.286	5717.438	6072.264	-0.57862	4.21E-19	RAB31
674.014	696.4847	647.9078	323.6451	366.2603	329.0024	0.985995	4.25E-19	SVIL
834.8786	883.7331	845.0178	482.3557	471.2059	412.5754	0.907643	4.31E-19	ABCB8
2293.445	2494.664	2525.928	3602.627	3992.132	3594.696	-0.61355	4.40E-19	OXSR1
905.8748	922.3717	892.4702	513.4754	456.5135	522.5955	0.866065	4.42E-19	ZNF652
2436.336	2573.922	2407.297	1682.54	1467.14	1576.25	0.650103	4.84E-19	EIF3G
1241.983	1203.74	1301.291	2007.222	1911.06	1911.599	-0.63748	4.89E-19	GLOD4
4744.16	4654.46	4639.385	6273.736	6576.943	6498.592	-0.46289	4.89E-19	PERP
1986.094	2094.408	2083.343	3062.181	3127.38	2920.822	-0.56386	5.08E-19	PHF20
212.9884	236.785	230.8742	541.4832	472.2554	488.7432	-1.14317	5.13E-19	LIN52
1889.036	2046.853	2027.678	2875.463	3005.643	2993.816	-0.57377	5.15E-19	PDCD11
1434.302	1460.339	1630.72	2299.748	2439.986	2377.069	-0.65303	5.16E-19	UBA6
1422.619	1507.894	1262.964	827.7847	775.5482	682.3363	0.875057	5.17E-19	SSR4
264.2135	243.7201	241.8247	71.57537	58.76956	88.8624	1.774755	5.17E-19	PDZD4
6033.773	6123.716	5910.562	9267.454	8739.872	8113.983	-0.53186	5.27E-19	LAPTM4B

904.9761	879.7702	948.1355	1439.806	1548.998	1458.824	-0.70231	5.32E-19	DCUN1D4
1498.108	1560.403	1484.713	2250.993	2266.826	2288.207	-0.58334	5.39E-19	MAPK9
588.6389	625.152	644.2576	1160.766	1079.891	1029.323	-0.81576	5.46E-19	NCOA5
119.5251	128.7952	139.6196	21.78381	22.03858	16.92617	2.674221	5.76E-19	SYT12
548.198	541.9305	556.6532	267.6296	259.2157	273.9924	1.040196	5.79E-19	CELSR3
150.9791	141.6747	146.0074	32.15705	9.445107	14.8104	2.956638	6.07E-19	TMEM238
699.1771	754.9379	766.5388	1186.699	1389.48	1504.313	-0.87784	6.38E-19	UTP3
94.36195	101.0547	111.3306	7.261269	1.049456	1.057886	5.024614	6.41E-19	IFI27
470.0124	484.468	548.4403	1022.802	930.8678	884.3924	-0.91704	6.43E-19	DDX31
262.4161	313.0714	287.4521	561.1924	668.5037	612.5158	-1.09518	6.63E-19	ZNF322
16.17634	20.80538	27.37639	129.6655	137.4788	126.9463	-2.61468	6.88E-19	STXBP6
570.6652	559.7637	625.0942	969.8981	1203.726	1268.405	-0.97094	7.08E-19	SRGAP1
2843.44	2812.689	2947.524	4138.923	4190.479	3964.956	-0.5149	7.31E-19	PGRMC1
486.1887	533.0139	471.7864	859.9417	917.2248	983.8337	-0.88957	7.45E-19	IFT57
891.4958	906.5199	927.147	530.0726	515.2831	478.1643	0.838786	7.62E-19	C9orf3
780.0588	764.8452	756.5008	441.9001	372.557	373.4336	0.953898	7.74E-19	PITPNM2
710.8601	650.911	636.0447	350.6156	274.9576	240.14	1.206158	7.87E-19	PIGQ
1516.082	1469.256	1386.158	927.3678	864.752	876.9872	0.711694	8.27E-19	DDA1
614.7007	601.3744	636.9573	1136.907	1025.319	1051.538	-0.7942	8.47E-19	SRSF8
454.7348	509.2363	445.3226	811.1875	989.6373	935.1709	-0.9578	8.71E-19	VPS41
710.8601	674.6886	730.9495	1154.542	1254.1	1174.253	-0.759	8.71E-19	SLMAP
915.7603	931.2882	1013.839	1470.926	1693.823	1658.765	-0.75343	8.88E-19	ME1
368.461	371.5246	395.1325	695.0072	737.7678	711.9571	-0.91777	8.97E-19	USPL1
1267.146	1089.805	1128.82	688.7832	617.0803	557.5058	0.903708	9.44E-19	UPP1
390.0294	441.8666	455.3606	773.8438	817.5265	890.7397	-0.94762	9.66E-19	MSANTD4
115.0317	127.8045	114.9808	18.67183	9.445107	17.98406	2.955529	9.93E-19	GPER1
1194.353	1120.518	1297.641	1952.244	1922.604	1897.847	-0.67577	1.01E-18	DESI2
1499.906	1615.884	1556.804	880.6882	671.6521	520.4798	1.172256	1.03E-18	RPL15P3
434.065	481.4958	495.5126	1039.399	824.8727	912.9553	-0.97727	1.04E-18	TPRKB
514.9467	553.8193	485.4746	948.1143	888.8895	1014.512	-0.87615	1.05E-18	LARP7
1007.426	1091.787	1022.052	608.9093	508.9863	437.9647	1.003871	1.06E-18	HS6ST1
243.5437	246.6923	236.3495	72.61269	86.05542	64.53103	1.70251	1.06E-18	FOXQ1
968.7827	958.038	911.6337	559.1177	505.838	449.6014	0.905902	1.07E-18	ABHD12
609.3086	636.0501	663.4211	1146.243	1065.198	1068.465	-0.78114	1.08E-18	MEAF6
6434.587	6691.405	6646.987	8789.247	9966.687	9921.91	-0.53642	1.08E-18	SMS
617.3968	609.3003	544.7901	289.4134	296.9961	261.2978	1.063027	1.09E-18	B3GNT3
305.553	334.8675	323.9539	605.7973	669.5531	633.6735	-0.98573	1.11E-18	THOC1
430.4702	349.7285	319.3912	96.47114	131.182	137.5251	1.591218	1.12E-18	TTLL3
461.9242	479.5144	437.1096	215.7634	185.7538	208.4035	1.176061	1.15E-18	OLFML2A
476.3032	539.949	459.0107	883.8002	892.0379	938.3446	-0.88054	1.15E-18	DHX57
115.0317	104.0269	83.95425	11.41057	5.247282	4.231543	3.854848	1.21E-18	C15orf59
1871.961	1894.28	1854.294	1213.669	1088.286	937.2867	0.79482	1.26E-18	CCDC124
939.1261	962.9917	1012.014	1686.689	1576.283	1496.908	-0.70792	1.31E-18	SPTY2D1
416.99	393.3207	448.0602	733.3882	844.8124	855.8295	-0.95106	1.38E-18	FAM210A
682.1021	728.1882	667.0713	368.2501	367.3097	345.9286	0.941253	1.39E-18	TOX2
393.6242	378.4597	336.7296	139.0014	89.20379	58.18371	1.95181	1.42E-18	NPEPL1
1398.354	1584.181	1475.587	2232.322	2345.535	2330.522	-0.63239	1.43E-18	POLR1A
544.6033	532.0232	527.4517	271.7789	251.8695	251.7768	1.048611	1.44E-18	MKL1
622.7889	674.6886	627.8318	1094.377	1174.342	1056.828	-0.78906	1.46E-18	NGLY1
1240.186	1325.6	1207.299	713.679	560.4097	446.4278	1.132431	1.47E-18	ANAPC11
3363.779	3369.48	3349.045	4555.928	4801.263	4724.517	-0.48199	1.48E-18	MOB1A
1793.776	1946.789	1791.328	1192.923	1001.181	920.3605	0.828396	1.48E-18	E2F1
623.6876	586.5134	560.3034	312.2346	264.463	264.4714	1.073531	1.48E-18	INO80E
603.0178	633.0779	688.9724	1197.072	1058.901	1142.517	-0.82001	1.48E-18	TXNDC9

226.4687	239.7572	196.1974	72.61269	43.02771	42.31543	2.066603	1.49E-18	PDLIM4
28.75793	34.67563	25.55129	125.5162	179.457	158.6829	-2.38457	1.51E-18	ZNF804A
140.1949	137.7118	124.1063	21.78381	22.03858	26.44714	2.516646	1.52E-18	FAM43A
1276.133	1375.136	1398.933	856.8297	778.6966	778.6039	0.746215	1.53E-18	ALDH2
1142.229	1184.916	1162.584	732.3508	663.2564	632.6156	0.782586	1.53E-18	AC007318.1
219.2792	208.0538	203.4978	59.12748	65.06629	41.25754	1.930345	1.58E-18	IDUA
940.0248	960.0195	915.2839	542.5205	380.9527	387.1862	1.10257	1.61E-18	ZNF316
487.9861	415.1168	380.5318	159.7479	180.5065	175.609	1.315673	1.63E-18	SLC2A13
592.2336	613.2632	605.0181	1012.428	1123.968	1010.281	-0.79761	1.66E-18	NVL
363.9675	336.8489	369.5812	705.3804	701.0368	652.7155	-0.94326	1.66E-18	HEATR3
142.891	161.4893	187.9845	391.0712	412.4363	406.2281	-1.29696	1.67E-18	FANCB
347.7912	346.7563	349.5052	147.3	125.9348	141.7567	1.330961	1.69E-18	KDELR3
511.3519	461.6812	438.0222	224.062	149.0228	158.6829	1.407529	1.69E-18	SPHK1
2009.46	2094.408	2274.978	3115.084	3287.947	3209.625	-0.5916	1.71E-18	NAA15
186.0279	222.9147	201.6727	453.3106	459.6619	451.7172	-1.16171	1.76E-18	RFFL
82.67905	111.9527	104.0303	4.149297	5.247282	0	4.984638	1.76E-18	EPS8L3
189.6226	173.3781	200.7602	394.1832	478.5521	461.2382	-1.24144	1.77E-18	TRMT13
1020.906	990.7322	1033.002	1555.986	1715.861	1591.06	-0.67532	1.78E-18	COPS8
189.6226	179.3225	147.8325	43.56761	22.03858	34.91023	2.361258	1.79E-18	ADM2
834.8786	913.4551	803.0407	484.4304	428.1782	449.6014	0.90473	1.89E-18	DMKN
98.85538	81.24004	96.7299	6.223945	4.197825	4.231543	4.23886	1.90E-18	SHISA4
1140.432	1217.61	1186.31	1769.675	1892.17	1842.837	-0.6354	1.94E-18	GFM2
848.3589	883.7331	897.0329	463.6839	453.3651	521.5376	0.869987	1.98E-18	CDYL2
724.3403	931.2882	866.9189	431.5268	397.744	322.6551	1.130028	2.01E-18	DMWD
984.9591	1143.305	1019.314	1655.569	1730.554	1750.801	-0.70737	2.04E-18	SH2D4A
471.8098	477.5329	507.3757	909.7333	880.4939	829.3824	-0.84659	2.09E-18	GATAD1
140.1949	147.6191	155.1329	29.04508	33.5826	17.98406	2.457097	2.10E-18	SLC25A35
414.2939	485.4588	395.1325	168.0465	76.61031	76.16777	2.011628	2.11E-18	ZNF428
1689.528	1844.743	1858.857	2690.819	2643.581	2820.323	-0.59671	2.13E-18	ZWINT
319.0333	377.469	323.9539	690.8579	647.5146	684.452	-0.98852	2.18E-18	GINS3
1347.129	1421.701	1366.994	879.6509	774.4988	843.1349	0.727547	2.19E-18	SCARA3
307.3504	285.3309	340.3797	604.76	638.0695	635.7893	-1.00864	2.22E-18	EIF4EP2
1552.928	1658.486	1616.119	2343.315	2426.343	2421.5	-0.57518	2.26E-18	RTF1
1680.541	1641.643	1737.488	2495.802	2541.783	2444.774	-0.56427	2.28E-18	PNRC2
939.1261	892.6497	918.0215	1418.022	1569.987	1465.172	-0.69521	2.28E-18	RAP1A
1244.679	1309.748	1251.101	1886.893	1939.395	2038.546	-0.62422	2.29E-18	KNSTRN
2419.261	2544.2	2752.239	3871.294	3823.169	3744.915	-0.56815	2.31E-18	THRAP3
919.355	986.7692	937.185	1539.389	1470.288	1510.661	-0.6693	2.32E-18	RAPGEF2
1495.412	1542.57	1624.332	2343.315	2333.991	2312.538	-0.58422	2.36E-18	CHAF1A
232.7595	280.3772	265.5509	562.2297	540.47	535.2902	-1.07407	2.40E-18	XRCC4
200.4068	177.3411	167.9085	51.86621	34.63206	41.25754	2.094241	2.41E-18	PRAF2
182.4331	186.2576	166.0834	51.86621	33.5826	27.50503	2.241471	2.42E-18	SYT7
1196.15	1291.915	1291.253	713.679	747.2129	790.2406	0.747386	2.47E-18	PDCD4
1615.836	1536.626	1623.42	2355.763	2514.497	2334.754	-0.59298	2.50E-18	RYK
1301.296	1305.785	1324.105	2073.611	2083.171	1902.078	-0.62412	2.51E-18	FTSJ1
124.0186	134.7396	126.8439	25.9331	7.346194	12.69463	3.065361	2.54E-18	GAGE1
2146.959	2286.61	2227.525	3891.003	3195.595	3480.444	-0.66593	2.55E-18	PTTG1
1925.883	1962.64	1958.324	2931.478	2841.928	2750.503	-0.54396	2.60E-18	FAM83D
883.4076	814.3819	824.0292	468.8705	453.3651	476.0486	0.851107	2.63E-18	GNE
841.1694	943.177	887.9075	497.9156	502.6896	430.5595	0.900295	2.68E-18	NR2F1
328.9188	323.9694	374.1439	630.6931	715.7292	701.3782	-0.99499	2.72E-18	MED6
418.7873	448.8017	507.3757	814.2994	890.9884	901.3186	-0.92265	2.72E-18	CEP192
1429.808	1437.552	1287.603	845.4192	638.0695	607.2264	0.99048	2.73E-18	SH3GLB2
456.5321	473.57	468.1362	782.1424	905.6808	889.6819	-0.88246	2.73E-18	RASA2

88.96984	91.14736	96.7299	6.223945	6.296738	7.4052	3.797006	2.74E-18	BAIAP3
11689.2	12671.46	11062.8	8081.792	6754.301	5995.038	0.765882	2.74E-18	KRT81
158.1686	155.545	167.9085	352.6902	392.4967	414.6912	-1.26746	2.76E-18	ARL13B
248.0371	215.9796	197.11	66.38874	31.48369	25.38926	2.421473	2.77E-18	CBARP
1800.067	1670.374	1712.849	1115.123	1127.116	1064.233	0.648763	2.80E-18	BTG1
1042.475	976.8619	954.5233	558.0804	419.7825	354.3917	1.158154	2.96E-18	BAX
237.2529	208.0538	219.9236	487.5423	498.4918	457.0066	-1.11627	2.97E-18	SLC20A2
1669.757	1763.503	1607.906	1082.966	1014.824	900.2607	0.749351	2.98E-18	PTPN23
567.0704	540.9398	569.4288	1082.966	1007.478	918.2448	-0.84267	2.98E-18	CENPW
1443.289	1407.83	1535.815	2179.418	2239.54	2217.328	-0.59689	3.03E-18	SART3
528.4269	515.1807	486.3871	251.0324	195.1989	159.7407	1.335584	3.12E-18	FAM53B
510.4532	411.1539	507.3757	862.0164	922.4721	992.2968	-0.95705	3.13E-18	CAAP1
1269.842	1293.896	1340.53	1956.393	2280.469	2025.851	-0.6817	3.14E-18	ABI2
741.4154	814.3819	902.5082	1331.924	1518.563	1475.751	-0.81546	3.20E-18	C5orf51
418.7873	413.1353	477.2617	773.8438	846.9113	834.6718	-0.90676	3.24E-18	MIER3
1078.422	1180.953	1093.23	1765.526	1835.499	1697.907	-0.66093	3.28E-18	ZNF185
608.4099	722.2438	662.5086	1326.738	1261.447	1087.506	-0.88381	3.29E-18	PSMD10
640.7626	628.1242	665.2462	1100.601	1131.314	1042.017	-0.75913	3.35E-18	AP1AR
826.7905	713.3272	793.9152	1298.73	1330.711	1286.389	-0.74567	3.36E-18	FAM126A
307.3504	313.0714	298.4026	615.1332	567.7559	668.5837	-1.011	3.44E-18	AC007969.1
743.2127	784.6599	699.0104	401.4444	400.8923	349.1023	0.951048	3.46E-18	EML3
2379.719	2253.916	2091.556	1336.073	1418.865	1485.271	0.665547	3.49E-18	COX5A
1112.572	1123.49	1253.839	1804.944	2012.857	1846.011	-0.69839	3.61E-18	PRKD3
396.3202	373.506	375.969	707.4551	690.5423	796.5879	-0.93719	3.88E-18	DUSP11
107.8422	112.9435	103.1177	9.335917	17.84076	7.4052	3.227001	3.89E-18	-
527.5283	641.0037	546.6152	270.7416	274.9576	257.0662	1.094357	3.91E-18	PPARA
995.7433	1107.639	1187.223	1732.331	1944.643	1815.332	-0.7392	3.97E-18	HELZ
1232.996	1279.035	1263.877	780.0678	792.3395	745.8094	0.703625	4.00E-18	PLEKHA6
748.6048	842.1224	874.2193	1369.268	1444.052	1341.399	-0.75353	4.00E-18	PDE8A
1341.737	1433.589	1572.317	2173.194	2595.306	2620.383	-0.76506	4.16E-18	DICER1
1650.885	1672.356	1572.317	2570.489	2421.096	2371.78	-0.5891	4.38E-18	KCMF1
71.89482	80.24931	85.77934	255.1817	298.0456	217.9244	-1.69704	4.52E-18	FLRT2
548.198	525.0881	530.1894	892.0988	939.2634	952.0971	-0.79537	4.73E-18	MTMR1
2050.8	2272.74	2098.856	3056.994	3332.024	3499.486	-0.6229	4.85E-18	RUVBL1
577.8546	598.4022	585.8547	302.8986	306.4413	267.6451	1.006347	4.99E-18	PRKCD
1111.674	1181.943	1077.717	665.9621	686.3445	631.5577	0.76461	5.08E-18	BIN1
1924.085	1926.974	1930.948	2796.626	2892.302	2713.477	-0.53925	5.09E-18	MPZL1
1049.664	1162.129	1100.531	1703.286	1708.515	1812.158	-0.65774	5.14E-18	RRP36
723.4417	723.2345	675.2842	402.4818	332.6777	340.6392	0.97962	5.22E-18	DNASE2
230.9621	220.9333	248.2126	493.7663	502.6896	480.2801	-1.07611	5.24E-18	AC245041.2
200.4068	215.9796	225.3989	58.09015	71.36303	46.54697	1.86605	5.41E-18	HRCT1
424.1795	398.2743	437.1096	747.9107	802.8341	761.6777	-0.87586	5.41E-18	SPRYD7
84.47642	104.0269	102.2052	5.186621	9.445107	0	4.308723	5.42E-18	-
383.7386	387.3763	393.3074	685.6713	755.6086	723.5938	-0.89461	5.48E-18	ZFYVE9
948.113	949.1214	1019.314	1487.523	1622.46	1558.266	-0.67842	5.51E-18	TMEM131
3361.083	3366.508	3253.227	4565.264	4716.257	5072.562	-0.52423	5.51E-18	ARPC2
3246.95	3162.417	3426.611	4646.175	4695.268	4556.314	-0.49856	5.53E-18	C11orf58
344.1965	367.5616	362.2808	648.3276	680.0477	706.6676	-0.92233	5.57E-18	ZNF518B
1985.196	1970.566	1815.967	1186.699	900.4335	778.6039	1.009837	5.60E-18	CAPN15
578.7533	603.3559	626.9193	1026.951	1003.28	1039.902	-0.76316	5.61E-18	OXR1
1407.341	1613.903	1489.275	2758.245	2438.937	2279.744	-0.72963	5.66E-18	SNRPE
745.9088	785.6506	784.7897	1259.312	1231.012	1262.058	-0.69619	5.70E-18	TRIM37
548.198	597.4115	576.7292	951.2262	1083.039	996.5283	-0.81575	5.72E-18	CDC40
949.0117	932.279	957.261	1460.552	1518.563	1470.461	-0.64836	5.81E-18	PPM1A

771.9707	735.1233	749.2004	1171.139	1281.386	1283.215	-0.72711	5.85E-18	STX7
355.8794	429.9778	410.6458	742.7241	770.301	763.7935	-0.9292	5.89E-18	UBXN2B
114.133	122.8508	137.7945	324.6825	303.2929	361.7969	-1.40096	6.01E-18	DTWD2
1110.775	1123.49	1163.496	1754.115	1695.921	1769.843	-0.61939	6.08E-18	DYNC1I2
631.7758	668.7442	688.9724	1079.854	1111.374	1157.327	-0.75125	6.09E-18	RNGTT
112.3357	96.10102	87.60444	10.37324	10.49456	4.231543	3.558127	6.23E-18	MIR210HG
886.1037	873.8258	936.2724	1405.574	1417.816	1485.271	-0.67604	6.28E-18	SCAF4
875.3195	1036.306	908.896	545.6325	472.2554	449.6014	0.941819	6.36E-18	KHNYN
1177.278	1158.166	1242.888	1839.176	1799.818	1840.721	-0.61459	6.56E-18	GLE1
686.5956	617.2261	714.5237	1107.862	1174.342	1181.658	-0.77838	6.59E-18	TRMT11
1252.767	1341.451	1455.511	2048.715	2218.551	2154.913	-0.66525	6.62E-18	KDM5A
877.1168	887.696	946.3104	1375.492	1563.69	1633.375	-0.75391	6.77E-18	HMMR
461.0255	457.7183	403.3454	172.1958	205.6934	194.651	1.20738	6.82E-18	MAP2K5
568.8678	554.81	526.5392	1035.249	1000.132	899.2028	-0.83063	6.88E-18	XXYLT1
480.7966	435.9222	427.0716	796.6649	869.9993	801.8773	-0.87691	6.89E-18	C12orf43
768.3759	814.3819	821.2916	1289.394	1344.354	1267.347	-0.69865	7.26E-18	CPSF3
1522.373	1545.542	1736.575	2416.965	2460.975	2534.694	-0.62542	7.38E-18	PPP2R2A
4931.985	4924.93	4527.142	1954.319	1497.574	1275.81	1.605198	7.47E-18	MAP2K2
94.36195	101.0547	93.07971	4.149297	12.59348	5.289428	3.711525	7.55E-18	TRIM22
1317.473	1447.46	1259.314	791.4783	582.4483	516.2482	1.089697	7.55E-18	NDUFB2
2594.504	2493.673	2502.202	3546.611	3563.954	3816.852	-0.52557	7.76E-18	UBXN4
541.9072	604.3466	546.6152	283.1895	284.4027	247.5452	1.053635	7.78E-18	VAMP2
23877.17	23551.69	22937.76	16966.47	15855.19	17879.33	0.472882	7.89E-18	YBX1
591.3349	632.0871	723.6492	1258.274	1160.699	1101.259	-0.85437	8.17E-18	ALG8
243.5437	224.8962	271.9388	572.6029	661.1575	484.5116	-1.21386	8.51E-18	NXT2
104.2475	84.21224	84.8668	296.6747	243.4739	273.9924	-1.57322	8.52E-18	ZNF616
2766.153	2569.959	2823.418	3782.084	4161.094	4180.764	-0.57101	8.61E-18	FUBP3
1108.978	1208.693	1028.44	605.7973	501.6401	369.2021	1.179611	8.91E-18	ATP6V1F
337.007	303.164	302.9653	677.3727	622.3276	586.0687	-0.99919	9.01E-18	MED20
1238.388	1412.784	1306.766	809.1128	794.4385	711.9571	0.772874	9.02E-18	MEGF8
2279.066	2209.333	2431.936	3457.401	3498.887	3250.883	-0.56049	9.07E-18	CMTM6
1581.686	1634.708	1580.53	983.3833	808.0814	660.1207	0.968077	9.11E-18	TSPAN4
9084.809	9535.797	8888.2	2709.491	1905.813	1501.14	2.169093	9.17E-18	SLC16A3
119.5251	115.9157	111.3306	15.55986	19.93967	16.92617	2.725961	9.17E-18	RGL3
279.4911	278.3957	284.7144	538.3712	586.6461	553.2742	-0.99394	9.25E-18	GID4
293.8701	364.5894	300.2277	628.6184	676.8993	641.0787	-1.02346	9.36E-18	NRF1
271.403	316.0436	317.5661	119.2923	91.3027	108.9622	1.501203	9.46E-18	RAET1E
227.3674	239.7572	247.3	556.0057	626.5254	457.0066	-1.1987	9.56E-18	DTD2
303.7556	252.6367	373.2314	669.0741	707.3336	679.1626	-1.1432	9.70E-18	ATP7A
1863.873	2143.944	1995.739	1240.64	1206.875	1317.068	0.673006	9.75E-18	STAT6
589.5375	627.1335	692.6226	1081.929	1284.535	1106.548	-0.86311	9.86E-18	EDEM3
177.9397	142.6654	175.2089	34.2317	43.02771	34.91023	2.145227	9.96E-18	IGFL2
434.065	395.3021	384.182	804.9635	817.5265	701.3782	-0.93703	9.96E-18	TSPAN6
1405.544	1335.507	1411.709	2201.202	2011.808	2117.887	-0.6081	1.00E-17	HMGB1P5
430.4702	488.431	475.4366	845.4192	895.1863	803.9931	-0.86855	1.01E-17	GGCT
358.5754	395.3021	421.5964	727.1642	766.1031	723.5938	-0.91554	1.01E-17	FAM129A
139.2962	161.4893	130.4941	28.00775	32.53315	23.27348	2.362148	1.02E-17	AL161421.1
1492.716	1548.514	1536.728	2238.545	2488.261	2252.239	-0.60844	1.03E-17	KDELC2
2641.236	2628.412	2809.73	3757.188	4249.249	4530.924	-0.63379	1.05E-17	ATRX
607.5112	601.3744	595.8927	1011.391	1004.33	1002.876	-0.74204	1.05E-17	TPMT
118.6265	146.6284	156.9579	31.11972	17.84076	24.33137	2.525005	1.07E-17	RTN2
298.3635	306.1362	263.7259	600.6107	546.7668	648.4839	-1.04906	1.07E-17	ZNF532
92.56458	96.10102	98.55499	3.111972	12.59348	5.289428	3.775547	1.08E-17	TJP3
1767.714	1777.374	1852.469	2563.228	2633.086	2671.161	-0.5435	1.08E-17	UBE2V2

201.3055	206.0723	207.148	67.42607	33.5826	32.79446	2.197719	1.09E-17	ICAM5
2017.548	1956.696	1955.587	1352.671	1220.518	1138.285	0.675909	1.09E-17	FXYD5
1361.508	1352.349	1366.082	1949.132	2181.82	2243.776	-0.64369	1.14E-17	ARIH1
166.2568	227.8684	181.5967	46.67959	38.82988	53.95217	2.044495	1.17E-17	PTPN6
884.3063	963.9824	988.2876	1439.806	1615.113	1645.012	-0.72863	1.20E-17	STRN
498.7703	512.2085	511.9384	263.4803	236.1277	230.6191	1.060098	1.21E-17	TDRD7
1393.861	1198.786	1283.953	521.774	677.9488	745.8094	0.995159	1.27E-17	NABP1
247.1385	228.8591	210.7982	54.97818	81.85759	47.60486	1.896694	1.27E-17	CDH24
112.3357	106.9991	102.2052	14.52254	15.74185	12.69463	2.903709	1.29E-17	PPFIA3
508.6559	543.912	478.1742	262.443	222.4847	217.9244	1.122194	1.32E-17	FAM3A
584.1454	483.4773	443.4975	209.5395	232.9793	204.1719	1.22484	1.36E-17	MTCO1P12
317.2359	283.3494	302.9653	113.0683	104.9456	121.6569	1.4121	1.36E-17	AP1G2
370.2583	412.1446	365.931	764.5079	736.7184	678.1047	-0.92534	1.38E-17	MRPS6
125.8159	103.0361	146.9199	21.78381	18.89021	17.98406	2.680024	1.39E-17	CYP4F12
754.8956	879.7702	782.0521	1428.395	1368.491	1287.447	-0.75794	1.39E-17	C12orf49
2242.22	2345.063	2490.339	3498.894	3565.003	3335.514	-0.5552	1.40E-17	SPIN1
955.3025	895.6219	918.0215	1539.389	1410.469	1467.287	-0.67351	1.41E-17	PDCD10
514.9467	452.7646	575.8167	906.6213	1086.187	982.7758	-0.94594	1.44E-17	SPIN4
570.6652	563.7266	688.9724	1052.884	1094.583	1189.063	-0.87119	1.46E-17	MAPKAPK5
175.2436	191.2113	140.5321	38.38099	25.18695	42.31543	2.258885	1.49E-17	PRDM16
372.0557	379.4504	449.8853	713.679	849.0102	946.8077	-1.06222	1.49E-17	SHPRH
173.4463	218.9518	184.3343	58.09015	47.22554	38.08388	2.005993	1.55E-17	MCRIP1
503.2638	449.7924	406.9956	835.0459	842.7134	850.5401	-0.89422	1.59E-17	CHCHD4
512.2506	491.4032	611.406	939.8157	1028.467	1026.149	-0.88997	1.61E-17	FKTN
1202.441	1290.924	1253.839	1931.498	1889.021	1861.879	-0.60104	1.65E-17	BZW2
550.8941	558.7729	567.6037	910.7706	982.2911	976.4285	-0.77463	1.71E-17	SSH2
984.0604	1111.602	1085.93	1710.548	1727.405	1621.739	-0.6698	1.76E-17	GPAT4
1092.801	1186.897	1052.166	688.7832	604.4869	575.4898	0.833675	1.78E-17	YDJC
4389.179	4513.776	4395.735	6099.466	6282.046	5887.134	-0.45818	1.79E-17	SDCBP
6345.617	6481.37	6470.865	8473.901	8571.959	8575.221	-0.40892	1.80E-17	MORF4L2
471.8098	476.5422	437.1096	233.3979	196.2483	192.5352	1.154434	1.84E-17	ZNF76
1328.257	1295.878	1233.762	832.9713	738.8173	710.8992	0.756893	1.85E-17	SUMF2
274.9977	267.4977	238.1746	576.7522	492.195	596.6475	-1.09344	1.89E-17	SERPINB8
1260.855	1350.368	1268.439	2145.186	1961.434	1924.294	-0.63686	1.92E-17	CERS2
1013.717	1119.527	885.1698	539.4086	447.0684	342.755	1.182548	1.92E-17	VSIR
2084.051	1968.585	2056.879	2839.156	3030.83	3100.663	-0.55388	1.92E-17	MAPK1IP1L
514.9467	403.228	446.2351	828.822	869.9993	896.0292	-0.92607	1.93E-17	LUC7L
384.6373	456.7275	389.6572	181.5317	162.6657	120.599	1.403976	1.95E-17	BAD
2019.346	2151.87	2237.563	3071.517	3183.001	3112.3	-0.5476	1.99E-17	YME1L1
380.1439	369.5431	321.2163	151.4493	146.9239	124.8305	1.338933	2.07E-17	BMP6
702.7719	815.3726	786.6148	1287.319	1380.035	1237.726	-0.76138	2.12E-17	PPP1R8
1381.279	1348.386	1607.906	2192.903	2450.481	2554.794	-0.7304	2.16E-17	RB1
1616.735	1639.662	1544.941	1075.705	939.2634	966.9075	0.686978	2.17E-17	PPP1R26
462.8229	387.3763	428.8967	784.217	824.8727	768.025	-0.89299	2.27E-17	FAM206A
1108.079	1274.082	1177.185	656.6262	652.7618	740.52	0.795724	2.38E-17	SULF2
80.88168	57.46247	74.82879	239.6219	216.188	233.7927	-1.69036	2.42E-17	FIGN
1447.782	1502.941	1500.226	2192.903	2158.732	2192.997	-0.55634	2.42E-17	DDX27
266.0108	309.1084	300.2277	549.7818	629.6738	593.4739	-1.019	2.44E-17	SLC35A5
925.6458	968.9361	909.8086	536.2966	567.7559	509.9009	0.796777	2.48E-17	RUSC1
955.3025	903.5477	940.8352	559.1177	446.0189	519.4219	0.876955	2.50E-17	AKAP8L
955.3025	973.8897	999.2381	1452.254	1728.455	1636.549	-0.71802	2.51E-17	ZNF292
1996.879	2075.584	2055.054	1409.724	1327.562	1238.784	0.623743	2.57E-17	DGKZ
2026.535	2178.62	2031.328	3488.521	3321.529	2920.822	-0.64212	2.58E-17	NDST1
382.8399	385.3948	384.182	181.5317	155.3195	131.1778	1.299431	2.66E-17	DNAJB2

241.7463	241.7387	233.6118	450.1987	541.5195	550.1006	-1.10433	2.71E-17	SGTB
248.0371	204.0908	179.7716	58.09015	45.12662	63.47314	1.923025	2.72E-17	D2HGDH
967.884	919.3995	991.9377	1440.843	1571.036	1638.665	-0.69129	2.73E-17	SECISBP2L
339.703	424.0334	355.893	679.4473	790.2406	761.6777	-0.99626	2.77E-17	ZMYM1
2636.743	2619.496	2346.156	1651.42	1471.338	1236.668	0.802109	2.85E-17	PLCD3
168.9528	186.2576	187.072	449.1614	418.7331	375.5494	-1.19793	3.02E-17	RCL1
1363.306	1494.024	1483.8	2224.023	2216.452	2118.945	-0.59583	3.03E-17	AP3B1
712.6574	747.0121	730.9495	420.1163	358.9141	313.1342	1.003632	3.04E-17	PTOV1
103.3488	106.0083	113.1557	0	4.197825	1.057886	5.943133	3.06E-17	DTNA
313.6412	340.8119	329.4292	668.0367	603.4374	617.8052	-0.94192	3.21E-17	DCLRE1B
2135.276	2342.091	2296.879	3219.854	3278.502	3280.503	-0.52986	3.27E-17	H2AFV
256.1253	264.5255	248.2126	521.774	518.4314	503.5536	-1.00611	3.31E-17	HASPIN
878.9142	1020.454	907.9835	508.2888	356.8152	291.9764	1.278039	3.38E-17	ILVBL
1857.582	1755.577	1837.868	2717.789	2577.465	2639.425	-0.54141	3.59E-17	TMEM50A
204.0016	221.924	202.5853	70.53804	31.48369	39.14177	2.152905	3.64E-17	SELENOM
1307.587	1222.564	1204.561	1891.042	1899.516	1870.342	-0.59983	3.72E-17	WSB2
732.4285	794.5672	764.7137	1221.968	1268.793	1214.453	-0.69359	3.84E-17	CENPN
788.147	772.7711	792.0901	1200.184	1286.633	1324.473	-0.69555	3.86E-17	EMSY
304.6543	342.7933	300.2277	130.7028	115.4402	122.7147	1.360512	4.01E-17	ABCB9
2538.786	2528.349	2410.035	1717.809	1620.361	1435.551	0.647215	4.07E-17	CDK2AP1
935.5314	925.3439	978.2495	1430.47	1501.772	1508.545	-0.64514	4.12E-17	PHF14
114.133	101.0547	140.5321	359.9515	322.1831	295.1501	-1.45636	4.20E-17	SMAGP
1045.171	1101.694	995.5879	626.5438	505.838	432.6752	1.005155	4.20E-17	GMEB2
4049.476	4000.577	4296.268	5546.572	6403.783	6459.45	-0.57629	4.21E-17	MBNL1
501.4664	461.6812	503.7255	831.934	877.3455	1021.918	-0.89611	4.21E-17	ITGB1P1
616.4981	657.8462	519.2388	299.7867	283.3532	249.661	1.106048	4.34E-17	TMUB1
518.5414	533.0139	576.7292	916.9945	923.5216	972.1969	-0.78846	4.37E-17	RSRC1
1396.557	1417.738	1254.751	871.3523	754.5591	728.8832	0.788728	4.41E-17	TRIM8
745.9088	825.2799	737.3373	446.0494	351.5679	384.0125	0.965546	4.49E-17	TKFC
778.2615	717.2901	789.3525	1226.117	1290.831	1214.453	-0.70701	4.50E-17	DPYD
705.4679	709.3642	819.4665	1188.773	1308.672	1336.11	-0.77849	4.74E-17	IPMK
706.3666	618.2169	624.1816	1040.436	1233.111	1178.485	-0.82429	4.84E-17	KCNK1
495.1756	597.4115	504.6381	272.8162	223.5342	217.9244	1.159923	4.84E-17	THAP7
754.8956	887.696	813.0787	466.7959	352.6173	325.8288	1.099699	4.90E-17	RNF5P1
861.8392	965.9639	997.413	1458.478	1595.174	1742.338	-0.76364	4.92E-17	DCAF6
667.7232	656.8554	646.9953	364.1008	303.2929	250.7189	1.102174	4.92E-17	MGAT5B
890.5971	829.2428	878.782	486.505	500.5907	508.843	0.797103	4.94E-17	CYTH1
661.4324	714.3179	625.0942	359.9515	286.5016	235.9085	1.180392	5.04E-17	FAAP20
8703.767	8834.359	9009.569	11682.34	11760.21	11485.46	-0.39581	5.10E-17	ILF3
737.8206	774.7526	756.5008	449.1614	390.3978	391.4177	0.881897	5.19E-17	LINC00963
2607.985	2720.551	2548.742	1854.736	1776.73	1769.843	0.544163	5.21E-17	STAT1
391.8268	378.4597	420.6838	751.0227	711.5314	714.0728	-0.8695	5.25E-17	RRP8
11638.87	11917.52	11229.79	15910.48	15010.37	16270.28	-0.44004	5.28E-17	RPS20
399.9149	375.4875	364.1059	147.3	143.7755	78.28354	1.624695	5.30E-17	HAGHL
1419.923	1404.858	1313.154	836.0833	667.4542	543.7532	1.014875	5.30E-17	SHKBP1
1374.09	1389.997	1366.082	1977.14	2485.113	2326.291	-0.71688	5.31E-17	NFAT5
221.9753	210.0352	255.5129	489.617	547.8162	463.3539	-1.12537	5.32E-17	AC099850.3
252.5306	269.4792	277.4141	542.5205	512.1347	540.5796	-0.99701	5.37E-17	MICU2
2377.921	2359.924	2303.267	1610.964	1647.646	1546.629	0.551145	5.39E-17	KIAA1191
471.8098	434.9314	422.5089	798.7396	767.1526	815.6299	-0.84093	5.41E-17	SYS1
440.3558	479.5144	453.5355	196.0543	203.5945	232.7348	1.118772	5.44E-17	ZFYVE19
58.41454	89.1659	83.95425	229.2486	244.5233	272.9345	-1.69207	5.60E-17	-
485.2901	539.949	545.7026	257.2564	178.4076	142.8146	1.440461	5.83E-17	MFSD10
1204.238	1275.072	1335.968	2032.118	1987.67	1867.168	-0.62587	5.84E-17	EXOSC2

478.9993	478.5236	502.813	811.1875	853.208	922.4763	-0.82465	5.85E-17	CHIC1
426.8755	463.6627	472.6989	213.6888	222.4847	196.7667	1.106548	5.96E-17	KSR1
1417.227	1380.09	1326.842	2511.362	2222.749	2016.33	-0.71097	6.00E-17	FURIN
850.1563	802.4931	780.227	1431.507	1395.777	1232.437	-0.73866	6.27E-17	SERPINE1
87.17247	78.26784	92.16717	6.223945	3.148369	1.057886	4.62103	6.28E-17	WNT11
9321.164	9948.933	9727.743	12595.19	13384.77	13119.9	-0.43128	6.38E-17	LAMC1
192.3186	177.3411	195.2849	420.1163	400.8923	412.5754	-1.12584	6.73E-17	CHAC2
3752.91	4076.863	3810.793	2653.475	2321.397	1976.13	0.743664	7.44E-17	NACC1
1410.037	1392.969	1515.739	2222.986	2097.863	2159.145	-0.5852	7.45E-17	EIF2AK4
878.0155	885.7146	992.8503	1649.345	1584.679	1398.525	-0.74883	7.57E-17	POLR2D
454.7348	431.9592	429.8093	822.598	746.1635	781.7775	-0.83609	7.58E-17	EBAG9
378.3465	377.469	354.0679	174.2705	152.1712	145.9882	1.231789	7.77E-17	LPIN3
444.8492	452.7646	430.7218	1103.713	903.5819	756.3883	-1.05731	7.83E-17	HGH1
1166.493	1176.99	1153.458	733.3882	713.6303	732.0569	0.682303	7.94E-17	LETMD1
4092.613	4248.26	4244.252	2818.41	2702.35	3100.663	0.545719	8.09E-17	UQCRB
1508.893	1726.846	1622.507	1052.884	936.1151	845.2507	0.776989	8.26E-17	SUN2
870.826	875.8072	871.4816	526.9607	453.3651	505.6694	0.816994	8.37E-17	CUEDC1
1023.603	1039.278	1003.801	1581.919	1637.152	1514.892	-0.62654	8.38E-17	CCNF
477.2019	457.7183	421.5964	35.26902	10.49456	9.520971	4.615294	8.46E-17	RN7SL2
545.502	544.9027	559.3908	964.7114	881.5433	979.6021	-0.77632	8.66E-17	RNF38
193.2173	193.1928	164.2583	46.67959	29.38478	55.01006	2.070821	8.88E-17	GAS1
2066.976	1990.381	2109.807	2845.38	3023.484	3029.785	-0.52874	8.91E-17	AP1G1
2349.163	2429.275	2336.118	1642.084	1406.272	1283.215	0.715647	9.65E-17	HDAC7
701.8732	711.3457	711.7861	1095.414	1180.638	1176.369	-0.70014	9.70E-17	CHD1L
333.4122	313.0714	283.8019	112.031	108.094	133.2936	1.396621	9.85E-17	PLAT
520.3388	538.9583	481.8244	271.7789	202.5451	191.4773	1.210069	1.02E-16	SIRT6
934.6327	1025.408	1021.139	1650.383	1487.08	1606.928	-0.6707	1.03E-16	KBTBD2
5186.313	5411.379	5311.932	3738.516	3947.005	3911.003	0.456154	1.04E-16	SPAG9
962.4919	988.7507	922.5842	1636.897	1590.976	1416.509	-0.69284	1.05E-16	DDX56
833.0813	929.3068	876.0444	1426.321	1489.179	1343.515	-0.69142	1.06E-16	ETV5
394.5228	440.8758	394.22	193.9796	183.6549	156.5671	1.201855	1.07E-16	MATN2
151.8778	128.7952	176.1214	358.9142	370.4581	386.1283	-1.28604	1.08E-16	CEP162
925.6458	759.8916	861.4436	1370.305	1422.013	1470.461	-0.74207	1.14E-16	NUF2
98.85538	106.9991	104.0303	289.4134	248.7212	295.1501	-1.42773	1.15E-16	PFN1P1
13.48028	13.87025	12.77565	81.94861	102.8467	119.5411	-2.92357	1.17E-16	AC026462.1
2323.101	2407.479	2272.24	1614.076	1620.361	1525.471	0.556789	1.18E-16	OGDH
469.1137	485.4588	465.3986	249.9951	185.7538	191.4773	1.178198	1.18E-16	C16orf58
627.2823	613.2632	531.1019	307.0479	282.3038	295.1501	1.001904	1.19E-16	LARP6
877.1168	730.1696	714.5237	429.4522	373.6065	381.8967	0.970687	1.22E-16	WDR90
81.78036	109.9713	100.3801	6.223945	14.69239	8.463085	3.31382	1.25E-16	MEOX1
810.6141	820.3262	834.0672	1244.789	1320.216	1402.756	-0.68665	1.26E-16	UBE4B
178.8384	187.2484	170.6461	50.82888	52.47282	46.54697	1.840173	1.28E-16	DYSF
683.8995	671.7164	753.7632	1102.676	1206.875	1246.189	-0.7529	1.29E-16	GPR180
2263.788	2006.233	2415.51	3250.974	3567.102	3583.059	-0.6372	1.30E-16	CYP1B1
1339.94	1418.728	1299.466	825.71	596.0912	524.7113	1.059505	1.31E-16	SLC25A22
694.6837	719.2716	713.6111	1101.638	1239.408	1156.269	-0.71716	1.32E-16	TRAK2
328.9188	340.8119	377.7941	680.4846	686.3445	623.0947	-0.92565	1.32E-16	PRKCE
274.099	267.4977	272.8513	102.6951	92.35216	108.9622	1.421922	1.34E-16	NMRK1
351.3859	369.5431	337.6421	751.0227	634.9211	649.5418	-0.9439	1.35E-16	STX10
539.2112	505.2734	455.3606	255.1817	215.1386	183.0142	1.198483	1.36E-16	PEX16
358.5754	378.4597	360.4558	691.8952	696.839	635.7893	-0.88389	1.37E-16	HSPA14
1379.482	1595.079	1408.971	936.7037	739.8667	729.9411	0.864601	1.39E-16	NAA38
790.843	701.4384	705.3982	414.9297	384.101	331.1182	0.959517	1.40E-16	CHTF18
10541.58	11425.12	10357.4	7661.676	6657.751	5799.329	0.683982	1.41E-16	EPHA2

591.3349	613.2632	558.4783	294.6001	232.9793	311.0184	1.071838	1.43E-16	DOCK6
1445.985	1432.599	1419.009	2089.171	2200.71	2053.356	-0.56168	1.44E-16	SLC30A5
406.2057	378.4597	373.2314	804.9635	703.1358	678.1047	-0.91677	1.46E-16	ISY1
518.5414	579.5783	555.7406	914.9199	948.7085	949.9813	-0.76719	1.51E-16	HSF2
164.4594	166.443	189.8096	370.3247	393.5461	413.6333	-1.17665	1.52E-16	ANKRD18A
1158.405	1229.499	1231.937	2339.166	2001.313	1803.695	-0.76353	1.53E-16	NOL6
2089.443	2207.351	2005.777	1444.993	1374.788	1304.373	0.611511	1.54E-16	HTATIP2
319.0333	348.7377	378.7067	151.4493	124.8853	89.92028	1.513802	1.57E-16	PDE4A
2401.287	2574.913	2507.677	3710.508	3493.64	3468.807	-0.51234	1.62E-16	ELAC2
468.215	432.95	501.9004	789.4037	864.752	862.1768	-0.84191	1.62E-16	MLLT10
87.17247	83.2215	73.0037	4.149297	4.197825	3.173657	4.400021	1.67E-16	FER1L4
843.8655	893.6404	998.3256	1450.179	1499.673	1486.329	-0.69727	1.69E-16	ZDHHC20
1217.719	1292.905	1398.021	2092.283	2105.209	1916.889	-0.64561	1.69E-16	BRI3BP
428.6729	498.3383	410.6458	220.95	175.2592	167.1459	1.246451	1.70E-16	GALK1
258.8214	357.6543	349.5052	666.9994	664.3059	681.2784	-1.06032	1.71E-16	MRPL48
470.9111	522.1159	460.8358	226.1367	158.4679	112.1359	1.548301	1.73E-16	ZNF444
346.8925	347.747	333.9919	648.3276	692.6412	600.8791	-0.9169	1.75E-16	C5orf30
685.6969	618.2169	626.0067	1026.951	1092.484	1107.606	-0.74112	1.77E-16	SENP5
2270.079	2201.407	2243.039	3066.33	3353.013	3575.654	-0.57377	1.78E-16	TUG1
8110.635	8346.919	8125.312	5398.235	5628.234	6241.525	0.509562	1.78E-16	SERPINB6
189.6226	163.4708	136.8819	42.53029	29.38478	39.14177	2.141294	1.80E-16	RAB43
106.0449	101.0547	101.2926	9.335917	11.54402	19.04194	2.951715	1.83E-16	CYP24A1
548.198	604.3466	489.1248	286.3015	228.7815	210.5192	1.17697	1.84E-16	PARP3
627.2823	738.0955	772.0141	1234.416	1222.617	1197.527	-0.77432	1.86E-16	UHRF1BP1
323.5267	323.9694	341.2923	619.2825	646.4651	591.3581	-0.90919	1.86E-16	ST7
266.0108	307.127	312.0908	665.9621	589.7945	549.0427	-1.02849	1.87E-16	ING3
297.4648	299.2011	339.4672	613.0586	617.0803	576.5477	-0.94823	1.88E-16	SLC25A30
2540.583	2649.218	2411.86	1749.966	1493.376	1365.73	0.72157	1.92E-16	REPIN1
3070.808	3024.705	3141.897	2172.157	2176.572	2223.676	0.49117	1.94E-16	PPP1R15B
1087.409	1063.056	1119.694	1748.928	1715.861	1584.713	-0.62665	1.95E-16	CHEK1
498.7703	596.4208	563.041	958.4875	945.5602	981.7179	-0.80021	1.97E-16	RNASEH1
3253.241	3253.564	2932.924	2102.656	2223.798	2067.109	0.562018	2.08E-16	CDC25B
248.0371	257.5904	234.5244	69.50072	86.05542	95.20971	1.561788	2.13E-16	DNAJC22
905.8748	848.0667	991.0252	1433.582	1475.536	1507.487	-0.68555	2.15E-16	CEBPZOS
116.8291	122.8508	140.5321	17.63451	26.23641	26.44714	2.43597	2.18E-16	MPP2
633.5731	584.532	567.6037	295.6374	315.8864	316.3078	0.944885	2.21E-16	CLTCL1
460.1269	435.9222	443.4975	212.6514	220.3858	211.5771	1.055468	2.28E-16	CCDC57
422.3821	439.8851	430.7218	215.7634	163.7152	135.4094	1.327596	2.30E-16	GPRIN1
3396.132	3344.712	2996.802	1048.735	693.6906	616.7473	2.045206	2.34E-16	CCDC85B
401.7123	384.4041	419.7713	188.793	177.3581	133.2936	1.271468	2.35E-16	PLD2
1873.759	1949.761	2018.552	1264.498	1345.403	1275.81	0.588273	2.35E-16	MGAT5
264.2135	240.7479	254.6004	100.6204	86.05542	68.76257	1.571869	2.36E-16	NUDT14
215.6845	187.2484	179.7716	396.2578	435.5244	473.9328	-1.16317	2.42E-16	CLK4
165.3581	220.9333	201.6727	442.9374	487.9972	415.7491	-1.19743	2.43E-16	NCR3LG1
919.355	979.8341	961.8237	1475.075	1445.101	1484.214	-0.62272	2.47E-16	RFC4
466.4177	430.9685	461.7484	741.6868	863.7026	863.2347	-0.86036	2.48E-16	STIM2
2277.268	2358.933	2348.894	3161.764	3510.431	3376.771	-0.52475	2.53E-16	MTHFD1
410.6992	360.6265	437.1096	192.9423	150.0723	165.0302	1.250499	2.54E-16	PNKP
1191.657	1205.721	1230.112	738.5748	693.6906	793.4143	0.7048	2.56E-16	CRTC3
502.3651	463.6627	551.1779	826.7473	1034.764	1013.454	-0.92137	2.58E-16	RAB11FIP2
1440.593	1394.951	1380.682	2038.342	2039.094	2202.518	-0.57466	2.59E-16	ARL1
426.8755	415.1168	491.8624	770.7318	842.7134	815.6299	-0.86417	2.61E-16	KLF12
44.93426	68.36052	64.79078	207.4648	209.8913	192.5352	-1.77916	2.62E-16	FAM196B
1414.531	1464.302	1510.264	2304.934	2061.132	2276.57	-0.59789	2.62E-16	DNAJC8

375.6504	409.1724	347.6801	159.7479	161.6163	171.3775	1.200108	2.62E-16	CASP1
88.96984	81.24004	79.39152	5.186621	4.197825	0	4.727172	2.64E-16	GRIP2
3500.379	3604.284	3298.855	2495.802	2076.874	2182.418	0.622845	2.77E-16	PPP1R14B
113.2343	108.9805	101.2926	267.6296	282.3038	282.4555	-1.36342	2.83E-16	PTPN21
470.9111	524.0973	490.0373	814.2994	906.7303	878.0451	-0.80811	2.85E-16	ATP6V0A2
521.2375	473.57	502.813	909.7333	809.1308	964.7917	-0.84094	2.86E-16	CLDND1
752.1996	746.0213	744.6377	439.8254	426.0793	388.244	0.838481	2.87E-16	KLC1
544.6033	533.0139	568.5163	866.1657	1002.231	1044.133	-0.82281	2.96E-16	POLK
888.7997	860.9463	918.934	1314.29	1569.987	1611.16	-0.75201	3.01E-16	XRN1
207.5963	219.9425	245.4749	72.61269	78.70923	70.87834	1.598796	3.11E-16	AC026271.1
199.5081	220.9333	187.9845	53.94086	52.47282	71.93623	1.770329	3.21E-16	AL391422.3
1603.255	1492.043	1532.165	2247.881	2241.639	2251.181	-0.54238	3.24E-16	SF3B4
1125.154	1146.277	1016.576	690.8579	553.0635	598.7633	0.835037	3.30E-16	RNF220
313.6412	310.0992	259.1631	549.7818	599.2396	687.6257	-1.057	3.33E-16	USP45
899.584	913.4551	818.554	519.6994	501.6401	434.791	0.853379	3.39E-16	PHLDB1
338.8044	360.6265	355.893	161.8226	153.2206	138.583	1.217595	3.40E-16	MAP4K2
325.3241	341.8026	285.627	141.0761	100.7478	112.1359	1.42762	3.47E-16	ZNF414
6675.434	7144.17	7210.028	9180.319	10180.78	10183.21	-0.49049	3.51E-16	PDIA3
101.5514	125.823	145.0948	298.7494	331.6282	352.2759	-1.40003	3.56E-16	MEF2C
1625.722	1593.097	1557.716	2233.359	2335.04	2411.979	-0.54727	3.60E-16	KCTD3
335.2096	384.4041	360.4558	691.8952	694.7401	630.4999	-0.90215	3.66E-16	B3GLCT
495.1756	452.7646	511.0259	851.6431	930.8678	812.4562	-0.83009	3.66E-16	NOL9
2876.692	3039.566	3138.246	4838.08	4593.47	4101.423	-0.57987	3.66E-16	ADH5
1584.382	1585.171	1534.903	2262.404	2219.6	2342.159	-0.53667	3.70E-16	CHCHD3
726.1377	744.0399	845.9303	1269.685	1270.892	1252.537	-0.71147	3.92E-16	R3HDM1
144.6883	157.5264	145.0948	38.38099	33.5826	37.026	2.036501	4.02E-16	NDRG4
1445.985	1557.431	1524.865	2339.166	2395.909	2122.119	-0.59895	4.11E-16	C16orf72
576.0573	587.5042	636.9573	1099.564	1068.347	942.5761	-0.78871	4.12E-16	HPS5
2335.683	2249.953	2249.426	1632.748	1459.794	1433.435	0.594687	4.13E-16	PACS1
215.6845	243.7201	262.8133	487.5423	518.4314	483.4538	-1.04463	4.23E-16	TOGARAM1
676.71	685.5867	605.0181	354.7649	343.1722	355.4496	0.900909	4.24E-16	PLCXD1
1170.987	1097.731	1290.34	679.4473	711.5314	729.9411	0.747282	4.26E-16	TMEM65
1147.621	1104.666	1063.116	663.8874	706.2841	661.1785	0.706799	4.27E-16	GRAMD1A
206.6976	143.6562	167.9085	46.67959	40.9288	41.25754	2.008796	4.40E-16	ATG16L2
3163.372	3237.713	3088.056	4438.71	4211.468	4494.956	-0.47029	4.49E-16	PPM1G
993.0472	1055.13	910.7211	603.7226	558.3108	545.869	0.792304	4.52E-16	AMPD2
82.67905	86.1937	87.60444	5.186621	11.54402	4.231543	3.612834	4.62E-16	DES
167.1555	148.6098	157.8705	39.41832	24.1375	44.4312	2.133525	4.79E-16	CCDC88B
567.0704	550.8471	587.6798	986.4953	945.5602	917.1869	-0.74005	4.89E-16	SLC25A17
215.6845	253.6274	232.6993	89.20988	70.31358	75.10988	1.580002	4.91E-16	CADM4
1086.511	1096.741	1074.979	1599.554	1616.163	1708.485	-0.59583	4.92E-16	REXO2
918.4564	993.7044	962.7363	1435.657	1480.783	1569.902	-0.64233	4.92E-16	HTT
205.7989	168.4245	169.7336	383.8099	459.6619	404.1123	-1.19636	4.94E-16	ZNF480
920.2537	855.9926	958.1735	1354.745	1593.075	1565.671	-0.72248	5.00E-16	FANCD2
93.46327	101.0547	62.05314	5.186621	7.346194	5.289428	3.846906	5.03E-16	APOE
68.30008	83.2215	85.77934	3.111972	5.247282	4.231543	4.236981	5.08E-16	EPHB3
452.0387	449.7924	471.7864	811.1875	760.8559	808.2247	-0.79298	5.10E-16	MED28
1215.022	1351.359	1134.295	770.7318	627.5749	573.374	0.907772	5.33E-16	ST3GAL2
598.5244	594.4393	630.5694	982.346	1016.923	991.2389	-0.71344	5.41E-16	VCPIP1
1218.617	1151.231	1315.892	1849.549	1912.109	1871.4	-0.61151	5.48E-16	LYN
300.1609	323.9694	321.2163	666.9994	631.7727	549.0427	-0.96746	5.51E-16	LRR1
98.85538	94.11956	94.90481	15.55986	11.54402	10.57886	2.932423	5.54E-16	GBP5
847.4602	822.3077	870.5691	1433.582	1312.87	1291.678	-0.6685	5.60E-16	MMS19
1866.569	1917.067	1961.974	2621.318	2919.588	2994.874	-0.57103	5.60E-16	SPTLC1

1288.715	1226.526	1193.61	786.2917	708.383	790.2406	0.698916	5.61E-16	GTPBP2
1029.893	1067.019	962.7363	590.2374	616.0309	629.442	0.736818	5.61E-16	TRIB1
828.5878	845.0945	847.7554	1282.133	1321.266	1303.315	-0.63177	5.62E-16	TOPORS
1149.418	1254.267	1141.595	754.1346	671.6521	724.6517	0.720819	5.64E-16	SLPI
7825.751	8160.661	8243.03	4799.699	5219.996	6000.328	0.596918	5.88E-16	DSP
87.17247	106.9991	92.16717	14.52254	9.445107	11.63674	3.005862	5.95E-16	APOL3
847.4602	896.6126	1013.839	1432.545	1559.492	1514.892	-0.70846	6.26E-16	MAP3K2
469.1137	469.6071	396.0451	826.7473	783.9439	794.4721	-0.85004	6.40E-16	STAMBPL1
1208.732	1308.757	1233.762	1785.235	1983.472	1988.825	-0.61837	6.45E-16	PHAX
674.014	636.0501	663.4211	1010.354	1185.886	1293.794	-0.82207	6.61E-16	ZNF451
268.7069	249.6645	256.4255	101.6578	96.54998	93.09394	1.411445	6.67E-16	KCTD13
32.35267	25.75904	23.7262	202.2782	131.182	112.1359	-2.44376	6.68E-16	AL009174.1
151.8778	137.7118	125.0188	31.11972	26.23641	33.85234	2.184816	6.70E-16	SPTBN5
728.8338	844.1038	773.8392	457.4599	376.7548	327.9446	1.013102	6.76E-16	CYB561A3
8213.983	8117.069	8241.205	10567.22	10937.43	10574.63	-0.38458	6.79E-16	GNB1
311.8438	316.0436	328.5166	569.491	630.7233	594.5317	-0.90798	6.90E-16	THUMPD2
1173.683	1251.295	1168.972	1710.548	1928.901	1933.815	-0.6332	7.19E-16	KIAA1551
578.7533	541.9305	558.4783	233.3979	315.8864	248.6031	1.073824	7.32E-16	SYNM
9648.285	10167.88	9462.192	7379.524	6247.414	6245.757	0.558959	7.36E-16	SLC25A6
583.2467	627.1335	642.4325	985.4579	1019.022	1076.928	-0.73405	7.39E-16	KDM1B
1473.844	1469.256	1486.538	2263.441	2068.478	2170.781	-0.55385	7.49E-16	ARFIP2
472.7085	479.5144	498.2502	774.8811	899.3841	878.0451	-0.81513	7.51E-16	PIK3CA
2822.77	2831.513	2638.171	1997.886	1774.631	1743.396	0.588026	7.51E-16	LTBR
2963.864	3020.742	3131.859	4275.85	4302.771	4058.049	-0.47109	7.56E-16	PRNP
638.9652	649.9203	716.3488	1038.361	1176.441	1157.327	-0.74964	7.68E-16	G2E3
1061.347	991.7229	1023.877	1539.389	1535.355	1618.565	-0.60873	7.71E-16	GLRX5
119.5251	117.8971	118.631	271.7789	315.8864	296.208	-1.31153	7.91E-16	PDCD2L
390.0294	425.0241	369.5812	189.8303	177.3581	166.0881	1.150688	8.66E-16	MMAB
83.57773	72.32345	83.95425	6.223945	1.049456	2.115771	4.670017	8.79E-16	MAPK4
3964.999	4091.724	3811.706	2940.814	2646.729	2717.708	0.51487	9.55E-16	CDK16
736.9219	714.3179	685.3222	1104.75	1137.611	1212.337	-0.69316	9.73E-16	UBLCP1
1088.308	1192.842	1245.626	1754.115	1885.873	1806.869	-0.62725	9.89E-16	SIKE1
393.6242	412.1446	371.4063	681.522	746.1635	692.9151	-0.84967	9.99E-16	EARS2
1753.335	1882.391	1762.127	1209.52	879.4444	943.634	0.831514	1.02E-15	MRPS34
2505.535	2588.783	2321.518	1684.614	1717.96	1592.118	0.569981	1.02E-15	TNIP1
519.4401	521.1251	540.2274	832.9713	941.3623	927.7657	-0.77325	1.04E-15	CETN2
1259.058	1260.211	1276.652	1913.863	1804.015	1874.573	-0.55903	1.06E-15	SUCLG1
274.9977	321.988	353.1554	139.0014	110.1929	107.9043	1.411121	1.07E-15	TNFAIP8L1
99.75407	82.23077	79.39152	12.44789	6.296738	5.289428	3.440477	1.07E-15	LINC02323
4681.252	5070.567	4627.522	3540.387	3087.501	3025.553	0.574681	1.11E-15	ERGIC3
270.5043	311.0899	304.7904	125.5162	101.7973	118.4832	1.357373	1.15E-15	PIK3CD
554.4888	529.051	477.2617	262.443	193.1	144.9303	1.37746	1.15E-15	TEDC1
144.6883	141.6747	166.0834	379.6606	316.9358	371.3179	-1.23832	1.19E-15	TAF4B
1308.486	1294.887	1339.618	1850.586	2181.82	2294.554	-0.68205	1.19E-15	TAF1
617.3968	665.772	639.6949	1072.593	994.8846	1085.391	-0.71385	1.20E-15	ATP5PO
412.4965	418.089	378.7067	203.3155	167.913	144.9303	1.227548	1.21E-15	TICAM1
688.3929	682.6145	733.6872	1269.685	1146.006	1085.391	-0.73411	1.21E-15	-
1159.304	1237.424	1269.352	683.5966	791.2901	756.3883	0.716367	1.24E-15	FAM168A
216.5832	247.683	237.262	70.53804	88.15433	83.57297	1.533739	1.24E-15	SMARCD3
541.0085	544.9027	499.1628	293.5627	247.6717	241.1979	1.01802	1.27E-15	-
228.2661	228.8591	227.224	495.8409	483.7994	424.2122	-1.03676	1.32E-15	ZNF202
33.25136	46.56441	44.71476	150.412	155.3195	171.3775	-1.94051	1.34E-15	AC012513.3
232.7595	237.7757	266.4635	98.54579	82.90705	66.6468	1.570176	1.36E-15	-
449.3426	536.9768	521.0639	221.9874	218.2869	266.5872	1.092261	1.38E-15	ZSCAN31

958.8972	919.3995	889.7326	560.155	548.8657	557.5058	0.732082	1.40E-15	DLGAP4
629.0797	558.7729	577.6418	936.7037	1027.418	1007.107	-0.75032	1.40E-15	SHOC2
304.6543	312.0806	318.4786	595.4241	547.8162	615.6895	-0.91137	1.41E-15	CEP63
1338.142	1365.229	1447.298	1948.095	2264.727	2185.592	-0.62426	1.42E-15	PCGF5
957.0998	1022.436	1011.101	1537.314	1514.366	1487.387	-0.60226	1.43E-15	GGH
1097.295	1147.268	1051.253	2594.348	1978.225	1714.833	-0.93209	1.43E-15	NELFB
595.8283	596.4208	549.3528	971.9727	950.8074	949.9813	-0.72228	1.45E-15	MIR4435-2HG
87.17247	70.34198	90.34208	6.223945	9.445107	6.347314	3.494078	1.47E-15	SPON2
829.4865	869.8629	921.6717	1316.364	1394.727	1411.219	-0.65331	1.47E-15	MKLN1
2510.927	2770.087	2608.97	4322.53	4140.105	3579.885	-0.6103	1.47E-15	PLXNA1
643.4587	610.291	634.2196	1129.646	1086.187	962.676	-0.75133	1.49E-15	BLOC1S2
358.5754	397.2836	357.7181	151.4493	154.2701	181.9563	1.190963	1.50E-15	RABL2B
2019.346	2027.038	2126.233	2791.439	3060.215	3117.589	-0.53898	1.50E-15	PARP4
232.7595	243.7201	260.0757	497.9156	487.9972	472.8749	-0.98598	1.50E-15	ATRIP
2860.515	3021.733	3028.741	4120.251	4040.407	4060.165	-0.45582	1.54E-15	RACGAP1
2157.743	2349.026	2157.259	1563.247	1323.364	1282.157	0.676422	1.54E-15	BCL2L1
328.0201	400.2558	339.4672	167.0092	136.4293	123.7726	1.320217	1.56E-15	OXLD1
305.553	339.8211	312.0908	577.7895	602.3879	600.8791	-0.89627	1.57E-15	EIF4ENIF1
86.27379	74.30491	75.74134	1.037324	2.098913	5.289428	4.816083	1.57E-15	KLHDC7B
249.8345	234.8035	250.0377	487.5423	461.7608	506.7272	-0.98626	1.61E-15	GAN
1082.916	1116.555	1172.622	732.3508	678.9983	659.0628	0.703596	1.62E-15	TCEAL9
1194.353	1262.193	1405.321	1920.087	2078.973	1999.404	-0.63523	1.63E-15	USP12
1333.649	1431.608	1376.12	2029.006	2021.253	1994.114	-0.54583	1.64E-15	TMX2
522.1361	547.8749	575.8167	882.7628	926.67	956.3287	-0.74887	1.65E-15	NT5C2
334.3109	277.405	322.1288	552.8938	643.3167	717.2465	-1.03361	1.67E-15	RBM41
198.6094	231.8313	208.0605	434.6388	441.8211	441.1383	-1.04645	1.70E-15	ZNF398
205.7989	209.0445	198.0225	431.5268	469.107	394.5914	-1.0799	1.72E-15	STARD13
586.8415	527.0695	553.003	207.4648	302.2434	259.182	1.116943	1.72E-15	SLC26A6
1202.441	1259.221	1234.675	1904.527	1747.345	1869.284	-0.5791	1.72E-15	SNW1
613.802	665.772	630.5694	1008.279	1045.259	1042.017	-0.69702	1.75E-15	RHOT1
408.0031	368.5524	386.9196	191.905	167.913	171.3775	1.131354	1.82E-15	PICK1
2042.712	2168.713	2148.134	2954.299	3115.836	2937.749	-0.50244	1.84E-15	SMAD3
5921.437	6420.935	5976.265	4397.217	3329.925	3282.619	0.734402	1.87E-15	MYL6
1064.942	1039.278	1043.04	1634.823	1519.613	1591.06	-0.59239	1.89E-15	COA4
84.47642	64.39759	83.04171	4.149297	6.296738	2.115771	4.206116	1.90E-15	FAM46C
1356.116	1290.924	1343.268	1865.109	2060.083	2043.835	-0.5807	1.90E-15	ADAM17
212.0897	276.4143	265.5509	608.9093	553.0635	478.1643	-1.12248	1.91E-15	MCAT
1249.173	1260.211	1289.428	1776.936	1970.879	1954.973	-0.58603	1.98E-15	ATAD1
29369.93	31381.44	30248.17	41911.01	38949.52	39477.12	-0.40319	2.01E-15	ANXA2
294.7688	286.3216	255.5129	103.7324	119.638	97.32548	1.383149	2.03E-15	PDE3A
1004.73	965.9639	1145.246	1647.271	1968.78	1640.781	-0.75417	2.06E-15	MTX3
594.9297	608.3096	626.9193	961.5995	1063.099	987.0073	-0.7186	2.10E-15	ABRACL
500.5677	496.3568	500.9879	910.7706	868.9499	792.3564	-0.78003	2.16E-15	TMEM126A
7237.113	7421.575	7615.198	5193.882	5730.032	5373.001	0.450762	2.19E-15	2-Sep
221.9753	250.6552	209.8856	466.7959	455.4641	467.5855	-1.02732	2.20E-15	TTC26
1428.91	1483.126	1549.503	2151.41	2151.386	2395.053	-0.58612	2.25E-15	HNRNPH2
8931.134	9216.781	9273.295	6464.604	6779.488	7096.297	0.430952	2.26E-15	MAP4
270.5043	275.4235	267.376	499.9902	552.014	513.0746	-0.94454	2.27E-15	FAT4
1623.026	1575.264	1679.085	2246.844	2466.222	2432.079	-0.55061	2.29E-15	CASC4
1501.703	1581.209	1471.937	1044.585	890.9884	874.8715	0.69623	2.30E-15	RETREG2
430.4702	405.2095	406.9956	766.5825	676.8993	812.4562	-0.85995	2.44E-15	FAIM
276.7951	181.304	229.0491	50.82888	79.75868	38.08388	2.027167	2.50E-15	-
860.9405	837.1687	905.2458	547.7071	471.2059	477.1064	0.799265	2.53E-15	REXO1
463.7216	478.5236	501.9004	790.441	847.9607	819.8614	-0.76739	2.71E-15	MAP3K21

6654.764	6764.719	6840.446	5160.688	4834.845	4487.551	0.484199	2.73E-15	LAPTM4A
627.2823	640.013	551.1779	1046.66	1074.643	963.7339	-0.76305	2.73E-15	RTCA
99.75407	101.0547	103.1177	18.67183	12.59348	16.92617	2.656451	2.73E-15	LINC00668
235.4555	218.9518	194.3723	76.76199	68.21466	43.37331	1.783386	2.77E-15	RTN4R
2142.466	2060.723	2113.457	2912.806	2919.588	2939.864	-0.47362	2.83E-15	DSTN
435.8624	449.7924	478.1742	733.3882	843.7629	834.6718	-0.82232	2.84E-15	COG3
101.5514	86.1937	106.7679	15.55986	14.69239	13.75251	2.743118	2.94E-15	AP001972.5
230.9621	226.8777	212.6233	474.0571	426.0793	458.0645	-1.01863	2.94E-15	AC125257.1
483.4927	574.6247	525.6266	283.1895	258.1663	245.4295	1.008394	3.04E-15	SHB
759.3891	858.9648	813.0787	467.8332	298.0456	282.4555	1.213065	3.05E-15	C19orf70
1827.926	1901.215	1905.397	2759.282	2604.751	2625.672	-0.50399	3.06E-15	NRBP1
1378.583	1392.969	1392.546	955.3755	866.8509	862.1768	0.633249	3.12E-15	P3H1
82.67905	73.31418	68.44097	2.074648	3.148369	5.289428	4.419795	3.16E-15	JAKMIP3
297.4648	285.3309	309.3532	600.6107	539.4206	549.0427	-0.92049	3.20E-15	POC5
99.75407	89.1659	62.96569	1.037324	3.148369	2.115771	5.32329	3.21E-15	DOC2A
307.3504	314.0621	316.6535	108.919	121.7369	148.104	1.308882	3.23E-15	MAPRE3
1077.524	1126.462	1137.033	1622.375	1673.883	1669.344	-0.57181	3.25E-15	INTS8
481.6953	509.2363	490.0373	983.3833	830.12	815.6299	-0.82852	3.25E-15	GINS2
538.3125	710.355	639.6949	331.9437	243.4739	203.114	1.277309	3.28E-15	LAMTOR4
956.2011	989.7414	1062.204	1465.739	1760.988	1635.491	-0.69264	3.30E-15	ZBTB44
97.05801	104.0269	77.56643	9.335917	7.346194	15.86829	3.098651	3.34E-15	CDH1
103.3488	123.8415	187.072	22.82113	27.28586	24.33137	2.476897	3.38E-15	-
1297.702	1260.211	1274.827	1932.535	1987.67	1789.943	-0.57508	3.38E-15	EXOC3
101.5514	100.0639	88.51698	16.59719	12.59348	13.75251	2.755398	3.41E-15	PNPLA7
238.1516	223.9055	239.9997	464.7212	524.7282	441.1383	-1.02642	3.43E-15	ABHD3
828.5878	772.7711	660.6835	1238.565	1258.298	1454.593	-0.80472	3.54E-15	EIF3CL
3397.03	3455.674	3026.916	2346.427	1969.83	1915.831	0.664543	3.54E-15	BCAP31
593.1323	560.7544	552.0905	320.5332	221.4353	209.4614	1.18246	3.64E-15	LRRC61
1404.645	1480.154	1384.333	953.3009	934.0161	868.5241	0.631098	3.66E-15	KLC2
4098.005	4412.721	3983.264	3059.069	2855.571	2688.088	0.538133	3.67E-15	CD276
613.802	604.3466	571.2539	965.7488	947.6591	1012.397	-0.7094	3.68E-15	CTAGE5
783.6536	864.9092	740.075	417.0043	418.7331	278.2239	1.09986	3.71E-15	WNT7B
1582.585	1712.976	1550.416	2282.113	2550.179	2386.59	-0.57534	3.79E-15	ZNF106
2087.646	2138	2093.381	706.4177	498.4918	387.1862	1.988647	3.85E-15	COL18A1
878.9142	777.7248	858.706	432.5642	494.2939	498.2642	0.820346	3.86E-15	ID2
727.0364	808.4375	699.0104	439.8254	333.7271	302.5553	1.053351	3.87E-15	STK40
1241.983	1287.952	1262.964	1766.563	1953.038	1968.725	-0.58479	3.87E-15	DHX40
190.5213	163.4708	173.3838	48.75423	25.18695	9.520971	2.657343	3.98E-15	PDXP
285.7819	425.0241	304.7904	133.8148	81.85759	59.2416	1.883447	4.05E-15	LY6E
487.9861	549.8564	546.6152	909.7333	876.296	882.2767	-0.75252	4.07E-15	ARNTL2
1036.184	975.8712	995.5879	1454.328	1542.701	1607.986	-0.61423	4.13E-15	ZCCHC11
417.8887	376.4782	433.4595	754.1346	801.7846	682.3363	-0.8655	4.13E-15	GLRX2
3604.627	3651.839	3776.116	4870.237	5132.891	4926.574	-0.43639	4.20E-15	SFXN1
377.4478	360.6265	302.9653	127.5909	128.0337	163.9723	1.311191	4.25E-15	TMEM44
517.6427	560.7544	541.1399	291.4881	285.4521	280.3397	0.917321	4.25E-15	CREM
5579.937	5582.776	5592.083	7188.656	7396.568	7774.402	-0.4163	4.40E-15	LIMCH1
562.577	545.8934	579.4668	942.9276	950.8074	886.5082	-0.71969	4.41E-15	CENPO
353.1833	276.4143	274.6764	110.9937	109.1435	125.8884	1.386804	4.42E-15	ADAMTSL5
714.4548	622.1798	721.8241	1085.041	1152.303	1147.806	-0.71673	4.44E-15	RIOK3
201.3055	251.646	251.8628	509.3262	475.4037	471.817	-1.04842	4.48E-15	KRBOX4
173.4463	152.5728	156.9579	362.0261	355.7657	345.9286	-1.13822	4.51E-15	TUBE1
183.3318	225.8869	202.5853	412.855	453.3651	424.2122	-1.07824	4.54E-15	EXOG
93.46327	59.44393	83.95425	4.149297	7.346194	5.289428	3.821288	4.57E-15	YPEL2
324.4254	254.6182	279.2391	88.17255	102.8467	122.7147	1.453279	4.64E-15	CDKN2B

843.8655	824.2892	803.0407	486.505	435.5244	334.2919	0.975665	4.67E-15	MED25
629.0797	704.4106	731.8621	1134.833	1140.759	1093.854	-0.7065	4.79E-15	RNMT
785.4509	683.6052	751.9381	1121.347	1291.881	1378.425	-0.77089	4.86E-15	TROVE2
354.9807	391.3392	321.2163	168.0465	147.9733	142.8146	1.217269	4.94E-15	ZBED6CL
542.8059	487.4402	543.8775	262.443	278.1059	275.0503	0.94927	4.94E-15	C19orf54
942.7209	1069.991	1014.751	1696.025	1699.07	1465.172	-0.68348	5.04E-15	USP36
184.2305	144.6469	143.2698	40.45564	44.07717	37.026	1.958184	5.05E-15	CDC42BPG
330.7162	335.8582	286.5395	135.8895	105.9951	67.70468	1.621169	5.12E-15	COMTD1
965.188	1023.426	1015.664	1489.597	1636.102	1486.329	-0.61861	5.13E-15	RUNX1
1088.308	996.6766	1197.261	1642.084	1958.286	2249.065	-0.83315	5.17E-15	RICTOR
622.7889	656.8554	597.7178	350.6156	342.1228	343.8128	0.8565	5.27E-15	TMEM63B
4856.495	4906.106	4612.921	3543.499	3301.59	2955.733	0.552519	5.52E-15	SERPINH1
86.27379	83.2215	102.2052	13.48521	12.59348	9.520971	2.931603	5.67E-15	HSF4
509.5546	456.7275	506.4632	810.1502	907.7797	825.1508	-0.78729	5.84E-15	STAM
103.3488	93.12882	104.9428	256.2191	244.5233	271.8766	-1.35674	5.84E-15	PUS3
9472.143	9324.771	8887.288	13847.24	12069.8	12562.39	-0.47505	5.90E-15	NACA
1446.883	1155.194	1450.036	2094.357	2264.727	2219.444	-0.69832	6.08E-15	CREBZF
655.1416	607.3188	629.6569	368.2501	343.1722	326.8867	0.865908	6.09E-15	NMNAT2
1127.85	1034.324	1106.919	1573.621	1703.268	1848.126	-0.64819	6.23E-15	NR2C2
197.7108	213.0074	230.8742	77.79931	73.46194	50.77851	1.666223	6.29E-15	TSC22D3
35.94741	26.74977	26.46384	125.5162	120.6875	149.1619	-2.14596	6.38E-15	-
127.6133	137.7118	124.1063	28.00775	30.43423	31.73657	2.110352	6.39E-15	PPP1R3G
2680.778	2397.572	2658.247	3570.47	3874.593	4299.247	-0.60188	6.39E-15	LUC7L3
3193.927	3320.934	3235.889	2428.376	2266.826	2166.55	0.50676	6.46E-15	SERP1
472.7085	550.8471	478.1742	905.584	818.5759	883.3345	-0.79711	6.50E-15	MRPL58
639.8639	612.2725	759.2385	1126.534	1102.979	1250.421	-0.79025	6.76E-15	GAS2L3
436.761	464.6534	420.6838	232.3606	206.7429	176.6669	1.101589	6.76E-15	CBX8
1356.116	1374.146	1383.42	1906.602	2032.797	2036.43	-0.53869	6.82E-15	TACC1
416.0913	395.3021	451.7104	851.6431	766.1031	696.0888	-0.87291	6.89E-15	NEIL3
886.1037	938.2234	972.7743	1475.075	1463.992	1371.02	-0.62397	6.99E-15	SMURF2
3317.047	3517.099	3433.911	4719.825	4720.455	4506.593	-0.44195	7.06E-15	RPA1
935.5314	864.9092	933.5348	540.4459	568.8053	543.7532	0.726317	7.10E-15	AACS
584.1454	532.0232	544.7901	308.0853	251.8695	204.1719	1.11988	7.10E-15	AP5Z1
727.0364	816.3633	772.0141	1233.378	1178.539	1247.247	-0.66085	7.19E-15	CSTF1
267.8082	270.4699	253.6878	91.28452	115.4402	99.44125	1.371157	7.22E-15	CCDC9
676.71	640.013	703.5731	1035.249	1284.535	1129.822	-0.77142	7.23E-15	KMT2E
5143.176	5089.391	5100.221	6759.204	6698.68	6596.975	-0.38732	7.25E-15	PRDX6
990.3512	1139.342	1046.691	1570.509	1671.784	1648.186	-0.62321	7.42E-15	PANX1
8005.488	8056.634	8382.65	10357.68	11092.75	10877.18	-0.40321	7.42E-15	ATP2A2
193.2173	213.9982	173.3838	60.1648	65.06629	38.08388	1.828544	7.49E-15	SLC35E4
1330.054	1356.312	1406.234	1892.079	2358.128	2337.927	-0.68676	7.59E-15	AKAP11
796.2352	854.0111	747.3754	1339.185	1195.331	1347.746	-0.69584	7.65E-15	IGBP1
782.7549	880.7609	782.0521	1346.447	1287.683	1247.247	-0.66719	7.65E-15	MRPL40
541.9072	553.8193	572.1665	285.2641	294.8972	314.192	0.899254	7.75E-15	MRPL33
1386.671	1476.191	1567.754	2243.732	2218.551	2103.077	-0.56747	7.75E-15	CCDC6
125.8159	237.7757	95.81735	26.97043	13.64293	23.27348	2.843807	8.10E-15	-
4902.328	4847.653	4958.776	3606.776	3740.262	3685.674	0.414946	8.40E-15	ESYT2
141.0936	121.8601	98.55499	20.74648	24.1375	25.38926	2.363612	8.50E-15	SPACA6
89.86853	101.0547	78.47897	12.44789	13.64293	8.463085	2.961113	8.57E-15	GJC2
2309.621	2376.766	2366.232	1672.167	1384.233	1265.231	0.706383	8.62E-15	UNC13D
114.133	91.14736	92.16717	12.44789	17.84076	15.86829	2.689425	8.63E-15	RHPN1
160.8647	187.2484	127.7565	43.56761	40.9288	29.6208	2.058253	8.67E-15	C21orf2
569.7665	487.4402	539.3148	270.7416	284.4027	270.8187	0.951484	8.71E-15	AIG1
664.1284	636.0501	649.7329	370.3247	356.8152	376.6073	0.821214	8.90E-15	WDR59

2373.428	2463.951	2422.81	1640.009	1513.316	1203.874	0.736404	9.19E-15	PPP4C
1178.176	1338.479	1239.238	762.4332	823.8232	759.5619	0.678616	9.21E-15	RGP1
3568.679	3683.542	3563.493	4754.057	4894.664	4797.512	-0.41767	9.24E-15	SCRN1
410.6992	379.4504	347.6801	853.7178	704.1852	655.8891	-0.96029	9.31E-15	DPH2
345.9938	366.5709	331.2543	160.7852	143.7755	98.38337	1.372267	9.34E-15	ZNF581
1298.6	1403.867	1332.317	2014.483	1925.752	1945.452	-0.54513	9.58E-15	GTF2A2
322.628	317.0343	277.4141	131.7402	132.2315	100.4991	1.330652	9.83E-15	TBC1D17
2202.678	2295.526	2309.654	1634.823	1310.771	1379.483	0.654295	1.01E-14	COX5B
737.8206	666.7628	772.9266	1140.019	1154.402	1268.405	-0.70958	1.01E-14	PHKB
753.9969	758.9008	848.668	1339.185	1189.034	1276.868	-0.688	1.02E-14	ANAPC13
124.0186	129.7859	110.4181	30.0824	13.64293	24.33137	2.418826	1.07E-14	LINC01679
327.1214	329.9138	344.9425	159.7479	133.281	149.1619	1.180016	1.08E-14	PGBD5
2743.686	2937.521	2934.749	3820.465	4182.084	4051.702	-0.48457	1.09E-14	NSD1
311.8438	350.7192	362.2808	150.412	158.4679	143.8725	1.178234	1.10E-14	USP18
248.0371	256.5996	281.0642	78.83663	116.4897	70.87834	1.561451	1.11E-14	RNF182
774.6667	804.4745	812.1661	490.6543	457.563	390.3598	0.836744	1.13E-14	SIPA1L3
1023.603	1024.417	1055.816	1459.515	1672.833	1689.443	-0.63536	1.14E-14	NIN
308.2491	328.9231	315.741	581.9388	555.1624	604.0527	-0.87009	1.18E-14	XPA
352.2846	358.645	313.9159	123.4416	167.913	136.4673	1.260238	1.18E-14	DNHD1
422.3821	348.7377	419.7713	692.9325	749.3118	720.4201	-0.8594	1.20E-14	PPIL3
2281.762	2027.038	1975.663	1326.738	1281.386	1452.477	0.630343	1.21E-14	USP11
3955.114	4336.435	4457.788	5781.007	6282.046	5861.745	-0.49163	1.23E-14	ADAM9
1087.409	1093.768	1000.151	685.6713	638.0695	648.4839	0.689554	1.23E-14	ARHGEF17
608.4099	611.2818	597.7178	355.8022	302.2434	257.0662	0.989405	1.23E-14	ZDHHC8
169.8515	149.6006	119.5436	32.15705	40.9288	21.15771	2.219143	1.24E-14	NXPH4
1902.517	2014.159	1975.663	2833.97	2720.191	2702.898	-0.48702	1.30E-14	SPAG5
681.2034	695.494	680.7595	1058.071	1061	1110.78	-0.65072	1.37E-14	DAAM1
829.4865	810.4189	759.2385	486.505	409.288	338.5234	0.958412	1.37E-14	PQLC1
1091.903	1158.166	1122.432	1620.3	1723.207	1650.302	-0.5666	1.38E-14	ABHD2
906.7734	836.178	880.6071	1328.812	1357.997	1317.068	-0.60942	1.39E-14	HERC3
1002.933	891.659	967.299	1384.828	1559.492	1587.886	-0.66262	1.40E-14	BRWD1
561.6783	527.0695	530.1894	285.2641	288.6005	297.2659	0.894391	1.43E-14	MAN2C1
1256.362	1292.905	1262.051	1753.078	2068.478	2037.488	-0.62041	1.44E-14	PCNX1
201.3055	241.7387	156.9579	63.27677	58.76956	41.25754	1.875609	1.45E-14	-
747.7062	727.1974	695.3602	1116.161	1141.809	1335.052	-0.72721	1.48E-14	RBAK
1896.226	1881.4	2062.354	2653.475	2923.785	3001.222	-0.55455	1.52E-14	PUM1
12427.02	13215.38	12661.58	16305.7	16725.19	17726.99	-0.40618	1.54E-14	PFN1
308.2491	262.544	271.9388	125.5162	105.9951	88.8624	1.395264	1.55E-14	PRR5L
924.7472	691.5311	878.782	1323.626	1571.036	1598.465	-0.84763	1.56E-14	SOCS4
521.2375	446.8202	464.486	935.6664	809.1308	799.7616	-0.82824	1.60E-14	TMEM69
1513.386	1523.746	1649.884	2176.306	2363.376	2299.843	-0.54507	1.60E-14	HSDL2
1652.682	1536.626	1497.488	1041.473	925.6205	775.4302	0.772983	1.61E-14	TBC1D10B
6705.091	7006.458	6724.553	9033.019	8865.807	8748.715	-0.38299	1.65E-14	CTSC
176.1423	167.4337	168.8211	331.9437	382.0021	437.9647	-1.16816	1.69E-14	AL354714.2
148.2831	169.4152	153.3078	47.71691	46.17608	33.85234	1.881168	1.69E-14	UCN2
403.5097	403.228	385.0945	655.5889	715.7292	838.9033	-0.89094	1.70E-14	ANKRD12
4638.115	4599.97	4590.107	3259.272	3340.42	3560.843	0.444698	1.76E-14	HK1
1277.032	1352.349	1210.036	2354.726	1963.533	1889.384	-0.69353	1.79E-14	POLR2C
266.0108	306.1362	272.8513	115.143	105.9951	122.7147	1.296528	1.87E-14	C1orf21
1091.903	1114.574	1192.698	728.2015	681.0972	565.9688	0.782954	1.88E-14	UNC93B1
760.2877	775.7433	792.0901	1192.923	1190.083	1185.89	-0.61635	1.88E-14	FUT8
399.9149	377.469	427.0716	186.7183	197.2978	196.7667	1.052858	1.89E-14	DGKA
229.1647	194.1835	221.7487	86.0979	51.42336	63.47314	1.682315	1.90E-14	ZNF513
653.3442	749.9843	835.8923	1281.095	1252.001	1217.626	-0.7444	1.97E-14	ABHD10

38192.33	41703.88	39921.16	31154.99	29563.19	27678.52	0.438744	1.98E-14	PGK1
1467.553	1423.682	1558.629	951.2262	999.0824	989.1231	0.5985	2.01E-14	MSMO1
1068.537	1034.324	1064.029	1594.367	1589.926	1508.545	-0.56724	2.01E-14	ERLIN2
165.3581	129.7859	142.3572	13.48521	38.82988	32.79446	2.364088	2.06E-14	PSG5
317.2359	316.0436	350.4177	587.1255	593.9923	597.7054	-0.85426	2.09E-14	AC005288.1
578.7533	515.1807	536.5772	891.0614	871.0488	978.5442	-0.74852	2.11E-14	ZNF195
538.3125	622.1798	610.4934	959.5248	969.6977	983.8337	-0.71863	2.12E-14	THADA
347.7912	333.8767	354.0679	587.1255	683.1961	615.6895	-0.86425	2.15E-14	CRNKL1
1004.73	1008.565	1032.09	622.3945	663.2564	634.7314	0.665299	2.20E-14	CASP7
595.8283	553.8193	536.5772	879.6509	1002.231	935.1709	-0.74009	2.23E-14	GJA1
124.0186	167.4337	156.9579	329.8691	345.2711	362.8548	-1.21273	2.25E-14	AC005034.3
81.78036	101.0547	100.3801	16.59719	12.59348	3.173657	3.125566	2.34E-14	PSMB10
1789.282	1781.336	1925.473	2512.399	2707.597	2927.17	-0.5677	2.38E-14	PSIP1
629.9784	709.3642	675.2842	395.2205	377.8043	355.4496	0.835519	2.39E-14	PPIC
416.0913	388.367	441.6724	725.0896	736.7184	698.2045	-0.7929	2.40E-14	CENPL
334.3109	416.1075	387.8321	669.0741	705.2347	692.9151	-0.86205	2.42E-14	BNC1
780.9575	730.1696	749.2004	1109.937	1219.468	1278.984	-0.6744	2.45E-14	TLK1
668.6218	679.6423	601.368	1107.862	1062.05	1003.934	-0.70347	2.45E-14	SLC23A2
258.8214	274.4328	236.3495	106.8444	74.5114	100.4991	1.44853	2.46E-14	TECPR1
1072.132	1081.88	1188.135	1631.711	1656.042	1737.048	-0.58808	2.50E-14	MON2
484.3914	524.0973	471.7864	783.1797	847.9607	879.103	-0.76252	2.51E-14	RSU1
182.4331	179.3225	202.5853	69.50072	57.7201	52.89428	1.647361	2.54E-14	NATD1
249.8345	234.8035	252.7753	452.2733	476.4532	494.0326	-0.94745	2.55E-14	MAP9
79.98299	101.0547	79.39152	7.261269	13.64293	11.63674	3.000778	2.56E-14	-
68.30008	76.28638	67.52842	5.186621	2.098913	6.347314	3.959717	2.60E-14	FXYD3
213.8871	178.3318	197.11	415.967	384.101	429.5016	-1.05963	2.62E-14	CASD1
349.5886	327.9324	313.9159	162.8599	126.9842	105.7886	1.32479	2.69E-14	UBTD1
3136.412	3333.814	3176.573	4319.418	4314.315	4338.389	-0.4275	2.73E-14	UBE3C
478.9993	442.8573	486.3871	783.1797	778.6966	799.7616	-0.74521	2.75E-14	RSPRY1
498.7703	513.1993	492.775	779.0304	871.0488	953.155	-0.79085	2.76E-14	AFTPH
7280.249	6669.609	6832.234	9267.454	9095.638	9163.406	-0.40536	2.77E-14	VIM
124.9173	119.8786	94.90481	24.89578	13.64293	23.27348	2.457703	2.83E-14	CMTM8
97.05801	96.10102	79.39152	13.48521	6.296738	15.86829	2.934588	2.91E-14	NPY4R
886.1037	980.8249	1003.801	1552.874	1423.063	1463.056	-0.62916	2.93E-14	ATG5
300.1609	270.4699	280.1517	542.5205	541.5195	505.6694	-0.90127	2.96E-14	FAM149B1
208.495	221.924	215.3609	490.6543	451.2662	391.4177	-1.04655	3.09E-14	USP31
14216.3	13519.53	14395.42	19447.75	18129.36	18032.72	-0.40041	3.16E-14	PTMA
357.6767	335.8582	342.2048	586.0881	614.9814	652.7155	-0.83932	3.18E-14	PPM1D
1051.462	1083.861	1096.881	1567.397	1606.718	1566.729	-0.55271	3.21E-14	NUDT19
461.9242	464.6534	409.7333	1192.923	924.571	732.0569	-1.09285	3.31E-14	TMEM250
1471.148	1438.543	1535.815	2058.051	2129.347	2223.676	-0.52797	3.32E-14	CSNK2A2
1548.435	1527.709	1571.405	2339.166	2311.952	2095.672	-0.53771	3.34E-14	CTBP2
1497.21	1579.227	1460.074	1054.959	919.3238	861.1189	0.67764	3.35E-14	TRIOBP
85.3751	86.1937	98.55499	10.37324	14.69239	14.8104	2.761267	3.36E-14	GNAO1
599.4231	594.4393	664.3336	950.1889	1086.187	1032.496	-0.72343	3.43E-14	DMXL1
162.662	168.4245	187.9845	51.86621	13.64293	35.96811	2.353807	3.43E-14	MRGPRF
1648.189	1704.059	1697.336	2309.084	2430.541	2355.911	-0.49085	3.44E-14	RNF145
211.191	211.026	233.6118	449.1614	461.7608	406.2281	-1.00576	3.50E-14	BBS7
105.1462	101.0547	78.47897	16.59719	15.74185	9.520971	2.76408	3.51E-14	C14orf132
13098.34	12804.22	12620.51	17241.36	15997.91	17569.37	-0.39934	3.64E-14	RPL14
7923.708	8179.485	8369.874	5616.073	5918.934	6364.24	0.451321	3.71E-14	SPTAN1
990.3512	1091.787	1030.265	657.6635	652.7618	556.4479	0.736897	3.73E-14	BAIAP2
79.98299	62.41613	75.74134	2.074648	5.247282	0	4.895248	3.73E-14	-
107.8422	107.9898	90.34208	18.67183	9.445107	21.15771	2.635332	3.85E-14	RAB24

79.0843	94.11956	86.69189	11.41057	13.64293	11.63674	2.823881	3.88E-14	RCOR2
87.17247	73.31418	57.49041	2.074648	4.197825	1.057886	4.893178	3.89E-14	STEAP4
188.7239	181.304	231.7867	423.2282	448.1179	401.9966	-1.08026	3.92E-14	SLC19A2
490.6822	489.4217	468.1362	791.4783	855.3069	775.4302	-0.74215	3.93E-14	ABRAXAS2
3177.751	3297.157	3346.307	4355.724	4453.893	4905.416	-0.48179	4.05E-14	FXR1
1206.934	1154.203	1273.915	1697.062	1923.653	1888.326	-0.59945	4.08E-14	GNAQ
124.9173	120.8693	107.6805	248.9578	334.7766	303.6132	-1.32794	4.09E-14	COBLL1
1081.118	1175.008	1199.998	1647.271	1747.345	1879.863	-0.61	4.25E-14	SETD3
719.8469	688.5589	663.4211	428.4149	365.2108	363.9127	0.839677	4.26E-14	TRMT2A
71.89482	75.29565	71.17861	6.223945	0	1.057886	4.896971	4.28E-14	QPRT
350.4873	354.6821	391.4823	615.1332	650.6629	722.5359	-0.85809	4.40E-14	DCTN6
176.1423	131.7674	126.8439	40.45564	34.63206	21.15771	2.174883	4.56E-14	FAM131C
574.2599	560.7544	560.3034	339.205	269.7103	277.166	0.9358	4.76E-14	POLR2J
342.3991	295.2382	338.5546	645.2156	564.6075	598.7633	-0.8886	4.78E-14	TAF1B
831.2839	798.5301	758.3259	476.1318	451.2662	491.9168	0.750761	4.80E-14	MID1IP1
991.2499	1070.981	1049.428	686.7086	525.7776	512.0167	0.851051	4.82E-14	EFNB1
277.6938	313.0714	315.741	573.6402	544.6678	550.1006	-0.88073	4.83E-14	VRK2
988.5538	930.2975	951.7857	1377.566	1480.783	1454.593	-0.58696	4.86E-14	GTF3C3
114.133	100.0639	93.99226	247.9205	247.6717	280.3397	-1.33133	4.87E-14	RAPH1
271.403	288.3031	292.9273	511.4008	568.8053	512.0167	-0.9013	4.98E-14	PRKACB
2166.73	2255.897	2326.993	1526.941	1554.245	1635.491	0.517048	5.07E-14	PARP14
260.6187	213.9982	238.1746	467.8332	507.9369	446.4278	-0.99524	5.07E-14	WDR47
754.8956	800.5116	817.6414	1205.371	1185.886	1306.489	-0.64002	5.13E-14	UGGT2
805.222	873.8258	823.1167	1375.492	1320.216	1212.337	-0.64381	5.24E-14	RABEPK
475.4045	528.0603	497.3377	793.553	921.4227	836.7876	-0.76632	5.28E-14	CCDC93
8410.796	8452.927	8526.832	6525.806	6700.779	6352.603	0.374962	5.37E-14	C6orf62
107.8422	148.6098	104.0303	31.11972	16.7913	17.98406	2.448781	5.52E-14	AC009299.2
3787.06	4082.807	4138.397	5208.404	5991.346	5961.186	-0.51515	5.52E-14	UBR5
461.9242	465.6441	459.9233	756.2093	762.9548	774.3723	-0.72515	5.64E-14	PIGX
2008.562	2158.805	1919.085	1387.94	1004.33	942.5761	0.867662	5.65E-14	UBL5
1084.713	1200.767	1123.344	2169.045	1989.769	1603.755	-0.75783	5.73E-14	GRWD1
4211.239	4329.5	4346.458	5631.633	5965.11	5572.942	-0.41397	5.80E-14	TGFBR2
926.5445	890.6682	996.5005	1436.694	1409.42	1410.162	-0.59672	5.84E-14	KDM3A
366.6636	302.1733	392.3949	124.4789	125.9348	174.5511	1.321603	5.91E-14	IL33
324.4254	325.9509	305.703	129.6655	145.8744	151.2777	1.163638	5.92E-14	RAB29
2066.976	2182.583	2042.278	2852.641	3017.187	2884.854	-0.47682	6.12E-14	PSMC5
243.5437	235.7943	271.9388	113.0683	71.36303	74.052	1.538774	6.18E-14	SRC
1617.634	1630.745	1563.192	2201.202	2256.331	2388.706	-0.50886	6.25E-14	ATP5PF
404.4084	381.4319	400.6078	645.2156	689.4928	723.5938	-0.79426	6.37E-14	S100PBP
683.8995	693.5125	638.7824	412.855	368.3592	350.1602	0.833131	6.51E-14	ZNF646
1506.197	1537.616	1387.983	915.9572	801.7846	1007.107	0.701624	6.58E-14	NFKBIA
234.5569	211.026	243.6498	445.0121	442.8706	457.0066	-0.96332	6.87E-14	LRIG2
586.8415	681.6237	615.9687	997.9058	1038.962	1008.165	-0.69322	6.87E-14	DPAGT1
115.9304	112.9435	79.39152	12.44789	20.98913	16.92617	2.614084	6.96E-14	RHBDL1
3581.261	3434.868	3482.276	2626.505	2502.953	2287.149	0.501334	6.98E-14	RALY
130.3094	186.2576	160.6081	382.7726	340.0239	371.3179	-1.1996	6.99E-14	DTD1
97.05801	98.08249	83.95425	16.59719	11.54402	16.92617	2.630225	7.20E-14	SULF1
1169.19	1110.611	1214.599	1633.786	1790.373	1788.885	-0.57667	7.21E-14	PAPD4
747.7062	734.1325	742.8126	433.6015	457.563	441.1383	0.739779	7.21E-14	CRTC2
1776.701	1867.53	1702.811	1240.64	944.5107	897.0871	0.794433	7.48E-14	DVL1
365.7649	388.367	365.0185	182.569	182.6054	178.7827	1.040443	7.84E-14	CXCL16
677.6087	675.6793	680.7595	1131.721	1093.534	998.6441	-0.66452	7.88E-14	RBM18
1156.608	1119.527	1135.207	1580.882	1709.564	1752.917	-0.56382	7.97E-14	UBE2E1
1942.059	1849.697	1895.358	2969.859	2723.339	2557.968	-0.53683	8.18E-14	MMADHC

359.4741	397.2836	355.893	187.7557	176.3087	154.4513	1.100675	8.25E-14	SLC2A8
4218.429	4066.956	4191.325	2937.702	3158.864	3120.763	0.43695	8.28E-14	AFF4
124.0186	130.7766	129.5816	32.15705	19.93967	35.96811	2.125872	8.38E-14	HHLA3
297.4648	308.1177	274.6764	109.9564	128.0337	135.4094	1.237228	8.55E-14	RILPL1
155.4726	160.4986	150.5701	356.8395	320.0842	327.9446	-1.10747	8.55E-14	GTPBP8
287.5793	311.0899	331.2543	547.7071	676.8993	560.6794	-0.9411	8.57E-14	HLCS
738.7193	773.7618	737.3373	1159.728	1187.985	1112.896	-0.62158	8.63E-14	HDAC8
1999.575	2105.306	2071.48	2901.396	2742.229	3017.09	-0.48787	8.89E-14	ACAT1
120.4238	116.9064	102.2052	28.00775	12.59348	5.289428	2.88365	8.92E-14	ADRA2C
642.56	679.6423	596.8052	376.5487	244.5233	235.9085	1.162324	8.92E-14	RARG
897.7866	845.0945	889.7326	535.2593	393.5461	314.192	1.082387	8.95E-14	RAVER1
725.239	678.6515	794.8278	1172.176	1128.166	1191.179	-0.66657	8.96E-14	IMPA1
39411.84	42971.03	40941.39	51868.28	53966.19	53502.57	-0.36965	8.98E-14	PKM
72.79351	71.33272	59.3155	4.149297	6.296738	3.173657	3.900101	9.05E-14	ODF3B
876.2181	803.4838	800.303	518.6621	496.3929	447.4856	0.761936	9.09E-14	ZMIZ2
1060.449	1094.759	1126.995	1516.568	1901.615	1853.416	-0.68351	9.10E-14	KNTC1
82.67905	77.27711	64.79078	2.074648	3.148369	0	5.422844	9.14E-14	AC099489.1
741.4154	792.5857	820.3791	1158.691	1257.249	1241.958	-0.63578	9.17E-14	ARMT1
4247.187	4202.686	4251.553	5394.086	6194.941	6328.272	-0.4963	9.31E-14	NUFIP2
103.3488	116.9064	93.07971	18.67183	24.1375	15.86829	2.415754	9.34E-14	PLEKHG6
327.1214	330.9045	380.5318	92.32185	171.0614	111.078	1.472118	9.39E-14	-
131.2081	115.9157	121.3686	34.2317	24.1375	27.50503	2.101189	9.55E-14	AC241585.2
113.2343	96.10102	105.8554	17.63451	19.93967	24.33137	2.349602	9.74E-14	SYNC
2279.965	2258.869	2298.704	1656.607	1609.866	1447.188	0.536565	9.75E-14	NFIC
440.3558	357.6543	374.1439	646.2529	754.5591	763.7935	-0.88386	9.91E-14	BAG4
746.8075	790.6043	712.6986	474.0571	410.3374	388.244	0.82158	1.00E-13	RFXANK
293.8701	268.4884	256.4255	485.4677	512.1347	589.2423	-0.95412	1.01E-13	ZNF473
2062.483	2156.824	2103.419	2777.954	3058.116	3043.537	-0.49005	1.03E-13	JAK1
859.1431	854.0111	896.1204	1261.386	1325.463	1452.477	-0.63024	1.03E-13	PAN3
364.8662	370.5338	428.8967	654.5515	685.295	734.1727	-0.83253	1.05E-13	SGO1
680.3048	645.9574	587.6798	1002.055	1021.121	1051.538	-0.68387	1.07E-13	EXOSC6
1619.431	1534.644	1572.317	1124.459	1015.874	966.9075	0.605117	1.08E-13	AKT1S1
410.6992	421.0612	371.4063	208.5022	196.2483	167.1459	1.072365	1.11E-13	USP20
1388.469	1247.332	1401.671	1884.818	2077.924	2051.24	-0.57432	1.12E-13	7-Mar
762.9838	771.7804	768.3639	1098.526	1281.386	1337.167	-0.69049	1.13E-13	C5orf42
144.6883	144.6469	136.8819	42.53029	18.89021	37.026	2.11357	1.13E-13	C6orf132
199.5081	238.7665	185.2469	501.0276	387.2494	459.1224	-1.11337	1.16E-13	ALG14
212.9884	163.4708	166.0834	64.3141	51.42336	38.08388	1.818255	1.16E-13	LHX2
195.9134	187.2484	208.0605	375.5113	394.5956	439.0226	-1.03144	1.17E-13	DNAH14
583.2467	695.494	630.5694	365.1381	347.3701	344.8707	0.851753	1.20E-13	PINK1
1103.586	1016.491	1090.493	1508.269	1652.894	1767.727	-0.61796	1.22E-13	ZMYM4
562.577	500.3197	572.1665	844.3818	928.7689	978.5442	-0.75008	1.24E-13	CEP95
544.6033	464.6534	502.813	884.8375	883.6422	794.4721	-0.76049	1.25E-13	PSMG2
1251.869	1364.238	1298.553	776.9558	838.5156	896.0292	0.64019	1.27E-13	RAB11B
20530.47	22507.45	20122.56	29413.33	28211.49	27136.88	-0.42445	1.28E-13	PPIA
404.4084	364.5894	392.3949	633.805	687.3939	697.1467	-0.7965	1.28E-13	FAM241A
343.2978	353.6914	333.0794	123.4416	137.4788	179.8406	1.225056	1.29E-13	PDK2
156.3712	154.5542	117.7185	30.0824	37.78043	41.25754	1.974123	1.30E-13	LINC01137
3524.644	3897.54	3594.52	2740.61	2602.652	2538.926	0.482744	1.31E-13	ECE1
866.3326	841.1316	908.896	1338.148	1264.595	1363.615	-0.59994	1.32E-13	SLAIN2
309.1477	269.4792	273.7639	507.2515	541.5195	533.1744	-0.89126	1.32E-13	R3HCC1L
1561.016	1446.469	1285.778	927.3678	736.7184	874.8715	0.757794	1.38E-13	IRX3
193.2173	166.443	184.3343	378.6233	351.5679	460.1803	-1.12853	1.40E-13	ADAT2
66.50271	79.25857	94.90481	236.5099	205.6934	226.3875	-1.47442	1.40E-13	AC083799.1

71.89482	95.11029	67.52842	5.186621	12.59348	5.289428	3.34484	1.42E-13	CCDC107
67.4014	58.4532	56.57787	219.9127	164.7646	178.7827	-1.62626	1.43E-13	NMRAL1
2820.074	3026.687	2711.175	4375.433	3800.081	4094.018	-0.52	1.43E-13	PSMD8
88.96984	63.40686	59.3155	1.037324	7.346194	4.231543	4.072679	1.46E-13	EFEMP2
213.8871	175.3596	198.0225	382.7726	422.9309	399.8808	-1.03608	1.46E-13	FAM200B
174.3449	218.9518	183.4218	473.0198	378.8537	394.5914	-1.11379	1.47E-13	AUNIP
240.8477	262.544	220.8362	98.54579	89.20379	96.2676	1.349734	1.48E-13	STARD10
1693.123	1738.735	1671.785	1221.968	1089.336	1001.818	0.623064	1.50E-13	TFE3
85.3751	79.25857	85.77934	10.37324	15.74185	5.289428	2.994459	1.53E-13	TBKBP1
1871.063	1761.522	1860.682	2505.138	2521.844	2678.567	-0.48793	1.55E-13	PTPN12
572.4625	551.8378	546.6152	958.4875	884.6917	861.1189	-0.69457	1.63E-13	GPN3
806.1207	808.4375	745.5503	1326.738	1173.292	1195.411	-0.64717	1.66E-13	SUPT4H1
587.7402	593.4486	529.2768	289.4134	331.6282	311.0184	0.875764	1.67E-13	CDK10
2138.871	2177.629	2150.871	3250.974	3006.692	2859.465	-0.49553	1.70E-13	MTA2
116.8291	134.7396	141.4447	38.38099	31.48369	28.56291	1.996397	1.70E-13	SP6
337.007	315.0528	344.0299	564.3043	631.7727	583.9529	-0.8369	1.70E-13	CENPC
1864.772	1808.086	1867.982	1327.775	1159.649	1043.075	0.650141	1.71E-13	ARHGEF1
2023.839	2171.685	2076.043	3233.339	2988.852	2797.05	-0.52446	1.72E-13	MAPKAP1
1513.386	1621.829	1435.435	1015.54	939.2634	762.7356	0.749681	1.72E-13	UBXN6
62.00928	63.40686	102.2052	260.3684	261.3146	196.7667	-1.65683	1.74E-13	HSPD1P1
238.1516	196.165	207.148	57.05283	66.11575	89.92028	1.591353	1.82E-13	RALGDS
821.3983	895.6219	850.4931	1382.753	1309.722	1240.9	-0.61593	1.89E-13	CCDC86
120.4238	126.8137	100.3801	24.89578	30.43423	17.98406	2.244203	1.90E-13	PPP1R3B
1623.026	1607.958	1656.271	2225.06	2301.458	2263.875	-0.47436	1.91E-13	RANBP9
1313.878	1252.285	1253.839	844.3818	874.1971	810.3404	0.595159	1.92E-13	C4orf3
2271.876	2103.324	2238.476	1478.187	1516.464	1614.334	0.521327	1.92E-13	MAP1LC3B
181.5344	193.1928	146.9199	57.05283	56.67064	45.48908	1.711009	1.93E-13	CASTOR3
866.3326	867.8814	884.2573	1346.447	1263.545	1300.141	-0.57849	1.93E-13	ZBED4
650.6481	645.9574	604.1056	940.853	1063.099	1044.133	-0.68141	1.96E-13	POU2F1
1337.244	1486.098	1461.899	981.3086	713.6303	734.1727	0.818574	2.01E-13	RAI1
578.7533	594.4393	678.0218	934.629	1266.694	1125.59	-0.84546	2.04E-13	CNOT6L
141.9923	71.33272	95.81735	16.59719	15.74185	13.75251	2.747162	2.05E-13	IGFL1P1
531.123	541.9305	545.7026	802.8889	1014.824	960.5602	-0.77923	2.07E-13	RAB27B
820.4997	725.216	697.1853	383.8099	455.4641	425.27	0.827163	2.07E-13	TMEM63A
179.7371	202.1094	150.5701	58.09015	58.76956	48.66274	1.684284	2.11E-13	TMEM8B
235.4555	222.9147	279.2391	525.9233	437.6233	527.885	-1.0149	2.15E-13	NAF1
2273.674	2381.72	2292.316	1640.009	1708.515	1545.571	0.505298	2.16E-13	MYO9B
248.9358	284.3401	216.2735	91.28452	107.0445	77.22565	1.442611	2.16E-13	HMOX1
1538.549	1653.532	1540.378	2318.419	2300.408	2142.218	-0.51505	2.20E-13	OAT
196.8121	187.2484	231.7867	404.5564	436.5738	408.3439	-1.01969	2.25E-13	RBBP9
557.1849	594.4393	650.6455	989.6072	996.9835	935.1709	-0.69709	2.26E-13	MFAP3
581.4494	560.7544	514.6761	302.8986	270.7597	318.4236	0.893232	2.26E-13	CARD16
849.2576	832.215	905.2458	1269.685	1310.771	1309.662	-0.58837	2.26E-13	HMGXB4
1476.54	1565.357	1511.177	982.346	1055.753	1047.307	0.561232	2.26E-13	MAVS
73.69219	85.20297	98.55499	239.6219	229.8309	212.635	-1.40605	2.27E-13	EIF4BP3
233.6582	239.7572	252.7753	435.6761	464.9092	476.0486	-0.92259	2.29E-13	ZNF614
735.1246	820.3262	835.8923	1182.55	1247.804	1343.515	-0.65847	2.35E-13	FBXO22
302.8569	287.3123	308.4406	558.0804	564.6075	507.7851	-0.8591	2.36E-13	SRBD1
360.3728	352.7007	389.6572	663.8874	625.476	623.0947	-0.79399	2.37E-13	COMMD8
6575.68	6696.359	6655.2	9052.728	8696.845	8318.155	-0.38757	2.37E-13	RAB7A
754.8956	667.7535	678.0218	1027.988	1237.309	1343.515	-0.78015	2.41E-13	CCDC82
116.8291	124.8323	147.8325	22.82113	37.78043	31.73657	2.077481	2.42E-13	DLX3
358.5754	383.4134	342.2048	187.7557	150.0723	118.4832	1.247544	2.43E-13	KCNJ12
457.4308	461.6812	419.7713	951.2262	750.3613	738.4042	-0.86626	2.44E-13	POLR1C

97.05801	92.13809	101.2926	10.37324	20.98913	19.04194	2.528654	2.48E-13	RAB11FIP4
316.3372	321.988	340.3797	154.5613	151.1217	153.3934	1.092183	2.48E-13	TRNP1
2173.021	2067.658	2223.875	2849.529	3507.283	3649.706	-0.63011	2.52E-13	ARRDC3
339.703	370.5338	382.3569	153.524	192.0505	163.9723	1.100399	2.52E-13	GSTM3
2857.819	2675.968	2865.395	1621.338	1902.664	2021.62	0.599156	2.52E-13	SPTSSA
953.5051	884.7238	953.6108	1409.724	1520.662	1335.052	-0.61109	2.54E-13	RPE
115.9304	114.9249	99.46754	255.1817	303.2929	247.5452	-1.28741	2.60E-13	PLS1
967.884	1256.248	905.2458	587.1255	519.4809	581.8371	0.889396	2.61E-13	-
125.8159	106.0083	121.3686	17.63451	19.93967	34.91023	2.286974	2.62E-13	ACVRL1
54.8198	80.24931	67.52842	7.261269	2.098913	2.115771	4.135602	2.64E-13	AC092384.2
321.7293	328.9231	348.5927	573.6402	632.8222	569.1425	-0.82932	2.69E-13	DNAJC16
249.8345	205.0816	206.2354	76.76199	88.15433	79.34143	1.437337	2.70E-13	OSBPL7
269.6056	248.6738	258.2506	91.28452	111.2424	115.3095	1.289537	2.71E-13	DNMT3A
746.8075	713.3272	866.0064	1191.885	1305.524	1245.131	-0.68551	2.72E-13	ARL5B
118.6265	67.36979	46.53986	1.037324	4.197825	1.057886	5.209209	2.73E-13	-
162.662	127.8045	117.7185	32.15705	36.73097	34.91023	1.976284	2.75E-13	-
357.6767	349.7285	360.4558	162.8599	146.9239	189.3615	1.097551	2.93E-13	CDON
1108.079	1032.343	983.7248	665.9621	561.4591	446.4278	0.900058	2.94E-13	NCDN
1216.82	1269.128	1269.352	1960.543	1826.054	1725.412	-0.55387	3.07E-13	ISG20L2
487.9861	568.6803	531.1019	945.0023	834.3178	868.5241	-0.73871	3.08E-13	FNTA
150.0804	182.2947	154.2203	51.86621	48.27499	50.77851	1.687862	3.12E-13	-
821.3983	890.6682	887.9075	1289.394	1326.513	1284.273	-0.58538	3.12E-13	CNDP2
1.797371	2.972197	1.825092	77.79931	50.3739	56.06794	-4.81692	3.18E-13	ESM1
2116.404	1737.744	1976.575	3906.563	3216.584	2781.181	-0.76416	3.25E-13	MTND2P28
903.1787	824.2892	872.3942	521.774	541.5195	549.0427	0.689755	3.30E-13	OSTF1
491.5808	477.5329	403.3454	246.8831	211.9902	224.2718	1.006139	3.30E-13	NUDT1
349.5886	384.4041	382.3569	189.8303	185.7538	152.3355	1.079762	3.31E-13	HTRA2
872.6234	864.9092	890.6451	1249.976	1333.859	1340.341	-0.57816	3.34E-13	TMEM87A
1236.591	1199.777	1289.428	855.7924	810.1803	756.3883	0.621199	3.34E-13	CDT1
1233.895	1279.035	1192.698	823.6354	606.5858	532.1165	0.916754	3.39E-13	SAPCD2
1853.988	1991.372	1878.02	1365.119	1184.836	1048.365	0.669207	3.45E-13	EMC10
494.2769	407.1909	481.8244	768.6572	773.4493	844.1928	-0.7854	3.47E-13	CTTNBP2NL
636.2692	653.8832	700.8355	1113.049	1035.813	999.702	-0.66123	3.54E-13	TNS3
789.0457	812.4004	810.341	1307.028	1211.073	1165.79	-0.61135	3.58E-13	PROSER1
184.2305	190.2206	166.0834	67.42607	59.81901	42.31543	1.671398	3.60E-13	PGAP3
492.4795	559.7637	540.2274	297.712	290.6994	282.4555	0.870158	3.68E-13	ANXA6
4262.464	3952.031	4136.572	5258.196	6073.204	5975.996	-0.48659	3.75E-13	CCNI
883.4076	922.3717	855.9684	1392.089	1293.98	1307.547	-0.58573	3.77E-13	SLC35B1
171.6489	157.5264	146.0074	51.86621	51.42336	42.31543	1.706177	3.79E-13	RGS14
159.0673	137.7118	121.3686	36.30634	41.97825	21.15771	2.071442	3.87E-13	LINC00623
98.85538	94.11956	71.17861	17.63451	11.54402	9.520971	2.768932	3.89E-13	RELL2
1636.506	1640.652	1722.887	2215.724	2475.668	2547.389	-0.53366	4.11E-13	DNAJC13
3645.966	3967.882	3543.417	2805.962	2514.497	2472.279	0.517516	4.15E-13	NNMT
155.4726	155.545	131.4067	337.1303	342.1228	295.1501	-1.13978	4.24E-13	DNER
221.9753	225.8869	196.1974	76.76199	76.61031	88.8624	1.410712	4.27E-13	CXCL11
598.5244	628.1242	583.117	986.4953	978.0933	903.4344	-0.66475	4.38E-13	JADE1
89.86853	82.23077	87.60444	215.7634	229.8309	212.635	-1.34085	4.41E-13	NALCN
210.2924	181.304	211.7107	373.4367	408.2385	467.5855	-1.0487	4.48E-13	FSD1L
355.8794	316.0436	313.0034	113.0683	140.6272	166.0881	1.231272	4.51E-13	ALPK1
211.191	258.5811	228.1366	446.0494	497.4423	430.5595	-0.9788	4.65E-13	ALKBH1
579.652	654.874	595.8927	1295.618	993.8352	975.3706	-0.83549	4.68E-13	RRP9
573.3612	539.949	555.7406	836.0833	921.4227	1021.918	-0.73531	4.69E-13	ATF2
375.6504	408.1817	458.0982	682.5593	734.6194	743.6936	-0.79898	4.75E-13	ETAA1
813.3102	844.1038	832.2422	1227.154	1299.227	1208.105	-0.58516	4.77E-13	OSBPL11

572.4625	587.5042	536.5772	342.317	243.4739	230.6191	1.054587	4.81E-13	GPR137
4327.17	4510.804	4130.184	2105.768	1528.008	1403.814	1.364103	4.82E-13	FSTL3
5179.123	5398.5	5628.585	6850.489	7553.987	7574.461	-0.43957	4.93E-13	TEAD1
619.1942	704.4106	554.8281	1016.578	1080.94	1019.802	-0.73176	4.94E-13	MRPS11
272.3016	250.6552	317.5661	92.32185	133.281	101.557	1.362084	5.02E-13	TMEM51
81.78036	100.0639	88.51698	8.298593	8.395651	21.15771	2.838291	5.03E-13	SULT1A2
1688.63	1740.716	1574.142	1105.788	1187.985	1092.796	0.562886	5.05E-13	GYS1
260.6187	228.8591	234.5244	112.031	76.61031	82.51508	1.416684	5.09E-13	XRCC3
849.2576	869.8629	841.3676	558.0804	517.382	451.7172	0.745182	5.12E-13	WDTC1
1099.092	964.9731	1148.896	1547.688	1679.13	1813.216	-0.64884	5.21E-13	PAXBP1
493.3782	467.6256	463.5735	263.4803	263.4135	231.677	0.909163	5.23E-13	ABHD4
535.6164	540.9398	511.0259	830.8966	833.2683	973.2548	-0.73236	5.25E-13	NFYC
1751.538	1708.022	1743.876	1209.52	1213.172	1243.016	0.50551	5.32E-13	LITAF
1978.905	2314.35	2164.56	3613	3240.721	2951.501	-0.60287	5.32E-13	PES1
187.8252	197.1557	192.5473	69.50072	76.61031	60.29948	1.483928	5.32E-13	PPP1R35
2457.904	2529.339	2510.415	3287.28	3606.981	3353.498	-0.45086	5.36E-13	PANK3
514.9467	584.532	469.9613	301.8613	217.2375	208.4035	1.108228	5.39E-13	SAC3D1
1054.158	1116.555	1144.333	1582.957	1607.767	1611.16	-0.53471	5.43E-13	RMDN1
114.133	113.9342	93.99226	25.9331	17.84076	24.33137	2.240875	5.45E-13	HOGA1
695.5824	623.1705	725.4742	1108.9	1026.368	1115.012	-0.66825	5.50E-13	TMEM126B
1407.341	1525.728	1690.036	2225.06	2268.925	2341.101	-0.5641	5.53E-13	FTSJ3
929.2406	921.3809	904.3333	521.774	608.6847	578.6635	0.688905	5.54E-13	JRK
3158.879	3066.316	3109.958	2348.502	2326.645	2189.823	0.443448	5.55E-13	TNS4
1710.198	1823.938	1775.815	2488.541	2436.838	2406.69	-0.4658	5.58E-13	YTHDF1
146.4857	124.8323	153.3078	31.11972	44.07717	41.25754	1.867908	5.60E-13	AC089983.1
276.7951	302.1733	271.0262	519.6994	492.195	534.2323	-0.86396	5.60E-13	MSANTD3
425.0781	423.0426	404.258	227.174	216.188	205.2298	0.949039	5.64E-13	MIER2
943.6195	895.6219	846.8429	557.0431	413.4858	326.8867	1.049504	5.66E-13	RIN1
282.1872	259.5718	264.6384	468.8705	506.8874	503.5536	-0.87476	5.67E-13	TRMT10A
3896.699	4165.038	4289.88	5284.129	5937.824	5896.655	-0.4709	5.73E-13	CD109
234.5569	182.2947	256.4255	425.3029	490.0961	487.6853	-1.05726	6.02E-13	CEP83
2442.627	2254.906	2216.575	1689.801	1424.112	1335.052	0.636027	6.08E-13	RELA
252.5306	255.6089	225.3989	431.5268	493.2445	472.8749	-0.93043	6.18E-13	FOXO1
2178.413	2113.232	1936.423	1331.924	1420.964	1490.561	0.553597	6.19E-13	GOLGA3
762.9838	776.734	699.0104	430.4895	470.1564	435.8489	0.743997	6.29E-13	INSR
840.2707	879.7702	854.1433	570.5283	459.6619	503.5536	0.746709	6.32E-13	ADCY9
5060.497	5183.511	5209.726	6904.429	6798.378	6458.392	-0.38368	6.37E-13	VAPA
78.18562	95.11029	90.34208	1.037324	2.098913	0	6.389235	6.50E-13	ARL2BP
337.9057	364.5894	357.7181	564.3043	644.3662	701.3782	-0.84947	6.51E-13	STAM2
1304.891	1295.878	1414.447	1938.759	1885.873	1906.31	-0.51311	6.63E-13	VHL
217.4818	285.3309	240.9122	107.8817	80.80814	93.09394	1.398763	6.64E-13	SEC14L2
89.86853	89.1659	81.21661	8.298593	12.59348	19.04194	2.706257	6.68E-13	HNF4A
378.3465	421.0612	359.5432	186.7183	172.1108	204.1719	1.041063	6.70E-13	IFT140
74.59088	61.4254	75.74134	8.298593	9.445107	4.231543	3.267761	6.71E-13	AC092902.2
263.3148	277.405	294.7524	127.5909	122.7864	118.4832	1.179333	6.78E-13	CCDC120
1009.224	985.7785	995.5879	650.4022	620.2287	665.4101	0.627439	7.03E-13	TOP1MT
1528.664	1459.348	1450.036	2335.017	2127.248	2011.041	-0.54455	7.24E-13	SF3B6
233.6582	210.0352	208.9731	85.06058	64.01684	90.97817	1.443378	7.26E-13	MYRF
643.4587	612.2725	555.7406	963.6741	935.0656	995.4704	-0.676	7.45E-13	GPX8
627.2823	597.4115	719.999	1021.764	1026.368	1099.143	-0.69391	7.54E-13	STXBP3
512.2506	544.9027	537.4897	814.2994	868.9499	863.2347	-0.67555	7.70E-13	LIG3
967.884	917.418	957.261	1320.514	1461.893	1548.745	-0.60718	7.90E-13	FBXL3
70.99614	85.20297	50.19004	2.074648	1.049456	8.463085	4.158896	8.12E-13	FRY
7527.388	7996.199	7398.012	13002.86	11165.17	9959.994	-0.57433	8.18E-13	HMGA1

464.6203	447.8109	490.9499	816.3741	771.3504	730.999	-0.7241	8.49E-13	LLPH
1052.36	1145.286	1078.63	1605.778	1649.745	1530.761	-0.54732	8.50E-13	C9orf78
299.2622	344.7748	323.9539	539.4086	587.6956	598.7633	-0.83507	8.59E-13	STK26
1022.704	1137.361	1037.565	1751.003	1582.58	1517.008	-0.60175	8.60E-13	MAPKAPK3
170.7502	173.3781	175.2089	64.3141	48.27499	61.35737	1.57783	8.61E-13	AKR1C2
222.8739	236.785	237.262	434.6388	449.1673	424.2122	-0.90868	8.63E-13	TADA1
2170.325	2265.804	2205.624	1641.047	1296.079	1276.868	0.656144	8.65E-13	MRPL28
279.4911	275.4235	276.5015	514.5128	471.2059	519.4219	-0.85616	8.77E-13	RPL24P4
151.8778	132.7581	121.3686	34.2317	38.82988	39.14177	1.855987	8.89E-13	NEURL1B
1722.78	1773.411	1729.275	1256.2	1140.759	1231.379	0.526105	9.04E-13	MED15
1382.178	1469.256	1361.519	2076.723	1977.176	1936.989	-0.50834	9.06E-13	LUZP1
5340.887	5425.249	5646.836	6951.109	7298.969	7853.743	-0.42942	9.06E-13	HIF1A
2105.62	2131.065	2135.358	2905.545	2903.846	2788.587	-0.4323	9.34E-13	RFWD3
779.1601	721.253	809.4285	1151.43	1220.518	1159.443	-0.61185	9.44E-13	SAV1
20.66976	18.82391	26.46384	94.3965	92.35216	135.4094	-2.28541	9.68E-13	H3F3AP6
16.17634	14.86098	10.95055	80.91128	72.41249	103.6728	-2.6142	9.73E-13	RPL5P4
1041.576	984.7878	975.5119	1416.985	1501.772	1470.461	-0.54787	9.78E-13	MTFR1
1270.741	1150.24	1252.926	1766.563	1720.059	1850.242	-0.53816	9.84E-13	ZCRB1
315.4385	303.164	318.4786	144.1881	150.0723	149.1619	1.079816	1.00E-12	NIT1
771.072	767.8174	720.9115	1281.095	1292.93	1063.175	-0.68685	1.00E-12	CHPT1
465.519	467.6256	515.5886	792.5156	818.5759	759.5619	-0.71027	1.01E-12	GATC
2218.854	2371.813	2420.985	3119.234	3295.293	3668.748	-0.52417	1.03E-12	NCOR1
677.6087	682.6145	622.3565	1122.385	1090.385	960.5602	-0.67896	1.03E-12	TUBBP2
873.5221	802.4931	850.4931	1285.245	1254.1	1231.379	-0.57724	1.06E-12	UBE2T
363.0689	365.5802	344.0299	170.1212	187.8527	172.4354	1.015925	1.07E-12	SMTN
71.89482	65.38832	52.01513	4.149297	6.296738	2.115771	3.9121	1.07E-12	SLC15A3
1978.006	2045.862	2097.944	2766.543	2771.614	2758.966	-0.43871	1.08E-12	SRP68
1393.861	1423.682	1377.945	1968.841	2098.913	1897.847	-0.50798	1.08E-12	GDE1
147.3844	149.6006	116.8059	44.60494	34.63206	31.73657	1.897425	1.08E-12	SPATA2L
351.3859	357.6543	344.9425	565.3417	613.932	656.947	-0.80095	1.08E-12	ASB7
9212.423	9487.251	9599.074	14354.49	13787.76	11744.65	-0.49521	1.09E-12	VCP
331.6149	293.2567	288.3646	152.4866	115.4402	89.92028	1.351162	1.09E-12	NUDT22
316.3372	305.1455	302.0528	160.7852	118.5886	102.6149	1.273009	1.10E-12	MSRB2
79.0843	70.34198	58.40296	7.261269	4.197825	9.520971	3.309154	1.13E-12	PDZD7
2862.313	3350.656	3036.954	2276.926	1885.873	1913.715	0.605873	1.13E-12	QSOX1
143.7896	145.6376	150.5701	292.5254	319.0347	320.5394	-1.08284	1.14E-12	RTKN2
550.8941	510.2271	509.2008	310.1599	264.463	216.8666	0.98817	1.15E-12	NDOR1
739.618	816.3633	728.2119	463.6839	361.013	273.9924	1.055228	1.17E-12	SELENOH
604.8152	548.8656	585.8547	888.9868	916.1754	935.1709	-0.65498	1.20E-12	ENOSF1
295.6675	328.9231	312.0908	547.7071	525.7776	604.0527	-0.84134	1.20E-12	TIMM9
543.7046	530.0417	532.927	326.7571	286.5016	261.2978	0.877219	1.20E-12	BRF1
395.4215	375.4875	443.4975	202.2782	217.2375	183.0142	1.011501	1.20E-12	ENGASE
536.5151	560.7544	528.3643	285.2641	316.9358	313.1342	0.8285	1.21E-12	DHRS3
60.21191	60.43466	52.92768	153.524	205.6934	166.0881	-1.59808	1.26E-12	HSP90AB3P
154.5739	162.4801	154.2203	12.44789	50.3739	33.85234	2.286559	1.27E-12	LINC00689
185.1292	261.5533	198.9351	54.97818	89.20379	62.41525	1.642863	1.31E-12	PKD1P6
1096.396	1090.796	1060.379	652.4769	691.5917	751.0988	0.632425	1.31E-12	PBX2P1
420.5847	474.5607	410.6458	691.8952	769.2515	743.6936	-0.75665	1.31E-12	ZBTB21
3868.84	3908.438	3983.264	5740.552	5314.447	4990.047	-0.44822	1.34E-12	PRDX3
877.1168	864.9092	880.6071	1491.672	1381.085	1216.569	-0.64089	1.38E-12	CTBP1
766.5785	858.9648	804.8658	1282.133	1280.337	1161.558	-0.61631	1.38E-12	UBE2E3
70.99614	61.4254	56.57787	2.074648	1.049456	7.4052	4.171175	1.39E-12	CSAG3
128.512	161.4893	128.669	297.712	294.8972	357.5654	-1.18432	1.42E-12	PANK1
522.1361	641.9945	624.1816	1051.847	1093.534	904.4923	-0.77093	1.42E-12	GRPEL2

2118.201	2190.509	2066.917	1547.688	1547.948	1445.072	0.489426	1.44E-12	GLB1
34.15004	39.62929	53.84023	128.6282	158.4679	161.8565	-1.81401	1.45E-12	AC113191.1
710.8601	630.1057	566.6912	1057.033	1035.813	1012.397	-0.70269	1.45E-12	METTL5
1232.098	1328.572	1254.751	1958.468	1999.214	1712.717	-0.57198	1.46E-12	POM121C
12773.01	13776.13	12967.28	10525.73	9999.22	9764.285	0.38356	1.46E-12	P4HB
203.1029	209.0445	271.9388	410.7804	499.5412	544.8111	-1.08815	1.50E-12	FAM133B
5584.43	5891.884	5336.57	4324.604	4021.517	3731.163	0.477109	1.51E-12	TNFRSF1A
3643.27	3603.293	3819.006	4676.257	5210.551	5381.464	-0.46435	1.51E-12	NAMPT
349.5886	394.3114	393.3074	209.5395	168.9625	167.1459	1.058708	1.52E-12	P4HTM
177.041	184.2762	203.4978	78.83663	58.76956	47.60486	1.607722	1.53E-12	ZGPAT
144.6883	138.7025	114.9808	36.30634	24.1375	41.25754	1.969794	1.55E-12	MDK
1250.97	1258.23	1224.637	883.8002	778.6966	811.3983	0.593702	1.56E-12	MEA1
854.6497	898.5941	793.9152	564.3043	469.107	470.7591	0.759377	1.58E-12	ASMTL
126.7146	144.6469	137.7945	37.34367	44.07717	21.15771	1.994604	1.59E-12	SPSB2
1013.717	1081.88	1037.565	1488.56	1486.03	1556.15	-0.53244	1.63E-12	IWS1
19693.79	21878.34	20691.07	30243.19	28143.27	26174.21	-0.44166	1.64E-12	LDHB
721.6443	629.1149	699.0104	396.2578	423.9804	373.4336	0.780593	1.66E-12	CCDC9B
378.3465	446.8202	421.5964	729.2389	730.4216	664.3522	-0.7696	1.66E-12	AC125807.2
1090.105	1037.297	1171.709	1688.764	1532.206	1682.038	-0.57121	1.67E-12	TPM1
226.4687	289.2938	256.4255	104.7697	110.1929	105.7886	1.266472	1.67E-12	PELI3
116.8291	146.6284	121.3686	40.45564	31.48369	26.44714	1.96565	1.69E-12	FP565260.6
289.3767	302.1733	284.7144	502.0649	531.0249	516.2482	-0.82262	1.70E-12	DCAF4
190.5213	156.5357	282.8893	47.71691	65.06629	68.76257	1.796503	1.72E-12	-
244.4424	242.7294	234.5244	484.4304	431.3266	432.6752	-0.90202	1.72E-12	MTFMT
230.0634	232.8221	226.3115	84.02326	62.96738	105.7886	1.447317	1.76E-12	PRKCZ
2520.812	2459.988	2451.099	3180.436	3536.668	3714.237	-0.48901	1.77E-12	PRKCI
779.1601	831.2243	761.0635	519.6994	464.9092	444.312	0.730283	1.80E-12	GORASP1
83.57773	138.7025	32.85166	6.223945	5.247282	4.231543	4.019055	1.80E-12	IFI27L1
158.1686	212.0167	201.6727	394.1832	371.5075	424.2122	-1.05867	1.83E-12	UBXN8
80.88168	74.30491	81.21661	8.298593	16.7913	5.289428	2.959954	1.83E-12	C5AR1
1137.736	1132.407	1225.55	1653.495	1643.449	1740.222	-0.52681	1.84E-12	RBM17
1525.069	1696.133	1424.485	2218.836	2276.271	2253.296	-0.5392	1.86E-12	FBXW11
179.7371	178.3318	135.0568	60.1648	38.82988	46.54697	1.759493	1.87E-12	LMF1
779.1601	746.0213	820.3791	1185.661	1208.974	1140.401	-0.59141	1.90E-12	TMEM230
3894.003	4084.789	3863.721	3018.613	2569.069	2355.911	0.575939	1.90E-12	TMED9
422.3821	462.6719	492.775	811.1875	738.8173	746.8673	-0.73757	1.91E-12	SMG8
762.0851	890.6682	813.0787	1451.216	1252.001	1226.089	-0.67296	1.93E-12	RPA2
680.3048	647.9388	636.9573	420.1163	321.1336	298.3238	0.918375	1.94E-12	BCL7C
650.6481	635.0593	620.5314	909.7333	1066.248	1109.722	-0.69469	1.94E-12	LSM12
741.4154	784.6599	837.7174	1135.87	1303.425	1228.205	-0.63368	1.98E-12	PARG
597.6257	642.9852	585.8547	362.0261	363.1119	303.6132	0.82755	1.98E-12	SIRT7
861.8392	861.937	803.9532	538.3712	532.0744	443.2541	0.739444	2.01E-12	PATZ1
579.652	600.3837	606.8432	918.0319	903.5819	941.5183	-0.62898	2.02E-12	NAB1
144.6883	131.7674	110.4181	29.04508	29.38478	41.25754	1.95714	2.02E-12	ACCS
23.36582	19.81464	29.20148	99.58312	110.1929	104.7307	-2.11602	2.03E-12	SEMA3D
121.3225	128.7952	120.4561	260.3684	287.551	271.8766	-1.14611	2.06E-12	BMP5
265.1122	268.4884	251.8628	121.3669	108.094	116.3674	1.183199	2.07E-12	AC104024.2
210.2924	211.026	198.0225	78.83663	73.46194	87.80451	1.367121	2.08E-12	TLCD1
584.1454	546.8842	593.155	877.5762	930.8678	894.9713	-0.64832	2.11E-12	RANBP6
122.2212	115.9157	113.1557	22.82113	32.53315	32.79446	1.99563	2.21E-12	ST6GAL1
1104.484	1138.351	1247.451	1610.964	1876.428	1764.553	-0.58937	2.23E-12	ACAP2
2003.169	1981.464	1898.096	2943.926	2655.125	2635.193	-0.48525	2.25E-12	ACP1
656.9389	613.2632	669.8089	1081.929	1059.951	935.1709	-0.66512	2.28E-12	RFC2
703.6706	643.9759	614.1436	315.3465	391.4472	387.1862	0.843011	2.33E-12	CLSTN3

218.3805	237.7757	225.3989	426.3402	456.5135	404.1123	-0.91778	2.33E-12	VPS50
386.4347	385.3948	333.9919	177.3824	189.9516	180.8985	1.012024	2.34E-12	PIM2
1025.4	1012.528	980.0746	695.0072	630.7233	615.6895	0.636307	2.37E-12	HIST1H2BK
286.6806	221.924	302.0528	65.35142	85.00596	124.8305	1.560366	2.40E-12	LUCAT1
2361.745	2269.767	2461.137	3120.271	3292.145	3197.988	-0.43804	2.45E-12	RBBP4
1596.065	1691.18	1766.689	2514.474	2488.261	2238.486	-0.51896	2.46E-12	GSR
1376.786	1299.841	1321.367	881.7255	676.8993	520.4798	0.943103	2.49E-12	RNF126
5694.969	5450.018	5711.627	7407.532	7157.292	7124.86	-0.3636	2.50E-12	YWHAG
602.1191	576.6061	529.2768	308.0853	346.3206	304.6711	0.832581	2.50E-12	SPSB1
326.2228	298.2104	323.9539	537.3339	552.014	559.6215	-0.79723	2.62E-12	LZTFL1
1822.534	1887.345	1873.457	2452.234	2557.525	2593.936	-0.44565	2.65E-12	TAX1BP1
1352.521	1395.942	1437.26	1869.258	2013.907	2067.109	-0.50745	2.66E-12	WWP1
463.7216	462.6719	476.3491	713.679	768.202	793.4143	-0.69761	2.68E-12	ARFIP1
991.2499	946.1492	996.5005	612.0212	668.5037	614.6316	0.630763	2.69E-12	TRERF1
989.4525	886.7053	965.4739	549.7818	587.6956	635.7893	0.680918	2.70E-12	EVPL
96.15932	78.26784	74.82879	11.41057	16.7913	12.69463	2.608527	2.71E-12	BTBD19
964.2893	1035.315	897.0329	650.4022	527.8765	563.8531	0.732982	2.76E-12	TTC7A
1650.885	1606.968	1732.013	1026.951	1151.254	1191.179	0.566811	2.77E-12	ZMYM2
6430.992	6554.684	6409.725	7973.911	8443.926	8547.716	-0.36425	2.78E-12	NT5E
961.5932	879.7702	905.2458	608.9093	457.563	404.1123	0.901088	2.79E-12	NR1H2
1560.118	1575.264	1498.401	2038.342	2412.7	2577.01	-0.60092	2.84E-12	DOCK5
1476.54	1533.653	1366.082	2121.328	2081.072	2008.925	-0.50556	2.92E-12	ARPC5L
502.3651	530.0417	448.0602	290.4508	237.1771	205.2298	1.013626	2.94E-12	NAGLU
81.78036	131.7674	94.90481	23.85846	19.93967	13.75251	2.419103	2.96E-12	PGF
1085.612	1123.49	1193.61	1576.733	1682.279	1656.649	-0.53065	2.96E-12	SEC22C
155.4726	138.7025	162.4332	35.26902	27.28586	58.18371	1.920552	2.98E-12	SERPINB3
7235.315	7463.185	7280.294	9137.788	9628.762	9237.458	-0.34958	3.01E-12	RPN1
1897.125	1964.622	1814.142	837.1206	614.9814	499.322	1.540184	3.02E-12	SSBP4
383.7386	382.4226	427.0716	670.1114	643.3167	685.5099	-0.74399	3.09E-12	IFIT5
4663.278	4804.06	4830.107	5953.203	6369.151	6260.567	-0.37824	3.10E-12	SSFA2
157.2699	206.0723	187.072	65.35142	65.06629	62.41525	1.512008	3.10E-12	ZNF784
1463.958	1510.867	1520.302	2052.864	2029.649	2149.624	-0.47143	3.10E-12	FOXJ3
647.0534	620.1983	570.3414	911.8079	974.9449	1045.191	-0.67395	3.12E-12	E2F7
79.98299	106.9991	99.46754	23.85846	16.7913	12.69463	2.422344	3.15E-12	SH3RF3-AS1
105.1462	103.0361	94.90481	26.97043	19.93967	20.09983	2.176382	3.16E-12	HLA-H
967.884	1071.972	1093.23	1509.307	1585.729	1508.545	-0.55547	3.18E-12	TTC19
138.3975	137.7118	120.4561	317.4212	288.6005	267.6451	-1.14013	3.21E-12	TBCCD1
98.85538	89.1659	86.69189	17.63451	22.03858	10.57886	2.450009	3.21E-12	GALNT9
148.2831	191.2113	139.6196	42.53029	45.12662	57.12583	1.725694	3.30E-12	DNAL4
142.891	107.9898	122.2812	270.7416	302.2434	276.1082	-1.18401	3.34E-12	RFXAP
374.7518	354.6821	338.5546	595.4241	590.8439	630.4999	-0.76627	3.39E-12	STRADB
296.5661	307.127	328.5166	142.1134	157.4185	142.8146	1.075534	3.41E-12	MBD5
106.9435	115.9157	119.5436	302.8986	285.4521	225.3296	-1.24935	3.45E-12	LINC01963
81.78036	115.9157	84.8668	23.85846	8.395651	8.463085	2.790704	3.45E-12	CYP51A1
430.4702	481.4958	479.0868	707.4551	798.6363	802.9352	-0.7315	3.46E-12	RNF20
75.48956	54.49027	84.8668	7.261269	11.54402	5.289428	3.157476	3.47E-12	MAFA
648.8508	638.0315	632.3945	407.6684	357.8646	373.4336	0.752732	3.47E-12	CSF1
162.662	103.0361	123.1937	292.5254	326.3809	308.9026	-1.25156	3.49E-12	MSANTD2
150.0804	206.0723	173.3838	70.53804	44.07717	45.48908	1.723704	3.54E-12	APC2
83.57773	73.31418	59.3155	5.186621	10.49456	10.57886	3.043475	3.55E-12	DNAAF3
325.3241	304.1548	279.2391	538.3712	595.0417	508.843	-0.85371	3.55E-12	LIG4
1780.296	1641.643	1665.397	2301.822	2487.212	2322.059	-0.4829	3.57E-12	SH3GLB1
696.4811	686.5774	763.8012	1030.063	1144.957	1163.674	-0.63666	3.59E-12	HOOK3
625.485	620.1983	579.4668	385.8846	296.9961	268.703	0.939118	3.64E-12	NAB2

1352.521	1375.136	1375.207	2102.656	1995.017	1815.332	-0.5274	3.69E-12	PPIAP22
4248.085	3497.285	4009.728	5179.359	6511.877	7038.113	-0.6718	3.69E-12	OGT
497.8716	491.4032	438.9347	253.1071	277.0565	226.3875	0.916408	3.74E-12	TXNDC5
325.3241	332.886	372.3189	588.1628	567.7559	674.9311	-0.82881	3.80E-12	DUXAP8
1230.3	1356.312	1296.728	2063.238	1852.29	1787.827	-0.55495	3.85E-12	PHC2
638.9652	653.8832	651.558	1017.615	961.302	976.4285	-0.60416	3.85E-12	DPY30
2630.452	2760.18	2413.685	1924.236	1526.959	1457.766	0.668593	3.87E-12	GUK1
677.6087	722.2438	622.3565	422.1909	362.0624	302.5553	0.895132	3.93E-12	MVB12A
11.68291	10.89805	20.98856	74.68734	109.1435	80.39931	-2.59608	3.96E-12	AC048344.4
788.147	660.8184	710.8735	1081.929	1203.726	1116.069	-0.65454	4.02E-12	PURA
340.6017	334.8675	355.893	183.6064	163.7152	165.0302	1.00934	4.07E-12	TBC1D2
105.1462	86.1937	124.1063	16.59719	11.54402	29.6208	2.451526	4.10E-12	TP53INP1
183.3318	188.2391	185.2469	77.79931	43.02771	62.41525	1.602795	4.12E-12	SOX17
916.659	1002.621	928.9721	624.4691	607.6352	608.2843	0.629592	4.17E-12	C1QTNF6
626.3836	669.735	631.482	417.0043	311.6885	319.4815	0.878333	4.21E-12	SIL1
902.28	873.8258	768.3639	501.0276	535.2227	401.9966	0.822789	4.25E-12	C15orf39
364.8662	338.8304	273.7639	51.86621	125.9348	125.8884	1.687203	4.26E-12	-
1076.625	1001.63	1048.516	1437.731	1615.113	1530.761	-0.55141	4.32E-12	FAM8A1
107.8422	142.6654	125.9314	310.1599	263.4135	282.4555	-1.18743	4.32E-12	KCNQ5
363.0689	359.6358	394.22	617.2079	653.8113	609.3421	-0.75115	4.46E-12	KATNA1
6276.418	6065.262	6122.273	7594.25	8732.526	8854.503	-0.44757	4.48E-12	WNK1
678.5074	651.9018	664.3336	433.6015	371.5075	339.5813	0.801076	4.60E-12	ZNF274
3894.003	4138.288	4226.002	5168.986	5642.927	5614.199	-0.42228	4.60E-12	RLIM
549.9954	663.7906	594.0676	1039.399	943.4613	937.2867	-0.69282	4.60E-12	CSTF2
15672.17	16074.63	15304.31	22199.77	19795.9	19878.73	-0.39515	4.61E-12	RPL7
2625.958	2540.237	2547.829	241.6965	128.0337	428.4437	3.272777	4.88E-12	-
15186.88	15673.38	14765.91	21952.89	19142.08	19671.38	-0.41345	4.89E-12	RPS8
2217.955	2547.172	2382.658	3327.736	3223.93	3303.777	-0.46382	4.91E-12	NDUFS5
1655.378	1723.874	1493.838	1160.766	1050.506	987.0073	0.607159	4.93E-12	CLPP
429.5716	461.6812	438.9347	229.2486	257.1168	232.7348	0.887042	5.14E-12	ZFYVE1
186.9265	185.2669	216.2735	360.9888	395.645	397.765	-0.97143	5.14E-12	PIGM
1838.71	1936.881	1699.161	1329.85	1153.353	1077.986	0.620066	5.22E-12	AP1M1
64.70534	59.44393	55.66532	1.037324	7.346194	1.057886	4.252418	5.27E-12	EMC6
117.7278	139.6932	130.4941	280.0775	289.65	271.8766	-1.11877	5.30E-12	HOXD13
252.5306	292.266	237.262	478.2064	481.7005	478.1643	-0.88024	5.42E-12	WARS2
315.4385	256.5996	305.703	523.8487	505.838	589.2423	-0.88168	5.43E-12	DENND6A
1312.979	1265.165	1318.629	1781.086	1809.263	2017.388	-0.52487	5.59E-12	SRP54
123.1199	131.7674	121.3686	42.53029	31.48369	28.56291	1.873681	5.66E-12	TCN1
889.6984	866.8907	892.4702	1220.931	1372.689	1332.936	-0.56754	5.67E-12	USP28
414.2939	401.2465	396.9576	221.9874	218.2869	205.2298	0.909455	5.68E-12	STN1
3622.6	3808.374	3860.071	4931.439	4868.428	4884.258	-0.37917	5.83E-12	NCOA4
3107.654	3423.97	3196.649	2426.301	2321.397	2047.009	0.517503	5.84E-12	ABL1
569.7665	516.1715	593.155	822.598	952.9064	1004.991	-0.72687	5.89E-12	GON4L
268.7069	243.7201	219.0111	117.2176	72.41249	85.68874	1.409136	5.91E-12	SNX21
4028.806	4252.223	3517.866	881.7255	465.9586	368.1442	2.781599	5.96E-12	TSPO
368.461	424.0334	381.4443	616.1705	672.7015	739.4621	-0.7897	5.98E-12	ZNF721
421.4834	379.4504	398.7827	625.5065	683.1961	698.2045	-0.74153	6.00E-12	GPALPP1
977.7696	948.1307	1010.189	1433.582	1489.179	1356.209	-0.54315	6.12E-12	SLF1
268.7069	360.6265	344.9425	551.8564	634.9211	635.7893	-0.90472	6.16E-12	EFL1
616.4981	613.2632	551.1779	348.5409	313.7874	360.739	0.799584	6.20E-12	UNC119
825.8918	855.0019	799.3905	1268.647	1215.27	1180.6	-0.56349	6.24E-12	RNF7
107.8422	125.823	136.8819	239.6219	328.4798	308.9026	-1.24322	6.29E-12	B4GALT6
1406.442	1367.21	1393.458	1840.213	1997.115	2013.156	-0.48931	6.38E-12	CCDC50
596.727	500.3197	615.9687	881.7255	940.3129	1063.175	-0.75106	6.49E-12	EYA4

1419.024	1264.174	1297.641	712.6417	777.6472	941.5183	0.711479	6.49E-12	RPL3P4
363.9675	402.2373	351.3303	203.3155	183.6549	175.609	0.989322	6.53E-12	METTL23
260.6187	316.0436	262.8133	128.6282	129.0831	94.15183	1.253147	6.64E-12	SLC48A1
2523.508	2612.561	2594.369	3404.498	3424.376	3342.919	-0.39607	6.65E-12	AHSA1
727.0364	699.4569	669.8089	1004.13	1133.413	1076.928	-0.61661	6.75E-12	APBB2
2305.128	2284.628	2307.829	1576.733	1724.257	1705.312	0.462454	6.84E-12	COL4A5
1598.761	1395.942	1400.758	2264.479	2029.649	2115.771	-0.54398	6.86E-12	SPATS2L
45.83295	38.63855	28.28893	173.2331	134.3304	114.2517	-1.90348	6.90E-12	SFTA1P
944.5182	987.76	939.9226	1505.157	1422.013	1317.068	-0.56371	6.95E-12	NXF1
1806.357	1659.476	1669.047	1025.914	1259.348	1099.143	0.60166	6.96E-12	SPG7
139.2962	145.6376	167.9085	35.26902	54.57173	48.66274	1.709883	7.22E-12	AL365181.2
612.0047	617.2261	653.3831	1019.69	967.5988	918.2448	-0.62604	7.25E-12	COMMD9
88.96984	113.9342	79.39152	12.44789	19.93967	21.15771	2.398256	7.30E-12	NUPR1
310.0464	327.9324	278.3266	109.9564	162.6657	101.557	1.291704	7.31E-12	WIPI1
348.6899	404.2187	409.7333	779.0304	713.6303	599.8212	-0.84849	7.43E-12	NIF3L1
159.0673	166.443	187.9845	362.0261	322.1831	354.3917	-1.01607	7.49E-12	AC113935.1
1374.988	1529.69	1488.363	2064.275	2139.841	1994.114	-0.49702	7.50E-12	TIMELESS
578.7533	631.0964	632.3945	395.2205	284.4027	298.3238	0.913087	7.53E-12	ACAA1
110.5383	104.0269	87.60444	25.9331	17.84076	23.27348	2.171622	7.56E-12	PLXNB3
62.90797	72.32345	73.0037	6.223945	9.445107	11.63674	2.932333	7.57E-12	ATP6V0C
164.4594	188.2391	122.2812	32.15705	52.47282	48.66274	1.833009	7.59E-12	VKORC1
489.7835	445.8295	448.9727	265.555	241.375	253.8926	0.864087	7.66E-12	ACOT8
1117.964	1114.574	1060.379	749.9854	669.5531	545.869	0.744226	7.85E-12	CHID1
700.9745	662.7998	715.4362	453.3106	373.6065	326.8867	0.849511	7.90E-12	SELENOO
2173.92	2258.869	2352.544	3210.518	3050.77	2963.138	-0.44311	7.91E-12	MFSD14B
738.7193	639.0223	746.4628	1031.1	1139.71	1233.495	-0.67957	8.10E-12	SCML1
81.78036	66.37906	107.6805	9.335917	16.7913	14.8104	2.646135	8.14E-12	HSPD1P11
259.72	285.3309	284.7144	476.1318	494.2939	497.2063	-0.82319	8.18E-12	TRIM23
1138.634	1192.842	1133.382	2024.857	1698.02	1606.928	-0.62159	8.21E-12	HSPE1
146.4857	177.3411	162.4332	328.8318	340.0239	320.5394	-1.0263	8.29E-12	WDR7
79.98299	60.43466	62.05314	8.298593	10.49456	4.231543	3.135908	8.30E-12	SCNN1D
665.9258	710.355	672.5466	425.3029	431.3266	387.1862	0.719579	8.33E-12	WDR45
209.3937	201.1186	167.9085	76.76199	51.42336	27.50503	1.89189	8.61E-12	TMEM54
237.2529	287.3123	222.6613	110.9937	80.80814	105.7886	1.327055	8.68E-12	RPP25L
764.7812	820.3262	852.3182	1136.907	1326.513	1277.926	-0.61827	8.90E-12	WDR11
224.6713	165.4523	185.2469	378.6233	402.9912	382.9546	-1.0153	8.93E-12	ZBTB26
1626.62	1635.699	1676.347	2425.264	2191.265	2235.312	-0.4724	8.96E-12	MRPL18
1466.654	1508.885	1386.158	1048.735	798.6363	892.8555	0.67031	9.01E-12	NDUFB7
125.8159	103.0361	96.7299	28.00775	28.33532	22.2156	2.051249	9.02E-12	MXRA8
78.18562	46.56441	53.84023	3.111972	5.247282	4.231543	3.828848	9.11E-12	LINC00173
302.8569	289.2938	284.7144	503.1022	490.0961	545.869	-0.81136	9.12E-12	THG1L
991.2499	983.7971	1004.713	1438.769	1374.788	1444.014	-0.51475	9.12E-12	AC118549.1
17836.21	18109.59	16852.9	24102.23	21547.44	23329.55	-0.38569	9.19E-12	RPL19
332.5136	358.645	289.2772	169.0838	151.1217	129.0621	1.124829	9.23E-12	POLL
753.0983	801.5023	776.5768	1076.742	1243.606	1253.595	-0.61664	9.24E-12	PCYT1A
2227.841	2300.48	2347.069	2989.568	3078.055	3072.1	-0.41075	9.25E-12	FAF2
1267.146	1189.869	1172.622	813.2621	843.7629	799.7616	0.563206	9.29E-12	FGD1
1731.767	1750.624	1815.054	2307.009	2449.431	2536.81	-0.46117	9.38E-12	OSBPL9
779.1601	753.9472	881.5196	1198.109	1280.337	1200.7	-0.60711	9.85E-12	PHF6
232.7595	232.8221	229.9616	100.6204	105.9951	93.09394	1.214425	9.88E-12	ENDOV
840.2707	863.9185	824.9418	533.1846	531.0249	399.8808	0.788258	9.93E-12	AHDC1
2771.545	2947.428	2806.992	2136.888	2133.545	2092.498	0.421952	1.01E-11	QARS
254.3279	248.6738	252.7753	108.919	122.7864	104.7307	1.167709	1.02E-11	FBXL19-AS1
264.2135	207.063	199.8476	84.02326	96.54998	79.34143	1.369193	1.02E-11	HYAL3

3160.676	3305.083	2979.463	2173.194	2286.765	2396.111	0.462085	1.03E-11	TNFAIP1
211.191	204.0908	207.148	73.65001	90.25325	86.74663	1.312721	1.03E-11	RAB6B
684.7982	752.9565	651.558	434.6388	420.832	400.9387	0.733037	1.03E-11	TBC1D22A
348.6899	345.7655	366.8436	558.0804	609.7341	629.442	-0.75962	1.04E-11	CCSAP
408.0031	460.6905	394.22	713.679	694.7401	678.1047	-0.72543	1.04E-11	CRBN
786.3496	830.2336	787.5274	501.0276	462.8102	535.2902	0.681179	1.05E-11	GPKOW
576.9559	562.7359	603.1931	888.9868	905.6808	880.1609	-0.61766	1.05E-11	STX3
79.0843	59.44393	64.79078	9.335917	7.346194	9.520971	2.956922	1.08E-11	SEMA6C
822.297	818.3448	793.9152	537.3339	487.9972	522.5955	0.653226	1.09E-11	CES2
635.3705	639.0223	590.4174	398.3325	354.7162	344.8707	0.763876	1.11E-11	TRIB3
1456.769	1432.599	1505.701	2248.919	2005.511	1988.825	-0.50636	1.12E-11	TIMM17A
79.0843	77.27711	73.0037	7.261269	6.296738	17.98406	2.864859	1.15E-11	DNM1
74.59088	83.2215	86.69189	16.59719	1.049456	10.57886	3.112531	1.17E-11	CALHM3
183.3318	162.4801	176.1214	308.0853	406.1396	369.2021	-1.05263	1.17E-11	RABIF
1508.893	1495.015	1511.177	1017.615	839.5651	616.7473	0.867726	1.20E-11	MBD3
293.8701	371.5246	373.2314	178.4198	139.5777	100.4991	1.310271	1.20E-11	PAQR4
651.5468	649.9203	675.2842	402.4818	372.557	423.1543	0.722414	1.21E-11	SZT2
1185.366	1239.406	1212.774	848.5311	841.664	777.546	0.559505	1.23E-11	CMTR1
1515.183	1474.209	1303.116	2300.785	2116.753	1958.146	-0.57093	1.23E-11	TXNL4A
793.5391	767.8174	733.6872	1126.534	1108.226	1166.848	-0.56764	1.24E-11	TOR1B
2551.368	2273.73	2521.365	3182.51	3520.926	3597.869	-0.48734	1.25E-11	NPEPPS
1123.357	1108.629	1099.618	771.7692	675.8499	757.4461	0.595381	1.25E-11	KIAA0319L
579.652	583.5413	522.889	337.1303	300.1445	225.3296	0.966326	1.26E-11	ZDHHC24
761.1864	750.975	758.3259	1160.766	1150.204	1063.175	-0.57151	1.27E-11	RAP2A
389.1307	418.089	427.9842	239.6219	209.8913	185.13	0.960174	1.27E-11	YIPF2
1299.499	1309.748	1330.492	1855.773	1807.164	1781.479	-0.4667	1.29E-11	CDK2
262.4161	244.7108	241.8247	97.50847	120.6875	66.6468	1.394449	1.30E-11	KCTD11
5817.19	5660.053	5920.6	3904.488	4545.195	4375.415	0.440037	1.30E-11	PLOD2
598.5244	552.8286	579.4668	998.9431	890.9884	854.7716	-0.66489	1.31E-11	SMYD2
217.4818	267.4977	245.4749	429.4522	443.92	514.1324	-0.92674	1.34E-11	TMF1
1163.797	1064.046	1203.648	1588.143	1660.24	1709.543	-0.53032	1.36E-11	PHC3
129.4107	127.8045	100.3801	37.34367	28.33532	28.56291	1.922837	1.38E-11	SMOC1
316.3372	313.0714	283.8019	509.3262	576.1515	517.3061	-0.81177	1.38E-11	TIMM8A
1590.673	1633.717	1459.161	2340.203	2150.336	2139.045	-0.50163	1.39E-11	DAD1
378.3465	335.8582	353.1554	203.3155	152.1712	150.2198	1.077618	1.39E-11	STXBP2
4859.191	4471.174	4993.453	6052.786	6697.63	6532.444	-0.4287	1.40E-11	KHDRBS1
104.2475	88.17516	104.0303	24.89578	25.18695	14.8104	2.191409	1.46E-11	ZNF524
59.31323	61.4254	86.69189	9.335917	10.49456	6.347314	2.985853	1.47E-11	PI16
53.02243	56.47173	55.66532	1.037324	3.148369	2.115771	4.714245	1.47E-11	SRRM3
2200.88	2136.019	2120.757	2857.828	2924.835	2804.455	-0.4111	1.49E-11	CPSF7
366.6636	328.9231	317.5661	559.1177	564.6075	638.9629	-0.79838	1.49E-11	CBWD1
82.67905	87.18443	95.81735	19.70916	14.69239	20.09983	2.285319	1.49E-11	HOXD11
97.9567	137.7118	150.5701	35.26902	36.73097	30.67868	1.910394	1.50E-11	NLRP1
492.4795	466.6349	501.9004	265.555	292.7983	267.6451	0.823133	1.51E-11	CNTNAP1
387.3334	332.886	399.6952	205.3902	163.7152	125.8884	1.177875	1.52E-11	SHROOM1
158.1686	151.582	143.2698	56.0155	43.02771	51.8364	1.585985	1.54E-11	TMEM53
5539.496	5887.921	5971.702	7264.381	7601.212	8026.179	-0.39585	1.57E-11	COPB2
647.0534	555.8008	592.2425	362.0261	331.6282	354.3917	0.776921	1.58E-11	EVA1C
1248.274	1190.86	1283.04	1671.129	1831.301	1773.016	-0.5028	1.60E-11	EBLN3P
264.2135	261.5533	249.1251	116.1803	128.0337	102.6149	1.159532	1.60E-11	PAQR7
845.6628	769.7989	659.7709	470.9452	434.4749	387.1862	0.815561	1.60E-11	INPP5E
1684.136	1820.966	1627.982	1205.371	1201.628	1202.816	0.507525	1.60E-11	MKRN1
81.78036	75.29565	91.25462	14.52254	12.59348	19.04194	2.428905	1.63E-11	CIITA
756.693	766.8267	757.4134	518.6621	442.8706	423.1543	0.719742	1.65E-11	ZNF746

1107.18	1161.138	1021.139	362.0261	196.2483	180.8985	2.153633	1.67E-11	ZDHHC12
1126.053	1190.86	1091.405	735.4628	571.9537	406.2281	0.991575	1.69E-11	NUBP2
270.5043	267.4977	290.1897	126.5535	140.6272	113.1938	1.122606	1.71E-11	PDK3
168.0541	204.0908	204.4104	365.1381	368.3592	392.4756	-0.96661	1.71E-11	THAP6
1139.533	1223.554	1221.899	673.2234	784.9933	836.7876	0.643516	1.71E-11	PARP9
785.4509	776.734	894.2953	490.6543	514.2336	523.6534	0.684805	1.72E-11	GRIPAP1
727.9351	654.874	695.3602	985.4579	1128.166	1087.506	-0.62265	1.73E-11	NCK1
767.4772	826.2706	811.2536	520.7367	407.1891	503.5536	0.748249	1.73E-11	GPR108
398.1176	380.4412	413.3834	604.76	712.5809	681.2784	-0.74514	1.73E-11	BACE1
337.9057	317.0343	339.4672	644.1783	548.8657	541.6375	-0.80243	1.75E-11	PDCL3
1212.326	1235.443	1229.2	1661.793	1746.295	1994.114	-0.55499	1.77E-11	LRRFIP2
390.9281	515.1807	523.8015	781.1051	982.2911	826.2087	-0.8575	1.77E-11	-
255.2266	212.0167	229.9616	112.031	85.00596	87.80451	1.291682	1.81E-11	GDF15
931.0379	1059.093	975.5119	418.0416	559.3602	644.2524	0.87092	1.82E-11	MT-TC
892.3945	876.798	911.6337	1519.68	1286.633	1272.636	-0.60551	1.82E-11	RRP1
915.7603	812.4004	894.2953	1281.095	1280.337	1287.447	-0.55284	1.84E-11	PPP1R2
57.51586	68.36052	88.51698	9.335917	6.296738	12.69463	2.920877	1.86E-11	NOVA2
1144.925	1069	1125.169	1474.038	2002.363	2115.771	-0.74368	1.86E-11	CCDC14
64.70534	49.53661	60.22805	6.223945	5.247282	0	3.922453	1.88E-11	BDKRB2
1107.18	1069	982.8123	714.7163	691.5917	673.8732	0.602648	1.90E-11	STUB1
1105.383	1008.565	974.5994	1574.658	1410.469	1664.054	-0.58981	1.91E-11	GNG5
532.0217	539.949	511.0259	291.4881	327.4304	243.3137	0.876189	1.92E-11	RARA
564.3744	684.5959	563.041	1179.438	980.1922	926.7079	-0.76936	1.93E-11	ABCF2
320.8306	309.1084	303.8779	493.7663	571.9537	555.39	-0.79551	1.96E-11	TDRD3
266.9095	287.3123	279.2391	145.2254	74.5114	87.80451	1.437342	1.96E-11	EME2
355.8794	390.3485	412.4709	617.2079	634.9211	709.8413	-0.75993	1.97E-11	DENND4C
1676.048	1851.678	1879.845	2356.8	2700.251	2793.876	-0.53799	1.98E-11	SPEN
754.8956	754.9379	754.6757	497.9156	471.2059	472.8749	0.651051	1.99E-11	HSPB8
4283.134	4608.886	4531.705	5759.224	5834.977	5711.525	-0.36661	2.01E-11	MORF4L1
567.0704	597.4115	528.3643	360.9888	258.1663	264.4714	0.937259	2.02E-11	MRPL23
245.3411	254.6182	228.1366	105.8071	76.61031	115.3095	1.289917	2.04E-11	CA2
540.1099	526.0788	583.117	906.6213	837.4662	836.7876	-0.64569	2.05E-11	COIL
121.3225	126.8137	132.3192	266.5923	252.919	306.7868	-1.11891	2.06E-11	C18orf21
371.157	390.3485	332.1668	204.3529	166.8636	173.4933	1.004866	2.12E-11	JDP2
230.9621	250.6552	223.5738	443.9747	419.7825	418.9227	-0.864	2.12E-11	ZIC2
1065.841	1101.694	1199.086	1526.941	1775.68	1675.691	-0.56424	2.14E-11	CLCN3
3507.569	3655.802	3819.006	4661.735	5134.99	4840.885	-0.4145	2.14E-11	CLIC4
143.7896	133.7488	102.2052	32.15705	35.68152	38.08388	1.842161	2.15E-11	WNT9A
125.8159	119.8786	130.4941	43.56761	34.63206	31.73657	1.774386	2.16E-11	FOXL1
54.8198	56.47173	62.96569	7.261269	5.247282	4.231543	3.378133	2.18E-11	RASSF4
550.8941	621.1891	457.1857	298.7494	228.7815	152.3355	1.259976	2.19E-11	C19orf25
1988.791	2085.491	2020.377	3108.86	2812.543	2636.251	-0.48989	2.22E-11	WDR5
1575.395	1589.134	1592.393	2204.314	2316.15	2064.993	-0.46931	2.23E-11	DARS2
733.3272	713.3272	781.1396	1106.825	1073.594	1126.648	-0.56958	2.31E-11	UBXN2A
411.5979	442.8573	446.2351	241.6965	248.7212	224.2718	0.863545	2.32E-11	ASB9
3815.818	4081.817	3933.074	5741.589	5137.089	5069.388	-0.43102	2.34E-11	PSMC3
582.3481	572.6432	536.5772	353.7275	308.5402	252.8347	0.885909	2.36E-11	RNPEPL1
116.8291	113.9342	114.9808	40.45564	24.1375	23.27348	1.97505	2.42E-11	GHDC
3075.301	3192.139	3209.425	4442.859	4637.548	3944.856	-0.45892	2.44E-11	UHRF1
365.7649	346.7563	345.855	196.0543	177.3581	172.4354	0.955248	2.45E-11	RAD9A
2012.156	2283.638	2045.929	2993.717	2913.291	2831.96	-0.46306	2.48E-11	SUMO3
1100.889	1061.074	1090.493	1451.216	1592.025	1689.443	-0.54085	2.49E-11	DPP8
182.4331	213.9982	215.3609	395.2205	365.2108	409.4018	-0.93599	2.49E-11	MIR4458HG
600.3218	657.8462	629.6569	940.853	982.2911	937.2867	-0.60003	2.50E-11	NAA30

498.7703	485.4588	515.5886	777.9931	771.3504	783.8933	-0.63723	2.50E-11	UBTD2
338.8044	320.0065	309.3532	162.8599	167.913	155.5092	0.993533	2.51E-11	IFT27
78.18562	93.12882	83.95425	12.44789	13.64293	22.2156	2.402483	2.54E-11	MAP2
188.7239	168.4245	161.5207	73.65001	32.53315	46.54697	1.76304	2.55E-11	ATOH8
328.9188	356.6636	367.7561	556.0057	628.6244	597.7054	-0.75902	2.56E-11	CLCC1
1765.018	1969.576	1984.788	1338.148	1324.414	1376.309	0.501697	2.57E-11	WEE1
97.9567	81.24004	93.07971	18.67183	22.03858	19.04194	2.188966	2.69E-11	DAPK2
1153.013	1137.361	1220.987	1551.837	1859.637	1777.248	-0.56311	2.72E-11	RALGPS2
54.8198	54.49027	56.57787	0	5.247282	1.057886	4.72067	2.78E-11	IGFBP5
872.6234	970.9175	966.3864	1340.223	1465.041	1333.994	-0.55921	2.81E-11	MAPK14
15005.35	15105.69	14847.13	18389.68	18449.44	19878.73	-0.33522	2.84E-11	TPM4
338.8044	566.6988	521.9764	244.8085	176.3087	160.7986	1.293442	2.84E-11	PUF60
69.19877	49.53661	48.36495	0	2.098913	4.231543	4.730925	2.85E-11	SMIM1
287.5793	269.4792	269.2011	499.9902	521.5798	446.4278	-0.82894	2.85E-11	GDAP2
612.9034	630.1057	592.2425	997.9058	893.0874	921.4184	-0.61619	2.86E-11	PPIH
489.7835	457.7183	492.775	725.0896	755.6086	816.6877	-0.67303	2.88E-11	MED4
256.1253	224.8962	260.0757	393.1458	559.3602	527.885	-0.9972	2.90E-11	SLC33A1
878.0155	892.6497	870.5691	592.3121	583.4977	561.7373	0.603981	2.91E-11	EDEM2
801.6273	905.5292	860.5311	1230.266	1250.952	1286.389	-0.55373	2.94E-11	ATP6V1D
349.5886	346.7563	365.0185	195.0169	185.7538	167.1459	0.953784	2.94E-11	SH2D3A
1624.823	1557.431	1538.553	2062.2	2195.463	2223.676	-0.45709	2.95E-11	DYRK2
84.47642	102.0454	71.17861	20.74648	14.69239	12.69463	2.41806	2.96E-11	EFR3B
231.8608	242.7294	185.2469	95.43382	89.20379	63.47314	1.409952	2.97E-11	AC126755.1
200.4068	213.9982	216.2735	366.1754	395.645	416.807	-0.90233	3.05E-11	ENTPD5
141.0936	142.6654	118.631	39.41832	38.82988	46.54697	1.688969	3.06E-11	PYROXD2
486.1887	401.2465	447.1476	234.4353	226.6826	256.0083	0.89701	3.07E-11	MLXIP
354.9807	370.5338	365.931	212.6514	161.6163	172.4354	0.996883	3.09E-11	NAGK
219.2792	219.9425	199.8476	428.4149	405.0901	367.0863	-0.91018	3.11E-11	MAIP1
841.1694	914.4458	897.9455	483.393	533.1238	612.5158	0.703929	3.15E-11	MYH10
391.8268	355.6729	318.4786	124.4789	166.8636	191.4773	1.143274	3.16E-11	ELF3
450.2413	348.7377	388.7447	217.8381	195.1989	165.0302	1.039369	3.17E-11	FADD
291.174	287.3123	271.9388	147.3	116.4897	82.51508	1.295305	3.17E-11	KRT86
89.86853	75.29565	75.74134	14.52254	12.59348	17.98406	2.418379	3.18E-11	RGS9
86.27379	74.30491	93.07971	10.37324	15.74185	21.15771	2.426343	3.19E-11	VIPR1
576.9559	523.1066	574.9041	337.1303	336.8755	278.2239	0.814978	3.19E-11	CDKN2A
2570.24	2184.564	2537.791	1470.926	1651.844	1769.843	0.576253	3.20E-11	KLF6
184.2305	180.3133	161.5207	73.65001	65.06629	49.72063	1.480315	3.39E-11	LINC00346
81.78036	98.08249	46.53986	12.44789	9.445107	6.347314	2.999904	3.51E-11	SMURF2P1
2239.524	2447.108	2271.328	3074.629	3115.836	3087.968	-0.41556	3.57E-11	PLK1
187.8252	187.2484	183.4218	365.1381	342.1228	351.218	-0.92248	3.57E-11	CCDC112
481.6953	443.848	425.2465	822.598	805.9825	659.0628	-0.75998	3.63E-11	CLN6
841.1694	938.2234	647.9078	493.7663	393.5461	350.1602	0.971216	3.64E-11	TARBP2
75.48956	62.41613	52.01513	10.37324	2.098913	6.347314	3.333041	3.65E-11	ANO9
1589.774	1660.467	1662.659	2138.962	2251.084	2309.364	-0.44754	3.68E-11	SIN3A
190.5213	130.7766	170.6461	482.3557	366.2603	309.9605	-1.23405	3.70E-11	AGK
250.7332	230.8406	240.9122	117.2176	86.05542	107.9043	1.215379	3.77E-11	SMIM29
833.9799	874.8165	882.4322	1318.439	1281.386	1188.006	-0.54798	3.77E-11	AFG3L2
12.58159	10.89805	18.25092	68.46339	82.90705	79.34143	-2.46264	3.78E-11	MBNL3
134.8028	137.7118	156.9579	51.86621	48.27499	30.67868	1.714241	3.80E-11	SSR4P1
281.2885	346.7563	323.0414	517.6247	558.3108	619.921	-0.83534	3.83E-11	ZRANB1
453.8361	421.0612	410.6458	717.8283	659.0586	697.1467	-0.6898	3.84E-11	GTF2B
840.2707	754.9379	849.5805	1308.066	1205.825	1160.501	-0.58726	3.86E-11	CHERP
784.5522	881.7516	704.4857	508.2888	447.0684	377.6652	0.829879	3.87E-11	ABHD14B
133.0054	127.8045	88.51698	32.15705	28.33532	29.6208	1.954097	3.93E-11	TMEM187

1004.73	987.76	1106.919	1467.814	1481.832	1493.735	-0.51934	3.94E-11	CTDSPL2
63.80665	73.31418	61.1406	2.074648	0	1.057886	5.978191	3.95E-11	SSC5D
200.4068	207.063	219.0111	466.7959	376.7548	371.3179	-0.95566	4.01E-11	EIF4A1P10
293.8701	380.4412	340.3797	622.3945	601.3385	559.6215	-0.81506	4.01E-11	SUMF1
351.3859	345.7655	348.5927	177.3824	147.9733	196.7667	1.002268	4.05E-11	HABP4
478.9993	461.6812	451.7104	281.1148	248.7212	257.0662	0.823239	4.05E-11	PIAS3
347.7912	316.0436	371.4063	569.491	585.5966	588.1844	-0.75086	4.13E-11	AASDH
344.1965	327.9324	365.0185	540.4459	589.7945	653.7733	-0.78183	4.21E-11	SNRNP27
115.9304	88.17516	145.0948	28.00775	26.23641	32.79446	2.006112	4.22E-11	POSTN
175.2436	177.3411	175.2089	53.94086	53.52227	80.39931	1.490973	4.24E-11	PRDM1
654.2429	634.0686	700.8355	979.234	1048.407	976.4285	-0.59438	4.27E-11	IQCB1
514.9467	493.3846	493.6875	731.3135	801.7846	856.8874	-0.66977	4.29E-11	CEP120
173.4463	177.3411	157.8705	63.27677	64.01684	64.53103	1.406611	4.38E-11	PPP2R2B
616.4981	636.0501	761.0635	1015.54	1049.456	1147.806	-0.6737	4.44E-11	TMEM161B
539.2112	565.7081	636.0447	851.6431	948.7085	963.7339	-0.66672	4.48E-11	CCDC43
384.6373	384.4041	337.6421	675.298	667.4542	561.7373	-0.78362	4.51E-11	IMP3
1131.445	1141.323	1301.291	1799.757	1722.158	1655.591	-0.53453	4.53E-11	WWC2
3058.226	2901.855	3040.604	3997.847	3829.466	3888.788	-0.38022	4.54E-11	PABPC4
90.76721	48.54588	41.06458	6.223945	3.148369	2.115771	3.97049	4.55E-11	SCX
89.86853	83.2215	76.65388	18.67183	15.74185	16.92617	2.282102	4.57E-11	LZTS3
117.7278	104.0269	91.25462	204.3529	263.4135	304.6711	-1.30261	4.70E-11	CCDC150
700.0758	746.0213	698.0979	996.8685	1113.473	1237.726	-0.6431	4.83E-11	LGALS8
1531.36	1400.895	1389.808	1039.399	909.8787	780.7196	0.66276	4.88E-11	CORO1B
579.652	539.949	496.4251	937.741	860.5542	798.7037	-0.68442	4.88E-11	CWF19L1
648.8508	583.5413	563.9536	387.9592	300.1445	320.5394	0.832621	4.89E-11	RPS27AP16
831.2839	867.8814	788.4399	575.7149	464.9092	480.2801	0.709341	4.93E-11	MTMR14
789.9444	824.2892	840.4551	513.4754	490.0961	561.7373	0.649102	4.97E-11	RPS6KC1
220.1779	262.544	243.6498	93.35917	107.0445	116.3674	1.196906	4.99E-11	MYOM3
138.3975	123.8415	123.1937	255.1817	295.9467	267.6451	-1.08625	4.99E-11	USP49
116.8291	156.5357	113.1557	34.2317	37.78043	40.19966	1.78308	4.99E-11	ARSD
269.6056	281.3679	226.3115	131.7402	102.8467	99.44125	1.217467	5.12E-11	TMEM200B
2245.815	2408.47	2385.396	3221.929	3112.688	3031.9	-0.41223	5.16E-11	ELAVL1
445.7479	428.987	454.448	673.2234	696.839	761.6777	-0.68104	5.34E-11	EIF3FP3
88.07116	121.8601	106.7679	260.3684	229.8309	240.14	-1.20798	5.43E-11	TUBD1
1428.91	1457.367	1429.96	1982.326	2073.726	1885.152	-0.46111	5.47E-11	SAR1B
2044.509	2098.371	2073.305	2664.886	2786.307	2763.197	-0.4022	5.47E-11	POLE
463.7216	483.4773	522.889	732.3508	780.7955	836.7876	-0.67657	5.48E-11	BRAF
2156.845	2234.101	2079.693	1644.159	1523.811	1405.93	0.500213	5.49E-11	PLOD3
274.099	243.7201	246.3875	103.7324	107.0445	131.1778	1.160971	5.50E-11	TPD52L1
1585.281	1599.042	1547.678	2039.379	2283.617	2201.46	-0.46344	5.52E-11	KIF1B
5386.72	5691.756	5404.099	4248.88	3806.378	3210.683	0.548849	5.54E-11	HNRNPA0
334.3109	303.164	306.6155	157.6733	168.9625	140.6988	1.014684	5.55E-11	CACNB3
403.5097	423.0426	441.6724	199.1662	211.9902	258.1241	0.922432	5.59E-11	CAVIN2
1372.292	1385.044	1188.135	938.7783	784.9933	820.9193	0.632379	5.64E-11	MAZ
570.6652	619.2076	526.5392	362.0261	240.3255	218.9823	1.062561	5.66E-11	C1orf122
925.6458	844.1038	926.2344	543.5578	573.0032	618.8631	0.636106	5.67E-11	PEG10
386.4347	379.4504	377.7941	195.0169	199.3967	221.0981	0.894081	5.71E-11	EVL
275.8964	278.3957	247.3	95.43382	141.6766	111.078	1.203042	5.90E-11	USP43
433.1663	450.7831	465.3986	244.8085	273.9081	210.5192	0.887577	5.92E-11	DUSP22
459.2282	493.3846	439.8473	289.4134	218.2869	231.677	0.912509	5.93E-11	GFER
299.2622	289.2938	334.9045	157.6733	119.638	156.5671	1.090073	5.94E-11	SLC29A2
195.9134	209.0445	173.3838	81.94861	78.70923	67.70468	1.339644	5.94E-11	FGFBP1
1542.144	1571.301	1502.051	2209.5	2001.313	2132.698	-0.45897	5.99E-11	UFD1
277.6938	276.4143	281.9768	130.7028	142.7261	133.2936	1.039719	6.15E-11	MAST4

328.9188	320.9972	300.2277	496.8783	554.113	582.895	-0.78201	6.22E-11	ZUFSP
144.6883	129.7859	149.6576	46.67959	48.27499	48.66274	1.563052	6.25E-11	-
352.2846	373.506	388.7447	589.2001	613.932	618.8631	-0.70919	6.31E-11	KATNBL1
426.8755	468.6163	479.9993	715.7537	715.7292	750.0409	-0.66571	6.33E-11	RNF115
130.3094	130.7766	112.2432	30.0824	40.9288	40.19966	1.747471	6.44E-11	SCN1B
6457.952	5918.634	6421.588	7752.961	8430.283	8731.788	-0.40627	6.44E-11	MAT2A
337.007	305.1455	327.6041	176.3451	132.2315	162.9144	1.040662	6.54E-11	SLC26A11
948.113	943.177	967.299	638.9917	660.108	619.921	0.574971	6.60E-11	CHMP7
5328.305	5862.162	5551.019	7176.208	7332.551	7045.519	-0.36473	6.62E-11	ADAR
599.4231	626.1427	559.3908	391.0712	314.8369	294.0922	0.835242	6.62E-11	RTL8A
252.5306	243.7201	276.5015	491.6916	485.8983	417.8648	-0.85224	6.80E-11	C16orf70
500.5677	458.709	478.1742	789.4037	702.0863	797.6458	-0.67079	6.88E-11	OSBPL3
819.601	781.6877	776.5768	1113.049	1252.001	1145.69	-0.5619	6.95E-11	BROX
282.1872	276.4143	262.8133	467.8332	452.3157	560.6794	-0.85011	6.99E-11	MEIS2
5011.069	5279.612	5315.582	3958.429	4093.929	4102.481	0.360543	7.05E-11	GLG1
3947.924	3969.864	4078.169	5036.209	5083.567	5089.488	-0.34237	7.05E-11	EIF2AK1
378.3465	402.2373	426.1591	660.7755	658.0091	631.5577	-0.69277	7.08E-11	FBXL4
602.1191	675.6793	591.3299	409.743	312.738	289.8607	0.883848	7.10E-11	FAM160A2
460.1269	491.4032	463.5735	281.1148	273.9081	259.182	0.796949	7.15E-11	GRK5
443.0518	455.7368	476.3491	742.7241	706.2841	709.8413	-0.65074	7.21E-11	BPNT1
35.04873	31.70343	29.20148	147.3	108.094	97.32548	-1.87805	7.37E-11	SETP14
149.1818	117.8971	137.7945	288.3761	263.4135	306.7868	-1.08257	7.38E-11	UMAD1
6688.915	6117.771	5875.885	9430.314	8067.171	8254.682	-0.46297	7.43E-11	RPS23
104.2475	102.0454	80.30407	21.78381	22.03858	24.33137	2.072043	7.49E-11	CYP11A1
1553.827	1627.773	1571.405	2057.014	2225.897	2189.823	-0.4457	7.60E-11	RALGAPB
62.90797	54.49027	41.06458	5.186621	1.049456	3.173657	4.07112	7.61E-11	PERM1
259.72	253.6274	246.3875	422.1909	478.5521	443.2541	-0.82286	7.68E-11	USP6NL
2211.664	2322.276	2142.659	1692.913	1560.542	1404.872	0.519009	7.71E-11	PCBP1
3198.421	3289.231	3253.227	4747.833	4245.051	4121.523	-0.42914	7.75E-11	FKBP1A
309.1477	355.6729	334.9045	179.4571	173.1603	151.2777	0.987543	7.80E-11	NPHP4
3169.663	3347.684	3295.204	4379.583	4217.765	4129.986	-0.3754	7.82E-11	NCAPD3
17.97371	8.91659	11.8631	73.65001	77.65977	69.82045	-2.50456	7.86E-11	RIMS2
536.5151	556.7915	542.0525	343.3543	313.7874	322.6551	0.738752	7.90E-11	MXI1
533.8191	519.1437	484.562	324.6825	277.0565	278.2239	0.804818	7.91E-11	C1orf35
2449.816	2541.228	2480.301	3098.487	3599.635	3676.153	-0.4736	7.93E-11	DROSHA
3883.219	4085.78	3871.934	5317.324	5064.676	4948.789	-0.37283	7.97E-11	BANF1
512.2506	559.7637	557.5657	768.6572	930.8678	922.4763	-0.68637	7.98E-11	FCHO2
266.0108	235.7943	277.4141	569.491	485.8983	421.0385	-0.92132	8.05E-11	MARS2
4585.991	4487.026	4857.484	3686.65	3405.486	3518.528	0.392802	8.12E-11	PLK2
174.3449	190.2206	180.6842	349.5782	328.4798	356.5075	-0.92473	8.13E-11	CDC37L1
1576.294	1616.875	1657.184	1196.035	1113.473	1168.964	0.479576	8.19E-11	C19orf53
1186.265	1218.601	1193.61	1711.585	1615.113	1643.954	-0.46623	8.32E-11	CMAS
912.1656	777.7248	914.3713	1213.669	1433.557	1355.152	-0.61919	8.39E-11	FGFR1OP2
54.8198	68.36052	51.10259	1.037324	1.049456	9.520971	3.91416	8.46E-11	GIPR
1422.619	1433.589	1575.055	1952.244	2214.353	2098.845	-0.49952	8.47E-11	PPFIBP1
293.8701	253.6274	223.5738	99.58312	123.8358	113.1938	1.196263	8.50E-11	ERO1B
1113.471	1149.249	1122.432	1601.628	1632.954	1502.198	-0.4849	8.56E-11	SENP2
904.9761	1016.491	1082.28	692.9325	575.1021	512.0167	0.75446	8.79E-11	RRAGA
434.065	398.2743	446.2351	251.0324	229.8309	228.5033	0.850312	8.83E-11	THBD
336.1083	288.3031	338.5546	549.7818	527.8765	563.8531	-0.76824	8.84E-11	AFG3L1P
2112.809	1982.455	2146.309	2784.178	2731.735	2785.413	-0.41113	8.85E-11	ANKLE2
230.0634	185.2669	263.7259	411.8177	464.9092	441.1383	-0.9547	8.89E-11	C16orf87
3804.135	3768.745	3780.679	5165.874	4853.736	4704.418	-0.37505	8.94E-11	PCBP2
239.0503	267.4977	274.6764	494.8036	423.9804	481.338	-0.84226	9.03E-11	MRPL50

419.686	479.5144	432.5469	268.667	220.3858	236.9664	0.874398	9.16E-11	PROCR
1318.371	1333.526	1373.382	1848.512	1794.57	1840.721	-0.44607	9.18E-11	LONP2
1146.722	1112.592	1049.428	801.8516	672.7015	658.0049	0.633485	9.27E-11	GIT1
184.2305	204.0908	192.5473	363.0634	368.3592	350.1602	-0.89771	9.36E-11	BEND3
227.3674	193.1928	205.3229	101.6578	68.21466	53.95217	1.482994	9.38E-11	MYPOP
893.2932	777.7248	782.9647	1126.534	1334.908	1327.647	-0.62619	9.40E-11	ZFAND1
150.9791	158.5171	131.4067	319.4958	277.0565	304.6711	-1.03243	9.41E-11	-
1067.638	1104.666	1095.968	1475.075	1546.899	1525.471	-0.47665	9.51E-11	MRPL16
8950.905	9352.512	10049.87	7459.398	6147.715	6896.357	0.46763	9.52E-11	RPL41
433.1663	425.0241	418.8587	659.7382	652.7618	728.8832	-0.67659	9.82E-11	FGD6
3147.196	3538.895	3191.174	5512.34	4794.966	4229.427	-0.55775	9.88E-11	TUBBP1
4744.16	4755.514	4596.495	6296.558	6079.501	5762.303	-0.36381	9.93E-11	SDC4
1206.036	1130.425	1130.645	832.9713	774.4988	707.7255	0.582601	9.94E-11	ANAPC2
68.30008	85.20297	114.9808	14.52254	12.59348	23.27348	2.414777	1.00E-10	MAP1A
97.9567	83.2215	88.51698	19.70916	25.18695	15.86829	2.150315	1.03E-10	TAGLN
161.7634	150.5913	157.8705	61.20212	53.52227	27.50503	1.724174	1.03E-10	AJM1
458.3295	464.6534	448.0602	713.679	782.8944	681.2784	-0.66782	1.03E-10	SLC7A6
474.5058	414.1261	414.296	690.8579	787.0923	671.7574	-0.72181	1.04E-10	C1orf52
1502.602	1538.607	1521.215	1145.206	867.9004	862.1768	0.665846	1.04E-10	MISP
203.1029	200.1279	314.8284	504.1395	450.2168	667.5259	-1.17453	1.05E-10	-
644.3573	726.2067	688.0598	1078.817	1034.764	979.6021	-0.5881	1.05E-10	BTBD10
158.1686	133.7488	122.2812	276.9655	278.1059	323.713	-1.08434	1.05E-10	ASTE1
2749.977	3080.186	3119.083	4014.444	4068.742	3904.656	-0.42193	1.06E-10	UGGT1
7422.242	7436.436	7383.411	11428.2	9749.449	9323.146	-0.45559	1.06E-10	RPS13
176.1423	125.823	141.4447	293.5627	305.3918	345.9286	-1.08927	1.06E-10	MIR222HG
631.7758	701.4384	726.3868	988.5699	1043.16	1082.217	-0.59662	1.08E-10	CDCA7L
947.2143	890.6682	863.2687	610.9839	410.3374	352.2759	0.975299	1.08E-10	PAXX
115.9304	121.8601	96.7299	36.30634	30.43423	20.09983	1.943912	1.08E-10	FAM131A
1013.717	969.9268	1039.39	1326.738	1608.817	1588.944	-0.58141	1.10E-10	ATP11B
3262.228	3411.091	3481.364	4296.597	4494.822	4334.158	-0.37029	1.10E-10	ADAM10
389.1307	501.3105	497.3377	812.2248	773.4493	739.4621	-0.74551	1.10E-10	RPIA
513.1493	577.5969	512.851	826.7473	882.5928	794.4721	-0.64366	1.13E-10	VPS54
239.0503	275.4235	223.5738	427.3775	487.9972	432.6752	-0.87043	1.15E-10	CD58
516.744	538.9583	543.8775	824.6727	771.3504	906.608	-0.64583	1.16E-10	CLEC2B
719.8469	732.1511	659.7709	462.6466	441.8211	414.6912	0.678435	1.18E-10	TUFT1
503.2638	444.8387	400.6078	270.7416	204.644	221.0981	0.953307	1.19E-10	FCHO1
13471.29	14114.96	12500.06	10719.71	9115.578	9798.137	0.435821	1.20E-10	RPL12
97.05801	64.39759	65.70333	15.55986	11.54402	11.63674	2.552195	1.22E-10	RPS10P3
299.2622	269.4792	281.9768	474.0571	491.1456	487.6853	-0.77142	1.25E-10	ZBTB39
11.68291	8.91659	17.33838	69.50072	87.10488	63.47314	-2.53059	1.25E-10	TDGP1
128.512	108.9805	112.2432	242.7338	281.2543	238.0243	-1.12243	1.26E-10	AC135050.6
328.9188	348.7377	331.2543	163.8972	189.9516	171.3775	0.941556	1.27E-10	CABLES2
1109.876	1063.056	1088.668	789.4037	685.295	720.4201	0.571326	1.29E-10	SLC25A23
1658.074	1706.041	1775.815	1270.722	1249.903	1185.89	0.47161	1.32E-10	KANK2
404.4084	397.2836	418.8587	208.5022	248.7212	207.3456	0.877236	1.34E-10	LPIN2
661.4324	632.0871	686.2348	936.7037	985.4395	1084.333	-0.60227	1.38E-10	EPM2AIP1
655.1416	663.7906	675.2842	965.7488	1088.286	951.0392	-0.59159	1.40E-10	TFCP2
650.6481	650.911	656.1207	448.124	348.4195	342.755	0.780675	1.41E-10	SMUG1
423.2808	448.8017	415.2085	660.7755	670.6026	701.3782	-0.65957	1.41E-10	RAB27A
1704.806	1899.234	1826.005	2410.741	2570.119	2416.211	-0.44633	1.41E-10	SH2B3
569.7665	564.7173	564.8661	797.7023	1056.803	917.1869	-0.70566	1.42E-10	SLC1A3
438.5584	459.6997	444.41	708.4924	696.839	685.5099	-0.63936	1.45E-10	RNASEH2B
210.2924	198.1464	209.8856	102.6951	76.61031	64.53103	1.341914	1.46E-10	PEX6
168.9528	164.4615	155.1329	348.5409	283.3532	340.6392	-0.99333	1.47E-10	ZNF92

575.1586	571.6525	522.889	810.1502	907.7797	854.7716	-0.62384	1.48E-10	RNF8
68.30008	102.0454	61.1406	12.44789	12.59348	14.8104	2.536545	1.50E-10	AC126564.1
120.4238	90.15663	109.5055	25.9331	28.33532	6.347314	2.399696	1.51E-10	SRXN1
83.57773	54.49027	85.77934	6.223945	14.69239	13.75251	2.694257	1.51E-10	GPS2
212.0897	254.6182	257.338	458.4973	431.3266	419.9806	-0.85616	1.52E-10	PPARGC1B
541.0085	593.4486	615.0562	860.979	895.1863	907.6659	-0.60679	1.53E-10	METTL3
813.3102	944.1678	779.3145	562.2297	323.2326	356.5075	1.029739	1.55E-10	GAS6
1079.321	1080.889	1127.907	1621.338	1560.542	1454.593	-0.49576	1.57E-10	ARHGAP1
3290.985	3301.12	3387.372	2443.936	2654.075	2510.363	0.391425	1.58E-10	LIPA
3998.251	3934.197	3672.999	5601.55	5148.633	4850.406	-0.42688	1.59E-10	EIF3I
77.28693	77.27711	101.2926	18.67183	16.7913	20.09983	2.203823	1.64E-10	-
402.611	376.4782	414.296	224.062	213.0396	225.3296	0.849669	1.66E-10	BCKDHB
1843.204	1990.381	1914.522	2537.295	2740.131	2499.784	-0.43643	1.66E-10	ARFGEF2
972.3775	1016.491	1041.215	1416.985	1466.091	1379.483	-0.49249	1.67E-10	CDK13
184.2305	195.1742	166.996	351.6529	352.6173	330.0603	-0.92161	1.68E-10	SOCS2
2416.565	2408.47	2328.818	1779.011	1842.845	1762.438	0.409918	1.68E-10	OAS3
2780.532	2923.651	2969.425	3602.627	3957.5	3921.582	-0.40467	1.70E-10	HLTF
817.8036	858.9648	821.2916	499.9902	562.5086	561.7373	0.620956	1.70E-10	B4GALT4
1348.028	1427.645	1356.956	1027.988	914.0765	839.9612	0.570543	1.71E-10	AGAP3
1150.317	1066.028	1144.333	772.8065	743.0151	792.3564	0.54238	1.73E-10	CAT
791.7417	748.9935	810.341	513.4754	525.7776	486.6274	0.623958	1.73E-10	TMEM94
80.88168	71.33272	61.1406	163.8972	202.5451	180.8985	-1.35876	1.76E-10	KCTD6
2508.231	2359.924	2613.532	3162.801	3471.602	3441.302	-0.42916	1.77E-10	SP1
1320.169	1426.654	1211.861	1932.535	1815.559	1862.937	-0.50377	1.78E-10	POLR1D
82.67905	87.18443	81.21661	14.52254	23.08804	16.92617	2.202988	1.78E-10	TCP11L2
265.1122	253.6274	261.9008	452.2733	432.376	461.2382	-0.78546	1.81E-10	MAP3K13
1142.229	1287.952	1175.36	1981.289	1720.059	1632.318	-0.56545	1.81E-10	TRUB2
432.2676	481.4958	432.5469	279.0402	237.1771	213.6929	0.882325	1.82E-10	ATG4D
54.8198	50.52734	56.57787	8.298593	4.197825	1.057886	3.57361	1.85E-10	AC061992.1
1881.847	1972.548	1852.469	1368.231	1381.085	1438.725	0.446245	1.86E-10	ZMYND8
83.57773	90.15663	107.6805	15.55986	29.38478	17.98406	2.161464	1.88E-10	AC105999.2
3160.676	3180.25	3174.748	2523.81	2133.545	1998.346	0.515594	1.88E-10	INTS1
112.3357	124.8323	104.0303	31.11972	39.87934	21.15771	1.887226	1.88E-10	CCDC24
22386.25	21862.49	21169.25	18112.72	17262.51	17519.64	0.306555	1.89E-10	GNAS
56.61717	51.51807	51.10259	4.149297	7.346194	4.231543	3.340431	1.89E-10	AC018521.5
480.7966	476.5422	406.0831	711.6044	765.0537	713.0149	-0.68384	1.90E-10	ZNF142
160.8647	194.1835	164.2583	59.12748	57.7201	77.22565	1.419512	1.90E-10	PIGV
181.5344	155.545	167.9085	314.3092	368.3592	311.0184	-0.97543	1.91E-10	ZBTB14
320.8306	441.8666	309.3532	85.06058	147.9733	174.5511	1.395026	1.92E-10	ATP5ME
404.4084	361.6172	355.893	202.2782	214.0891	171.3775	0.932835	1.95E-10	UBXN11
169.8515	198.1464	166.0834	313.2719	351.5679	375.5494	-0.96326	1.95E-10	BMP2K
900.4827	910.4829	862.3562	1245.826	1248.853	1290.621	-0.50192	1.96E-10	PKP4
212.9884	207.063	202.5853	100.6204	58.76956	83.57297	1.357268	1.98E-10	AIF1L
64.70534	62.41613	67.52842	10.37324	7.346194	12.69463	2.678833	2.00E-10	STYK1
169.8515	155.545	135.9694	37.34367	67.16521	42.31543	1.652169	2.01E-10	VASH1
1624.823	1645.606	1850.644	2257.217	2512.398	2390.822	-0.48343	2.02E-10	RB1CC1
639.8639	638.0315	615.0562	370.3247	365.2108	426.3279	0.704316	2.07E-10	PACS2
230.9621	213.0074	159.6956	417.0043	419.7825	366.0284	-0.995	2.07E-10	YAE1D1
948.113	997.6673	981.8997	1303.916	1540.602	1414.393	-0.54086	2.13E-10	WDFY1
308.2491	321.988	292.9273	158.7106	142.7261	167.1459	0.977982	2.19E-10	CNNM2
747.7062	713.3272	703.5731	1086.078	1140.759	992.2968	-0.57248	2.21E-10	TSPYL1
55.71849	54.49027	46.53986	7.261269	2.098913	4.231543	3.524913	2.21E-10	EDN2
555.3875	534.9954	570.3414	792.5156	847.9607	928.8236	-0.62912	2.23E-10	UBE2Q2
553.5901	582.5505	572.1665	802.8889	883.6422	949.9813	-0.62618	2.26E-10	ZCCHC6

276.7951	268.4884	246.3875	448.124	437.6233	502.4957	-0.81028	2.30E-10	NBPF9
134.8028	139.6932	121.3686	45.64226	32.53315	48.66274	1.641685	2.30E-10	AC027309.2
79.98299	70.34198	72.09115	19.70916	9.445107	9.520971	2.522024	2.31E-10	IL17RE
1192.555	1349.377	1314.979	870.315	926.67	847.3664	0.544157	2.34E-10	GAK
70.09745	59.44393	41.06458	7.261269	6.296738	4.231543	3.260008	2.34E-10	TMEM121
1724.577	1826.91	1755.739	2518.623	2348.683	2295.612	-0.43288	2.37E-10	SDCCAG3
314.5398	327.9324	328.5166	585.0508	496.3929	550.1006	-0.74899	2.37E-10	DNAAF2
488.8848	462.6719	532.927	782.1424	761.9053	760.6198	-0.63399	2.42E-10	SNRK
1519.677	1606.968	1625.245	1183.587	1129.215	1100.201	0.477252	2.46E-10	SETD1A
1672.453	1538.607	1631.633	2214.687	2172.375	2134.813	-0.42914	2.55E-10	IFRD1
345.0951	387.3763	334.9045	211.6141	160.5668	163.9723	0.99233	2.56E-10	CAPN10
736.9219	681.6237	813.0787	1023.839	1226.814	1318.126	-0.67672	2.56E-10	FNBP1L
4504.211	4812.977	4548.13	3647.232	3329.925	2912.359	0.487344	2.56E-10	SLC2A1
321.7293	330.9045	284.7144	165.9719	129.0831	163.9723	1.029567	2.57E-10	ZBTB47
685.6969	748.0028	722.7366	470.9452	436.5738	471.817	0.644302	2.58E-10	MAD1L1
519.4401	567.6895	509.2008	337.1303	301.194	309.9605	0.750699	2.58E-10	ARL8A
2574.733	2714.606	2517.715	1977.14	1989.769	1811.1	0.433951	2.61E-10	KIAA0930
2282.661	2453.053	2222.963	1784.198	1456.645	1326.589	0.607132	2.63E-10	RABL6
268.7069	256.5996	286.5395	432.5642	472.2554	532.1165	-0.823	2.65E-10	SPATA13
1024.501	1062.065	987.375	1624.45	1399.975	1426.03	-0.53421	2.69E-10	KANSL2
207.5963	218.9518	216.2735	474.0571	373.6065	369.2021	-0.9212	2.70E-10	C1orf112
1110.775	1052.158	1043.04	730.2762	762.9548	707.7255	0.542759	2.71E-10	LSM14B
1832.419	1948.77	1928.21	2516.548	2483.014	2539.984	-0.40134	2.74E-10	DERL1
395.4215	397.2836	375.969	193.9796	234.0288	211.5771	0.869687	2.78E-10	RECQL5
965.188	1089.805	1023.877	718.8656	670.6026	681.2784	0.57171	2.80E-10	ADCY6
1030.792	1029.371	959.0861	690.8579	524.7282	400.9387	0.900918	2.82E-10	ISOC2
1555.624	1628.764	1600.606	2467.794	2230.095	2050.182	-0.49618	2.92E-10	MRPL11
229.1647	216.9703	250.9502	400.4071	453.3651	400.9387	-0.84719	2.95E-10	CNTLN
32.35267	29.72197	40.15203	99.58312	134.3304	113.1938	-1.76152	2.99E-10	SNORD17
474.5058	457.7183	478.1742	242.7338	278.1059	291.9764	0.795586	3.07E-10	NAV1
2324	2508.534	2368.97	1874.445	1719.009	1718.006	0.438885	3.10E-10	ECHS1
47.63032	54.49027	40.15203	2.074648	2.098913	2.115771	4.498769	3.12E-10	GPT
70.09745	105.0176	74.82879	14.52254	22.03858	12.69463	2.341357	3.14E-10	CYSRT1
1765.917	1880.41	1919.085	2395.181	2494.558	2647.888	-0.43769	3.19E-10	OSGIN2
558.0836	495.3661	602.2805	784.217	983.3406	947.8656	-0.71285	3.19E-10	SLC35A3
518.5414	507.2549	511.0259	743.7614	777.6472	829.3824	-0.61297	3.22E-10	NT5DC1
566.1717	541.9305	488.2122	293.5627	317.9853	330.0603	0.761661	3.23E-10	MOB3A
1187.163	1184.916	1179.01	1675.278	1769.383	1539.224	-0.48906	3.32E-10	PDHX
205.7989	281.3679	253.6878	116.1803	110.1929	100.4991	1.179095	3.39E-10	ZNF503
1480.135	1600.032	1492.926	1151.43	1009.577	926.7079	0.566217	3.42E-10	GTPBP6
341.5004	400.2558	307.5281	187.7557	179.457	135.4094	1.060617	3.43E-10	NARFL
11874.33	12032.44	11263.56	9455.21	8411.393	9319.973	0.371428	3.44E-10	RPS11
473.6071	421.0612	480.9119	692.9325	738.8173	728.8832	-0.65048	3.44E-10	RAP2C
350.4873	322.9787	313.0034	189.8303	161.6163	156.5671	0.957306	3.45E-10	BCAR1
236.3542	218.9518	208.9731	414.9297	389.3483	384.0125	-0.83882	3.47E-10	INTS9
327.1214	343.7841	350.4177	526.9607	570.9043	570.2004	-0.70785	3.48E-10	VTI1A
781.8562	741.0677	810.341	438.7881	514.2336	522.5955	0.66155	3.51E-10	PML
264.2135	230.8406	222.6613	116.1803	95.50053	114.2517	1.139172	3.66E-10	LMBR1L
38.64347	36.65709	43.80222	114.1057	131.182	119.5411	-1.61374	3.74E-10	COX16
1577.193	1571.301	1373.382	1073.63	1067.297	993.3546	0.52855	3.81E-10	AMFR
61.1106	60.43466	80.30407	12.44789	8.395651	12.69463	2.589904	3.82E-10	-
170.7502	185.2669	171.5587	66.38874	61.91792	81.4572	1.33053	3.83E-10	GEM
2329.392	2628.412	2556.042	1788.347	1879.576	1867.168	0.440711	3.85E-10	OS9
557.1849	568.6803	505.5506	810.1502	820.6749	847.3664	-0.60353	3.87E-10	CINP

44.93426	48.54588	57.49041	5.186621	5.247282	0	3.850228	3.92E-10	LMO1
1312.979	1425.664	1351.481	1836.064	1830.252	1890.442	-0.44247	3.98E-10	RALBP1
327.1214	311.0899	311.1783	468.8705	578.2504	610.4	-0.80353	4.03E-10	NBPF8
63.80665	70.34198	65.70333	8.298593	3.148369	15.86829	2.872707	4.05E-10	BMF
417.8887	419.0797	368.6687	232.3606	215.1386	153.3934	1.003954	4.07E-10	TESK1
155.4726	129.7859	111.3306	44.60494	45.12662	23.27348	1.810558	4.07E-10	PAPLN
312.7425	279.3865	306.6155	527.998	518.4314	471.817	-0.75568	4.09E-10	NEU3
210.2924	242.7294	255.5129	110.9937	108.094	106.8465	1.119819	4.17E-10	HMGCL
964.2893	936.2419	945.3979	1443.955	1320.216	1283.215	-0.50806	4.24E-10	TRAF3
410.6992	355.6729	364.1059	199.1662	203.5945	214.7508	0.87307	4.27E-10	MXD1
194.116	252.6367	217.186	107.8817	75.56086	49.72063	1.507944	4.31E-10	LSS
5833.366	6513.073	6480.903	8349.422	8230.886	7831.528	-0.37488	4.38E-10	DDB1
2783.228	2825.568	2577.031	2152.448	1878.527	1797.348	0.489841	4.49E-10	TCF3
292.9714	384.4041	351.3303	574.6776	578.2504	596.6475	-0.76755	4.51E-10	FP565260.1
454.7348	390.3485	409.7333	708.4924	674.8004	632.6156	-0.68324	4.56E-10	CXorf56
667.7232	716.2994	667.9838	1053.921	978.0933	978.5442	-0.55355	4.65E-10	NEDD4L
52.12375	87.18443	63.87824	14.52254	8.395651	3.173657	2.95602	4.77E-10	PTGS1
53.02243	66.37906	60.22805	3.111972	10.49456	9.520971	2.959146	4.77E-10	-
181.5344	173.3781	156.9579	74.68734	67.16521	61.35737	1.332391	4.82E-10	ADORA1
530.2243	598.4022	509.2008	353.7275	285.4521	239.0822	0.898147	4.83E-10	PIP4P1
803.4246	887.696	866.9189	1214.707	1239.408	1193.295	-0.5123	4.85E-10	ABHD17C
4830.433	5133.974	4783.567	7445.913	6803.625	6020.427	-0.45896	4.87E-10	PPIF
1603.255	1612.912	1533.99	1196.035	865.8015	812.4562	0.724512	4.87E-10	FBXW5
125.8159	116.9064	138.707	304.9733	281.2543	229.5612	-1.09607	4.88E-10	MRPS17
2484.865	2645.255	2511.327	2007.222	1643.449	1542.397	0.557012	4.90E-10	TRMT112
1967.222	2123.139	1836.043	1269.685	1288.732	1510.661	0.542295	4.91E-10	DBI
212.9884	282.3587	237.262	436.7135	420.832	485.5695	-0.87621	4.95E-10	DOCK4
367.5623	344.7748	337.6421	543.5578	598.1901	563.8531	-0.69961	5.00E-10	RNF169
710.8601	620.1983	744.6377	1018.652	1013.775	1142.517	-0.61224	5.04E-10	ORC2
168.0541	172.3874	181.5967	77.79931	69.26412	65.58891	1.295342	5.07E-10	PLA2G6
3841.88	4367.147	4112.846	5556.945	5335.436	5239.708	-0.38897	5.08E-10	MCM3
619.1942	667.7535	657.9458	998.9431	974.9449	905.5501	-0.56654	5.18E-10	GOSR2
1058.651	1027.389	1057.641	1362.007	1579.432	1541.339	-0.51169	5.21E-10	RAB3GAP2
37.74478	61.4254	75.74134	0	3.148369	0	5.79691	5.24E-10	C2CD4C
2939.6	2899.873	3092.619	2344.353	2242.688	2241.66	0.38746	5.28E-10	MARCKS
1245.578	1295.878	1221.899	940.853	813.3287	746.8673	0.589113	5.33E-10	BCKDK
1151.216	1163.12	1187.223	1523.829	1669.685	1636.549	-0.46398	5.35E-10	SOAT1
463.7216	470.5978	458.0982	303.936	234.0288	240.14	0.839108	5.37E-10	ARRDC1-AS1
59.31323	56.47173	66.61587	8.298593	12.59348	4.231543	2.859301	5.40E-10	BRSK1
752.1996	794.5672	740.075	1108.9	1075.693	1090.68	-0.5187	5.43E-10	SLC25A44
601.2205	520.1344	595.8927	819.4861	901.483	935.1709	-0.62827	5.43E-10	ITSN2
2183.805	2168.713	2136.271	2769.655	2773.713	2873.217	-0.37527	5.45E-10	SAFB
273.2003	272.4513	227.224	411.8177	479.6016	497.2063	-0.84567	5.46E-10	BICRAL
352.2846	340.8119	342.2048	532.1473	567.7559	570.2004	-0.68962	5.51E-10	PDE10A
809.7154	824.2892	826.7669	1101.638	1277.188	1194.353	-0.53809	5.52E-10	UBR2
2213.462	2233.11	2247.601	2835.007	2914.34	3245.593	-0.42615	5.57E-10	IFI16
3202.914	3562.673	3514.215	4298.671	4628.102	4602.861	-0.39647	5.59E-10	ARHGEF12
248.9358	276.4143	283.8019	456.4226	477.5026	447.4856	-0.77213	5.63E-10	PGBD1
432.2676	441.8666	453.5355	275.9282	230.8804	256.0083	0.799324	5.64E-10	SLC17A5
702.7719	747.0121	775.6643	1014.503	1109.275	1194.353	-0.57629	5.76E-10	ZNF791
506.8585	569.671	556.6532	792.5156	877.3455	823.0351	-0.61065	5.79E-10	MIS12
163.5607	172.3874	171.5587	288.3761	327.4304	382.9546	-0.97675	5.79E-10	ANKEF1
1116.167	1083.861	1158.934	787.329	764.0042	798.7037	0.515565	5.79E-10	TMEM135
180.6357	184.2762	209.8856	320.5332	439.7222	368.1442	-0.97274	5.85E-10	TTC14

1371.394	1294.887	1387.983	1758.264	1993.967	1866.11	-0.47037	5.85E-10	PURB
3038.455	2964.271	2976.726	2373.398	2314.051	2236.37	0.375073	5.92E-10	SOWAHC
226.4687	225.8869	240.9122	408.7057	425.0298	382.9546	-0.8113	5.94E-10	MITD1
85.3751	97.09175	75.74134	19.70916	24.1375	8.463085	2.301322	5.99E-10	HES6
1079.321	1214.638	1161.671	1812.205	1662.339	1522.297	-0.53256	6.00E-10	NUP85
761.1864	767.8174	845.0178	1215.744	1067.297	1256.768	-0.57616	6.08E-10	PTRH2
205.7989	197.1557	225.3989	350.6156	389.3483	403.0544	-0.8624	6.10E-10	ERI2
460.1269	454.7461	493.6875	736.5001	751.4107	687.6257	-0.62692	6.13E-10	VPS45
87.17247	96.10102	97.64245	24.89578	8.395651	26.44714	2.233129	6.41E-10	SLC4A3
638.0665	636.0501	581.2919	392.1085	388.2988	389.3019	0.665399	6.43E-10	MTSS1L
55.71849	61.4254	59.3155	8.298593	11.54402	4.231543	2.872201	6.46E-10	SYT15
147.3844	149.6006	167.9085	283.1895	307.4907	308.9026	-0.95193	6.47E-10	SIAH1
283.9845	289.2938	288.3646	163.8972	136.4293	131.1778	0.997251	6.48E-10	DDIT3
382.8399	395.3021	363.1934	619.2825	588.745	600.8791	-0.66486	6.48E-10	B4GALT3
1812.648	1680.282	1823.267	1346.447	1269.842	1252.537	0.458736	6.59E-10	PAIP2
856.4471	879.7702	750.113	543.5578	482.7499	345.9286	0.856985	6.79E-10	STARD3
457.4308	429.9778	413.3834	622.3945	719.9271	758.504	-0.69124	6.85E-10	HIST1H4I
4679.454	4846.662	4604.708	3803.868	3668.899	3411.681	0.376433	6.87E-10	WDR1
2253.004	2411.442	2361.67	3290.392	3249.117	2902.838	-0.42664	6.93E-10	MCM5
895.0905	950.1122	868.744	1279.021	1378.986	1229.263	-0.5188	6.97E-10	SBF2
294.7688	295.2382	267.376	136.9268	156.369	140.6988	0.982112	6.97E-10	MTHFSD
674.9126	693.5125	719.999	955.3755	1040.011	1064.233	-0.55086	7.07E-10	MED23
2004.068	2138.991	2131.708	2722.976	2923.785	2684.914	-0.40922	7.08E-10	DPY19L1
179.7371	143.6562	159.6956	66.38874	65.06629	46.54697	1.440705	7.09E-10	TGFB1I1
6530.746	5938.449	6180.676	5016.5	4514.761	4792.222	0.380881	7.11E-10	S100A10
805.222	862.9277	879.6946	1284.207	1253.051	1142.517	-0.53063	7.12E-10	PREP
183.3318	172.3874	177.9465	69.50072	67.16521	83.57297	1.27749	7.15E-10	WDR25
358.5754	329.9138	360.4558	577.7895	563.5581	547.9848	-0.68687	7.23E-10	PFKFB2
371.157	394.3114	336.7296	191.905	173.1603	218.9823	0.915899	7.32E-10	TEP1
779.1601	936.2419	861.4436	601.648	506.8874	491.9168	0.686368	7.33E-10	AIP
2832.656	2998.946	2921.973	3755.113	3687.79	3731.163	-0.35239	7.48E-10	ANTXR1
42.23821	40.62002	41.97713	0	0	0	7.753859	7.50E-10	-
22650.46	23940.05	22576.39	19379.29	17809.27	17824.32	0.330268	7.50E-10	RPS3
1050.563	1032.343	1113.306	751.0227	743.0151	729.9411	0.523388	7.52E-10	SNX19
563.4757	621.1891	541.1399	383.8099	274.9576	241.1979	0.938527	7.60E-10	MSRB1
46.73163	70.34198	59.3155	10.37324	6.296738	7.4052	2.870473	7.67E-10	AC007255.1
4396.368	4547.461	4314.519	3486.446	3555.558	3351.382	0.351105	7.87E-10	MYO1C
780.9575	764.8452	670.7215	1286.282	1117.671	1031.439	-0.63247	8.01E-10	ALG3
301.9583	331.8953	377.7941	582.9762	523.6787	614.6316	-0.76678	8.09E-10	GPATCH2
3711.57	3970.855	3758.778	3076.703	2488.261	2405.632	0.521297	8.15E-10	ATP5MC3
235.4555	236.785	230.8742	385.8846	407.1891	428.4437	-0.79685	8.28E-10	ZNF280D
4930.187	5321.223	5015.354	4061.124	3872.494	3447.649	0.423562	8.36E-10	ITGB5
79.98299	83.2215	61.1406	8.298593	14.69239	19.04194	2.417423	8.49E-10	AC026471.1
1242.882	1173.027	1169.884	1624.45	1613.014	1637.607	-0.44291	8.54E-10	DUSP3
44.03558	41.61075	59.3155	4.149297	3.148369	5.289428	3.527176	8.73E-10	FAM83F
549.9954	584.532	566.6912	851.6431	871.0488	807.1668	-0.5729	8.95E-10	TGFBRAP1
1533.157	1621.829	1575.967	2061.163	2267.875	2068.166	-0.43552	9.01E-10	TMEM43
101.5514	80.24931	75.74134	22.82113	18.89021	20.09983	2.059214	9.07E-10	ROM1
967.884	978.8434	969.1241	1503.083	1438.805	1261	-0.52763	9.09E-10	POLA2
81.78036	68.36052	81.21661	17.63451	9.445107	20.09983	2.294909	9.33E-10	WDR31
1751.538	1895.271	1862.507	1368.231	1384.233	1249.363	0.460934	9.42E-10	SLC39A9
188.7239	156.5357	162.4332	72.61269	65.06629	66.6468	1.313648	9.58E-10	EPOR
478.9993	507.2549	445.3226	767.6199	757.7075	693.973	-0.63319	9.63E-10	AIMP2
1046.07	1069.991	1051.253	1557.024	1370.59	1512.777	-0.48757	9.69E-10	PMPCB

1102.687	1079.898	928.9721	739.6121	597.1407	517.3061	0.746583	9.80E-10	DPP7
810.6141	784.6599	719.999	507.2515	518.4314	490.859	0.610307	9.86E-10	HNRNPUL2
338.8044	286.3216	346.7676	156.6359	187.8527	138.583	1.009248	9.88E-10	PLSCR4
1469.35	1595.079	1518.477	2094.357	2049.588	1996.23	-0.42241	9.90E-10	SLC31A1
6069.72	6226.752	5991.778	8230.13	7805.856	7329.032	-0.35352	9.91E-10	CD59
373.8531	389.3577	437.1096	618.2452	663.2564	632.6156	-0.67308	9.91E-10	RAB4A
819.601	841.1316	770.189	1125.497	1147.056	1188.006	-0.50978	9.93E-10	GCLC
401.7123	437.9036	386.0071	227.174	249.7706	211.5771	0.831303	1.00E-09	BCORL1
188.7239	190.2206	193.4598	86.0979	66.11575	89.92028	1.241263	1.00E-09	LINC00339
90.76721	82.23077	113.1557	23.85846	28.33532	23.27348	1.923777	1.01E-09	WBP1
1951.046	2017.131	1965.625	2628.579	2740.131	2479.684	-0.4036	1.03E-09	TNPO3
59.31323	48.54588	53.84023	4.149297	9.445107	6.347314	3.02145	1.04E-09	PIP5KL1
329.8175	333.8767	310.2657	511.4008	537.3216	528.9428	-0.69612	1.04E-09	AP3M2
74.59088	56.47173	74.82879	11.41057	14.69239	12.69463	2.409504	1.04E-09	FAM117A
140.1949	129.7859	150.5701	289.4134	253.9684	293.0343	-0.99098	1.05E-09	RP9
603.9165	656.8554	670.7215	896.2481	1020.072	965.8496	-0.57764	1.06E-09	MYO9A
2205.374	2365.868	2110.719	1155.579	879.4444	706.6676	1.285088	1.07E-09	GPAA1
1389.367	1381.081	1498.401	1915.938	1958.286	1881.979	-0.4311	1.07E-09	HADHB
401.7123	444.8387	473.6115	702.2684	696.839	669.6416	-0.64835	1.09E-09	DHX35
2607.985	2779.994	2646.384	3503.044	3603.833	3332.34	-0.37799	1.09E-09	MARS
103.3488	108.9805	88.51698	12.44789	28.33532	31.73657	2.053832	1.09E-09	LRG1
45.83295	60.43466	55.66532	10.37324	4.197825	3.173657	3.185235	1.10E-09	AL513304.1
116.8291	113.9342	93.07971	38.38099	23.08804	10.57886	2.165868	1.11E-09	INAFM1
154.5739	106.0083	121.3686	252.0698	278.1059	285.6291	-1.09231	1.11E-09	TEFM
1009.224	1174.018	1053.991	792.5156	595.0417	596.6475	0.705595	1.14E-09	CENPX
448.444	367.5616	442.5849	663.8874	685.295	666.468	-0.67813	1.15E-09	CHMP2B
252.5306	268.4884	236.3495	399.3698	442.8706	492.9747	-0.8185	1.16E-09	ZNF304
444.8492	491.4032	491.8624	696.0445	772.3999	733.1148	-0.6248	1.16E-09	RUNDC1
38.64347	45.57368	51.10259	4.149297	3.148369	2.115771	3.842981	1.16E-09	HSH2D
499.669	469.6071	466.3111	792.5156	726.2238	695.0309	-0.62472	1.17E-09	MCUB
688.3929	807.4467	729.1244	519.6994	417.6836	407.286	0.725735	1.19E-09	COX14
148.2831	186.2576	180.6842	334.0184	323.2326	319.4815	-0.92414	1.20E-09	PSMD9
1387.57	1433.589	1293.991	975.0847	1005.379	956.3287	0.486423	1.20E-09	PPP6R2
1065.841	1103.676	1053.078	761.3959	603.4374	458.0645	0.82161	1.21E-09	BRAT1
417.8887	454.7461	408.8207	647.2903	672.7015	666.468	-0.63312	1.22E-09	FAM173B
66.50271	89.1659	66.61587	188.793	193.1	162.9144	-1.29654	1.22E-09	CCBE1
7683.759	7755.451	7515.731	9164.759	9478.69	9715.622	-0.30502	1.23E-09	SERPINE2
36.8461	44.58295	40.15203	122.4042	119.638	115.3095	-1.55745	1.24E-09	KBTBD8
212.0897	185.2669	226.3115	88.17255	95.50053	95.20971	1.162141	1.24E-09	AKNA
154.5739	140.684	127.7565	53.94086	51.42336	50.77851	1.437869	1.25E-09	LPAR2
907.6721	915.4365	866.0064	636.917	519.4809	417.8648	0.772058	1.26E-09	SNHG15
3277.505	3353.628	3287.904	2632.729	2208.056	1938.047	0.549019	1.26E-09	POLR2E
905.8748	861.937	867.8315	1166.99	1268.793	1308.605	-0.50628	1.27E-09	SOCS5
360.3728	367.5616	375.0565	592.3121	548.8657	613.5737	-0.66986	1.28E-09	IFT81
1782.093	2053.788	1945.549	1480.262	1276.139	1185.89	0.551986	1.29E-09	CIB1
984.9591	1008.565	981.8997	1314.29	1374.788	1451.419	-0.47678	1.31E-09	TRA2A
897.7866	956.0566	916.1964	629.6558	601.3385	658.0049	0.552041	1.32E-09	CLASRP
1335.446	1347.396	1302.203	1013.466	834.3178	759.5619	0.611737	1.35E-09	C7orf50
8424.276	8538.13	8781.432	5255.084	6418.475	6793.742	0.479301	1.35E-09	SLC2A3
266.0108	359.6358	226.3115	704.3431	499.5412	527.885	-1.02525	1.36E-09	DDX52
1502.602	1538.607	1505.701	1057.033	1053.654	1171.079	0.470413	1.36E-09	CHD2
2835.352	2794.855	2840.756	3646.194	3577.597	3518.528	-0.34267	1.37E-09	TAB2
248.9358	260.5626	277.4141	141.0761	105.9951	132.2357	1.052657	1.37E-09	GABPB1-IT1
5165.643	5525.313	5347.521	6611.904	6751.153	6642.464	-0.319	1.38E-09	EPAS1

242.645	214.9889	203.4978	80.91128	97.59944	113.1938	1.181281	1.39E-09	CREG2
228.2661	188.2391	188.8971	103.7324	50.3739	60.29948	1.497048	1.40E-09	ARID5A
59.31323	44.58295	50.19004	3.111972	9.445107	2.115771	3.393326	1.43E-09	KCNH3
774.6667	804.4745	772.0141	1078.817	1095.632	1170.022	-0.5086	1.43E-09	ITFG1
410.6992	409.1724	416.1211	238.5846	241.375	245.4295	0.768942	1.44E-09	MFAP5
563.4757	642.9852	605.0181	373.4367	361.013	396.7071	0.678879	1.44E-09	VWA5A
554.4888	505.2734	540.2274	937.741	749.3118	826.2087	-0.65112	1.45E-09	CHURC1
2642.135	2760.18	2741.289	3471.924	3485.245	3412.739	-0.3488	1.45E-09	YY1
1026.299	1039.278	981.8997	1373.417	1450.349	1379.483	-0.46406	1.47E-09	COX15
1666.163	1580.218	1531.253	2042.491	2215.402	2165.492	-0.42689	1.48E-09	CDK17
118.6265	99.07322	132.3192	229.2486	267.6114	246.4874	-1.08457	1.48E-09	CEP76
1553.827	1645.606	1594.218	2082.947	2243.738	2078.745	-0.4184	1.51E-09	TYMS
668.6218	647.9388	685.3222	910.7706	1030.566	993.3546	-0.5515	1.51E-09	PPM1B
6935.154	7740.591	7801.358	10240.46	9907.917	9197.258	-0.38479	1.51E-09	LARP1
1334.548	1514.83	1409.884	1074.668	916.1754	849.4822	0.584049	1.53E-09	RCC1L
565.273	568.6803	511.0259	297.712	331.6282	360.739	0.732489	1.53E-09	IRF2
2077.76	2248.962	2120.757	2880.649	2738.032	2808.686	-0.38667	1.53E-09	ATP6V1E1
201.3055	187.2484	221.7487	369.2874	425.0298	338.5234	-0.89155	1.55E-09	PYGO1
868.13	918.4087	896.1204	1219.893	1239.408	1276.868	-0.47815	1.56E-09	UXS1
307.3504	320.0065	296.5775	173.2331	167.913	142.8146	0.932298	1.57E-09	PHC1
53.92112	57.46247	63.87824	13.48521	5.247282	5.289428	2.864052	1.57E-09	CXCL10
1290.512	1316.683	1403.496	979.234	963.4009	836.7876	0.528977	1.57E-09	BCR
44.93426	49.53661	52.01513	4.149297	0	7.4052	3.666236	1.59E-09	CALHM5
1422.619	1580.218	1423.572	1097.489	1030.566	903.4344	0.545613	1.61E-09	PLPP2
674.9126	665.772	627.8318	961.5995	913.027	992.2968	-0.54246	1.62E-09	KMT5A
782.7549	836.178	798.4779	509.3262	549.9151	556.4479	0.581116	1.63E-09	DAB2
1430.707	1527.709	1406.234	1071.556	1020.072	1066.349	0.466525	1.63E-09	CTDNEP1
805.222	769.7989	807.6034	1069.481	1152.303	1170.022	-0.50913	1.64E-09	TARBP1
77.28693	59.44393	61.1406	15.55986	11.54402	5.289428	2.609148	1.65E-09	KCNIP3
959.7959	1015.5	911.6337	695.0072	593.9923	502.4957	0.687857	1.68E-09	RUSC2
798.0325	757.9101	785.7023	1188.773	1053.654	1119.243	-0.52143	1.68E-09	CHAF1B
798.9312	740.0769	767.4514	1025.914	1147.056	1203.874	-0.54952	1.69E-09	MID1
73.69219	85.20297	68.44097	9.335917	10.49456	23.27348	2.400367	1.70E-09	AC008443.1
268.7069	300.1918	284.7144	131.7402	158.4679	145.9882	0.968281	1.71E-09	USP35
135.7015	144.6469	139.6196	244.8085	290.6994	295.1501	-0.98428	1.72E-09	RAPGEF6
299.2622	278.3957	264.6384	438.7881	478.5521	505.6694	-0.75618	1.72E-09	WDR72
246.2398	223.9055	223.5738	399.3698	419.7825	384.0125	-0.79391	1.75E-09	MRPS9
1629.316	1557.431	1549.503	1225.08	1070.445	992.2968	0.526527	1.75E-09	CCND3
446.6466	433.9407	460.8358	737.5375	656.9597	674.9311	-0.62526	1.76E-09	ASF1A
760.2877	891.659	815.8163	1444.993	1264.595	1109.722	-0.63079	1.76E-09	COMT
444.8492	469.6071	491.8624	765.5452	689.4928	705.6097	-0.61974	1.78E-09	NGDN
132.1067	123.8415	74.82879	35.26902	28.33532	19.04194	1.999268	1.79E-09	CICP14
178.8384	125.823	169.7336	53.94086	47.22554	68.76257	1.482993	1.80E-09	PAQR6
661.4324	654.874	578.5543	406.6311	370.4581	410.4596	0.673904	1.81E-09	ALAD
274.099	275.4235	288.3646	156.6359	139.5777	135.4094	0.95681	1.82E-09	SNRNP35
771.072	803.4838	734.5997	523.8487	519.4809	433.7331	0.644165	1.87E-09	SLC35A2
241.7463	212.0167	231.7867	126.5535	75.56086	85.68874	1.251974	1.88E-09	DGKQ
127.6133	104.0269	106.7679	35.26902	39.87934	32.79446	1.649158	1.89E-09	RORA
452.9374	422.0519	397.8702	230.286	258.1663	248.6031	0.788489	1.91E-09	ABLIM3
866.3326	905.5292	865.0938	616.1705	611.833	569.1425	0.552829	1.93E-09	ADGRL1
296.5661	330.9045	361.3683	563.267	522.6293	542.6954	-0.72009	1.94E-09	HPF1
142.891	111.9527	133.2317	244.8085	270.7597	272.9345	-1.02071	1.94E-09	RBM48
462.8229	477.5329	477.2617	681.522	739.8667	723.5938	-0.59758	1.97E-09	DYNLT3
330.7162	359.6358	344.9425	168.0465	205.6934	189.3615	0.878395	1.98E-09	ZNF226

318.1346	302.1733	250.0377	150.412	146.9239	140.6988	0.990265	2.00E-09	IL11
345.9938	354.6821	314.8284	202.2782	136.4293	101.557	1.204821	2.04E-09	BLOC1S4
937.3287	888.6868	881.5196	1181.512	1457.695	1330.82	-0.55194	2.05E-09	STK3
1730.868	1811.058	1929.123	2317.382	2630.987	2488.147	-0.44278	2.06E-09	OTULIN
1185.366	1119.527	1166.234	1463.664	1774.631	1894.673	-0.56409	2.07E-09	N4BP2L2
632.6744	640.013	585.8547	430.4895	314.8369	352.2759	0.75939	2.12E-09	ZCCHC24
2162.237	2302.462	2185.548	3617.149	3174.605	2756.85	-0.52209	2.13E-09	STOML2
36.8461	45.57368	34.67676	119.2923	123.8358	106.8465	-1.58257	2.19E-09	PSMA6P1
1362.407	1422.691	1385.245	1802.869	1995.017	1839.663	-0.43507	2.20E-09	RAB6A
1135.04	1119.527	1147.983	1668.017	1630.855	1446.13	-0.47979	2.21E-09	PFAS
206.6976	249.6645	211.7107	109.9564	103.8962	95.20971	1.110836	2.26E-09	UQCC3
113.2343	130.7766	107.6805	47.71691	31.48369	22.2156	1.791823	2.27E-09	ALDH4A1
147.3844	186.2576	149.6576	74.68734	55.62119	49.72063	1.422792	2.28E-09	SLC27A5
166.2568	147.6191	154.2203	60.1648	71.36303	45.48908	1.402996	2.29E-09	TMEM37
654.2429	583.5413	610.4934	831.934	1014.824	1177.427	-0.70982	2.29E-09	AKAP9
238.1516	262.544	280.1517	406.6311	503.739	455.9487	-0.80732	2.34E-09	-
964.2893	915.4365	996.5005	1390.014	1259.348	1402.756	-0.49414	2.36E-09	CENPH
559.8809	472.5792	392.3949	288.3761	216.188	169.2617	1.080024	2.36E-09	BX255925.3
283.0859	260.5626	292.9273	482.3557	430.2771	499.322	-0.75435	2.38E-09	TMEM206
150.9791	135.7303	118.631	46.67959	45.12662	53.95217	1.475951	2.39E-09	AC018638.5
135.7015	123.8415	118.631	270.7416	222.4847	286.687	-1.04384	2.39E-09	EPCAM
1486.425	1628.764	1565.017	2089.171	2085.27	2042.777	-0.41008	2.43E-09	RTCB
203.1029	231.8313	248.2126	101.6578	114.3907	104.7307	1.090396	2.43E-09	SH3PXD2A
61.1106	63.40686	97.64245	237.5472	195.1989	159.7407	-1.41418	2.45E-09	HTR7
952.6064	1069	980.0746	728.2015	644.3662	559.6215	0.63493	2.46E-09	PICSAR
673.1153	686.5774	552.0905	1103.713	928.7689	962.676	-0.64826	2.47E-09	TMA7
126.7146	100.0639	102.2052	42.53029	27.28586	17.98406	1.90473	2.56E-09	MAP1LC3B2
455.6334	466.6349	444.41	748.948	683.1961	662.2364	-0.61622	2.59E-09	PIGU
306.4517	318.025	344.9425	533.1846	513.1842	517.3061	-0.68971	2.65E-09	ETFDH
613.802	665.772	610.4934	423.2282	407.1891	356.5075	0.670574	2.65E-09	C11orf49
116.8291	118.8879	105.8554	39.41832	41.97825	27.50503	1.648256	2.68E-09	LINC01085
381.9412	438.8944	410.6458	577.7895	708.383	753.2146	-0.72822	2.68E-09	USP3
838.4734	962.9917	1001.976	1309.103	1329.661	1389.004	-0.52309	2.69E-09	RAB3GAP1
1513.386	1457.367	1523.04	1892.079	2045.39	2099.903	-0.42572	2.70E-09	SMAD2
424.1795	424.0334	448.9727	274.8909	219.3364	253.8926	0.794008	2.70E-09	NAT9
334.3109	365.5802	362.2808	570.5283	525.7776	622.0368	-0.69437	2.70E-09	CNOT4
119.5251	139.6932	111.3306	50.82888	37.78043	28.56291	1.659032	2.71E-09	LMX1B
330.7162	377.469	378.7067	218.8754	195.1989	180.8985	0.868599	2.71E-09	ADCK2
1188.062	1208.693	1095.968	1780.048	1820.807	1477.866	-0.54038	2.74E-09	IL18
119.5251	109.9713	102.2052	34.2317	34.63206	39.14177	1.619216	2.78E-09	MMP25-AS1
321.7293	310.0992	341.2923	584.0135	523.6787	491.9168	-0.71675	2.79E-09	-
470.9111	400.2558	407.9082	651.4396	658.0091	698.2045	-0.64956	2.79E-09	ZBTB6
514.048	543.912	490.0373	787.329	796.5374	738.4042	-0.58573	2.82E-09	PLPBP
138.3975	137.7118	163.3458	53.94086	45.12662	64.53103	1.426225	2.85E-09	AC084033.3
3533.631	3397.221	3647.447	2325.681	2645.679	2811.86	0.442857	2.89E-09	PDP1
2170.325	1977.501	2017.64	1595.405	1391.579	1510.661	0.455199	2.90E-09	CNN2
1003.831	1001.63	996.5005	1339.185	1452.448	1342.457	-0.46166	2.92E-09	TCF12
57.51586	58.4532	32.85166	5.186621	6.296738	3.173657	3.341936	2.92E-09	TBX2-AS1
569.7665	566.6988	502.813	362.0261	280.2048	326.8867	0.757971	2.97E-09	PRR14
5.392112	1.981464	8.212916	52.90353	43.02771	47.60486	-3.18793	2.97E-09	LINC00973
373.8531	372.5153	359.5432	217.8381	213.0396	204.1719	0.800124	3.09E-09	ZC3H3
472.7085	477.5329	530.1894	327.7944	258.1663	279.2818	0.77476	3.16E-09	CYB5R1
1923.186	2082.519	1875.282	1473	1444.052	1215.511	0.508662	3.19E-09	DGCR2
3666.636	4033.271	3838.169	2915.918	3036.077	3023.437	0.362162	3.27E-09	ATP11A

462.8229	501.3105	501.9004	762.4332	735.6689	707.7255	-0.58984	3.29E-09	ASB8
3.594741	0.990732	1.825092	35.26902	47.22554	35.96811	-4.18799	3.33E-09	-
81.78036	54.49027	59.3155	12.44789	13.64293	9.520971	2.458185	3.33E-09	KLHL3
192.3186	201.1186	245.4749	74.68734	113.3413	78.28354	1.262868	3.33E-09	PRX
3467.128	3487.377	3270.566	2720.901	2044.341	1834.374	0.631517	3.34E-09	SLC1A5
26.06187	19.81464	23.7262	76.76199	82.90705	101.557	-1.90481	3.37E-09	BRD7P2
81.78036	76.28638	97.64245	26.97043	20.98913	12.69463	2.074887	3.42E-09	PRRT3
264.2135	222.9147	243.6498	391.0712	431.3266	440.0804	-0.7875	3.46E-09	BX890604.1
2180.21	2280.665	2458.4	2913.844	3082.253	3331.282	-0.43079	3.46E-09	EPB41L2
276.7951	330.9045	271.9388	479.2438	528.926	474.9907	-0.75512	3.53E-09	POLH
1089.207	1146.277	1041.215	1543.538	1468.189	1474.693	-0.45375	3.59E-09	MLH1
104.2475	131.7674	166.0834	29.04508	39.87934	53.95217	1.711255	3.67E-09	CNN3
490.6822	510.2271	512.851	755.172	732.5205	754.2725	-0.56681	3.68E-09	FAHD1
473.6071	508.2456	495.5126	754.1346	697.8885	770.1408	-0.58932	3.74E-09	C18orf25
2109.214	2360.915	2294.141	1762.414	1648.696	1568.844	0.44147	3.82E-09	AP1B1
45.83295	35.66636	48.36495	4.149297	4.197825	1.057886	3.785367	3.82E-09	LIPE
67.4014	98.08249	85.77934	193.9796	205.6934	180.8985	-1.21108	3.88E-09	XYLB
1146.722	1207.703	1139.77	857.8671	819.6254	825.1508	0.48118	3.89E-09	RREB1
978.6683	931.2882	921.6717	1241.677	1347.502	1343.515	-0.47365	3.90E-09	MIER1
697.3798	644.9666	594.0676	940.853	955.0053	942.5761	-0.55157	3.93E-09	NOA1
68.30008	85.20297	78.47897	16.59719	13.64293	23.27348	2.115972	3.98E-09	ZFP90
877.1168	959.0287	953.6108	631.7304	648.564	647.426	0.532977	3.99E-09	ZFHX3
289.3767	287.3123	299.3152	162.8599	157.4185	150.2198	0.896735	4.00E-09	RFX1
556.2862	538.9583	530.1894	372.3994	315.8864	281.3976	0.744949	4.02E-09	SERTAD3
1404.645	1370.183	1472.85	1068.444	945.5602	783.8933	0.602237	4.07E-09	ZNF512B
405.3071	371.5246	403.3454	244.8085	204.644	226.3875	0.804571	4.08E-09	LRRC45
330.7162	314.0621	289.2772	498.9529	494.2939	520.4798	-0.69646	4.12E-09	TBX18
250.7332	251.646	261.9008	421.1536	406.1396	455.9487	-0.74743	4.14E-09	COMMD10
423.2808	427.9963	472.6989	680.4846	691.5917	652.7155	-0.6127	4.14E-09	PI4K2B
71.89482	106.0083	71.17861	23.85846	18.89021	14.8104	2.110535	4.23E-09	MMP17
705.4679	632.0871	632.3945	941.8903	1022.17	924.5921	-0.5518	4.25E-09	NPAS2
397.2189	447.8109	441.6724	604.76	764.0042	701.3782	-0.68642	4.27E-09	CXADR
59.31323	47.55514	56.57787	5.186621	8.395651	10.57886	2.76094	4.33E-09	ATP2A3
1206.036	1149.249	1325.017	1716.771	1664.438	1671.459	-0.45683	4.34E-09	FBXO30
124.0186	134.7396	119.5436	279.0402	252.919	227.4454	-1.00646	4.34E-09	UBE2F
453.8361	460.6905	494.6001	697.0818	691.5917	736.2884	-0.59245	4.36E-09	PLEKHF2
48.52901	46.56441	43.80222	7.261269	4.197825	3.173657	3.243992	4.38E-09	AC007785.1
54.8198	46.56441	34.67676	5.186621	3.148369	4.231543	3.435766	4.43E-09	PRR13P5
1836.014	1905.178	1822.355	2472.981	2436.838	2329.464	-0.38007	4.46E-09	ARIH2
141.0936	82.23077	86.69189	24.89578	25.18695	31.73657	1.923849	4.47E-09	SEC31B
612.0047	736.114	640.6074	935.6664	1003.28	1018.744	-0.57355	4.48E-09	HNRNPH1
1329.156	1277.054	1324.105	1682.54	1753.642	1871.4	-0.43314	4.48E-09	CDYL
257.9227	225.8869	226.3115	398.3325	412.4363	401.9966	-0.77141	4.51E-09	SFMBT2
1817.142	1990.381	1808.667	1443.955	1316.018	1144.632	0.524024	4.59E-09	KIAA2013
852.8523	901.5663	901.5957	515.5501	609.7341	631.5577	0.596352	4.61E-09	OPTN
736.0232	671.7164	725.4742	1015.54	984.3901	1048.365	-0.51438	4.68E-09	COL4A3BP
88.96984	95.11029	105.8554	22.82113	29.38478	32.79446	1.77099	4.70E-09	ANGPTL2
40.44084	46.56441	40.15203	4.149297	3.148369	3.173657	3.600364	4.71E-09	FLVCR2
206.6976	234.8035	294.7524	418.0416	468.0575	450.6593	-0.86027	4.75E-09	ICK
352.2846	392.3299	422.5089	622.3945	595.0417	623.0947	-0.65743	4.76E-09	KIAA0753
2710.435	2706.68	2759.54	2225.06	2008.659	1965.552	0.399346	4.81E-09	SZRD1
78.18562	87.18443	65.70333	14.52254	19.93967	20.09983	2.08224	4.85E-09	ACER2
990.3512	998.658	970.9492	1281.095	1420.964	1381.599	-0.46431	4.89E-09	SERTAD2
73.69219	97.09175	65.70333	23.85846	13.64293	14.8104	2.173739	4.91E-09	LIMS2

287.5793	336.8489	374.1439	505.1769	609.7341	581.8371	-0.76504	4.94E-09	LRRC57
414.2939	376.4782	379.6192	604.76	574.0526	679.1626	-0.66619	4.98E-09	ZNF655
646.1547	646.9481	709.961	1025.914	920.3732	967.9654	-0.54075	5.00E-09	TP53BP2
723.4417	717.2901	745.5503	981.3086	1053.654	1066.349	-0.50419	5.04E-09	USP13
114.133	82.23077	73.91624	23.85846	18.89021	26.44714	1.966744	5.07E-09	ROBO4
331.6149	393.3207	393.3074	210.5768	213.0396	142.8146	0.980457	5.10E-09	STK11IP
745.9088	909.4921	745.5503	541.4832	508.9863	473.9328	0.654417	5.14E-09	SMPD1
2930.613	2702.717	2732.163	3715.695	3418.079	3765.015	-0.38147	5.24E-09	AP3S1
452.0387	483.4773	457.1857	287.3388	279.1554	194.651	0.870999	5.26E-09	RHBDD3
906.7734	888.6868	926.2344	1178.4	1316.018	1316.01	-0.48519	5.27E-09	VPS13B
84.47642	72.32345	75.74134	19.70916	14.69239	22.2156	2.039	5.27E-09	BTN2A2
340.6017	372.5153	312.0908	645.2156	563.5581	507.7851	-0.7446	5.30E-09	UBIAD1
1601.457	1765.485	1708.287	2184.605	2257.381	2246.949	-0.39863	5.30E-09	TIMMDC1
485.2901	448.8017	511.9384	708.4924	741.9656	728.8832	-0.59106	5.37E-09	SLC25A43
685.6969	718.2808	726.3868	985.4579	1001.181	1025.091	-0.49963	5.39E-09	DNMBP
1786.586	1844.743	1808.667	1429.433	1338.057	1264.173	0.432011	5.39E-09	CKS2
1226.705	1118.537	1204.561	1619.263	1586.778	1590.002	-0.43364	5.41E-09	RBMS1
4713.604	4939.791	4645.773	2559.079	1944.643	1541.339	1.242055	5.50E-09	MLF2
41.33952	53.49954	38.32694	2.074648	6.296738	3.173657	3.527885	5.50E-09	FPR3
45.83295	36.65709	40.15203	2.074648	3.148369	3.173657	3.870788	5.58E-09	AC008147.2
44.93426	41.61075	37.41439	4.149297	0	2.115771	4.302107	5.68E-09	ESPN
629.0797	637.0408	612.3185	916.9945	895.1863	878.0451	-0.5184	5.70E-09	EAPP
66.50271	54.49027	47.4524	6.223945	11.54402	8.463085	2.684217	5.70E-09	SYT17
1029.893	1037.297	900.6831	732.3508	557.2613	597.7054	0.652717	5.71E-09	TCEA2
499.669	461.6812	503.7255	915.9572	707.3336	727.8253	-0.6821	5.73E-09	BOLA3
64.70534	70.34198	57.49041	11.41057	7.346194	16.92617	2.432221	5.77E-09	ZNF837
328.9188	312.0806	311.1783	520.7367	506.8874	489.8011	-0.67211	5.81E-09	RFT1
152.7765	143.6562	158.783	311.1972	288.6005	262.3556	-0.92088	5.82E-09	PRMT9
330.7162	320.0065	344.0299	484.4304	601.3385	544.8111	-0.71252	5.84E-09	NDUFAF7
756.693	764.8452	792.0901	1233.378	1071.495	1047.307	-0.53503	5.85E-09	DERL2
53.92112	57.46247	46.53986	7.261269	4.197825	10.57886	2.841666	5.87E-09	AC023043.1
613.802	603.3559	537.4897	362.0261	367.3097	382.9546	0.657554	5.87E-09	SCNN1A
79.98299	100.0639	93.99226	31.11972	25.18695	17.98406	1.881162	5.90E-09	BIK
3966.797	4180.89	3911.173	5073.552	5188.512	4901.184	-0.33067	5.91E-09	XPO6
366.6636	355.6729	297.4901	203.3155	175.2592	143.8725	0.964321	5.92E-09	WTIP
111.437	94.11956	102.2052	213.6888	224.5837	204.1719	-1.06043	5.96E-09	MITF
308.2491	321.988	285.627	130.7028	188.9021	137.5251	1.002319	5.97E-09	CAMK1D
803.4246	828.2521	826.7669	1103.713	1199.529	1128.764	-0.48141	6.04E-09	BACE2
61.1106	45.57368	43.80222	5.186621	3.148369	9.520971	3.077847	6.05E-09	STARD4-AS1
1249.173	1272.1	1285.778	1800.795	1752.592	1602.697	-0.43772	6.05E-09	GNPDA1
337.9057	283.3494	274.6764	178.4198	124.8853	134.3515	1.033592	6.05E-09	HDHD3
121.3225	172.3874	150.5701	66.38874	49.32445	43.37331	1.479635	6.13E-09	ERFE
158.1686	230.8406	209.8856	82.98593	90.25325	82.51508	1.226268	6.17E-09	RNF144A
1508.893	1640.652	1583.268	2042.491	2374.92	2073.456	-0.45595	6.17E-09	DYRK1A
43.13689	66.37906	62.05314	1.037324	1.049456	0	6.354083	6.17E-09	PRSS56
1056.854	1208.693	1101.443	1566.359	1665.487	1475.751	-0.48413	6.18E-09	KCTD10
74.59088	43.59222	54.75277	11.41057	6.296738	8.463085	2.724983	6.19E-09	FA2H
55.71849	47.55514	69.35351	9.335917	7.346194	11.63674	2.609487	6.20E-09	LINC01348
102.4501	115.9157	100.3801	31.11972	34.63206	38.08388	1.617777	6.20E-09	AC098614.1
115.0317	96.10102	83.95425	31.11972	28.33532	28.56291	1.74546	6.25E-09	MMP14
401.7123	451.7739	396.9576	275.9282	177.3581	184.0721	0.971331	6.31E-09	ZNRF1
1802.763	2063.695	1842.431	2768.618	2490.36	2494.494	-0.44212	6.40E-09	KIF2C
39.54215	32.69416	28.28893	0	0	0	7.441555	6.43E-09	KLK10
220.1779	191.2113	205.3229	376.5487	356.8152	346.9865	-0.80791	6.47E-09	SEPSECS

162.662	155.545	134.1443	56.0155	59.81901	65.58891	1.318246	6.61E-09	SELPLG
546.4007	486.4495	523.8015	731.3135	797.5868	799.7616	-0.58027	6.74E-09	UBE2D1
4204.948	4418.666	4382.959	6534.105	5678.608	5291.544	-0.42857	6.74E-09	MYBL2
6929.762	7337.363	7617.936	5557.983	5891.648	5913.581	0.333903	6.75E-09	LAMB1
103.3488	66.37906	77.56643	20.74648	23.08804	15.86829	2.051439	6.75E-09	EGLN2
984.0604	1022.436	918.0215	715.7537	655.9102	638.9629	0.540117	6.78E-09	PREB
1409.139	1325.6	1398.021	945.0023	1008.528	1046.249	0.462613	6.80E-09	CCNL2
1508.893	1352.349	1450.948	1962.617	2103.111	1837.547	-0.45273	6.92E-09	NR2F2
47.63032	56.47173	42.88967	5.186621	5.247282	8.463085	2.959924	7.10E-09	PIGZ
203.1029	187.2484	180.6842	103.7324	58.76956	62.41525	1.343451	7.22E-09	FUOM
983.1617	1032.343	1074.979	713.679	759.8064	628.3841	0.555993	7.45E-09	VPS26C
416.99	444.8387	387.8321	261.4057	217.2375	246.4874	0.784613	7.48E-09	DDRGK1
112.3357	109.9713	129.5816	48.75423	36.73097	23.27348	1.693007	7.50E-09	TTYH2
798.0325	863.9185	804.8658	1121.347	1138.66	1179.543	-0.48003	7.51E-09	DCAF16
44.93426	44.58295	48.36495	8.298593	0	1.057886	3.873244	7.55E-09	NECTIN4
610.2073	591.4671	603.1931	891.0614	932.9667	814.572	-0.54777	7.57E-09	GNPAT
161.7634	158.5171	181.5967	74.68734	58.76956	77.22565	1.25263	7.57E-09	TPT1-AS1
1809.053	1918.057	1773.99	2652.438	2378.068	2337.927	-0.42196	7.61E-09	PEA15
665.0271	739.0862	687.1473	435.6761	492.195	439.0226	0.613019	7.72E-09	CREBL2
2650.223	2761.171	2855.357	3368.191	3601.734	3701.542	-0.36836	7.77E-09	CLIP1
993.9459	922.3717	980.9872	1344.372	1281.386	1346.688	-0.4549	7.77E-09	ISCU
519.4401	510.2271	474.524	712.6417	829.0705	738.4042	-0.60018	7.83E-09	FUT11
97.05801	91.14736	109.5055	34.2317	23.08804	32.79446	1.724457	7.86E-09	GNAZ
147.3844	150.5913	126.8439	38.38099	60.86847	57.12583	1.442167	7.94E-09	LGALS9B
1202.441	1144.296	1148.896	1587.106	1517.514	1580.481	-0.42234	7.97E-09	ITGB1BP1
331.6149	331.8953	342.2048	210.5768	172.1108	140.6988	0.941775	7.98E-09	TEDC2
219.2792	199.1372	197.11	110.9937	86.05542	84.63085	1.127572	7.99E-09	AC006262.2
81.78036	66.37906	62.96569	18.67183	13.64293	14.8104	2.163475	8.02E-09	MSLNL
464.6203	451.7739	500.0753	712.6417	727.2732	683.3941	-0.58363	8.18E-09	PCGF6
1292.309	1308.757	1130.645	921.1438	840.6145	842.077	0.518866	8.21E-09	ETV4
2228.739	2304.443	2240.301	1803.907	1474.486	1296.968	0.565802	8.29E-09	MGAT1
743.2127	811.4097	855.9684	1127.571	1102.979	1219.742	-0.51746	8.31E-09	SDE2
71.89482	69.35125	75.74134	158.7106	180.5065	159.7407	-1.20069	8.31E-09	AC093297.2
1928.579	1966.603	1708.287	1412.835	1214.221	971.139	0.638771	8.35E-09	MRPL41
1238.388	1312.72	1174.447	926.3305	866.8509	871.6978	0.482959	8.39E-09	AP1S1
470.0124	461.6812	504.6381	728.2015	729.3722	687.6257	-0.57844	8.39E-09	DPH5
2313.216	2317.323	2264.94	3179.398	3015.088	2802.339	-0.38387	8.47E-09	PMPCA
31.45398	33.68489	63.87824	1.037324	4.197825	1.057886	4.359217	8.47E-09	AC117422.1
764.7812	762.8638	760.151	1021.764	1163.847	1061.059	-0.50496	8.48E-09	VPS36
84.47642	74.30491	65.70333	21.78381	17.84076	12.69463	2.10019	8.51E-09	TSNARE1
1179.075	1146.277	1122.432	879.6509	759.8064	789.1827	0.505436	8.54E-09	NKIRAS2
490.6822	523.1066	484.562	339.205	284.4027	290.9186	0.711675	8.58E-09	TRIM16
1039.779	1069.991	1065.854	767.6199	778.6966	684.452	0.509291	8.60E-09	ACSL1
1553.827	1495.015	1654.446	1970.916	2155.583	2277.628	-0.44501	8.65E-09	BOD1L1
405.3071	468.6163	459.0107	663.8874	677.9488	687.6257	-0.60717	8.70E-09	FAM13A
1526.866	1541.579	1460.986	1089.19	1169.094	1088.564	0.436417	8.70E-09	MEN1
2172.122	2201.407	1988.438	1682.54	1517.514	1463.056	0.447937	8.73E-09	TADA3
160.8647	166.443	169.7336	75.72466	69.26412	71.93623	1.19597	8.81E-09	GET4
177.9397	180.3133	198.9351	303.936	357.8646	341.6971	-0.84842	8.85E-09	C15orf41
232.7595	231.8313	241.8247	396.2578	405.0901	381.8967	-0.74403	8.91E-09	TCEAL1
79.98299	84.21224	78.47897	174.2705	174.2098	185.13	-1.13734	8.94E-09	FAM9B
66.50271	54.49027	118.631	16.59719	16.7913	14.8104	2.314973	8.97E-09	VPS28
460.1269	488.431	496.4251	288.3761	321.1336	275.0503	0.70781	9.03E-09	MRFAP1L1
52.12375	65.38832	62.96569	126.5535	182.6054	154.4513	-1.36244	9.04E-09	AC015813.6

396.3202	421.0612	420.6838	589.2001	634.9211	722.5359	-0.653	9.06E-09	MTRF1L
35.94741	35.66636	25.55129	0	0	0	7.391732	9.06E-09	MIR4697HG
604.8152	621.1891	620.5314	822.598	975.9944	919.3027	-0.55768	9.07E-09	KIF3A
733.3272	760.8823	787.5274	532.1473	514.2336	504.6115	0.556815	9.09E-09	SLCO3A1
228.2661	201.1186	229.9616	350.6156	389.3483	408.3439	-0.79919	9.13E-09	LIAS
62.90797	87.18443	61.1406	13.48521	12.59348	19.04194	2.226083	9.20E-09	METTL27
3615.411	3785.588	3585.394	2939.777	2909.093	2882.738	0.331228	9.20E-09	GPR107
314.5398	253.6274	271.9388	144.1881	145.8744	148.104	0.940028	9.21E-09	SLC6A9
1088.308	1108.629	1073.154	767.6199	800.7352	788.1248	0.472606	9.28E-09	DOT1L
7645.116	7559.287	7465.541	9544.419	9042.116	9085.122	-0.28764	9.28E-09	ARL6IP1
55.71849	65.38832	57.49041	12.44789	12.59348	8.463085	2.412614	9.32E-09	HSPA2
374.7518	412.1446	464.486	607.8719	690.5423	689.7415	-0.66786	9.54E-09	LMBRD2
338.8044	278.3957	288.3646	180.4944	147.9733	130.1199	0.98179	9.58E-09	RMI2
100.6528	57.46247	65.70333	16.59719	14.69239	16.92617	2.216565	9.61E-09	ISYNA1
259.72	245.7016	239.0871	145.2254	101.7973	114.2517	1.042877	9.61E-09	RTKN
63299.8	65209	62359.76	54359.93	48925.65	45562.08	0.358731	9.75E-09	GAPDH
53.02243	37.64782	60.22805	149.3747	116.4897	152.3355	-1.46698	9.85E-09	NEXN
433.1663	438.8944	405.1705	613.0586	668.5037	659.0628	-0.60372	9.98E-09	HEATR6
123.1199	122.8508	93.99226	44.60494	29.38478	35.96811	1.627418	1.00E-08	2-Mar
450.2413	427.0056	474.524	733.3882	697.8885	635.7893	-0.61235	1.01E-08	P3H2
42.23821	48.54588	52.01513	9.335917	4.197825	3.173657	3.091707	1.01E-08	UGT1A7
2362.644	2383.702	2365.32	3026.912	3262.76	2912.359	-0.37181	1.02E-08	UBFD1
458.3295	429.9778	456.2731	288.3761	273.9081	259.182	0.711086	1.02E-08	GSKIP
141.9923	113.9342	117.7185	45.64226	43.02771	47.60486	1.456069	1.02E-08	TESMIN
1099.991	1111.602	1018.402	1540.426	1431.458	1438.725	-0.44972	1.03E-08	SFSWAP
530.2243	562.7359	574.9041	753.0973	845.8618	917.1869	-0.59323	1.03E-08	NR2C1
310.0464	288.3031	357.7181	533.1846	504.7885	519.4219	-0.70305	1.04E-08	FAM122A
825.8918	810.4189	708.1359	1193.96	1053.654	1153.095	-0.53682	1.04E-08	MRPL22
1632.911	1710.994	1733.838	2164.895	2167.127	2336.869	-0.39332	1.05E-08	AIFM1
984.0604	965.9639	1025.702	699.1565	733.57	643.1945	0.519575	1.05E-08	ZFAND3
2854.224	2841.42	2893.684	3413.834	3950.154	3893.019	-0.39012	1.07E-08	TRIM44
376.5491	381.4319	342.2048	235.4726	191.0011	188.3037	0.839011	1.08E-08	CRY2
69.19877	58.4532	78.47897	14.52254	6.296738	19.04194	2.371976	1.08E-08	INPP5J
2595.403	2567.978	2552.392	2076.723	1982.423	1835.432	0.388347	1.08E-08	ATP6AP1
16.17634	22.78684	17.33838	75.72466	76.61031	69.82045	-1.98492	1.08E-08	AC027228.2
1903.415	2113.232	1833.305	2656.587	2532.338	2573.836	-0.40862	1.08E-08	ANXA2P2
750.4022	683.6052	657.0333	456.4226	469.107	459.1224	0.59495	1.09E-08	COQ4
368.461	414.1261	381.4443	612.0212	579.2999	600.8791	-0.62345	1.09E-08	ERI1
1188.062	1242.378	1314.979	1754.115	1595.174	1744.453	-0.44364	1.10E-08	EMC4
94.36195	91.14736	82.12916	12.44789	32.53315	22.2156	1.994839	1.10E-08	DNAJC15
667.7232	715.3086	707.2233	923.2185	1067.297	1026.149	-0.52947	1.11E-08	FBXO11
2288.053	2065.677	2362.582	2744.76	3245.968	3360.903	-0.47722	1.12E-08	SLC25A36
337.007	312.0806	348.5927	511.4008	505.838	618.8631	-0.71284	1.13E-08	CCDC186
204.0016	189.2298	200.7602	363.0634	330.5787	340.6392	-0.79957	1.14E-08	ZNF720
341.5004	381.4319	375.969	168.0465	211.9902	225.3296	0.860225	1.16E-08	ATP8B3
845.6628	887.696	886.0824	658.7008	536.2722	540.5796	0.593508	1.19E-08	OCIAD2
25.16319	18.82391	18.25092	76.76199	77.65977	79.34143	-1.90671	1.20E-08	ICA1L
218.3805	248.6738	227.224	121.3669	117.5391	107.9043	1.000477	1.20E-08	STARD8
274.099	318.025	271.9388	559.1177	453.3651	455.9487	-0.76633	1.21E-08	-
516.744	570.6617	558.4783	851.6431	802.8341	768.025	-0.55821	1.21E-08	SMIM7
302.8569	340.8119	331.2543	537.3339	495.3434	515.1903	-0.66765	1.21E-08	HEATR5B
3053.733	2979.132	2853.532	2374.435	1868.032	1595.292	0.606058	1.21E-08	MTCH1
98.85538	94.11956	93.99226	25.9331	36.73097	24.33137	1.722024	1.22E-08	FBF1
502.3651	571.6525	592.2425	828.822	830.12	807.1668	-0.56607	1.22E-08	TRAF3IP1

62.90797	65.38832	42.88967	12.44789	7.346194	8.463085	2.596825	1.23E-08	PKIB
186.0279	152.5728	159.6956	300.824	285.4521	351.218	-0.91049	1.23E-08	ZNF286A
711.7587	761.873	736.4248	497.9156	522.6293	430.5595	0.60652	1.25E-08	ATG2A
3900.294	3942.123	3915.736	5174.173	5080.418	4640.944	-0.34128	1.26E-08	ARF3
216.5832	191.2113	201.6727	372.3994	332.6777	356.5075	-0.7998	1.27E-08	HOXA1
38.64347	30.7127	40.15203	105.8071	97.59944	121.6569	-1.56682	1.29E-08	TLK2P1
116.8291	94.11956	123.1937	35.26902	36.73097	41.25754	1.562397	1.29E-08	AC006262.1
570.6652	576.6061	607.7558	835.0459	904.6314	812.4562	-0.54013	1.30E-08	TMX4
2270.978	2380.729	2187.373	2963.635	2860.818	2938.806	-0.35793	1.30E-08	DAP3
319.932	308.1177	395.1325	499.9902	599.2396	701.3782	-0.81462	1.31E-08	GCC2
192.3186	181.304	156.0454	89.20988	52.47282	32.79446	1.600683	1.31E-08	SDSL
302.8569	287.3123	301.1403	180.4944	154.2701	147.0461	0.887404	1.31E-08	BCAS4
333.4122	352.7007	331.2543	187.7557	187.8527	206.2877	0.805792	1.34E-08	R3HDM2
429.5716	407.1909	426.1591	256.2191	262.3641	251.7768	0.713449	1.36E-08	MST1R
343.2978	321.988	354.9805	581.9388	537.3216	501.4378	-0.66723	1.38E-08	PDSS2
140.1949	131.7674	132.3192	54.97818	57.7201	45.48908	1.353641	1.39E-08	AC012146.1
141.9923	208.0538	178.8591	351.6529	389.3483	296.208	-0.97376	1.39E-08	POMK
200.4068	153.5635	135.9694	57.05283	77.65977	46.54697	1.434926	1.40E-08	TRIM62
371.157	307.127	348.5927	203.3155	191.0011	178.7827	0.842099	1.41E-08	MAPK11
176.1423	180.3133	170.6461	282.1522	322.1831	421.0385	-0.95995	1.42E-08	ZNF431
376.5491	373.506	435.2845	577.7895	621.2782	664.3522	-0.65209	1.42E-08	SOS2
329.8175	304.1548	271.9388	157.6733	162.6657	170.3196	0.884843	1.44E-08	LAPTM5
265.1122	281.3679	288.3646	435.6761	460.7113	452.7751	-0.69267	1.45E-08	ZNF41
58.41454	50.52734	39.23949	2.074648	6.296738	9.520971	3.053646	1.45E-08	CMPK2
102.4501	111.9527	113.1557	30.0824	38.82988	42.31543	1.558659	1.45E-08	SDHAF4
322.628	348.7377	287.4521	497.9156	580.3494	497.2063	-0.71731	1.45E-08	MLKL
916.659	969.9268	950.8732	638.9917	638.0695	706.6676	0.516283	1.46E-08	LTBP1
48.52901	51.51807	30.11403	1.037324	2.098913	0	5.370882	1.47E-08	GLIS2
261.5174	275.4235	260.9882	155.5986	137.4788	108.9622	0.988124	1.48E-08	COL17A1
48.52901	50.52734	55.66532	0	2.098913	0	6.205605	1.49E-08	KRT16P6
574.2599	565.7081	552.0905	921.1438	736.7184	943.634	-0.62056	1.50E-08	EEF1B2P3
258.8214	224.8962	180.6842	102.6951	96.54998	106.8465	1.118241	1.50E-08	ROGDI
39.54215	44.58295	59.3155	7.261269	6.296738	5.289428	2.927325	1.54E-08	GPC2
343.2978	346.7563	366.8436	218.8754	174.2098	204.1719	0.823351	1.54E-08	CLCF1
264.2135	282.3587	304.7904	446.0494	452.3157	490.859	-0.70643	1.54E-08	TMEM168
409.8005	412.1446	438.9347	641.0663	641.2178	614.6316	-0.5891	1.58E-08	TRMT1L
471.8098	494.3754	475.4366	336.093	251.8695	252.8347	0.777284	1.60E-08	ADCY7
1917.794	1940.844	1976.575	2426.301	2618.394	2473.337	-0.36555	1.60E-08	HERC4
2803.898	2920.678	2991.327	4051.788	3722.422	3544.975	-0.37715	1.62E-08	UQCRFS1
2028.333	1990.381	2066.005	2540.407	2587.959	2617.209	-0.34804	1.63E-08	TM9SF4
160.8647	159.5079	148.745	253.1071	324.282	306.7868	-0.91437	1.63E-08	PTGES3P1
47.63032	59.44393	49.2775	9.335917	6.296738	9.520971	2.634805	1.64E-08	LINC01615
4615.648	4428.573	4592.845	3652.418	3574.448	3750.205	0.313161	1.64E-08	SAR1A
1145.824	1271.109	1129.732	864.091	836.4167	837.8455	0.482027	1.65E-08	FDPS
3152.588	3100.992	3414.748	3904.488	4299.623	4329.926	-0.37435	1.65E-08	AGO2
1335.446	1387.025	1325.93	1827.765	1764.136	1733.875	-0.39591	1.65E-08	PPRC1
1055.955	1195.814	1089.58	1598.516	1525.91	1458.824	-0.45659	1.66E-08	TEX261
975.9722	1018.473	949.9606	1415.947	1348.551	1273.694	-0.45612	1.66E-08	TEAD4
51.22506	42.60148	49.2775	8.298593	3.148369	7.4052	2.924353	1.67E-08	STBD1
824.9931	866.8907	850.4931	1075.705	1285.584	1293.794	-0.52379	1.68E-08	ARHGAP42
809.7154	856.9833	861.4436	1169.064	1128.166	1179.543	-0.45988	1.69E-08	SEC23B
765.6799	720.2623	735.5123	454.348	428.1782	549.0427	0.634297	1.69E-08	KDM5B
998.4393	1035.315	939.9226	1548.725	1382.134	1285.331	-0.50409	1.69E-08	CTNNBL1
2093.038	2081.528	2245.776	2670.072	2850.323	2764.255	-0.36763	1.72E-08	SMAD5

317.2359	330.9045	321.2163	461.6092	570.9043	536.348	-0.69475	1.73E-08	FANCL
37.74478	50.52734	45.62731	8.298593	1.049456	0	3.830627	1.76E-08	SNAI3-AS1
1157.507	1215.628	1211.861	1618.226	1573.135	1562.497	-0.40734	1.77E-08	GPBP1
79.98299	77.27711	103.1177	15.55986	19.93967	31.73657	1.955371	1.82E-08	MARCOL
56.61717	71.33272	94.90481	183.6064	194.1494	161.8565	-1.27595	1.82E-08	ADSL
18843.63	19955.33	19633.43	16224.79	16165.83	16304.13	0.262966	1.83E-08	APP
75.48956	78.26784	74.82879	26.97043	6.296738	9.520971	2.414509	1.86E-08	ASPHD1
5426.262	5965.198	5951.626	4564.226	4728.85	4416.673	0.33904	1.88E-08	PRRC2B
47.63032	58.4532	44.71476	8.298593	2.098913	9.520971	2.919572	1.88E-08	AC138150.2
35.94741	53.49954	59.3155	0	0	1.057886	7.044205	1.88E-08	FOXO6
472.7085	491.4032	473.6115	330.9064	259.2157	279.2818	0.72523	1.89E-08	ELL
151696.3	166019	156543.7	205645.4	191945.6	188009.6	-0.30425	1.89E-08	EEF1A1
1481.033	1488.08	1564.104	1127.571	1086.187	1166.848	0.423356	1.90E-08	CHD6
105.1462	141.6747	131.4067	248.9578	252.919	244.3716	-0.98234	1.90E-08	ZYG11A
339.703	358.645	308.4406	206.4275	180.5065	176.6669	0.836256	1.91E-08	CASKIN2
873.5221	823.2984	898.858	1229.229	1200.578	1150.98	-0.46379	1.91E-08	OSTM1
458.3295	473.57	509.2008	691.8952	768.202	696.0888	-0.58124	1.92E-08	METTL17
1719.185	1789.262	1734.75	1356.82	1345.403	1274.752	0.398553	1.94E-08	AKIRIN1
148.2831	129.7859	135.0568	63.27677	52.47282	46.54697	1.347896	1.94E-08	ZYX
1260.855	1291.915	1224.637	991.6819	814.3781	823.0351	0.522523	1.95E-08	UNC45A
142.891	122.8508	139.6196	43.56761	47.22554	63.47314	1.395225	1.96E-08	LINC01588
930.1393	1306.776	512.851	135.8895	215.1386	173.4933	2.390154	2.00E-08	RPL41P1
142.891	168.4245	175.2089	67.42607	68.21466	74.052	1.213927	2.01E-08	KIF13B
364.8662	328.9231	396.0451	558.0804	590.8439	560.6794	-0.64857	2.03E-08	DNAL1
18.87239	30.7127	102.2052	0	2.098913	2.115771	5.177122	2.06E-08	-
591.3349	642.9852	599.5429	443.9747	334.7766	339.5813	0.712894	2.07E-08	ZDHHC18
2364.441	2346.054	2357.107	2971.934	2891.252	3059.405	-0.3362	2.08E-08	APEX1
228.2661	233.8128	198.0225	114.1057	108.094	103.6728	1.017763	2.08E-08	TMEM120A
21.56845	34.67563	31.02657	91.28452	107.0445	86.74663	-1.71174	2.09E-08	ST13P4
150.0804	161.4893	111.3306	43.56761	54.57173	59.2416	1.42571	2.10E-08	TMEM92
1939.363	2054.779	1928.21	753.0973	416.6342	332.1761	1.979269	2.10E-08	LRFN4
192.3186	168.4245	150.5701	73.65001	80.80814	67.70468	1.202742	2.11E-08	AMH
712.6574	845.0945	699.0104	520.7367	489.0467	422.0964	0.655438	2.14E-08	DEF8
47.63032	38.63855	30.11403	1.037324	2.098913	4.231543	3.986311	2.16E-08	BX539320.1
978.6683	936.2419	1039.39	1495.821	1295.029	1339.283	-0.48312	2.17E-08	FCF1
454.7348	526.0788	539.3148	779.0304	766.1031	729.9411	-0.5823	2.18E-08	ZSCAN29
296.5661	320.9972	304.7904	465.7585	541.5195	474.9907	-0.68492	2.19E-08	SPIRE1
941.8222	916.4273	1026.614	716.791	651.7124	607.2264	0.546198	2.20E-08	FOXP4
99.75407	77.27711	61.1406	23.85846	19.93967	11.63674	2.102176	2.23E-08	CHST7
616.4981	598.4022	611.406	808.0755	898.3346	957.3865	-0.54424	2.23E-08	GPD1L
93.46327	85.20297	53.84023	19.70916	17.84076	17.98406	2.065211	2.25E-08	TESK2
1200.644	1354.331	1342.355	1664.905	1897.417	1777.248	-0.45453	2.25E-08	ADSS
56.61717	39.62929	47.4524	0	1.049456	0	6.996089	2.26E-08	PHOSPHO1
430.4702	426.0148	392.3949	202.2782	269.7103	249.661	0.79144	2.27E-08	C21orf33
340.6017	324.9602	325.779	476.1318	527.8765	585.0108	-0.68027	2.28E-08	DCUN1D2
1687.731	1567.338	1589.656	2104.731	2173.424	2045.951	-0.38421	2.28E-08	ZFP36L1
196.8121	178.3318	181.5967	336.093	313.7874	322.6551	-0.80416	2.30E-08	MTERF1
549.9954	598.4022	566.6912	410.7804	325.3315	309.9605	0.712639	2.31E-08	MPV17L2
52.12375	35.66636	33.76421	2.074648	4.197825	4.231543	3.535803	2.32E-08	HCAR2
635.3705	677.6608	691.71	989.6072	955.0053	903.4344	-0.50685	2.33E-08	TEX2
249.8345	278.3957	317.5661	148.3374	155.3195	142.8146	0.921637	2.34E-08	ANKRD13B
412.4965	367.5616	395.1325	257.2564	219.3364	203.114	0.790077	2.34E-08	SAT2
1329.156	1329.563	1232.85	1948.095	1830.252	1613.276	-0.47058	2.37E-08	VOPP1
470.0124	533.0139	458.0982	309.1226	230.8804	141.7567	1.098907	2.37E-08	SLC39A3

387.3334	347.747	374.1439	593.3494	538.3711	581.8371	-0.62675	2.39E-08	SH3RF1
449.3426	452.7646	438.0222	300.824	210.9407	261.2978	0.793408	2.42E-08	TOM1
60.21191	66.37906	79.39152	12.44789	14.69239	20.09983	2.12565	2.42E-08	SNED1
66.50271	76.28638	73.91624	21.78381	18.89021	10.57886	2.078068	2.43E-08	AL590666.2
913.0642	994.6951	985.5499	732.3508	634.9211	614.6316	0.5454	2.46E-08	GTPBP3
298.3635	272.4513	329.4292	464.7212	474.3543	534.2323	-0.70962	2.48E-08	FAM126B
714.4548	693.5125	682.5846	1115.123	1092.484	889.6819	-0.56719	2.49E-08	SIAH2
407.1044	446.8202	423.4214	286.3015	241.375	230.6191	0.751585	2.49E-08	TMEM14A
699.1771	722.2438	740.075	535.2593	425.0298	382.9546	0.685933	2.51E-08	TRIM16L
1032.589	973.8897	1026.614	766.5825	597.1407	498.2642	0.703787	2.52E-08	REEP4
131.2081	97.09175	131.4067	52.90353	31.48369	34.91023	1.592662	2.53E-08	SMIM4
957.9985	1038.287	991.9377	751.0227	677.9488	675.9889	0.505039	2.53E-08	NOP10
352.2846	349.7285	393.3074	578.8269	533.1238	590.3002	-0.63571	2.55E-08	ZNF143
17.97371	26.74977	21.90111	72.61269	88.15433	78.28354	-1.84737	2.66E-08	RTN3P1
311.8438	326.9416	301.1403	178.4198	189.9516	142.8146	0.878097	2.67E-08	RFX2
295.6675	293.2567	269.2011	155.5986	155.3195	162.9144	0.856721	2.68E-08	ULBP2
362.1702	360.6265	326.6915	584.0135	577.201	498.2642	-0.66142	2.71E-08	TRMT2B
38.64347	45.57368	32.85166	0	1.049456	5.289428	4.214794	2.71E-08	SCN4A
1248.274	1289.933	1310.416	867.203	944.5107	985.9494	0.46019	2.72E-08	AFF1
278.5924	231.8313	235.4369	490.6543	425.0298	380.8388	-0.79701	2.75E-08	PRMT6
40.44084	45.57368	42.88967	6.223945	5.247282	4.231543	3.035535	2.75E-08	ITGA2B
461.9242	414.1261	389.6572	865.1283	602.3879	666.468	-0.75354	2.75E-08	FTH1P8
410.6992	439.8851	457.1857	628.6184	660.108	667.5259	-0.58113	2.78E-08	NSL1
677.6087	558.7729	653.3831	863.0537	953.9558	1002.876	-0.57638	2.79E-08	FAM177A1
4057.564	4272.037	4194.062	3494.745	3147.32	3222.32	0.344214	2.79E-08	TAF7
219.2792	240.7479	233.6118	374.474	397.744	379.781	-0.73248	2.80E-08	LCORL
878.0155	903.5477	1077.717	1408.686	1423.063	1282.157	-0.52468	2.80E-08	LIN7C
94.36195	106.9991	94.90481	40.45564	25.18695	27.50503	1.667714	2.80E-08	PRRG4
12296.71	12798.28	11672.38	16744.49	14348.17	15952.92	-0.35569	2.80E-08	RPS24
471.8098	477.5329	475.4366	729.2389	706.2841	661.1785	-0.55755	2.82E-08	DYM
1057.753	1017.482	1012.014	1433.582	1339.106	1375.251	-0.42598	2.85E-08	RNF14
176.1423	194.1835	158.783	291.4881	334.7766	325.8288	-0.84862	2.86E-08	MPC1
33.25136	49.53661	31.93912	3.111972	3.148369	0	4.188945	2.95E-08	SLC22A18
1203.34	1134.388	1270.264	1559.098	1658.141	1627.028	-0.42464	2.96E-08	ERAP1
45.83295	56.47173	42.88967	6.223945	7.346194	8.463085	2.719886	2.97E-08	CBS
45.83295	38.63855	45.62731	131.7402	142.7261	93.09394	-1.4969	2.97E-08	THAP10
703.6706	863.9185	778.4019	1189.811	1086.187	1107.606	-0.52929	2.98E-08	INO80
1195.251	1277.054	1210.949	1602.666	1698.02	1591.06	-0.40971	2.99E-08	GID8
1838.71	1920.039	1859.769	1497.896	1303.425	1131.938	0.514204	2.99E-08	NDUFA8
677.6087	752.9565	740.9875	514.5128	486.9477	457.0066	0.573761	3.00E-08	RETREG3
39.54215	37.64782	75.74134	129.6655	147.9733	169.2617	-1.54429	3.01E-08	CSNK2A3
839.3721	803.4838	861.4436	1071.556	1220.518	1221.858	-0.48827	3.02E-08	NOV
486.1887	496.3568	471.7864	337.1303	209.8913	180.8985	0.997869	3.03E-08	DOHH
341.5004	377.469	396.0451	568.4536	569.8548	575.4898	-0.62045	3.04E-08	SRP19
326.2228	364.5894	337.6421	498.9529	537.3216	585.0108	-0.65726	3.08E-08	TMEM131L
102.4501	106.9991	102.2052	40.45564	35.68152	31.73657	1.529829	3.10E-08	-
189.6226	217.9611	214.4484	98.54579	111.2424	92.03605	1.042671	3.11E-08	GALT
1211.428	1234.452	1232.85	963.6741	830.12	755.3304	0.528951	3.12E-08	TSPAN17
120.4238	97.09175	109.5055	198.1289	219.3364	256.0083	-1.04041	3.14E-08	BCLAF3
237.2529	262.544	249.1251	385.8846	453.3651	405.1702	-0.73321	3.15E-08	SDHAF3
1278.829	1296.868	1315.892	1629.636	1876.428	1722.238	-0.42595	3.19E-08	NFRKB
1119.762	1195.814	1072.242	868.2403	753.5097	758.504	0.508754	3.20E-08	WDR13
3110.35	3005.881	3113.608	3752.001	3924.967	3803.099	-0.31461	3.23E-08	RAB10
1957.337	2047.843	1969.275	1590.218	1524.86	1426.03	0.395506	3.24E-08	ESYT1

36.8461	38.63855	36.50185	1.037324	0	3.173657	4.738096	3.31E-08	MGAT2
3573.173	3827.198	3717.713	3037.285	2732.784	2379.185	0.447992	3.32E-08	NCS1
33.25136	22.78684	32.85166	90.2472	110.1929	84.63085	-1.67715	3.34E-08	-
1341.737	1224.545	1414.447	1743.742	1810.312	1785.711	-0.42321	3.38E-08	NDFIP2
316.3372	327.9324	349.5052	215.7634	147.9733	167.1459	0.904131	3.42E-08	HIC2
36.8461	53.49954	37.41439	6.223945	1.049456	5.289428	3.343432	3.44E-08	AC133644.2
416.99	383.4134	426.1591	594.3867	660.108	607.2264	-0.60131	3.47E-08	NAMPTP1
1108.079	1203.74	1037.565	523.8487	364.1614	273.9924	1.527084	3.54E-08	TIMM17B
65.60403	52.50881	52.01513	148.3374	131.182	136.4673	-1.28848	3.55E-08	FAM92A1P1
263.3148	246.6923	245.4749	407.6684	447.0684	388.244	-0.71804	3.59E-08	ZMAT3
108.7409	109.9713	113.1557	37.34367	48.27499	28.56291	1.538941	3.60E-08	F12
341.5004	368.5524	346.7676	552.8938	505.838	582.895	-0.6359	3.67E-08	MRPS31
1083.814	1130.425	1053.078	842.3072	745.114	716.1886	0.503806	3.68E-08	ZNF598
75.48956	87.18443	83.04171	7.261269	24.1375	25.38926	2.114962	3.69E-08	AC080112.1
1086.511	1224.545	1056.729	849.5685	756.658	725.7096	0.529619	3.71E-08	RPUSD3
35.94741	36.65709	44.71476	0	1.049456	2.115771	5.220613	3.74E-08	TBATA
247.1385	264.5255	252.7753	133.8148	139.5777	138.583	0.891532	3.78E-08	BCAS3
934.6327	781.6877	849.5805	1109.937	1283.485	1375.251	-0.55387	3.78E-08	CAPRIN2
459.2282	453.7553	459.9233	269.7043	316.9358	247.5452	0.718786	3.79E-08	ESRP2
60.21191	64.39759	32.85166	9.335917	8.395651	5.289428	2.771462	3.79E-08	INHA
61.1106	69.35125	85.77934	12.44789	12.59348	24.33137	2.132446	3.84E-08	EGFLAM
2259.295	2152.861	2426.46	2812.186	3138.924	3006.511	-0.38913	3.85E-08	PDLIM5
1068.537	1030.361	1137.033	1423.209	1466.091	1448.245	-0.42234	3.92E-08	ZFAND6
18.87239	12.87952	23.7262	58.09015	97.59944	74.052	-2.04464	3.96E-08	SMN2
518.5414	466.6349	509.2008	703.3058	795.4879	717.2465	-0.56774	3.98E-08	C8orf59
373.8531	373.506	351.3303	662.8501	544.6678	535.2902	-0.66597	4.07E-08	FABP5
192.3186	189.2298	198.9351	311.1972	329.5293	362.8548	-0.78942	4.14E-08	KYAT3
164.4594	140.684	156.9579	56.0155	74.5114	66.6468	1.229847	4.19E-08	NMRAL2P
114.133	128.7952	144.1823	221.9874	262.3641	270.8187	-0.9641	4.19E-08	AC024896.1
50.32638	57.46247	43.80222	8.298593	1.049456	10.57886	2.927148	4.21E-08	SSC4D
380.1439	394.3114	356.8056	253.1071	187.8527	200.9983	0.816728	4.29E-08	RABEP2
39.54215	59.44393	31.93912	110.9937	135.3799	125.8884	-1.51273	4.39E-08	AC090833.1
746.8075	821.317	730.9495	539.4086	537.3216	466.5276	0.574426	4.42E-08	PYGO2
289.3767	299.2011	280.1517	459.5346	441.8211	469.7012	-0.65869	4.43E-08	ALG2
82.67905	96.10102	73.91624	22.82113	16.7913	30.67868	1.845613	4.51E-08	AC145098.2
270.5043	268.4884	249.1251	136.9268	138.5282	69.82045	1.189952	4.53E-08	APBA3
52.12375	57.46247	69.35351	116.1803	157.4185	187.2458	-1.36426	4.55E-08	NUP62CL
534.7177	510.2271	473.6115	726.1269	715.7292	799.7616	-0.56174	4.57E-08	TERF2
61.1106	51.51807	90.34208	18.67183	13.64293	5.289428	2.431107	4.57E-08	THAP8
1052.36	1015.5	1145.246	1456.403	1462.942	1415.451	-0.43164	4.58E-08	PPIP5K2
90.76721	91.14736	85.77934	219.9127	182.6054	168.2038	-1.09272	4.58E-08	GPR87
285.7819	270.4699	289.2772	456.4226	444.9695	437.9647	-0.66315	4.62E-08	FAM118B
251.6319	267.4977	276.5015	413.8923	423.9804	441.1383	-0.685	4.67E-08	TIMM23B
2395.895	2528.349	2629.958	1773.824	1946.742	2017.388	0.396779	4.69E-08	PYGL
97.9567	117.8971	104.9428	234.4353	216.188	194.651	-1.00998	4.73E-08	DCLK1
1270.741	1382.071	1309.504	983.3833	987.5384	974.3127	0.427597	4.80E-08	BCL2L13
632.6744	616.2354	584.0296	367.2127	396.6945	435.8489	0.611621	4.86E-08	AL365181.3
120.4238	124.8323	104.9428	225.0993	209.8913	254.9504	-0.979	4.90E-08	PAX9
382.8399	379.4504	324.8665	214.7261	173.1603	224.2718	0.828361	4.92E-08	SIRT3
1201.542	1105.657	1294.903	1497.896	1786.175	1874.573	-0.51768	4.93E-08	DNAJB14
318.1346	333.8767	366.8436	539.4086	487.9972	606.1685	-0.68095	4.94E-08	UBA3
7787.108	7551.361	7919.076	9465.583	9530.113	9123.206	-0.27378	4.94E-08	YWHAB
204.9002	230.8406	213.5358	339.205	365.2108	395.6492	-0.76142	4.98E-08	ZFP62
729.7324	695.494	714.5237	925.2931	1040.011	1218.684	-0.57304	5.04E-08	SYNE2

176.1423	153.5635	154.2203	44.60494	56.67064	86.74663	1.36528	5.06E-08	SERPINB4
13.48028	21.79611	17.33838	60.1648	62.96738	93.09394	-2.04364	5.13E-08	CCDC58P3
41.33952	59.44393	34.67676	7.261269	3.148369	6.347314	3.012315	5.13E-08	NKD2
10921.72	11101.15	10412.15	13405.34	13571.57	17335.57	-0.45016	5.16E-08	RPL11
2057.091	2083.51	2086.081	2591.236	2597.404	2634.135	-0.32924	5.20E-08	WAC
561.6783	500.3197	518.3263	726.1269	780.7955	799.7616	-0.54492	5.26E-08	ZDHHC17
307.3504	321.988	315.741	553.9311	486.9477	460.1803	-0.66793	5.27E-08	TXNL4B
224.6713	294.2475	228.1366	146.2627	98.6489	75.10988	1.221345	5.34E-08	PRR5
59.31323	68.36052	62.05314	13.48521	17.84076	12.69463	2.106956	5.36E-08	GPR173
219.2792	238.7665	285.627	395.2205	447.0684	418.9227	-0.76185	5.46E-08	DLEU2
1032.589	1002.621	1059.466	1313.252	1398.925	1460.94	-0.43103	5.51E-08	PLEKHA5
45.83295	42.60148	46.53986	0	2.098913	0	6.008873	5.52E-08	SEMA5B
2246.713	2235.092	2262.202	2957.411	2870.263	2716.65	-0.34138	5.55E-08	SF3A1
7688.253	8078.43	7831.472	9285.088	9543.756	9621.47	-0.26984	5.57E-08	HDLBP
908.5708	922.3717	897.0329	1214.707	1265.644	1192.237	-0.42911	5.58E-08	EIF3C
3677.42	3703.357	3679.386	4366.097	4619.707	4665.276	-0.30363	5.62E-08	CTNND1
1109.876	1070.981	994.6754	823.6354	677.9488	659.0628	0.555311	5.63E-08	TMEM189
49.42769	41.61075	35.5893	6.223945	2.098913	6.347314	3.109671	5.75E-08	PEX11G
575.1586	704.4106	628.7443	452.2733	384.101	356.5075	0.676897	5.80E-08	CCDC12
1728.172	1757.559	1691.861	1349.559	1290.831	1338.225	0.379886	5.88E-08	HTRA1
111.437	104.0269	97.64245	176.3451	232.9793	240.14	-1.05213	5.89E-08	EFCAB7
281.2885	258.5811	229.0491	146.2627	123.8358	77.22565	1.145877	5.89E-08	FAM173A
211.191	246.6923	254.6004	402.4818	393.5461	386.1283	-0.73121	5.89E-08	BABAM2
367.5623	366.5709	344.9425	573.6402	544.6678	524.7113	-0.60681	5.94E-08	LZIC
227.3674	220.9333	223.5738	125.5162	117.5391	101.557	0.963002	5.95E-08	HEXDC
1079.321	1036.306	1153.458	1400.388	1673.883	1474.693	-0.47632	5.96E-08	CSNK1G3
1332.75	1154.203	1248.363	743.7614	818.5759	970.0812	0.561252	5.96E-08	ARID5B
380.1439	360.6265	367.7561	185.681	239.276	222.156	0.777041	5.97E-08	PIR
1676.947	1844.743	1716.499	1412.835	1113.473	1038.844	0.554744	6.05E-08	ATXN7L3
1394.76	1427.645	1523.04	1786.272	2053.786	1981.42	-0.42177	6.06E-08	PRRC1
46.73163	33.68489	28.28893	2.074648	0	3.173657	4.374764	6.08E-08	-
369.3596	382.4226	323.0414	229.2486	200.4462	158.6829	0.868459	6.13E-08	TAZ
1818.04	1768.457	1799.541	1402.462	1330.711	1413.335	0.377435	6.14E-08	USF2
18.87239	17.83318	30.11403	86.0979	70.31358	83.57297	-1.84175	6.22E-08	PABPC3
355.8794	478.5236	436.1971	685.6713	667.4542	642.1366	-0.65241	6.24E-08	TRIM13
79.98299	69.35125	46.53986	12.44789	15.74185	14.8104	2.187802	6.26E-08	CPT1C
223.7726	228.8591	224.4864	136.9268	99.69835	86.74663	1.065397	6.28E-08	IBA57
308.2491	334.8675	345.855	493.7663	516.3325	518.364	-0.62834	6.37E-08	ZNF28
1011.021	955.0658	1133.382	1351.633	1490.228	1455.651	-0.47096	6.47E-08	RFK
2714.03	2390.637	2598.019	3358.856	3368.755	3176.831	-0.36238	6.54E-08	NDFIP1
2000.473	2050.816	2055.967	1642.084	1573.135	1453.535	0.387334	6.55E-08	C6orf106
352.2846	416.1075	348.5927	226.1367	204.644	224.2718	0.768949	6.63E-08	SLC37A1
2324.899	2498.627	2315.13	2878.574	3157.814	3122.879	-0.3598	6.63E-08	ABLIM1
690.1903	731.1603	768.3639	945.0023	1061	1140.401	-0.52291	6.63E-08	FNIP1
112.3357	70.34198	93.99226	18.67183	31.48369	29.6208	1.796729	6.65E-08	CCDC18-AS1
528.4269	557.7822	515.5886	864.091	793.389	711.9571	-0.56541	6.65E-08	ADORA2B
1695.819	1429.627	1606.081	2004.11	2144.039	2337.927	-0.45448	6.65E-08	ZBED5
74.59088	63.40686	73.0037	148.3374	175.2592	150.2198	-1.16548	6.67E-08	LRRC49
438.5584	383.4134	507.3757	243.7712	237.1771	289.8607	0.787249	6.72E-08	EDN1
263.3148	260.5626	285.627	406.6311	455.4641	437.9647	-0.68304	6.73E-08	SATB2
44.03558	37.64782	32.85166	1.037324	6.296738	0	3.964804	6.74E-08	MRC2
381.9412	397.2836	353.1554	230.286	236.1277	215.8087	0.730548	6.78E-08	HOXC13
344.1965	341.8026	385.0945	214.7261	203.5945	217.9244	0.751736	6.80E-08	ARL3
52.12375	41.61075	46.53986	5.186621	8.395651	8.463085	2.671945	6.84E-08	IL3RA

263.3148	236.785	249.1251	397.2951	401.9418	411.5175	-0.69166	6.88E-08	TTC8
75.48956	60.43466	60.22805	3.111972	13.64293	19.04194	2.457629	6.93E-08	AC008105.3
212.9884	233.8128	246.3875	414.9297	435.5244	339.5813	-0.7801	6.94E-08	ACTR5
4241.795	4568.266	4472.389	6085.981	5527.487	5309.528	-0.34961	6.97E-08	PEBP1
53.02243	51.51807	66.61587	2.074648	5.247282	16.92617	2.824043	7.00E-08	BTBD8
452.0387	528.0603	477.2617	723.0149	779.7461	674.9311	-0.58038	7.03E-08	CXorf38
133.0054	106.9991	114.9808	48.75423	47.22554	38.08388	1.4053	7.03E-08	LINC01232
1117.066	985.7785	1115.131	1459.515	1493.376	1406.988	-0.43752	7.10E-08	SNX4
637.1679	693.5125	582.2045	448.124	408.2385	391.4177	0.615639	7.11E-08	NDUFA2
33.25136	33.68489	42.88967	0	4.197825	0	4.711228	7.18E-08	USH1C
851.055	843.1131	878.782	1073.63	1260.397	1282.157	-0.49081	7.20E-08	RASA1
120.4238	129.7859	129.5816	204.3529	256.0673	290.9186	-0.98434	7.24E-08	ZMYM6
1334.548	1295.878	1316.804	1019.69	992.7857	901.3186	0.43793	7.27E-08	TMBIM1
2619.668	2756.217	2778.703	2012.409	2063.231	2249.065	0.366602	7.29E-08	NBR1
102.4501	132.7581	121.3686	232.3606	225.6331	231.677	-0.95354	7.33E-08	ZNF184
718.9482	848.0667	716.3488	1038.361	1091.435	1111.838	-0.50645	7.36E-08	KMT5B
258.8214	232.8221	249.1251	371.362	407.1891	445.3699	-0.72351	7.36E-08	ZC2HC1A
244.4424	250.6552	255.5129	432.5642	374.6559	413.6333	-0.70189	7.39E-08	HIRA
916.659	971.9083	949.0481	1625.487	1342.255	1203.874	-0.55624	7.40E-08	DHX37
42.23821	42.60148	28.28893	4.149297	3.148369	3.173657	3.431778	7.41E-08	LINC01693
31.45398	22.78684	22.81366	0	0	0	7.05849	7.45E-08	CSPG4P13
2996.217	3339.758	3134.596	2553.892	2421.096	2130.582	0.414228	7.50E-08	LONP1
420.5847	299.2011	359.5432	105.8071	194.1494	198.8825	1.114734	7.51E-08	KRT7-AS
161.7634	185.2669	160.6081	88.17255	65.06629	75.10988	1.151431	7.59E-08	TMEM175
225.57	239.7572	194.3723	347.5036	418.7331	367.0863	-0.78152	7.61E-08	TAPT1
41.33952	48.54588	40.15203	0	1.049456	1.057886	5.953581	7.63E-08	-
1083.814	1318.665	1303.116	1638.972	1717.96	1773.016	-0.46968	7.70E-08	MYCBP2
161.7634	179.3225	147.8325	288.3761	312.738	275.0503	-0.84287	7.81E-08	ADAMTS16
502.3651	527.0695	563.041	829.8593	720.9765	778.6039	-0.5488	7.84E-08	EPC2
219.2792	160.4986	166.996	316.3839	343.1722	330.0603	-0.85422	7.88E-08	ZIC5
273.2003	208.0538	292.0148	415.967	498.4918	430.5595	-0.79677	7.99E-08	SCAI
1059.55	1075.935	1001.063	808.0755	668.5037	561.7373	0.621431	8.03E-08	MEPCE
5.392112	4.953661	8.212916	40.45564	51.42336	37.026	-2.79217	8.08E-08	AC080013.4
2949.485	3107.927	3097.182	3639.97	3874.593	4055.934	-0.33794	8.12E-08	DDX42
44.93426	33.68489	48.36495	5.186621	7.346194	0	3.338713	8.19E-08	PCSK4
635.3705	625.152	643.3451	827.7847	934.0161	936.2288	-0.50272	8.22E-08	SHTN1
987.6551	1019.463	966.3864	759.3213	695.7896	678.1047	0.478797	8.24E-08	C12orf10
1030.792	950.1122	969.1241	1419.059	1353.799	1249.363	-0.44704	8.27E-08	TNPO2
374.7518	452.7646	421.5964	590.2374	634.9211	782.8354	-0.68545	8.33E-08	MNAT1
148.2831	144.6469	109.5055	60.1648	53.52227	42.31543	1.366178	8.47E-08	AK1
322.628	308.1177	298.4026	448.124	506.8874	499.322	-0.64612	8.49E-08	C2orf49
1366.9	1401.886	1319.542	962.6368	1080.94	979.6021	0.435306	8.52E-08	MAST2
620.9915	647.9388	654.2956	880.6882	943.4613	869.582	-0.48624	8.54E-08	AKAP10
54.8198	72.32345	46.53986	13.48521	10.49456	11.63674	2.283636	8.55E-08	KRT8P42
1945.654	1921.03	1951.936	2994.755	2649.877	2327.348	-0.45431	8.56E-08	DDX39A
1694.92	1630.745	1480.15	1270.722	1116.622	1101.259	0.46198	8.60E-08	XBP1
14265.73	14608.35	13784.01	12175.07	10564.88	9517.797	0.403128	8.61E-08	TAGLN2
925.6458	920.3902	941.7477	665.9621	698.9379	641.0787	0.474842	8.62E-08	RDH10
190.5213	242.7294	198.0225	115.143	100.7478	86.74663	1.059032	8.79E-08	MOB3B
166.2568	115.9157	166.0834	265.555	273.9081	352.2759	-0.98976	8.81E-08	AL161891.1
373.8531	431.9592	368.6687	136.9268	53.52227	64.53103	2.203055	8.81E-08	TMEM160
833.9799	793.5765	785.7023	1045.623	1118.72	1140.401	-0.4533	8.85E-08	NGRN
591.3349	618.2169	569.4288	945.0023	854.2575	786.0091	-0.53977	8.85E-08	KLHL18
1323.763	1415.756	1569.579	1821.541	2011.808	2008.925	-0.43909	8.92E-08	UBXN7

87.17247	84.21224	72.09115	20.74648	18.89021	30.67868	1.792532	8.98E-08	CASZ1
250.7332	255.6089	253.6878	425.3029	433.4255	373.4336	-0.69731	8.99E-08	SRRD
675.8113	550.8471	665.2462	876.5389	930.8678	959.5023	-0.5473	9.12E-08	FKBP14
73.69219	61.4254	60.22805	9.335917	11.54402	22.2156	2.182909	9.14E-08	AL158206.1
690.1903	672.7071	700.8355	479.2438	460.7113	487.6853	0.531823	9.18E-08	PSRC1
450.2413	622.1798	508.2882	359.9515	221.4353	183.0142	1.047133	9.26E-08	-
895.0905	939.2141	817.6414	1364.081	1159.649	1211.279	-0.49455	9.32E-08	PRKAG1
102.4501	106.0083	101.2926	240.6592	213.0396	176.6669	-1.02572	9.34E-08	RPSAP19
1326.459	1322.627	1254.751	1020.727	938.214	939.4025	0.429487	9.34E-08	CDK9
3096.869	3395.239	3019.615	1884.818	1483.931	1183.774	1.062956	9.41E-08	ADAM15
446.6466	402.2373	459.0107	588.1628	707.3336	708.7834	-0.61498	9.45E-08	ZNF37A
471.8098	485.4588	491.8624	801.8516	671.6521	682.3363	-0.5733	9.54E-08	RNF216P1
959.7959	1071.972	946.3104	453.3106	348.4195	220.0402	1.543094	9.58E-08	NBL1
4178.887	4471.174	4176.724	6418.962	5518.041	5126.514	-0.4119	9.62E-08	MYL12B
130.3094	154.5542	134.1443	236.5099	271.8092	266.5872	-0.8884	9.65E-08	PLAG1
124.9173	143.6562	131.4067	49.79156	57.7201	59.2416	1.261831	9.72E-08	DAGLA
502.3651	486.4495	507.3757	710.567	697.8885	738.4042	-0.52063	9.85E-08	EAF1
378.3465	372.5153	415.2085	544.5952	607.6352	617.8052	-0.60163	9.87E-08	PWWP2A
85.3751	110.962	100.3801	22.82113	34.63206	39.14177	1.619086	9.92E-08	NCK1-AS1
418.7873	420.0704	411.5583	288.3761	243.4739	210.5192	0.751739	9.93E-08	ASPSCR1
1833.318	1756.568	1741.138	2404.517	2205.957	2243.776	-0.36253	9.96E-08	NUTF2
983.1617	981.8156	972.7743	1252.05	1394.727	1299.084	-0.4256	1.00E-07	KDSR
420.5847	414.1261	462.6609	645.2156	619.1792	651.6576	-0.56215	1.00E-07	KDELC1
360.3728	336.8489	328.5166	507.2515	507.9369	558.5636	-0.61719	1.01E-07	SNX25
465.519	493.3846	453.5355	290.4508	322.1831	287.7449	0.649193	1.02E-07	PPP1R37
132.1067	138.7025	136.8819	228.2113	251.8695	272.9345	-0.88534	1.03E-07	VDAC1P8
1999.575	2237.073	2070.567	2658.662	2765.317	2654.235	-0.35743	1.03E-07	DIDO1
1242.882	1331.544	1208.211	1874.445	1682.279	1585.771	-0.44352	1.03E-07	GNG11
387.3334	342.7933	377.7941	224.062	209.8913	233.7927	0.731183	1.04E-07	PRICKLE3
212.0897	181.304	197.11	108.919	91.3027	88.8624	1.030819	1.04E-07	ALX4
1530.461	1602.014	1638.02	2005.148	2044.341	2064.993	-0.35817	1.05E-07	AP3M1
632.6744	670.7257	726.3868	956.4129	927.7194	961.6181	-0.48751	1.06E-07	DCP1A
12.58159	2.972197	8.212916	54.97818	53.52227	41.25754	-2.64091	1.06E-07	AP000526.1
117.7278	106.0083	99.46754	215.7634	197.2978	218.9823	-0.96713	1.06E-07	CYP4V2
322.628	290.2845	337.6421	485.4677	504.7885	486.6274	-0.63475	1.07E-07	CWF19L2
762.9838	789.6135	866.0064	1118.235	1166.995	1065.291	-0.47016	1.07E-07	SUCO
738.7193	866.8907	712.6986	527.998	474.3543	309.9605	0.820274	1.08E-07	WDR83OS
242.645	230.8406	247.3	129.6655	129.0831	132.2357	0.882847	1.08E-07	TWIST1
240.8477	232.8221	281.9768	135.8895	139.5777	129.0621	0.901932	1.08E-07	ABCC10
193.2173	136.721	156.9579	68.46339	75.56086	68.76257	1.195612	1.08E-07	AL049840.4
67.4014	63.40686	44.71476	152.4866	129.0831	143.8725	-1.27815	1.09E-07	RWDD4P2
79.0843	76.28638	49.2775	14.52254	15.74185	19.04194	2.053313	1.10E-07	BAIAP2L2
960.6946	1007.575	901.5957	1378.604	1255.15	1269.463	-0.44415	1.11E-07	DNTTIP1
3396.132	3825.217	3317.106	2895.172	2519.745	2392.937	0.432373	1.11E-07	LMAN2
97.05801	62.41613	91.25462	23.85846	26.23641	23.27348	1.774819	1.12E-07	HIST1H4H
57.51586	92.13809	60.22805	22.82113	10.49456	13.75251	2.153337	1.12E-07	TRAPPC6A
89.86853	53.49954	83.04171	188.793	164.7646	169.2617	-1.20351	1.12E-07	AC115618.2
1390.266	1330.553	1393.458	1698.1	1852.29	1817.448	-0.38342	1.13E-07	TOR1AIP2
448.444	432.95	455.3606	654.5515	672.7015	623.0947	-0.54473	1.13E-07	ILKAP
2417.463	2504.571	2359.845	2020.707	1742.098	1635.491	0.431575	1.14E-07	SAMD4B
86.27379	86.1937	90.34208	29.04508	33.5826	20.09983	1.667077	1.14E-07	ADRA1B
20.66976	20.80538	25.55129	59.12748	85.00596	92.03605	-1.81571	1.15E-07	AC092017.1
157.2699	201.1186	163.3458	87.13523	79.75868	71.93623	1.125699	1.15E-07	KPTN
185.1292	138.7025	151.4827	86.0979	41.97825	39.14177	1.506642	1.16E-07	TPGS1

62.00928	62.41613	69.35351	18.67183	14.69239	15.86829	1.976472	1.19E-07	HOTAIR
265.1122	295.2382	307.5281	410.7804	489.0467	515.1903	-0.70541	1.19E-07	TTC21B
2907.247	2844.392	2915.585	2246.844	2340.288	2358.027	0.319686	1.20E-07	CD46
45.83295	45.57368	40.15203	9.335917	2.098913	6.347314	2.885229	1.23E-07	AQP1
683.0008	709.3642	693.5351	977.1593	936.1151	947.8656	-0.45618	1.23E-07	NSRP1
648.8508	673.6979	683.4971	395.2205	462.8102	482.3959	0.581818	1.23E-07	SIAE
361.2715	412.1446	382.3569	529.0353	616.0309	633.6735	-0.6226	1.24E-07	DPY19L3
509.5546	550.8471	515.5886	369.2874	265.5125	190.4194	0.932727	1.26E-07	SENP3
300.1609	278.3957	270.1137	443.9747	434.4749	451.7172	-0.648	1.27E-07	IFT74
1351.623	1522.755	1460.986	2042.491	1905.813	1813.216	-0.41075	1.28E-07	GALNT10
2572.936	2766.124	2590.719	3312.176	3315.233	3260.404	-0.31865	1.28E-07	ARF4
954.4038	1065.037	960.9112	737.5375	611.833	443.2541	0.732905	1.28E-07	RECQL4
2887.476	3284.277	3129.121	3844.323	3932.313	3979.766	-0.33828	1.29E-07	MBTPS1
233.6582	269.4792	278.3266	368.2501	471.2059	490.859	-0.76778	1.29E-07	TAF3
42.23821	37.64782	48.36495	6.223945	7.346194	5.289428	2.766075	1.29E-07	SCARF2
761.1864	658.8369	735.5123	1031.1	1067.297	947.8656	-0.49831	1.29E-07	DCBLD1
60.21191	64.39759	73.0037	16.59719	13.64293	20.09983	1.973365	1.30E-07	CCDC136
195.0147	179.3225	232.6993	104.7697	92.35216	100.4991	1.029025	1.31E-07	SDR16C5
354.082	364.5894	282.8893	195.0169	168.9625	197.8246	0.833559	1.32E-07	SDHAF1
451.14	465.6441	483.6495	740.6494	691.5917	631.5577	-0.55961	1.33E-07	TMEM268
1130.546	1062.065	1100.531	843.3445	800.7352	692.9151	0.49484	1.34E-07	FHL1
38.64347	66.37906	47.4524	11.41057	7.346194	7.4052	2.539547	1.35E-07	VIPR2
337.9057	377.469	360.4558	534.2219	534.1733	552.2163	-0.59175	1.35E-07	INPP1
507.7572	472.5792	533.8395	696.0445	725.1743	790.2406	-0.54575	1.36E-07	NAPA
106.0449	87.18443	83.95425	16.59719	29.38478	37.026	1.741488	1.36E-07	PECAM1
830.3852	841.1316	876.9569	1138.982	1148.105	1132.996	-0.42435	1.36E-07	RFC5
103.3488	94.11956	93.99226	35.26902	29.38478	38.08388	1.504792	1.37E-07	AC093673.1
110.5383	114.9249	112.2432	202.2782	214.0891	227.4454	-0.93107	1.37E-07	REL
465.519	408.1817	429.8093	230.286	282.3038	283.5134	0.71217	1.38E-07	CCNG2
368.461	283.3494	343.1174	503.1022	613.932	509.9009	-0.70808	1.38E-07	PTBP2
1007.426	1023.426	994.6754	1322.588	1291.881	1408.046	-0.41099	1.39E-07	EMC3
50.32638	56.47173	96.7299	9.335917	10.49456	21.15771	2.314315	1.39E-07	CTIF
34.15004	39.62929	35.5893	96.47114	107.0445	95.20971	-1.45123	1.40E-07	MATR3
3217.293	3230.778	3405.622	4014.444	4019.418	4082.381	-0.29814	1.40E-07	KDM1A
95.26064	80.24931	93.07971	33.19437	16.7913	31.73657	1.717121	1.41E-07	DNAJB5
617.3968	563.7266	612.3185	379.6606	405.0901	418.9227	0.575905	1.43E-07	KIAA0895
76382.86	70973.08	69960.36	102975.2	97291.95	82546.82	-0.38006	1.43E-07	MT-ND4
17.07502	21.79611	25.55129	62.23945	70.31358	102.6149	-1.8685	1.43E-07	CCT8P1
148.2831	151.582	139.6196	240.6592	259.2157	307.8447	-0.87819	1.43E-07	KLHL28
64.70534	65.38832	62.96569	20.74648	15.74185	7.4052	2.134809	1.44E-07	CSPG4P12
45.83295	27.7405	32.85166	4.149297	3.148369	1.057886	3.668915	1.46E-07	PAEP
309.1477	336.8489	388.7447	581.9388	492.195	572.3162	-0.67008	1.46E-07	CDC26
19.77108	21.79611	30.11403	0	0	0	6.953662	1.47E-07	RORC
1026.299	1105.657	1004.713	817.4114	632.8222	539.5217	0.656167	1.47E-07	TOMM7
61.1106	66.37906	66.61587	18.67183	14.69239	16.92617	1.947905	1.47E-07	TLE6
390.9281	389.3577	387.8321	558.0804	585.5966	579.7213	-0.56102	1.48E-07	CNKSR3
66.50271	57.46247	53.84023	117.2176	194.1494	136.4673	-1.33187	1.50E-07	ZC3HAV1L
33.25136	59.44393	41.97713	127.5909	109.1435	124.8305	-1.43002	1.51E-07	SOX7
780.9575	747.0121	791.1776	1115.123	1122.918	980.66	-0.47269	1.51E-07	FAM57A
554.4888	529.051	599.5429	815.3368	768.202	816.6877	-0.51155	1.51E-07	BUD31
42.23821	41.61075	37.41439	5.186621	6.296738	5.289428	2.853923	1.52E-07	AL645608.8
213.8871	231.8313	236.3495	102.6951	118.5886	133.2936	0.944	1.53E-07	ANK3
859.1431	915.4365	743.7252	1231.304	1139.71	1172.137	-0.49321	1.53E-07	ACAA2
1199.745	1140.333	1116.957	1502.045	1639.251	1464.114	-0.41368	1.54E-07	TTC17

696.4811	782.6784	778.4019	568.4536	477.5026	448.5435	0.594547	1.54E-07	TUSC1
287.5793	265.5162	270.1137	148.3374	165.8141	151.2777	0.823097	1.55E-07	TSPYL4
1130.546	1210.675	1181.747	1831.914	1524.86	1509.603	-0.46638	1.56E-07	VPS25
485.2901	497.3476	452.6229	657.6635	702.0863	722.5359	-0.53713	1.57E-07	UEVLD
77.28693	79.25857	76.65388	28.00775	24.1375	13.75251	1.821765	1.57E-07	MFSD14A
523.9335	559.7637	549.3528	322.6078	344.2217	393.5335	0.623012	1.57E-07	MCTP2
655.1416	684.5959	706.3108	910.7706	939.2634	976.4285	-0.46619	1.57E-07	PTPRA
66.50271	73.31418	60.22805	160.7852	134.3304	152.3355	-1.16291	1.58E-07	SLC35A1
13360.75	13852.42	12709.94	20000.65	17330.72	15525.53	-0.40491	1.59E-07	RPS5
151.8778	201.1186	161.5207	90.2472	50.3739	27.50503	1.611648	1.61E-07	TBC1D10A
1135.04	1148.259	1136.12	1610.964	1426.211	1495.85	-0.40685	1.61E-07	HJURP
409.8005	427.9963	427.0716	725.0896	642.2673	565.9688	-0.61247	1.61E-07	WDR4
1792.877	1970.566	1808.667	1011.391	632.8222	585.0108	1.321566	1.61E-07	AURKAIP1
187.8252	166.443	198.0225	101.6578	85.00596	76.16777	1.071547	1.62E-07	-
3608.221	3599.33	3732.314	4251.992	4894.664	4694.897	-0.33934	1.63E-07	USP9X
166.2568	186.2576	156.9579	328.8318	277.0565	291.9764	-0.81881	1.64E-07	TIFA
423.2808	385.3948	385.0945	230.286	245.5728	262.3556	0.693949	1.65E-07	WDR27
879.8129	858.9648	868.744	1142.094	1179.589	1156.269	-0.41541	1.65E-07	SPRED2
616.4981	591.4671	691.71	870.315	1040.011	874.8715	-0.55157	1.66E-07	LPCAT2
1417.227	1390.988	1353.306	1867.183	1727.405	1789.943	-0.37174	1.67E-07	SNX2
695.5824	596.4208	677.1093	395.2205	427.1287	476.0486	0.601666	1.68E-07	LSM8
420.5847	437.9036	438.0222	628.6184	619.1792	632.6156	-0.53661	1.68E-07	FBXO33
180.6357	141.6747	160.6081	70.53804	76.61031	74.052	1.127646	1.68E-07	APBB3
802.526	835.1872	850.4931	1046.66	1256.199	1161.558	-0.47752	1.69E-07	RPRD1A
112.3357	120.8693	105.8554	216.8007	237.1771	197.8246	-0.94389	1.69E-07	E2F5
685.6969	741.0677	661.596	973.01	950.8074	959.5023	-0.46599	1.69E-07	CDKN3
24.2645	20.80538	24.63875	0	0	0	6.913735	1.70E-07	TNFRSF8
44.93426	48.54588	61.1406	109.9564	122.7864	165.0302	-1.36241	1.71E-07	ENSAP2
80.88168	78.26784	124.1063	205.3902	200.4462	203.114	-1.10257	1.72E-07	-
70.09745	50.52734	48.36495	10.37324	15.74185	8.463085	2.289725	1.72E-07	CARD14
580.5507	537.9676	563.041	768.6572	903.5819	769.0829	-0.53745	1.72E-07	SAP130
1190.758	1230.489	1140.683	1688.764	1583.63	1477.866	-0.41568	1.74E-07	NUP62
79.98299	85.20297	71.17861	23.85846	27.28586	21.15771	1.708102	1.75E-07	HEIH
143.7896	119.8786	121.3686	214.7261	266.5619	248.6031	-0.92147	1.77E-07	ZNF254
2133.479	2305.434	2149.046	2992.68	2792.603	2669.046	-0.36019	1.77E-07	PSMA5
43.13689	45.57368	53.84023	6.223945	12.59348	3.173657	2.695879	1.78E-07	RAET1L
345.0951	320.9972	346.7676	531.11	520.5303	486.6274	-0.60237	1.79E-07	HIBCH
129.4107	133.7488	124.1063	61.20212	53.52227	47.60486	1.253625	1.79E-07	LINC02009
451.14	464.6534	507.3757	668.0367	695.7896	697.1467	-0.53406	1.80E-07	MORC3
398.1176	405.2095	391.4823	561.1924	619.1792	583.9529	-0.56247	1.80E-07	KIAA1468
35.94741	33.68489	30.11403	3.111972	1.049456	3.173657	3.765098	1.80E-07	AL049836.1
256.1253	249.6645	244.5624	366.1754	401.9418	464.4118	-0.7157	1.81E-07	PDIK1L
984.0604	921.3809	794.8278	1212.632	1274.04	1289.563	-0.48385	1.82E-07	PSMF1
575.1586	625.152	664.3336	911.8079	874.1971	848.4243	-0.49883	1.84E-07	HIVEP1
1125.154	1159.157	1168.972	1520.717	1479.733	1489.503	-0.37885	1.85E-07	TBC1D5
1157.507	1055.13	1122.432	812.2248	836.4167	815.6299	0.436968	1.86E-07	BTF3L4
1915.098	1829.882	1847.906	1450.179	1496.525	1293.794	0.399393	1.86E-07	ATXN7L3B
813.3102	905.5292	800.303	625.5065	514.2336	582.895	0.547727	1.88E-07	MRPL24
1605.951	1696.133	1631.633	2093.32	2131.446	2045.951	-0.34622	1.89E-07	OXA1L
64.70534	72.32345	58.40296	22.82113	6.296738	12.69463	2.222207	1.90E-07	SLC25A42
231.8608	226.8777	245.4749	368.2501	405.0901	366.0284	-0.69377	1.92E-07	MFSD8
651.5468	593.4486	601.368	457.4599	376.7548	376.6073	0.608751	1.93E-07	TRIM11
337.9057	373.506	346.7676	481.3184	584.5472	575.4898	-0.63378	1.93E-07	OXNAD1
16321.02	16654.21	14919.22	13711.35	11895.59	11109.92	0.38337	1.94E-07	RPL13

138.3975	125.823	151.4827	67.42607	58.76956	51.8364	1.223649	1.96E-07	CD99P1
78.18562	61.4254	73.0037	142.1134	165.8141	158.6829	-1.13172	1.96E-07	GCSHP5
59.31323	52.50881	35.5893	7.261269	11.54402	6.347314	2.550766	1.96E-07	USP2
1508.893	1524.737	1459.161	1032.138	1096.682	1213.395	0.42686	1.96E-07	TRIP10
2013.954	1974.529	1971.1	2368.211	2596.355	2795.992	-0.38082	1.97E-07	UBE2I
124.0186	97.09175	94.90481	40.45564	38.82988	37.026	1.442668	1.97E-07	CCDC78
56.61717	59.44393	48.36495	7.261269	16.7913	2.115771	2.650153	1.99E-07	MYO1G
584.1454	550.8471	608.6683	400.4071	398.7934	375.5494	0.570103	1.99E-07	PARP12
2060.685	1937.872	2004.864	2407.629	2592.157	2818.207	-0.38076	2.01E-07	PHF10
337.007	333.8767	339.4672	213.6888	204.644	192.5352	0.725835	2.02E-07	PROSER3
1546.637	1607.958	1648.058	2077.76	1998.165	2043.835	-0.34973	2.02E-07	RTF2
124.0186	108.9805	110.4181	36.30634	56.67064	30.67868	1.473928	2.02E-07	GSEC
857.3458	912.4643	888.82	1397.276	1109.275	1239.842	-0.49513	2.03E-07	MRPL21
76.38825	86.1937	84.8668	31.11972	19.93967	26.44714	1.673823	2.04E-07	NFATC1
52.12375	56.47173	47.4524	10.37324	12.59348	9.520971	2.263268	2.04E-07	EFNA3
55.71849	71.33272	74.82879	12.44789	22.03858	16.92617	1.973384	2.04E-07	AAMDC
120.4238	106.0083	90.34208	30.0824	33.5826	47.60486	1.510399	2.05E-07	TRABD2B
903.1787	905.5292	859.6185	1301.842	1193.232	1135.111	-0.44434	2.08E-07	YARS2
84.47642	118.8879	72.09115	33.19437	28.33532	23.27348	1.696966	2.08E-07	SPINK4
1645.493	1654.523	1640.758	1341.26	991.7362	847.3664	0.635333	2.08E-07	RPL10P16
638.0665	730.1696	729.1244	430.4895	421.8815	524.7113	0.606816	2.09E-07	BBS2
1483.729	1559.412	1481.975	2163.858	1862.785	1938.047	-0.39878	2.17E-07	HDDC2
337.007	309.1084	295.665	196.0543	170.0119	184.0721	0.775706	2.17E-07	YBX1P1
787.2483	830.2336	848.668	645.2156	467.0081	461.2382	0.647946	2.22E-07	TMEM258
288.478	236.785	254.6004	402.4818	450.2168	408.3439	-0.69214	2.23E-07	SLC25A40
484.3914	491.4032	531.1019	273.8536	369.4086	278.2239	0.709693	2.23E-07	CUL9
55.71849	61.4254	59.3155	14.52254	15.74185	12.69463	2.037622	2.24E-07	FOLR1
144.6883	130.7766	152.3952	234.4353	259.2157	286.687	-0.86572	2.26E-07	TXNDC16
656.9389	689.5496	614.1436	883.8002	910.9281	926.7079	-0.47351	2.28E-07	CWC27
133.0054	106.9991	99.46754	46.67959	44.07717	39.14177	1.38623	2.28E-07	ENO3
190.5213	205.0816	140.5321	92.32185	68.21466	78.28354	1.165491	2.30E-07	AL390719.1
32.35267	50.52734	31.93912	7.261269	2.098913	2.115771	3.31632	2.32E-07	B4GALNT3
59.31323	38.63855	48.36495	13.48521	4.197825	5.289428	2.669569	2.33E-07	-
1677.845	1773.411	1666.309	1292.506	1372.689	1267.347	0.379738	2.35E-07	EHMT1
283.9845	257.5904	261.9008	123.4416	174.2098	131.1778	0.906325	2.36E-07	PALMD
283.0859	272.4513	276.5015	431.5268	411.3869	452.7751	-0.6387	2.38E-07	COX18
417.8887	453.7553	407.9082	233.3979	299.0951	181.9563	0.840231	2.39E-07	FZD7
399.0163	360.6265	381.4443	216.8007	251.8695	233.7927	0.700529	2.39E-07	KLHL29
363.9675	391.3392	383.2694	579.8642	564.6075	540.5796	-0.56606	2.40E-07	PTCD2
748.6048	732.1511	834.0672	1068.444	1086.187	1027.207	-0.45861	2.44E-07	ZC3H12C
1037.981	1111.602	967.299	807.0382	670.6026	561.7373	0.611463	2.46E-07	CYB561
132.1067	152.5728	152.3952	71.57537	48.27499	20.09983	1.641278	2.47E-07	PDF
896.8879	926.3346	952.6983	1192.923	1223.666	1281.1	-0.41365	2.49E-07	NDUFA12
874.4208	764.8452	862.3562	519.6994	597.1407	609.3421	0.536003	2.49E-07	RNF217
660.5337	649.9203	636.9573	340.2423	385.1505	495.0905	0.674464	2.50E-07	PPL
4461.074	4778.301	4764.404	6005.069	5693.301	5544.379	-0.30031	2.54E-07	CDV3
649.7495	604.3466	585.8547	820.5234	867.9004	887.5661	-0.48515	2.55E-07	STK17B
925.6458	983.7971	1035.74	642.1036	743.0151	714.0728	0.488567	2.56E-07	DPYSL2
270.5043	252.6367	226.3115	419.079	426.0793	371.3179	-0.69886	2.57E-07	TADA2B
137.4988	176.3503	156.9579	254.1444	317.9853	282.4555	-0.86161	2.57E-07	ZNF283
339.703	314.0621	337.6421	499.9902	523.6787	479.2222	-0.59962	2.57E-07	ZNF761
900.4827	925.3439	947.223	539.4086	686.3445	678.1047	0.542736	2.57E-07	ETV3
578.7533	553.8193	669.8089	851.6431	926.67	828.3245	-0.53173	2.59E-07	PDE12
32.35267	33.68489	31.93912	4.149297	0	2.115771	3.962702	2.62E-07	3-Sep

137.4988	130.7766	125.0188	50.82888	67.16521	45.48908	1.266451	2.63E-07	TRIM6
653.3442	658.8369	627.8318	462.6466	448.1179	435.8489	0.52651	2.65E-07	ARFRP1
104.2475	87.18443	88.51698	38.38099	32.53315	24.33137	1.555198	2.66E-07	SRPX
3073.504	3207.991	2985.851	2534.183	2453.629	2461.7	0.314807	2.67E-07	TNFRSF10B
2819.176	3010.835	3119.083	2249.956	2470.42	2299.843	0.350171	2.68E-07	AARS
12.58159	33.68489	23.7262	0	0	0	6.916333	2.69E-07	IGSF9B
37.74478	35.66636	31.02657	5.186621	3.148369	2.115771	3.317983	2.71E-07	SBK3
791.7417	874.8165	788.4399	632.7677	523.6787	487.6853	0.577744	2.72E-07	TLE1
488.8848	483.4773	511.9384	659.7382	717.8281	772.2565	-0.5341	2.72E-07	NCOA1
0	3.962929	0.912546	30.0824	36.73097	25.38926	-4.29054	2.74E-07	MMP10
352.2846	315.0528	339.4672	469.9078	583.4977	516.2482	-0.63989	2.74E-07	CEP57L1
265.1122	273.4421	257.338	170.1212	139.5777	113.1938	0.911495	2.79E-07	CTDP1
502.3651	485.4588	451.7104	343.3543	293.8478	259.182	0.683044	2.80E-07	FKRP
2851.528	2860.244	2834.369	3438.73	3677.295	3437.071	-0.30434	2.81E-07	SEC23A
1437.896	1433.589	1323.192	736.5001	517.382	396.7071	1.345455	2.82E-07	APRT
648.8508	631.0964	601.368	450.1987	425.0298	418.9227	0.539632	2.84E-07	VPS18
133.9041	98.08249	111.3306	30.0824	34.63206	56.06794	1.509263	2.85E-07	RNASEL
2696.955	2876.096	2817.943	3702.21	3463.206	3354.555	-0.32643	2.85E-07	CLSTN1
566.1717	572.6432	540.2274	741.6868	787.0923	868.5241	-0.51383	2.86E-07	SH3D19
641.6613	631.0964	577.6418	880.6882	842.7134	852.6559	-0.47751	2.88E-07	STMP1
459.2282	457.7183	477.2617	716.791	694.7401	617.8052	-0.54157	2.90E-07	HSDL1
1215.921	1275.072	1246.538	1620.3	1606.718	1583.655	-0.3644	2.95E-07	CAMKK2
183.3318	167.4337	163.3458	66.38874	90.25325	86.74663	1.079557	2.96E-07	VPS9D1
1246.476	1319.655	1269.352	962.6368	1005.379	912.9553	0.412568	2.96E-07	GTPBP1
223.7726	173.3781	163.3458	309.1226	336.8755	340.6392	-0.8146	2.98E-07	RMC1
263.3148	278.3957	259.1631	395.2205	416.6342	446.4278	-0.6522	2.99E-07	ZNF621
264.2135	270.4699	252.7753	452.2733	391.4472	403.0544	-0.66339	3.05E-07	INTS12
904.0774	933.2697	1036.653	1461.59	1346.452	1202.816	-0.48087	3.06E-07	SNRNP40
1119.762	1132.407	1085.017	1518.643	1439.854	1405.93	-0.38736	3.06E-07	ZDHHC3
45.83295	43.59222	46.53986	1.037324	11.54402	6.347314	2.847456	3.06E-07	ZC3H12D
68.30008	51.51807	62.96569	155.5986	281.2543	103.6728	-1.56281	3.10E-07	GPR89B
303.7556	345.7655	301.1403	199.1662	183.6549	126.9463	0.898026	3.11E-07	CNPPD1
1094.599	1074.944	1110.569	762.4332	812.2792	852.6559	0.434595	3.13E-07	APOL6
1557.422	1640.652	1610.644	1936.684	2087.369	2210.981	-0.37483	3.15E-07	RCOR1
443.9505	365.5802	400.6078	259.331	250.8201	239.0822	0.692416	3.15E-07	CAMTA2
260.6187	204.0908	231.7867	100.6204	120.6875	136.4673	0.962505	3.16E-07	RNF207
834.8786	877.7887	784.7897	1149.355	1138.66	1086.449	-0.4347	3.16E-07	APTX
571.5638	614.254	624.1816	442.9374	395.645	335.3498	0.624167	3.16E-07	RASSF1
1782.093	1840.78	1876.195	1383.79	1463.992	1447.188	0.35653	3.17E-07	GBF1
174.3449	191.2113	195.2849	299.7867	353.6668	301.4974	-0.76822	3.18E-07	FNIP2
81.78036	68.36052	67.52842	17.63451	22.03858	24.33137	1.767216	3.18E-07	AC098613.1
223.7726	263.5348	264.6384	421.1536	396.6945	388.244	-0.68251	3.18E-07	KIAA2026
523.9335	628.1242	499.1628	399.3698	285.4521	300.4395	0.743946	3.19E-07	PGLS
610.2073	582.5505	584.0296	451.236	322.1831	338.5234	0.675973	3.21E-07	SNHG8
623.6876	632.0871	633.3071	456.4226	438.6728	407.286	0.536339	3.21E-07	PRR12
510.4532	612.2725	608.6683	383.8099	346.3206	397.765	0.617795	3.22E-07	PIN4
394.5228	449.7924	434.372	293.5627	251.8695	256.0083	0.67327	3.22E-07	KATNB1
225.57	275.4235	271.9388	158.7106	129.0831	128.0042	0.893451	3.23E-07	GAPDHP1
11234.46	11086.29	10466.91	9548.569	7752.334	7793.444	0.385751	3.25E-07	MT-ND3
1117.066	1190.86	1204.561	1442.918	1685.427	1570.96	-0.42006	3.25E-07	XIAP
8.986853	5.944393	10.03801	42.53029	38.82988	56.06794	-2.45394	3.28E-07	CCDC150P1
344.1965	360.6265	352.2428	506.2142	526.8271	537.4059	-0.57136	3.28E-07	TYW1
151.8778	139.6932	181.5967	243.7712	307.4907	322.6551	-0.8838	3.28E-07	KIN
830.3852	846.0853	954.5233	1146.243	1269.842	1189.063	-0.45423	3.30E-07	XPR1

261.5174	292.266	283.8019	121.3669	153.2206	178.7827	0.885688	3.31E-07	HSPA12A
204.9002	243.7201	202.5853	383.8099	379.9032	325.8288	-0.7441	3.32E-07	RPUSD2
1871.961	1834.836	1802.279	1513.456	1260.397	1084.333	0.513726	3.35E-07	GRK2
18879.58	20147.53	19982.94	17128.3	15120.57	15846.07	0.295039	3.35E-07	UBC
102.4501	102.0454	108.593	42.53029	36.73097	42.31543	1.364814	3.37E-07	ZNF446
75.48956	58.4532	79.39152	15.55986	27.28586	12.69463	1.942563	3.40E-07	CREB3L1
124.9173	158.5171	172.4712	268.667	287.551	269.7608	-0.85822	3.43E-07	RBM45
709.9614	741.0677	716.3488	449.1614	549.9151	489.8011	0.541651	3.47E-07	ATP6V0A1
1359.711	1278.045	1419.922	1635.86	1901.615	1894.673	-0.42047	3.51E-07	AEBP2
1054.158	1138.351	1111.481	774.8811	828.0211	843.1349	0.433579	3.54E-07	NPC1
989.4525	948.1307	995.5879	1321.551	1342.255	1226.089	-0.40706	3.56E-07	CDC42EP3
595.8283	620.1983	566.6912	338.1677	387.2494	439.0226	0.614468	3.58E-07	C2orf68
274.099	263.5348	319.3912	145.2254	139.5777	185.13	0.867686	3.60E-07	YBX1P10
301.0596	329.9138	267.376	450.1987	458.6124	500.3799	-0.65045	3.60E-07	STX18
41.33952	46.56441	59.3155	9.335917	12.59348	3.173657	2.550547	3.61E-07	-
115.9304	156.5357	162.4332	259.331	298.0456	252.8347	-0.89888	3.65E-07	MFHAS1
13762.47	14617.26	13810.47	16626.23	16566.72	16791.82	-0.24465	3.69E-07	CALR
278.5924	289.2938	340.3797	445.0121	492.195	487.6853	-0.64928	3.71E-07	UTP14C
391.8268	352.7007	401.5203	232.3606	244.5233	239.0822	0.67942	3.73E-07	AC073869.1
1171.886	1078.907	1266.614	1484.411	1593.075	1652.417	-0.42674	3.75E-07	GOPC
79.98299	80.24931	67.52842	23.85846	22.03858	25.38926	1.675562	3.79E-07	CASP10
177.041	172.3874	138.707	71.57537	87.10488	63.47314	1.135283	3.79E-07	TMEM198B
123.1199	121.8601	87.60444	34.2317	28.33532	52.89428	1.526583	3.86E-07	DLGAP1-AS1
1424.416	1505.913	1586.918	1969.879	1887.972	1953.915	-0.36361	3.90E-07	DFFA
3089.68	3252.574	3189.349	3860.92	3851.505	3897.251	-0.28468	3.90E-07	TM9SF2
86.27379	81.24004	61.1406	16.59719	20.98913	28.56291	1.790202	3.92E-07	NLGN3
550.8941	563.7266	608.6683	757.2466	897.2852	811.3983	-0.5168	3.98E-07	PTPRG
1071.233	1099.713	1138.858	1466.776	1457.695	1390.062	-0.38252	4.02E-07	FRMD6
52.12375	64.39759	56.57787	125.5162	138.5282	129.0621	-1.18526	4.03E-07	NR5A2
33.25136	32.69416	31.02657	0	0	3.173657	4.944674	4.05E-07	CCR1
35.94741	40.62002	27.37639	0	1.049456	6.347314	3.820092	4.06E-07	ARHGAP4
70.99614	77.27711	94.90481	30.0824	23.08804	24.33137	1.649381	4.08E-07	BORCS6
34.15004	32.69416	32.85166	1.037324	0	0	6.467615	4.08E-07	CASP14
233.6582	229.8499	222.6613	117.2176	108.094	141.7567	0.902796	4.10E-07	BPHL
585.9428	707.3828	667.0713	975.0847	953.9558	863.2347	-0.51115	4.10E-07	GINS1
35.94741	50.52734	31.93912	5.186621	7.346194	0	3.235312	4.11E-07	LDB3
75.48956	72.32345	72.09115	23.85846	24.1375	20.09983	1.691042	4.14E-07	CFAP57
29.65661	33.68489	79.39152	10.37324	3.148369	3.173657	3.093315	4.15E-07	-
660.5337	624.1613	675.2842	872.3896	874.1971	989.1231	-0.48057	4.22E-07	NEDD1
381.9412	422.0519	434.372	631.7304	593.9923	589.2423	-0.55194	4.22E-07	SNX7
719.8469	686.5774	691.71	1053.921	1015.874	874.8715	-0.48895	4.23E-07	ZDHHC9
2194.589	2526.367	2322.43	2992.68	2930.082	3021.321	-0.34508	4.29E-07	PRPF6
29.65661	22.78684	28.28893	73.65001	92.35216	78.28354	-1.59442	4.39E-07	AC122718.1
1042.475	1051.167	1096.881	1305.991	1518.563	1438.725	-0.41804	4.39E-07	ATF7IP
692.8864	716.2994	766.5388	558.0804	450.2168	388.244	0.639331	4.41E-07	UNK
99.75407	86.1937	102.2052	201.2409	203.5945	167.1459	-0.98788	4.47E-07	SLC10A7
82.67905	54.49027	78.47897	19.70916	22.03858	20.09983	1.803868	4.47E-07	IL11RA
25.16319	19.81464	32.85166	85.06058	61.91792	114.2517	-1.74336	4.48E-07	AL391058.1
122.2212	100.0639	111.3306	219.9127	216.188	195.7088	-0.91988	4.50E-07	UBE3D
2563.949	2700.736	2479.388	2174.231	1885.873	1760.322	0.411707	4.51E-07	AP2S1
4169.9	4475.137	4395.735	5132.68	5302.903	5526.395	-0.29171	4.52E-07	CHD4
23.36582	20.80538	18.25092	0	0	0	6.753935	4.52E-07	AC018638.1
47390.37	50290.56	46817.27	42028.22	38565.42	35757.59	0.312544	4.61E-07	EEF2
1591.572	1673.347	1652.621	1298.73	1210.023	952.0971	0.506558	4.62E-07	TRAF7

555.3875	448.8017	441.6724	317.4212	285.4521	305.729	0.67077	4.66E-07	GNPTG
559.8809	463.6627	480.9119	660.7755	776.5977	829.3824	-0.59052	4.67E-07	TRAPPC6B
6501.089	7037.171	6703.565	5402.384	5578.91	5702.004	0.27883	4.67E-07	HEG1
575.1586	552.8286	511.0259	395.2205	357.8646	312.0763	0.62146	4.67E-07	TJAP1
125.8159	79.25857	103.1177	34.2317	34.63206	41.25754	1.486915	4.68E-07	SPINK13
110.5383	147.6191	127.7565	224.062	224.5837	290.9186	-0.94007	4.72E-07	AP4S1
23.36582	14.86098	24.63875	0	0	0	6.765737	4.76E-07	CD52
4333.46	4240.334	4079.994	3587.067	3379.249	3288.967	0.303152	4.77E-07	DUSP4
2155.946	2242.027	1962.887	1731.294	1577.333	1444.014	0.420186	4.80E-07	EHD1
57.51586	44.58295	45.62731	9.335917	4.197825	13.75251	2.438426	4.85E-07	SLC16A13
1534.954	1650.56	1497.488	2170.082	1906.862	2026.909	-0.38268	4.90E-07	PFDN1
308.2491	284.3401	265.5509	152.4866	126.9842	188.3037	0.875793	4.91E-07	MLLT3
226.4687	241.7387	215.3609	360.9888	356.8152	373.4336	-0.67552	4.93E-07	PLCE1
33.25136	53.49954	17.33838	0	4.197825	1.057886	4.308344	4.93E-07	RTP4
35.94741	33.68489	26.46384	3.111972	0	0	4.935691	4.94E-07	PLPPR3
127.6133	117.8971	104.0303	50.82888	52.47282	32.79446	1.360205	4.95E-07	PCSK5
62.90797	39.62929	60.22805	15.55986	9.445107	9.520971	2.237643	4.99E-07	NTN1
334.3109	329.9138	333.0794	463.6839	498.4918	653.7733	-0.69598	5.04E-07	RHEBP2
234.5569	225.8869	237.262	108.919	132.2315	138.583	0.878268	5.04E-07	AC005392.2
1313.878	1416.747	1264.789	1067.407	950.8074	846.3085	0.47958	5.06E-07	SLC35C2
296.5661	367.5616	332.1668	485.4677	616.0309	491.9168	-0.67849	5.06E-07	MSRB3
315.4385	345.7655	363.1934	449.1614	600.289	588.1844	-0.67686	5.10E-07	INPP5F
2478.574	2567.978	2322.43	1393.126	967.5988	803.9931	1.219309	5.15E-07	CAVIN3
176.1423	162.4801	195.2849	326.7571	292.7983	289.8607	-0.76748	5.16E-07	AFAP1L1
1676.048	1666.412	1731.1	2043.529	2181.82	2187.708	-0.33788	5.16E-07	GPBP1L1
530.2243	516.1715	510.1133	709.5297	714.6798	758.504	-0.48765	5.16E-07	LDLRAD3
161.7634	168.4245	161.5207	84.02326	71.36303	84.63085	1.03439	5.17E-07	SPRED3
1278.829	1275.072	1279.39	1015.54	975.9944	902.3765	0.405457	5.20E-07	PLEKHM2
1253.666	1411.793	1340.53	1718.846	1886.923	1675.691	-0.3992	5.20E-07	CAMSAP1
147.3844	147.6191	136.8819	69.50072	64.01684	67.70468	1.101612	5.24E-07	MSX1
717.1509	799.5209	776.5768	519.6994	561.4591	544.8111	0.49574	5.25E-07	ARHGAP26
505.0611	523.1066	548.4403	770.7318	720.9765	724.6517	-0.49146	5.28E-07	THOC5
698.2785	793.5765	740.9875	1019.69	1027.418	989.1231	-0.4441	5.29E-07	PRDM4
514.9467	573.6339	559.3908	726.1269	800.7352	802.9352	-0.49993	5.29E-07	LMBRD1
621.8902	615.2447	667.9838	844.3818	878.395	897.0871	-0.45929	5.31E-07	FRS2
1244.679	1340.461	1285.778	1049.772	908.8292	886.5082	0.44379	5.32E-07	EIF4EBP1
1782.992	2087.473	1840.606	2625.467	2471.47	2369.664	-0.38723	5.32E-07	ZNF622
492.4795	533.0139	513.7635	645.2156	828.0211	823.0351	-0.57723	5.33E-07	AHI1
37.74478	25.75904	30.11403	1.037324	1.049456	4.231543	3.895451	5.34E-07	PRICKLE2
907.6721	960.0195	880.6071	1169.064	1272.991	1216.569	-0.41313	5.40E-07	POLR3A
127.6133	192.202	152.3952	269.7043	355.7657	271.8766	-0.92854	5.40E-07	AC240274.1
51.22506	47.55514	42.88967	10.37324	11.54402	5.289428	2.379235	5.40E-07	ELMO1
396.3202	456.7275	387.8321	630.6931	585.5966	611.4579	-0.55983	5.41E-07	NARS2
456.5321	441.8666	464.486	768.6572	630.7233	624.1525	-0.57015	5.41E-07	TSFM
1730.868	1912.113	1877.108	1414.91	1479.733	1371.02	0.371638	5.44E-07	NOTCH1
80.88168	106.0083	97.64245	204.3529	159.5174	218.9823	-1.03622	5.44E-07	ZNF343
451.14	444.8387	478.1742	334.0184	272.8586	261.2978	0.662332	5.45E-07	PRKRIP1
1149.418	1206.712	1255.664	1673.204	1564.739	1500.082	-0.39172	5.45E-07	FAF1
69.19877	79.25857	16.42583	11.41057	2.098913	4.231543	3.212079	5.49E-07	-
272.3016	248.6738	239.9997	394.1832	387.2494	413.6333	-0.65074	5.51E-07	GPATCH1
1803.661	1886.354	1678.172	1449.142	1348.551	1229.263	0.414397	5.52E-07	RBM42
508.6559	550.8471	556.6532	745.8361	740.9162	775.4302	-0.48544	5.57E-07	SNX13
303.7556	259.5718	308.4406	161.8226	183.6549	157.625	0.793967	5.66E-07	PLAGL1
16.17634	20.80538	23.7262	69.50072	59.81901	77.22565	-1.76755	5.67E-07	AC073073.2

518.5414	533.0139	498.2502	382.7726	298.0456	239.0822	0.751945	5.69E-07	BOK
47.63032	49.53661	31.02657	5.186621	10.49456	3.173657	2.763966	5.73E-07	C17orf100
70.99614	69.35125	62.96569	13.48521	26.23641	5.289428	2.174458	5.80E-07	ACTC1
665.0271	637.0408	637.8698	488.5797	430.2771	416.807	0.538389	5.81E-07	LIMK1
969.6814	1023.426	937.185	741.6868	692.6412	530.0007	0.576568	5.89E-07	RITA1
13.48028	22.78684	31.02657	68.46339	77.65977	84.63085	-1.77904	5.93E-07	CNTF
1177.278	1225.536	1273.002	1498.933	1813.461	1624.912	-0.42565	5.94E-07	SPPL2A
609.3086	628.1242	626.0067	827.7847	838.5156	886.5082	-0.4542	5.95E-07	SYF2
70.09745	63.40686	69.35351	22.82113	20.98913	14.8104	1.79062	5.98E-07	NXNL2
18.87239	15.85171	26.46384	0	0	0	6.726154	5.99E-07	PCK1
281.2885	257.5904	274.6764	460.5719	412.4363	397.765	-0.64292	5.99E-07	OSGEP
592.2336	664.7813	619.6189	903.5093	861.6037	833.6139	-0.47039	5.99E-07	SLC29A1
2830.859	3032.631	3053.38	3574.619	3669.949	3736.452	-0.30052	6.02E-07	NRDC
67.4014	99.07322	57.49041	4.149297	9.445107	28.56291	2.411125	6.02E-07	TNXB
789.9444	738.0955	776.5768	963.6741	1096.682	1088.564	-0.44987	6.05E-07	RRM2B
452.0387	481.4958	468.1362	634.8424	650.6629	755.3304	-0.5422	6.07E-07	TCAIM
314.5398	304.1548	255.5129	432.5642	457.563	477.1064	-0.64542	6.08E-07	PRDM10
160.8647	181.304	156.9579	92.32185	79.75868	61.35737	1.095023	6.13E-07	CATSPER1
256.1253	245.7016	263.7259	161.8226	130.1326	139.6409	0.826854	6.24E-07	MYORG
480.7966	532.0232	547.5277	701.2311	737.7678	787.0669	-0.51286	6.26E-07	CCDC90B
329.8175	319.0158	318.4786	445.0121	552.014	489.8011	-0.61993	6.31E-07	GOLPH3L
147.3844	159.5079	117.7185	274.8909	244.5233	250.7189	-0.86045	6.32E-07	GPR157
224.6713	264.5255	226.3115	149.3747	122.7864	102.6149	0.93158	6.33E-07	HEMK1
479.8979	501.3105	542.965	749.9854	704.1852	706.6676	-0.50359	6.33E-07	ACBD5
628.181	638.0315	628.7443	1023.839	876.296	810.3404	-0.51664	6.33E-07	UBL7
894.1919	895.6219	901.5957	1102.676	1238.358	1284.273	-0.42961	6.34E-07	DDHD2
187.8252	162.4801	186.1594	95.43382	90.25325	85.68874	0.983842	6.35E-07	PLEKHM1P1
854.6497	917.418	917.1089	1159.728	1227.864	1174.253	-0.40572	6.42E-07	NIPA1
41.33952	47.55514	57.49041	113.0683	114.3907	123.7726	-1.26261	6.44E-07	YWHAZP3
38.64347	33.68489	31.93912	105.8071	81.85759	94.15183	-1.43381	6.48E-07	PKIA
1162.899	1363.247	1264.789	1021.764	799.6857	824.0929	0.518459	6.52E-07	UBXN1
471.8098	514.19	437.1096	657.6635	711.5314	680.2205	-0.52693	6.57E-07	CYTOR
26.06187	46.56441	22.81366	2.074648	0	1.057886	4.921948	6.72E-07	CPAMD8
40.44084	45.57368	39.23949	6.223945	7.346194	8.463085	2.507241	6.75E-07	LINC00239
8.088168	8.91659	5.475277	47.71691	29.38478	52.89428	-2.53533	6.75E-07	AL390039.1
599.4231	534.9954	485.4746	382.7726	335.826	335.3498	0.620114	6.84E-07	AC115223.1
156.3712	162.4801	177.9465	256.2191	285.4521	319.4815	-0.79329	6.85E-07	STXBP4
2094.835	2043.88	2039.541	1729.219	1541.651	1415.451	0.398651	6.86E-07	PNMA1
228.2661	243.7201	235.4369	124.4789	139.5777	133.2936	0.83195	6.90E-07	POMT1
10148.85	9903.359	9560.747	8632.611	7631.647	7117.455	0.340813	6.90E-07	TMSB4X
425.9768	427.0056	441.6724	632.7677	610.7836	604.0527	-0.51305	6.91E-07	TUBGCP4
27452.14	27557.22	28612.89	31707.89	33085.16	33940.15	-0.23964	6.93E-07	ASPH
91.6659	116.9064	113.1557	43.56761	47.22554	33.85234	1.366743	6.97E-07	B3GNT9
584.1454	597.4115	553.9156	869.2776	837.4662	741.5779	-0.49686	6.99E-07	ADO
124.0186	90.15663	105.8554	189.8303	203.5945	230.6191	-0.96104	7.06E-07	AKAP7
27.85924	20.80538	44.71476	112.031	79.75868	95.20971	-1.61572	7.10E-07	SNX18P3
516.744	558.7729	530.1894	959.5248	867.9004	656.947	-0.63016	7.11E-07	TGIF2
1344.433	1430.617	1363.344	1051.847	1098.781	1018.744	0.384579	7.15E-07	SMARCC2
47.63032	117.8971	156.9579	35.26902	24.1375	24.33137	1.943982	7.18E-07	-
882.5089	852.0297	837.7174	664.9248	575.1021	612.5158	0.473494	7.18E-07	AKIP1
2342.873	2561.043	2494.901	3298.691	3178.803	2910.243	-0.34374	7.19E-07	PCMT1
101.5514	113.9342	93.07971	35.26902	48.27499	33.85234	1.393372	7.30E-07	CREB3L4
30.5553	45.57368	26.46384	6.223945	2.098913	1.057886	3.443731	7.31E-07	CERS4
74.59088	84.21224	73.91624	22.82113	24.1375	29.6208	1.603384	7.35E-07	NBR2

1174.582	1092.778	1223.724	1438.769	1659.19	1546.629	-0.41142	7.44E-07	CNOT2
169.8515	202.1094	212.6233	88.17255	91.3027	114.2517	0.992892	7.47E-07	ING4
38.64347	52.50881	50.19004	107.8817	119.638	113.1938	-1.27151	7.50E-07	ZNF449
4368.509	4652.478	4485.165	6072.495	5440.382	5320.107	-0.31782	7.51E-07	PSMB1
88.96984	120.8693	114.0683	201.2409	216.188	202.0562	-0.93737	7.52E-07	ANKRD46
118.6265	136.721	141.4447	67.42607	55.62119	31.73657	1.356752	7.53E-07	MORN1
357.6767	366.5709	359.5432	520.7367	522.6293	538.4638	-0.54566	7.54E-07	FUNDC1
1542.144	1525.728	1545.853	1930.46	2042.242	1874.573	-0.34179	7.56E-07	SDHD
541.0085	533.0139	608.6683	736.5001	830.12	823.0351	-0.50556	7.62E-07	STAU2
271.403	273.4421	254.6004	375.5113	423.9804	459.1224	-0.65481	7.65E-07	AL589743.1
4359.522	4208.63	4560.906	5042.433	5677.559	5650.167	-0.31816	7.67E-07	LMAN1
34.15004	42.60148	46.53986	4.149297	4.197825	10.57886	2.705823	7.67E-07	AXIN2
966.0867	997.6673	834.9798	712.6417	623.3771	633.6735	0.506332	7.68E-07	CRELD2
206.6976	228.8591	229.9616	121.3669	113.3413	130.1199	0.86699	7.69E-07	TMEM107
69.19877	88.17516	105.8554	31.11972	26.23641	31.73657	1.562632	7.75E-07	CPQ
190.5213	171.3967	165.1709	267.6296	309.5896	319.4815	-0.76594	7.79E-07	HNRNPKP4
54.8198	81.24004	74.82879	149.3747	151.1217	154.4513	-1.1118	7.82E-07	DLEU1
14935.25	15845.77	15378.23	18103.38	17903.73	19900.95	-0.27646	7.89E-07	TPT1
84.47642	62.41613	117.7185	207.4648	187.8527	181.9563	-1.12248	7.91E-07	GIN1
72.79351	48.54588	54.75277	154.5613	124.8853	129.0621	-1.21134	7.97E-07	ARMCX5
51.22506	48.54588	68.44097	13.48521	11.54402	15.86829	2.041387	8.02E-07	PPP1R3C
35.94741	37.64782	26.46384	1.037324	6.296738	2.115771	3.405419	8.06E-07	WFDC21P
202.2042	230.8406	230.8742	379.6606	361.013	331.1182	-0.69175	8.20E-07	DHODH
1619.431	1658.486	1653.534	1359.932	1097.731	1225.032	0.421102	8.27E-07	ATP5PD
35.94741	33.68489	23.7262	3.111972	2.098913	3.173657	3.476542	8.28E-07	AATK
658.7363	670.7257	589.5049	458.4973	398.7934	453.833	0.549196	8.31E-07	SGSM2
50.32638	52.50881	53.84023	6.223945	13.64293	14.8104	2.177511	8.36E-07	CSPG4P10
366.6636	416.1075	344.9425	219.9127	258.1663	205.2298	0.72197	8.37E-07	NDUFS7
193.2173	214.9889	168.8211	300.824	329.5293	336.4076	-0.74572	8.37E-07	ZCCHC2
178.8384	225.8869	219.9236	348.5409	332.6777	346.9865	-0.72025	8.40E-07	ETFRF1
214.7858	214.9889	236.3495	113.0683	128.0337	125.8884	0.860457	8.42E-07	KYAT1
242.645	209.0445	180.6842	114.1057	116.4897	93.09394	0.966147	8.43E-07	CROCC
217.4818	225.8869	166.996	340.2423	321.1336	378.7231	-0.76987	8.45E-07	UFSP2
57.51586	50.52734	53.84023	16.59719	12.59348	9.520971	2.063332	8.46E-07	AL121832.2
12.58159	33.68489	15.51329	0	0	0	6.735421	8.51E-07	AC026336.3
1482.831	1518.792	1398.933	1198.109	1067.297	943.634	0.455228	8.64E-07	NACC2
734.2259	651.9018	655.2082	427.3775	465.9586	508.843	0.542413	8.68E-07	LOX
496.973	518.1529	527.4517	343.3543	355.7657	351.218	0.55441	8.70E-07	MPI
2032.826	2325.248	2137.183	1770.712	1636.102	1598.465	0.375504	8.72E-07	SNX12
1203.34	1358.294	1219.162	1003.092	906.7303	887.5661	0.43402	8.73E-07	ATP13A1
3148.095	3242.666	3205.775	2649.326	2667.718	2486.031	0.298356	8.75E-07	FKBP9
564.3744	537.9676	595.8927	788.3663	819.6254	754.2725	-0.4757	8.80E-07	FBXO38
472.7085	460.6905	459.9233	289.4134	341.0733	269.7608	0.630164	8.80E-07	ANKRD13D
722.543	751.9657	754.6757	971.9727	992.7857	1010.281	-0.41652	8.84E-07	RAD54L2
41.33952	66.37906	42.88967	9.335917	13.64293	7.4052	2.306837	8.90E-07	RASD2
430.4702	453.7553	452.6229	301.8613	211.9902	299.3816	0.716899	9.02E-07	FADS2
169.8515	159.5079	146.0074	76.76199	81.85759	74.052	1.030755	9.03E-07	FAM98C
403.5097	443.848	430.7218	623.4318	574.0526	653.7733	-0.535	9.04E-07	C1orf174
15.27765	7.925857	10.95055	46.67959	52.47282	51.8364	-2.13713	9.08E-07	AKAP5
263.3148	222.9147	187.072	122.4042	107.0445	121.6569	0.939578	9.25E-07	STX11
319.932	296.2289	356.8056	453.3106	559.3602	498.2642	-0.63411	9.43E-07	PIAS2
292.9714	325.9509	290.1897	189.8303	187.8527	166.0881	0.740602	9.44E-07	COL27A1
58.41454	48.54588	46.53986	8.298593	14.69239	11.63674	2.149622	9.45E-07	SLC9A5
11380.05	11426.11	11312.84	13507	13887.46	12930.54	-0.24112	9.49E-07	PODXL

1110.775	1047.204	1106.006	1383.79	1394.727	1740.222	-0.46896	9.60E-07	FAM192A
369.3596	395.3021	375.969	269.7043	205.6934	227.4454	0.69796	9.64E-07	CMC1
243.5437	237.7757	239.9997	386.9219	367.3097	368.1442	-0.63774	9.68E-07	ZNF778
70.09745	85.20297	79.39152	15.55986	24.1375	32.79446	1.695579	9.72E-07	RALY-AS1
237.2529	196.165	216.2735	117.2176	108.094	125.8884	0.888455	9.75E-07	CCDC130
1303.992	1391.979	1214.599	1052.884	892.0379	764.8513	0.528765	9.80E-07	RAB32
5959.182	6230.715	5550.106	5121.269	3901.879	4225.195	0.421085	9.84E-07	RPL36
933.734	889.6775	935.3599	1129.646	1330.711	1249.363	-0.42695	9.93E-07	SKAP2
2159.541	2121.158	2149.959	2494.765	2909.093	2783.297	-0.34828	9.97E-07	PALLD
36.8461	40.62002	37.41439	6.223945	8.395651	2.115771	2.77691	1.00E-06	HAR1A
97.9567	20.80538	41.06458	405.5937	412.4363	411.5175	-2.94156	1.01E-06	KIAA1143
131.2081	135.7303	130.4941	61.20212	67.16521	51.8364	1.140614	1.01E-06	CACNB1
1547.536	1322.627	1346.006	1144.169	926.67	833.6139	0.53772	1.01E-06	PITX1
18.87239	12.87952	26.46384	0	0	0	6.654933	1.02E-06	GOLGA6L7
1420.821	1341.451	1268.439	1011.391	1022.17	1040.96	0.390776	1.02E-06	PDLIM1
519.4401	440.8758	464.486	188.793	313.7874	305.729	0.818546	1.02E-06	-
394.5228	410.1631	354.0679	273.8536	223.5342	187.2458	0.758375	1.03E-06	PYCR2
36.8461	48.54588	39.23949	6.223945	10.49456	4.231543	2.570872	1.03E-06	BASP1-AS1
337.007	336.8489	293.8399	224.062	171.0614	162.9144	0.793505	1.03E-06	FBXO41
649.7495	640.013	706.3108	891.0614	951.8569	883.3345	-0.44945	1.04E-06	RP2
158.1686	194.1835	184.3343	306.0106	314.8369	281.3976	-0.75075	1.04E-06	RAB36
30.5553	32.69416	25.55129	1.037324	0	0	6.299946	1.04E-06	VPS37D
11902.19	11063.51	11260.82	8470.789	8746.169	10048.86	0.328088	1.04E-06	RPL28
26.96056	26.74977	32.85166	0	3.148369	1.057886	4.367231	1.04E-06	ETV7
632.6744	599.393	636.9573	901.4347	836.4167	823.0351	-0.45407	1.04E-06	PTS
42.23821	44.58295	21.90111	87.13523	123.8358	100.4991	-1.52096	1.04E-06	AL365273.1
1514.285	1494.024	1569.579	2086.059	2006.561	1813.216	-0.36743	1.05E-06	YIPF5
59.31323	45.57368	75.74134	7.261269	18.89021	15.86829	2.106771	1.06E-06	MYLK3
88.07116	106.0083	78.47897	42.53029	25.18695	15.86829	1.702578	1.06E-06	AC005077.4
133.0054	141.6747	167.9085	70.53804	68.21466	69.82045	1.085565	1.07E-06	SDK1
35.04873	25.75904	34.67676	4.149297	1.049456	4.231543	3.340892	1.07E-06	AKR1B15
227.3674	197.1557	239.0871	130.7028	96.54998	48.66274	1.265627	1.07E-06	ACADS
57.51586	75.29565	69.35351	28.00775	8.395651	6.347314	2.237815	1.08E-06	DMBX1
79.98299	90.15663	103.1177	43.56761	25.18695	17.98406	1.653826	1.08E-06	ITPKA
765.6799	819.3355	706.3108	591.2748	421.8815	326.8867	0.773345	1.09E-06	HYAL2
1000.237	1054.139	1034.827	1449.142	1255.15	1382.657	-0.40404	1.09E-06	PDZD11
156.3712	216.9703	176.1214	85.06058	97.59944	88.8624	1.015504	1.09E-06	PER3
943.6195	926.3346	981.8997	1180.475	1305.524	1270.521	-0.39723	1.10E-06	FRYL
2067.875	2156.824	2006.689	2534.183	2576.415	2605.572	-0.30855	1.10E-06	QRICH1
867.2313	823.2984	876.9569	1039.399	1220.518	1257.826	-0.45388	1.11E-06	STS
9184.564	10170.86	9379.15	14128.35	11842.07	11284.47	-0.37473	1.11E-06	RPS27A
159.966	126.8137	167.9085	49.79156	77.65977	74.052	1.175833	1.12E-06	AC087741.1
440.3558	469.6071	467.2237	735.4628	619.1792	636.8472	-0.53254	1.13E-06	MRPS14
43.13689	40.62002	30.11403	1.037324	3.148369	9.520971	3.059255	1.13E-06	MTUS2
72.79351	3.962929	57.49041	2.074648	2.098913	3.173657	4.195276	1.13E-06	-
36.8461	48.54588	40.15203	10.37324	3.148369	7.4052	2.582201	1.14E-06	-
1133.242	990.7322	1085.93	1365.119	1429.36	1422.856	-0.39315	1.14E-06	ELK4
19.77108	20.80538	15.51329	0	0	0	6.598902	1.14E-06	CEBPE
110.5383	122.8508	103.1177	312.2346	184.7043	200.9983	-1.05372	1.15E-06	LSM14A
2154.149	2176.639	2187.373	2806.999	2742.229	2553.736	-0.31406	1.15E-06	MTHFD2
591.3349	641.0037	709.0484	932.5544	883.6422	881.2188	-0.47459	1.16E-06	NAPG
57.51586	67.36979	44.71476	20.74648	4.197825	5.289428	2.483576	1.16E-06	LIME1
310.9451	344.7748	282.8893	201.2409	189.9516	160.7986	0.764834	1.18E-06	TPST1
36.8461	44.58295	55.66532	7.261269	12.59348	6.347314	2.387289	1.18E-06	FBXO27

150.9791	182.2947	151.4827	255.1817	350.5184	266.5872	-0.84888	1.18E-06	HELQ
34.15004	22.78684	30.11403	3.111972	1.049456	2.115771	3.793213	1.18E-06	AL391056.1
150.9791	198.1464	177.9465	285.2641	299.0951	309.9605	-0.76444	1.19E-06	WDYHV1
106.0449	88.17516	79.39152	42.53029	16.7913	8.463085	2.010942	1.19E-06	TSPAN10
115.0317	170.4059	147.8325	251.0324	267.6114	265.5293	-0.85821	1.20E-06	AL078621.1
608.4099	638.0315	506.4632	419.079	314.8369	380.8388	0.652457	1.21E-06	FAM207A
282.1872	283.3494	292.9273	453.3106	459.6619	397.765	-0.61059	1.21E-06	PNPO
502.3651	603.3559	584.9421	808.0755	758.7569	825.1508	-0.50137	1.23E-06	FAM216A
105.1462	108.9805	114.0683	43.56761	39.87934	52.89428	1.267657	1.23E-06	URB1-AS1
1270.741	1341.451	1283.04	1896.229	1795.62	1528.645	-0.42278	1.23E-06	GEMIN4
1442.39	1442.506	1522.127	1788.347	1936.247	1869.284	-0.3439	1.24E-06	C6orf89
41.33952	43.59222	37.41439	81.94861	115.4402	114.2517	-1.34957	1.24E-06	-
20.66976	14.86098	20.07602	0	0	0	6.588251	1.24E-06	-
99.75407	68.36052	83.95425	29.04508	31.48369	26.44714	1.536611	1.26E-06	DNAH1
195.0147	202.1094	210.7982	126.5535	100.7478	76.16777	1.001586	1.27E-06	KCNK5
69.19877	70.34198	59.3155	12.44789	16.7913	26.44714	1.837539	1.29E-06	SARM1
276.7951	297.2197	323.0414	429.4522	440.7717	532.1165	-0.64446	1.29E-06	IKZF5
1639.202	1671.365	1800.454	2055.976	2359.178	2172.897	-0.36614	1.30E-06	CREB3L2
20.66976	10.89805	12.77565	33.19437	81.85759	83.57297	-2.15773	1.30E-06	-
799.8299	842.1224	907.9835	664.9248	565.657	466.5276	0.587097	1.31E-06	BCL7B
4387.382	4677.247	4705.088	5430.392	5540.08	5595.157	-0.26678	1.31E-06	NCAPD2
150.0804	179.3225	154.2203	96.47114	64.01684	50.77851	1.193117	1.32E-06	CTU1
856.4471	952.0936	906.1584	1170.102	1212.122	1190.121	-0.39658	1.32E-06	NKAPD1
244.4424	206.0723	232.6993	356.8395	369.4086	356.5075	-0.66304	1.32E-06	BBS10
336.1083	326.9416	325.779	220.95	206.7429	180.8985	0.700051	1.32E-06	SUOX
87.17247	81.24004	79.39152	39.41832	15.74185	20.09983	1.717847	1.33E-06	-
1744.348	1835.827	1575.055	1415.947	1173.292	1014.512	0.516193	1.33E-06	PRELID1
1009.224	946.1492	937.185	1207.445	1314.969	1273.694	-0.39191	1.33E-06	ENTPD4
80.88168	93.12882	96.7299	171.1585	174.2098	177.7248	-0.95081	1.34E-06	NSUN3
910.3682	869.8629	857.7934	1122.385	1161.748	1174.253	-0.39045	1.35E-06	HPS3
807.9181	801.5023	761.9761	617.2079	553.0635	481.338	0.521553	1.35E-06	FAM83G
27.85924	37.64782	36.50185	96.47114	86.05542	87.80451	-1.40836	1.36E-06	TXK
55.71849	48.54588	55.66532	15.55986	8.395651	14.8104	2.044986	1.36E-06	AC016876.1
69.19877	85.20297	62.96569	150.412	173.1603	136.4673	-1.08409	1.36E-06	FAM86C1
75.48956	68.36052	83.95425	23.85846	24.1375	28.56291	1.574305	1.36E-06	ITGA11
749.5035	786.6413	748.2879	606.8346	492.195	467.5855	0.543708	1.37E-06	FN3KRP
71.89482	56.47173	46.53986	14.52254	17.84076	12.69463	1.957046	1.37E-06	HAGLROS
2044.509	2102.334	1979.313	1654.532	1662.339	1507.487	0.344425	1.38E-06	RNPEP
612.0047	601.3744	573.9916	431.5268	415.5847	406.2281	0.51194	1.38E-06	HAGH
1150.317	1136.37	1130.645	1337.111	1656.042	1629.144	-0.4356	1.38E-06	VPS13A
168.0541	163.4708	153.3078	81.94861	90.25325	59.2416	1.066364	1.38E-06	KLHL22
119.5251	120.8693	101.2926	206.4275	222.4847	197.8246	-0.87589	1.39E-06	ULK4
22.46713	36.65709	27.37639	1.037324	2.098913	0	4.780608	1.39E-06	C11orf96
389.1307	376.4782	368.6687	544.5952	526.8271	567.0267	-0.53041	1.39E-06	ACVR1B
38.64347	53.49954	30.11403	3.111972	10.49456	0	3.164941	1.40E-06	IRF7
41.33952	46.56441	38.32694	12.44789	4.197825	4.231543	2.592117	1.41E-06	TNFSF13
13227.75	13347.14	12646.98	15314.02	15111.12	15651.42	-0.2324	1.41E-06	KRT18
171.6489	194.1835	141.4447	60.1648	95.50053	78.28354	1.116058	1.42E-06	LZTR1
730.6311	743.0491	765.6263	1170.102	1072.544	906.608	-0.49211	1.42E-06	MCTS1
815.1075	886.7053	820.3791	652.4769	603.4374	553.2742	0.47876	1.43E-06	NFKB2
763.8825	770.7896	695.3602	1012.428	972.846	1004.991	-0.4235	1.43E-06	PHF23
23.36582	26.74977	50.19004	1.037324	5.247282	3.173657	3.409921	1.43E-06	RTL9
53.92112	52.50881	53.84023	19.70916	7.346194	8.463085	2.171644	1.44E-06	LRRC75B
98.85538	103.0361	91.25462	186.7183	216.188	163.9723	-0.95217	1.44E-06	GDAP1

181.5344	186.2576	186.1594	112.031	78.70923	92.03605	0.969613	1.44E-06	RASSF8-AS1
647.0534	687.5681	678.0218	930.4798	885.7412	890.7397	-0.42793	1.44E-06	RIPK2
433.1663	491.4032	515.5886	708.4924	671.6521	673.8732	-0.51265	1.45E-06	COG2
1939.363	2234.101	2191.936	2550.78	2851.373	2785.413	-0.36343	1.46E-06	TRRAP
255.2266	276.4143	250.0377	441.9001	374.6559	398.8229	-0.63758	1.46E-06	GEMIN6
3140.905	3412.082	3473.151	3876.48	4310.117	4634.597	-0.3548	1.46E-06	MACF1
24.2645	47.55514	26.46384	0	0	2.115771	5.546918	1.47E-06	NACAD
112.3357	89.1659	96.7299	39.41832	28.33532	45.48908	1.39815	1.47E-06	HOXC4
132.1067	141.6747	150.5701	214.7261	268.6608	269.7608	-0.8276	1.47E-06	C18orf54
2436.336	2764.143	2601.669	2188.754	1906.862	1689.443	0.431238	1.47E-06	CAPN1
1114.37	1255.258	1144.333	949.1516	756.658	625.2104	0.591653	1.47E-06	SSNA1
3886.814	3903.485	4022.504	3387.901	3192.446	3041.421	0.295939	1.48E-06	HDAC1
5852.239	5804.7	5757.254	3960.504	3195.595	2600.283	0.835816	1.49E-06	CD81
2031.927	1899.234	1834.218	2403.48	2449.431	2363.317	-0.32371	1.50E-06	TMEM165
61.1106	80.24931	51.10259	18.67183	17.84076	17.98406	1.818389	1.51E-06	SRGAP3
3092.376	3142.602	2943.874	2618.206	2267.875	2384.474	0.336097	1.51E-06	TRIR
418.7873	376.4782	426.1591	544.5952	639.1189	605.1106	-0.54958	1.51E-06	GFOD1
677.6087	740.0769	721.8241	937.741	1047.357	926.7079	-0.44502	1.52E-06	PRKX
139.2962	122.8508	141.4447	57.05283	66.11575	64.53103	1.105487	1.53E-06	LINC02434
999.338	1096.741	965.4739	817.4114	706.2841	665.4101	0.48332	1.53E-06	AKR7A2
965.188	1008.565	1003.801	801.8516	666.4048	560.6794	0.553052	1.55E-06	DNPEP
65.60403	72.32345	51.10259	24.89578	12.59348	10.57886	1.972638	1.55E-06	CA11
87.17247	14.86098	116.8059	11.41057	7.346194	13.75251	2.753393	1.56E-06	RUFY1
115.9304	101.0547	88.51698	39.41832	46.17608	34.91023	1.342182	1.57E-06	HIST1H2BC
469.1137	511.2178	543.8775	683.5966	769.2515	708.7834	-0.5042	1.58E-06	APOOL
38.64347	32.69416	36.50185	5.186621	5.247282	6.347314	2.685277	1.58E-06	-
728.8338	715.3086	769.2765	904.5466	1059.951	1086.449	-0.46263	1.60E-06	TAB3
243.5437	248.6738	229.9616	135.8895	152.1712	122.7147	0.813632	1.60E-06	DUSP10
6263.836	6007.8	6107.672	7015.423	7428.052	7989.153	-0.28743	1.61E-06	CAST
438.5584	376.4782	396.0451	283.1895	252.919	230.6191	0.659836	1.61E-06	FBXO46
645.256	563.7266	629.6569	357.8768	428.1782	449.6014	0.574173	1.61E-06	CPEB4
175.2436	152.5728	167.9085	92.32185	85.00596	66.6468	1.022973	1.62E-06	DMTN
211.191	255.6089	239.9997	348.5409	389.3483	381.8967	-0.66489	1.63E-06	GPHN
966.0867	1009.556	963.6488	745.8361	728.3227	740.52	0.408108	1.63E-06	TFAP2C
48.52901	53.49954	35.5893	9.335917	7.346194	11.63674	2.280262	1.63E-06	LINC01569
28.75793	29.72197	25.55129	1.037324	2.098913	3.173657	3.738439	1.64E-06	AC092720.1
661.4324	602.3652	595.8927	874.4642	813.3287	857.9453	-0.45268	1.64E-06	DERA
51.22506	54.49027	55.66532	114.1057	115.4402	130.1199	-1.15637	1.65E-06	AP001486.2
70.09745	53.49954	49.2775	15.55986	11.54402	17.98406	1.939964	1.66E-06	ZNF503-AS2
10307.02	10706.84	10475.12	12568.22	12263.95	12073.65	-0.22906	1.66E-06	NONO
82.67905	82.23077	63.87824	33.19437	13.64293	21.15771	1.748954	1.67E-06	MX1
451.14	411.1539	535.6646	630.6931	710.4819	746.8673	-0.57788	1.68E-06	ZBTB41
203.1029	167.4337	156.9579	291.4881	311.6885	287.7449	-0.75529	1.68E-06	-
81.78036	59.44393	76.65388	20.74648	25.18695	24.33137	1.634541	1.68E-06	STAG3L5P
323.5267	321.988	306.6155	212.6514	198.3472	163.9723	0.727228	1.69E-06	KIAA0556
1693.123	1668.393	1501.139	2214.687	2051.687	1994.114	-0.36471	1.70E-06	ADIPOR1
365.7649	320.9972	348.5927	249.9951	159.5174	177.7248	0.818098	1.71E-06	ALDH16A1
15963.35	16248.01	16105.53	18227.86	18929.04	19226.01	-0.22274	1.71E-06	MSN
2650.223	2841.42	2791.479	3546.611	3468.453	3225.493	-0.30626	1.72E-06	ICMT
209.3937	178.3318	214.4484	115.143	98.6489	108.9622	0.900589	1.74E-06	NOG
148.2831	186.2576	177.9465	96.47114	71.36303	84.63085	1.020309	1.75E-06	PLEKHO2
2205.374	2207.351	2454.749	2778.991	2889.153	2919.764	-0.32236	1.76E-06	YIPF6
28.75793	27.7405	26.46384	2.074648	0	1.057886	4.722456	1.76E-06	THPO
106.9435	106.0083	105.8554	181.5317	203.5945	199.9404	-0.87575	1.76E-06	AC009948.1

68.30008	65.38832	70.26606	30.0824	12.59348	12.69463	1.879284	1.79E-06	LBH
545.502	584.532	545.7026	757.2466	758.7569	776.4881	-0.45261	1.80E-06	AGO1
35.94741	35.66636	32.85166	1.037324	3.148369	8.463085	3.050831	1.80E-06	NRG2
1374.988	1461.33	1348.743	1918.012	1863.834	1656.649	-0.37834	1.82E-06	ALDH3A2
244.4424	220.9333	227.224	119.2923	149.0228	117.4253	0.844836	1.83E-06	LHPP
312.7425	334.8675	299.3152	177.3824	203.5945	200.9983	0.701976	1.83E-06	PAQR5
1568.206	1745.67	1718.325	2015.521	2186.018	2226.849	-0.3535	1.84E-06	NSD3
62.00928	68.36052	74.82879	14.52254	27.28586	20.09983	1.729398	1.86E-06	TMOD1
846.5615	837.1687	775.6643	629.6558	600.289	541.6375	0.472997	1.86E-06	MAP3K3
184.2305	201.1186	226.3115	108.919	99.69835	119.5411	0.898438	1.88E-06	ATP8A1
339.703	281.3679	299.3152	458.4973	477.5026	453.833	-0.59353	1.88E-06	MT-TP
790.843	871.8443	878.782	591.2748	653.8113	601.9369	0.460211	1.89E-06	LATS2
554.4888	508.2456	540.2274	398.3325	349.469	341.6971	0.557281	1.90E-06	PANX2
3069.909	3310.036	3123.646	2670.072	2555.426	2487.089	0.300999	1.91E-06	AP3D1
295.6675	354.6821	313.9159	216.8007	167.913	190.4194	0.744475	1.91E-06	STK16
156.3712	163.4708	157.8705	232.3606	306.4413	285.6291	-0.78739	1.91E-06	IL1B
135.7015	155.545	105.8554	255.1817	225.6331	244.3716	-0.87078	1.91E-06	ZNF586
1059.55	1131.416	1022.964	864.091	761.9053	739.4621	0.441719	1.92E-06	S100A4
1625.722	1643.625	1504.789	1324.663	1155.451	1152.038	0.394152	1.95E-06	PELP1
321.7293	298.2104	291.1022	423.2282	528.926	448.5435	-0.62021	1.95E-06	ANKRD42
1527.765	1547.524	1457.336	2044.566	1801.917	1943.336	-0.35335	1.96E-06	RPL6P27
5938.512	6130.651	6122.273	5173.135	5179.067	4929.747	0.251364	1.96E-06	ACLY
191.42	190.2206	211.7107	344.3916	353.6668	281.3976	-0.72291	1.97E-06	ROR1
1306.688	1333.526	1335.055	1676.316	1664.438	1652.417	-0.32899	1.98E-06	COX11
68.30008	68.36052	65.70333	14.52254	29.38478	8.463085	1.94963	1.98E-06	-
927.4432	869.8629	1035.74	1173.214	1322.315	1361.499	-0.44457	1.98E-06	FMNL2
33.25136	25.75904	21.90111	1.037324	1.049456	2.115771	4.270268	1.99E-06	ADAMTS4
1354.319	1521.765	1388.895	1815.317	1879.576	1761.38	-0.3559	2.00E-06	TM7SF3
462.8229	413.1353	421.5964	586.0881	642.2673	623.0947	-0.51222	2.02E-06	TCTN1
375.6504	351.7099	356.8056	231.3233	234.0288	234.8506	0.631063	2.03E-06	DIS3L2
268.7069	240.7479	277.4141	408.7057	404.0407	391.4177	-0.6129	2.04E-06	SLC25A16
35.94741	42.60148	36.50185	4.149297	3.148369	10.57886	2.688052	2.05E-06	JMJD1C-AS1
3860.752	3642.922	3804.405	4417.963	4745.642	4523.519	-0.2753	2.06E-06	GOLPH3
124.9173	110.962	106.7679	51.86621	38.82988	55.01006	1.234066	2.06E-06	IRX4
33.25136	40.62002	32.85166	6.223945	5.247282	5.289428	2.66911	2.06E-06	CAMK2N2
30.5553	27.7405	21.90111	2.074648	1.049456	1.057886	4.258278	2.07E-06	EBF4
94.36195	100.0639	84.8668	186.7183	198.3472	155.5092	-0.9538	2.08E-06	-
601.2205	615.2447	732.7746	871.3523	928.7689	919.3027	-0.48004	2.08E-06	TBC1D23
389.1307	393.3207	386.9196	230.286	267.6114	261.2978	0.623308	2.08E-06	ABHD11
902.28	855.9926	862.3562	659.7382	653.8113	499.322	0.531545	2.08E-06	ELFN2
402.611	364.5894	309.3532	221.9874	234.0288	154.4513	0.818138	2.09E-06	C1orf159
78.18562	89.1659	89.42953	23.85846	24.1375	41.25754	1.525213	2.09E-06	FAM122C
408.9018	369.5431	411.5583	610.9839	566.7064	543.7532	-0.53203	2.09E-06	MYNN
923.8485	892.6497	871.4816	723.0149	607.6352	528.9428	0.53131	2.10E-06	RPS19BP1
164.4594	164.4615	162.4332	91.28452	81.85759	79.34143	0.960282	2.11E-06	CRYL1
30.5553	35.66636	31.02657	1.037324	5.247282	5.289428	3.073618	2.11E-06	TRPV4
324.4254	396.2929	379.6192	512.4381	563.5581	554.3321	-0.56811	2.11E-06	DCAF17
746.8075	752.9565	775.6643	568.4536	514.2336	570.2004	0.461172	2.14E-06	FIS1
377.4478	370.5338	392.3949	280.0775	206.7429	215.8087	0.698461	2.15E-06	CTBP1-AS2
310.9451	262.544	260.9882	164.9345	144.825	177.7248	0.776186	2.18E-06	MIR100HG
164.4594	168.4245	129.5816	80.91128	68.21466	74.052	1.05035	2.18E-06	MTMR11
254.3279	250.6552	277.4141	383.8099	417.6836	393.5335	-0.61064	2.20E-06	ZBTB18
38.64347	31.70343	36.50185	2.074648	9.445107	2.115771	2.971438	2.22E-06	ST6GALNAC2
3255.937	3324.897	3417.486	3804.905	4291.227	4750.965	-0.36163	2.24E-06	DST

34.15004	34.67563	31.02657	6.223945	5.247282	1.057886	2.990758	2.25E-06	AL355001.2
194.116	185.2669	147.8325	94.3965	64.01684	95.20971	1.055422	2.25E-06	AGFG2
561.6783	502.3012	535.6646	264.5177	290.6994	415.7491	0.720946	2.26E-06	PCMTD1
46.73163	65.38832	60.22805	19.70916	10.49456	14.8104	1.934829	2.26E-06	AC002310.6
337.007	318.025	366.8436	474.0571	497.4423	550.1006	-0.57359	2.26E-06	CEPT1
32.35267	37.64782	32.85166	81.94861	118.5886	77.22565	-1.43486	2.26E-06	DNAH5
72.79351	81.24004	84.8668	37.34367	14.69239	22.2156	1.684411	2.27E-06	CCDC69
62.00928	63.40686	78.47897	15.55986	27.28586	19.04194	1.720949	2.28E-06	PRSS16
1484.628	1543.561	1551.329	1125.497	1194.281	1249.363	0.359622	2.29E-06	SUDS3
53.92112	57.46247	65.70333	19.70916	13.64293	16.92617	1.816078	2.30E-06	LRRC20
85.3751	96.10102	97.64245	34.2317	30.43423	43.37331	1.369388	2.32E-06	AC018638.2
58.41454	42.60148	69.35351	15.55986	6.296738	17.98406	2.098047	2.33E-06	C10orf91
25.16319	38.63855	29.20148	2.074648	4.197825	4.231543	3.146543	2.34E-06	CAMSAP3
35.04873	38.63855	35.5893	75.72466	105.9951	99.44125	-1.36416	2.37E-06	-
581.4494	582.5505	580.3794	403.5191	439.7222	359.6811	0.536041	2.37E-06	CYP26B1
788.147	831.2243	843.1927	1043.548	1063.099	1160.501	-0.40795	2.37E-06	RARS2
301.9583	359.6358	315.741	467.8332	463.8597	562.7952	-0.61364	2.38E-06	ZNF33A
1142.229	1198.786	1098.706	943.965	805.9825	758.504	0.455067	2.38E-06	MICALL1
1038.88	1069.991	1022.964	865.1283	608.6847	530.0007	0.643911	2.39E-06	TPRA1
88.96984	74.30491	80.30407	29.04508	22.03858	34.91023	1.503181	2.42E-06	AL109615.3
26.06187	33.68489	21.90111	1.037324	0	3.173657	4.280725	2.42E-06	HBB
10.78422	17.83318	7.30037	35.26902	54.57173	72.99411	-2.1887	2.43E-06	-
1013.717	1006.584	1106.006	1249.976	1443.002	1488.445	-0.41924	2.43E-06	TMTC3
2426.45	2656.153	2441.061	2148.298	1806.114	1635.491	0.428323	2.47E-06	APH1A
204.0016	168.4245	167.9085	246.8831	357.8646	356.5075	-0.83002	2.47E-06	TMEM260
156.3712	159.5079	179.7716	85.06058	86.05542	84.63085	0.954815	2.47E-06	DGAT2
540.1099	559.7637	530.1894	825.71	708.383	735.2305	-0.47774	2.49E-06	PODXL2
204.0016	219.9425	199.8476	121.3669	115.4402	112.1359	0.837402	2.51E-06	LRFN3
3477.912	3713.264	3579.006	4378.545	4479.08	4179.706	-0.27579	2.51E-06	FKBP4
787.2483	980.8249	868.744	1350.596	1140.759	1174.253	-0.47611	2.52E-06	ZNF263
38.64347	27.7405	30.11403	4.149297	5.247282	3.173657	2.941106	2.53E-06	NYAP1
187.8252	235.7943	174.2963	112.031	92.35216	106.8465	0.940465	2.54E-06	H1F0
2517.217	2657.144	2785.091	3225.041	3216.584	3330.224	-0.296	2.56E-06	ALYREF
61.1106	35.66636	35.5893	4.149297	9.445107	10.57886	2.456636	2.56E-06	CORO6
98.85538	96.10102	94.90481	32.15705	45.12662	39.14177	1.316444	2.56E-06	CRIP1
2561.253	2565.996	2373.533	2876.5	3298.441	3359.845	-0.34618	2.56E-06	SEC61G
44.03558	60.43466	66.61587	17.63451	6.296738	16.92617	2.06512	2.57E-06	SCAMP5
629.9784	664.7813	640.6074	492.729	453.3651	418.9227	0.503228	2.58E-06	PYM1
1241.084	1132.407	1137.945	919.0692	905.6808	825.1508	0.406288	2.60E-06	GSTK1
284.8832	182.2947	217.186	108.919	122.7864	123.7726	0.946672	2.63E-06	CSAD
576.0573	590.4764	558.4783	798.7396	738.8173	819.8614	-0.45084	2.64E-06	GLT8D1
395.4215	348.7377	310.2657	207.4648	230.8804	213.6929	0.693703	2.64E-06	DMRTA2
185.1292	191.2113	166.996	95.43382	105.9951	83.57297	0.93028	2.64E-06	CALHM2
695.5824	660.8184	690.7975	1017.615	945.5602	844.1928	-0.45543	2.65E-06	TBC1D13
346.8925	325.9509	296.5775	484.4304	511.0852	450.6593	-0.57706	2.65E-06	SLC25A33
279.4911	262.544	252.7753	144.1881	153.2206	173.4933	0.755624	2.68E-06	TTC28
832.1826	712.3364	673.4591	578.8269	452.3157	430.5595	0.601596	2.68E-06	LIF
194.116	183.2855	194.3723	295.6374	333.7271	293.0343	-0.68944	2.70E-06	ZNF407
51.22506	44.58295	32.85166	13.48521	4.197825	2.115771	2.695686	2.71E-06	AC138932.1
1474.743	1567.338	1659.009	1923.199	2095.764	1966.609	-0.34854	2.71E-06	HIPK3
67.4014	54.49027	86.69189	16.59719	15.74185	28.56291	1.778493	2.72E-06	DNAH2
208.495	213.9982	183.4218	125.5162	102.8467	97.32548	0.894777	2.73E-06	BIN3
822.297	879.7702	836.8049	1290.431	1123.968	1044.133	-0.44642	2.74E-06	EED
140.1949	145.6376	156.0454	233.3979	241.375	285.6291	-0.78288	2.74E-06	METTL1

57.51586	54.49027	61.1406	13.48521	19.93967	15.86829	1.813382	2.77E-06	BLACAT1
927.4432	1001.63	855.9684	507.2515	355.7657	263.4135	1.305729	2.77E-06	TSSC4
545.502	547.8749	550.2654	802.8889	764.0042	699.2624	-0.46349	2.78E-06	RMDN3
97.9567	122.8508	84.8668	47.71691	31.48369	39.14177	1.367089	2.78E-06	ZBTB42
110.5383	120.8693	147.8325	204.3529	264.463	227.4454	-0.87617	2.78E-06	BNC2
78.18562	84.21224	78.47897	38.38099	23.08804	14.8104	1.656851	2.80E-06	DOK1
2654.716	2369.831	2492.164	1877.557	1970.879	2097.787	0.338462	2.82E-06	SH3BGRL3
623.6876	572.6432	537.4897	754.1346	792.3395	887.5661	-0.48908	2.83E-06	USP42
673.1153	716.2994	615.9687	524.886	418.7331	407.286	0.569295	2.84E-06	MGST3
123.1199	166.443	129.5816	280.0775	251.8695	221.0981	-0.84778	2.85E-06	SYTL3
646.1547	726.2067	649.7329	518.6621	459.6619	429.5016	0.521612	2.86E-06	LDOC1
121.3225	159.5079	134.1443	202.2782	281.2543	280.3397	-0.88181	2.88E-06	PTGR2
3769.086	3890.605	3920.299	3217.779	3293.194	3076.332	0.272364	2.90E-06	KDM5C
466.4177	428.987	369.5812	249.9951	299.0951	250.7189	0.661435	2.91E-06	OPHN1
365.7649	301.1826	343.1174	152.4866	215.1386	221.0981	0.779936	2.92E-06	PABPC1L
1334.548	1348.386	1273.002	1653.495	1714.812	1622.797	-0.3355	2.92E-06	NUP214
504.1624	600.3837	562.1285	733.3882	849.0102	766.9671	-0.49604	2.93E-06	GPSM2
269.6056	239.7572	251.8628	381.7353	390.3978	386.1283	-0.60477	2.99E-06	FAM222B
9.885538	1.981464	6.387824	37.34367	51.42336	28.56291	-2.66851	3.00E-06	AC108463.2
1183.569	1262.193	1231.937	1009.316	887.8401	890.7397	0.399279	3.00E-06	DBNL
736.9219	839.1502	769.2765	546.6698	487.9972	611.4579	0.510341	3.00E-06	HERC6
159.966	155.545	156.9579	218.8754	295.9467	319.4815	-0.81988	3.01E-06	FUT10
24.2645	36.65709	18.25092	1.037324	0	0	6.132758	3.02E-06	CNGB1
201.3055	190.2206	205.3229	304.9733	308.5402	335.3498	-0.66818	3.03E-06	PPP2R3C
2504.636	2577.885	2606.232	3094.338	3121.083	3071.042	-0.27244	3.05E-06	SEC31A
44.93426	55.481	51.10259	105.8071	111.2424	123.7726	-1.17111	3.08E-06	FLG
303.7556	242.7294	260.9882	422.1909	397.744	424.2122	-0.62241	3.13E-06	ING2
731.5298	729.1789	616.8812	537.3339	447.0684	390.3598	0.595237	3.15E-06	BAK1
2607.086	2224.194	1895.358	1385.865	987.5384	990.181	0.999855	3.17E-06	RPS28
1067.638	1284.98	1114.219	935.6664	723.0754	579.7213	0.630559	3.18E-06	LLGL1
103.3488	148.6098	107.6805	51.86621	56.67064	44.4312	1.231227	3.18E-06	DNAJC4
264.2135	227.8684	259.1631	374.474	404.0407	376.6073	-0.61955	3.19E-06	ZNF529
253.4292	246.6923	239.0871	383.8099	387.2494	356.5075	-0.60919	3.20E-06	ARAP2
846.5615	869.8629	893.3827	662.8501	656.9597	634.7314	0.417024	3.22E-06	GATD1
615.5994	591.4671	600.4554	840.2326	838.5156	768.025	-0.43674	3.24E-06	TMEM19
764.7812	768.8082	777.4894	935.6664	1066.248	1108.664	-0.42847	3.24E-06	RBM12B
22.46713	23.77757	30.11403	1.037324	0	0	6.083011	3.27E-06	-
66.50271	61.4254	66.61587	21.78381	16.7913	23.27348	1.65382	3.28E-06	ATP2A1
592.2336	568.6803	653.3831	371.362	463.8597	399.8808	0.555237	3.28E-06	NFIA
247.1385	305.1455	301.1403	469.9078	462.8102	393.5335	-0.63707	3.29E-06	SLC25A15
790.843	803.4838	803.9532	527.998	542.5689	646.3681	0.482332	3.29E-06	TRIM38
7110.398	7905.052	6791.169	6335.976	5342.782	5002.741	0.386378	3.29E-06	RPL31
60.21191	48.54588	49.2775	15.55986	14.69239	11.63674	1.915755	3.29E-06	LINC00601
150.9791	140.684	146.0074	69.50072	79.75868	68.76257	1.005747	3.32E-06	HOXD9
521.2375	578.5876	536.5772	730.2762	718.8776	838.9033	-0.48407	3.33E-06	PARP2
50.32638	53.49954	70.26606	137.9641	159.5174	102.6149	-1.20003	3.33E-06	MCTS2P
32.35267	27.7405	21.90111	4.149297	1.049456	1.057886	3.706796	3.34E-06	AL731577.2
8.088168	29.72197	15.51329	0	0	0	6.522865	3.35E-06	MSMB
796.2352	745.0306	754.6757	963.6741	1047.357	1025.091	-0.40278	3.39E-06	SOCS6
1086.511	1152.222	1162.584	1403.5	1467.14	1464.114	-0.35001	3.39E-06	PGM3
38.64347	31.70343	36.50185	98.54579	79.75868	92.03605	-1.33736	3.40E-06	NPM1P6
299.2622	285.3309	278.3266	185.681	183.6549	128.0042	0.794663	3.43E-06	VPS16
1120.661	1131.416	1188.135	879.6509	897.2852	878.0451	0.373864	3.43E-06	CNTROB
1912.402	2107.287	2071.48	2594.348	2572.218	2420.442	-0.31711	3.43E-06	KIRREL1

2253.004	2493.673	2595.281	2874.425	3183.001	3321.761	-0.35336	3.44E-06	BBX
653.3442	683.6052	735.5123	853.7178	1004.33	987.0073	-0.45699	3.44E-06	ANKRD13C
186.9265	231.8313	258.2506	378.6233	363.1119	352.2759	-0.69302	3.45E-06	FAM172A
966.0867	1063.056	1060.379	755.172	760.8559	810.3404	0.409085	3.47E-06	OST4
94.36195	89.1659	89.42953	23.85846	45.12662	32.79446	1.424023	3.48E-06	CDKL5
14.37896	13.87025	20.07602	50.82888	59.81901	58.18371	-1.80247	3.49E-06	IER3
754.8956	879.7702	728.2119	605.7973	529.9755	523.6534	0.508996	3.52E-06	SPAG7
1355.217	1431.608	1444.561	1702.249	1772.532	1899.963	-0.34513	3.53E-06	RABGAP1
41.33952	55.481	54.75277	9.335917	17.84076	8.463085	2.087629	3.55E-06	TAX1BP3
476.3032	454.7461	489.1248	269.7043	266.5619	367.0863	0.653272	3.55E-06	GAGE2A
6644.879	7583.064	7298.545	8567.26	8865.807	8630.231	-0.27605	3.56E-06	AP2B1
622.7889	604.3466	619.6189	779.0304	860.5542	854.7716	-0.43339	3.56E-06	VPS37A
2353.657	2505.562	2565.167	3074.629	2985.703	3009.685	-0.28896	3.60E-06	NELFCD
35.04873	31.70343	41.97713	7.261269	5.247282	6.347314	2.52802	3.62E-06	SOCS1
18374.52	18818.96	17230.7	23277.55	21133.95	21094.24	-0.26741	3.63E-06	RACK1
90.76721	72.32345	64.79078	25.9331	23.08804	29.6208	1.535777	3.63E-06	NME2
19971.48	21364.15	18071.15	24767.15	23377.69	24491.11	-0.29011	3.64E-06	MT-ND5
160.8647	161.4893	119.5436	86.0979	54.57173	57.12583	1.158493	3.66E-06	FDXR
5595.215	5479.74	5782.805	6593.232	6611.575	6663.622	-0.23698	3.67E-06	SRSF6
3995.555	4316.62	4061.743	5766.485	5271.419	4680.086	-0.34528	3.69E-06	MCM7
26.96056	23.77757	28.28893	3.111972	1.049456	2.115771	3.652844	3.72E-06	ACTG2
439.4571	430.9685	484.562	314.3092	316.9358	265.5293	0.595565	3.74E-06	RAB40C
392.7255	386.3855	350.4177	223.0247	253.9684	253.8926	0.628048	3.74E-06	ZBTB46
591.3349	623.1705	549.3528	421.1536	369.4086	431.6174	0.52887	3.77E-06	MYL6B
947.2143	1055.13	876.0444	1313.252	1324.414	1205.99	-0.41794	3.78E-06	TVP23B
2013.055	2207.351	2140.833	2727.125	2690.806	2497.668	-0.3157	3.80E-06	UBTF
914.8616	879.7702	963.6488	1200.184	1304.474	1136.169	-0.40021	3.82E-06	SPPL3
695.5824	740.0769	688.0598	553.9311	498.4918	446.4278	0.502181	3.83E-06	COMMD5
1460.364	1465.293	1444.561	1893.117	1817.658	1750.801	-0.32171	3.85E-06	ZDHHC7
122.2212	137.7118	97.64245	199.1662	219.3364	234.8506	-0.8712	3.85E-06	ZSCAN26
116.8291	116.9064	92.16717	53.94086	46.17608	34.91023	1.269993	3.88E-06	MGMT
75.48956	59.44393	82.12916	30.0824	23.08804	10.57886	1.767036	3.90E-06	KRBA1
55.71849	57.46247	61.1406	23.85846	12.59348	10.57886	1.888193	3.95E-06	RNF208
119.5251	130.7766	108.593	67.42607	38.82988	45.48908	1.240478	3.96E-06	ADAM8
104.2475	156.5357	191.6347	247.9205	293.8478	326.8867	-0.94171	3.99E-06	-
18.87239	14.86098	12.77565	44.60494	62.96738	59.2416	-1.84095	3.99E-06	BEND6
120.4238	68.36052	74.82879	30.0824	36.73097	19.04194	1.619493	3.99E-06	AL357060.1
443.9505	505.2734	526.5392	785.2544	643.3167	695.0309	-0.52552	4.00E-06	PRIM2
121.3225	134.7396	146.9199	66.38874	66.11575	61.35737	1.055381	4.00E-06	BTG2
44.93426	19.81464	40.15203	6.223945	4.197825	4.231543	2.841402	4.06E-06	-
1363.306	1355.322	1244.713	1081.929	854.2575	637.9051	0.622386	4.06E-06	CD320
474.5058	511.2178	436.1971	673.2234	658.0091	655.8891	-0.48362	4.07E-06	FAM210B
107.8422	99.07322	140.5321	49.79156	45.12662	55.01006	1.213652	4.08E-06	FTH1P11
840.2707	822.3077	796.6528	665.9621	556.2119	539.5217	0.48102	4.11E-06	SNX8
18665.69	19070.6	17148.57	13148.08	10363.38	9177.158	0.747606	4.13E-06	RPLP1
1337.244	1328.572	1196.348	1703.286	1675.982	1572.018	-0.35861	4.13E-06	ELF4
195.0147	190.2206	195.2849	304.9733	307.4907	302.5553	-0.65623	4.15E-06	PIGL
1494.514	1496.006	1586.918	1923.199	1845.994	1940.162	-0.31867	4.16E-06	CLMP
4797.182	4768.394	4558.168	4170.043	3468.453	3222.32	0.3789	4.21E-06	AC011495.1
65.60403	66.37906	65.70333	28.00775	18.89021	14.8104	1.678236	4.22E-06	BEGAIN
3520.15	3226.815	3590.869	4027.93	4281.782	4385.994	-0.29613	4.24E-06	SRSF11
66.50271	88.17516	54.75277	23.85846	23.08804	5.289428	2.000282	4.28E-06	SH3BP1
32.35267	36.65709	29.20148	0	8.395651	1.057886	3.378245	4.31E-06	LMCD1
62.90797	49.53661	65.70333	8.298593	10.49456	25.38926	2.014775	4.31E-06	MAP3K14-AS1

460.1269	387.3763	408.8207	307.0479	255.0179	242.2558	0.643677	4.32E-06	ULK3
303.7556	338.8304	333.0794	504.1395	463.8597	460.1803	-0.5504	4.34E-06	USP46
885.205	723.2345	903.4208	1108.9	1224.716	1116.069	-0.45679	4.34E-06	PPP3R1
471.8098	489.4217	478.1742	377.586	247.6717	260.2399	0.700393	4.35E-06	EPB41L4A-AS1
36.8461	36.65709	31.02657	8.298593	3.148369	5.289428	2.640846	4.39E-06	TUBB3
73.69219	67.36979	96.7299	133.8148	168.9625	191.4773	-1.05367	4.42E-06	1-Mar
2301.533	2562.033	2504.939	2985.419	2966.813	3133.457	-0.30246	4.42E-06	SLC39A6
605.7139	621.1891	608.6683	439.8254	447.0684	307.8447	0.619171	4.47E-06	IFFO2
130.3094	153.5635	162.4332	13.48521	5.247282	0	4.572218	4.48E-06	RAET1E-AS1
76.38825	71.33272	78.47897	30.0824	23.08804	28.56291	1.468883	4.57E-06	NDUFV2P1
85.3751	73.31418	108.593	186.7183	176.3087	160.7986	-0.96881	4.57E-06	IL1A
30.5553	23.77757	29.20148	3.111972	4.197825	1.057886	3.318455	4.57E-06	AC022075.1
288.478	341.8026	280.1517	191.905	193.1	170.3196	0.712031	4.58E-06	RPL39L
903.1787	856.9833	959.0861	1131.721	1256.199	1182.716	-0.39252	4.59E-06	WASHC2C
98.85538	86.1937	81.21661	43.56761	31.48369	23.27348	1.436427	4.59E-06	SAMD10
218.3805	238.7665	195.2849	325.7198	352.6173	346.9865	-0.6532	4.60E-06	NUTM2B-AS1
26.06187	21.79611	26.46384	1.037324	2.098913	1.057886	4.148579	4.61E-06	-
1540.347	1546.533	1581.443	2094.357	1975.077	1828.026	-0.33725	4.63E-06	KIF22
247.1385	268.4884	230.8742	365.1381	359.9635	446.4278	-0.6508	4.64E-06	ZNF512
515.8454	532.0232	512.851	802.8889	712.5809	664.3522	-0.48238	4.65E-06	MUL1
205.7989	210.0352	177.034	289.4134	365.2108	313.1342	-0.70743	4.66E-06	ENOX2
28.75793	29.72197	29.20148	2.074648	3.148369	5.289428	3.062824	4.66E-06	AC092171.3
843.8655	871.8443	759.2385	1149.355	1094.583	1044.133	-0.41033	4.68E-06	XPNPEP1
286.6806	235.7943	283.8019	159.7479	154.2701	168.2038	0.742717	4.69E-06	PHYKPL
1381.279	1413.775	1463.724	1747.891	1773.581	1780.422	-0.31606	4.74E-06	TDP2
1622.127	1725.855	1630.72	2166.97	2005.511	2032.198	-0.31792	4.74E-06	SDHB
58.41454	56.47173	43.80222	3.111972	11.54402	19.04194	2.238196	4.74E-06	ABHD8
30.5553	58.4532	51.10259	9.335917	8.395651	12.69463	2.201875	4.75E-06	SRMS
36.8461	28.73123	20.07602	4.149297	1.049456	3.173657	3.353594	4.76E-06	KCNN1
170.7502	220.9333	197.11	113.0683	108.094	97.32548	0.885132	4.77E-06	AP2A2
386.4347	426.0148	353.1554	286.3015	225.6331	198.8825	0.712533	4.77E-06	NAGPA
1736.26	1910.132	1786.765	1466.776	1424.112	1435.551	0.328233	4.78E-06	KCTD20
867.2313	771.7804	909.8086	1042.511	1191.133	1227.147	-0.44055	4.81E-06	ATP11C
56.61717	55.481	73.0037	137.9641	134.3304	119.5411	-1.08089	4.81E-06	LINC01291
309.1477	358.645	341.2923	252.0698	144.825	142.8146	0.901857	4.83E-06	NUDT16L1
42.23821	74.30491	48.36495	12.44789	11.54402	17.98406	1.972267	4.90E-06	FNDC11
903.1787	942.1863	968.2115	1337.111	1150.204	1212.337	-0.39515	4.90E-06	PSMA1
69.19877	66.37906	80.30407	22.82113	31.48369	17.98406	1.578621	4.90E-06	ACOT11
945.4169	1036.306	985.5499	1660.756	1369.541	1172.137	-0.50247	4.90E-06	DDX49
401.7123	364.5894	404.258	584.0135	559.3602	526.8271	-0.5122	4.91E-06	PLEKHA3
852.8523	826.2706	840.4551	1052.884	1264.595	1068.465	-0.42622	4.92E-06	ZCCHC14
1141.33	1168.073	1114.219	937.741	850.0596	820.9193	0.391888	4.95E-06	AKAP17A
72.79351	63.40686	69.35351	16.59719	26.23641	25.38926	1.592713	4.97E-06	CHRM3
439.4571	394.3114	386.9196	744.7987	554.113	557.5058	-0.60471	4.97E-06	PEMT
41.33952	34.67563	30.11403	7.261269	7.346194	1.057886	2.757468	4.98E-06	LMO2
2730.206	2918.697	2915.585	3442.879	3460.058	3424.376	-0.27018	4.99E-06	RAB1A
19.77108	14.86098	20.98856	57.05283	65.06629	57.12583	-1.68457	5.03E-06	METTL25
243.5437	182.2947	192.5473	106.8444	128.0337	86.74663	0.943871	5.04E-06	IFNGR2
109.6396	103.0361	97.64245	178.4198	184.7043	197.8246	-0.85381	5.05E-06	RANBP17
425.0781	415.1168	389.6572	276.9655	291.7489	243.3137	0.598684	5.07E-06	PLEKHG2
644.3573	675.6793	671.634	485.4677	507.9369	411.5175	0.50317	5.08E-06	ARHGAP39
2891.969	2863.216	2846.232	2499.951	2098.913	1831.2	0.41963	5.11E-06	AKR1B1
302.8569	258.5811	318.4786	168.0465	158.4679	199.9404	0.742176	5.12E-06	FDFT1
24.2645	28.73123	21.90111	2.074648	2.098913	0	4.159426	5.14E-06	SLC1A7

399.0163	437.9036	394.22	303.936	252.919	242.2558	0.622695	5.17E-06	ZBTB17
46.73163	47.55514	37.41439	13.48521	8.395651	7.4052	2.166879	5.19E-06	ASIC3
137.4988	174.3689	131.4067	251.0324	238.2266	273.9924	-0.78604	5.20E-06	ZNF430
1688.63	1768.457	1602.431	2410.741	2232.194	1952.857	-0.38285	5.24E-06	TUBA4A
136.6002	177.3411	109.5055	255.1817	270.7597	240.14	-0.85794	5.26E-06	SRD5A3
27.85924	35.66636	23.7262	4.149297	3.148369	3.173657	3.056241	5.27E-06	DDO
821.3983	956.0566	880.6071	700.1938	586.6461	635.7893	0.466646	5.28E-06	TXNDC17
29.65661	36.65709	24.63875	2.074648	4.197825	5.289428	2.976881	5.31E-06	LINC00482
950.809	914.4458	1078.63	1225.08	1348.551	1319.183	-0.40262	5.50E-06	PIKFYVE
336.1083	253.6274	251.8628	178.4198	154.2701	155.5092	0.78634	5.52E-06	IFT43
447.5453	511.2178	431.6344	343.3543	294.8972	275.0503	0.605328	5.55E-06	FAHD2A
931.9366	958.038	827.6794	746.8734	553.0635	561.7373	0.545309	5.55E-06	POLE4
108.7409	106.0083	95.81735	41.49297	38.82988	52.89428	1.221441	5.58E-06	LINC01089
2512.724	2318.313	2403.647	1781.086	1976.126	2013.156	0.326548	5.58E-06	KLF10
29.65661	47.55514	39.23949	11.41057	5.247282	2.115771	2.627611	5.59E-06	AL121906.2
105.1462	116.9064	104.9428	51.86621	38.82988	52.89428	1.186651	5.62E-06	KANK1
291.174	328.9231	271.0262	215.7634	141.6766	139.6409	0.84099	5.65E-06	PRADC1
33.25136	41.61075	24.63875	90.2472	96.54998	75.10988	-1.40014	5.69E-06	UGT8
38.64347	44.58295	52.92768	12.44789	12.59348	4.231543	2.21569	5.70E-06	AC079807.1
98.85538	92.13809	115.8934	56.0155	32.53315	31.73657	1.350772	5.74E-06	SDR39U1
455.6334	430.9685	456.2731	337.1303	278.1059	283.5134	0.579385	5.77E-06	ZFYVE21
135.7015	128.7952	168.8211	249.9951	218.2869	311.0184	-0.84564	5.77E-06	CEP85L
6.290797	3.962929	1.825092	30.0824	25.18695	34.91023	-2.8974	5.78E-06	MMP3
335.2096	342.7933	354.0679	236.5099	215.1386	220.0402	0.619545	5.79E-06	MTFR1L
1309.384	1372.164	1365.169	1708.473	1708.515	1635.491	-0.32044	5.80E-06	ALDH9A1
868.13	891.659	850.4931	1165.952	1109.275	1095.97	-0.36934	5.85E-06	SAP18
23.36582	34.67563	42.88967	3.111972	4.197825	7.4052	2.77983	5.86E-06	ACE2
74.59088	89.1659	74.82879	31.11972	34.63206	19.04194	1.490781	5.89E-06	RABGEF1
126.7146	118.8879	118.631	210.5768	235.0782	192.5352	-0.80883	5.89E-06	MFSD4B
1333.649	1277.054	1143.42	982.346	961.302	910.8396	0.395128	5.91E-06	SPRY4
50.32638	80.24931	38.32694	12.44789	15.74185	14.8104	1.971427	5.91E-06	DGCR5
560.7796	594.4393	667.9838	768.6572	873.1477	889.6819	-0.47334	5.93E-06	TRAPPC11
34.15004	23.77757	36.50185	7.261269	1.049456	3.173657	3.037611	5.93E-06	WNT5A-AS1
896.8879	922.3717	928.0595	1147.28	1168.045	1209.163	-0.35944	5.93E-06	TRIM24
261.5174	303.164	278.3266	443.9747	389.3483	431.6174	-0.58646	5.94E-06	MRPS18A
228.2661	193.1928	197.11	118.255	102.8467	124.8305	0.839167	5.98E-06	MR1
16.17634	23.77757	12.77565	80.91128	48.27499	55.01006	-1.81078	6.02E-06	C12orf60
170.7502	193.1928	149.6576	95.43382	80.80814	92.03605	0.935811	6.05E-06	INHBB
254.3279	274.4328	263.7259	407.6684	425.0298	362.8548	-0.59382	6.05E-06	LINC00857
154.5739	159.5079	167.9085	73.65001	58.76956	101.557	1.043113	6.10E-06	PLPP1
454.7348	499.329	386.9196	311.1972	285.4521	280.3397	0.611761	6.10E-06	KLHL36
11087.08	11778.81	10353.75	15305.72	13637.69	12660.78	-0.32477	6.12E-06	RPS15
64.70534	52.50881	73.91624	132.7775	146.9239	121.6569	-1.06802	6.12E-06	GULP1
1331.852	1354.331	1354.219	979.234	911.9776	1143.574	0.412982	6.12E-06	NUCB2
96.15932	78.26784	87.60444	37.34367	32.53315	34.91023	1.323118	6.15E-06	PDGFRL
1838.71	1796.197	1730.188	2335.017	2234.293	2103.077	-0.31466	6.17E-06	TMEM106C
692.8864	672.7071	711.7861	977.1593	926.67	863.2347	-0.41345	6.18E-06	GLTP
1050.563	1039.278	991.0252	866.1657	664.3059	619.921	0.518453	6.20E-06	TRABD
1900.719	1794.216	1792.241	1522.792	1462.942	1393.235	0.325541	6.25E-06	CMIP
16.17634	12.87952	16.42583	0	0	0	6.297964	6.25E-06	CYSLTR2
22.46713	34.67563	41.06458	5.186621	6.296738	0	3.092212	6.27E-06	-
74.59088	78.26784	75.74134	20.74648	16.7913	38.08388	1.596927	6.27E-06	DENND6B
332.5136	385.3948	359.5432	237.5472	227.732	236.9664	0.616846	6.30E-06	METTL21A
177.9397	154.5542	174.2963	81.94861	86.05542	100.4991	0.917569	6.41E-06	CMAHP

1137.736	1134.388	1085.017	1509.307	1366.392	1404.872	-0.35072	6.47E-06	ARAF
43.13689	38.63855	34.67676	7.261269	9.445107	7.4052	2.272253	6.54E-06	EWSAT1
38.64347	30.7127	49.2775	6.223945	4.197825	11.63674	2.430129	6.57E-06	-
211.191	248.6738	242.7373	128.6282	154.2701	86.74663	0.925756	6.63E-06	CLBA1
2295.242	2190.509	2233.001	2561.153	2892.302	2890.144	-0.31229	6.64E-06	AGFG1
1020.906	1064.046	994.6754	832.9713	696.839	773.3144	0.418842	6.66E-06	TLDC1
2759.862	2907.799	2909.197	3345.37	3550.311	3443.418	-0.26969	6.67E-06	IL13RA1
9682.435	10297.67	9888.351	13341.03	11503.09	11628.28	-0.28825	6.67E-06	RPL10A
4.493426	4.953661	2.737639	24.89578	23.08804	42.31543	-2.89379	6.74E-06	AP001282.1
63.80665	52.50881	46.53986	18.67183	10.49456	15.86829	1.854406	6.81E-06	STAG3
66.50271	62.41613	74.82879	28.00775	22.03858	19.04194	1.560038	6.81E-06	TMEM191A
2901.855	2826.559	2852.619	2437.712	2380.167	2231.081	0.283747	6.90E-06	PPP6R1
125.8159	135.7303	100.3801	71.57537	33.5826	34.91023	1.367757	6.91E-06	CCDC102A
308.2491	351.7099	302.0528	466.7959	479.6016	457.0066	-0.54592	6.94E-06	ZNF845
759.3891	777.7248	758.3259	586.0881	540.47	580.7792	0.426858	6.94E-06	LDLRAP1
505.0611	485.4588	547.5277	688.7832	715.7292	698.2045	-0.45064	7.10E-06	TDP1
1309.384	1260.211	1329.58	966.7861	1064.149	1014.512	0.35678	7.13E-06	WIPF2
264.2135	273.4421	281.0642	418.0416	397.744	393.5335	-0.56291	7.14E-06	C9orf85
2911.74	3135.667	2991.327	2644.139	2202.809	1981.42	0.40437	7.21E-06	TK1
1197.049	1173.027	1076.805	932.5544	822.7738	637.9051	0.526063	7.22E-06	ELK1
1009.224	1149.249	1127.907	1407.649	1360.095	1474.693	-0.3688	7.25E-06	RIPK1
263.3148	294.2475	242.7373	429.4522	412.4363	373.4336	-0.60395	7.25E-06	TBC1D14
25.16319	32.69416	32.85166	88.17255	81.85759	68.76257	-1.39843	7.27E-06	AC145207.5
203.1029	167.4337	177.034	285.2641	305.3918	288.8028	-0.68235	7.28E-06	ZSWIM1
559.8809	553.8193	535.6646	413.8923	394.5956	338.5234	0.523763	7.31E-06	SH3BP5L
32.35267	35.66636	42.88967	8.298593	8.395651	4.231543	2.404674	7.32E-06	IQCD
689.2916	802.4931	696.2728	598.536	389.3483	356.5075	0.702025	7.32E-06	COPS9
826.7905	842.1224	827.6794	989.6072	1134.462	1216.569	-0.42011	7.35E-06	ITGA1
607.5112	606.3281	587.6798	471.9825	408.2385	339.5813	0.56219	7.40E-06	TMEM8A
136.6002	135.7303	109.5055	76.76199	40.9288	29.6208	1.372488	7.42E-06	MNX1
28.75793	28.73123	16.42583	0	1.049456	2.115771	4.552435	7.48E-06	TMEM229B
56.61717	96.10102	77.56643	32.15705	10.49456	25.38926	1.756711	7.49E-06	FRK
301.0596	402.2373	312.0908	498.9529	541.5195	492.9747	-0.5965	7.49E-06	ZNF45
647.0534	709.3642	695.3602	523.8487	497.4423	468.6434	0.461135	7.52E-06	ANXA4
3733.139	3841.069	3453.075	3203.257	2826.186	2800.223	0.320464	7.59E-06	CYC1
13.48028	23.77757	9.125462	0	0	0	6.322445	7.59E-06	SNAI3
3758.302	3883.67	3792.542	3081.89	3260.661	3228.667	0.256562	7.60E-06	STAT3
16.17634	11.88879	16.42583	0	0	0	6.266479	7.60E-06	LSP1
362.1702	399.2651	379.6192	628.6184	593.9923	472.8749	-0.57201	7.65E-06	STEAP3
111.437	110.962	114.0683	31.11972	40.9288	65.58891	1.290827	7.68E-06	TNFRSF25
647.0534	643.9759	644.2576	859.9417	877.3455	819.8614	-0.40201	7.69E-06	KDM2B
29.65661	26.74977	23.7262	0	4.197825	3.173657	3.447084	7.69E-06	YPEL4
337.007	306.1362	338.5546	160.7852	213.0396	226.3875	0.710708	7.69E-06	FAM214A
445.7479	465.6441	388.7447	336.093	225.6331	202.0562	0.766654	7.75E-06	BCL2L12
569.7665	627.1335	579.4668	842.3072	814.3781	747.9252	-0.43757	7.87E-06	GTF2E2
35.04873	25.75904	34.67676	3.111972	5.247282	6.347314	2.702146	7.92E-06	AL645608.3
332.5136	342.7933	324.8665	252.0698	162.6657	176.6669	0.7574	7.93E-06	PQLC2
452.9374	502.3012	521.0639	319.4958	314.8369	370.26	0.555301	8.02E-06	ENPP1
40.44084	18.82391	17.33838	0	2.098913	2.115771	4.191539	8.05E-06	TMEM63C
16.17634	20.80538	23.7262	51.86621	61.91792	75.10988	-1.63834	8.10E-06	RASGRF2
1322.865	1370.183	1251.101	1120.31	875.2466	911.8975	0.43962	8.11E-06	CENPB
83.57773	67.36979	63.87824	7.261269	18.89021	32.79446	1.868365	8.12E-06	ASMTL-AS1
26.96056	31.70343	14.60074	1.037324	1.049456	1.057886	4.540598	8.15E-06	AC136475.3
53.02243	52.50881	44.71476	82.98593	137.4788	130.1199	-1.22254	8.16E-06	AC000123.3

22.46713	15.85171	30.11403	1.037324	0	0	5.926336	8.17E-06	AVPR2
53.02243	67.36979	63.87824	105.8071	146.9239	138.583	-1.08782	8.26E-06	CCDC169
204.9002	153.5635	201.6727	290.4508	329.5293	299.3816	-0.71279	8.28E-06	TYW5
598.5244	560.7544	631.482	779.0304	845.8618	790.2406	-0.43095	8.30E-06	MFSD14C
130.3094	94.11956	90.34208	50.82888	41.97825	39.14177	1.254896	8.30E-06	SLC25A45
15.27765	8.91659	18.25092	39.41832	48.27499	79.34143	-1.96986	8.33E-06	AC093536.1
596.727	580.5691	574.9041	760.3586	770.301	807.1668	-0.41584	8.34E-06	RAB8A
759.3891	654.874	676.1967	543.5578	468.0575	479.2222	0.488038	8.36E-06	SGSH
2348.265	2398.563	2222.05	2867.164	2863.966	2739.924	-0.28181	8.38E-06	CD9
30.5553	29.72197	26.46384	66.38874	82.90705	79.34143	-1.39836	8.43E-06	KCNIP4
1145.824	1266.156	1294.903	1565.322	1672.833	1511.719	-0.35794	8.46E-06	MICU1
70.99614	62.41613	72.09115	28.00775	22.03858	22.2156	1.508082	8.54E-06	HOXB3
271.403	264.5255	277.4141	186.7183	159.5174	153.3934	0.702891	8.56E-06	TGFBR1
468.215	452.7646	483.6495	315.3465	342.1228	318.4236	0.5257	8.59E-06	PAN2
119.5251	127.8045	137.7945	198.1289	221.4353	246.4874	-0.79018	8.60E-06	ACVR2A
455.6334	446.8202	526.5392	644.1783	681.0972	664.3522	-0.47697	8.67E-06	CERS6
1117.964	1193.832	1116.044	1613.039	1473.437	1359.383	-0.37558	8.73E-06	ZMAT2
120.4238	125.823	154.2203	220.95	246.6222	222.156	-0.78386	8.74E-06	CHRNA5
9429.905	9793.388	9171.089	8180.338	8186.809	7506.757	0.250088	8.77E-06	CAVIN1
144.6883	148.6098	129.5816	226.1367	241.375	236.9664	-0.73687	8.83E-06	CGRRF1
179.7371	207.063	176.1214	114.1057	89.20379	104.7307	0.868744	8.88E-06	AIM2
1501.703	1528.7	1488.363	1299.767	1080.94	1015.57	0.411733	9.01E-06	COPE
93.46327	105.0176	109.5055	175.3078	178.4076	199.9404	-0.84649	9.01E-06	ZNF124
17.97371	14.86098	10.95055	0	0	0	6.242325	9.06E-06	AC120498.3
783.6536	730.1696	800.303	612.0212	552.014	538.4638	0.443054	9.08E-06	E4F1
1454.971	1414.766	1332.317	1184.624	1034.764	979.6021	0.393306	9.14E-06	SLCO4A1
17742.74	19032.96	18856.86	16308.81	15538.25	13790.6	0.285664	9.22E-06	FLNA
1080.22	967.9453	1102.356	1273.834	1392.629	1433.435	-0.37936	9.31E-06	USP48
5.392112	2.972197	5.475277	26.97043	25.18695	39.14177	-2.71282	9.33E-06	SEPT7P6
85.3751	83.2215	92.16717	174.2705	136.4293	189.3615	-0.93882	9.36E-06	ENTPD1-AS1
192.3186	176.3503	182.5092	102.6951	110.1929	95.20971	0.839453	9.36E-06	DLGAP1-AS2
75.48956	87.18443	85.77934	26.97043	39.87934	29.6208	1.364523	9.39E-06	PLAU
79.0843	79.25857	72.09115	19.70916	38.82988	22.2156	1.512883	9.40E-06	SLC35E2
2167.629	2371.813	2307.829	2676.296	2804.147	2929.285	-0.29672	9.51E-06	TNC
17.97371	10.89805	14.60074	0	0	0	6.23348	9.54E-06	CHST1
206.6976	209.0445	216.2735	307.0479	321.1336	344.8707	-0.62238	9.56E-06	-
851.9536	836.178	884.2573	1257.237	1096.682	1052.596	-0.40516	9.58E-06	PEX26
35.94741	36.65709	27.37639	6.223945	3.148369	7.4052	2.574892	9.59E-06	P2RY6
48.52901	66.37906	72.09115	25.9331	13.64293	16.92617	1.724878	9.64E-06	SPRN
767.4772	730.1696	751.9381	542.5205	593.9923	530.0007	0.433044	9.71E-06	ARAP3
1232.996	1091.787	1143.42	1404.537	1501.772	1531.818	-0.35523	9.75E-06	UBAP2
21.56845	29.72197	27.37639	4.149297	2.098913	2.115771	3.229915	9.76E-06	AL021392.1
458.3295	489.4217	523.8015	701.2311	643.3167	680.2205	-0.46055	9.77E-06	-
74.59088	66.37906	63.87824	24.89578	16.7913	28.56291	1.544534	9.82E-06	CNFN
939.1261	953.0844	952.6983	1156.616	1246.754	1223.974	-0.35053	9.85E-06	PHACTR4
1322.865	1266.156	1317.717	846.4565	952.9064	1104.433	0.428352	9.85E-06	NHP2
1200.644	1076.926	1115.131	1419.059	1479.733	1411.219	-0.34482	9.87E-06	ANAPC7
961.5932	1134.388	1062.204	1516.568	1401.024	1271.579	-0.4082	9.92E-06	APMAP
31.45398	35.66636	43.80222	5.186621	8.395651	8.463085	2.332291	1.00E-05	TTC39B
337.007	373.506	366.8436	521.774	538.3711	478.1643	-0.51446	1.00E-05	MTERF4
112.3357	102.0454	87.60444	52.90353	40.9288	30.67868	1.277318	1.00E-05	ACOT2
79.98299	69.35125	65.70333	140.0388	128.0337	157.625	-0.98441	1.01E-05	-
18.87239	29.72197	18.25092	66.38874	60.86847	69.82045	-1.56566	1.02E-05	GPR65
76.38825	84.21224	73.91624	143.1507	138.5282	170.3196	-0.94753	1.02E-05	EOGT

26.06187	25.75904	21.90111	4.149297	0	0	4.139578	1.02E-05	TMEM59L
1571.801	1622.819	1650.796	1863.034	2069.528	2225.791	-0.34588	1.02E-05	VAPB
327.1214	283.3494	356.8056	487.5423	478.5521	457.0066	-0.55588	1.03E-05	KLHL2
89.86853	94.11956	74.82879	39.41832	23.08804	37.026	1.377854	1.03E-05	FRS3
148.2831	148.6098	156.0454	281.1148	241.375	228.5033	-0.72957	1.03E-05	PIGH
1129.647	1127.453	1068.592	832.9713	861.6037	889.6819	0.363882	1.03E-05	CALCOCO2
115.9304	125.823	153.3078	57.05283	66.11575	67.70468	1.049938	1.03E-05	UCP2
27.85924	35.66636	33.76421	6.223945	5.247282	5.289428	2.535804	1.04E-05	NAGS
47.63032	45.57368	45.62731	14.52254	13.64293	6.347314	2.006703	1.04E-05	LINC02298
204.0016	231.8313	203.4978	433.6015	325.3315	297.2659	-0.72537	1.04E-05	TSEN54
421.4834	416.1075	429.8093	549.7818	606.5858	601.9369	-0.47206	1.04E-05	STARD3NL
2484.865	2356.952	2404.559	2787.29	3018.236	2994.874	-0.28011	1.06E-05	EFCAB14
135.7015	146.6284	129.5816	78.83663	68.21466	57.12583	1.011401	1.06E-05	CCNO
172.5476	175.3596	156.0454	89.20988	72.41249	103.6728	0.925596	1.07E-05	TWIST2
529.3256	511.2178	537.4897	649.3649	761.9053	758.504	-0.45902	1.08E-05	ARHGAP12
90.76721	69.35125	51.10259	13.48521	22.03858	29.6208	1.698793	1.08E-05	FHDC1
370.2583	357.6543	349.5052	491.6916	501.6401	526.8271	-0.49646	1.09E-05	HIVEP2
1287.816	1453.404	1419.922	1692.913	1793.521	1751.859	-0.33248	1.09E-05	NCSTN
1006.528	1120.518	960.9112	1370.305	1259.348	1389.004	-0.38067	1.09E-05	UBQLN4
280.3898	256.5996	281.0642	172.1958	178.4076	160.7986	0.678201	1.09E-05	SFI1
2142.466	2259.86	2159.084	2747.872	2556.476	2669.046	-0.28142	1.10E-05	MRPL17
259.72	293.2567	265.5509	399.3698	425.0298	386.1283	-0.56548	1.11E-05	TTC5
88.96984	72.32345	65.70333	25.9331	29.38478	29.6208	1.419047	1.11E-05	NUDT8
243.5437	245.7016	245.4749	118.255	123.8358	176.6669	0.811584	1.11E-05	ROBO1
45.83295	65.38832	50.19004	20.74648	11.54402	12.69463	1.840289	1.11E-05	MYCL
41.33952	29.72197	34.67676	1.037324	9.445107	6.347314	2.655262	1.11E-05	AL606489.1
330.7162	314.0621	315.741	480.2811	440.7717	457.0066	-0.52054	1.11E-05	HACD1
303.7556	323.9694	372.3189	510.3635	528.926	442.1962	-0.56698	1.12E-05	ADAT1
51.22506	50.52734	36.50185	9.335917	16.7913	5.289428	2.13663	1.12E-05	NKILA
54.8198	61.4254	76.65388	135.8895	123.8358	134.3515	-1.03042	1.13E-05	-
44.93426	47.55514	58.40296	8.298593	19.93967	10.57886	1.959591	1.13E-05	AL691515.2
26.06187	35.66636	32.85166	80.91128	67.16521	97.32548	-1.3778	1.13E-05	HMGN1P38
1991.487	2160.787	1993.001	1783.16	1495.475	1465.172	0.373093	1.14E-05	ADRM1
38.64347	27.7405	46.53986	10.37324	6.296738	5.289428	2.362396	1.15E-05	KRT6A
1385.773	1408.821	1398.021	1664.905	1727.405	1808.985	-0.31103	1.15E-05	GPATCH8
36.8461	33.68489	39.23949	10.37324	7.346194	3.173657	2.391313	1.17E-05	CASC15
100.6528	122.8508	126.8439	197.0916	205.6934	207.3456	-0.80124	1.17E-05	TARSL2
60.21191	51.51807	60.22805	15.55986	20.98913	17.98406	1.65808	1.17E-05	WFDC3
655.1416	742.0584	718.1739	552.8938	486.9477	496.1484	0.461152	1.18E-05	IL17RA
2565.746	2637.329	2574.293	3036.248	3061.264	3410.623	-0.28991	1.19E-05	SUPT6H
252.5306	233.8128	307.5281	162.8599	162.6657	150.2198	0.739412	1.19E-05	CPEB2
24.2645	29.72197	22.81366	2.074648	3.148369	3.173657	3.193386	1.19E-05	AL118508.1
2723.016	2734.421	2836.194	3195.996	3309.985	3551.322	-0.27808	1.19E-05	ENAH
61.1106	48.54588	66.61587	8.298593	12.59348	26.44714	1.899912	1.19E-05	ANKRD37
144.6883	129.7859	137.7945	232.3606	209.8913	248.6031	-0.74394	1.21E-05	LONRF3
31.45398	28.73123	26.46384	3.111972	5.247282	4.231543	2.783887	1.21E-05	CDH10
3920.065	3651.839	3867.371	4289.335	4757.186	5047.173	-0.30087	1.22E-05	HP1BP3
185.1292	195.1742	204.4104	282.1522	306.4413	324.7709	-0.64343	1.22E-05	FZD3
217.4818	247.683	249.1251	346.4663	363.1119	367.0863	-0.59256	1.23E-05	LNX2
35.94741	18.82391	20.07602	3.111972	2.098913	1.057886	3.576384	1.23E-05	RNF223
486.1887	437.9036	451.7104	335.0557	325.3315	260.2399	0.579685	1.23E-05	DCAKD
68.30008	29.72197	38.32694	13.48521	9.445107	4.231543	2.327739	1.24E-05	REC8
52.12375	36.65709	36.50185	4.149297	8.395651	13.75251	2.255787	1.25E-05	LINC00106
1822.534	1779.355	1854.294	2345.39	2207.007	2142.218	-0.29504	1.25E-05	PRPS1

132.1067	136.721	133.2317	66.38874	53.52227	78.28354	1.020558	1.25E-05	KRT15
113.2343	96.10102	93.99226	48.75423	44.07717	42.31543	1.166665	1.25E-05	ZMAT5
88.07116	82.23077	76.65388	144.1881	152.1712	167.1459	-0.90798	1.26E-05	ZNF138
1368.698	1323.618	1372.47	1019.69	1035.813	1139.343	0.347694	1.26E-05	MED12
471.8098	514.19	440.7598	698.1191	669.5531	613.5737	-0.47452	1.27E-05	BCS1L
4208.543	4183.862	4118.321	3308.027	3589.141	3578.827	0.256119	1.27E-05	ZC3HAV1
33.25136	14.86098	22.81366	1.037324	2.098913	0	4.498622	1.27E-05	APOC3
128.512	165.4523	113.1557	65.35142	71.36303	58.18371	1.060927	1.30E-05	DOPEY2
24.2645	18.82391	29.20148	1.037324	0	1.057886	5.10928	1.31E-05	LINC02361
625.485	668.7442	657.9458	881.7255	810.1803	889.6819	-0.40349	1.31E-05	ELP3
78.18562	71.33272	78.47897	140.0388	143.7755	149.1619	-0.92427	1.31E-05	ZNF567
87.17247	70.34198	77.56643	26.97043	37.78043	24.33137	1.400802	1.32E-05	-
694.6837	758.9008	687.1473	544.5952	523.6787	515.1903	0.434425	1.32E-05	FBXO18
1352.521	1452.413	1448.211	1830.877	1901.615	1651.36	-0.34036	1.32E-05	CLPTM1L
90.76721	76.28638	72.09115	13.48521	37.78043	29.6208	1.565471	1.33E-05	PILRB
2370.732	2401.535	2431.936	2786.253	2987.802	2893.317	-0.26674	1.34E-05	TFG
135.7015	180.3133	162.4332	60.1648	96.54998	80.39931	1.012168	1.34E-05	RGS12
770.1733	741.0677	700.8355	589.2001	500.5907	528.9428	0.450414	1.35E-05	DHRSX
35.94741	41.61075	27.37639	9.335917	6.296738	3.173657	2.476449	1.37E-05	JMJD7-PLA2G4B
598.5244	593.4486	534.7521	446.0494	400.8923	364.9706	0.510352	1.38E-05	NELFA
115.0317	104.0269	114.0683	51.86621	56.67064	48.66274	1.083987	1.38E-05	ARHGEF18
957.9985	998.658	938.0975	783.1797	737.7678	664.3522	0.405209	1.38E-05	LEMD2
4586.89	4223.491	4376.572	5061.104	5292.408	5317.991	-0.24884	1.39E-05	PHLDA1
317.2359	355.6729	379.6192	228.2113	235.0782	227.4454	0.607403	1.39E-05	AMPD3
29.65661	28.73123	24.63875	5.186621	4.197825	0	3.140311	1.39E-05	CD74
597.6257	565.7081	599.5429	716.791	824.8727	850.5401	-0.43992	1.40E-05	APPL2
18.87239	24.7683	28.28893	3.111972	2.098913	1.057886	3.516573	1.41E-05	AC012307.1
894.1919	923.3624	953.6108	710.567	727.2732	689.7415	0.381207	1.41E-05	6-Sep
29.65661	27.7405	28.28893	2.074648	7.346194	1.057886	3.031558	1.42E-05	CD248
609.3086	654.874	669.8089	879.6509	863.7026	814.572	-0.4037	1.42E-05	SLC4A1AP
143.7896	111.9527	109.5055	239.6219	241.375	178.7827	-0.85214	1.43E-05	CYP20A1
746.8075	809.4282	810.341	640.029	554.113	547.9848	0.441533	1.43E-05	SPR
56.61717	54.49027	44.71476	21.78381	9.445107	10.57886	1.896	1.44E-05	HOXB6
389.1307	488.431	393.3074	313.2719	232.9793	263.4135	0.649225	1.44E-05	TAB1
851.9536	894.6312	863.2687	1075.705	1135.512	1121.359	-0.35291	1.45E-05	SRI
124.9173	147.6191	125.0188	59.12748	73.46194	65.58891	1.003537	1.45E-05	SPACA9
555.3875	574.6247	511.0259	423.2282	378.8537	335.3498	0.528239	1.46E-05	ARRDC2
87.17247	79.25857	106.7679	39.41832	37.78043	38.08388	1.245681	1.49E-05	ST13P6
100.6528	80.24931	76.65388	34.2317	41.97825	19.04194	1.434958	1.50E-05	C6orf226
690.1903	696.4847	680.7595	897.2854	941.3623	861.1189	-0.38512	1.50E-05	MFSD1
48.52901	72.32345	56.57787	14.52254	18.89021	22.2156	1.672382	1.51E-05	ADGRV1
193.2173	228.8591	208.9731	395.2205	362.0624	272.9345	-0.7083	1.52E-05	MB21D2
53.02243	87.18443	54.75277	28.00775	17.84076	11.63674	1.75812	1.52E-05	HRAS
8.986853	40.62002	36.50185	3.111972	3.148369	1.057886	3.551815	1.53E-05	MRM1
193.2173	171.3967	165.1709	117.2176	73.46194	85.68874	0.938582	1.56E-05	C7orf43
26.06187	34.67563	34.67676	2.074648	9.445107	2.115771	2.806685	1.56E-05	B4GALNT1
1177.278	1230.489	1289.428	879.6509	1033.715	925.65	0.381063	1.56E-05	HECTD4
34.15004	39.62929	32.85166	88.17255	73.46194	95.20971	-1.27005	1.57E-05	ELAC1
40.44084	34.67563	43.80222	13.48521	6.296738	1.057886	2.509058	1.57E-05	ADAMTS15
8160.961	7470.121	10068.12	2699.117	3398.14	4873.679	1.228059	1.58E-05	MALAT1
17.97371	17.83318	5.475277	64.3141	49.32445	45.48908	-1.95065	1.59E-05	RMDN2
38.64347	41.61075	35.5893	10.37324	6.296738	9.520971	2.144085	1.59E-05	AL731571.1
27.85924	18.82391	35.5893	3.111972	4.197825	3.173657	2.974509	1.61E-05	AC108058.1
87.17247	85.20297	70.26606	25.9331	39.87934	28.56291	1.362298	1.61E-05	CCDC61

13.48028	9.907322	17.33838	0	0	0	6.139194	1.61E-05	EVA1B
17.97371	35.66636	36.50185	4.149297	6.296738	1.057886	2.966691	1.63E-05	ODF3L1
1827.027	2028.029	1817.792	1612.002	1368.491	1454.593	0.354705	1.63E-05	GRHPR
1053.259	1175.999	1064.029	1485.448	1347.502	1375.251	-0.35428	1.63E-05	IVD
4090.815	4455.323	4372.921	3771.711	3511.481	3249.825	0.294407	1.63E-05	KIF1C
1838.71	1856.632	2047.754	2213.65	2433.689	2652.119	-0.34578	1.63E-05	CDK5RAP2
3455.445	3305.083	3878.321	2687.707	2678.213	3066.811	0.335476	1.64E-05	VMP1
366.6636	346.7563	337.6421	272.8162	173.1603	185.13	0.735516	1.66E-05	B9D1
144.6883	142.6654	137.7945	212.6514	229.8309	263.4135	-0.7313	1.66E-05	WDR5B
2581.024	2656.153	2448.362	3310.101	3000.396	3054.116	-0.28528	1.66E-05	MRPL37
88.07116	92.13809	94.90481	40.45564	45.12662	32.79446	1.216226	1.66E-05	CAPS2
1286.917	1298.85	1283.04	1614.076	1648.696	1532.876	-0.30993	1.67E-05	KIF18B
130.3094	138.7025	145.0948	264.5177	214.0891	216.8666	-0.7484	1.68E-05	VPS9D1-AS1
408.0031	415.1168	397.8702	312.2346	261.3146	234.8506	0.594403	1.68E-05	H6PD
425.0781	397.2836	387.8321	503.1022	605.5363	614.6316	-0.50951	1.69E-05	PTPN4
2346.467	2513.488	2405.472	3044.546	2866.065	2861.581	-0.27216	1.70E-05	MYL12A
16.17634	8.91659	15.51329	0	0	0	6.13552	1.70E-05	IFNL1
7.189482	2.972197	1.825092	13.48521	31.48369	52.89428	-3.02047	1.71E-05	AC245060.5
295.6675	278.3957	268.2886	400.4071	399.8429	424.2122	-0.53939	1.71E-05	C9orf72
369.3596	353.6914	320.3037	268.667	163.7152	184.0721	0.758678	1.72E-05	MOB2
931.0379	1041.26	999.2381	807.0382	736.7184	600.8791	0.469975	1.73E-05	GRK6
40.44084	25.75904	41.97713	7.261269	7.346194	7.4052	2.299188	1.73E-05	GJA5
26.96056	21.79611	19.16347	1.037324	3.148369	0	4.018899	1.74E-05	ARL11
20.66976	25.75904	22.81366	3.111972	0	2.115771	3.723944	1.75E-05	NKX2-1
2111.012	1898.243	1849.731	1692.913	1324.414	1082.217	0.515111	1.75E-05	ATP5F1D
36.8461	20.80538	31.93912	7.261269	3.148369	2.115771	2.837035	1.76E-05	LINC00887
30.5553	33.68489	40.15203	5.186621	7.346194	8.463085	2.315152	1.77E-05	AC132872.3
1065.841	1176.99	1089.58	1465.739	1436.706	1330.82	-0.34575	1.77E-05	HKDC1
773.768	689.5496	730.9495	891.0614	1001.181	1009.223	-0.4024	1.77E-05	GK5
2214.361	2352.989	2052.316	2893.097	2738.032	2591.82	-0.31324	1.78E-05	TTLL12
40.44084	14.86098	34.67676	4.149297	2.098913	5.289428	2.966966	1.79E-05	HOXD-AS2
1525.968	1396.932	1470.112	1137.945	1179.589	1189.063	0.325524	1.79E-05	INHBA
419.686	378.4597	401.5203	238.5846	208.8418	312.0763	0.660148	1.80E-05	TUBG2
211.191	199.1372	204.4104	273.8536	351.5679	339.5813	-0.64999	1.81E-05	CRYBG3
487.9861	401.2465	378.7067	224.062	247.6717	321.5972	0.677249	1.84E-05	TRIM3
303.7556	284.3401	286.5395	387.9592	435.5244	450.6593	-0.54227	1.85E-05	SFMBT1
32.35267	28.73123	30.11403	5.186621	7.346194	2.115771	2.637206	1.88E-05	CHRM4
692.8864	773.7618	725.4742	546.6698	552.014	539.5217	0.419671	1.89E-05	DENND1A
247.1385	249.6645	293.8399	467.8332	357.8646	390.3598	-0.62092	1.90E-05	ZNF180
927.4432	951.1029	907.0709	1465.739	1261.447	1072.696	-0.44824	1.90E-05	MPDU1
37.74478	26.74977	39.23949	9.335917	6.296738	4.231543	2.38429	1.91E-05	-
156.3712	130.7766	149.6576	74.68734	69.26412	84.63085	0.935427	1.91E-05	MAPK13
501.4664	587.5042	545.7026	413.8923	385.1505	342.755	0.516844	1.92E-05	MRPS26
585.9428	630.1057	591.3299	764.5079	815.4276	806.1089	-0.40115	1.93E-05	SIK2
32.35267	21.79611	46.53986	82.98593	85.00596	92.03605	-1.36402	1.93E-05	BCLAF1P2
641.6613	654.874	657.0333	826.7473	825.9221	919.3027	-0.39674	1.95E-05	MRPL9
102.4501	106.9991	131.4067	186.7183	192.0505	218.9823	-0.80974	1.96E-05	HOXA10
28.75793	13.87025	24.63875	1.037324	0	2.115771	4.420663	1.96E-05	NHLRC4
65.60403	54.49027	70.26606	20.74648	28.33532	10.57886	1.673995	1.97E-05	LRRC23
75.48956	82.23077	68.44097	22.82113	34.63206	29.6208	1.376857	1.98E-05	AP006621.3
118.6265	136.721	111.3306	57.05283	67.16521	55.01006	1.031679	1.98E-05	SHROOM2
40.44084	21.79611	20.07602	5.186621	1.049456	3.173657	3.128315	1.98E-05	NOXA1
33.25136	24.7683	19.16347	5.186621	2.098913	0	3.398992	1.99E-05	MYBPC3
40.44084	30.7127	31.93912	3.111972	10.49456	5.289428	2.449883	1.99E-05	SULT1C2

332.5136	342.7933	311.1783	230.286	189.9516	222.156	0.618431	2.00E-05	TEAD2
5295.054	5341.037	5317.407	4622.316	4515.811	4574.298	0.218362	2.00E-05	COPG1
44.93426	17.83318	31.93912	3.111972	3.148369	7.4052	2.798141	2.01E-05	AL451069.3
329.8175	371.5246	339.4672	533.1846	463.8597	485.5695	-0.51135	2.02E-05	C1orf123
1818.04	1656.504	1653.534	1485.448	1244.655	1142.517	0.405097	2.04E-05	STRN4
499.669	527.0695	453.5355	716.791	639.1189	670.6995	-0.45387	2.04E-05	RPL14P1
92.56458	115.9157	89.42953	185.681	179.457	168.2038	-0.84254	2.05E-05	FAM103A2P
54.8198	62.41613	66.61587	22.82113	14.69239	24.33137	1.571574	2.05E-05	LRP1B
505.0611	603.3559	522.889	790.441	749.3118	695.0309	-0.45521	2.07E-05	DSTYK
34.15004	41.61075	50.19004	13.48521	5.247282	10.57886	2.102276	2.08E-05	ZDHHC11B
320.8306	290.2845	321.2163	209.5395	215.1386	168.2038	0.653376	2.08E-05	CNNM4
291.174	293.2567	310.2657	385.8846	515.2831	436.9068	-0.58045	2.08E-05	TRNT1
339.703	337.8397	324.8665	219.9127	240.3255	200.9983	0.600064	2.10E-05	NUDT16
87.17247	74.30491	94.90481	127.5909	167.913	214.7508	-0.99111	2.10E-05	PTPRB
387.3334	330.9045	396.0451	470.9452	554.113	610.4	-0.55244	2.11E-05	CBR4
5102.735	5234.038	5354.821	6801.734	6084.748	5977.054	-0.26566	2.11E-05	LASP1
453.8361	568.6803	692.6226	895.2107	909.8787	751.0988	-0.57589	2.12E-05	UTP4
844.7642	902.557	834.9798	692.9325	626.5254	641.0787	0.396934	2.12E-05	CLEC16A
1452.275	1362.257	1391.633	1123.422	1141.809	1107.606	0.318768	2.14E-05	TRAK1
39.54215	28.73123	35.5893	9.335917	7.346194	1.057886	2.547362	2.14E-05	TMC8
1339.94	1295.878	1420.834	1830.877	1656.042	1622.797	-0.33279	2.15E-05	ELOC
75.48956	64.39759	57.49041	120.3296	128.0337	143.8725	-0.98993	2.15E-05	PARD6B
214.7858	269.4792	228.1366	355.8022	355.7657	363.9127	-0.59582	2.16E-05	MRPS28
398.1176	450.7831	377.7941	321.5705	223.5342	222.156	0.676007	2.16E-05	SDF2L1
424.1795	460.6905	447.1476	576.7522	595.0417	663.2943	-0.46254	2.17E-05	ZEB2
3538.124	3627.071	3508.74	4188.715	4066.643	4394.457	-0.24509	2.17E-05	UBAP2L
61.1106	55.481	62.05314	16.59719	24.1375	20.09983	1.555155	2.18E-05	SH3D21
171.6489	194.1835	207.148	303.936	278.1059	312.0763	-0.64236	2.18E-05	KPNA5
119.5251	107.9898	96.7299	45.64226	60.86847	41.25754	1.13382	2.19E-05	DPF1
2.696056	1.981464	8.212916	25.9331	31.48369	29.6208	-2.74506	2.21E-05	AC127024.5
753.0983	722.2438	634.2196	514.5128	541.5195	477.1064	0.460293	2.21E-05	MPV17
14.37896	26.74977	18.25092	0	0	1.057886	5.717643	2.21E-05	RGS11
139.2962	151.582	152.3952	84.02326	65.06629	84.63085	0.923076	2.23E-05	PHF21B
2344.67	1977.501	2299.616	2511.362	2942.676	3595.753	-0.45035	2.24E-05	PNISR
1016.413	1127.453	1063.116	1288.357	1357.997	1426.03	-0.34504	2.24E-05	SNX14
61.1106	48.54588	55.66532	107.8817	131.182	105.7886	-1.05878	2.24E-05	DOK3
31.45398	24.7683	21.90111	5.186621	3.148369	1.057886	3.052699	2.26E-05	ENTPD2
1715.59	1912.113	1630.72	1494.784	1300.276	1244.074	0.380108	2.26E-05	KLHDC3
61.1106	59.44393	68.44097	22.82113	23.08804	22.2156	1.472473	2.28E-05	LINC00847
324.4254	318.025	372.3189	432.5642	499.5412	575.4898	-0.57045	2.28E-05	ARID4A
446.6466	450.7831	461.7484	337.1303	259.2157	326.8867	0.55791	2.28E-05	PEX14
58.41454	32.69416	52.92768	10.37324	10.49456	16.92617	1.93361	2.29E-05	MASP2
976.8709	946.1492	1025.702	1198.109	1257.249	1273.694	-0.33837	2.29E-05	WDR41
65.60403	54.49027	55.66532	119.2923	109.1435	128.0042	-1.01871	2.29E-05	ZNF860
1658.973	1600.032	1632.545	2276.926	1932.049	1954.973	-0.33354	2.30E-05	RER1
766.5785	763.8545	779.3145	554.9684	619.1792	570.2004	0.405149	2.30E-05	ZNF335
11044.84	10984.25	10186.75	9219.737	8717.834	9406.719	0.236506	2.30E-05	RPL27
79.0843	66.37906	65.70333	129.6655	144.825	132.2357	-0.94456	2.31E-05	ZNF786
697.3798	649.9203	734.5997	810.1502	1033.715	1017.686	-0.45835	2.31E-05	DLG1
159.0673	140.684	125.9314	77.79931	74.5114	69.82045	0.938437	2.32E-05	FAM110A
30.5553	43.59222	27.37639	7.261269	6.296738	6.347314	2.348055	2.33E-05	AASS
494.2769	571.6525	620.5314	768.6572	757.7075	773.3144	-0.44774	2.34E-05	EVI5
974.1748	1065.037	1054.903	825.71	818.5759	703.494	0.397837	2.34E-05	AMBRA1
3403.321	3270.407	3346.307	2891.022	2828.285	2644.714	0.260665	2.35E-05	RNF10

708.164	781.6877	647.9078	557.0431	477.5026	510.9588	0.467305	2.37E-05	SFXN3
53.92112	50.52734	57.49041	114.1057	125.9348	97.32548	-1.05811	2.38E-05	-
325.3241	301.1826	333.9919	423.2282	483.7994	468.6434	-0.51752	2.40E-05	BET1
231.8608	219.9425	239.0871	149.3747	144.825	128.0042	0.710684	2.40E-05	C21orf58
72.79351	71.33272	83.95425	37.34367	26.23641	25.38926	1.357762	2.41E-05	LINC01003
11.68291	11.88879	14.60074	0	0	0	6.044572	2.42E-05	FGF7
744.1114	748.9935	834.0672	984.4206	1035.813	996.5283	-0.37418	2.42E-05	LHFPL2
322.628	325.9509	361.3683	449.1614	468.0575	535.2902	-0.52387	2.43E-05	TTLL5
29.65661	24.7683	20.98856	1.037324	2.098913	5.289428	3.166774	2.43E-05	AC091435.2
81.78036	93.12882	65.70333	26.97043	36.73097	31.73657	1.333213	2.43E-05	PDE2A
1359.711	1194.823	1262.051	1498.933	1625.608	1816.39	-0.37202	2.44E-05	SPCS2
559.8809	553.8193	565.7787	424.2656	397.744	406.2281	0.451447	2.45E-05	NDUFC1
705.4679	694.5033	694.4477	818.4487	961.302	1057.886	-0.43792	2.45E-05	ERP44
215.6845	198.1464	168.8211	117.2176	108.094	108.9622	0.801477	2.46E-05	TFEB
607.5112	605.3374	605.0181	769.6945	803.8836	801.8773	-0.3859	2.48E-05	ACTR6
124.9173	117.8971	130.4941	195.0169	207.7924	221.0981	-0.74014	2.51E-05	CEP41
245.3411	234.8035	226.3115	164.9345	138.5282	104.7307	0.790832	2.53E-05	PEX10
11.68291	13.87025	11.8631	54.97818	35.68152	44.4312	-1.85468	2.53E-05	PTGFR
727.0364	695.494	735.5123	901.4347	902.5325	1024.033	-0.38966	2.54E-05	MPHOSPH8
1606.849	1753.596	1617.944	1446.03	1211.073	1087.506	0.410488	2.55E-05	ST3GAL4
563.4757	574.6247	602.2805	413.8923	426.0793	436.9068	0.446891	2.56E-05	WNT5A
30.5553	39.62929	37.41439	7.261269	10.49456	5.289428	2.221704	2.56E-05	IL2RB
117.7278	112.9435	108.593	197.0916	171.0614	217.9244	-0.78849	2.57E-05	-
11137.41	11230.94	11602.11	12522.58	13384.77	13954.57	-0.2307	2.58E-05	SRRM2
51.22506	50.52734	66.61587	125.5162	107.0445	115.3095	-1.04589	2.59E-05	LINC00997
527.5283	569.671	579.4668	405.5937	415.5847	269.7608	0.619603	2.59E-05	TYRO3
293.8701	117.8971	126.8439	1067.407	1126.067	513.0746	-2.32872	2.59E-05	MTCH2
1143.128	1173.027	1054.903	949.1516	795.4879	816.6877	0.395943	2.59E-05	MRPL43
900.4827	947.14	833.1547	762.4332	556.2119	564.911	0.508709	2.60E-05	SPINDOC
203.1029	185.2669	209.8856	328.8318	286.5016	303.6132	-0.61847	2.61E-05	STK36
101.5514	102.0454	93.99226	42.53029	35.68152	55.01006	1.159739	2.61E-05	STAG3L3
2319.507	2622.468	2681.061	3044.546	3096.946	3204.336	-0.29413	2.61E-05	CSNK1A1
204.9002	213.0074	219.0111	141.0761	121.7369	118.4832	0.739816	2.61E-05	P2RX4
679.4061	739.0862	671.634	521.774	548.8657	453.833	0.454714	2.62E-05	TRIM65
301.0596	238.7665	297.4901	411.8177	413.4858	412.5754	-0.56243	2.63E-05	ZBTB34
130.3094	172.3874	150.5701	96.47114	70.31358	50.77851	1.057066	2.65E-05	XKR8
160.8647	143.6562	160.6081	244.8085	263.4135	235.9085	-0.67697	2.67E-05	SYT14
174.3449	226.8777	210.7982	115.143	120.6875	119.5411	0.78321	2.68E-05	SYNE1
40.44084	24.7683	25.55129	3.111972	7.346194	4.231543	2.629687	2.68E-05	-
441.2545	452.7646	392.3949	331.9437	270.7597	258.1241	0.578899	2.70E-05	SP2
517.6427	572.6432	624.1816	719.903	831.1694	784.9512	-0.44637	2.71E-05	JMY
223.7726	185.2669	200.7602	301.8613	291.7489	361.7969	-0.64652	2.71E-05	ACTR8
110.5383	127.8045	131.4067	74.68734	28.33532	51.8364	1.254591	2.71E-05	CNIH3
393.6242	380.4412	391.4823	290.4508	265.5125	206.2877	0.612436	2.72E-05	TRIM47
805.222	769.7989	711.7861	609.9466	537.3216	554.3321	0.426323	2.72E-05	CREB3
567.9691	603.3559	607.7558	453.3106	394.5956	445.3699	0.459867	2.73E-05	NAGA
53.92112	47.55514	39.23949	90.2472	105.9951	110.0201	-1.12159	2.76E-05	PGM5P2
168.9528	162.4801	138.707	257.2564	230.8804	272.9345	-0.69515	2.78E-05	-
64.70534	68.36052	46.53986	21.78381	23.08804	13.75251	1.613597	2.79E-05	FBXO32
398.1176	439.8851	406.9956	554.9684	605.5363	552.2163	-0.46082	2.79E-05	ZCCHC7
65.60403	53.49954	48.36495	23.85846	15.74185	12.69463	1.678309	2.79E-05	MST1
169.8515	165.4523	185.2469	85.06058	114.3907	88.8624	0.852855	2.80E-05	CCDC142
7.189482	17.83318	13.68819	0	0	0	6.062029	2.81E-05	XAF1
22.46713	24.7683	18.25092	3.111972	1.049456	1.057886	3.644117	2.81E-05	AC006001.2

1157.507	1213.647	1166.234	1575.695	1523.811	1367.846	-0.33705	2.81E-05	BIRC2
149.1818	144.6469	123.1937	67.42607	68.21466	81.4572	0.941864	2.83E-05	AC010457.1
525.7309	618.2169	642.4325	801.8516	820.6749	783.8933	-0.43034	2.83E-05	ZNF445
6108.364	6220.807	6377.786	7209.403	7156.243	7249.691	-0.20848	2.84E-05	EIF4A2
176.1423	154.5542	203.4978	266.5923	327.4304	270.8187	-0.69372	2.84E-05	FAM239B
1533.157	1770.438	1632.545	1393.126	1269.842	1162.616	0.367209	2.85E-05	CD2BP2
310.9451	266.507	259.1631	412.855	402.9912	405.1702	-0.54478	2.85E-05	RBFOX2
185.1292	209.0445	166.0834	125.5162	96.54998	80.39931	0.88787	2.86E-05	TLX3
1274.336	1284.98	1212.774	1093.34	892.0379	843.1349	0.415038	2.87E-05	NAXE
3021.38	3220.87	3178.398	2697.043	2648.828	2520.942	0.259862	2.87E-05	BIRC5
30.5553	24.7683	37.41439	6.223945	7.346194	2.115771	2.563082	2.88E-05	SARDH
79.0843	84.21224	93.07971	38.38099	40.9288	29.6208	1.234449	2.89E-05	TMEM234
109.6396	110.962	122.2812	190.8676	192.0505	195.7088	-0.75461	2.92E-05	ATF7IP2
399.9149	446.8202	427.0716	550.8191	600.289	594.5317	-0.45516	2.92E-05	PMS2
2210.766	2528.349	2493.989	3711.546	3136.825	2716.65	-0.40337	2.97E-05	PHB
88.96984	106.0083	101.2926	54.97818	40.9288	35.96811	1.166251	2.97E-05	IL15
36.8461	61.4254	41.06458	14.52254	10.49456	12.69463	1.882572	3.00E-05	ZNF815P
52.12375	54.49027	62.05314	124.4789	113.3413	104.7307	-1.02209	3.01E-05	PA2G4P4
600.3218	615.2447	670.7215	799.7769	877.3455	812.4562	-0.40021	3.02E-05	APAF1
26.96056	20.80538	16.42583	2.074648	0	2.115771	3.937106	3.02E-05	ADGRG3
255.2266	270.4699	250.9502	382.7726	399.8429	352.2759	-0.54783	3.04E-05	CRIPT
262.4161	286.3216	313.0034	431.5268	444.9695	384.0125	-0.54891	3.07E-05	INAFM2
384.6373	392.3299	436.1971	517.6247	563.5581	622.0368	-0.48914	3.08E-05	UHRF2
345.9938	374.4968	306.6155	252.0698	213.0396	153.3934	0.730769	3.09E-05	IL27RA
123.1199	95.11029	86.69189	53.94086	44.07717	35.96811	1.186406	3.09E-05	TBX2
806.1207	792.5857	782.0521	673.2234	537.3216	497.2063	0.479063	3.10E-05	RHOBTB2
35.94741	43.59222	39.23949	103.7324	77.65977	88.8624	-1.18795	3.11E-05	AC025048.6
3205.61	3142.602	2800.604	2613.02	2465.173	2317.828	0.306707	3.12E-05	KDELR1
97.05801	87.18443	114.9808	189.8303	160.5668	183.0142	-0.83261	3.12E-05	RPL21P75
76.38825	85.20297	55.66532	33.19437	24.1375	23.27348	1.428525	3.14E-05	NUDT18
164.4594	139.6932	147.8325	86.0979	89.20379	61.35737	0.933685	3.14E-05	COL16A1
705.4679	738.0955	730.037	894.1734	959.2031	942.5761	-0.36341	3.15E-05	GNAI1
266.0108	218.9518	187.9845	95.43382	133.281	143.8725	0.853749	3.15E-05	PLCD4
470.0124	455.7368	529.2768	377.586	327.4304	279.2818	0.563887	3.16E-05	COX19
1083.814	1252.285	1176.272	869.2776	977.0439	844.1928	0.384108	3.17E-05	ASAP2
34.15004	31.70343	33.76421	8.298593	2.098913	8.463085	2.40111	3.17E-05	CD14
15.27765	30.7127	19.16347	2.074648	0	2.115771	3.955413	3.18E-05	TBC1D3L
1118.863	1127.453	1020.227	1453.291	1306.573	1375.251	-0.34043	3.19E-05	MRPL4
452.0387	518.1529	441.6724	355.8022	305.3918	318.4236	0.526368	3.21E-05	DOLK
120.4238	111.9527	127.7565	205.3902	226.6826	179.8406	-0.76409	3.22E-05	SP4
19.77108	22.78684	20.98856	3.111972	0	1.057886	3.923282	3.23E-05	ZBED6
122.2212	112.9435	117.7185	181.5317	221.4353	194.651	-0.75936	3.23E-05	SGK3
1557.422	1631.736	1531.253	2429.413	2115.704	1745.511	-0.41453	3.25E-05	TMED3
1300.398	1353.34	1269.352	1816.355	1668.636	1504.313	-0.34716	3.25E-05	TOR3A
29.65661	50.52734	22.81366	4.149297	8.395651	6.347314	2.44474	3.25E-05	CFD
35.94741	44.58295	39.23949	12.44789	8.395651	9.520971	1.978013	3.26E-05	STX1B
1132.343	1296.868	1136.12	992.7192	869.9993	699.2624	0.476233	3.26E-05	FRMD8
205.7989	212.0167	178.8591	135.8895	104.9456	99.44125	0.809298	3.28E-05	TMEM205
962.4919	978.8434	850.4931	727.1642	733.57	583.9529	0.448941	3.30E-05	PEPD
180.6357	167.4337	186.1594	309.1226	241.375	297.2659	-0.6656	3.31E-05	CHMP4C
232.7595	206.0723	218.0985	300.824	389.3483	322.6551	-0.62378	3.34E-05	POPDC3
725.239	843.1131	817.6414	957.4502	1085.138	1098.085	-0.39692	3.35E-05	MBTPS2
12.58159	32.69416	22.81366	1.037324	1.049456	3.173657	3.695039	3.35E-05	SIGLEC15
474.5058	466.6349	409.7333	365.1381	248.7212	246.4874	0.650364	3.36E-05	NLRX1

469.1137	499.329	430.7218	335.0557	295.9467	344.8707	0.519296	3.37E-05	KIF7
218.3805	213.0074	228.1366	155.5986	119.638	116.3674	0.751795	3.38E-05	ACACB
416.0913	396.2929	470.8738	561.1924	612.8825	599.8212	-0.4664	3.39E-05	ALG10B
71.89482	91.14736	80.30407	40.45564	23.08804	33.85234	1.319547	3.40E-05	WNK2
42.23821	24.7683	41.06458	85.06058	99.69835	77.22565	-1.27285	3.41E-05	LINC01806
156.3712	150.5913	155.1329	86.0979	92.35216	77.22565	0.853944	3.42E-05	SDK2
34.15004	26.74977	33.76421	88.17255	74.5114	68.76257	-1.28753	3.42E-05	GJA3
1199.745	1272.1	1263.877	1496.859	1611.965	1518.066	-0.30885	3.43E-05	RBMS2
406.2057	381.4319	446.2351	534.2219	611.833	570.2004	-0.47534	3.43E-05	SLC35E3
122.2212	108.9805	157.8705	224.062	224.5837	214.7508	-0.76823	3.44E-05	PCBD2
727.9351	755.9287	639.6949	592.3121	402.9912	443.2541	0.561345	3.44E-05	C19orf24
186.9265	174.3689	158.783	246.8831	283.3532	288.8028	-0.65494	3.46E-05	SMAP1
542.8059	547.8749	563.041	735.4628	692.6412	754.2725	-0.40013	3.46E-05	UIMC1
2781.431	2771.078	2875.433	3246.825	3295.293	3457.17	-0.24655	3.47E-05	ELL2
133.9041	112.9435	138.707	213.6888	219.3364	206.2877	-0.72803	3.48E-05	ZNF440
27.85924	52.50881	38.32694	76.76199	104.9456	102.6149	-1.26491	3.49E-05	TBC1D30
1193.454	1292.905	1269.352	1902.452	1561.591	1472.577	-0.39475	3.53E-05	BOP1
2693.36	2956.345	2600.757	2420.077	2060.083	2082.977	0.329772	3.55E-05	ELOB
261.5174	277.405	306.6155	424.2656	451.2662	369.2021	-0.55798	3.55E-05	ZNF271P
2103.822	2103.324	1956.499	2367.174	2531.289	3115.473	-0.3787	3.56E-05	RPS15A
603.9165	524.0973	579.4668	763.4706	735.6689	768.025	-0.40819	3.58E-05	CDKN2AIP
612.9034	603.3559	614.1436	810.1502	762.9548	810.3404	-0.38078	3.59E-05	CCNB1IP1
135.7015	178.3318	176.1214	253.1071	256.0673	281.3976	-0.69089	3.59E-05	ZNF557
36.8461	45.57368	45.62731	15.55986	10.49456	7.4052	1.933822	3.60E-05	AL513477.1
0	1.981464	1.825092	10.37324	16.7913	37.026	-4.09597	3.61E-05	NUFIP1P
148.2831	110.962	168.8211	255.1817	240.3255	229.5612	-0.75802	3.61E-05	VCPKMT
671.3179	719.2716	717.2613	571.5656	510.0358	473.9328	0.437929	3.63E-05	NOSIP
2162.237	2219.24	2328.818	1910.751	1845.994	1760.322	0.282421	3.63E-05	DCTN1
196.8121	126.8137	180.6842	113.0683	62.96738	58.18371	1.106895	3.64E-05	FAM83H
226.4687	243.7201	232.6993	173.2331	120.6875	116.3674	0.775766	3.66E-05	TPI1P1
50.32638	35.66636	51.10259	122.4042	87.10488	96.2676	-1.15443	3.69E-05	C4BPB
28.75793	204.0908	73.0037	11.41057	8.395651	5.289428	3.605766	3.70E-05	-
1329.156	1417.738	1273.915	1166.99	934.0161	942.5761	0.401302	3.70E-05	WIZ
26.06187	30.7127	27.37639	7.261269	4.197825	1.057886	2.744308	3.72E-05	SH2D6
904.0774	966.9546	926.2344	1090.228	1256.199	1239.842	-0.35867	3.72E-05	WDFY3
63.80665	49.53661	59.3155	19.70916	17.84076	22.2156	1.532029	3.72E-05	SH2B2
701.8732	674.6886	643.3451	547.7071	476.4532	460.1803	0.444295	3.73E-05	HAUS5
347.7912	398.2743	365.931	264.5177	255.0179	236.9664	0.55488	3.75E-05	NT5C
79.98299	86.1937	80.30407	41.49297	29.38478	33.85234	1.23388	3.75E-05	LRRC3
27.85924	37.64782	29.20148	8.298593	3.148369	6.347314	2.40953	3.75E-05	AC012358.2
492.4795	512.2085	475.4366	338.1677	337.9249	381.8967	0.48424	3.76E-05	ETHE1
694.6837	756.9194	741.9001	928.4051	973.8955	919.3027	-0.36368	3.77E-05	TMEM39A
975.0735	954.0751	931.7097	815.3368	680.0477	607.2264	0.444036	3.78E-05	AGBL5
388.232	330.9045	389.6572	211.6141	228.7815	282.4555	0.618312	3.80E-05	THUMPD3-AS1
357.6767	344.7748	344.9425	490.6543	523.6787	451.7172	-0.48502	3.81E-05	OTUD3
1827.027	1615.884	1700.074	1311.178	1361.145	1445.072	0.321304	3.81E-05	SLC25A37
7425.836	7528.574	8052.308	5961.502	6653.553	6663.622	0.255104	3.82E-05	NCOA7
235.4555	250.6552	242.7373	173.2331	115.4402	147.0461	0.741658	3.83E-05	SP110
432.2676	411.1539	425.2465	517.6247	658.0091	599.8212	-0.48446	3.85E-05	CREB1
283.0859	294.2475	309.3532	402.4818	406.1396	467.5855	-0.52525	3.87E-05	RPGRIP1L
1460.364	1466.284	1484.713	1253.088	1108.226	1177.427	0.317926	3.88E-05	AURKB
511.3519	477.5329	483.6495	649.3649	662.207	647.426	-0.41146	3.89E-05	TBC1D20
7919.215	8368.715	7878.924	9761.22	9250.958	9134.843	-0.22006	3.89E-05	AP2M1
435.8624	471.5885	456.2731	633.805	618.1298	585.0108	-0.4303	3.89E-05	STK39

1283.323	1226.526	1186.31	1452.254	1613.014	1542.397	-0.31787	3.92E-05	ZNF706
147.3844	170.4059	157.8705	311.1972	216.188	259.182	-0.72672	3.93E-05	FAM104B
27.85924	22.78684	29.20148	7.261269	2.098913	1.057886	2.933799	3.93E-05	NPY4R2
164.4594	151.582	123.1937	80.91128	70.31358	81.4572	0.916514	3.96E-05	-
2879.388	3006.872	3042.429	4334.978	3752.856	3295.314	-0.35049	3.97E-05	RANGAP1
31.45398	30.7127	31.93912	9.335917	3.148369	5.289428	2.402679	3.98E-05	AL078590.3
673.1153	715.3086	818.554	912.8452	1051.555	976.4285	-0.41401	3.98E-05	FAM111A
2385.111	2520.423	2477.563	2825.671	2934.28	3100.663	-0.26328	4.02E-05	CBX1
51.22506	40.62002	37.41439	15.55986	8.395651	10.57886	1.903729	4.02E-05	POLR2A
23.36582	37.64782	17.33838	3.111972	3.148369	4.231543	2.898656	4.05E-05	TRPM3
26.06187	16.84245	18.25092	1.037324	1.049456	2.115771	3.86714	4.05E-05	AL139289.2
89.86853	33.68489	154.2203	36.30634	16.7913	23.27348	1.864045	4.09E-05	AKT3
28.75793	20.80538	31.93912	6.223945	0	4.231543	2.961857	4.11E-05	AC093535.2
3811.324	3816.3	3887.447	3148.279	3257.512	3374.655	0.235613	4.11E-05	MYO1E
386.4347	366.5709	369.5812	476.1318	536.2722	544.8111	-0.47173	4.11E-05	SETDB1
1195.251	1241.387	1133.382	1514.493	1430.409	1484.214	-0.31142	4.12E-05	NUDCD2
2618.769	2659.125	2574.293	3167.988	3100.094	3018.148	-0.24212	4.13E-05	SND1
2536.989	2577.885	2490.339	2999.941	2908.044	3166.252	-0.25485	4.15E-05	PPIB
89.86853	107.9898	86.69189	146.2627	182.6054	180.8985	-0.84273	4.16E-05	ZDHHC23
496.973	473.57	460.8358	648.3276	597.1407	688.6836	-0.43411	4.18E-05	MIS18A
566.1717	626.1427	562.1285	764.5079	753.5097	788.1248	-0.39516	4.18E-05	ZNF330
79.0843	69.35125	66.61587	125.5162	133.281	145.9882	-0.91162	4.23E-05	RPGR
229.1647	238.7665	248.2126	309.1226	384.101	380.8388	-0.58463	4.23E-05	CCPG1
200.4068	239.7572	222.6613	161.8226	110.1929	100.4991	0.83006	4.26E-05	TMED1
598.5244	604.3466	643.3451	767.6199	854.2575	797.6458	-0.38999	4.28E-05	ALS2
20.66976	16.84245	31.93912	3.111972	1.049456	3.173657	3.244435	4.29E-05	MAPK8IP2
296.5661	328.9231	275.589	430.4895	405.0901	461.2382	-0.52618	4.29E-05	LSM1
539.2112	561.7451	571.2539	704.3431	772.3999	725.7096	-0.39745	4.30E-05	LRCH3
113.2343	91.14736	104.0303	47.71691	57.7201	27.50503	1.21428	4.30E-05	ZFYVE28
3863.448	4270.056	4203.188	5291.39	4736.196	4823.959	-0.26784	4.34E-05	STMN1
645.256	636.0501	594.0676	1039.399	840.6145	743.6936	-0.48469	4.35E-05	ARMC6
1147.621	1085.842	1068.592	895.2107	866.8509	851.598	0.337388	4.35E-05	TBC1D8
433.1663	428.987	466.3111	350.6156	290.6994	234.8506	0.600227	4.36E-05	GM2A
2303.33	2484.756	2384.483	2130.664	1820.807	1673.575	0.350365	4.38E-05	TMEM109
343.2978	426.0148	390.5698	260.3684	276.007	248.6031	0.56229	4.42E-05	SIRT5
151.8778	115.9157	115.8934	209.5395	222.4847	211.5771	-0.74475	4.43E-05	ZNF749
230.0634	180.3133	227.224	387.9592	315.8864	299.3816	-0.65262	4.43E-05	BORCS7
17.07502	7.925857	10.95055	0	0	0	5.960046	4.45E-05	LINC00898
1179.974	1137.361	1200.911	903.5093	977.0439	916.129	0.331381	4.47E-05	TSC2
21.56845	21.79611	16.42583	1.037324	0	2.115771	4.247732	4.47E-05	PODNL1
1427.112	1340.461	1311.329	1826.728	1634.004	1628.086	-0.31909	4.49E-05	AUP1
525.7309	508.2456	533.8395	657.6635	690.5423	730.999	-0.40689	4.51E-05	FAM104A
467.3163	500.3197	596.8052	714.7163	768.202	678.1047	-0.46593	4.52E-05	SMIM30
805.222	815.3726	843.1927	1053.921	1026.368	1029.323	-0.33585	4.54E-05	TSG101
728.8338	742.0584	685.3222	536.2966	565.657	533.1744	0.398863	4.55E-05	DAGLB
230.9621	231.8313	237.262	349.5782	312.738	374.4915	-0.5665	4.56E-05	PACRGL
93.46327	123.8415	98.55499	186.7183	197.2978	167.1459	-0.80563	4.56E-05	ZSCAN12
1852.19	1966.603	1881.67	1592.293	1476.585	1597.407	0.288579	4.57E-05	CHTOP
44.03558	32.69416	2.737639	1.037324	2.098913	1.057886	4.243865	4.58E-05	DYRK1B
6471.433	6529.916	6207.139	7972.873	7576.025	7105.818	-0.23814	4.58E-05	ARF1
544.6033	536.9768	515.5886	385.8846	261.3146	406.2281	0.600415	4.63E-05	UQCC2
38.64347	47.55514	38.32694	75.72466	87.10488	123.7726	-1.20423	4.68E-05	-
824.9931	937.2326	877.8695	700.1938	691.5917	604.0527	0.40298	4.72E-05	MAU2
3230.774	3133.686	3059.767	3930.421	3592.289	3680.384	-0.24945	4.72E-05	DYNLL1

92.56458	81.24004	79.39152	41.49297	32.53315	37.026	1.189205	4.73E-05	MLYCD
344.1965	357.6543	288.3646	445.0121	490.0961	479.2222	-0.51495	4.78E-05	ACSL5
162.662	153.5635	152.3952	253.1071	235.0782	245.4295	-0.64634	4.79E-05	LINC01234
17.97371	20.80538	24.63875	3.111972	0	2.115771	3.598006	4.79E-05	GAS6-AS1
1050.563	946.1492	991.0252	862.0164	682.1466	625.2104	0.461782	4.85E-05	RBM38
158.1686	172.3874	173.3838	92.32185	102.8467	95.20971	0.79506	4.85E-05	RNF165
17.07502	18.82391	25.55129	0	0	3.173657	4.285883	4.86E-05	MAN1C1
225.57	226.8777	205.3229	129.6655	145.8744	130.1199	0.697084	4.88E-05	C1orf226
1065.841	1170.055	1001.063	904.5466	802.8341	718.3044	0.415654	4.88E-05	RNASEH2C
745.0101	847.076	783.8772	955.3755	1077.792	1042.017	-0.3727	4.89E-05	SPOP
10.78422	6.935125	18.25092	0	0	0	5.960677	4.89E-05	SMPX
52.12375	60.43466	41.97713	12.44789	14.69239	21.15771	1.677757	4.93E-05	AP001816.1
25.16319	24.7683	29.20148	66.38874	90.25325	52.89428	-1.40421	4.95E-05	FRMD3
961.5932	936.2419	875.1318	693.9698	743.0151	717.2465	0.364261	4.95E-05	OSBPL2
925.6458	998.658	1018.402	800.8142	766.1031	713.0149	0.367843	4.98E-05	BSDC1
195.0147	242.7294	197.11	136.9268	111.2424	128.0042	0.75365	4.99E-05	SEMA4F
124.9173	159.5079	159.6956	291.4881	213.0396	238.0243	-0.74281	5.01E-05	FAHD2P1
62.00928	69.35125	68.44097	119.2923	134.3304	124.8305	-0.92223	5.01E-05	SLC9B2
624.5863	679.6423	603.1931	533.1846	410.3374	368.1442	0.539545	5.03E-05	FAAP100
287.5793	283.3494	277.4141	391.0712	420.832	392.4756	-0.50554	5.03E-05	DENND1B
30.5553	37.64782	25.55129	4.149297	9.445107	4.231543	2.39382	5.06E-05	CCDC33
573.3612	620.1983	586.7672	380.698	422.9309	480.2801	0.471515	5.06E-05	MAP1B
631.7758	654.874	598.6303	519.6994	416.6342	423.1543	0.471209	5.08E-05	WDR54
1387.57	1463.311	1374.295	1206.408	1100.88	1058.944	0.327513	5.08E-05	ASF1B
2187.4	2342.091	2345.244	2685.632	2712.845	2821.381	-0.25795	5.08E-05	TARDBP
193.2173	177.3411	185.2469	293.5627	263.4135	289.8607	-0.60693	5.10E-05	DYRK3
119.5251	136.721	160.6081	87.13523	54.57173	67.70468	0.992663	5.11E-05	C5orf38
149.1818	119.8786	127.7565	208.5022	226.6826	214.7508	-0.71037	5.13E-05	C3orf38
8.088168	19.81464	8.212916	0	0	0	5.960713	5.13E-05	NECAB2
123.1199	86.1937	91.25462	56.0155	35.68152	41.25754	1.177408	5.14E-05	PARD6G
115.9304	100.0639	129.5816	210.5768	186.8032	188.3037	-0.75965	5.17E-05	LHFPL6
3105.856	3195.111	3456.725	2454.309	2816.741	2709.245	0.290181	5.17E-05	LANCL1
406.2057	434.9314	417.0336	624.4691	553.0635	541.6375	-0.45095	5.19E-05	TMEM18
258.8214	294.2475	279.2391	355.8022	427.1287	432.6752	-0.54702	5.22E-05	NFATC3
29.65661	17.83318	20.98856	5.186621	0	0	3.713984	5.27E-05	DUSP13
1358.812	1323.618	1353.306	1158.691	1046.308	906.608	0.375073	5.30E-05	NADK
372.9544	407.1909	426.1591	580.9015	556.2119	519.4219	-0.45794	5.31E-05	ARPC1A
450.2413	422.0519	419.7713	545.6325	593.9923	611.4579	-0.43817	5.32E-05	CPNE8
137.4988	171.3967	166.996	63.27677	68.21466	110.0201	0.978645	5.35E-05	KIAA1211
35.04873	38.63855	50.19004	13.48521	10.49456	9.520971	1.88608	5.35E-05	NCKAP5
91.6659	49.53661	74.82879	32.15705	23.08804	24.33137	1.442639	5.36E-05	PKDCC
5251.018	4682.2	3998.778	2995.792	2071.627	1811.1	1.018209	5.38E-05	MT-ND6
259.72	265.5162	248.2126	202.2782	111.2424	113.1938	0.857226	5.41E-05	CYB561D2
9181.868	9394.123	9328.96	11773.63	11335.18	10077.42	-0.25011	5.44E-05	TRIM28
12030.7	12224.64	11975.34	14482.08	13458.23	13707.02	-0.20105	5.45E-05	HDGF
503.2638	450.7831	510.1133	585.0508	723.0754	714.0728	-0.46505	5.45E-05	VWA8
88.96984	97.09175	87.60444	43.56761	38.82988	44.4312	1.108948	5.45E-05	LAMC3
21.56845	17.83318	19.16347	2.074648	1.049456	1.057886	3.805705	5.47E-05	LINC02320
1527.765	1599.042	1528.515	1355.783	1199.529	1092.796	0.351424	5.49E-05	TBCB
9.885538	16.84245	8.212916	0	0	0	5.914082	5.50E-05	CDH7
1072.132	1026.399	1005.626	1312.215	1266.694	1272.636	-0.31115	5.50E-05	PPARG
132.1067	115.9157	149.6576	198.1289	217.2375	246.4874	-0.73329	5.51E-05	RNFT1
922.9498	937.2326	798.4779	1246.864	1129.215	1083.275	-0.38023	5.54E-05	MKRN2
33.25136	27.7405	31.93912	3.111972	3.148369	10.57886	2.468243	5.54E-05	CDK15

62.90797	64.39759	45.62731	21.78381	23.08804	13.75251	1.559154	5.60E-05	AL713998.1
634.4718	608.3096	597.7178	753.0973	824.8727	817.7456	-0.38014	5.62E-05	RBM19
63.80665	59.44393	70.26606	16.59719	32.53315	21.15771	1.462171	5.63E-05	GDPD1
1419.923	1445.478	1400.758	1783.16	1661.289	1746.569	-0.28323	5.67E-05	RNF216
209.3937	210.0352	219.9236	289.4134	320.0842	346.9865	-0.58081	5.70E-05	LRRC1
35.04873	48.54588	35.5893	112.031	87.10488	74.052	-1.20035	5.79E-05	PUS10
60.21191	57.46247	53.84023	17.63451	22.03858	22.2156	1.471115	5.82E-05	TP73
941.8222	884.7238	946.3104	698.1191	760.8559	700.3203	0.361161	5.82E-05	ARHGAP32
823.1957	834.1965	808.5159	662.8501	607.6352	638.9629	0.368793	5.85E-05	ATOX1
164.4594	218.9518	256.4255	61.20212	25.18695	10.57886	2.721371	5.86E-05	-
768.3759	743.0491	760.151	921.1438	959.2031	1015.57	-0.35005	5.88E-05	DSE
75.48956	192.202	108.593	52.90353	44.07717	56.06794	1.295236	5.91E-05	-
9.885538	9.907322	20.98856	40.45564	47.22554	53.95217	-1.79233	5.92E-05	KLRA1P
56.61717	69.35125	63.87824	20.74648	25.18695	26.44714	1.390717	5.98E-05	CARD9
49.42769	47.55514	79.39152	120.3296	120.6875	122.7147	-1.04226	5.99E-05	AC115618.3
636.2692	601.3744	609.5809	798.7396	808.0814	778.6039	-0.36862	5.99E-05	SECISBP2
343.2978	395.3021	396.9576	239.6219	289.65	236.9664	0.567109	6.00E-05	SCD5
198.6094	216.9703	209.8856	324.6825	299.0951	304.6711	-0.57055	6.00E-05	MMD
232.7595	291.2753	251.8628	406.6311	355.7657	378.7231	-0.55804	6.01E-05	C2CD2L
21.56845	17.83318	20.07602	2.074648	0	0	4.82939	6.05E-05	ZDHHC1
182.4331	213.9982	189.8096	133.8148	109.1435	100.4991	0.770074	6.09E-05	NFKBIE
55.71849	56.47173	51.10259	18.67183	24.1375	10.57886	1.611843	6.12E-05	KIAA1211L
16.17634	8.91659	9.125462	0	0	0	5.88813	6.14E-05	FOXN1
324.4254	332.886	323.0414	497.9156	456.5135	421.0385	-0.48893	6.14E-05	ATG14
433.1663	529.051	502.813	679.4473	659.0586	643.1945	-0.43677	6.15E-05	GALNT11
62.00928	92.13809	71.17861	153.524	155.3195	121.6569	-0.93725	6.15E-05	ZBED8
97.9567	84.21224	83.95425	40.45564	47.22554	19.04194	1.317787	6.18E-05	UQCRFS1P1
38.64347	29.72197	31.02657	9.335917	3.148369	8.463085	2.246705	6.18E-05	AL162231.2
358.5754	335.8582	270.1137	439.8254	479.6016	477.1064	-0.53406	6.19E-05	ADI1
1146.722	1256.248	1209.124	1547.688	1603.569	1386.888	-0.32966	6.21E-05	VANGL1
24.2645	36.65709	34.67676	11.41057	2.098913	2.115771	2.607495	6.24E-05	TMEM198
314.5398	291.2753	324.8665	447.0867	398.7934	480.2801	-0.5102	6.26E-05	WBP4
26.96056	52.50881	34.67676	78.83663	104.9456	86.74663	-1.25034	6.28E-05	-
25.16319	36.65709	21.90111	5.186621	4.197825	5.289428	2.51003	6.29E-05	MAPK8IP1
377.4478	351.7099	353.1554	451.236	557.2613	509.9009	-0.48799	6.30E-05	ZNF260
433.1663	490.4124	474.524	350.6156	332.6777	316.3078	0.483385	6.35E-05	PCTP
1414.531	1543.561	1497.488	1741.667	1813.461	1899.963	-0.29224	6.38E-05	ZNF609
262.4161	274.4328	228.1366	164.9345	167.913	155.5092	0.646825	6.44E-05	BLOC1S3
53.02243	52.50881	60.22805	22.82113	17.84076	17.98406	1.498774	6.44E-05	ZNF514
35.04873	13.87025	38.32694	79.87396	67.16521	90.97817	-1.44093	6.45E-05	KIAA1024
120.4238	109.9713	104.0303	46.67959	57.7201	62.41525	1.004177	6.46E-05	MHENCR
395.4215	399.2651	378.7067	285.2641	257.1168	281.3976	0.510158	6.46E-05	ACAD10
70.09745	68.36052	82.12916	33.19437	31.48369	27.50503	1.258936	6.48E-05	GRTP1
34.15004	28.73123	17.33838	5.186621	0	5.289428	2.936301	6.50E-05	LINC00324
701.8732	780.697	774.7517	928.4051	984.3901	976.4285	-0.35644	6.50E-05	TRAPPC10
585.9428	1698.115	1591.481	267.6296	73.46194	50.77851	3.305787	6.52E-05	-
5.392112	17.83318	11.8631	0	0	0	5.919556	6.60E-05	KRT9
292.9714	242.7294	281.9768	171.1585	151.1217	195.7088	0.659689	6.61E-05	RPL32P3
30.5553	41.61075	28.28893	10.37324	5.247282	6.347314	2.189798	6.68E-05	ALPPL2
342.3991	397.2836	407.9082	517.6247	538.3711	525.7692	-0.46347	6.71E-05	CASK
62.90797	81.24004	53.84023	33.19437	14.69239	21.15771	1.517232	6.71E-05	SOX5
86.27379	84.21224	76.65388	41.49297	34.63206	33.85234	1.167587	6.80E-05	AL162427.1
138.3975	131.7674	133.2317	64.3141	55.62119	88.8624	0.950781	6.82E-05	SORBS2
4.493426	7.925857	7.30037	49.79156	18.89021	30.67868	-2.33846	6.83E-05	AP000786.1

20.66976	18.82391	38.32694	82.98593	59.81901	68.76257	-1.43986	6.85E-05	AC104447.1
49.42769	51.51807	62.05314	13.48521	19.93967	22.2156	1.551928	6.85E-05	PRUNE2
82.67905	61.4254	94.90481	35.26902	26.23641	37.026	1.280151	6.89E-05	HIST1H2BN
191.42	213.0074	184.3343	278.0029	319.0347	291.9764	-0.59547	6.90E-05	IMMP1L
182.4331	167.4337	141.4447	218.8754	290.6994	289.8607	-0.70212	6.91E-05	ASAH2B
32.35267	32.69416	47.4524	68.46339	92.35216	101.557	-1.21997	6.98E-05	AL353743.1
1213.225	1216.619	1189.96	1558.061	1480.783	1414.393	-0.29908	6.98E-05	ATXN1L
32.35267	33.68489	30.11403	6.223945	10.49456	3.173657	2.271897	7.01E-05	CYP4F11
18.87239	11.88879	8.212916	42.53029	43.02771	48.66274	-1.78107	7.01E-05	DNAJC8P1
28.75793	33.68489	32.85166	5.186621	7.346194	8.463085	2.182921	7.02E-05	AC132872.4
614.7007	647.9388	674.3717	452.2733	527.8765	455.9487	0.431622	7.04E-05	ANKS1A
370.2583	373.506	396.9576	516.5874	518.4314	512.0167	-0.43943	7.05E-05	SS18L2
595.8283	612.2725	553.9156	841.2699	734.6194	747.9252	-0.39972	7.07E-05	NSMCE4A
649.7495	683.6052	653.3831	957.4502	803.8836	841.0191	-0.38982	7.07E-05	RPS3AP6
92.56458	71.33272	107.6805	134.8521	181.5559	190.4194	-0.89756	7.08E-05	SMARCE1
1479.236	1367.21	1378.857	1027.988	1104.028	1213.395	0.33721	7.09E-05	VEGFA
851.055	946.1492	993.7628	738.5748	718.8776	696.0888	0.373781	7.09E-05	HMGN3
79.98299	88.17516	62.05314	154.5613	111.2424	190.4194	-0.98809	7.10E-05	SYNRG
267.8082	295.2382	297.4901	473.0198	391.4472	382.9546	-0.53625	7.11E-05	COMMD7
961.5932	1002.621	1013.839	863.0537	689.4928	699.2624	0.403002	7.13E-05	GALE
110.5383	129.7859	100.3801	168.0465	184.7043	248.6031	-0.82087	7.17E-05	PTPMT1
504.1624	516.1715	535.6646	374.474	390.3978	382.9546	0.43897	7.17E-05	ANO10
137.4988	124.8323	105.8554	43.56761	50.3739	80.39931	1.079442	7.17E-05	PRR34-AS1
985.8578	1193.832	1112.394	1739.593	1424.112	1281.1	-0.43366	7.20E-05	ATP5MPL
328.0201	304.1548	307.5281	217.8381	208.8418	211.5771	0.558353	7.21E-05	CARD11
17.07502	17.83318	31.02657	62.23945	51.42336	71.93623	-1.49079	7.21E-05	AC078817.1
274.099	327.9324	292.9273	422.1909	427.1287	416.807	-0.50174	7.24E-05	ZNF26
1767.714	1666.412	1856.119	2015.521	2349.733	2183.476	-0.30756	7.26E-05	REEP3
1051.462	1129.435	1083.192	1299.767	1321.266	1459.882	-0.32244	7.26E-05	TERF2IP
201.3055	180.3133	167.9085	100.6204	97.59944	122.7147	0.776476	7.27E-05	PPFIBP2
2953.979	2891.947	2834.369	3521.715	3444.316	3270.983	-0.238	7.30E-05	DNAJB1
30.5553	29.72197	38.32694	53.94086	107.0445	86.74663	-1.32763	7.36E-05	-
680.3048	696.4847	757.4134	822.598	1001.181	1009.223	-0.4084	7.37E-05	STXBP5
76.38825	69.35125	79.39152	32.15705	23.08804	38.08388	1.271254	7.42E-05	TSPAN1
17.07502	31.70343	15.51329	2.074648	3.148369	1.057886	3.3512	7.43E-05	FAM110D
39.54215	28.73123	28.28893	5.186621	9.445107	6.347314	2.204145	7.48E-05	CCDC189
84.47642	104.0269	101.2926	34.2317	37.78043	58.18371	1.15452	7.53E-05	FRMD4B
477.2019	453.7553	495.5126	568.4536	775.5482	653.7733	-0.48551	7.54E-05	DGKH
26.96056	22.78684	20.98856	1.037324	1.049456	6.347314	3.073696	7.58E-05	FEZ1
14.37896	44.58295	62.05314	4.149297	12.59348	0	2.85188	7.61E-05	PCK2
311.8438	247.683	211.7107	182.569	130.1326	139.6409	0.769833	7.64E-05	STX1A
19.77108	19.81464	16.42583	1.037324	2.098913	0	4.155313	7.65E-05	GDF6
5115.317	4941.772	5358.471	5664.827	6261.057	6412.903	-0.25038	7.65E-05	PICALM
18.87239	23.77757	14.60074	0	1.049456	1.057886	4.76868	7.68E-05	NCALD
185.1292	176.3503	185.2469	122.4042	95.50053	105.7886	0.75621	7.70E-05	TP53I3
32.35267	21.79611	21.90111	6.223945	3.148369	2.115771	2.724961	7.76E-05	NPAS1
2672.69	2625.44	2751.327	3059.069	3253.315	3181.062	-0.2379	7.79E-05	AHCYL1
297.4648	279.3865	310.2657	398.3325	436.5738	413.6333	-0.49238	7.81E-05	CCDC174
620.9915	602.3652	574.9041	749.9854	778.6966	798.7037	-0.37203	7.81E-05	PPP2R2D
1165.595	1096.741	1152.546	1577.77	1416.766	1319.183	-0.33696	7.82E-05	ESPL1
196.8121	166.443	189.8096	267.6296	288.6005	283.5134	-0.60116	7.82E-05	TTC13
55.71849	65.38832	46.53986	119.2923	105.9951	107.9043	-0.99335	7.83E-05	IARS2P1
24.2645	22.78684	39.23949	1.037324	4.197825	8.463085	2.66035	7.91E-05	EXOC3L4
74.59088	98.08249	104.9428	41.49297	43.02771	43.37331	1.117467	7.95E-05	SPOCK2

174.3449	140.684	153.3078	91.28452	95.50053	75.10988	0.839258	8.01E-05	ZDHHC14
7.189482	13.87025	11.8631	0	0	0	5.829034	8.02E-05	AC090114.3
447.5453	422.0519	480.9119	564.3043	644.3662	616.7473	-0.43406	8.03E-05	ZNF587
30.5553	31.70343	35.5893	2.074648	1.049456	12.69463	2.633311	8.04E-05	NGFR
88.96984	50.52734	94.90481	18.67183	33.5826	34.91023	1.430288	8.04E-05	THBS3
8.088168	5.944393	3.650185	22.82113	44.07717	24.33137	-2.36587	8.08E-05	AP000919.1
19691.09	20083.13	19308.57	22985.03	22629.43	21680.31	-0.18779	8.11E-05	TMBIM6
549.0967	566.6988	501.9004	818.4487	665.3553	701.3782	-0.43432	8.11E-05	EIF2B2
546.4007	571.6525	547.5277	385.8846	369.4086	454.8908	0.460751	8.13E-05	SUGP1
778.2615	845.0945	740.9875	665.9621	549.9151	532.1165	0.435098	8.13E-05	FZD8
287.5793	255.6089	272.8513	383.8099	357.8646	442.1962	-0.53597	8.13E-05	TRABD2A
32.35267	23.77757	32.85166	9.335917	0	4.231543	2.711233	8.14E-05	SLC49A3
23.36582	8.91659	21.90111	46.67959	57.7201	63.47314	-1.62296	8.16E-05	C2orf48
71.89482	89.1659	95.81735	133.8148	159.5174	171.3775	-0.85585	8.16E-05	ZBTB8A
46.73163	46.56441	41.06458	12.44789	10.49456	17.98406	1.715558	8.18E-05	AL449212.1
7.189482	15.85171	10.03801	0	0	0	5.834949	8.21E-05	AC018816.1
106.0449	107.9898	114.0683	167.0092	187.8527	191.4773	-0.7354	8.22E-05	INPP5B
23.36582	20.80538	13.68819	0	2.098913	0	4.786084	8.25E-05	AL009178.2
1593.369	1571.301	1726.537	1935.647	2127.248	1929.583	-0.29277	8.25E-05	CBFB
893.2932	806.456	758.3259	1079.854	1005.379	1069.522	-0.35977	8.26E-05	BRPF3
1458.566	1626.782	1647.146	1936.684	1966.681	1880.921	-0.28982	8.34E-05	NEMP1
40.44084	57.46247	57.49041	16.59719	16.7913	19.04194	1.566578	8.39E-05	AMACR
674.9126	613.2632	605.9307	496.8783	470.1564	451.7172	0.41709	8.40E-05	PIAS4
257.9227	253.6274	298.4026	377.586	393.5461	388.244	-0.51684	8.41E-05	ZNF765
72.79351	72.32345	75.74134	33.19437	34.63206	26.44714	1.227944	8.42E-05	RPL23AP42
17.07502	16.84245	23.7262	0	0	3.173657	4.193794	8.42E-05	AZIN2
177.9397	183.2855	172.4712	127.5909	87.10488	87.80451	0.818253	8.46E-05	FBXL15
1672.453	1209.684	1571.405	1698.1	2167.127	2280.802	-0.46411	8.50E-05	NKTR
31.45398	29.72197	20.07602	5.186621	4.197825	5.289428	2.468653	8.57E-05	KMT2E-AS1
161.7634	189.2298	182.5092	246.8831	299.0951	275.0503	-0.62271	8.57E-05	SIRPA
460.1269	393.3207	441.6724	261.4057	321.1336	317.3657	0.526193	8.58E-05	CAMK2D
62.00928	59.44393	52.92768	15.55986	15.74185	29.6208	1.518383	8.59E-05	RIPOR2
980.4656	1010.547	1031.177	1158.691	1286.633	1389.004	-0.3433	8.60E-05	HINT3
1046.968	1037.297	1054.903	1202.259	1348.551	1375.251	-0.32253	8.65E-05	AC004057.1
210.2924	181.304	180.6842	96.47114	97.59944	136.4673	0.792882	8.68E-05	HIST2H2BE
63.80665	70.34198	73.0037	33.19437	29.38478	21.15771	1.305685	8.72E-05	TRAPPC3L
13.48028	15.85171	4.562731	0	0	0	5.870944	8.74E-05	OVOL1
15.27765	18.82391	22.81366	2.074648	0	0	4.765134	8.80E-05	AL355312.1
376.5491	343.7841	372.3189	476.1318	489.0467	534.2323	-0.45581	8.86E-05	CRYZL1
880.7116	887.696	910.7211	1169.064	1134.462	1054.712	-0.326	8.87E-05	HSD17B11
4337.055	4501.887	4235.127	5371.264	4963.929	4986.873	-0.22905	8.87E-05	PTTG1IP
1241.983	1258.23	1273.915	1086.078	989.6373	921.4184	0.332391	8.91E-05	MAFG
57.51586	62.41613	44.71476	6.223945	26.23641	15.86829	1.768993	8.94E-05	LRRC75A
112.3357	140.684	144.1823	71.57537	77.65977	61.35737	0.914495	8.95E-05	TMC7
191.42	248.6738	227.224	336.093	333.7271	322.6551	-0.5742	8.98E-05	NDUFAF5
610.2073	618.2169	542.0525	462.6466	387.2494	443.2541	0.452891	8.98E-05	GATA2-AS1
49.42769	67.36979	59.3155	23.85846	17.84076	23.27348	1.437089	9.04E-05	CHST2
141.9923	153.5635	158.783	99.58312	82.90705	68.76257	0.853846	9.05E-05	ADIPOR2
485.2901	587.5042	480.9119	402.4818	366.2603	312.0763	0.522478	9.06E-05	BAHD1
16.17634	64.39759	21.90111	5.186621	7.346194	4.231543	2.607051	9.07E-05	UPK3B
179.7371	219.9425	177.034	141.0761	55.62119	83.57297	1.039725	9.08E-05	B9D2
29.65661	35.66636	38.32694	12.44789	3.148369	7.4052	2.16976	9.14E-05	RIMS3
97.05801	97.09175	87.60444	41.49297	43.02771	50.77851	1.058409	9.22E-05	NBPF3
1667.96	1661.458	1675.435	1348.521	1390.53	1409.104	0.27093	9.24E-05	HIF1AN

44.03558	35.66636	18.25092	79.87396	75.56086	88.8624	-1.31876	9.26E-05	FAM200A
23.36582	31.70343	23.7262	40.45564	85.00596	93.09394	-1.47411	9.31E-05	XPOTP1
1987.892	2059.732	2105.244	1839.176	1580.481	1511.719	0.319104	9.33E-05	WDR6
15.27765	16.84245	24.63875	0	0	2.115771	4.757149	9.34E-05	MYO15A
30.5553	49.53661	24.63875	5.186621	12.59348	3.173657	2.31862	9.34E-05	MYO7A
21.56845	20.80538	20.98856	1.037324	2.098913	4.231543	3.108421	9.40E-05	-
413.3952	443.848	399.6952	571.5656	531.0249	591.3581	-0.43106	9.41E-05	NECAP1
867.2313	863.9185	852.3182	676.3353	684.2455	677.0468	0.342406	9.42E-05	SMURF1
67.4014	91.14736	82.12916	35.26902	32.53315	38.08388	1.183368	9.44E-05	-
16.17634	22.78684	20.98856	2.074648	2.098913	2.115771	3.251688	9.45E-05	RHOH
14.37896	16.84245	15.51329	0	0	1.057886	5.373873	9.48E-05	AC008440.1
101.5514	109.9713	94.90481	52.90353	55.62119	42.31543	1.021532	9.49E-05	AC009133.1
575.1586	605.3374	621.444	703.3058	880.4939	816.6877	-0.41377	9.50E-05	LRBA
24.2645	13.87025	26.46384	3.111972	3.148369	1.057886	3.141908	9.55E-05	MT1F
374.7518	367.5616	418.8587	477.1691	585.5966	551.1584	-0.47446	9.60E-05	TET2
165.3581	145.6376	144.1823	91.28452	56.67064	94.15183	0.911145	9.60E-05	CNPY4
27.85924	20.80538	24.63875	2.074648	5.247282	4.231543	2.668532	9.62E-05	APOBEC3G
59.31323	60.43466	68.44097	33.19437	10.49456	20.09983	1.559726	9.64E-05	WFIKKN1
60.21191	81.24004	54.75277	23.85846	26.23641	26.44714	1.356254	9.65E-05	FSD1
79.98299	60.43466	102.2052	14.52254	38.82988	35.96811	1.444148	9.66E-05	LINC00342
88.96984	96.10102	72.09115	45.64226	38.82988	28.56291	1.184266	9.68E-05	PCED1B
1737.159	1842.762	1605.169	1480.262	1385.282	1275.81	0.323872	9.71E-05	TOB2
24.2645	27.7405	29.20148	5.186621	1.049456	7.4052	2.574249	9.71E-05	CLIC5
28.75793	27.7405	31.02657	7.261269	7.346194	3.173657	2.298017	9.72E-05	ACTBL2
282.1872	312.0806	288.3646	214.7261	200.4462	154.4513	0.630917	9.74E-05	SHISAL1
66.50271	56.47173	54.75277	26.97043	14.69239	23.27348	1.452634	9.74E-05	SMIM3
675.8113	777.7248	795.7403	960.5622	951.8569	978.5442	-0.36251	9.81E-05	WWC3
173.4463	214.9889	214.4484	281.1148	328.4798	303.6132	-0.59997	9.87E-05	ALG9
20.66976	24.7683	26.46384	2.074648	6.296738	2.115771	2.777477	9.89E-05	C1QTNF12
232.7595	281.3679	238.1746	357.8768	377.8043	353.3338	-0.53508	9.89E-05	NKIRAS1
107.8422	106.0083	79.39152	157.6733	165.8141	188.3037	-0.80406	9.89E-05	THNSL1
494.2769	445.8295	412.4709	322.6078	303.2929	336.4076	0.491399	9.93E-05	MMP24OS
603.9165	675.6793	699.0104	940.853	885.7412	784.9512	-0.40084	9.94E-05	LIG1
117.7278	93.12882	97.64245	177.3824	150.0723	214.7508	-0.81209	9.96E-05	-
35.94741	53.49954	109.5055	9.335917	6.296738	31.73657	2.072534	0.0001001	TINF2
116.8291	93.12882	82.12916	162.8599	174.2098	171.3775	-0.79869	0.0001004	FAM72A
17.07502	17.83318	21.90111	1.037324	0	3.173657	3.758764	0.0001007	ATP2B2
58.41454	50.52734	73.0037	15.55986	22.03858	28.56291	1.461714	0.000102	FLNB-AS1
9.885538	19.81464	27.37639	1.037324	0	1.057886	4.766403	0.0001021	AL135838.1
385.536	356.6636	421.5964	490.6543	582.4483	536.348	-0.46692	0.0001021	POC1B
69.19877	62.41613	72.09115	21.78381	33.5826	27.50503	1.298694	0.0001026	COL15A1
8952.703	8714.48	8577.934	10989.41	10123.06	9638.396	-0.22859	0.0001027	PTBP1
35.94741	40.62002	26.46384	4.149297	12.59348	6.347314	2.157541	0.0001033	AP002851.1
8826.887	9327.743	8989.493	8354.609	7288.474	6907.993	0.267363	0.0001037	NQO1
43.13689	48.54588	35.5893	5.186621	1.049456	19.04194	2.334162	0.0001038	ARHGEF37
999.338	974.8805	1100.531	1234.416	1280.337	1327.647	-0.32116	0.0001039	SLU7
84.47642	93.12882	85.77934	45.64226	41.97825	35.96811	1.090613	0.0001044	NUDT3
452.0387	468.6163	424.334	598.536	580.3494	605.1106	-0.40796	0.0001049	CCNDBP1
1713.793	1762.513	1674.522	1488.56	1310.771	966.9075	0.451457	0.0001052	CHPF
735.1246	658.8369	675.2842	512.4381	508.9863	552.2163	0.395511	0.0001055	CAMLG
687.4942	753.9472	712.6986	859.9417	959.2031	937.2867	-0.35609	0.0001055	SPG11
911.2669	990.7322	980.0746	1163.878	1197.43	1216.569	-0.31229	0.0001059	GTF2H3
2670.893	2836.466	2604.407	3254.086	3157.814	3156.731	-0.23857	0.0001061	RAF1
56.61717	33.68489	64.79078	6.223945	13.64293	23.27348	1.850067	0.0001062	AL031123.2

54.8198	46.56441	41.06458	15.55986	14.69239	16.92617	1.594782	0.0001065	AC092811.1
173.4463	170.4059	196.1974	261.4057	278.1059	275.0503	-0.59232	0.0001071	ACTR3B
228.2661	178.3318	230.8742	262.443	338.9744	478.1643	-0.75866	0.0001071	-
6006.812	5735.349	6205.314	6786.175	7313.661	6827.594	-0.2215	0.0001074	6-Mar
53.02243	63.40686	48.36495	107.8817	116.4897	99.44125	-0.97676	0.0001082	AC126474.2
713.5561	786.6413	774.7517	930.4798	965.4998	984.8916	-0.34103	0.0001084	WDR48
24.2645	22.78684	30.11403	4.149297	6.296738	3.173657	2.502605	0.0001092	IL20RA
1316.574	1244.36	1453.686	1682.54	1619.311	1658.765	-0.30485	0.0001094	DCTN4
35.94741	27.7405	31.93912	2.074648	12.59348	1.057886	2.605154	0.0001096	AC092368.3
82.67905	75.29565	93.07971	127.5909	179.457	148.104	-0.85702	0.0001096	CLN5
53.02243	61.4254	61.1406	11.41057	25.18695	25.38926	1.503062	0.0001096	PCOLCE
1158.405	1175.999	1098.706	1402.462	1406.272	1384.772	-0.28886	0.0001097	ERCC3
487.0874	429.9778	465.3986	375.5113	301.194	228.5033	0.610855	0.0001111	HPS6
437.6597	432.95	481.8244	349.5782	308.5402	314.192	0.476187	0.0001117	RPAP1
86.27379	66.37906	99.46754	132.7775	149.0228	186.1879	-0.88972	0.0001121	AL355596.1
17.97371	22.78684	30.11403	5.186621	2.098913	3.173657	2.758592	0.0001128	FAM167B
38.64347	30.7127	44.71476	75.72466	78.70923	99.44125	-1.15121	0.0001141	RPL21P28
115.0317	98.08249	111.3306	43.56761	67.16521	49.72063	1.016897	0.0001144	CDK5RAP3
50.32638	57.46247	52.01513	20.74648	23.08804	10.57886	1.55261	0.000115	FOXD2
122.2212	116.9064	120.4561	67.42607	70.31358	51.8364	0.923402	0.0001152	EFNA4
213.8871	221.924	199.8476	122.4042	136.4293	140.6988	0.669763	0.0001152	CREBRF
877.1168	940.2048	930.7971	1121.347	1153.353	1132.996	-0.31063	0.0001154	ANKRD13A
22.46713	11.88879	20.07602	1.037324	0	1.057886	4.70095	0.0001155	WT1
232.7595	281.3679	278.3266	356.8395	389.3483	393.5335	-0.52507	0.0001155	ITFG2
363.9675	402.2373	341.2923	637.9543	483.7994	474.9907	-0.52873	0.0001155	ORMDL3
458.3295	448.8017	482.7369	587.1255	616.0309	631.5577	-0.40023	0.0001159	ELMOD2
21.56845	22.78684	24.63875	2.074648	4.197825	4.231543	2.717409	0.000116	SERPING1
612.0047	613.2632	640.6074	845.4192	765.0537	788.1248	-0.36231	0.0001161	TRIM14
3208.306	3287.249	3573.531	3815.278	4112.819	4022.081	-0.24703	0.0001163	LIFR
234.5569	251.646	208.0605	337.1303	349.469	323.713	-0.54221	0.0001165	ATP5S
666.8245	624.1613	641.52	803.9262	844.8124	816.6877	-0.35099	0.0001167	MEX3C
690.1903	722.2438	767.4514	845.4192	978.0933	1009.223	-0.37783	0.0001168	AGL
371.157	409.1724	417.0336	497.9156	582.4483	551.1584	-0.44661	0.0001178	TULP4
17.07502	13.87025	22.81366	2.074648	1.049456	0	4.09904	0.0001183	AC027682.6
1129.647	966.9546	1014.751	730.2762	801.7846	866.4084	0.37603	0.0001186	IQGAP3
16.17634	20.80538	17.33838	0	0	2.115771	4.692348	0.0001188	AQP7
83.57773	99.07322	94.90481	37.34367	56.67064	31.73657	1.141456	0.0001189	C11orf45
4915.808	4692.108	4625.697	4290.373	3891.384	3404.276	0.29688	0.0001191	LRRC75A-AS1
530.2243	536.9768	591.3299	657.6635	764.0042	781.7775	-0.40953	0.0001192	DDHD1
180.6357	175.3596	140.5321	282.1522	240.3255	252.8347	-0.64349	0.0001193	LYRM1
496.973	492.3939	517.4137	644.1783	642.2673	680.2205	-0.38405	0.0001195	TAOK3
355.8794	349.7285	351.3303	154.5613	243.4739	264.4714	0.674429	0.0001195	DOCK11
50.32638	37.64782	38.32694	9.335917	13.64293	14.8104	1.742874	0.0001199	STOX2
1403.746	1581.209	1457.336	1302.879	1121.869	1006.049	0.3723	0.0001199	B4GALT2
1035.285	1044.232	1058.554	1196.035	1402.074	1326.589	-0.32262	0.0001203	GLCE
4301.108	4350.305	4420.374	5065.254	5032.143	4930.805	-0.20123	0.0001203	DNMT1
18.87239	29.72197	18.25092	5.186621	2.098913	1.057886	2.995383	0.0001208	GGN
290.2753	341.8026	287.4521	225.0993	201.4956	183.0142	0.591784	0.0001216	CCDC115
44.03558	41.61075	27.37639	3.111972	15.74185	6.347314	2.165794	0.0001218	SLC23A1
1395.658	1365.229	1421.747	1168.027	1127.116	1140.401	0.283992	0.0001225	MAP2K1
478.9993	477.5329	440.7598	657.6635	555.1624	673.8732	-0.4334	0.0001228	MACO1
8.088168	4.953661	10.03801	40.45564	43.02771	19.04194	-2.14528	0.0001236	LYPLA1P3
239.949	234.8035	219.0111	154.5613	150.0723	143.8725	0.629127	0.0001236	MPZL3
1583.483	1715.948	1753.914	2261.367	2200.71	1861.879	-0.3238	0.0001241	UNG

563.4757	554.81	524.7141	384.8473	444.9695	382.9546	0.437982	0.0001244	SLC50A1
17.07502	20.80538	27.37639	3.111972	4.197825	1.057886	2.961347	0.0001249	TMEM105
299.2622	287.3123	321.2163	462.6466	453.3651	374.4915	-0.50722	0.0001251	GINS4
71.89482	75.29565	67.52842	163.8972	115.4402	121.6569	-0.9021	0.0001253	-
2117.303	2260.851	2062.354	1915.938	1685.427	1595.292	0.309266	0.0001255	NDUFV1
62.90797	55.481	52.92768	88.17255	123.8358	128.0042	-0.98796	0.0001257	U91328.1
6.290797	11.88879	12.77565	0	0	0	5.740712	0.0001263	SLC43A2
24.2645	42.60148	28.28893	6.223945	9.445107	5.289428	2.179943	0.0001265	PON3
67.4014	55.481	61.1406	33.19437	13.64293	19.04194	1.481448	0.0001267	AC107375.1
29.65661	60.43466	44.71476	108.919	86.05542	101.557	-1.14228	0.0001267	ARL14EPL
893.2932	885.7146	889.7326	652.4769	710.4819	735.2305	0.347191	0.0001271	-
683.8995	690.5403	735.5123	832.9713	903.5819	988.0652	-0.3686	0.0001276	C2CD3
346.8925	328.9231	289.2772	208.5022	195.1989	242.2558	0.579351	0.0001277	AGPAT4
47.63032	64.39759	52.01513	20.74648	16.7913	22.2156	1.45548	0.0001277	-
141.0936	145.6376	112.2432	64.3141	76.61031	75.10988	0.884618	0.0001281	SLC1A4
40.44084	32.69416	28.28893	6.223945	8.395651	10.57886	2.010729	0.0001284	ANO7
160.8647	145.6376	186.1594	241.6965	238.2266	296.208	-0.65438	0.0001287	PTER
1338.142	1350.368	1281.215	1591.255	1600.421	1596.349	-0.27053	0.0001288	SAMM50
970.5801	1044.232	919.8466	1179.438	1247.804	1230.321	-0.31818	0.000129	PHF12
351.3859	361.6172	335.817	481.3184	440.7717	519.4219	-0.45909	0.0001292	ARL14
81.78036	60.43466	83.95425	31.11972	25.18695	39.14177	1.246589	0.0001296	AC132872.1
34.15004	76.28638	57.49041	21.78381	6.296738	20.09983	1.798802	0.0001297	-
316.3372	362.608	356.8056	459.5346	460.7113	504.6115	-0.46071	0.0001302	FOCAD
45.83295	64.39759	24.63875	11.41057	10.49456	14.8104	1.875051	0.0001302	PLCD1
140.1949	138.7025	143.2698	242.7338	207.7924	211.5771	-0.64925	0.0001305	C8orf37
110.5383	134.7396	102.2052	52.90353	66.11575	60.29948	0.953273	0.0001306	PCYOX1L
17.07502	16.84245	10.95055	1.037324	0	0	5.315047	0.0001307	DUOX1
17.07502	16.84245	10.95055	1.037324	0	0	5.315047	0.0001307	PRR36
328.9188	288.3031	322.1288	237.5472	199.3967	194.651	0.573065	0.0001317	PLK3
73.69219	67.36979	73.0037	118.255	153.2206	121.6569	-0.8761	0.0001318	ZNF519
314.5398	385.3948	374.1439	421.1536	565.657	577.6056	-0.54301	0.0001327	TMEM67
149.1818	145.6376	165.1709	243.7712	246.6222	220.0402	-0.62666	0.0001334	SNX10
1004.73	1076.926	929.8846	775.9185	811.2298	783.8933	0.344519	0.0001336	GBA2
67.4014	75.29565	60.22805	20.74648	36.73097	23.27348	1.32885	0.0001339	C1QTNF1-AS1
92.56458	115.9157	104.9428	179.4571	167.913	174.5511	-0.7374	0.0001343	EEF1AKMT1
9.885538	13.87025	12.77565	24.89578	38.82988	71.93623	-1.89466	0.0001349	SLC6A14
20.66976	29.72197	13.68819	0	5.247282	0	3.609466	0.0001351	AC233724.15
518.5414	520.1344	525.6266	383.8099	357.8646	416.807	0.433391	0.0001362	TRIM2
797.1338	858.9648	886.9949	1017.615	1073.594	1105.491	-0.33012	0.0001365	NEO1
184.2305	190.2206	195.2849	280.0775	284.4027	275.0503	-0.55939	0.0001372	ALG6
39.54215	36.65709	45.62731	13.48521	13.64293	9.520971	1.733021	0.0001374	PRKAR2A-AS1
130.3094	115.9157	131.4067	86.0979	46.17608	57.12583	0.995374	0.0001381	ROBO3
73.69219	77.27711	62.05314	20.74648	15.74185	42.31543	1.435404	0.0001382	-
257.9227	249.6645	311.1783	360.9888	405.0901	415.7491	-0.52864	0.0001382	GIT2
75.48956	85.20297	89.42953	150.412	143.7755	140.6988	-0.7986	0.000139	ZNF17
807.0194	810.4189	838.63	992.7192	1038.962	1026.149	-0.31605	0.0001391	SNX30
229.1647	221.924	261.9008	338.1677	321.1336	380.8388	-0.5441	0.0001395	CACHD1
37.74478	17.83318	24.63875	4.149297	7.346194	0	2.802619	0.0001398	MYO1F
10.78422	22.78684	23.7262	43.56761	65.06629	58.18371	-1.54546	0.0001399	ZNF556
240.8477	236.785	231.7867	446.0494	335.826	300.4395	-0.60971	0.0001406	SLC25A19
170.7502	180.3133	330.3417	62.23945	14.69239	12.69463	2.925922	0.0001407	-
10.78422	9.907322	9.125462	0	0	0	5.68799	0.0001416	FZD9
19.77108	28.73123	19.16347	1.037324	6.296738	1.057886	3.010305	0.0001423	EFHD1
17515.38	17510.2	18302.94	19082.61	21379.52	21819.95	-0.22389	0.0001427	SLC38A2

5274.384	5557.017	5241.665	4930.402	4267.09	4212.501	0.261199	0.0001431	COX4I1
899.584	929.3068	1006.538	1142.094	1227.864	1166.848	-0.31879	0.0001436	MTF2
331.6149	392.3299	406.0831	510.3635	573.0032	485.5695	-0.474	0.0001439	ERCC4
28.75793	32.69416	34.67676	9.335917	3.148369	9.520971	2.12666	0.0001443	PRSS53
10.78422	8.91659	10.03801	0	0	0	5.684761	0.0001443	AC245100.8
17.97371	23.77757	13.68819	0	3.148369	1.057886	3.721932	0.0001444	SMIM6
38.64347	52.50881	44.71476	15.55986	11.54402	16.92617	1.624306	0.0001449	VAMP5
168.0541	185.2669	164.2583	123.4416	69.26412	94.15183	0.850463	0.0001469	TPST2
168.0541	112.9435	135.0568	68.46339	79.75868	76.16777	0.892601	0.0001472	ZNF341
102.4501	81.24004	94.90481	145.2254	165.8141	167.1459	-0.77744	0.0001472	AC004704.1
21.56845	21.79611	30.11403	4.149297	0	6.347314	2.809101	0.0001485	AC084125.2
333.4122	258.5811	279.2391	375.5113	430.2771	455.9487	-0.53292	0.0001488	DNAJC24
28.75793	38.63855	22.81366	3.111972	8.395651	7.4052	2.253784	0.0001492	MELTF-AS1
169.8515	157.5264	148.745	220.95	292.7983	234.8506	-0.65256	0.0001494	PIGB
325.3241	390.3485	397.8702	654.5515	574.0526	426.3279	-0.57229	0.0001495	TSPAN5
255.2266	231.8313	208.9731	155.5986	158.4679	120.599	0.679077	0.0001499	ST6GALNAC4
13.48028	15.85171	39.23949	1.037324	5.247282	1.057886	3.225049	0.0001506	-
165.3581	134.7396	154.2203	203.3155	280.2048	244.3716	-0.67854	0.0001508	ANKRD26
21.56845	14.86098	16.42583	1.037324	1.049456	2.115771	3.656508	0.0001513	ASGR1
806.1207	769.7989	840.4551	954.3382	1062.05	1030.381	-0.334	0.0001514	PDPR
1011.92	1099.713	1073.154	1275.909	1453.497	1257.826	-0.32448	0.0001514	ZNF367
7.189482	9.907322	12.77565	0	0	0	5.690313	0.0001514	AL161668.1
1285.12	1284.98	1299.466	1054.959	1107.176	942.5761	0.317643	0.0001518	SETD1B
28.75793	32.69416	26.46384	2.074648	9.445107	6.347314	2.30007	0.0001523	AC131212.3
37.74478	47.55514	46.53986	13.48521	14.69239	14.8104	1.616124	0.0001527	LINC00869
38.64347	20.80538	29.20148	39.41832	90.25325	124.8305	-1.51739	0.0001528	RBMS2P1
847.4602	933.2697	880.6071	780.0678	603.4374	494.0326	0.502823	0.0001529	TELO2
1552.029	1651.551	1635.283	1797.683	2010.758	2164.434	-0.30381	0.0001536	YLPM1
325.3241	305.1455	345.855	431.5268	448.1179	460.1803	-0.45588	0.0001542	AC138392.1
146.4857	97.09175	114.9808	47.71691	66.11575	66.6468	0.992467	0.0001544	ASB16-AS1
363.0689	369.5431	438.0222	300.824	267.6114	232.7348	0.547082	0.0001544	FIZ1
459.2282	417.0982	403.3454	560.155	563.5581	579.7213	-0.41228	0.0001546	FANCA
4239.997	4234.389	4087.295	3770.673	3518.827	3095.373	0.274453	0.0001547	PXN
68.30008	48.54588	62.05314	28.00775	24.1375	12.69463	1.464379	0.0001553	FBXL8
1964.526	1960.659	1842.431	1719.883	1515.415	1386.888	0.319204	0.0001556	GSTO1
600.3218	568.6803	681.672	782.1424	841.664	789.1827	-0.38216	0.0001573	ELF2
25.16319	14.86098	18.25092	1.037324	2.098913	3.173657	3.212274	0.0001575	AL022328.4
257.024	286.3216	273.7639	339.205	422.9309	408.3439	-0.51892	0.0001575	PEX1
281.2885	317.0343	336.7296	425.3029	425.0298	448.5435	-0.47445	0.0001577	KIAA1841
3459.04	3420.998	3642.885	4032.079	4084.484	4084.497	-0.21336	0.0001577	RTN3
1460.364	1448.45	1552.241	1706.398	1903.714	1806.869	-0.27991	0.0001579	USO1
409.8005	376.4782	367.7561	269.7043	294.8972	250.7189	0.501442	0.0001582	NOCT
42.23821	41.61075	34.67676	8.298593	17.84076	4.231543	1.963645	0.0001583	CAPS
24.2645	25.75904	27.37639	7.261269	4.197825	3.173657	2.400765	0.0001585	GADD45G
1383.077	1659.476	1462.812	1988.55	1734.751	1914.773	-0.32414	0.0001587	SNRPG
3612.715	3850.976	3800.755	3238.526	3294.243	3115.473	0.223288	0.0001588	MLEC
36.8461	45.57368	27.37639	8.298593	6.296738	13.75251	1.953	0.0001594	TUBB2B
148.2831	114.9249	147.8325	198.1289	217.2375	249.661	-0.69202	0.0001598	RNPC3
108.7409	106.0083	102.2052	183.6064	182.6054	156.5671	-0.72207	0.0001602	HENMT1
3.594741	6.935125	8.212916	31.11972	20.98913	34.91023	-2.21859	0.0001602	AL133520.1
43.13689	68.36052	49.2775	25.9331	11.54402	15.86829	1.588396	0.0001611	GAS7
220.1779	211.026	182.5092	140.0388	121.7369	119.5411	0.686171	0.0001616	SNN
1046.07	981.8156	1056.729	911.8079	694.7401	537.4059	0.524687	0.0001619	COMMD4
17.97371	15.85171	19.16347	0	0	3.173657	4.072205	0.000162	RIMBP3

577.8546	568.6803	602.2805	774.8811	706.2841	769.0829	-0.36351	0.0001624	C12orf65
24.2645	15.85171	32.85166	69.50072	65.06629	55.01006	-1.3727	0.0001627	SEMA5A
85.3751	89.1659	80.30407	139.0014	155.3195	143.8725	-0.78254	0.0001632	LDAH
28.75793	22.78684	16.42583	4.149297	3.148369	3.173657	2.697914	0.0001632	EFNA2
1307.587	1366.22	1304.941	1619.263	1583.63	1582.597	-0.26663	0.0001632	TRAM2
51.22506	66.37906	25.55129	11.41057	19.93967	6.347314	1.922601	0.0001635	-
23.36582	33.68489	33.76421	8.298593	6.296738	6.347314	2.114612	0.0001637	AL390957.1
648.8508	679.6423	689.8849	464.7212	536.2722	535.2902	0.393802	0.0001639	FKBP5
64.70534	55.481	67.52842	23.85846	32.53315	15.86829	1.377647	0.000164	ADM5
797.1338	781.6877	874.2193	1132.758	1004.33	988.0652	-0.34918	0.0001645	MRPL27
34.15004	39.62929	33.76421	9.335917	13.64293	5.289428	1.926061	0.000165	LINC00525
1421.72	1380.09	1383.42	1198.109	1078.841	1144.632	0.290679	0.0001654	NQO2
336.1083	330.9045	324.8665	439.8254	499.5412	425.27	-0.46027	0.0001655	PKNOX1
68.30008	100.0639	83.04171	31.11972	46.17608	34.91023	1.162429	0.0001657	GPR37
413.3952	429.9778	405.1705	518.6621	566.7064	575.4898	-0.41192	0.0001658	RMND1
27.85924	30.7127	22.81366	6.223945	2.098913	7.4052	2.37086	0.000166	PROM2
400.8136	443.848	397.8702	590.2374	568.8053	512.0167	-0.42835	0.0001674	SDF2
430.4702	411.1539	494.6001	571.5656	646.4651	585.0108	-0.43162	0.0001677	DYNC2H1
245.3411	231.8313	237.262	179.4571	142.7261	133.2936	0.649228	0.000168	GPATCH3
401.7123	409.1724	438.9347	283.1895	329.5293	281.3976	0.483293	0.0001697	ORMDL2
843.8655	809.4282	884.2573	1010.354	1107.176	1056.828	-0.32263	0.0001703	SLC35F5
35.04873	37.64782	41.06458	9.335917	11.54402	12.69463	1.761216	0.0001717	NTRK1
3134.614	3370.471	3285.166	2909.694	2746.427	2490.263	0.26498	0.0001719	PRDX2
583.2467	605.3374	631.482	837.1206	761.9053	744.7515	-0.365	0.000172	LIMD1
1262.653	1173.027	1133.382	932.5544	1020.072	886.5082	0.330208	0.0001721	NBEAL2
2100.227	2114.222	2185.548	1712.622	1884.824	1756.09	0.25764	0.0001723	BRD4
140.1949	140.684	156.9579	106.8444	62.96738	46.54697	1.016084	0.0001727	GNB1L
1153.013	1180.953	1164.409	1020.727	906.7303	843.1349	0.33623	0.0001728	FXR2
2371.63	2567.978	2416.422	3148.279	2702.35	3031.9	-0.27228	0.000174	PFDN5
87.17247	64.39759	69.35351	124.4789	147.9733	129.0621	-0.85986	0.0001742	EFCAB2
1815.344	1935.891	1800.454	2237.508	2195.463	2161.26	-0.24862	0.0001745	PRDX4
415.1926	387.3763	340.3797	292.5254	261.3146	235.9085	0.533026	0.0001751	TMSB4XP8
33.25136	21.79611	20.07602	7.261269	2.098913	3.173657	2.581809	0.0001754	RARRES2
44.93426	49.53661	37.41439	21.78381	5.247282	8.463085	1.890564	0.0001759	GCNT3
59.31323	57.46247	41.97713	15.55986	16.7913	24.33137	1.486206	0.0001761	DLX4
17.97371	26.74977	20.07602	52.90353	49.32445	67.70468	-1.39481	0.0001767	LNX1
593.1323	662.7998	688.0598	489.617	487.9972	500.3799	0.395119	0.0001768	TBC1D9
21.56845	15.85171	15.51329	0	2.098913	2.115771	3.657045	0.000177	MT-TL2
7.189482	11.88879	10.03801	0	0	0	5.652046	0.0001771	GAL3ST1
44.93426	56.47173	68.44097	8.298593	29.38478	16.92617	1.637824	0.0001775	RTEL1-TNFRSF6B
636.2692	718.2808	675.2842	835.0459	892.0379	855.8295	-0.34824	0.0001781	CEP85
15.27765	11.88879	3.650185	0	0	0	5.735109	0.0001791	FOXH1
370.2583	334.8675	413.3834	480.2811	495.3434	583.9529	-0.47858	0.0001797	RIC8B
157.2699	198.1464	197.11	263.4803	250.8201	374.4915	-0.68639	0.0001799	ZNF510
61.1106	47.55514	44.71476	130.7028	87.10488	96.2676	-1.03303	0.0001805	HS3ST1
132.1067	145.6376	111.3306	201.2409	231.9299	193.5931	-0.68924	0.000181	FPGT
17.97371	15.85171	9.125462	1.037324	0	0	5.252289	0.0001823	EXOC3L1
67.4014	87.18443	110.4181	54.97818	19.93967	28.56291	1.355347	0.0001826	-
867.2313	894.6312	925.3219	1124.459	1136.561	1064.233	-0.30745	0.0001827	CHMP3
40.44084	38.63855	28.28893	9.335917	13.64293	3.173657	2.035882	0.0001829	AL139220.2
44.03558	24.7683	28.28893	9.335917	8.395651	5.289428	2.077274	0.0001831	AC008894.3
4131.256	4479.1	4382.047	5054.881	4938.742	5034.478	-0.21016	0.0001834	IMPDH2
23.36582	13.87025	21.90111	3.111972	3.148369	0	3.237442	0.0001837	NKX6-1
18.87239	19.81464	22.81366	2.074648	4.197825	2.115771	2.874616	0.0001843	GLI1

312.7425	323.9694	336.7296	457.4599	442.8706	425.27	-0.44562	0.0001844	SERINC5
1392.962	1373.155	1346.918	1717.809	1650.795	1584.713	-0.26825	0.0001861	COPS6
810.6141	882.7424	832.2422	1087.116	993.8352	1075.87	-0.32232	0.0001866	NOB1
382.8399	272.4513	373.2314	152.4866	241.375	238.0243	0.704224	0.0001883	AC015813.1
125.8159	105.0176	102.2052	39.41832	57.7201	68.76257	1.00676	0.0001888	TLL1
635.3705	624.1613	678.9344	775.9185	828.0211	879.103	-0.35681	0.000189	TMEM87B
183.3318	209.0445	167.9085	122.4042	115.4402	103.6728	0.71294	0.0001891	SLC22A4
79.0843	125.823	97.64245	149.3747	191.0011	191.4773	-0.81658	0.0001902	RAD54B
187.8252	192.202	189.8096	119.2923	115.4402	123.7726	0.668478	0.0001907	KIZ
18532.69	20424.93	19122.41	25336.64	23088.04	20908.05	-0.25554	0.0001924	ATP5F1B
469.1137	460.6905	489.1248	550.8191	647.5146	735.2305	-0.44606	0.0001927	NFIB
195.0147	163.4708	187.072	286.3015	266.5619	261.2978	-0.57638	0.0001933	MTHFD2L
365.7649	417.0982	376.8816	635.8797	534.1733	462.296	-0.49404	0.0001938	ALG1
23.36582	36.65709	28.28893	6.223945	3.148369	9.520971	2.22388	0.0001941	MATN1-AS1
1184.467	1440.525	1490.188	2753.058	2339.238	1796.29	-0.74335	0.0001944	SNHG16
27.85924	32.69416	22.81366	1.037324	2.098913	10.57886	2.607569	0.0001946	F10
113.2343	90.15663	100.3801	64.3141	36.73097	45.48908	1.052001	0.0001958	DHRS13
384.6373	388.367	484.562	548.7445	554.113	632.6156	-0.46413	0.0001964	AP1S3
491.5808	507.2549	544.7901	635.8797	675.8499	698.2045	-0.38067	0.0001966	SS18L1
107.8422	73.31418	82.12916	140.0388	151.1217	176.6669	-0.8268	0.0001966	NBPF11
53.02243	70.34198	84.8668	39.41832	22.03858	11.63674	1.508314	0.0001969	AC116347.1
302.8569	301.1826	300.2277	206.4275	198.3472	221.0981	0.530987	0.0001991	GSDME
102.4501	117.8971	90.34208	171.1585	195.1989	155.5092	-0.74992	0.0001992	SLC38A9
2170.325	2150.88	2203.799	1927.348	1819.757	1693.675	0.262122	0.0001995	OTUB1
1209.63	1128.444	1169.884	919.0692	988.5879	938.3446	0.302011	0.0001998	KAT7
91.6659	73.31418	74.82879	123.4416	154.2701	147.0461	-0.8232	0.0002002	PLA2G4A
106.9435	111.9527	84.8668	59.12748	48.27499	44.4312	0.99919	0.0002023	CASC10
184.2305	184.2762	220.8362	279.0402	298.0456	293.0343	-0.56149	0.0002029	MDM1
39.54215	32.69416	46.53986	96.47114	101.7973	63.47314	-1.138	0.0002037	RGPD5
440.3558	466.6349	489.1248	599.5734	571.9537	702.4361	-0.42465	0.0002041	CETN3
113.2343	112.9435	101.2926	187.7557	166.8636	176.6669	-0.69856	0.0002058	DYRK4
53.92112	60.43466	53.84023	14.52254	10.49456	31.73657	1.568304	0.0002066	FRAT1
46.73163	46.56441	44.71476	16.59719	8.395651	20.09983	1.614058	0.0002071	GOLGA2P10
58.41454	64.39759	51.10259	135.8895	103.8962	96.2676	-0.95206	0.0002077	PARS2
13.48028	12.87952	24.63875	0	1.049456	2.115771	4.018461	0.000208	COL3A1
16.17634	17.83318	15.51329	2.074648	1.049456	0	3.97883	0.0002087	ADAMTS10
17.07502	13.87025	19.16347	2.074648	0	0	4.5828	0.0002094	AC010616.1
36.8461	46.56441	37.41439	10.37324	16.7913	9.520971	1.718345	0.0002098	HIST1H3E
93.46327	98.08249	158.783	191.905	259.2157	180.8985	-0.85026	0.0002101	ACACA
3.594741	8.91659	4.562731	18.67183	35.68152	28.56291	-2.29179	0.0002109	AC006111.2
216.5832	167.4337	196.1974	123.4416	128.0337	101.557	0.717742	0.0002117	ZBTB48
997.5407	942.1863	992.8503	1146.243	1195.331	1285.331	-0.30617	0.0002126	TFPI
9.885538	13.87025	6.387824	26.97043	40.9288	41.25754	-1.86272	0.0002132	AL132656.2
332.5136	386.3855	367.7561	298.7494	217.2375	203.114	0.594712	0.0002135	MTX1
736.0232	724.2252	746.4628	924.2558	903.5819	920.3605	-0.31645	0.0002138	PPP1R7
1031.691	994.6951	1003.801	747.9107	808.0814	860.0611	0.327032	0.0002144	GLRX
22.46713	15.85171	16.42583	3.111972	0	2.115771	3.387085	0.0002161	-
1293.208	1412.784	1330.492	1208.483	998.033	903.4344	0.375801	0.0002168	CTDSP1
408.0031	369.5431	405.1705	313.2719	277.0565	236.9664	0.515786	0.0002172	TIMM29
58.41454	53.49954	45.62731	23.85846	20.98913	8.463085	1.561692	0.0002189	AL360181.2
192.3186	220.9333	198.0225	297.712	329.5293	271.8766	-0.55778	0.0002193	FBXO25
1534.056	1564.366	1562.279	1375.492	1255.15	1144.632	0.303774	0.0002194	USP19
433.1663	400.2558	482.7369	609.9466	542.5689	616.7473	-0.42608	0.0002223	TFIP11
457.4308	474.5607	386.0071	338.1677	321.1336	239.0822	0.552232	0.0002225	MIGA2

1848.596	1872.484	1832.393	2491.653	2181.82	2088.266	-0.28416	0.0002228	FGFRL1
107.8422	125.823	134.1443	176.3451	196.2483	222.156	-0.69364	0.0002229	ZNF527
74.59088	66.37906	62.05314	132.7775	116.4897	117.4253	-0.85257	0.0002245	PGBD2
60.21191	54.49027	53.84023	108.919	100.7478	106.8465	-0.90858	0.0002245	IQCC
53.02243	51.51807	71.17861	16.59719	31.48369	16.92617	1.435491	0.0002247	CASTOR2
27.85924	29.72197	33.76421	5.186621	6.296738	10.57886	2.051524	0.0002247	AL109811.3
87.17247	72.32345	93.99226	148.3374	126.9842	171.3775	-0.81549	0.0002248	HPGD
403.5097	409.1724	370.4938	514.5128	527.8765	527.885	-0.40867	0.0002248	CPPED1
331.6149	313.0714	320.3037	482.3557	406.1396	436.9068	-0.45764	0.0002261	NME7
17.97371	21.79611	12.77565	3.111972	1.049456	0	3.649298	0.0002269	GACAT2
29.65661	31.70343	17.33838	2.074648	6.296738	6.347314	2.419825	0.0002276	TRAPPC5
110.5383	106.0083	100.3801	151.4493	181.5559	188.3037	-0.71769	0.000228	IRAK1BP1
281.2885	275.4235	246.3875	368.2501	358.9141	398.8229	-0.48767	0.0002281	SNX24
4422.43	4608.886	4510.716	4961.521	5299.755	5408.969	-0.21063	0.0002286	PTK2
150.9791	157.5264	187.072	113.0683	93.40161	83.57297	0.772619	0.0002287	FUK
234.5569	297.2197	253.6878	154.5613	193.1	161.8565	0.623366	0.000229	ELFN1-AS1
5.392112	17.83318	6.387824	0	0	0	5.673512	0.0002292	GIMAP2
9.885538	10.89805	6.387824	32.15705	40.9288	27.50503	-1.89181	0.0002294	OR51B5
19.77108	15.85171	14.60074	2.074648	1.049456	1.057886	3.584002	0.00023	-
2.696056	2.972197	3.650185	22.82113	15.74185	22.2156	-2.70504	0.0002301	PTPN22
597.6257	598.4022	553.9156	447.0867	442.8706	447.4856	0.38765	0.0002302	SIK3
854.6497	882.7424	890.6451	1020.727	1197.43	1077.986	-0.32683	0.0002307	CCSER2
41.33952	33.68489	30.11403	10.37324	11.54402	7.4052	1.842133	0.0002308	AC091271.1
329.8175	317.0343	276.5015	483.393	409.288	404.1123	-0.49033	0.0002311	ZFAND2A
239.949	237.7757	243.6498	190.8676	124.8853	133.2936	0.683436	0.0002327	SPHK2
1257.261	1277.054	1331.405	1516.568	1525.91	1622.797	-0.27114	0.0002329	INCENP
585.9428	607.3188	615.9687	732.3508	758.7569	819.8614	-0.35313	0.0002329	BDNF
180.6357	191.2113	166.996	263.4803	293.8478	246.4874	-0.57776	0.0002341	COMMD1
407.1044	455.7368	385.0945	310.1599	306.4413	280.3397	0.475552	0.0002341	ANGEL1
44.03558	60.43466	52.01513	19.70916	17.84076	21.15771	1.413013	0.0002342	ZNRF2P1
2883.881	3113.871	3110.87	4381.657	3306.837	3702.6	-0.32273	0.000236	COX7B
11.68291	9.907322	6.387824	0	0	0	5.59595	0.0002366	ADGRB1
484.3914	427.0056	511.0259	642.1036	649.6135	592.416	-0.4047	0.0002366	TRIT1
19.77108	32.69416	21.90111	5.186621	6.296738	0	2.68994	0.000237	LYNX1
21.56845	15.85171	13.68819	63.27677	46.17608	38.08388	-1.52811	0.0002377	RPL7P23
53.92112	30.7127	40.15203	10.37324	2.098913	17.98406	2.037331	0.0002382	AC027796.4
21.56845	16.84245	12.77565	0	0	3.173657	4.02183	0.0002401	NHSL2
797.1338	811.4097	785.7023	922.1812	1046.308	1031.439	-0.32539	0.0002414	UBR1
34.15004	15.85171	24.63875	3.111972	7.346194	2.115771	2.571871	0.0002417	AC133785.1
2724.814	2832.503	2551.479	3472.961	3282.699	2988.527	-0.26528	0.000242	SSR2
10.78422	14.86098	3.650185	0	0	0	5.659887	0.0002433	TEX45
673.1153	740.0769	657.9458	566.379	486.9477	528.9428	0.387798	0.0002435	IPO13
1087.409	1282.007	1160.759	1586.069	1449.299	1377.367	-0.32261	0.0002435	PNP
130.3094	117.8971	129.5816	79.87396	71.36303	57.12583	0.858531	0.0002441	S100A2
124.9173	138.7025	127.7565	74.68734	55.62119	84.63085	0.864221	0.0002449	MAP3K8
634.4718	549.8564	563.9536	454.348	417.6836	446.4278	0.407597	0.0002452	SLC25A28
35.04873	36.65709	26.46384	67.42607	87.10488	65.58891	-1.16645	0.0002459	PCDHGA9
281.2885	335.8582	281.0642	407.6684	439.7222	401.9966	-0.47742	0.000246	TMEM39B
89.86853	90.15663	76.65388	37.34367	46.17608	41.25754	1.040436	0.0002467	GALNT6
169.8515	197.1557	192.5473	278.0029	288.6005	258.1241	-0.56055	0.0002471	-
587.7402	585.5227	573.9916	442.9374	412.4363	473.9328	0.39446	0.0002476	ING1
57.51586	58.4532	62.96569	105.8071	118.5886	105.7886	-0.88364	0.0002476	DPY19L1P1
104.2475	146.6284	105.8554	71.57537	61.91792	51.8364	0.942391	0.0002484	AC004656.1
299.2622	332.886	310.2657	431.5268	434.4749	415.7491	-0.44447	0.0002497	ZBTB8OS

1141.33	1140.333	1141.595	1511.381	1365.343	1319.183	-0.29372	0.0002497	SLC35E1
148.2831	150.5913	147.8325	105.8071	80.80814	53.95217	0.891949	0.0002511	ZNF771
21.56845	19.81464	30.11403	1.037324	7.346194	3.173657	2.631923	0.0002512	SEC14L4
450.2413	459.6997	448.0602	330.9064	352.6173	324.7709	0.42941	0.0002518	TAF6
24.2645	11.88879	12.77565	1.037324	1.049456	1.057886	3.961975	0.000252	SLC6A12
997.5407	1039.278	900.6831	1397.276	1119.77	1243.016	-0.35657	0.0002522	RPS7P1
72.79351	56.47173	56.57787	18.67183	13.64293	35.96811	1.4464	0.0002523	RNF43
576.9559	639.0223	626.0067	824.6727	782.8944	745.8094	-0.35403	0.0002524	FBXO34
195.9134	240.7479	233.6118	332.981	317.9853	315.2499	-0.52873	0.0002528	RGMB
32.35267	23.77757	18.25092	0	6.296738	5.289428	2.686843	0.0002529	IRF5
846.5615	944.1678	901.5957	1119.273	1136.561	1074.812	-0.30749	0.000253	EIF2B5
104.2475	104.0269	88.51698	147.3	165.8141	180.8985	-0.73539	0.000254	EEF1B2P6
335.2096	399.2651	330.3417	669.0741	522.6293	406.2281	-0.58661	0.0002545	INTS5
879.8129	861.937	831.3296	1337.111	1144.957	939.4025	-0.41129	0.000255	ERI3
35.94741	26.74977	44.71476	11.41057	9.445107	9.520971	1.823552	0.0002555	BCO2
2049.002	2036.945	2141.746	2399.331	2457.827	2442.658	-0.22904	0.0002559	KDM3B
97.05801	83.2215	104.9428	63.27677	36.73097	29.6208	1.137312	0.0002563	AC102953.2
21.56845	21.79611	14.60074	1.037324	3.148369	3.173657	2.979613	0.0002566	P2RX1
46.73163	20.80538	23.7262	10.37324	4.197825	4.231543	2.279132	0.0002572	PNMA2
61.1106	43.59222	58.40296	20.74648	23.08804	19.04194	1.376851	0.0002575	THAP7-AS1
922.9498	957.0473	859.6185	1110.974	1092.484	1198.584	-0.31272	0.0002583	YY1AP1
59.31323	64.39759	70.26606	26.97043	15.74185	34.91023	1.321679	0.0002583	EIF4E3
110.5383	102.0454	107.6805	42.53029	47.22554	71.93623	0.987215	0.0002593	RHOBTB1
33.25136	32.69416	37.41439	9.335917	13.64293	3.173657	1.981627	0.0002598	SRP14-AS1
407.1044	339.8211	335.817	246.8831	251.8695	262.3556	0.509231	0.0002617	SIDT2
33.25136	24.7683	31.02657	7.261269	6.296738	8.463085	2.017431	0.0002639	AL590560.2
1203.34	1213.647	1204.561	1635.86	1452.448	1376.309	-0.30209	0.000264	SKI
1338.142	1398.914	1373.382	1179.438	1117.671	1092.796	0.277798	0.0002649	PRKACA
842.9668	789.6135	815.8163	1012.428	992.7857	1018.744	-0.30425	0.000267	TGFA
708.164	643.9759	656.1207	890.0241	865.8015	795.53	-0.345	0.0002671	CYTH3
1656.277	1645.606	1648.971	1355.783	1383.183	1416.509	0.252731	0.0002678	TPP1
758.4904	893.6404	794.8278	681.522	617.0803	579.7213	0.380731	0.0002683	DDB2
19.77108	16.84245	28.28893	3.111972	4.197825	3.173657	2.631745	0.0002695	-
38.64347	44.58295	46.53986	9.335917	10.49456	21.15771	1.663959	0.0002699	TTLL1
689.2916	656.8554	694.4477	491.6916	577.201	495.0905	0.384056	0.0002703	INPP4A
110.5383	116.9064	109.5055	62.23945	69.26412	48.66274	0.902506	0.0002707	CLDN15
9.885538	7.925857	5.475277	40.45564	29.38478	23.27348	-1.99947	0.0002712	-
155.4726	130.7766	169.7336	233.3979	244.5233	223.2139	-0.61913	0.0002715	NEMP2
19.77108	16.84245	16.42583	2.074648	3.148369	0	3.341194	0.0002725	TENM2
26.06187	30.7127	31.02657	9.335917	4.197825	7.4052	2.06669	0.0002739	BOLA1
21.56845	25.75904	21.90111	2.074648	7.346194	2.115771	2.58479	0.0002759	ACAP1
44.93426	26.74977	31.93912	12.44789	9.445107	5.289428	1.930777	0.0002761	TSSK6
157.2699	151.582	164.2583	231.3233	230.8804	246.4874	-0.58244	0.0002767	FLI1
225.57	237.7757	268.2886	300.824	358.9141	421.0385	-0.5625	0.0002783	TRAF6
26.06187	31.70343	32.85166	61.20212	59.81901	87.80451	-1.20515	0.0002786	RGPD8
3733.139	3665.709	3644.71	3020.688	3325.727	3139.805	0.219354	0.0002791	ARF6
186.0279	211.026	203.4978	140.0388	116.4897	124.8305	0.654352	0.0002796	LINC00294
350.4873	352.7007	348.5927	221.9874	240.3255	278.2239	0.506443	0.0002806	CYB5A
380.1439	346.7563	394.22	463.6839	519.4809	526.8271	-0.42874	0.0002818	RCOR3
567.9691	770.7896	671.634	405.5937	541.5195	484.5116	0.489187	0.0002825	SSBP1
200.4068	144.6469	173.3838	296.6747	273.9081	232.7348	-0.63017	0.000283	-
10.78422	4.953661	11.8631	0	0	0	5.57912	0.0002833	PLPP7
1101.788	1120.518	1119.694	1502.045	1275.089	1344.573	-0.30267	0.0002857	ERAL1
1318.371	1244.36	1289.428	1644.159	1514.366	1499.024	-0.27373	0.0002866	PPP2CB

13.48028	23.77757	20.98856	4.149297	2.098913	1.057886	2.989337	0.0002868	MAG
379.2452	367.5616	441.6724	513.4754	532.0744	550.1006	-0.42436	0.000287	NEK4
146.4857	121.8601	146.0074	51.86621	80.80814	90.97817	0.891233	0.0002871	MPPE1
20.66976	7.925857	11.8631	0	0	1.057886	5.169218	0.0002882	TCF20
17.07502	17.83318	27.37639	4.149297	3.148369	2.115771	2.724978	0.0002888	CACNA1G
775.5654	850.0482	762.8886	544.5952	672.7015	615.6895	0.381602	0.000291	LPIN1
24.2645	5.944393	20.07602	1.037324	1.049456	0	4.589227	0.0002935	ZG16B
9.885538	19.81464	25.55129	2.074648	3.148369	0	3.398919	0.0002957	-
114.133	146.6284	147.8325	75.72466	90.25325	53.95217	0.892538	0.0002963	ACOX3
147.3844	152.5728	121.3686	88.17255	83.95651	67.70468	0.811892	0.0002967	DUSP23
233.6582	252.6367	272.8513	184.6437	171.0614	144.9303	0.600193	0.0002968	PKIG
27.85924	16.84245	30.11403	64.3141	55.62119	63.47314	-1.28863	0.0002969	NEK10
148.2831	152.5728	126.8439	85.06058	85.00596	80.39931	0.771344	0.0002975	SH3RF2
8.986853	12.87952	5.475277	0	0	0	5.560582	0.000298	AC034213.1
399.0163	433.9407	411.5583	504.1395	553.0635	599.8212	-0.41334	0.0002987	ITPR2
564.3744	584.532	580.3794	719.903	780.7955	703.494	-0.35025	0.0002993	UBE3B
36.8461	35.66636	37.41439	12.44789	12.59348	8.463085	1.713586	0.0003004	-
56.61717	63.40686	62.96569	24.89578	18.89021	31.73657	1.276794	0.0003008	AC012306.2
48.52901	38.63855	62.96569	23.85846	15.74185	12.69463	1.521792	0.0003013	PNPLA1
90.76721	94.11956	81.21661	39.41832	45.12662	48.66274	0.998198	0.0003029	SERPINB9P1
212.0897	278.3957	257.338	149.3747	142.7261	186.1879	0.644084	0.0003056	C21orf59
144.6883	120.8693	135.0568	182.569	238.2266	212.635	-0.65958	0.0003063	N6AMT1
17.97371	12.87952	15.51329	1.037324	0	1.057886	4.468524	0.000307	CPZ
129.4107	123.8415	102.2052	69.50072	70.31358	51.8364	0.890556	0.0003092	HIST1H2BJ
438.5584	420.0704	412.4709	557.0431	574.0526	532.1165	-0.38778	0.0003094	CENPI
1098.193	1100.703	1059.466	961.5995	838.5156	743.6936	0.356891	0.0003106	NDUFB6
195.0147	232.8221	244.5624	297.712	322.1831	359.6811	-0.54328	0.0003109	PRRG1
21.56845	15.85171	20.07602	50.82888	40.9288	61.35737	-1.40987	0.0003111	DPF3
666.8245	748.0028	692.6226	824.6727	919.3238	927.7657	-0.34279	0.0003115	PPP2R5D
56.61717	77.27711	51.10259	19.70916	32.53315	21.15771	1.331944	0.0003154	AC020915.3
30.5553	23.77757	25.55129	3.111972	9.445107	4.231543	2.252144	0.0003162	AC144831.1
899.584	1035.315	1021.139	1147.28	1215.27	1379.483	-0.34043	0.0003172	PFDN2
87.17247	92.13809	96.7299	43.56761	47.22554	50.77851	0.963528	0.0003178	MT-TH
563.4757	544.9027	614.1436	435.6761	438.6728	442.1962	0.388171	0.0003191	HECA
994.8446	959.0287	972.7743	1258.274	1180.638	1141.459	-0.29078	0.0003196	CDC42SE1
1035.285	962.9917	986.4625	1270.722	1187.985	1189.063	-0.28916	0.0003203	ZBTB2
1237.49	1342.442	1312.241	929.4424	1133.413	1049.423	0.322492	0.0003213	MARK2
184.2305	189.2298	166.0834	77.79931	126.9842	113.1938	0.762983	0.0003221	DNASE1
56.61717	60.43466	64.79078	18.67183	23.08804	32.79446	1.287302	0.0003222	AL031714.1
424.1795	464.6534	443.4975	612.0212	546.7668	585.0108	-0.38887	0.0003226	C17orf75
124.9173	196.165	171.5587	212.6514	282.3038	309.9605	-0.70992	0.0003227	CNTNAP3
42.23821	52.50881	121.3686	21.78381	31.48369	22.2156	1.518559	0.0003239	-
157.2699	127.8045	146.9199	109.9564	55.62119	43.37331	1.047362	0.0003243	EXOC3-AS1
187.8252	171.3967	156.9579	100.6204	105.9951	112.1359	0.695793	0.0003245	AL445524.1
1099.092	1228.508	1129.732	1542.501	1441.953	1304.373	-0.31147	0.000325	ALDH1B1
118.6265	96.10102	108.593	48.75423	58.76956	64.53103	0.911621	0.0003253	PLCB2
1057.753	1210.675	1309.504	1422.171	1511.217	1530.761	-0.31941	0.0003257	PRKCA
1771.309	1843.753	1854.294	2562.191	2155.583	2040.661	-0.30548	0.0003265	DNAJC5
98.85538	87.18443	86.69189	44.60494	46.17608	48.66274	0.968459	0.0003267	PHF7
437.6597	451.7739	472.6989	589.2001	585.5966	589.2423	-0.37303	0.0003277	TIAM1
4.493426	14.86098	8.212916	0	0	0	5.571012	0.0003278	AC005186.1
78.18562	71.33272	57.49041	24.89578	32.53315	33.85234	1.181787	0.0003291	TRIM45
5359.759	5508.471	5529.118	4284.149	4916.703	4808.09	0.227132	0.0003297	UBR4
18.87239	18.82391	15.51329	3.111972	1.049456	2.115771	3.081369	0.0003304	BX640514.2

133.0054	136.721	132.3192	71.57537	86.05542	76.16777	0.782032	0.0003304	AP002884.1
97.9567	103.0361	110.4181	72.61269	41.97825	28.56291	1.119961	0.0003311	RNF122
8.088168	10.89805	7.30037	22.82113	30.43423	45.48908	-1.91328	0.0003317	ZC3H12B
6403.133	6924.227	6584.934	8106.688	7594.916	7332.206	-0.21023	0.0003319	ATP5F1A
76.38825	76.28638	86.69189	115.143	164.7646	140.6988	-0.8126	0.0003333	ZNF112
20499.91	21539.51	20624.46	27132.25	23595.98	22856.68	-0.23181	0.0003339	RPL15
391.8268	382.4226	392.3949	512.4381	515.2831	503.5536	-0.39219	0.0003348	NFU1
16.17634	5.944393	5.475277	0	0	0	5.578709	0.0003355	RPL7AP34
23.36582	16.84245	13.68819	1.037324	4.197825	0	3.36352	0.0003366	AC138207.2
154.5739	106.9991	181.5967	62.23945	44.07717	105.7886	1.064855	0.0003376	LINC01836
1050.563	1162.129	1182.66	1355.783	1473.437	1338.225	-0.29587	0.0003379	ZZEF1
262.4161	231.8313	264.6384	342.317	397.744	334.2919	-0.5006	0.0003381	MLH3
42.23821	34.67563	47.4524	11.41057	17.84076	11.63674	1.606275	0.0003382	TMEM91
46.73163	58.4532	49.2775	136.9268	90.25325	83.57297	-1.01079	0.0003393	ISCA1P1
26.96056	12.87952	23.7262	3.111972	5.247282	0	2.926667	0.0003401	TMEM159
1073.03	1113.583	1044.865	1255.162	1347.502	1316.01	-0.2784	0.0003402	DDX41
275.8964	258.5811	272.8513	359.9515	398.7934	357.5654	-0.46702	0.0003403	ELP4
8.986853	8.91659	8.212916	0	0	0	5.496597	0.0003406	TCP1P3
806.1207	882.7424	937.185	1068.444	1140.759	1064.233	-0.31808	0.0003421	RBSN
16.17634	14.86098	23.7262	4.149297	1.049456	1.057886	3.125993	0.0003422	LEMD1
52.12375	56.47173	45.62731	24.89578	15.74185	17.98406	1.393972	0.0003434	HOXB13
1693.123	1754.587	1753.914	1962.617	2077.924	2106.25	-0.24092	0.000345	LTA4H
645.256	687.5681	699.0104	896.2481	797.5868	869.582	-0.33553	0.0003491	AZI2
56.61717	42.60148	40.15203	77.79931	108.094	95.20971	-1.01038	0.0003493	TMEM117
52.12375	52.50881	50.19004	21.78381	22.03858	16.92617	1.349104	0.0003496	C5orf56
36.8461	29.72197	33.76421	8.298593	13.64293	5.289428	1.881859	0.0003507	AP001107.9
1027.197	1041.26	1076.805	878.6135	855.3069	829.3824	0.295134	0.0003515	LGMN
186.9265	203.1001	193.4598	147.3	99.69835	112.1359	0.699338	0.0003516	PEX7
1234.794	1284.98	1181.747	1718.846	1505.97	1387.946	-0.31786	0.0003542	RPSAP58
20.66976	53.49954	23.7262	2.074648	6.296738	11.63674	2.29009	0.0003545	TSTA3
21.56845	19.81464	23.7262	50.82888	47.22554	64.53103	-1.31869	0.0003554	AC084824.1
26.96056	47.55514	6.387824	2.074648	4.197825	5.289428	2.805499	0.0003557	CCHCR1
307.3504	305.1455	336.7296	412.855	463.8597	414.6912	-0.44379	0.0003561	CNNM3
8.088168	18.82391	11.8631	0	0	1.057886	5.102095	0.0003564	AC068580.4
246.2398	188.2391	203.4978	151.4493	137.4788	99.44125	0.716517	0.0003575	ZNF764
649.7495	634.0686	580.3794	476.1318	501.6401	455.9487	0.378656	0.0003588	IQCE
39.54215	29.72197	41.06458	14.52254	11.54402	6.347314	1.767001	0.0003597	RPL37AP1
6.290797	8.91659	10.95055	0	0	0	5.49859	0.0003605	MGAT3
57.51586	83.2215	96.7299	149.3747	128.0337	149.1619	-0.84619	0.0003617	TSPAN8
62.90797	72.32345	58.40296	36.30634	20.98913	24.33137	1.244385	0.0003618	RSG1
83.57773	75.29565	93.07971	129.6655	136.4293	167.1459	-0.78051	0.0003622	AC007406.5
89.86853	69.35125	73.0037	108.919	165.8141	143.8725	-0.84833	0.0003637	MIR137HG
579.652	642.9852	621.444	504.1395	477.5026	361.7969	0.456395	0.0003642	OGFOD3
1566.408	1420.71	1669.047	1796.645	1933.099	1926.41	-0.28017	0.0003667	YOD1
206.6976	152.5728	175.2089	112.031	92.35216	118.4832	0.72854	0.0003671	ALPK2
878.0155	883.7331	793.0027	1115.123	1054.704	1007.107	-0.31479	0.0003683	APEX2
19.77108	21.79611	26.46384	50.82888	56.67064	58.18371	-1.28378	0.0003686	GCNA
5310.331	5643.21	5592.996	5104.672	4574.58	4261.163	0.247141	0.0003696	UBB
2644.831	2980.122	2639.084	2456.384	2252.133	2148.566	0.268882	0.0003705	ATP5MC2
78.18562	94.11956	104.0303	38.38099	56.67064	41.25754	1.01934	0.000371	MAMDC4
21.56845	15.85171	18.25092	5.186621	0	0	3.415357	0.0003713	EGR4
39.54215	56.47173	48.36495	88.17255	96.54998	96.2676	-0.96336	0.0003716	YY2
302.8569	301.1826	279.2391	208.5022	208.8418	202.0562	0.511792	0.0003719	SNHG12
8.088168	6.935125	5.475277	34.2317	18.89021	31.73657	-2.04931	0.0003737	NEGR1

77.28693	94.11956	100.3801	46.67959	33.5826	52.89428	1.029007	0.0003752	CEACAM19
447.5453	469.6071	438.9347	384.8473	301.194	279.2818	0.489743	0.0003758	SNX11
37.74478	44.58295	30.11403	10.37324	14.69239	9.520971	1.699415	0.0003771	-
319.932	311.0899	323.9539	452.2733	406.1396	428.4437	-0.43013	0.0003776	RNF121
77.28693	104.0269	86.69189	34.2317	50.3739	46.54697	1.030069	0.0003786	FAM50B
187.8252	229.8499	207.148	322.6078	319.0347	268.703	-0.54438	0.0003835	RPP14
1120.661	1110.611	998.3256	878.6135	838.5156	890.7397	0.30833	0.0003848	TROAP
34.15004	20.80538	34.67676	10.37324	6.296738	5.289428	2.029796	0.0003856	SRRM2-AS1
322.628	311.0899	392.3949	450.1987	500.5907	459.1224	-0.45767	0.0003873	ZNF561
711.7587	635.0593	739.1624	810.1502	938.214	921.4184	-0.35522	0.0003873	KDM6A
17.97371	17.83318	16.42583	1.037324	3.148369	2.115771	3.052956	0.0003873	LINC01273
128.512	114.9249	108.593	168.0465	177.3581	222.156	-0.68837	0.0003877	ZNF654
70.09745	88.17516	61.1406	30.0824	43.02771	19.04194	1.249717	0.0003878	NT5M
6863.259	7048.069	6563.945	8134.696	7651.586	7643.224	-0.19454	0.0003883	RPL30
215.6845	236.785	238.1746	338.1677	319.0347	313.1342	-0.49109	0.0003896	IPPK
522.1361	442.8573	526.5392	573.6402	701.0368	737.3463	-0.43085	0.0003903	PSMA3-AS1
1302.195	1308.757	1322.279	1506.195	1604.619	1579.423	-0.2539	0.0003907	MAT2B
77.28693	77.27711	64.79078	34.2317	26.23641	40.19966	1.123537	0.0003909	-
32.35267	32.69416	35.5893	88.17255	56.67064	75.10988	-1.12801	0.0003913	PIPSL
606.6126	597.4115	622.3565	739.6121	788.1417	768.025	-0.32978	0.0003928	ZNF562
25.16319	8.91659	46.53986	65.35142	78.70923	83.57297	-1.49082	0.0003936	-
21.56845	24.7683	29.20148	3.111972	8.395651	4.231543	2.263596	0.000394	SSTR5
71.89482	96.10102	77.56643	47.71691	29.38478	38.08388	1.090145	0.000394	NEK8
9422.715	10148.07	9892.914	11315.13	11262.77	10859.2	-0.1826	0.0003947	RPN2
269.6056	230.8406	245.4749	143.1507	191.0011	156.5671	0.604941	0.0003949	KLHL24
30.5553	40.62002	32.85166	10.37324	13.64293	5.289428	1.825354	0.000395	SLC6A16
112.3357	121.8601	139.6196	175.3078	207.7924	206.2877	-0.65667	0.0003962	KANSL1L
74.59088	56.47173	34.67676	22.82113	17.84076	20.09983	1.447673	0.0003974	TBXA2R
2379.719	2547.172	2548.742	2863.015	3001.445	2857.349	-0.22259	0.0003979	CREBBP
86.27379	78.26784	71.17861	45.64226	10.49456	33.85234	1.388666	0.0004026	FOXL2
16.17634	22.78684	12.77565	2.074648	3.148369	0	3.302986	0.0004049	ELOA-AS1
87.17247	75.29565	74.82879	114.1057	144.825	153.3934	-0.79596	0.000405	CBWD3
81.78036	93.12882	89.42953	53.94086	34.63206	42.31543	1.012962	0.000406	SPOCD1
422.3821	386.3855	479.9993	279.0402	327.4304	321.5972	0.474549	0.0004063	HNRNPA1L2
10.78422	22.78684	14.60074	2.074648	1.049456	1.057886	3.520302	0.0004068	SBSN
629.0797	619.2076	580.3794	734.4255	758.7569	829.3824	-0.3449	0.0004078	AGTPBP1
474.5058	431.9592	475.4366	579.8642	612.8825	596.6475	-0.37211	0.000408	DDX59
122.2212	131.7674	117.7185	179.4571	191.0011	207.3456	-0.63706	0.0004081	LETM2
109.6396	106.9991	106.7679	56.0155	69.26412	49.72063	0.885957	0.0004083	TMTC2
293.8701	334.8675	291.1022	228.2113	224.5837	161.8565	0.580593	0.0004091	RNF166
268.7069	246.6923	237.262	346.4663	336.8755	362.8548	-0.47471	0.0004094	SNAPC1
729.7324	839.1502	697.1853	978.1967	892.0379	1019.802	-0.35168	0.0004097	TIMM8B
8.986853	12.87952	11.8631	29.04508	37.78043	41.25754	-1.68287	0.0004101	SMG1P6
146.4857	159.5079	149.6576	231.3233	229.8309	217.9244	-0.57641	0.0004103	ANAPC15
16.17634	28.73123	25.55129	3.111972	5.247282	5.289428	2.367385	0.0004124	HAPLN3
763.8825	791.595	803.0407	1033.175	924.571	965.8496	-0.31004	0.000414	PEX13
95.26064	71.33272	71.17861	26.97043	35.68152	46.54697	1.124504	0.0004143	AC021054.1
696.4811	643.9759	556.6532	476.1318	512.1347	429.5016	0.420099	0.0004153	HIGD2A
1923.186	1829.882	1912.697	1456.403	1463.992	1726.469	0.286269	0.0004156	LRP11
38.64347	24.7683	37.41439	8.298593	7.346194	12.69463	1.834031	0.0004169	PSD4
693.785	730.1696	680.7595	840.2326	876.296	904.4923	-0.31682	0.0004172	ARL6IP5
399.0163	449.7924	417.0336	327.7944	323.2326	261.2978	0.471682	0.0004202	OGG1
17.07502	30.7127	22.81366	4.149297	6.296738	3.173657	2.371179	0.0004203	SORCS1
1399.253	1514.83	1267.527	1229.229	1061	1024.033	0.334876	0.0004206	MRPL36

93.46327	92.13809	82.12916	165.9719	134.3304	145.9882	-0.73767	0.000423	KIAA1324L
24.2645	26.74977	32.85166	69.50072	54.57173	66.6468	-1.18509	0.0004255	AC113404.3
478.1006	567.6895	479.0868	412.855	375.7054	306.7868	0.47628	0.0004268	EFNA1
226.4687	303.164	237.262	386.9219	352.6173	356.5075	-0.51718	0.0004272	SFXN4
189.6226	113.9342	169.7336	239.6219	290.6994	233.7927	-0.68845	0.0004275	ZNF680
295.6675	259.5718	281.0642	382.7726	357.8646	413.6333	-0.46398	0.0004294	AHCYL2
543.7046	620.1983	491.8624	449.1614	407.1891	338.5234	0.469691	0.0004302	HEXA
3509.366	3389.295	3515.128	3020.688	3091.698	2920.822	0.20528	0.0004304	ERRFI1
2829.061	3019.752	2909.197	2670.072	2406.403	2219.444	0.263291	0.0004311	AKT2
8.986853	6.935125	12.77565	32.15705	34.63206	31.73657	-1.77487	0.0004321	-
2541.482	2637.329	2465.7	2733.349	3141.023	3404.276	-0.27953	0.0004322	GOLGB1
82.67905	62.41613	49.2775	29.04508	30.43423	22.2156	1.250734	0.0004326	AL590004.4
9.885538	19.81464	22.81366	0	3.148369	2.115771	3.321396	0.0004333	AL358334.2
268.7069	202.1094	258.2506	186.7183	147.9733	108.9622	0.717076	0.0004377	ZNF213
606.6126	573.6339	626.0067	505.1769	455.4641	411.5175	0.396663	0.0004378	SOX4
216.5832	217.9611	219.9236	338.1677	277.0565	317.3657	-0.51102	0.000438	PEX11B
80.88168	79.25857	61.1406	119.2923	120.6875	151.2777	-0.82269	0.0004381	PARGP1
21.56845	16.84245	14.60074	4.149297	1.049456	1.057886	3.078617	0.0004386	AC092903.2
2.696056	9.907322	14.60074	0	0	0	5.554511	0.0004391	AC074212.1
2092.139	2214.286	1912.697	2703.267	2431.59	2374.953	-0.2724	0.0004419	ATP5MG
9.885538	12.87952	13.68819	0	1.049456	0	5.015435	0.0004423	LINC01126
47.63032	78.26784	57.49041	131.7402	116.4897	98.38337	-0.9224	0.0004423	DYNC1I2P1
573.3612	526.0788	522.889	642.1036	731.4711	711.9571	-0.36191	0.0004432	POT1
158.1686	154.5542	152.3952	101.6578	102.8467	72.99411	0.74473	0.0004452	AC016065.1
2616.972	2822.596	2731.251	2361.987	2275.221	2369.664	0.221497	0.0004456	CIZ1
35.94741	25.75904	23.7262	10.37324	3.148369	6.347314	2.103591	0.0004458	NALT1
567.9691	600.3837	571.2539	787.329	715.7292	704.5519	-0.3442	0.0004464	ASH2L
1614.937	1743.689	1591.481	1860.96	2001.313	2052.298	-0.2571	0.0004471	SLC11A2
101.5514	101.0547	107.6805	173.2331	141.6766	191.4773	-0.70633	0.0004473	LINC00839
124.0186	121.8601	135.9694	72.61269	59.81901	84.63085	0.815419	0.0004484	GASAL1
891.4958	901.5663	880.6071	1035.249	1107.176	1122.417	-0.28821	0.0004497	RPRD2
65.60403	91.14736	55.66532	30.0824	12.59348	39.14177	1.375135	0.0004499	AC004943.2
18.87239	10.89805	31.02657	3.111972	1.049456	4.231543	2.860158	0.0004508	ADAM11
124.0186	121.8601	112.2432	157.6733	209.8913	203.114	-0.67212	0.000451	ZNF182
29.65661	39.62929	28.28893	12.44789	7.346194	7.4052	1.839867	0.0004526	OSER1-AS1
59.31323	5.944393	5.475277	3.111972	0	2.115771	3.75913	0.0004537	-
78.18562	67.36979	77.56643	48.75423	25.18695	21.15771	1.229562	0.000454	SLC26A1
8.986853	10.89805	5.475277	0	0	0	5.452772	0.0004553	UNC5CL
217.4818	267.4977	235.4369	139.0014	172.1108	163.9723	0.599856	0.0004553	ZNF559
16.17634	6.935125	3.650185	0	0	0	5.533732	0.0004565	AFDN-DT
241.7463	224.8962	211.7107	332.981	316.9358	304.6711	-0.49274	0.0004584	ZCCHC4
1316.574	1404.858	1319.542	1194.997	1006.429	1081.159	0.299517	0.0004596	AXIN1
56.61717	65.38832	51.10259	23.85846	23.08804	26.44714	1.23698	0.0004651	DNAJC12
1846.798	1948.77	1978.4	2208.463	2213.303	2396.111	-0.23982	0.0004654	NDRG3
204.9002	240.7479	187.072	162.8599	112.2918	114.2517	0.698838	0.0004655	CYP1A1
753.9969	747.0121	699.9229	644.1783	528.926	509.9009	0.386754	0.0004655	PI4K2A
868.13	934.2604	905.2458	771.7692	723.0754	658.0049	0.330343	0.0004659	ZC3H4
597.6257	607.3188	534.7521	417.0043	481.7005	421.0385	0.398308	0.0004659	MRPL52
362.1702	376.4782	341.2923	473.0198	446.0189	526.8271	-0.42132	0.0004674	LSM12P1
239.0503	264.5255	262.8133	339.205	336.8755	392.4756	-0.47986	0.0004675	NCK2
26.96056	25.75904	26.46384	8.298593	6.296738	4.231543	2.071015	0.0004689	AL365205.1
93.46327	88.17516	87.60444	49.79156	45.12662	45.48908	0.939337	0.000469	AP4M1
222.8739	228.8591	203.4978	174.2705	112.2918	121.6569	0.681843	0.0004709	ZNF205
26.06187	15.85171	9.125462	2.074648	2.098913	1.057886	3.285607	0.0004721	CCDC194

3.594741	6.935125	1.825092	21.78381	26.23641	17.98406	-2.43221	0.0004745	ZNRF2P2
3579.463	3700.385	3602.732	3177.324	3214.485	3099.605	0.197209	0.0004755	SMARCA4
26.96056	30.7127	19.16347	3.111972	3.148369	9.520971	2.285123	0.000476	AL137002.2
22.46713	44.58295	34.67676	7.261269	11.54402	9.520971	1.842481	0.0004774	-
295.6675	287.3123	289.2772	388.9966	380.9527	408.3439	-0.43365	0.0004787	ZKSCAN5
2580.125	2584.82	2733.988	2933.553	3127.38	3127.11	-0.21799	0.0004794	SLC30A1
26.06187	40.62002	31.93912	11.41057	5.247282	10.57886	1.854022	0.0004802	SLC44A3
259.72	310.0992	281.9768	402.4818	407.1891	362.8548	-0.46223	0.0004805	ZNRF3
233.6582	292.266	249.1251	183.6064	180.5065	154.4513	0.57839	0.0004827	ZNF710
598.5244	562.7359	534.7521	684.6339	727.2732	744.7515	-0.34643	0.0004852	NME6
3987.467	4335.444	3985.089	3894.115	3062.314	2806.571	0.334035	0.000486	SNRPD2
1822.534	1862.576	1693.686	1642.084	1383.183	1295.91	0.315588	0.0004898	SF3B5
62.00928	67.36979	56.57787	28.00775	28.33532	26.44714	1.166582	0.0004924	GLIPR2
88.07116	66.37906	92.16717	42.53029	46.17608	27.50503	1.086442	0.0004928	OGDHL
687.4942	622.1798	635.1322	492.729	465.9586	543.7532	0.372792	0.0004959	ZBTB5
99.75407	97.09175	93.99226	136.9268	168.9625	168.2038	-0.70467	0.000498	INTU
31.45398	31.70343	20.98856	7.261269	5.247282	8.463085	2.003954	0.0005001	CDNF
1027.197	1019.463	973.6868	731.3135	882.5928	797.6458	0.324793	0.0005021	SRGAP2
1440.593	1368.201	1444.561	1555.986	1783.026	1823.795	-0.27924	0.000503	GIGYF2
1.797371	5.944393	3.650185	25.9331	17.84076	19.04194	-2.47749	0.0005036	B3GALT5
88.07116	130.7766	132.3192	182.569	209.8913	180.8985	-0.70893	0.0005057	KLHL11
303.7556	343.7841	344.9425	479.2438	428.1782	429.5016	-0.43047	0.0005063	KLHL20
404.4084	456.7275	396.9576	310.1599	322.1831	295.1501	0.438996	0.0005069	PAAF1
4.493426	8.91659	11.8631	0	0	0	5.448586	0.0005074	SLC2A5
16.17634	30.7127	23.7262	7.261269	4.197825	0	2.616892	0.0005081	TGFBR3L
12.58159	12.87952	10.03801	1.037324	0	0	4.977268	0.0005106	KLRC4-KLRK1
274.099	229.8499	244.5624	325.7198	349.469	372.3758	-0.4838	0.0005114	RTTN
640.7626	652.8925	644.2576	550.8191	484.8488	466.5276	0.367135	0.0005135	FAM219A
85.3751	66.37906	81.21661	35.26902	46.17608	28.56291	1.083573	0.0005158	NFATC2
17.97371	44.58295	24.63875	7.261269	8.395651	4.231543	2.127661	0.0005158	AC108515.1
705.4679	803.4838	793.0027	624.4691	602.3879	589.2423	0.341502	0.0005162	CDK11B
224.6713	195.1742	215.3609	270.7416	328.4798	312.0763	-0.5196	0.0005165	JAK2
36.8461	45.57368	62.05314	82.98593	91.3027	115.3095	-1.00283	0.0005194	FRAS1
1054.158	1129.435	1063.116	958.4875	851.1091	716.1886	0.361818	0.0005194	YIPF3
143.7896	145.6376	166.996	102.6951	96.54998	70.87834	0.756504	0.0005199	ZFP41
10.78422	10.89805	13.68819	0	1.049456	0	4.972868	0.0005201	MAGEB17
689.2916	674.6886	759.2385	832.9713	904.6314	927.7657	-0.32765	0.0005208	EHBP1
1250.97	1280.026	1137.945	1042.511	1020.072	881.2188	0.317334	0.0005211	GSK3A
231.8608	229.8499	204.4104	161.8226	153.2206	110.0201	0.647454	0.0005237	MFSD2A
1348.927	1338.479	1410.796	1469.888	1792.471	1798.406	-0.30417	0.0005248	LPP
152.7765	154.5542	187.072	114.1057	86.05542	100.4991	0.717702	0.0005252	AC012085.1
383.7386	394.3114	397.8702	227.174	338.9744	245.4295	0.535048	0.0005253	-
1159.304	1147.268	1239.238	920.1065	1008.528	979.6021	0.286212	0.0005255	HIPK2
16.17634	23.77757	10.95055	0	4.197825	0	3.599138	0.0005261	LINC02475
24.2645	20.80538	24.63875	59.12748	72.41249	40.19966	-1.29948	0.0005262	AC026167.1
16.17634	18.82391	15.51329	1.037324	4.197825	0	3.268577	0.0005265	OPN4
207.5963	203.1001	229.0491	117.2176	116.4897	169.2617	0.667599	0.0005268	PPARGC1A
90.76721	99.07322	91.25462	161.8226	151.1217	142.8146	-0.69817	0.0005296	DPH6
817.8036	722.2438	725.4742	566.379	585.5966	627.3262	0.349053	0.0005309	AREG
10.78422	18.82391	18.25092	39.41832	43.02771	49.72063	-1.46906	0.0005322	CEP170P1
14.37896	16.84245	16.42583	2.074648	1.049456	2.115771	3.184082	0.0005327	RAB7B
12.58159	11.88879	18.25092	0	1.049456	1.057886	4.348466	0.0005357	LINC01010
4235.504	4477.119	4341.895	3648.269	3528.272	4013.618	0.222259	0.0005362	B2M
1528.664	1653.532	1586.918	1356.82	1375.837	1218.684	0.271042	0.0005377	ALAS1

10.78422	9.907322	14.60074	1.037324	0	0	4.97017	0.0005379	CU639417.4
20.66976	9.907322	13.68819	0	0	2.115771	4.399774	0.0005392	RNF112
264.2135	218.9518	248.2126	294.6001	362.0624	399.8808	-0.52937	0.0005415	PMS1
4.493426	5.944393	3.650185	23.85846	15.74185	28.56291	-2.27993	0.0005432	AC097534.1
1118.863	1061.074	1054.903	1213.669	1334.908	1399.583	-0.28722	0.0005447	WASHC2A
897.7866	1068.009	1025.702	1212.632	1206.875	1270.521	-0.30333	0.0005453	CCDC25
339.703	365.5802	347.6801	414.9297	501.6401	501.4378	-0.42967	0.0005463	TRAPPC13
36.8461	41.61075	43.80222	16.59719	16.7913	6.347314	1.619709	0.0005474	F2RL2
3.594741	2.972197	77.56643	1.037324	2.098913	3.173657	3.740818	0.000549	-
6.290797	10.89805	7.30037	0	0	0	5.401786	0.0005504	EMID1
963.3906	1029.371	1083.192	1303.916	1226.814	1215.511	-0.28458	0.0005522	COPS5
16.17634	18.82391	17.33838	3.111972	2.098913	2.115771	2.83468	0.0005531	-
8.986853	7.925857	7.30037	0	0	0	5.387754	0.0005538	DBH-AS1
392.7255	432.95	426.1591	537.3339	527.8765	560.6794	-0.37764	0.0005549	PIK3C3
660.5337	728.1882	630.5694	539.4086	538.3711	490.859	0.363621	0.0005565	PXDC1
111.437	106.9991	131.4067	52.90353	57.7201	80.39931	0.874166	0.000558	RALGAPA2
153.6752	179.3225	177.034	78.83663	103.8962	123.7726	0.734763	0.0005596	AL356740.3
80.88168	65.38832	77.56643	123.4416	119.638	141.7567	-0.77988	0.0005605	SETD9
1814.446	1645.606	1979.313	1539.389	1470.288	1472.577	0.279592	0.0005613	JUN
435.8624	431.9592	434.372	585.0508	567.7559	528.9428	-0.36907	0.0005634	FANCC
93.46327	117.8971	112.2432	66.38874	38.82988	63.47314	0.938837	0.0005644	AC012512.1
19.77108	14.86098	27.37639	47.71691	60.86847	48.66274	-1.33843	0.0005646	AC009302.1
26.96056	48.54588	50.19004	94.3965	80.80814	84.63085	-1.05053	0.0005659	AC009053.1
18.87239	19.81464	16.42583	0	4.197825	3.173657	2.906004	0.0005666	AC097478.1
539.2112	422.0519	488.2122	246.8831	353.6668	390.3598	0.549703	0.000567	AHSA2P
303.7556	360.6265	275.589	236.5099	222.4847	169.2617	0.579958	0.0005675	PDGFB
2313.216	2483.766	2452.012	2644.139	2941.626	2952.559	-0.23628	0.0005689	TTC3
38.64347	30.7127	35.5893	8.298593	14.69239	9.520971	1.692069	0.00057	LINC01311
23.36582	16.84245	24.63875	5.186621	4.197825	3.173657	2.368855	0.0005711	PIK3IP1
148.2831	154.5542	147.8325	78.83663	85.00596	107.9043	0.730029	0.0005717	STAG3L2
24.2645	22.78684	23.7262	6.223945	5.247282	4.231543	2.171594	0.0005722	CCDC183
605.7139	634.0686	567.6037	510.3635	457.563	373.4336	0.429625	0.000574	FJX1
1056.854	1024.417	985.5499	1184.624	1270.892	1251.479	-0.27342	0.0005759	FNBP1
75.48956	51.51807	66.61587	18.67183	32.53315	31.73657	1.225649	0.0005768	ADGRD2
410.6992	396.2929	357.7181	287.3388	286.5016	283.5134	0.441903	0.0005774	ACD
5.392112	8.91659	10.03801	0	0	0	5.394589	0.0005774	MIR4442
12.58159	56.47173	115.8934	12.44789	16.7913	17.98406	1.969808	0.0005781	-
596.727	590.4764	627.8318	701.2311	794.4385	801.8773	-0.33978	0.0005782	ELF1
20.66976	25.75904	14.60074	4.149297	2.098913	4.231543	2.53994	0.0005806	INSL3
16.17634	11.88879	7.30037	0	1.049456	0	4.972636	0.0005846	LINC00589
107.8422	67.36979	88.51698	181.5317	125.9348	155.5092	-0.80928	0.0005856	AC008560.1
37.74478	38.63855	31.93912	3.111972	5.247282	19.04194	1.985868	0.0005863	AC003102.1
341.5004	353.6914	333.0794	433.6015	468.0575	457.0066	-0.40226	0.0005874	PEX2
1970.817	2058.741	1941.898	1853.698	1518.563	1449.303	0.308326	0.0005897	NDUFS8
4089.917	4039.215	4215.051	3582.918	3691.987	3533.338	0.191765	0.000591	OSBP2
9.885538	20.80538	13.68819	1.037324	0	2.115771	3.815148	0.0005931	SMIM5
5.392112	8.91659	5.475277	30.0824	23.08804	25.38926	-1.99703	0.0005967	AC017100.1
434.9637	407.1909	457.1857	549.7818	589.7945	545.869	-0.37475	0.0005983	ALG13
496.973	496.3568	485.4746	289.4134	377.8043	407.286	0.461074	0.000599	GTF2IP4
133.9041	122.8508	109.5055	65.35142	77.65977	67.70468	0.797756	0.0005991	TEPSIN
135.7015	143.6562	149.6576	199.1662	221.4353	221.0981	-0.58091	0.0006006	RYR2
328.0201	335.8582	298.4026	236.5099	240.3255	208.4035	0.489331	0.0006017	LIMK2
4035.996	3999.586	4206.838	3224.003	3517.778	3724.815	0.226214	0.0006021	ACSL3
44.03558	40.62002	28.28893	16.59719	6.296738	11.63674	1.708291	0.0006028	LPAR5

532.0217	489.4217	531.1019	658.7008	625.476	698.2045	-0.35199	0.0006031	LRCH1
24.2645	24.7683	28.28893	3.111972	3.148369	10.57886	2.202198	0.0006055	PIK3AP1
335.2096	300.1918	345.855	406.6311	440.7717	478.1643	-0.43291	0.000607	FRA10AC1
40.44084	54.49027	42.88967	77.79931	99.69835	89.92028	-0.95891	0.0006083	DHFR2
215.6845	192.202	210.7982	291.4881	298.0456	283.5134	-0.496	0.0006089	ACVR2B
44.93426	45.57368	48.36495	12.44789	17.84076	22.2156	1.404468	0.0006092	AC007191.1
22.46713	18.82391	17.33838	3.111972	1.049456	5.289428	2.635104	0.0006106	FAM71E1
1339.041	1330.553	1330.492	1503.083	1621.41	1630.202	-0.24923	0.000611	SCAMP1
11.68291	9.907322	12.77565	1.037324	0	0	4.931779	0.0006112	AC241585.3
629.0797	641.9945	571.2539	787.329	739.8667	790.2406	-0.33137	0.0006117	CMC2
6401.335	6934.135	6872.386	7277.866	7871.972	8271.608	-0.21296	0.0006134	SPTBN1
21.56845	23.77757	31.02657	8.298593	4.197825	5.289428	2.101233	0.0006152	AC087623.3
1270.741	1192.842	1275.74	913.8826	1027.418	1093.854	0.301417	0.0006184	CEP250
1406.442	1543.561	1532.165	1770.712	1805.065	1739.164	-0.24616	0.0006201	ATP6AP2
329.8175	351.7099	333.0794	240.6592	229.8309	263.4135	0.466988	0.000621	MAP3K6
19.77108	17.83318	28.28893	3.111972	7.346194	1.057886	2.516601	0.0006221	ABLIM2
27.85924	47.55514	30.11403	13.48521	11.54402	2.115771	1.954344	0.0006232	AP001453.2
1512.487	1573.283	1646.233	1808.056	1915.258	1868.226	-0.2408	0.000626	CBL
17.97371	12.87952	14.60074	2.074648	0	2.115771	3.439891	0.0006262	CES1
26.06187	15.85171	18.25092	0	2.098913	6.347314	2.839909	0.0006271	NSG1
1244.679	1336.498	1294.903	1575.695	1607.767	1457.766	-0.26024	0.0006284	AREL1
14.37896	4.953661	2.737639	38.38099	24.1375	30.67868	-2.07031	0.0006286	-
164.4594	155.545	161.5207	215.7634	238.2266	253.8926	-0.5553	0.0006287	AC092718.4
302.8569	337.8397	377.7941	253.1071	219.3364	248.6031	0.498113	0.0006309	GLI2
2.696056	1.981464	2.737639	15.55986	12.59348	23.27348	-2.78861	0.0006335	SERPINB2
366.6636	386.3855	394.22	448.124	528.926	550.1006	-0.41261	0.0006359	NEK1
145.587	122.8508	140.5321	74.68734	74.5114	93.09394	0.756525	0.0006379	RPL35P1
155.4726	149.6006	138.707	190.8676	269.7103	221.0981	-0.6191	0.0006386	SYDE2
726.1377	726.2067	729.1244	920.1065	872.0982	885.4503	-0.2957	0.0006406	ACBD6
327.1214	340.8119	334.9045	456.4226	435.5244	431.6174	-0.40067	0.0006422	ARL4C
1548.435	1414.766	1470.112	1192.923	1202.677	1295.91	0.264553	0.0006422	RAB5A
905.8748	893.6404	891.5577	731.3135	769.2515	664.3522	0.313821	0.0006435	NARF
194.116	129.7859	190.7222	224.062	270.7597	314.192	-0.65025	0.0006438	AC060780.1
9.885538	4.953661	9.125462	0	0	0	5.374987	0.0006453	GRM4
14.37896	21.79611	12.77565	3.111972	2.098913	0	3.224098	0.0006463	LINC00319
19.77108	17.83318	16.42583	1.037324	5.247282	1.057886	2.88023	0.0006464	MGP
310.9451	331.8953	290.1897	178.4198	195.1989	259.182	0.560159	0.0006488	AGO4
213.8871	251.646	245.4749	180.4944	150.0723	143.8725	0.582671	0.0006493	HINT2
1551.131	1667.402	1736.575	1421.134	1333.859	1389.004	0.257753	0.0006502	HMCES
41.33952	31.70343	51.10259	93.35917	76.61031	80.39931	-1.00913	0.000651	LIPT1
1742.551	1851.678	1805.016	2019.67	2096.814	2276.57	-0.24387	0.0006538	SRSF4
399.0163	383.4134	375.0565	501.0276	582.4483	461.2382	-0.41628	0.0006581	BCL10
34.15004	27.7405	27.37639	9.335917	9.445107	6.347314	1.828773	0.0006589	NME3
8.986853	11.88879	3.650185	0	0	0	5.403751	0.0006589	APOBR
254.3279	280.3772	245.4749	212.6514	158.4679	138.583	0.613088	0.0006599	ITPKC
160.8647	138.7025	147.8325	221.9874	220.3858	220.0402	-0.56516	0.0006611	TWF1P1
20.66976	10.89805	24.63875	49.79156	36.73097	67.70468	-1.45008	0.0006623	Z68871.1
108.7409	122.8508	109.5055	176.3451	171.0614	180.8985	-0.63228	0.0006649	DMRTA1
21.56845	14.86098	31.02657	57.05283	45.12662	66.6468	-1.31884	0.0006651	LRRC8B
19.77108	27.7405	33.76421	4.149297	8.395651	7.4052	2.027171	0.0006683	ZNF710-AS1
13.48028	11.88879	17.33838	1.037324	2.098913	0	3.765547	0.0006695	SLC2A14
195.0147	182.2947	165.1709	113.0683	91.3027	133.2936	0.68423	0.0006719	ENOX1
18.87239	28.73123	30.11403	4.149297	4.197825	9.520971	2.121782	0.0006748	PPM1N
5817.19	7827.775	6687.139	4870.237	3003.544	3414.855	0.848853	0.0006752	MT-RNR1

95.26064	87.18443	69.35351	162.8599	153.2206	116.3674	-0.78071	0.000676	TMBIM4
701.8732	717.2901	691.71	590.2374	571.9537	472.8749	0.368148	0.0006786	PIGO
54.8198	61.4254	45.62731	21.78381	24.1375	22.2156	1.247275	0.0006792	AC009779.2
880.7116	886.7053	874.2193	1081.929	1104.028	1019.802	-0.27934	0.0006796	HIKESHI
100.6528	114.9249	79.39152	153.524	172.1108	156.5671	-0.71093	0.0006798	SLC38A6
556.2862	549.8564	615.0562	671.1487	791.2901	738.4042	-0.35425	0.0006837	RABL3
203.1029	238.7665	203.4978	293.5627	305.3918	308.9026	-0.49361	0.0006842	SLC36A1
18.87239	18.82391	13.68819	31.11972	50.3739	58.18371	-1.44333	0.0006858	MTMR9
5.392112	22.78684	0.912546	0	0	0	5.647016	0.0006867	-
353.1833	369.5431	320.3037	565.3417	436.5738	433.7331	-0.46163	0.0006898	CLP1
182.4331	198.1464	200.7602	318.4585	258.1663	262.3556	-0.52983	0.0006925	BNIP1
1170.088	1198.786	1172.622	1686.689	1397.876	1324.473	-0.31633	0.0006934	TIMM50
23.36582	31.70343	22.81366	7.261269	8.395651	2.115771	2.128166	0.0006967	HDAC10
141.9923	137.7118	139.6196	102.6951	71.36303	72.99411	0.762803	0.0006968	HIST1H2AG
385.536	283.3494	372.3189	422.1909	512.1347	522.5955	-0.48305	0.0006983	SCLT1
351.3859	323.9694	328.5166	414.9297	429.2276	512.0167	-0.43339	0.0006986	NUDT9
27.85924	24.7683	31.02657	5.186621	4.197825	11.63674	1.995315	0.0007011	GPD1
38.64347	49.53661	33.76421	16.59719	16.7913	6.347314	1.614756	0.0007029	FERMT3
1056.854	1187.888	1057.641	1432.545	1364.293	1247.247	-0.29294	0.0007036	PLAGL2
792.6404	791.595	750.113	682.5593	569.8548	585.0108	0.345062	0.0007071	B4GAT1
6.290797	7.925857	9.125462	0	0	0	5.334383	0.0007074	TOB1-AS1
331.6149	334.8675	328.5166	450.1987	407.1891	462.296	-0.40751	0.000709	TRIP4
198.6094	208.0538	182.5092	126.5535	134.3304	128.0042	0.598841	0.0007113	NSUN5P1
8.088168	9.907322	5.475277	0	0	0	5.341156	0.000714	AF213884.3
70.09745	98.08249	88.51698	48.75423	45.12662	34.91023	0.993097	0.0007168	ZNF688
27.85924	24.7683	23.7262	53.94086	48.27499	74.052	-1.20604	0.0007184	GABPAP
13.48028	10.89805	9.125462	0	1.049456	0	4.894652	0.0007187	SH2D3C
55.71849	62.41613	52.92768	20.74648	33.5826	15.86829	1.284081	0.0007191	RCN3
410.6992	394.3114	438.0222	532.1473	531.0249	542.6954	-0.36899	0.0007206	PRDM2
50.32638	56.47173	48.36495	23.85846	19.93967	21.15771	1.255147	0.000725	CTSK
441.2545	428.987	452.6229	571.5656	571.9537	549.0427	-0.3553	0.0007264	DCPS
17.97371	20.80538	10.03801	0	0	4.231543	3.53556	0.0007268	FOXD3-AS1
2087.646	2082.519	1951.936	2592.273	2343.436	2297.728	-0.24081	0.0007332	SGTA
199.5081	168.4245	158.783	262.443	307.4907	222.156	-0.58813	0.0007364	EEF1AKMT2
198.6094	166.443	171.5587	94.3965	135.3799	103.6728	0.687253	0.0007383	MED11
13.48028	12.87952	7.30037	0	1.049456	0	4.900335	0.0007423	LINC00612
925.6458	1072.963	996.5005	1346.447	1186.935	1168.964	-0.30648	0.0007438	CIAPIN1
186.0279	211.026	207.148	257.2564	305.3918	298.3238	-0.51144	0.0007472	DIAPH2
762.9838	739.0862	813.9912	928.4051	972.846	940.4604	-0.29472	0.0007479	CSTF3
144.6883	154.5542	188.8971	221.9874	264.463	242.2558	-0.57763	0.0007537	HECTD2
14.37896	20.80538	11.8631	3.111972	1.049456	1.057886	3.166034	0.0007554	AC022916.1
133.9041	152.5728	132.3192	94.3965	68.21466	86.74663	0.747011	0.0007578	SERGEF
849.2576	810.4189	826.7669	640.029	688.4434	683.3941	0.305842	0.0007594	APOBEC3C
683.0008	731.1603	671.634	973.01	787.0923	881.2188	-0.34115	0.0007595	LYRM4
56.61717	38.63855	22.81366	82.98593	75.56086	98.38337	-1.12053	0.0007599	AC018645.2
26.06187	32.69416	22.81366	3.111972	10.49456	6.347314	2.031289	0.0007605	LINC01132
190.5213	136.721	201.6727	114.1057	105.9951	105.7886	0.700286	0.0007607	ZNF704
5689.577	5929.532	5816.57	5193.882	5190.611	5071.504	0.173783	0.0007618	COPA
316.3372	293.2567	270.1137	215.7634	223.5342	152.3355	0.572072	0.0007627	TRAPPC12
241.7463	257.5904	239.0871	178.4198	160.5668	172.4354	0.529413	0.0007637	MACROD1
233.6582	294.2475	276.5015	171.1585	203.5945	174.5511	0.549444	0.0007657	EIF4BP6
531.123	608.3096	611.406	727.1642	766.1031	719.3623	-0.33823	0.0007712	CDK8
18.87239	13.87025	14.60074	2.074648	0	3.173657	3.175598	0.0007746	PLA2G4D
15.27765	8.91659	9.125462	0	1.049456	0	4.887902	0.000776	MIRLET7D

124.9173	122.8508	114.0683	60.1648	75.56086	75.10988	0.779489	0.0007772	DENND2D
236.3542	215.9796	238.1746	170.1212	153.2206	147.0461	0.554169	0.0007773	ZNF319
38.64347	47.55514	45.62731	19.70916	11.54402	17.98406	1.419667	0.0007785	-
19.77108	10.89805	10.95055	2.074648	0	0	4.316167	0.000779	AC147067.1
46.73163	53.49954	32.85166	9.335917	6.296738	25.38926	1.698634	0.0007795	OBSCN-AS1
157.2699	144.6469	178.8591	221.9874	235.0782	251.7768	-0.55891	0.0007816	ERCC8
258.8214	275.4235	296.5775	163.8972	211.9902	197.8246	0.534428	0.0007829	ANKRA2
73.69219	65.38832	62.05314	113.0683	130.1326	105.7886	-0.79452	0.0007844	AC073857.1
97.05801	96.10102	104.0303	117.2176	187.8527	200.9983	-0.76728	0.000785	AC138409.2
2587.315	2794.855	2646.384	2997.867	3024.533	3672.979	-0.27224	0.0007895	IK
38.64347	32.69416	47.4524	85.06058	91.3027	64.53103	-1.018	0.0007919	PLCB4
45.83295	41.61075	34.67676	17.63451	14.69239	11.63674	1.473081	0.0007932	DHRS4
224.6713	230.8406	250.9502	328.8318	317.9853	324.7709	-0.45962	0.0007962	KIF24
404.4084	413.1353	390.5698	554.9684	523.6787	488.7432	-0.376	0.0007978	QDPR
7.189482	20.80538	15.51329	1.037324	2.098913	0	3.788431	0.0007981	LINC02328
216.5832	304.1548	238.1746	335.0557	377.8043	366.0284	-0.50944	0.0008002	ATG4A
27.85924	21.79611	23.7262	5.186621	7.346194	5.289428	2.042916	0.000805	FENDRR
1506.197	1622.819	1397.108	1406.612	1089.336	999.702	0.372331	0.0008072	GNA11
639.8639	648.9296	598.6303	800.8142	766.1031	775.4302	-0.31184	0.0008078	SNAP29
26.96056	15.85171	19.16347	54.97818	43.02771	55.01006	-1.29966	0.0008112	RPL7AP66
2677.183	2630.394	2711.175	2946.001	3122.133	3165.194	-0.20334	0.0008122	MGEA5
123.1199	109.9713	135.9694	71.57537	76.61031	68.76257	0.767394	0.0008156	NMNAT1
62.90797	46.56441	68.44097	21.78381	25.18695	30.67868	1.198517	0.000817	OSMR-AS1
1016.413	1173.027	1095.055	934.629	907.7797	794.4721	0.316249	0.0008229	IQSEC1
72.79351	79.25857	73.0037	30.0824	50.3739	22.2156	1.131595	0.0008242	LINC01547
49.42769	54.49027	73.91624	38.38099	8.395651	13.75251	1.553192	0.0008252	-
105.1462	129.7859	147.8325	64.3141	67.16521	86.74663	0.810701	0.0008322	FBXO6
39.54215	35.66636	33.76421	69.50072	60.86847	95.20971	-1.0489	0.0008344	GPR75
27.85924	29.72197	29.20148	9.335917	7.346194	8.463085	1.786551	0.0008361	NTSR1
196.8121	216.9703	205.3229	307.0479	286.5016	273.9924	-0.48756	0.0008396	SETMAR
2595.403	2730.458	2741.289	3182.51	3229.177	2943.038	-0.21378	0.0008397	UPF1
51.22506	23.77757	37.41439	11.41057	7.346194	15.86829	1.702372	0.000843	CYTH4
32.35267	30.7127	21.90111	5.186621	9.445107	8.463085	1.879959	0.0008466	MST1P2
47.63032	43.59222	38.32694	72.61269	89.20379	87.80451	-0.94589	0.0008498	AC112220.4
57.51586	50.52734	47.4524	20.74648	24.1375	21.15771	1.235881	0.0008512	FGGY
3423.092	3634.006	3462.2	3287.28	2830.384	2400.343	0.304288	0.0008526	IRAK1
1.797371	0	1.825092	5.186621	23.08804	15.86829	-3.5791	0.0008542	GRM7-AS3
16043.33	16438.23	14979.45	14943.69	13154.94	12155.11	0.237575	0.0008566	RPL18A
14.37896	3.962929	5.475277	0	0	0	5.367026	0.0008568	STAB1
2158.642	2524.386	2682.886	2075.686	2104.16	1847.068	0.289256	0.0008603	PLEKHA2
146.4857	137.7118	146.9199	91.28452	81.85759	94.15183	0.690096	0.000865	CBR3
110.5383	94.11956	101.2926	163.8972	189.9516	138.583	-0.6857	0.0008687	NSUN6
23.36582	18.82391	10.95055	0	1.049456	5.289428	3.074258	0.0008713	S100A9
17.07502	19.81464	16.42583	3.111972	4.197825	1.057886	2.668982	0.0008743	CLDN23
10.78422	18.82391	19.16347	1.037324	3.148369	2.115771	2.952641	0.0008744	CFI
984.0604	1012.528	955.4359	758.2839	820.6749	836.7876	0.289137	0.0008749	DCAF5
2.696056	8.91659	5.475277	22.82113	33.5826	17.98406	-2.13436	0.0008758	AL356019.2
205.7989	177.3411	175.2089	289.4134	276.007	240.14	-0.5283	0.0008765	MON1A
48.52901	30.7127	51.10259	8.298593	16.7913	20.09983	1.53185	0.0008792	ARSI
20.66976	16.84245	10.03801	0	0	4.231543	3.498871	0.0008806	AC008105.2
308.2491	310.0992	309.3532	386.9219	431.3266	412.5754	-0.40785	0.0008816	TMEM52B
7740.376	5303.389	7046.682	2040.417	3551.36	4103.539	1.051182	0.0008874	NEAT1
6.290797	9.907322	6.387824	0	0	0	5.285228	0.0008879	RNA5-8SN4
263.3148	253.6274	307.5281	154.5613	217.2375	186.1879	0.563962	0.0008883	FARP1

1777.599	1994.344	1921.822	1735.443	1517.514	1238.784	0.341804	0.0008901	SLC4A2
727.0364	717.2901	667.0713	607.8719	545.7173	497.2063	0.354734	0.0008903	CDC34
17.97371	23.77757	16.42583	3.111972	5.247282	2.115771	2.471284	0.0008907	MLC1
25.16319	37.64782	29.20148	9.335917	7.346194	10.57886	1.753215	0.0008911	HEXIM2
102.4501	101.0547	118.631	163.8972	161.6163	176.6669	-0.63983	0.0008975	DEXI
33.25136	38.63855	38.32694	12.44789	9.445107	15.86829	1.545294	0.0008975	GSTA4
358.5754	348.7377	289.2772	259.331	208.8418	235.9085	0.500856	0.0009015	PDE6D
629.9784	641.9945	628.7443	815.3368	813.3287	733.1148	-0.31356	0.0009018	MRPL44
1820.736	2029.02	1811.404	2602.646	2399.057	1996.23	-0.30618	0.0009052	CDCA5
1580.787	1614.893	1554.066	1401.425	1339.106	1264.173	0.245938	0.0009052	SMARCD2
378.3465	397.2836	355.893	258.2937	273.9081	302.5553	0.438611	0.0009209	ARRB2
491.5808	458.709	530.1894	624.4691	634.9211	623.0947	-0.34591	0.000923	TBCEL
27.85924	40.62002	31.93912	16.59719	3.148369	7.4052	1.883278	0.0009236	NEDD9
8.986853	6.935125	6.387824	0	0	0	5.27002	0.0009248	UGT3A2
395.4215	390.3485	440.7598	316.3839	322.1831	267.6451	0.436776	0.0009251	MFSD11
225.57	221.924	261.9008	299.7867	334.7766	354.3917	-0.4786	0.0009256	L3MBTL3
8.986853	7.925857	5.475277	0	0	0	5.27433	0.0009277	CALY
682.1021	696.4847	680.7595	806.0009	820.6749	938.3446	-0.31678	0.0009281	ZKSCAN1
11.68291	3.962929	7.30037	0	0	0	5.313071	0.0009288	CDH16
368.461	397.2836	342.2048	252.0698	301.194	252.8347	0.458309	0.0009308	SAP30
15.27765	25.75904	21.90111	2.074648	2.098913	7.4052	2.443965	0.0009352	KLHL32
206.6976	213.0074	177.034	301.8613	298.0456	252.8347	-0.51584	0.0009355	TMEM147-AS1
32.35267	15.85171	29.20148	57.05283	76.61031	49.72063	-1.23882	0.0009377	ADTRP
28.75793	14.86098	14.60074	54.97818	46.17608	47.60486	-1.34895	0.0009405	ZNF566
339.703	341.8026	333.0794	378.6233	512.1347	494.0326	-0.44864	0.0009406	NHLRC3
17.07502	11.88879	4.562731	1.037324	0	0	4.895425	0.0009436	ABHD11-AS1
3805.932	4009.493	3526.079	5073.552	4363.639	4146.912	-0.26049	0.0009472	FAU
267.8082	231.8313	235.4369	106.8444	183.6549	175.609	0.658131	0.0009477	PZP
419.686	483.4773	424.334	597.4987	566.7064	546.9269	-0.36728	0.0009498	METTL2A
203.1029	171.3967	179.7716	259.331	255.0179	276.1082	-0.51101	0.0009525	RAD52
3423.092	3301.12	3384.634	3770.673	3861.999	3843.299	-0.18287	0.0009558	SNX3
22.46713	22.78684	27.37639	3.111972	9.445107	4.231543	2.11419	0.0009558	AC099778.1
89.86853	86.1937	73.91624	38.38099	41.97825	49.72063	0.94268	0.0009564	RBP5
5.392112	1.981464	7.30037	19.70916	24.1375	23.27348	-2.18147	0.0009585	LINC01776
745.0101	732.1511	825.8543	841.2699	1032.665	1051.538	-0.34475	0.0009596	MEF2A
614.7007	633.0779	632.3945	821.5607	800.7352	720.4201	-0.31756	0.0009634	COP1
250.7332	214.9889	234.5244	310.1599	317.9853	337.4655	-0.46247	0.0009706	ST7L
30.5553	39.62929	35.5893	9.335917	13.64293	12.69463	1.567535	0.0009738	AC073957.3
956.2011	930.2975	913.4588	803.9262	768.202	709.8413	0.295047	0.0009786	P3H4
78.18562	58.4532	51.10259	22.82113	37.78043	20.09983	1.2188	0.0009828	ISL2
74.59088	69.35125	69.35351	34.2317	31.48369	40.19966	1.010516	0.0009893	ATP2B1-AS1
725.239	735.1233	763.8012	932.5544	905.6808	875.9293	-0.28727	0.0009902	OSER1
188.7239	207.063	180.6842	121.3669	122.7864	137.5251	0.594357	0.0009906	RPL23AP82
275.8964	270.4699	250.0377	211.6141	161.6163	177.7248	0.531192	0.0009918	SNHG19
2594.504	2692.81	2446.536	2295.598	2204.908	2136.929	0.220313	0.000996	PIGT
357.6767	386.3855	442.5849	458.4973	595.0417	547.9848	-0.43237	0.0009992	AGO3
11.68291	16.84245	11.8631	1.037324	2.098913	0	3.682316	0.0010076	LXN
580.5507	525.0881	590.4174	676.3353	736.7184	719.3623	-0.32956	0.0010104	MTA3
1837.811	1860.595	1887.146	2208.463	2204.908	2072.398	-0.21563	0.0010164	TUBGCP2
709.9614	829.2428	726.3868	618.2452	625.476	521.5376	0.359217	0.0010167	CC2D1B
3.594741	8.91659	2.737639	25.9331	19.93967	22.2156	-2.17434	0.0010187	AC010226.1
52.12375	37.64782	25.55129	17.63451	2.098913	11.63674	1.877384	0.0010218	1-Sep
1816.243	1955.705	1831.48	2119.253	2289.914	2144.334	-0.22625	0.001025	AK4
594.9297	610.291	633.3071	443.9747	487.9972	512.0167	0.348658	0.0010277	ZBTB10

369.3596	354.6821	365.0185	493.7663	446.0189	476.0486	-0.37835	0.0010279	PIGC
285.7819	314.0621	333.9919	198.1289	231.9299	236.9664	0.485481	0.0010299	DNAJC18
93.46327	55.481	97.64245	134.8521	129.0831	170.3196	-0.81285	0.0010326	ZNF354B
30.5553	30.7127	39.23949	64.3141	74.5114	66.6468	-1.03063	0.0010358	AC012360.3
10.78422	9.907322	9.125462	23.85846	36.73097	33.85234	-1.66261	0.0010372	AC097468.3
8.986853	4.953661	19.16347	0	0	1.057886	4.879706	0.0010373	MAPK15
128.512	123.8415	112.2432	174.2705	186.8032	190.4194	-0.59704	0.0010401	DCLRE1C
14.37896	8.91659	16.42583	37.34367	43.02771	32.79446	-1.50505	0.0010417	AL163051.2
12.58159	32.69416	10.95055	3.111972	0	4.231543	2.933261	0.0010428	C19orf81
29.65661	35.66636	37.41439	67.42607	62.96738	78.28354	-1.0231	0.0010453	FBXO48
416.0913	416.1075	445.3226	554.9684	584.5472	506.7272	-0.36573	0.0010474	ARHGEF28
906.7734	975.8712	903.4208	1229.229	1104.028	1067.407	-0.2881	0.0010477	ATRAID
11.68291	11.88879	15.51329	1.037324	1.049456	1.057886	3.636166	0.0010483	-
5.392112	0	1.825092	19.70916	25.18695	9.520971	-2.88944	0.0010492	AC079907.1
272.3016	237.7757	301.1403	200.2036	195.1989	165.0302	0.534243	0.0010496	ZNF8
51.22506	47.55514	61.1406	18.67183	30.43423	17.98406	1.254026	0.001053	EPHX2
60.21191	55.481	55.66532	30.0824	22.03858	25.38926	1.144488	0.0010565	LINC01521
18.87239	7.925857	9.125462	28.00775	39.87934	43.37331	-1.6238	0.0010581	ISM1
139.2962	136.721	152.3952	177.3824	230.8804	239.0822	-0.59483	0.00106	ALG10
221.9753	210.0352	248.2126	141.0761	170.0119	151.2777	0.557683	0.0010601	WASH3P
267.8082	263.5348	307.5281	387.9592	373.6065	368.1442	-0.42897	0.0010624	MOSPD1
236.3542	235.7943	221.7487	347.5036	322.1831	288.8028	-0.4664	0.0010631	SEC22A
44.93426	70.34198	33.76421	19.70916	23.08804	12.69463	1.421991	0.0010634	SORCS2
0.898685	2.972197	0.912546	8.298593	24.1375	11.63674	-3.2267	0.0010644	TNPO1P3
31.45398	11.88879	16.42583	4.149297	2.098913	4.231543	2.514827	0.0010644	LINC00659
133.9041	155.545	157.8705	205.3902	216.188	236.9664	-0.55853	0.0010648	ZFHX4
128.512	90.15663	92.16717	157.6733	173.1603	168.2038	-0.68102	0.001071	ZFPM2-AS1
46.73163	63.40686	65.70333	23.85846	30.43423	24.33137	1.160363	0.0010725	SORBS1
466.4177	514.19	525.6266	403.5191	401.9418	337.4655	0.397754	0.0010726	CCDC71
26.06187	21.79611	22.81366	42.53029	64.01684	56.06794	-1.20058	0.0010777	AL365203.2
159.0673	225.8869	182.5092	130.7028	81.85759	130.1199	0.72628	0.0010788	MARCKSL1
1137.736	1175.999	1241.975	1354.745	1489.179	1403.814	-0.25652	0.0010814	LCOR
50.32638	39.62929	46.53986	14.52254	18.89021	21.15771	1.324615	0.0010825	KIRREL3
47.63032	45.57368	38.32694	11.41057	17.84076	21.15771	1.384623	0.0010843	BEND3P1
15.27765	20.80538	13.68819	0	0	5.289428	3.240869	0.0010851	SESN3
11355.79	11760.98	10779.91	13982.09	11836.82	13601.24	-0.21784	0.0010874	RPL37
70.09745	78.26784	83.04171	43.56761	33.5826	41.25754	0.966245	0.0010893	MAGEB2
869.0287	863.9185	858.706	975.0847	1057.852	1122.417	-0.2838	0.0010921	ATXN2
40.44084	28.73123	42.88967	80.91128	62.96738	82.51508	-1.01129	0.0010986	RBBP4P1
278.5924	264.5255	264.6384	193.9796	177.3581	203.114	0.492	0.0011013	TPCN2
152.7765	155.545	184.3343	223.0247	278.1059	225.3296	-0.55985	0.0011025	IFT88
57.51586	40.62002	29.20148	22.82113	10.49456	8.463085	1.606553	0.0011046	AC015912.3
65.60403	58.4532	65.70333	31.11972	23.08804	34.91023	1.091241	0.0011071	COL5A2
194.116	244.7108	219.0111	296.6747	353.6668	283.5134	-0.50679	0.0011109	GMEB1
52.12375	62.41613	69.35351	104.7697	116.4897	100.4991	-0.80772	0.0011113	FAM81A
533.8191	585.5227	574.9041	641.0663	730.4216	787.0669	-0.34962	0.0011114	BTRC
17.97371	10.89805	18.25092	1.037324	2.098913	3.173657	2.90599	0.0011129	KLHL30
106.9435	99.07322	100.3801	151.4493	193.1	142.8146	-0.66918	0.0011144	TAMM41
26.06187	18.82391	14.60074	3.111972	3.148369	5.289428	2.366842	0.0011146	RPS10P7
10.78422	10.89805	9.125462	1.037324	0	0	4.772926	0.0011148	HCAR3
312.7425	296.2289	289.2772	203.3155	240.3255	199.9404	0.481158	0.0011188	9-Mar
154.5739	127.8045	102.2052	65.35142	81.85759	75.10988	0.791185	0.00112	-
26.96056	13.87025	24.63875	1.037324	7.346194	4.231543	2.380316	0.0011201	AC090589.3
515.8454	518.1529	540.2274	462.6466	378.8537	326.8867	0.429835	0.0011208	LRRC14

206.6976	194.1835	253.6878	291.4881	340.0239	298.3238	-0.50543	0.0011224	LMLN
239.949	232.8221	270.1137	328.8318	325.3315	361.7969	-0.45096	0.0011272	ATXN3
8.088168	1.981464	10.95055	24.89578	40.9288	20.09983	-2.01939	0.0011277	ARPC3P1
20.66976	10.89805	20.98856	4.149297	2.098913	2.115771	2.652206	0.0011297	-
274.9977	350.7192	285.627	419.079	390.3978	424.2122	-0.43849	0.0011325	METTL6
3.594741	0.990732	37.41439	1.037324	0	0	5.22273	0.0011338	-
21.56845	23.77757	15.51329	6.223945	4.197825	0	2.540537	0.0011355	PRSS36
11.68291	4.953661	5.475277	32.15705	30.43423	20.09983	-1.89577	0.0011449	AL049869.1
1383.975	1420.71	1407.146	1062.22	718.8776	593.4739	0.826691	0.0011496	ATP6V0B
3858.056	3118.825	4033.454	2623.393	2911.192	3313.298	0.315752	0.00115	CCNL1
144.6883	145.6376	132.3192	96.47114	82.90705	83.57297	0.684135	0.0011524	GSTM4
81.78036	61.4254	83.04171	46.67959	34.63206	29.6208	1.028976	0.0011546	MIRLET7BHG
1599.66	1946.789	1708.287	2236.471	2145.089	1971.899	-0.27451	0.0011552	RNF4
190.5213	222.9147	220.8362	143.1507	142.7261	144.9303	0.557432	0.0011564	TMEM80
266.9095	293.2567	279.2391	370.3247	400.8923	353.3338	-0.42255	0.0011621	GEMIN2
60.21191	37.64782	55.66532	62.23945	134.3304	117.4253	-1.02906	0.0011694	HELLPAR
63.80665	69.35125	56.57787	30.0824	26.23641	33.85234	1.072627	0.0011703	THTPA
150.0804	180.3133	156.9579	221.9874	257.1168	228.5033	-0.53932	0.001171	LINP1
123.1199	133.7488	160.6081	77.79931	78.70923	96.2676	0.724296	0.0011731	PLCB1
23.36582	28.73123	23.7262	8.298593	7.346194	4.231543	1.929038	0.0011742	H1FX-AS1
323.5267	326.9416	321.2163	490.6543	393.5461	413.6333	-0.41783	0.0011764	HAUS1
1623.026	1773.411	1594.218	1486.485	1309.722	1375.251	0.258254	0.0011765	GADD45GIP1
148.2831	146.6284	144.1823	91.28452	92.35216	95.20971	0.655132	0.0011816	VAMP1
32.35267	33.68489	36.50185	6.223945	13.64293	13.75251	1.610341	0.001184	PDCD4-AS1
509.5546	528.0603	465.3986	429.4522	372.557	276.1082	0.478737	0.0011888	TMEM101
23.36582	26.74977	19.16347	39.41832	58.76956	63.47314	-1.22424	0.0011938	-
407.1044	437.9036	443.4975	320.5332	349.469	307.8447	0.397741	0.0012021	WRB
141.9923	144.6469	125.9314	94.3965	74.5114	84.63085	0.701816	0.0012169	AMDHD2
9.885538	13.87025	14.60074	1.037324	1.049456	1.057886	3.607707	0.0012176	TGFB2-AS1
161.7634	128.7952	177.034	210.5768	253.9684	232.7348	-0.57454	0.0012188	MED7
13.48028	7.925857	5.475277	45.64226	24.1375	24.33137	-1.80505	0.0012211	SETSIP
23.36582	28.73123	30.11403	66.38874	58.76956	51.8364	-1.10768	0.0012249	MTSS1
62.00928	49.53661	50.19004	17.63451	24.1375	28.56291	1.203074	0.001225	NEIL1
62.90797	68.36052	57.49041	38.38099	28.33532	17.98406	1.154242	0.0012271	BMP4
68.30008	62.41613	52.92768	35.26902	29.38478	13.75251	1.226575	0.0012307	ADAT3
3.594741	15.85171	24.63875	1.037324	2.098913	0	3.809575	0.0012316	LENG8
216.5832	258.5811	200.7602	298.7494	326.3809	317.3657	-0.48112	0.0012329	MTRF1
6.290797	5.944393	15.51329	26.97043	25.18695	44.4312	-1.79439	0.0012345	CCDC144CP
911.2669	990.7322	991.9377	1160.766	1178.539	1130.88	-0.2623	0.0012442	PHKA1
2500.142	2604.635	2531.403	3201.182	2999.346	2734.634	-0.22682	0.0012443	COPZ1
17.07502	18.82391	21.90111	54.97818	37.78043	47.60486	-1.2802	0.0012489	AC097263.1
142.891	137.7118	113.1557	182.569	198.3472	211.5771	-0.5897	0.0012527	RBFA
2290.749	2334.165	2294.141	2761.357	2630.987	2563.257	-0.20152	0.0012559	MYBBP1A
211.191	213.0074	272.8513	178.4198	154.2701	124.8305	0.607451	0.0012587	CCDC22
39.54215	21.79611	23.7262	4.149297	9.445107	9.520971	1.883343	0.0012682	RPS24P17
185.1292	168.4245	172.4712	126.5535	116.4897	100.4991	0.614732	0.001269	ZFAT
20.66976	14.86098	12.77565	0	4.197825	2.115771	2.939882	0.0012726	DACT3
31.45398	25.75904	29.20148	3.111972	10.49456	10.57886	1.840114	0.001273	KCNK7
16322.82	18174.98	16925.91	16233.09	14327.18	13406.59	0.225956	0.001273	TPI1
709.9614	675.6793	766.5388	931.5171	888.8895	835.7297	-0.30317	0.0012756	AAAS
21.56845	23.77757	23.7262	8.298593	4.197825	4.231543	2.043625	0.0012771	CDH8
4249.883	4637.617	4512.541	4861.938	5340.683	5151.903	-0.19655	0.0012782	NOTCH2
35.04873	41.61075	41.06458	11.41057	19.93967	11.63674	1.452992	0.0012784	HOXA4
25.16319	34.67563	25.55129	9.335917	6.296738	9.520971	1.761394	0.0012811	AC105285.1

31.45398	26.74977	31.93912	8.298593	11.54402	8.463085	1.672201	0.0012817	CD22
207.5963	217.9611	204.4104	279.0402	284.4027	304.6711	-0.46297	0.0012943	LINC00667
0.898685	0.990732	0.912546	4.149297	16.7913	16.92617	-3.75747	0.0013019	SYT16
743.2127	759.8916	730.9495	620.3198	625.476	550.1006	0.314677	0.0013025	DGUOK
50.32638	48.54588	62.96569	23.85846	30.43423	10.57886	1.318567	0.0013052	MPND
1019.109	1101.694	1054.903	1310.14	1249.903	1225.032	-0.25368	0.0013062	SAP30BP
30.5553	13.87025	22.81366	64.3141	78.70923	31.73657	-1.3732	0.0013135	OXTR
305.553	292.266	292.9273	375.5113	388.2988	412.5754	-0.40094	0.0013151	CEP128
6.290797	18.82391	27.37639	3.111972	2.098913	2.115771	2.838583	0.0013173	WNT10A
16.17634	11.88879	13.68819	3.111972	1.049456	0	3.32055	0.0013176	GATA5
0	0.990732	0	12.44789	9.445107	6.347314	-4.81597	0.0013203	AC084357.2
984.0604	962.9917	1015.664	767.6199	838.5156	838.9033	0.27734	0.0013219	SEC24D
10.78422	13.87025	11.8631	1.037324	0	1.057886	4.121793	0.0013224	NKX3-2
451.14	452.7646	407.9082	370.3247	307.4907	306.7868	0.413479	0.0013263	ZNF384
65.60403	77.27711	62.05314	37.34367	22.03858	38.08388	1.071059	0.0013287	AC012640.2
5270.789	5682.84	5071.932	4447.009	3386.596	3048.827	0.558328	0.0013299	GPX1
17.07502	12.87952	9.125462	1.037324	0	2.115771	3.634854	0.0013368	GUCY2EP
485.2901	457.7183	472.6989	381.7353	352.6173	362.8548	0.367915	0.0013382	PGAP2
63.80665	56.47173	97.64245	130.7028	125.9348	123.7726	-0.80164	0.001339	KIAA0391
1036.184	1049.185	897.0329	853.7178	811.2298	732.0569	0.314847	0.0013399	RAC2
1013.717	1068.009	987.375	841.2699	866.8509	838.9033	0.268665	0.0013438	HGSNAT
0	3.962929	2.737639	14.52254	16.7913	15.86829	-2.8376	0.0013451	IL1RAPL1
39.54215	25.75904	32.85166	11.41057	9.445107	11.63674	1.59689	0.0013488	AC144652.1
132.1067	142.6654	120.4561	75.72466	82.90705	84.63085	0.699615	0.0013488	PXMP4
1104.484	1161.138	1157.109	1382.753	1416.766	1275.81	-0.252	0.0013527	UQCC1
183.3318	183.2855	175.2089	127.5909	119.638	114.2517	0.58362	0.0013558	BORCS8
8.986853	14.86098	12.77565	1.037324	1.049456	0	4.126555	0.0013583	SLC2A12
66.50271	70.34198	70.26606	33.19437	35.68152	35.96811	0.982081	0.0013611	ERCC5
57.51586	34.67563	49.2775	21.78381	22.03858	10.57886	1.379837	0.0013642	-
281.2885	331.8953	323.9539	237.5472	213.0396	228.5033	0.463916	0.0013652	MOB3C
304.6543	349.7285	328.5166	409.743	477.5026	414.6912	-0.40629	0.0013655	BUD13
1889.935	1783.318	1895.358	1562.21	1611.965	1610.102	0.219305	0.0013668	ZNF217
32.35267	46.56441	31.93912	59.12748	85.00596	79.34143	-1.01478	0.001378	ZNF420
81.78036	80.24931	84.8668	48.75423	48.27499	32.79446	0.926958	0.0013833	PXMP2
8.088168	8.91659	12.77565	0	1.049456	0	4.724734	0.0013833	4-Sep
26.96056	17.83318	15.51329	5.186621	3.148369	4.231543	2.263288	0.001385	CD36
37.74478	38.63855	40.15203	80.91128	72.41249	69.82045	-0.93739	0.0013915	AC007382.1
17.07502	14.86098	20.07602	6.223945	0	0	3.055106	0.0013924	-
73.69219	83.2215	74.82879	132.7775	120.6875	123.7726	-0.70416	0.0013928	FBXO4
623.6876	603.3559	602.2805	512.4381	500.5907	387.1862	0.385502	0.0013932	DCAF15
2253.903	2431.257	2219.312	2134.813	1879.576	1751.859	0.259581	0.0013951	RIC8A
351.3859	350.7192	330.3417	446.0494	501.6401	411.5175	-0.3969	0.0013966	ELAVL2
29.65661	19.81464	29.20148	43.56761	67.16521	65.58891	-1.16024	0.0013993	AC010326.3
38.64347	51.51807	32.85166	10.37324	14.69239	20.09983	1.444793	0.0014033	C2orf16
647.9521	612.2725	563.041	766.5825	782.8944	720.4201	-0.31611	0.0014035	ERG28
35.94741	15.85171	28.28893	6.223945	10.49456	3.173657	2.011545	0.001404	AC099343.3
310.9451	245.7016	285.627	375.5113	375.7054	385.0704	-0.43051	0.0014071	LINC00941
1113.471	1185.906	1064.029	1413.873	1352.749	1265.231	-0.26201	0.0014084	PIGS
20.66976	35.66636	20.98856	73.65001	51.42336	50.77851	-1.19129	0.0014101	TSPAN2
12.58159	7.925857	9.125462	0	1.049456	0	4.718654	0.0014136	AC084346.2
1470.249	1696.133	1664.484	1353.708	1401.024	1280.042	0.259415	0.0014158	TP53
16.17634	18.82391	20.07602	2.074648	5.247282	3.173657	2.392394	0.0014172	TM4SF1-AS1
1.797371	1.981464	1.825092	14.52254	16.7913	10.57886	-2.90406	0.0014257	C9orf135
215.6845	163.4708	203.4978	146.2627	113.3413	119.5411	0.620915	0.0014258	CDPF1

414.2939	475.5514	427.9842	653.5142	553.0635	516.2482	-0.38755	0.0014283	FASTKD5
317.2359	336.8489	285.627	372.3994	431.3266	455.9487	-0.42324	0.0014305	EFNA5
80.88168	71.33272	59.3155	31.11972	43.02771	29.6208	1.027618	0.0014321	ICOSLG
26.96056	29.72197	30.11403	11.41057	5.247282	9.520971	1.72829	0.0014346	AL357033.4
8089.965	9299.012	8712.991	9117.042	10532.34	11210.41	-0.24163	0.0014362	FAT1
937.3287	1062.065	1041.215	1350.596	1225.765	1139.343	-0.28977	0.0014366	CCNY
233.6582	227.8684	210.7982	328.8318	307.4907	285.6291	-0.45579	0.0014407	RRP7BP
185.1292	210.0352	227.224	121.3669	155.3195	139.6409	0.580056	0.0014456	HMBOX1
614.7007	730.1696	680.7595	941.8903	871.0488	756.3883	-0.34381	0.001447	RTL6
641.6613	606.3281	553.9156	481.3184	474.3543	466.5276	0.341383	0.001448	DHRS7
110.5383	126.8137	158.783	202.2782	209.8913	189.3615	-0.6026	0.0014535	HELB
46.73163	43.59222	40.15203	10.37324	16.7913	23.27348	1.372746	0.0014586	MIR503HG
337.007	306.1362	292.9273	434.6388	418.7331	385.0704	-0.40362	0.0014599	RUBCN
23.36582	31.70343	28.28893	11.41057	5.247282	7.4052	1.789977	0.0014603	EOMES
11835.69	12530.78	11919.68	11008.08	11024.54	9809.774	0.188401	0.0014644	PTPRF
788.147	786.6413	848.668	955.3755	986.489	983.8337	-0.27149	0.0014684	SMAD4
14.37896	18.82391	20.98856	6.223945	2.098913	0	2.696493	0.0014732	LZTS1
21.56845	18.82391	24.63875	4.149297	8.395651	1.057886	2.256807	0.0014735	LIN37
325.3241	275.4235	253.6878	346.4663	392.4967	436.9068	-0.45999	0.0014746	AC132008.2
3137.31	3507.192	3245.927	2927.329	2863.966	2788.587	0.204768	0.0014777	PARK7
113.2343	47.55514	54.75277	98.54579	126.9842	228.5033	-1.07179	0.0014806	PAK1IP1
221.9753	222.9147	265.5509	363.0634	313.7874	305.729	-0.46758	0.0014853	VPS33A
533.8191	474.5607	528.3643	646.2529	629.6738	650.5997	-0.32535	0.0014901	NRBF2
23.36582	21.79611	22.81366	3.111972	3.148369	9.520971	2.109813	0.0015005	AC006042.3
6894.713	7536.5	6317.557	5570.431	4022.566	3679.326	0.644571	0.001504	RPS21
20559.22	20024.68	19750.24	17773.51	18240.6	18482.32	0.14683	0.001504	PRSS23
16.17634	15.85171	14.60074	4.149297	0	2.115771	2.892394	0.0015083	MIR4767
1596.065	1532.663	1552.241	1414.91	1314.969	1125.59	0.279847	0.0015134	HGS
1197.049	1250.304	1277.565	1032.138	1040.011	1068.465	0.246102	0.0015157	MRPS5
40.44084	12.87952	21.90111	6.223945	5.247282	6.347314	2.081439	0.0015222	AC108134.2
59.31323	48.54588	36.50185	19.70916	25.18695	12.69463	1.325456	0.0015264	ALOX12
13.48028	12.87952	15.51329	1.037324	2.098913	2.115771	2.997611	0.0015269	AC092171.5
24.2645	16.84245	33.76421	5.186621	9.445107	4.231543	1.990869	0.0015346	FRMD6-AS1
20.66976	21.79611	32.85166	8.298593	4.197825	7.4052	1.920361	0.0015356	AL031186.1
8.088168	5.944393	6.387824	0	0	0	5.142668	0.0015358	PRR25
277.6938	280.3772	271.0262	434.6388	386.1999	316.3078	-0.4562	0.0015412	EIF5AP4
537.4138	444.8387	594.9801	684.6339	630.7233	743.6936	-0.38349	0.0015418	COMMD6
19.77108	7.925857	9.125462	0	1.049456	1.057886	4.135638	0.0015432	LINC01050
68.30008	64.39759	95.81735	35.26902	44.07717	35.96811	0.987854	0.001544	TSPAN13
63.80665	59.44393	62.05314	25.9331	27.28586	35.96811	1.055955	0.0015445	RPL13AP25
24.2645	30.7127	47.4524	10.37324	9.445107	13.75251	1.610245	0.0015452	-
369.3596	371.5246	394.22	591.2748	478.5521	441.1383	-0.41279	0.0015494	NOC4L
22.46713	20.80538	24.63875	4.149297	1.049456	9.520971	2.208589	0.0015499	COL9A2
132.1067	150.5913	137.7945	221.9874	193.1	199.9404	-0.54971	0.0015543	C19orf44
136.6002	123.8415	116.8059	95.43382	69.26412	45.48908	0.843113	0.0015622	CTSF
51.22506	43.59222	79.39152	25.9331	28.33532	22.2156	1.189013	0.0015631	-
345.0951	385.3948	370.4938	304.9733	272.8586	218.9823	0.465699	0.0015665	PLEKHG4
127.6133	103.0361	114.9808	48.75423	72.41249	77.22565	0.80249	0.0015723	PCDH9
141.0936	153.5635	119.5436	79.87396	91.3027	85.68874	0.688512	0.0015729	POLR3GL
1212.326	1136.37	1106.919	1274.871	1516.464	1382.657	-0.27228	0.0015777	ARHGEF7
8.088168	3.962929	14.60074	24.89578	29.38478	37.026	-1.76763	0.0015793	AC126474.1
4.493426	6.935125	9.125462	0	0	0	5.150756	0.0015798	-
12.58159	3.962929	13.68819	1.037324	0	0	4.749634	0.0015801	AL031123.1
137.4988	125.823	124.1063	227.174	192.0505	166.0881	-0.5952	0.0015837	ZKSCAN4

11.68291	6.935125	2.737639	0	0	0	5.207089	0.0015855	VWA1
12.58159	36.65709	9.125462	4.149297	2.098913	3.173657	2.624922	0.001586	PPP2R3B
95.26064	106.9991	115.8934	71.57537	46.17608	62.41525	0.819868	0.0015965	SLC1A1
133.0054	136.721	135.9694	94.3965	75.56086	83.57297	0.677874	0.0015966	LARGE1
95.26064	116.9064	82.12916	142.1134	156.369	168.2038	-0.66724	0.0016087	FAM239A
10.78422	13.87025	15.51329	2.074648	2.098913	0	3.261774	0.0016093	MGAT4B
366.6636	387.3763	344.9425	306.0106	229.8309	267.6451	0.451205	0.0016117	MCOLN1
921.1524	966.9546	945.3979	1174.251	1113.473	1092.796	-0.25496	0.0016122	CNIH4
1537.651	1591.116	1590.568	1421.134	1319.167	1256.768	0.239441	0.0016162	FAM53C
143.7896	157.5264	159.6956	122.4042	83.95651	75.10988	0.710853	0.0016175	FAM241B
49.42769	32.69416	34.67676	15.55986	14.69239	13.75251	1.409705	0.0016178	NINL
1888.138	2035.955	1938.248	2293.524	2241.639	2224.734	-0.20584	0.0016181	CHSY1
33.25136	33.68489	33.76421	14.52254	7.346194	12.69463	1.542239	0.0016182	SLC25A27
4.493426	3.962929	8.212916	30.0824	11.54402	29.6208	-2.09134	0.0016199	EVI2A
32.35267	14.86098	16.42583	6.223945	3.148369	4.231543	2.227312	0.0016203	CPSF1P1
16.17634	10.89805	13.68819	1.037324	0	3.173657	3.280678	0.0016226	AGT
3496.784	3689.487	3341.744	2908.657	2972.06	3224.436	0.209352	0.0016282	CDC37
149.1818	93.12882	52.01513	37.34367	49.32445	49.72063	1.110677	0.0016325	BFAR
57.51586	62.41613	68.44097	20.74648	33.5826	34.91023	1.078644	0.0016399	KCNQ1OT1
145.587	129.7859	139.6196	90.2472	85.00596	87.80451	0.658313	0.0016587	PGM2L1
42.23821	34.67563	19.16347	5.186621	15.74185	6.347314	1.816927	0.001664	HIST1H3B
402.611	333.8767	405.1705	455.3853	556.2119	497.2063	-0.40109	0.0016672	ALKBH3
871.7247	874.8165	851.4056	747.9107	707.3336	683.3941	0.280502	0.0016678	CNPY3
1564.611	1457.367	1690.036	1783.16	1911.06	1876.689	-0.24113	0.0016689	MSI2
796.2352	888.6868	846.8429	981.3086	1061	1016.628	-0.27336	0.0016721	SLC41A1
1044.272	1009.556	937.185	869.2776	780.7955	810.3404	0.281625	0.001674	USB1
352.2846	366.5709	352.2428	453.3106	492.195	436.9068	-0.36842	0.0016781	TBC1D2B
103.3488	140.684	112.2432	157.6733	206.7429	186.1879	-0.63002	0.0016835	EIF4BP7
424.1795	461.6812	386.9196	673.2234	480.651	551.1584	-0.42257	0.0016863	CCDC58
33.25136	21.79611	31.02657	11.41057	8.395651	6.347314	1.719318	0.0016865	NLRP3
855.5484	802.4931	734.5997	726.1269	567.7559	565.9688	0.36323	0.0016867	NENF
44.93426	38.63855	47.4524	18.67183	17.84076	17.98406	1.266558	0.0017012	-
378.3465	433.9407	368.6687	551.8564	483.7994	495.0905	-0.37538	0.001707	LCMT1
53.92112	59.44393	51.10259	31.11972	13.64293	26.44714	1.20654	0.001708	MOCS1
148.2831	212.0167	177.9465	269.7043	260.2652	246.4874	-0.53081	0.0017098	NANP
301.9583	306.1362	296.5775	380.698	399.8429	400.9387	-0.38523	0.0017123	HIVEP3
2.696056	4.953661	8.212916	16.59719	30.43423	20.09983	-2.08166	0.0017268	-
623.6876	581.5598	614.1436	825.71	701.0368	738.4042	-0.31588	0.0017273	ADRB2
740.5167	729.1789	731.8621	608.9093	581.3988	607.2264	0.292562	0.0017325	SMG9
1248.274	1250.304	1279.39	1038.361	1123.968	1019.802	0.247648	0.0017334	STK38
629.0797	660.8184	646.0827	538.3712	527.8765	487.6853	0.316789	0.0017337	GDF11
39.54215	27.7405	32.85166	52.90353	67.16521	86.74663	-1.04333	0.0017379	AC007066.2
2177.514	2419.368	2163.647	1689.801	1292.93	912.9553	0.795181	0.0017411	SNRPB
13.48028	14.86098	11.8631	0	1.049456	3.173657	3.257265	0.0017471	SYCE1L
26.96056	33.68489	23.7262	8.298593	5.247282	11.63674	1.743514	0.0017525	CCDC106
812.4115	910.4829	882.4322	733.3882	711.5314	688.6836	0.287665	0.001767	ITPR1
1028.995	1061.074	1013.839	836.0833	864.752	892.8555	0.258953	0.0017677	TUSC3
136.6002	130.7766	112.2432	164.9345	207.7924	197.8246	-0.58781	0.0017847	SVIL-AS1
8.986853	18.82391	17.33838	1.037324	2.098913	3.173657	2.839815	0.0017852	-
98.85538	94.11956	91.25462	59.12748	43.02771	58.18371	0.825967	0.0017915	LINC00674
834.8786	869.8629	840.4551	673.2234	703.1358	721.478	0.278734	0.001792	SYVN1
160.8647	152.5728	174.2963	264.5177	208.8418	229.5612	-0.5268	0.0017922	TPM3P9
22.46713	20.80538	10.03801	4.149297	0	4.231543	2.668138	0.001794	AC096733.2
31.45398	21.79611	20.98856	57.05283	52.47282	52.89428	-1.12712	0.0017945	FDXACB1

15.27765	21.79611	22.81366	7.261269	3.148369	2.115771	2.253315	0.0018042	SNORC
75.48956	61.4254	54.75277	29.04508	28.33532	35.96811	1.038867	0.0018074	S1PR3
286.6806	268.4884	283.8019	367.2127	380.9527	357.5654	-0.39784	0.0018089	ACAT2
1428.91	1472.228	1318.629	1236.49	1142.858	1173.195	0.247995	0.0018113	TRAPPC1
10.78422	4.953661	4.562731	0	0	0	5.135292	0.0018147	LDLRAD1
160.8647	152.5728	136.8819	91.28452	99.69835	99.44125	0.632899	0.001822	AC091729.3
842.0681	857.9741	858.706	1079.854	1025.319	969.0233	-0.26494	0.0018236	MANBAL
33.25136	39.62929	32.85166	18.67183	8.395651	9.520971	1.528351	0.001832	TPI1P2
17.07502	7.925857	4.562731	1.037324	0	0	4.715552	0.0018375	SCN1A
220.1779	270.4699	215.3609	167.0092	147.9733	172.4354	0.533267	0.0018537	NDUFB8
416.99	412.1446	388.7447	323.6451	306.4413	304.6711	0.381526	0.0018551	FYCO1
1913.301	1886.354	1903.571	2607.833	2122.001	2113.656	-0.26302	0.0018568	SNRPC
50.32638	63.40686	44.71476	84.02326	122.7864	83.57297	-0.87634	0.001859	C8orf76
2090.342	2477.821	2298.704	1903.49	2046.44	1846.011	0.244231	0.0018597	ARHGAP35
35.94741	19.81464	22.81366	7.261269	9.445107	5.289428	1.838608	0.0018665	-
70.99614	79.25857	71.17861	45.64226	29.38478	39.14177	0.954662	0.0018687	ABALON
811.5128	931.2882	829.5045	1251.013	987.5384	995.4704	-0.33092	0.0018763	ELP5
31.45398	40.62002	34.67676	7.261269	8.395651	20.09983	1.578818	0.0018791	CARMIL2
4261.566	4465.23	4588.282	3785.196	4046.704	3903.598	0.182155	0.0018812	SERINC3
1381.279	1547.524	1434.523	2285.225	1760.988	1532.876	-0.35492	0.0018825	IFRD2
35.04873	31.70343	42.88967	19.70916	9.445107	9.520971	1.502586	0.0018874	AP002478.1
249.8345	322.9787	282.8893	216.8007	217.2375	143.8725	0.565006	0.0018993	ALG12
40.44084	56.47173	26.46384	14.52254	9.445107	20.09983	1.483396	0.0019037	DISP2
115.9304	152.5728	126.8439	94.3965	74.5114	70.87834	0.71949	0.0019057	TRPM2
81.78036	54.49027	87.60444	125.5162	122.7864	128.0042	-0.74593	0.0019086	AC107068.1
1791.08	1840.78	1815.054	1620.3	1565.789	1513.834	0.212638	0.0019108	RNF40
104.2475	91.14736	110.4181	157.6733	173.1603	141.7567	-0.62673	0.0019116	LEPR
40.44084	23.77757	25.55129	11.41057	7.346194	9.520971	1.668041	0.0019179	TMPRSS9
213.8871	200.1279	199.8476	285.2641	277.0565	276.1082	-0.44946	0.0019217	TCTN2
1965.425	2016.14	1957.412	1783.16	1663.388	1692.617	0.208513	0.0019349	-
248.9358	239.7572	271.9388	178.4198	176.3087	190.4194	0.4811	0.0019402	ARHGEF3
18.87239	8.91659	16.42583	0	1.049456	4.231543	3.074937	0.0019436	AL445490.1
13.48028	10.89805	13.68819	1.037324	2.098913	1.057886	3.183485	0.0019515	SLC16A8
15.27765	10.89805	20.07602	37.34367	40.9288	40.19966	-1.35237	0.0019522	HNRNPA3P10
1274.336	1284.98	1201.823	1576.733	1517.514	1373.136	-0.2485	0.0019602	NFS1
795.3365	815.3726	857.7934	1066.369	989.6373	934.1131	-0.27666	0.0019623	ATP6V1H
83.57773	87.18443	125.9314	61.20212	51.42336	51.8364	0.851629	0.0019698	DTX2
564.3744	571.6525	535.6646	471.9825	452.3157	368.1442	0.370802	0.0019728	ATG4B
617.3968	584.532	622.3565	462.6466	511.0852	486.6274	0.321422	0.0019766	MUM1
302.8569	251.646	258.2506	379.6606	334.7766	378.7231	-0.42672	0.001982	TCEANC2
144.6883	129.7859	98.55499	63.27677	80.80814	77.22565	0.753408	0.0019843	NR6A1
5.392112	0.990732	0.912546	28.00775	22.03858	4.231543	-2.8805	0.0019881	AL139094.1
931.0379	1023.426	992.8503	1201.221	1211.073	1109.722	-0.25745	0.0019887	SORD
935.5314	947.14	796.6528	1087.116	1065.198	1103.375	-0.28145	0.0019942	RNF130
28.75793	40.62002	40.15203	14.52254	11.54402	14.8104	1.420731	0.0019944	CIRBP-AS1
662.3311	686.5774	730.037	891.0614	862.6531	791.2985	-0.29192	0.0019944	KIAA1549
51.22506	85.20297	80.30407	34.2317	37.78043	35.96811	1.003471	0.0020021	OTX1
523.9335	498.3383	512.851	402.4818	415.5847	396.7071	0.337923	0.0020055	C19orf12
77.28693	77.27711	93.99226	40.45564	43.02771	51.8364	0.878091	0.0020141	KDM4D
26.96056	32.69416	45.62731	8.298593	9.445107	17.98406	1.560826	0.0020149	AC092910.3
1033.488	1062.065	985.5499	953.3009	779.7461	576.5477	0.415478	0.0020153	POLD1
3350.299	3812.337	3559.843	3304.915	3046.572	2620.383	0.256919	0.0020169	DDOST
610.2073	605.3374	605.0181	490.6543	493.2445	484.5116	0.310145	0.0020226	SLC22A5
86.27379	61.4254	88.51698	119.2923	145.8744	124.8305	-0.72058	0.0020283	ZFP30

1.797371	10.89805	8.212916	0	0	0	5.172386	0.0020303	ATP1A3
13.48028	9.907322	12.77565	2.074648	0	1.057886	3.526501	0.0020313	AL157871.2
323.5267	392.3299	360.4558	535.2593	458.6124	428.4437	-0.40327	0.0020328	DCXR
8.088168	4.953661	15.51329	1.037324	0	0	4.666246	0.0020336	LINC00313
32.35267	37.64782	38.32694	75.72466	61.91792	70.87834	-0.94576	0.0020356	FRMD5
39.54215	29.72197	29.20148	15.55986	10.49456	7.4052	1.556668	0.0020419	C19orf33
168.0541	210.0352	155.1329	137.9641	94.45107	105.7886	0.655123	0.0020428	NRGN
532.9204	544.9027	589.5049	730.2762	655.9102	683.3941	-0.31177	0.002049	UROS
17595.36	17937.21	17177.77	20570.14	18396.97	20420.37	-0.17209	0.0020527	RPL35
7.189482	8.91659	3.650185	0	0	0	5.092208	0.0020585	KLRD1
12.58159	12.87952	13.68819	1.037324	0	3.173657	3.220992	0.002069	LINC01124
529.3256	554.81	534.7521	636.917	697.8885	665.4101	-0.3054	0.0020698	BICD1
9.885538	12.87952	11.8631	2.074648	0	0	4.048623	0.0020729	CYP2W1
10.78422	5.944393	10.95055	0	0	1.057886	4.621073	0.0020732	CABP1
223.7726	205.0816	242.7373	264.5177	309.5896	372.3758	-0.49396	0.0020788	CCDC68
59.31323	63.40686	62.05314	32.15705	31.48369	28.56291	1.002322	0.0020852	ASTN2
20330.96	20706.3	20364.38	27628.09	24684.26	20610.79	-0.24812	0.0020857	SLC7A5
725.239	833.2058	847.7554	671.1487	620.2287	653.7733	0.306461	0.0020861	DHTKD1
5.392112	10.89805	3.650185	0	0	0	5.103833	0.0021032	CHCHD2P4
984.0604	1087.824	1147.071	1268.647	1328.612	1253.595	-0.25879	0.0021106	ZFX
159.0673	114.9249	93.99226	84.02326	56.67064	67.70468	0.820697	0.0021256	-
133.0054	119.8786	125.0188	177.3824	205.6934	174.5511	-0.56059	0.0021349	JPH1
15.27765	25.75904	16.42583	6.223945	0	4.231543	2.454302	0.0021403	SKOR1
11.68291	11.88879	15.51329	1.037324	3.148369	0	3.221915	0.0021422	LRRN2
35.04873	22.78684	20.07602	4.149297	12.59348	3.173657	1.969047	0.002144	CROCCP3
355.8794	352.7007	328.5166	241.6965	261.3146	276.1082	0.41269	0.0021491	PDGFA
42.23821	44.58295	34.67676	14.52254	17.84076	16.92617	1.30112	0.0021531	AL590617.2
232.7595	220.9333	214.4484	186.7183	136.4293	129.0621	0.562768	0.0021657	SPRY1
453.8361	437.9036	477.2617	542.5205	566.7064	615.6895	-0.3329	0.0021661	MBTD1
21.56845	9.907322	4.562731	1.037324	1.049456	0	4.106954	0.0021749	RNLS
1419.024	1396.932	1320.454	1795.608	1606.718	1518.066	-0.25055	0.0021754	FIBP
68.30008	54.49027	75.74134	109.9564	111.2424	111.078	-0.7407	0.0021755	UBE2V1P2
13.48028	5.944393	8.212916	1.037324	0	0	4.619166	0.0021764	AL031846.2
93.46327	75.29565	75.74134	53.94086	39.87934	37.026	0.902135	0.0021778	AC018653.3
1948.35	2250.944	1951.936	1739.593	1461.893	1849.184	0.2841	0.002179	PDAP1
69.19877	86.1937	80.30407	49.79156	27.28586	44.4312	0.954672	0.0021912	HECW1
15.27765	25.75904	25.55129	4.149297	5.247282	7.4052	1.986403	0.0021935	EGF
125.8159	98.08249	125.9314	68.46339	61.91792	79.34143	0.739813	0.0021955	KIAA0319
217.4818	213.0074	217.186	290.4508	312.738	275.0503	-0.43922	0.0021997	FZD5
139.2962	131.7674	117.7185	199.1662	176.3087	194.651	-0.5524	0.0022008	SPATA33
24.2645	31.70343	20.07602	10.37324	1.049456	7.4052	2.011099	0.0022092	AC145285.6
145.587	140.684	125.9314	95.43382	87.10488	79.34143	0.654034	0.0022102	PPOX
925.6458	928.3161	976.4245	1363.044	1115.572	1036.728	-0.31276	0.0022139	PSMC1
146.4857	116.9064	115.8934	206.4275	188.9021	171.3775	-0.57837	0.0022204	TMEM243
3112.147	3167.371	3193.912	3421.095	3658.405	3649.706	-0.17957	0.0022238	EXT1
15.27765	23.77757	28.28893	3.111972	7.346194	6.347314	2.003266	0.0022285	AC147651.1
14.37896	15.85171	15.51329	5.186621	1.049456	0	2.867416	0.0022328	TSPAN11
8.986853	11.88879	6.387824	0	1.049456	0	4.595043	0.002236	TBC1D10C
30.5553	19.81464	20.98856	3.111972	8.395651	7.4052	1.918949	0.0022415	MAOB
2000.473	2023.075	1955.587	1757.227	1778.829	1637.607	0.208616	0.0022591	TIPARP
27.85924	20.80538	16.42583	56.0155	44.07717	47.60486	-1.18098	0.0022596	AC097461.1
229.1647	206.0723	248.2126	173.2331	149.0228	156.5671	0.513998	0.0022617	ZNF16
38.64347	28.73123	31.02657	8.298593	11.54402	14.8104	1.508091	0.0022619	NPTN-IT1
126.7146	132.7581	129.5816	175.3078	178.4076	220.0402	-0.56054	0.0022658	ZNF888

14.37896	29.72197	20.07602	39.41832	58.76956	51.8364	-1.23091	0.0022716	GPR160
27.85924	17.83318	14.60074	3.111972	6.296738	4.231543	2.146173	0.0022758	CDK5R2
18.87239	27.7405	25.55129	4.149297	7.346194	8.463085	1.854355	0.0022766	TCN2
26.06187	22.78684	19.16347	5.186621	2.098913	9.520971	2.018419	0.0022773	AC111200.2
16.17634	15.85171	18.25092	0	3.148369	5.289428	2.580301	0.0022786	THSD7B
1411.835	1410.803	1441.823	1206.408	1285.584	1111.838	0.242811	0.0022849	RPTOR
193.2173	179.3225	148.745	100.6204	126.9842	114.2517	0.608787	0.0022851	-
78.18562	88.17516	77.56643	124.4789	119.638	143.8725	-0.67055	0.002287	AC127502.2
1898.922	1955.705	1945.549	1478.187	1672.833	1766.669	0.238171	0.0023066	CIT
344.1965	284.3401	282.8893	208.5022	251.8695	189.3615	0.488821	0.002313	CLCN6
9.885538	11.88879	12.77565	1.037324	1.049456	1.057886	3.457367	0.0023139	IDO1
272.3016	228.8591	264.6384	307.0479	309.5896	533.1744	-0.58546	0.0023191	-
2011.258	2083.51	2122.583	2409.704	2316.15	2381.301	-0.19305	0.0023201	HARS
475.4045	410.1631	448.9727	341.2796	334.7766	357.5654	0.369437	0.0023216	CENPT
106.9435	8.91659	18.25092	8.298593	12.59348	9.520971	2.143645	0.0023225	-
8.986853	6.935125	10.95055	1.037324	0	0	4.577473	0.0023247	AC135782.1
24.2645	17.83318	13.68819	6.223945	2.098913	3.173657	2.277161	0.0023406	TRIM74
41.33952	55.481	49.2775	107.8817	94.45107	66.6468	-0.88298	0.0023436	FAM133DP
587.7402	642.9852	542.965	534.2219	418.7331	396.7071	0.393394	0.002351	KEAP1
17.97371	22.78684	15.51329	4.149297	0	6.347314	2.42228	0.0023534	AL118558.3
81.78036	59.44393	79.39152	34.2317	31.48369	47.60486	0.963318	0.0023614	SDHAP3
924.7472	967.9453	1012.926	1065.332	1236.26	1195.411	-0.26723	0.0023625	STK17A
138.3975	161.4893	160.6081	107.8817	89.20379	102.6149	0.618939	0.0023648	BBS9
44.93426	42.60148	0.912546	0	0	0	7.256402	0.0023685	-
1030.792	1121.509	1066.767	844.3818	889.939	948.9235	0.262432	0.0023705	CEP164
41.33952	31.70343	41.06458	16.59719	15.74185	12.69463	1.342323	0.0023828	AC245297.3
19.77108	8.91659	11.8631	1.037324	2.098913	2.115771	2.953545	0.0023835	CEACAMP11
5369.645	5605.563	5770.942	5110.896	4972.324	4783.759	0.171657	0.0023885	LAMP1
60.21191	62.41613	54.75277	29.04508	36.73097	13.75251	1.156137	0.0024055	ARVCF
759.3891	732.1511	697.1853	1024.876	819.6254	870.6399	-0.31105	0.0024091	COX17
57.51586	129.7859	111.3306	41.49297	56.67064	53.95217	0.971433	0.0024128	GMPR2
258.8214	259.5718	233.6118	364.1008	336.8755	306.7868	-0.42283	0.0024169	FAM120C
44.03558	70.34198	41.97713	21.78381	23.08804	24.33137	1.173222	0.0024189	-
2541.482	2396.581	2452.012	2663.848	3009.841	2840.423	-0.20408	0.0024228	GSK3B
46.73163	41.61075	47.4524	18.67183	15.74185	24.33137	1.210101	0.002423	SLFNL1
657.8376	741.0677	734.5997	876.5389	846.9113	864.2926	-0.27896	0.0024311	ST3GAL1
116.8291	118.8879	102.2052	59.12748	70.31358	75.10988	0.724191	0.0024337	DNAJC30
322.628	279.3865	309.3532	233.3979	238.2266	193.5931	0.45472	0.0024344	AL391244.1
68.30008	92.13809	111.3306	140.0388	130.1326	174.5511	-0.71096	0.0024355	-
18.87239	13.87025	11.8631	2.074648	2.098913	3.173657	2.603982	0.0024431	AL645608.9
36.8461	50.52734	29.20148	20.74648	12.59348	9.520971	1.439872	0.0024445	CLEC11A
29.65661	34.67563	28.28893	9.335917	11.54402	11.63674	1.509562	0.0024476	MGAT4A
8.986853	5.944393	11.8631	0	0	1.057886	4.5739	0.0024489	AP001626.1
288.478	273.4421	335.817	245.8458	203.5945	196.7667	0.474593	0.0024499	KAT5
37.74478	50.52734	31.02657	16.59719	13.64293	16.92617	1.336529	0.0024638	AL591845.1
21.56845	20.80538	20.98856	3.111972	8.395651	4.231543	2.010178	0.0024671	-
54.8198	63.40686	78.47897	76.76199	137.4788	139.6409	-0.84676	0.0024685	MINOS1
4.493426	14.86098	8.212916	0	1.049456	0	4.608901	0.0024745	REEP2
46.73163	45.57368	38.32694	29.04508	9.445107	8.463085	1.47376	0.0024781	EBI3
52.12375	53.49954	55.66532	108.919	72.41249	103.6728	-0.82145	0.0024786	AC007996.1
35.94741	17.83318	12.77565	6.223945	5.247282	4.231543	2.084668	0.0024828	SLC7A8
876.2181	867.8814	891.5577	716.791	773.4493	692.9151	0.271812	0.002485	MBD1
21.56845	13.87025	11.8631	3.111972	3.148369	2.115771	2.497955	0.0024939	TTC9B
404.4084	336.8489	313.0034	272.8162	276.007	211.5771	0.471594	0.0024953	TMEM223

31.45398	32.69416	35.5893	82.98593	57.7201	57.12583	-0.98842	0.0024963	ENDOD1
19.77108	34.67563	22.81366	56.0155	44.07717	68.76257	-1.13293	0.0024965	AC024075.1
3841.88	3891.596	3871.021	4662.772	4309.068	4188.169	-0.18155	0.0024987	RNPS1
547.2993	578.5876	520.1513	744.7987	662.207	642.1366	-0.31663	0.0025028	KCTD2
814.2089	772.7711	732.7746	671.1487	629.6738	585.0108	0.298694	0.0025041	GTF2IRD1
30.5553	34.67563	28.28893	60.1648	53.52227	72.99411	-0.99865	0.002508	LNP1
4.493426	4.953661	6.387824	36.30634	13.64293	15.86829	-2.0556	0.0025151	AC022968.1
585.9428	492.3939	337.6421	309.1226	325.3315	358.6232	0.511924	0.0025161	ABCC1
364.8662	345.7655	319.3912	252.0698	242.4244	278.2239	0.414821	0.0025189	AC026412.1
248.0371	276.4143	321.2163	375.5113	340.0239	435.8489	-0.44511	0.0025255	MORC4
13.48028	25.75904	39.23949	11.41057	4.197825	4.231543	1.981165	0.0025256	-
524.8322	492.3939	472.6989	869.2776	671.6521	507.7851	-0.45963	0.0025322	NPM3
10.78422	5.944393	2.737639	0	0	0	5.073777	0.0025345	-
127.6133	98.08249	132.3192	161.8226	185.7538	192.5352	-0.59098	0.0025356	ZNF589
152.7765	130.7766	139.6196	81.94861	96.54998	94.15183	0.635213	0.002539	PBLD
119.5251	113.9342	113.1557	174.2705	145.8744	204.1719	-0.59674	0.0025392	RPL5P34
19.77108	10.89805	20.07602	0	3.148369	5.289428	2.595464	0.0025462	CDC37P2
8.986853	15.85171	8.212916	0	1.049456	1.057886	3.974044	0.002547	AK7
342.3991	308.1177	296.5775	409.743	425.0298	395.6492	-0.3772	0.0025549	NUTM2A-AS1
9.885538	18.82391	14.60074	34.2317	52.47282	28.56291	-1.41753	0.002557	IL1RL1
26.96056	17.83318	18.25092	6.223945	6.296738	3.173657	2.00632	0.002564	CXXC4
385.536	393.3207	357.7181	267.6296	298.0456	301.4974	0.390168	0.0025652	ARHGEF39
512.2506	512.2085	509.2008	425.3029	397.744	396.7071	0.330274	0.002569	RNF19B
27.85924	23.77757	32.85166	6.223945	9.445107	11.63674	1.632039	0.0025773	IL22RA1
310.9451	316.0436	372.3189	447.0867	438.6728	414.6912	-0.37971	0.0025775	ZNF468
14.37896	6.935125	4.562731	32.15705	24.1375	28.56291	-1.70842	0.0025821	AC046185.2
35.04873	22.78684	23.7262	7.261269	11.54402	6.347314	1.698697	0.0025863	C19orf57
212.9884	186.2576	220.8362	263.4803	296.9961	288.8028	-0.45255	0.0025954	ZNF227
8.088168	10.89805	7.30037	1.037324	0	0	4.542693	0.0025984	PRPF31
69.19877	56.47173	63.87824	41.49297	28.33532	22.2156	1.042048	0.0026014	RPS13P2
79.0843	102.0454	100.3801	50.82888	59.81901	50.77851	0.801345	0.0026125	KLHL25
79.98299	64.39759	37.41439	40.45564	15.74185	17.98406	1.291821	0.0026212	C20orf194
7.189482	4.953661	6.387824	0	0	0	5.002972	0.0026278	AL121845.3
7.189482	4.953661	6.387824	0	0	0	5.002972	0.0026278	S1PR4
125.8159	113.9342	126.8439	79.87396	64.01684	83.57297	0.689219	0.0026308	PPP1R3D
159.0673	198.1464	178.8591	132.7775	94.45107	124.8305	0.605487	0.0026335	PARL
29.65661	25.75904	31.93912	7.261269	12.59348	9.520971	1.57382	0.0026569	AC127496.7
35.94741	35.66636	47.4524	14.52254	11.54402	21.15771	1.335533	0.002659	ZNF821
18.87239	31.70343	11.8631	4.149297	7.346194	2.115771	2.19354	0.0026657	AP003733.4
14.37896	18.82391	11.8631	1.037324	2.098913	4.231543	2.614478	0.0026725	IZUMO4
73.69219	85.20297	141.4447	269.7043	140.6272	136.4673	-0.86416	0.0026915	-
144.6883	149.6006	156.9579	220.95	216.188	200.9983	-0.50008	0.0026944	DEPDC7
9.885538	11.88879	12.77565	1.037324	2.098913	0	3.458518	0.0026958	LINC00638
2137.972	2038.927	1861.594	1801.832	1706.416	1648.186	0.227745	0.0026959	DCTN2
2.696056	0.990732	4.562731	13.48521	16.7913	15.86829	-2.47014	0.0027054	AC120053.1
317.2359	400.2558	330.3417	475.0945	461.7608	430.5595	-0.38557	0.002711	KLHL7
508.6559	524.0973	526.5392	449.1614	393.5461	389.3019	0.339587	0.0027185	PGS1
12.58159	12.87952	9.125462	2.074648	0	1.057886	3.460297	0.0027263	ACSM5
647.0534	661.8091	657.0333	835.0459	726.2238	844.1928	-0.29123	0.0027267	FAM120AOS
294.7688	270.4699	305.703	218.8754	223.5342	200.9983	0.437364	0.0027273	MAPK7
31.45398	38.63855	57.49041	20.74648	15.74185	14.8104	1.314107	0.0027291	CD37
6073.315	6278.27	5970.79	5769.597	5303.952	5024.957	0.186569	0.0027408	EIF4H
42.23821	52.50881	19.16347	8.298593	6.296738	21.15771	1.671514	0.0027434	AC008429.1
2103.822	2253.916	2258.552	2392.069	2624.69	2602.399	-0.20371	0.0027481	HMGCR

413.3952	343.7841	366.8436	275.9282	301.194	273.9924	0.402064	0.0027484	RECK
5.392112	9.907322	10.95055	0	1.049456	0	4.541061	0.0027578	NRN1
58.41454	79.25857	63.87824	97.50847	115.4402	121.6569	-0.73355	0.0027812	ZNF175
344.1965	358.645	404.258	476.1318	489.0467	452.7751	-0.35692	0.0027856	KNOP1
1468.452	1533.653	1408.059	2107.843	1712.713	1578.365	-0.29212	0.0027871	UBL4A
20.66976	16.84245	5.475277	3.111972	2.098913	0	3.038881	0.0027928	AC068580.3
564.3744	548.8656	623.2691	683.5966	724.1249	727.8253	-0.29793	0.0028025	METTL2B
112.3357	104.0269	125.0188	162.8599	165.8141	177.7248	-0.56778	0.0028183	RSPH4A
45.83295	68.36052	50.19004	25.9331	32.53315	9.520971	1.271128	0.0028196	TMCC2
453.8361	539.949	483.6495	379.6606	386.1999	391.4177	0.35152	0.0028228	GLI3
2.696056	9.907322	6.387824	0	0	0	5.033546	0.0028275	NPNT
3340.413	3364.526	3467.676	4015.482	3788.537	3696.253	-0.177	0.0028309	RBM12
411.5979	404.2187	378.7067	556.0057	482.7499	481.338	-0.34797	0.0028328	ZC3HC1
1563.712	1511.857	1416.272	1702.249	1746.295	1763.495	-0.21453	0.0028517	5-Mar
44.03558	64.39759	52.01513	17.63451	22.03858	32.79446	1.145984	0.0028525	PTK7
321.7293	343.7841	264.6384	252.0698	229.8309	153.3934	0.549102	0.0028527	PLA2G15
125.8159	106.0083	122.2812	154.5613	185.7538	186.1879	-0.57074	0.0028539	RAB33B
86.27379	69.35125	97.64245	113.0683	164.7646	134.3515	-0.70051	0.0028554	TRMT10B
1282.424	1342.442	1328.667	1612.002	1540.602	1459.882	-0.22265	0.0028707	ADD1
102.4501	93.12882	91.25462	53.94086	57.7201	58.18371	0.756494	0.0028758	CSRP2
8.986853	1.981464	8.212916	0	0	0	5.055321	0.0028781	S100A8
106.0449	89.1659	108.593	139.0014	146.9239	181.9563	-0.62128	0.0028858	SUPT3H
354.9807	337.8397	334.9045	425.3029	468.0575	422.0964	-0.35589	0.0029022	PIBF1
20.66976	15.85171	14.60074	28.00775	38.82988	62.41525	-1.33601	0.0029037	KCNAB1
3.594741	7.925857	7.30037	22.82113	28.33532	16.92617	-1.86124	0.0029044	FAM69B
308.2491	301.1826	337.6421	425.3029	408.2385	390.3598	-0.3696	0.0029046	GFOD2
5.392112	4.953661	9.125462	21.78381	22.03858	24.33137	-1.80362	0.0029094	GMPSP1
98.85538	79.25857	118.631	112.031	178.4076	204.1719	-0.73502	0.0029197	ERVK3-1
274.9977	293.2567	314.8284	223.0247	220.3858	212.635	0.428613	0.0029222	AC016747.1
199.5081	204.0908	207.148	293.5627	262.3641	270.8187	-0.43705	0.0029252	C12orf66
77.28693	75.29565	67.52842	41.49297	32.53315	44.4312	0.893792	0.0029287	CISH
91.6659	93.12882	80.30407	117.2176	130.1326	177.7248	-0.68129	0.0029361	ZNF225
363.9675	332.886	327.6041	379.6606	549.9151	447.4856	-0.42633	0.0029445	B3GNT2
129.4107	125.823	140.5321	74.68734	77.65977	98.38337	0.659299	0.0029477	PAXIP1-AS2
23.36582	11.88879	10.95055	48.75423	34.63206	35.96811	-1.36486	0.0029583	TOX
3772.681	3866.828	3666.611	3707.396	2932.181	2607.688	0.289882	0.0029591	LSM4
415.1926	401.2465	360.4558	318.4585	308.5402	268.703	0.393597	0.0029673	INPP5K
408.9018	421.0612	379.6192	294.6001	294.8972	339.5813	0.38047	0.0029697	UBE2E2
353.1833	298.2104	302.0528	260.3684	232.9793	206.2877	0.446879	0.0029747	ACSF3
16.17634	15.85171	16.42583	3.111972	5.247282	0	2.532812	0.0029822	ZBTB7C
58.41454	56.47173	51.10259	29.04508	22.03858	29.6208	1.040252	0.002984	AC104794.2
462.8229	463.6627	453.5355	319.4958	392.4967	361.7969	0.362093	0.0029928	SUFU
29.65661	30.7127	27.37639	47.71691	64.01684	64.53103	-1.00668	0.0029975	LHX4
12.58159	5.944393	7.30037	0	0	1.057886	4.521138	0.0030018	EPN3
26.06187	36.65709	26.46384	8.298593	6.296738	14.8104	1.600019	0.003006	HSD11B1L
35.94741	26.74977	31.93912	12.44789	10.49456	11.63674	1.453775	0.0030119	HSD11B1
2379.719	2391.627	2395.434	2266.553	1985.571	1843.895	0.233337	0.0030172	TUBB6
9.885538	15.85171	13.68819	4.149297	0	0	3.237399	0.0030248	PSG2
37.74478	14.86098	46.53986	10.37324	13.64293	7.4052	1.660804	0.0030302	GMPR
71.89482	123.8415	69.35351	46.67959	48.27499	45.48908	0.913512	0.0030369	NEIL2
2562.152	2346.054	2399.084	2075.686	2212.254	2076.63	0.1995	0.0030558	ITPRIPL2
20.66976	27.7405	20.07602	41.49297	65.06629	44.4312	-1.14368	0.0030687	IL17RB
64.70534	85.20297	71.17861	116.1803	119.638	118.4832	-0.68258	0.0030702	ZFP1
844.7642	883.7331	858.706	947.0769	1081.989	1071.638	-0.26131	0.0030755	NRIP1

1045.171	976.8619	1074.067	876.5389	867.9004	862.1768	0.248664	0.003082	SP100
128.512	93.12882	80.30407	33.19437	69.26412	58.18371	0.911817	0.0030924	POU6F1
15.27765	12.87952	15.51329	4.149297	2.098913	1.057886	2.576763	0.0030926	LSMEM2
396.3202	415.1168	415.2085	513.4754	524.7282	496.1484	-0.32316	0.0030981	METTL14
62.00928	47.55514	58.40296	23.85846	26.23641	31.73657	1.039411	0.0031168	GOLGA2P5
17.97371	17.83318	12.77565	5.186621	0	3.173657	2.535975	0.0031236	HNRNPA1P4
39.54215	23.77757	31.02657	13.48521	10.49456	9.520971	1.495279	0.0031281	HSD17B1P1
11.68291	118.8879	27.37639	15.55986	10.49456	16.92617	1.873923	0.0031289	SMIM11A
12.58159	4.953661	8.212916	0	0	1.057886	4.5174	0.0031343	NKPD1
290.2753	350.7192	357.7181	262.443	235.0782	245.4295	0.426102	0.0031403	DPCD
919.355	962.0009	876.0444	857.8671	660.108	695.0309	0.316878	0.0031471	GAL
112.3357	116.9064	90.34208	171.1585	156.369	153.3934	-0.59075	0.0031473	CBWD6
76.38825	47.55514	51.10259	32.15705	25.18695	26.44714	1.064342	0.0031511	AC098864.1
656.0403	673.6979	680.7595	894.1734	772.3999	782.8354	-0.28509	0.0031595	RPL26L1
116.8291	95.11029	140.5321	171.1585	174.2098	185.13	-0.58774	0.0031622	PRTG
113.2343	110.962	95.81735	57.05283	71.36303	65.58891	0.72203	0.0031733	AL139095.2
7.189482	7.925857	10.03801	0	0	1.057886	4.480885	0.0031806	CCDC177
12.58159	18.82391	10.03801	1.037324	3.148369	2.115771	2.716516	0.0031835	LRRC73
95.26064	73.31418	73.0037	40.45564	32.53315	56.06794	0.9059	0.003188	STK11
333.4122	387.3763	365.931	335.0557	225.6331	189.3615	0.534056	0.0031916	LRP3
177.041	207.063	155.1329	118.255	137.4788	100.4991	0.596777	0.0031955	CRELD1
35.04873	49.53661	36.50185	25.9331	10.49456	7.4052	1.462228	0.0031991	IL1R1
307.3504	306.1362	295.665	365.1381	409.288	399.8808	-0.36919	0.0031995	MEGF9
541.0085	610.291	550.2654	795.6276	668.5037	662.2364	-0.32233	0.0032075	EDC3
26.06187	14.86098	38.32694	6.223945	10.49456	6.347314	1.783693	0.0032081	PMEL
312.7425	363.5987	306.6155	398.3325	439.7222	434.791	-0.3739	0.0032101	ZNF267
17.07502	13.87025	21.90111	5.186621	4.197825	2.115771	2.199673	0.0032138	IQCN
79.0843	78.26784	100.3801	51.86621	47.22554	47.60486	0.813532	0.0032199	AC100861.1
374.7518	316.0436	369.5812	442.9374	484.8488	437.9647	-0.36402	0.0032208	TMEM68
16889.89	18236.41	16850.17	14641.83	11537.72	9577.039	0.539636	0.0032215	RPL10
15.27765	8.91659	5.475277	20.74648	48.27499	25.38926	-1.66694	0.0032283	LINC01297
10.78422	15.85171	13.68819	4.149297	0	1.057886	2.946104	0.0032291	OR52A1
123.1199	122.8508	96.7299	216.8007	152.1712	157.625	-0.62068	0.0032294	RPS7P10
44.93426	48.54588	35.5893	24.89578	8.395651	17.98406	1.330121	0.0032375	RNASEK
7.189482	9.907322	1.825092	0	0	0	5.029046	0.0032411	MEFV
846.5615	854.0111	848.668	685.6713	773.4493	615.6895	0.296978	0.0032444	KLF11
123.1199	115.9157	135.9694	157.6733	204.644	190.4194	-0.5587	0.003245	SHQ1
2630.452	2243.018	2341.594	2892.06	2976.258	2598.167	-0.23049	0.0032459	RHEB
14.37896	6.935125	16.42583	1.037324	3.148369	0	3.173826	0.0032514	GRIK5
0.898685	0	0.912546	13.48521	9.445107	6.347314	-3.98372	0.0032556	AC017074.1
137.4988	138.7025	156.0454	93.35917	101.7973	88.8624	0.60608	0.0032603	TRPC1
1.797371	2.972197	4.562731	16.59719	11.54402	19.04194	-2.33858	0.0032684	AC234772.2
43.13689	42.60148	23.7262	22.82113	6.296738	5.289428	1.666357	0.0032757	MDFI
15.27765	14.86098	29.20148	34.2317	61.91792	46.54697	-1.26294	0.0032885	AC011043.1
10.78422	7.925857	6.387824	1.037324	0	0	4.478162	0.0032922	CASKIN1
170.7502	177.3411	167.9085	206.4275	245.5728	272.9345	-0.49053	0.0032956	SUZ12P1
10.78422	8.91659	21.90111	4.149297	1.049456	0	2.9953	0.0033126	MAPT
121.3225	156.5357	154.2203	88.17255	96.54998	95.20971	0.625353	0.0033233	PMVK
393.6242	441.8666	427.9842	350.6156	341.0733	249.661	0.423911	0.0033367	CCDC94
107.8422	115.9157	117.7185	75.72466	54.57173	78.28354	0.710946	0.0033457	JMJD4
8.986853	7.925857	1.825092	0	0	0	5.016733	0.0033457	AC016737.1
23.36582	16.84245	26.46384	5.186621	9.445107	3.173657	1.906033	0.0033477	ACTA2
656.9389	695.494	566.6912	834.0086	801.7846	739.4621	-0.30833	0.0033478	VAC14
22.46713	23.77757	13.68819	7.261269	4.197825	3.173657	2.03091	0.0033576	LINC01703

989.4525	1005.593	1028.44	1205.371	1160.699	1174.253	-0.22771	0.0033588	SPATS2
1059.55	1135.379	1014.751	1013.466	816.477	640.0208	0.377527	0.0033593	PSMG3
428.6729	409.1724	448.0602	477.1691	578.2504	579.7213	-0.34606	0.0033773	GMCL1
48.52901	29.72197	41.06458	14.52254	20.98913	12.69463	1.309607	0.0033792	THSD1
8.088168	5.944393	7.30037	14.52254	28.33532	29.6208	-1.76062	0.0033798	AP001992.1
70.09745	74.30491	58.40296	116.1803	108.094	104.7307	-0.69932	0.0033803	ZNF607
1132.343	1092.778	1094.143	995.8312	873.1477	925.65	0.248231	0.0033833	INTS11
29.65661	35.66636	18.25092	4.149297	5.247282	14.8104	1.788426	0.0033899	CLTRN
26.96056	30.7127	25.55129	11.41057	9.445107	7.4052	1.556365	0.0033917	SNHG9
521.2375	558.7729	495.5126	450.1987	372.557	416.807	0.345404	0.0033918	VDR
1469.35	1527.709	1349.656	1848.512	1738.949	1563.555	-0.24529	0.0033993	POLR3H
107.8422	95.11029	113.1557	71.57537	67.16521	50.77851	0.738432	0.0034019	PPM1M
5.392112	8.91659	3.650185	0	0	0	4.953657	0.0034088	AC104667.2
5.392112	8.91659	3.650185	0	0	0	4.953657	0.0034088	AL390778.2
234.5569	217.9611	239.0871	275.9282	313.7874	340.6392	-0.42701	0.003415	MTURN
16.17634	30.7127	15.51329	2.074648	2.098913	9.520971	2.188357	0.0034218	INSL4
42.23821	31.70343	23.7262	10.37324	12.59348	12.69463	1.454552	0.0034272	RARA-AS1
7.189482	4.953661	5.475277	0	0	0	4.929944	0.0034307	LINC01301
385.536	353.6914	343.1174	247.9205	269.7103	302.5553	0.400647	0.0034317	ZNF462
1530.461	1567.338	1513.002	1412.835	1285.584	1246.189	0.224902	0.0034405	CCAR2
882.5089	960.0195	786.6148	796.6649	682.1466	597.7054	0.339795	0.0034414	MRPL14
27.85924	38.63855	46.53986	16.59719	15.74185	12.69463	1.326671	0.0034465	AC092651.2
131.2081	142.6654	138.707	162.8599	215.1386	224.2718	-0.54589	0.0034473	ZNF81
39.54215	24.7683	34.67676	57.05283	62.96738	74.052	-0.96724	0.0034539	-
31.45398	16.84245	19.16347	10.37324	4.197825	2.115771	2.014046	0.0034543	UCN
124.0186	99.07322	116.8059	172.1958	163.7152	166.0881	-0.56093	0.003461	FAM198B
4920.302	4468.202	4733.377	5172.098	5618.789	5199.508	-0.1791	0.0034685	F3
9.885538	31.70343	12.77565	40.45564	40.9288	57.12583	-1.35912	0.0034721	LINC01446
419.686	469.6071	468.1362	325.7198	319.0347	398.8229	0.379199	0.003475	RPL22P1
13.48028	6.935125	20.07602	2.074648	3.148369	0	2.954564	0.0034821	CSRNP3
1012.818	1104.666	964.5614	939.8157	834.3178	663.2943	0.338021	0.0034856	TSR3
367.5623	392.3299	391.4823	450.1987	518.4314	487.6853	-0.33917	0.0035044	SMYD4
47.63032	26.74977	33.76421	17.63451	7.346194	14.8104	1.444077	0.0035092	PROCA1
1974.412	2048.834	1932.773	1635.86	1701.169	1821.679	0.207246	0.0035105	PON2
24.2645	15.85171	21.90111	59.12748	48.27499	33.85234	-1.18443	0.0035117	AL132656.4
697.3798	727.1974	699.0104	596.4614	601.3385	545.869	0.28404	0.0035179	FOXF2
57.51586	72.32345	65.70333	45.64226	25.18695	26.44714	1.005322	0.0035245	DLK2
41.33952	46.56441	41.06458	15.55986	17.84076	23.27348	1.186258	0.0035275	PDLIM3
83.57773	61.4254	61.1406	20.74648	45.12662	35.96811	1.019089	0.0035371	ATP6AP1L
983.1617	989.7414	1016.576	1236.49	1163.847	1119.243	-0.23557	0.0035548	DDX50
16.17634	31.70343	14.60074	6.223945	5.247282	4.231543	1.987593	0.0035909	AL139352.1
91.6659	60.43466	77.56643	116.1803	109.1435	153.3934	-0.71872	0.0036018	ECHDC2
37.74478	38.63855	52.01513	25.9331	13.64293	13.75251	1.266904	0.0036118	MFSD13A
12.58159	15.85171	8.212916	3.111972	0	1.057886	3.128752	0.0036255	IL12RB1
45.83295	49.53661	62.96569	81.94861	109.1435	84.63085	-0.79971	0.0036261	-
8914.958	9427.807	8661.889	10824.48	9675.988	9948.357	-0.17328	0.0036336	RPL37A
4533.867	4837.745	4568.206	5150.314	5271.419	5141.324	-0.15908	0.0036396	USP22
4476.351	4441.452	4579.157	4149.297	4078.187	3495.254	0.203297	0.0036404	AXL
1497.21	1523.746	1349.656	1314.29	1244.655	1100.201	0.256014	0.0036441	LRRC41
35.94741	24.7683	24.63875	9.335917	8.395651	11.63674	1.541048	0.0036525	DZANK1
1736.26	1793.225	1608.819	1921.124	1999.214	2004.693	-0.20577	0.0036558	ARID1A
20.66976	7.925857	13.68819	1.037324	4.197825	1.057886	2.752019	0.0036632	NRTN
264.2135	300.1918	270.1137	201.2409	200.4462	216.8666	0.43136	0.0036689	PCCA
11.68291	7.925857	15.51329	2.074648	1.049456	1.057886	3.069892	0.0036735	CYS1

7.189482	7.925857	2.737639	0	0	0	4.946324	0.0036748	KCNG1
2522.61	2698.754	2552.392	2970.896	2916.439	2897.549	-0.17668	0.0036762	PLAUR
1933.072	1907.159	2019.465	2236.471	2301.458	2153.855	-0.19145	0.0036829	DLST
37.74478	48.54588	43.80222	63.27677	75.56086	99.44125	-0.87442	0.0036855	HDX
20.66976	30.7127	13.68819	4.149297	4.197825	8.463085	1.951384	0.0036911	AP000924.1
112.3357	91.14736	114.0683	160.7852	152.1712	160.7986	-0.57537	0.0036965	BEND7
21.56845	22.78684	19.16347	4.149297	9.445107	3.173657	1.920831	0.0037031	LINC01315
24.2645	21.79611	16.42583	35.26902	48.27499	56.06794	-1.15957	0.0037203	MIR4453HG
106.9435	104.0269	100.3801	160.7852	134.3304	171.3775	-0.58323	0.0037231	TBC1D19
600.3218	768.8082	634.2196	564.3043	537.3216	466.5276	0.35227	0.0037246	CCDC97
1386.671	1475.2	1402.584	1251.013	1173.292	1240.9	0.218174	0.0037529	GSS
16.17634	21.79611	10.03801	4.149297	2.098913	3.173657	2.345784	0.0037558	STXBP5-AS1
168.0541	159.5079	187.072	210.5768	258.1663	249.661	-0.48035	0.0037608	TNRC6C
14.37896	7.925857	8.212916	1.037324	0	1.057886	3.865782	0.0037654	PKD1L2
4.493426	10.89805	2.737639	0	0	0	4.965637	0.0037747	SCO2
1995.081	2000.288	1979.313	1581.919	1650.795	1881.979	0.224309	0.0037823	GSE1
1394.76	1423.682	1325.93	1264.498	1176.441	1025.091	0.257579	0.0037836	PYGB
7.189482	8.91659	10.95055	21.78381	34.63206	24.33137	-1.57772	0.0038136	HNRNPA1P33
3701.685	3513.136	3618.246	3148.279	3251.216	3258.288	0.165841	0.0038161	RCN1
5.392112	9.907322	9.125462	0	0	1.057886	4.436611	0.0038226	LINC00602
10.78422	14.86098	12.77565	0	3.148369	2.115771	2.870319	0.0038324	AC093535.1
53.02243	47.55514	36.50185	23.85846	18.89021	19.04194	1.148991	0.003833	GPR155
1205.137	1188.879	1137.945	1474.038	1391.579	1285.331	-0.23311	0.0038511	UBE2Q1
212.0897	177.3411	190.7222	256.2191	282.3038	252.8347	-0.4468	0.0038723	AP4B1
124.9173	116.9064	125.0188	78.83663	74.5114	82.51508	0.637724	0.0038763	-
78.18562	96.10102	124.1063	63.27677	51.42336	58.18371	0.787213	0.003885	NOP14-AS1
603.9165	576.6061	620.5314	710.567	719.9271	747.9252	-0.274	0.0038882	AK3
8.986853	11.88879	16.42583	2.074648	2.098913	1.057886	2.832289	0.00389	AL157935.2
562.577	564.7173	539.3148	445.0121	438.6728	465.4697	0.30479	0.0038931	SGMS1
502.3651	488.431	560.3034	590.2374	662.207	673.8732	-0.312	0.0038986	STYX
31.45398	23.77757	24.63875	11.41057	9.445107	5.289428	1.610735	0.0039061	CCDC190
1629.316	1806.105	1746.613	1606.815	1433.557	1346.688	0.239896	0.0039103	RAB35
134.8028	132.7581	168.8211	100.6204	65.06629	106.8465	0.679689	0.0039128	CPEB3
8.088168	6.935125	3.650185	21.78381	24.1375	19.04194	-1.79979	0.0039267	AC021016.2
22.46713	30.7127	22.81366	5.186621	9.445107	9.520971	1.653119	0.0039272	TP53TG1
2057.091	2217.259	2033.153	1989.588	1741.048	1579.423	0.248021	0.0039318	THOP1
648.8508	659.8276	730.9495	764.5079	889.939	832.556	-0.28586	0.0039372	PDPK1
11.68291	9.907322	9.125462	2.074648	0	0	3.877169	0.0039453	AP003119.1
11.68291	8.91659	11.8631	0	1.049456	2.115771	3.366209	0.0039613	AC105020.1
17.97371	11.88879	14.60074	2.074648	2.098913	4.231543	2.406927	0.0039631	AL356215.1
6.290797	2.972197	8.212916	0	0	0	4.919845	0.0039673	NTN3
505.0611	571.6525	502.813	430.4895	436.5738	386.1283	0.333057	0.0039732	TMEM120B
1318.371	1359.285	1256.576	1550.8	1585.729	1442.956	-0.21939	0.0039766	ACOT9
717.1509	772.7711	761.9761	895.2107	859.5047	935.1709	-0.25669	0.0039822	LAP3
258.8214	236.785	209.8856	186.7183	133.281	173.4933	0.515541	0.0039987	NRIP3
9.885538	8.91659	11.8631	0	2.098913	0	3.870943	0.004005	DPY19L2P1
1531.36	1603.005	1615.207	2108.88	1845.994	1680.98	-0.24703	0.0040061	EIF6
570.6652	559.7637	530.1894	423.2282	456.5135	460.1803	0.309635	0.0040091	PPP2R5A
104.2475	99.07322	68.44097	225.0993	130.1326	112.1359	-0.78318	0.0040112	DCAF11
124.9173	97.09175	85.77934	67.42607	68.21466	32.79446	0.869578	0.0040181	ZNHIT2
40.44084	41.61075	41.06458	15.55986	23.08804	14.8104	1.203418	0.0040223	LINC01980
7.189482	10.89805	11.8631	0	1.049456	1.057886	3.833875	0.0040302	CPS1-IT1
807.0194	790.6043	671.634	690.8579	586.6461	453.833	0.389949	0.0040331	RPL13P12
9.885538	9.907322	4.562731	0	1.049456	0	4.433123	0.0040352	CCR5

77.28693	116.9064	90.34208	63.27677	44.07717	55.01006	0.807082	0.0040369	APLN
1017.312	1001.63	1018.402	1378.604	1177.49	1101.259	-0.26807	0.0040394	IP6K1
2052.597	2155.833	2105.244	1962.617	1814.51	1615.391	0.22729	0.0040478	KIFC3
227.3674	222.9147	210.7982	318.4585	266.5619	299.3816	-0.42003	0.0040497	C10orf88
23.36582	15.85171	14.60074	42.53029	43.02771	38.08388	-1.19755	0.004053	AC097376.2
64.70534	52.50881	49.2775	29.04508	10.49456	33.85234	1.18241	0.0040542	SNRPGP15
5.392112	2.972197	9.125462	0	0	0	4.920815	0.0040562	PTGER1
816.9049	933.2697	883.3447	832.9713	654.8608	561.7373	0.36113	0.0040575	SLC25A11
15.27765	12.87952	10.03801	0	2.098913	3.173657	2.862295	0.0040766	MMP24
638.0665	641.0037	614.1436	451.236	565.657	501.4378	0.318385	0.0040783	RERE
514.9467	533.0139	563.041	473.0198	384.101	418.9227	0.336116	0.0040865	GNB5
1.797371	10.89805	5.475277	0	0	0	4.96872	0.0041124	SERPINA5
243.5437	293.2567	225.3989	364.1008	344.2217	312.0763	-0.42253	0.004114	POMGNT2
10.78422	14.86098	13.68819	3.111972	2.098913	1.057886	2.64551	0.0041202	AL592071.1
30.5553	18.82391	19.16347	11.41057	4.197825	2.115771	1.94936	0.0041335	SMPD3
27.85924	28.73123	32.85166	51.86621	52.47282	71.93623	-0.97812	0.0041338	ZNF234
556.2862	545.8934	512.851	426.3402	460.7113	409.4018	0.316892	0.0041364	PHKG2
1229.401	1334.516	1207.299	1160.766	1038.962	927.7657	0.269547	0.0041396	KCTD5
44.93426	44.58295	46.53986	11.41057	25.18695	23.27348	1.185545	0.0041452	KBTBD3
64.70534	42.60148	58.40296	34.2317	23.08804	22.2156	1.060188	0.0041548	PSMG3-AS1
53.02243	40.62002	35.5893	23.85846	18.89021	12.69463	1.220548	0.0041641	AP002990.1
197.7108	126.8137	210.7982	128.6282	66.11575	124.8305	0.745551	0.0041916	MICA
8.986853	4.953661	10.03801	1.037324	0	0	4.414129	0.0042059	AC011448.1
11.68291	14.86098	16.42583	41.49297	34.63206	29.6208	-1.30068	0.0042071	ACTL8
2.696056	8.91659	0	21.78381	13.64293	22.2156	-2.3346	0.0042101	RELN
26.06187	9.907322	11.8631	42.53029	32.53315	46.54697	-1.33958	0.0042149	EPHX4
11.68291	5.944393	6.387824	0	1.049456	0	4.415944	0.004215	CRYAB
35.04873	30.7127	40.15203	9.335917	14.69239	17.98406	1.336242	0.0042335	SENCR
27.85924	29.72197	23.7262	5.186621	11.54402	10.57886	1.574623	0.0042455	HRK
14.37896	13.87025	20.07602	2.074648	6.296738	1.057886	2.357997	0.0042523	SEMA3G
6.290797	14.86098	13.68819	2.074648	1.049456	1.057886	3.053709	0.0042583	PLCL1
2997.115	3154.491	2990.414	2809.074	2708.647	2509.305	0.18743	0.0042619	TSPAN14
213.8871	198.1464	178.8591	139.0014	139.5777	138.583	0.502371	0.0042639	NAIF1
94.36195	75.29565	75.74134	118.255	151.1217	117.4253	-0.6552	0.0042665	TIGD1
14.37896	14.86098	11.8631	2.074648	3.148369	2.115771	2.485259	0.0042828	LINC00917
15.27765	11.88879	12.77565	1.037324	1.049456	4.231543	2.665188	0.0042846	BEAN1
5.392112	5.944393	5.475277	0	0	0	4.860834	0.0042859	AL391832.2
51.22506	35.66636	37.41439	17.63451	17.84076	19.04194	1.190769	0.0042915	MTMR9LP
79.98299	71.33272	65.70333	37.34367	40.9288	42.31543	0.848258	0.0043002	PBX4
95.26064	89.1659	81.21661	39.41832	52.47282	61.35737	0.794365	0.0043039	IQCG
122.2212	136.721	162.4332	186.7183	195.1989	225.3296	-0.52696	0.0043085	SETDB2
175.2436	159.5079	140.5321	102.6951	112.2918	105.7886	0.567393	0.0043202	FAHD2B
12.58159	15.85171	11.8631	38.38099	32.53315	29.6208	-1.32231	0.0043232	SNX25P1
38.64347	28.73123	45.62731	21.78381	13.64293	8.463085	1.364427	0.0043414	KCND1
10.78422	16.84245	14.60074	1.037324	2.098913	4.231543	2.520677	0.0043443	-
242.645	223.9055	264.6384	281.1148	330.5787	376.6073	-0.43372	0.004347	ZNF236
11.68291	11.88879	20.07602	5.186621	1.049456	1.057886	2.577327	0.0043499	GRAMD2A
163.5607	149.6006	160.6081	110.9937	108.094	104.7307	0.549469	0.0043647	-
393.6242	300.1918	375.0565	252.0698	280.2048	270.8187	0.413811	0.0043648	ZNF692
15.27765	6.935125	11.8631	0	0	3.173657	3.435864	0.0043733	AC005622.1
2603.491	2690.829	2776.878	2629.617	2110.457	2032.198	0.253023	0.0043786	ARHGDIA
960.6946	957.0473	946.3104	1119.273	1113.473	1109.722	-0.22288	0.0043796	RANBP3
24.2645	35.66636	41.06458	15.55986	10.49456	12.69463	1.380701	0.0043994	-
5804.608	5303.389	5531.855	5609.849	6750.103	7064.561	-0.22312	0.0044034	DDX17

6.290797	4.953661	5.475277	0	0	0	4.854062	0.0044236	AC015660.1
9.885538	14.86098	10.03801	0	2.098913	2.115771	3.047797	0.0044243	ROR2
7.189482	8.91659	7.30037	0	1.049456	0	4.375877	0.0044492	-
195.9134	151.582	161.5207	235.4726	242.4244	229.5612	-0.47352	0.0044503	-
4.493426	8.91659	3.650185	0	0	0	4.879066	0.0044766	CX3CL1
4.493426	8.91659	3.650185	0	0	0	4.879066	0.0044766	CBFA2T3
472.7085	452.7646	507.3757	548.7445	613.932	611.4579	-0.30766	0.0044788	PPM1L
10.78422	12.87952	17.33838	0	2.098913	4.231543	2.700895	0.0044789	FSCN2
58.41454	53.49954	66.61587	96.47114	118.5886	83.57297	-0.74113	0.0045083	ZFAND4
187.8252	164.4615	222.6613	261.4057	265.5125	263.4135	-0.45762	0.0045088	PPP3CC
4.493426	4.953661	7.30037	0	0	0	4.856125	0.0045092	-
26.96056	12.87952	12.77565	49.79156	28.33532	51.8364	-1.29992	0.0045139	ABCA11P
35.94741	33.68489	48.36495	10.37324	10.49456	25.38926	1.353447	0.0045197	-
1383.975	1450.432	1637.108	1131.721	1326.513	1285.331	0.256501	0.0045242	USP33
58.41454	55.481	63.87824	34.2317	23.08804	33.85234	0.963801	0.0045262	FOXD2-AS1
17.07502	24.7683	15.51329	3.111972	6.296738	5.289428	1.96315	0.0045327	AC010186.2
110.5383	84.21224	116.8059	137.9641	158.4679	176.6669	-0.60025	0.0045374	AC068831.7
26.96056	27.7405	30.11403	41.49297	74.5114	56.06794	-1.02018	0.0045561	-
1041.576	1041.26	1086.843	1118.235	1295.029	1369.962	-0.25507	0.0045733	PDK1
2566.645	2426.303	2335.206	2649.326	2807.296	2907.07	-0.19056	0.0045736	WDR26
8.088168	4.953661	20.98856	1.037324	2.098913	0	3.440009	0.004587	TBC1D3D
2720.32	2722.532	2757.715	3035.21	2994.099	3187.41	-0.16848	0.0046117	NUDC
54.8198	51.51807	79.39152	36.30634	26.23641	31.73657	0.979049	0.0046256	EEPD1
3292.783	3640.941	3322.581	2958.448	3128.429	2946.212	0.182957	0.0046284	PTPRJ
17.07502	4.953661	9.125462	2.074648	0	0	3.901082	0.0046289	CDH23
149.1818	189.2298	144.1823	221.9874	228.7815	224.2718	-0.48616	0.0046333	MKKS
7626.243	7795.081	7775.806	9058.952	8447.074	8276.898	-0.15252	0.0046424	CTNNB1
377.4478	362.608	359.5432	311.1972	255.0179	280.3397	0.37727	0.0046425	NT5DC3
142.891	133.7488	123.1937	96.47114	86.05542	79.34143	0.610333	0.0046478	CA13
8.986853	13.87025	18.25092	4.149297	2.098913	0	2.712097	0.0046654	CAMK1G
369.3596	438.8944	482.7369	497.9156	565.657	591.3581	-0.35849	0.0046772	KAT2B
62.90797	49.53661	83.04171	96.47114	134.3304	100.4991	-0.75855	0.0046785	FAM76A
5.392112	7.925857	10.03801	0	0	1.057886	4.373296	0.0046833	AC124067.4
5.392112	7.925857	10.03801	1.037324	0	0	4.373297	0.0046894	AC040170.1
1937.565	1990.381	1954.674	1919.05	1539.552	1372.078	0.284042	0.004691	AAMP
25.16319	41.61075	37.41439	53.94086	61.91792	86.74663	-0.96203	0.004694	TARID
139.2962	118.8879	105.8554	174.2705	173.1603	180.8985	-0.53676	0.004695	ZNF232
5.392112	8.91659	2.737639	0	0	0	4.878099	0.0047027	TREH
203.1029	195.1742	198.9351	248.9578	282.3038	268.703	-0.42133	0.0047205	SUSD1
76.38825	105.0176	94.90481	61.20212	48.27499	52.89428	0.76528	0.0047206	ITPKB
686.5956	654.874	719.0864	878.6135	855.3069	758.504	-0.27427	0.0047221	SCOC
16.17634	6.935125	9.125462	1.037324	0	2.115771	3.359298	0.0047322	ANKRD24
42.23821	36.65709	39.23949	11.41057	16.7913	22.2156	1.230314	0.0047384	PLTP
278.5924	268.4884	310.2657	231.3233	178.4076	221.0981	0.442966	0.0047466	CCRL2
26.06187	16.84245	30.11403	6.223945	7.346194	9.520971	1.664259	0.0047492	PURPL
171.6489	190.2206	188.8971	127.5909	137.4788	121.6569	0.509632	0.0047592	CYB5D2
13.48028	10.89805	14.60074	31.11972	39.87934	27.50503	-1.33492	0.0047598	AC012615.1
52.12375	47.55514	34.67676	74.68734	79.75868	82.51508	-0.81889	0.0047683	SNORA33
16.17634	16.84245	17.33838	1.037324	7.346194	2.115771	2.263074	0.0047748	AC026471.4
18.87239	13.87025	10.95055	2.074648	2.098913	4.231543	2.380707	0.0047758	AC018665.1
8.088168	15.85171	20.98856	2.074648	2.098913	4.231543	2.41921	0.0047789	C4orf47
42.23821	59.44393	45.62731	21.78381	22.03858	26.44714	1.066229	0.0047872	LINC01138
17.07502	5.944393	14.60074	3.111972	1.049456	1.057886	2.849728	0.0047908	AC025031.2
17.97371	5.944393	16.42583	1.037324	3.148369	2.115771	2.684145	0.0048089	IL20

215.6845	251.646	260.9882	193.9796	180.5065	145.9882	0.483966	0.0048105	APOBEC3B
4.493426	3.962929	8.212916	0	0	0	4.850331	0.0048175	LY6D
17.97371	12.87952	10.03801	0	5.247282	0	2.963712	0.0048428	ANO7L1
86.27379	67.36979	76.65388	58.09015	36.73097	25.38926	0.937722	0.0048474	RASSF7
44.93426	59.44393	32.85166	17.63451	17.84076	25.38926	1.171275	0.004851	TJP2
60.21191	45.57368	49.2775	116.1803	91.3027	66.6468	-0.82086	0.0048612	RPS2P55
386.4347	409.1724	400.6078	505.1769	467.0081	517.3061	-0.31665	0.0048634	JADE3
154.5739	92.13809	111.3306	101.6578	35.68152	48.66274	0.945095	0.0048654	VPS53
1092.801	1112.592	1043.953	924.2558	917.2248	934.1131	0.22717	0.0048824	ARHGAP17
579.652	577.5969	589.5049	827.7847	664.3059	670.6995	-0.30835	0.0048897	NLE1
11.68291	10.89805	10.03801	3.111972	0	0	3.379834	0.0049002	CD79B
124.9173	102.0454	123.1937	161.8226	186.8032	161.8565	-0.54225	0.0049217	LRRC37B
18.87239	11.88879	30.11403	6.223945	6.296738	3.173657	1.95711	0.0049295	BATF2
3274.809	3311.027	3130.034	2974.008	2925.884	2629.904	0.187696	0.0049334	PPP1CA
491.5808	434.9314	500.0753	302.8986	374.6559	417.8648	0.381958	0.0049532	WDFY2
28.75793	29.72197	27.37639	11.41057	12.59348	7.4052	1.449701	0.0049675	TAS1R3
0	0	0.912546	7.261269	13.64293	2.115771	-4.52096	0.0049685	IRF4
45.83295	32.69416	39.23949	21.78381	12.59348	15.86829	1.229692	0.0049721	-
385.536	490.4124	493.6875	377.586	354.7162	319.4815	0.380128	0.0049729	COL13A1
273.2003	238.7665	251.8628	287.3388	367.3097	362.8548	-0.41279	0.0049786	ZSCAN9
571.5638	574.6247	528.3643	734.4255	647.5146	661.1785	-0.28733	0.0049826	CUTC
39.54215	38.63855	40.15203	16.59719	23.08804	10.57886	1.234875	0.0049861	RAB11B-AS1
587.7402	649.9203	678.0218	822.598	702.0863	821.9772	-0.293	0.0049924	MAGOH
669.5205	690.5403	618.7063	890.0241	825.9221	713.0149	-0.29621	0.004997	DDX51
17.97371	20.80538	20.07602	8.298593	1.049456	5.289428	2.005285	0.0050159	YTHDF3-AS1
4229.213	4262.13	4062.656	4927.29	4505.316	4635.655	-0.16439	0.0050185	EIF3F
38.64347	26.74977	20.07602	66.38874	52.47282	53.95217	-1.01435	0.0050508	SNORA73B
2217.057	2092.426	2143.571	1681.502	1856.488	2015.272	0.216891	0.0050531	SAMD9
348.6899	358.645	274.6764	254.1444	265.5125	193.5931	0.460578	0.0050535	THAP11
6.290797	2.972197	7.30037	0	0	0	4.842404	0.0050615	-
9.885538	15.85171	10.03801	2.074648	2.098913	1.057886	2.769523	0.0050724	C1QL4
731.5298	732.1511	750.113	845.4192	892.0379	884.3924	-0.24396	0.0050732	SESTD1
813.3102	767.8174	756.5008	699.1565	616.0309	619.921	0.272652	0.0050768	ERCC1
20.66976	17.83318	11.8631	4.149297	5.247282	2.115771	2.127985	0.0050903	AL359091.4
847.4602	898.5941	772.0141	789.4037	645.4157	467.5855	0.404027	0.0051028	KLHL21
330.7162	293.2567	371.4063	402.4818	479.6016	411.5175	-0.37706	0.0051038	HACE1
13.48028	11.88879	23.7262	29.04508	36.73097	55.01006	-1.29503	0.0051158	ZBED3-AS1
76.38825	61.4254	55.66532	33.19437	25.18695	42.31543	0.943165	0.005124	MYO7B
13.48028	6.935125	9.125462	2.074648	0	0	3.822907	0.0051253	KCP
66.50271	48.54588	64.79078	92.32185	101.7973	101.557	-0.71457	0.0051392	ZNF525
565.273	512.2085	497.3377	438.7881	449.1673	327.9446	0.373146	0.00514	FLAD1
258.8214	288.3031	246.3875	339.205	372.557	323.713	-0.38495	0.0051464	ZMYM5
808.8168	826.2706	812.1661	987.5326	956.0547	935.1709	-0.23447	0.0051496	JMJD6
17.97371	18.82391	20.98856	6.223945	3.148369	6.347314	1.87812	0.005159	AC112907.2
1.797371	8.91659	6.387824	0	0	0	4.882353	0.0051624	LINC00475
62.90797	44.58295	55.66532	31.11972	29.38478	20.09983	1.018333	0.0051676	MYL5
22.46713	45.57368	20.98856	1.037324	10.49456	13.75251	1.815794	0.0051764	MAGIX
179.7371	158.5171	201.6727	223.0247	247.6717	276.1082	-0.46656	0.0051777	ZNF248
40.44084	40.62002	70.26606	23.85846	26.23641	20.09983	1.109174	0.0051866	DNAH17
86.27379	104.0269	103.1177	53.94086	60.86847	64.53103	0.709792	0.0051982	CCDC134
268.7069	213.0074	244.5624	273.8536	356.8152	353.3338	-0.43672	0.005209	AL390728.4
494.2769	519.1437	508.2882	642.1036	618.1298	591.3581	-0.28344	0.0052103	L3MBTL2
52.12375	45.57368	45.62731	20.74648	27.28586	21.15771	1.051294	0.0052167	AC068888.1
1101.788	1222.564	1232.85	1339.185	1371.639	1442.956	-0.22392	0.0052196	ACADM

7.189482	6.935125	2.737639	29.04508	17.84076	14.8104	-1.87517	0.0052211	NOX1
6.290797	8.91659	1.825092	0	0	0	4.87719	0.0052293	CCL5
252.5306	262.544	209.8856	189.8303	162.6657	172.4354	0.464983	0.0052381	POLR2I
70.99614	64.39759	81.21661	50.82888	34.63206	32.79446	0.87327	0.0052456	-
9.885538	16.84245	5.475277	0	2.098913	1.057886	3.350933	0.0052644	CIDEC
157.2699	138.7025	36.50185	49.79156	59.81901	45.48908	1.099438	0.0052731	-
3775.377	3805.402	3724.101	4513.397	4356.293	3930.045	-0.17925	0.0052811	PSMD3
9.885538	1.981464	6.387824	20.74648	20.98913	23.27348	-1.81905	0.0052862	FRG1EP
509.5546	464.6534	532.927	581.9388	607.6352	672.8153	-0.30455	0.0052906	KIAA0232
15.27765	2.972197	11.8631	0	1.049456	1.057886	3.846762	0.0052945	DHRS7C
75.48956	81.24004	70.26606	114.1057	124.8853	112.1359	-0.63028	0.0053029	-
8.088168	20.80538	10.03801	4.149297	1.049456	0	2.894195	0.0053195	PAOX
421.4834	399.2651	434.372	530.0726	528.926	494.0326	-0.30686	0.0053375	NUPL2
867.2313	1028.38	928.9721	859.9417	731.4711	725.7096	0.284977	0.0053499	POLR2G
2142.466	2169.703	2292.316	2289.374	2659.322	2700.782	-0.2118	0.0053727	CHD8
3790.655	3636.978	3768.816	4302.821	4112.819	4070.744	-0.15722	0.0053775	ZFAND5
257.024	258.5811	245.4749	155.5986	207.7924	192.5352	0.453312	0.0053823	PIK3R3
7.189482	12.87952	13.68819	28.00775	36.73097	25.38926	-1.42012	0.0054064	AP000766.1
103.3488	95.11029	110.4181	58.09015	68.21466	66.6468	0.679737	0.0054173	KRCC1
416.99	457.7183	389.6572	649.3649	537.3216	455.9487	-0.37842	0.0054177	MAD2L1BP
53.02243	38.63855	41.06458	28.00775	7.346194	19.04194	1.286931	0.0054271	MMP13
140.1949	155.545	120.4561	214.7261	199.3967	177.7248	-0.50945	0.0054454	C12orf73
1055.057	1047.204	1044.865	864.091	873.1477	944.6919	0.230876	0.0054518	MAGEF1
212.9884	219.9425	248.2126	293.5627	269.7103	355.4496	-0.43131	0.0054526	TMEM199
2777.836	2763.152	2696.574	2265.516	1576.283	1277.926	0.68611	0.0054563	TNFRSF12A
50.32638	51.51807	34.67676	20.74648	25.18695	16.92617	1.11775	0.0054773	LAMA1
7.189482	3.962929	11.8631	1.037324	0	0	4.355316	0.0054775	C4A
753.0983	712.3364	719.0864	539.4086	642.2673	614.6316	0.28263	0.0054787	BIRC3
430.4702	405.2095	472.6989	526.9607	556.2119	538.4638	-0.30899	0.0054895	EIF2B4
121.3225	133.7488	117.7185	103.7324	56.67064	67.70468	0.707414	0.0054909	STMN3
9.885538	13.87025	7.30037	0	2.098913	1.057886	3.299925	0.0054915	AC021594.1
3907.484	3865.837	3828.131	3233.339	3499.937	3588.348	0.1687	0.005503	ABCC2
8.088168	8.91659	11.8631	2.074648	0	0	3.787767	0.0055057	-
2.696056	3.962929	10.03801	0	0	0	4.852354	0.0055128	IFITM10
567.0704	592.4578	599.5429	498.9529	484.8488	454.8908	0.289804	0.0055156	HEBP2
1038.88	1015.5	970.0366	733.3882	918.2743	855.8295	0.270541	0.0055327	DIP2A
18.87239	17.83318	28.28893	42.53029	38.82988	59.2416	-1.11082	0.0055361	NEB
366.6636	152.5728	674.3717	135.8895	122.7864	128.0042	1.626337	0.0055413	TMEM14C
11.68291	8.91659	12.77565	34.2317	29.38478	24.33137	-1.39489	0.0055424	FAM60CP
5.392112	7.925857	9.125462	0	0	1.057886	4.315568	0.0055475	BX537318.2
1263.552	1289.933	1234.675	1435.657	1540.602	1402.756	-0.20927	0.0055519	SNX9
21.56845	14.86098	13.68819	3.111972	6.296738	2.115771	2.121432	0.005569	NID1
23.36582	20.80538	24.63875	5.186621	9.445107	7.4052	1.644251	0.0055702	LCMT1-AS1
104.2475	105.0176	110.4181	60.1648	76.61031	64.53103	0.667496	0.0055714	ITGB2
8.986853	4.953661	3.650185	25.9331	18.89021	16.92617	-1.80763	0.0055756	TMEM121B
112.3357	111.9527	113.1557	63.27677	66.11575	84.63085	0.657373	0.005582	RETREG1
7953.365	8294.41	7945.54	9848.355	8808.087	8570.99	-0.17053	0.0056018	ITGA3
227.3674	259.5718	213.5358	176.3451	188.9021	121.6569	0.523589	0.0056146	KLHL26
7.189482	14.86098	20.07602	4.149297	1.049456	2.115771	2.522514	0.0056219	SHANK2
27.85924	30.7127	21.90111	3.111972	12.59348	10.57886	1.615188	0.0056265	EMILIN1
9.885538	3.962929	2.737639	0	0	0	4.843943	0.0056297	AC092809.2
354.082	357.6543	320.3037	247.9205	283.3532	264.4714	0.374926	0.0056341	SNX29
7.189482	3.962929	9.125462	15.55986	23.08804	28.56291	-1.7211	0.0056449	SERBP1P6
8.088168	9.907322	4.562731	0	0	1.057886	4.32176	0.0056456	KCNK6

0	0	0	2.074648	4.197825	9.520971	-4.9379	0.0056856	CTHRC1
5.392112	5.944393	4.562731	0	0	0	4.78003	0.0056863	FCGR3B
17.07502	19.81464	27.37639	6.223945	2.098913	9.520971	1.849823	0.0056897	ARHGAP30
511.3519	539.949	523.8015	480.2811	393.5461	370.26	0.339843	0.0056898	PLEKHG3
30.5553	21.79611	20.07602	6.223945	5.247282	11.63674	1.650575	0.0057179	AL513318.2
18.87239	13.87025	10.03801	2.074648	4.197825	2.115771	2.351407	0.0057365	RAPGEF4
12.58159	11.88879	6.387824	2.074648	1.049456	0	3.297058	0.0057384	TNFRSF19
12.58159	13.87025	10.95055	3.111972	2.098913	1.057886	2.573302	0.0057406	NTS
12.58159	2.972197	1.825092	0	0	0	4.91238	0.0057474	AL359715.2
47.63032	40.62002	52.92768	20.74648	26.23641	21.15771	1.052217	0.0057557	DIABLO
95.26064	102.0454	104.0303	74.68734	60.86847	47.60486	0.717371	0.005763	ZNF362
178.8384	160.4986	149.6576	197.0916	241.375	240.14	-0.47219	0.0057638	NAPB
8.088168	10.89805	14.60074	38.38099	22.03858	29.6208	-1.42338	0.0057937	PDCL3P5
18.87239	11.88879	18.25092	3.111972	3.148369	5.289428	2.088828	0.005794	AC118344.2
4.493426	7.925857	3.650185	0	0	0	4.793249	0.0058057	MIR4258
9.885538	5.944393	6.387824	0	0	1.057886	4.303256	0.0058072	KRT16P2
17.97371	18.82391	11.8631	1.037324	6.296738	3.173657	2.21219	0.0058115	LINC01629
1080.22	1160.147	1157.109	1249.976	1338.057	1362.557	-0.21777	0.0058207	F2R
46.73163	33.68489	46.53986	23.85846	19.93967	12.69463	1.168891	0.005825	RNF152
785.4509	815.3726	877.8695	1002.055	964.4504	960.5602	-0.23988	0.005837	IDH3B
8.986853	4.953661	7.30037	17.63451	24.1375	25.38926	-1.6544	0.0058466	NPM1P39
14.37896	15.85171	25.55129	4.149297	4.197825	6.347314	1.926303	0.0058468	CCDC151
47.63032	45.57368	44.71476	21.78381	22.03858	23.27348	1.039782	0.0058554	LRRC27
6.290797	8.91659	6.387824	30.0824	25.18695	13.75251	-1.68136	0.0058576	P2RX5-TAX1BP3
383.7386	390.3485	351.3303	506.2142	452.3157	451.7172	-0.32597	0.0058708	ERICH1
22.46713	16.84245	17.33838	6.223945	4.197825	5.289428	1.850846	0.0058732	WDR97
596.727	597.4115	635.1322	668.0367	781.845	770.1408	-0.27904	0.0058747	CCNYL1
778.2615	772.7711	677.1093	592.3121	654.8608	583.9529	0.282892	0.0058755	ZFP64
19.77108	20.80538	14.60074	3.111972	2.098913	8.463085	2.014288	0.0059092	RGL1
26.06187	34.67563	34.67676	6.223945	17.84076	11.63674	1.41836	0.0059171	LINC01910
74.59088	85.20297	179.7716	1.037324	30.43423	5.289428	3.207744	0.0059179	-
10.78422	9.907322	16.42583	1.037324	4.197825	0	2.825933	0.0059237	AC026801.2
1357.015	1387.025	1440.91	1546.65	1657.092	1599.523	-0.19877	0.0059412	ATP6V1A
603.9165	688.5589	616.8812	796.6649	724.1249	782.8354	-0.2716	0.0059436	TMEM185B
37.74478	40.62002	13.68819	12.44789	10.49456	8.463085	1.548985	0.0059537	TRIM7
523.9335	531.0324	528.3643	445.0121	423.9804	422.0964	0.294232	0.0059586	FBXL20
12.58159	12.87952	13.68819	2.074648	3.148369	2.115771	2.415613	0.0059683	FILIP1
273.2003	266.507	295.665	220.95	201.4956	209.4614	0.402977	0.006005	LSM6
168.9528	168.4245	184.3343	137.9641	116.4897	108.9622	0.521499	0.0060146	ZNF668
9.885538	6.935125	12.77565	1.037324	1.049456	1.057886	3.236549	0.0060155	U62317.1
50.32638	52.50881	62.96569	94.3965	85.00596	93.09394	-0.71638	0.0060359	LINC00630
2.696056	7.925857	5.475277	0	0	0	4.795473	0.0060378	FLT4
2.696056	7.925857	5.475277	0	0	0	4.795473	0.0060378	CASTOR1
311.8438	305.1455	319.3912	365.1381	436.5738	392.4756	-0.35055	0.0060466	DDX19A
117.7278	94.11956	85.77934	80.91128	39.87934	47.60486	0.821361	0.0060513	RASSF5
5.392112	3.962929	6.387824	0	0	0	4.767852	0.0060514	AC103740.1
41.33952	37.64782	31.93912	18.67183	13.64293	15.86829	1.202639	0.0060514	AC099518.1
18.87239	25.75904	19.16347	77.79931	44.07717	26.44714	-1.22068	0.006061	NACA3P
6.290797	3.962929	5.475277	0	0	0	4.766752	0.0060682	PSAPL1
44.93426	59.44393	55.66532	19.70916	32.53315	27.50503	1.004366	0.0060697	FP565260.3
16.17634	7.925857	4.562731	0	1.049456	1.057886	3.77301	0.0060765	AC109460.3
220.1779	211.026	229.9616	247.9205	330.5787	309.9605	-0.42566	0.0060953	TLR6
23.36582	6.935125	10.95055	2.074648	4.197825	0	2.718701	0.006115	SSTR5-AS1
32.35267	10.89805	7.30037	2.074648	6.296738	0	2.595439	0.0061169	SP5

305.553	324.9602	335.817	383.8099	389.3483	463.3539	-0.35568	0.006124	BLOC1S5
6329.44	6522.981	6404.249	7517.488	7025.061	6845.578	-0.15153	0.0061253	MYC
24.2645	10.89805	28.28893	6.223945	7.346194	4.231543	1.83651	0.006143	RASSF6
1522.373	1641.643	1519.389	1675.278	1795.62	1984.594	-0.22034	0.0061434	ODF2
443.0518	406.2002	442.5849	325.7198	369.4086	329.0024	0.335573	0.0061463	SLC45A4
141.0936	174.3689	187.072	224.062	221.4353	249.661	-0.46873	0.0061517	LRRC37BP1
25.16319	32.69416	18.25092	52.90353	40.9288	62.41525	-1.04136	0.0061591	MALINC1
71.89482	275.4235	90.34208	54.97818	39.87934	31.73657	1.788547	0.0061686	NAPRT
0	0.990732	0.912546	10.37324	4.197825	11.63674	-3.80471	0.0061802	AL138785.1
118.6265	112.9435	118.631	162.8599	175.2592	160.7986	-0.51019	0.0061867	ZNF767P
926.5445	930.2975	961.8237	811.1875	787.0923	803.9931	0.230657	0.0061921	ARL4A
133.9041	132.7581	146.9199	92.32185	83.95651	101.557	0.574365	0.0061932	LRP4
8.986853	7.925857	4.562731	21.78381	18.89021	26.44714	-1.64492	0.0061942	RPSAP52
51.22506	65.38832	57.49041	86.0979	96.54998	100.4991	-0.70332	0.0062045	ADGRL2
17.97371	10.89805	12.77565	3.111972	0	4.231543	2.506082	0.0062091	GMDS-AS1
20.66976	22.78684	30.11403	9.335917	7.346194	8.463085	1.548917	0.00621	AL031123.3
183.3318	189.2298	181.5967	269.7043	255.0179	222.156	-0.43107	0.0062197	MRPL46
1819.838	1830.873	1925.473	2105.768	2043.292	2181.36	-0.18293	0.006223	PPP5C
7.189482	3.962929	4.562731	0	0	0	4.765636	0.0062234	AL592295.4
258.8214	187.2484	221.7487	155.5986	158.4679	163.9723	0.483871	0.0062272	FKBP11
89.86853	61.4254	76.65388	49.79156	38.82988	40.19966	0.824796	0.0062597	BACH2
643.4587	607.3188	605.0181	539.4086	463.8597	515.1903	0.289554	0.0062769	MALSU1
7.189482	10.89805	13.68819	0	0	3.173657	3.331906	0.0062948	AC083902.2
10.78422	12.87952	12.77565	0	1.049456	4.231543	2.792606	0.0063143	CKMT1B
882.5089	870.8536	855.9684	655.5889	724.1249	794.4721	0.263403	0.0063264	TANC1
8.088168	3.962929	10.03801	0	0	1.057886	4.296153	0.0063267	PACERR
155.4726	156.5357	147.8325	202.2782	201.4956	227.4454	-0.45709	0.0063337	KLHL4
111.437	68.36052	51.10259	153.524	99.69835	143.8725	-0.78076	0.0063358	AC083899.1
1167.392	1316.683	1176.272	1640.009	1404.173	1322.357	-0.25505	0.0063358	MAPKAPK2
2.696056	9.907322	3.650185	0	0	0	4.807678	0.0063385	NXPH3
133.9041	112.9435	140.5321	98.54579	83.95651	67.70468	0.631158	0.0063455	HNRNPA1P7
9.885538	3.962929	8.212916	0	1.049456	0	4.294603	0.0063509	AP003064.2
73.69219	76.28638	74.82879	45.64226	36.73097	47.60486	0.790259	0.0063574	AC010761.1
447.5453	456.7275	389.6572	392.1085	322.1831	248.6031	0.425663	0.0063599	CENPM
4617.445	4345.351	4373.834	4942.85	4851.637	5000.626	-0.14961	0.0063834	ITM2B
536.5151	500.3197	486.3871	455.3853	379.9032	378.7231	0.327299	0.0063923	TONSL
426.8755	386.3855	385.0945	520.7367	476.4532	489.8011	-0.31097	0.0064076	NLK
17.07502	20.80538	14.60074	6.223945	4.197825	3.173657	1.945548	0.0064126	ABCA4
9.885538	9.907322	7.30037	1.037324	1.049456	0	3.693157	0.0064202	-
67.4014	79.25857	73.0037	116.1803	108.094	114.2517	-0.62524	0.006423	TMEM183B
753.9969	760.8823	701.748	692.9325	583.4977	467.5855	0.345559	0.0064248	RCE1
6.290797	11.88879	13.68819	0	3.148369	0	3.339476	0.0064316	VASN
23.36582	15.85171	20.07602	8.298593	3.148369	5.289428	1.825211	0.0064326	TMEM52
54.8198	49.53661	50.19004	74.68734	76.61031	112.1359	-0.76838	0.0064393	AC007485.2
3.594741	3.962929	8.212916	0	0	0	4.770019	0.0064503	Z98884.1
16.17634	5.944393	11.8631	1.037324	3.148369	0	3.023107	0.0064615	JPH3
315.4385	238.7665	342.2048	214.7261	241.375	197.8246	0.456328	0.0064675	C17orf58
20.66976	9.907322	21.90111	47.71691	32.53315	41.25754	-1.2051	0.0065195	AC025678.3
6.290797	7.925857	7.30037	1.037324	0	0	4.254632	0.0065217	SYT11
19.77108	16.84245	15.51329	6.223945	4.197825	3.173657	1.937686	0.0065259	RPL24P2
76.38825	82.23077	55.66532	40.45564	36.73097	42.31543	0.841381	0.0065326	PTCD1
40.44084	63.40686	54.75277	23.85846	33.5826	20.09983	1.030358	0.0065414	AC005256.1
222.8739	202.1094	209.8856	262.443	290.6994	283.5134	-0.39747	0.0065428	ABHD18
8.986853	8.91659	10.95055	1.037324	1.049456	1.057886	3.198286	0.0065477	PPDPFL

249.8345	251.646	290.1897	294.6001	369.4086	381.8967	-0.40122	0.006549	MINDY3
5.392112	8.91659	7.30037	0	0	1.057886	4.259827	0.0065544	HM13-AS1
113.2343	108.9805	110.4181	140.0388	163.7152	177.7248	-0.53302	0.0065565	C9orf84
63.80665	83.2215	65.70333	45.64226	40.9288	31.73657	0.844314	0.0065585	PKN3
375.6504	408.1817	350.4177	486.505	465.9586	461.2382	-0.31859	0.006563	DHDDS
3238.862	3586.45	3248.665	3793.494	3687.79	3855.993	-0.17073	0.006566	COX6C
96.15932	89.1659	76.65388	130.7028	136.4293	125.8884	-0.58522	0.0065921	FRG1BP
8.986853	6.935125	16.42583	1.037324	2.098913	1.057886	2.949727	0.0065982	BRWD1-AS2
25.16319	32.69416	35.5893	13.48521	8.395651	14.8104	1.34813	0.0066085	SDAD1P1
798.0325	833.2058	722.7366	1045.623	931.9172	863.2347	-0.27171	0.006613	EIF2D
740.5167	808.4375	801.2156	642.1036	656.9597	672.8153	0.252919	0.0066169	JARID2
12.58159	8.91659	10.03801	1.037324	2.098913	1.057886	2.912226	0.0066241	PDE6G
1960.033	1603.995	1950.111	1868.221	2364.425	2469.105	-0.28096	0.0066421	SREK1
837.5747	946.1492	976.4245	1026.951	1161.748	1090.68	-0.24892	0.0066587	PRR14L
895.9892	933.2697	943.5728	1063.257	1093.534	1068.465	-0.21818	0.0066652	DSN1
1896.226	1882.391	1811.404	2050.79	2137.743	2130.582	-0.17688	0.0066671	UBE2A
20.66976	31.70343	27.37639	6.223945	11.54402	10.57886	1.491497	0.0066706	COL4A4
297.4648	351.7099	342.2048	270.7416	231.9299	256.0083	0.385181	0.0066843	HIRIP3
44.93426	70.34198	38.32694	32.15705	11.54402	24.33137	1.171872	0.0066853	-
163.5607	165.4523	164.2583	207.4648	220.3858	242.2558	-0.44191	0.0066886	TMTC4
26.06187	30.7127	24.63875	9.335917	9.445107	11.63674	1.419628	0.0066902	LINC01876
207.5963	213.9982	227.224	307.0479	307.4907	248.6031	-0.41195	0.0067021	WHAMM
408.0031	460.6905	444.41	376.5487	340.0239	323.713	0.335285	0.0067157	MAP3K9
26.06187	16.84245	15.51329	5.186621	5.247282	6.347314	1.801512	0.0067162	AL139089.1
66.50271	44.58295	52.01513	24.89578	36.73097	17.98406	1.036027	0.0067228	SLFNL1-AS1
1123.357	1070.981	1131.557	1006.204	879.4444	947.8656	0.231317	0.0067234	POLR2H
464.6203	448.8017	500.0753	561.1924	637.02	544.8111	-0.30194	0.0067305	TMEM167B
6.290797	7.925857	1.825092	0	0	0	4.79122	0.0067328	CDH15
210.2924	250.6552	227.224	330.9064	296.9961	279.2818	-0.39991	0.0067461	RAB28
532.9204	491.4032	467.2237	405.5937	342.1228	432.6752	0.337763	0.0067601	HERC2P2
12.58159	7.925857	9.125462	1.037324	2.098913	0	3.239144	0.0067674	PTGDS
4613.85	4624.738	4421.286	4421.075	3884.038	3567.19	0.202249	0.0067712	AL080243.2
192.3186	211.026	185.2469	178.4198	116.4897	89.92028	0.611909	0.0067744	CRK
1428.011	1433.589	1430.872	1349.559	1213.172	1061.059	0.244136	0.0067828	PLBD2
266.9095	268.4884	279.2391	164.9345	185.7538	243.3137	0.456196	0.0067899	PAG1
17.97371	11.88879	10.03801	1.037324	2.098913	4.231543	2.441631	0.0068048	LINC01433
925.6458	907.5107	825.8543	1029.026	1004.33	1101.259	-0.23747	0.006811	RASSF3
6.290797	10.89805	4.562731	1.037324	0	0	4.267896	0.0068127	DLK1
39.54215	13.87025	62.05314	16.59719	15.74185	8.463085	1.5031	0.0068274	LONRF1
244.4424	280.3772	250.0377	223.0247	182.6054	158.6829	0.456305	0.0068396	TAF6L
4.493426	2.972197	58.40296	3.111972	4.197825	3.173657	2.653744	0.0068452	-
35.04873	34.67563	26.46384	19.70916	7.346194	9.520971	1.39281	0.0068473	-
10.78422	12.87952	12.77565	2.074648	1.049456	3.173657	2.5332	0.0068551	SELENOP
91.6659	69.35125	99.46754	121.3669	130.1326	147.0461	-0.61098	0.0068622	AC092747.4
155.4726	145.6376	140.5321	119.2923	96.54998	78.28354	0.586023	0.0069032	TSPAN12
8.986853	23.77757	16.42583	3.111972	1.049456	6.347314	2.225318	0.0069266	NXF3
88.96984	82.23077	74.82879	136.9268	109.1435	128.0042	-0.60445	0.0069429	SERAC1
291.174	290.2845	274.6764	216.8007	221.4353	217.9244	0.383673	0.0069574	CRNDE
583.2467	511.2178	548.4403	443.9747	458.6124	436.9068	0.295189	0.0069627	PTPRM
0.898685	0.990732	0.912546	5.186621	8.395651	13.75251	-3.28719	0.0069668	TNFSF15
30.5553	32.69416	30.11403	8.298593	17.84076	10.57886	1.346464	0.0069876	CCDC144B
198.6094	179.3225	185.2469	232.3606	283.3532	242.2558	-0.42789	0.0070235	FAM234B
658.7363	632.0871	682.5846	873.4269	776.5977	729.9411	-0.27011	0.007036	DHX32
337.007	360.6265	325.779	424.2656	419.7825	433.7331	-0.32086	0.0070538	FCHSD2

107.8422	95.11029	122.2812	77.79931	55.62119	70.87834	0.671603	0.0070553	PNPLA3
35.94741	41.61075	47.4524	20.74648	17.84076	20.09983	1.090637	0.0070563	MTFP1
91.6659	81.24004	110.4181	56.0155	65.06629	51.8364	0.713429	0.0070744	ZNF256
392.7255	507.2549	430.7218	499.9902	602.3879	580.7792	-0.34005	0.0070762	USF3
158.1686	158.5171	132.3192	191.905	219.3364	209.4614	-0.46761	0.0070816	FAM117B
27.85924	29.72197	32.85166	5.186621	8.395651	19.04194	1.473166	0.0070861	AC093495.1
1893.53	1945.798	1998.476	1827.765	1648.696	1614.334	0.197414	0.0071073	POLDIP3
1661.669	1817.994	1754.826	1940.833	1983.472	2024.793	-0.18491	0.0071175	SART1
42.23821	37.64782	40.15203	69.50072	66.11575	72.99411	-0.79632	0.0071313	ALMS1-IT1
168.9528	176.3503	179.7716	134.8521	126.9842	108.9622	0.501454	0.007149	ST3GAL5
10.78422	20.80538	8.212916	2.074648	3.148369	2.115771	2.435297	0.0071692	SLC45A1
221.0766	225.8869	202.5853	177.3824	98.6489	165.0302	0.558079	0.0071761	SQOR
67.4014	87.18443	75.74134	52.90353	46.17608	30.67868	0.825846	0.0071841	BTF3L4P2
97.05801	103.0361	101.2926	67.42607	67.16521	56.06794	0.660046	0.0072036	MIR600HG
17.07502	7.925857	5.475277	0	2.098913	1.057886	3.276854	0.007227	TSPAN19
1170.088	1326.59	1097.793	1495.821	1390.53	1348.804	-0.23732	0.0072316	HAX1
23.36582	16.84245	26.46384	10.37324	4.197825	6.347314	1.672799	0.0072338	SHC3
171.6489	182.2947	140.5321	116.1803	115.4402	111.078	0.528043	0.0072348	HOXB9
327.1214	235.7943	579.4668	696.0445	752.4602	969.0233	-1.08117	0.0072375	FLII
84.47642	84.21224	97.64245	40.45564	60.86847	58.18371	0.740514	0.0072631	C5orf17
79.98299	76.28638	93.07971	51.86621	46.17608	51.8364	0.735034	0.007271	SVBP
154.5739	169.4152	128.669	117.2176	100.7478	84.63085	0.579653	0.0072723	IQCJ-SCHIP1
159.0673	120.8693	167.9085	103.7324	103.8962	94.15183	0.571026	0.0072804	CLCN2
0.898685	0	0	8.298593	7.346194	4.231543	-4.30923	0.0072837	AP000223.1
16.17634	6.935125	12.77565	14.52254	12.59348	100.4991	-1.82537	0.0072841	-
77.28693	68.36052	93.99226	103.7324	120.6875	151.2777	-0.64686	0.007289	LRRC37A16P
16.17634	14.86098	11.8631	3.111972	4.197825	2.115771	2.185565	0.0072919	HM13-IT1
311.8438	296.2289	324.8665	365.1381	383.0516	441.1383	-0.34964	0.0072924	TM2D1
3.594741	0	1.825092	13.48521	14.69239	7.4052	-2.69007	0.0073226	NPM1P24
25.16319	11.88879	18.25092	1.037324	5.247282	7.4052	2.02002	0.007328	DM1-AS
30.5553	25.75904	30.11403	19.70916	2.098913	3.173657	1.78776	0.0073331	ZNF775
1186.265	1280.026	1251.101	1298.73	1493.376	1576.25	-0.2329	0.0073531	MIA3
434.065	437.9036	441.6724	397.2951	315.8864	321.5972	0.343963	0.0073603	ADPRHL2
867.2313	930.2975	907.9835	1037.324	1067.297	1044.133	-0.2192	0.0073612	AGAP1
247.1385	224.8962	231.7867	191.905	182.6054	130.1199	0.479925	0.0073683	RPL17
31.45398	25.75904	32.85166	50.82888	59.81901	58.18371	-0.90428	0.0073915	DDX47
1326.459	1209.684	1273.915	1419.059	1578.382	1420.74	-0.21327	0.0073935	ANKIB1
8.986853	11.88879	10.03801	2.074648	1.049456	1.057886	2.882349	0.007398	CSDC2
8.088168	10.89805	10.03801	2.074648	1.049456	0	3.20838	0.0073993	SLC7A4
2471.385	2464.942	2435.586	2017.595	2239.54	2248.007	0.180525	0.0074117	SUGP2
318.1346	484.468	347.6801	496.8783	518.4314	492.9747	-0.39275	0.0074138	PDXDC1
28.75793	32.69416	22.81366	57.05283	47.22554	57.12583	-0.93989	0.0074274	AL355987.4
1378.583	1368.201	1387.983	1493.747	1560.542	1715.891	-0.20609	0.0074374	SLC16A6
38.64347	40.62002	28.28893	60.1648	53.52227	85.68874	-0.89155	0.0074483	HORMAD1
79.0843	73.31418	70.26606	57.05283	36.73097	29.6208	0.850618	0.0074646	CHAC1
30.5553	27.7405	21.90111	13.48521	8.395651	7.4052	1.451916	0.007468	PAQR8
86.27379	70.34198	105.8554	132.7775	122.7864	144.9303	-0.60742	0.00749	ZNF615
9.885538	4.953661	11.8631	0	1.049456	1.057886	3.671898	0.0074911	AC007686.1
79.98299	48.54588	56.57787	20.74648	34.63206	39.14177	0.972435	0.0074996	FAM229A
181.5344	202.1094	204.4104	241.6965	267.6114	270.8187	-0.40812	0.007505	TMPRSS15
4.493426	5.944393	4.562731	0	0	0	4.695585	0.0075214	TSPOAP1-AS1
8.088168	1.981464	5.475277	0	0	0	4.751963	0.0075324	AARD
173.4463	155.545	180.6842	187.7557	278.1059	247.5452	-0.48409	0.0075389	ZNF550
7.189482	12.87952	9.125462	2.074648	0	1.057886	3.214211	0.007555	HPD

271.403	388.367	278.3266	206.4275	262.3641	210.5192	0.46419	0.0075563	-
360.3728	361.6172	311.1783	281.1148	238.2266	277.166	0.374941	0.0075576	WASHC3
124.0186	98.08249	151.4827	174.2705	174.2098	194.651	-0.53767	0.0075625	SMIM8
88.07116	123.8415	94.90481	59.12748	58.76956	71.93623	0.690995	0.0075646	ZNF554
198.6094	216.9703	229.9616	158.7106	143.7755	169.2617	0.45237	0.0075784	SCAPER
87.17247	94.11956	75.74134	51.86621	57.7201	45.48908	0.727969	0.0075911	RFX3
331.6149	330.9045	340.3797	433.6015	387.2494	434.791	-0.32417	0.0075977	SDHAP1
541.9072	533.0139	486.3871	610.9839	652.7618	624.1525	-0.27415	0.0076072	SMAP2
8.986853	7.925857	9.125462	1.037324	1.049456	0	3.637041	0.0076217	CEACAMP10
1254.565	1318.665	1386.158	1392.089	1652.894	1585.771	-0.22593	0.0076255	CDC42BPA
177.041	189.2298	175.2089	145.2254	116.4897	122.7147	0.49344	0.0076269	SLC29A3
14.37896	24.7683	24.63875	11.41057	1.049456	4.231543	1.92999	0.0076313	AL121944.1
14.37896	10.89805	8.212916	2.074648	1.049456	2.115771	2.676515	0.0076519	ZFHX2
35916.86	38714.84	34686.79	36301.16	30024.95	27860.48	0.21492	0.0076575	RPS2
2173.021	2259.86	2159.997	2095.395	1805.065	1843.895	0.198542	0.0076657	NDUFB11
221.0766	252.6367	233.6118	308.0853	307.4907	299.3816	-0.37228	0.0076718	ZNF511
5.392112	5.944393	3.650185	0	0	0	4.694437	0.0076736	LEXM
43.13689	32.69416	62.96569	19.70916	19.93967	25.38926	1.096133	0.0076776	AP000759.1
97.9567	91.14736	94.90481	159.7479	116.4897	145.9882	-0.57172	0.007678	EPM2A
736.9219	696.4847	631.482	1034.212	856.3564	709.8413	-0.33286	0.0076855	SYNGR2
4080.031	3974.817	4120.146	4192.864	4641.745	4957.252	-0.17978	0.00771	LMO7
253.4292	285.3309	271.9388	196.0543	195.1989	222.156	0.401888	0.0077164	JCAD
13.48028	4.953661	9.125462	0	2.098913	0	3.719207	0.0077422	AC136475.1
31.45398	22.78684	30.11403	10.37324	6.296738	14.8104	1.424322	0.0077576	LINC01133
69.19877	46.56441	62.96569	34.2317	24.1375	35.96811	0.923877	0.0077726	DOCK3
8848.455	9202.911	8672.839	10642.95	10023.36	9236.4	-0.16221	0.0077797	COTL1
2113.708	2209.333	2236.651	2345.39	2453.629	2654.235	-0.18425	0.0077958	OSBP
72.79351	47.55514	42.88967	25.9331	16.7913	35.96811	1.054419	0.0078169	AC122688.3
394.5228	411.1539	350.4177	344.3916	278.1059	268.703	0.374698	0.0078592	RELT
85.3751	92.13809	91.25462	70.53804	28.33532	51.8364	0.833769	0.0078636	ICAM1
26.06187	26.74977	27.37639	13.48521	10.49456	5.289428	1.452298	0.0078736	AL121832.3
2.696056	6.935125	5.475277	0	0	0	4.704377	0.0079035	SMPDL3B
11.68291	7.925857	9.125462	1.037324	0	2.115771	3.19198	0.0079102	CEP295NL
10.78422	12.87952	10.03801	0	4.197825	0	3.005283	0.0079211	ERVH48-1
1345.332	1234.452	1417.184	1384.828	1649.745	1696.849	-0.24291	0.0079235	AHR
7.189482	8.91659	12.77565	2.074648	1.049456	0	3.20256	0.0079338	CCR3
289.3767	364.5894	368.6687	263.4803	256.0673	264.4714	0.38259	0.0079401	DIP2C
17.07502	10.89805	20.98856	39.41832	29.38478	43.37331	-1.19115	0.0079686	HOXA2
440.3558	363.5987	391.4823	532.1473	511.0852	454.8908	-0.32485	0.0079747	EID2
112.3357	78.26784	111.3306	125.5162	156.369	174.5511	-0.59346	0.0079953	ZNF674-AS1
60.21191	43.59222	37.41439	26.97043	17.84076	23.27348	1.05316	0.008018	FYB1
8.986853	13.87025	9.125462	1.037324	3.148369	0	2.930145	0.0080197	DCST2
8.088168	12.87952	10.95055	1.037324	3.148369	0	2.927718	0.0080371	MLXIPL
3093.275	3006.872	3082.581	3220.891	3445.365	3693.079	-0.1738	0.0080467	ELOVL5
416.99	429.9778	477.2617	523.8487	546.7668	551.1584	-0.29221	0.0080475	DOCK9
445.7479	487.4402	548.4403	649.3649	564.6075	614.6316	-0.30366	0.0080596	KLHL12
3.594741	1.981464	10.03801	0	0	0	4.757464	0.0080629	AC025857.2
337.9057	302.1733	390.5698	373.4367	498.4918	471.817	-0.38169	0.0080915	KANTR
41.33952	28.73123	33.76421	77.79931	50.3739	63.47314	-0.88185	0.0080946	POLR2J4
17.07502	14.86098	14.60074	7.261269	2.098913	0	2.308617	0.0080961	AC244090.1
23.36582	10.89805	22.81366	36.30634	48.27499	41.25754	-1.13413	0.0080991	ZNF547
111.437	112.9435	94.90481	168.0465	152.1712	140.6988	-0.53048	0.0081035	RP9P
31.45398	23.77757	30.11403	4.149297	16.7913	9.520971	1.488776	0.0081043	AC092053.3
28.75793	21.79611	38.32694	14.52254	9.445107	10.57886	1.364619	0.0081131	AC012615.6

56.61717	62.41613	62.05314	30.0824	38.82988	30.67868	0.862269	0.0081445	LINC00958
53.92112	43.59222	51.10259	30.0824	25.18695	20.09983	0.980004	0.0081504	AC244669.1
2619.668	2839.438	2741.289	3204.294	3024.533	2981.122	-0.16776	0.0081989	C1orf43
8.088168	5.944393	6.387824	0	0	1.057886	4.180975	0.0082061	GPIHBP1
366.6636	341.8026	396.0451	410.7804	476.4532	505.6694	-0.33387	0.0082086	SNHG14
554.4888	517.1622	455.3606	485.4677	355.7657	316.3078	0.399304	0.0082493	FZD2
8458.426	8984.95	8869.037	10151.25	9172.248	9849.974	-0.14898	0.0082515	EEF1A1P5
4.493426	7.925857	10.03801	19.70916	30.43423	17.98406	-1.60322	0.0082592	LINC01094
18.87239	36.65709	30.11403	16.59719	7.346194	6.347314	1.495199	0.0082713	PINX1
21.56845	4.953661	10.95055	1.037324	4.197825	0	2.842496	0.0082734	GAL3ST4
16.17634	7.925857	8.212916	1.037324	0	3.173657	2.945994	0.0082784	MRPL23-AS1
18.87239	15.85171	10.03801	3.111972	3.148369	4.231543	2.09364	0.0082971	MYH7B
1181.771	1268.137	1250.188	1453.291	1415.717	1374.193	-0.1979	0.0083097	WDR33
31.45398	23.77757	30.11403	61.20212	45.12662	56.06794	-0.92577	0.0083113	WNT7A
16.17634	27.7405	16.42583	7.261269	7.346194	3.173657	1.75841	0.0083451	ETV2
68.30008	79.25857	80.30407	44.60494	52.47282	35.96811	0.775441	0.0083559	TMEM143
132.1067	106.0083	97.64245	157.6733	168.9625	157.625	-0.5274	0.0083594	SERBP1P5
10741.99	10513.65	10549.03	11429.24	11346.72	11995.37	-0.12863	0.0083785	MCL1
11.68291	12.87952	14.60074	1.037324	5.247282	1.057886	2.415684	0.0083824	Z95152.1
27.85924	30.7127	30.11403	10.37324	15.74185	9.520971	1.314931	0.0084133	VWCE
2670.893	2906.808	2579.768	2476.093	2466.222	2167.608	0.197978	0.0084211	SDF4
15.27765	23.77757	11.8631	8.298593	1.049456	2.115771	2.144738	0.0084419	GLTPD2
7.189482	4.953661	2.737639	0	0	0	4.685646	0.0084426	AC241377.3
583.2467	590.4764	568.5163	542.5205	440.7717	418.9227	0.312854	0.008457	TNIP2
7.189482	9.907322	3.650185	0	0	1.057886	4.200298	0.0084606	RHBDL2
692.8864	738.0955	701.748	634.8424	603.4374	525.7692	0.273322	0.0084701	TBL2
40.44084	31.70343	50.19004	11.41057	27.28586	14.8104	1.195061	0.0084814	AC022211.2
29.65661	35.66636	22.81366	3.111972	17.84076	9.520971	1.532219	0.0084901	AC055713.1
5.392112	2.972197	6.387824	0	0	0	4.674958	0.0084954	AC107959.4
270.5043	281.3679	232.6993	219.9127	201.4956	144.9303	0.469366	0.0085565	MAPKBP1
26.96056	41.61075	44.71476	76.76199	61.91792	64.53103	-0.84513	0.0085962	-
2.696056	9.907322	2.737639	0	0	0	4.723847	0.0085989	SNORA5C
96.15932	86.1937	119.5436	74.68734	51.42336	60.29948	0.69626	0.0086075	ZNF469
561.6783	549.8564	540.2274	475.0945	410.3374	466.5276	0.288951	0.0086101	PLEKHA7
39.54215	30.7127	30.11403	47.71691	69.26412	67.70468	-0.8779	0.0086679	AC093909.6
3494.088	3476.479	3644.71	3616.112	4185.232	4268.569	-0.18517	0.0086693	SETD5
2219.753	2159.796	2286.841	2005.148	1980.324	1947.568	0.168272	0.0086737	FAM84B
48.52901	50.52734	48.36495	24.89578	30.43423	20.09983	0.966195	0.0087104	PPP1R3F
148.2831	137.7118	126.8439	168.0465	229.8309	184.0721	-0.49506	0.0087152	ZRANB3
1457.668	1456.376	1575.055	1659.719	1706.416	1750.801	-0.18867	0.0087309	UBE2B
54.8198	81.24004	66.61587	39.41832	36.73097	38.08388	0.825094	0.0087501	AP003486.1
1103.586	1115.564	1082.28	1033.175	936.1151	747.9252	0.280706	0.0087583	HSPBP1
189.6226	175.3596	144.1823	158.7106	81.85759	78.28354	0.674443	0.0087625	-
38.64347	32.69416	24.63875	52.90353	59.81901	64.53103	-0.88463	0.0087691	ZNF695
274.099	324.9602	295.665	219.9127	264.463	174.5511	0.440476	0.0087695	PTDSS2
18.87239	30.7127	30.11403	47.71691	72.41249	40.19966	-1.01102	0.0087707	UCHL3
340.6017	363.5987	352.2428	318.4585	258.1663	234.8506	0.379948	0.0088162	TBC1D7
26.06187	34.67563	27.37639	12.44789	15.74185	5.289428	1.39356	0.0088173	ACVR2B-AS1
0	0.990732	0.912546	4.149297	10.49456	9.520971	-3.68674	0.0088311	AC135279.2
44.93426	42.60148	65.70333	73.65001	109.1435	79.34143	-0.77283	0.0088337	MTMR10
8.088168	14.86098	3.650185	1.037324	1.049456	0	3.663231	0.0088343	DPYSL4
8.986853	13.87025	10.95055	3.111972	2.098913	0	2.69111	0.0088398	-
9.885538	4.953661	5.475277	0	1.049456	0	4.174609	0.0088398	GJD3
4.493426	1.981464	2.737639	12.44789	12.59348	15.86829	-2.14172	0.0088454	LRRC69

12.58159	0.990732	2.737639	0	0	0	4.822212	0.0088503	AC233723.1
370.2583	297.2197	346.7676	187.7557	231.9299	315.2499	0.465846	0.0088585	AC021078.1
10.78422	1.981464	2.737639	0	0	0	4.748324	0.0088731	AC239803.4
27.85924	23.77757	28.28893	15.55986	8.395651	1.057886	1.673585	0.0088791	SPANXB1
16.17634	6.935125	3.650185	1.037324	1.049456	0	3.678278	0.0088868	STX16-NPEPL1
958.8972	1051.167	964.5614	1152.467	1132.363	1159.443	-0.21201	0.00889	TBC1D4
319.0333	348.7377	335.817	393.1458	421.8815	435.8489	-0.31818	0.0088922	SLC9A6
190.5213	225.8869	169.7336	156.6359	144.825	103.6728	0.53132	0.008925	GCAT
133.0054	148.6098	108.593	81.94861	91.3027	86.74663	0.584657	0.0089502	SYTL4
3742.126	3807.384	3819.006	3688.725	3177.754	3148.268	0.182827	0.0089544	POLDIP2
16.17634	15.85171	9.125462	0	0	6.347314	2.702913	0.0089553	CCR10
278.5924	251.646	251.8628	183.6064	222.4847	181.9563	0.411841	0.008969	DDX12P
26.96056	37.64782	37.41439	78.83663	48.27499	61.35737	-0.88755	0.0089731	CMTR2
9.885538	15.85171	7.30037	2.074648	1.049456	2.115771	2.65315	0.0089837	TVP23A
203.1029	205.0816	167.9085	142.1134	147.9733	122.7147	0.480189	0.0089895	RHBDF2
15.27765	13.87025	15.51329	6.223945	2.098913	2.115771	2.094568	0.0090218	TIGD5
108.7409	116.9064	79.39152	65.35142	58.76956	66.6468	0.676057	0.0090284	PXYLP1
7.189482	0.990732	7.30037	0	0	0	4.746514	0.0090335	KLHDC7A
715.3535	675.6793	663.4211	593.3494	574.0526	554.3321	0.254986	0.0090494	EPGN
25.16319	21.79611	32.85166	8.298593	10.49456	11.63674	1.393177	0.0090729	ATP2A1-AS1
117.7278	136.721	146.0074	71.57537	99.69835	95.20971	0.587638	0.009073	-
8.088168	5.944393	3.650185	15.55986	15.74185	27.50503	-1.73207	0.009111	XK
8.088168	2.972197	3.650185	0	0	0	4.671356	0.0091133	AC004637.1
6.290797	3.962929	10.03801	1.037324	0	0	4.173266	0.009119	APOBEC3H
22.46713	17.83318	63.87824	22.82113	3.148369	3.173657	1.836528	0.0091242	-
28.75793	38.63855	25.55129	4.149297	9.445107	20.09983	1.464682	0.0091302	PPP1R1C
26.06187	17.83318	30.11403	56.0155	35.68152	58.18371	-1.01438	0.0091392	SNORD100
17.97371	14.86098	13.68819	6.223945	2.098913	3.173657	2.015155	0.0091475	PLA2G4C
1789.282	1803.133	1648.058	2257.217	1902.664	1919.005	-0.21432	0.0091513	GPS1
36.8461	33.68489	40.15203	60.1648	61.91792	71.93623	-0.80838	0.009198	MCIDAS
16.17634	9.907322	13.68819	5.186621	0	2.115771	2.443514	0.009203	AC002456.1
38.64347	33.68489	39.23949	19.70916	13.64293	17.98406	1.12049	0.0092331	PET117
43.13689	36.65709	57.49041	88.17255	98.6489	56.06794	-0.82143	0.0092613	ABCA10
70.09745	72.32345	83.04171	104.7697	128.0337	110.0201	-0.60419	0.0092634	OPN1SW
216.5832	244.7108	246.3875	339.205	315.8864	269.7608	-0.38686	0.0092704	TATDN3
7.189482	16.84245	19.16347	3.111972	4.197825	2.115771	2.193816	0.0092746	ABR
354.9807	329.9138	279.2391	397.2951	406.1396	417.8648	-0.34113	0.0092798	DEDD
35.04873	28.73123	19.16347	16.59719	7.346194	5.289428	1.502397	0.0093124	TSPAN33
37.74478	33.68489	38.32694	59.12748	73.46194	60.29948	-0.81226	0.0093256	-
279.4911	239.7572	246.3875	312.2346	305.3918	379.781	-0.38062	0.0093323	MDFIC
1204.238	1287.952	1179.01	981.3086	985.4395	1156.269	0.23314	0.0093459	PAF1
2865.907	2884.021	2927.448	3613	3234.424	3023.437	-0.18599	0.0093592	POMP
37.74478	41.61075	35.5893	56.0155	62.96738	83.57297	-0.81808	0.0094007	ZNF117
27.85924	27.7405	21.90111	4.149297	8.395651	14.8104	1.504193	0.0094186	AC013444.1
12.58159	14.86098	22.81366	46.67959	46.17608	23.27348	-1.20799	0.0094301	CACYBPP2
1147.621	1060.083	1004.713	962.6368	932.9667	817.7456	0.243584	0.0094471	NPRL3
1162	1208.693	1239.238	1303.916	1462.942	1392.178	-0.20429	0.0094501	SAMD8
545.502	552.8286	488.2122	491.6916	368.3592	398.8229	0.333275	0.0094712	NDUFB1
11.68291	7.925857	10.03801	20.74648	28.33532	28.56291	-1.38495	0.0094778	MIR3685
424.1795	399.2651	434.372	339.205	329.5293	345.9286	0.31039	0.0094837	COA5
231.8608	233.8128	224.4864	196.0543	157.4185	158.6829	0.429896	0.0094928	SLC39A11
557.1849	604.3466	488.2122	479.2438	411.3869	437.9647	0.311577	0.0095019	PSMG4
214.7858	200.1279	237.262	284.2268	298.0456	268.703	-0.38308	0.0095022	CABLES1
425.9768	419.0797	395.1325	536.2966	486.9477	492.9747	-0.2901	0.0095068	NSUN4

58.41454	46.56441	52.92768	101.6578	80.80814	76.16777	-0.7105	0.0095217	SFXN2
249.8345	223.9055	227.224	127.5909	194.1494	183.0142	0.474551	0.0095258	TRIML2
35.04873	34.67563	39.23949	15.55986	17.84076	16.92617	1.114979	0.0095273	QRICH2
666.8245	684.5959	672.5466	1016.578	786.0428	711.9571	-0.31343	0.0095298	SMYD5
101.5514	84.21224	83.04171	68.46339	53.52227	39.14177	0.738212	0.0095516	LCAT
23.36582	9.907322	15.51329	33.19437	41.97825	35.96811	-1.18124	0.0095743	LINC01881
17.07502	28.73123	18.25092	50.82888	48.27499	33.85234	-1.05901	0.0095929	CHDH
32.35267	45.57368	36.50185	70.53804	55.62119	75.10988	-0.81749	0.0096001	ZNF34
150.9791	171.3967	139.6196	130.7028	99.69835	77.22565	0.585229	0.0096011	SLC25A10
940.9235	955.0658	1056.729	1103.713	1115.572	1238.784	-0.22769	0.009605	MIB1
11.68291	8.91659	13.68819	2.074648	2.098913	2.115771	2.448441	0.0096178	AL121987.2
424.1795	446.8202	412.4709	358.9142	343.1722	334.2919	0.307992	0.0096208	RIT1
38.64347	45.57368	53.84023	57.05283	100.7478	82.51508	-0.79954	0.0096228	ZNF674
338.8044	350.7192	310.2657	257.2564	277.0565	248.6031	0.352303	0.0096353	IRX2
17.07502	8.91659	9.125462	22.82113	24.1375	44.4312	-1.37476	0.0096635	AL049844.2
204.9002	175.3596	247.3	260.3684	282.3038	299.3816	-0.42259	0.009664	SLC25A4
2.696056	3.962929	1.825092	16.59719	10.49456	11.63674	-2.19931	0.0096923	AC034198.2
280.3898	280.3772	277.4141	365.1381	362.0624	330.0603	-0.33514	0.0097052	FAM220A
547.2993	508.2456	527.4517	454.348	430.2771	417.8648	0.28165	0.009706	TLN2
12.58159	19.81464	15.51329	8.298593	1.049456	1.057886	2.196362	0.0097345	AL590705.1
248.0371	238.7665	270.1137	308.0853	311.6885	352.2759	-0.36023	0.0097401	RNF214
32.35267	36.65709	57.49041	18.67183	24.1375	15.86829	1.108755	0.0098043	GREB1
6.290797	5.944393	6.387824	16.59719	20.98913	20.09983	-1.63013	0.0098067	TMED6
779.1601	885.7146	898.858	940.853	1043.16	1042.017	-0.23946	0.0098712	ASCC1
20.66976	12.87952	20.07602	5.186621	6.296738	4.231543	1.772851	0.0099299	IL9RP3
1.797371	3.962929	2.737639	16.59719	9.445107	12.69463	-2.19832	0.0099325	AL138976.2
2422.856	2593.737	2603.494	2276.926	2350.782	2114.713	0.176369	0.0099352	SLC39A14
2007.663	1902.206	1908.134	2003.073	2373.87	2299.843	-0.19844	0.0099379	DYNC1LI2
11.68291	13.87025	15.51329	4.149297	4.197825	0	2.294935	0.009966	-
29.65661	51.51807	27.37639	18.67183	10.49456	16.92617	1.232179	0.0099704	RAB43P1
226.4687	221.924	184.3343	259.331	266.5619	311.0184	-0.40369	0.0100256	HNRNPA1P10
2.696056	14.86098	3.650185	1.037324	0	0	4.228107	0.0100307	ADAMTS5
121.3225	169.4152	143.2698	201.2409	185.7538	215.8087	-0.47595	0.0100379	NEK3
79.98299	104.0269	83.04171	58.09015	56.67064	50.77851	0.688079	0.0100493	-
7.189482	2.972197	20.07602	0	1.049456	2.115771	3.265233	0.0100932	BGN
3.594741	5.944393	4.562731	0	0	0	4.605898	0.0101098	AC018641.1
8.986853	4.953661	10.95055	0	1.049456	1.057886	3.570258	0.0101201	PCBP2-OT1
15.27765	25.75904	10.95055	8.298593	3.148369	1.057886	2.048818	0.0101298	ZBTB32
136.6002	106.9991	121.3686	56.0155	72.41249	102.6149	0.661341	0.0101336	AC018638.4
4.493426	5.944393	3.650185	0	0	0	4.604667	0.0101397	NOS2
37.74478	36.65709	28.28893	78.83663	69.26412	42.31543	-0.892	0.0101674	TMEM216
662.3311	572.6432	668.8964	520.7367	536.2722	520.4798	0.272102	0.010212	ZSWIM6
2426.45	2400.544	2361.67	3079.815	2516.596	2657.409	-0.19941	0.0102254	CLTA
0.898685	4.953661	5.475277	15.55986	9.445107	22.2156	-2.06729	0.0102508	AC087071.2
128.512	101.0547	107.6805	147.3	157.4185	176.6669	-0.51176	0.0102583	ZNF83
11.68291	4.953661	8.212916	1.037324	0	1.057886	3.57056	0.0102766	PFN4
2.696056	1.981464	2.737639	12.44789	12.59348	10.57886	-2.25975	0.0103226	AC002056.1
466.4177	479.5144	461.7484	406.6311	374.6559	367.0863	0.293385	0.0103341	TRAF3IP2
5.392112	4.953661	3.650185	0	0	0	4.596565	0.0103386	KRT17P6
0	0.990732	0.912546	6.223945	3.148369	14.8104	-3.68708	0.0103604	RAB1C
0	0.990732	0	4.149297	3.148369	11.63674	-4.2379	0.0103648	AP3B2
6.290797	18.82391	2.737639	0	2.098913	0	3.725921	0.0103673	FLRT3
900.4827	1031.352	876.9569	878.6135	757.7075	671.7574	0.282556	0.0103682	RPUSD1
50.32638	54.49027	73.0037	38.38099	23.08804	33.85234	0.89966	0.0103816	AL669831.1

789.0457	860.9463	804.8658	962.6368	937.1645	957.3865	-0.21947	0.0103857	NSFL1C
128.512	143.6562	125.0188	102.6951	91.3027	74.052	0.566074	0.0103929	SYBU
1270.741	1243.369	1205.474	1193.96	1017.973	920.3605	0.247745	0.0104123	CSK
30.5553	24.7683	20.07602	2.074648	11.54402	11.63674	1.580537	0.0104151	AC008013.1
13.48028	15.85171	7.30037	0	4.197825	2.115771	2.537907	0.0104163	FP325330.3
1290.512	1289.933	1210.036	1443.955	1482.882	1399.583	-0.19094	0.0104296	TRMU
4.493426	3.962929	5.475277	0	0	0	4.590909	0.010482	MAF
75.48956	77.27711	68.44097	45.64226	43.02771	44.4312	0.732408	0.0105116	NOS1AP
21.56845	24.7683	13.68819	6.223945	8.395651	4.231543	1.668665	0.010518	TMEM38A
305.553	250.6552	288.3646	358.9142	388.2988	332.1761	-0.35279	0.0105405	TTC33
29.65661	18.82391	15.51329	9.335917	5.247282	6.347314	1.612742	0.0105892	LY6E-DT
85.3751	98.08249	59.3155	46.67959	55.62119	39.14177	0.777686	0.0105963	AOC2
619.1942	636.0501	636.9573	614.0959	419.7825	466.5276	0.334402	0.0106202	BX679664.3
221.9753	194.1835	186.1594	251.0324	303.2929	245.4295	-0.40851	0.0106328	FBXO8
243.5437	236.785	280.1517	321.5705	382.0021	289.8607	-0.38506	0.0106339	PCMTD2
16.17634	17.83318	25.55129	11.41057	2.098913	3.173657	1.833196	0.010634	LYPD5
25.16319	43.59222	39.23949	16.59719	17.84076	13.75251	1.161727	0.0106402	SLC4A11
1347.129	1414.766	1374.295	1464.702	1613.014	1658.765	-0.19561	0.0106641	RPL7P9
7.189482	9.907322	10.03801	1.037324	0	2.115771	3.107031	0.0106646	SLC30A3
178.8384	212.0167	242.7373	142.1134	137.4788	175.609	0.477069	0.0106712	MAML3
312.7425	337.8397	296.5775	454.348	350.5184	399.8808	-0.34782	0.0106966	APOO
7.189482	3.962929	8.212916	0	1.049456	0	4.105934	0.0107119	RGMA
270.5043	281.3679	301.1403	318.4585	399.8429	370.26	-0.35161	0.0107299	DENND4A
13.48028	4.953661	12.77565	0	1.049456	3.173657	2.895261	0.0107483	C1orf228
12.58159	13.87025	18.25092	4.149297	3.148369	4.231543	1.955362	0.0107945	FTH1P2
5.392112	11.88879	7.30037	1.037324	1.049456	0	3.550149	0.0108237	ADGRD1
6.290797	3.962929	3.650185	0	0	0	4.58841	0.0108265	GBP1P1
211.191	197.1557	166.996	142.1134	141.6766	134.3515	0.46027	0.010885	THRB
10.78422	7.925857	10.95055	2.074648	0	2.115771	2.824371	0.010894	AL121772.1
6.290797	4.953661	2.737639	0	0	0	4.595327	0.0108972	AL353743.2
54.8198	66.37906	76.65388	40.45564	39.87934	32.79446	0.805812	0.0108996	AL596244.1
185.1292	139.6932	146.0074	97.50847	113.3413	116.3674	0.526374	0.0109036	HRASLS2
574.2599	678.6515	621.444	725.0896	789.1912	725.7096	-0.2579	0.0109077	CEP104
2.696056	8.91659	6.387824	15.55986	12.59348	32.79446	-1.76805	0.010908	ATL1
7.189482	8.91659	10.95055	0	1.049456	2.115771	3.101663	0.0109093	AC087392.5
706.3666	770.7896	829.5045	968.8607	850.0596	917.1869	-0.2465	0.0109199	TFDP2
225.57	235.7943	263.7259	324.6825	289.65	317.3657	-0.36161	0.0109454	C17orf51
39.54215	20.80538	43.80222	13.48521	7.346194	21.15771	1.314042	0.0109461	FAM86C2P
16.17634	15.85171	18.25092	5.186621	4.197825	5.289428	1.777083	0.0109637	ZCWPW1
53.02243	53.49954	56.57787	37.34367	26.23641	24.33137	0.890932	0.0109727	DNAJC3-AS1
125.8159	106.9991	100.3801	57.05283	72.41249	85.68874	0.63207	0.010975	JHY
21.56845	29.72197	26.46384	9.335917	11.54402	9.520971	1.353398	0.0109874	MPZ
195.9134	204.0908	203.4978	161.8226	146.9239	137.5251	0.43501	0.0109912	SCAMP1-AS1
1001.135	1086.833	1010.189	1418.022	1171.193	1100.201	-0.25243	0.0110392	MRPS23
98.85538	110.962	93.07971	117.2176	152.1712	175.609	-0.55564	0.0110502	ANKRD36B
27.85924	31.70343	25.55129	15.55986	12.59348	4.231543	1.391337	0.0111166	ST7-AS1
221.9753	249.6645	266.4635	162.8599	198.3472	192.5352	0.41457	0.0111704	BMT2
156.3712	189.2298	175.2089	127.5909	126.9842	120.599	0.472128	0.0111845	LYPD6
2817.378	2840.429	2817.943	2956.374	3333.073	3200.104	-0.16298	0.0112141	HMGCS1
28.75793	32.69416	37.41439	59.12748	64.01684	52.89428	-0.83265	0.0112199	ZNF30
29.65661	14.86098	17.33838	37.34367	46.17608	45.48908	-1.05552	0.0112213	AL138756.1
68.30008	75.29565	62.05314	129.6655	77.65977	118.4832	-0.66503	0.0112228	ZNF230
48.52901	83.2215	78.47897	117.2176	117.5391	97.32548	-0.66227	0.011228	ENTPD1
33.25136	32.69416	50.19004	19.70916	18.89021	15.86829	1.093037	0.0112286	APOBEC3F

33.25136	23.77757	11.8631	8.298593	7.346194	7.4052	1.579644	0.0112525	AL353807.3
212.9884	223.9055	213.5358	308.0853	279.1554	256.0083	-0.37516	0.0112536	EPC1
3986.568	4380.027	4035.279	4704.265	4592.421	4475.914	-0.1515	0.011262	SOD1
509.5546	510.2271	501.9004	453.3106	398.7934	395.6492	0.286109	0.0112683	BTBD6
7.189482	12.87952	4.562731	1.037324	1.049456	0	3.552745	0.0113038	TMEM239
285.7819	280.3772	328.5166	190.8676	214.0891	268.703	0.410085	0.0113083	ARNTL
18.87239	29.72197	16.42583	9.335917	6.296738	6.347314	1.560746	0.0113228	ARHGEF35
54.8198	41.61075	55.66532	20.74648	27.28586	31.73657	0.933429	0.0113497	FAM227A
238.1516	211.026	215.3609	180.4944	152.1712	163.9723	0.420539	0.011373	THAP3
5.392112	13.87025	5.475277	1.037324	0	1.057886	3.556363	0.0113921	FABP4
32.35267	29.72197	28.28893	13.48521	10.49456	14.8104	1.220415	0.0113993	KDM4A-AS1
12.58159	19.81464	24.63875	8.298593	6.296738	0	1.96252	0.0114016	MISP3
7.189482	5.944393	10.95055	0	1.049456	1.057886	3.521604	0.0114209	FOXB1
959.7959	919.3995	907.0709	1102.676	1034.764	1077.986	-0.20657	0.0114333	FAM162A
8.986853	13.87025	10.95055	2.074648	3.148369	1.057886	2.425285	0.0114341	MRPS30-DT
0.898685	0.990732	2.737639	7.261269	10.49456	11.63674	-2.66054	0.0114837	C3orf49
110.5383	98.08249	119.5436	140.0388	144.825	187.2458	-0.52328	0.0114858	AFG1L
7.189482	3.962929	2.737639	0	0	0	4.587129	0.0114866	TFF2
270.5043	240.7479	260.0757	368.2501	319.0347	301.4974	-0.35781	0.0115173	-
104.2475	70.34198	79.39152	135.8895	115.4402	130.1199	-0.58452	0.0115231	ZNF77
17.07502	21.79611	14.60074	2.074648	3.148369	9.520971	1.860194	0.0115495	AL358472.4
12.58159	15.85171	17.33838	5.186621	5.247282	1.057886	1.990996	0.0115509	AC011005.1
0	0	0.912546	4.149297	8.395651	5.289428	-4.15215	0.0115717	-
0	0	0	1.037324	6.296738	6.347314	-4.7309	0.0115803	AL590302.2
13.48028	13.87025	9.125462	2.074648	4.197825	1.057886	2.313388	0.0115929	SPTBN4
13.48028	16.84245	18.25092	6.223945	3.148369	4.231543	1.834448	0.0116058	AC116366.1
114.133	93.12882	88.51698	68.46339	72.41249	40.19966	0.707946	0.0116062	B3GNTL1
12.58159	9.907322	6.387824	1.037324	1.049456	2.115771	2.782878	0.0116326	AC002553.1
1264.45	1313.711	1356.956	1170.102	1193.232	1042.017	0.208466	0.0116343	SEC24C
1000.237	933.2697	967.299	1156.616	1125.017	1066.349	-0.20658	0.0116355	BRD1
8.986853	2.972197	7.30037	1.037324	0	0	4.099142	0.0116383	SCN4B
13.48028	10.89805	11.8631	0	1.049456	5.289428	2.522154	0.0116591	AC007114.1
306.4517	317.0343	338.5546	227.174	270.7597	254.9504	0.353865	0.0116617	ZNF276
1931.275	1961.65	1920.91	2330.867	2177.622	2057.588	-0.17569	0.0116695	WDR45B
44.93426	63.40686	41.06458	69.50072	96.54998	82.51508	-0.73757	0.01167	MRPL45
13.48028	11.88879	14.60074	4.149297	3.148369	2.115771	2.085335	0.0116803	CD7
303.7556	280.3772	274.6764	262.443	205.6934	169.2617	0.429873	0.0116866	TOR2A
1242.882	1241.387	1182.66	1422.171	1340.156	1416.509	-0.18863	0.0117419	DNAJB12
17.07502	20.80538	11.8631	3.111972	7.346194	3.173657	1.865989	0.0117489	CYGB
87.17247	65.38832	62.05314	28.00775	43.02771	50.77851	0.818846	0.011753	DUSP28
250.7332	231.8313	209.8856	155.5986	192.0505	169.2617	0.422024	0.0118014	MAML2
70.99614	67.36979	71.17861	49.79156	161.6163	170.3196	-0.86458	0.0118033	SMG1P2
11.68291	14.86098	11.8631	4.149297	1.049456	3.173657	2.195173	0.0118053	PRDM6
13.48028	12.87952	4.562731	0	1.049456	3.173657	2.877272	0.0118056	AC073896.4
5.392112	0	2.737639	12.44789	11.54402	15.86829	-2.27149	0.0118264	AP002761.4
8.986853	13.87025	4.562731	1.037324	2.098913	0	3.122441	0.0118852	AC145676.1
65.60403	67.36979	62.05314	46.67959	25.18695	38.08388	0.826075	0.0119134	FAM89B
52.12375	70.34198	72.09115	86.0979	100.7478	118.4832	-0.65121	0.011972	TRDMT1
70.09745	61.4254	67.52842	95.43382	102.8467	104.7307	-0.60503	0.011976	FP565260.7
110.5383	128.7952	113.1557	84.02326	85.00596	66.6468	0.579505	0.0119881	LINC02035
742.314	810.4189	824.9418	665.9621	707.3336	638.9629	0.240453	0.0120215	CHST11
3.594741	0.990732	0	10.37324	10.49456	9.520971	-2.71532	0.012043	LPAL2
451.14	415.1168	434.372	494.8036	533.1238	547.9848	-0.27638	0.0120615	PARP6
17.07502	10.89805	3.650185	0	1.049456	3.173657	2.911038	0.0120987	HCG4P7

144.6883	142.6654	133.2317	183.6064	184.7043	200.9983	-0.43685	0.0121245	NRAV
756.693	825.2799	685.3222	657.6635	676.8993	439.0226	0.353783	0.0121486	PDRG1
415.1926	452.7646	459.0107	362.0261	377.8043	337.4655	0.300349	0.0121706	MRPL55
211.191	161.4893	171.5587	258.2937	238.2266	230.6191	-0.41671	0.0121922	EEF1AKMT3
2.696056	2.972197	8.212916	0	0	0	4.586381	0.0122097	LINC01269
1417.227	1477.182	1337.793	1620.3	1528.008	1680.98	-0.19068	0.0122321	CASP4
13.48028	1.981464	4.562731	0	0	1.057886	4.156409	0.0122382	SHF
44.03558	48.54588	61.1406	87.13523	89.20379	72.99411	-0.69759	0.0122505	AC019069.1
54.8198	36.65709	55.66532	18.67183	33.5826	22.2156	0.984898	0.0122623	RAD51-AS1
289.3767	339.8211	307.5281	369.2874	447.0684	368.1442	-0.33946	0.012286	SNAP23
103.3488	90.15663	109.5055	177.3824	128.0337	135.4094	-0.53997	0.0122941	CD3EAP
13.48028	6.935125	10.95055	3.111972	1.049456	1.057886	2.586496	0.0122998	AC005993.1
16.17634	11.88879	17.33838	32.15705	32.53315	34.91023	-1.12983	0.0123064	CAPSL
7.189482	4.953661	6.387824	0	0	1.057886	4.041333	0.0123208	DGCR9
1136.837	1231.48	1224.637	1032.138	1015.874	1077.986	0.200636	0.0123654	ZNF629
162.662	199.1372	163.3458	142.1134	117.5391	115.3095	0.4844	0.0123723	LCMT2
1049.664	1074.944	1120.607	1236.49	1349.601	1166.848	-0.20973	0.0123732	KIAA1671
14.37896	10.89805	6.387824	1.037324	1.049456	3.173657	2.59299	0.0123816	RHCE
6553.213	6807.321	6609.572	6520.62	5826.582	5352.902	0.173995	0.0123839	GSTP1
8.088168	5.944393	4.562731	0	0	1.057886	4.045492	0.0123944	TPPP3
26.06187	37.64782	27.37639	10.37324	12.59348	15.86829	1.228481	0.0124066	AC004967.1
162.662	149.6006	140.5321	92.32185	116.4897	110.0201	0.506539	0.0124116	SLCO1B3
8.088168	2.972197	2.737639	0	0	0	4.578821	0.012427	TM4SF5
97.9567	98.08249	79.39152	58.09015	70.31358	41.25754	0.698227	0.0124473	GJB5
15.27765	1.981464	9.125462	0	2.098913	0	3.65768	0.0124722	PRRT2
6.290797	4.953661	13.68819	2.074648	0	0	3.578787	0.0124947	ASPDH
15.27765	10.89805	12.77565	39.41832	25.18695	26.44714	-1.22264	0.0124999	RPS24P8
126.7146	108.9805	133.2317	151.4493	176.3087	187.2458	-0.47969	0.0125464	MFSD6
12.58159	5.944393	7.30037	1.037324	1.049456	1.057886	3.040645	0.0125632	RTCA-AS1
8.986853	9.907322	7.30037	2.074648	0	1.057886	3.059408	0.0125694	AP001528.1
1354.319	1368.201	1233.762	1041.473	1117.671	1238.784	0.219452	0.0125864	CHMP2A
7.189482	10.89805	8.212916	0	1.049456	2.115771	3.059042	0.012623	AC008443.5
65.60403	58.4532	69.35351	35.26902	46.17608	29.6208	0.800903	0.0126314	C1orf56
1188.961	1226.526	1287.603	1325.7	1447.2	1469.403	-0.19604	0.0126773	CNOT8
612.9034	621.1891	641.52	587.1255	501.6401	445.3699	0.289683	0.0126846	ECSIT
14.37896	16.84245	19.16347	3.111972	4.197825	7.4052	1.777403	0.0127066	PLIN1
20.66976	23.77757	14.60074	47.71691	25.18695	52.89428	-1.09361	0.0127073	UBE2D3P1
188.7239	281.3679	250.0377	307.0479	332.6777	309.9605	-0.40091	0.0127208	SLIT2
11.68291	14.86098	9.125462	3.111972	3.148369	1.057886	2.281216	0.0127494	MED14OS
27.85924	20.80538	8.212916	8.298593	5.247282	2.115771	1.858197	0.0127623	-
931.9366	892.6497	989.2001	1173.214	1088.286	1017.686	-0.22061	0.0127642	NEDD4
20.66976	22.78684	24.63875	46.67959	51.42336	34.91023	-0.96649	0.012767	AC026412.3
2102.025	2056.76	2101.594	1895.191	1881.675	1828.026	0.15963	0.0127837	SYMPK
956.2011	966.9546	950.8732	1243.752	1106.127	1014.512	-0.22749	0.0128002	COPS7A
7.189482	3.962929	7.30037	0	0	1.057886	4.036195	0.0128759	AC010890.1
884.3063	1022.436	959.0861	901.4347	768.202	733.1148	0.253642	0.0128763	CCDC85C
188.7239	203.1001	183.4218	330.9064	206.7429	249.661	-0.45353	0.0128824	AC026403.1
240.8477	248.6738	231.7867	199.1662	179.457	171.3775	0.390646	0.0128998	SLC46A1
452.9374	447.8109	449.8853	544.5952	527.8765	545.869	-0.26081	0.0129222	HECTD3
35.04873	28.73123	30.11403	9.335917	17.84076	13.75251	1.199479	0.0129287	GEMIN8P4
29.65661	29.72197	33.76421	11.41057	17.84076	11.63674	1.188199	0.0129633	MINDY1
41.33952	59.44393	63.87824	31.11972	25.18695	32.79446	0.884981	0.0129766	ARHGAP44
51.22506	49.53661	50.19004	67.42607	96.54998	80.39931	-0.69456	0.0130082	KIF27
147.3844	97.09175	144.1823	60.1648	70.31358	113.1938	0.675813	0.0130125	EEF1D

816.9049	744.0399	699.0104	878.6135	874.1971	899.2028	-0.23056	0.0130376	ECI2
65.60403	70.34198	94.90481	46.67959	39.87934	51.8364	0.73879	0.0130504	ZC3H6
36.8461	34.67563	44.71476	22.82113	12.59348	20.09983	1.066551	0.0130867	RPS6KA2
1727.273	1684.245	1665.397	1455.366	1535.355	1515.95	0.171987	0.0130987	RDH11
195.9134	213.0074	209.8856	223.0247	304.3423	290.9186	-0.40324	0.0131019	SCRN3
897.7866	927.3253	937.185	681.522	756.658	879.103	0.25358	0.0131173	SVEP1
8.088168	3.962929	3.650185	17.63451	12.59348	22.2156	-1.73381	0.0131598	AC087477.2
83.57773	30.7127	31.02657	158.7106	56.67064	80.39931	-1.02268	0.0131623	ERMARD
53.92112	28.73123	24.63875	79.87396	45.12662	79.34143	-0.92668	0.0131649	SNRPGP10
17.97371	11.88879	10.03801	2.074648	3.148369	4.231543	2.08027	0.0131793	DNAH17-AS1
65.60403	98.08249	64.79078	154.5613	110.1929	95.20971	-0.65914	0.0131812	-
11.68291	11.88879	10.03801	18.67183	26.23641	38.08388	-1.30419	0.0131924	AC008669.1
95.26064	103.0361	106.7679	75.72466	52.47282	69.82045	0.623075	0.0132062	WDR91
136.6002	118.8879	134.1443	73.65001	87.10488	104.7307	0.554784	0.0132222	TMEM161B-AS1
12.58159	3.962929	2.737639	1.037324	0	0	4.100294	0.0132675	TPSG1
11.68291	0.990732	7.30037	0	0	1.057886	4.1531	0.0132988	SAMD14
644.3573	671.7164	666.1587	766.5825	736.7184	826.2087	-0.23302	0.0133178	FTO
457.4308	384.4041	485.4746	515.5501	542.5689	572.3162	-0.29554	0.0133262	CGGBP1
6.290797	6.935125	10.95055	18.67183	23.08804	24.33137	-1.44896	0.0133274	GLIDR
2012.156	2305.434	2121.67	1869.258	1474.486	1137.227	0.522986	0.0133338	C19orf48
30.5553	25.75904	25.55129	4.149297	15.74185	11.63674	1.37866	0.0133886	AL591895.1
0.898685	5.944393	7.30037	0	0	0	4.609863	0.0133904	RFX6
1.797371	2.972197	9.125462	0	0	0	4.587581	0.0134036	SLC39A4
125.8159	132.7581	140.5321	107.8817	90.25325	75.10988	0.545988	0.0134483	NEURL1
22.46713	15.85171	11.8631	4.149297	4.197825	6.347314	1.773718	0.0134483	TRPM2-AS
1911.504	2113.232	2073.305	2293.524	2335.04	2221.56	-0.16808	0.0134583	KPNA6
18.87239	16.84245	28.28893	37.34367	46.17608	44.4312	-0.99657	0.0134611	ZNF573
26.96056	40.62002	42.88967	12.44789	8.395651	26.44714	1.224322	0.0134762	ST3GAL3
162.662	161.4893	172.4712	142.1134	93.40161	114.2517	0.505559	0.0134963	RCN1P2
92.56458	80.24931	80.30407	63.27677	53.52227	39.14177	0.698662	0.0135494	AL021453.1
22.46713	31.70343	14.60074	52.90353	38.82988	45.48908	-1.00176	0.0135651	ATP6V0A4
115.0317	88.17516	114.9808	134.8521	152.1712	168.2038	-0.51447	0.0135692	GORAB
22.46713	40.62002	30.11403	15.55986	15.74185	7.4052	1.264065	0.0135779	TOB2P1
79.0843	70.34198	67.52842	79.87396	167.913	103.6728	-0.69536	0.0135829	-
3.594741	5.944393	3.650185	0	0	0	4.508947	0.0136023	ABCG4
181.5344	510.2271	85.77934	29.04508	66.11575	93.09394	2.046087	0.0136029	-
0.898685	0	0	4.149297	1.049456	13.75251	-4.23911	0.0136109	AC024909.2
461.0255	393.3207	297.4901	226.1367	293.8478	332.1761	0.435077	0.0136218	FAM193B
526.6296	454.7461	498.2502	613.0586	616.0309	554.3321	-0.26871	0.0136558	IFT52
441.2545	523.1066	510.1133	414.9297	412.4363	373.4336	0.295462	0.0136624	SMARCAL1
1997.777	2116.204	2054.142	2121.328	2452.579	2422.558	-0.18188	0.0136736	EP300
35.04873	36.65709	35.5893	49.79156	66.11575	69.82045	-0.7916	0.0136803	AC112220.2
4.493426	4.953661	3.650185	0	0	0	4.500277	0.0136836	COL8A2
1739.855	1789.262	1740.226	2062.2	2016.006	1858.705	-0.17228	0.0136957	QSOX2
79.0843	107.9898	76.65388	130.7028	122.7864	133.2936	-0.55523	0.0137045	ZNF416
12.58159	9.907322	12.77565	2.074648	4.197825	1.057886	2.266769	0.0137361	-
546.4007	577.5969	567.6037	747.9107	697.8885	593.4739	-0.27008	0.0137425	DOLPP1
645.256	659.8276	709.0484	755.172	843.7629	773.3144	-0.23598	0.0137487	PARD3
143.7896	126.8137	164.2583	187.7557	197.2978	205.2298	-0.4394	0.0137511	EMC3-AS1
8.088168	6.935125	16.42583	25.9331	20.98913	33.85234	-1.35635	0.0137572	-
6.290797	0.990732	8.212916	21.78381	12.59348	20.09983	-1.80057	0.0137659	IL13RA2
89.86853	112.9435	88.51698	54.97818	73.46194	58.18371	0.641143	0.0137868	TMEM116
204.9002	181.304	189.8096	220.95	267.6114	267.6451	-0.39175	0.0138089	ZNF700
33.25136	40.62002	31.02657	60.1648	52.47282	69.82045	-0.80067	0.0138448	PI3

2.696056	5.944393	4.562731	0	0	0	4.510287	0.0138557	AL031728.1
818.7023	893.6404	912.5462	1001.018	1094.583	964.7917	-0.22172	0.0138794	SAMHD1
2.696056	12.87952	3.650185	0	0	1.057886	4.086833	0.0138876	UGT1A6
1046.07	1063.056	1064.941	1208.483	1179.589	1227.147	-0.1878	0.0138982	GLA
7.189482	4.953661	4.562731	14.52254	19.93967	17.98406	-1.64737	0.0139428	EFCAB10
5.392112	14.86098	6.387824	0	2.098913	1.057886	3.076056	0.0139699	GRHL2
5946.6	6030.587	5901.436	5536.199	5615.641	5013.32	0.145285	0.0139926	KHSRP
4061.159	4273.028	4218.701	3791.42	3791.686	3847.53	0.135012	0.0139946	PFKP
614.7007	632.0871	641.52	609.9466	494.2939	389.3019	0.338002	0.0140291	DGCR6L
1.797371	6.935125	2.737639	16.59719	10.49456	16.92617	-1.95669	0.0140345	AC098679.2
5.392112	1.981464	1.825092	12.44789	12.59348	13.75251	-2.06675	0.0140638	AC021851.1
26.96056	20.80538	20.07602	39.41832	48.27499	43.37331	-0.9483	0.0140808	AC021106.1
2.696056	4.953661	5.475277	0	0	0	4.502938	0.0140953	AC022398.1
1369.596	1341.451	1195.436	1213.669	1052.605	1094.912	0.216701	0.0141057	PRCC
651.5468	675.6793	780.227	772.8065	872.0982	863.2347	-0.25085	0.0141217	CHM
14.37896	22.78684	24.63875	9.335917	8.395651	2.115771	1.635597	0.0141284	-
21.56845	9.907322	16.42583	5.186621	4.197825	4.231543	1.8176	0.0141387	AL160408.4
591.3349	578.5876	532.927	499.9902	477.5026	434.791	0.269671	0.0142252	C9orf40
5.392112	4.953661	2.737639	0	0	0	4.498962	0.0142256	ACY3
5.392112	4.953661	2.737639	0	0	0	4.498962	0.0142256	TBC1D29
8.088168	8.91659	1.825092	0	0	1.057886	4.060883	0.0142413	LRRC56
57.51586	48.54588	55.66532	35.26902	28.33532	26.44714	0.845211	0.0142453	AC139530.1
55.71849	65.38832	52.92768	93.35917	90.25325	85.68874	-0.63167	0.0142787	PARD3B
712.6574	700.4476	636.0447	561.1924	528.926	623.0947	0.258296	0.0143472	TACC2
108.7409	98.08249	105.8554	137.9641	159.5174	141.7567	-0.48947	0.0143547	KIF9
12.58159	12.87952	16.42583	7.261269	1.049456	1.057886	2.156169	0.0143548	AP001351.1
152.7765	101.0547	141.4447	82.98593	83.95651	99.44125	0.571594	0.0143548	HOMEZ
77.28693	82.23077	87.60444	54.97818	60.86847	34.91023	0.712386	0.0144419	ACTR3C
37.74478	31.70343	24.63875	61.20212	45.12662	63.47314	-0.85145	0.0144609	DAW1
14.37896	8.91659	12.77565	5.186621	1.049456	1.057886	2.303557	0.0144712	KLK14
45.83295	67.36979	65.70333	39.41832	30.43423	30.67868	0.829678	0.0144902	ETNK2
4.493426	7.925857	5.475277	0	0	1.057886	3.987121	0.0145083	AC145124.1
971.4788	980.8249	862.3562	783.1797	796.5374	832.556	0.22236	0.0145164	GUSB
240.8477	243.7201	268.2886	295.6374	326.3809	330.0603	-0.33835	0.0145164	RPS6KA5
143.7896	110.962	139.6196	73.65001	93.40161	101.557	0.555895	0.0145294	ZSCAN30
142.891	155.545	165.1709	89.20988	115.4402	122.7147	0.502279	0.014537	GAS8
499.669	496.3568	544.7901	682.5593	639.1189	545.869	-0.27741	0.0145616	MED27
381.0426	440.8758	388.7447	584.0135	481.7005	443.2541	-0.31876	0.0145701	EXOSC4
6.290797	5.944393	5.475277	0	1.049456	0	3.974588	0.0146237	CST2
0.898685	46.56441	5.475277	4.149297	3.148369	1.057886	2.656016	0.0146261	MIEF2
168.0541	167.4337	168.8211	208.5022	243.4739	212.635	-0.39811	0.0146759	ZNF189
6.290797	0.990732	6.387824	0	0	0	4.566967	0.0146761	ALDH1A1
2.696056	3.962929	6.387824	0	0	0	4.495556	0.01468	C2orf54
2.696056	3.962929	6.387824	0	0	0	4.495556	0.01468	DHDH
4.493426	2.972197	5.475277	0	0	0	4.485469	0.0146806	SLC51B
3.594741	13.87025	9.125462	2.074648	1.049456	0	3.079432	0.0146966	PRRT4
89.86853	94.11956	99.46754	61.20212	71.36303	50.77851	0.628308	0.0147003	HS3ST3A1
53.92112	50.52734	42.88967	69.50072	71.36303	99.44125	-0.70549	0.0147068	C1orf50
14.37896	30.7127	15.51329	3.111972	5.247282	10.57886	1.676612	0.0147175	SMG7-AS1
8.986853	13.87025	14.60074	1.037324	4.197825	3.173657	2.156242	0.0147369	SLC34A3
2249.409	2212.305	2147.221	1989.588	1904.763	2025.851	0.158779	0.0147739	NUMA1
1743.449	1827.901	1646.233	1716.771	1429.36	1265.231	0.241856	0.0147753	MAP2K3
100.6528	107.9898	93.07971	70.53804	67.16521	62.41525	0.591475	0.0148043	MTMR3
195.0147	185.2669	225.3989	255.1817	276.007	254.9504	-0.37541	0.014827	SCCPDH

4.493426	6.935125	1.825092	0	0	0	4.515089	0.0148343	DEFB1
3012.393	3147.556	3130.034	3398.274	3311.035	3570.364	-0.14612	0.014854	DAZAP1
6.290797	3.962929	2.737639	0	0	0	4.49022	0.0148612	LCNL1
2819.176	2959.317	2646.384	2644.139	2504.003	2304.075	0.176727	0.014874	SEC13
1.797371	7.925857	3.650185	0	0	0	4.526433	0.0148842	AGAP1-IT1
17.97371	13.87025	10.03801	75.72466	20.98913	15.86829	-1.42696	0.0149119	-
781.8562	896.6126	763.8012	991.6819	941.3623	924.5921	-0.22745	0.0149121	PUDP
7.189482	5.944393	4.562731	0	1.049456	0	3.973628	0.0149183	AC010491.1
8.088168	8.91659	8.212916	1.037324	2.098913	0	3.004401	0.0149267	SHBG
6.290797	7.925857	3.650185	0	1.049456	0	3.985243	0.0149345	AC125616.1
6.290797	7.925857	3.650185	1.037324	0	0	3.985244	0.0149472	AC010883.1
0	0	0.912546	3.111972	7.346194	6.347314	-4.06613	0.0149502	AC007610.2
0	1.981464	1.825092	9.335917	7.346194	9.520971	-2.80063	0.0149727	TAF7L
29.65661	41.61075	26.46384	21.78381	10.49456	9.520971	1.22161	0.0149787	ZNF623
8.088168	7.925857	6.387824	1.037324	0	1.057886	3.417599	0.0149888	ABCA9
226.4687	228.8591	222.6613	267.6296	284.4027	312.0763	-0.34991	0.0150319	EIF2AK3
6.290797	6.935125	10.03801	2.074648	0	0	3.47718	0.0150635	COL21A1
1.797371	2.972197	4.562731	8.298593	17.84076	12.69463	-2.05727	0.0150731	AL034370.1
101.5514	90.15663	98.55499	122.4042	159.5174	133.2936	-0.51549	0.0150871	AL138789.1
12.58159	14.86098	14.60074	3.111972	6.296738	1.057886	2.004492	0.0150897	AC009271.1
53.92112	32.69416	50.19004	10.37324	24.1375	30.67868	1.072864	0.0151101	SLC44A5
443.9505	438.8944	468.1362	580.9015	533.1238	510.9588	-0.26634	0.0151106	NANS
48.52901	41.61075	41.97713	68.46339	87.10488	62.41525	-0.72153	0.0151203	KIAA1324
2275.471	2389.646	2431.023	2733.349	2450.481	2809.744	-0.17187	0.0151269	CHMP4B
267.8082	273.4421	251.8628	347.5036	312.738	333.234	-0.32536	0.0151359	PIGP
178.8384	184.2762	164.2583	140.0388	129.0831	117.4253	0.447504	0.0151486	CXorf40A
4.493426	9.907322	9.125462	2.074648	0	0	3.490941	0.0151514	AC120498.1
4.493426	7.925857	2.737639	8.298593	17.84076	26.44714	-1.80331	0.0151704	SOS1-IT1
8.088168	5.944393	9.125462	2.074648	0	0	3.471627	0.0152133	AC010894.2
8.986853	5.944393	8.212916	2.074648	0	0	3.470891	0.0152354	AC004596.1
754.8956	775.7433	801.2156	832.9713	914.0765	981.7179	-0.22665	0.0152458	PDCL
9.885538	7.925857	5.475277	0	2.098913	0	3.473174	0.0152459	AC148477.3
31.45398	30.7127	19.16347	13.48521	9.445107	10.57886	1.277836	0.01526	KCNS3
1374.09	1473.219	1395.283	1328.812	1211.073	1166.848	0.19441	0.0152711	PRR13
2433.64	2565.996	2479.388	2980.232	2743.279	2646.83	-0.16266	0.0152742	MDH2
663.2297	454.7461	506.4632	347.5036	460.7113	451.7172	0.367637	0.0152818	GOLGA8B
35.94741	45.57368	41.06458	58.09015	68.21466	78.28354	-0.74036	0.0153041	AC121761.1
319.0333	295.2382	326.6915	363.0634	393.5461	407.286	-0.30599	0.0153108	ZNF3
47.63032	37.64782	34.67676	10.37324	32.53315	8.463085	1.22411	0.0153171	AL137003.1
5.392112	8.91659	8.212916	17.63451	24.1375	20.09983	-1.46178	0.01533	RPSAP54
24.2645	32.69416	33.76421	6.223945	18.89021	12.69463	1.262964	0.015337	EHHADH
709.9614	746.0213	778.4019	791.4783	895.1863	944.6919	-0.23585	0.015359	RAB30
111.437	106.0083	120.4561	159.7479	165.8141	142.8146	-0.47051	0.0153616	HOXA11
8.986853	6.935125	6.387824	0	1.049456	1.057886	3.410242	0.0153678	GNG10
159.0673	157.5264	168.8211	229.2486	188.9021	226.3875	-0.40883	0.0153866	MED31
83.57773	101.0547	94.90481	53.94086	46.17608	76.16777	0.664802	0.015387	APOLD1
9.885538	20.80538	23.7262	4.149297	5.247282	7.4052	1.694984	0.0153941	MRO
8878.112	8551.009	8755.881	8090.091	8245.579	7644.282	0.126947	0.0154763	GPI
3.594741	0.990732	9.125462	0	0	0	4.570707	0.0154956	CDHR5
58.41454	9.907322	11.8631	10.37324	5.247282	8.463085	1.738463	0.0154996	-
339.703	305.1455	301.1403	409.743	396.6945	364.9706	-0.30801	0.0155093	ZNF580
6.290797	10.89805	7.30037	18.67183	17.84076	29.6208	-1.43874	0.0155162	FSIP1
1118.863	1157.175	1216.424	1307.028	1317.068	1344.573	-0.18439	0.0155263	TBC1D1
6.290797	1.981464	10.03801	0	0	1.057886	4.026481	0.0155417	LINC00479

13.48028	6.935125	16.42583	0	3.148369	4.231543	2.326691	0.0155583	MMP25
3.594741	3.962929	0.912546	14.52254	9.445107	12.69463	-2.12086	0.0155643	CR383656.1
6.290797	3.962929	7.30037	0	0	1.057886	3.963872	0.0155768	RPS19P3
9.885538	4.953661	10.95055	23.85846	23.08804	21.15771	-1.39393	0.0155929	ARHGEF33
9.885538	5.944393	14.60074	4.149297	0	0	2.868388	0.0156007	AC090587.2
337.9057	299.2011	316.6535	251.0324	242.4244	264.4714	0.332261	0.015649	EZH1
1277.93	1292.905	1310.416	1149.355	1152.303	1125.59	0.179439	0.0156576	EIF4E2
16.17634	20.80538	24.63875	4.149297	11.54402	5.289428	1.554397	0.015676	AC005674.2
167.1555	153.5635	177.034	231.3233	196.2483	231.677	-0.40459	0.0156994	KCTD18
17.07502	8.91659	9.125462	4.149297	2.098913	1.057886	2.263982	0.0157299	RASA4
14.37896	13.87025	10.95055	5.186621	3.148369	1.057886	2.057719	0.0157676	C1QTNF2
1934.869	2152.861	2029.503	1903.49	1854.389	1506.429	0.216322	0.0157752	ZC3H7B
1745.247	1849.697	1829.655	1929.423	2069.528	2086.151	-0.16588	0.0157809	ERO1A
57.51586	39.62929	44.71476	70.53804	65.06629	100.4991	-0.73233	0.0157831	ZNF816
537.4138	451.7739	500.9879	566.379	595.0417	631.5577	-0.26588	0.015786	SNAPC3
7.189482	9.907322	13.68819	3.111972	2.098913	0	2.557828	0.0157884	LGR6
2.696056	12.87952	16.42583	1.037324	2.098913	2.115771	2.606822	0.0158038	GRPR
10.78422	15.85171	11.8631	2.074648	3.148369	4.231543	2.025387	0.0158119	AC073389.1
36.8461	40.62002	39.23949	11.41057	20.98913	24.33137	1.041455	0.0158499	ANKLE1
167.1555	158.5171	131.4067	213.6888	197.2978	200.9983	-0.42138	0.0158576	MFSD9
264.2135	275.4235	220.8362	175.3078	206.7429	197.8246	0.390678	0.0158919	DCUN1D3
9.885538	7.925857	4.562731	1.037324	1.049456	0	3.41782	0.0159225	SPSB3
520.3388	598.4022	593.155	514.5128	485.8983	384.0125	0.305689	0.0159353	COPG2
1395.658	1396.932	1335.055	1826.728	1572.086	1413.335	-0.22155	0.0159675	E2F4
3.594741	4.953661	9.125462	0	0	1.057886	3.972115	0.0159691	C8orf74
14.37896	11.88879	16.42583	2.074648	7.346194	1.057886	2.027466	0.0160076	AC025178.1
12.58159	6.935125	11.8631	16.59719	31.48369	30.67868	-1.3216	0.0160241	CHRNA3
44.93426	28.73123	40.15203	69.50072	60.86847	63.47314	-0.76477	0.0160255	PABPC4L
195.0147	179.3225	195.2849	244.8085	243.4739	246.4874	-0.36658	0.0160382	ZNF101
88.07116	81.24004	97.64245	120.3296	121.7369	141.7567	-0.52265	0.0160513	HHEX
94.36195	73.31418	91.25462	47.71691	45.12662	68.76257	0.681969	0.0160695	TVP23C
257.024	282.3587	245.4749	207.4648	207.7924	194.651	0.363002	0.016113	ARID3A
1159.304	1147.268	1045.778	1070.519	905.6808	883.3345	0.229128	0.0161227	KMT2B
179.7371	158.5171	164.2583	110.9937	118.5886	136.4673	0.458024	0.0161592	ABHD6
452.0387	506.2641	504.6381	598.536	587.6956	559.6215	-0.25561	0.0161809	WRAP53
8.088168	7.925857	17.33838	1.037324	1.049456	4.231543	2.405341	0.0162131	-
0.898685	0	0	7.261269	2.098913	7.4052	-4.06326	0.0162436	AL031736.1
11.68291	8.91659	6.387824	1.037324	1.049456	2.115771	2.685484	0.0162441	HPCAL4
6897.41	7518.666	7055.807	6793.436	6282.046	6428.771	0.138515	0.0162764	ATF4
1472.945	1613.903	1587.83	1613.039	1897.417	1839.663	-0.19485	0.0163002	AKAP13
93.46327	114.9249	139.6196	175.3078	192.0505	133.2936	-0.52512	0.0163012	MANEAL
319.0333	384.4041	365.931	396.2578	478.5521	450.6593	-0.31045	0.0163242	OARD1
16.17634	12.87952	7.30037	3.111972	0	4.231543	2.308295	0.016363	MAP6D1
209.3937	263.5348	231.7867	176.3451	193.1	163.9723	0.400526	0.0163793	CEP68
222.8739	202.1094	196.1974	188.793	142.7261	117.4253	0.468177	0.0164482	RAB3IL1
39.54215	40.62002	35.5893	15.55986	28.33532	10.57886	1.086749	0.016477	AL031058.1
328.0201	339.8211	300.2277	369.2874	392.4967	436.9068	-0.30858	0.0164886	NMI
2.696056	11.88879	3.650185	1.037324	0	0	4.010691	0.0165088	SLC2A4
26.06187	26.74977	27.37639	37.34367	45.12662	67.70468	-0.90465	0.0165255	ZNF555
187.8252	136.721	181.5967	94.3965	136.4293	124.8305	0.510899	0.0165292	TBC1D32
89.86853	98.08249	89.42953	106.8444	151.1217	142.8146	-0.53136	0.016537	ABRAXAS1
11.68291	9.907322	11.8631	3.111972	2.098913	2.115771	2.190722	0.0165614	LINC01504
79.98299	70.34198	80.30407	51.86621	34.63206	55.01006	0.705493	0.0166124	TIAM2
317.2359	398.2743	410.6458	443.9747	465.9586	484.5116	-0.30894	0.0166278	ZFYVE26

35.04873	35.66636	20.07602	7.261269	18.89021	11.63674	1.264312	0.0166381	INCA1
12.58159	11.88879	14.60074	25.9331	32.53315	28.56291	-1.15403	0.0166396	CDR2
10.78422	4.953661	10.95055	33.19437	19.93967	17.98406	-1.4075	0.0166413	AC005332.4
702.7719	706.392	649.7329	951.2262	799.6857	721.478	-0.26438	0.0166534	BYSL
67.4014	46.56441	54.75277	79.87396	92.35216	92.03605	-0.64467	0.0166654	TRAM2-AS1
89.86853	117.8971	110.4181	146.2627	139.5777	160.7986	-0.49072	0.0167067	ZNF273
354.9807	137.7118	190.7222	347.5036	653.8113	495.0905	-1.13014	0.0167569	YTHDC1
11.68291	7.925857	3.650185	25.9331	16.7913	22.2156	-1.48044	0.0168072	AL035252.3
9.885538	4.953661	7.30037	14.52254	30.43423	17.98406	-1.50108	0.0168396	CCDC7
159.966	101.0547	133.2317	183.6064	202.5451	167.1459	-0.4865	0.0168889	NPIPB5
408.9018	380.4412	388.7447	352.6902	316.9358	276.1082	0.316953	0.0169251	SLC8B1
64.70534	83.2215	84.8668	52.90353	41.97825	49.72063	0.685913	0.0169295	TERT
26.96056	16.84245	12.77565	7.261269	5.247282	6.347314	1.586282	0.0169422	LEKR1
843.8655	836.178	857.7934	718.8656	759.8064	715.1307	0.210258	0.0170005	KLHL15
4.493426	7.925857	0.912546	0	0	0	4.522717	0.0170027	NPR1
1.797371	2.972197	8.212916	0	0	0	4.489394	0.0170536	-
101.5514	112.9435	87.60444	143.1507	135.3799	145.9882	-0.49219	0.0170988	METTL18
130.3094	128.7952	104.0303	87.13523	74.5114	87.80451	0.541286	0.0171021	PRKAA2
9.885538	3.962929	3.650185	0	1.049456	0	3.959865	0.017107	AC004847.1
10.78422	5.944393	17.33838	2.074648	4.197825	0	2.4421	0.0171171	LINC00484
35.04873	37.64782	29.20148	58.09015	58.76956	57.12583	-0.77325	0.0171363	SPEF2
931.0379	948.1307	887.9075	881.7255	771.3504	680.2205	0.245639	0.0171707	DMAC2
0.898685	0.990732	1.825092	3.111972	15.74185	7.4052	-2.81773	0.0171853	AL513174.1
703.6706	618.2169	626.0067	550.8191	523.6787	567.0267	0.247384	0.0172075	CDK2AP2
0.898685	2.972197	2.737639	9.335917	6.296738	16.92617	-2.30932	0.0172077	NGF
17.07502	16.84245	9.125462	1.037324	2.098913	7.4052	2.032637	0.0172212	AC092117.1
911.2669	837.1687	850.4931	1017.615	957.1042	1018.744	-0.20355	0.0172257	GOLGA5
79.98299	95.11029	99.46754	108.919	129.0831	162.9144	-0.54642	0.0172801	MIPOL1
15.27765	24.7683	11.8631	38.38099	29.38478	41.25754	-1.07664	0.0173518	AC016394.2
3.594741	3.962929	3.650185	8.298593	22.03858	11.63674	-1.90588	0.0173574	RASL10A
14.37896	29.72197	21.90111	6.223945	7.346194	10.57886	1.448432	0.017381	AC123595.1
26.06187	16.84245	21.90111	3.111972	9.445107	10.57886	1.489828	0.0174103	CCL20
10.78422	15.85171	7.30037	2.074648	3.148369	2.115771	2.206363	0.01745	C16orf71
0.898685	0	0.912546	1.037324	9.445107	11.63674	-3.58145	0.0174553	AC069499.2
49.42769	63.40686	42.88967	81.94861	92.35216	72.99411	-0.67009	0.0174752	AL513165.1
15.27765	6.935125	22.81366	25.9331	39.87934	37.026	-1.18439	0.0174993	AC093227.1
1399.253	1489.07	1498.401	1331.924	1322.315	1207.048	0.183824	0.0175519	PRCP
45.83295	32.69416	20.07602	20.74648	8.395651	12.69463	1.236312	0.0175791	PET100
87.17247	64.39759	89.42953	56.0155	50.3739	44.4312	0.677671	0.0175869	LINC00265
598.5244	537.9676	703.5731	615.1332	793.389	892.8555	-0.32202	0.0176047	ODF2L
6.290797	6.935125	9.125462	2.074648	0	0	3.419352	0.0176182	-
14.37896	12.87952	14.60074	4.149297	5.247282	2.115771	1.86192	0.0176378	DBNDD1
19.77108	9.907322	14.60074	5.186621	4.197825	3.173657	1.819885	0.0177032	TRPV2
44.03558	36.65709	48.36495	14.52254	16.7913	32.79446	1.011859	0.0177264	AC006547.1
2163.135	2062.704	2075.13	2379.622	2287.815	2324.175	-0.14993	0.0177358	SH3KBP1
644.3573	564.7173	605.0181	580.9015	447.0684	454.8908	0.291076	0.0177419	ZNF768
437.6597	455.7368	396.0451	534.2219	477.5026	546.9269	-0.27405	0.0177433	DIRC2
18.87239	28.73123	22.81366	42.53029	41.97825	47.60486	-0.91126	0.0177477	RYR3
13.48028	14.86098	21.90111	44.60494	28.33532	32.79446	-1.0728	0.0177626	C2orf27A
2.696056	0.990732	1.825092	6.223945	9.445107	13.75251	-2.404	0.017764	AC002059.1
1090.105	1123.49	1031.177	1298.73	1241.507	1168.964	-0.19335	0.0177662	SPRY2
1310.283	1337.488	1233.762	1215.744	1081.989	1092.796	0.194823	0.0178295	EHD4
5.392112	6.935125	4.562731	0	0	1.057886	3.904591	0.0178373	SLC5A10
201.3055	210.0352	209.8856	122.4042	161.6163	176.6669	0.431644	0.0178399	ALDH5A1

14.37896	7.925857	7.30037	0	0	4.231543	2.815515	0.0178419	LUM
887.9011	852.0297	845.0178	959.5248	984.3901	1021.918	-0.1981	0.0178575	MKL2
18.87239	38.63855	18.25092	13.48521	3.148369	10.57886	1.472882	0.0178863	MNX1-AS1
113.2343	96.10102	104.0303	71.57537	76.61031	63.47314	0.566833	0.0179954	HOXC11
4.493426	14.86098	16.42583	38.38099	23.08804	26.44714	-1.30205	0.0179979	RGS7
104.2475	75.29565	89.42953	58.09015	53.52227	62.41525	0.629902	0.0180018	CREB5
570.6652	629.1149	558.4783	633.805	752.4602	703.494	-0.2497	0.0180548	INTS4
16.17634	21.79611	19.16347	2.074648	8.395651	8.463085	1.594393	0.0181075	GRIK2
2673.589	2939.502	2653.684	3343.296	3037.127	2918.707	-0.17009	0.0181182	COX7A2
6.290797	8.91659	9.125462	0	2.098913	1.057886	2.94901	0.0181538	CPXM1
13.48028	8.91659	5.475277	3.111972	0	1.057886	2.736953	0.0181819	AC020558.2
3.594741	6.935125	6.387824	1.037324	0	0	3.90663	0.0182102	RIMBP3B
32.35267	30.7127	48.36495	14.52254	24.1375	14.8104	1.060636	0.0182142	-
14.37896	21.79611	14.60074	10.37324	0	2.115771	2.017746	0.0182383	FLRT1
271.403	249.6645	245.4749	325.7198	289.65	350.1602	-0.33255	0.0182726	KCTD1
8.088168	4.953661	6.387824	20.74648	14.69239	20.09983	-1.5106	0.0182995	CYB5R2
225.57	267.4977	212.6233	204.3529	175.2592	148.104	0.41783	0.018305	SLC45A3
8.088168	5.944393	7.30037	1.037324	1.049456	0	3.350392	0.0183126	AL844908.2
0	0	0.912546	4.149297	6.296738	5.289428	-3.97152	0.0183269	AP001178.2
12.58159	10.89805	11.8631	0	5.247282	2.115771	2.266178	0.018374	PRSS8
3.594741	4.953661	3.650185	0	0	0	4.397101	0.0183771	GJA10
984.9591	983.7971	1017.489	1228.192	1034.764	1185.89	-0.20776	0.0183805	ATRN
31.45398	30.7127	30.11403	13.48521	9.445107	19.04194	1.137267	0.0183819	HMOX2
402.611	416.1075	417.0336	394.1832	299.0951	289.8607	0.329481	0.0184146	TBC1D25
0.898685	0	0	6.223945	4.197825	5.289428	-3.96968	0.0184166	AC093801.1
774.6667	781.6877	724.5617	755.172	558.3108	558.5636	0.284632	0.0184383	NTMT1
6.290797	1.981464	9.125462	1.037324	0	0	3.952752	0.018475	ZMYND15
961.5932	1016.491	1009.276	826.7473	814.3781	938.3446	0.211734	0.0184777	TGFBR3
35.94741	34.67563	35.5893	16.59719	14.69239	21.15771	1.018652	0.0184785	C7orf31
860.0418	954.0751	869.6565	881.7255	699.9874	642.1366	0.270645	0.0185093	WDR18
344.1965	371.5246	397.8702	359.9515	264.463	230.6191	0.380673	0.0185335	THEM6
13.48028	11.88879	15.51329	1.037324	3.148369	6.347314	1.960885	0.0185361	AL031282.2
5.392112	12.87952	4.562731	0	2.098913	0	3.440352	0.018576	MUC5B
903.1787	987.76	943.5728	1108.9	1088.286	1045.191	-0.19443	0.0186175	CLPB
63.80665	82.23077	52.92768	84.02326	99.69835	123.7726	-0.63026	0.0186246	AC124798.1
0	0	0	0	8.395651	5.289428	-4.7314	0.0186273	RNU6-1301P
9.885538	22.78684	20.98856	8.298593	6.296738	1.057886	1.772553	0.0186288	AL022328.2
509.5546	523.1066	536.5772	483.393	429.2276	379.781	0.279712	0.0186299	TRAPPC2L
3.594741	5.944393	2.737639	0	0	0	4.405028	0.0186428	IFNL3
8.088168	0	6.387824	0	0	0	4.649931	0.0186608	AC067930.3
23.36582	26.74977	26.46384	52.90353	44.07717	42.31543	-0.86429	0.0186622	GJB7
161.7634	174.3689	178.8591	127.5909	139.5777	112.1359	0.440802	0.0186668	TTC32
2.696056	4.953661	4.562731	0	0	0	4.398536	0.0186808	LINC02518
152.7765	158.5171	142.3572	114.1057	115.4402	99.44125	0.462926	0.0186867	C12orf76
133.0054	118.8879	146.0074	76.76199	102.8467	98.38337	0.518617	0.0187134	IKZF2
697.3798	710.355	633.3071	701.2311	475.4037	411.5175	0.361521	0.0187343	C20orf27
23.36582	12.87952	20.98856	2.074648	4.197825	11.63674	1.68148	0.0187386	BNIP3P11
3.594741	4.953661	0.912546	11.41057	14.69239	11.63674	-2.00617	0.0187596	-
124.0186	131.7674	110.4181	147.3	188.9021	166.0881	-0.45656	0.0187692	TMLHE
4.493426	3.962929	3.650185	0	0	0	4.387751	0.0187704	PDZK1IP1
12.58159	2.972197	11.8631	1.037324	2.098913	1.057886	2.713658	0.0187915	AC010654.1
452.9374	457.7183	386.0071	390.0339	327.4304	330.0603	0.30725	0.0187954	CHCHD6
29.65661	33.68489	45.62731	13.48521	15.74185	23.27348	1.054769	0.0188003	KLRC2
11.68291	6.935125	25.55129	5.186621	3.148369	3.173657	1.94233	0.0188127	CCL2

2.696056	7.925857	6.387824	0	0	1.057886	3.913309	0.018841	PON1
28.75793	17.83318	21.90111	3.111972	10.49456	11.63674	1.443902	0.0188936	CTBP1-AS
13.48028	16.84245	20.07602	7.261269	2.098913	6.347314	1.681038	0.0189017	CFAP58
18.87239	31.70343	20.98856	48.75423	64.01684	28.56291	-0.98635	0.0189293	IFNAR2
1106.282	1309.748	1132.47	1352.671	1318.117	1406.988	-0.20126	0.0189594	EFNB2
11.68291	14.86098	13.68819	1.037324	1.049456	7.4052	2.086956	0.0189961	ALDH1A2
5.392112	3.962929	7.30037	1.037324	0	0	3.887718	0.0190325	RGS16
555.3875	550.8471	520.1513	443.9747	415.5847	495.0905	0.263773	0.0190403	IDH3G
72.79351	89.1659	101.2926	134.8521	111.2424	134.3515	-0.5318	0.0190585	CD274
1408.24	1454.395	1471.937	1330.887	1229.963	1281.1	0.173933	0.0190665	VAV2
11.68291	21.79611	4.562731	4.149297	2.098913	2.115771	2.178945	0.0190812	AC007773.1
44.03558	39.62929	43.80222	57.05283	77.65977	72.99411	-0.70318	0.0190813	-
0	0	0.912546	5.186621	3.148369	7.4052	-3.97188	0.0190946	Z94721.2
34.15004	26.74977	25.55129	16.59719	3.148369	14.8104	1.32315	0.0191361	VGF
72.79351	90.15663	161.5207	41.49297	41.97825	97.32548	0.844867	0.0191362	-
44.03558	55.481	43.80222	28.00775	18.89021	30.67868	0.884135	0.0191428	-
146.4857	175.3596	153.3078	192.9423	236.1277	200.9983	-0.40832	0.0191431	HTR1D
0	0.990732	0	7.261269	5.247282	3.173657	-3.96747	0.0191486	AC073343.1
3.594741	4.953661	8.212916	0	1.049456	0	3.895395	0.0191504	IRF6
706.3666	786.6413	726.3868	845.4192	850.0596	872.7557	-0.21119	0.0191603	CDK5RAP1
48.52901	29.72197	37.41439	59.12748	59.81901	77.22565	-0.75856	0.0191906	AC006329.1
851.9536	975.8712	925.3219	1082.966	998.033	1097.027	-0.20759	0.0191917	ASCC2
2558.557	2609.589	2615.357	2180.455	2307.755	2484.973	0.158648	0.0192338	PAM
17.07502	21.79611	12.77565	10.37324	3.148369	1.057886	1.8192	0.0192369	ESPNL
697.3798	754.9379	678.9344	876.5389	795.4879	809.2825	-0.22003	0.0192433	HACL1
2.696056	3.962929	5.475277	0	0	0	4.390609	0.0192578	AC131009.2
171.6489	142.6654	234.5244	109.9564	118.5886	154.4513	0.520652	0.0192629	TLR3
12.58159	9.907322	10.95055	41.49297	24.1375	15.86829	-1.28426	0.0193734	EIF4A1P2
150.9791	149.6006	170.6461	187.7557	197.2978	243.3137	-0.4145	0.0193747	HNRNPA3P6
39.54215	53.49954	45.62731	79.87396	72.41249	68.76257	-0.67525	0.0194556	ZNF57
521.2375	535.9861	529.2768	402.4818	458.6124	464.4118	0.259351	0.0194732	SLC12A6
8.986853	3.962929	19.16347	26.97043	26.23641	28.56291	-1.34046	0.0195033	AC078778.1
20.66976	23.77757	18.25092	8.298593	8.395651	7.4052	1.378009	0.0195129	ALG1L6P
143.7896	155.545	128.669	98.54579	110.1929	98.38337	0.47801	0.0195152	PTPRE
43.13689	73.31418	121.3686	39.41832	54.57173	37.026	0.86008	0.0195294	POLR3E
53.92112	63.40686	69.35351	80.91128	98.6489	103.6728	-0.60165	0.0195454	TATDN2P2
26.06187	26.74977	23.7262	17.63451	6.296738	6.347314	1.335396	0.0195502	AC106782.2
17.07502	13.87025	10.95055	6.223945	3.148369	2.115771	1.864488	0.0195615	LRFN1
18.87239	20.80538	19.16347	32.15705	43.02771	39.14177	-0.95899	0.0196136	ANKHD1-EIF4EBP3
16.17634	8.91659	6.387824	3.111972	2.098913	1.057886	2.327423	0.0196191	AC093904.4
9.885538	7.925857	5.475277	1.037324	1.049456	1.057886	2.888785	0.0196395	AC010531.6
3.594741	2.972197	5.475277	0	0	0	4.381197	0.0196689	SBK2
40.44084	44.58295	33.76421	26.97043	18.89021	13.75251	0.992539	0.0197423	RPL41P5
57.51586	43.59222	61.1406	19.70916	39.87934	29.6208	0.865116	0.0197692	DEPTOR
231.8608	221.924	208.9731	291.4881	299.0951	251.7768	-0.34605	0.0198261	MORN2
583.2467	669.735	585.8547	613.0586	432.376	421.0385	0.325702	0.019833	THOC6
250.7332	290.2845	278.3266	238.5846	202.5451	197.8246	0.357842	0.019834	SRGAP2B
4.493426	8.91659	6.387824	16.59719	16.7913	22.2156	-1.49636	0.0198351	AC078899.1
1.797371	4.953661	5.475277	0	0	0	4.400012	0.019859	B3GNT8
17.07502	15.85171	7.30037	2.074648	4.197825	4.231543	1.937765	0.0199265	AL031727.1
44.03558	33.68489	51.10259	25.9331	8.395651	27.50503	1.060293	0.019929	YJEFN3
1915.098	2075.584	2028.59	2164.895	2259.48	2275.512	-0.15476	0.0199393	ANO6
501.4664	567.6895	529.2768	464.7212	423.9804	447.4856	0.257855	0.0199826	RCAN1
993.9459	1017.482	1012.926	1093.34	1172.243	1172.137	-0.18485	0.0199873	SUPT20H

1112.572	1102.685	1136.12	1398.313	1217.369	1218.684	-0.19427	0.0200501	RHNO1
6.290797	2.972197	7.30037	0	0	1.057886	3.880956	0.0200773	MAP7D2
307.3504	197.1557	209.8856	303.936	402.9912	803.9931	-1.08035	0.0200778	-
1.797371	7.925857	2.737639	0	0	0	4.423838	0.0200835	MPPED1
15.27765	16.84245	10.95055	1.037324	1.049456	8.463085	2.032699	0.0201073	CACNB4
16.17634	8.91659	8.212916	16.59719	31.48369	32.79446	-1.27543	0.0201224	AP001350.2
7.189482	2.972197	6.387824	0	1.049456	0	3.879928	0.0201298	RASGRP2
770.1733	819.3355	774.7517	641.0663	695.7896	698.2045	0.216076	0.020144	PTPN13
1210.529	1210.675	1138.858	1162.84	978.0933	897.0871	0.228509	0.0201496	CYFIP1
374.7518	440.8758	441.6724	680.4846	502.6896	426.3279	-0.35692	0.020212	TRMT61A
19.77108	18.82391	24.63875	10.37324	5.247282	8.463085	1.392811	0.0202171	KIF1A
70.09745	76.28638	80.30407	35.26902	47.22554	58.18371	0.688872	0.0202334	AL928654.1
1738.956	1981.464	1807.754	1951.207	1981.374	2519.884	-0.22322	0.0202354	RPL36AL
4.493426	17.83318	13.68819	5.186621	1.049456	1.057886	2.296129	0.020237	AC022413.1
3332.325	3376.415	3240.452	4231.245	3117.935	4249.527	-0.22135	0.020266	SUMO2
8.088168	13.87025	12.77565	26.97043	22.03858	30.67868	-1.2017	0.0202825	AL589182.2
445.7479	388.367	382.3569	324.6825	350.5184	316.3078	0.29548	0.0202898	STRIP2
5.392112	2.972197	8.212916	0	1.049456	0	3.881962	0.0202939	MOV10L1
609.3086	703.4198	634.2196	580.9015	528.926	533.1744	0.244071	0.0202968	ELP6
133.9041	140.684	140.5321	117.2176	80.80814	95.20971	0.500912	0.0203194	Z74021.1
26.06187	27.7405	26.46384	7.261269	15.74185	11.63674	1.212866	0.0203225	C20orf96
27.85924	44.58295	27.37639	16.59719	16.7913	13.75251	1.078982	0.0203502	PRR34
0	0	0.912546	6.223945	2.098913	7.4052	-3.97096	0.0203581	AC097451.1
439.4571	498.3383	469.0488	595.4241	496.3929	605.1106	-0.27106	0.0203668	CDC25C
110.5383	105.0176	94.90481	68.46339	68.21466	75.10988	0.551908	0.0203674	NEBL
28.75793	36.65709	34.67676	12.44789	19.93967	15.86829	1.051953	0.0204223	LIN7B
6.290797	41.61075	50.19004	6.223945	6.296738	20.09983	1.588243	0.0204726	ARHGAP27
182.4331	203.1001	169.7336	144.1881	120.6875	148.104	0.426248	0.0205415	CBY1
78.18562	86.1937	83.95425	45.64226	61.91792	52.89428	0.629909	0.0205526	WNT5B
10.78422	30.7127	5.475277	0	0	8.463085	2.473948	0.0206221	-
34.15004	20.80538	13.68819	36.30634	52.47282	46.54697	-0.97687	0.0206344	AP001318.2
237.2529	246.6923	204.4104	319.4958	270.7597	284.5712	-0.34667	0.0206556	SFR1
20.66976	22.78684	15.51329	12.44789	4.197825	3.173657	1.569194	0.02067	-
69.19877	52.50881	63.87824	104.7697	86.05542	89.92028	-0.59529	0.0206915	AC087392.1
17.07502	11.88879	26.46384	9.335917	6.296738	2.115771	1.642853	0.0207709	NEDD8
37.74478	20.80538	31.93912	10.37324	14.69239	15.86829	1.148035	0.0208404	ETFBKMT
14.37896	21.79611	22.81366	2.074648	7.346194	10.57886	1.561877	0.0208456	AL121753.2
7.189482	3.962929	4.562731	18.67183	13.64293	15.86829	-1.61119	0.0208578	AL627402.1
888.7997	919.3995	963.6488	1024.876	1045.259	1097.027	-0.1923	0.0208996	UGDH
18.87239	14.86098	10.95055	26.97043	39.87934	28.56291	-1.0936	0.0209312	AL121929.2
6.290797	3.962929	1.825092	0	0	0	4.384856	0.0209413	INSRR
6.290797	3.962929	1.825092	0	0	0	4.384856	0.0209413	LRRC32
345.0951	344.7748	329.4292	297.712	283.3532	232.7348	0.32445	0.0209532	TBC1D24
40.44084	39.62929	48.36495	77.79931	59.81901	69.82045	-0.69099	0.0209558	IQCK
253.4292	325.9509	307.5281	346.4663	350.5184	419.9806	-0.33363	0.0209632	PDIA3P1
8.986853	2.972197	4.562731	0	1.049456	0	3.877794	0.020981	IGF2
10.78422	10.89805	13.68819	5.186621	2.098913	1.057886	2.080577	0.0210573	LINC01786
14.37896	24.7683	20.07602	2.074648	2.098913	13.75251	1.725664	0.0210794	CCDC17
14.37896	20.80538	9.125462	6.223945	2.098913	4.231543	1.815218	0.0210943	PPP1R32
4.493426	1.981464	5.475277	0	0	0	4.371656	0.0211466	MIR4260
11.68291	10.89805	25.55129	2.074648	3.148369	8.463085	1.818761	0.0212067	OLFM2
4.493426	8.91659	11.8631	0	3.148369	0	3.005161	0.0212352	AC092123.1
70.09745	85.20297	93.07971	167.0092	133.281	81.4572	-0.62105	0.0212358	-
8.088168	11.88879	8.212916	0	4.197825	0	2.746775	0.0212383	AC136285.1

7.189482	8.91659	7.30037	1.037324	2.098913	0	2.896311	0.0212417	SCN2A
11.68291	12.87952	11.8631	1.037324	6.296738	1.057886	2.117974	0.0212492	THAP12P3
587.7402	484.468	555.7406	588.1628	671.6521	690.7993	-0.25983	0.0212563	FAM160B1
12.58159	10.89805	2.737639	0	0	3.173657	3.053169	0.0212925	NTN5
9.885538	9.907322	14.60074	3.111972	2.098913	3.173657	2.037138	0.0213446	BTBD16
444.8492	372.5153	427.0716	323.6451	312.738	373.4336	0.302414	0.0213672	PDE4B
21.56845	21.79611	25.55129	8.298593	5.247282	13.75251	1.337346	0.021373	WDR88
23.36582	37.64782	24.63875	10.37324	16.7913	10.57886	1.179604	0.0214048	AC008267.5
70.99614	82.23077	74.82879	107.8817	111.2424	108.9622	-0.52585	0.0214086	TMCC3
0	1.981464	1.825092	8.298593	12.59348	4.231543	-2.7394	0.02145	AC004233.3
8.986853	14.86098	8.212916	0	4.197825	2.115771	2.344502	0.0214648	GCOM1
3.594741	1.981464	6.387824	0	0	0	4.373082	0.0214943	CHKB-CPT1B
8.986853	0.990732	7.30037	1.037324	0	0	3.943878	0.0215083	AC009242.1
84.47642	50.52734	74.82879	124.4789	113.3413	84.63085	-0.61706	0.0215467	-
683.8995	750.975	715.4362	858.9044	847.9607	783.8933	-0.2126	0.0215558	NAA20
75.48956	77.27711	63.87824	120.3296	116.4897	84.63085	-0.5703	0.0215583	-
102.4501	106.9991	83.04171	58.09015	73.46194	63.47314	0.584113	0.0215605	ERMAP
30.5553	26.74977	31.02657	68.46339	46.17608	42.31543	-0.82866	0.0215653	CKS1BP3
26.06187	22.78684	19.16347	12.44789	6.296738	8.463085	1.320975	0.0215985	-
480.7966	505.2734	516.5012	601.648	574.0526	592.416	-0.23496	0.0216014	MSL3
1405.544	1515.82	1435.435	1469.888	1149.155	1064.233	0.241947	0.0216724	RNF26
338.8044	295.2382	316.6535	370.3247	408.2385	385.0704	-0.29071	0.0216824	OFD1
21.56845	16.84245	9.125462	0	9.445107	2.115771	2.041337	0.0217009	AJ239328.1
59.31323	71.33272	69.35351	87.13523	87.10488	129.0621	-0.60134	0.0217149	AL592183.1
7.189482	8.91659	5.475277	15.55986	22.03858	20.09983	-1.42239	0.0217515	AC115837.1
28.75793	12.87952	26.46384	11.41057	6.296738	8.463085	1.382559	0.0217885	DICER1-AS1
4.493426	7.925857	8.212916	0	1.049456	1.057886	3.295164	0.0217924	VSIG10L2
19.77108	14.86098	12.77565	5.186621	7.346194	2.115771	1.693992	0.0218004	AC024451.2
271.403	261.5533	226.3115	192.9423	198.3472	202.0562	0.355619	0.0218132	CROCCP2
181.5344	174.3689	153.3078	128.6282	118.5886	131.1778	0.42825	0.0218331	ELMOD3
6.290797	3.962929	8.212916	10.37324	25.18695	19.04194	-1.55784	0.0219245	RPL12P12
263.3148	283.3494	238.1746	342.317	306.4413	329.0024	-0.31797	0.0219503	TECPR2
7.189482	1.981464	7.30037	0	0	1.057886	3.874021	0.0219709	LGI4
3.594741	9.907322	8.212916	0	0	2.115771	3.365598	0.0219743	FBN3
8.088168	11.88879	1.825092	1.037324	1.049456	0	3.377108	0.0220335	ACP5
320.8306	305.1455	330.3417	264.5177	264.463	242.2558	0.310608	0.0220622	HERC2P3
660.5337	611.2818	659.7709	830.8966	707.3336	728.8832	-0.23077	0.0220729	SELENOS
8.986853	11.88879	9.125462	3.111972	1.049456	2.115771	2.253768	0.0220831	TTC25
154.5739	189.2298	178.8591	215.7634	234.0288	227.4454	-0.37487	0.0220842	RPL24P8
1392.962	1382.071	1395.283	1160.766	1227.864	1303.315	0.175922	0.0220907	IDI1
4.493426	6.935125	9.125462	1.037324	1.049456	0	3.295022	0.0221401	KALRN
10.78422	8.91659	13.68819	31.11972	33.5826	14.8104	-1.24965	0.0221605	DLC1
24.2645	25.75904	31.93912	15.55986	10.49456	10.57886	1.161296	0.0221622	C22orf23
306.4517	329.9138	325.779	290.4508	244.5233	231.677	0.327081	0.0221983	NAAA
25.16319	10.89805	20.07602	6.223945	3.148369	9.520971	1.575294	0.0222296	KRT8P36
313.6412	276.4143	280.1517	302.8986	372.557	427.3858	-0.34102	0.0222368	AGAP6
7433.925	7823.812	7241.054	7024.759	5500.201	4864.158	0.371634	0.0222789	CHCHD2
8.986853	6.935125	9.125462	2.074648	1.049456	1.057886	2.581544	0.0222922	DANT2
530.2243	479.5144	479.0868	552.8938	588.745	624.1525	-0.2456	0.0223171	ABI1
7.189482	2.972197	1.825092	0	0	0	4.37531	0.0223269	RN7SKP80
21.56845	73.31418	31.93912	13.48521	3.148369	0	2.928196	0.022374	C16orf45
12.58159	14.86098	10.95055	7.261269	0	1.057886	2.200171	0.0223743	DACH1
2173.021	2420.359	2384.483	2349.539	1942.544	1594.234	0.245201	0.0224093	SLC39A1
88.07116	98.08249	92.16717	77.79931	51.42336	52.89428	0.61068	0.0224354	UAP1L1

45.83295	44.58295	54.75277	23.85846	19.93967	35.96811	0.865121	0.0224378	ABHD14A
160.8647	172.3874	150.5701	206.4275	211.9902	209.4614	-0.37679	0.0224404	GNAL
10.78422	13.87025	7.30037	37.34367	13.64293	27.50503	-1.30099	0.0224453	LDHAP3
13.48028	7.925857	10.03801	4.149297	2.098913	0	2.328286	0.0224458	AL583832.1
79.0843	54.49027	52.92768	36.30634	39.87934	34.91023	0.748808	0.0224765	ZNF585B
8.986853	1.981464	5.475277	1.037324	0	0	3.871885	0.022542	FSBP
8.986853	1.981464	5.475277	1.037324	0	0	3.871885	0.022542	AC018845.3
27.85924	7.925857	55.66532	18.67183	3.148369	8.463085	1.595846	0.0225435	FAM120B
26.06187	29.72197	27.37639	9.335917	13.64293	14.8104	1.13806	0.0225553	AC004816.1
13.48028	16.84245	11.8631	3.111972	5.247282	4.231543	1.743451	0.0225649	KANK4
502.3651	491.4032	482.7369	463.6839	336.8755	403.0544	0.294669	0.0225696	NAA10
36.8461	32.69416	33.76421	70.53804	57.7201	46.54697	-0.7585	0.0226101	-
33.25136	55.481	38.32694	22.82113	26.23641	16.92617	0.942141	0.0226258	MB
23.36582	18.82391	15.51329	20.74648	55.62119	43.37331	-1.05193	0.0226554	AC026356.1
341.5004	354.6821	350.4177	452.2733	430.2771	386.1283	-0.27799	0.0227009	SUCLG2
6.290797	2.972197	0.912546	12.44789	12.59348	13.75251	-1.92564	0.0227334	-
32.35267	28.73123	29.20148	33.19437	73.46194	57.12583	-0.85807	0.0227503	FRMD4A
2.696056	5.944393	4.562731	14.52254	11.54402	16.92617	-1.71057	0.0227511	-
26.06187	16.84245	17.33838	11.41057	6.296738	4.231543	1.456935	0.0227566	BMPER
41.33952	31.70343	35.5893	23.85846	18.89021	10.57886	1.026435	0.0227632	AC069547.1
64.70534	89.1659	62.05314	107.8817	101.7973	107.9043	-0.55957	0.022819	LINC00460
3.594741	8.91659	3.650185	1.037324	0	0	3.838128	0.0228273	MT-TK
26.06187	23.77757	41.06458	16.59719	12.59348	12.69463	1.118899	0.02288	AC006273.1
71.89482	71.33272	56.57787	100.6204	109.1435	86.74663	-0.5703	0.0228923	RCBTB2
8.088168	7.925857	8.212916	0	0	3.173657	2.940574	0.0228925	BDKRB1
26.96056	18.82391	6.387824	3.111972	7.346194	5.289428	1.729195	0.0229288	ATP1A1-AS1
1246.476	1362.257	1356.044	1242.714	1176.441	1024.033	0.203152	0.0229413	DPP3
2.696056	7.925857	5.475277	0	0	1.057886	3.833271	0.0229548	AC100786.1
860.0418	933.2697	967.299	1038.361	1089.336	1030.381	-0.19426	0.0229616	LMTK2
215.6845	185.2669	198.0225	310.1599	242.4244	227.4454	-0.38056	0.022985	ZNF526
20.66976	2.972197	4.562731	16.59719	34.63206	29.6208	-1.5088	0.0229977	AC090607.2
169.8515	145.6376	143.2698	187.7557	202.5451	212.635	-0.39337	0.0230078	INVS
8.088168	25.75904	14.60074	4.149297	4.197825	6.347314	1.717259	0.0230291	AC008946.1
214.7858	180.3133	210.7982	265.555	270.7597	238.0243	-0.35276	0.0230501	THAP1
210.2924	167.4337	146.0074	134.8521	114.3907	132.2357	0.45778	0.0230599	DZIP1L
13.48028	14.86098	9.125462	21.78381	32.53315	28.56291	-1.14769	0.0231227	AC246787.1
421.4834	442.8573	427.0716	414.9297	328.4798	293.0343	0.316741	0.0231229	ATF5
108.7409	132.7581	114.0683	160.7852	155.3195	163.9723	-0.43469	0.0231574	RASSF9
10.78422	10.89805	14.60074	0	3.148369	5.289428	2.109236	0.0231678	LARGE2
305.553	297.2197	289.2772	344.3916	368.3592	376.6073	-0.28812	0.0231811	CBWD2
324.4254	318.025	327.6041	391.0712	390.3978	391.4177	-0.27374	0.0231875	CBLB
8.088168	14.86098	5.475277	3.111972	1.049456	1.057886	2.437849	0.023197	AL117336.2
9.885538	1.981464	4.562731	1.037324	0	0	3.87077	0.0231985	NRSN2
24.2645	22.78684	44.71476	62.23945	50.3739	51.8364	-0.83928	0.0232231	FCMR
1018.21	1130.425	1029.352	1220.931	1184.836	1200.7	-0.18305	0.0232339	AKAP1
6.290797	0	7.30037	0	0	0	4.558788	0.023237	-
9.885538	3.962929	7.30037	2.074648	0	0	3.342682	0.0233887	AC004069.1
2644.831	2791.883	2740.376	3126.495	2882.857	3006.511	-0.14105	0.0234305	NMT1
10.78422	13.87025	7.30037	2.074648	3.148369	2.115771	2.120309	0.0234902	ROPN1L
4.493426	6.935125	17.33838	1.037324	2.098913	2.115771	2.45633	0.0234918	GLIPR1L1
24.2645	19.81464	29.20148	114.1057	19.93967	32.79446	-1.18632	0.0235034	-
631.7758	647.9388	623.2691	654.5515	799.6857	787.0669	-0.23608	0.0235159	C6orf120
39.54215	36.65709	47.4524	17.63451	23.08804	25.38926	0.904846	0.0235427	GSDMB
30.5553	28.73123	29.20148	16.59719	11.54402	13.75251	1.078586	0.0235464	AC069360.1

162.662	166.443	195.2849	220.95	224.5837	231.677	-0.36853	0.0235685	PRELID2
357.6767	367.5616	359.5432	413.8923	441.8211	446.4278	-0.26358	0.0235703	MAPKAPK5-AS1
338.8044	338.8304	308.4406	311.1972	255.0179	190.4194	0.381513	0.0235723	B4GALT7
24.2645	13.87025	16.42583	3.111972	7.346194	8.463085	1.531867	0.0236301	CSF1R
83.57773	105.0176	83.95425	78.83663	39.87934	52.89428	0.665518	0.0236625	-
1601.457	1529.69	1750.264	1295.618	1458.744	1520.182	0.191892	0.0236656	ADD3
145.587	110.962	177.9465	287.3388	987.5384	163.9723	-1.72736	0.0236665	AC005000.1
17.97371	14.86098	10.95055	5.186621	4.197825	4.231543	1.684827	0.0237019	AC091982.3
113.2343	130.7766	114.0683	169.0838	143.7755	171.3775	-0.43663	0.0237167	SPA17
7.189482	5.944393	2.737639	1.037324	0	0	3.816028	0.0237629	TMEM240
336.1083	391.3392	323.0414	294.6001	235.0782	303.6132	0.333291	0.0238221	CXorf40B
8.986853	2.972197	2.737639	22.82113	13.64293	11.63674	-1.70253	0.0239763	TAGLN2P1
24.2645	11.88879	15.51329	4.149297	3.148369	9.520971	1.623641	0.0239938	AP002761.3
247.1385	235.7943	226.3115	204.3529	182.6054	163.9723	0.364205	0.024004	SLC47A1
102.4501	92.13809	104.9428	66.38874	73.46194	65.58891	0.544776	0.0240187	HOMER2
71.89482	51.51807	49.2775	18.67183	39.87934	38.08388	0.839549	0.0240497	MIR3936HG
250.7332	271.4606	257.338	272.8162	345.2711	364.9706	-0.33484	0.0241013	ATG4C
8.986853	15.85171	4.562731	2.074648	3.148369	0	2.486419	0.0241251	FAM57B
3.594741	3.962929	8.212916	0	1.049456	0	3.808364	0.0241463	COX7A1
10.78422	11.88879	8.212916	4.149297	2.098913	0	2.299532	0.0241849	AP001453.4
285.7819	274.4328	307.5281	218.8754	231.9299	243.3137	0.322679	0.0242427	DZIP3
5192.604	5492.619	5441.513	4664.847	5127.644	4930.805	0.131299	0.0242656	ITPR3
31.45398	26.74977	23.7262	1.037324	17.84076	11.63674	1.426913	0.0242682	EIF4HP2
16.17634	8.91659	10.95055	4.149297	2.098913	3.173657	1.937215	0.0243308	AL365356.4
6.290797	2.972197	6.387824	0	0	1.057886	3.799116	0.0243562	SPDYE2B
49.42769	77.27711	84.8668	88.17255	143.7755	96.2676	-0.63473	0.0243798	TMPPE
0.898685	0	0.912546	3.111972	9.445107	6.347314	-3.35488	0.0244386	TEX12
951.7077	1031.352	1061.291	1112.011	1208.974	1146.748	-0.18803	0.0244438	BMPR2
2534.292	2615.533	2631.783	2962.598	2952.121	2691.261	-0.14538	0.0244599	SMARCD1
103.3488	86.1937	128.669	71.57537	82.90705	58.18371	0.582597	0.0244619	AC068152.1
661.4324	698.4662	674.3717	856.8297	805.9825	711.9571	-0.2237	0.0244628	PRPSAP2
271.403	224.8962	218.0985	248.9578	326.3809	348.0444	-0.36928	0.0244656	ZNF107
1.797371	8.91659	5.475277	0	1.049456	0	3.840461	0.0244834	AC087289.5
1.797371	5.944393	6.387824	15.55986	20.98913	9.520971	-1.71157	0.0244868	AL049779.2
5.392112	0.990732	5.475277	0	0	0	4.361819	0.0244982	TPRXL
5.392112	5.944393	10.95055	1.037324	1.049456	1.057886	2.826358	0.0245268	HPX
8.088168	4.953661	2.737639	0	0	1.057886	3.808954	0.0245306	MIR4645
16.17634	17.83318	14.60074	2.074648	8.395651	5.289428	1.625776	0.0245613	AL355994.2
152.7765	124.8323	140.5321	75.72466	79.75868	131.1778	0.546087	0.0246012	SRGAP2D
115.9304	124.8323	105.8554	66.38874	75.56086	97.32548	0.534544	0.0246379	SPTLC3
296.5661	292.266	299.3152	228.2113	234.0288	252.8347	0.31297	0.0246733	NIPSNAP3A
3295.479	3385.332	3244.102	3866.107	3484.195	3580.943	-0.13947	0.0246761	JPT1
115.0317	120.8693	111.3306	139.0014	144.825	193.5931	-0.45949	0.0247027	AL136164.4
576.9559	526.0788	519.2388	648.3276	641.2178	611.4579	-0.22844	0.0247308	PMP22
117.7278	98.08249	122.2812	173.2331	140.6272	149.1619	-0.45235	0.0247412	LDHAP4
2.696056	3.962929	14.60074	1.037324	1.049456	0	3.346332	0.0247488	HIST2H4B
6.290797	0.990732	4.562731	0	0	0	4.360327	0.0247579	MASP1
1700.313	1809.077	1763.952	1993.737	2021.253	1859.763	-0.15607	0.0247743	DESI1
82.67905	73.31418	66.61587	54.97818	47.22554	39.14177	0.655045	0.024783	GPX1P1
18.87239	11.88879	16.42583	23.85846	37.78043	35.96811	-1.04427	0.0247839	ASS1P12
16.17634	3.962929	10.03801	1.037324	0	4.231543	2.525871	0.0248062	DEF6
1.797371	1.981464	0.912546	8.298593	6.296738	10.57886	-2.42887	0.024808	LINC02211
36.8461	44.58295	20.07602	14.52254	17.84076	15.86829	1.071383	0.0248132	TIAF1
1784.789	1959.668	1970.187	1582.957	1719.009	1777.248	0.169947	0.0248403	RBM6

14.37896	9.907322	17.33838	22.82113	31.48369	34.91023	-1.09526	0.0249489	AL592148.3
0	0	0	7.261269	0	5.289428	-4.60762	0.0249505	FSTL5
23.36582	23.77757	33.76421	12.44789	7.346194	15.86829	1.183019	0.024956	LINC00910
139.2962	175.3596	155.1329	193.9796	202.5451	217.9244	-0.38866	0.0249593	IFT46
824.9931	809.4282	816.7289	779.0304	689.4928	629.442	0.224333	0.0249622	VPS4A
9.885538	17.83318	14.60074	31.11972	31.48369	26.44714	-1.07809	0.0249747	PRKG1-AS1
1107.18	1123.49	1226.462	1293.543	1279.287	1335.052	-0.17666	0.0249787	PSEN1
254.3279	213.0074	231.7867	170.1212	151.1217	211.5771	0.392919	0.024983	NOMO1
51486.58	50095.38	48113.09	62848.36	53257.81	50903.34	-0.15791	0.0249984	MT-CO2
17.97371	16.84245	9.125462	5.186621	4.197825	4.231543	1.688937	0.0250229	TCF7L1
9200.74	9559.575	8577.934	11064.1	9882.73	9416.24	-0.15148	0.025034	PTGES
807.0194	798.5301	801.2156	1002.055	935.0656	842.077	-0.20768	0.0250469	AIFM2
1.797371	0	0	2.074648	9.445107	8.463085	-3.43985	0.025074	RNU6-652P
3691.799	4040.206	3993.302	4064.236	4658.537	4293.958	-0.15085	0.0250775	CPD
58.41454	62.41613	47.4524	23.85846	41.97825	31.73657	0.785932	0.0251092	ARHGAP24
