# Supplementary material for: A novel oncogenic seRNA promotes nasopharyngeal carcinoma metastasis
Source: Cell Death Dis. 2022 Apr 23;13(4):401. doi: 10.1038/s41419-022-04846-1 (PMC9035166; doi:10.1038/s41419-022-04846-1)
Supplement: Supplementary file 5 — Supplementary Material 5 [file 41419_2022_4846_MOESM5_ESM.pdf]

Supplementary Data 8, ChIRP-Seq data of seRNA LOC100506178

Refseq_name	Symbol	Summit_ position	TagCounts _in_peak	-10*LOG (p-value)	Fold_ enrichment	Peak_ Classification	Peak_ to_TSS
NM_001291745	ZNF419	1005	93	413.72	39.72	promoter	-278
NM_001098491	ZNF419	1005	93	413.72	39.72	promoter	-278
NM_001291744	ZNF419	1005	93	413.72	39.72	promoter	-278
NM_001098494	ZNF419	1005	93	413.72	39.72	promoter	-278
NM_001098495	ZNF419	1005	93	413.72	39.72	promoter	-278
NM_001098492	ZNF419	1005	93	413.72	39.72	promoter	-278
NM_001098496	ZNF419	1005	93	413.72	39.72	promoter	-278
NM_001291743	ZNF419	1005	93	413.72	39.72	promoter	-278
NM_001098493	ZNF419	1005	93	413.72	39.72	promoter	-278
NM_024691	ZNF419	1005	93	413.72	39.72	promoter	-278
NM_001304337	ZNF773	800	60	216.83	23.83	promoter	-183
NM_001304335	ZNF773	800	60	216.83	23.83	promoter	-183
NM_001304334	ZNF773	800	60	216.83	23.83	promoter	-99
NM_001304336	ZNF773	800	60	216.83	23.83	promoter	-99
NR_130705	ZNF773	800	60	216.83	23.83	promoter	-183
NM_198542	ZNF773	800	60	216.83	23.83	promoter	-183
NM_001324512	ADAMTS12	763	43	100	14.69	promoter	197
NM_030955	ADAMTS12	763	43	100	14.69	promoter	197
NM_001324511	ADAMTS12	763	43	100	14.69	promoter	197
NM_001130142	VWA5A	534	27	72.04	14.04	promoter	-1283
NM_014622	VWA5A	534	27	72.04	14.04	promoter	-1237
NM_198315	VWA5A	534	27	72.04	14.04	promoter	-1237
NM_001321117	ZSCAN22	780	43	55.7	10.85	promoter	-328
NM_001321116	ZSCAN22	780	43	55.7	10.85	promoter	-328
NM_181846	ZSCAN22	780	43	55.7	10.85	promoter	-328
NR_144444	LOC105372480	780	43	55.7	10.85	promoter	583
NM_145804	ABTB2	484	20	41.22	10.33	promoter	-1342
NM_001190460	KRTAP9-1	629	26	43.53	9.72	promoter	224
NM_001256526	TMEM250	756	30	58.03	9.6	promoter	-426
NR_134506	TMEM250	756	30	58.03	9.6	promoter	18
NM_152833	TMEM250	756	30	58.03	9.6	promoter	18
NR_077244	LOC729930	856	28	51.82	9.53	promoter	1320
NM_022060	ABHD4	269	16	34.11	9.53	promoter	-943
NM_001204403	ANK3	510	19	32.76	9.53	promoter	781
NM_018372	LRIF1	706	21	34.5	9.47	promoter	338
NM_001006945	LRIF1	706	21	34.5	9.47	promoter	360
NM_001099669	HIGD1A	736	27	42.38	9.03	promoter	110
NM_014056	HIGD1A	736	27	42.38	9.03	promoter	110
NM_001099668	HIGD1A	736	27	42.38	9.03	promoter	17
NR_110954	LOC101927040	670	17	31.53	8.87	promoter	606
NR_135636	LOC102724908	264	12	32.91	8.87	promoter	-667

NM_017515	SLC35F2	591	15	32.08	8.74	promoter	831
NM_001145658	RAP1GAP	286	16	34.54	8.74	promoter	261
NM_001353795	APBA2	258	11	35.33	8.74	promoter	-1029
NM_001353792	APBA2	258	11	35.33	8.74	promoter	-1029
NM_001353789	APBA2	258	11	35.33	8.74	promoter	-1029
NM_001353790	APBA2	258	11	35.33	8.74	promoter	-1029
NM_001353788	APBA2	258	11	35.33	8.74	promoter	-1029
NM_001164425	MBD3L3	786	18	34.63	8.74	promoter	-1282
NM_002067	GNA11	394	14	36.11	8.74	promoter	670
NM_002455	MTX1	412	18	33.45	8.74	promoter	350
NM_198883	MTX1	412	18	33.45	8.74	promoter	350
NM_001252607	THBS3	412	18	33.45	8.74	promoter	-1067
NM_001252608	THBS3	412	18	33.45	8.74	promoter	-1151
NM_007112	THBS3	412	18	33.45	8.74	promoter	-1151
NR_045553	THBS3	412	18	33.45	8.74	promoter	-1151
NM_001351702	GGT7	493	27	42.88	8.68	promoter	440
NM_178026	GGT7	493	27	42.88	8.68	promoter	438
NM_001290230	CDK2	393	18	30.84	8.49	promoter	-241
NM_052827	CDK2	393	18	30.84	8.49	promoter	-241
NM_001798	CDK2	393	18	30.84	8.49	promoter	-241
NM_006928	PMEL	393	18	30.84	8.49	promoter	124
NM_001200054	PMEL	393	18	30.84	8.49	promoter	-526
NM_001200053	PMEL	393	18	30.84	8.49	promoter	-526
NM_001320122	PMEL	393	18	30.84	8.49	promoter	-526
NM_001320121	PMEL	393	18	30.84	8.49	promoter	-526
NR_034090	DDX11L9	362	22	42.47	8.33	promoter	462
NR_051985	DDX11L9	362	22	42.47	8.33	promoter	462
NR_148357	LOC102725121	362	22	42.47	8.33	promoter	467
NR_046696	IPO9-AS1	505	20	39.27	8.33	promoter	36
NM_018085	IPO9	505	20	39.27	8.33	promoter	367
NM_001137601	ZBTB42	651	19	30.86	7.94	promoter	83
NM_001370342	ZBTB42	651	19	30.86	7.94	promoter	630
NM_031216	SEH1L	403	19	38.11	7.94	promoter	370
NM_001013437	SEH1L	403	19	38.11	7.94	promoter	343
NR_033908	LOC100288069	445	16	30.25	7.94	promoter	-7
NR_126333	LOC101929058	711	20	36.43	7.94	promoter	-168
NM_000562	C8A	471	18	39.13	7.94	promoter	-1982
NM_133170	PTPRT	338	16	33.16	7.94	promoter	1978
NM_007050	PTPRT	338	16	33.16	7.94	promoter	1978
NM_003091	SNRPB	993	22	30.34	7.94	promoter	239
NM_198216	SNRPB	993	22	30.34	7.94	promoter	239
NM_182905	WASHC1	556	20	36.2	7.89	promoter	-388
NR_036051	MIR1302-2	556	20	36.2	7.89	promoter	-16
NR_036266	MIR1302-9	556	20	36.2	7.89	promoter	-16

NR_036267	MIR1302-10	556	20	36.2	7.89	promoter	-16
NR_036268	MIR1302-11	556	20	36.2	7.89	promoter	-16
NM_003362	UNG	652	20	30.86	7.52	promoter	-467
NM_080911	UNG	652	20	30.86	7.52	promoter	108
NM_022361	POPDC3	616	22	32.65	7.49	promoter	653
NM_001368123	CRISP3	609	31	38.33	7.49	promoter	-73
NM_006061	CRISP3	609	31	38.33	7.49	promoter	-73
NM_001190986	CRISP3	609	31	38.33	7.49	promoter	-6
NM_001533	HNRNPL	1821	90	31.24	7.29	promoter	-185
NM_001319652	OSBPL8	684	34	48.37	7.15	promoter	1991
NM_020841	OSBPL8	684	34	48.37	7.15	promoter	1991
NM_001319653	OSBPL8	684	34	48.37	7.15	promoter	1991
NM_001003712	OSBPL8	684	34	48.37	7.15	promoter	1991
NR_134283	LOC100996419	240	12	32.45	7.15	promoter	1729
NM_016175	MRNIP	240	12	32.45	7.15	promoter	-1914
NM_001017987	MRNIP	240	12	32.45	7.15	promoter	-1914
NR_073385	LRRC2-AS1	287	12	30.25	7.15	promoter	-1057
NM_001252377	NT5C	1153	23	35.08	7.1	promoter	42
NR_045513	NT5C	1153	23	35.08	7.1	promoter	-11
NM_014595	NT5C	1153	23	35.08	7.1	promoter	-11
NM_012469	PRPF6	373	20	40.67	7.09	promoter	-240
NM_080621	SAMD10	373	20	40.67	7.09	promoter	-1249
NM_003195	TCEA2	684	24	40.61	6.94	promoter	-1168
NR_046215	LINC00536	740	23	38.98	6.94	promoter	1606
NM_025029	MZT2B	718	23	34.67	6.94	promoter	-60
NM_001330284	MZT2B	718	23	34.67	6.94	promoter	-60
NM_001330282	MZT2B	718	23	34.67	6.94	promoter	-60
NM_017951	SMPD4	718	23	34.67	6.94	promoter	-444
NM_001171083	SMPD4	718	23	34.67	6.94	promoter	-444
NM_017751	SMPD4	718	23	34.67	6.94	promoter	-444
NR_033232	SMPD4	718	23	34.67	6.94	promoter	-599
NR_033231	SMPD4	718	23	34.67	6.94	promoter	-599
NR_146190	FAM185BP	772	21	33.74	6.94	promoter	192
NM_020879	CCDC146	772	21	33.74	6.94	promoter	-517
NM_024410	ODF1	272	18	30.43	6.94	promoter	1205
NM_080598	DDX39B	1319	35	38.75	6.68	promoter	452
NM_004640	DDX39B	1319	35	38.75	6.68	promoter	452
NR_037852	DDX39B	1319	35	38.75	6.68	promoter	452
NR_133674	DDX39B-AS1	1319	35	38.75	6.68	promoter	-752
NR_133675	DDX39B-AS1	1319	35	38.75	6.68	promoter	-752
NR_003065	SNORD84	1319	35	38.75	6.68	promoter	-373
NM_001145641	SRRM5	1399	51	41.11	6.61	promoter	1279
NR_026751	ZNRD1ASP	587	29	33.94	6.45	promoter	406
NM_170783	ZNRD1	587	29	33.94	6.45	promoter	-481

NM_001278785	ZNRD1	587	29	33.94	6.45	promoter	-481
NM_001278786	ZNRD1	587	29	33.94	6.45	promoter	-481
NM_014596	ZNRD1	587	29	33.94	6.45	promoter	-481
NR_103864	ZNRD1	587	29	33.94	6.45	promoter	-481
NR_145418	ZNRD1ASP	587	29	33.94	6.45	promoter	406
NR_145416	ZNRD1ASP	587	29	33.94	6.45	promoter	406
NM_001289972	ZNF84	433	25	32.09	6.43	promoter	-855
NR_110091	ZNF84-DT	433	25	32.09	6.43	promoter	496
NM_001127372	ZNF84	433	25	32.09	6.43	promoter	-782
NM_001289971	ZNF84	433	25	32.09	6.43	promoter	-782
NM_003428	ZNF84	433	25	32.09	6.43	promoter	-559
NR_122031	GAS8-AS1	596	21	30.28	6.38	promoter	-1398
NM_020453	ATP10D	1338	27	36.07	6.38	promoter	-378
NM_001256855	USP17L13	284	18	34.37	6.37	promoter	1898
NM_001369148	ALDH3A2	704	16	32.51	6.35	promoter	972
NM_001369146	ALDH3A2	704	16	32.51	6.35	promoter	972
NM_001369138	ALDH3A2	704	16	32.51	6.35	promoter	972
NM_001369137	ALDH3A2	704	16	32.51	6.35	promoter	972
NM_000382	ALDH3A2	704	16	32.51	6.35	promoter	371
NM_001369139	ALDH3A2	704	16	32.51	6.35	promoter	371
NM_001369136	ALDH3A2	704	16	32.51	6.35	promoter	371
NM_001031806	ALDH3A2	704	16	32.51	6.35	promoter	371
NR_120435	WWOX	508	16	30.14	6.35	promoter	800
NM_001291997	WWOX	508	16	30.14	6.35	promoter	800
NM_016373	WWOX	508	16	30.14	6.35	promoter	800
NR_120436	WWOX	508	16	30.14	6.35	promoter	800
NM_130791	WWOX	508	16	30.14	6.35	promoter	800
NM_032959	POLR2J2	1565	40	42.22	6.15	promoter	-1474
NM_001172774	DPY19L3	660	31	32.75	6.09	promoter	327
NR_046201	LOC400684	660	31	32.75	6.09	promoter	-557
NM_207325	DPY19L3	660	31	32.75	6.09	promoter	-28
NM_145871	GSTZ1	727	45	40.62	6.07	promoter	-753
NM_145870	GSTZ1	727	45	40.62	6.07	promoter	-753
NM_001312660	GSTZ1	727	45	40.62	6.07	promoter	-753
NM_013382	POMT2	727	45	40.62	6.07	promoter	600
NR_158217	LOC105370708	1008	25	41.4	6.04	promoter	481
NM_015656	KIF26A	1008	25	41.4	6.04	promoter	-574
NM_001271156	ZFP41	549	45	64.8	5.9	promoter	685
NM_173832	ZFP41	549	45	64.8	5.9	promoter	685
NR_109945	CTD-2350J17.1	931	30	32.82	5.89	promoter	-1950
NM_001011667	CHCHD7	1335	36	35.15	5.79	promoter	448
NM_001011668	CHCHD7	1335	36	35.15	5.79	promoter	448
NM_001011669	CHCHD7	1335	36	35.15	5.79	promoter	448
NM_001011670	CHCHD7	1335	36	35.15	5.79	promoter	448

NM_001317858	CHCHD7	1335	36	35.15	5.79	promoter	448
NM_001317859	CHCHD7	1335	36	35.15	5.79	promoter	448
NM_024300	CHCHD7	1335	36	35.15	5.79	promoter	448
NR_133934	CHCHD7	1335	36	35.15	5.79	promoter	448
NR_133935	CHCHD7	1335	36	35.15	5.79	promoter	448
NR_133936	CHCHD7	1335	36	35.15	5.79	promoter	448
NR_133937	CHCHD7	1335	36	35.15	5.79	promoter	448
NM_001114634	PLAG1	1335	36	35.15	5.79	promoter	-812
NM_001114635	PLAG1	1335	36	35.15	5.79	promoter	-812
NM_002655	PLAG1	1335	36	35.15	5.79	promoter	-812
NM_001011671	CHCHD7	1335	36	35.15	5.79	promoter	297
NR_004400	RNVU1-18	1607	53	45.95	5.77	promoter	700
NR_004408	RNU1-3	1607	53	45.95	5.77	promoter	700
NR_004421	RNU1-4	1607	53	45.95	5.77	promoter	700
NR_004427	RNU1-2	1607	53	45.95	5.77	promoter	700
NR_004430	RNU1-1	1607	53	45.95	5.77	promoter	700
NM_013449	BAZ2A	272	20	30.66	5.67	promoter	-62
NM_001351156	BAZ2A	272	20	30.66	5.67	promoter	-62
NM_001244390	NUDT2	474	43	30.18	5.64	promoter	14
NM_194313	KIF24	474	43	30.18	5.64	promoter	-251
NM_147172	NUDT2	474	43	30.18	5.64	promoter	-49
NM_147173	NUDT2	474	43	30.18	5.64	promoter	-49
NM_001161	NUDT2	474	43	30.18	5.64	promoter	-49
NR_135155	LINC02329	275	22	31.53	5.56	promoter	-861
NM_001363650	MYL5	260	35	54.23	5.56	promoter	171
NM_007100	ATP5ME	260	35	54.23	5.56	promoter	-436
NR_033743	ATP5ME	260	35	54.23	5.56	promoter	-436
NM_001007075	KLHL5	1175	27	30.11	5.48	promoter	186
NM_001171654	KLHL5	1175	27	30.11	5.48	promoter	186
NR_040095	MIR663AHG	1437	435	41.71	5.42	promoter	331
NR_030386	MIR663A	1437	435	41.71	5.42	promoter	-624
NM_006366	CAP2	1111	27	32.11	5.39	promoter	1346
NM_001363534	CAP2	1111	27	32.11	5.39	promoter	1436
NM_001363533	CAP2	1111	27	32.11	5.39	promoter	1436
NM_001314033	ZNF526	1032	66	36.69	5.21	promoter	-38
NM_133444	ZNF526	1032	66	36.69	5.21	promoter	-38
NR_073047	DEDD2	1032	66	36.69	5.21	promoter	-139
NM_001270614	DEDD2	1032	66	36.69	5.21	promoter	-139
NR_027620	SCGB1B2P	383	30	30.27	4.96	promoter	-1580
NM_001289188	ZNF302	383	30	30.27	4.96	promoter	934
NM_001289189	ZNF302	383	30	30.27	4.96	promoter	934
NM_001289190	ZNF302	383	30	30.27	4.96	promoter	934
NM_001289191	ZNF302	383	30	30.27	4.96	promoter	934
NM_001289192	ZNF302	383	30	30.27	4.96	promoter	934

NM_018675	ZNF302	383	30	30.27	4.96	promoter	934
NM_001289187	ZNF302	383	30	30.27	4.96	promoter	934
NM_018443	ZNF302	383	30	30.27	4.96	promoter	973
NR_110322	ZNF302	383	30	30.27	4.96	promoter	973
NM_001289185	ZNF302	383	30	30.27	4.96	promoter	973
NM_001289184	ZNF302	383	30	30.27	4.96	promoter	973
NM_001289183	ZNF302	383	30	30.27	4.96	promoter	973
NM_001289182	ZNF302	383	30	30.27	4.96	promoter	973
NM_001289181	ZNF302	383	30	30.27	4.96	promoter	973
NM_001289186	ZNF302	383	30	30.27	4.96	promoter	973
NM_001012320	ZNF302	383	30	30.27	4.96	promoter	973
NM_199135	FOXD4L3	1457	31	32.88	4.88	promoter	1535
NR_003594	REXO1L2P	4057	294	71.58	4.77	promoter	-925
NR_003594	REXO1L2P	373	100	37.16	4.38	promoter	-1652
NR_037962	RHOU	2553	101	47.03	4.22	promoter	728
NR_023363	RNA5S1	2553	101	47.03	4.22	promoter	-1447
NR_023364	RNA5S2	2553	101	47.03	4.22	promoter	-1447
NR_023365	RNA5S3	2553	101	47.03	4.22	promoter	-1447
NR_023366	RNA5S4	2553	101	47.03	4.22	promoter	-1447
NR_023367	RNA5S5	2553	101	47.03	4.22	promoter	-1447
NR_023368	RNA5S6	2553	101	47.03	4.22	promoter	-1447
NR_023369	RNA5S7	2553	101	47.03	4.22	promoter	-1447
NR_023370	RNA5S8	2553	101	47.03	4.22	promoter	-1447
NR_023372	RNA5S10	2553	101	47.03	4.22	promoter	-1447
NR_023373	RNA5S11	2553	101	47.03	4.22	promoter	-1447
NR_023374	RNA5S12	2553	101	47.03	4.22	promoter	-1447
NR_023375	RNA5S13	2553	101	47.03	4.22	promoter	-1447
NR_023376	RNA5S14	2553	101	47.03	4.22	promoter	-1447
NR_023377	RNA5S15	2553	101	47.03	4.22	promoter	-1447
NR_023378	RNA5S16	2553	101	47.03	4.22	promoter	-1447
NR_023379	RNA5S17	2553	101	47.03	4.22	promoter	-1447
NR_023363	RNA5S1	2553	101	47.03	4.22	promoter	784
NR_023364	RNA5S2	2553	101	47.03	4.22	promoter	784
NR_023365	RNA5S3	2553	101	47.03	4.22	promoter	784
NR_023366	RNA5S4	2553	101	47.03	4.22	promoter	784
NR_023367	RNA5S5	2553	101	47.03	4.22	promoter	784
NR_023368	RNA5S6	2553	101	47.03	4.22	promoter	784
NR_023369	RNA5S7	2553	101	47.03	4.22	promoter	784
NR_023370	RNA5S8	2553	101	47.03	4.22	promoter	784
NR_023372	RNA5S10	2553	101	47.03	4.22	promoter	784
NR_023373	RNA5S11	2553	101	47.03	4.22	promoter	784
NR_023374	RNA5S12	2553	101	47.03	4.22	promoter	784
NR_023375	RNA5S13	2553	101	47.03	4.22	promoter	784
NR_023376	RNA5S14	2553	101	47.03	4.22	promoter	784

NR_023377	RNA5S15	2553	101	47.03	4.22	promoter	784
NR_023378	RNA5S16	2553	101	47.03	4.22	promoter	784
NR_023379	RNA5S17	2553	101	47.03	4.22	promoter	784
NR_002834	DUSP5P1	2553	101	47.03	4.22	promoter	465
NR_111000	LOC101060524	1556	134	37.05	3.63	promoter	-1129
NR_111001	DRD5P2	1556	134	37.05	3.63	promoter	-1129

Supplementary Data 9, ChIP-Seq data of hnRNPK

GeneID	Chr	Start	End	PeakID	Annotation	Annotated_Transcript
WBP1LP6	1	661476	661714	S18_peak_1	Intergenic between ENST00000416718 and ENST00000438434	ENST00000438434
DDI2	1	15632299	15632466	S18_peak_10	intron (ENST00000480945, intron 3 of 9)	ENST00000480945
RNA5SP377	12	131656275	131656478	S18_peak_1000	Intergenic between ENST00000537280 and ENST00000390852	ENST00000390852
RP11-495K9.6	12	131662469	131662634	S18_peak_1001	promoter-TSS (ENST00000508145)	ENST00000508145
ULK1	12	131911305	131911500	S18_peak_1002	intron (ENST00000321867, intron 12 of 27)	ENST00000321867
RPS11P5	12	132825049	132825316	S18_peak_1003	Intergenic between ENST00000545875 and ENST00000488441	ENST00000488441
ZNF26	12	132980559	132980724	S18_peak_1004	Intergenic between ENST00000544753 and ENST00000540238	ENST00000540238
ZMYM2	13	19957511	19957680	S18_peak_1005	Intergenic between ENST00000620617 and ENST00000382871	ENST00000382871
GJA3	13	20121483	20121648	S18_peak_1006	Intergenic between ENST00000455848 and ENST00000241125	ENST00000241125
CRYL1	13	20386531	20386696	S18_peak_1007	Intergenic between ENST00000356192 and ENST00000298248	ENST00000298248
CRYL1	13	20481186	20481372	S18_peak_1008	intron (ENST00000298248, intron 3 of 7)	ENST00000298248
MIPEP	13	23870810	23870975	S18_peak_1009	intron (ENST00000382172, intron 5 of 18)	ENST00000382172
RP11-307N16.6	13	24090284	24090526	S18_peak_1010	intron (ENST00000382141, intron 3 of 15)	ENST00000382141
RP11-307N16.6	13	24296759	24296924	S18_peak_1011	intron (ENST00000382141, intron 11 of 15)	ENST00000382141
ATP8A2	13	25433145	25433310	S18_peak_1012	intron (ENST00000381655, intron 1 of 36)	ENST00000381655
ATP8A2P3	13	26139093	26139290	S18_peak_1013	intron (ENST00000426792, intron 5 of 16)	ENST00000426792
CDK8	13	26265384	26265549	S18_peak_1014	intron (ENST00000536792, intron 1 of 11)	ENST00000536792
RP11-545M8.4	13	26936760	26936925	S18_peak_1015	Intergenic between ENST00000395943 and ENST00000637583	ENST00000637583
RNU6-63P	13	27502082	27502278	S18_peak_1016	Intergenic between ENST00000516690 and ENST00000316334	ENST00000516690
NPM1P4	13	27701910	27702075	S18_peak_1017	Intergenic between ENST00000414095 and ENST00000302945	ENST00000414095
FLT3	13	28032810	28032975	S18_peak_1018	intron (ENST00000241453, intron 15 of 23)	ENST00000241453
FLT1	13	28455436	28455601	S18_peak_1019	intron (ENST00000282397, intron 3 of 29)	ENST00000282397
CH17-13I23.3	1	143405566	143405731	S18_peak_102	Intergenic between ENST00000615738 and ENST00000622095	ENST00000622095
RP11-161P17.2	13	28768702	28768867	S18_peak_1020	Intergenic between ENST00000419457 and	ENST00000621262

					ENST00000621262	
MTUS2-AS2	13	29049561	29049726	S18_peak_1021	Intergenic between ENST00000422362 and ENST00000434779	ENST00000434779
LINC00365	13	30065234	30065399	S18_peak_1022	Intergenic between ENST00000585222 and ENST00000413591	ENST00000413591
LINC00365	13	30096414	30096579	S18_peak_1023	Intergenic between ENST00000585222 and ENST00000413591	ENST00000413591
KATNAL1	13	30227017	30227182	S18_peak_1024	intron (ENST00000380615, intron 9 of 10)	ENST00000380615
LINC00398	13	30761334	30761544	S18_peak_1025	Intergenic between ENST00000612609 and ENST00000414407	ENST00000414407
RP11-326L9.2	13	31565055	31565220	S18_peak_1026	Intergenic between ENST00000611867 and ENST00000298386	ENST00000611867
PDS5B	13	32565290	32565455	S18_peak_1027	Intergenic between ENST00000629393 and ENST00000315596	ENST00000315596
STARD13	13	33117118	33117292	S18_peak_1028	intron (ENST00000336934, intron 8 of 13)	ENST00000336934
RP11-141M1.3	13	33381354	33381519	S18_peak_1029	intron (ENST00000454681, intron 4 of 5)	ENST00000454681
SRGAP2D	1	143985326	143985491	S18_peak_103	intron (ENST00000605551, intron 1 of 6)	ENST00000605551
RP11-141M1.3	13	33399269	33399434	S18_peak_1030	intron (ENST00000454681, intron 3 of 5)	ENST00000454681
RP11-141M1.3	13	33541868	33542111	S18_peak_1031	intron (ENST00000454681, intron 1 of 5)	ENST00000454681
Metazoa_SRP	13	34745689	34745987	S18_peak_1032	Intergenic between ENST00000430917 and ENST00000614194	ENST00000614194
LINC00445	13	35688059	35688224	S18_peak_1033	Intergenic between ENST00000379919 and ENST00000422430	ENST00000422430
SPG20-AS1	13	36364621	36364893	S18_peak_1034	intron (ENST00000488319, intron 1 of 4)	ENST00000488319
ALG5	13	36998651	36998816	S18_peak_1035	promoter-TSS (ENST00000239891)	ENST00000239891
UFM1	13	38228242	38228407	S18_peak_1036	Intergenic between ENST00000451826 and ENST00000379649	ENST00000379649
LINC00332	13	40129513	40129750	S18_peak_1037	Intergenic between ENST00000391251 and ENST00000455241	ENST00000455241
RN7SKP2	13	40434306	40434719	S18_peak_1038	Intergenic between ENST00000517003 and ENST00000379561	ENST00000517003
FOXO1	13	40552876	40553041	S18_peak_1039	Intergenic between ENST00000517003 and ENST00000379561	ENST00000379561
RP11-666A1.7	1	144391771	144391936	S18_peak_104	Intergenic between ENST00000581164 and ENST00000605374	ENST00000605374
MIR3168	13	41097460	41097702	S18_peak_1040	Intergenic between ENST00000379487 and ENST00000577639	ENST00000577639
LINC00400	13	43149939	43150104	S18_peak_1041	Intergenic between ENST00000379221 and ENST00000425609	ENST00000425609
ENOX1	13	43629447	43629733	S18_peak_1042	intron (ENST00000261488, intron 2 of 16)	ENST00000261488
ENOX1	13	43752665	43752830	S18_peak_1043	intron (ENST00000261488, intron 1 of 16)	ENST00000261488
LINC00390	13	44095315	44095535	S18_peak_1044	Intergenic between ENST00000423211 and ENST00000432331	ENST00000432331
LINC00407	13	44675733	44675962	S18_peak_1045	intron (ENST00000426509, intron 2 of 2)	ENST00000426509

KIAA0226L	13	46320952	46321162	S18_peak_1046	Intergenic between ENST00000566370 and ENST00000389908	ENST00000389908
HTR2A-AS1	13	46835185	46835552	S18_peak_1047	Intergenic between ENST00000378720 and ENST00000455126	ENST00000455126
RN7SL700P	13	47416391	47416750	S18_peak_1048	Intergenic between ENST00000420444 and ENST00000496982	ENST00000496982
LINC00444	13	47900488	47900653	S18_peak_1049	Intergenic between ENST00000417987 and ENST00000435617	ENST00000435617
U1	1	144525174	144525339	S18_peak_105	Intergenic between ENST00000618578 and ENST00000617626	ENST00000618578
MED4-AS1	13	48106480	48106663	S18_peak_1050	Intergenic between ENST00000422483 and ENST00000438155	ENST00000422483
ITM2B	13	48195585	48195780	S18_peak_1051	Intergenic between ENST00000438155 and ENST00000378565	ENST00000378565
EBPL	13	49662038	49662296	S18_peak_1052	intron (ENST00000378284, intron 3 of 4)	ENST00000378284
ST13P4	13	50222413	50222578	S18_peak_1053	Intergenic between ENST00000495613 and ENST00000445636	ENST00000495613
DHRS12	13	51779798	51779963	S18_peak_1054	intron (ENST00000444610, intron 5 of 9)	ENST00000444610
RP11-248G5.8	13	52232940	52233105	S18_peak_1055	intron (ENST00000451298, intron 11 of 21)	ENST00000451298
LINC00558	13	53879796	53879961	S18_peak_1056	Intergenic between ENST00000569422 and ENST00000423442	ENST00000569422
LINC00458	13	54061699	54061928	S18_peak_1057	Intergenic between ENST00000569422 and ENST00000423442	ENST00000423442
RPL13AP25	13	54557799	54558012	S18_peak_1058	Intergenic between ENST00000484130 and ENST00000622486	ENST00000484130
RPL13AP25	13	54654049	54654238	S18_peak_1059	Intergenic between ENST00000484130 and ENST00000622486	ENST00000484130
RP11-289H16.1	1	144800985	144801241	S18_peak_106	intron (ENST00000441760, intron 2 of 3)	ENST00000441760
RP11-78L16.1	13	55052194	55052359	S18_peak_1060	Intergenic between ENST00000616396 and ENST00000616578	ENST00000616578
SPATA2P1	13	56263022	56263187	S18_peak_1061	Intergenic between ENST00000431223 and ENST00000364795	ENST00000431223
RN7SKP6	13	56748127	56748292	S18_peak_1062	Intergenic between ENST00000431223 and ENST00000364795	ENST00000364795
RN7SKP6	13	56766864	56767029	S18_peak_1063	Intergenic between ENST00000431223 and ENST00000364795	ENST00000364795
RN7SKP6	13	56949871	56950105	S18_peak_1064	Intergenic between ENST00000364795 and ENST00000377931	ENST00000364795
RP11-95F22.1	13	57781494	57781659	S18_peak_1065	Intergenic between ENST00000610846 and ENST00000365552	ENST00000610846
CTAGE16P	13	58578100	58578292	S18_peak_1066	Intergenic between ENST00000435601 and ENST00000425106	ENST00000435601
RNY3P5	13	60783939	60784104	S18_peak_1067	Intergenic between ENST00000624954 and ENST00000384127	ENST00000384127

PCDH20	13	61388158	61388334	S18_peak_1068	Intergenic between ENST00000578032 and ENST00000409204	ENST00000409204
RP11-432J3.4	13	61512932	61513171	S18_peak_1069	Intergenic between ENST00000472649 and ENST00000427086	ENST00000472649
RP11-289H16.1	1	144818151	144818316	S18_peak_107	intron (ENST00000441760, intron 2 of 3)	ENST00000441760
LINC00358	13	62032835	62033049	S18_peak_1070	Intergenic between ENST00000432697 and ENST00000455977	ENST00000432697
LINC01075	13	62246193	62246358	S18_peak_1071	intron (ENST00000455977, intron 3 of 3)	ENST00000455977
RP11-527N12.2	13	63055532	63055734	S18_peak_1072	intron (ENST00000618134, intron 2 of 2)	ENST00000618134
LGMNP1	13	65019406	65019597	S18_peak_1073	Intergenic between ENST00000441282 and ENST00000397973	ENST00000441282
HNRNPA3P5	13	65657743	65657998	S18_peak_1074	Intergenic between ENST00000397973 and ENST00000455201	ENST00000455201
PCDH9	13	67113442	67113646	S18_peak_1075	Intergenic between ENST00000456459 and ENST00000377861	ENST00000377861
PCDH9	13	67132836	67133001	S18_peak_1076	Intergenic between ENST00000456459 and ENST00000377861	ENST00000377861
RPSAP53	13	67305434	67305599	S18_peak_1077	Intergenic between ENST00000433498 and ENST00000415120	ENST00000433498
BCRP9	13	67665188	67665528	S18_peak_1078	Intergenic between ENST00000415120 and ENST00000605312	ENST00000605312
KLHL1	13	69597208	69597476	S18_peak_1079	Intergenic between ENST00000424058 and ENST00000377844	ENST00000377844
SRGAP2B	1	144982728	144982893	S18_peak_108	intron (ENST00000612199, intron 3 of 9)	ENST00000612199
ATXN8OS	13	70180318	70180496	S18_peak_1080	Intergenic between ENST00000424524 and ENST00000365563	ENST00000424524
LINC00348	13	71075392	71075557	S18_peak_1081	intron (ENST00000428761, intron 1 of 3)	ENST00000428761
DACH1	13	71394349	71394514	S18_peak_1082	Intergenic between ENST00000440529 and ENST00000613252	ENST00000613252
SNORD37	13	72381423	72381712	S18_peak_1083	Intergenic between ENST00000430654 and ENST00000391075	ENST00000391075
SNORA9	13	72585972	72586215	S18_peak_1084	Intergenic between ENST00000612038 and ENST00000362412	ENST00000362412
PIBF1	13	72923801	72923997	S18_peak_1085	intron (ENST00000326291, intron 13 of 17)	ENST00000326291
RNU4-10P	13	73162651	73162818	S18_peak_1086	Intergenic between ENST00000363397 and ENST00000364383	ENST00000364383
LINC00393	13	73433174	73433341	S18_peak_1087	intron (ENST00000443621, intron 1 of 1)	ENST00000443621
LINC00393	13	73494282	73494460	S18_peak_1088	intron (ENST00000443621, intron 1 of 1)	ENST00000443621
LINC00393	13	73542173	73542338	S18_peak_1089	intron (ENST00000443621, intron 1 of 1)	ENST00000443621
FAM72D	1	145100873	145101038	S18_peak_109	intron (ENST00000400889, intron 2 of 3)	ENST00000400889
KLF12	13	73878476	73878808	S18_peak_1090	intron (ENST00000377669, intron 2 of 6)	ENST00000377669
RP11-157H4.1	13	74027336	74027542	S18_peak_1091	Intergenic between ENST00000625150 and ENST00000419499	ENST00000625150
RNY1P5	13	74303615	74303780	S18_peak_1092	Intergenic between ENST00000383890 and	ENST00000383890

					ENST00000413857	
RP11-285E18.2	13	74453371	74453552	S18_peak_1093	intron (ENST00000446691, intron 1 of 6)	ENST00000446691
RP11-285E18.2	13	74502708	74502953	S18_peak_1094	intron (ENST00000446691, intron 1 of 6)	ENST00000446691
RP11-285E18.2	13	74528236	74528401	S18_peak_1095	intron (ENST00000446691, intron 1 of 6)	ENST00000446691
LINC00347	13	74626593	74626758	S18_peak_1096	Intergenic between ENST00000597518 and ENST00000418778	ENST00000597518
RP11-226E21.4	13	77074883	77075176	S18_peak_1097	intron (ENST00000638147, intron 3 of 4)	ENST00000638147
MYCBP2	13	77323317	77323482	S18_peak_1098	intron (ENST00000544440, intron 1 of 82)	ENST00000544440
RNF219-AS1	13	78444024	78444220	S18_peak_1099	intron (ENST00000607862, intron 2 of 2)	ENST00000607862
CLCNKB	1	16051757	16052035	S18_peak_11	intron (ENST00000375679, intron 14 of 19)	ENST00000375679
U1	1	145423027	145423204	S18_peak_110	Intergenic between ENST00000443612 and ENST00000580816	ENST00000580816
LINC00331	13	78776800	78777079	S18_peak_1100	Intergenic between ENST00000418453 and ENST00000446391	ENST00000446391
PTMAP5	13	81846120	81846285	S18_peak_1101	Intergenic between ENST00000393073 and ENST00000426437	ENST00000393073
PTMAP5	13	82084475	82084640	S18_peak_1102	Intergenic between ENST00000393073 and ENST00000426437	ENST00000393073
LINC00351	13	85342138	85342391	S18_peak_1103	Intergenic between ENST00000433074 and ENST00000424926	ENST00000424926
MOB1AP1	13	85926389	85926577	S18_peak_1104	Intergenic between ENST00000620433 and ENST00000430903	ENST00000430903
PEX12P1	13	89997294	89997516	S18_peak_1105	Intergenic between ENST00000400284 and ENST00000569476	ENST00000400284
LINC00559	13	90028103	90028268	S18_peak_1106	Intergenic between ENST00000400284 and ENST00000569476	ENST00000569476
KRT18P27	13	90205162	90205438	S18_peak_1107	Intergenic between ENST00000427163 and ENST00000427733	ENST00000427733
KRT18P27	13	90293826	90294065	S18_peak_1108	Intergenic between ENST00000427733 and ENST00000426840	ENST00000427733
RP11-487A2.1	13	91526828	91527171	S18_peak_1109	Intergenic between ENST00000635272 and ENST00000634535	ENST00000635272
NBPF25P	1	145574669	145574838	S18_peak_111	intron (ENST00000619932, intron 18 of 19)	ENST00000619932
GPC5-AS1	13	92785024	92785301	S18_peak_1110	Intergenic between ENST00000419288 and ENST00000443228	ENST00000419288
GPC6-AS2	13	93835367	93835633	S18_peak_1111	promoter-TSS (ENST00000445540)	ENST00000445540
TGDS	13	94541334	94541527	S18_peak_1112	Intergenic between ENST00000377028 and ENST00000261296	ENST00000261296
SOX21-AS1	13	94718060	94718363	S18_peak_1113	Intergenic between ENST00000438290 and ENST00000299197	ENST00000438290
ABCC4	13	95208090	95208255	S18_peak_1114	intron (ENST00000376887, intron 6 of 30)	ENST00000376887
LINC00456	13	97115186	97115479	S18_peak_1115	Intergenic between ENST00000298440 and ENST00000453862	ENST00000453862
LINC00456	13	97198882	97199143	S18_peak_1116	Intergenic between ENST00000453862 and	ENST00000453862

					ENST00000469707	
RPL7AP61	13	97912719	97912884	S18_peak_1117	Intergenic between ENST00000443410 and ENST00000604129	ENST00000443410
FARP1	13	98272278	98272443	S18_peak_1118	intron (ENST00000595437, intron 2 of 27)	ENST00000595437
FARP1	13	98445821	98445986	S18_peak_1119	intron (ENST00000595437, intron 25 of 27)	ENST00000595437
GPR89A	1	145681535	145681779	S18_peak_112	Intergenic between ENST00000313835 and ENST00000582693	ENST00000313835
STK24	13	98560092	98560257	S18_peak_1120	intron (ENST00000539966, intron 1 of 10)	ENST00000539966
SLC15A1	13	98691342	98691507	S18_peak_1121	intron (ENST00000376503, intron 18 of 22)	ENST00000376503
RP11-155N3.4	13	98909257	98909422	S18_peak_1122	Intergenic between ENST00000619393 and ENST00000417594	ENST00000619393
DOCK9-AS2	13	99078958	99079123	S18_peak_1123	Intergenic between ENST00000384568 and ENST00000562781	ENST00000562781
NDUFA12P1	13	100019925	100020090	S18_peak_1124	Intergenic between ENST00000564841 and ENST00000455914	ENST00000455914
PCCA	13	100517841	100518006	S18_peak_1125	intron (ENST00000376285, intron 22 of 23)	ENST00000376285
MIR4705	13	102152863	102153063	S18_peak_1126	Intergenic between ENST00000577547 and ENST00000447779	ENST00000577547
TPP2	13	102628126	102628291	S18_peak_1127	intron (ENST00000376065, intron 8 of 28)	ENST00000376065
METTL21C	13	102686731	102686896	S18_peak_1128	intron (ENST00000267273, intron 3 of 3)	ENST00000267273
RPL7P45	13	104832339	104832504	S18_peak_1129	Intergenic between ENST00000455851 and ENST00000448407	ENST00000455851
CH17-78J1.1	1	149113951	149114119	S18_peak_113	intron (ENST00000622818, intron 1 of 2)	ENST00000622818
DAOA-AS1	13	105304593	105304835	S18_peak_1130	Intergenic between ENST00000455851 and ENST00000448407	ENST00000448407
LINC00343	13	105737828	105737993	S18_peak_1131	intron (ENST00000454555, intron 3 of 3)	ENST00000454555
LINC00460	13	106334486	106334653	S18_peak_1132	Intergenic between ENST00000410750 and ENST00000439790	ENST00000439790
FAM155A	13	107423618	107423783	S18_peak_1133	intron (ENST00000375915, intron 1 of 2)	ENST00000375915
MYO16-AS1	13	109222951	109223136	S18_peak_1134	Intergenic between ENST00000439299 and ENST00000618966	ENST00000439299
RP11-40E6.1	13	109787793	109787958	S18_peak_1135	intron (ENST00000615635, intron 1 of 2)	ENST00000615635
COL4A2	13	110460313	110460478	S18_peak_1136	intron (ENST00000360467, intron 22 of 47)	ENST00000360467
RPL21P107	13	110777066	110777263	S18_peak_1137	Intergenic between ENST00000411449 and ENST00000569854	ENST00000411449
TEX29	13	111424166	111424434	S18_peak_1138	Intergenic between ENST00000283547 and ENST00000607406	ENST00000283547
RP11-65D24.2	13	111578174	111578339	S18_peak_1139	Intergenic between ENST00000283547 and ENST00000607406	ENST00000607406
WI2-1896O14.1	1	149272556	149272878	S18_peak_114	intron (ENST00000313342, intron 1 of 6)	ENST00000313342
RP11-120K24.3	13	112935790	112936004	S18_peak_1140	Intergenic between ENST00000615844 and ENST00000602192	ENST00000602192
MCF2L	13	113002887	113003109	S18_peak_1141	intron (ENST00000535094, intron 1 of 29)	ENST00000535094
PCID2	13	113194437	113194739	S18_peak_1142	intron (ENST00000375457, intron 6 of 13)	ENST00000375457

GRTP1-AS1	13	113366842	113367007	S18_peak_1143	Intergenic between ENST00000423246 and ENST00000356501	ENST00000423246
ADPRHL1	13	113450502	113450692	S18_peak_1144	intron (ENST00000375418, intron 1 of 6)	ENST00000375418
5_8S_rRNA	14	16045016	16045181	S18_peak_1145	Intergenic before ENST00000636054	ENST00000636054
5_8S_rRNA	14	16117260	16117425	S18_peak_1146	Intergenic between ENST00000636054 and ENST00000514609	ENST00000636054
5_8S_rRNA	14	16237396	16237561	S18_peak_1147	Intergenic between ENST00000636054 and ENST00000514609	ENST00000636054
5_8S_rRNA	14	16261585	16261753	S18_peak_1148	Intergenic between ENST00000636054 and ENST00000514609	ENST00000636054
5_8S_rRNA	14	16364425	16364590	S18_peak_1149	Intergenic between ENST00000636054 and ENST00000514609	ENST00000636054
RP11-544O24.2	1	149369563	149369728	S18_peak_115	Intergenic between ENST00000618702 and ENST00000585245	ENST00000618702
5_8S_rRNA	14	16987322	16987498	S18_peak_1150	Intergenic between ENST00000636054 and ENST00000514609	ENST00000636054
RP11-754I20.1	14	17445364	17445538	S18_peak_1151	Intergenic between ENST00000636054 and ENST00000514609	ENST00000514609
RP11-754I20.1	14	17533606	17533777	S18_peak_1152	Intergenic between ENST00000636054 and ENST00000514609	ENST00000514609
RP11-754I20.1	14	17773606	17773775	S18_peak_1153	Intergenic between ENST00000636054 and ENST00000514609	ENST00000514609
RP11-754I20.3	14	18370395	18370580	S18_peak_1154	Intergenic between ENST00000384179 and ENST00000547576	ENST00000547576
POTEM	14	18984365	18984538	S18_peak_1155	intron (ENST00000547889, intron 6 of 10)	ENST00000547889
CTD-2314B22.1	14	19002724	19002960	S18_peak_1156	Intergenic between ENST00000391310 and ENST00000611785	ENST00000611785
LINC01296	14	19287313	19287555	S18_peak_1157	intron (ENST00000614614, intron 5 of 5)	ENST00000614614
MED15P6	14	19480550	19480728	S18_peak_1158	Intergenic between ENST00000391214 and ENST00000621415	ENST00000621415
ARHGAP42P4	14	19687308	19687517	S18_peak_1159	Intergenic between ENST00000547175 and ENST00000549782	ENST00000549782
HIST2H3D	1	149804810	149804975	S18_peak_116	Intergenic between ENST00000428289 and ENST00000331491	ENST00000331491
OR11H2	14	19716740	19716905	S18_peak_1160	Intergenic between ENST00000556246 and ENST00000553311	ENST00000556246
RNASE1	14	20822808	20823016	S18_peak_1161	Intergenic between ENST00000397970 and ENST00000556197	ENST00000397970
SLC39A2	14	21003420	21003585	S18_peak_1162	Intergenic between ENST00000298681 and ENST00000298687	ENST00000298681
RPGRIP1	14	21342848	21343013	S18_peak_1163	intron (ENST00000400017, intron 20 of 23)	ENST00000400017
RP11-124D2.1	14	23216551	23216733	S18_peak_1164	Intergenic between ENST00000365359 and ENST00000465411	ENST00000465411
HEATR5A	14	31355686	31355865	S18_peak_1165	intron (ENST00000543095, intron 16 of 35)	ENST00000543095

NPAS3	14	33679045	33679210	S18_peak_1166	intron (ENST00000356141, intron 6 of 11)	ENST00000356141
NPAS3	14	33793995	33794160	S18_peak_1167	intron (ENST00000356141, intron 10 of 11)	ENST00000356141
EAPP	14	34528921	34529096	S18_peak_1168	intron (ENST00000250454, intron 4 of 5)	ENST00000250454
RNU1-27P	14	34547307	34547684	S18_peak_1169	TTS (ENST00000383869)	ENST00000383869
HIST2H2AA3	1	149841742	149841935	S18_peak_117	TTS (ENST00000369159)	ENST00000369159
RALGAPA1	14	35630657	35630822	S18_peak_1170	intron (ENST00000307138, intron 32 of 39)	ENST00000307138
PTCSC3	14	36188447	36188627	S18_peak_1171	Intergenic between ENST00000556013 and ENST00000556542	ENST00000556013
RP11-129M6.1	14	40699926	40700091	S18_peak_1172	Intergenic between ENST00000557101 and ENST00000515218	ENST00000515218
RP11-816J8.1	14	49221365	49221573	S18_peak_1173	Intergenic between ENST00000557062 and ENST00000554200	ENST00000557062
RP11-326E7.1	14	49271028	49271193	S18_peak_1174	Intergenic between ENST00000557062 and ENST00000554200	ENST00000554200
SOS2	14	50171409	50171574	S18_peak_1175	intron (ENST00000216373, intron 8 of 22)	ENST00000216373
MAP4K5	14	50464762	50464981	S18_peak_1176	intron (ENST00000013125, intron 11 of 31)	ENST00000013125
RP11-1140I5.1	14	51130623	51130788	S18_peak_1177	Intergenic between ENST00000554475 and ENST00000457354	ENST00000554475
TXNDC16	14	52415864	52416029	S18_peak_1178	Intergenic between ENST00000245457 and ENST00000281741	ENST00000281741
MIR5580	14	53940073	53940394	S18_peak_1179	Intergenic between ENST00000426913 and ENST00000580850	ENST00000580850
OTUD7B	1	149968872	149969089	S18_peak_118	intron (ENST00000581312, intron 3 of 11)	ENST00000581312
ATP5C1P1	14	54045004	54045169	S18_peak_1180	Intergenic between ENST00000441945 and ENST00000335183	ENST00000441945
C14orf105	14	57510627	57510792	S18_peak_1181	Intergenic between ENST00000529860 and ENST00000555600	ENST00000529860
DACT1	14	58626844	58627073	S18_peak_1182	Intergenic between ENST00000479477 and ENST00000395153	ENST00000395153
LRRC9	14	60051290	60051455	S18_peak_1183	intron (ENST00000445360, intron 29 of 31)	ENST00000445360
HIF1A	14	61741430	61741638	S18_peak_1184	intron (ENST00000337138, intron 12 of 14)	ENST00000337138
RP11-395B20.2	14	62424383	62424640	S18_peak_1185	Intergenic between ENST00000604527 and ENST00000554127	ENST00000554127
PARP1P2	14	63113014	63113179	S18_peak_1186	Intergenic between ENST00000394964 and ENST00000556806	ENST00000556806
RHOJ	14	63200883	63201048	S18_peak_1187	Intergenic between ENST00000554921 and ENST00000316754	ENST00000316754
ZBTB25	14	64479445	64479610	S18_peak_1188	exon (ENST00000608382, exon 3 of 3)	ENST00000608382
CTD-2014B16.1	14	65881076	65881325	S18_peak_1189	Intergenic between ENST00000555712 and ENST00000557194	ENST00000555712
SNORA40	1	150593725	150593890	S18_peak_119	Intergenic between ENST00000307940 and ENST00000517238	ENST00000517238
CCDC177	14	69592602	69592767	S18_peak_1190	Intergenic between ENST00000599174 and ENST00000342745	ENST00000599174

RP11-718G2.5	14	70268871	70269050	S18_peak_1191	Intergenic between ENST00000530196 and ENST00000553791	ENST00000553791
PCNX1	14	71054638	71054899	S18_peak_1192	intron (ENST00000304743, intron 24 of 35)	ENST00000304743
RGS6	14	72294989	72295154	S18_peak_1193	Intergenic between ENST00000363428 and ENST00000554782	ENST00000554782
ZFYVE1	14	73006934	73007099	S18_peak_1194	intron (ENST00000556143, intron 2 of 11)	ENST00000556143
ZFYVE1	14	73014382	73014547	S18_peak_1195	intron (ENST00000556143, intron 2 of 11)	ENST00000556143
SUB1P2	14	74322303	74322468	S18_peak_1196	Intergenic between ENST00000556401 and ENST00000256362	ENST00000556401
FLVCR2	14	75597709	75597887	S18_peak_1197	intron (ENST00000238667, intron 1 of 9)	ENST00000238667
LRRC74A	14	76830692	76830857	S18_peak_1198	intron (ENST00000393774, intron 2 of 13)	ENST00000393774
LINC01629	14	76960045	76960210	S18_peak_1199	promoter-TSS (ENST00000553613)	ENST00000553613
RNU1-6P	1	16536003	16536235	S18_peak_12	Intergenic between ENST00000516290 and ENST00000600179	ENST00000516290
RP11-316M1.12	1	150966978	150967248	S18_peak_120	TTS (ENST00000561111)	ENST00000561111
SPTLC2	14	77572739	77572904	S18_peak_1200	intron (ENST00000216484, intron 4 of 11)	ENST00000216484
NRXN3	14	79297748	79297993	S18_peak_1201	intron (ENST00000557594, intron 1 of 5)	ENST00000557594
RP11-526N18.1	14	79930184	79930399	S18_peak_1202	intron (ENST00000554307, intron 2 of 2)	ENST00000554307
DIO2-AS1	14	80544257	80544505	S18_peak_1203	Intergenic between ENST00000553979 and ENST00000555320	ENST00000553979
RNU6ATAC28P	14	83565681	83565846	S18_peak_1204	Intergenic between ENST00000516560 and ENST00000459249	ENST00000459249
RP11-789A21.1	14	85255892	85256057	S18_peak_1205	Intergenic between ENST00000556016 and ENST00000557547	ENST00000556016
Y_RNA	14	88834010	88834379	S18_peak_1206	Intergenic between ENST00000363783 and ENST00000384612	ENST00000384612
MPPE1P1	14	89088968	89089133	S18_peak_1207	Intergenic between ENST00000637466 and ENST00000557286	ENST00000557286
MPPE1P1	14	89130669	89130897	S18_peak_1208	Intergenic between ENST00000557286 and ENST00000612252	ENST00000557286
CPSF2	14	92142670	92142835	S18_peak_1209	intron (ENST00000298875, intron 8 of 15)	ENST00000298875
SCNM1	1	151169235	151169626	S18_peak_121	TTS (ENST00000368902)	ENST00000368902
SYNE3	14	95429018	95429202	S18_peak_1210	intron (ENST00000334258, intron 16 of 16)	ENST00000334258
AK7	14	96452486	96452682	S18_peak_1211	intron (ENST00000267584, intron 10 of 17)	ENST00000267584
RP11-1029J19.4	14	101613213	101613378	S18_peak_1212	Intergenic between ENST00000510508 and ENST00000553270	ENST00000553270
PPP2R5C	14	101798511	101798726	S18_peak_1213	intron (ENST00000328724, intron 3 of 14)	ENST00000328724
AMN	14	102912092	102912257	S18_peak_1214	Intergenic between ENST00000384242 and ENST00000299155	ENST00000299155
CTD-2134A5.4	14	103877660	103877825	S18_peak_1215	intron (ENST00000555967, intron 2 of 3)	ENST00000555967
RP11-79C23.1	15	19563267	19563552	S18_peak_1216	Intergenic before ENST00000524329	ENST00000524329
RN7SL584P	15	20086743	20086908	S18_peak_1217	Intergenic between ENST00000553953 and ENST00000492309	ENST00000492309
RP11-492D6.3	15	20241563	20241739	S18_peak_1218	intron (ENST00000557586, intron 10 of 12)	ENST00000557586

HERC2P3	15	20499896	20500061	S18_peak_1219	intron (ENST00000428453, intron 2 of 26)	ENST00000428453
PI4KB	1	151302021	151302186	S18_peak_122	intron (ENST00000368874, intron 6 of 10)	ENST00000368874
RP11-467L19.16	15	20642758	20642923	S18_peak_1220	promoter-TSS (ENST00000613130)	ENST00000613130
RP11-403B2.10	15	20802821	20803004	S18_peak_1221	TTS (ENST00000621235)	ENST00000621235
HERC2P2	15	22566751	22566961	S18_peak_1222	intron (ENST00000613386, intron 24 of 31)	ENST00000613386
CTD-3092A11.3	15	30508594	30508769	S18_peak_1223	promoter-TSS (ENST00000636170)	ENST00000636170
RNU6-18P	15	32277962	32278127	S18_peak_1224	Intergenic between ENST00000605648 and ENST00000384527	ENST00000384527
RP11-1000B6.3	15	32540930	32541162	S18_peak_1225	intron (ENST00000564670, intron 1 of 2)	ENST00000564670
HNRNPLP2	15	34454704	34454869	S18_peak_1226	Intergenic between ENST00000623619 and ENST00000568980	ENST00000568980
RP11-184D12.1	15	36203933	36204179	S18_peak_1227	intron (ENST00000561394, intron 2 of 4)	ENST00000561394
FSIP1	15	39754451	39754616	S18_peak_1228	intron (ENST00000350221, intron 5 of 11)	ENST00000350221
OIP5	15	41322583	41322848	S18_peak_1229	intron (ENST00000220514, intron 2 of 4)	ENST00000220514
RIIAD1	1	151732494	151732659	S18_peak_123	Intergenic between ENST00000479191 and ENST00000384253	ENST00000479191
TTBK2	15	42805994	42806159	S18_peak_1230	intron (ENST00000267890, intron 9 of 14)	ENST00000267890
AC011330.5	15	43687920	43688085	S18_peak_1231	Intergenic between ENST00000510070 and ENST00000434505	ENST00000510070
FRMD5	15	44010525	44010698	S18_peak_1232	intron (ENST00000484674, intron 2 of 15)	ENST00000484674
SPATA5L1	15	45404546	45404711	S18_peak_1233	intron (ENST00000305560, intron 1 of 7)	ENST00000305560
USP8	15	50492669	50492873	S18_peak_1234	exon (ENST00000396444, exon 15 of 20)	ENST00000396444
DMXL2	15	51544338	51544503	S18_peak_1235	intron (ENST00000543779, intron 8 of 42)	ENST00000543779
MAPK6	15	52039678	52039843	S18_peak_1236	intron (ENST00000261845, intron 1 of 5)	ENST00000261845
GNB5	15	52146304	52146486	S18_peak_1237	intron (ENST00000261837, intron 6 of 12)	ENST00000261837
ARPP19	15	52540425	52540590	S18_peak_1238	Intergenic between ENST00000567221 and ENST00000249822	ENST00000249822
RAB27A	15	55217120	55217285	S18_peak_1239	intron (ENST00000396307, intron 5 of 5)	ENST00000396307
SNAPIN	1	153656580	153656934	S18_peak_124	Intergenic between ENST00000469931 and ENST00000368685	ENST00000368685
FAM81A	15	59518204	59518369	S18_peak_1240	intron (ENST00000288228, intron 8 of 8)	ENST00000288228
RP11-82L7.4	15	60190641	60190922	S18_peak_1241	Intergenic between ENST00000560503 and ENST00000396024	ENST00000560503
RP11-244F12.2	15	63084349	63084723	S18_peak_1242	Intergenic between ENST00000558905 and ENST00000560238	ENST00000558905
RPS27L	15	63159983	63160148	S18_peak_1243	Intergenic between ENST00000330964 and ENST00000321437	ENST00000330964
FBXL22	15	63601832	63602029	S18_peak_1244	exon (ENST00000360587, exon 2 of 2)	ENST00000360587
HNRNPA1P44	15	65825826	65825991	S18_peak_1245	Intergenic between ENST00000503999 and ENST00000636829	ENST00000503999
MIR4311	15	66091745	66092015	S18_peak_1246	Intergenic between ENST00000580794 and ENST00000564269	ENST00000580794
RP11-34F13.3	15	67837516	67837732	S18_peak_1247	intron (ENST00000558889, intron 2 of 2)	ENST00000558889
RPL5P3	15	71072714	71072888	S18_peak_1248	Intergenic between ENST00000493817 and	ENST00000493817

					ENST00000449977	
CD276	15	73639148	73639313	S18_peak_1249	Intergenic between ENST00000628401 and ENST00000318443	ENST00000318443
MIR8083	1	153695418	153695672	S18_peak_125	Intergenic between ENST00000620823 and ENST00000461982	ENST00000620823
EDC3	15	74672082	74672247	S18_peak_1250	intron (ENST00000315127, intron 2 of 6)	ENST00000315127
SIN3A	15	75391077	75391242	S18_peak_1251	intron (ENST00000394947, intron 15 of 20)	ENST00000394947
MORF4L1	15	78887754	78888115	S18_peak_1252	intron (ENST00000426013, intron 5 of 11)	ENST00000426013
GOLGA6L17P	15	82521726	82521911	S18_peak_1253	intron (ENST00000611297, intron 2 of 5)	ENST00000611297
RP11-382A20.5	15	82954250	82954415	S18_peak_1254	intron (ENST00000566841, intron 1 of 1)	ENST00000566841
DNM1P41	15	84168692	84168927	S18_peak_1255	promoter-TSS (ENST00000623634)	ENST00000623634
GOLGA2P7	15	84213481	84213699	S18_peak_1256	intron (ENST00000400817, intron 1 of 6)	ENST00000400817
LINC00933	15	84586724	84586889	S18_peak_1257	Intergenic between ENST00000557887 and ENST00000618330	ENST00000557887
SLC28A1	15	84943763	84943928	S18_peak_1258	intron (ENST00000286749, intron 15 of 17)	ENST00000286749
AKAP13	15	85748621	85748786	S18_peak_1259	exon (ENST00000394518, exon 37 of 37)	ENST00000394518
GATAD2B	1	153880219	153880436	S18_peak_126	intron (ENST00000368655, intron 1 of 10)	ENST00000368655
MRPL46	15	88377428	88377593	S18_peak_1260	Intergenic between ENST00000569588 and ENST00000312475	ENST00000312475
ABHD2	15	89089139	89089304	S18_peak_1261	promoter-TSS (ENST00000352732)	ENST00000352732
FES	15	90901861	90902045	S18_peak_1262	Intergenic between ENST00000444422 and ENST00000560522	ENST00000444422
IGF1R	15	98953956	98954167	S18_peak_1263	intron (ENST00000268035, intron 20 of 20)	ENST00000268035
TTC23	15	99231497	99231662	S18_peak_1264	intron (ENST00000558663, intron 2 of 11)	ENST00000558663
MPG	16	82311	82500	S18_peak_1265	intron (ENST00000356432, intron 2 of 3)	ENST00000356432
RAB11FIP3	16	453495	453677	S18_peak_1266	intron (ENST00000262305, intron 1 of 13)	ENST00000262305
WDR90	16	652230	652395	S18_peak_1267	intron (ENST00000420061, intron 9 of 16)	ENST00000420061
METRN	16	720032	720356	S18_peak_1268	TTS (ENST00000568223)	ENST00000568223
LMF1	16	861275	861458	S18_peak_1269	intron (ENST00000262301, intron 10 of 10)	ENST00000262301
NUP210L	1	154140413	154140578	S18_peak_127	intron (ENST00000368559, intron 4 of 39)	ENST00000368559
SSTR5-AS1	16	1046627	1046792	S18_peak_1270	Intergenic between ENST00000293894 and ENST00000569832	ENST00000569832
CACNA1H	16	1200842	1201007	S18_peak_1271	intron (ENST00000348261, intron 8 of 34)	ENST00000348261
LA16c-431H6.6	16	1670506	1670793	S18_peak_1272	intron (ENST00000454337, intron 19 of 22)	ENST00000454337
LINC00254	16	1885237	1885529	S18_peak_1273	Intergenic between ENST00000633813 and ENST00000570087	ENST00000633813
NDUFB10	16	1961229	1961394	S18_peak_1274	promoter-TSS (ENST00000268668)	ENST00000268668
TSC2	16	2067559	2067740	S18_peak_1275	intron (ENST00000219476, intron 16 of 41)	ENST00000219476
ABCA17P	16	2416216	2416381	S18_peak_1276	intron (ENST00000469908, intron 13 of 16)	ENST00000469908
CCNF	16	2441181	2441448	S18_peak_1277	intron (ENST00000397066, intron 8 of 16)	ENST00000397066
PDPK2P	16	2640709	2640933	S18_peak_1278	intron (ENST00000382326, intron 1 of 8)	ENST00000382326
SNORA3	16	2796726	2797002	S18_peak_1279	Intergenic between ENST00000408792 and ENST00000399677	ENST00000408792
DAP3P1	1	155587309	155587474	S18_peak_128	promoter-TSS (ENST00000441522)	ENST00000441522

HCFC1R1	16	3022947	3023378	S18_peak_1280	promoter-TSS (ENST00000248089)	ENST00000248089
HCFC1R1	16	3023665	3024152	S18_peak_1281	promoter-TSS (ENST00000248089)	ENST00000248089
RP11-473M20.5	16	3042643	3042876	S18_peak_1282	Intergenic between ENST00000382225 and ENST00000336577	ENST00000382225
ZNF213	16	3141165	3141385	S18_peak_1283	exon (ENST00000396878, exon 6 of 6)	ENST00000396878
LA16c-360H6.1	16	3294317	3294540	S18_peak_1284	Intergenic between ENST00000622977 and ENST00000396862	ENST00000622977
LA16c-360H6.1	16	3295722	3296130	S18_peak_1285	Intergenic between ENST00000622977 and ENST00000396862	ENST00000622977
RP11-433P17.1	16	3334856	3335025	S18_peak_1286	Intergenic between ENST00000572039 and ENST00000304936	ENST00000572039
NLRC3	16	3547947	3548112	S18_peak_1287	intron (ENST00000359128, intron 15 of 19)	ENST00000359128
TRAP1	16	3704570	3704795	S18_peak_1288	intron (ENST00000246957, intron 1 of 17)	ENST00000246957
RP11-462G12.2	16	3940161	3940326	S18_peak_1289	intron (ENST00000571302, intron 2 of 2)	ENST00000571302
DAP3	1	155737855	155738067	S18_peak_129	intron (ENST00000343043, intron 12 of 12)	ENST00000343043
RP11-462G12.1	16	3953861	3954120	S18_peak_1290	Intergenic between ENST00000576810 and ENST00000294016	ENST00000576810
ADCY9	16	3958192	3958357	S18_peak_1291	Intergenic between ENST00000576810 and ENST00000294016	ENST00000294016
HMOX2	16	4502531	4502745	S18_peak_1292	intron (ENST00000575120, intron 1 of 4)	ENST00000575120
C16orf96	16	4587725	4587902	S18_peak_1293	intron (ENST00000444310, intron 8 of 15)	ENST00000444310
MGRN1	16	4683714	4683879	S18_peak_1294	intron (ENST00000399577, intron 15 of 16)	ENST00000399577
MGRN1	16	4686720	4686895	S18_peak_1295	exon (ENST00000399577, exon 17 of 17)	ENST00000399577
NUDT16L1	16	4694205	4694397	S18_peak_1296	promoter-TSS (ENST00000586536)	ENST00000586536
UBN1	16	4881560	4881725	S18_peak_1297	exon (ENST00000262376, exon 18 of 18)	ENST00000262376
SEC14L5	16	4990772	4990937	S18_peak_1298	exon (ENST00000251170, exon 5 of 16)	ENST00000251170
SEC14L5	16	5007331	5007581	S18_peak_1299	exon (ENST00000251170, exon 13 of 16)	ENST00000251170
RP5-875O13.6	1	16567069	16567296	S18_peak_13	Intergenic between ENST00000438396 and ENST00000607700	ENST00000438396
MSTO2P	1	155739334	155739513	S18_peak_130	Intergenic between ENST00000432858 and ENST00000314835	ENST00000314835
ENPP7P14	16	5146381	5146710	S18_peak_1300	intron (ENST00000621765, intron 1 of 2)	ENST00000621765
RP11-420N3.3	16	5259759	5260006	S18_peak_1301	intron (ENST00000569895, intron 1 of 6)	ENST00000569895
RP11-420N3.3	16	5405635	5405811	S18_peak_1302	intron (ENST00000569895, intron 1 of 6)	ENST00000569895
RP11-420N3.3	16	5530707	5530970	S18_peak_1303	intron (ENST00000569895, intron 2 of 6)	ENST00000569895
RP11-420N3.3	16	5865608	5865896	S18_peak_1304	intron (ENST00000569895, intron 3 of 6)	ENST00000569895
RP11-420N3.3	16	5965574	5965739	S18_peak_1305	intron (ENST00000569895, intron 4 of 6)	ENST00000569895
RP11-420N3.3	16	6107600	6107828	S18_peak_1306	intron (ENST00000569895, intron 4 of 6)	ENST00000569895
RP11-420N3.3	16	6611288	6611453	S18_peak_1307	intron (ENST00000569895, intron 5 of 6)	ENST00000569895
RNU7-99P	16	6802270	6802435	S18_peak_1308	Intergenic between ENST00000516271 and ENST00000363999	ENST00000516271
RBFOX1	16	7319186	7319351	S18_peak_1309	Intergenic between ENST00000495947 and ENST00000355637	ENST00000355637
LMNA	1	156112023	156112306	S18_peak_131	Intergenic between ENST00000532414 and	ENST00000368300

					ENST00000368300	
RBFOX1	16	7505636	7505801	S18_peak_1310	intron (ENST00000355637, intron 1 of 13)	ENST00000355637
RBFOX1	16	7562318	7562518	S18_peak_1311	intron (ENST00000355637, intron 2 of 13)	ENST00000355637
RP11-483K5.2	16	8513526	8513691	S18_peak_1312	Intergenic between ENST00000567734 and ENST00000562516	ENST00000567734
METTL22	16	8629546	8629772	S18_peak_1313	intron (ENST00000381920, intron 3 of 10)	ENST00000381920
METTL22	16	8649838	8650003	S18_peak_1314	TTS (ENST00000381920)	ENST00000381920
ABAT	16	8779112	8779277	S18_peak_1315	intron (ENST00000268251, intron 14 of 15)	ENST00000268251
PMM2	16	8815256	8815421	S18_peak_1316	intron (ENST00000268261, intron 7 of 7)	ENST00000268261
RP11-77H9.8	16	9015580	9015803	S18_peak_1317	Intergenic between ENST00000564485 and ENST00000562893	ENST00000564485
RPL21P119	16	9184777	9184976	S18_peak_1318	Intergenic between ENST00000484462 and ENST00000564305	ENST00000484462
RPL21P119	16	9240659	9240824	S18_peak_1319	Intergenic between ENST00000484462 and ENST00000564305	ENST00000484462
ARHGEF11	1	156972456	156972621	S18_peak_132	intron (ENST00000368194, intron 7 of 40)	ENST00000368194
RP11-418I22.2	16	9525310	9525531	S18_peak_1320	Intergenic between ENST00000570038 and ENST00000634367	ENST00000570038
RP11-297M9.1	16	9679205	9679370	S18_peak_1321	Intergenic between ENST00000561538 and ENST00000636913	ENST00000561538
RP11-297M9.1	16	9696640	9696805	S18_peak_1322	Intergenic between ENST00000561538 and ENST00000636913	ENST00000561538
RP11-895K13.2	16	10037723	10037960	S18_peak_1323	TTS (ENST00000569218)	ENST00000569218
MTATP6P24	16	10729346	10729651	S18_peak_1324	Intergenic between ENST00000576835 and ENST00000283027	ENST00000576835
CLEC16A	16	10965407	10965572	S18_peak_1325	intron (ENST00000409790, intron 3 of 23)	ENST00000409790
CLEC16A	16	11077003	11077168	S18_peak_1326	intron (ENST00000409790, intron 19 of 23)	ENST00000409790
TNP2	16	11263471	11263733	S18_peak_1327	Intergenic between ENST00000332029 and ENST00000312693	ENST00000312693
Y_RNA	16	11326450	11326615	S18_peak_1328	Intergenic between ENST00000362798 and ENST00000572913	ENST00000362798
CTD-3088G3.8	16	11385820	11385985	S18_peak_1329	intron (ENST00000599216, intron 5 of 10)	ENST00000599216
ATP1A4	1	160179669	160179900	S18_peak_133	intron (ENST00000368081, intron 18 of 21)	ENST00000368081
LITAF	16	11573846	11574011	S18_peak_1330	intron (ENST00000339430, intron 1 of 3)	ENST00000339430
SNX29	16	12117871	12118117	S18_peak_1331	intron (ENST00000566228, intron 11 of 20)	ENST00000566228
SNX29	16	12529328	12529493	S18_peak_1332	intron (ENST00000566228, intron 20 of 20)	ENST00000566228
CPPED1	16	12763228	12763393	S18_peak_1333	intron (ENST00000381774, intron 2 of 3)	ENST00000381774
CPPED1	16	12764973	12765152	S18_peak_1334	intron (ENST00000381774, intron 2 of 3)	ENST00000381774
CPPED1	16	12800673	12800890	S18_peak_1335	intron (ENST00000381774, intron 1 of 3)	ENST00000381774
SHISA9	16	13012464	13012707	S18_peak_1336	Intergenic between ENST00000423335 and ENST00000571939	ENST00000423335
AC009134.1	16	13209716	13209948	S18_peak_1337	Intergenic between ENST00000571939 and ENST00000571619	ENST00000571939
U91319.1	16	13301147	13301312	S18_peak_1338	intron (ENST00000571619, intron 2 of 4)	ENST00000571619

U91319.1	16	13528891	13529160	S18_peak_1339	intron (ENST00000571619, intron 4 of 4)	ENST00000571619
RP11-122G18.11	1	161471109	161471345	S18_peak_134	Intergenic between ENST00000636824 and ENST00000271450	ENST00000636824
ERCC4	16	13864780	13865064	S18_peak_1340	Intergenic between ENST00000576944 and ENST00000311895	ENST00000311895
ERCC4	16	13893101	13893266	S18_peak_1341	Intergenic between ENST00000576944 and ENST00000311895	ENST00000311895
MIR193BHG	16	14338252	14338417	S18_peak_1342	Intergenic between ENST00000575792 and ENST00000566054	ENST00000575792
RP11-65J21.1	16	14385669	14385834	S18_peak_1343	Intergenic between ENST00000566054 and ENST00000572479	ENST00000566054
RP11-65J21.4	16	14414654	14414861	S18_peak_1344	intron (ENST00000572479, intron 1 of 2)	ENST00000572479
PDXDC1	16	15071533	15071698	S18_peak_1345	intron (ENST00000535621, intron 16 of 16)	ENST00000535621
MIR3180-4	16	15256828	15256993	S18_peak_1346	Intergenic between ENST00000552015 and ENST00000611162	ENST00000552015
RP11-72I8.2	16	15333630	15334048	S18_peak_1347	Intergenic between ENST00000552015 and ENST00000611162	ENST00000611162
RP11-72I8.2	16	15336928	15337163	S18_peak_1348	Intergenic between ENST00000552015 and ENST00000611162	ENST00000611162
C16orf45	16	15573154	15573420	S18_peak_1349	intron (ENST00000300006, intron 3 of 5)	ENST00000300006
UQCRBP2	1	162550831	162550996	S18_peak_135	Intergenic between ENST00000447827 and ENST00000563991	ENST00000447827
RPL15P20	16	15938381	15938657	S18_peak_1350	Intergenic between ENST00000482912 and ENST00000399410	ENST00000482912
RPL15P20	16	15938988	15939153	S18_peak_1351	Intergenic between ENST00000482912 and ENST00000399410	ENST00000482912
ABCC1	16	15959080	15959245	S18_peak_1352	intron (ENST00000399410, intron 1 of 30)	ENST00000399410
ABCC6	16	16182192	16182357	S18_peak_1353	intron (ENST00000205557, intron 17 of 30)	ENST00000205557
RP11-958N24.4	16	16342474	16342729	S18_peak_1354	Intergenic between ENST00000618304 and ENST00000620593	ENST00000620593
RP11-958N24.4	16	16348468	16348685	S18_peak_1355	Intergenic between ENST00000618304 and ENST00000620593	ENST00000620593
RP11-467M13.2	16	16396461	16396626	S18_peak_1356	intron (ENST00000564015, intron 1 of 1)	ENST00000564015
RP11-14N9.2	16	16599891	16600246	S18_peak_1357	Intergenic between ENST00000563361 and ENST00000567465	ENST00000567465
RP11-14N9.2	16	16604827	16605048	S18_peak_1358	Intergenic between ENST00000563361 and ENST00000567465	ENST00000567465
RP11-14N9.1	16	16733763	16733947	S18_peak_1359	Intergenic between ENST00000567465 and ENST00000624772	ENST00000624772
RP11-331H2.4	1	162852676	162852911	S18_peak_136	Intergenic between ENST00000451015 and ENST00000434394	ENST00000451015
RP11-419L9.1	16	17009883	17010048	S18_peak_1360	Intergenic between ENST00000564217 and ENST00000623235	ENST00000564217
RP11-567P19.1	16	17486965	17487130	S18_peak_1361	Intergenic between ENST00000618290 and	ENST00000618290

					ENST00000568026	
RPL7P47	16	17914323	17914488	S18_peak_1362	Intergenic between ENST00000564852 and ENST00000497299	ENST00000497299
CTA-481E9.4	16	18007336	18007501	S18_peak_1363	intron (ENST00000567304, intron 1 of 2)	ENST00000567304
CTA-481E9.4	16	18054242	18054407	S18_peak_1364	intron (ENST00000567304, intron 1 of 2)	ENST00000567304
RP11-1212A22.7	16	18317163	18317334	S18_peak_1365	TTS (ENST00000622756)	ENST00000622756
RP11-1212A22.1	16	18341582	18341825	S18_peak_1366	exon (ENST00000525846, exon 20 of 31)	ENST00000525846
RP11-1212A22.1	16	18351287	18351527	S18_peak_1367	promoter-TSS (ENST00000525846)	ENST00000525846
RP11-1212A22.8	16	18354208	18354385	S18_peak_1368	Intergenic between ENST00000539740 and ENST00000615944	ENST00000615944
RP11-1212A22.8	16	18360863	18361161	S18_peak_1369	Intergenic between ENST00000615944 and ENST00000532415	ENST00000615944
U3	1	163967701	163967898	S18_peak_137	Intergenic between ENST00000391236 and ENST00000435192	ENST00000391236
PKD1P5	16	18370102	18370276	S18_peak_1370	Intergenic between ENST00000615944 and ENST00000532415	ENST00000532415
RPS15A	16	18689412	18689677	S18_peak_1371	Intergenic between ENST00000565118 and ENST00000322989	ENST00000322989
SMG1	16	18889319	18889484	S18_peak_1372	exon (ENST00000446231, exon 6 of 63)	ENST00000446231
TMC7	16	19043636	19043816	S18_peak_1373	intron (ENST00000304381, intron 9 of 15)	ENST00000304381
COQ7	16	19076738	19076922	S18_peak_1374	intron (ENST00000321998, intron 4 of 5)	ENST00000321998
KNOP1	16	19702956	19703121	S18_peak_1375	exon (ENST00000219837, exon 5 of 5)	ENST00000219837
ACSM5	16	20427139	20427304	S18_peak_1376	intron (ENST00000331849, intron 6 of 13)	ENST00000331849
ACSM1	16	20663028	20663193	S18_peak_1377	intron (ENST00000307493, intron 5 of 12)	ENST00000307493
ANKS4B	16	21250427	21250824	S18_peak_1378	exon (ENST00000311620, exon 2 of 2)	ENST00000311620
CTB-31N19.2	16	21547171	21547336	S18_peak_1379	intron (ENST00000550637, intron 3 of 4)	ENST00000550637
RP11-280O1.2	1	165510252	165510417	S18_peak_138	intron (ENST00000416424, intron 3 of 5)	ENST00000416424
SCARNA6	16	21567527	21567695	S18_peak_1380	Intergenic between ENST00000605544 and ENST00000516989	ENST00000516989
C16orf52	16	22074211	22074446	S18_peak_1381	intron (ENST00000542527, intron 1 of 2)	ENST00000542527
VWA3A	16	22094155	22094320	S18_peak_1382	promoter-TSS (ENST00000389398)	ENST00000389398
RRN3P3	16	22433089	22433254	S18_peak_1383	intron (ENST00000551766, intron 3 of 6)	ENST00000551766
SMG1P1	16	22443709	22444014	S18_peak_1384	Intergenic between ENST00000549207 and ENST00000337787	ENST00000337787
RP11-368J21.4	16	22514322	22514487	S18_peak_1385	Intergenic between ENST00000337787 and ENST00000610646	ENST00000610646
RP11-368J21.3	16	22553665	22553844	S18_peak_1386	intron (ENST00000550753, intron 2 of 8)	ENST00000550753
RP11-105C19.1	16	22663515	22663728	S18_peak_1387	Intergenic between ENST00000566098 and ENST00000636354	ENST00000566098
RP11-105C19.1	16	22701175	22701340	S18_peak_1388	Intergenic between ENST00000566098 and ENST00000636354	ENST00000566098
HS3ST2	16	22944625	22944790	S18_peak_1389	Intergenic between ENST00000261374 and ENST00000567395	ENST00000261374
ADCY10	1	167816909	167817088	S18_peak_139	intron (ENST00000545172, intron 28 of 29)	ENST00000545172

SCNN1B	16	23381390	23381654	S18_peak_1390	TTS (ENST00000343070)	ENST00000343070
COG7	16	23400723	23400888	S18_peak_1391	intron (ENST00000307149, intron 13 of 16)	ENST00000307149
KDM8	16	27212370	27212601	S18_peak_1392	intron (ENST00000286096, intron 2 of 7)	ENST00000286096
FLJ21408	16	27288782	27288947	S18_peak_1393	exon (ENST00000566854, exon 4 of 5)	ENST00000566854
GSG1L	16	28034322	28034487	S18_peak_1394	intron (ENST00000447459, intron 1 of 6)	ENST00000447459
RP11-57A19.4	16	28707318	28707507	S18_peak_1395	intron (ENST00000562217, intron 1 of 2)	ENST00000562217
RP11-57A19.4	16	28723338	28723667	S18_peak_1396	intron (ENST00000562217, intron 1 of 2)	ENST00000562217
RP11-22P6.2	16	28854857	28855090	S18_peak_1397	Intergenic between ENST00000313511 and ENST00000567731	ENST00000567731
RABEP2	16	28907865	28908030	S18_peak_1398	intron (ENST00000358201, intron 8 of 12)	ENST00000358201
RP11-264B17.5	16	28992563	28992878	S18_peak_1399	Intergenic between ENST00000561471 and ENST00000424293	ENST00000561471
RP5-1182A14.5	1	16606428	16606596	S18_peak_14	Intergenic between ENST00000438396 and ENST00000607700	ENST00000607700
DCAF6	1	167978390	167978636	S18_peak_140	intron (ENST00000367843, intron 4 of 19)	ENST00000367843
RP11-231C14.7	16	29298912	29299077	S18_peak_1400	intron (ENST00000604430, intron 1 of 10)	ENST00000604430
RP11-231C14.5	16	29478707	29478887	S18_peak_1401	promoter-TSS (ENST00000546408)	ENST00000546408
RP11-231C14.5	16	29482914	29483079	S18_peak_1402	TTS (ENST00000546408)	ENST00000546408
RP11-231C14.5	16	29499623	29500033	S18_peak_1403	Intergenic between ENST00000546408 and ENST00000566127	ENST00000546408
CA5AP1	16	29621262	29621693	S18_peak_1404	intron (ENST00000568175, intron 4 of 4)	ENST00000568175
HIRIP3	16	29993633	29993844	S18_peak_1405	exon (ENST00000279392, exon 5 of 7)	ENST00000279392
INO80E	16	30003846	30004062	S18_peak_1406	intron (ENST00000563197, intron 6 of 6)	ENST00000563197
BOLA2B	16	30191811	30191976	S18_peak_1407	Intergenic between ENST00000567153 and ENST00000305321	ENST00000305321
ITGAL	16	30519226	30519430	S18_peak_1408	intron (ENST00000356798, intron 29 of 30)	ENST00000356798
SRCAP	16	30719254	30719419	S18_peak_1409	intron (ENST00000262518, intron 18 of 33)	ENST00000262518
RP5-968D22.3	1	168488270	168488435	S18_peak_141	intron (ENST00000636459, intron 2 of 8)	ENST00000636459
SETD1A	16	30974968	30975239	S18_peak_1410	intron (ENST00000262519, intron 13 of 18)	ENST00000262519
PYDC1	16	31233408	31233653	S18_peak_1411	Intergenic between ENST00000302964 and ENST00000544665	ENST00000302964
ITGAM	16	31342778	31342943	S18_peak_1412	Intergenic between ENST00000544665 and ENST00000268296	ENST00000544665
HERC2P4	16	32107075	32107240	S18_peak_1413	intron (ENST00000564145, intron 4 of 4)	ENST00000564145
RP11-19N8.2	16	32949976	32950203	S18_peak_1414	Intergenic between ENST00000563896 and ENST00000567619	ENST00000567619
RP11-19N8.2	16	32999269	32999573	S18_peak_1415	intron (ENST00000567619, intron 1 of 2)	ENST00000567619
RP11-19N8.4	16	33065772	33065993	S18_peak_1416	intron (ENST00000561541, intron 1 of 1)	ENST00000561541
HERC2P8	16	33116361	33116681	S18_peak_1417	promoter-TSS (ENST00000564334)	ENST00000564334
ABHD17AP9	16	33140912	33141077	S18_peak_1418	promoter-TSS (ENST00000561925)	ENST00000561925
RP11-23E10.4	16	33563968	33564149	S18_peak_1419	promoter-TSS (ENST00000568520)	ENST00000568520
RP4-738P11.3	1	168548603	168548822	S18_peak_142	Intergenic between ENST00000449688 and ENST00000367818	ENST00000449688
RP11-104C4.6	16	33741519	33741711	S18_peak_1420	intron (ENST00000634254, intron 9 of 18)	ENST00000634254

IGHV3OR16-12	16	33798243	33798463	S18_peak_1421	Intergenic between ENST00000563187 and ENST00000570121	ENST00000570121
RP11-812E19.10	16	33880746	33880981	S18_peak_1422	Intergenic between ENST00000566360 and ENST00000562773	ENST00000566360
CTD-2522B17.8	16	34122622	34122787	S18_peak_1423	Intergenic between ENST00000574771 and ENST00000566406	ENST00000574771
BCLAF1P2	16	34256920	34257165	S18_peak_1424	Intergenic between ENST00000363564 and ENST00000624956	ENST00000624956
BCLAF1P2	16	34273970	34274290	S18_peak_1425	Intergenic between ENST00000624956 and ENST00000624300	ENST00000624956
BCLAF1P2	16	34280718	34280896	S18_peak_1426	Intergenic between ENST00000624956 and ENST00000624300	ENST00000624956
CTD-2144E22.9	16	34826676	34826841	S18_peak_1428	Intergenic between ENST00000624956 and ENST00000624300	ENST00000624300
PPP1R1AP2	16	38267116	38267297	S18_peak_1429	Intergenic between ENST00000561752 and ENST00000566201	ENST00000561752
PRRX1	1	170730353	170730573	S18_peak_143	intron (ENST00000367760, intron 4 of 4)	ENST00000367760
PPP1R1AP2	16	38268497	38268727	S18_peak_1430	Intergenic between ENST00000561752 and ENST00000566201	ENST00000561752
N4BP1	16	48527754	48527966	S18_peak_1432	Intergenic between ENST00000564020 and ENST00000262384	ENST00000262384
N4BP1	16	48588459	48588624	S18_peak_1433	intron (ENST00000262384, intron 1 of 6)	ENST00000262384
RP11-883G14.4	16	51074023	51074191	S18_peak_1434	Intergenic between ENST00000569009 and ENST00000563903	ENST00000569009
RP11-132F7.2	16	52368996	52369161	S18_peak_1435	Intergenic between ENST00000628396 and ENST00000624321	ENST00000624321
RNU6-269P	16	58340793	58340958	S18_peak_1436	Intergenic between ENST00000219299 and ENST00000391077	ENST00000391077
PLA2G15	16	68256644	68257059	S18_peak_1437	intron (ENST00000219345, intron 5 of 5)	ENST00000219345
RP11-140H17.1	16	69490421	69490586	S18_peak_1438	Intergenic between ENST00000567834 and ENST00000411177	ENST00000567834
PDXDC2P	16	69986566	69986731	S18_peak_1439	intron (ENST00000531894, intron 20 of 25)	ENST00000531894
FAM163A	1	179785286	179785555	S18_peak_144	intron (ENST00000341785, intron 1 of 4)	ENST00000341785
PHLPP2	16	71696776	71696941	S18_peak_1440	intron (ENST00000568954, intron 3 of 18)	ENST00000568954
CLEC18B	16	74404348	74404513	S18_peak_1441	Intergenic between ENST00000614366 and ENST00000339953	ENST00000339953
RP11-525K10.1	16	80148566	80148731	S18_peak_1442	intron (ENST00000567851, intron 2 of 3)	ENST00000567851
RN7SKP190	16	82301733	82301898	S18_peak_1443	Intergenic between ENST00000516079 and ENST00000563948	ENST00000516079
RP11-542M13.2	16	85980730	85980936	S18_peak_1444	Intergenic between ENST00000598933 and ENST00000599411	ENST00000599411
KLHDC4	16	87729207	87729372	S18_peak_1445	intron (ENST00000353170, intron 4 of 9)	ENST00000353170
LA16c-444G7.1	16	88269730	88269895	S18_peak_1446	intron (ENST00000563190, intron 1 of 3)	ENST00000563190
RP11-1228E12.1	17	73891	74072	S18_peak_1447	intron (ENST00000623180, intron 2 of 4)	ENST00000623180

RP11-1228E12.2	17	99403	99568	S18_peak_1448	intron (ENST00000624936, intron 5 of 7)	ENST00000624936
SCGB1C2	17	139134	139331	S18_peak_1449	TTS (ENST00000595228)	ENST00000595228
XPR1	1	180874138	180874315	S18_peak_145	intron (ENST00000367590, intron 13 of 14)	ENST00000367590
DOC2B	17	158330	158609	S18_peak_1450	intron (ENST00000613549, intron 5 of 8)	ENST00000613549
TUSC5	17	1289553	1289920	S18_peak_1451	intron (ENST00000333813, intron 1 of 2)	ENST00000333813
INPP5K	17	1494296	1494461	S18_peak_1452	TTS (ENST00000421807)	ENST00000421807
SLC43A2	17	1599098	1599278	S18_peak_1453	intron (ENST00000301335, intron 5 of 13)	ENST00000301335
RTN4RL1	17	1964517	1964714	S18_peak_1454	intron (ENST00000331238, intron 1 of 1)	ENST00000331238
RN7SL33P	17	2538739	2538904	S18_peak_1455	Intergenic between ENST00000624486 and ENST00000462543	ENST00000462543
PAFAH1B1	17	2668504	2668748	S18_peak_1456	intron (ENST00000397195, intron 5 of 10)	ENST00000397195
RAP1GAP2	17	2811721	2811886	S18_peak_1457	intron (ENST00000254695, intron 2 of 24)	ENST00000254695
OR1E3	17	3114264	3114482	S18_peak_1458	Intergenic between ENST00000331459 and ENST00000575608	ENST00000575608
SPATA22	17	3456920	3457085	S18_peak_1459	intron (ENST00000397168, intron 5 of 8)	ENST00000397168
IER5	1	181057729	181057966	S18_peak_146	Intergenic between ENST00000258301 and ENST00000367577	ENST00000367577
CTNS	17	3642929	3643094	S18_peak_1460	intron (ENST00000046640, intron 3 of 11)	ENST00000046640
PITPNM3	17	6511395	6511560	S18_peak_1461	intron (ENST00000421306, intron 2 of 18)	ENST00000421306
CTC-281F24.3	17	6657360	6657525	S18_peak_1462	promoter-TSS (ENST00000634558)	ENST00000634558
ACAP1	17	7347538	7347784	S18_peak_1463	intron (ENST00000158762, intron 14 of 21)	ENST00000158762
CNTROB	17	7947447	7947686	S18_peak_1464	intron (ENST00000380262, intron 13 of 18)	ENST00000380262
RP11-849F2.5	17	8355911	8356189	S18_peak_1465	Intergenic between ENST00000328248 and ENST00000583963	ENST00000583963
MFSD6L	17	8827553	8827754	S18_peak_1466	Intergenic between ENST00000329805 and ENST00000585297	ENST00000329805
MYHAS	17	10542134	10542334	S18_peak_1467	intron (ENST00000587182, intron 2 of 10)	ENST00000587182
RN7SL601P	17	11127688	11127853	S18_peak_1468	Intergenic between ENST00000585243 and ENST00000471221	ENST00000471221
RN7SL601P	17	11188724	11188889	S18_peak_1469	Intergenic between ENST00000471221 and ENST00000432116	ENST00000471221
C1orf21	1	184418320	184418485	S18_peak_147	intron (ENST00000235307, intron 1 of 5)	ENST00000235307
DNAH9	17	11875273	11875438	S18_peak_1470	intron (ENST00000262442, intron 53 of 68)	ENST00000262442
MYOCD	17	12677402	12677567	S18_peak_1471	intron (ENST00000425538, intron 1 of 13)	ENST00000425538
MYOCD	17	12723379	12723580	S18_peak_1472	intron (ENST00000425538, intron 5 of 13)	ENST00000425538
RP11-1090M7.1	17	12770457	12770622	S18_peak_1473	intron (ENST00000584772, intron 1 of 1)	ENST00000584772
PIGL	17	16244636	16244930	S18_peak_1474	intron (ENST00000225609, intron 2 of 6)	ENST00000225609
PIGL	17	16249304	16249594	S18_peak_1475	intron (ENST00000225609, intron 2 of 6)	ENST00000225609
TRPV2	17	16436451	16436616	S18_peak_1476	intron (ENST00000338560, intron 14 of 14)	ENST00000338560
LRRC75A-AS1	17	16443678	16444053	S18_peak_1477	intron (ENST00000584177, intron 4 of 4)	ENST00000584177
RP11-219A15.1	17	16735426	16735591	S18_peak_1478	exon (ENST00000448331, exon 12 of 26)	ENST00000448331
RP11-219A15.1	17	16788155	16788320	S18_peak_1479	intron (ENST00000448331, intron 21 of 25)	ENST00000448331
HMCN1	1	185845314	185845521	S18_peak_148	intron (ENST00000271588, intron 1 of 106)	ENST00000271588
SMCR2	17	17652043	17652213	S18_peak_1480	Intergenic between ENST00000623335 and	ENST00000456090

					ENST00000456090	
RAI1	17	17732616	17732845	S18_peak_1481	intron (ENST00000353383, intron 2 of 5)	ENST00000353383
DRC3	17	17981362	17981637	S18_peak_1482	intron (ENST00000399182, intron 3 of 12)	ENST00000399182
TOP3A	17	18303458	18303867	S18_peak_1483	intron (ENST00000321105, intron 5 of 18)	ENST00000321105
TBC1D3P4	17	18397414	18397658	S18_peak_1484	intron (ENST00000338790, intron 6 of 11)	ENST00000338790
KRT16P4	17	18465210	18465450	S18_peak_1485	intron (ENST00000580052, intron 1 of 1)	ENST00000580052
CCDC144B	17	18581292	18581457	S18_peak_1486	intron (ENST00000618081, intron 11 of 16)	ENST00000618081
TRIM16L	17	18729299	18729576	S18_peak_1487	intron (ENST00000395671, intron 2 of 4)	ENST00000395671
AC107982.4	17	18823238	18823478	S18_peak_1488	Intergenic between ENST00000584957 and ENST00000507171	ENST00000507171
SLC5A10	17	18955803	18956031	S18_peak_1489	intron (ENST00000317977, intron 1 of 14)	ENST00000317977
GS1-304P7.2	1	186556591	186556756	S18_peak_149	Intergenic between ENST00000609032 and ENST00000456921	ENST00000456921
SLC5A10	17	18958721	18958886	S18_peak_1490	intron (ENST00000317977, intron 2 of 14)	ENST00000317977
RP11-160E2.17	17	19057050	19057220	S18_peak_1491	Intergenic between ENST00000625108 and ENST00000442355	ENST00000442355
RP11-160E2.17	17	19062218	19062500	S18_peak_1492	Intergenic between ENST00000625108 and ENST00000442355	ENST00000442355
RP11-160E2.16	17	19207371	19207564	S18_peak_1493	intron (ENST00000428348, intron 2 of 2)	ENST00000428348
RP11-744A16.4	17	19222393	19222647	S18_peak_1494	exon (ENST00000628484, exon 3 of 3)	ENST00000628484
EPN2	17	19232744	19233138	S18_peak_1495	Intergenic between ENST00000628484 and ENST00000314728	ENST00000314728
EPN2	17	19262051	19262262	S18_peak_1496	intron (ENST00000314728, intron 1 of 10)	ENST00000314728
B9D1	17	19338641	19338806	S18_peak_1497	intron (ENST00000575403, intron 6 of 6)	ENST00000575403
ULK2	17	19794023	19794222	S18_peak_1498	intron (ENST00000361658, intron 20 of 27)	ENST00000361658
AKAP10	17	19942883	19943048	S18_peak_1499	intron (ENST00000225737, intron 5 of 14)	ENST00000225737
RP1-163M9.8	1	16666347	16666549	S18_peak_15	intron (ENST00000633763, intron 2 of 2)	ENST00000633763
LINC01036	1	187354251	187354494	S18_peak_150	intron (ENST00000458683, intron 3 of 3)	ENST00000458683
RP11-434D2.10	17	20541191	20541403	S18_peak_1500	Intergenic between ENST00000584274 and ENST00000578659	ENST00000578659
MAP2K3	17	21331700	21331865	S18_peak_1501	Intergenic between ENST00000342679 and ENST00000583088	ENST00000342679
CH17-53B9.2	17	21634406	21634626	S18_peak_1502	intron (ENST00000618784, intron 2 of 2)	ENST00000618784
AC144838.3	17	21664588	21664753	S18_peak_1503	Intergenic between ENST00000584107 and ENST00000598588	ENST00000584107
KCNJ18	17	21709462	21709733	S18_peak_1504	Intergenic between ENST00000567955 and ENST00000637164	ENST00000567955
RP11-1109M24.5	17	22286384	22286553	S18_peak_1505	exon (ENST00000578881, exon 3 of 3)	ENST00000578881
RP11-846F4.11	17	23225215	23225380	S18_peak_1506	Intergenic between ENST00000578745 and ENST00000580677	ENST00000578745
RP11-720N19.2	17	27480467	27480632	S18_peak_1507	Intergenic between ENST00000583643 and ENST00000583159	ENST00000583643
RP11-720N19.1	17	27501681	27501929	S18_peak_1508	Intergenic between ENST00000583643 and ENST00000583159	ENST00000583159

RP1-66C13.4	17	27830430	27830755	S18_peak_1509	intron (ENST00000582441, intron 3 of 4)	ENST00000582441
RP5-1011O1.2	1	192560899	192561064	S18_peak_151	intron (ENST00000434300, intron 1 of 1)	ENST00000434300
RP11-218F4.1	17	27942616	27942781	S18_peak_1510	intron (ENST00000567574, intron 1 of 3)	ENST00000567574
CTD-2008P7.9	17	28238621	28238786	S18_peak_1511	Intergenic between ENST00000592016 and ENST00000579045	ENST00000592016
KRT18P55	17	28292003	28292247	S18_peak_1512	intron (ENST00000577198, intron 2 of 2)	ENST00000577198
TNFAIP1	17	28341717	28341882	S18_peak_1513	intron (ENST00000226225, intron 5 of 6)	ENST00000226225
H3F3BP2	17	28420606	28420815	S18_peak_1514	Intergenic between ENST00000583907 and ENST00000579654	ENST00000583907
CRYBA1	17	29226262	29226464	S18_peak_1515	Intergenic between ENST00000580812 and ENST00000225387	ENST00000225387
CRYBA1	17	29233545	29233710	S18_peak_1516	Intergenic between ENST00000580812 and ENST00000225387	ENST00000225387
CRYBA1	17	29246439	29246604	S18_peak_1517	Intergenic between ENST00000580812 and ENST00000225387	ENST00000225387
TAOK1	17	29492718	29492960	S18_peak_1518	intron (ENST00000261716, intron 10 of 19)	ENST00000261716
SSH2	17	29770247	29770412	S18_peak_1519	intron (ENST00000269033, intron 2 of 14)	ENST00000269033
LINC01031	1	193369983	193370185	S18_peak_152	Intergenic between ENST00000420807 and ENST00000448412	ENST00000420807
AC005562.1	17	30652483	30652722	S18_peak_1520	intron (ENST00000578265, intron 4 of 4)	ENST00000578265
RP11-848P1.3	17	30964430	30964595	S18_peak_1521	TTS (ENST00000579561)	ENST00000579561
RNF135	17	30972684	30972958	S18_peak_1522	intron (ENST00000328381, intron 1 of 4)	ENST00000328381
NF1	17	31175896	31176169	S18_peak_1523	intron (ENST00000358273, intron 5 of 57)	ENST00000358273
NF1	17	31210109	31210319	S18_peak_1524	intron (ENST00000358273, intron 12 of 57)	ENST00000358273
RP11-227G15.8	17	32414015	32414256	S18_peak_1525	Intergenic between ENST00000584721 and ENST00000625127	ENST00000584721
RP11-227G15.12	17	32435231	32435396	S18_peak_1526	intron (ENST00000625127, intron 2 of 2)	ENST00000625127
MYO1D	17	32664782	32664947	S18_peak_1527	intron (ENST00000318217, intron 16 of 21)	ENST00000318217
ASIC2	17	33167484	33167667	S18_peak_1528	intron (ENST00000225823, intron 1 of 9)	ENST00000225823
RP11-642M2.1	17	34818021	34818186	S18_peak_1529	Intergenic between ENST00000577556 and ENST00000579327	ENST00000579327
RP11-476B1.1	1	195763300	195763546	S18_peak_153	Intergenic between ENST00000446021 and ENST00000430519	ENST00000430519
RP11-47L3.1	17	35320907	35321072	S18_peak_1530	intron (ENST00000591886, intron 1 of 2)	ENST00000591886
AC015849.2	17	35881126	35881291	S18_peak_1531	intron (ENST00000605548, intron 1 of 4)	ENST00000605548
CTB-91J4.1	17	36117924	36118209	S18_peak_1532	promoter-TSS (ENST00000617914)	ENST00000617914
AC131056.3	17	36181043	36181213	S18_peak_1533	Intergenic between ENST00000613949 and ENST00000615128	ENST00000615128
TBC1D3G	17	36337617	36337953	S18_peak_1534	Intergenic between ENST00000569055 and ENST00000610350	ENST00000569055
TBC1D3F	17	36428203	36428369	S18_peak_1535	TTS (ENST00000620210)	ENST00000620210
TBC1D3F	17	36442778	36442943	S18_peak_1536	Intergenic between ENST00000620210 and ENST00000620719	ENST00000620210
RP11-445F12.1	17	36825992	36826245	S18_peak_1537	Intergenic between ENST00000610398 and	ENST00000616341

					ENST00000616341	
ACACA	17	37116676	37116891	S18_peak_1538	intron (ENST00000614428, intron 50 of 55)	ENST00000614428
ACACA	17	37310504	37310848	S18_peak_1539	intron (ENST00000614428, intron 3 of 55)	ENST00000614428
KCNT2	1	196538635	196538800	S18_peak_154	intron (ENST00000367433, intron 1 of 26)	ENST00000367433
ACACA	17	37320244	37320475	S18_peak_1540	intron (ENST00000614428, intron 3 of 55)	ENST00000614428
DUSP14	17	37482848	37483013	S18_peak_1541	Intergenic between ENST00000612843 and ENST00000617516	ENST00000617516
SYNRG	17	37537284	37537569	S18_peak_1542	intron (ENST00000612223, intron 18 of 21)	ENST00000612223
AC091199.1	17	37792402	37792662	S18_peak_1543	Intergenic between ENST00000613727 and ENST00000615743	ENST00000615743
5S_rRNA	17	37950172	37950337	S18_peak_1544	Intergenic between ENST00000618379 and ENST00000616101	ENST00000618379
5S_rRNA	17	38161285	38161450	S18_peak_1545	Intergenic between ENST00000619782 and ENST00000620215	ENST00000619782
TBC1D3	17	38163461	38163626	S18_peak_1546	Intergenic between ENST00000619782 and ENST00000620215	ENST00000620215
MRPL45	17	38271784	38271949	S18_peak_1547	Intergenic between ENST00000614695 and ENST00000613675	ENST00000613675
SOCS7	17	38349231	38349586	S18_peak_1548	Intergenic between ENST00000613675 and ENST00000612932	ENST00000612932
FBXL20	17	39292969	39293134	S18_peak_1549	intron (ENST00000264658, intron 6 of 14)	ENST00000264658
ATP6V1G3	1	198427183	198427348	S18_peak_155	Intergenic between ENST00000456678 and ENST00000309309	ENST00000309309
CASC3	17	40144040	40144205	S18_peak_1550	intron (ENST00000264645, intron 3 of 13)	ENST00000264645
WIPF2	17	40275587	40275759	S18_peak_1551	intron (ENST00000323571, intron 6 of 7)	ENST00000323571
CTD-2267D19.4	17	40368391	40368556	S18_peak_1552	promoter-TSS (ENST00000583752)	ENST00000583752
KRT26	17	40768915	40769096	S18_peak_1553	exon (ENST00000335552, exon 6 of 8)	ENST00000335552
KRTAP4-6	17	41142676	41142841	S18_peak_1554	Intergenic between ENST00000345847 and ENST00000343246	ENST00000345847
STAT5B	17	42198839	42199094	S18_peak_1555	TTS (ENST00000293328)	ENST00000293328
ATP6V0A1	17	42465330	42465495	S18_peak_1556	intron (ENST00000264649, intron 2 of 20)	ENST00000264649
ATP6V0A1	17	42504151	42504334	S18_peak_1557	intron (ENST00000264649, intron 17 of 20)	ENST00000264649
SNORA40	17	42942449	42942735	S18_peak_1558	Intergenic between ENST00000390847 and ENST00000360221	ENST00000390847
BRCA1	17	43112190	43112355	S18_peak_1559	intron (ENST00000357654, intron 3 of 22)	ENST00000357654
NR5A2	1	200068457	200068640	S18_peak_156	intron (ENST00000367362, intron 5 of 7)	ENST00000367362
LINC00854	17	43238109	43238330	S18_peak_1560	intron (ENST00000594691, intron 1 of 3)	ENST00000594691
LINC00854	17	43288495	43288666	S18_peak_1561	intron (ENST00000594691, intron 1 of 3)	ENST00000594691
LINC00910	17	43375832	43375997	S18_peak_1562	intron (ENST00000341011, intron 4 of 5)	ENST00000341011
RNU6-406P	17	43431551	43431894	S18_peak_1563	Intergenic between ENST00000515954 and ENST00000516470	ENST00000516470
MPP3	17	43820006	43820171	S18_peak_1564	intron (ENST00000398389, intron 11 of 19)	ENST00000398389
HDAC5	17	44122089	44122367	S18_peak_1565	promoter-TSS (ENST00000225983)	ENST00000225983
GPATCH8	17	44402809	44403125	S18_peak_1566	intron (ENST00000591680, intron 7 of 7)	ENST00000591680

GPATCH8	17	44403974	44404173	S18_peak_1567	intron (ENST00000591680, intron 7 of 7)	ENST00000591680
PLEKHM1	17	45447191	45447356	S18_peak_1568	intron (ENST00000430334, intron 8 of 11)	ENST00000430334
LRRC37A4P	17	45498145	45498310	S18_peak_1569	Intergenic between ENST00000433601 and ENST00000579913	ENST00000579913
PPFIA4	1	203030455	203030620	S18_peak_157	Intergenic between ENST00000367242 and ENST00000447715	ENST00000447715
LRRC37A4P	17	45547227	45547420	S18_peak_1570	intron (ENST00000581296, intron 1 of 13)	ENST00000581296
RP11-798G7.4	17	45570091	45570256	S18_peak_1571	Intergenic between ENST00000579701 and ENST00000580842	ENST00000579701
ARL17B	17	46349037	46349259	S18_peak_1572	intron (ENST00000434041, intron 3 of 4)	ENST00000434041
NSFP1	17	46493684	46493901	S18_peak_1573	Intergenic between ENST00000570034 and ENST00000582765	ENST00000570034
RP11-995C19.2	17	46503790	46504010	S18_peak_1574	promoter-TSS (ENST00000582765)	ENST00000582765
ARL17A	17	46536282	46536559	S18_peak_1575	intron (ENST00000445552, intron 3 of 4)	ENST00000445552
ARL17A	17	46548622	46548889	S18_peak_1576	intron (ENST00000445552, intron 3 of 4)	ENST00000445552
ARL17A	17	46571820	46572040	S18_peak_1577	intron (ENST00000445552, intron 2 of 4)	ENST00000445552
RP11-63A1.1	17	46920230	46920470	S18_peak_1578	intron (ENST00000572349, intron 1 of 2)	ENST00000572349
EFCAB13	17	47364498	47364703	S18_peak_1579	intron (ENST00000331493, intron 10 of 24)	ENST00000331493
LARP7P1	1	203434388	203434565	S18_peak_158	Intergenic between ENST00000605576 and ENST00000343110	ENST00000605576
NPEPPS	17	47527004	47527169	S18_peak_1580	Intergenic between ENST00000573318 and ENST00000322157	ENST00000322157
NPEPPS	17	47584931	47585157	S18_peak_1581	intron (ENST00000322157, intron 5 of 22)	ENST00000322157
TBKBP1	17	47712361	47712551	S18_peak_1582	TTS (ENST00000361722)	ENST00000361722
RP11-6N17.10	17	47987670	47987835	S18_peak_1583	intron (ENST00000578239, intron 1 of 1)	ENST00000578239
TTLL6	17	48779253	48779418	S18_peak_1584	intron (ENST00000433608, intron 6 of 8)	ENST00000433608
B4GALNT2	17	49158478	49158727	S18_peak_1585	intron (ENST00000504681, intron 5 of 10)	ENST00000504681
ZNF652	17	49330518	49330750	S18_peak_1586	intron (ENST00000362063, intron 1 of 5)	ENST00000362063
RP11-1094H24.3	17	50009648	50009813	S18_peak_1587	intron (ENST00000511867, intron 1 of 1)	ENST00000511867
CACNA1G	17	50621109	50621274	S18_peak_1588	intron (ENST00000505165, intron 33 of 36)	ENST00000505165
MIR8059	17	50774129	50774294	S18_peak_1589	Intergenic between ENST00000620427 and ENST00000510589	ENST00000620427
LINC00303	1	204042857	204043022	S18_peak_159	Intergenic between ENST00000427799 and ENST00000367202	ENST00000427799
TOB1-AS1	17	50888190	50888355	S18_peak_1590	intron (ENST00000523470, intron 1 of 1)	ENST00000523470
SPAG9	17	50962686	50962851	S18_peak_1591	exon (ENST00000262013, exon 30 of 30)	ENST00000262013
SPAG9	17	51072992	51073157	S18_peak_1592	intron (ENST00000262013, intron 2 of 29)	ENST00000262013
RP11-421E14.2	17	51234675	51234919	S18_peak_1593	Intergenic between ENST00000624148 and ENST00000581917	ENST00000581917
CA10	17	51940483	51940824	S18_peak_1594	intron (ENST00000442502, intron 3 of 9)	ENST00000442502
RP11-429O1.1	17	52271007	52271172	S18_peak_1595	Intergenic between ENST00000625000 and ENST00000572848	ENST00000572848
RP11-429O1.1	17	52401838	52402003	S18_peak_1596	intron (ENST00000572848, intron 1 of 3)	ENST00000572848
RP11-429O1.1	17	52667843	52668008	S18_peak_1597	Intergenic between ENST00000572848 and	ENST00000572848

					ENST00000412360	
AC102948.2	17	52936088	52936253	S18_peak_1598	Intergenic between ENST00000412360 and ENST00000623702	ENST00000412360
MTCO1P40	17	53196329	53196494	S18_peak_1599	Intergenic between ENST00000574371 and ENST00000571086	ENST00000574371
FAM231C	1	16724298	16724525	S18_peak_16	Intergenic between ENST00000492551 and ENST00000624031	ENST00000624031
RP11-430C7.4	1	204604641	204604806	S18_peak_160	promoter-TSS (ENST00000453895)	ENST00000453895
ISCA1P3	17	54555856	54556021	S18_peak_1600	Intergenic between ENST00000577104 and ENST00000576282	ENST00000577104
RP11-372K20.1	17	54708451	54708686	S18_peak_1601	Intergenic between ENST00000576282 and ENST00000576706	ENST00000576282
HLF	17	55217794	55217999	S18_peak_1602	Intergenic between ENST00000398391 and ENST00000226067	ENST00000226067
RP11-515E23.1	17	55453950	55454157	S18_peak_1603	intron (ENST00000636752, intron 4 of 4)	ENST00000636752
CTD-2033D24.2	17	55530376	55530541	S18_peak_1604	intron (ENST00000455347, intron 1 of 3)	ENST00000455347
ANKFN1	17	56143858	56144104	S18_peak_1605	Intergenic between ENST00000571972 and ENST00000318698	ENST00000318698
RP11-670E13.3	17	56882263	56882485	S18_peak_1606	Intergenic between ENST00000391167 and ENST00000571053	ENST00000571053
COIL	17	56957763	56957928	S18_peak_1607	intron (ENST00000240316, intron 1 of 6)	ENST00000240316
MSI2	17	57386630	57386795	S18_peak_1608	intron (ENST00000284073, intron 5 of 13)	ENST00000284073
MSI2	17	57585862	57586027	S18_peak_1609	intron (ENST00000284073, intron 7 of 13)	ENST00000284073
RAB7B	1	205976236	205976401	S18_peak_161	TTS (ENST00000617070)	ENST00000617070
MKS1	17	58227746	58227934	S18_peak_1610	Intergenic between ENST00000537529 and ENST00000262290	ENST00000537529
SEPT4-AS1	17	58528042	58528226	S18_peak_1611	intron (ENST00000580589, intron 2 of 4)	ENST00000580589
RP11-579A4.1	17	58741827	58741992	S18_peak_1612	Intergenic between ENST00000337432 and ENST00000621624	ENST00000621624
PPM1E	17	58801854	58802055	S18_peak_1613	intron (ENST00000308249, intron 1 of 6)	ENST00000308249
PPM1E	17	58953315	58953480	S18_peak_1614	intron (ENST00000308249, intron 1 of 6)	ENST00000308249
PRR11	17	59187493	59187658	S18_peak_1615	intron (ENST00000262293, intron 4 of 9)	ENST00000262293
GDPD1	17	59267553	59267789	S18_peak_1616	intron (ENST00000284116, intron 7 of 9)	ENST00000284116
AC111155.1	17	60726150	60726394	S18_peak_1617	TTS (ENST00000437414)	ENST00000437414
RP11-332H18.7	17	61282042	61282282	S18_peak_1618	Intergenic between ENST00000585921 and ENST00000614751	ENST00000614751
MED13	17	61980405	61980632	S18_peak_1619	intron (ENST00000397786, intron 16 of 29)	ENST00000397786
RAB7B	1	205979416	205979581	S18_peak_162	intron (ENST00000617070, intron 5 of 5)	ENST00000617070
MED13	17	62034904	62035133	S18_peak_1620	intron (ENST00000397786, intron 4 of 29)	ENST00000397786
EFCAB3	17	62410072	62410316	S18_peak_1621	intron (ENST00000305286, intron 8 of 9)	ENST00000305286
METTL2A	17	62463465	62463776	S18_peak_1622	Intergenic between ENST00000311506 and ENST00000346027	ENST00000311506
TANC2	17	63160908	63161167	S18_peak_1623	intron (ENST00000424789, intron 3 of 24)	ENST00000424789
CCDC47	17	63777654	63777819	S18_peak_1624	Intergenic between ENST00000225726 and	ENST00000225726

					ENST00000427159	
RN7SL404P	17	64871079	64871285	S18_peak_1625	TTS (ENST00000582421)	ENST00000582421
RP11-927P21.1	17	64892833	64893065	S18_peak_1626	promoter-TSS (ENST00000577938)	ENST00000577938
AXIN2	17	65520551	65520716	S18_peak_1627	Intergenic between ENST00000579474 and ENST00000307078	ENST00000307078
PRKCA	17	66294184	66294405	S18_peak_1628	Intergenic between ENST00000516844 and ENST00000413366	ENST00000413366
PRKCA	17	66325731	66325947	S18_peak_1629	intron (ENST00000413366, intron 2 of 16)	ENST00000413366
MAPKAPK2	1	206716122	206716287	S18_peak_163	intron (ENST00000294981, intron 1 of 9)	ENST00000294981
PRKCA	17	66693053	66693306	S18_peak_1630	intron (ENST00000413366, intron 8 of 16)	ENST00000413366
PRKCA	17	66714546	66714793	S18_peak_1631	intron (ENST00000413366, intron 8 of 16)	ENST00000413366
CACNG4	17	66983763	66984044	S18_peak_1632	intron (ENST00000262138, intron 1 of 3)	ENST00000262138
ARSG	17	68326408	68326636	S18_peak_1633	intron (ENST00000448504, intron 2 of 11)	ENST00000448504
ABCA8	17	68852186	68852351	S18_peak_1634	Intergenic between ENST00000591379 and ENST00000269080	ENST00000269080
ABCA8	17	68933347	68933595	S18_peak_1635	intron (ENST00000269080, intron 4 of 37)	ENST00000269080
ABCA5	17	69252572	69252737	S18_peak_1636	intron (ENST00000392676, intron 34 of 38)	ENST00000392676
AC005152.3	17	72023984	72024149	S18_peak_1637	promoter-TSS (ENST00000430908)	ENST00000430908
SLC39A11	17	72807893	72808058	S18_peak_1638	intron (ENST00000542342, intron 6 of 9)	ENST00000542342
SLC39A11	17	72815695	72815937	S18_peak_1639	intron (ENST00000542342, intron 6 of 9)	ENST00000542342
PFKFB2	1	207069566	207069731	S18_peak_164	intron (ENST00000367080, intron 11 of 14)	ENST00000367080
RP11-143K11.5	17	73189726	73189923	S18_peak_1640	intron (ENST00000580671, intron 2 of 2)	ENST00000580671
CTD-2582D11.1	17	73888808	73888973	S18_peak_1641	Intergenic between ENST00000580776 and ENST00000581911	ENST00000581911
HID1-AS1	17	74973443	74973686	S18_peak_1642	intron (ENST00000577295, intron 1 of 2)	ENST00000577295
KCTD2	17	75056494	75056659	S18_peak_1643	intron (ENST00000322444, intron 3 of 5)	ENST00000322444
GGA3	17	75259565	75259952	S18_peak_1644	intron (ENST00000537686, intron 1 of 16)	ENST00000537686
GRB2	17	75315682	75315896	S18_peak_1645	Intergenic between ENST00000442286 and ENST00000316804	ENST00000316804
SAP30BP	17	75674023	75674229	S18_peak_1646	intron (ENST00000584667, intron 3 of 10)	ENST00000584667
ITGB4	17	75744483	75744765	S18_peak_1647	intron (ENST00000200181, intron 26 of 39)	ENST00000200181
TEN1	17	75998119	75998293	S18_peak_1648	intron (ENST00000397640, intron 3 of 3)	ENST00000397640
EVPL	17	76012910	76013075	S18_peak_1649	intron (ENST00000586740, intron 18 of 21)	ENST00000586740
CD46	1	207787307	207787510	S18_peak_165	intron (ENST00000358170, intron 12 of 13)	ENST00000358170
METTL23	17	76730710	76730926	S18_peak_1650	intron (ENST00000588822, intron 1 of 3)	ENST00000588822
MGAT5B	17	76875811	76876007	S18_peak_1651	intron (ENST00000301618, intron 2 of 16)	ENST00000301618
SEC14L1	17	77106366	77106531	S18_peak_1652	intron (ENST00000392476, intron 3 of 19)	ENST00000392476
C17orf99	17	78155330	78155495	S18_peak_1653	intron (ENST00000340363, intron 2 of 4)	ENST00000340363
TIMP2	17	78889038	78889238	S18_peak_1654	intron (ENST00000262768, intron 1 of 4)	ENST00000262768
TBC1D16	17	79971947	79972112	S18_peak_1655	intron (ENST00000310924, intron 3 of 11)	ENST00000310924
RNF213	17	80369557	80369722	S18_peak_1656	exon (ENST00000582970, exon 45 of 68)	ENST00000582970
RNF213	17	80395946	80396111	S18_peak_1657	exon (ENST00000582970, exon 68 of 68)	ENST00000582970
ENDOV	17	80413242	80413581	S18_peak_1658	Intergenic between ENST00000427003 and ENST00000518137	ENST00000518137

CTD-2561B21.11	17	81017693	81017858	S18_peak_1659	TTS (ENST00000575647)	ENST00000575647
CD34	1	207961434	207961599	S18_peak_166	Intergenic between ENST00000356522 and ENST00000367033	ENST00000356522
RP11-1055B8.2	17	81359117	81359381	S18_peak_1660	Intergenic between ENST00000617652 and ENST00000574472	ENST00000574472
RP13-1032I1.10	17	81709068	81709279	S18_peak_1661	intron (ENST00000571730, intron 5 of 14)	ENST00000571730
RP13-1032I1.10	17	81729021	81729240	S18_peak_1662	Intergenic between ENST00000571730 and ENST00000400723	ENST00000571730
CSNK1D	17	82266676	82266861	S18_peak_1663	intron (ENST00000314028, intron 1 of 8)	ENST00000314028
NARF	17	82471531	82471773	S18_peak_1664	intron (ENST00000390006, intron 4 of 10)	ENST00000390006
NARF-IT1	17	82494393	82494558	S18_peak_1665	Intergenic between ENST00000584012 and ENST00000473637	ENST00000584012
FN3K	17	82743719	82743884	S18_peak_1666	intron (ENST00000300784, intron 4 of 5)	ENST00000300784
METRNL	17	83082239	83082404	S18_peak_1667	intron (ENST00000320095, intron 1 of 3)	ENST00000320095
AC139099.7	17	83135723	83135891	S18_peak_1668	promoter-TSS (ENST00000625083)	ENST00000625083
GAPLINC	18	3474397	3474654	S18_peak_1669	intron (ENST00000581442, intron 2 of 2)	ENST00000581442
RCOR3	1	211289236	211289401	S18_peak_167	exon (ENST00000367006, exon 8 of 11)	ENST00000367006
DLGAP1	18	3525651	3525816	S18_peak_1670	intron (ENST00000400155, intron 7 of 9)	ENST00000400155
DLGAP1-AS5	18	4286390	4286667	S18_peak_1671	intron (ENST00000565759, intron 2 of 2)	ENST00000565759
RP11-91I8.1	18	6625989	6626154	S18_peak_1672	Intergenic between ENST00000479676 and ENST00000563503	ENST00000563503
RP11-678G15.2	18	7582429	7582594	S18_peak_1673	Intergenic between ENST00000391324 and ENST00000579509	ENST00000579509
PIEZO2	18	10759417	10759582	S18_peak_1674	exon (ENST00000503781, exon 24 of 52)	ENST00000503781
ROCK1	18	19413628	19413793	S18_peak_1675	Intergenic between ENST00000410155 and ENST00000399799	ENST00000399799
ROCK1	18	20561901	20562070	S18_peak_1676	Intergenic between ENST00000410155 and ENST00000399799	ENST00000399799
RP11-27G24.1	18	59405302	59405471	S18_peak_1677	Intergenic between ENST00000591331 and ENST00000589242	ENST00000591331
LINC01002	19	214897	215083	S18_peak_1678	intron (ENST00000631644, intron 2 of 3)	ENST00000631644
PLPP2	19	286733	286898	S18_peak_1679	intron (ENST00000434325, intron 3 of 5)	ENST00000434325
RP11-15I11.2	1	212178643	212178815	S18_peak_168	intron (ENST00000444750, intron 3 of 3)	ENST00000444750
THEG	19	356505	356726	S18_peak_1680	Intergenic between ENST00000591533 and ENST00000346878	ENST00000346878
CFD	19	866531	866891	S18_peak_1681	Intergenic between ENST00000327726 and ENST00000384776	ENST00000327726
HMGB2P1	19	1190421	1190586	S18_peak_1682	Intergenic between ENST00000438103 and ENST00000591146	ENST00000591146
AC005330.2	19	1331711	1331942	S18_peak_1683	Intergenic between ENST00000590086 and ENST00000591806	ENST00000590086
AC005330.2	19	1334706	1334906	S18_peak_1684	Intergenic between ENST00000590086 and ENST00000591806	ENST00000590086
NDUFS7	19	1381100	1381265	S18_peak_1685	Intergenic between ENST00000589673 and	ENST00000233627

					ENST00000233627	
Metazoa_SRP	19	1674775	1674940	S18_peak_1686	Intergenic between ENST00000517124 and ENST00000622745	ENST00000622745
AP3D1	19	2143855	2144125	S18_peak_1687	intron (ENST00000345016, intron 1 of 29)	ENST00000345016
LLfos-48D6.2	19	2293177	2293345	S18_peak_1688	intron (ENST00000621615, intron 1 of 7)	ENST00000621615
LLfos-48D6.2	19	2319761	2319930	S18_peak_1689	intron (ENST00000621615, intron 1 of 7)	ENST00000621615
TMEM206	1	212381984	212382199	S18_peak_169	intron (ENST00000261455, intron 4 of 7)	ENST00000261455
GNG7	19	2608052	2608217	S18_peak_1690	intron (ENST00000382159, intron 2 of 4)	ENST00000382159
GNA15	19	3136814	3136990	S18_peak_1691	promoter-TSS (ENST00000262958)	ENST00000262958
SMIM24	19	3484215	3484380	S18_peak_1692	Intergenic between ENST00000215531 and ENST00000441788	ENST00000215531
PIAS4	19	4026801	4027006	S18_peak_1693	intron (ENST00000262971, intron 3 of 10)	ENST00000262971
CTB-50L17.16	19	4466300	4466465	S18_peak_1694	intron (ENST00000590989, intron 1 of 1)	ENST00000590989
SEMA6B	19	4555170	4555389	S18_peak_1695	intron (ENST00000586582, intron 7 of 16)	ENST00000586582
TNFAIP8L1	19	4631776	4631941	S18_peak_1696	Intergenic between ENST00000592027 and ENST00000327473	ENST00000327473
TINCR	19	5561620	5561892	S18_peak_1697	intron (ENST00000448587, intron 2 of 2)	ENST00000448587
LONP1	19	5696653	5696975	S18_peak_1698	intron (ENST00000360614, intron 10 of 17)	ENST00000360614
AC024592.9	19	5854943	5855112	S18_peak_1699	intron (ENST00000589276, intron 3 of 3)	ENST00000589276
MST1L	1	16780895	16781103	S18_peak_17	Intergenic between ENST00000545160 and ENST00000414340	ENST00000545160
C1orf143	1	218513757	218513952	S18_peak_170	intron (ENST00000443836, intron 1 of 2)	ENST00000443836
FCER2	19	7701456	7701640	S18_peak_1700	promoter-TSS (ENST00000597921)	ENST00000597921
MAP2K7	19	7908816	7908989	S18_peak_1701	intron (ENST00000397983, intron 2 of 11)	ENST00000397983
CTD-3193O13.13	19	7914566	7914770	S18_peak_1702	Intergenic between ENST00000595655 and ENST00000565886	ENST00000595655
MYO1F	19	8531288	8531453	S18_peak_1703	intron (ENST00000338257, intron 19 of 27)	ENST00000338257
MYO1F	19	8538602	8538889	S18_peak_1704	intron (ENST00000338257, intron 16 of 27)	ENST00000338257
ZNF121	19	9566492	9566657	S18_peak_1705	exon (ENST00000320451, exon 4 of 4)	ENST00000320451
CTD-3116E22.7	19	9735041	9735206	S18_peak_1706	Intergenic between ENST00000585880 and ENST00000588113	ENST00000585880
C3P1	19	10043965	10044130	S18_peak_1707	intron (ENST00000497718, intron 13 of 38)	ENST00000497718
P2RY11	19	10114956	10115234	S18_peak_1708	exon (ENST00000321826, exon 2 of 2)	ENST00000321826
KANK2	19	11175157	11175372	S18_peak_1709	intron (ENST00000586659, intron 8 of 12)	ENST00000586659
C1orf143	1	218525712	218525877	S18_peak_171	exon (ENST00000443836, exon 3 of 3)	ENST00000443836
ZNF627	19	11623964	11624129	S18_peak_1710	Intergenic between ENST00000361113 and ENST00000344893	ENST00000361113
CTD-3105H18.5	19	12372731	12372907	S18_peak_1711	Intergenic between ENST00000293725 and ENST00000458635	ENST00000458635
CTD-3105H18.16	19	12439207	12439487	S18_peak_1712	promoter-TSS (ENST00000595562)	ENST00000595562
TNPO2	19	12710560	12710725	S18_peak_1713	exon (ENST00000356861, exon 12 of 25)	ENST00000356861
C19orf43	19	12742963	12743128	S18_peak_1714	Intergenic between ENST00000242784 and ENST00000397668	ENST00000242784
HOOK2	19	12751340	12751614	S18_peak_1715	Intergenic between ENST00000242784 and	ENST00000397668

					ENST00000397668	
SYCE2	19	12908252	12908478	S18_peak_1716	intron (ENST00000293695, intron 2 of 5)	ENST00000293695
NFIX	19	13036044	13036235	S18_peak_1717	intron (ENST00000397661, intron 2 of 9)	ENST00000397661
PRKACA	19	14106253	14106418	S18_peak_1718	intron (ENST00000308677, intron 3 of 9)	ENST00000308677
ADGRE5	19	14373672	14373943	S18_peak_1719	Intergenic between ENST00000588275 and ENST00000358600	ENST00000358600
PRELID3BP1	1	220468371	220468583	S18_peak_172	promoter-TSS (ENST00000457142)	ENST00000457142
DNAJB1	19	14514473	14514638	S18_peak_1720	TTS (ENST00000254322)	ENST00000254322
CLEC17A	19	14594911	14595087	S18_peak_1721	intron (ENST00000547437, intron 7 of 12)	ENST00000547437
ADGRE3	19	14641533	14641736	S18_peak_1722	intron (ENST00000443157, intron 6 of 12)	ENST00000443157
CCDC105	19	15031896	15032084	S18_peak_1723	Intergenic between ENST00000292574 and ENST00000427043	ENST00000292574
OR10H2	19	15723871	15724085	S18_peak_1724	Intergenic between ENST00000550308 and ENST00000305899	ENST00000305899
HSH2D	19	16142183	16142370	S18_peak_1725	Intergenic between ENST00000599676 and ENST00000613986	ENST00000613986
AP1M1	19	16216136	16216301	S18_peak_1726	intron (ENST00000291439, intron 5 of 11)	ENST00000291439
CALR3	19	16492648	16492813	S18_peak_1727	intron (ENST00000269881, intron 2 of 8)	ENST00000269881
CTD-3222D19.2	19	16557533	16557698	S18_peak_1728	intron (ENST00000409035, intron 5 of 11)	ENST00000409035
CTD-3222D19.2	19	16580635	16580800	S18_peak_1729	intron (ENST00000409035, intron 2 of 11)	ENST00000409035
C1orf140	1	221385188	221385374	S18_peak_173	Intergenic between ENST00000434398 and ENST00000440104	ENST00000434398
CTD-2538G9.3	19	16705467	16705644	S18_peak_1730	Intergenic between ENST00000187762 and ENST00000599446	ENST00000599446
NWD1	19	16801666	16801831	S18_peak_1731	intron (ENST00000438489, intron 16 of 18)	ENST00000438489
HAUS8	19	17052994	17053364	S18_peak_1732	intron (ENST00000253669, intron 9 of 10)	ENST00000253669
CTD-3032J10.2	19	17139914	17140079	S18_peak_1733	Intergenic between ENST00000597045 and ENST00000597216	ENST00000597216
PGLS	19	17517540	17517779	S18_peak_1734	intron (ENST00000252603, intron 3 of 4)	ENST00000252603
UNC13A	19	17598138	17598338	S18_peak_1735	Intergenic between ENST00000469924 and ENST00000519716	ENST00000519716
INSL3	19	17823598	17823763	S18_peak_1736	Intergenic between ENST00000379695 and ENST00000222247	ENST00000379695
MAST3	19	18116421	18116586	S18_peak_1737	intron (ENST00000262811, intron 2 of 26)	ENST00000262811
KIAA1683	19	18265871	18266036	S18_peak_1738	exon (ENST00000392413, exon 3 of 4)	ENST00000392413
LSM4	19	18316100	18316265	S18_peak_1739	intron (ENST00000593829, intron 1 of 4)	ENST00000593829
RP11-103C3.1	1	221443096	221443341	S18_peak_174	Intergenic between ENST00000434398 and ENST00000440104	ENST00000440104
UPF1	19	18817719	18817884	S18_peak_1740	Intergenic between ENST00000222271 and ENST00000599848	ENST00000599848
COPE	19	18898196	18898361	S18_peak_1741	Intergenic between ENST00000597769 and ENST00000262812	ENST00000262812
AC002985.3	19	18928528	18928693	S18_peak_1742	intron (ENST00000593484, intron 4 of 5)	ENST00000593484
LINC00662	19	27755476	27755792	S18_peak_1743	intron (ENST00000588027, intron 1 of 2)	ENST00000588027

CTC-459F4.3	19	27901846	27902011	S18_peak_1744	intron (ENST00000588122, intron 3 of 3)	ENST00000588122
CTC-459F4.3	19	27903885	27904050	S18_peak_1745	intron (ENST00000588122, intron 3 of 3)	ENST00000588122
CCNE1	19	29822747	29822933	S18_peak_1746	intron (ENST00000262643, intron 11 of 11)	ENST00000262643
URI1	19	29940883	29941048	S18_peak_1747	intron (ENST00000360605, intron 1 of 10)	ENST00000360605
CTC-439O9.3	19	31156976	31157141	S18_peak_1748	Intergenic between ENST00000587702 and ENST00000589511	ENST00000589511
CEP89	19	32876829	32877021	S18_peak_1749	exon (ENST00000305768, exon 19 of 19)	ENST00000305768
RP11-358H9.1	1	222394269	222394434	S18_peak_175	Intergenic between ENST00000418582 and ENST00000455363	ENST00000455363
CEP89	19	32890229	32890442	S18_peak_1750	intron (ENST00000305768, intron 16 of 18)	ENST00000305768
CEP89	19	32942047	32942212	S18_peak_1751	intron (ENST00000305768, intron 5 of 18)	ENST00000305768
KCTD15	19	33814775	33814977	S18_peak_1752	exon (ENST00000284006, exon 7 of 7)	ENST00000284006
CTC-526N19.1	19	33884697	33884898	S18_peak_1753	Intergenic between ENST00000284006 and ENST00000610908	ENST00000610908
PDCD2L	19	34415001	34415166	S18_peak_1754	intron (ENST00000246535, intron 5 of 6)	ENST00000246535
KIRREL2	19	35861882	35862047	S18_peak_1755	exon (ENST00000347900, exon 10 of 15)	ENST00000347900
Y_RNA	19	36237575	36237802	S18_peak_1756	Intergenic between ENST00000363309 and ENST00000585356	ENST00000363309
CTD-3220F14.3	19	37293507	37293848	S18_peak_1757	Intergenic between ENST00000587477 and ENST00000611171	ENST00000611171
PPP1R14A	19	38254902	38255122	S18_peak_1758	promoter-TSS (ENST00000347262)	ENST00000347262
RYR1	19	38530078	38530243	S18_peak_1759	intron (ENST00000355481, intron 75 of 104)	ENST00000355481
RP11-378J18.9	1	222483436	222483609	S18_peak_176	intron (ENST00000621440, intron 2 of 4)	ENST00000621440
FBXO27	19	39010329	39010548	S18_peak_1760	Intergenic between ENST00000292852 and ENST00000292853	ENST00000292853
NCCRP1	19	39184882	39185256	S18_peak_1761	Intergenic between ENST00000599484 and ENST00000339852	ENST00000339852
PAF1	19	39386095	39386334	S18_peak_1762	exon (ENST00000221265, exon 14 of 14)	ENST00000221265
PLEKHG2	19	39426737	39426988	S18_peak_1763	exon (ENST00000425673, exon 19 of 19)	ENST00000425673
TIMM50	19	39484817	39485049	S18_peak_1764	intron (ENST00000314349, intron 4 of 10)	ENST00000314349
ZNF546	19	40002857	40003022	S18_peak_1765	intron (ENST00000347077, intron 3 of 6)	ENST00000347077
MAP3K10	19	40189300	40189465	S18_peak_1766	Intergenic between ENST00000446052 and ENST00000253055	ENST00000253055
PLD3	19	40355799	40355964	S18_peak_1767	intron (ENST00000409735, intron 1 of 12)	ENST00000409735
SERTAD1	19	40419429	40419594	S18_peak_1768	Intergenic between ENST00000324001 and ENST00000357949	ENST00000357949
NUMBL	19	40657109	40657394	S18_peak_1769	Intergenic between ENST00000624832 and ENST00000252891	ENST00000252891
DEGS1	1	224194110	224194312	S18_peak_177	TTS (ENST00000323699)	ENST00000323699
CYP2S1	19	41211959	41212246	S18_peak_1770	Intergenic between ENST00000310054 and ENST00000301178	ENST00000310054
RABAC1	19	41943558	41943943	S18_peak_1771	Intergenic between ENST00000598387 and ENST00000601891	ENST00000601891
LIPE-AS1	19	42455595	42455770	S18_peak_1772	intron (ENST00000597203, intron 1 of 1)	ENST00000597203

CD177P1	19	43393226	43393455	S18_peak_1773	Intergenic between ENST00000606252 and ENST00000598265	ENST00000606252
ETHE1	19	43510411	43510576	S18_peak_1774	intron (ENST00000292147, intron 4 of 6)	ENST00000292147
RNU6-902P	19	44089638	44089860	S18_peak_1775	Intergenic between ENST00000517212 and ENST00000336976	ENST00000517212
CEACAM22P	19	44570589	44570847	S18_peak_1776	intron (ENST00000455455, intron 1 of 3)	ENST00000455455
AC005779.2	19	45199999	45200200	S18_peak_1777	intron (ENST00000593083, intron 4 of 4)	ENST00000593083
AC006126.3	19	45208988	45209197	S18_peak_1778	intron (ENST00000591569, intron 3 of 3)	ENST00000591569
FOSB	19	45455452	45455751	S18_peak_1779	Intergenic between ENST00000611232 and ENST00000353609	ENST00000353609
ENAH	1	225555431	225555596	S18_peak_178	intron (ENST00000366844, intron 2 of 14)	ENST00000366844
RTN2	19	45487613	45487809	S18_peak_1780	intron (ENST00000245923, intron 9 of 10)	ENST00000245923
HIF3A	19	46290401	46290669	S18_peak_1781	Intergenic between ENST00000593820 and ENST00000377670	ENST00000377670
CTB-147N14.4	19	46828866	46829056	S18_peak_1782	Intergenic between ENST00000597106 and ENST00000263270	ENST00000597106
ARHGAP35	19	46889076	46889350	S18_peak_1783	Intergenic between ENST00000601649 and ENST00000404338	ENST00000404338
GYS1	19	48967910	48968156	S18_peak_1784	TTS (ENST00000323798)	ENST00000323798
IZUMO2	19	50119470	50119642	S18_peak_1785	Intergenic between ENST00000600998 and ENST00000293405	ENST00000293405
SIGLEC8	19	51446744	51446974	S18_peak_1786	Intergenic between ENST00000594327 and ENST00000321424	ENST00000321424
AC008753.6	19	53844599	53844764	S18_peak_1787	Intergenic between ENST00000391773 and ENST00000455835	ENST00000455835
LAIR2	19	54489289	54489454	S18_peak_1788	Intergenic between ENST00000301200 and ENST00000301202	ENST00000301202
CTB-61M7.1	19	54775268	54775456	S18_peak_1789	intron (ENST00000400864, intron 2 of 5)	ENST00000400864
ACBD3	1	226150285	226150450	S18_peak_179	intron (ENST00000366812, intron 7 of 7)	ENST00000366812
BRSK1	19	55297291	55297534	S18_peak_1790	intron (ENST00000309383, intron 7 of 18)	ENST00000309383
TMEM190	19	55375197	55375429	S18_peak_1791	Intergenic between ENST00000590625 and ENST00000291934	ENST00000291934
NLRP13	19	55910346	55910562	S18_peak_1792	intron (ENST00000342929, intron 6 of 10)	ENST00000342929
CTC-258N23.3	19	56921332	56921551	S18_peak_1793	Intergenic between ENST00000614523 and ENST00000593294	ENST00000593294
AC011995.3	2	2877429	2877594	S18_peak_1794	Intergenic between ENST00000437610 and ENST00000457478	ENST00000437610
AC010729.1	2	5706382	5706547	S18_peak_1795	intron (ENST00000455579, intron 3 of 3)	ENST00000455579
HPCAL1	2	10343752	10343917	S18_peak_1796	intron (ENST00000307845, intron 1 of 4)	ENST00000307845
MIR3681HG	2	12466049	12466214	S18_peak_1797	intron (ENST00000412294, intron 4 of 5)	ENST00000412294
MIR3125	2	12789604	12789769	S18_peak_1798	Intergenic between ENST00000579927 and ENST00000425974	ENST00000579927
MIR3125	2	12826069	12826317	S18_peak_1799	Intergenic between ENST00000579927 and ENST00000425974	ENST00000579927

RNU1-2	1	16896579	16896846	S18_peak_18	TTS (ENST00000384278)	ENST00000384278
ITPKB-IT1	1	226638197	226638473	S18_peak_180	Intergenic between ENST00000366788 and ENST00000443422	ENST00000443422
AC092635.1	2	13471028	13471193	S18_peak_1800	Intergenic between ENST00000414661 and ENST00000434509	ENST00000434509
GEN1	2	17778996	17779161	S18_peak_1801	intron (ENST00000317402, intron 12 of 13)	ENST00000317402
AC012361.1	2	20896520	20896729	S18_peak_1802	Intergenic between ENST00000447260 and ENST00000567376	ENST00000447260
ATAD2B	2	23863092	23863257	S18_peak_1803	intron (ENST00000238789, intron 12 of 27)	ENST00000238789
ASXL2	2	25768159	25768429	S18_peak_1804	intron (ENST00000404843, intron 3 of 9)	ENST00000404843
NDUFB4P4	2	25895335	25895500	S18_peak_1805	Intergenic between ENST00000404822 and ENST00000264712	ENST00000404822
ADGRF3	2	26351956	26352213	S18_peak_1806	Intergenic between ENST00000311519 and ENST00000288710	ENST00000311519
AC015977.6	2	26662612	26662825	S18_peak_1807	Intergenic between ENST00000288861 and ENST00000420852	ENST00000420852
CDKN2AIPNLP2	2	26836756	26836964	S18_peak_1808	Intergenic between ENST00000274516 and ENST00000288699	ENST00000274516
BRE	2	28018888	28019053	S18_peak_1809	intron (ENST00000344773, intron 4 of 12)	ENST00000344773
CTD-2090I13.1	1	227383185	227383350	S18_peak_181	Intergenic between ENST00000420014 and ENST00000445817	ENST00000445817
BRE	2	28294350	28294515	S18_peak_1810	intron (ENST00000344773, intron 10 of 12)	ENST00000344773
TRMT61B	2	28876000	28876165	S18_peak_1811	Intergenic between ENST00000306108 and ENST00000407426	ENST00000306108
CLIP4	2	29158928	29159093	S18_peak_1812	intron (ENST00000401605, intron 11 of 14)	ENST00000401605
AL133249.1	2	31750854	31751180	S18_peak_1813	Intergenic between ENST00000603236 and ENST00000416279	ENST00000416279
BIRC6	2	32437460	32437625	S18_peak_1814	intron (ENST00000421745, intron 15 of 73)	ENST00000421745
LINC01317	2	33841041	33841206	S18_peak_1815	intron (ENST00000366209, intron 1 of 5)	ENST00000366209
LINC01317	2	34028053	34028218	S18_peak_1816	intron (ENST00000366209, intron 4 of 5)	ENST00000366209
AC012593.1	2	34935982	34936180	S18_peak_1817	intron (ENST00000453451, intron 2 of 5)	ENST00000453451
MIR548AD	2	35384578	35384743	S18_peak_1818	Intergenic between ENST00000397347 and ENST00000584780	ENST00000584780
CEBPZOS	2	37202167	37202332	S18_peak_1819	intron (ENST00000397064, intron 4 of 4)	ENST00000397064
CTD-2090I13.1	1	227404693	227404858	S18_peak_182	intron (ENST00000445817, intron 2 of 3)	ENST00000445817
AC011247.3	2	38613708	38613873	S18_peak_1820	Intergenic between ENST00000457097 and ENST00000272252	ENST00000457097
AC013480.2	2	42001789	42002084	S18_peak_1821	Intergenic between ENST00000436551 and ENST00000451514	ENST00000451514
PLEKHH2	2	43730747	43731022	S18_peak_1822	intron (ENST00000282406, intron 18 of 29)	ENST00000282406
RP11-333I13.1	2	46785698	46785936	S18_peak_1823	Intergenic between ENST00000568862 and ENST00000479311	ENST00000568862
AC016722.4	2	46881868	46882033	S18_peak_1824	Intergenic between ENST00000495449 and ENST00000429761	ENST00000429761

AC073283.4	2	47299095	47299308	S18_peak_1825	intron (ENST00000448713, intron 2 of 2)	ENST00000448713
AC139712.1	2	52444666	52444831	S18_peak_1826	Intergenic between ENST00000435357 and ENST00000424302	ENST00000424302
RNU6-997P	2	53549657	53549867	S18_peak_1827	Intergenic between ENST00000421749 and ENST00000384725	ENST00000384725
SPTBN1	2	54454245	54454410	S18_peak_1828	Intergenic between ENST00000398634 and ENST00000356805	ENST00000356805
EFEMP1	2	55855659	55855860	S18_peak_1829	Intergenic between ENST00000447944 and ENST00000394555	ENST00000394555
OBSCN	1	228221800	228221965	S18_peak_183	intron (ENST00000284548, intron 8 of 80)	ENST00000284548
RP11-481J13.1	2	56181302	56181467	S18_peak_1830	exon (ENST00000606639, exon 7 of 7)	ENST00000606639
USP34	2	61308851	61309016	S18_peak_1831	intron (ENST00000398571, intron 27 of 79)	ENST00000398571
USP34	2	61454524	61454689	S18_peak_1832	intron (ENST00000398571, intron 1 of 79)	ENST00000398571
RP11-355B11.2	2	61475981	61476146	S18_peak_1833	intron (ENST00000603652, intron 1 of 2)	ENST00000603652
COMMD1	2	62014643	62014891	S18_peak_1834	intron (ENST00000311832, intron 2 of 2)	ENST00000311832
AC007880.1	2	64844919	64845326	S18_peak_1835	Intergenic between ENST00000363094 and ENST00000445865	ENST00000445865
AC010987.6	2	67907012	67907177	S18_peak_1836	Intergenic between ENST00000457448 and ENST00000454972	ENST00000457448
PCBP1-AS1	2	70100457	70100622	S18_peak_1837	intron (ENST00000628659, intron 1 of 5)	ENST00000628659
ZNF638	2	71279913	71280078	S18_peak_1838	Intergenic between ENST00000244221 and ENST00000409544	ENST00000409544
DCTN1	2	74382855	74383023	S18_peak_1839	intron (ENST00000409240, intron 1 of 27)	ENST00000409240
ABCB10	1	229540078	229540348	S18_peak_184	intron (ENST00000344517, intron 5 of 12)	ENST00000344517
POLE4	2	74986145	74986310	S18_peak_1840	Intergenic between ENST00000483063 and ENST00000305249	ENST00000483063
AC079117.1	2	76997521	76997686	S18_peak_1841	intron (ENST00000445178, intron 3 of 3)	ENST00000445178
MIR4264	2	79749010	79749175	S18_peak_1842	Intergenic between ENST00000583520 and ENST00000622224	ENST00000583520
GGCX	2	85546373	85546633	S18_peak_1843	exon (ENST00000233838, exon 15 of 15)	ENST00000233838
VAMP8	2	85576060	85576323	S18_peak_1844	Intergenic between ENST00000630104 and ENST00000263864	ENST00000263864
RNF103-CHMP3	2	86681226	86681391	S18_peak_1845	intron (ENST00000604011, intron 2 of 7)	ENST00000604011
RNF103-CHMP3	2	86702376	86702608	S18_peak_1846	intron (ENST00000604011, intron 1 of 7)	ENST00000604011
PLGLB1	2	86980755	86981119	S18_peak_1847	Intergenic between ENST00000415445 and ENST00000355705	ENST00000355705
RGPD2	2	87792916	87793160	S18_peak_1849	intron (ENST00000398146, intron 15 of 22)	ENST00000398146
RP5-858B6.1	1	230823918	230824083	S18_peak_185	promoter-TSS (ENST00000436739)	ENST00000436739
CH17-132F21.5	2	90400127	90400351	S18_peak_1850	Intergenic between ENST00000631132 and ENST00000454518	ENST00000631132
AC018696.4	2	91566010	91566220	S18_peak_1852	Intergenic between ENST00000631132 and ENST00000454518	ENST00000454518
RNA5SP100	2	91667098	91667285	S18_peak_1853	Intergenic between ENST00000423774 and ENST00000410903	ENST00000410903

AC027612.3	2	91709278	91709531	S18_peak_1854	intron (ENST00000436174, intron 2 of 9)	ENST00000436174
IGKV1OR2-2	2	92582444	92582661	S18_peak_1855	Intergenic between ENST00000451163 and ENST00000562821	ENST00000451163
IGKV1OR2-2	2	92709331	92709515	S18_peak_1856	Intergenic between ENST00000451163 and ENST00000562821	ENST00000451163
IGKV1OR2-2	2	92869594	92869759	S18_peak_1857	Intergenic between ENST00000451163 and ENST00000562821	ENST00000451163
RP11-407P15.1	2	93313101	93313272	S18_peak_1858	Intergenic between ENST00000451163 and ENST00000562821	ENST00000562821
RN7SL313P	2	97147978	97148158	S18_peak_1859	Intergenic between ENST00000497618 and ENST00000621110	ENST00000497618
C1orf198	1	230831780	230831945	S18_peak_186	Intergenic between ENST00000436739 and ENST00000470540	ENST00000470540
RN7SL313P	2	97148448	97148613	S18_peak_1860	Intergenic between ENST00000497618 and ENST00000621110	ENST00000497618
RP11-734K23.9	2	97258548	97258754	S18_peak_1861	Intergenic between ENST00000497618 and ENST00000621110	ENST00000621110
RP11-734K23.9	2	97267303	97267468	S18_peak_1862	Intergenic between ENST00000497618 and ENST00000621110	ENST00000621110
KIAA1211L	2	98870930	98871095	S18_peak_1863	intron (ENST00000397899, intron 1 of 9)	ENST00000397899
REV1	2	99447687	99447852	S18_peak_1864	intron (ENST00000258428, intron 4 of 22)	ENST00000258428
NPAS2	2	100917324	100917559	S18_peak_1865	intron (ENST00000335681, intron 2 of 20)	ENST00000335681
RGPD5	2	109802608	109802891	S18_peak_1866	intron (ENST00000016946, intron 2 of 22)	ENST00000016946
MIR4435-2HG	2	111366114	111366299	S18_peak_1867	promoter-TSS (ENST00000609902)	ENST00000609902
MERTK	2	112027596	112027761	S18_peak_1868	intron (ENST00000295408, intron 18 of 18)	ENST00000295408
RGPD8	2	112399768	112399933	S18_peak_1869	intron (ENST00000302558, intron 13 of 22)	ENST00000302558
TTC13	1	230940982	230941147	S18_peak_187	intron (ENST00000366662, intron 4 of 20)	ENST00000366662
RABL2A	2	113641307	113641487	S18_peak_1870	exon (ENST00000393167, exon 7 of 9)	ENST00000393167
AC104653.1	2	113860850	113861087	S18_peak_1871	Intergenic between ENST00000446401 and ENST00000602760	ENST00000446401
RP11-338I24.1	2	116121711	116121876	S18_peak_1872	Intergenic between ENST00000446095 and ENST00000605241	ENST00000605241
AC097499.1	2	125426019	125426184	S18_peak_1873	Intergenic between ENST00000383999 and ENST00000430692	ENST00000430692
POTEJ	2	130633985	130634237	S18_peak_1874	intron (ENST00000409602, intron 9 of 14)	ENST00000409602
POTEE	2	131248804	131248986	S18_peak_1875	intron (ENST00000356920, intron 10 of 14)	ENST00000356920
AC093375.1	2	155970380	155970545	S18_peak_1876	Intergenic between ENST00000383991 and ENST00000635799	ENST00000635799
CDK7PS	2	157078781	157078946	S18_peak_1877	Intergenic between ENST00000416050 and ENST00000434574	ENST00000434574
TANC1	2	159205678	159205957	S18_peak_1878	intron (ENST00000263635, intron 19 of 26)	ENST00000263635
SPC25	2	168878021	168878186	S18_peak_1879	intron (ENST00000282074, intron 3 of 6)	ENST00000282074
MIR4427	1	233708522	233708687	S18_peak_188	Intergenic between ENST00000585213 and ENST00000453379	ENST00000585213

AC093899.3	2	169577028	169577193	S18_peak_1880	TTS (ENST00000457984)	ENST00000457984
CHN1	2	174862270	174862631	S18_peak_1881	intron (ENST00000409900, intron 6 of 12)	ENST00000409900
OSBPL6	2	178328164	178328329	S18_peak_1882	intron (ENST00000392505, intron 4 of 25)	ENST00000392505
CWC22	2	180065415	180065675	S18_peak_1883	Intergenic between ENST00000410053 and ENST00000603209	ENST00000410053
AC020595.1	2	181434170	181434335	S18_peak_1884	Intergenic between ENST00000456446 and ENST00000339307	ENST00000456446
RN7SL267P	2	182256187	182256352	S18_peak_1885	Intergenic between ENST00000410486 and ENST00000485107	ENST00000485107
CACYBPP2	2	183569390	183569555	S18_peak_1886	Intergenic between ENST00000429574 and ENST00000453625	ENST00000453625
AC007319.1	2	187212293	187212458	S18_peak_1887	intron (ENST00000453517, intron 2 of 3)	ENST00000453517
WDR75	2	189448237	189448402	S18_peak_1888	intron (ENST00000314761, intron 1 of 20)	ENST00000314761
AC010983.1	2	195043780	195043945	S18_peak_1889	Intergenic between ENST00000418387 and ENST00000413290	ENST00000418387
TARBP1	1	234399182	234399347	S18_peak_189	intron (ENST00000040877, intron 25 of 29)	ENST00000040877
AC013264.1	2	197192118	197192283	S18_peak_1890	intron (ENST00000282272, intron 1 of 27)	ENST00000282272
AC093590.1	2	199598779	199599044	S18_peak_1891	Intergenic between ENST00000416200 and ENST00000419243	ENST00000419243
TYW5	2	200046337	200046502	S18_peak_1892	Intergenic between ENST00000354611 and ENST00000409718	ENST00000354611
ALS2	2	201719823	201720079	S18_peak_1893	intron (ENST00000264276, intron 23 of 33)	ENST00000264276
AC125238.2	2	203632848	203633013	S18_peak_1894	Intergenic between ENST00000308091 and ENST00000430003	ENST00000430003
DSTNP5	2	204323993	204324158	S18_peak_1895	Intergenic between ENST00000427921 and ENST00000425086	ENST00000427921
AC007879.5	2	207356949	207357114	S18_peak_1896	intron (ENST00000412387, intron 3 of 4)	ENST00000412387
AC012362.3	2	208359224	208359494	S18_peak_1897	Intergenic between ENST00000429485 and ENST00000434906	ENST00000434906
UNC80	2	209929975	209930168	S18_peak_1898	intron (ENST00000272845, intron 36 of 62)	ENST00000272845
ATIC	2	215329907	215330094	S18_peak_1899	intron (ENST00000236959, intron 7 of 15)	ENST00000236959
RP11-473A10.2	1	17846719	17846884	S18_peak_19	Intergenic between ENST00000430540 and ENST00000569711	ENST00000430540
B3GALNT2	1	235476794	235477011	S18_peak_190	intron (ENST00000366600, intron 5 of 11)	ENST00000366600
4-Mar	2	216343954	216344249	S18_peak_1900	intron (ENST00000273067, intron 1 of 3)	ENST00000273067
SERPINE2	2	224098488	224098653	S18_peak_1901	Intergenic between ENST00000447280 and ENST00000401725	ENST00000447280
DOCK10	2	224836750	224836915	S18_peak_1902	intron (ENST00000258390, intron 25 of 55)	ENST00000258390
RHBDD1	2	226867771	226867936	S18_peak_1903	intron (ENST00000341329, intron 3 of 6)	ENST00000341329
RP11-499P1.2	2	230938436	230938742	S18_peak_1904	Intergenic between ENST00000637275 and ENST00000452636	ENST00000637275
PSMD1	2	231124181	231124346	S18_peak_1905	intron (ENST00000308696, intron 16 of 24)	ENST00000308696
LINC00471	2	231510978	231511143	S18_peak_1906	intron (ENST00000313064, intron 2 of 2)	ENST00000313064
MSL3P1	2	233884444	233884720	S18_peak_1907	Intergenic between ENST00000440028 and	ENST00000440028

					ENST00000324695	
AC097713.5	2	234337122	234337323	S18_peak_1908	Intergenic between ENST00000430199 and ENST00000418025	ENST00000430199
AGXT	2	240872308	240872474	S18_peak_1909	intron (ENST00000307503, intron 4 of 10)	ENST00000307503
MIPEPP2	1	238722159	238722324	S18_peak_191	Intergenic between ENST00000424695 and ENST00000422560	ENST00000422560
FARP2	2	241381233	241381398	S18_peak_1910	intron (ENST00000264042, intron 2 of 26)	ENST00000264042
POLR3F	20	18464767	18464985	S18_peak_1911	Intergenic between ENST00000516613 and ENST00000462997	ENST00000462997
FRG1CP	20	28495761	28495960	S18_peak_1912	Intergenic between ENST00000416638 and ENST00000358464	ENST00000358464
FRG1CP	20	28564336	28564557	S18_peak_1913	Intergenic between ENST00000416638 and ENST00000358464	ENST00000358464
FRG1CP	20	28788798	28789023	S18_peak_1914	Intergenic between ENST00000358464 and ENST00000635586	ENST00000358464
FRG1CP	20	28816380	28816818	S18_peak_1915	Intergenic between ENST00000358464 and ENST00000635586	ENST00000358464
FRG1DP	20	29027716	29027881	S18_peak_1916	Intergenic between ENST00000358464 and ENST00000635586	ENST00000635586
FRG1DP	20	29029281	29029446	S18_peak_1917	Intergenic between ENST00000358464 and ENST00000635586	ENST00000635586
FRG1DP	20	29118367	29118583	S18_peak_1918	Intergenic between ENST00000635586 and ENST00000637374	ENST00000635586
FRG1DP	20	29120559	29120765	S18_peak_1919	Intergenic between ENST00000635586 and ENST00000637374	ENST00000635586
RGS7	1	241328388	241328556	S18_peak_192	intron (ENST00000348120, intron 1 of 14)	ENST00000348120
DUX4L33	20	29331959	29332124	S18_peak_1920	Intergenic between ENST00000635217 and ENST00000634362	ENST00000635217
DUX4L34	20	29405593	29405758	S18_peak_1921	Intergenic between ENST00000635217 and ENST00000634362	ENST00000634362
CFTRP2	20	29681214	29681379	S18_peak_1922	Intergenic between ENST00000634304 and ENST00000638132	ENST00000634304
LINC01597	20	30275072	30275237	S18_peak_1923	Intergenic between ENST00000376400 and ENST00000380888	ENST00000380888
DEFB115	20	31106717	31106933	S18_peak_1924	Intergenic between ENST00000612928 and ENST00000400552	ENST00000400552
DEFB123	20	31446885	31447137	S18_peak_1925	intron (ENST00000376309, intron 1 of 1)	ENST00000376309
MIR3193	20	31607067	31607320	S18_peak_1926	promoter-TSS (ENST00000578262)	ENST00000578262
DUSP15	20	31866671	31866836	S18_peak_1927	intron (ENST00000398084, intron 3 of 6)	ENST00000398084
XKR7	20	31963896	31964090	S18_peak_1928	Intergenic between ENST00000202017 and ENST00000562532	ENST00000562532
HCK	20	32101093	32101258	S18_peak_1929	intron (ENST00000629881, intron 13 of 13)	ENST00000629881
SDCCAG8	1	243428790	243428955	S18_peak_193	intron (ENST00000366541, intron 16 of 17)	ENST00000366541
TM9SF4	20	32163354	32163534	S18_peak_1930	intron (ENST00000398022, intron 17 of 17)	ENST00000398022

BPIFA3	20	33221233	33221398	S18_peak_1931	intron (ENST00000375454, intron 1 of 6)	ENST00000375454
RPL12P3	20	33273287	33273452	S18_peak_1932	Intergenic between ENST00000440080 and ENST00000253354	ENST00000440080
BPIFB5P	20	33328990	33329155	S18_peak_1933	intron (ENST00000457066, intron 8 of 9)	ENST00000457066
CBFA2T2	20	33515599	33515957	S18_peak_1934	intron (ENST00000342704, intron 1 of 10)	ENST00000342704
CBFA2T2	20	33579830	33579999	S18_peak_1935	intron (ENST00000342704, intron 1 of 10)	ENST00000342704
CBFA2T2	20	33604779	33604944	S18_peak_1936	intron (ENST00000342704, intron 1 of 10)	ENST00000342704
NECAB3	20	33664845	33665125	S18_peak_1937	intron (ENST00000375238, intron 5 of 12)	ENST00000375238
TPM3P2	20	33933981	33934210	S18_peak_1938	Intergenic between ENST00000397772 and ENST00000425307	ENST00000397772
CTD-3216D2.4	20	34330654	34330819	S18_peak_1939	Intergenic between ENST00000609218 and ENST00000437424	ENST00000437424
C1orf100	1	244266844	244267009	S18_peak_194	Intergenic between ENST00000440148 and ENST00000308105	ENST00000308105
DYNLRB1	20	34514750	34515034	S18_peak_1940	Intergenic between ENST00000618799 and ENST00000357156	ENST00000357156
MAP1LC3A	20	34542591	34542895	S18_peak_1941	Intergenic between ENST00000383855 and ENST00000374837	ENST00000374837
EDEM2	20	35116893	35117141	S18_peak_1942	intron (ENST00000374492, intron 10 of 10)	ENST00000374492
RPL37P1	20	35587939	35588104	S18_peak_1943	Intergenic between ENST00000616283 and ENST00000432310	ENST00000432310
NFS1	20	35688899	35689064	S18_peak_1944	intron (ENST00000374092, intron 5 of 12)	ENST00000374092
PHF20	20	35809935	35810100	S18_peak_1945	intron (ENST00000374012, intron 2 of 17)	ENST00000374012
CNBD2	20	35984471	35984636	S18_peak_1946	intron (ENST00000349339, intron 5 of 11)	ENST00000349339
EPB41L1	20	36221660	36221903	S18_peak_1947	intron (ENST00000202028, intron 17 of 19)	ENST00000202028
AAR2	20	36291900	36292065	S18_peak_1948	Intergenic between ENST00000373932 and ENST00000475894	ENST00000373932
AAR2	20	36296666	36296909	S18_peak_1949	Intergenic between ENST00000373932 and ENST00000475894	ENST00000373932
EFCAB2	1	245045153	245045318	S18_peak_195	intron (ENST00000366523, intron 3 of 7)	ENST00000366523
DLGAP4	20	36423029	36423205	S18_peak_1950	Intergenic between ENST00000373932 and ENST00000475894	ENST00000475894
DLGAP4	20	36480933	36481131	S18_peak_1951	intron (ENST00000475894, intron 1 of 6)	ENST00000475894
TGIF2	20	36576197	36576455	S18_peak_1952	intron (ENST00000373874, intron 1 of 2)	ENST00000373874
SAMHD1	20	36902729	36903073	S18_peak_1953	intron (ENST00000262878, intron 13 of 15)	ENST00000262878
SAMHD1	20	36957789	36957954	S18_peak_1954	Intergenic between ENST00000262878 and ENST00000373664	ENST00000262878
MANBAL	20	37332856	37333104	S18_peak_1955	Intergenic between ENST00000373606 and ENST00000424041	ENST00000373606
BLCAP	20	37514012	37514258	S18_peak_1956	Intergenic between ENST00000424041 and ENST00000373537	ENST00000373537
LINC00489	20	37618690	37618856	S18_peak_1957	TTS (ENST00000457816)	ENST00000457816
RP4-640H8.2	20	37673055	37673330	S18_peak_1958	Intergenic between ENST00000457816 and ENST00000373508	ENST00000373508

CTNNBL1	20	37754117	37754296	S18_peak_1959	intron (ENST00000373473, intron 2 of 12)	ENST00000373473
RNU6-1283P	1	246194717	246194933	S18_peak_196	Intergenic between ENST00000365314 and ENST00000426929	ENST00000365314
CTNNBL1	20	37843500	37843665	S18_peak_1960	intron (ENST00000373473, intron 10 of 12)	ENST00000373473
TTI1	20	38010618	38010783	S18_peak_1961	intron (ENST00000373447, intron 2 of 7)	ENST00000373447
RPRD1B	20	38053354	38053519	S18_peak_1962	intron (ENST00000373433, intron 3 of 6)	ENST00000373433
BPI	20	38327773	38327938	S18_peak_1963	intron (ENST00000262865, intron 11 of 14)	ENST00000262865
RP4-705O1.1	20	39106078	39106243	S18_peak_1964	Intergenic between ENST00000450070 and ENST00000412672	ENST00000412672
RP4-705O1.1	20	39188157	39188393	S18_peak_1965	Intergenic between ENST00000450070 and ENST00000412672	ENST00000412672
RP4-705O1.1	20	39265428	39265646	S18_peak_1966	Intergenic between ENST00000412672 and ENST00000419507	ENST00000412672
RP4-705O1.1	20	39274811	39274976	S18_peak_1967	Intergenic between ENST00000412672 and ENST00000419507	ENST00000412672
RP5-1031J8.1	20	39622304	39622517	S18_peak_1968	Intergenic between ENST00000620379 and ENST00000444436	ENST00000444436
RP5-1031J8.1	20	39698591	39698815	S18_peak_1969	Intergenic between ENST00000620379 and ENST00000444436	ENST00000444436
ZNF670-ZNF695	1	247003945	247004134	S18_peak_197	intron (ENST00000465049, intron 1 of 6)	ENST00000465049
MAFB	20	40561094	40561259	S18_peak_1970	Intergenic between ENST00000445278 and ENST00000373313	ENST00000373313
MAFB	20	40682735	40682900	S18_peak_1971	Intergenic between ENST00000445278 and ENST00000373313	ENST00000373313
RP5-1121H13.4	20	41927815	41928071	S18_peak_1972	Intergenic between ENST00000390922 and ENST00000431589	ENST00000431589
RP5-1121H13.3	20	42244936	42245129	S18_peak_1973	Intergenic between ENST00000412348 and ENST00000446195	ENST00000412348
RP4-730D4.1	20	42353485	42353665	S18_peak_1974	Intergenic between ENST00000412348 and ENST00000446195	ENST00000446195
RP4-753D4.2	20	42970428	42970593	S18_peak_1975	promoter-TSS (ENST00000412519)	ENST00000412519
RN7SKP100	20	43100188	43100353	S18_peak_1976	Intergenic between ENST00000411258 and ENST00000611791	ENST00000411258
RNU6-1251P	20	43493166	43493348	S18_peak_1977	Intergenic between ENST00000364502 and ENST00000432727	ENST00000364502
MYBL2	20	43719076	43719241	S18_peak_1978	Intergenic between ENST00000217026 and ENST00000373005	ENST00000217026
TOX2	20	43999934	44000099	S18_peak_1979	intron (ENST00000341197, intron 2 of 8)	ENST00000341197
ZNF669	1	247099557	247099722	S18_peak_198	TTS (ENST00000343381)	ENST00000343381
R3HDML	20	44336905	44337070	S18_peak_1980	promoter-TSS (ENST00000217043)	ENST00000217043
KCNK15-AS1	20	44740234	44740427	S18_peak_1981	intron (ENST00000445420, intron 1 of 2)	ENST00000445420
RP1-300I2.2	20	45230819	45230984	S18_peak_1982	promoter-TSS (ENST00000461248)	ENST00000461248
WFDC9	20	45591205	45591412	S18_peak_1983	Intergenic between ENST00000432876 and ENST00000326000	ENST00000326000

CDH22	20	46253749	46254063	S18_peak_1984	Intergenic between ENST00000474438 and ENST00000243896	ENST00000474438
RP11-394O2.3	20	46380749	46380935	S18_peak_1985	intron (ENST00000612368, intron 3 of 4)	ENST00000612368
SLC13A3	20	46598760	46598958	S18_peak_1986	intron (ENST00000279027, intron 4 of 12)	ENST00000279027
EYA2	20	47091017	47091298	S18_peak_1987	intron (ENST00000357410, intron 8 of 13)	ENST00000357410
ZMYND8	20	47248446	47248611	S18_peak_1988	intron (ENST00000540497, intron 12 of 20)	ENST00000540497
NCOA3	20	47643155	47643320	S18_peak_1989	intron (ENST00000371998, intron 17 of 22)	ENST00000371998
RP11-488L18.8	1	247236133	247236298	S18_peak_199	intron (ENST00000428687, intron 1 of 3)	ENST00000428687
RP11-321P16.1	20	48136508	48136741	S18_peak_1990	Intergenic between ENST00000622092 and ENST00000446280	ENST00000622092
RP11-321P16.1	20	48197253	48197418	S18_peak_1991	Intergenic between ENST00000622092 and ENST00000446280	ENST00000622092
RNU7-144P	20	48523867	48524177	S18_peak_1992	Intergenic between ENST00000459405 and ENST00000371941	ENST00000459405
STAU1	20	49136514	49136843	S18_peak_1993	intron (ENST00000371856, intron 5 of 13)	ENST00000371856
SNORD12	20	49290261	49290586	S18_peak_1994	Intergenic between ENST00000391002 and ENST00000636663	ENST00000391002
RP4-791K14.2	20	49376017	49376182	S18_peak_1995	intron (ENST00000637341, intron 2 of 7)	ENST00000637341
KCNB1	20	49476124	49476415	S18_peak_1996	intron (ENST00000371741, intron 1 of 1)	ENST00000371741
PTGIS	20	49498035	49498200	S18_peak_1997	Intergenic between ENST00000371741 and ENST00000244043	ENST00000244043
PTGIS	20	49543349	49543604	S18_peak_1998	intron (ENST00000244043, intron 4 of 9)	ENST00000244043
B4GALT5	20	49679392	49679658	S18_peak_1999	intron (ENST00000371711, intron 1 of 8)	ENST00000371711
LINC01342	1	1119690	1119897	S18_peak_2	Intergenic between ENST00000465822 and ENST00000416774	ENST00000416774
IFFO2	1	18976580	18976748	S18_peak_20	Intergenic between ENST00000455833 and ENST00000606379	ENST00000455833
RP11-195B3.1	10	3314579	3314747	S18_peak_200	Intergenic between ENST00000436340 and ENST00000413993	ENST00000436340
RP5-1041C10.3	20	49854772	49854991	S18_peak_2000	Intergenic between ENST00000429893 and ENST00000289431	ENST00000429893
KRT18P4	20	49959833	49959998	S18_peak_2001	Intergenic between ENST00000422599 and ENST00000383983	ENST00000422599
TRERNA1	20	50026884	50027049	S18_peak_2002	Intergenic between ENST00000244050 and ENST00000431460	ENST00000431460
LINC01270	20	50263803	50263968	S18_peak_2003	Intergenic between ENST00000303004 and ENST00000371639	ENST00000371639
MIR1302-5	20	50601213	50601469	S18_peak_2004	Intergenic between ENST00000385283 and ENST00000408164	ENST00000408164
RPL36P2	20	50631591	50631756	S18_peak_2005	Intergenic between ENST00000426026 and ENST00000452336	ENST00000426026
PARD6B	20	50766856	50767042	S18_peak_2006	Intergenic between ENST00000371610 and ENST00000371608	ENST00000371610
BCAS4	20	50779149	50779314	S18_peak_2007	Intergenic between ENST00000371610 and	ENST00000371608

					ENST00000371608	
NFATC2	20	51295780	51295965	S18_peak_2008	Intergenic between ENST00000421328 and ENST00000371564	ENST00000371564
NFATC2	20	51314326	51314521	S18_peak_2009	Intergenic between ENST00000421328 and ENST00000371564	ENST00000371564
RP11-96F8.1	10	39620950	39621295	S18_peak_201	Intergenic between ENST00000427864 and ENST00000446298	ENST00000427864
NFATC2	20	51457794	51458013	S18_peak_2010	intron (ENST00000371564, intron 5 of 10)	ENST00000371564
ATP9A	20	51658038	51658203	S18_peak_2011	intron (ENST00000338821, intron 13 of 27)	ENST00000338821
LINC01429	20	51868586	51868751	S18_peak_2012	Intergenic between ENST00000438154 and ENST00000516658	ENST00000438154
ZFP64	20	52018831	52019078	S18_peak_2013	Intergenic between ENST00000516845 and ENST00000361387	ENST00000361387
ZFP64	20	52131313	52131478	S18_peak_2014	intron (ENST00000361387, intron 5 of 8)	ENST00000361387
RP11-80K6.2	20	52839250	52839415	S18_peak_2015	Intergenic between ENST00000422666 and ENST00000426963	ENST00000426963
RN7SKP184	20	53040643	53040920	S18_peak_2016	Intergenic between ENST00000419720 and ENST00000362348	ENST00000362348
RN7SKP184	20	53083496	53083661	S18_peak_2017	Intergenic between ENST00000419720 and ENST00000362348	ENST00000362348
RN7SKP184	20	53088844	53089009	S18_peak_2018	Intergenic between ENST00000419720 and ENST00000362348	ENST00000362348
RN7SKP184	20	53106596	53106761	S18_peak_2019	Intergenic between ENST00000362348 and ENST00000432706	ENST00000362348
RP11-96F8.1	10	40135150	40135315	S18_peak_202	Intergenic between ENST00000427864 and ENST00000446298	ENST00000427864
RP4-678D15.1	20	53368067	53368300	S18_peak_2020	intron (ENST00000606932, intron 1 of 2)	ENST00000606932
RP5-823G15.5	20	53496466	53496748	S18_peak_2021	intron (ENST00000558738, intron 1 of 3)	ENST00000558738
AC006076.1	20	53752650	53752818	S18_peak_2022	Intergenic between ENST00000459331 and ENST00000423233	ENST00000423233
AL133335.1	20	54362601	54362766	S18_peak_2023	Intergenic between ENST00000624441 and ENST00000262593	ENST00000624441
DOK5	20	54609167	54609332	S18_peak_2024	intron (ENST00000262593, intron 5 of 7)	ENST00000262593
LINC01440	20	55283256	55283421	S18_peak_2025	Intergenic between ENST00000432629 and ENST00000627529	ENST00000627529
RP5-837G17.1	20	55510140	55510305	S18_peak_2026	Intergenic between ENST00000622780 and ENST00000064571	ENST00000622780
RP5-837G17.1	20	55586441	55586606	S18_peak_2027	Intergenic between ENST00000622780 and ENST00000064571	ENST00000622780
CBLN4	20	56073638	56073803	S18_peak_2028	Intergenic between ENST00000064571 and ENST00000410186	ENST00000064571
RTFDC1	20	56507098	56507263	S18_peak_2029	intron (ENST00000357348, intron 5 of 8)	ENST00000357348
RP11-96F8.1	10	40344926	40345134	S18_peak_203	Intergenic between ENST00000427864 and ENST00000446298	ENST00000427864

TFAP2C	20	56627651	56627947	S18_peak_2030	Intergenic between ENST00000398541 and ENST00000201031	ENST00000201031
PTMAP6	20	56715465	56715630	S18_peak_2031	Intergenic between ENST00000439110 and ENST00000458293	ENST00000439110
MIR4532	20	57889213	57889464	S18_peak_2032	Intergenic between ENST00000614771 and ENST00000582082	ENST00000582082
PIEZO1P2	20	58745242	58745488	S18_peak_2033	intron (ENST00000416241, intron 11 of 13)	ENST00000416241
EDN3	20	59207632	59208014	S18_peak_2034	Intergenic between ENST00000445106 and ENST00000311585	ENST00000311585
EDN3	20	59303883	59304048	S18_peak_2035	intron (ENST00000311585, intron 2 of 4)	ENST00000311585
RP4-614C15.3	20	59369716	59369881	S18_peak_2036	Intergenic between ENST00000440889 and ENST00000485427	ENST00000440889
MIR646HG	20	60122280	60122573	S18_peak_2037	intron (ENST00000431181, intron 5 of 7)	ENST00000431181
MIR646HG	20	60319230	60319395	S18_peak_2038	intron (ENST00000432910, intron 4 of 4)	ENST00000432910
RP11-151E14.1	20	60823615	60823790	S18_peak_2039	Intergenic between ENST00000569070 and ENST00000420710	ENST00000569070
RP11-96F8.1	10	40390419	40390613	S18_peak_204	Intergenic between ENST00000427864 and ENST00000446298	ENST00000427864
WI2-8325B5.1	20	61272947	61273206	S18_peak_2040	Intergenic between ENST00000441660 and ENST00000617215	ENST00000617215
RP5-827E24.1	20	61419862	61420109	S18_peak_2041	Intergenic between ENST00000617215 and ENST00000615619	ENST00000615619
CDH4	20	61837021	61837300	S18_peak_2042	intron (ENST00000543233, intron 3 of 14)	ENST00000543233
SS18L1	20	62180723	62180926	S18_peak_2043	exon (ENST00000331758, exon 11 of 11)	ENST00000331758
HRH3	20	62210778	62210968	S18_peak_2044	Intergenic between ENST00000370823 and ENST00000340177	ENST00000340177
OSBPL2	20	62232416	62232607	S18_peak_2045	Intergenic between ENST00000340177 and ENST00000313733	ENST00000313733
OSBPL2	20	62242826	62243071	S18_peak_2046	intron (ENST00000313733, intron 1 of 13)	ENST00000313733
LAMA5	20	62364107	62364272	S18_peak_2047	intron (ENST00000252999, intron 1 of 79)	ENST00000252999
SLCO4A1-AS1	20	62663754	62663981	S18_peak_2048	intron (ENST00000433126, intron 3 of 3)	ENST00000433126
NTSR1	20	62706588	62706753	S18_peak_2049	Intergenic between ENST00000435412 and ENST00000370501	ENST00000370501
KSR1P1	10	41072342	41072536	S18_peak_205	Intergenic between ENST00000427864 and ENST00000446298	ENST00000446298
SLC17A9	20	62958561	62958762	S18_peak_2050	intron (ENST00000370351, intron 3 of 12)	ENST00000370351
KCNQ2	20	63469209	63469486	S18_peak_2051	intron (ENST00000626839, intron 1 of 15)	ENST00000626839
HELZ2	20	63559615	63559904	S18_peak_2052	intron (ENST00000467148, intron 18 of 18)	ENST00000467148
RTEL1-TNFRSF6B	20	63696133	63696408	S18_peak_2053	intron (ENST00000492259, intron 32 of 34)	ENST00000492259
RP4-591C20.9	20	63852819	63852987	S18_peak_2054	Intergenic between ENST00000623918 and ENST00000601296	ENST00000601296
TPD52L2	20	63868158	63868369	S18_peak_2055	intron (ENST00000346249, intron 1 of 6)	ENST00000346249
TPD52L2	20	63869479	63869668	S18_peak_2056	intron (ENST00000346249, intron 2 of 6)	ENST00000346249
TPD52L2	20	63870821	63870986	S18_peak_2057	intron (ENST00000346249, intron 2 of 6)	ENST00000346249

TPD52L2	20	63885863	63886028	S18_peak_2058	intron (ENST00000346249, intron 5 of 6)	ENST00000346249
OPRL1	20	64088696	64088874	S18_peak_2059	intron (ENST00000336866, intron 1 of 4)	ENST00000336866
KSR1P1	10	41516301	41516506	S18_peak_206	Intergenic between ENST00000427864 and ENST00000446298	ENST00000446298
MYT1	20	64223520	64223685	S18_peak_2060	intron (ENST00000360149, intron 14 of 15)	ENST00000360149
MYT1	20	64238500	64238736	S18_peak_2061	Intergenic between ENST00000360149 and ENST00000308824	ENST00000360149
RP11-717F1.1	21	5230380	5230793	S18_peak_2062	Intergenic between ENST00000623744 and ENST00000623753	ENST00000623753
CH507-513H4.1	21	8207773	8208024	S18_peak_2064	intron (ENST00000623664, intron 2 of 6)	ENST00000623664
CH507-513H4.1	21	8208802	8208973	S18_peak_2065	intron (ENST00000623664, intron 2 of 6)	ENST00000623664
CH507-513H4.1	21	8218827	8219131	S18_peak_2067	intron (ENST00000623664, intron 2 of 6)	ENST00000623664
CH507-513H4.1	21	8226079	8226405	S18_peak_2068	exon (ENST00000623664, exon 6 of 7)	ENST00000623664
pRNA	21	8251248	8251508	S18_peak_2069	Intergenic between ENST00000618423 and ENST00000629969	ENST00000618423
CH507-528H12.1	21	8395446	8395611	S18_peak_2070	intron (ENST00000623860, intron 2 of 6)	ENST00000623860
RNA5-8S5	21	8413404	8413621	S18_peak_2075	Intergenic between ENST00000613359 and ENST00000617390	ENST00000613359
BMS1	10	42788601	42788766	S18_peak_208	intron (ENST00000374518, intron 4 of 22)	ENST00000374518
bP-2189O9.3	21	8765054	8765219	S18_peak_2080	Intergenic between ENST00000624813 and ENST00000625014	ENST00000624813
bP-2171C21.3	21	8991817	8992518	S18_peak_2081	Intergenic between ENST00000577708 and ENST00000623236	ENST00000623236
bP-2171C21.3	21	9015293	9015564	S18_peak_2082	intron (ENST00000623236, intron 1 of 1)	ENST00000623236
bP-21264C1.2	21	9246215	9246389	S18_peak_2083	Intergenic between ENST00000623217 and ENST00000622961	ENST00000622961
bP-21264C1.2	21	9248607	9248839	S18_peak_2084	Intergenic between ENST00000623217 and ENST00000622961	ENST00000622961
bP-21264C1.2	21	9313410	9313589	S18_peak_2085	Intergenic between ENST00000623217 and ENST00000622961	ENST00000622961
bP-21264C1.2	21	9315551	9315719	S18_peak_2086	Intergenic between ENST00000623217 and ENST00000622961	ENST00000622961
U1	21	9644424	9644712	S18_peak_2087	Intergenic between ENST00000623408 and ENST00000620684	ENST00000620684
U1	21	9676741	9676906	S18_peak_2088	Intergenic between ENST00000620684 and ENST00000623554	ENST00000620684
MGC39584	21	9740571	9740856	S18_peak_2089	Intergenic between ENST00000620684 and ENST00000623554	ENST00000623554
RP11-168L22.2	10	43323594	43323863	S18_peak_209	promoter-TSS (ENST00000451438)	ENST00000451438
RN7SL52P	21	9881620	9881785	S18_peak_2090	Intergenic between ENST00000624130 and ENST00000584271	ENST00000584271
IGHV1OR21-1	21	10728057	10728293	S18_peak_2091	Intergenic between ENST00000622028 and ENST00000457565	ENST00000622028
IGHV1OR21-1	21	10778403	10778612	S18_peak_2092	Intergenic between ENST00000622028 and	ENST00000622028

					ENST00000457565	
AP001464.4	21	11968916	11969090	S18_peak_2093	Intergenic between ENST00000622028 and ENST00000457565	ENST00000457565
AP001464.4	21	12106231	12106396	S18_peak_2094	Intergenic between ENST00000622028 and ENST00000457565	ENST00000457565
AP001464.4	21	12169110	12169275	S18_peak_2095	Intergenic between ENST00000622028 and ENST00000457565	ENST00000457565
AP001464.4	21	12607374	12607542	S18_peak_2096	Intergenic between ENST00000622028 and ENST00000457565	ENST00000457565
AP001464.4	21	12616591	12616812	S18_peak_2097	Intergenic between ENST00000622028 and ENST00000457565	ENST00000457565
AP001464.4	21	12744148	12744324	S18_peak_2098	Intergenic between ENST00000622028 and ENST00000457565	ENST00000457565
RBM11	21	14244944	14245109	S18_peak_2099	Intergenic between ENST00000400577 and ENST00000467409	ENST00000400577
AKR7L	1	19264700	19264865	S18_peak_21	Intergenic between ENST00000330263 and ENST00000457194	ENST00000457194
RP11-517P14.7	10	43490808	43491079	S18_peak_210	Intergenic between ENST00000516723 and ENST00000421913	ENST00000421913
SAMSN1	21	14580852	14581017	S18_peak_2100	intron (ENST00000285670, intron 2 of 8)	ENST00000285670
AJ006998.2	21	15442908	15443073	S18_peak_2101	intron (ENST00000634644, intron 8 of 11)	ENST00000634644
USP25	21	15722595	15722826	S18_peak_2102	Intergenic between ENST00000454157 and ENST00000285681	ENST00000285681
MIR99AHG	21	16007131	16007296	S18_peak_2103	intron (ENST00000635845, intron 1 of 5)	ENST00000635845
NEK4P1	21	17067225	17067441	S18_peak_2104	Intergenic between ENST00000430064 and ENST00000433383	ENST00000433383
NEK4P1	21	17183931	17184179	S18_peak_2105	Intergenic between ENST00000430064 and ENST00000433383	ENST00000433383
AP000457.2	21	17259343	17259547	S18_peak_2106	Intergenic between ENST00000433383 and ENST00000620221	ENST00000620221
RNU6-113P	21	17392616	17392781	S18_peak_2107	Intergenic between ENST00000620221 and ENST00000516653	ENST00000516653
TMPRSS15	21	18410297	18410462	S18_peak_2108	Intergenic between ENST00000284885 and ENST00000455391	ENST00000284885
KRT18P2	21	20411645	20411810	S18_peak_2109	Intergenic between ENST00000425091 and ENST00000447938	ENST00000447938
RP11-20J15.2	10	44131332	44131614	S18_peak_211	Intergenic between ENST00000451929 and ENST00000437014	ENST00000437014
AP000959.2	21	22587714	22587879	S18_peak_2110	Intergenic between ENST00000441759 and ENST00000438328	ENST00000441759
AP000476.1	21	24607738	24607961	S18_peak_2111	Intergenic between ENST00000415182 and ENST00000441009	ENST00000415182
APP	21	25903314	25903534	S18_peak_2112	intron (ENST00000348990, intron 13 of 15)	ENST00000348990
MIR4759	21	26987584	26987814	S18_peak_2113	Intergenic between ENST00000584048 and	ENST00000584048

					ENST00000429271	
MIR4759	21	27013844	27014075	S18_peak_2114	Intergenic between ENST00000584048 and ENST00000429271	ENST00000584048
LINC00113	21	27703951	27704116	S18_peak_2115	Intergenic between ENST00000426418 and ENST00000411460	ENST00000411460
MAP3K7CL	21	29097002	29097167	S18_peak_2116	intron (ENST00000339024, intron 1 of 6)	ENST00000339024
GRIK1	21	29769646	29769881	S18_peak_2117	intron (ENST00000327783, intron 1 of 17)	ENST00000327783
TPT1P1	21	31791933	31792122	S18_peak_2118	Intergenic between ENST00000610276 and ENST00000458495	ENST00000458495
MRAP	21	32302055	32302220	S18_peak_2119	intron (ENST00000303645, intron 1 of 2)	ENST00000303645
RP11-733D4.2	10	44452317	44452551	S18_peak_212	Intergenic between ENST00000392269 and ENST00000605122	ENST00000605122
EVA1C	21	32511070	32511235	S18_peak_2120	intron (ENST00000300255, intron 7 of 7)	ENST00000300255
RNA5SP490	21	32561076	32561241	S18_peak_2121	Intergenic between ENST00000516236 and ENST00000517141	ENST00000517141
SYNJ1	21	32631255	32631526	S18_peak_2122	exon (ENST00000382499, exon 33 of 33)	ENST00000382499
AP000289.6	21	33121529	33121794	S18_peak_2123	intron (ENST00000421051, intron 1 of 2)	ENST00000421051
AP000320.7	21	34301486	34301651	S18_peak_2124	intron (ENST00000362077, intron 5 of 5)	ENST00000362077
HLCS	21	36850353	36850518	S18_peak_2125	intron (ENST00000336648, intron 7 of 11)	ENST00000336648
TTC3	21	37186542	37186790	S18_peak_2126	intron (ENST00000399017, intron 37 of 45)	ENST00000399017
KCNJ6	21	37630124	37630328	S18_peak_2127	intron (ENST00000609713, intron 3 of 3)	ENST00000609713
AF064858.6	21	38946704	38947048	S18_peak_2128	intron (ENST00000415824, intron 1 of 3)	ENST00000415824
SH3BGR	21	39440149	39440314	S18_peak_2129	Intergenic between ENST00000380708 and ENST00000380637	ENST00000380637
TMEM72-AS1	10	44915058	44915223	S18_peak_213	intron (ENST00000450287, intron 2 of 7)	ENST00000450287
SH3BGR	21	39498929	39499094	S18_peak_2130	intron (ENST00000380637, intron 3 of 6)	ENST00000380637
B3GALT5	21	39619129	39619364	S18_peak_2131	intron (ENST00000380620, intron 2 of 4)	ENST00000380620
LINC00323	21	41085323	41085505	S18_peak_2132	Intergenic between ENST00000456507 and ENST00000441268	ENST00000441268
FAM3B	21	41308719	41308884	S18_peak_2133	Intergenic between ENST00000414699 and ENST00000357985	ENST00000357985
RSPH1	21	42474461	42474755	S18_peak_2134	intron (ENST00000291536, intron 8 of 8)	ENST00000291536
CRYAA	21	43191640	43191868	S18_peak_2135	Intergenic between ENST00000291554 and ENST00000450205	ENST00000291554
RRP1B	21	43692297	43692532	S18_peak_2136	intron (ENST00000340648, intron 15 of 15)	ENST00000340648
RNU6-1150P	21	43999039	43999244	S18_peak_2137	Intergenic between ENST00000516797 and ENST00000380221	ENST00000516797
LINC00163	21	44987261	44987426	S18_peak_2138	Intergenic between ENST00000616815 and ENST00000439088	ENST00000439088
LINC00165	21	44995108	44995394	S18_peak_2139	promoter-TSS (ENST00000569966)	ENST00000569966
RASSF4	10	44986764	44987001	S18_peak_214	intron (ENST00000340258, intron 6 of 10)	ENST00000340258
LINC00334	21	45243691	45243858	S18_peak_2140	intron (ENST00000584169, intron 2 of 2)	ENST00000584169
AL592528.1	21	45827337	45827652	S18_peak_2141	TTS (ENST00000380008)	ENST00000380008
DIP2A	21	46554332	46554624	S18_peak_2142	intron (ENST00000417564, intron 26 of 37)	ENST00000417564

DIP2A	21	46558221	46558386	S18_peak_2143	exon (ENST00000417564, exon 32 of 38)	ENST00000417564
DIP2A	21	46559407	46559921	S18_peak_2144	intron (ENST00000417564, intron 32 of 37)	ENST00000417564
DIP2A	21	46563488	46563741	S18_peak_2145	intron (ENST00000417564, intron 34 of 37)	ENST00000417564
DIP2A	21	46563894	46564145	S18_peak_2146	intron (ENST00000417564, intron 35 of 37)	ENST00000417564
DIP2A	21	46564856	46565475	S18_peak_2147	intron (ENST00000417564, intron 35 of 37)	ENST00000417564
DIP2A	21	46565667	46565838	S18_peak_2148	exon (ENST00000417564, exon 36 of 38)	ENST00000417564
DIP2A	21	46566502	46566732	S18_peak_2149	exon (ENST00000417564, exon 37 of 38)	ENST00000417564
8-Mar	10	45468810	45469002	S18_peak_215	intron (ENST00000453424, intron 3 of 7)	ENST00000453424
U2	22	10667056	10667221	S18_peak_2150	Intergenic before ENST00000615943	ENST00000615943
U2	22	10680491	10680656	S18_peak_2151	Intergenic before ENST00000615943	ENST00000615943
FRG1FP	22	10954369	10954656	S18_peak_2152	intron (ENST00000635667, intron 2 of 8)	ENST00000635667
5_8S_rRNA	22	11804790	11804955	S18_peak_2154	Intergenic between ENST00000612732 and ENST00000634617	ENST00000612732
FRG1GP	22	12037621	12037786	S18_peak_2155	Intergenic between ENST00000612732 and ENST00000634617	ENST00000634617
FRG1GP	22	12183328	12183493	S18_peak_2156	Intergenic between ENST00000612732 and ENST00000634617	ENST00000634617
FRG1GP	22	12498120	12498405	S18_peak_2157	Intergenic between ENST00000612732 and ENST00000634617	ENST00000634617
FRG1GP	22	12513770	12514003	S18_peak_2158	Intergenic between ENST00000612732 and ENST00000634617	ENST00000634617
FRG1GP	22	12900471	12900636	S18_peak_2159	Intergenic between ENST00000634617 and ENST00000613107	ENST00000634617
AGAP10P	10	45691343	45691675	S18_peak_216	intron (ENST00000454844, intron 3 of 6)	ENST00000454844
LA16c-60G3.6	22	15558630	15558833	S18_peak_2160	intron (ENST00000412729, intron 2 of 3)	ENST00000412729
POTEH	22	15682244	15682429	S18_peak_2161	Intergenic between ENST00000435410 and ENST00000343518	ENST00000343518
POTEH	22	15716790	15716955	S18_peak_2162	intron (ENST00000343518, intron 9 of 10)	ENST00000343518
DUXAP8	22	15804360	15804558	S18_peak_2163	intron (ENST00000413768, intron 6 of 7)	ENST00000413768
U6	22	16256326	16256491	S18_peak_2164	Intergenic between ENST00000612550 and ENST00000399687	ENST00000612550
KB-67B5.12	22	16553935	16554100	S18_peak_2165	Intergenic between ENST00000609641 and ENST00000454360	ENST00000454360
XKR3	22	16813065	16813323	S18_peak_2166	intron (ENST00000331428, intron 1 of 3)	ENST00000331428
IL17RA	22	17091556	17091721	S18_peak_2167	intron (ENST00000319363, intron 1 of 12)	ENST00000319363
CECR3	22	17272813	17273150	S18_peak_2168	Intergenic between ENST00000428828 and ENST00000453421	ENST00000428828
RN7SL843P	22	17352300	17352515	S18_peak_2169	TTS (ENST00000485262)	ENST00000485262
FAM21C	10	45796379	45796688	S18_peak_217	Intergenic between ENST00000374362 and ENST00000603431	ENST00000374362
AC004019.10	22	17395186	17395385	S18_peak_2170	Intergenic between ENST00000485262 and ENST00000455617	ENST00000455617
PEX26	22	18091496	18091687	S18_peak_2171	exon (ENST00000329627, exon 6 of 6)	ENST00000329627
AC008079.10	22	18109838	18110003	S18_peak_2172	TTS (ENST00000623543)	ENST00000623543

USP18	22	18176805	18177044	S18_peak_2173	exon (ENST00000215794, exon 11 of 11)	ENST00000215794
GGT3P	22	18773842	18774212	S18_peak_2174	intron (ENST00000412448, intron 14 of 14)	ENST00000412448
DGCR5	22	18994797	18995037	S18_peak_2175	intron (ENST00000440005, intron 4 of 5)	ENST00000440005
XXbac-B33L19.4	22	20364462	20364700	S18_peak_2176	Intergenic between ENST00000458154 and ENST00000400451	ENST00000458154
PI4KA	22	20808379	20808544	S18_peak_2177	intron (ENST00000255882, intron 9 of 54)	ENST00000255882
PI4KA	22	20847965	20848218	S18_peak_2178	intron (ENST00000255882, intron 1 of 54)	ENST00000255882
KB-1592A4.16	22	21213003	21213202	S18_peak_2179	Intergenic between ENST00000417463 and ENST00000637734	ENST00000637734
FAM21C	10	45797389	45797554	S18_peak_218	Intergenic between ENST00000374362 and ENST00000603431	ENST00000374362
TOP3B	22	21982385	21982550	S18_peak_2180	promoter-TSS (ENST00000398793)	ENST00000398793
ADORA2A-AS1	22	24472044	24472209	S18_peak_2181	intron (ENST00000326341, intron 2 of 6)	ENST00000326341
GGT1	22	24598614	24598779	S18_peak_2182	intron (ENST00000248923, intron 1 of 15)	ENST00000248923
CTA-796E4.4	22	26164817	26164982	S18_peak_2183	Intergenic between ENST00000434510 and ENST00000360929	ENST00000434510
MIAT	22	26675297	26675514	S18_peak_2184	exon (ENST00000616469, exon 4 of 4)	ENST00000616469
RP1-40G4P.1	22	26876988	26877153	S18_peak_2185	Intergenic between ENST00000434868 and ENST00000422915	ENST00000422915
THOC5	22	29519653	29519818	S18_peak_2186	intron (ENST00000490103, intron 14 of 19)	ENST00000490103
OSBP2	22	30905422	30905609	S18_peak_2187	intron (ENST00000332585, intron 12 of 13)	ENST00000332585
PATZ1	22	31367000	31367270	S18_peak_2188	Intergenic between ENST00000215919 and ENST00000383888	ENST00000215919
EIF4ENIF1	22	31471262	31471427	S18_peak_2189	intron (ENST00000344710, intron 3 of 16)	ENST00000344710
AGAP4	10	45824596	45824897	S18_peak_219	TTS (ENST00000448048)	ENST00000448048
PISD	22	31642756	31642921	S18_peak_2190	intron (ENST00000439502, intron 3 of 7)	ENST00000439502
SOX10	22	37990645	37990978	S18_peak_2191	Intergenic between ENST00000396884 and ENST00000410099	ENST00000396884
RP1-199H16.6	22	38577215	38577380	S18_peak_2192	Intergenic between ENST00000446147 and ENST00000216029	ENST00000446147
JOSD1	22	38690501	38690666	S18_peak_2193	intron (ENST00000216039, intron 1 of 3)	ENST00000216039
DNAL4	22	38795489	38795705	S18_peak_2194	Intergenic between ENST00000216068 and ENST00000333039	ENST00000216068
L3MBTL2	22	41224677	41224842	S18_peak_2195	exon (ENST00000216237, exon 10 of 17)	ENST00000216237
ZC3H7B	22	41303734	41303899	S18_peak_2196	intron (ENST00000352645, intron 1 of 22)	ENST00000352645
SREBF2	22	41907273	41907522	S18_peak_2197	TTS (ENST00000361204)	ENST00000361204
OGFRP1	22	42272423	42272699	S18_peak_2198	intron (ENST00000332965, intron 1 of 1)	ENST00000332965
Y_RNA	22	42756754	42756939	S18_peak_2199	Intergenic between ENST00000401850 and ENST00000363578	ENST00000363578
EIF4G3	1	20910390	20910555	S18_peak_22	intron (ENST00000602326, intron 12 of 34)	ENST00000602326
ANXA8L1	10	46391669	46391889	S18_peak_220	exon (ENST00000622769, exon 9 of 9)	ENST00000622769
PACSIN2	22	42926757	42926983	S18_peak_2200	intron (ENST00000263246, intron 1 of 10)	ENST00000263246
TTLL1	22	43054391	43054556	S18_peak_2201	intron (ENST00000266254, intron 8 of 10)	ENST00000266254
TTLL1	22	43072844	43073056	S18_peak_2202	intron (ENST00000266254, intron 3 of 10)	ENST00000266254

SULT4A1	22	43874911	43875187	S18_peak_2203	Intergenic between ENST00000330884 and ENST00000216180	ENST00000330884
PNPLA3	22	43914911	43915194	S18_peak_2204	Intergenic between ENST00000330884 and ENST00000216180	ENST00000216180
PHF21B	22	44929468	44929633	S18_peak_2205	intron (ENST00000313237, intron 2 of 12)	ENST00000313237
CTA-268H5.9	22	45270368	45270533	S18_peak_2206	Intergenic between ENST00000609206 and ENST00000445867	ENST00000445867
RP3-439F8.1	22	46603055	46603276	S18_peak_2207	Intergenic between ENST00000426112 and ENST00000216264	ENST00000426112
TBC1D22A	22	46991801	46992086	S18_peak_2208	intron (ENST00000355704, intron 7 of 10)	ENST00000355704
TBC1D22A	22	47019866	47020109	S18_peak_2209	intron (ENST00000355704, intron 8 of 10)	ENST00000355704
HNRNPA1P33	10	46436691	46436856	S18_peak_221	Intergenic between ENST00000421426 and ENST00000612632	ENST00000421426
FAM19A5	22	48527329	48527552	S18_peak_2210	intron (ENST00000402357, intron 1 of 3)	ENST00000402357
MIR4535	22	48789069	48789234	S18_peak_2211	Intergenic between ENST00000580946 and ENST00000380981	ENST00000580946
RP1-34P24.3	22	49282729	49282894	S18_peak_2212	Intergenic between ENST00000623446 and ENST00000625038	ENST00000623446
MIR6821	22	49977504	49977700	S18_peak_2213	Intergenic between ENST00000617625 and ENST00000433387	ENST00000617625
PANX2	22	50179768	50180057	S18_peak_2214	exon (ENST00000395842, exon 3 of 3)	ENST00000395842
TRABD	22	50197948	50198125	S18_peak_2215	intron (ENST00000303434, intron 8 of 9)	ENST00000303434
SELO	22	50204686	50204966	S18_peak_2216	intron (ENST00000380903, intron 1 of 8)	ENST00000380903
DENND6B	22	50298168	50298454	S18_peak_2217	Intergenic between ENST00000330651 and ENST00000413817	ENST00000413817
PPP6R2	22	50382735	50382919	S18_peak_2218	intron (ENST00000395741, intron 2 of 22)	ENST00000395741
LINC01266	3	661388	661660	S18_peak_2219	intron (ENST00000420823, intron 2 of 4)	ENST00000420823
RN7SL248P	10	46678153	46678336	S18_peak_222	Intergenic between ENST00000615117 and ENST00000628708	ENST00000615117
LINC01266	3	701686	701851	S18_peak_2220	intron (ENST00000420823, intron 2 of 4)	ENST00000420823
RPL23AP38	3	1546831	1546996	S18_peak_2221	Intergenic between ENST00000449338 and ENST00000423801	ENST00000423801
HINT2P1	3	2630122	2630287	S18_peak_2222	Intergenic between ENST00000419438 and ENST00000440115	ENST00000419438
HINT2P1	3	2632668	2632833	S18_peak_2223	Intergenic between ENST00000419438 and ENST00000440115	ENST00000419438
DNAJC19P4	3	2767054	2767219	S18_peak_2224	Intergenic between ENST00000419438 and ENST00000440115	ENST00000440115
DNAJC19P4	3	2851214	2851379	S18_peak_2225	Intergenic between ENST00000419438 and ENST00000440115	ENST00000440115
RP11-97C16.1	3	3199377	3199542	S18_peak_2226	Intergenic between ENST00000607052 and ENST00000420000	ENST00000607052
AC026188.1	3	3325125	3325290	S18_peak_2227	intron (ENST00000420000, intron 4 of 4)	ENST00000420000
ITPR1	3	4651370	4651569	S18_peak_2228	intron (ENST00000357086, intron 10 of 58)	ENST00000357086

ITPR1	3	4700430	4700595	S18_peak_2229	intron (ENST00000357086, intron 35 of 58)	ENST00000357086
FRMPD2B	10	46867059	46867231	S18_peak_223	Intergenic between ENST00000417888 and ENST00000431305	ENST00000431305
AC026202.5	3	5445262	5445427	S18_peak_2230	Intergenic between ENST00000443087 and ENST00000425894	ENST00000443087
GRM7-AS1	3	7558874	7559039	S18_peak_2231	promoter-TSS (ENST00000427273)	ENST00000427273
LMCD1-AS1	3	8209289	8209454	S18_peak_2232	intron (ENST00000446281, intron 3 of 5)	ENST00000446281
LMCD1-AS1	3	8609097	8609276	S18_peak_2233	intron (ENST00000439407, intron 2 of 3)	ENST00000439407
RP11-380O24.1	3	9282966	9283290	S18_peak_2234	Intergenic between ENST00000449023 and ENST00000491930	ENST00000491930
DUSP5P2	3	9578081	9578442	S18_peak_2235	Intergenic between ENST00000287585 and ENST00000426873	ENST00000426873
DUSP5P2	3	9617877	9618137	S18_peak_2236	Intergenic between ENST00000426873 and ENST00000353332	ENST00000426873
MTMR14	3	9701741	9702016	S18_peak_2237	exon (ENST00000353332, exon 18 of 18)	ENST00000353332
VHL	3	10151677	10151892	S18_peak_2238	exon (ENST00000256474, exon 3 of 3)	ENST00000256474
TATDN2	3	10271665	10271830	S18_peak_2239	intron (ENST00000448281, intron 4 of 7)	ENST00000448281
PTPN20	10	46928820	46929072	S18_peak_224	intron (ENST00000374342, intron 1 of 3)	ENST00000374342
TAMM41	3	11815127	11815293	S18_peak_2240	intron (ENST00000455809, intron 5 of 7)	ENST00000455809
GSTM5P1	3	12269080	12269255	S18_peak_2241	Intergenic between ENST00000434751 and ENST00000309576	ENST00000434751
RAF1	3	12655159	12655382	S18_peak_2242	intron (ENST00000251849, intron 1 of 16)	ENST00000251849
CAND2	3	12804584	12804961	S18_peak_2243	intron (ENST00000295989, intron 2 of 12)	ENST00000295989
SNORA7A	3	12842117	12842282	S18_peak_2244	Intergenic between ENST00000384765 and ENST00000502828	ENST00000384765
AC027124.2	3	13464827	13464992	S18_peak_2245	Intergenic between ENST00000440852 and ENST00000424112	ENST00000440852
LINC00620	3	13719548	13719775	S18_peak_2246	intron (ENST00000438915, intron 1 of 1)	ENST00000438915
TPRXL	3	13945071	13945236	S18_peak_2247	intron (ENST00000532880, intron 1 of 4)	ENST00000532880
RP11-536I6.2	3	14287408	14287573	S18_peak_2248	intron (ENST00000525575, intron 1 of 1)	ENST00000525575
FGD5	3	14910773	14910938	S18_peak_2249	intron (ENST00000285046, intron 10 of 19)	ENST00000285046
CH17-360D5.2	10	47929105	47929270	S18_peak_225	intron (ENST00000479781, intron 2 of 2)	ENST00000479781
ANKRD28	3	15791302	15791477	S18_peak_2250	intron (ENST00000399451, intron 2 of 27)	ENST00000399451
AC090945.1	3	15893918	15894126	S18_peak_2251	Intergenic between ENST00000413639 and ENST00000339732	ENST00000413639
GALNT15	3	16077445	16077764	S18_peak_2252	Intergenic between ENST00000413639 and ENST00000339732	ENST00000339732
GALNT15	3	16135515	16135680	S18_peak_2253	Intergenic between ENST00000413639 and ENST00000339732	ENST00000339732
AC091493.2	3	16697433	16697598	S18_peak_2254	promoter-TSS (ENST00000426218)	ENST00000426218
PLCL2	3	16805738	16805905	S18_peak_2255	Intergenic between ENST00000419017 and ENST00000615277	ENST00000615277
PLCL2	3	17008803	17009208	S18_peak_2256	intron (ENST00000615277, intron 1 of 5)	ENST00000615277
TBC1D5	3	17554660	17554994	S18_peak_2257	intron (ENST00000253692, intron 2 of 21)	ENST00000253692

KCNH8	3	19455710	19455892	S18_peak_2258	intron (ENST00000328405, intron 10 of 15)	ENST00000328405
SGO1	3	20172143	20172402	S18_peak_2259	intron (ENST00000421451, intron 6 of 8)	ENST00000421451
PTPN20CP	10	48085712	48085909	S18_peak_226	intron (ENST00000614090, intron 5 of 6)	ENST00000614090
RPL24P7	3	23085650	23085815	S18_peak_2260	Intergenic between ENST00000419001 and ENST00000399435	ENST00000399435
UBE2E2-AS1	3	23201078	23201243	S18_peak_2261	promoter-TSS (ENST00000452251)	ENST00000452251
UBE2E2-AS1	3	23223259	23223424	S18_peak_2262	Intergenic between ENST00000430018 and ENST00000384001	ENST00000430018
AC121251.1	3	23346094	23346513	S18_peak_2263	Intergenic between ENST00000384001 and ENST00000451481	ENST00000451481
UBE2E1	3	23811365	23811530	S18_peak_2264	intron (ENST00000306627, intron 2 of 5)	ENST00000306627
AC133680.1	3	24810803	24811028	S18_peak_2265	intron (ENST00000455576, intron 2 of 5)	ENST00000455576
AC133680.1	3	24900387	24900552	S18_peak_2266	intron (ENST00000455576, intron 4 of 5)	ENST00000455576
RNA5SP126	3	25386540	25386757	S18_peak_2267	Intergenic between ENST00000365092 and ENST00000330688	ENST00000365092
RARB	3	25479740	25479905	S18_peak_2268	intron (ENST00000330688, intron 2 of 7)	ENST00000330688
RARB	3	25531588	25531753	S18_peak_2269	intron (ENST00000330688, intron 3 of 7)	ENST00000330688
C10orf71-AS1	10	49275068	49275330	S18_peak_227	Intergenic between ENST00000453436 and ENST00000437677	ENST00000437677
CMC1	3	28291979	28292144	S18_peak_2270	intron (ENST00000466830, intron 2 of 3)	ENST00000466830
RP11-9J18.1	3	29226550	29226715	S18_peak_2271	intron (ENST00000635992, intron 8 of 13)	ENST00000635992
TGFBR2	3	30646687	30646852	S18_peak_2272	intron (ENST00000295754, intron 2 of 6)	ENST00000295754
OSBPL10	3	31803186	31803354	S18_peak_2273	intron (ENST00000396556, intron 4 of 11)	ENST00000396556
OSBPL10	3	31824621	31824870	S18_peak_2274	intron (ENST00000396556, intron 4 of 11)	ENST00000396556
RP11-384L8.1	3	32234967	32235132	S18_peak_2275	Intergenic between ENST00000443010 and ENST00000565519	ENST00000565519
CMTM8	3	32262260	32262472	S18_peak_2276	intron (ENST00000307526, intron 1 of 3)	ENST00000307526
CMTM8	3	32277008	32277173	S18_peak_2277	intron (ENST00000307526, intron 1 of 3)	ENST00000307526
AC104306.4	3	32604928	32605093	S18_peak_2278	Intergenic between ENST00000451877 and ENST00000447449	ENST00000451877
CNOT10	3	32746527	32746692	S18_peak_2279	intron (ENST00000331889, intron 12 of 17)	ENST00000331889
ERCC6	10	49519486	49519735	S18_peak_228	intron (ENST00000355832, intron 5 of 20)	ENST00000355832
CLASP2	3	33651652	33651842	S18_peak_2280	intron (ENST00000480013, intron 1 of 32)	ENST00000480013
SDAD1P3	3	33770100	33770265	S18_peak_2281	Intergenic between ENST00000434628 and ENST00000427380	ENST00000427380
AC018359.1	3	34059029	34059194	S18_peak_2282	Intergenic between ENST00000605513 and ENST00000424786	ENST00000424786
ARPP21	3	35585287	35585538	S18_peak_2283	Intergenic between ENST00000391140 and ENST00000428373	ENST00000428373
DCLK3	3	36745752	36745932	S18_peak_2284	Intergenic between ENST00000416516 and ENST00000388967	ENST00000416516
LRRFIP2	3	37059972	37060137	S18_peak_2285	intron (ENST00000336686, intron 24 of 27)	ENST00000336686
LRRFIP2	3	37105775	37105940	S18_peak_2286	intron (ENST00000336686, intron 13 of 27)	ENST00000336686
RNA5SP129	3	37379786	37379980	S18_peak_2287	Intergenic between ENST00000410276 and	ENST00000410276

					ENST00000264741	
ITGA9	3	37544954	37545144	S18_peak_2288	intron (ENST00000264741, intron 15 of 27)	ENST00000264741
XYLB	3	38339934	38340099	S18_peak_2289	Intergenic between ENST00000427139 and ENST00000207870	ENST00000207870
RP11-140C5.3	10	49574191	49574362	S18_peak_229	Intergenic between ENST00000605814 and ENST00000339797	ENST00000605814
TTC21A	3	39124884	39125049	S18_peak_2290	intron (ENST00000431162, intron 9 of 28)	ENST00000431162
MYRIP	3	40073788	40073953	S18_peak_2291	intron (ENST00000444716, intron 3 of 16)	ENST00000444716
RP11-761N21.1	3	40820879	40821044	S18_peak_2292	intron (ENST00000412811, intron 2 of 2)	ENST00000412811
RP11-372H2.1	3	41146630	41146795	S18_peak_2293	Intergenic between ENST00000453828 and ENST00000415774	ENST00000415774
ULK4	3	41311460	41311625	S18_peak_2294	intron (ENST00000301831, intron 35 of 36)	ENST00000301831
RPL36P20	3	41964419	41964694	S18_peak_2295	Intergenic between ENST00000423702 and ENST00000418195	ENST00000423702
RP4-613B23.5	3	42792674	42792839	S18_peak_2296	intron (ENST00000431549, intron 2 of 4)	ENST00000431549
ABHD5	3	43653017	43653182	S18_peak_2297	Intergenic between ENST00000413663 and ENST00000458276	ENST00000458276
ABHD5	3	43703228	43703393	S18_peak_2298	intron (ENST00000458276, intron 3 of 6)	ENST00000458276
TCAIM	3	44353826	44354118	S18_peak_2299	intron (ENST00000417237, intron 1 of 10)	ENST00000417237
SRRM1	1	24676253	24676418	S18_peak_23	Intergenic between ENST00000323848 and ENST00000374379	ENST00000323848
CHAT	10	49617079	49617304	S18_peak_230	intron (ENST00000339797, intron 2 of 14)	ENST00000339797
LIMD1	3	45553414	45553620	S18_peak_2300	Intergenic between ENST00000442534 and ENST00000273317	ENST00000273317
LZTFL1	3	45856003	45856358	S18_peak_2301	Intergenic between ENST00000296135 and ENST00000425889	ENST00000296135
FLT1P1	3	46163782	46163978	S18_peak_2302	Intergenic between ENST00000451650 and ENST00000296140	ENST00000451650
TDGF1	3	46577058	46577320	S18_peak_2303	promoter-TSS (ENST00000296145)	ENST00000296145
RN7SL870P	3	47544328	47544493	S18_peak_2304	Intergenic between ENST00000603861 and ENST00000460111	ENST00000460111
MAP4	3	47924888	47925087	S18_peak_2305	intron (ENST00000395734, intron 4 of 17)	ENST00000395734
MAP4	3	47975449	47975735	S18_peak_2306	intron (ENST00000395734, intron 3 of 17)	ENST00000395734
CDC25A	3	48176824	48176989	S18_peak_2307	intron (ENST00000302506, intron 8 of 14)	ENST00000302506
Y_RNA	3	48282786	48282951	S18_peak_2308	Intergenic between ENST00000414458 and ENST00000362606	ENST00000362606
MIR2115	3	48330839	48331074	S18_peak_2309	Intergenic between ENST00000516657 and ENST00000296438	ENST00000516657
PARG	10	49925957	49926127	S18_peak_231	intron (ENST00000616448, intron 4 of 17)	ENST00000616448
BSN	3	49585685	49585850	S18_peak_2310	intron (ENST00000296452, intron 1 of 11)	ENST00000296452
BSN	3	49589715	49589880	S18_peak_2311	intron (ENST00000296452, intron 1 of 11)	ENST00000296452
MON1A	3	49930281	49930479	S18_peak_2312	Intergenic between ENST00000455683 and ENST00000442092	ENST00000455683
RBM6	3	50038694	50038859	S18_peak_2313	intron (ENST00000442092, intron 2 of 16)	ENST00000442092

C3orf18	3	50561755	50561932	S18_peak_2314	intron (ENST00000426034, intron 2 of 4)	ENST00000426034
DOCK3	3	50697411	50697576	S18_peak_2315	intron (ENST00000266037, intron 1 of 52)	ENST00000266037
PPM1M	3	52247493	52247658	S18_peak_2316	promoter-TSS (ENST00000409502)	ENST00000409502
MIR4272	3	67202478	67202643	S18_peak_2317	Intergenic between ENST00000417314 and ENST00000635924	ENST00000635924
RP11-383I23.2	3	99807463	99807628	S18_peak_2318	Intergenic between ENST00000608028 and ENST00000354552	ENST00000608028
Y_RNA	3	106630205	106630370	S18_peak_2319	Intergenic between ENST00000363740 and ENST00000363491	ENST00000363491
AGAP6	10	49986221	49986386	S18_peak_232	Intergenic between ENST00000410176 and ENST00000412531	ENST00000412531
SPICE1	3	113514096	113514272	S18_peak_2320	promoter-TSS (ENST00000295872)	ENST00000295872
RN7SL815P	3	115796837	115797002	S18_peak_2321	Intergenic between ENST00000491321 and ENST00000470970	ENST00000470970
RN7SL815P	3	115926132	115926297	S18_peak_2322	Intergenic between ENST00000470970 and ENST00000490351	ENST00000470970
LSAMP-AS1	3	116416200	116416365	S18_peak_2323	Intergenic between ENST00000490351 and ENST00000497854	ENST00000490351
RP11-384F7.2	3	117778530	117778800	S18_peak_2324	intron (ENST00000484092, intron 1 of 1)	ENST00000484092
RP11-384F7.2	3	117987913	117988078	S18_peak_2325	intron (ENST00000484092, intron 1 of 1)	ENST00000484092
RP11-384F7.1	3	118624807	118624972	S18_peak_2326	Intergenic between ENST00000476460 and ENST00000393775	ENST00000476460
IGSF11	3	118995756	118995921	S18_peak_2327	intron (ENST00000393775, intron 1 of 6)	ENST00000393775
IGSF11	3	118999968	119000332	S18_peak_2328	intron (ENST00000393775, intron 1 of 6)	ENST00000393775
UPK1B	3	119195912	119196077	S18_peak_2329	intron (ENST00000264234, intron 6 of 7)	ENST00000264234
SGMS1	10	50406709	50406932	S18_peak_233	intron (ENST00000361781, intron 6 of 10)	ENST00000361781
ARHGAP31	3	119321713	119321896	S18_peak_2330	intron (ENST00000264245, intron 1 of 11)	ENST00000264245
ADPRH	3	119593017	119593332	S18_peak_2331	Intergenic between ENST00000357003 and ENST00000273371	ENST00000357003
Y_RNA	3	120230623	120230788	S18_peak_2332	Intergenic between ENST00000383994 and ENST00000498792	ENST00000383994
Y_RNA	3	120242511	120242800	S18_peak_2333	Intergenic between ENST00000383994 and ENST00000498792	ENST00000383994
RP11-174O3.3	3	120404337	120404645	S18_peak_2334	intron (ENST00000494869, intron 3 of 4)	ENST00000494869
NDUFB4	3	120604982	120605147	S18_peak_2335	Intergenic between ENST00000485064 and ENST00000283871	ENST00000485064
GTF2E1	3	120756634	120756876	S18_peak_2336	intron (ENST00000283875, intron 2 of 4)	ENST00000283875
RP11-500K19.1	3	120855177	120855342	S18_peak_2337	Intergenic between ENST00000490647 and ENST00000273666	ENST00000490647
STXBP5L	3	121020236	121020401	S18_peak_2338	intron (ENST00000273666, intron 3 of 27)	ENST00000273666
STXBP5L	3	121055527	121055712	S18_peak_2339	intron (ENST00000273666, intron 5 of 27)	ENST00000273666
SGMS1	10	50612961	50613162	S18_peak_234	intron (ENST00000361781, intron 1 of 10)	ENST00000361781
STXBP5L	3	121062077	121062242	S18_peak_2340	intron (ENST00000273666, intron 5 of 27)	ENST00000273666
STXBP5L	3	121071066	121071241	S18_peak_2341	intron (ENST00000273666, intron 5 of 27)	ENST00000273666

STXBP5L	3	121095098	121095326	S18_peak_2342	intron (ENST00000273666, intron 5 of 27)	ENST00000273666
STXBP5L	3	121315489	121315657	S18_peak_2343	intron (ENST00000273666, intron 20 of 27)	ENST00000273666
EAF2	3	121844000	121844210	S18_peak_2344	intron (ENST00000273668, intron 1 of 5)	ENST00000273668
EAF2	3	121856556	121856721	S18_peak_2345	intron (ENST00000273668, intron 3 of 5)	ENST00000273668
HNRNPA1P23	3	122303980	122304217	S18_peak_2346	Intergenic between ENST00000490131 and ENST00000491091	ENST00000491091
DIRC2	3	122872752	122873030	S18_peak_2347	intron (ENST00000261038, intron 8 of 8)	ENST00000261038
PDIA5	3	123073792	123074084	S18_peak_2348	intron (ENST00000316218, intron 1 of 16)	ENST00000316218
PDIA5	3	123119905	123120108	S18_peak_2349	intron (ENST00000316218, intron 8 of 16)	ENST00000316218
A1CF	10	50866366	50866534	S18_peak_235	intron (ENST00000373995, intron 1 of 12)	ENST00000373995
PDIA5	3	123131510	123131675	S18_peak_2350	intron (ENST00000316218, intron 11 of 16)	ENST00000316218
PDIA5	3	123140902	123141164	S18_peak_2351	intron (ENST00000316218, intron 11 of 16)	ENST00000316218
ADCY5	3	123358949	123359145	S18_peak_2352	intron (ENST00000462833, intron 1 of 20)	ENST00000462833
RP11-9N20.3	3	123742073	123742238	S18_peak_2353	Intergenic between ENST00000608701 and ENST00000384275	ENST00000608701
CCDC14	3	123927968	123928133	S18_peak_2354	intron (ENST00000485727, intron 8 of 8)	ENST00000485727
KALRN	3	124038018	124038190	S18_peak_2355	Intergenic between ENST00000433542 and ENST00000240874	ENST00000240874
KALRN	3	124152084	124152249	S18_peak_2356	intron (ENST00000240874, intron 1 of 33)	ENST00000240874
KALRN	3	124490098	124490263	S18_peak_2357	intron (ENST00000240874, intron 29 of 33)	ENST00000240874
RNU6-232P	3	125416893	125417235	S18_peak_2358	Intergenic between ENST00000517144 and ENST00000251775	ENST00000517144
GS1-388B5.8	3	125760619	125760784	S18_peak_2359	Intergenic between ENST00000467186 and ENST00000610060	ENST00000467186
CSTF2T	10	51817150	51817386	S18_peak_236	Intergenic between ENST00000331173 and ENST00000452247	ENST00000331173
SLC41A3	3	126010387	126010578	S18_peak_2360	intron (ENST00000360370, intron 9 of 10)	ENST00000360370
KLF15	3	126332033	126332320	S18_peak_2361	Intergenic between ENST00000514281 and ENST00000296233	ENST00000296233
KLF15	3	126369014	126369243	S18_peak_2362	Intergenic between ENST00000296233 and ENST00000506660	ENST00000296233
TXNRD3NB	3	126578546	126578749	S18_peak_2363	Intergenic between ENST00000404489 and ENST00000524230	ENST00000404489
RP11-305F5.2	3	127219119	127219422	S18_peak_2364	Intergenic between ENST00000391254 and ENST00000492080	ENST00000492080
RNU6-1047P	3	127241163	127241328	S18_peak_2365	Intergenic between ENST00000364418 and ENST00000465482	ENST00000364418
RNU6-1047P	3	127249480	127249645	S18_peak_2366	Intergenic between ENST00000364418 and ENST00000465482	ENST00000364418
RP11-88I21.2	3	127355913	127356078	S18_peak_2367	intron (ENST00000488425, intron 2 of 4)	ENST00000488425
LINC01471	3	127446758	127446923	S18_peak_2368	Intergenic between ENST00000488425 and ENST00000461398	ENST00000461398
RP11-59J16.3	3	127538217	127538382	S18_peak_2369	promoter-TSS (ENST00000635368)	ENST00000635368
MBL2	10	52809498	52809663	S18_peak_237	Intergenic between ENST00000373968 and	ENST00000373968

					ENST00000364326	
MGLL	3	127760292	127760513	S18_peak_2370	Intergenic between ENST00000398101 and ENST00000364340	ENST00000398101
RNA5SP139	3	127915465	127915640	S18_peak_2371	Intergenic between ENST00000398101 and ENST00000364340	ENST00000364340
RNA5SP139	3	128001547	128001891	S18_peak_2372	Intergenic between ENST00000364340 and ENST00000243253	ENST00000364340
EEFSEC	3	128308371	128308536	S18_peak_2373	intron (ENST00000254730, intron 4 of 6)	ENST00000254730
DNAJB8	3	128458358	128458523	S18_peak_2374	Intergenic between ENST00000254730 and ENST00000319153	ENST00000319153
RP11-723O4.6	3	128915635	128915800	S18_peak_2375	intron (ENST00000508239, intron 5 of 7)	ENST00000508239
EFCC1	3	129041948	129042176	S18_peak_2376	Intergenic between ENST00000436022 and ENST00000510422	ENST00000436022
RP11-434H6.2	3	129049681	129049954	S18_peak_2377	Intergenic between ENST00000510422 and ENST00000307395	ENST00000510422
CNBP	3	129191410	129191575	S18_peak_2378	Intergenic between ENST00000441626 and ENST00000464146	ENST00000441626
RP13-685P2.8	3	129336305	129336566	S18_peak_2379	Intergenic between ENST00000604126 and ENST00000603123	ENST00000604126
RP11-71J2.1	10	53249516	53249797	S18_peak_238	Intergenic between ENST00000442866 and ENST00000449272	ENST00000449272
RP11-529F4.1	3	129371591	129371756	S18_peak_2380	Intergenic between ENST00000603123 and ENST00000499853	ENST00000499853
TMCC1	3	129721901	129722144	S18_peak_2381	intron (ENST00000393238, intron 3 of 5)	ENST00000393238
RP11-93K22.6	3	129944920	129945085	S18_peak_2382	Intergenic between ENST00000604747 and ENST00000509201	ENST00000509201
RP11-93K22.6	3	129949826	129950046	S18_peak_2383	Intergenic between ENST00000604747 and ENST00000509201	ENST00000509201
ALG1L2	3	130092100	130092327	S18_peak_2384	exon (ENST00000425059, exon 3 of 8)	ENST00000425059
ENPP7P3	3	130154170	130154335	S18_peak_2385	Intergenic between ENST00000504808 and ENST00000509123	ENST00000509123
COL6A4P2	3	130275731	130275896	S18_peak_2386	Intergenic between ENST00000509855 and ENST00000508850	ENST00000509855
ATP2C1	3	130900102	130900267	S18_peak_2387	intron (ENST00000504381, intron 1 of 26)	ENST00000504381
NEK11	3	131207233	131207567	S18_peak_2388	intron (ENST00000510688, intron 13 of 15)	ENST00000510688
RP11-517B11.4	3	131505385	131505562	S18_peak_2389	Intergenic between ENST00000505219 and ENST00000503006	ENST00000503006
PCDH15	10	54149850	54150092	S18_peak_239	intron (ENST00000395430, intron 14 of 31)	ENST00000395430
NPHP3	3	132695547	132695813	S18_peak_2390	intron (ENST00000337331, intron 15 of 26)	ENST00000337331
NPHP3-AS1	3	132820378	132820578	S18_peak_2391	intron (ENST00000504440, intron 8 of 10)	ENST00000504440
TMEM108	3	133187107	133187454	S18_peak_2392	intron (ENST00000321871, intron 2 of 5)	ENST00000321871
RP11-91K8.4	3	133498401	133498756	S18_peak_2393	Intergenic between ENST00000512176 and ENST00000608823	ENST00000512176
RAB6B	3	133850125	133850290	S18_peak_2394	intron (ENST00000285208, intron 2 of 7)	ENST00000285208

RAB6B	3	133862766	133863201	S18_peak_2395	intron (ENST00000285208, intron 2 of 7)	ENST00000285208
SLCO2A1	3	133931732	133931917	S18_peak_2396	TTS (ENST00000310926)	ENST00000310926
KY	3	134583890	134584155	S18_peak_2397	Intergenic between ENST00000509658 and ENST00000423778	ENST00000423778
EPHB1	3	134961105	134961270	S18_peak_2398	intron (ENST00000398015, intron 3 of 15)	ENST00000398015
EPHB1	3	135046921	135047183	S18_peak_2399	intron (ENST00000398015, intron 3 of 15)	ENST00000398015
CLIC4	1	24758464	24758671	S18_peak_24	intron (ENST00000374379, intron 1 of 5)	ENST00000374379
PCDH15	10	54638037	54638203	S18_peak_240	intron (ENST00000395430, intron 2 of 31)	ENST00000395430
RP11-237P21.1	3	135907084	135907389	S18_peak_2400	Intergenic between ENST00000473001 and ENST00000481235	ENST00000481235
MSL2	3	136156407	136156625	S18_peak_2401	intron (ENST00000309993, intron 1 of 1)	ENST00000309993
IL20RB	3	136962913	136963078	S18_peak_2402	intron (ENST00000329582, intron 1 of 6)	ENST00000329582
IL20RB-AS1	3	137136505	137136711	S18_peak_2403	Intergenic between ENST00000462176 and ENST00000364455	ENST00000462176
HSPA8P9	3	137932066	137932231	S18_peak_2404	Intergenic between ENST00000485881 and ENST00000343735	ENST00000485881
ARMC8	3	138226255	138226466	S18_peak_2405	intron (ENST00000358441, intron 6 of 12)	ENST00000358441
ARMC8	3	138245778	138245943	S18_peak_2406	exon (ENST00000358441, exon 13 of 13)	ENST00000358441
CEP70	3	138576415	138576795	S18_peak_2407	intron (ENST00000264982, intron 2 of 17)	ENST00000264982
PIK3CB	3	138719271	138719436	S18_peak_2408	Intergenic between ENST00000481749 and ENST00000481866	ENST00000481749
BPESC1	3	139111738	139112140	S18_peak_2409	intron (ENST00000418282, intron 2 of 2)	ENST00000418282
RP11-168O22.1	10	55365995	55366160	S18_peak_241	intron (ENST00000457975, intron 3 of 3)	ENST00000457975
MRPS22	3	139334043	139334211	S18_peak_2410	Intergenic between ENST00000616930 and ENST00000478464	ENST00000478464
RP11-319G6.1	3	139511649	139511946	S18_peak_2411	intron (ENST00000515247, intron 3 of 4)	ENST00000515247
NMNAT3	3	139586781	139586946	S18_peak_2412	intron (ENST00000506254, intron 3 of 6)	ENST00000506254
CLSTN2	3	140007303	140007468	S18_peak_2413	intron (ENST00000458420, intron 1 of 16)	ENST00000458420
CLSTN2	3	140136617	140136806	S18_peak_2414	intron (ENST00000458420, intron 1 of 16)	ENST00000458420
CLSTN2	3	140521807	140522200	S18_peak_2415	intron (ENST00000458420, intron 8 of 16)	ENST00000458420
GRK7	3	141821263	141821577	S18_peak_2416	Intergenic between ENST00000264952 and ENST00000486956	ENST00000264952
TFDP2	3	142097615	142097843	S18_peak_2417	intron (ENST00000489671, intron 2 of 12)	ENST00000489671
GK5	3	142201968	142202215	S18_peak_2418	intron (ENST00000392993, intron 4 of 15)	ENST00000392993
XRN1	3	142422145	142422310	S18_peak_2419	intron (ENST00000264951, intron 8 of 41)	ENST00000264951
RP11-168O22.1	10	55429342	55429507	S18_peak_242	intron (ENST00000457975, intron 2 of 3)	ENST00000457975
PLS1	3	142642896	142643319	S18_peak_2420	intron (ENST00000457734, intron 1 of 15)	ENST00000457734
PCOLCE2	3	142812822	142812987	S18_peak_2421	Intergenic between ENST00000515978 and ENST00000295992	ENST00000295992
PCOLCE2	3	142842322	142842487	S18_peak_2422	intron (ENST00000295992, intron 4 of 8)	ENST00000295992
PCOLCE2	3	142884671	142884912	S18_peak_2423	intron (ENST00000295992, intron 2 of 8)	ENST00000295992
SLC9A9	3	143434747	143434912	S18_peak_2424	intron (ENST00000316549, intron 12 of 15)	ENST00000316549
SLC9A9	3	143620907	143621121	S18_peak_2425	intron (ENST00000316549, intron 6 of 15)	ENST00000316549
SLC9A9	3	143691043	143691284	S18_peak_2426	intron (ENST00000316549, intron 5 of 15)	ENST00000316549

RNA5SP144	3	144213320	144213485	S18_peak_2427	Intergenic between ENST00000410846 and ENST00000624647	ENST00000410846
RP11-42F12.1	3	144364532	144364697	S18_peak_2428	Intergenic between ENST00000410846 and ENST00000624647	ENST00000624647
RP11-42F12.1	3	144485981	144486149	S18_peak_2429	Intergenic between ENST00000624647 and ENST00000481075	ENST00000624647
RP11-168O22.1	10	55556622	55556787	S18_peak_243	intron (ENST00000457975, intron 2 of 3)	ENST00000457975
RP11-42F12.1	3	144862654	144862854	S18_peak_2430	Intergenic between ENST00000624647 and ENST00000481075	ENST00000624647
RP11-42F12.1	3	144936840	144937005	S18_peak_2431	Intergenic between ENST00000624647 and ENST00000481075	ENST00000624647
LARP7P4	3	145000037	145000275	S18_peak_2432	Intergenic between ENST00000624647 and ENST00000481075	ENST00000481075
LARP7P4	3	145601642	145601807	S18_peak_2433	Intergenic between ENST00000481075 and ENST00000491450	ENST00000481075
GM2AP1	3	145713940	145714105	S18_peak_2434	Intergenic between ENST00000491450 and ENST00000463605	ENST00000491450
RP11-274H2.5	3	146045806	146045971	S18_peak_2435	Intergenic between ENST00000494509 and ENST00000567714	ENST00000567714
RP11-758I14.2	3	146334105	146334317	S18_peak_2436	Intergenic between ENST00000493382 and ENST00000464158	ENST00000464158
PLSCR2	3	146426411	146426576	S18_peak_2437	Intergenic between ENST00000482888 and ENST00000497985	ENST00000497985
PLSCR5-AS1	3	146732825	146733023	S18_peak_2438	Intergenic between ENST00000473817 and ENST00000473299	ENST00000473817
RP11-649A16.1	3	147152066	147152231	S18_peak_2439	intron (ENST00000473299, intron 3 of 5)	ENST00000473299
RP11-478B11.2	10	55965237	55965454	S18_peak_244	Intergenic between ENST00000605671 and ENST00000373944	ENST00000605671
RP11-649A16.1	3	147357224	147357522	S18_peak_2440	intron (ENST00000473299, intron 2 of 5)	ENST00000473299
NPM1P28	3	147712464	147712649	S18_peak_2441	Intergenic between ENST00000492269 and ENST00000470739	ENST00000492269
RP11-78O22.1	3	147747890	147748055	S18_peak_2442	Intergenic between ENST00000492269 and ENST00000470739	ENST00000470739
RP11-501O2.3	3	148186481	148186649	S18_peak_2443	intron (ENST00000477153, intron 1 of 2)	ENST00000477153
HMGB1P30	3	148803847	148804114	S18_peak_2444	Intergenic between ENST00000349243 and ENST00000492455	ENST00000492455
COMMD2	3	149704128	149704339	S18_peak_2445	Intergenic between ENST00000479752 and ENST00000473414	ENST00000473414
COMMD2	3	149746015	149746180	S18_peak_2446	intron (ENST00000473414, intron 4 of 4)	ENST00000473414
LINC01214	3	150277196	150277361	S18_peak_2447	intron (ENST00000471222, intron 1 of 2)	ENST00000471222
EIF2A	3	150570971	150571203	S18_peak_2448	intron (ENST00000460851, intron 9 of 13)	ENST00000460851
CLRN1-AS1	3	150978897	150979112	S18_peak_2449	intron (ENST00000476886, intron 1 of 4)	ENST00000476886
ZWINT	10	56170694	56170859	S18_peak_245	Intergenic between ENST00000605671 and ENST00000373944	ENST00000373944

CLRN1-AS1	3	151080005	151080170	S18_peak_2450	exon (ENST00000476886, exon 5 of 5)	ENST00000476886
MED12L	3	151413772	151413937	S18_peak_2451	intron (ENST00000474524, intron 40 of 42)	ENST00000474524
MBNL1-AS1	3	152225220	152225387	S18_peak_2452	Intergenic between ENST00000482382 and ENST00000608395	ENST00000608395
ATP5LP5	3	152769989	152770189	S18_peak_2453	Intergenic between ENST00000496084 and ENST00000483050	ENST00000483050
RP11-292E2.1	3	153340450	153340678	S18_peak_2454	Intergenic between ENST00000582522 and ENST00000498457	ENST00000498457
RP11-292E2.1	3	153346669	153346834	S18_peak_2455	Intergenic between ENST00000582522 and ENST00000498457	ENST00000498457
RP11-23D24.2	3	153534487	153534718	S18_peak_2456	exon (ENST00000493214, exon 5 of 7)	ENST00000493214
RP11-23D24.2	3	153561920	153562085	S18_peak_2457	intron (ENST00000493214, intron 4 of 6)	ENST00000493214
RP11-23D24.2	3	153668344	153668509	S18_peak_2458	intron (ENST00000493214, intron 3 of 6)	ENST00000493214
RP11-23D24.2	3	153884807	153884972	S18_peak_2459	intron (ENST00000488210, intron 3 of 3)	ENST00000488210
ZWINT	10	56446098	56446263	S18_peak_246	Intergenic between ENST00000373944 and ENST00000458762	ENST00000373944
ARHGEF26-AS1	3	154102564	154102797	S18_peak_2460	intron (ENST00000491862, intron 2 of 3)	ENST00000491862
GPR149	3	154371292	154371457	S18_peak_2461	intron (ENST00000389740, intron 3 of 3)	ENST00000389740
RP11-656A15.1	3	154468210	154468375	S18_peak_2462	Intergenic between ENST00000463545 and ENST00000498604	ENST00000498604
RPL9P15	3	154708227	154708392	S18_peak_2463	Intergenic between ENST00000491096 and ENST00000478814	ENST00000491096
RP11-439C8.1	3	154865097	154865319	S18_peak_2464	Intergenic between ENST00000478814 and ENST00000486545	ENST00000478814
RP11-439C8.2	3	154982728	154982901	S18_peak_2465	Intergenic between ENST00000486545 and ENST00000484721	ENST00000486545
PLCH1	3	155493488	155493653	S18_peak_2466	intron (ENST00000340059, intron 16 of 22)	ENST00000340059
KCNAB1	3	156531655	156531820	S18_peak_2467	intron (ENST00000302490, intron 13 of 13)	ENST00000302490
LINC00880	3	157096329	157096709	S18_peak_2468	intron (ENST00000471357, intron 3 of 3)	ENST00000471357
CCNL1	3	157135839	157136004	S18_peak_2469	Intergenic between ENST00000467995 and ENST00000295926	ENST00000295926
RP11-179B15.6	10	58326342	58326507	S18_peak_247	promoter-TSS (ENST00000562575)	ENST00000562575
PQLC2L	3	157641617	157641913	S18_peak_2470	Intergenic between ENST00000426338 and ENST00000515942	ENST00000426338
RN7SKP46	3	157825130	157825295	S18_peak_2471	Intergenic between ENST00000426338 and ENST00000515942	ENST00000515942
RSRC1	3	158225226	158225563	S18_peak_2472	intron (ENST00000312179, intron 3 of 8)	ENST00000312179
RSRC1	3	158244230	158244472	S18_peak_2473	intron (ENST00000312179, intron 3 of 8)	ENST00000312179
RSRC1	3	158388644	158388809	S18_peak_2474	intron (ENST00000312179, intron 5 of 8)	ENST00000312179
RSRC1	3	158416454	158416660	S18_peak_2475	intron (ENST00000312179, intron 5 of 8)	ENST00000312179
RSRC1	3	158537070	158537235	S18_peak_2476	exon (ENST00000312179, exon 7 of 9)	ENST00000312179
MFSD1	3	158820713	158820878	S18_peak_2477	intron (ENST00000415822, intron 9 of 15)	ENST00000415822
RP11-170K4.2	3	158914123	158914461	S18_peak_2478	Intergenic between ENST00000624004 and ENST00000482126	ENST00000624004

IQCJ-SCHIP1	3	159355545	159355710	S18_peak_2479	intron (ENST00000476809, intron 3 of 9)	ENST00000476809
UBE2D1	10	58334262	58334427	S18_peak_248	Intergenic between ENST00000562575 and ENST00000373910	ENST00000373910
RP11-774I5.1	3	161830828	161831003	S18_peak_2480	Intergenic between ENST00000497285 and ENST00000465035	ENST00000497285
MIR1263	3	164131034	164131199	S18_peak_2481	Intergenic between ENST00000483240 and ENST00000408324	ENST00000408324
LINC01323	3	164694762	164694955	S18_peak_2482	Intergenic between ENST00000486767 and ENST00000463978	ENST00000486767
LINC01322	3	165445009	165445174	S18_peak_2483	intron (ENST00000470138, intron 2 of 3)	ENST00000470138
MECOM	3	169225788	169225953	S18_peak_2484	Intergenic between ENST00000468789 and ENST00000480669	ENST00000468789
RP11-3K16.2	3	169636596	169636810	S18_peak_2485	Intergenic between ENST00000469794 and ENST00000484597	ENST00000469794
RP11-3K16.1	3	169675233	169675398	S18_peak_2486	Intergenic between ENST00000469794 and ENST00000484597	ENST00000484597
TERC	3	169751119	169751309	S18_peak_2487	Intergenic between ENST00000384050 and ENST00000602385	ENST00000602385
LRRC31	3	169850959	169851141	S18_peak_2488	intron (ENST00000264676, intron 6 of 7)	ENST00000264676
KLF7P1	3	170938880	170939053	S18_peak_2489	Intergenic between ENST00000295822 and ENST00000490941	ENST00000490941
BICC1	10	58773177	58773342	S18_peak_249	intron (ENST00000373886, intron 3 of 20)	ENST00000373886
SLC2A2	3	171038889	171039054	S18_peak_2490	Intergenic between ENST00000497642 and ENST00000436636	ENST00000497642
TNIK	3	171107660	171107825	S18_peak_2491	intron (ENST00000436636, intron 20 of 32)	ENST00000436636
TNIK	3	171332495	171332696	S18_peak_2492	intron (ENST00000436636, intron 2 of 32)	ENST00000436636
TNIK	3	171343265	171343430	S18_peak_2493	intron (ENST00000436636, intron 2 of 32)	ENST00000436636
RP11-185E8.1	3	171475385	171475550	S18_peak_2494	Intergenic between ENST00000467896 and ENST00000384386	ENST00000467896
PLD1	3	171785894	171786059	S18_peak_2495	intron (ENST00000356327, intron 1 of 25)	ENST00000356327
TMEM212-IT1	3	171916204	171916413	S18_peak_2496	Intergenic between ENST00000456146 and ENST00000415807	ENST00000456146
ECT2	3	172796513	172796836	S18_peak_2497	intron (ENST00000392692, intron 18 of 24)	ENST00000392692
SPATA16	3	172871736	172872039	S18_peak_2498	Intergenic between ENST00000428618 and ENST00000351008	ENST00000351008
RP11-324C10.1	3	173382262	173382455	S18_peak_2499	Intergenic between ENST00000447709 and ENST00000449016	ENST00000447709
EPB41	1	29010948	29011113	S18_peak_25	intron (ENST00000349460, intron 5 of 20)	ENST00000349460
BICC1	10	58816235	58816400	S18_peak_250	intron (ENST00000373886, intron 18 of 20)	ENST00000373886
RP11-521A24.1	3	173505201	173505366	S18_peak_2500	Intergenic between ENST00000447709 and ENST00000449016	ENST00000449016
NLGN1-AS1	3	173977274	173977439	S18_peak_2501	Intergenic between ENST00000457192 and ENST00000517173	ENST00000457192
RN7SKP234	3	174120121	174120286	S18_peak_2502	Intergenic between ENST00000517173 and	ENST00000517173

					ENST00000411665	
NAALADL2	3	175432681	175432846	S18_peak_2503	intron (ENST00000454872, intron 5 of 13)	ENST00000454872
LINC00578	3	177466544	177466795	S18_peak_2504	intron (ENST00000442937, intron 1 of 3)	ENST00000442937
RNU6-1120P	3	177983720	177983885	S18_peak_2505	Intergenic between ENST00000603299 and ENST00000362988	ENST00000362988
FLJ46066	3	182452347	182452545	S18_peak_2506	intron (ENST00000496220, intron 2 of 3)	ENST00000496220
LAMP3	3	183118081	183118284	S18_peak_2507	Intergenic between ENST00000471731 and ENST00000265598	ENST00000265598
YEATS2	3	183753431	183753604	S18_peak_2508	intron (ENST00000305135, intron 10 of 30)	ENST00000305135
RP11-433C9.2	3	184458795	184459027	S18_peak_2509	Intergenic between ENST00000419960 and ENST00000637035	ENST00000419960
RP11-135D11.2	10	59576837	59577057	S18_peak_251	Intergenic between ENST00000446432 and ENST00000450677	ENST00000450677
C3orf70	3	184930701	184930866	S18_peak_2510	Intergenic between ENST00000457449 and ENST00000335012	ENST00000335012
LIPH	3	185555942	185556186	S18_peak_2511	Intergenic between ENST00000424591 and ENST00000296257	ENST00000424591
IGF2BP2	3	185813886	185814151	S18_peak_2512	intron (ENST00000382199, intron 2 of 15)	ENST00000382199
ADIPOQ-AS1	3	186859541	186859740	S18_peak_2513	Intergenic between ENST00000422718 and ENST00000412545	ENST00000422718
SST	3	187591303	187591468	S18_peak_2514	Intergenic between ENST00000440726 and ENST00000287641	ENST00000287641
RP11-430L16.1	3	188128676	188128841	S18_peak_2515	intron (ENST00000449845, intron 1 of 1)	ENST00000449845
FLJ42393	3	188221508	188221681	S18_peak_2516	Intergenic between ENST00000623801 and ENST00000434996	ENST00000623801
MIR28	3	188741271	188741436	S18_peak_2517	Intergenic between ENST00000384918 and ENST00000444488	ENST00000384918
TPRG1	3	189088481	189088667	S18_peak_2518	Intergenic between ENST00000411493 and ENST00000345063	ENST00000345063
TPRG1	3	189105688	189105853	S18_peak_2519	Intergenic between ENST00000411493 and ENST00000345063	ENST00000345063
ANK3	10	60146541	60146708	S18_peak_252	intron (ENST00000280772, intron 23 of 43)	ENST00000280772
TP63	3	189808582	189808747	S18_peak_2520	intron (ENST00000264731, intron 4 of 13)	ENST00000264731
TP63	3	189823163	189823328	S18_peak_2521	intron (ENST00000264731, intron 4 of 13)	ENST00000264731
NMNAT1P3	3	190226516	190226774	S18_peak_2522	Intergenic between ENST00000457517 and ENST00000295522	ENST00000457517
CLDN1	3	190312923	190313088	S18_peak_2523	exon (ENST00000295522, exon 2 of 4)	ENST00000295522
OSTN	3	191126378	191126588	S18_peak_2524	Intergenic between ENST00000442080 and ENST00000339051	ENST00000339051
RP11-655G22.2	3	191877893	191878058	S18_peak_2525	Intergenic between ENST00000364990 and ENST00000434379	ENST00000434379
FGF12	3	192239399	192239564	S18_peak_2526	intron (ENST00000454309, intron 3 of 4)	ENST00000454309
RP11-384A12.1	3	194478406	194478571	S18_peak_2527	Intergenic between ENST00000605225 and ENST00000448892	ENST00000605225

XXYLT1	3	195163296	195163461	S18_peak_2528	intron (ENST00000310380, intron 2 of 3)	ENST00000310380
ACAP2	3	195416240	195416405	S18_peak_2529	intron (ENST00000326793, intron 1 of 22)	ENST00000326793
ANK3	10	60200793	60201058	S18_peak_253	intron (ENST00000280772, intron 12 of 43)	ENST00000280772
RNU6-910P	3	196341823	196341988	S18_peak_2530	Intergenic between ENST00000390844 and ENST00000296328	ENST00000390844
RNF168	3	196491443	196491608	S18_peak_2531	intron (ENST00000318037, intron 1 of 5)	ENST00000318037
AC128709.3	3	197377840	197378071	S18_peak_2532	Intergenic between ENST00000414529 and ENST00000438408	ENST00000438408
BDH1	3	197539344	197539675	S18_peak_2533	intron (ENST00000392378, intron 2 of 6)	ENST00000392378
LMLN	3	197971354	197971519	S18_peak_2534	intron (ENST00000330198, intron 1 of 15)	ENST00000330198
LMLN-AS1	3	198045774	198045959	S18_peak_2535	Intergenic between ENST00000423460 and ENST00000455355	ENST00000423460
ZNF595	4	65560	65765	S18_peak_2536	intron (ENST00000610261, intron 3 of 3)	ENST00000610261
RP11-478C6.6	4	267862	268027	S18_peak_2537	Intergenic between ENST00000398732 and ENST00000511111	ENST00000511111
MIR573	4	24291031	24291268	S18_peak_2538	Intergenic between ENST00000264867 and ENST00000384964	ENST00000384964
MIR573	4	24373133	24373363	S18_peak_2539	Intergenic between ENST00000264867 and ENST00000384964	ENST00000384964
ANK3	10	60296043	60296208	S18_peak_254	intron (ENST00000280772, intron 1 of 43)	ENST00000280772
ZCCHC4	4	25355370	25355535	S18_peak_2540	intron (ENST00000302874, intron 8 of 12)	ENST00000302874
RP11-302F12.2	4	25624334	25624499	S18_peak_2541	TTS (ENST00000506450)	ENST00000506450
SEL1L3	4	25755721	25756039	S18_peak_2542	intron (ENST00000399878, intron 23 of 23)	ENST00000399878
RP11-293A21.1	4	26798704	26798919	S18_peak_2543	Intergenic between ENST00000364129 and ENST00000467484	ENST00000467484
RP11-415C15.3	4	27419414	27419579	S18_peak_2544	Intergenic between ENST00000513616 and ENST00000507524	ENST00000513616
IGBP1P5	4	27433210	27433375	S18_peak_2545	Intergenic between ENST00000513616 and ENST00000507524	ENST00000507524
RP11-180C1.1	4	28265204	28265380	S18_peak_2546	Intergenic between ENST00000509666 and ENST00000507759	ENST00000507759
RP11-472K22.1	4	29187715	29187880	S18_peak_2547	intron (ENST00000503299, intron 2 of 2)	ENST00000503299
RP11-68D16.1	4	29346750	29346915	S18_peak_2548	Intergenic between ENST00000512268 and ENST00000513866	ENST00000513866
RP11-617I14.1	4	31135387	31135552	S18_peak_2549	Intergenic between ENST00000513621 and ENST00000515292	ENST00000515292
RP11-491H19.1	10	61849172	61849337	S18_peak_255	Intergenic between ENST00000441001 and ENST00000279873	ENST00000441001
RP11-315A17.1	4	31395391	31395688	S18_peak_2550	Intergenic between ENST00000505636 and ENST00000510420	ENST00000505636
RP11-734I18.1	4	32176545	32176710	S18_peak_2551	Intergenic between ENST00000513211 and ENST00000515181	ENST00000513211
RP11-640B6.1	4	35852440	35852605	S18_peak_2552	Intergenic between ENST00000383938 and ENST00000514630	ENST00000514630

Y_RNA	4	37723704	37723869	S18_peak_2553	Intergenic between ENST00000384347 and ENST00000493466	ENST00000384347
RP11-600L4.1	4	38313410	38313575	S18_peak_2554	Intergenic between ENST00000506010 and ENST00000503465	ENST00000506010
RP11-213G21.2	4	38582623	38582797	S18_peak_2555	Intergenic between ENST00000512170 and ENST00000515436	ENST00000512170
FAM114A1	4	38893988	38894153	S18_peak_2556	intron (ENST00000358869, intron 4 of 14)	ENST00000358869
UGDH-AS1	4	39586775	39586940	S18_peak_2557	intron (ENST00000504032, intron 2 of 4)	ENST00000504032
PDS5A	4	39861599	39861764	S18_peak_2558	intron (ENST00000303538, intron 26 of 32)	ENST00000303538
PDS5A	4	39935136	39935444	S18_peak_2559	intron (ENST00000303538, intron 2 of 32)	ENST00000303538
ARID5B	10	62011499	62011664	S18_peak_256	intron (ENST00000279873, intron 4 of 9)	ENST00000279873
NSUN7	4	40702628	40702793	S18_peak_2560	Intergenic between ENST00000581881 and ENST00000381782	ENST00000381782
APBB2	4	40895610	40895827	S18_peak_2561	intron (ENST00000513140, intron 9 of 16)	ENST00000513140
APBB2	4	40938701	40938866	S18_peak_2562	intron (ENST00000513140, intron 7 of 16)	ENST00000513140
Y_RNA	4	41324371	41324607	S18_peak_2563	Intergenic between ENST00000384750 and ENST00000313860	ENST00000384750
SLC30A9	4	42072871	42073036	S18_peak_2564	intron (ENST00000264451, intron 15 of 17)	ENST00000264451
RP11-109E24.1	4	42694312	42694492	S18_peak_2565	Intergenic between ENST00000562054 and ENST00000511279	ENST00000511279
RP11-109E24.1	4	42733142	42733307	S18_peak_2566	Intergenic between ENST00000511279 and ENST00000516786	ENST00000511279
RP11-109E24.1	4	42780810	42780984	S18_peak_2567	Intergenic between ENST00000511279 and ENST00000516786	ENST00000511279
GRXCR1	4	43025662	43025889	S18_peak_2568	intron (ENST00000399770, intron 3 of 3)	ENST00000399770
RN7SKP199	4	45955490	45955713	S18_peak_2569	Intergenic between ENST00000363850 and ENST00000410325	ENST00000410325
ARID5B	10	62141850	62142072	S18_peak_257	Intergenic between ENST00000309334 and ENST00000373789	ENST00000309334
GABRA4	4	46855820	46856053	S18_peak_2570	Intergenic between ENST00000511164 and ENST00000264318	ENST00000264318
GABRB1	4	47385738	47386051	S18_peak_2571	intron (ENST00000295454, intron 5 of 8)	ENST00000295454
SLAIN2	4	48314211	48314386	S18_peak_2572	Intergenic between ENST00000365144 and ENST00000264313	ENST00000264313
RP11-1281K21.2	4	49157830	49157996	S18_peak_2573	Intergenic between ENST00000513630 and ENST00000512142	ENST00000512142
RP11-1281K21.2	4	49166219	49166384	S18_peak_2574	Intergenic between ENST00000513630 and ENST00000512142	ENST00000512142
MTND3P22	4	49247828	49248025	S18_peak_2575	Intergenic between ENST00000510932 and ENST00000399720	ENST00000510932
SNX18P25	4	50000076	50000241	S18_peak_2577	Intergenic between ENST00000510539 and ENST00000381441	ENST00000510539
RP11-752D24.2	4	52957502	52957750	S18_peak_2578	exon (ENST00000508813, exon 3 of 3)	ENST00000508813
RP11-231C18.3	4	53863002	53863191	S18_peak_2579	intron (ENST00000507166, intron 12 of 23)	ENST00000507166

REEP3	10	63592668	63592883	S18_peak_258	intron (ENST00000373758, intron 2 of 7)	ENST00000373758
RP11-273B19.2	4	54788947	54789112	S18_peak_2580	Intergenic between ENST00000288135 and ENST00000512898	ENST00000512898
RP11-530I17.2	4	55228025	55228190	S18_peak_2581	Intergenic between ENST00000507445 and ENST00000384112	ENST00000507445
CLOCK	4	55528435	55528600	S18_peak_2582	intron (ENST00000513440, intron 1 of 22)	ENST00000513440
EXOC1	4	55894709	55894927	S18_peak_2583	intron (ENST00000381295, intron 15 of 18)	ENST00000381295
KIAA1211	4	56181295	56181564	S18_peak_2584	intron (ENST00000264229, intron 2 of 10)	ENST00000264229
KIAA1211	4	56209866	56210031	S18_peak_2585	intron (ENST00000264229, intron 2 of 10)	ENST00000264229
KIAA1211	4	56212436	56212630	S18_peak_2586	intron (ENST00000264229, intron 2 of 10)	ENST00000264229
KIAA1211	4	56251194	56251359	S18_peak_2587	intron (ENST00000264229, intron 2 of 10)	ENST00000264229
AASDH	4	56359284	56359449	S18_peak_2588	intron (ENST00000205214, intron 5 of 14)	ENST00000205214
PAICS	4	56457251	56457416	S18_peak_2589	intron (ENST00000399688, intron 9 of 9)	ENST00000399688
RP11-170M17.1	10	63699083	63699354	S18_peak_259	intron (ENST00000444770, intron 2 of 2)	ENST00000444770
THEGL	4	56535773	56535948	S18_peak_2590	intron (ENST00000512175, intron 1 of 8)	ENST00000512175
THEGL	4	56601236	56601448	S18_peak_2591	intron (ENST00000512175, intron 8 of 8)	ENST00000512175
HOPX	4	56623477	56623642	S18_peak_2592	Intergenic between ENST00000510885 and ENST00000337881	ENST00000337881
RP11-533F5.2	4	56770081	56770246	S18_peak_2593	Intergenic between ENST00000514020 and ENST00000460700	ENST00000514020
RN7SL492P	4	56789126	56789291	S18_peak_2594	Intergenic between ENST00000514020 and ENST00000460700	ENST00000460700
Y_RNA	4	56824968	56825133	S18_peak_2595	Intergenic between ENST00000515990 and ENST00000309042	ENST00000515990
Y_RNA	4	56845216	56845525	S18_peak_2596	Intergenic between ENST00000515990 and ENST00000309042	ENST00000515990
Y_RNA	4	56846893	56847058	S18_peak_2597	Intergenic between ENST00000515990 and ENST00000309042	ENST00000515990
RP11-738E22.3	4	56942148	56942313	S18_peak_2598	Intergenic between ENST00000503522 and ENST00000602820	ENST00000602820
IGFBP7	4	57096340	57096545	S18_peak_2599	intron (ENST00000295666, intron 1 of 4)	ENST00000295666
RP5-1125N11.1	1	30810227	30810392	S18_peak_26	TTS (ENST00000440358)	ENST00000440358
RP11-170M17.1	10	63804496	63804661	S18_peak_260	intron (ENST00000444770, intron 2 of 2)	ENST00000444770
IGFBP7-AS1	4	57221654	57222053	S18_peak_2600	Intergenic between ENST00000515865 and ENST00000497656	ENST00000515865
RP11-685F15.1	4	57587637	57587802	S18_peak_2601	Intergenic between ENST00000511962 and ENST00000509641	ENST00000509641
RP11-4O3.2	4	57696952	57697117	S18_peak_2602	Intergenic between ENST00000507808 and ENST00000508135	ENST00000508135
RP11-257A22.1	4	63003078	63003257	S18_peak_2603	Intergenic between ENST00000510543 and ENST00000507857	ENST00000507857
NPFFR2	4	72003963	72004185	S18_peak_2604	Intergenic between ENST00000507156 and ENST00000308744	ENST00000308744
CDS1	4	84592745	84592910	S18_peak_2605	intron (ENST00000295887, intron 1 of 12)	ENST00000295887

RP11-84C13.2	4	89093438	89093691	S18_peak_2606	Intergenic between ENST00000513837 and ENST00000603220	ENST00000603220
GPRIN3	4	89256503	89256668	S18_peak_2607	intron (ENST00000609438, intron 1 of 1)	ENST00000609438
CCSER1	4	90497692	90497857	S18_peak_2608	intron (ENST00000432775, intron 5 of 7)	ENST00000432775
RP11-710C12.1	4	95405390	95405555	S18_peak_2609	Intergenic between ENST00000264568 and ENST00000605849	ENST00000605849
RP11-170M17.1	10	63887359	63887588	S18_peak_261	intron (ENST00000444770, intron 2 of 2)	ENST00000444770
COX7A2P2	4	96953009	96953372	S18_peak_2610	Intergenic between ENST00000505291 and ENST00000517407	ENST00000505291
RP11-681L8.1	4	97600660	97600845	S18_peak_2611	intron (ENST00000521680, intron 1 of 2)	ENST00000521680
RP11-571L19.8	4	99066950	99067115	S18_peak_2612	TTS (ENST00000609071)	ENST00000609071
RP11-696N14.3	4	99492114	99492279	S18_peak_2613	intron (ENST00000506494, intron 1 of 3)	ENST00000506494
EMCN	4	100569249	100569491	S18_peak_2614	Intergenic between ENST00000511970 and ENST00000515728	ENST00000511970
RN7SL89P	4	105302952	105303117	S18_peak_2615	Intergenic between ENST00000480252 and ENST00000341695	ENST00000480252
LRIT3	4	109859609	109859776	S18_peak_2616	intron (ENST00000594814, intron 2 of 3)	ENST00000594814
AC004062.2	4	111108948	111109132	S18_peak_2617	Intergenic between ENST00000509926 and ENST00000364093	ENST00000509926
ANK2	4	113228315	113228495	S18_peak_2618	intron (ENST00000394537, intron 4 of 44)	ENST00000394537
ANXA5	4	121595938	121596154	S18_peak_2619	Intergenic between ENST00000512282 and ENST00000296511	ENST00000296511
RP11-210G22.1	10	66157010	66157179	S18_peak_262	Intergenic between ENST00000608793 and ENST00000620045	ENST00000608793
BBS12	4	122776325	122776490	S18_peak_2620	Intergenic between ENST00000314218 and ENST00000480069	ENST00000314218
RP11-18O11.1	4	127488352	127488690	S18_peak_2621	Intergenic between ENST00000509671 and ENST00000514125	ENST00000514125
RP11-400D2.2	4	134236491	134236656	S18_peak_2622	Intergenic between ENST00000421491 and ENST00000504728	ENST00000504728
RP11-223C24.1	4	142638603	142638772	S18_peak_2623	intron (ENST00000509497, intron 2 of 2)	ENST00000509497
FREM3	4	143655051	143655297	S18_peak_2624	intron (ENST00000329798, intron 2 of 7)	ENST00000329798
POU4F2	4	146618041	146618206	S18_peak_2625	Intergenic between ENST00000384453 and ENST00000281321	ENST00000281321
POU4F2	4	146723677	146724118	S18_peak_2626	Intergenic between ENST00000281321 and ENST00000515530	ENST00000281321
LRBA	4	150660504	150660690	S18_peak_2627	intron (ENST00000510413, intron 37 of 56)	ENST00000510413
FBXW7	4	152483297	152483462	S18_peak_2628	intron (ENST00000281708, intron 1 of 11)	ENST00000281708
RP11-502M1.2	4	160669116	160669281	S18_peak_2629	Intergenic between ENST00000512652 and ENST00000473701	ENST00000512652
RP11-210G22.1	10	66329465	66329630	S18_peak_263	Intergenic between ENST00000608793 and ENST00000620045	ENST00000608793
FSTL5	4	161724798	161724963	S18_peak_2630	intron (ENST00000306100, intron 6 of 15)	ENST00000306100
RP11-475B2.1	4	173583924	173584089	S18_peak_2631	Intergenic between ENST00000359562 and	ENST00000561655

					ENST00000561655	
AGA	4	177396888	177397053	S18_peak_2632	Intergenic between ENST00000506895 and ENST00000264595	ENST00000264595
PRIMPOL	4	184672467	184672632	S18_peak_2633	intron (ENST00000314970, intron 7 of 13)	ENST00000314970
SNORA31	4	186117686	186117945	S18_peak_2634	Intergenic between ENST00000296795 and ENST00000517204	ENST00000517204
DUX4L2	4	190099739	190099904	S18_peak_2635	Intergenic between ENST00000561772 and ENST00000571193	ENST00000561772
Y_RNA	5	126845	127112	S18_peak_2637	Intergenic between ENST00000506872 and ENST00000362670	ENST00000362670
AHRR	5	360822	361301	S18_peak_2638	intron (ENST00000316418, intron 3 of 11)	ENST00000316418
AHRR	5	362370	362599	S18_peak_2639	intron (ENST00000316418, intron 3 of 11)	ENST00000316418
RP11-210G22.1	10	66448247	66448412	S18_peak_264	Intergenic between ENST00000608793 and ENST00000620045	ENST00000608793
MIR4456	5	557952	558144	S18_peak_2640	Intergenic between ENST00000582112 and ENST00000506629	ENST00000582112
ZDHHC11	5	825759	825924	S18_peak_2641	intron (ENST00000283441, intron 7 of 12)	ENST00000283441
ZDHHC11	5	842969	843215	S18_peak_2642	intron (ENST00000283441, intron 4 of 12)	ENST00000283441
BRD9	5	864355	864561	S18_peak_2643	exon (ENST00000483173, exon 16 of 16)	ENST00000483173
BRD9	5	878202	878480	S18_peak_2644	intron (ENST00000483173, intron 11 of 15)	ENST00000483173
RP11-661C8.3	5	968691	968955	S18_peak_2645	Intergenic between ENST00000507989 and ENST00000511936	ENST00000511936
TERT	5	1260164	1260344	S18_peak_2646	intron (ENST00000310581, intron 12 of 15)	ENST00000310581
SLC6A3	5	1424434	1424685	S18_peak_2647	intron (ENST00000270349, intron 4 of 14)	ENST00000270349
LPCAT1	5	1491261	1491435	S18_peak_2648	intron (ENST00000283415, intron 3 of 13)	ENST00000283415
MRPL36	5	1769908	1770073	S18_peak_2649	Intergenic between ENST00000565428 and ENST00000505059	ENST00000505059
RP11-93L14.1	10	67258038	67258203	S18_peak_265	Intergenic between ENST00000422963 and ENST00000422016	ENST00000422016
RP11-259O2.3	5	1974770	1974953	S18_peak_2650	Intergenic between ENST00000514519 and ENST00000364156	ENST00000514519
RP11-259O2.3	5	2005245	2005491	S18_peak_2651	Intergenic between ENST00000514519 and ENST00000364156	ENST00000514519
RP11-259O2.3	5	2060307	2060493	S18_peak_2652	Intergenic between ENST00000514519 and ENST00000364156	ENST00000514519
Y_RNA	5	2092727	2092943	S18_peak_2653	Intergenic between ENST00000514519 and ENST00000364156	ENST00000364156
RP11-24P24.1	5	2262631	2263027	S18_peak_2654	TTS (ENST00000610958)	ENST00000610958
RP11-24P24.1	5	2343134	2343299	S18_peak_2655	Intergenic between ENST00000610958 and ENST00000626215	ENST00000610958
LSINCT5	5	2585435	2585725	S18_peak_2656	Intergenic between ENST00000610958 and ENST00000626215	ENST00000626215
IRX2	5	2749647	2749920	S18_peak_2657	promoter-TSS (ENST00000382611)	ENST00000382611
RP11-468D11.1	5	2838268	2838433	S18_peak_2658	Intergenic between ENST00000509745 and	ENST00000509745

					ENST00000515086	
RP11-35O7.1	5	3001720	3001912	S18_peak_2659	Intergenic between ENST00000515086 and ENST00000505396	ENST00000515086
MIR7151	10	67492970	67493135	S18_peak_266	Intergenic between ENST00000617477 and ENST00000425765	ENST00000617477
RP11-35O7.1	5	3009860	3010025	S18_peak_2660	Intergenic between ENST00000515086 and ENST00000505396	ENST00000515086
LINC01019	5	3535491	3535873	S18_peak_2661	promoter-TSS (ENST00000505443)	ENST00000505443
CTD-2012M11.3	5	3694816	3694981	S18_peak_2662	Intergenic between ENST00000559410 and ENST00000513859	ENST00000559410
CTD-2012M11.3	5	3706815	3706980	S18_peak_2663	Intergenic between ENST00000559410 and ENST00000513859	ENST00000559410
CTD-2287N17.1	5	3990873	3991038	S18_peak_2664	Intergenic between ENST00000559410 and ENST00000513859	ENST00000513859
RP11-445O3.3	5	4432900	4433156	S18_peak_2665	Intergenic between ENST00000503415 and ENST00000562127	ENST00000562127
RP11-445O3.2	5	4682263	4682462	S18_peak_2666	intron (ENST00000507435, intron 3 of 5)	ENST00000507435
RP11-445O3.2	5	4740994	4741207	S18_peak_2667	intron (ENST00000507435, intron 3 of 5)	ENST00000507435
CTD-2247C11.5	5	5087017	5087205	S18_peak_2668	Intergenic between ENST00000507950 and ENST00000411075	ENST00000507950
CTD-2247C11.5	5	5098891	5099056	S18_peak_2669	Intergenic between ENST00000507950 and ENST00000411075	ENST00000507950
AKR1B10P1	10	67750570	67750796	S18_peak_267	promoter-TSS (ENST00000425765)	ENST00000425765
ADAMTS16	5	5186981	5187146	S18_peak_2670	intron (ENST00000274181, intron 5 of 22)	ENST00000274181
ADAMTS16	5	5195945	5196110	S18_peak_2671	intron (ENST00000274181, intron 8 of 22)	ENST00000274181
ADAMTS16	5	5234615	5234780	S18_peak_2672	intron (ENST00000274181, intron 12 of 22)	ENST00000274181
MTCO1P31	5	5401369	5401534	S18_peak_2673	Intergenic between ENST00000506355 and ENST00000296564	ENST00000506355
ICE1	5	5639955	5640123	S18_peak_2674	Intergenic between ENST00000296564 and ENST00000564741	ENST00000296564
ICE1	5	5644457	5644622	S18_peak_2675	Intergenic between ENST00000296564 and ENST00000564741	ENST00000296564
CTC-471C19.1	5	5961597	5961821	S18_peak_2676	Intergenic between ENST00000296564 and ENST00000564741	ENST00000564741
HMGB3P3	5	6216745	6216910	S18_peak_2677	Intergenic between ENST00000624837 and ENST00000510526	ENST00000510526
UBE2QL1	5	6449652	6449988	S18_peak_2678	promoter-TSS (ENST00000399816)	ENST00000399816
RN7SKP79	5	6851199	6851387	S18_peak_2679	Intergenic between ENST00000363373 and ENST00000510622	ENST00000363373
SIRT1	10	67895217	67895382	S18_peak_268	intron (ENST00000212015, intron 4 of 8)	ENST00000212015
RP11-332J15.2	5	6895907	6896101	S18_peak_2680	Intergenic between ENST00000510622 and ENST00000512838	ENST00000510622
RP11-122F24.1	5	6981363	6981615	S18_peak_2681	intron (ENST00000512838, intron 1 of 5)	ENST00000512838
ADCY2	5	7520526	7520702	S18_peak_2682	intron (ENST00000338316, intron 2 of 24)	ENST00000338316

ADCY2	5	7598746	7598992	S18_peak_2683	intron (ENST00000338316, intron 3 of 24)	ENST00000338316
ADCY2	5	7633176	7633409	S18_peak_2684	intron (ENST00000338316, intron 4 of 24)	ENST00000338316
RP11-215I16.1	5	8174427	8174632	S18_peak_2685	Intergenic between ENST00000511351 and ENST00000501071	ENST00000511351
RP11-215I16.1	5	8202211	8202376	S18_peak_2686	Intergenic between ENST00000511351 and ENST00000501071	ENST00000511351
RP11-143A12.1	5	8957733	8957951	S18_peak_2687	Intergenic between ENST00000510067 and ENST00000624494	ENST00000624494
SEMA5A	5	9059679	9059939	S18_peak_2688	intron (ENST00000382496, intron 18 of 22)	ENST00000382496
SEMA5A	5	9129059	9129272	S18_peak_2689	intron (ENST00000382496, intron 13 of 22)	ENST00000382496
HNRNPH3	10	68315421	68315626	S18_peak_269	Intergenic between ENST00000443208 and ENST00000265866	ENST00000265866
SEMA5A	5	9445554	9445756	S18_peak_2690	intron (ENST00000382496, intron 1 of 22)	ENST00000382496
SEMA5A	5	9509408	9509650	S18_peak_2691	intron (ENST00000382496, intron 1 of 22)	ENST00000382496
SEMA5A	5	9515583	9515794	S18_peak_2692	intron (ENST00000382496, intron 1 of 22)	ENST00000382496
SNHG18	5	9549771	9549990	S18_peak_2693	exon (ENST00000508179, exon 3 of 3)	ENST00000508179
SNORD123	5	9566689	9566904	S18_peak_2694	Intergenic between ENST00000459473 and ENST00000504182	ENST00000459473
CTD-2001E22.1	5	9587558	9587723	S18_peak_2695	Intergenic between ENST00000459473 and ENST00000504182	ENST00000504182
CTD-2143L24.1	5	9860605	9860790	S18_peak_2696	intron (ENST00000511616, intron 4 of 12)	ENST00000511616
CTD-2143L24.1	5	9914052	9914217	S18_peak_2697	Intergenic between ENST00000606169 and ENST00000623143	ENST00000606169
RP11-191F9.1	5	10077437	10077756	S18_peak_2698	Intergenic between ENST00000624400 and ENST00000623432	ENST00000623432
CTD-2199O4.4	5	10159424	10159634	S18_peak_2699	Intergenic between ENST00000506299 and ENST00000623704	ENST00000623704
PUM1	1	30927360	30927525	S18_peak_27	Intergenic between ENST00000578099 and ENST00000373747	ENST00000373747
DNA2	10	68426715	68426960	S18_peak_270	intron (ENST00000358410, intron 14 of 20)	ENST00000358410
FAM173B	5	10222550	10222826	S18_peak_2700	Intergenic between ENST00000607861 and ENST00000511437	ENST00000511437
CTNND2	5	11027774	11027939	S18_peak_2701	intron (ENST00000304623, intron 16 of 21)	ENST00000304623
CTNND2	5	11150802	11150967	S18_peak_2702	intron (ENST00000304623, intron 12 of 21)	ENST00000304623
CTNND2	5	11645058	11645272	S18_peak_2703	intron (ENST00000304623, intron 2 of 21)	ENST00000304623
RNU6-679P	5	11916110	11916346	S18_peak_2704	Intergenic between ENST00000384582 and ENST00000391003	ENST00000391003
RP11-308B16.2	5	12493842	12494075	S18_peak_2705	Intergenic between ENST00000391003 and ENST00000502209	ENST00000502209
RP11-308B16.2	5	12501592	12501757	S18_peak_2706	Intergenic between ENST00000391003 and ENST00000502209	ENST00000502209
LINC01194	5	12723530	12723695	S18_peak_2707	intron (ENST00000505196, intron 3 of 3)	ENST00000505196
RP11-419C19.2	5	13061019	13061270	S18_peak_2708	Intergenic between ENST00000510065 and ENST00000513283	ENST00000510065

RPS23P5	5	13257860	13258119	S18_peak_2709	Intergenic between ENST00000495419 and ENST00000503735	ENST00000495419
HKDC1	10	69263167	69263332	S18_peak_271	intron (ENST00000354624, intron 16 of 17)	ENST00000354624
NENFP3	5	13503094	13503267	S18_peak_2710	Intergenic between ENST00000495419 and ENST00000503735	ENST00000503735
NENFP3	5	13508412	13508612	S18_peak_2711	Intergenic between ENST00000495419 and ENST00000503735	ENST00000503735
AC016549.1	5	14325451	14325652	S18_peak_2712	Intergenic between ENST00000503244 and ENST00000627417	ENST00000627417
ANKH	5	14745954	14746223	S18_peak_2713	intron (ENST00000284268, intron 6 of 11)	ENST00000284268
ANKH	5	14754347	14754616	S18_peak_2714	intron (ENST00000284268, intron 4 of 11)	ENST00000284268
U8	5	15094481	15094789	S18_peak_2715	Intergenic between ENST00000511618 and ENST00000365399	ENST00000365399
RP1-251I12.1	5	15148603	15148800	S18_peak_2716	Intergenic between ENST00000502680 and ENST00000511443	ENST00000502680
RP1-137K24.1	5	15299060	15299307	S18_peak_2717	Intergenic between ENST00000511443 and ENST00000507649	ENST00000511443
CTD-2350J17.1	5	15591413	15591679	S18_peak_2718	Intergenic between ENST00000502306 and ENST00000510456	ENST00000510456
MIR887	5	15806922	15807164	S18_peak_2719	Intergenic between ENST00000509228 and ENST00000401258	ENST00000401258
RP11-242G20.1	10	69734484	69734734	S18_peak_272	Intergenic between ENST00000434931 and ENST00000438138	ENST00000434931
MIR887	5	15924360	15924565	S18_peak_2720	Intergenic between ENST00000509228 and ENST00000401258	ENST00000401258
RNA5SP178	5	15993815	15994054	S18_peak_2721	Intergenic between ENST00000401258 and ENST00000410380	ENST00000410380
11-Mar	5	16133261	16133426	S18_peak_2722	intron (ENST00000332432, intron 2 of 3)	ENST00000332432
11-Mar	5	16170947	16171152	S18_peak_2723	intron (ENST00000332432, intron 2 of 3)	ENST00000332432
RP1-167G20.1	5	16395136	16395301	S18_peak_2724	intron (ENST00000513157, intron 6 of 7)	ENST00000513157
ZNF622	5	16446442	16446626	S18_peak_2725	Intergenic between ENST00000513041 and ENST00000308683	ENST00000308683
FAM134B	5	16602751	16603019	S18_peak_2726	intron (ENST00000306320, intron 1 of 8)	ENST00000306320
MYO10	5	16814584	16814797	S18_peak_2727	exon (ENST00000507288, exon 4 of 4)	ENST00000507288
CTD-2228A4.1	5	17105816	17105981	S18_peak_2728	Intergenic between ENST00000364566 and ENST00000606282	ENST00000606282
RP11-321E2.8	5	17524586	17524759	S18_peak_2729	Intergenic between ENST00000512357 and ENST00000510009	ENST00000510009
H2AFY2	10	70048395	70048560	S18_peak_273	Intergenic between ENST00000427247 and ENST00000373255	ENST00000373255
RP11-432M8.2	5	17584220	17584459	S18_peak_2731	TTS (ENST00000506051)	ENST00000506051
RP11-432M8.3	5	17592157	17592322	S18_peak_2732	Intergenic between ENST00000505742 and ENST00000505258	ENST00000505742
RP11-432M8.4	5	17593519	17593693	S18_peak_2733	TTS (ENST00000505258)	ENST00000505258

RP11-432M8.5	5	17597185	17597380	S18_peak_2734	promoter-TSS (ENST00000503077)	ENST00000503077
RP11-432M8.7	5	17599094	17599268	S18_peak_2735	Intergenic between ENST00000512613 and ENST00000511476	ENST00000512613
RP11-432M8.9	5	17610473	17610711	S18_peak_2736	Intergenic between ENST00000505613 and ENST00000503184	ENST00000503184
RP11-432M8.16	5	17649001	17649166	S18_peak_2737	promoter-TSS (ENST00000509526)	ENST00000509526
SNORD81	5	18201993	18202169	S18_peak_2738	Intergenic between ENST00000490372 and ENST00000390976	ENST00000390976
CTD-2023M8.1	5	18793898	18794089	S18_peak_2739	Intergenic between ENST00000512978 and ENST00000503954	ENST00000512978
NPFFR1	10	70266713	70266878	S18_peak_274	intron (ENST00000277942, intron 1 of 3)	ENST00000277942
HSPD1P15	5	19235467	19235794	S18_peak_2740	Intergenic between ENST00000505573 and ENST00000514549	ENST00000505573
HSPD1P15	5	19661680	19661903	S18_peak_2741	Intergenic between ENST00000505573 and ENST00000514549	ENST00000505573
RP11-420O16.1	5	20337976	20338354	S18_peak_2742	Intergenic between ENST00000513573 and ENST00000502427	ENST00000513573
RP11-774D14.1	5	20591360	20591525	S18_peak_2743	Intergenic between ENST00000513573 and ENST00000502427	ENST00000502427
RP11-774D14.1	5	20617052	20617323	S18_peak_2744	intron (ENST00000502427, intron 1 of 11)	ENST00000502427
RP11-774D14.1	5	20663825	20664033	S18_peak_2745	intron (ENST00000502427, intron 2 of 11)	ENST00000502427
RP11-774D14.1	5	20935175	20935647	S18_peak_2746	intron (ENST00000502427, intron 8 of 11)	ENST00000502427
RP11-697E23.1	5	20940876	20941048	S18_peak_2747	Intergenic between ENST00000515431 and ENST00000502928	ENST00000515431
RP11-811J10.1	5	21137896	21138122	S18_peak_2748	Intergenic between ENST00000515431 and ENST00000502928	ENST00000502928
RP11-811J10.1	5	21216524	21216792	S18_peak_2749	Intergenic between ENST00000502928 and ENST00000503488	ENST00000502928
ADAMTS14	10	70601774	70601978	S18_peak_275	Intergenic between ENST00000427625 and ENST00000373208	ENST00000373208
RP11-811J10.1	5	21218372	21218643	S18_peak_2750	Intergenic between ENST00000502928 and ENST00000503488	ENST00000502928
RP11-811J10.1	5	21223332	21223497	S18_peak_2751	Intergenic between ENST00000502928 and ENST00000503488	ENST00000502928
GUSBP1	5	21387376	21387627	S18_peak_2752	intron (ENST00000508260, intron 1 of 8)	ENST00000508260
RP11-804N13.1	5	21679139	21679354	S18_peak_2753	intron (ENST00000522350, intron 2 of 3)	ENST00000522350
CDH12	5	22018862	22019027	S18_peak_2754	intron (ENST00000504376, intron 4 of 13)	ENST00000504376
CDH12	5	22125502	22125758	S18_peak_2755	intron (ENST00000504376, intron 3 of 13)	ENST00000504376
CDH12	5	22446211	22446450	S18_peak_2756	intron (ENST00000504376, intron 2 of 13)	ENST00000504376
CDH12	5	22615935	22616100	S18_peak_2757	intron (ENST00000504376, intron 1 of 13)	ENST00000504376
CDH12	5	22697538	22697703	S18_peak_2758	intron (ENST00000504376, intron 1 of 13)	ENST00000504376
CDH12	5	22777877	22778104	S18_peak_2759	intron (ENST00000504376, intron 1 of 13)	ENST00000504376
MIR7152	10	71790227	71790392	S18_peak_276	Intergenic between ENST00000394957 and ENST00000619512	ENST00000619512

CTD-2272G21.3	5	23270465	23270667	S18_peak_2760	Intergenic between ENST00000509054 and ENST00000384185	ENST00000509054
RP11-197O8.1	5	23697125	23697370	S18_peak_2761	Intergenic between ENST00000511286 and ENST00000512559	ENST00000511286
C5orf17	5	23993298	23993463	S18_peak_2762	intron (ENST00000512559, intron 4 of 4)	ENST00000512559
Metazoa_SRP	5	24465935	24466235	S18_peak_2763	Intergenic between ENST00000612585 and ENST00000264463	ENST00000612585
RP11-549K20.1	5	25105367	25105683	S18_peak_2764	Intergenic between ENST00000363041 and ENST00000507600	ENST00000507600
RP11-184E9.1	5	25265131	25265477	S18_peak_2765	intron (ENST00000502100, intron 1 of 4)	ENST00000502100
RP11-184E9.3	5	25338573	25338806	S18_peak_2766	Intergenic between ENST00000562632 and ENST00000623006	ENST00000623006
CTD-2306M5.1	5	25429968	25430133	S18_peak_2767	intron (ENST00000507887, intron 1 of 2)	ENST00000507887
RNU6-374P	5	25625714	25626014	S18_peak_2768	Intergenic between ENST00000623500 and ENST00000364146	ENST00000364146
RNU4-43P	5	25963178	25963394	S18_peak_2769	Intergenic between ENST00000511640 and ENST00000410628	ENST00000410628
MIR7152	10	71806506	71806671	S18_peak_277	Intergenic between ENST00000619512 and ENST00000459501	ENST00000619512
RP11-351N6.1	5	26321188	26321462	S18_peak_2770	Intergenic between ENST00000410628 and ENST00000512939	ENST00000512939
CTD-2533K21.1	5	26679678	26679959	S18_peak_2771	Intergenic between ENST00000509113 and ENST00000506032	ENST00000509113
CDH9	5	26961970	26962172	S18_peak_2772	intron (ENST00000231021, intron 2 of 11)	ENST00000231021
CDH9	5	27064476	27064641	S18_peak_2773	Intergenic between ENST00000231021 and ENST00000384531	ENST00000231021
RNU6-738P	5	27179614	27179779	S18_peak_2774	Intergenic between ENST00000384531 and ENST00000510512	ENST00000384531
RNU6-738P	5	27230235	27230443	S18_peak_2775	Intergenic between ENST00000384531 and ENST00000510512	ENST00000384531
LINC01021	5	27530899	27531064	S18_peak_2776	Intergenic between ENST00000514255 and ENST00000510368	ENST00000514255
LINC01021	5	27620032	27620197	S18_peak_2777	Intergenic between ENST00000514255 and ENST00000510368	ENST00000514255
LINC01021	5	27695111	27695287	S18_peak_2778	Intergenic between ENST00000514255 and ENST00000510368	ENST00000514255
LINC01021	5	27769350	27769558	S18_peak_2779	Intergenic between ENST00000514255 and ENST00000510368	ENST00000514255
CHST3	10	72014133	72014298	S18_peak_278	TTS (ENST00000373115)	ENST00000373115
RP11-560A7.1	5	27993511	27993680	S18_peak_2780	Intergenic between ENST00000514255 and ENST00000510368	ENST00000510368
RP11-560A7.1	5	28077952	28078178	S18_peak_2781	Intergenic between ENST00000514255 and ENST00000510368	ENST00000510368
CTD-2134P3.2	5	28844529	28844792	S18_peak_2782	Intergenic between ENST00000603374 and	ENST00000603374

					ENST00000603743	
SNORA18	5	29047798	29047963	S18_peak_2783	Intergenic between ENST00000514739 and ENST00000516792	ENST00000516792
SNORA18	5	29081934	29082205	S18_peak_2784	Intergenic between ENST00000516792 and ENST00000512207	ENST00000516792
CTD-2345H1.1	5	29522920	29523144	S18_peak_2785	Intergenic between ENST00000611900 and ENST00000507979	ENST00000611900
CTD-2351A16.1	5	29988634	29988799	S18_peak_2786	Intergenic between ENST00000623357 and ENST00000505250	ENST00000623357
HPRT1P2	5	30199263	30199541	S18_peak_2787	Intergenic between ENST00000623357 and ENST00000505250	ENST00000505250
RP11-136H13.2	5	30593808	30593995	S18_peak_2788	Intergenic between ENST00000502347 and ENST00000495944	ENST00000502347
RPL19P11	5	31037415	31037580	S18_peak_2789	Intergenic between ENST00000502347 and ENST00000495944	ENST00000495944
SPOCK2	10	72089305	72089470	S18_peak_279	Intergenic between ENST00000373109 and ENST00000317168	ENST00000373109
RP11-152K4.2	5	31195841	31196051	S18_peak_2790	intron (ENST00000523584, intron 1 of 4)	ENST00000523584
PDZD2	5	31687723	31687950	S18_peak_2791	intron (ENST00000438447, intron 1 of 24)	ENST00000438447
PDZD2	5	31755899	31756198	S18_peak_2792	intron (ENST00000438447, intron 1 of 24)	ENST00000438447
PDZD2	5	31924310	31924475	S18_peak_2793	intron (ENST00000438447, intron 2 of 24)	ENST00000438447
PDZD2	5	31930202	31930372	S18_peak_2794	intron (ENST00000438447, intron 2 of 24)	ENST00000438447
PDZD2	5	31944252	31944469	S18_peak_2795	intron (ENST00000438447, intron 2 of 24)	ENST00000438447
PDZD2	5	32060919	32061197	S18_peak_2796	exon (ENST00000438447, exon 14 of 25)	ENST00000438447
GOLPH3	5	32131060	32131225	S18_peak_2797	intron (ENST00000265070, intron 3 of 3)	ENST00000265070
CTD-2186M15.3	5	32182104	32182368	S18_peak_2798	Intergenic between ENST00000606994 and ENST00000477507	ENST00000606994
MTMR12	5	32244361	32244540	S18_peak_2799	intron (ENST00000382142, intron 10 of 15)	ENST00000382142
Metazoa_SRP	1	33044394	33044624	S18_peak_28	Intergenic between ENST00000610352 and ENST00000294517	ENST00000610352
RP11-167P22.4	10	72546753	72546982	S18_peak_280	Intergenic between ENST00000431638 and ENST00000425543	ENST00000425543
MTMR12	5	32254850	32255015	S18_peak_2800	intron (ENST00000382142, intron 8 of 15)	ENST00000382142
MTMR12	5	32302673	32302863	S18_peak_2801	intron (ENST00000382142, intron 1 of 15)	ENST00000382142
CTD-2004A9.1	5	32649666	32649924	S18_peak_2802	intron (ENST00000514225, intron 1 of 1)	ENST00000514225
CTD-2066L21.3	5	33096874	33097045	S18_peak_2803	intron (ENST00000510327, intron 2 of 2)	ENST00000510327
CTD-2066L21.3	5	33217408	33217688	S18_peak_2804	intron (ENST00000510327, intron 2 of 2)	ENST00000510327
ADAMTS12	5	33582002	33582184	S18_peak_2805	intron (ENST00000504830, intron 18 of 23)	ENST00000504830
ADAMTS12	5	33729613	33729779	S18_peak_2806	intron (ENST00000504830, intron 3 of 23)	ENST00000504830
ADAMTS12	5	33740782	33741027	S18_peak_2807	intron (ENST00000504830, intron 3 of 23)	ENST00000504830
ADAMTS12	5	33803536	33803760	S18_peak_2808	intron (ENST00000504830, intron 2 of 23)	ENST00000504830
SLC45A2	5	33949869	33950112	S18_peak_2809	intron (ENST00000296589, intron 5 of 6)	ENST00000296589
HMGN2P34	10	72636176	72636341	S18_peak_281	TTS (ENST00000437647)	ENST00000437647
SLC45A2	5	33963255	33963420	S18_peak_2810	intron (ENST00000296589, intron 3 of 6)	ENST00000296589

C1QTNF3-AMACR	5	34031793	34031980	S18_peak_2811	intron (ENST00000382079, intron 3 of 8)	ENST00000382079
RP11-1250I15.1	5	34141125	34141327	S18_peak_2812	Intergenic between ENST00000623525 and ENST00000607051	ENST00000623525
RP11-1023L17.1	5	34293023	34293188	S18_peak_2814	Intergenic between ENST00000512782 and ENST00000503549	ENST00000512782
RP11-1325J9.1	5	34371897	34372062	S18_peak_2815	Intergenic between ENST00000512782 and ENST00000503549	ENST00000503549
RP11-1325J9.1	5	34410797	34410962	S18_peak_2816	Intergenic between ENST00000503549 and ENST00000606491	ENST00000503549
RP11-1325J9.1	5	34456325	34456549	S18_peak_2817	Intergenic between ENST00000503549 and ENST00000606491	ENST00000503549
CTD-2517O10.6	5	34837367	34837539	S18_peak_2818	TTS (ENST00000606401)	ENST00000606401
PRLR	5	35121491	35121694	S18_peak_2819	intron (ENST00000618457, intron 1 of 9)	ENST00000618457
AGAP5	10	73684781	73684946	S18_peak_282	intron (ENST00000443782, intron 3 of 6)	ENST00000443782
U3	5	35516578	35516743	S18_peak_2820	Intergenic between ENST00000364498 and ENST00000356031	ENST00000364498
SPEF2	5	35634371	35634554	S18_peak_2821	intron (ENST00000356031, intron 2 of 36)	ENST00000356031
SPEF2	5	35730259	35730424	S18_peak_2822	intron (ENST00000356031, intron 21 of 36)	ENST00000356031
NADK2	5	36202610	36202799	S18_peak_2823	intron (ENST00000397338, intron 8 of 11)	ENST00000397338
RANBP3L	5	36323566	36323731	S18_peak_2824	Intergenic between ENST00000502994 and ENST00000410246	ENST00000502994
CTD-2282P23.1	5	36584498	36584691	S18_peak_2825	Intergenic between ENST00000410246 and ENST00000474150	ENST00000474150
CTD-2653M23.3	5	36805976	36806141	S18_peak_2826	Intergenic between ENST00000508745 and ENST00000605892	ENST00000605892
NIPBL	5	36970885	36971050	S18_peak_2827	exon (ENST00000282516, exon 7 of 47)	ENST00000282516
NIPBL	5	37063519	37063832	S18_peak_2828	intron (ENST00000282516, intron 45 of 46)	ENST00000282516
WDR70	5	37386286	37386508	S18_peak_2829	intron (ENST00000265107, intron 3 of 17)	ENST00000265107
PLAU	10	73956834	73957092	S18_peak_283	Intergenic between ENST00000372764 and ENST00000372755	ENST00000372764
WDR70	5	37388497	37388733	S18_peak_2830	intron (ENST00000265107, intron 3 of 17)	ENST00000265107
GDNF-AS1	5	37880858	37881023	S18_peak_2831	Intergenic between ENST00000510986 and ENST00000513673	ENST00000510986
CTD-2116N24.1	5	37996928	37997241	S18_peak_2832	Intergenic between ENST00000506429 and ENST00000513039	ENST00000513039
LIFR	5	38565243	38565417	S18_peak_2833	intron (ENST00000263409, intron 1 of 19)	ENST00000263409
LIFR	5	38573917	38574207	S18_peak_2834	intron (ENST00000263409, intron 1 of 19)	ENST00000263409
RICTOR	5	39018506	39018671	S18_peak_2835	intron (ENST00000357387, intron 3 of 37)	ENST00000357387
FYB	5	39102196	39102361	S18_peak_2836	Intergenic between ENST00000503376 and ENST00000515010	ENST00000515010
INTS6P1	5	39746600	39746765	S18_peak_2837	Intergenic between ENST00000504674 and ENST00000512554	ENST00000504674
GCSHP1	5	39851610	39851775	S18_peak_2838	Intergenic between ENST00000504674 and ENST00000512554	ENST00000512554

MROH2B	5	41068205	41068370	S18_peak_2839	intron (ENST00000399564, intron 2 of 41)	ENST00000399564
PPIAP13	10	75086016	75086206	S18_peak_284	Intergenic between ENST00000338487 and ENST00000434706	ENST00000434706
MTHFD2P6	5	41975843	41976008	S18_peak_2840	Intergenic between ENST00000506170 and ENST00000564402	ENST00000506170
MTHFD2P6	5	42050923	42051100	S18_peak_2841	Intergenic between ENST00000506170 and ENST00000564402	ENST00000506170
RP11-112L7.1	5	42211921	42212091	S18_peak_2842	Intergenic between ENST00000564402 and ENST00000230882	ENST00000564402
GHR	5	42393672	42393879	S18_peak_2843	Intergenic between ENST00000564402 and ENST00000230882	ENST00000230882
PRELID3BP6	5	42941358	42941523	S18_peak_2844	Intergenic between ENST00000503839 and ENST00000503606	ENST00000503839
RP11-159F24.2	5	43337771	43337960	S18_peak_2845	promoter-TSS (ENST00000511991)	ENST00000511991
RP11-8L21.1	5	43949716	43950013	S18_peak_2846	Intergenic between ENST00000508829 and ENST00000391259	ENST00000508829
HCN1	5	45352448	45352705	S18_peak_2847	intron (ENST00000303230, intron 5 of 7)	ENST00000303230
HCN1	5	45466827	45467008	S18_peak_2848	intron (ENST00000303230, intron 2 of 7)	ENST00000303230
HCN1	5	45585091	45585281	S18_peak_2849	intron (ENST00000303230, intron 2 of 7)	ENST00000303230
SAMD8	10	75124609	75124774	S18_peak_285	intron (ENST00000372687, intron 1 of 4)	ENST00000372687
CTD-2013M15.1	5	45976602	45976815	S18_peak_2850	Intergenic between ENST00000510987 and ENST00000303221	ENST00000510987
CTD-2013M15.1	5	45983163	45983328	S18_peak_2851	Intergenic between ENST00000510987 and ENST00000303221	ENST00000510987
CTD-2013M15.1	5	46305124	46305346	S18_peak_2852	Intergenic between ENST00000510987 and ENST00000303221	ENST00000510987
CTD-2013M15.1	5	47299263	47299428	S18_peak_2853	Intergenic between ENST00000510987 and ENST00000303221	ENST00000510987
CTD-2013M15.1	5	47619910	47620085	S18_peak_2855	Intergenic between ENST00000510987 and ENST00000303221	ENST00000510987
CTD-2013M15.1	5	47638138	47638429	S18_peak_2856	Intergenic between ENST00000510987 and ENST00000303221	ENST00000510987
CTD-2013M15.1	5	47656211	47656418	S18_peak_2857	Intergenic between ENST00000510987 and ENST00000303221	ENST00000510987
CTD-2013M15.1	5	47778505	47778670	S18_peak_2858	Intergenic between ENST00000510987 and ENST00000303221	ENST00000510987
COMTD1	10	75233958	75234181	S18_peak_286	promoter-TSS (ENST00000372538)	ENST00000372538
CTD-2013M15.1	5	47899868	47900149	S18_peak_2860	Intergenic between ENST00000510987 and ENST00000303221	ENST00000510987
CTD-2013M15.1	5	47989054	47989225	S18_peak_2861	Intergenic between ENST00000510987 and ENST00000303221	ENST00000510987
CTD-2013M15.1	5	48036774	48036939	S18_peak_2862	Intergenic between ENST00000510987 and ENST00000303221	ENST00000510987
EMB	5	48165993	48166158	S18_peak_2863	Intergenic between ENST00000510987 and	ENST00000303221

					ENST00000303221	
EMB	5	48312054	48312219	S18_peak_2864	Intergenic between ENST00000510987 and ENST00000303221	ENST00000303221
EMB	5	48316814	48317032	S18_peak_2865	Intergenic between ENST00000510987 and ENST00000303221	ENST00000303221
EMB	5	48505951	48506123	S18_peak_2866	Intergenic between ENST00000510987 and ENST00000303221	ENST00000303221
EMB	5	48559589	48559815	S18_peak_2867	Intergenic between ENST00000510987 and ENST00000303221	ENST00000303221
EMB	5	48637575	48637906	S18_peak_2868	Intergenic between ENST00000510987 and ENST00000303221	ENST00000303221
EMB	5	48659182	48659370	S18_peak_2869	Intergenic between ENST00000510987 and ENST00000303221	ENST00000303221
RNU6-673P	10	76259505	76259730	S18_peak_287	Intergenic between ENST00000491887 and ENST00000365084	ENST00000365084
EMB	5	48838321	48838627	S18_peak_2870	Intergenic between ENST00000510987 and ENST00000303221	ENST00000303221
EMB	5	48848616	48848781	S18_peak_2871	Intergenic between ENST00000510987 and ENST00000303221	ENST00000303221
EMB	5	48924289	48924488	S18_peak_2872	Intergenic between ENST00000510987 and ENST00000303221	ENST00000303221
EMB	5	48960920	48961093	S18_peak_2873	Intergenic between ENST00000510987 and ENST00000303221	ENST00000303221
EMB	5	48990334	48990558	S18_peak_2874	Intergenic between ENST00000510987 and ENST00000303221	ENST00000303221
EMB	5	49027254	49027421	S18_peak_2875	Intergenic between ENST00000510987 and ENST00000303221	ENST00000303221
EMB	5	49087314	49087543	S18_peak_2877	Intergenic between ENST00000510987 and ENST00000303221	ENST00000303221
EMB	5	49169518	49169861	S18_peak_2878	Intergenic between ENST00000510987 and ENST00000303221	ENST00000303221
EMB	5	49306920	49307123	S18_peak_2879	Intergenic between ENST00000510987 and ENST00000303221	ENST00000303221
COX6CP15	10	77410758	77410923	S18_peak_288	Intergenic between ENST00000418515 and ENST00000426313	ENST00000426313
EMB	5	49403752	49403917	S18_peak_2880	Intergenic between ENST00000510987 and ENST00000303221	ENST00000303221
RNA5SP182	5	51789556	51789822	S18_peak_2883	Intergenic between ENST00000509423 and ENST00000516064	ENST00000516064
CTD-2037L6.2	5	58130444	58130609	S18_peak_2884	Intergenic between ENST00000505861 and ENST00000504014	ENST00000505861
CWC27	5	64893627	64893861	S18_peak_2885	intron (ENST00000381070, intron 11 of 13)	ENST00000381070
NLN	5	65769611	65769864	S18_peak_2886	intron (ENST00000380985, intron 3 of 12)	ENST00000380985
ERBIN	5	65997986	65998169	S18_peak_2887	intron (ENST00000284037, intron 4 of 25)	ENST00000284037

CTD-2016O11.1	5	66245750	66245915	S18_peak_2888	Intergenic between ENST00000509924 and ENST00000510964	ENST00000509924
CTD-2016O11.1	5	66253149	66253360	S18_peak_2889	Intergenic between ENST00000509924 and ENST00000510964	ENST00000509924
RP11-90J7.3	10	78348031	78348348	S18_peak_289	intron (ENST00000634930, intron 1 of 3)	ENST00000634930
RP11-5P22.1	5	66558592	66558846	S18_peak_2890	Intergenic between ENST00000508486 and ENST00000414951	ENST00000414951
MAST4	5	66658292	66658457	S18_peak_2891	intron (ENST00000403625, intron 1 of 28)	ENST00000403625
MAST4	5	66664987	66665152	S18_peak_2892	intron (ENST00000403625, intron 1 of 28)	ENST00000403625
MAST4	5	67046127	67046380	S18_peak_2893	intron (ENST00000403625, intron 4 of 28)	ENST00000403625
EEF1B2P2	5	68171231	68171396	S18_peak_2894	Intergenic between ENST00000519625 and ENST00000520762	ENST00000519625
CTC-340D7.1	5	68835794	68836147	S18_peak_2895	intron (ENST00000503268, intron 2 of 2)	ENST00000503268
SNORA50	5	69149692	69149873	S18_peak_2896	Intergenic between ENST00000504129 and ENST00000408059	ENST00000408059
MRPS36	5	69215732	69216001	S18_peak_2897	Intergenic between ENST00000283006 and ENST00000256441	ENST00000256441
MARVELD2	5	69448157	69448383	S18_peak_2898	Intergenic between ENST00000436532 and ENST00000496370	ENST00000436532
RP11-974F13.5	5	69725628	69725827	S18_peak_2899	Intergenic between ENST00000514492 and ENST00000506490	ENST00000514492
RP5-884C9.2	1	38060167	38060419	S18_peak_29	intron (ENST00000432922, intron 1 of 3)	ENST00000432922
RP11-90J7.3	10	78587343	78587582	S18_peak_290	intron (ENST00000421324, intron 2 of 2)	ENST00000421324
RP11-98J23.2	5	69858901	69859066	S18_peak_2900	Intergenic between ENST00000514492 and ENST00000506490	ENST00000506490
RP11-98J23.2	5	69900219	69900503	S18_peak_2901	intron (ENST00000506490, intron 1 of 4)	ENST00000506490
CDH12P2	5	69947308	69947556	S18_peak_2902	Intergenic between ENST00000505635 and ENST00000504393	ENST00000504393
SERF1B	5	70018255	70018420	S18_peak_2903	Intergenic between ENST00000504393 and ENST00000380751	ENST00000380751
SERF1B	5	70038327	70038540	S18_peak_2904	intron (ENST00000380750, intron 2 of 2)	ENST00000380750
SERF1B	5	70056467	70056739	S18_peak_2905	Intergenic between ENST00000380750 and ENST00000511830	ENST00000380750
RP11-1415C14.3	5	70219777	70219985	S18_peak_2906	TTS (ENST00000514522)	ENST00000514522
RP11-497H16.9	5	70456071	70456289	S18_peak_2907	Intergenic between ENST00000602697 and ENST00000425529	ENST00000602697
RP11-497H16.8	5	70502472	70502735	S18_peak_2908	Intergenic between ENST00000509969 and ENST00000508439	ENST00000509969
RP11-589F5.4	5	70772676	70772847	S18_peak_2909	intron (ENST00000505516, intron 1 of 4)	ENST00000505516
RP11-31E13.2	10	78728631	78728796	S18_peak_291	Intergenic between ENST00000455498 and ENST00000456353	ENST00000455498
NAIP	5	71012505	71012867	S18_peak_2910	exon (ENST00000508426, exon 2 of 14)	ENST00000508426
GTF2H2	5	71069147	71069312	S18_peak_2911	Intergenic between ENST00000519783 and ENST00000445744	ENST00000519783

GUSBP9	5	71204285	71204498	S18_peak_2912	intron (ENST00000510138, intron 1 of 3)	ENST00000510138
GUSBP9	5	71211992	71212185	S18_peak_2913	Intergenic between ENST00000510138 and ENST00000505946	ENST00000510138
MCCC2	5	71569664	71569829	S18_peak_2914	Intergenic between ENST00000499125 and ENST00000340941	ENST00000340941
MCCC2	5	71575288	71575473	S18_peak_2915	Intergenic between ENST00000499125 and ENST00000340941	ENST00000340941
MCCC2	5	71658393	71658558	S18_peak_2916	exon (ENST00000340941, exon 17 of 17)	ENST00000340941
MAP1B	5	71999632	71999797	S18_peak_2917	Intergenic between ENST00000614164 and ENST00000296755	ENST00000296755
MAP1B	5	72202521	72202711	S18_peak_2918	intron (ENST00000296755, intron 5 of 6)	ENST00000296755
CTC-347C20.2	5	72608989	72609179	S18_peak_2919	intron (ENST00000512573, intron 1 of 2)	ENST00000512573
PPIF	10	79368076	79368263	S18_peak_292	Intergenic between ENST00000225174 and ENST00000372336	ENST00000225174
CTD-2372A4.1	5	73799383	73799563	S18_peak_2920	Intergenic between ENST00000506717 and ENST00000508746	ENST00000508746
CTD-2292M14.1	5	74092131	74092296	S18_peak_2921	intron (ENST00000511395, intron 1 of 1)	ENST00000511395
ENC1	5	74617150	74617460	S18_peak_2922	Intergenic between ENST00000507781 and ENST00000302351	ENST00000302351
HEXB	5	74655511	74655676	S18_peak_2923	intron (ENST00000511181, intron 1 of 13)	ENST00000511181
HEXB	5	74678100	74678265	S18_peak_2924	intron (ENST00000511181, intron 1 of 13)	ENST00000511181
LINC01336	5	75114850	75115015	S18_peak_2925	Intergenic between ENST00000506086 and ENST00000506790	ENST00000506086
SUMO2P5	5	75162070	75162235	S18_peak_2926	Intergenic between ENST00000506086 and ENST00000506790	ENST00000506790
SUMO2P5	5	75203261	75203426	S18_peak_2927	Intergenic between ENST00000506086 and ENST00000506790	ENST00000506790
POLK	5	75512752	75512917	S18_peak_2928	promoter-TSS (ENST00000241436)	ENST00000241436
SNRPCP2	5	76365660	76365890	S18_peak_2929	Intergenic between ENST00000502589 and ENST00000504370	ENST00000504370
NUTM2B-AS1	10	79817732	79818010	S18_peak_293	intron (ENST00000601369, intron 1 of 6)	ENST00000601369
IQGAP2	5	76516962	76517219	S18_peak_2930	intron (ENST00000274364, intron 2 of 35)	ENST00000274364
AGGF1	5	77053240	77053504	S18_peak_2931	intron (ENST00000312916, intron 9 of 13)	ENST00000312916
TBCA	5	77720466	77720876	S18_peak_2932	intron (ENST00000380377, intron 1 of 3)	ENST00000380377
RP11-107N7.1	5	77971536	77971701	S18_peak_2933	Intergenic between ENST00000523413 and ENST00000519295	ENST00000523413
SCAMP1-AS1	5	78358701	78358903	S18_peak_2934	promoter-TSS (ENST00000513755)	ENST00000513755
HMGB1P21	5	78627302	78627467	S18_peak_2935	Intergenic between ENST00000621999 and ENST00000502313	ENST00000502313
HMGB1P21	5	78670079	78670244	S18_peak_2936	Intergenic between ENST00000502313 and ENST00000519121	ENST00000502313
CTD-2045M21.1	5	78746987	78747321	S18_peak_2937	Intergenic between ENST00000519121 and ENST00000521306	ENST00000521306
ARSB	5	78933839	78934004	S18_peak_2938	intron (ENST00000264914, intron 4 of 7)	ENST00000264914

DMGDH	5	79034101	79034281	S18_peak_2939	intron (ENST00000255189, intron 7 of 15)	ENST00000255189
TSPAN14	10	80530067	80530232	S18_peak_294	exon (ENST00000429989, exon 9 of 9)	ENST00000429989
BHMT2	5	79079348	79079578	S18_peak_2940	intron (ENST00000255192, intron 3 of 7)	ENST00000255192
HOMER1	5	79417120	79417339	S18_peak_2941	intron (ENST00000334082, intron 5 of 8)	ENST00000334082
LINC01455	5	79964406	79964632	S18_peak_2942	intron (ENST00000503167, intron 2 of 2)	ENST00000503167
SERINC5	5	80236123	80236332	S18_peak_2943	intron (ENST00000507668, intron 1 of 11)	ENST00000507668
RASGRF2	5	81102272	81102505	S18_peak_2944	intron (ENST00000265080, intron 12 of 26)	ENST00000265080
RASGRF2	5	81190159	81190343	S18_peak_2945	intron (ENST00000265080, intron 18 of 26)	ENST00000265080
ZCCHC9	5	81317568	81317757	S18_peak_2946	Intergenic between ENST00000438268 and ENST00000307624	ENST00000438268
ACOT12	5	81370306	81370471	S18_peak_2947	intron (ENST00000307624, intron 3 of 14)	ENST00000307624
SHFM1P1	5	81928470	81928635	S18_peak_2948	Intergenic between ENST00000506694 and ENST00000282185	ENST00000506694
ATG10	5	81991929	81992094	S18_peak_2949	intron (ENST00000282185, intron 2 of 7)	ENST00000282185
RP11-202D18.2	10	81871099	81871264	S18_peak_295	Intergenic between ENST00000419698 and ENST00000440589	ENST00000440589
ATG10	5	82227511	82227749	S18_peak_2950	intron (ENST00000282185, intron 5 of 7)	ENST00000282185
CTD-2015A6.2	5	82613103	82613332	S18_peak_2951	Intergenic between ENST00000510845 and ENST00000509506	ENST00000510845
CTD-2015A6.2	5	82644977	82645142	S18_peak_2952	Intergenic between ENST00000510845 and ENST00000509506	ENST00000510845
VCAN	5	83574159	83574324	S18_peak_2953	intron (ENST00000265077, intron 13 of 14)	ENST00000265077
HAPLN1	5	83695253	83695425	S18_peak_2954	intron (ENST00000274341, intron 1 of 4)	ENST00000274341
EDIL3	5	84358703	84358868	S18_peak_2955	intron (ENST00000296591, intron 1 of 10)	ENST00000296591
RP11-509M23.1	5	84733667	84733949	S18_peak_2956	Intergenic between ENST00000502253 and ENST00000425840	ENST00000425840
CTC-534B23.1	5	85605749	85605914	S18_peak_2957	Intergenic between ENST00000512074 and ENST00000507387	ENST00000512074
CTD-2036A18.2	5	85887853	85888018	S18_peak_2958	Intergenic between ENST00000504046 and ENST00000512479	ENST00000504046
CTC-261N6.1	5	86346956	86347121	S18_peak_2959	Intergenic between ENST00000505348 and ENST00000510946	ENST00000510946
RNU6-441P	10	82354010	82354225	S18_peak_296	Intergenic between ENST00000384545 and ENST00000363904	ENST00000384545
CTD-2299E8.1	5	86407882	86408047	S18_peak_2960	Intergenic between ENST00000502592 and ENST00000247655	ENST00000502592
RP11-72L22.1	5	87007314	87007480	S18_peak_2961	intron (ENST00000503349, intron 1 of 2)	ENST00000503349
RP11-72L22.1	5	87160099	87160264	S18_peak_2962	Intergenic between ENST00000515750 and ENST00000611331	ENST00000515750
CTD-2316B1.1	5	87600252	87600417	S18_peak_2963	Intergenic between ENST00000363592 and ENST00000504287	ENST00000504287
CTD-2316B1.2	5	87702750	87702943	S18_peak_2964	Intergenic between ENST00000504287 and ENST00000511417	ENST00000511417
TMEM161B	5	88038046	88038211	S18_peak_2965	Intergenic between ENST00000511417 and	ENST00000514135

					ENST00000514135	
TMEM161B-AS1	5	88364521	88364686	S18_peak_2966	intron (ENST00000504922, intron 2 of 2)	ENST00000504922
TMEM161B-AS1	5	88396652	88396845	S18_peak_2967	intron (ENST00000504922, intron 2 of 2)	ENST00000504922
MEF2C-AS1	5	89095460	89095708	S18_peak_2968	intron (ENST00000512585, intron 3 of 5)	ENST00000512585
RP11-61G23.2	5	90338488	90338659	S18_peak_2969	Intergenic between ENST00000519336 and ENST00000520032	ENST00000520032
RNU6-129P	10	83776310	83776475	S18_peak_297	Intergenic between ENST00000391022 and ENST00000432108	ENST00000391022
ADGRV1	5	90791852	90792097	S18_peak_2970	intron (ENST00000405460, intron 70 of 89)	ENST00000405460
ADGRV1	5	90839335	90839500	S18_peak_2971	intron (ENST00000405460, intron 77 of 89)	ENST00000405460
ADGRV1	5	91015308	91015473	S18_peak_2972	intron (ENST00000405460, intron 85 of 89)	ENST00000405460
LUCAT1	5	91298843	91299008	S18_peak_2973	Intergenic between ENST00000440769 and ENST00000513492	ENST00000513492
ARRDC3-AS1	5	91416982	91417147	S18_peak_2974	intron (ENST00000625713, intron 1 of 2)	ENST00000625713
uc_338	5	91913660	91913873	S18_peak_2975	Intergenic between ENST00000621804 and ENST00000507217	ENST00000621804
RP11-348J24.2	5	92104795	92105066	S18_peak_2976	intron (ENST00000507217, intron 1 of 3)	ENST00000507217
RP11-348J24.2	5	92302039	92302252	S18_peak_2977	intron (ENST00000507217, intron 2 of 3)	ENST00000507217
CTC-529L17.2	5	92690319	92690519	S18_peak_2978	Intergenic between ENST00000503489 and ENST00000502383	ENST00000503489
CTC-458G6.4	5	92762465	92762714	S18_peak_2979	Intergenic between ENST00000503489 and ENST00000502383	ENST00000502383
HMGN2P8	10	84080016	84080318	S18_peak_298	Intergenic between ENST00000363919 and ENST00000391458	ENST00000391458
POLD2P1	5	93172354	93172624	S18_peak_2980	Intergenic between ENST00000515153 and ENST00000511220	ENST00000511220
POLD2P1	5	93338081	93338270	S18_peak_2981	Intergenic between ENST00000511220 and ENST00000607797	ENST00000511220
FAM172A	5	93734858	93735023	S18_peak_2982	intron (ENST00000395965, intron 10 of 10)	ENST00000395965
FAM172A	5	93955812	93956093	S18_peak_2983	intron (ENST00000395965, intron 6 of 10)	ENST00000395965
MCTP1	5	94813655	94813868	S18_peak_2984	intron (ENST00000515393, intron 17 of 22)	ENST00000515393
MCTP1	5	94845701	94845909	S18_peak_2985	intron (ENST00000515393, intron 17 of 22)	ENST00000515393
TTC37	5	95545712	95545939	S18_peak_2986	intron (ENST00000358746, intron 4 of 42)	ENST00000358746
CTD-2337A12.1	5	96036818	96036983	S18_peak_2987	intron (ENST00000502645, intron 1 of 4)	ENST00000502645
CTD-2337A12.1	5	96262605	96262813	S18_peak_2988	intron (ENST00000502645, intron 1 of 4)	ENST00000502645
CAST	5	96654249	96654414	S18_peak_2989	Intergenic between ENST00000513158 and ENST00000338252	ENST00000338252
LINC01519	10	84998500	84998665	S18_peak_299	Intergenic between ENST00000389400 and ENST00000454178	ENST00000454178
CTD-2215E18.1	5	97114074	97114239	S18_peak_2990	intron (ENST00000504578, intron 3 of 6)	ENST00000504578
CTD-2215E18.1	5	97431903	97432079	S18_peak_2991	intron (ENST00000504578, intron 6 of 6)	ENST00000504578
AC008834.2	5	98045951	98046116	S18_peak_2992	Intergenic between ENST00000511612 and ENST00000505908	ENST00000511612
RP11-274E7.2	5	98211488	98211653	S18_peak_2993	Intergenic between ENST00000514912 and	ENST00000442824

					ENST00000442824	
CSNK1A1P3	5	98842936	98843153	S18_peak_2994	Intergenic between ENST00000504105 and ENST00000410678	ENST00000504105
CTD-2007H13.3	5	98973705	98973935	S18_peak_2995	intron (ENST00000513175, intron 1 of 2)	ENST00000513175
CTD-2007H13.1	5	99069238	99069486	S18_peak_2996	Intergenic between ENST00000484584 and ENST00000510435	ENST00000484584
CTD-2007H13.1	5	99128261	99128568	S18_peak_2997	Intergenic between ENST00000484584 and ENST00000510435	ENST00000484584
RP11-93O17.1	5	99354995	99355160	S18_peak_2998	Intergenic between ENST00000484584 and ENST00000510435	ENST00000510435
EEF1A1P20	5	99987326	99987491	S18_peak_2999	Intergenic between ENST00000511386 and ENST00000514852	ENST00000514852
KCNAB2	1	6074340	6074568	S18_peak_3	intron (ENST00000378097, intron 5 of 15)	ENST00000378097
CTPS1	1	40996199	40996395	S18_peak_30	intron (ENST00000372621, intron 8 of 18)	ENST00000372621
GRID1	10	85732033	85732274	S18_peak_300	intron (ENST00000327946, intron 8 of 15)	ENST00000327946
CTD-2001C12.1	5	100435975	100436140	S18_peak_3000	Intergenic between ENST00000511468 and ENST00000499025	ENST00000499025
MIR548P	5	100929238	100929403	S18_peak_3001	Intergenic between ENST00000408336 and ENST00000577858	ENST00000408336
MIR548P	5	101042079	101042244	S18_peak_3002	Intergenic between ENST00000408336 and ENST00000577858	ENST00000408336
SLCO6A1	5	102479038	102479203	S18_peak_3003	intron (ENST00000506729, intron 2 of 13)	ENST00000506729
SLCO6A1	5	102483991	102484156	S18_peak_3004	intron (ENST00000506729, intron 1 of 13)	ENST00000506729
LINC00491	5	102618054	102618219	S18_peak_3005	intron (ENST00000510145, intron 1 of 1)	ENST00000510145
PAM	5	102983136	102983348	S18_peak_3006	intron (ENST00000346918, intron 14 of 22)	ENST00000346918
RN7SL255P	5	104281760	104281925	S18_peak_3007	Intergenic between ENST00000470639 and ENST00000524336	ENST00000470639
RP11-6N13.1	5	104938099	104938343	S18_peak_3008	intron (ENST00000503650, intron 1 of 2)	ENST00000503650
RNA5SP189	5	105626361	105626526	S18_peak_3009	Intergenic between ENST00000519581 and ENST00000364920	ENST00000364920
GRID1	10	85967445	85967610	S18_peak_301	intron (ENST00000327946, intron 4 of 15)	ENST00000327946
RNA5SP189	5	105899493	105899658	S18_peak_3010	Intergenic between ENST00000519581 and ENST00000364920	ENST00000364920
CTC-278L1.1	5	106271986	106272151	S18_peak_3011	Intergenic between ENST00000364920 and ENST00000509159	ENST00000509159
CTC-254B4.1	5	107025566	107025731	S18_peak_3012	Intergenic between ENST00000505997 and ENST00000512850	ENST00000505997
RN7SKP122	5	107890522	107890687	S18_peak_3013	Intergenic between ENST00000515923 and ENST00000466800	ENST00000515923
RN7SKP122	5	108012641	108012954	S18_peak_3014	Intergenic between ENST00000515923 and ENST00000466800	ENST00000515923
CTD-2197I11.1	5	108811854	108812019	S18_peak_3015	Intergenic between ENST00000506742 and ENST00000510935	ENST00000510935
CTD-2197I11.1	5	108855465	108855630	S18_peak_3016	Intergenic between ENST00000510935 and	ENST00000510935

					ENST00000384673	
RP11-47F1.1	5	108951037	108951252	S18_peak_3017	Intergenic between ENST00000509173 and ENST00000383866	ENST00000509173
GJA1P1	5	109043687	109043925	S18_peak_3018	Intergenic between ENST00000383866 and ENST00000511478	ENST00000511478
GJA1P1	5	109142703	109142878	S18_peak_3019	Intergenic between ENST00000511478 and ENST00000512693	ENST00000511478
GRID1	10	86165020	86165243	S18_peak_302	intron (ENST00000327946, intron 3 of 15)	ENST00000327946
MAN2A1	5	109713499	109713664	S18_peak_3020	exon (ENST00000261483, exon 2 of 22)	ENST00000261483
PGAM5P1	5	109898003	109898168	S18_peak_3021	Intergenic between ENST00000518923 and ENST00000455884	ENST00000518923
TMEM232	5	110408265	110408430	S18_peak_3022	Intergenic between ENST00000518923 and ENST00000455884	ENST00000455884
TMEM232	5	110612455	110612693	S18_peak_3023	intron (ENST00000455884, intron 8 of 13)	ENST00000455884
TMEM232	5	110684794	110684959	S18_peak_3024	intron (ENST00000455884, intron 1 of 13)	ENST00000455884
CTD-2026D23.1	5	110853576	110853741	S18_peak_3025	Intergenic between ENST00000355943 and ENST00000603386	ENST00000603386
STARD4-AS1	5	111554314	111554580	S18_peak_3026	intron (ENST00000500779, intron 1 of 6)	ENST00000500779
HMGB3P16	5	112443874	112444058	S18_peak_3027	Intergenic between ENST00000513509 and ENST00000508037	ENST00000508037
MCC	5	113287317	113287558	S18_peak_3028	intron (ENST00000302475, intron 1 of 16)	ENST00000302475
YTHDC2	5	113740340	113740505	S18_peak_3029	Intergenic between ENST00000161863 and ENST00000512927	ENST00000161863
BMPR1A	10	86762312	86762551	S18_peak_303	intron (ENST00000372037, intron 1 of 12)	ENST00000372037
RP11-371M22.1	5	113946589	113946776	S18_peak_3030	Intergenic between ENST00000161863 and ENST00000512927	ENST00000512927
RP11-492A10.1	5	114444050	114444215	S18_peak_3031	Intergenic between ENST00000410774 and ENST00000514115	ENST00000514115
TRIM36	5	115117693	115117884	S18_peak_3032	Intergenic between ENST00000515822 and ENST00000282369	ENST00000282369
TRIM36	5	115125129	115125301	S18_peak_3033	exon (ENST00000282369, exon 10 of 10)	ENST00000282369
CTC-505O3.3	5	115707107	115707290	S18_peak_3034	Intergenic between ENST00000333482 and ENST00000512900	ENST00000333482
CTC-505O3.1	5	115767498	115767686	S18_peak_3035	Intergenic between ENST00000512900 and ENST00000505098	ENST00000505098
HMGN2P27	5	116216586	116216836	S18_peak_3036	Intergenic between ENST00000391155 and ENST00000427352	ENST00000427352
CTC-339F2.2	5	116326215	116326486	S18_peak_3037	Intergenic between ENST00000507813 and ENST00000343348	ENST00000507813
RP11-74D3.2	5	117614505	117614680	S18_peak_3038	Intergenic between ENST00000504107 and ENST00000506771	ENST00000506771
RN7SL174P	5	119319537	119319702	S18_peak_3039	Intergenic between ENST00000475578 and ENST00000513374	ENST00000475578
MMRN2	10	86952002	86952219	S18_peak_304	intron (ENST00000372027, intron 1 of 6)	ENST00000372027

TNFAIP8	5	119376216	119376431	S18_peak_3040	intron (ENST00000513374, intron 1 of 1)	ENST00000513374
TNFAIP8	5	119382087	119382252	S18_peak_3041	intron (ENST00000513374, intron 1 of 1)	ENST00000513374
HSD17B4	5	119474190	119474355	S18_peak_3042	intron (ENST00000256216, intron 3 of 23)	ENST00000256216
CTD-2158P22.4	5	119665531	119665804	S18_peak_3043	Intergenic between ENST00000505780 and ENST00000511500	ENST00000505780
RNU4-69P	5	120708141	120708306	S18_peak_3044	Intergenic between ENST00000604148 and ENST00000410677	ENST00000410677
CTC-546K23.2	5	121453244	121453409	S18_peak_3045	Intergenic between ENST00000623862 and ENST00000482216	ENST00000623862
RP11-510I6.3	5	121679343	121679508	S18_peak_3046	Intergenic between ENST00000509365 and ENST00000321339	ENST00000509365
SNCAIP	5	122416918	122417083	S18_peak_3047	intron (ENST00000261368, intron 3 of 10)	ENST00000261368
RP11-166A12.1	5	122580193	122580466	S18_peak_3048	Intergenic between ENST00000505546 and ENST00000511194	ENST00000511194
PRDM6	5	123213715	123213880	S18_peak_3049	Intergenic between ENST00000407847 and ENST00000513479	ENST00000407847
AGAP11	10	86996714	86996879	S18_peak_305	intron (ENST00000433214, intron 6 of 11)	ENST00000433214
CTD-2169H9.1	5	123491930	123492095	S18_peak_3050	Intergenic between ENST00000504195 and ENST00000623961	ENST00000623961
KRT18P16	5	123648298	123648487	S18_peak_3051	Intergenic between ENST00000510337 and ENST00000628324	ENST00000510337
KRT18P16	5	123719125	123719313	S18_peak_3052	Intergenic between ENST00000510337 and ENST00000628324	ENST00000510337
RP11-284A20.2	5	124862740	124862951	S18_peak_3053	Intergenic between ENST00000511091 and ENST00000510242	ENST00000510242
HMGB1P22	5	125285481	125285765	S18_peak_3054	Intergenic between ENST00000505315 and ENST00000625219	ENST00000505315
RP11-395P13.7	5	125302005	125302170	S18_peak_3055	Intergenic between ENST00000505315 and ENST00000625219	ENST00000625219
RP11-395P13.7	5	125330190	125330416	S18_peak_3056	intron (ENST00000625219, intron 1 of 1)	ENST00000625219
GRAMD3	5	126283031	126283236	S18_peak_3057	Intergenic between ENST00000507428 and ENST00000513040	ENST00000513040
GRAMD3	5	126323673	126323888	S18_peak_3058	Intergenic between ENST00000507428 and ENST00000513040	ENST00000513040
GRAMD3	5	126481818	126481987	S18_peak_3059	intron (ENST00000513040, intron 8 of 13)	ENST00000513040
AGAP11	10	86998925	86999166	S18_peak_306	intron (ENST00000433214, intron 6 of 11)	ENST00000433214
3-Mar	5	126912746	126913025	S18_peak_3060	intron (ENST00000308660, intron 3 of 4)	ENST00000308660
MRPS5P3	5	127156321	127156501	S18_peak_3061	Intergenic between ENST00000515577 and ENST00000514947	ENST00000515577
CCDC192	5	127699638	127699888	S18_peak_3062	Intergenic between ENST00000512352 and ENST00000514853	ENST00000514853
LINC01184	5	127966083	127966248	S18_peak_3063	intron (ENST00000499346, intron 2 of 3)	ENST00000499346
Y_RNA	5	128297057	128297222	S18_peak_3064	Intergenic between ENST00000512118 and ENST00000384560	ENST00000384560

Y_RNA	5	128322788	128323005	S18_peak_3065	Intergenic between ENST00000512118 and ENST00000384560	ENST00000384560
FBN2	5	128527260	128527513	S18_peak_3066	intron (ENST00000502468, intron 4 of 7)	ENST00000502468
CTC-573M9.1	5	128769955	128770261	S18_peak_3067	Intergenic between ENST00000514867 and ENST00000262462	ENST00000514867
SLC27A6	5	128877706	128877871	S18_peak_3068	Intergenic between ENST00000514867 and ENST00000262462	ENST00000262462
SLC27A6	5	128911332	128911508	S18_peak_3069	Intergenic between ENST00000514867 and ENST00000262462	ENST00000262462
NPAP1P3	10	87377871	87378166	S18_peak_307	promoter-TSS (ENST00000432327)	ENST00000432327
SLC27A6	5	128936770	128936962	S18_peak_3070	Intergenic between ENST00000514867 and ENST00000262462	ENST00000262462
RP11-114H7.1	5	130814734	130814931	S18_peak_3071	Intergenic between ENST00000511094 and ENST00000498453	ENST00000498453
RP11-114H7.1	5	130880416	130880650	S18_peak_3072	Intergenic between ENST00000511094 and ENST00000498453	ENST00000498453
RAPGEF6	5	131437989	131438208	S18_peak_3073	intron (ENST00000509018, intron 24 of 27)	ENST00000509018
P4HA2	5	132205803	132205968	S18_peak_3074	intron (ENST00000360568, intron 8 of 14)	ENST00000360568
P4HA2	5	132214024	132214379	S18_peak_3075	intron (ENST00000360568, intron 4 of 14)	ENST00000360568
IL5	5	132533976	132534190	S18_peak_3076	Intergenic between ENST00000612967 and ENST00000231454	ENST00000231454
RAD50	5	132594993	132595158	S18_peak_3077	intron (ENST00000378823, intron 12 of 24)	ENST00000378823
8-Sep	5	132769183	132769438	S18_peak_3078	intron (ENST00000378719, intron 1 of 9)	ENST00000378719
AFF4	5	132916666	132916831	S18_peak_3079	intron (ENST00000265343, intron 5 of 20)	ENST00000265343
RP11-399L7.3	10	87443876	87444091	S18_peak_308	Intergenic between ENST00000417713 and ENST00000636979	ENST00000417713
ZCCHC10	5	133005600	133005861	S18_peak_3080	intron (ENST00000324170, intron 2 of 3)	ENST00000324170
FSTL4	5	133268957	133269122	S18_peak_3081	intron (ENST00000265342, intron 6 of 15)	ENST00000265342
FSTL4	5	133356673	133356948	S18_peak_3082	intron (ENST00000265342, intron 4 of 15)	ENST00000265342
FSTL4	5	133362557	133362802	S18_peak_3083	intron (ENST00000265342, intron 4 of 15)	ENST00000265342
C5orf15	5	133944503	133944921	S18_peak_3084	Intergenic between ENST00000513561 and ENST00000231512	ENST00000231512
CTB-113I20.1	5	134045828	134046241	S18_peak_3085	Intergenic between ENST00000606089 and ENST00000603910	ENST00000603910
CTB-113I20.1	5	134055083	134055248	S18_peak_3086	Intergenic between ENST00000606089 and ENST00000603910	ENST00000603910
CTB-113I20.1	5	134086065	134086230	S18_peak_3087	Intergenic between ENST00000603910 and ENST00000342854	ENST00000603910
CTB-113I20.1	5	134088142	134088307	S18_peak_3088	Intergenic between ENST00000603910 and ENST00000342854	ENST00000603910
JADE2	5	134574912	134575108	S18_peak_3089	intron (ENST00000395003, intron 10 of 10)	ENST00000395003
MINPP1	10	87513055	87513220	S18_peak_309	intron (ENST00000371994, intron 2 of 2)	ENST00000371994
SEC24A	5	134668390	134668555	S18_peak_3090	intron (ENST00000398844, intron 3 of 22)	ENST00000398844
SEC24A	5	134715082	134715247	S18_peak_3091	intron (ENST00000398844, intron 19 of 22)	ENST00000398844

CATSPER3	5	134996460	134996727	S18_peak_3092	intron (ENST00000282611, intron 3 of 7)	ENST00000282611
H2AFY	5	135357462	135357736	S18_peak_3093	intron (ENST00000511689, intron 5 of 8)	ENST00000511689
H2AFY	5	135361045	135361273	S18_peak_3094	intron (ENST00000511689, intron 4 of 8)	ENST00000511689
RP11-114H21.2	5	136501058	136501312	S18_peak_3095	intron (ENST00000511165, intron 1 of 2)	ENST00000511165
RP11-114H21.2	5	136574663	136574828	S18_peak_3096	Intergenic between ENST00000446720 and ENST00000502421	ENST00000446720
RNA5SP193	5	136957128	136957383	S18_peak_3097	Intergenic between ENST00000511236 and ENST00000410353	ENST00000410353
KLHL3	5	137482115	137482280	S18_peak_3098	Intergenic between ENST00000410353 and ENST00000508657	ENST00000508657
KLHL3	5	137517497	137517662	S18_peak_3099	Intergenic between ENST00000410353 and ENST00000508657	ENST00000508657
RP11-399E6.1	1	41264004	41264217	S18_peak_31	intron (ENST00000425554, intron 2 of 2)	ENST00000425554
RP11-57C13.6	10	87655393	87655605	S18_peak_310	intron (ENST00000438082, intron 2 of 3)	ENST00000438082
KLHL3	5	137541318	137541483	S18_peak_3100	Intergenic between ENST00000410353 and ENST00000508657	ENST00000508657
KLHL3	5	137689259	137689424	S18_peak_3101	intron (ENST00000508657, intron 5 of 14)	ENST00000508657
NPY6R	5	137796637	137796802	S18_peak_3102	Intergenic between ENST00000604680 and ENST00000510937	ENST00000510937
RP11-325L7.2	5	138046846	138047011	S18_peak_3103	Intergenic between ENST00000506911 and ENST00000410992	ENST00000506911
CDC23	5	138203575	138203754	S18_peak_3104	intron (ENST00000394886, intron 3 of 15)	ENST00000394886
CDC23	5	138230479	138230644	S18_peak_3105	Intergenic between ENST00000394886 and ENST00000274721	ENST00000394886
CDC25C	5	138281764	138281929	S18_peak_3106	Intergenic between ENST00000515536 and ENST00000323760	ENST00000323760
CDC25C	5	138306365	138306697	S18_peak_3107	intron (ENST00000323760, intron 7 of 13)	ENST00000323760
snoU13	5	138657857	138658083	S18_peak_3108	Intergenic between ENST00000459407 and ENST00000520838	ENST00000459407
AC034243.1	5	138750672	138750863	S18_peak_3109	intron (ENST00000520838, intron 1 of 1)	ENST00000520838
PTEN	10	87895435	87895695	S18_peak_311	intron (ENST00000371953, intron 2 of 8)	ENST00000371953
RNU6-572P	5	139210314	139210479	S18_peak_3110	Intergenic between ENST00000472214 and ENST00000516724	ENST00000516724
AC138517.4	5	139503875	139504123	S18_peak_3111	Intergenic between ENST00000513338 and ENST00000384293	ENST00000513338
Y_RNA	5	139552460	139552652	S18_peak_3112	Intergenic between ENST00000513338 and ENST00000384293	ENST00000384293
CTD-2062A1.2	5	139619611	139619779	S18_peak_3113	Intergenic between ENST00000619270 and ENST00000606683	ENST00000619270
ANKHD1	5	140417838	140418003	S18_peak_3114	intron (ENST00000360839, intron 1 of 33)	ENST00000360839
SRA1	5	140555305	140555524	S18_peak_3115	intron (ENST00000336283, intron 2 of 4)	ENST00000336283
CH17-140K24.2	5	141202623	141202998	S18_peak_3116	intron (ENST00000624192, intron 1 of 1)	ENST00000624192
PCDHGA1	5	141428885	141429165	S18_peak_3117	intron (ENST00000517417, intron 1 of 3)	ENST00000517417
AC005753.1	5	141833263	141833437	S18_peak_3118	Intergenic between ENST00000623837 and	ENST00000623837

					ENST00000287008	
PCDH12	5	141957216	141957404	S18_peak_3119	promoter-TSS (ENST00000231484)	ENST00000231484
PTEN	10	87902343	87902508	S18_peak_312	intron (ENST00000371953, intron 2 of 8)	ENST00000371953
AC005592.2	5	142399635	142399935	S18_peak_3120	intron (ENST00000510311, intron 3 of 3)	ENST00000510311
ARHGAP26	5	142930450	142930615	S18_peak_3121	intron (ENST00000274498, intron 10 of 22)	ENST00000274498
ARHGAP26	5	142961307	142961536	S18_peak_3122	intron (ENST00000274498, intron 11 of 22)	ENST00000274498
NR3C1	5	143310586	143310826	S18_peak_3123	intron (ENST00000231509, intron 3 of 8)	ENST00000231509
RP11-278J6.4	5	143446841	143447043	S18_peak_3124	Intergenic between ENST00000623204 and ENST00000340585	ENST00000623204
KCTD16	5	144349820	144349985	S18_peak_3125	intron (ENST00000512467, intron 3 of 3)	ENST00000512467
KCTD16	5	144468239	144468510	S18_peak_3126	intron (ENST00000512467, intron 3 of 3)	ENST00000512467
NAMPTP2	5	144893755	144893920	S18_peak_3127	Intergenic between ENST00000364553 and ENST00000505629	ENST00000505629
CTB-73N10.1	5	145522113	145522278	S18_peak_3128	Intergenic between ENST00000513657 and ENST00000509592	ENST00000513657
CTB-78O21.1	5	145643593	145643758	S18_peak_3129	Intergenic between ENST00000513657 and ENST00000509592	ENST00000509592
PTEN	10	87915595	87915851	S18_peak_313	intron (ENST00000371953, intron 2 of 8)	ENST00000371953
PRELID2	5	145809406	145809571	S18_peak_3130	intron (ENST00000334744, intron 5 of 7)	ENST00000334744
RP11-118M9.3	5	146107446	146107611	S18_peak_3131	intron (ENST00000514002, intron 2 of 2)	ENST00000514002
JAKMIP2	5	147747536	147747701	S18_peak_3132	intron (ENST00000507386, intron 1 of 20)	ENST00000507386
JAKMIP2	5	147764418	147764583	S18_peak_3133	intron (ENST00000507386, intron 1 of 20)	ENST00000507386
ADRB2	5	148868758	148868923	S18_peak_3134	Intergenic between ENST00000305988 and ENST00000606841	ENST00000305988
ARHGEF37	5	149586204	149586506	S18_peak_3135	intron (ENST00000333677, intron 1 of 12)	ENST00000333677
PDE6A	5	149866007	149866213	S18_peak_3136	intron (ENST00000255266, intron 20 of 21)	ENST00000255266
CD74	5	150439493	150439728	S18_peak_3137	Intergenic between ENST00000009530 and ENST00000519040	ENST00000009530
CTC-367J11.1	5	150466911	150467179	S18_peak_3138	Intergenic between ENST00000009530 and ENST00000519040	ENST00000519040
CTB-12O2.1	5	152292960	152293125	S18_peak_3139	Intergenic between ENST00000523605 and ENST00000255262	ENST00000523605
Y_RNA	10	88436251	88436481	S18_peak_314	Intergenic between ENST00000610667 and ENST00000364678	ENST00000364678
CTD-2311A18.1	5	155089445	155089646	S18_peak_3140	Intergenic between ENST00000435029 and ENST00000519111	ENST00000519111
ZBED8	5	160385376	160385664	S18_peak_3141	Intergenic between ENST00000393975 and ENST00000408953	ENST00000408953
GABRG2	5	162182917	162183082	S18_peak_3142	Intergenic between ENST00000361925 and ENST00000522274	ENST00000361925
CTC-425K20.1	5	165908941	165909137	S18_peak_3143	Intergenic between ENST00000524271 and ENST00000520308	ENST00000524271
CTB-63M22.1	5	166637799	166637993	S18_peak_3144	Intergenic between ENST00000518938 and ENST00000523742	ENST00000518938

CTC-264O10.2	5	167037478	167037761	S18_peak_3145	Intergenic between ENST00000523742 and ENST00000522292	ENST00000522292
TENM2	5	168065036	168065344	S18_peak_3146	intron (ENST00000518659, intron 7 of 28)	ENST00000518659
TENM2	5	168165121	168165286	S18_peak_3147	intron (ENST00000518659, intron 13 of 28)	ENST00000518659
WWC1	5	168289442	168289662	S18_peak_3148	Intergenic between ENST00000519795 and ENST00000521089	ENST00000521089
Y_RNA	5	168852219	168852456	S18_peak_3149	Intergenic between ENST00000365475 and ENST00000514811	ENST00000365475
LIPJ	10	88593420	88593585	S18_peak_315	intron (ENST00000371939, intron 4 of 10)	ENST00000371939
CTB-174D11.1	5	169084567	169084737	S18_peak_3150	Intergenic between ENST00000508470 and ENST00000384887	ENST00000508470
DOCK2	5	169960465	169960630	S18_peak_3151	intron (ENST00000256935, intron 27 of 51)	ENST00000256935
LINC01187	5	170182523	170182834	S18_peak_3152	Intergenic between ENST00000518924 and ENST00000506431	ENST00000506431
MIR3912	5	171385041	171385206	S18_peak_3153	Intergenic between ENST00000522888 and ENST00000577566	ENST00000577566
STK10	5	172108553	172108718	S18_peak_3154	intron (ENST00000176763, intron 4 of 18)	ENST00000176763
UBTD2	5	172283508	172283673	S18_peak_3155	promoter-TSS (ENST00000393792)	ENST00000393792
SH3PXD2B	5	172444280	172444445	S18_peak_3156	intron (ENST00000519643, intron 1 of 12)	ENST00000519643
RP11-536N17.1	5	172826334	172826499	S18_peak_3157	Intergenic between ENST00000518260 and ENST00000393784	ENST00000518260
CTB-32H22.1	5	173440600	173440765	S18_peak_3158	intron (ENST00000523205, intron 1 of 3)	ENST00000523205
LINC01484	5	173732483	173732770	S18_peak_3159	intron (ENST00000517733, intron 1 of 2)	ENST00000517733
ACTA2-AS1	10	88964590	88964755	S18_peak_316	Intergenic between ENST00000625277 and ENST00000355740	ENST00000625277
MSX2	5	174680594	174680761	S18_peak_3160	Intergenic between ENST00000515857 and ENST00000239243	ENST00000239243
RNU6-226P	5	175717851	175718111	S18_peak_3161	Intergenic between ENST00000363778 and ENST00000359546	ENST00000363778
RP11-826N14.1	5	176040322	176040487	S18_peak_3162	Intergenic between ENST00000606159 and ENST00000509643	ENST00000509643
CDHR2	5	176557291	176557456	S18_peak_3163	intron (ENST00000510636, intron 1 of 31)	ENST00000510636
ZNF346	5	177057658	177057883	S18_peak_3164	intron (ENST00000358149, intron 5 of 6)	ENST00000358149
FAM153A	5	177720995	177721160	S18_peak_3165	Intergenic between ENST00000509363 and ENST00000440605	ENST00000440605
HNRNPAB	5	178201252	178201417	S18_peak_3166	Intergenic between ENST00000463439 and ENST00000358344	ENST00000358344
RN7SKP70	5	178630954	178631179	S18_peak_3167	Intergenic between ENST00000516655 and ENST00000335815	ENST00000516655
RN7SKP70	5	178653677	178653886	S18_peak_3168	Intergenic between ENST00000516655 and ENST00000335815	ENST00000516655
RNU1-39P	5	178889757	178890034	S18_peak_3169	Intergenic between ENST00000383897 and ENST00000361362	ENST00000383897
RP11-341B24.3	10	89107218	89107403	S18_peak_317	Intergenic between ENST00000637619 and	ENST00000453728

					ENST00000453728	
GFPT2	5	180302218	180302493	S18_peak_3170	intron (ENST00000253778, intron 18 of 18)	ENST00000253778
RNU1-17P	5	180724613	180724778	S18_peak_3171	Intergenic between ENST00000448534 and ENST00000363022	ENST00000363022
CTC-338M12.9	5	181281998	181282163	S18_peak_3172	promoter-TSS (ENST00000412295)	ENST00000412295
DUSP22	6	300027	300271	S18_peak_3173	intron (ENST00000344450, intron 1 of 7)	ENST00000344450
DUSP22	6	371002	371167	S18_peak_3174	Intergenic between ENST00000419235 and ENST00000380956	ENST00000419235
IRF4	6	381818	382084	S18_peak_3175	Intergenic between ENST00000419235 and ENST00000380956	ENST00000380956
EXOC2	6	675328	675542	S18_peak_3176	intron (ENST00000230449, intron 1 of 27)	ENST00000230449
RP5-856G1.2	6	1161406	1161637	S18_peak_3177	Intergenic between ENST00000314040 and ENST00000296839	ENST00000314040
RP11-157J24.2	6	1522168	1522333	S18_peak_3178	Intergenic between ENST00000404600 and ENST00000607350	ENST00000607350
GMDS	6	1700485	1700650	S18_peak_3179	intron (ENST00000380815, intron 9 of 10)	ENST00000380815
SLC16A12	10	89468614	89468822	S18_peak_318	intron (ENST00000371790, intron 2 of 7)	ENST00000371790
GMDS	6	1885744	1885950	S18_peak_3180	intron (ENST00000380815, intron 7 of 10)	ENST00000380815
GMDS	6	2140396	2140561	S18_peak_3181	intron (ENST00000380815, intron 1 of 10)	ENST00000380815
GMDS-AS1	6	2257881	2258046	S18_peak_3182	intron (ENST00000530346, intron 2 of 3)	ENST00000530346
RP1-90J20.2	6	2914961	2915266	S18_peak_3183	Intergenic between ENST00000454998 and ENST00000437718	ENST00000437718
BPHL	6	3108660	3108825	S18_peak_3184	Intergenic between ENST00000410316 and ENST00000380379	ENST00000380379
TUBB2B	6	3225026	3225283	S18_peak_3185	exon (ENST00000259818, exon 4 of 4)	ENST00000259818
RP1-223B1.1	6	3583974	3584154	S18_peak_3186	Intergenic between ENST00000607565 and ENST00000443445	ENST00000443445
FAM50B	6	3861047	3861240	S18_peak_3187	Intergenic between ENST00000380272 and ENST00000450255	ENST00000380272
RP3-400B16.2	6	4217539	4217727	S18_peak_3188	Intergenic between ENST00000403917 and ENST00000437430	ENST00000403917
RP4-529N6.2	6	4590329	4590623	S18_peak_3189	Intergenic between ENST00000603277 and ENST00000568110	ENST00000568110
SLC16A12	10	89520723	89520888	S18_peak_319	intron (ENST00000371790, intron 2 of 7)	ENST00000371790
RP3-430A16.1	6	4745791	4745956	S18_peak_3190	Intergenic between ENST00000405257 and ENST00000436283	ENST00000436283
RP11-530A18.1	6	5063141	5063306	S18_peak_3191	Intergenic between ENST00000606227 and ENST00000405549	ENST00000405549
LYRM4	6	5243008	5243173	S18_peak_3192	intron (ENST00000330636, intron 1 of 2)	ENST00000330636
FARS2	6	5450710	5450875	S18_peak_3193	intron (ENST00000274680, intron 4 of 6)	ENST00000274680
FARS2	6	5509113	5509278	S18_peak_3194	intron (ENST00000274680, intron 4 of 6)	ENST00000274680
FARS2	6	5581716	5581951	S18_peak_3195	intron (ENST00000274680, intron 5 of 6)	ENST00000274680
FARS2	6	5631011	5631213	S18_peak_3196	intron (ENST00000274680, intron 6 of 6)	ENST00000274680
RP1-182O16.2	6	5812248	5812413	S18_peak_3197	Intergenic between ENST00000407959 and	ENST00000403053

					ENST00000403053	
F13A1	6	6202440	6202616	S18_peak_3198	intron (ENST00000264870, intron 8 of 14)	ENST00000264870
LY86-AS1	6	6608151	6608316	S18_peak_3199	intron (ENST00000429345, intron 1 of 7)	ENST00000429345
HIVEP3	1	41531377	41531542	S18_peak_32	intron (ENST00000372584, intron 4 of 7)	ENST00000372584
LINC00865	10	89836428	89836622	S18_peak_320	intron (ENST00000448963, intron 3 of 3)	ENST00000448963
LY86	6	6641859	6642024	S18_peak_3200	intron (ENST00000230568, intron 3 of 4)	ENST00000230568
LY86	6	6653528	6653693	S18_peak_3201	intron (ENST00000230568, intron 4 of 4)	ENST00000230568
RP3-429O6.1	6	6878812	6878977	S18_peak_3202	Intergenic between ENST00000407097 and ENST00000422310	ENST00000422310
RN7SL554P	6	6946570	6946735	S18_peak_3203	Intergenic between ENST00000468197 and ENST00000423568	ENST00000468197
RP11-556O15.1	6	6980025	6980190	S18_peak_3204	Intergenic between ENST00000468197 and ENST00000423568	ENST00000423568
DSP	6	7573340	7573576	S18_peak_3205	intron (ENST00000379802, intron 15 of 23)	ENST00000379802
SNRNP48	6	7605092	7605350	S18_peak_3206	intron (ENST00000342415, intron 6 of 8)	ENST00000342415
BLOC1S5-TXNDC5	6	7920133	7920298	S18_peak_3207	intron (ENST00000439343, intron 4 of 12)	ENST00000439343
RP11-203H2.1	6	8250178	8250405	S18_peak_3208	Intergenic between ENST00000379715 and ENST00000439891	ENST00000439891
RP11-314C16.1	6	8756445	8756610	S18_peak_3209	Intergenic between ENST00000612720 and ENST00000429060	ENST00000429060
SNRPD2P1	10	90020485	90020669	S18_peak_321	Intergenic between ENST00000489253 and ENST00000410519	ENST00000489253
RP11-354I10.1	6	8965291	8965479	S18_peak_3210	Intergenic between ENST00000429060 and ENST00000431369	ENST00000431369
RP11-354I10.1	6	9092859	9093195	S18_peak_3211	Intergenic between ENST00000429060 and ENST00000431369	ENST00000431369
RP11-354I10.1	6	9138311	9138568	S18_peak_3212	intron (ENST00000431369, intron 2 of 2)	ENST00000431369
RP11-354I10.1	6	9218028	9218193	S18_peak_3213	Intergenic between ENST00000431369 and ENST00000401715	ENST00000431369
RP11-354I10.1	6	9243324	9243494	S18_peak_3214	Intergenic between ENST00000431369 and ENST00000401715	ENST00000431369
RP11-354I10.1	6	9355003	9355294	S18_peak_3215	Intergenic between ENST00000431369 and ENST00000401715	ENST00000431369
GCNT2	6	10611376	10611585	S18_peak_3216	intron (ENST00000495262, intron 3 of 4)	ENST00000495262
GCNT2	6	10615814	10616023	S18_peak_3217	intron (ENST00000495262, intron 3 of 4)	ENST00000495262
GCNT6	6	10637618	10637790	S18_peak_3218	Intergenic between ENST00000379591 and ENST00000364178	ENST00000379591
NEDD9	6	11351276	11351623	S18_peak_3219	intron (ENST00000504387, intron 1 of 7)	ENST00000504387
RN7SKP143	10	90235088	90235253	S18_peak_322	Intergenic between ENST00000410519 and ENST00000418379	ENST00000410519
NEDD9	6	11380251	11380505	S18_peak_3220	promoter-TSS (ENST00000504387)	ENST00000504387
RP3-413H6.2	6	11831055	11831295	S18_peak_3221	Intergenic between ENST00000412132 and ENST00000299540	ENST00000412132

AMD1P4	6	11847252	11847496	S18_peak_3222	Intergenic between ENST00000412132 and ENST00000299540	ENST00000299540
EDN1	6	12247678	12247934	S18_peak_3223	Intergenic between ENST00000607445 and ENST00000379375	ENST00000379375
RN7SKP293	6	12425558	12425723	S18_peak_3224	Intergenic between ENST00000411389 and ENST00000391491	ENST00000411389
RP11-125M16.1	6	12556194	12556359	S18_peak_3225	Intergenic between ENST00000391491 and ENST00000457945	ENST00000457945
PHACTR1	6	12669550	12669715	S18_peak_3226	Intergenic between ENST00000457945 and ENST00000332995	ENST00000332995
PHACTR1	6	12723114	12723339	S18_peak_3227	intron (ENST00000332995, intron 3 of 14)	ENST00000332995
PHACTR1	6	12855244	12855414	S18_peak_3228	intron (ENST00000332995, intron 4 of 14)	ENST00000332995
PHACTR1	6	12866898	12867223	S18_peak_3229	intron (ENST00000332995, intron 4 of 14)	ENST00000332995
RPP30	10	90887723	90887888	S18_peak_323	intron (ENST00000371703, intron 6 of 10)	ENST00000371703
RNF182	6	13977713	13977923	S18_peak_3230	exon (ENST00000488300, exon 3 of 3)	ENST00000488300
CD83	6	14095906	14096071	S18_peak_3231	Intergenic between ENST00000427011 and ENST00000379153	ENST00000379153
RP11-359N11.1	6	14255116	14255513	S18_peak_3232	Intergenic between ENST00000455973 and ENST00000427276	ENST00000455973
LINC01108	6	14267612	14267805	S18_peak_3233	Intergenic between ENST00000455973 and ENST00000427276	ENST00000427276
RP3-448I9.2	6	14376306	14376471	S18_peak_3234	Intergenic between ENST00000635227 and ENST00000456440	ENST00000456440
RP3-448I9.1	6	14404013	14404347	S18_peak_3235	intron (ENST00000434947, intron 2 of 2)	ENST00000434947
RP11-330A16.1	6	14575625	14575881	S18_peak_3236	Intergenic between ENST00000434947 and ENST00000414740	ENST00000414740
RP11-146I2.1	6	15035322	15035487	S18_peak_3237	intron (ENST00000437648, intron 2 of 3)	ENST00000437648
JARID2	6	15378299	15378464	S18_peak_3238	intron (ENST00000341776, intron 2 of 17)	ENST00000341776
GMPR	6	16277048	16277247	S18_peak_3239	intron (ENST00000259727, intron 5 of 8)	ENST00000259727
LINC00502	10	91024064	91024279	S18_peak_324	Intergenic between ENST00000607979 and ENST00000423621	ENST00000423621
ATXN1	6	16462525	16462731	S18_peak_3240	intron (ENST00000244769, intron 7 of 8)	ENST00000244769
ATXN1	6	16633434	16633691	S18_peak_3241	intron (ENST00000244769, intron 4 of 8)	ENST00000244769
RBM24	6	17251623	17251788	S18_peak_3242	Intergenic between ENST00000606924 and ENST00000379052	ENST00000379052

RP1-273P12.1	6	17348984	17349251	S18_peak_3243	Intergenic between ENST00000425446 and ENST00000407586	ENST0000040758 6
CAP2	6	17511379	17511547	S18_peak_3244	intron (ENST00000229922, intron 6 of 12)	ENST0000022992 2
NUP153	6	17622307	17622481	S18_peak_3245	intron (ENST00000262077, intron 20 of 21)	ENST0000026207 7
KDM1B	6	18162970	18163154	S18_peak_3246	intron (ENST00000546309, intron 1 of 3)	ENST0000054630 9
RNF144B	6	18485724	18485894	S18_peak_3247	Intergenic between ENST00000259939 and ENST00000636739	ENST0000025993 9
RP1-242N11.1	6	18577863	18578184	S18_peak_3248	intron (ENST00000637804, intron 3 of 5)	ENST0000063780 4
RP11-254A17.1	6	19028083	19028284	S18_peak_3249	Intergenic between ENST00000385041 and ENST00000402485	ENST0000040248 5
BTAF1	10	91987775	91987990	S18_peak_325	intron (ENST00000265990, intron 19 of 37)	ENST0000026599 0
RP11-254A17.1	6	19183024	19183189	S18_peak_3250	Intergenic between ENST00000402485 and ENST00000443504	ENST0000040248 5
RP11-686D16.1	6	19276395	19276760	S18_peak_3251	Intergenic between ENST00000402485 and ENST00000443504	ENST0000044350 4
RP4-625H18.2	6	19529276	19529457	S18_peak_3252	intron (ENST00000636202, intron 7 of 9)	ENST0000063620 2
ID4	6	19908328	19908493	S18_peak_3253	Intergenic between ENST00000378700 and ENST00000446953	ENST0000037870 0
RP1-130G2.1	6	19988841	19989035	S18_peak_3254	Intergenic between ENST00000378700 and ENST00000446953	ENST0000044695 3
MBOAT1	6	20139891	20140061	S18_peak_3255	intron (ENST00000324607, intron 4 of 12)	ENST0000032460 7
MBOAT1	6	20177051	20177217	S18_peak_3256	intron (ENST00000324607, intron 1 of 12)	ENST0000032460 7
RNU6-150P	6	20967804	20967994	S18_peak_3257	Intergenic between ENST00000421167 and ENST00000516237	ENST0000051623 7
LINC00581	6	21491615	21491885	S18_peak_3258	intron (ENST00000630914, intron 2 of 3)	ENST0000063091 4
RP11-204E9.3	6	21561405	21561842	S18_peak_3259	intron (ENST00000637901, intron 1 of 2)	ENST0000063790 1
LGI1	10	93755599	93755764	S18_peak_326	Intergenic between ENST00000359204 and ENST00000371418	ENST0000037141 8
BOLA2P3	6	21605370	21605581	S18_peak_3260	Intergenic between ENST00000404566 and ENST00000606336	ENST0000040456 6
CASC15	6	21709906	21710071	S18_peak_3261	intron (ENST00000606336, intron 3 of 6)	ENST0000060633 6
CASC15	6	22140891	22141088	S18_peak_3262	intron (ENST00000606851, intron 9 of 11)	ENST0000060685 1
RP1-309H15.2	6	22624261	22624426	S18_peak_3263	Intergenic between ENST00000427775 and ENST00000420572	ENST0000042777 5
RP11-108N13.1	6	23097817	23098034	S18_peak_3264	Intergenic between	ENST0000040401

					ENST00000420572 and ENST00000404015	5
RNU6-1060P	6	23224594	23224964	S18_peak_3265	Intergenic between ENST00000384664 and ENST00000431001	ENST0000038466 4
RP11-439H9.1	6	23283177	23283444	S18_peak_3266	Intergenic between ENST00000384664 and ENST00000431001	ENST0000043100 1
RP4-810F7.1	6	23607375	23607540	S18_peak_3267	Intergenic between ENST00000431001 and ENST00000402635	ENST0000040263 5
RP4-810F7.1	6	23654864	23655029	S18_peak_3268	Intergenic between ENST00000402635 and ENST00000622029	ENST0000040263 5
SNORD46	6	24159085	24159250	S18_peak_3269	Intergenic between ENST00000378491 and ENST00000516021	ENST0000051602 1
RP11-437J2.4	10	93804232	93804402	S18_peak_327	Intergenic between ENST00000627075 and ENST00000442978	ENST0000062707 5
DCDC2	6	24286529	24286724	S18_peak_3270	intron (ENST00000378454, intron 6 of 9)	ENST0000037845 4
ALDH5A1	6	24493414	24493679	S18_peak_3271	Intergenic between ENST00000230036 and ENST00000357578	ENST0000035757 8
ALDH5A1	6	24532857	24533076	S18_peak_3272	intron (ENST00000357578, intron 9 of 9)	ENST0000035757 8
RP11-191A15.1	6	25158037	25158213	S18_peak_3273	Intergenic between ENST00000573276 and ENST00000407885	ENST0000057327 6
LRRC16A	6	25449089	25449345	S18_peak_3274	intron (ENST00000329474, intron 5 of 36)	ENST0000032947 4
LRRC16A	6	25607764	25607955	S18_peak_3275	intron (ENST00000329474, intron 35 of 36)	ENST0000032947 4
SCGN	6	25701073	25701316	S18_peak_3276	intron (ENST00000377961, intron 10 of 10)	ENST0000037796 1
SLC17A1	6	25787736	25787915	S18_peak_3277	intron (ENST00000244527, intron 12 of 12)	ENST0000024452 7
HIST1H2BC	6	26130406	26130663	S18_peak_3278	Intergenic between ENST00000396984 and ENST00000304218	ENST0000039698 4
BTN2A3P	6	26432881	26433049	S18_peak_3279	Intergenic between ENST00000463944 and ENST00000244519	ENST0000046394 4
HELLS	10	94562086	94562270	S18_peak_328	intron (ENST00000348459, intron 4 of 21)	ENST0000034845 9
ABT1	6	26581345	26581510	S18_peak_3280	Intergenic between ENST00000377575 and ENST00000274849	ENST0000027484 9
RP11-457M11.5	6	26730061	26730226	S18_peak_3281	Intergenic between ENST00000562904 and ENST00000479900	ENST0000056290 4
GUSBP2	6	26791972	26792234	S18_peak_3282	Intergenic between ENST00000562904 and ENST00000479900	ENST0000047990 0
LINC00240	6	26988052	26988217	S18_peak_3283	intron (ENST00000606878, intron 2 of 2)	ENST0000060687 8
VN1R12P	6	27040081	27040246	S18_peak_3284	Intergenic between	ENST0000040377

					ENST00000403778 and ENST00000406212	8
VN1R10P	6	27337145	27337340	S18_peak_3285	Intergenic between ENST00000447106 and ENST00000416749	ENST0000044710 6
ZNF204P	6	27366519	27366684	S18_peak_3286	Intergenic between ENST00000416749 and ENST00000244576	ENST0000041674 9
MCFD2P1	6	27447353	27447562	S18_peak_3287	Intergenic between ENST00000417812 and ENST00000613415	ENST0000041781 2
XXbac-BPG34I8.3	6	27577420	27577585	S18_peak_3288	Intergenic between ENST00000405625 and ENST00000383944	ENST0000040562 5
ZKSCAN3	6	28344527	28344703	S18_peak_3289	Intergenic between ENST00000446474 and ENST00000341464	ENST0000034146 4
CYP2C59P	10	94990242	94990407	S18_peak_329	Intergenic between ENST00000424125 and ENST00000457790	ENST0000045779 0
LINC01623	6	28876091	28876320	S18_peak_3290	Intergenic between ENST00000606624 and ENST00000419323	ENST0000060662 4
XXbac-BPG13B8.10	6	29510743	29510983	S18_peak_3291	TTS (ENST00000436804)	ENST0000043680 4
HCG4P11	6	29721547	29721866	S18_peak_3292	Intergenic between ENST00000435787 and ENST00000427340	ENST0000042734 0
HCP5B	6	29868973	29869138	S18_peak_3293	Intergenic between ENST00000432679 and ENST00000630472	ENST0000063047 2
TRIM26	6	30180654	30180819	S18_peak_3294	Intergenic between ENST00000376694 and ENST00000453195	ENST0000045319 5
HCG17	6	30283715	30283895	S18_peak_3295	intron (ENST00000453558, intron 1 of 4)	ENST0000045355 8
PPP1R10	6	30603112	30603328	S18_peak_3296	exon (ENST00000376511, exon 17 of 20)	ENST0000037651 1
DDR1-AS1	6	30877005	30877170	S18_peak_3297	Intergenic between ENST00000458361 and ENST00000508312	ENST0000045836 1
MUC22	6	31005952	31006208	S18_peak_3298	Intergenic between ENST00000376296 and ENST00000561890	ENST0000056189 0
RNU6-1133P	6	31078930	31079095	S18_peak_3299	Intergenic between ENST00000561890 and ENST00000410963	ENST0000041096 3
ERMAP	1	42826658	42826823	S18_peak_33	intron (ENST00000372517, intron 2 of 11)	ENST0000037251 7
SORBS1	10	95345281	95345448	S18_peak_330	intron (ENST00000371249, intron 19 of 24)	ENST0000037124 9
RNU6-1133P	6	31079533	31079698	S18_peak_3300	Intergenic between ENST00000561890 and ENST00000410963	ENST0000041096 3
USP8P1	6	31277618	31277785	S18_peak_3301	exon (ENST00000494673, exon 1 of 1)	ENST0000049467 3
LINC01149	6	31440518	31440778	S18_peak_3302	Intergenic between ENST00000449934 and	ENST0000043036 4

					ENST00000430364	
XXbac-BPG181B23.6	6	31466005	31466223	S18_peak_3303	Intergenic between ENST00000440087 and ENST00000383850	ENST0000044008 7
MCCD1	6	31528910	31529083	S18_peak_3304	promoter-TSS (ENST00000376191)	ENST0000037619 1
LTB	6	31579836	31580001	S18_peak_3305	TTS (ENST00000446745)	ENST0000044674 5
SNORD52	6	31842476	31842769	S18_peak_3306	Intergenic between ENST00000364884 and ENST00000375631	ENST0000036488 4
C4A	6	31982481	31982685	S18_peak_3307	promoter-TSS (ENST00000428956)	ENST0000042895 6
C4A	6	31986911	31987078	S18_peak_3308	intron (ENST00000428956, intron 9 of 40)	ENST0000042895 6
ATF6B	6	32098584	32098855	S18_peak_3309	Intergenic between ENST00000516703 and ENST00000375203	ENST0000037520 3
DNTT	10	96315895	96316060	S18_peak_331	intron (ENST00000371174, intron 1 of 10)	ENST0000037117 4
PRRT1	6	32141000	32141165	S18_peak_3310	Intergenic between ENST00000375156 and ENST00000211413	ENST0000021141 3
NOTCH4	6	32215527	32215769	S18_peak_3311	intron (ENST00000375023, intron 11 of 29)	ENST0000037502 3
NOTCH4	6	32234997	32235234	S18_peak_3312	Intergenic between ENST00000375023 and ENST00000611838	ENST0000037502 3
HLA-DOB	6	32797297	32797515	S18_peak_3313	Intergenic between ENST00000437316 and ENST00000438763	ENST0000043876 3
COL11A2P1	6	33103796	33104073	S18_peak_3314	intron (ENST00000441798, intron 5 of 5)	ENST0000044179 8
B3GALT4	6	33277046	33277233	S18_peak_3315	promoter-TSS (ENST00000451237)	ENST0000045123 7
MYL8P	6	33338090	33338255	S18_peak_3316	Intergenic between ENST00000374542 and ENST00000431574	ENST0000043157 4
ZBTB9	6	33474602	33474842	S18_peak_3317	Intergenic between ENST00000395064 and ENST00000612409	ENST0000039506 4
UQCC2	6	33693632	33693797	S18_peak_3318	Intergenic between ENST00000477984 and ENST00000607484	ENST0000060748 4
MLN	6	33819576	33819743	S18_peak_3319	Intergenic between ENST00000430124 and ENST00000525912	ENST0000043012 4
TLL2	10	96426262	96426427	S18_peak_332	intron (ENST00000357947, intron 5 of 20)	ENST0000035794 7
LINC01016	6	33835983	33836232	S18_peak_3320	Intergenic between ENST00000430124 and ENST00000525912	ENST0000052591 2
ANKS1A	6	35055389	35055554	S18_peak_3321	intron (ENST00000360359, intron 12 of 23)	ENST0000036035 9
PPARD	6	35431923	35432105	S18_peak_3322	Intergenic between ENST00000311565 and ENST00000436521	ENST0000031156 5

SRPK1	6	35876897	35877062	S18_peak_3323	intron (ENST00000373825, intron 6 of 15)	ENST0000037382 5
SLC26A8	6	36010513	36010741	S18_peak_3324	intron (ENST00000490799, intron 3 of 19)	ENST0000049079 9
RP3-441A12.1	6	37780947	37781199	S18_peak_3325	Intergenic between ENST00000434837 and ENST00000415890	ENST0000041589 0
ADCY10P1	6	41117054	41117219	S18_peak_3326	intron (ENST00000457653, intron 7 of 22)	ENST0000045765 3
C6orf132	6	42103307	42103472	S18_peak_3327	exon (ENST00000341865, exon 5 of 5)	ENST0000034186 5
RP11-256G5.1	6	42455207	42455433	S18_peak_3328	Intergenic between ENST00000053468 and ENST00000464297	ENST0000046429 7
CLIC5	6	45854557	45854738	S18_peak_3329	Intergenic between ENST00000563807 and ENST00000185206	ENST0000018520 6
MIR607	10	96827397	96827562	S18_peak_333	Intergenic between ENST00000439189 and ENST00000385241	ENST0000038524 1
RNU6-464P	6	53175202	53175406	S18_peak_3330	Intergenic between ENST00000517217 and ENST00000407803	ENST0000051721 7
NANOGP3	6	53434442	53434607	S18_peak_3331	Intergenic between ENST00000605855 and ENST00000229416	ENST0000060585 5
KRASP1	6	54746762	54746927	S18_peak_3332	Intergenic between ENST00000406712 and ENST00000407852	ENST0000040785 2
COL21A1	6	56155617	56155916	S18_peak_3333	intron (ENST00000244728, intron 10 of 29)	ENST0000024472 8
RP11-472M19.2	6	56791616	56791781	S18_peak_3334	Intergenic between ENST00000370765 and ENST00000426453	ENST0000042645 3
PRIM2	6	57402068	57402375	S18_peak_3335	intron (ENST00000615550, intron 7 of 13)	ENST0000061555 0
PRIM2	6	57572930	57573095	S18_peak_3336	intron (ENST00000615550, intron 10 of 13)	ENST0000061555 0
GUSBP4	6	57926395	57926560	S18_peak_3337	exon (ENST00000423899, exon 3 of 5)	ENST0000042389 9
RP11-136G2.1	6	58970693	58970863	S18_peak_3338	Intergenic between ENST00000619504 and ENST00000637553	ENST0000061950 4
RP3-401D24.1	6	60414055	60414220	S18_peak_3339	intron (ENST00000637553, intron 3 of 8)	ENST0000063755 3
ARHGAP19-SLIT1	10	97189663	97189870	S18_peak_334	intron (ENST00000479633, intron 11 of 14)	ENST0000047963 3
RP3-401D24.1	6	60603224	60603389	S18_peak_3340	Intergenic between ENST00000637553 and ENST00000613707	ENST0000063755 3
RP11-325M4.2	6	60841470	60841651	S18_peak_3341	Intergenic between ENST00000618593 and ENST00000619705	ENST0000061859 3
RP1-91N13.1	6	61251631	61251796	S18_peak_3342	Intergenic between ENST00000405826 and ENST00000622562	ENST0000040582 6
AL356135.1	6	61592847	61593039	S18_peak_3343	Intergenic between ENST00000624857 and	ENST0000062485 7

					ENST00000511849	
KHDRBS2	6	62035792	62035978	S18_peak_3344	intron (ENST00000281156, intron 3 of 8)	ENST0000028115 6
RP11-448N11.3	6	62898356	62898521	S18_peak_3345	Intergenic between ENST00000574825 and ENST00000403497	ENST0000057482 5
RP3-525N10.2	6	68868738	68868903	S18_peak_3346	Intergenic between ENST00000419979 and ENST00000370577	ENST0000041997 9
RP3-525N10.2	6	68883388	68883553	S18_peak_3347	Intergenic between ENST00000419979 and ENST00000370577	ENST0000041997 9
LMBRD1	6	69469437	69469646	S18_peak_3348	Intergenic between ENST00000419979 and ENST00000370577	ENST0000037057 7
LMBRD1	6	69687094	69687259	S18_peak_3349	intron (ENST00000370577, intron 14 of 15)	ENST0000037057 7
ARHGAP19-SLIT1	10	97211109	97211274	S18_peak_335	intron (ENST00000479633, intron 11 of 14)	ENST0000047963 3
COL19A1	6	69860855	69861020	S18_peak_3350	Intergenic between ENST00000406848 and ENST00000620364	ENST0000062036 4
SMAP1	6	70861701	70861911	S18_peak_3351	exon (ENST00000370455, exon 11 of 11)	ENST0000037045 5
RIMS1	6	72079625	72079790	S18_peak_3352	intron (ENST00000521978, intron 2 of 33)	ENST0000052197 8
RIMS1	6	72255701	72255896	S18_peak_3353	intron (ENST00000521978, intron 16 of 33)	ENST0000052197 8
PAICSP3	6	73328316	73328557	S18_peak_3354	promoter-TSS (ENST00000428007)	ENST0000042800 7
MB21D1	6	73427960	73428125	S18_peak_3355	intron (ENST00000370315, intron 4 of 4)	ENST0000037031 5
MB21D1	6	73436804	73437011	S18_peak_3356	intron (ENST00000370315, intron 3 of 4)	ENST0000037031 5
MTO1	6	73477185	73477350	S18_peak_3357	intron (ENST00000415954, intron 4 of 12)	ENST0000041595 4
MTO1	6	73482090	73482279	S18_peak_3358	exon (ENST00000415954, exon 9 of 13)	ENST0000041595 4
RP11-554D15.1	6	74685523	74685688	S18_peak_3359	intron (ENST00000435946, intron 3 of 3)	ENST0000043594 6
UBTD1	10	97574257	97574422	S18_peak_336	Intergenic between ENST00000370664 and ENST00000370647	ENST0000037066 4
RP11-560O20.1	6	74899028	74899212	S18_peak_3360	Intergenic between ENST00000406203 and ENST00000322507	ENST0000040620 3
FILIP1	6	75476106	75476271	S18_peak_3361	intron (ENST00000237172, intron 1 of 5)	ENST0000023717 2
RP11-354K4.2	6	76719929	76720094	S18_peak_3362	Intergenic between ENST00000440811 and ENST00000607287	ENST0000060728 7
MEI4	6	78241826	78242068	S18_peak_3363	Intergenic between ENST00000602452 and ENST00000449463	ENST0000060245 2
IRAK1BP1	6	78905465	78905630	S18_peak_3364	Intergenic between ENST00000369940 and ENST00000275034	ENST0000036994 0

LCA5	6	79540226	79540391	S18_peak_3365	Intergenic between ENST00000369846 and ENST00000571144	ENST0000036984 6
RP11-486E2.1	6	80801746	80802095	S18_peak_3366	Intergenic between ENST00000403828 and ENST00000435858	ENST0000040382 8
RP11-379B8.1	6	81893676	81893841	S18_peak_3367	intron (ENST00000418567, intron 2 of 2)	ENST0000041856 7
RP11-801I18.1	6	82050687	82050906	S18_peak_3368	Intergenic between ENST00000565962 and ENST00000306270	ENST0000056596 2
IBTK	6	82175788	82176001	S18_peak_3369	intron (ENST00000306270, intron 26 of 28)	ENST0000030627 0
RP11-548K23.11	10	97638550	97638739	S18_peak_337	intron (ENST00000370649, intron 2 of 9)	ENST0000037064 9
RP3-492P14.2	6	82379334	82379499	S18_peak_3370	Intergenic between ENST00000401636 and ENST00000369747	ENST0000040163 6
UBE3D	6	82718992	82719244	S18_peak_3371	Intergenic between ENST00000401636 and ENST00000369747	ENST0000036974 7
UBE3D	6	83012921	83013172	S18_peak_3372	intron (ENST00000369747, intron 8 of 9)	ENST0000036974 7
DOPEY1	6	83115560	83115766	S18_peak_3373	intron (ENST00000349129, intron 7 of 38)	ENST0000034912 9
ME1	6	83204678	83204843	S18_peak_3374	Intergenic between ENST00000369724 and ENST00000369705	ENST0000036970 5
MRAP2	6	84090433	84090598	S18_peak_3375	exon (ENST00000257776, exon 4 of 4)	ENST0000025777 6
CEP162	6	84248493	84248658	S18_peak_3376	Intergenic between ENST00000403245 and ENST00000454981	ENST0000040324 5
LINC01611	6	84402676	84402841	S18_peak_3377	Intergenic between ENST00000403245 and ENST00000454981	ENST0000045498 1
TBX18-AS1	6	84573483	84573690	S18_peak_3378	Intergenic between ENST00000442896 and ENST00000626169	ENST0000062616 9
RP3-435K13.1	6	84930418	84930583	S18_peak_3379	Intergenic between ENST00000369663 and ENST00000401925	ENST0000040192 5
MARVELD1	10	97707828	97707993	S18_peak_338	Intergenic between ENST00000370626 and ENST00000285605	ENST0000028560 5
RP3-455E7.1	6	85093602	85093767	S18_peak_3380	Intergenic between ENST00000401925 and ENST00000403057	ENST0000040305 7
RP3-455E7.1	6	85189763	85189929	S18_peak_3381	Intergenic between ENST00000403057 and ENST00000403365	ENST0000040305 7
RP1-161C16.1	6	85236190	85236409	S18_peak_3382	Intergenic between ENST00000403057 and ENST00000403365	ENST0000040336 5
SNX14	6	85540165	85540363	S18_peak_3383	intron (ENST00000346348, intron 13 of 25)	ENST0000034634 8
RPL7P27	6	86104873	86105066	S18_peak_3384	Intergenic between ENST00000403775 and	ENST0000040377 5

					ENST00000401856	
RPL7P27	6	86119378	86119543	S18_peak_3385	Intergenic between ENST00000403775 and ENST00000401856	ENST0000040377 5
ZNF292	6	87193384	87193735	S18_peak_3386	intron (ENST00000369577, intron 1 of 7)	ENST0000036957 7
GJB7	6	87317366	87317532	S18_peak_3387	intron (ENST00000525899, intron 2 of 2)	ENST0000052589 9
RP11-453D21.1	6	87910213	87910555	S18_peak_3388	Intergenic between ENST00000604960 and ENST00000237201	ENST0000060496 0
RNGTT	6	88822194	88822416	S18_peak_3389	intron (ENST00000369485, intron 11 of 15)	ENST0000036948 5
DNMBP	10	99954325	99954490	S18_peak_339	intron (ENST00000324109, intron 4 of 16)	ENST0000032410 9
ANKRD6	6	89549667	89549922	S18_peak_3390	intron (ENST00000369408, intron 1 of 14)	ENST0000036940 8
RP1-45N11.1	6	90345186	90345372	S18_peak_3391	promoter-TSS (ENST00000569865)	ENST0000056986 5
RP1-45N11.1	6	90394201	90394428	S18_peak_3392	Intergenic between ENST00000569865 and ENST00000369332	ENST0000056986 5
MAP3K7	6	90464748	90465059	S18_peak_3393	Intergenic between ENST00000569865 and ENST00000369332	ENST0000036933 2
MAP3K7	6	90476839	90477004	S18_peak_3394	Intergenic between ENST00000569865 and ENST00000369332	ENST0000036933 2
RP1-177I10.1	6	90995493	90995831	S18_peak_3395	Intergenic between ENST00000369325 and ENST00000434493	ENST0000043449 3
RP1-149C7.1	6	92466709	92466904	S18_peak_3396	Intergenic between ENST00000564251 and ENST00000404689	ENST0000056425 1
EPHA7	6	93454681	93454932	S18_peak_3397	Intergenic between ENST00000369303 and ENST00000624663	ENST0000036930 3
RP11-64I5.1	6	93715967	93716243	S18_peak_3398	Intergenic between ENST00000369303 and ENST00000624663	ENST0000062466 3
CYCSP17	6	95361828	95361993	S18_peak_3399	Intergenic between ENST00000404146 and ENST00000407870	ENST0000040787 0
FAM183A	1	43160984	43161149	S18_peak_34	Intergenic between ENST00000335282 and ENST00000362370	ENST0000033528 2
PDCD11	10	103415083	10341534 9	S18_peak_340	intron (ENST00000369797, intron 12 of 35)	ENST0000036979 7
RP11-374I15.1	6	95636670	95636835	S18_peak_3400	Intergenic between ENST00000401718 and ENST00000404337	ENST0000040171 8
GPR63	6	96801082	96801247	S18_peak_3401	intron (ENST00000229955, intron 1 of 1)	ENST0000022995 5
GPR63	6	96842980	96843145	S18_peak_3402	Intergenic between ENST00000229955 and ENST00000316149	ENST0000022995 5
KLHL32	6	97019736	97019901	S18_peak_3403	intron (ENST00000369261, intron 3 of 10)	ENST0000036926 1

MMS22L	6	97198355	97198574	S18_peak_3404	intron (ENST00000275053, intron 14 of 24)	ENST0000027505 3
FBXL4	6	98867143	98867365	S18_peak_3405	Intergenic between ENST00000328345 and ENST00000369244	ENST0000036924 4
MCHR2-AS1	6	99964480	99964645	S18_peak_3406	Intergenic between ENST00000362433 and ENST00000443234	ENST0000044323 4
RP1-121G13.2	6	100436811	10043702 7	S18_peak_3407	intron (ENST00000411442, intron 1 of 1)	ENST0000041144 2
ACTG1P18	6	101163722	10116392 4	S18_peak_3408	Intergenic between ENST00000604292 and ENST00000401561	ENST0000040156 1
RP11-793L10.1	6	102453903	10245414 2	S18_peak_3409	Intergenic between ENST00000407792 and ENST00000407015	ENST0000040779 2
CFAP58	10	104441081	10444132 0	S18_peak_341	intron (ENST00000369704, intron 15 of 17)	ENST0000036970 4
RP1-76C18.1	6	102798719	10279888 4	S18_peak_3410	Intergenic between ENST00000407792 and ENST00000407015	ENST0000040701 5
HACE1	6	104766229	10476645 5	S18_peak_3411	intron (ENST00000262903, intron 19 of 23)	ENST0000026290 3
RN7SKP211	6	105906807	10590697 2	S18_peak_3412	Intergenic between ENST00000363328 and ENST00000369096	ENST0000036332 8
QRSL1	6	106646727	10664701 9	S18_peak_3413	intron (ENST00000369046, intron 4 of 10)	ENST0000036904 6
PDSS2	6	107350538	10735084 0	S18_peak_3414	intron (ENST00000369037, intron 1 of 7)	ENST0000036903 7
SOBP	6	107484453	10748474 5	S18_peak_3415	Intergenic between ENST00000369031 and ENST00000317357	ENST0000031735 7
SCML4	6	107838660	10783891 0	S18_peak_3416	Intergenic between ENST00000369020 and ENST00000369002	ENST0000036902 0
SEC63	6	107882673	10788286 2	S18_peak_3417	intron (ENST00000369002, intron 17 of 20)	ENST0000036900 2
SEC63	6	107906995	10790717 7	S18_peak_3418	intron (ENST00000369002, intron 8 of 20)	ENST0000036900 2
ARMC2	6	108952862	10895305 2	S18_peak_3419	intron (ENST00000392644, intron 12 of 17)	ENST0000039264 4
RNU7-165P	10	113322506	11332267 1	S18_peak_342	Intergenic between ENST00000420976 and ENST00000458864	ENST0000045886 4
DDO	6	110375739	11037602 4	S18_peak_3420	Intergenic between ENST00000338882 and ENST00000368923	ENST0000036892 3
CDK19	6	110796125	11079629 0	S18_peak_3421	intron (ENST00000368911, intron 1 of 12)	ENST0000036891 1
SNORA40	6	110838627	11083879 2	S18_peak_3422	Intergenic between ENST00000363652 and ENST00000391285	ENST0000039128 5
RP1-159M24.1	6	112717924	11271815 4	S18_peak_3423	Intergenic between ENST00000418595 and ENST00000604713	ENST0000041859 5
FCF1P10	6	112999678	11299984 3	S18_peak_3424	Intergenic between ENST00000364516 and	ENST0000040759 0

					ENST00000407590	
RNU6-475P	6	114721276	114721441	S18_peak_3425	Intergenic between ENST00000435802 and ENST00000516073	ENST00000516073
RPS29P13	6	117041665	117041830	S18_peak_3426	Intergenic between ENST00000332958 and ENST00000402041	ENST00000402041
RP1-179P9.3	6	117474869	117475145	S18_peak_3427	intron (ENST00000467125, intron 4 of 6)	ENST00000467125
RP11-632C17__A.1	6	117936510	117936675	S18_peak_3428	Intergenic between ENST00000368494 and ENST00000443998	ENST00000443998
ASF1A	6	118874939	118875107	S18_peak_3429	Intergenic between ENST00000404721 and ENST00000229595	ENST00000229595
GFRA1	10	116107049	116107240	S18_peak_343	intron (ENST00000355422, intron 6 of 10)	ENST00000355422
RNU6-214P	6	120308665	120308911	S18_peak_3430	Intergenic between ENST00000579460 and ENST00000384130	ENST00000384130
COX6A1P3	6	120803699	120803864	S18_peak_3431	Intergenic between ENST00000404167 and ENST00000398212	ENST00000404167
COX6A1P3	6	120857316	120857497	S18_peak_3432	Intergenic between ENST00000404167 and ENST00000398212	ENST00000404167
TBC1D32	6	121163318	121163483	S18_peak_3433	intron (ENST00000398212, intron 22 of 31)	ENST00000398212
TBC1D32	6	121282990	121283166	S18_peak_3434	intron (ENST00000398212, intron 13 of 31)	ENST00000398212
HMGB3P18	6	121811619	121811819	S18_peak_3435	Intergenic between ENST00000403062 and ENST00000406327	ENST00000406327
RP3-425C14.5	6	122488457	122488622	S18_peak_3436	Intergenic between ENST00000624363 and ENST00000619967	ENST00000624363
RP11-374A22.1	6	123862369	123862534	S18_peak_3437	Intergenic between ENST00000441521 and ENST00000439075	ENST00000441521
TPD52L1	6	125170419	125170655	S18_peak_3438	intron (ENST00000534000, intron 1 of 6)	ENST00000534000
NCOA7	6	125834635	125834800	S18_peak_3439	intron (ENST00000368357, intron 3 of 16)	ENST00000368357
SLC18A2	10	117254476	117254641	S18_peak_344	intron (ENST00000298472, intron 6 of 15)	ENST00000298472
RP11-527F13.1	6	126186948	126187209	S18_peak_3440	intron (ENST00000444229, intron 1 of 3)	ENST00000444229
CENPW	6	126313176	126313341	S18_peak_3441	Intergenic between ENST00000440334 and ENST00000368325	ENST00000368325
RPS4XP9	6	126837476	126837752	S18_peak_3442	Intergenic between ENST00000405151 and ENST00000356698	ENST00000405151
RP11-325O24.6	6	127691008	127691173	S18_peak_3443	Intergenic between ENST00000625799 and ENST00000622571	ENST00000625799
RP11-325O24.6	6	127769462	127769627	S18_peak_3444	Intergenic between ENST00000625799 and	ENST00000625799

					ENST00000622571	
PTPRK	6	128012094	128012439	S18_peak_3445	intron (ENST00000368226, intron 13 of 29)	ENST00000368226
PTPRK	6	128027757	128027922	S18_peak_3446	intron (ENST00000368226, intron 13 of 29)	ENST00000368226
PTPRK	6	128185683	128185895	S18_peak_3447	intron (ENST00000368226, intron 6 of 29)	ENST00000368226
LAMA2	6	128835674	128835839	S18_peak_3448	Intergenic between ENST00000365330 and ENST00000421865	ENST00000421865
LAMA2	6	129451816	129451981	S18_peak_3449	intron (ENST00000421865, intron 45 of 64)	ENST00000421865
SEC23IP	10	119945393	119945558	S18_peak_345	TTS (ENST00000369075)	ENST00000369075
LAMA2	6	129508675	129508983	S18_peak_3450	intron (ENST00000421865, intron 62 of 64)	ENST00000421865
TMEM200A	6	130317809	130317974	S18_peak_3451	Intergenic between ENST00000324172 and ENST00000296978	ENST00000296978
SMLR1	6	130825423	130825666	S18_peak_3452	Intergenic between ENST00000454262 and ENST00000541421	ENST00000541421
ARG1	6	131553374	131553566	S18_peak_3453	Intergenic between ENST00000402896 and ENST00000356962	ENST00000356962
OR2A4	6	131688202	131688367	S18_peak_3454	Intergenic between ENST00000516751 and ENST00000315453	ENST00000315453
OR2A4	6	131700903	131701068	S18_peak_3455	promoter-TSS (ENST00000315453)	ENST00000315453
RP5-988G15.1	6	131760072	131760237	S18_peak_3456	Intergenic between ENST00000314099 and ENST00000401879	ENST00000401879
LINC01013	6	132141788	132141953	S18_peak_3457	intron (ENST00000458028, intron 1 of 1)	ENST00000458028
LINC00326	6	133073691	133073888	S18_peak_3458	Intergenic between ENST00000438548 and ENST00000457339	ENST00000457339
EYA4	6	133308575	133308740	S18_peak_3459	intron (ENST00000367895, intron 2 of 19)	ENST00000367895
RP11-781P14.3	10	120116476	120116641	S18_peak_346	Intergenic between ENST00000425391 and ENST00000495531	ENST00000425391
RP11-557H15.4	6	134527646	134527811	S18_peak_3460	intron (ENST00000607531, intron 3 of 3)	ENST00000607531
PDE7B	6	135934418	135934583	S18_peak_3461	intron (ENST00000308191, intron 1 of 12)	ENST00000308191
PDE7B	6	135968514	135968723	S18_peak_3462	intron (ENST00000308191, intron 2 of 12)	ENST00000308191
MTFR2	6	136235070	136235302	S18_peak_3463	intron (ENST00000420702, intron 6 of 7)	ENST00000420702
BCLAF1	6	136266314	136266479	S18_peak_3464	intron (ENST00000531224, intron 11 of 12)	ENST00000531224
RP3-406A7.7	6	136330731	136331090	S18_peak_3465	Intergenic between ENST00000403956 and ENST00000564248	ENST00000564248
MAP7	6	136523940	136524145	S18_peak_3466	intron (ENST00000354570, intron 1 of 17)	ENST00000354570

SLC35D3	6	136918365	13691853 0	S18_peak_3467	Intergenic between ENST00000425027 and ENST00000331858	ENST0000033185 8
BTF3L4P3	6	137577805	13757797 0	S18_peak_3468	Intergenic between ENST00000418879 and ENST00000638039	ENST0000041887 9
RP11-356I2.4	6	137826115	13782629 3	S18_peak_3469	intron (ENST00000606998, intron 3 of 3)	ENST0000060699 8
RPS27P18	10	125470394	12547055 9	S18_peak_347	Intergenic between ENST00000415184 and ENST00000431701	ENST0000043170 1
ARFGEF3	6	138207131	13820729 6	S18_peak_3470	intron (ENST00000251691, intron 3 of 33)	ENST0000025169 1
ARFGEF3	6	138245148	13824531 3	S18_peak_3471	intron (ENST00000251691, intron 7 of 33)	ENST0000025169 1
RP3-509I19.6	6	138853137	13885330 2	S18_peak_3472	Intergenic between ENST00000449542 and ENST00000572284	ENST0000057228 4
RP1-225E12.3	6	139250539	13925072 9	S18_peak_3473	intron (ENST00000621913, intron 5 of 6)	ENST0000062191 3
RP11-445F6.2	6	139294609	13929482 7	S18_peak_3474	Intergenic between ENST00000440518 and ENST00000403909	ENST0000044051 8
LINC01625	6	139444474	13944468 5	S18_peak_3475	Intergenic between ENST00000367651 and ENST00000454788	ENST0000045478 8
LINC01625	6	139498941	13949912 6	S18_peak_3476	Intergenic between ENST00000454788 and ENST00000402768	ENST0000045478 8
MIR3668	6	140232730	14023295 0	S18_peak_3477	Intergenic between ENST00000582138 and ENST00000583434	ENST0000058213 8
MIR3668	6	140407954	14040820 5	S18_peak_3478	Intergenic between ENST00000582138 and ENST00000583434	ENST0000058213 8
MIR4465	6	140613523	14061368 8	S18_peak_3479	Intergenic between ENST00000582138 and ENST00000583434	ENST0000058343 4
SNORD60	10	126815109	12681527 4	S18_peak_348	Intergenic between ENST00000362451 and ENST00000280333	ENST0000036245 1
RPS3AP23	6	141712952	14171311 7	S18_peak_3480	Intergenic between ENST00000401762 and ENST00000604304	ENST0000040176 2
RP11-137J7.3	6	141910910	14191111 2	S18_peak_3481	Intergenic between ENST00000401762 and ENST00000604304	ENST0000060430 4
VTA1	6	142141417	14214158 2	S18_peak_3482	Intergenic between ENST00000450456 and ENST00000367630	ENST0000036763 0
ADGRG6	6	142288545	14228875 2	S18_peak_3483	Intergenic between ENST00000426166 and ENST00000230173	ENST0000023017 3
RP1-20N2.7	6	143365087	14336525 2	S18_peak_3484	Intergenic between ENST00000402907 and ENST00000604177	ENST0000060417 7
PHACTR2	6	143719395	14371962 2	S18_peak_3485	intron (ENST00000427704, intron 2 of 12)	ENST0000042770 4

TPT1P4	6	144203324	144203489	S18_peak_3486	Intergenic between ENST00000403538 and ENST00000404655	ENST00000403538
UTRN	6	144331005	144331170	S18_peak_3487	intron (ENST00000367545, intron 1 of 73)	ENST00000367545
UTRN	6	144377867	144378032	S18_peak_3488	intron (ENST00000367545, intron 1 of 73)	ENST00000367545
RNU1-33P	6	145271012	145271177	S18_peak_3489	Intergenic between ENST00000402316 and ENST00000364249	ENST00000364249
BUB1P1	10	127814941	127815124	S18_peak_349	Intergenic between ENST00000388920 and ENST00000603541	ENST00000603541
RP11-545I5.3	6	145901995	145902160	S18_peak_3490	Intergenic between ENST00000626833 and ENST00000492807	ENST00000626833
RP11-715G15.1	6	146578593	146578777	S18_peak_3491	Intergenic between ENST00000367495 and ENST00000419168	ENST00000419168
RP11-307P5.1	6	147788964	147789153	S18_peak_3492	intron (ENST00000427015, intron 1 of 2)	ENST00000427015
RP1-111D6.4	6	149263568	149263733	S18_peak_3493	Intergenic between ENST00000637865 and ENST00000485574	ENST00000637865
GINM1	6	149580853	149581018	S18_peak_3494	intron (ENST00000367419, intron 6 of 7)	ENST00000367419
LATS1	6	149713507	149713672	S18_peak_3495	intron (ENST00000543571, intron 1 of 7)	ENST00000543571
RAET1E-AS1	6	149883945	149884110	S18_peak_3496	intron (ENST00000606915, intron 1 of 1)	ENST00000606915
ULBP1	6	149960875	149961152	S18_peak_3497	Intergenic between ENST00000399570 and ENST00000229708	ENST00000229708
ULBP1	6	149965573	149965738	S18_peak_3498	promoter-TSS (ENST00000229708)	ENST00000229708
RAET1K	6	150012322	150012571	S18_peak_3499	Intergenic between ENST00000403651 and ENST00000405282	ENST00000403651
KLF18	1	44137134	44137299	S18_peak_35	TTS (ENST00000634670)	ENST00000634670
RP11-45A17.3	10	132015694	132015859	S18_peak_350	Intergenic between ENST00000623138 and ENST00000298622	ENST00000623138
IYD	6	150377491	150377664	S18_peak_3500	intron (ENST00000344419, intron 1 of 4)	ENST00000344419
IYD	6	150377999	150378184	S18_peak_3501	intron (ENST00000344419, intron 1 of 4)	ENST00000344419
PLEKHG1	6	150763265	150763571	S18_peak_3502	intron (ENST00000358517, intron 2 of 15)	ENST00000358517
RNU6-302P	6	150952666	150952831	S18_peak_3503	Intergenic between ENST00000365249 and ENST00000445833	ENST00000365249
RP1-292B18.3	6	151051706	151051871	S18_peak_3504	Intergenic between ENST00000365249 and ENST00000445833	ENST00000445833
RP11-613F7.1	6	151246467	151246632	S18_peak_3505	Intergenic between ENST00000440335 and ENST00000516622	ENST00000440335

CCDC170	6	151520159	151520405	S18_peak_3506	intron (ENST00000239374, intron 1 of 10)	ENST00000239374
RP3-472M2.2	6	154845575	154845740	S18_peak_3507	promoter-TSS (ENST00000404382)	ENST00000404382
NOX3	6	155414497	155414662	S18_peak_3508	intron (ENST00000159060, intron 10 of 13)	ENST00000159060
SNX9	6	157823746	157823911	S18_peak_3509	intron (ENST00000614703, intron 3 of 5)	ENST00000614703
RP11-432J24.3	10	132469510	132469675	S18_peak_351	Intergenic between ENST00000450206 and ENST00000455414	ENST00000450206
SERAC1	6	158115105	158115270	S18_peak_3510	intron (ENST00000367104, intron 14 of 16)	ENST00000367104
CACYBPP3	6	158523707	158523946	S18_peak_3511	Intergenic between ENST00000406011 and ENST00000367090	ENST00000406011
RP1-111C20.4	6	159013679	159013909	S18_peak_3512	intron (ENST00000607391, intron 2 of 4)	ENST00000607391
AGPAT4	6	161262312	161262616	S18_peak_3513	intron (ENST00000320285, intron 1 of 8)	ENST00000320285
AGPAT4-IT1	6	161356610	161356775	S18_peak_3514	Intergenic between ENST00000624499 and ENST00000441609	ENST00000624499
KRT8P44	6	162557415	162557580	S18_peak_3515	Intergenic between ENST00000624499 and ENST00000441609	ENST00000441609
QKI	6	163500897	163501201	S18_peak_3516	intron (ENST00000275262, intron 3 of 6)	ENST00000275262
RP11-534P19.1	6	165004505	165004696	S18_peak_3517	Intergenic between ENST00000605741 and ENST00000407696	ENST00000407696
RP11-534P19.1	6	165195367	165195585	S18_peak_3518	Intergenic between ENST00000407696 and ENST00000230301	ENST00000407696
PDE10A	6	165655768	165655933	S18_peak_3519	intron (ENST00000539869, intron 1 of 21)	ENST00000539869
ADGRA1	10	133111704	133111953	S18_peak_352	intron (ENST00000392607, intron 5 of 6)	ENST00000392607
RP11-514O12.4	6	166849102	166849308	S18_peak_3520	Intergenic between ENST00000454907 and ENST00000507747	ENST00000507747
RP3-366N23.4	6	167404581	167404746	S18_peak_3521	Intergenic between ENST00000402817 and ENST00000609107	ENST00000402817
MLLT4	6	167977610	167977775	S18_peak_3522	Intergenic between ENST00000392108 and ENST00000456585	ENST00000392108
RPL12P23	6	170196507	170196785	S18_peak_3523	Intergenic between ENST00000610240 and ENST00000405880	ENST00000405880
FAM120B	6	170397953	170398164	S18_peak_3524	intron (ENST00000630384, intron 9 of 10)	ENST00000630384
ABC7-481722F1.2	7	9918	10140	S18_peak_3525	Intergenic before ENST00000635602	ENST00000635602
RP11-90P13.1	7	167080	167248	S18_peak_3526	Intergenic between ENST00000567533 and ENST00000497017	ENST00000567533
FAM20C	7	206614	206779	S18_peak_3527	intron (ENST00000313766,	ENST0000031376

					intron 2 of 9)	6
C7orf50	7	1117633	1117798	S18_peak_3528	intron (ENST00000397098, intron 2 of 4)	ENST0000039709 8
LFNG	7	2507651	2507816	S18_peak_3529	Intergenic between ENST00000611693 and ENST00000402506	ENST0000040250 6
AGGF1P2	10	133642639	13364286 6	S18_peak_353	promoter-TSS (ENST00000426656)	ENST0000042665 6
IQCE	7	2579601	2579766	S18_peak_3530	intron (ENST00000402050, intron 8 of 21)	ENST0000040205 0
IQCE	7	2599864	2600029	S18_peak_3531	intron (ENST00000402050, intron 17 of 21)	ENST0000040205 0
SDK1	7	4070448	4070613	S18_peak_3532	Intergenic between ENST00000427920 and ENST00000476701	ENST0000047670 1
SDK1	7	4151180	4151350	S18_peak_3533	intron (ENST00000476701, intron 4 of 19)	ENST0000047670 1
CYP3A54P	7	4589263	4589428	S18_peak_3534	Intergenic between ENST00000566950 and ENST00000328914	ENST0000056695 0
ZNF890P	7	5121018	5121338	S18_peak_3535	TTS (ENST00000530367)	ENST0000053036 7
WIPI2	7	5185585	5185750	S18_peak_3536	Intergenic between ENST00000365169 and ENST00000288828	ENST0000028882 8
WIPI2	7	5191151	5191339	S18_peak_3537	promoter-TSS (ENST00000288828)	ENST0000028882 8
FBXL18	7	5485239	5485484	S18_peak_3538	intron (ENST00000382368, intron 4 of 4)	ENST0000038236 8
FBXL18	7	5493199	5493464	S18_peak_3539	intron (ENST00000382368, intron 3 of 4)	ENST0000038236 8
RP11-304M2.3	11	184034	184263	S18_peak_354	intron (ENST00000527297, intron 2 of 2)	ENST0000052729 7
FBXL18	7	5493817	5494087	S18_peak_3540	intron (ENST00000382368, intron 3 of 4)	ENST0000038236 8
ZNF815P	7	5854856	5855290	S18_peak_3541	TTS (ENST00000422825)	ENST0000042282 5
CCZ1	7	5945321	5945486	S18_peak_3542	Intergenic between ENST00000325974 and ENST00000265849	ENST0000032597 4
KDELR2	7	6459192	6459357	S18_peak_3543	Intergenic between ENST00000425398 and ENST00000258739	ENST0000025873 9
AC079742.4	7	6579216	6579381	S18_peak_3544	intron (ENST00000434951, intron 1 of 1)	ENST0000043495 1
OR7E59P	7	6881183	6881458	S18_peak_3545	Intergenic between ENST00000508367 and ENST00000490376	ENST0000050836 7
ALG1L5P	7	6919013	6919178	S18_peak_3546	Intergenic between ENST00000490376 and ENST00000482043	ENST0000048204 3
Y_RNA	7	7124418	7124590	S18_peak_3547	Intergenic between ENST00000364348 and ENST00000467154	ENST0000036434 8
RP4-733B9.1	7	7192251	7192467	S18_peak_3548	Intergenic between ENST00000467154 and ENST00000430266	ENST0000046715 4

AC005532.5	7	7268281	7268446	S18_peak_3549	intron (ENST00000430266, intron 3 of 3)	ENST0000043026 6
PSMD13	11	249407	249677	S18_peak_355	intron (ENST00000532097, intron 9 of 12)	ENST0000053209 7
ICA1	7	8122114	8122279	S18_peak_3550	intron (ENST00000402384, intron 13 of 13)	ENST0000040238 4
ICA1	7	8216454	8216619	S18_peak_3551	intron (ENST00000402384, intron 6 of 13)	ENST0000040238 4
AC007128.1	7	8300134	8300299	S18_peak_3552	intron (ENST00000424460, intron 3 of 6)	ENST0000042446 0
AC011288.2	7	13478736	13478912	S18_peak_3553	intron (ENST00000411542, intron 3 of 4)	ENST0000041154 2
ITGB8	7	20337956	20338121	S18_peak_3554	intron (ENST00000222573, intron 1 of 13)	ENST0000022257 3
JAZF1	7	27943380	27943545	S18_peak_3555	intron (ENST00000283928, intron 2 of 4)	ENST0000028392 8
CHN2	7	29377679	29377844	S18_peak_3556	intron (ENST00000222792, intron 3 of 12)	ENST0000022279 2
snoU13	7	29853194	29853359	S18_peak_3557	Intergenic between ENST00000602980 and ENST00000459368	ENST0000045936 8
ZNRF2	7	30270110	30270275	S18_peak_3558	Intergenic between ENST00000430537 and ENST00000323037	ENST0000032303 7
NOD1	7	30463522	30463687	S18_peak_3559	intron (ENST00000222823, intron 1 of 13)	ENST0000022282 3
AP006621.8	11	827503	827668	S18_peak_356	intron (ENST00000532946, intron 2 of 2)	ENST0000053294 6
PDE1C	7	31787251	31787501	S18_peak_3560	intron (ENST00000396191, intron 16 of 17)	ENST0000039619 1
PDE1C	7	31886716	31886957	S18_peak_3561	intron (ENST00000396191, intron 2 of 17)	ENST0000039619 1
SNX2P2	7	32170895	32171075	S18_peak_3562	Intergenic between ENST00000424553 and ENST00000435950	ENST0000042455 3
AC018641.7	7	32322102	32322267	S18_peak_3563	Intergenic between ENST00000424553 and ENST00000435950	ENST0000043595 0
AC018641.7	7	32376776	32376947	S18_peak_3564	Intergenic between ENST00000424553 and ENST00000435950	ENST0000043595 0
AC018641.7	7	32387523	32387721	S18_peak_3565	Intergenic between ENST00000424553 and ENST00000435950	ENST0000043595 0
BBS9	7	33254049	33254214	S18_peak_3566	intron (ENST00000242067, intron 5 of 22)	ENST0000024206 7
BBS9	7	33481943	33482108	S18_peak_3567	intron (ENST00000242067, intron 19 of 22)	ENST0000024206 7
RP11-89N17.4	7	33765803	33765968	S18_peak_3568	Intergenic between ENST00000455149 and ENST00000420185	ENST0000042018 5
RP11-89N17.4	7	33775870	33776035	S18_peak_3569	Intergenic between ENST00000455149 and ENST00000420185	ENST0000042018 5
BRSK2	11	1351757	1352232	S18_peak_357	Intergenic between ENST00000530897 and ENST00000531197	ENST0000053119 7
TRGVA	7	38323744	38323942	S18_peak_3570	Intergenic between	ENST0000041381

					ENST00000413819 and ENST00000609522	9
AMPH	7	38467189	38467354	S18_peak_3571	intron (ENST00000325590, intron 7 of 19)	ENST0000032559 0
VPS41	7	38911862	38912027	S18_peak_3572	Intergenic between ENST00000395969 and ENST00000403058	ENST0000039596 9
POU6F2	7	39230129	39230294	S18_peak_3573	intron (ENST00000403058, intron 5 of 10)	ENST0000040305 8
MPLKIP	7	40121623	40121827	S18_peak_3574	Intergenic between ENST00000603944 and ENST00000306984	ENST0000030698 4
SUGCT	7	40140444	40140646	S18_peak_3575	intron (ENST00000416370, intron 1 of 12)	ENST0000041637 0
C7orf57	7	48048815	48049057	S18_peak_3576	intron (ENST00000435376, intron 4 of 6)	ENST0000043537 6
C7orf72	7	50131564	50131734	S18_peak_3577	intron (ENST00000297001, intron 3 of 8)	ENST0000029700 1
C7orf72	7	50153677	50153949	S18_peak_3578	intron (ENST00000297001, intron 8 of 8)	ENST0000029700 1
AC004920.3	7	50813054	50813240	S18_peak_3579	Intergenic between ENST00000357271 and ENST00000422831	ENST0000042283 1
BRSK2	11	1352421	1352680	S18_peak_358	Intergenic between ENST00000530897 and ENST00000531197	ENST0000053119 7
COBL	7	51206118	51206283	S18_peak_3580	intron (ENST00000265136, intron 2 of 12)	ENST0000026513 6
CTD-2021A8.3	7	51429956	51430316	S18_peak_3581	Intergenic between ENST00000446669 and ENST00000422054	ENST0000044666 9
RP11-153N17.1	7	52026093	52026258	S18_peak_3582	Intergenic between ENST00000473305 and ENST00000441295	ENST0000044129 5
HPVC1	7	54153606	54153772	S18_peak_3583	Intergenic between ENST00000418679 and ENST00000629899	ENST0000062989 9
LINC01445	7	54299100	54299265	S18_peak_3584	Intergenic between ENST00000629899 and ENST00000458615	ENST0000045861 5
LINC01445	7	54374579	54374744	S18_peak_3585	Intergenic between ENST00000426205 and ENST00000444439	ENST0000042620 5
VSTM2A	7	54563525	54563690	S18_peak_3586	intron (ENST00000407838, intron 4 of 4)	ENST0000040783 8
RNU6-1125P	7	54612127	54612292	S18_peak_3587	Intergenic between ENST00000439027 and ENST00000363601	ENST0000036360 1
RP4-791C19.1	7	54917778	54918081	S18_peak_3588	Intergenic between ENST00000439413 and ENST00000407916	ENST0000040791 6
RP4-791C19.1	7	54921624	54921882	S18_peak_3589	Intergenic between ENST00000439413 and ENST00000407916	ENST0000040791 6
ASCL2	11	2237672	2237959	S18_peak_359	Intergenic between ENST00000584128 and ENST00000331289	ENST0000033128 9
ELDR	7	55213481	55213646	S18_peak_3590	Intergenic between	ENST0000062653

					ENST00000442411 and ENST00000626532	2
LANCL2	7	55416387	55416605	S18_peak_3591	intron (ENST00000254770, intron 6 of 8)	ENST0000025477 0
LANCL2	7	55449505	55449670	S18_peak_3592	Intergenic between ENST00000254770 and ENST00000285279	ENST0000025477 0
RP11-310H4.1	7	55586313	55586562	S18_peak_3593	intron (ENST00000454777, intron 1 of 2)	ENST0000045477 7
RP11-310H4.6	7	55673892	55674057	S18_peak_3594	intron (ENST00000432235, intron 3 of 5)	ENST0000043223 5
PSPHP1	7	55758176	55758414	S18_peak_3595	Intergenic between ENST00000619081 and ENST00000450062	ENST0000045006 2
14-Sep	7	55811555	55811778	S18_peak_3596	intron (ENST00000388975, intron 7 of 9)	ENST0000038897 5
14-Sep	7	55854603	55854778	S18_peak_3597	intron (ENST00000388975, intron 2 of 9)	ENST0000038897 5
SUMF2	7	56084240	56084589	S18_peak_3598	Intergenic between ENST00000342190 and ENST00000395422	ENST0000034219 0
NUPR2	7	56135916	56136081	S18_peak_3599	Intergenic between ENST00000329309 and ENST00000442064	ENST0000032930 9
TMEM53	1	44675567	44675732	S18_peak_36	Intergenic between ENST00000372237 and ENST00000362507	ENST0000037223 7
NAP1L4	11	2984314	2984479	S18_peak_360	intron (ENST00000380542, intron 1 of 15)	ENST0000038054 2
CICP8	7	56372097	56372279	S18_peak_3600	Intergenic between ENST00000455800 and ENST00000450682	ENST0000045580 0
CICP8	7	56372561	56372904	S18_peak_3601	Intergenic between ENST00000455800 and ENST00000450682	ENST0000045580 0
RP11-814E24.3	7	56374511	56374685	S18_peak_3603	Intergenic between ENST00000455800 and ENST00000450682	ENST0000045068 2
RP11-10F11.4	7	56748382	56748564	S18_peak_3604	Intergenic between ENST00000634439 and ENST00000599370	ENST0000063443 9
RP11-715L17.1	7	60917450	60917723	S18_peak_3605	Intergenic between ENST00000604991 and ENST00000454392	ENST0000045439 2
RP11-715L17.1	7	61378789	61378954	S18_peak_3606	Intergenic between ENST00000604991 and ENST00000454392	ENST0000045439 2
RP11-715L17.1	7	61654350	61654552	S18_peak_3607	Intergenic between ENST00000604991 and ENST00000454392	ENST0000045439 2
RP11-715L17.1	7	61760209	61760497	S18_peak_3608	Intergenic between ENST00000604991 and ENST00000454392	ENST0000045439 2
OSBPL5	11	3090524	3090689	S18_peak_361	exon (ENST00000263650, exon 20 of 22)	ENST0000026365 0
RP11-715L17.1	7	62366695	62367018	S18_peak_3610	Intergenic between ENST00000454392 and ENST00000384712	ENST0000045439 2

RNU6-912P	7	65819188	65819353	S18_peak_3611	Intergenic between ENST00000363800 and ENST00000447086	ENST00000363800
RABGEF1	7	66786366	66786609	S18_peak_3612	intron (ENST00000284957, intron 4 of 8)	ENST00000284957
TMEM248	7	66940876	66941095	S18_peak_3613	intron (ENST00000341567, intron 1 of 6)	ENST00000341567
RNA5SP231	7	68729351	68729516	S18_peak_3614	Intergenic between ENST00000410496 and ENST00000447158	ENST00000410496
RP11-3P22.2	7	69424456	69424673	S18_peak_3615	intron (ENST00000435148, intron 1 of 2)	ENST00000435148
NSUN5P2	7	72951638	72951803	S18_peak_3616	intron (ENST00000602348, intron 1 of 4)	ENST00000602348
RP11-313P13.4	7	73105038	73105210	S18_peak_3617	intron (ENST00000333674, intron 5 of 5)	ENST00000333674
SPDYE10P	7	73131389	73131688	S18_peak_3618	Intergenic between ENST00000612005 and ENST00000544802	ENST00000612005
GTF2IP4	7	73197509	73197699	S18_peak_3619	intron (ENST00000544802, intron 15 of 23)	ENST00000544802
RP13-726E6.2	11	3452394	3452731	S18_peak_362	intron (ENST00000637148, intron 1 of 3)	ENST00000637148
NCF1B	7	73225244	73225449	S18_peak_3620	intron (ENST00000432102, intron 2 of 5)	ENST00000432102
SPDYE12P	7	74902917	74903108	S18_peak_3621	Intergenic between ENST00000459002 and ENST00000618416	ENST00000618416
NCF1C	7	75172077	75172284	S18_peak_3622	Intergenic between ENST00000438382 and ENST00000618412	ENST00000438382
SPDYE13P	7	75264155	75264320	S18_peak_3623	Intergenic between ENST00000621495 and ENST00000445314	ENST00000445314
SPDYE13P	7	75286554	75286761	S18_peak_3624	intron (ENST00000445314, intron 5 of 6)	ENST00000445314
AC006014.7	7	75490472	75490637	S18_peak_3625	Intergenic between ENST00000415277 and ENST00000529061	ENST00000415277
PMS2P3	7	75521652	75521817	S18_peak_3626	intron (ENST00000529061, intron 2 of 9)	ENST00000529061
RSBN1L	7	77749162	77749395	S18_peak_3627	intron (ENST00000334955, intron 2 of 7)	ENST00000334955
GNAT3	7	80510063	80510228	S18_peak_3628	promoter-TSS (ENST00000398291)	ENST00000398291
PQLC1P1	7	88608163	88608328	S18_peak_3629	Intergenic between ENST00000419546 and ENST00000434950	ENST00000434950
RP13-726E6.1	11	3508811	3509031	S18_peak_363	intron (ENST00000534291, intron 2 of 2)	ENST00000534291
MTERF1	7	91824990	91825155	S18_peak_3630	Intergenic between ENST00000415249 and ENST00000351870	ENST00000351870
RN7SL7P	7	92958933	92959115	S18_peak_3631	Intergenic between ENST00000424523 and ENST00000467823	ENST00000467823
BET1	7	93987081	93987283	S18_peak_3632	intron (ENST00000357520, intron 4 of 6)	ENST00000357520

COL1A2	7	94412048	94412213	S18_peak_3633	intron (ENST00000297268, intron 24 of 51)	ENST00000297268
ARPC1B	7	99372904	99373114	S18_peak_3634	Intergenic between ENST00000262942 and ENST00000252725	ENST00000252725
RP11-758P17.3	7	100444414	100444579	S18_peak_3635	Intergenic between ENST00000475250 and ENST00000332375	ENST00000475250
CLDN15	7	101233822	101234045	S18_peak_3636	intron (ENST00000401528, intron 3 of 5)	ENST00000401528
AC004953.1	7	101661402	101661602	S18_peak_3637	Intergenic between ENST00000624170 and ENST00000292538	ENST00000624170
RASA4	7	102609495	102609690	S18_peak_3638	intron (ENST00000262940, intron 2 of 20)	ENST00000262940
RP11-514P8.6	7	102624636	102624803	S18_peak_3639	intron (ENST00000519541, intron 5 of 25)	ENST00000519541
RRM1	11	4128425	4128663	S18_peak_364	intron (ENST00000300738, intron 14 of 18)	ENST00000300738
RP11-577H5.5	7	102649356	102649630	S18_peak_3640	intron (ENST00000476151, intron 4 of 8)	ENST00000476151
FBXL13	7	102890439	102890648	S18_peak_3641	intron (ENST00000379305, intron 11 of 18)	ENST00000379305
CTB-111H14.1	7	106752657	106752830	S18_peak_3642	intron (ENST00000592441, intron 2 of 4)	ENST00000592441
CTB-111H14.1	7	106767224	106767389	S18_peak_3643	intron (ENST00000592441, intron 1 of 4)	ENST00000592441
PRKAR2B	7	107090215	107090430	S18_peak_3644	intron (ENST00000265717, intron 2 of 10)	ENST00000265717
COG5	7	107213310	107213475	S18_peak_3645	intron (ENST00000347053, intron 18 of 20)	ENST00000347053
COG5	7	107220839	107221004	S18_peak_3646	intron (ENST00000347053, intron 18 of 20)	ENST00000347053
NRCAM	7	108250192	108250357	S18_peak_3647	intron (ENST00000351718, intron 3 of 27)	ENST00000351718
NRCAM	7	108327071	108327254	S18_peak_3648	intron (ENST00000351718, intron 2 of 27)	ENST00000351718
NRCAM	7	108388816	108389010	S18_peak_3649	intron (ENST00000351718, intron 2 of 27)	ENST00000351718
SSU72P4	11	4288955	4289120	S18_peak_365	Intergenic between ENST00000530088 and ENST00000531780	ENST00000530088
RP11-745H7.1	7	110144184	110144401	S18_peak_3650	Intergenic between ENST00000432928 and ENST00000612027	ENST00000612027
AC073326.3	7	110675891	110676089	S18_peak_3651	Intergenic between ENST00000435466 and ENST00000451832	ENST00000451832
AC003989.4	7	111431030	111431195	S18_peak_3652	Intergenic between ENST00000434569 and ENST00000445943	ENST00000434569
PPP1R3A	7	113929940	113930105	S18_peak_3653	Intergenic between ENST00000284602 and ENST00000441290	ENST00000284602
FOXP2	7	114686326	114686491	S18_peak_3654	intron (ENST00000408937, intron 17 of 17)	ENST00000408937
MDFIC	7	114870945	114871134	S18_peak_3655	Intergenic between ENST00000607845 and	ENST00000257724

					ENST00000257724	
TFEC	7	116025347	116025614	S18_peak_3656	intron (ENST00000265440, intron 1 of 7)	ENST00000265440
ST7	7	116987844	116988070	S18_peak_3657	intron (ENST00000265437, intron 1 of 15)	ENST00000265437
ST7	7	117016934	117017099	S18_peak_3658	intron (ENST00000265437, intron 1 of 15)	ENST00000265437
RN7SKP277	7	121738710	121738875	S18_peak_3659	Intergenic between ENST00000516895 and ENST00000456768	ENST00000516895
SSU72P7	11	4346979	4347182	S18_peak_366	Intergenic between ENST00000532284 and ENST00000624801	ENST00000532284
CADPS2	7	122789940	122790213	S18_peak_3660	intron (ENST00000412584, intron 1 of 27)	ENST00000412584
AC000374.1	7	126537630	126537795	S18_peak_3661	Intergenic between ENST00000434698 and ENST00000452711	ENST00000434698
SND1	7	127706806	127706992	S18_peak_3662	intron (ENST00000354725, intron 8 of 23)	ENST00000354725
SND1	7	127835498	127835663	S18_peak_3663	intron (ENST00000354725, intron 11 of 23)	ENST00000354725
LEP	7	128252917	128253214	S18_peak_3664	intron (ENST00000308868, intron 2 of 2)	ENST00000308868
RP11-62J1.4	7	128276507	128276672	S18_peak_3665	Intergenic between ENST00000608625 and ENST00000223073	ENST00000608625
METTL2B	7	128499490	128499709	S18_peak_3666	intron (ENST00000262432, intron 7 of 8)	ENST00000262432
CCDC136	7	128785750	128785942	S18_peak_3667	Intergenic between ENST00000249389 and ENST00000378685	ENST00000378685
FLNC	7	128838184	128838349	S18_peak_3668	intron (ENST00000325888, intron 6 of 47)	ENST00000325888
SMKR1	7	129504693	129504861	S18_peak_3669	intron (ENST00000462322, intron 1 of 1)	ENST00000462322
SSU72P7	11	4349644	4349835	S18_peak_367	Intergenic between ENST00000532284 and ENST00000624801	ENST00000532284
RP11-306G20.1	7	130016939	130017104	S18_peak_3670	intron (ENST00000587038, intron 3 of 4)	ENST00000587038
SSMEM1	7	130209075	130209240	S18_peak_3671	promoter-TSS (ENST00000297819)	ENST00000297819
AC009362.2	7	131194454	131194619	S18_peak_3672	Intergenic between ENST00000362757 and ENST00000427253	ENST00000427253
AC011625.1	7	132323159	132323345	S18_peak_3673	Intergenic between ENST00000445459 and ENST00000455442	ENST00000455442
SLC13A4	7	135701801	135702016	S18_peak_3674	intron (ENST00000354042, intron 6 of 15)	ENST00000354042
AC009264.1	7	136816716	136816881	S18_peak_3675	intron (ENST00000586239, intron 2 of 4)	ENST00000586239
TRIM24	7	138543873	138544100	S18_peak_3676	intron (ENST00000343526, intron 7 of 18)	ENST00000343526
HIPK2	7	139639124	139639355	S18_peak_3677	intron (ENST00000406875, intron 2 of 14)	ENST00000406875

RNU6-267P	7	143726045	14372621 0	S18_peak_3678	Intergenic between ENST00000638055 and ENST00000516714	ENST0000051671 4
RP4-545C24.1	7	144262344	14426258 5	S18_peak_3679	intron (ENST00000460955, intron 2 of 2)	ENST0000046095 5
C11orf40	11	4569588	4569802	S18_peak_368	Intergenic between ENST00000360213 and ENST00000307616	ENST0000030761 6
TPK1	7	144662302	14466246 7	S18_peak_3680	intron (ENST00000360057, intron 4 of 8)	ENST0000036005 7
AC005229.5	7	148581988	14858218 5	S18_peak_3681	Intergenic between ENST00000456995 and ENST00000307003	ENST0000045699 5
RN7SL569P	7	148891894	14889208 1	S18_peak_3682	Intergenic between ENST00000494652 and ENST00000516507	ENST0000049465 2
LRRC61	7	150260484	15026064 9	S18_peak_3683	Intergenic between ENST00000495144 and ENST00000323078	ENST0000032307 8
LRRC61	7	150295972	15029615 3	S18_peak_3684	Intergenic between ENST00000495144 and ENST00000323078	ENST0000032307 8
LRRC61	7	150297540	15029783 9	S18_peak_3685	Intergenic between ENST00000495144 and ENST00000323078	ENST0000032307 8
PRKAG2-AS1	7	151879745	15187991 0	S18_peak_3686	TTS (ENST00000467458)	ENST0000046745 8
LINC01287	7	153343723	15334388 8	S18_peak_3687	Intergenic between ENST00000415468 and ENST00000454441	ENST0000045444 1
INSIG1	7	155307370	15530753 5	S18_peak_3688	intron (ENST00000340368, intron 5 of 5)	ENST0000034036 8
INSIG1	7	155327842	15532801 5	S18_peak_3689	Intergenic between ENST00000342407 and ENST00000401499	ENST0000034240 7
OR51F5P	11	4710105	4710303	S18_peak_369	promoter-TSS (ENST00000609912)	ENST0000060991 2
RBM33	7	155750758	15575092 3	S18_peak_3690	intron (ENST00000401878, intron 14 of 17)	ENST0000040187 8
AC021218.2	7	155923279	15592348 3	S18_peak_3691	Intergenic between ENST00000297261 and ENST00000377722	ENST0000037772 2
Y_RNA	7	156132225	15613249 3	S18_peak_3692	Intergenic between ENST00000377722 and ENST00000384333	ENST0000038433 3
RNF32	7	156646814	15664697 9	S18_peak_3693	intron (ENST00000392741, intron 2 of 6)	ENST0000039274 1
LMBR1	7	156795769	15679593 4	S18_peak_3694	intron (ENST00000353442, intron 5 of 16)	ENST0000035344 2
NOM1	7	156973963	15697417 3	S18_peak_3695	TTS (ENST00000275820)	ENST0000027582 0
RP5-1142J19.1	7	157302638	15730283 9	S18_peak_3696	Intergenic between ENST00000442017 and ENST00000262177	ENST0000044201 7
MIR153-2	7	157574464	15757486 4	S18_peak_3697	Intergenic between ENST00000385225 and ENST00000409610	ENST0000038522 5
MIR595	7	158355840	15835619	S18_peak_3698	Intergenic between	ENST0000038490

			3		ENST00000436489 and ENST00000384905	5
AC078942.1	7	158555552	158555717	S18_peak_3699	Intergenic between ENST00000448698 and ENST00000415652	ENST00000448698
AKR1A1	1	45566749	45566914	S18_peak_37	intron (ENST00000351829, intron 3 of 8)	ENST00000351829
OR51F2	11	4822975	4823140	S18_peak_370	TTS (ENST00000322110)	ENST00000322110
ESYT2	7	158775968	158776133	S18_peak_3700	intron (ENST00000251527, intron 6 of 21)	ENST00000251527
WDR60	7	158915563	158915763	S18_peak_3701	intron (ENST00000407559, intron 14 of 24)	ENST00000407559
AC144568.2	8	87002	87167	S18_peak_3702	Intergenic between ENST00000518652 and ENST00000520478	ENST00000518652
FBXO25	8	472085	472250	S18_peak_3703	exon (ENST00000382824, exon 11 of 11)	ENST00000382824
FBXO25	8	473262	473503	S18_peak_3704	exon (ENST00000382824, exon 11 of 11)	ENST00000382824
RP11-91J19.2	8	510020	510185	S18_peak_3705	Intergenic between ENST00000623988 and ENST00000262109	ENST00000623988
RP11-555E9.1	8	849400	849576	S18_peak_3706	Intergenic between ENST00000631653 and ENST00000633456	ENST00000633456
CTD-2281E23.3	8	1206371	1206572	S18_peak_3707	Intergenic between ENST00000634128 and ENST00000517950	ENST00000517950
KBTBD11-OT1	8	1806354	1806519	S18_peak_3708	intron (ENST00000635855, intron 2 of 29)	ENST00000635855
AC133633.2	8	2285946	2286122	S18_peak_3709	Intergenic between ENST00000619295 and ENST00000522799	ENST00000522799
TRIM22	11	5698243	5698519	S18_peak_371	exon (ENST00000379965, exon 4 of 8)	ENST00000379965
AC133633.2	8	2466197	2466362	S18_peak_3710	Intergenic between ENST00000619295 and ENST00000522799	ENST00000522799
RNA5SP251	8	3732844	3733009	S18_peak_3711	Intergenic between ENST00000410614 and ENST00000517543	ENST00000410614
RNA5SP251	8	4070423	4070588	S18_peak_3712	Intergenic between ENST00000410614 and ENST00000517543	ENST00000410614
PAICSP4	8	4882292	4882494	S18_peak_3713	Intergenic between ENST00000517543 and ENST00000518519	ENST00000517543
RP11-728L1.1	8	5829624	5829789	S18_peak_3714	Intergenic between ENST00000622736 and ENST00000520775	ENST00000520775
MCPH1	8	6466610	6466775	S18_peak_3715	intron (ENST00000344683, intron 9 of 13)	ENST00000344683
MCPH1	8	6484228	6484474	S18_peak_3716	intron (ENST00000344683, intron 11 of 13)	ENST00000344683
DEFB107B	8	7500093	7500505	S18_peak_3717	intron (ENST00000355602, intron 1 of 1)	ENST00000355602
FAM90A22P	8	7573790	7574043	S18_peak_3718	promoter-TSS	ENST0000040014

					(ENST00000400142)	2
FAM90A23P	8	7583220	7583525	S18_peak_3719	Intergenic between ENST00000400136 and ENST00000494376	ENST0000040013 6
OR56B4	11	6106137	6106371	S18_peak_372	Intergenic between ENST00000446117 and ENST00000316529	ENST0000031652 9
FAM90A11P	8	8017778	8017972	S18_peak_3720	Intergenic between ENST00000518378 and ENST00000520792	ENST0000051837 8
ENPP7P1	8	8166204	8166369	S18_peak_3722	intron (ENST00000509557, intron 1 of 2)	ENST0000050955 7
ENPP7P1	8	8191459	8191786	S18_peak_3723	intron (ENST00000509557, intron 1 of 2)	ENST0000050955 7
CTA-398F10.1	8	8335617	8335821	S18_peak_3724	Intergenic between ENST00000587202 and ENST00000517681	ENST0000051768 1
CTA-398F10.2	8	8443758	8443923	S18_peak_3725	Intergenic between ENST00000519814 and ENST00000521218	ENST0000052121 8
CLDN23	8	8650704	8650903	S18_peak_3726	Intergenic between ENST00000582930 and ENST00000519106	ENST0000051910 6
MSRA	8	10296819	10297140	S18_peak_3727	intron (ENST00000317173, intron 3 of 5)	ENST0000031717 3
DEFB134	8	11992873	11993038	S18_peak_3728	TTS (ENST00000526438)	ENST0000052643 8
ENPP7P6	8	12488796	12489333	S18_peak_3729	intron (ENST00000529817, intron 1 of 2)	ENST0000052981 7
RP11-290F24.3	11	6150566	6150870	S18_peak_373	intron (ENST00000529961, intron 1 of 2)	ENST0000052996 1
ENPP7P6	8	12489515	12489732	S18_peak_3730	intron (ENST00000529817, intron 1 of 2)	ENST0000052981 7
RP11-436P7.1	8	13689845	13690010	S18_peak_3731	Intergenic between ENST00000530718 and ENST00000532664	ENST0000053266 4
RP11-480O10.2	8	13898202	13898398	S18_peak_3732	Intergenic between ENST00000525673 and ENST00000526681	ENST0000052668 1
SGCZ	8	14807200	14807365	S18_peak_3733	intron (ENST00000382080, intron 1 of 7)	ENST0000038208 0
RP11-782K4.1	8	15354353	15354518	S18_peak_3734	Intergenic between ENST00000506833 and ENST00000382020	ENST0000050683 3
CSGALNACT1	8	19515346	19515563	S18_peak_3735	intron (ENST00000454498, intron 3 of 9)	ENST0000045449 8
DMTN	8	22053339	22053504	S18_peak_3736	Intergenic between ENST00000359441 and ENST00000443491	ENST0000044349 1
RP11-624C23.1	8	24361510	24361675	S18_peak_3737	intron (ENST00000518988, intron 1 of 3)	ENST0000051898 8
DOCK5	8	25302888	25303053	S18_peak_3738	intron (ENST00000276440, intron 10 of 51)	ENST0000027644 0
CDCA2	8	25481455	25481765	S18_peak_3739	intron (ENST00000330560, intron 8 of 14)	ENST0000033056 0
RRP8	11	6601342	6601507	S18_peak_374	exon (ENST00000254605, exon 3 of 7)	ENST0000025460 5

BNIP3L	8	26400736	26400949	S18_peak_3740	intron (ENST00000380629, intron 3 of 5)	ENST0000038062 9
DPYSL2	8	26525196	26525381	S18_peak_3741	intron (ENST00000521913, intron 1 of 13)	ENST0000052191 3
NRG1-IT3	8	32444300	32444465	S18_peak_3742	Intergenic between ENST00000519023 and ENST00000356819	ENST0000051902 3
NRG1	8	32567832	32568126	S18_peak_3743	intron (ENST00000356819, intron 1 of 11)	ENST0000035681 9
RP11-317N12.1	8	33718343	33718565	S18_peak_3744	intron (ENST00000523063, intron 2 of 5)	ENST0000052306 3
RP11-593P24.4	8	36394022	36394303	S18_peak_3745	Intergenic between ENST00000522004 and ENST00000524132	ENST0000052200 4
RP11-150O12.5	8	37563422	37563587	S18_peak_3746	intron (ENST00000522643, intron 1 of 2)	ENST0000052264 3
RP11-863K10.2	8	37722985	37723181	S18_peak_3747	intron (ENST00000523507, intron 1 of 2)	ENST0000052350 7
PLEKHA2	8	38877449	38877763	S18_peak_3748	Intergenic between ENST00000459965 and ENST00000617275	ENST0000061727 5
PLEKHA2	8	38962227	38962505	S18_peak_3749	intron (ENST00000617275, intron 10 of 11)	ENST0000061727 5
RP11-413N10.3	11	6847781	6847990	S18_peak_375	intron (ENST00000637205, intron 3 of 3)	ENST0000063720 5
SNORD112	8	42071996	42072161	S18_peak_3750	Intergenic between ENST00000459551 and ENST00000490411	ENST0000045955 1
IKBKB	8	42316636	42316898	S18_peak_3751	exon (ENST00000520810, exon 11 of 22)	ENST0000052081 0
VDAC3	8	42407097	42407326	S18_peak_3752	Intergenic between ENST00000022615 and ENST00000342228	ENST0000002261 5
RP11-643N23.1	8	43773061	43773283	S18_peak_3753	Intergenic between ENST00000519417 and ENST00000618609	ENST0000051941 7
SPIDR	8	47308554	47308822	S18_peak_3754	intron (ENST00000297423, intron 5 of 19)	ENST0000029742 3
SPIDR	8	47315681	47315881	S18_peak_3755	intron (ENST00000297423, intron 5 of 19)	ENST0000029742 3
SPIDR	8	47350406	47350571	S18_peak_3756	intron (ENST00000297423, intron 5 of 19)	ENST0000029742 3
SPIDR	8	47715503	47715668	S18_peak_3757	intron (ENST00000297423, intron 16 of 19)	ENST0000029742 3
RP11-769N21.2	8	48240793	48241072	S18_peak_3758	Intergenic between ENST00000519711 and ENST00000520250	ENST0000051971 1
EFCAB1	8	48806085	48806250	S18_peak_3759	Intergenic between ENST00000262103 and ENST00000522657	ENST0000026210 3
RP11-413N10.3	11	6910763	6910973	S18_peak_376	intron (ENST00000637205, intron 2 of 3)	ENST0000063720 5
RP11-22C8.1	8	48878983	48879268	S18_peak_3760	Intergenic between ENST00000262103 and ENST00000522657	ENST0000052265 7
SNORA7	8	51070646	51070980	S18_peak_3761	Intergenic between ENST00000364446 and ENST00000521294	ENST0000036444 6

PCMTD1	8	51871388	51871553	S18_peak_3762	intron (ENST00000519559, intron 1 of 5)	ENST0000051955 9
PCMTD1	8	51882042	51882207	S18_peak_3763	intron (ENST00000519559, intron 1 of 5)	ENST0000051955 9
ST18	8	52328854	52329098	S18_peak_3764	intron (ENST00000276480, intron 2 of 25)	ENST0000027648 0
RB1CC1	8	52715812	52715977	S18_peak_3765	Intergenic between ENST00000025008 and ENST00000605049	ENST0000002500 8
RP11-550I15.1	8	54752570	54752753	S18_peak_3766	Intergenic between ENST00000520784 and ENST00000522186	ENST0000052078 4
RP11-628E19.2	8	55585802	55585967	S18_peak_3767	Intergenic between ENST00000522918 and ENST00000334667	ENST0000052291 8
LYN	8	55884599	55884908	S18_peak_3768	intron (ENST00000519728, intron 1 of 12)	ENST0000051972 8
LYN	8	55974873	55975071	S18_peak_3769	intron (ENST00000519728, intron 10 of 12)	ENST0000051972 8
NLRP14	11	7047318	7047483	S18_peak_377	intron (ENST00000299481, intron 5 of 11)	ENST0000029948 1
RP11-17A4.2	8	56464374	56464578	S18_peak_3770	intron (ENST00000518662, intron 1 of 3)	ENST0000051866 2
LINC01606	8	57205805	57205970	S18_peak_3771	intron (ENST00000519241, intron 3 of 3)	ENST0000051924 1
LINC01606	8	57210266	57210451	S18_peak_3772	intron (ENST00000519241, intron 3 of 3)	ENST0000051924 1
RP11-246K15.1	8	57584453	57584618	S18_peak_3773	intron (ENST00000520881, intron 1 of 1)	ENST0000052088 1
NSMAF	8	58611463	58611675	S18_peak_3774	intron (ENST00000038176, intron 9 of 30)	ENST0000003817 6
RP11-379I19.1	8	59575224	59575389	S18_peak_3775	intron (ENST00000521456, intron 1 of 1)	ENST0000052145 6
RP11-27P7.1	8	60017580	60017745	S18_peak_3776	Intergenic between ENST00000615967 and ENST00000531077	ENST0000053107 7
NKAIN3	8	62835829	62836109	S18_peak_3777	intron (ENST00000519049, intron 5 of 7)	ENST0000051904 9
RP11-32K4.1	8	64235244	64235409	S18_peak_3778	intron (ENST00000523191, intron 1 of 5)	ENST0000052319 1
CPA6	8	67437994	67438311	S18_peak_3779	intron (ENST00000297770, intron 8 of 10)	ENST0000029777 0
SYT9	11	7256090	7256318	S18_peak_378	intron (ENST00000318881, intron 1 of 6)	ENST0000031888 1
NDUFS5P6	8	67837378	67837543	S18_peak_3780	Intergenic between ENST00000526382 and ENST00000529136	ENST0000052913 6
NDUFS5P6	8	67888138	67888303	S18_peak_3781	Intergenic between ENST00000529136 and ENST00000288368	ENST0000052913 6
GS1-44D20.1	8	69139798	69140051	S18_peak_3782	Intergenic between ENST00000520239 and ENST00000520780	ENST0000052023 9
LINC01603	8	69384987	69385188	S18_peak_3783	Intergenic between ENST00000520780 and ENST00000517343	ENST0000051734 3
LINC01603	8	69433695	69433860	S18_peak_3784	intron (ENST00000517343, intron 1 of 2)	ENST0000051734 3

NCOA2	8	70310048	70310213	S18_peak_3785	intron (ENST00000452400, intron 1 of 22)	ENST00000452400
RP11-351E7.2	8	72437197	72437476	S18_peak_3786	Intergenic between ENST00000530034 and ENST00000521224	ENST00000530034
RP11-531A24.3	8	72945514	72945679	S18_peak_3787	Intergenic between ENST00000523881 and ENST00000564832	ENST00000564832
STAU2	8	73642823	73642988	S18_peak_3788	intron (ENST00000524300, intron 6 of 14)	ENST00000524300
RP11-38H17.1	8	77759461	77759626	S18_peak_3789	Intergenic between ENST00000524014 and ENST00000521879	ENST00000524014
CTD-2516F10.2	11	7479449	7479762	S18_peak_379	intron (ENST00000622955, intron 1 of 7)	ENST00000622955
PKIA	8	78581056	78581273	S18_peak_3790	intron (ENST00000396418, intron 2 of 3)	ENST00000396418
RP11-594N15.3	8	78635575	78635740	S18_peak_3791	Intergenic between ENST00000565862 and ENST00000263849	ENST00000565862
RP11-79H23.3	8	78908273	78908450	S18_peak_3792	Intergenic between ENST00000565297 and ENST00000519983	ENST00000565297
RP11-1114I9.1	8	79405088	79405362	S18_peak_3793	Intergenic between ENST00000519983 and ENST00000522035	ENST00000519983
RP11-758H6.1	8	79444016	79444195	S18_peak_3794	Intergenic between ENST00000519983 and ENST00000522035	ENST00000522035
MIR5708	8	80239833	80239998	S18_peak_3795	Intergenic between ENST00000476144 and ENST00000579723	ENST00000579723
RP11-775E10.1	8	80359876	80360041	S18_peak_3796	Intergenic between ENST00000520293 and ENST00000517075	ENST00000520293
RNU6-1213P	8	80403929	80404220	S18_peak_3797	Intergenic between ENST00000520293 and ENST00000517075	ENST00000517075
PAG1	8	81012446	81012611	S18_peak_3798	intron (ENST00000220597, intron 3 of 8)	ENST00000220597
FABP5	8	81287004	81287193	S18_peak_3799	Intergenic between ENST00000297258 and ENST00000524085	ENST00000297258
RPS15AP10	1	45641608	45641773	S18_peak_38	Intergenic between ENST00000445048 and ENST00000432472	ENST00000432472
CTD-2516F10.2	11	7505682	7505847	S18_peak_380	intron (ENST00000530201, intron 1 of 1)	ENST00000530201
RP11-157I4.4	8	81474323	81474598	S18_peak_3800	intron (ENST00000524085, intron 2 of 3)	ENST00000524085
HNRNPA1P4	8	82259951	82260116	S18_peak_3801	Intergenic between ENST00000520988 and ENST00000509706	ENST00000509706
LINC01419	8	83578866	83579046	S18_peak_3802	Intergenic between ENST00000522365 and ENST00000523678	ENST00000522365
RP11-120I21.3	8	84310706	84310949	S18_peak_3803	Intergenic between ENST00000518452 and	ENST00000518452

					ENST00000383974	
RP11-120I21.3	8	84360722	84360896	S18_peak_3804	Intergenic between ENST00000518452 and ENST00000383974	ENST0000051845 2
RNU6-1040P	8	84490203	84490368	S18_peak_3805	Intergenic between ENST00000518452 and ENST00000383974	ENST0000038397 4
RNU6-1040P	8	84523445	84523644	S18_peak_3806	Intergenic between ENST00000518452 and ENST00000383974	ENST0000038397 4
ACTBP6	8	84942814	84943085	S18_peak_3807	Intergenic between ENST00000521595 and ENST00000520036	ENST0000052003 6
RP11-146E23.2	8	85089030	85089485	S18_peak_3808	Intergenic between ENST00000522168 and ENST00000360375	ENST0000052216 8
CA1	8	85377991	85378236	S18_peak_3809	promoter-TSS (ENST00000523953)	ENST0000052395 3
PPFIBP2	11	7515539	7515704	S18_peak_381	promoter-TSS (ENST00000299492)	ENST0000029949 2
REXO1L10P	8	85752835	85753003	S18_peak_3810	Intergenic between ENST00000615707 and ENST00000604264	ENST0000061570 7
REXO1L9P	8	85765220	85765388	S18_peak_3811	promoter-TSS (ENST00000604264)	ENST0000060426 4
REXO1L4P	8	85800052	85800251	S18_peak_3812	Intergenic between ENST00000540724 and ENST00000605109	ENST0000060510 9
REXO1L5P	8	85812256	85812540	S18_peak_3815	Intergenic between ENST00000605109 and ENST00000622260	ENST0000062226 0
REXO1L5P	8	85813653	85813875	S18_peak_3816	Intergenic between ENST00000605109 and ENST00000622260	ENST0000062226 0
ATP6V0D2	8	86146432	86146597	S18_peak_3818	intron (ENST00000285393, intron 5 of 7)	ENST0000028539 3
CTD-2284J15.1	8	86393354	86393547	S18_peak_3819	Intergenic between ENST00000523218 and ENST00000406452	ENST0000052321 8
PPFIBP2	11	7558472	7558637	S18_peak_382	intron (ENST00000299492, intron 2 of 23)	ENST0000029949 2
CNGB3	8	86609880	86610195	S18_peak_3820	intron (ENST00000320005, intron 14 of 17)	ENST0000032000 5
RP11-386D6.3	8	86829888	86830146	S18_peak_3821	Intergenic between ENST00000519251 and ENST00000440763	ENST0000051925 1
RP11-386D6.3	8	86960656	86960909	S18_peak_3822	Intergenic between ENST00000519251 and ENST00000440763	ENST0000051925 1
AF121898.3	8	87535410	87535575	S18_peak_3823	Intergenic between ENST00000519251 and ENST00000440763	ENST0000044076 3
CTB-118P15.3	8	87869211	87869475	S18_peak_3824	TTS (ENST00000613582)	ENST0000061358 2
DCAF4L2	8	87900816	87901025	S18_peak_3825	Intergenic between ENST00000319675 and ENST00000518129	ENST0000031967 5

RP11-586K2.1	8	88607565	88607741	S18_peak_3826	intron (ENST00000521433, intron 3 of 4)	ENST0000052143 3
RP11-642C5.1	8	88823610	88823775	S18_peak_3827	Intergenic between ENST00000604955 and ENST00000522047	ENST0000060495 5
RPSAP74	8	89209376	89209580	S18_peak_3828	Intergenic between ENST00000604955 and ENST00000522047	ENST0000052204 7
RNU6-925P	8	89874285	89874597	S18_peak_3829	Intergenic between ENST00000619538 and ENST00000384629	ENST0000038462 9
PPFIBP2	11	7581888	7582104	S18_peak_383	intron (ENST00000299492, intron 3 of 23)	ENST0000029949 2
DECR1	8	90016454	90016619	S18_peak_3830	intron (ENST00000220764, intron 1 of 9)	ENST0000022076 4
LINC00534	8	90178703	90178871	S18_peak_3831	Intergenic between ENST00000265431 and ENST00000517400	ENST0000051740 0
AB015752.3	8	90586728	90586894	S18_peak_3832	intron (ENST00000521975, intron 2 of 4)	ENST0000052197 5
SLC26A7	8	91427776	91427965	S18_peak_3833	Intergenic between ENST00000309536 and ENST00000521199	ENST0000030953 6
RP11-122C21.1	8	91651711	91651980	S18_peak_3834	intron (ENST00000521199, intron 2 of 3)	ENST0000052119 9
MRPS16P1	8	91927712	91927877	S18_peak_3835	Intergenic between ENST00000523462 and ENST00000523629	ENST0000052346 2
RUNX1T1	8	92063626	92063791	S18_peak_3836	intron (ENST00000523629, intron 2 of 11)	ENST0000052362 9
RP11-587H10.1	8	92489897	92490062	S18_peak_3837	Intergenic between ENST00000523385 and ENST00000521955	ENST0000052195 5
RP11-587H10.2	8	92552744	92552909	S18_peak_3838	Intergenic between ENST00000521955 and ENST00000523284	ENST0000052328 4
LINC00535	8	93392678	93392908	S18_peak_3839	intron (ENST00000520096, intron 1 of 5)	ENST0000052009 6
TUB	11	8052693	8052858	S18_peak_384	intron (ENST00000305253, intron 2 of 12)	ENST0000030525 3
LINC00535	8	93677703	93677974	S18_peak_3840	intron (ENST00000501400, intron 3 of 10)	ENST0000050140 0
RP11-328L11.1	8	95415989	95416154	S18_peak_3841	Intergenic between ENST00000523014 and ENST00000519366	ENST0000052301 4
RNU6-1172P	8	96452616	96452812	S18_peak_3842	Intergenic between ENST00000365225 and ENST00000302190	ENST0000036522 5
CPQ	8	96652667	96652894	S18_peak_3843	intron (ENST00000220763, intron 1 of 7)	ENST0000022076 3
CPQ	8	96766824	96767013	S18_peak_3844	intron (ENST00000220763, intron 1 of 7)	ENST0000022076 3
CPQ	8	96781891	96782056	S18_peak_3845	intron (ENST00000220763, intron 1 of 7)	ENST0000022076 3
CPQ	8	97042115	97042280	S18_peak_3846	intron (ENST00000220763, intron 6 of 7)	ENST0000022076 3
U3	8	97381808	97382056	S18_peak_3847	Intergenic between ENST00000384486 and	ENST0000038448 6

					ENST00000492365	
KB-1683C8.1	8	97558585	97558750	S18_peak_3848	Intergenic between ENST00000384486 and ENST00000492365	ENST00000492365
MTDH	8	97649737	97649980	S18_peak_3849	intron (ENST00000336273, intron 1 of 11)	ENST00000336273
STK33	11	8558365	8558617	S18_peak_385	intron (ENST00000315204, intron 1 of 13)	ENST00000315204
RPL30	8	98031700	98031865	S18_peak_3850	Intergenic between ENST00000491662 and ENST00000287038	ENST00000287038
STK3	8	98452633	98452849	S18_peak_3851	Intergenic between ENST00000605923 and ENST00000419617	ENST00000419617
RP11-410L14.2	8	99010001	99010166	S18_peak_3852	intron (ENST00000521696, intron 1 of 1)	ENST00000521696
VPS13B	8	99630106	99630271	S18_peak_3853	intron (ENST00000358544, intron 33 of 61)	ENST00000358544
RGS22	8	100076711	100076876	S18_peak_3854	intron (ENST00000360863, intron 4 of 27)	ENST00000360863
KB-173C10.2	8	100387186	100387402	S18_peak_3855	intron (ENST00000519844, intron 2 of 2)	ENST00000519844
Y_RNA	8	100759500	100759678	S18_peak_3856	Intergenic between ENST00000365131 and ENST00000461683	ENST00000365131
RNU4-83P	8	100859708	100859976	S18_peak_3857	Intergenic between ENST00000410863 and ENST00000475156	ENST00000410863
KB-1396H2.2	8	101875308	101875557	S18_peak_3858	Intergenic between ENST00000635554 and ENST00000577577	ENST00000635554
KB-1396H2.2	8	101907816	101908066	S18_peak_3859	Intergenic between ENST00000635554 and ENST00000577577	ENST00000635554
RP11-152H18.4	11	8623390	8623580	S18_peak_386	Intergenic between ENST00000516969 and ENST00000534169	ENST00000534169
KB-1980E6.2	8	102417048	102417213	S18_peak_3860	Intergenic between ENST00000606361 and ENST00000517692	ENST00000517692
BAALC	8	103215593	103215780	S18_peak_3861	intron (ENST00000309982, intron 2 of 2)	ENST00000309982
FZD6	8	103335180	103335420	S18_peak_3862	Intergenic between ENST00000523739 and ENST00000330295	ENST00000523739
CTHRC1	8	103355753	103356056	S18_peak_3863	Intergenic between ENST00000523739 and ENST00000330295	ENST00000330295
SLC25A32	8	103410416	103410607	S18_peak_3864	intron (ENST00000297578, intron 1 of 6)	ENST00000297578
RP11-200A13.1	8	104880880	104881079	S18_peak_3865	Intergenic between ENST00000636802 and ENST00000483263	ENST00000483263
ZFPM2	8	105620932	105621160	S18_peak_3866	intron (ENST00000407775, intron 4 of 7)	ENST00000407775
ZFPM2	8	105670853	105671018	S18_peak_3867	intron (ENST00000407775, intron 5 of 7)	ENST00000407775

OXR1	8	106604830	106605086	S18_peak_3868	intron (ENST00000442977, intron 2 of 15)	ENST00000442977
HMGB1P46	8	107143580	107143755	S18_peak_3869	Intergenic between ENST00000617737 and ENST00000520062	ENST00000520062
ST5	11	8721249	8721455	S18_peak_387	intron (ENST00000526757, intron 3 of 18)	ENST00000526757
ANGPT1	8	107275439	107275680	S18_peak_3870	intron (ENST00000517746, intron 7 of 8)	ENST00000517746
RNA5SP275	8	107783741	107783906	S18_peak_3871	Intergenic between ENST00000507497 and ENST00000363936	ENST00000363936
RSPO2	8	108022564	108022734	S18_peak_3872	intron (ENST00000517781, intron 2 of 4)	ENST00000517781
EIF3E	8	108180740	108180905	S18_peak_3873	Intergenic between ENST00000413008 and ENST00000220849	ENST00000220849
RPS17P14	8	108382367	108382532	S18_peak_3874	Intergenic between ENST00000382740 and ENST00000220853	ENST00000382740
RP11-25P11.2	8	108545724	108545940	S18_peak_3875	Intergenic between ENST00000220853 and ENST00000520765	ENST00000520765
TMEM74	8	108783196	108783365	S18_peak_3876	TTS (ENST00000297459)	ENST00000297459
PKHD1L1	8	109454235	109454400	S18_peak_3877	intron (ENST00000378402, intron 44 of 77)	ENST00000378402
CTD-2544L4.1	8	110471297	110471471	S18_peak_3878	Intergenic between ENST00000522896 and ENST00000523800	ENST00000523800
RP11-238I10.1	8	110692280	110692460	S18_peak_3879	Intergenic between ENST00000523264 and ENST00000520749	ENST00000523264
TMEM9B	11	8953676	8953841	S18_peak_388	intron (ENST00000534025, intron 3 of 4)	ENST00000534025
NDUFB9P3	8	110837944	110838109	S18_peak_3880	Intergenic between ENST00000521269 and ENST00000523889	ENST00000521269
LINC01608	8	111043917	111044088	S18_peak_3881	Intergenic between ENST00000523557 and ENST00000519506	ENST00000523557
CSMD3	8	112507569	112507734	S18_peak_3882	intron (ENST00000343508, intron 29 of 71)	ENST00000343508
CSMD3	8	112987622	112987905	S18_peak_3883	intron (ENST00000343508, intron 7 of 71)	ENST00000343508
CSMD3	8	113002900	113003104	S18_peak_3884	intron (ENST00000343508, intron 7 of 71)	ENST00000343508
CSMD3	8	113271187	113271352	S18_peak_3885	intron (ENST00000343508, intron 4 of 71)	ENST00000343508
Y_RNA	8	113802826	113803029	S18_peak_3886	Intergenic between ENST00000518558 and ENST00000383992	ENST00000383992
Y_RNA	8	114008544	114008818	S18_peak_3887	Intergenic between ENST00000383992 and ENST00000521523	ENST00000383992
CARSP2	8	114558081	114558246	S18_peak_3888	Intergenic between ENST00000518851 and ENST00000520688	ENST00000520688

CARSP2	8	114559359	114559586	S18_peak_3889	Intergenic between ENST00000518851 and ENST00000520688	ENST00000520688
DENND5A	11	9171585	9171817	S18_peak_389	intron (ENST00000328194, intron 8 of 22)	ENST00000328194
TRPS1	8	115120798	115120963	S18_peak_3890	Intergenic between ENST00000520688 and ENST00000395715	ENST00000395715
AF178030.2	8	115679905	115680146	S18_peak_3891	Intergenic between ENST00000415088 and ENST00000505156	ENST00000415088
RP11-536K17.1	8	116365017	116365232	S18_peak_3892	Intergenic between ENST00000362580 and ENST00000523110	ENST00000523110
EIF3H	8	116585551	116585716	S18_peak_3893	Intergenic between ENST00000523110 and ENST00000521861	ENST00000521861
EIF3H	8	116608979	116609188	S18_peak_3894	Intergenic between ENST00000523110 and ENST00000521861	ENST00000521861
RAD21	8	116856597	116856762	S18_peak_3895	exon (ENST00000297338, exon 7 of 14)	ENST00000297338
AARD	8	116938024	116938189	S18_peak_3896	Intergenic between ENST00000521487 and ENST00000378279	ENST00000378279
SLC30A8	8	117079060	117079225	S18_peak_3897	intron (ENST00000521243, intron 2 of 9)	ENST00000521243
EXT1	8	117686162	117686390	S18_peak_3898	Intergenic between ENST00000483812 and ENST00000378204	ENST00000378204
EXT1	8	117921283	117921566	S18_peak_3899	intron (ENST00000378204, intron 1 of 10)	ENST00000378204
IPP	1	45704965	45705276	S18_peak_39	intron (ENST00000359942, intron 8 of 9)	ENST00000359942
Metazoa_SRP	11	9268803	9269092	S18_peak_390	Intergenic between ENST00000528520 and ENST00000613468	ENST00000613468
COLEC10	8	119106031	119106247	S18_peak_3900	exon (ENST00000332843, exon 6 of 6)	ENST00000332843
COLEC10	8	119129368	119129533	S18_peak_3901	Intergenic between ENST00000332843 and ENST00000614891	ENST00000332843
DSCC1	8	119837680	119837903	S18_peak_3902	intron (ENST00000313655, intron 8 of 8)	ENST00000313655
KB-1471A8.1	8	119871212	119871377	S18_peak_3903	intron (ENST00000500705, intron 1 of 1)	ENST00000500705
DEPTOR	8	120041260	120041499	S18_peak_3904	intron (ENST00000286234, intron 8 of 8)	ENST00000286234
MRPL13	8	120388002	120388200	S18_peak_3905	Intergenic between ENST00000297848 and ENST00000306185	ENST00000306185
SNTB1	8	120572637	120572888	S18_peak_3906	intron (ENST00000517992, intron 4 of 6)	ENST00000517992
SNTB1	8	120781932	120782097	S18_peak_3907	intron (ENST00000517992, intron 1 of 6)	ENST00000517992
RP11-369K17.1	8	120944809	120945129	S18_peak_3908	intron (ENST00000517739, intron 1 of 3)	ENST00000517739
RP11-369K17.2	8	121181286	12118150	S18_peak_3909	Intergenic between	ENST0000060471

			8		ENST00000604710 and ENST00000469425	0
IPO7	11	9396274	9396566	S18_peak_391	intron (ENST00000379719, intron 1 of 24)	ENST0000037971 9
RPL35AP19	8	121254845	12125501 0	S18_peak_3910	Intergenic between ENST00000604710 and ENST00000469425	ENST0000046942 5
RPL35AP19	8	121421012	12142117 7	S18_peak_3911	Intergenic between ENST00000469425 and ENST00000303924	ENST0000046942 5
RP11-398G24.2	8	121987104	12198729 1	S18_peak_3912	intron (ENST00000523792, intron 1 of 2)	ENST0000052379 2
MRPS36P3	8	122243779	12224397 1	S18_peak_3913	Intergenic between ENST00000519284 and ENST00000533992	ENST0000051928 4
RP11-973F15.2	8	122735003	12273520 3	S18_peak_3914	Intergenic between ENST00000577765 and ENST00000442838	ENST0000057776 5
TBC1D31	8	123150308	12315048 0	S18_peak_3915	intron (ENST00000287380, intron 21 of 21)	ENST0000028738 0
RP11-174I12.2	8	123557437	12355760 2	S18_peak_3916	Intergenic between ENST00000443022 and ENST00000518970	ENST0000051897 0
FER1L6	8	123903759	12390392 4	S18_peak_3917	intron (ENST00000522917, intron 1 of 40)	ENST0000052291 7
FER1L6-AS2	8	124163212	12416349 5	S18_peak_3918	intron (ENST00000520031, intron 1 of 4)	ENST0000052003 1
LINC00964	8	124912132	12491229 7	S18_peak_3919	intron (ENST00000528090, intron 3 of 5)	ENST0000052809 0
RPL23AP65	11	9601859	9602024	S18_peak_392	Intergenic between ENST00000458785 and ENST00000475608	ENST0000047560 8
KIAA0196	8	125023315	12502357 9	S18_peak_3920	TTS (ENST00000318410)	ENST0000031841 0
KIAA0196	8	125031906	12503207 1	S18_peak_3921	intron (ENST00000318410, intron 27 of 28)	ENST0000031841 0
KIAA0196	8	125037686	12503791 4	S18_peak_3922	intron (ENST00000318410, intron 25 of 28)	ENST0000031841 0
NSMCE2	8	125093597	12509382 9	S18_peak_3923	promoter-TSS (ENST00000287437)	ENST0000028743 7
NSMCE2	8	125250007	12525017 2	S18_peak_3924	intron (ENST00000287437, intron 5 of 7)	ENST0000028743 7
RP11-136O12.2	8	125497876	12549804 1	S18_peak_3925	intron (ENST00000522815, intron 2 of 3)	ENST0000052281 5
RP11-697B24.1	8	125749385	12574955 3	S18_peak_3926	promoter-TSS (ENST00000518339)	ENST0000051833 9
LINC00861	8	125938896	12593915 4	S18_peak_3927	intron (ENST00000522865, intron 1 of 2)	ENST0000052286 5
RP11-622O11.4	8	126000696	12600094 6	S18_peak_3928	promoter-TSS (ENST00000523525)	ENST0000052352 5
RP11-89K10.1	8	126597266	12659749 3	S18_peak_3929	intron (ENST00000519880, intron 2 of 4)	ENST0000051988 0
SBF2-AS1	11	9781007	9781206	S18_peak_393	intron (ENST00000498905, intron 1 of 2)	ENST0000049890 5
RP11-351C8.1	8	126766521	12676681 2	S18_peak_3930	TTS (ENST00000520224)	ENST0000052022 4
PCAT1	8	126973021	12697327 5	S18_peak_3931	intron (ENST00000519319, intron 3 of 4)	ENST0000051931 9

PCAT1	8	126981017	126981182	S18_peak_3932	intron (ENST00000519319, intron 3 of 4)	ENST00000519319
MIR1207	8	128033336	128033628	S18_peak_3933	Intergenic between ENST00000410569 and ENST00000408249	ENST00000408249
MIR1207	8	128056410	128056575	S18_peak_3934	Intergenic between ENST00000408249 and ENST00000623475	ENST00000408249
RN7SKP226	8	128190705	128190920	S18_peak_3935	Intergenic between ENST00000408334 and ENST00000364912	ENST00000364912
LINC00976	8	128761697	128761935	S18_peak_3936	Intergenic between ENST00000518044 and ENST00000630386	ENST00000630386
LINC00976	8	128805126	128805291	S18_peak_3937	Intergenic between ENST00000518044 and ENST00000630386	ENST00000630386
LINC00977	8	129104004	129104169	S18_peak_3938	Intergenic between ENST00000625513 and ENST00000509893	ENST00000509893
LINC00977	8	129122885	129123113	S18_peak_3939	Intergenic between ENST00000625513 and ENST00000509893	ENST00000509893
SBF2	11	9996466	9996686	S18_peak_394	intron (ENST00000256190, intron 9 of 39)	ENST00000256190
FAM49B	8	129917476	129917689	S18_peak_3940	intron (ENST00000519824, intron 1 of 11)	ENST00000519824
ASAP1	8	130045020	130045185	S18_peak_3941	Intergenic between ENST00000582634 and ENST00000518721	ENST00000518721
ASAP1	8	130057434	130057738	S18_peak_3942	intron (ENST00000518721, intron 29 of 29)	ENST00000518721
ASAP1	8	130159425	130159727	S18_peak_3943	intron (ENST00000518721, intron 12 of 29)	ENST00000518721
KB-1568E2.1	8	130638938	130639103	S18_peak_3944	intron (ENST00000523502, intron 1 of 2)	ENST00000523502
KB-1568E2.1	8	130658323	130658488	S18_peak_3945	Intergenic between ENST00000523502 and ENST00000286355	ENST00000523502
ADCY8	8	130805522	130805687	S18_peak_3946	intron (ENST00000286355, intron 14 of 17)	ENST00000286355
CTD-2501M5.1	8	131338619	131338867	S18_peak_3947	Intergenic between ENST00000519005 and ENST00000516349	ENST00000519005
CTD-2008O4.1	8	131746614	131746812	S18_peak_3948	Intergenic between ENST00000521318 and ENST00000254624	ENST00000521318
TMEM71	8	132705554	132705719	S18_peak_3949	Intergenic between ENST00000520457 and ENST00000377901	ENST00000377901
SBF2	11	10285234	10285473	S18_peak_395	intron (ENST00000256190, intron 1 of 39)	ENST00000256190
TG	8	132875266	132875454	S18_peak_3950	intron (ENST00000220616, intron 5 of 47)	ENST00000220616
NDRG1	8	133305419	133305611	S18_peak_3951	Intergenic between ENST00000522476 and ENST00000479066	ENST00000522476
ST13P6	8	133422461	13342269	S18_peak_3952	Intergenic between	ENST0000052243

			7		ENST00000522430 and ENST00000638080	0
Metazoa_SRP	8	133662719	133663014	S18_peak_3953	Intergenic between ENST00000390971 and ENST00000611670	ENST00000611670
ZFAT	8	134576257	134576422	S18_peak_3954	intron (ENST00000520356, intron 10 of 13)	ENST00000520356
RPL23AP56	8	134971308	134971473	S18_peak_3955	Intergenic between ENST00000505083 and ENST00000460778	ENST00000460778
LINC01591	8	135226140	135226318	S18_peak_3956	Intergenic between ENST00000460778 and ENST00000522279	ENST00000522279
RP11-30J20.1	8	136543617	136543782	S18_peak_3957	intron (ENST00000524346, intron 2 of 5)	ENST00000524346
RP11-30J20.1	8	136744270	136744435	S18_peak_3958	intron (ENST00000524346, intron 2 of 5)	ENST00000524346
RP11-30J20.1	8	136752109	136752440	S18_peak_3959	intron (ENST00000524346, intron 2 of 5)	ENST00000524346
Y_RNA	11	10728576	10728769	S18_peak_396	Intergenic between ENST00000384131 and ENST00000361367	ENST00000384131
RP11-30J20.1	8	136816071	136816342	S18_peak_3960	intron (ENST00000524346, intron 4 of 5)	ENST00000524346
RP11-30J20.1	8	136935475	136935793	S18_peak_3961	intron (ENST00000521034, intron 2 of 3)	ENST00000521034
RNU6-144P	8	137051348	137051539	S18_peak_3962	Intergenic between ENST00000521034 and ENST00000383951	ENST00000383951
FAM135B	8	138160514	138160679	S18_peak_3963	intron (ENST00000395297, intron 12 of 19)	ENST00000395297
COL22A1	8	138656012	138656323	S18_peak_3964	intron (ENST00000303045, intron 44 of 64)	ENST00000303045
COL22A1	8	138948465	138948692	S18_peak_3965	Intergenic between ENST00000303045 and ENST00000519306	ENST00000303045
RP11-324F11.1	8	139070644	139070817	S18_peak_3966	Intergenic between ENST00000303045 and ENST00000519306	ENST00000519306
TRAPPC9	8	140275295	140275467	S18_peak_3967	intron (ENST00000389328, intron 15 of 22)	ENST00000389328
AGO2	8	140544049	140544323	S18_peak_3968	intron (ENST00000220592, intron 14 of 18)	ENST00000220592
RP11-10J21.5	8	141273287	141273452	S18_peak_3969	Intergenic between ENST00000518520 and ENST00000520606	ENST00000520606
GALNT18	11	11372668	11372833	S18_peak_397	intron (ENST00000227756, intron 5 of 10)	ENST00000227756
RP11-10J21.6	8	141328467	141328632	S18_peak_3970	Intergenic between ENST00000523604 and ENST00000521073	ENST00000523604
GPR20	8	141374732	141374897	S18_peak_3971	Intergenic between ENST00000377741 and ENST00000501440	ENST00000377741
AC138647.1	8	141546996	141547161	S18_peak_3972	Intergenic between ENST00000427937 and ENST00000408841	ENST00000427937
AC138647.1	8	141636137	14163630	S18_peak_3973	Intergenic between	ENST0000042793

			5		ENST00000427937 and ENST00000408841	7
MIR1302-7	8	141797243	141797408	S18_peak_3974	Intergenic between ENST00000408841 and ENST00000523974	ENST00000408841
RP11-953B20.2	8	141934758	141934938	S18_peak_3975	Intergenic between ENST00000408841 and ENST00000523974	ENST00000523974
RN7SL260P	8	142260222	142260387	S18_peak_3976	Intergenic between ENST00000521103 and ENST00000569285	ENST00000521103
MROH4P	8	142569974	142570245	S18_peak_3977	intron (ENST00000431302, intron 10 of 11)	ENST00000431302
RP11-706C16.13	8	142814343	142814508	S18_peak_3978	Intergenic between ENST00000637396 and ENST00000519411	ENST00000637396
RP11-520P18.1	8	143063718	143063883	S18_peak_3979	Intergenic between ENST00000507976 and ENST00000485679	ENST00000485679
GALNT18	11	11521301	11521520	S18_peak_398	intron (ENST00000227756, intron 1 of 10)	ENST00000227756
RP13-582O9.6	8	143269065	143269326	S18_peak_3980	intron (ENST00000522452, intron 2 of 3)	ENST00000522452
TOP1MT	8	143324244	143324493	S18_peak_3981	intron (ENST00000329245, intron 6 of 13)	ENST00000329245
RP11-909N17.2	8	143413641	143413806	S18_peak_3982	promoter-TSS (ENST00000523002)	ENST00000523002
ZC3H3	8	143471411	143471576	S18_peak_3983	intron (ENST00000262577, intron 5 of 11)	ENST00000262577
PYCRL	8	143609070	143609237	S18_peak_3984	promoter-TSS (ENST00000220966)	ENST00000220966
FAM83H-AS1	8	143746811	143747017	S18_peak_3985	intron (ENST00000527139, intron 3 of 13)	ENST00000527139
WDR97	8	144125678	144126004	S18_peak_3986	Intergenic between ENST00000323662 and ENST00000347708	ENST00000323662
MROH1	8	144217613	144217778	S18_peak_3987	intron (ENST00000534366, intron 11 of 41)	ENST00000534366
MROH1	8	144233424	144233594	S18_peak_3988	intron (ENST00000534366, intron 13 of 41)	ENST00000534366
DGAT1	8	144317468	144317633	S18_peak_3989	exon (ENST00000528718, exon 12 of 17)	ENST00000528718
GALNT18	11	11525708	11525873	S18_peak_399	intron (ENST00000227756, intron 1 of 10)	ENST00000227756
LRRC14	8	144521224	144521389	S18_peak_3990	exon (ENST00000529022, exon 5 of 5)	ENST00000529022
ARHGAP39	8	144547373	144547538	S18_peak_3991	exon (ENST00000377307, exon 4 of 11)	ENST00000377307
AF186192.6	8	144713306	144713637	S18_peak_3992	Intergenic between ENST00000529026 and ENST00000583427	ENST00000583427
CBWD1	9	128123	128288	S18_peak_3993	intron (ENST00000356521, intron 11 of 14)	ENST00000356521
RP11-20A20.2	9	24594798	24594963	S18_peak_3994	Intergenic between ENST00000602614 and ENST00000411156	ENST00000602614
TUSC1	9	25422598	25422772	S18_peak_3995	Intergenic between ENST00000410761 and	ENST00000358022

					ENST00000358022	
LINC01241	9	25816081	25816388	S18_peak_3996	Intergenic between ENST00000627303 and ENST00000604431	ENST00000627303
CAAP1	9	26892394	26892584	S18_peak_3997	promoter-TSS (ENST00000333916)	ENST00000333916
RP11-20P5.2	9	28113121	28113286	S18_peak_3998	Intergenic between ENST00000566293 and ENST00000478520	ENST00000478520
LINC01242	9	30378199	30378364	S18_peak_3999	Intergenic between ENST00000426028 and ENST00000629369	ENST00000629369
RP11-334N17.1	1	6968860	6969072	S18_peak_4	Intergenic between ENST00000433407 and ENST00000456701	ENST00000456701
TRABD2B	1	47846938	47847103	S18_peak_40	intron (ENST00000606738, intron 2 of 6)	ENST00000606738
MICAL2	11	12111606	12111784	S18_peak_400	promoter-TSS (ENST00000531732)	ENST00000531732
ACO1	9	32428364	32428529	S18_peak_4000	intron (ENST00000309951, intron 12 of 20)	ENST00000309951
ASS1P12	9	32946962	32947163	S18_peak_4001	promoter-TSS (ENST00000452496)	ENST00000452496
NOL6	9	33471170	33471418	S18_peak_4002	intron (ENST00000353159, intron 3 of 15)	ENST00000353159
NOL6	9	33471890	33472159	S18_peak_4003	promoter-TSS (ENST00000353159)	ENST00000353159
RP11-255A11.4	9	33574368	33574538	S18_peak_4004	promoter-TSS (ENST00000437894)	ENST00000437894
UBE2R2	9	33880652	33880893	S18_peak_4005	intron (ENST00000263228, intron 1 of 4)	ENST00000263228
UBAP1	9	34175940	34176105	S18_peak_4006	Intergenic between ENST00000435838 and ENST00000297661	ENST00000297661
NUDT2	9	34326259	34326424	S18_peak_4007	Intergenic between ENST00000419794 and ENST00000379158	ENST00000379158
UNC13B	9	35220340	35220505	S18_peak_4008	intron (ENST00000378495, intron 1 of 38)	ENST00000378495
UNC13B	9	35326470	35326635	S18_peak_4009	intron (ENST00000378495, intron 10 of 38)	ENST00000378495
PARVA	11	12359234	12359474	S18_peak_401	Intergenic between ENST00000526616 and ENST00000334956	ENST00000334956
RNF38	9	36414453	36414618	S18_peak_4010	Intergenic between ENST00000353739 and ENST00000298048	ENST00000353739
MELK	9	36736911	36737134	S18_peak_4011	Intergenic between ENST00000545008 and ENST00000580479	ENST00000545008
POLR1E	9	37495137	37495302	S18_peak_4012	exon (ENST00000377798, exon 7 of 12)	ENST00000377798
RP11-613M10.9	9	37726309	37726474	S18_peak_4013	intron (ENST00000540557, intron 10 of 11)	ENST00000540557
RP13-198D9.4	9	38725372	38725607	S18_peak_4014	Intergenic between ENST00000637691 and ENST00000444791	ENST00000637691

RP13-198D9.4	9	38741502	38741667	S18_peak_4015	Intergenic between ENST00000637691 and ENST00000444791	ENST0000063769 1
RP11-402N8.1	9	38853618	38853873	S18_peak_4016	Intergenic between ENST00000444791 and ENST00000422626	ENST0000044479 1
VN2R3P	9	38945036	38945236	S18_peak_4017	Intergenic between ENST00000444791 and ENST00000422626	ENST0000042262 6
VN2R3P	9	38951875	38952040	S18_peak_4018	Intergenic between ENST00000444791 and ENST00000422626	ENST0000042262 6
CNTNAP3	9	39078678	39078920	S18_peak_4019	exon (ENST00000297668, exon 22 of 24)	ENST0000029766 8
PARVA	11	12382503	12382835	S18_peak_402	intron (ENST00000334956, intron 1 of 12)	ENST0000033495 6
CNTNAP3	9	39175207	39175477	S18_peak_4020	intron (ENST00000297668, intron 7 of 23)	ENST0000029766 8
FKBP4P2	9	39632206	39632371	S18_peak_4021	promoter-TSS (ENST00000598909)	ENST0000059890 9
SDR42E1P1	9	39652370	39652568	S18_peak_4022	promoter-TSS (ENST00000611327)	ENST0000061132 7
RP11-104G3.7	9	39860362	39860529	S18_peak_4023	Intergenic between ENST00000454645 and ENST00000622735	ENST0000062273 5
ANKRD20A2	9	40249576	40249803	S18_peak_4024	intron (ENST00000377601, intron 9 of 14)	ENST0000037760 1
RP11-341A11.3	9	40389099	40389306	S18_peak_4025	Intergenic between ENST00000621855 and ENST00000428759	ENST0000062185 5
RP11-341A11.3	9	40396624	40396854	S18_peak_4026	Intergenic between ENST00000621855 and ENST00000428759	ENST0000062185 5
AC129778.2	9	40523719	40523900	S18_peak_4027	intron (ENST00000618223, intron 2 of 2)	ENST0000061822 3
CH17-80A12.1	9	40607058	40607354	S18_peak_4028	Intergenic between ENST00000615056 and ENST00000616097	ENST0000061609 7
FRG1HP	9	41007146	41007341	S18_peak_4029	intron (ENST00000615326, intron 3 of 3)	ENST0000061532 6
LINC00958	11	12954700	12954914	S18_peak_403	Intergenic between ENST00000454086 and ENST00000527945	ENST0000052794 5
PTGER4P2-CDK2AP2P 2	9	41266118	41266289	S18_peak_4030	Intergenic between ENST00000612104 and ENST00000619442	ENST0000061944 2
RN7SL565P	9	41582159	41582324	S18_peak_4031	Intergenic between ENST00000618246 and ENST00000612308	ENST0000061824 6
RP11-157L3.12	9	41683440	41683711	S18_peak_4032	intron (ENST00000611780, intron 2 of 2)	ENST0000061178 0
RP11-292F9.1	9	41807370	41807591	S18_peak_4033	Intergenic between ENST00000612900 and ENST00000377561	ENST0000061290 0
CNTNAP3B	9	42034208	42034426	S18_peak_4034	intron (ENST00000377561, intron 3 of 23)	ENST0000037756 1
RP11-286O1.1	9	42332463	42332660	S18_peak_4035	intron (ENST00000614030, intron 2 of 3)	ENST0000061403 0

RAB28P3	9	42459692	42459933	S18_peak_4036	Intergenic between ENST00000454088 and ENST00000448325	ENST0000044832 5
RP11-475I24.8	9	42580451	42580663	S18_peak_4037	intron (ENST00000425493, intron 2 of 2)	ENST0000042549 3
RP11-475I24.1	9	42622043	42622243	S18_peak_4038	Intergenic between ENST00000425493 and ENST00000441829	ENST0000044182 9
CYP4F60P	9	42941883	42942055	S18_peak_4039	Intergenic between ENST00000619873 and ENST00000439449	ENST0000043944 9
ARNTL	11	13374893	13375058	S18_peak_404	intron (ENST00000389707, intron 14 of 19)	ENST0000038970 7
FP325317.1	9	43650847	43651114	S18_peak_4040	Intergenic between ENST00000539936 and ENST00000610784	ENST0000053993 6
FP325317.1	9	43938539	43938736	S18_peak_4041	Intergenic between ENST00000539936 and ENST00000610784	ENST0000053993 6
FP325317.1	9	44602748	44602919	S18_peak_4042	Intergenic between ENST00000539936 and ENST00000610784	ENST0000053993 6
FP325317.1	9	45430770	45430935	S18_peak_4043	Intergenic between ENST00000539936 and ENST00000610784	ENST0000053993 6
FP325317.1	9	45473456	45473726	S18_peak_4044	Intergenic between ENST00000539936 and ENST00000610784	ENST0000053993 6
RP11-49E18.1	9	60558819	60558984	S18_peak_4045	Intergenic between ENST00000610784 and ENST00000437823	ENST0000061078 4
RP11-49E18.1	9	60659709	60659880	S18_peak_4046	Intergenic between ENST00000610784 and ENST00000437823	ENST0000061078 4
RP11-144A16.6	9	60927326	60927491	S18_peak_4047	promoter-TSS (ENST00000611916)	ENST0000061191 6
SPATA31A7	9	61158665	61158870	S18_peak_4048	Intergenic between ENST00000637145 and ENST00000619167	ENST0000061916 7
CH17-296N19.1	9	61308508	61308801	S18_peak_4049	Intergenic between ENST00000622281 and ENST00000636699	ENST0000063669 9
RP11-231N3.1	11	13919858	13920023	S18_peak_405	Intergenic between ENST00000364986 and ENST00000532065	ENST0000053206 5
FAM27C	9	61824886	61825051	S18_peak_4050	Intergenic between ENST00000617780 and ENST00000377542	ENST0000037754 2
RP11-374M1.9	9	61941947	61942328	S18_peak_4051	Intergenic between ENST00000618472 and ENST00000610388	ENST0000061038 8
RP11-96J15.2	9	62466473	62466739	S18_peak_4052	Intergenic between ENST00000438252 and ENST00000514120	ENST0000051412 0
CNTNAP3P5	9	62582053	62582260	S18_peak_4053	Intergenic between ENST00000611892 and ENST00000442803	ENST0000061189 2
CDK2AP2P2	9	62844658	62844934	S18_peak_4054	promoter-TSS	ENST0000047851

					(ENST00000478512)	2
BMS1P10	9	63391554	63391930	S18_peak_4055	Intergenic between ENST00000443754 and ENST00000433508	ENST0000044375 4
BMS1P10	9	63468016	63468181	S18_peak_4056	Intergenic between ENST00000443754 and ENST00000433508	ENST0000044375 4
FRG1JP	9	63905306	63905638	S18_peak_4057	Intergenic between ENST00000376334 and ENST00000485382	ENST0000037633 4
ANKRD20A4	9	64382169	64382429	S18_peak_4058	intron (ENST00000357336, intron 6 of 14)	ENST0000035733 6
SNX18P9	9	64422378	64422584	S18_peak_4059	Intergenic between ENST00000459461 and ENST00000440580	ENST0000044058 0
SPON1	11	14072396	14072659	S18_peak_406	intron (ENST00000576479, intron 3 of 15)	ENST0000057647 9
IGKV1OR-2	9	64780340	64780505	S18_peak_4060	Intergenic between ENST00000457995 and ENST00000604946	ENST0000045799 5
IGKV1OR9-1	9	65253235	65253400	S18_peak_4061	Intergenic between ENST00000424370 and ENST00000377420	ENST0000042437 0
AQP7P3	9	65928511	65928676	S18_peak_4062	Intergenic between ENST00000624547 and ENST00000619851	ENST0000062454 7
RP11-399F4.1	9	66036481	66036687	S18_peak_4063	Intergenic between ENST00000618497 and ENST00000620352	ENST0000062035 2
FAM95B1	9	66056724	66056889	S18_peak_4064	Intergenic between ENST00000610473 and ENST00000456554	ENST0000061047 3
ATP5A1P10	9	66682059	66682224	S18_peak_4065	Intergenic between ENST00000622233 and ENST00000622403	ENST0000062240 3
RP11-1082I11.2	9	66840610	66840842	S18_peak_4066	Intergenic between ENST00000610931 and ENST00000610519	ENST0000061051 9
CNTNAP3P2	9	67195866	67196101	S18_peak_4067	intron (ENST00000617175, intron 8 of 23)	ENST0000061717 5
RP11-203I2.2	9	67356029	67356197	S18_peak_4068	Intergenic between ENST00000618097 and ENST00000616196	ENST0000061809 7
RP11-203I2.2	9	67414956	67415121	S18_peak_4069	Intergenic between ENST00000618097 and ENST00000616196	ENST0000061809 7
SPON1	11	14185718	14185893	S18_peak_407	intron (ENST00000576479, intron 6 of 15)	ENST0000057647 9
RP11-118H15.1	9	67443854	67444019	S18_peak_4070	Intergenic between ENST00000618097 and ENST00000616196	ENST0000061619 6
RP11-118H15.1	9	67447676	67447845	S18_peak_4071	Intergenic between ENST00000618097 and ENST00000616196	ENST0000061619 6
Y_RNA	9	67694712	67694877	S18_peak_4072	Intergenic between ENST00000616196 and ENST00000619475	ENST0000061947 5

ANKRD20A1	9	67875322	67875490	S18_peak_4073	intron (ENST00000562196, intron 6 of 14)	ENST0000056219 6
TJP2	9	69141988	69142262	S18_peak_4074	Intergenic between ENST00000605734 and ENST00000377245	ENST0000037724 5
APBA1	9	69433857	69434022	S18_peak_4075	intron (ENST00000265381, intron 11 of 12)	ENST0000026538 1
RP11-141J10.1	9	70687645	70687810	S18_peak_4076	Intergenic between ENST00000605957 and ENST00000436491	ENST0000043649 1
RP11-141J10.1	9	70693761	70694063	S18_peak_4077	Intergenic between ENST00000605957 and ENST00000436491	ENST0000043649 1
RP11-63P12.2	9	72276270	72276440	S18_peak_4078	TTS (ENST00000446146)	ENST0000044614 6
RP11-63P12.2	9	72278382	72278570	S18_peak_4079	Intergenic between ENST00000446146 and ENST00000516619	ENST0000044614 6
PDE3B	11	14826750	14826915	S18_peak_408	intron (ENST00000282096, intron 7 of 15)	ENST0000028209 6
ZFAND5	9	72352078	72352269	S18_peak_4080	Intergenic between ENST00000451152 and ENST00000376960	ENST0000037696 0
RP11-313E4.1	9	72518083	72518248	S18_peak_4081	Intergenic between ENST00000415024 and ENST00000457473	ENST0000041502 4
RP11-171A24.2	9	74282484	74282649	S18_peak_4082	Intergenic between ENST00000450410 and ENST00000423681	ENST0000042368 1
RORB	9	74550899	74551104	S18_peak_4083	intron (ENST00000376896, intron 1 of 9)	ENST0000037689 6
RP11-197P3.1	9	74903526	74903705	S18_peak_4084	Intergenic between ENST00000516949 and ENST00000414735	ENST0000041473 5
CARNMT1	9	75007423	75007588	S18_peak_4085	intron (ENST00000376834, intron 3 of 7)	ENST0000037683 4
RBM22P5	9	76296643	76296897	S18_peak_4086	Intergenic between ENST00000449536 and ENST00000376736	ENST0000044953 6
DNAJB5P1	9	76935775	76936040	S18_peak_4087	Intergenic between ENST00000454004 and ENST00000417429	ENST0000045400 4
RP11-336N8.4	9	78221880	78222045	S18_peak_4088	Intergenic between ENST00000449661 and ENST00000415759	ENST0000044966 1
CEP78	9	78228866	78229031	S18_peak_4089	Intergenic between ENST00000449661 and ENST00000415759	ENST0000041575 9
INSC	11	15128798	15129005	S18_peak_409	intron (ENST00000379554, intron 1 of 12)	ENST0000037955 4
RP11-216F5.1	9	78546056	78546221	S18_peak_4090	Intergenic between ENST00000376588 and ENST00000605266	ENST0000060526 6
MTND2P8	9	78800553	78800744	S18_peak_4091	Intergenic between ENST00000450555 and ENST00000344739	ENST0000045055 5
RP11-375O18.2	9	79502658	79502842	S18_peak_4092	Intergenic between ENST00000461726 and	ENST0000042498 0

					ENST00000424980	
LINC01507	9	80036946	80037111	S18_peak_4093	Intergenic between ENST00000417801 and ENST00000443325	ENST0000041780 1
TLE1	9	81541007	81541172	S18_peak_4094	Intergenic between ENST00000457409 and ENST00000376499	ENST0000037649 9
RNA5SP287	9	81869888	81870053	S18_peak_4095	Intergenic between ENST00000413050 and ENST00000516358	ENST0000051635 8
RNA5SP287	9	81878102	81878267	S18_peak_4096	Intergenic between ENST00000413050 and ENST00000516358	ENST0000051635 8
UBQLN1	9	83696468	83696653	S18_peak_4097	intron (ENST00000376395, intron 1 of 10)	ENST0000037639 5
KIF27	9	83913448	83913684	S18_peak_4098	intron (ENST00000297814, intron 2 of 17)	ENST0000029781 4
SLC28A3	9	84298313	84298533	S18_peak_4099	intron (ENST00000376238, intron 6 of 17)	ENST0000037623 8
FAF1	1	50467275	50467533	S18_peak_41	intron (ENST00000396153, intron 18 of 18)	ENST0000039615 3
INSC	11	15245965	15246222	S18_peak_410	exon (ENST00000379554, exon 13 of 13)	ENST0000037955 4
SLC28A3	9	84334371	84334572	S18_peak_4100	intron (ENST00000376238, intron 1 of 17)	ENST0000037623 8
RP11-380F14.2	9	84414155	84414322	S18_peak_4101	Intergenic between ENST00000419815 and ENST00000423044	ENST0000041981 5
NTRK2	9	84772294	84772459	S18_peak_4102	intron (ENST00000304053, intron 12 of 14)	ENST0000030405 3
UBE2V1P10	9	85126159	85126358	S18_peak_4103	Intergenic between ENST00000304053 and ENST00000414685	ENST0000041468 5
GAS1	9	86907509	86907690	S18_peak_4104	Intergenic between ENST00000443163 and ENST00000298743	ENST0000029874 3
SPIN1	9	88393650	88393815	S18_peak_4105	intron (ENST00000375859, intron 1 of 5)	ENST0000037585 9
GADD45G	9	89611838	89612185	S18_peak_4106	Intergenic between ENST00000252506 and ENST00000617436	ENST0000025250 6
OR7E31P	9	90198925	90199090	S18_peak_4107	Intergenic between ENST00000625060 and ENST00000426212	ENST0000042621 2
PAICSP2	9	91524808	91524973	S18_peak_4108	Intergenic between ENST00000442072 and ENST00000429118	ENST0000042911 8
RP11-62C3.6	9	92199297	92199487	S18_peak_4109	Intergenic between ENST00000611097 and ENST00000457003	ENST0000045700 3
RP11-449L13.2	11	16520175	16520544	S18_peak_411	Intergenic between ENST00000525311 and ENST00000469842	ENST0000052531 1
BICD2	9	92712093	92712258	S18_peak_4110	exon (ENST00000356884, exon 7 of 7)	ENST0000035688 4
ANKRD19P	9	92849699	92849864	S18_peak_4111	intron (ENST00000473204, intron 8 of 10)	ENST0000047320 4

RP11-53B5.1	9	93841407	93841572	S18_peak_4112	Intergenic between ENST00000584984 and ENST00000443771	ENST00000443771
MFSD14B	9	94432730	94432895	S18_peak_4113	intron (ENST00000375344, intron 3 of 11)	ENST00000375344
RP11-533K9.3	9	94466608	94466773	S18_peak_4114	Intergenic between ENST00000621419 and ENST00000448030	ENST00000448030
C9orf3	9	94911626	94911791	S18_peak_4115	intron (ENST00000277198, intron 5 of 7)	ENST00000277198
FANCC	9	95307580	95307783	S18_peak_4116	intron (ENST00000289081, intron 1 of 14)	ENST00000289081
MT1P1	9	95427404	95427605	S18_peak_4117	Intergenic between ENST00000396160 and ENST00000331920	ENST00000396160
LINC00476	9	95750069	95750262	S18_peak_4118	Intergenic between ENST00000445213 and ENST00000433656	ENST00000433656
HSD17B3	9	96258850	96259024	S18_peak_4119	intron (ENST00000375263, intron 2 of 10)	ENST00000375263
RN7SKP90	11	16840341	16840561	S18_peak_412	Intergenic between ENST00000530489 and ENST00000363013	ENST00000363013
RP11-535M15.2	9	96764866	96765059	S18_peak_4120	Intergenic between ENST00000602289 and ENST00000602494	ENST00000602289
NUTM2G	9	96942236	96942401	S18_peak_4121	Intergenic between ENST00000372322 and ENST00000424606	ENST00000372322
RNU6-918P	9	98028050	98028218	S18_peak_4122	Intergenic between ENST00000411981 and ENST00000391219	ENST00000391219
FKTN	9	105594411	105594611	S18_peak_4123	intron (ENST00000223528, intron 2 of 9)	ENST00000223528
TMEM38B	9	105684369	105684619	S18_peak_4124	Intergenic between ENST00000334077 and ENST00000374692	ENST00000374692
LINC01505	9	106280705	106280870	S18_peak_4125	intron (ENST00000637185, intron 1 of 7)	ENST00000637185
RP11-508N12.2	9	107065967	107066164	S18_peak_4126	intron (ENST00000439901, intron 3 of 4)	ENST00000439901
RP11-417L14.1	9	107211869	107212135	S18_peak_4127	Intergenic between ENST00000415694 and ENST00000358015	ENST00000415694
RNU6-1064P	9	107747454	107747619	S18_peak_4128	Intergenic between ENST00000365438 and ENST00000410626	ENST00000410626
PALM2	9	109827486	109827758	S18_peak_4129	intron (ENST00000374531, intron 2 of 6)	ENST00000374531
RP11-466H18.1	11	16992104	16992289	S18_peak_413	Intergenic between ENST00000459748 and ENST00000530490	ENST00000459748
PALM2	9	109845147	109845398	S18_peak_4130	intron (ENST00000374531, intron 2 of 6)	ENST00000374531
PALM2-AKAP2	9	110024308	110024584	S18_peak_4131	intron (ENST00000374530, intron 7 of 10)	ENST00000374530
TXN	9	110274847	110275012	S18_peak_4132	Intergenic between ENST00000374517 and	ENST00000374517

					ENST00000374510	
RP11-410K21.2	9	110566096	110566261	S18_peak_4133	Intergenic between ENST00000383931 and ENST00000451108	ENST00000451108
MUSK	9	110658933	110659098	S18_peak_4134	Intergenic between ENST00000451108 and ENST00000374448	ENST00000374448
RP11-202G18.1	9	111141199	111141364	S18_peak_4135	intron (ENST00000426204, intron 2 of 2)	ENST00000426204
ZNF483	9	111574336	111574540	S18_peak_4136	intron (ENST00000358151, intron 5 of 5)	ENST00000358151
RNU6-1013P	9	111863958	111864184	S18_peak_4137	Intergenic between ENST00000384180 and ENST00000374279	ENST00000384180
UGCG	9	111899371	111899536	S18_peak_4138	intron (ENST00000374279, intron 1 of 8)	ENST00000374279
PTBP3	9	112245958	112246123	S18_peak_4139	intron (ENST00000374257, intron 7 of 13)	ENST00000374257
RP11-452G18.2	11	17248425	17248632	S18_peak_414	Intergenic between ENST00000494359 and ENST00000338965	ENST00000494359
PTBP3	9	112246657	112246822	S18_peak_4140	intron (ENST00000374257, intron 7 of 13)	ENST00000374257
KIAA1958	9	112561248	112561525	S18_peak_4141	intron (ENST00000337530, intron 1 of 3)	ENST00000337530
SNX30	9	112812024	112812189	S18_peak_4142	intron (ENST00000374232, intron 2 of 8)	ENST00000374232
FAM225A	9	113130053	113130218	S18_peak_4143	Intergenic between ENST00000453010 and ENST00000259392	ENST00000453010
RGS3	9	113489687	113489935	S18_peak_4144	intron (ENST00000478599, intron 5 of 22)	ENST00000478599
RP11-534I8.1	9	113868941	113869106	S18_peak_4145	Intergenic between ENST00000437852 and ENST00000265132	ENST00000437852
RP11-445L6.3	9	115486795	115486960	S18_peak_4146	Intergenic between ENST00000444701 and ENST00000619197	ENST00000444701
U2	9	115720801	115721050	S18_peak_4147	Intergenic between ENST00000444701 and ENST00000619197	ENST00000619197
LINC00474	9	115938356	115938644	S18_peak_4148	Intergenic between ENST00000374014 and ENST00000416507	ENST00000374014
RP11-787B4.2	9	115978070	115978235	S18_peak_4149	Intergenic between ENST00000374014 and ENST00000416507	ENST00000416507
KCNJ11	11	17411170	17411335	S18_peak_415	Intergenic between ENST00000528731 and ENST00000532834	ENST00000528731
ASTN2	9	116489818	116490028	S18_peak_4150	intron (ENST00000288520, intron 4 of 7)	ENST00000288520
RP11-349E4.1	9	118556989	118557237	S18_peak_4151	Intergenic between ENST00000413759 and ENST00000450292	ENST00000450292
MIR147A	9	120156156	120156389	S18_peak_4152	Intergenic between ENST00000454204 and ENST00000385079	ENST00000385079

TRAF1	9	120922409	120922651	S18_peak_4153	intron (ENST00000373887, intron 3 of 7)	ENST00000373887
RP11-477J21.2	9	121259823	121260100	S18_peak_4154	Intergenic between ENST00000455437 and ENST00000437135	ENST00000455437
AL513122.1	9	121310767	121311011	S18_peak_4155	intron (ENST00000449733, intron 4 of 16)	ENST00000449733
RP11-244O19.1	9	121825286	121825604	S18_peak_4156	Intergenic between ENST00000567068 and ENST00000411790	ENST00000567068
TTLL11-IT1	9	121861103	121861448	S18_peak_4157	Intergenic between ENST00000567068 and ENST00000411790	ENST00000411790
TTLL11-IT1	9	121914657	121914962	S18_peak_4158	intron (ENST00000411790, intron 1 of 1)	ENST00000411790
MIR4478	9	122112887	122113111	S18_peak_4159	Intergenic between ENST00000487468 and ENST00000584530	ENST00000584530
USH1C	11	17491264	17491436	S18_peak_416	Intergenic between ENST00000532834 and ENST00000527720	ENST00000527720
MRRF	9	122341857	122342022	S18_peak_4160	Intergenic between ENST00000344641 and ENST00000602325	ENST00000344641
RP11-498E2.9	9	122382776	122383008	S18_peak_4161	Intergenic between ENST00000602325 and ENST00000439471	ENST00000602325
RP11-64P14.7	9	122620838	122621003	S18_peak_4162	intron (ENST00000431442, intron 1 of 2)	ENST00000431442
OR1L4	9	122723880	122724060	S18_peak_4163	promoter-TSS (ENST00000259466)	ENST00000259466
SKA2P1	9	122760663	122760927	S18_peak_4164	Intergenic between ENST00000259466 and ENST00000425592	ENST00000425592
CRB2	9	123327471	123327718	S18_peak_4165	Intergenic between ENST00000407982 and ENST00000373631	ENST00000373631
CRB2	9	123329157	123329422	S18_peak_4166	Intergenic between ENST00000407982 and ENST00000373631	ENST00000373631
CRB2	9	123369180	123369345	S18_peak_4167	intron (ENST00000373631, intron 6 of 12)	ENST00000373631
RP11-230L22.4	9	123537500	123537665	S18_peak_4168	Intergenic between ENST00000458025 and ENST00000423942	ENST00000458025
RP11-85O21.2	9	123949612	123949777	S18_peak_4169	Intergenic between ENST00000421400 and ENST00000453529	ENST00000453529
RP11-113D6.6	11	18132769	18133018	S18_peak_417	Intergenic between ENST00000534768 and ENST00000527671	ENST00000527671
RP11-85O21.5	9	124089152	124089317	S18_peak_4170	Intergenic between ENST00000429482 and ENST00000320246	ENST00000429482
PSMB7	9	124356162	124356327	S18_peak_4171	intron (ENST00000259457, intron 7 of 7)	ENST00000259457
PSMB7	9	124376723	124376926	S18_peak_4172	intron (ENST00000259457, intron 6 of 7)	ENST00000259457

GOLGA1	9	124906744	12490690<br>9	S18_peak_4173	intron (ENST00000373555, intron 12 of 22)	ENST0000037355<br>5
Metazoa_SRP	9	125401847	12540201<br>2	S18_peak_4174	Intergenic between ENST00000416693 and ENST00000618720	ENST0000061872<br>0
MAPKAP1	9	125460884	12546119<br>4	S18_peak_4175	intron (ENST00000373511, intron 9 of 10)	ENST0000037351<br>1
MAPKAP1	9	125664074	12566427<br>0	S18_peak_4176	intron (ENST00000373511, intron 3 of 10)	ENST0000037351<br>1
RP11-343J18.2	9	126070729	12607089<br>4	S18_peak_4177	intron (ENST00000441473, intron 1 of 1)	ENST0000044147<br>3
RP11-343J18.2	9	126084916	12608508<br>1	S18_peak_4178	intron (ENST00000441473, intron 1 of 1)	ENST0000044147<br>3
RP11-343J18.2	9	126135926	12613614<br>7	S18_peak_4179	intron (ENST00000441473, intron 1 of 1)	ENST0000044147<br>3
RP11-113D6.6	11	18171463	18171678	S18_peak_418	intron (ENST00000527671, intron 1 of 1)	ENST0000052767<br>1
RP11-343J18.1	9	126274568	12627473<br>3	S18_peak_4180	promoter-TSS (ENST00000419853)	ENST0000041985<br>3
RP11-123K19.2	9	126717482	12671771<br>7	S18_peak_4181	Intergenic between ENST00000457964 and ENST00000373464	ENST0000045796<br>4
RP13-225O21.2	9	127227302	12722757<br>0	S18_peak_4182	Intergenic between ENST00000453199 and ENST00000373387	ENST0000045319<br>9
GARNL3	9	127243897	12724406<br>2	S18_peak_4183	Intergenic between ENST00000453199 and ENST00000373387	ENST0000037338<br>7
SLC2A8	9	127414974	12741519<br>5	S18_peak_4184	Intergenic between ENST00000373360 and ENST00000361436	ENST0000037336<br>0
CFAP157	9	127706540	12770670<br>5	S18_peak_4185	Intergenic between ENST00000373299 and ENST00000373293	ENST0000037329<br>3
SH2D3C	9	127746656	12774691<br>2	S18_peak_4186	intron (ENST00000373277, intron 5 of 10)	ENST0000037327<br>7
SLC27A4	9	128361024	12836118<br>9	S18_peak_4187	exon (ENST00000372870, exon 6 of 6)	ENST0000037287<br>0
SPTAN1	9	128629949	12863029<br>3	S18_peak_4188	intron (ENST00000372731, intron 50 of 55)	ENST0000037273<br>1
HMGA1P4	9	128661488	12866166<br>5	S18_peak_4189	Intergenic between ENST00000427516 and ENST00000428643	ENST0000042864<br>3
LDHAL6A	11	18474013	18474408	S18_peak_419	intron (ENST00000396213, intron 4 of 7)	ENST0000039621<br>3
RP11-545E17.3	9	128729066	12872927<br>7	S18_peak_4190	intron (ENST00000443631, intron 1 of 1)	ENST0000044363<br>1
PTPA	9	129158494	12915866<br>5	S18_peak_4191	Intergenic between ENST00000355007 and ENST00000600965	ENST0000035500<br>7
RP11-344B5.2	9	129278256	12927842<br>1	S18_peak_4192	Intergenic between ENST00000435157 and ENST00000455981	ENST0000045598<br>1
C9orf106	9	129326377	12932659<br>3	S18_peak_4193	Intergenic between ENST00000316786 and ENST00000599815	ENST0000031678<br>6
GPR107	9	130079115	13007928<br>0	S18_peak_4194	intron (ENST00000347136, intron 4 of 17)	ENST0000034713<br>6

RN7SL665P	9	130329428	130329593	S18_peak_4195	Intergenic between ENST00000372398 and ENST00000578793	ENST00000578793
FUBP3	9	130562017	130562337	S18_peak_4196	Intergenic between ENST00000372393 and ENST00000319725	ENST00000319725
FUBP3	9	130594096	130594461	S18_peak_4197	intron (ENST00000319725, intron 1 of 18)	ENST00000319725
RP11-83J21.3	9	130951529	130951694	S18_peak_4198	Intergenic between ENST00000421067 and ENST00000361069	ENST00000421067
SNORD62A	9	131412410	131412576	S18_peak_4199	Intergenic between ENST00000372264 and ENST00000428514	ENST00000428514
LINC01562	1	51213256	51213449	S18_peak_42	intron (ENST00000366181, intron 1 of 1)	ENST00000366181
RP11-583F24.8	11	18940656	18940878	S18_peak_420	Intergenic between ENST00000528646 and ENST00000531736	ENST00000528646
SNORD62A	9	131470641	131470838	S18_peak_4200	Intergenic between ENST00000372264 and ENST00000428514	ENST00000428514
UCK1	9	131530235	131530400	S18_peak_4201	promoter-TSS (ENST00000372211)	ENST00000372211
RP11-323H21.3	9	131813189	131813444	S18_peak_4202	Intergenic between ENST00000391093 and ENST00000444708	ENST00000444708
MED27	9	132110616	132110781	S18_peak_4203	Intergenic between ENST00000474263 and ENST00000393229	ENST00000474263
SETX	9	132291328	132291493	S18_peak_4204	intron (ENST00000224140, intron 15 of 25)	ENST00000224140
GFI1B	9	132964110	132964298	S18_peak_4205	intron (ENST00000339463, intron 1 of 10)	ENST00000339463
GTF3C5	9	133044176	133044341	S18_peak_4206	intron (ENST00000372097, intron 3 of 10)	ENST00000372097
CEL	9	133067619	133067784	S18_peak_4207	intron (ENST00000372080, intron 7 of 10)	ENST00000372080
GBGT1	9	133157595	133157760	S18_peak_4208	intron (ENST00000372038, intron 3 of 6)	ENST00000372038
GBGT1	9	133167268	133167475	S18_peak_4209	Intergenic between ENST00000372038 and ENST00000372031	ENST00000372038
NAV2	11	19767032	19767345	S18_peak_421	intron (ENST00000396085, intron 1 of 38)	ENST00000396085
VAV2	9	133752313	133752478	S18_peak_4210	Intergenic between ENST00000427237 and ENST00000406606	ENST00000406606
VAV2	9	133951388	133951656	S18_peak_4211	intron (ENST00000406606, intron 1 of 26)	ENST00000406606
RP11-473E2.2	9	134477948	134478259	S18_peak_4212	Intergenic between ENST00000585099 and ENST00000444936	ENST00000444936
RP11-473E2.2	9	134493452	134493617	S18_peak_4213	Intergenic between ENST00000585099 and ENST00000444936	ENST00000444936
RP11-473E2.3	9	134558264	134558429	S18_peak_4214	Intergenic between ENST00000446184 and	ENST00000446184

					ENST00000371817	
RP11-399H11.3	9	135198982	135199168	S18_peak_4215	Intergenic between ENST00000371793 and ENST00000452377	ENST00000452377
RP11-555H7.2	9	135360280	135360445	S18_peak_4216	Intergenic between ENST00000450850 and ENST00000449637	ENST00000450850
CAMSAP1	9	135818527	135818752	S18_peak_4217	intron (ENST00000389532, intron 12 of 16)	ENST00000389532
UBAC1	9	135992371	135992536	S18_peak_4218	Intergenic between ENST00000371756 and ENST00000566567	ENST00000371756
RP11-83N9.5	9	136080469	136080663	S18_peak_4219	Intergenic between ENST00000371756 and ENST00000566567	ENST00000566567
NAV2	11	19889811	19890065	S18_peak_422	intron (ENST00000396085, intron 5 of 38)	ENST00000396085
SNAPC4	9	136385032	136385197	S18_peak_4220	intron (ENST00000298532, intron 12 of 22)	ENST00000298532
NOTCH1	9	136492841	136493006	S18_peak_4221	Intergenic between ENST00000371712 and ENST00000277541	ENST00000277541
HSPC324	9	136638302	136638547	S18_peak_4222	Intergenic between ENST00000450304 and ENST00000411904	ENST00000411904
AGPAT2	9	136688624	136688849	S18_peak_4223	Intergenic between ENST00000371694 and ENST00000371692	ENST00000371694
TRAF2	9	136922609	136922780	S18_peak_4224	intron (ENST00000247668, intron 9 of 10)	ENST00000247668
MAN1B1-AS1	9	137084667	137084832	S18_peak_4225	TTS (ENST00000596585)	ENST00000596585
EXD3	9	137313713	137313969	S18_peak_4226	intron (ENST00000340951, intron 19 of 21)	ENST00000340951
PNPLA7	9	137480660	137480825	S18_peak_4227	intron (ENST00000277531, intron 21 of 33)	ENST00000277531
PNPLA7	9	137501877	137502042	S18_peak_4228	intron (ENST00000277531, intron 13 of 33)	ENST00000277531
RP11-48C7.8	9	137625043	137625220	S18_peak_4229	intron (ENST00000637510, intron 1 of 1)	ENST00000637510
NAV2	11	19931930	19932095	S18_peak_423	intron (ENST00000396085, intron 6 of 38)	ENST00000396085
BX004987.1	GL000009.2	119929	120195	S18_peak_4231	Intergenic after ENST00000618686	ENST00000618686
AC145212.1	GL000194.1	168552	168718	S18_peak_4232	Intergenic after ENST00000400754	ENST00000400754
pRNA	GL000220.1	18817	19062	S18_peak_4235	Intergenic before ENST00000619317	ENST00000619317
RNA5-8S5	GL000220.1	115421	115603	S18_peak_4238	Intergenic between ENST00000611446 and ENST00000614535	ENST00000611446
RNA5-8S5	GL000220.1	122564	122847	S18_peak_4239	Intergenic between ENST00000611446 and ENST00000614535	ENST00000611446
NELL1	11	20889751	20890017	S18_peak_424	intron (ENST00000298925, intron 6 of 20)	ENST00000298925
RNA5-8S5	GL000220.1	124509	124674	S18_peak_4240	Intergenic between ENST00000611446 and	ENST00000611446

					ENST00000614535	
NELL1	11	21543719	21543885	S18_peak_425	intron (ENST00000298925, intron 17 of 20)	ENST00000298925
5_8S_rRNA	KI270442.1	111039	111314	S18_peak_4256	Intergenic before ENST00000611690	ENST00000611690
5_8S_rRNA	KI270442.1	223069	223234	S18_peak_4257	Intergenic between ENST00000611690 and ENST00000620265	ENST00000611690
RP11-945A11.1	11	23717264	23717429	S18_peak_426	Intergenic between ENST00000474589 and ENST00000534068	ENST00000534068
AC240274.1	KI270711.1	1849	2099	S18_peak_4263	Intergenic before ENST00000614336	ENST00000614336
AC141272.1	KI270728.1	1439427	1439627	S18_peak_4266	Intergenic after ENST00000611746	ENST00000611746
AC141272.1	KI270728.1	1539936	1540105	S18_peak_4267	Intergenic after ENST00000611746	ENST00000611746
AC141272.1	KI270728.1	1734005	1734258	S18_peak_4268	Intergenic after ENST00000611746	ENST00000611746
RP11-747E23.1	11	24004004	24004265	S18_peak_427	Intergenic between ENST00000619919 and ENST00000527283	ENST00000619919
pRNA	KI270733.1	85859	86135	S18_peak_4270	Intergenic before ENST00000618675	ENST00000618675
pRNA	KI270733.1	155250	155428	S18_peak_4278	Intergenic between ENST00000616292 and ENST00000615130	ENST00000615130
RP11-46P12.1	11	25629322	25629581	S18_peak_428	Intergenic between ENST00000473882 and ENST00000532310	ENST00000532310
pRNA	KI270733.1	168096	168261	S18_peak_4282	TTS (ENST00000615130)	ENST00000615130
RNA5-8S5	KI270733.1	175091	175257	S18_peak_4283	Intergenic after ENST00000618998	ENST00000618998
AC007325.1	KI270734.1	71040	71205	S18_peak_4284	Intergenic between ENST00000615362 and ENST00000617983	ENST00000617983
U6	KI270744.1	133944	134125	S18_peak_4286	Intergenic after ENST00000616830	ENST00000616830
U6	KI270744.1	154185	154360	S18_peak_4287	Intergenic after ENST00000616830	ENST00000616830
RP11-405K6.1	11	25833364	25833529	S18_peak_429	Intergenic between ENST00000533942 and ENST00000531157	ENST00000533942
U1	KI270750.1	149	418	S18_peak_4293	Intergenic before ENST00000612925	ENST00000612925
TTC39A	1	51311347	51311552	S18_peak_43	intron (ENST00000413473, intron 4 of 17)	ENST00000413473
FIBIN	11	26878905	26879176	S18_peak_430	Intergenic between ENST00000396005 and ENST00000318627	ENST00000318627
RP13-465B17.4	X	397410	397575	S18_peak_4300	intron (ENST00000627721, intron 1 of 1)	ENST00000627721
FABP5P13	X	528232	528403	S18_peak_4301	Intergenic between ENST00000452144 and ENST00000431919	ENST00000452144
RP11-309M23.1	X	881935	882100	S18_peak_4302	Intergenic between ENST00000431919 and	ENST00000420865

					ENST00000420865	
CSF2RA	X	1297817	1297982	S18_peak_4303	intron (ENST00000432318, intron 10 of 13)	ENST0000043231 8
IL3RA	X	1382306	1382501	S18_peak_4304	exon (ENST00000331035, exon 12 of 12)	ENST0000033103 5
ASMT	X	1651592	1651810	S18_peak_4305	Intergenic between ENST00000381233 and ENST00000427886	ENST0000038123 3
MIR6089	X	2586874	2587039	S18_peak_4306	Intergenic between ENST00000381218 and ENST00000616698	ENST0000061669 8
LINC01546	X	3284162	3284327	S18_peak_4307	exon (ENST00000457435, exon 3 of 3)	ENST0000045743 5
SNORA48	X	3522994	3523295	S18_peak_4308	Intergenic between ENST00000439462 and ENST00000390912	ENST0000039091 2
RP11-706O15.3	X	3853499	3853664	S18_peak_4309	exon (ENST00000381108, exon 3 of 3)	ENST0000038110 8
BBOX1-AS1	11	27148987	27149211	S18_peak_431	intron (ENST00000526061, intron 3 of 3)	ENST0000052606 1
MIR4770	X	6298578	6298743	S18_peak_4310	Intergenic between ENST00000516482 and ENST00000579149	ENST0000057914 9
RP13-926M18.1	X	7001891	7002056	S18_peak_4311	Intergenic between ENST00000463344 and ENST00000381077	ENST0000046334 4
RP11-692P14.1	X	8051150	8051315	S18_peak_4312	Intergenic between ENST00000422160 and ENST00000384896	ENST0000042216 0
VCX2	X	8250471	8250636	S18_peak_4313	Intergenic between ENST00000317103 and ENST00000381032	ENST0000031710 3
CLDN34	X	9962608	9962773	S18_peak_4314	Intergenic between ENST00000384117 and ENST00000445307	ENST0000044530 7
WWC3	X	10012689	10012862	S18_peak_4315	Intergenic between ENST00000445307 and ENST00000380861	ENST0000038086 1
WWC3	X	10060925	10061090	S18_peak_4316	intron (ENST00000380861, intron 1 of 22)	ENST0000038086 1
WWC3	X	10134464	10134629	S18_peak_4317	exon (ENST00000380861, exon 19 of 23)	ENST0000038086 1
RP6-1O2.1	X	10253487	10253652	S18_peak_4318	intron (ENST00000454113, intron 1 of 4)	ENST0000045411 3
RP11-120D5.1	X	10814737	10814906	S18_peak_4319	Intergenic between ENST00000384473 and ENST00000608176	ENST0000060817 6
HSP90AA2P	11	27892210	27892375	S18_peak_432	Intergenic between ENST00000530115 and ENST00000525309	ENST0000053011 5
RP11-120D5.1	X	10875536	10875704	S18_peak_4320	intron (ENST00000608176, intron 2 of 3)	ENST0000060817 6
RP11-120D5.1	X	10965082	10965247	S18_peak_4321	intron (ENST00000608176, intron 2 of 3)	ENST0000060817 6
AC002366.3	X	11438067	11438232	S18_peak_4322	Intergenic between ENST00000446186 and ENST00000634846	ENST0000044618 6

MSL3	X	11897978	11898143	S18_peak_4323	Intergenic between ENST00000337339 and ENST00000384065	ENST0000033733 9
TMSB4X	X	12983389	12983554	S18_peak_4324	Intergenic between ENST00000451311 and ENST00000380625	ENST0000045131 1
GS1-600G8.5	X	13182275	13182440	S18_peak_4325	Intergenic between ENST00000568788 and ENST00000412485	ENST0000041248 5
GS1-600G8.5	X	13227392	13227719	S18_peak_4326	Intergenic between ENST00000568788 and ENST00000412485	ENST0000041248 5
RP11-1L9.1	X	13433739	13433904	S18_peak_4327	Intergenic between ENST00000420403 and ENST00000380602	ENST0000042040 3
GPM6B	X	13776236	13776413	S18_peak_4328	intron (ENST00000316715, intron 6 of 7)	ENST0000031671 5
GPM6B	X	13791673	13791838	S18_peak_4329	intron (ENST00000316715, intron 2 of 7)	ENST0000031671 5
RP11-22P4.2	11	28516967	28517132	S18_peak_433	promoter-TSS (ENST00000524707)	ENST0000052470 7
PIR	X	15393698	15393901	S18_peak_4330	intron (ENST00000380421, intron 8 of 9)	ENST0000038042 1
RPL6P30	X	16271344	16271509	S18_peak_4331	Intergenic between ENST00000443382 and ENST00000447112	ENST0000044338 2
REPS2	X	17047741	17047926	S18_peak_4332	intron (ENST00000357277, intron 6 of 17)	ENST0000035727 7
LINC01456	X	18072678	18072843	S18_peak_4333	intron (ENST00000453902, intron 1 of 4)	ENST0000045390 2
SCML2	X	18268361	18268560	S18_peak_4334	intron (ENST00000251900, intron 7 of 14)	ENST0000025190 0
CDKL5	X	18641292	18641564	S18_peak_4335	intron (ENST00000379996, intron 18 of 20)	ENST0000037999 6
PHKA2-AS1	X	18878846	18879011	S18_peak_4336	Intergenic between ENST00000439908 and ENST00000452900	ENST0000045290 0
ADGRG2	X	19144568	19144733	S18_peak_4337	Intergenic between ENST00000356606 and ENST00000338883	ENST0000035660 6
ADGRG2	X	19165368	19165533	S18_peak_4338	Intergenic between ENST00000356606 and ENST00000338883	ENST0000035660 6
SH3KBP1	X	19554419	19554584	S18_peak_4339	intron (ENST00000397821, intron 13 of 17)	ENST0000039782 1
OR2BH1P	11	29064996	29065192	S18_peak_434	Intergenic between ENST00000608850 and ENST00000530960	ENST0000060885 0
SH3KBP1	X	19772758	19772969	S18_peak_4340	intron (ENST00000397821, intron 2 of 17)	ENST0000039782 1
SH3KBP1	X	19809581	19809746	S18_peak_4341	intron (ENST00000397821, intron 2 of 17)	ENST0000039782 1
MBTPS2	X	21816312	21816477	S18_peak_4342	Intergenic between ENST00000379494 and ENST00000379484	ENST0000037948 4
MBTPS2	X	21848489	21848654	S18_peak_4343	intron (ENST00000379484, intron 3 of 10)	ENST0000037948 4
SMS	X	21990849	21991082	S18_peak_4344	intron (ENST00000404933,	ENST0000040493

					intron 9 of 10)	3
PHEX	X	22122768	22122933	S18_peak_4345	intron (ENST00000379374, intron 11 of 21)	ENST0000037937 4
PHEX	X	22165809	22165974	S18_peak_4346	intron (ENST00000379374, intron 12 of 21)	ENST0000037937 4
PTCHD1-AS	X	22283768	22283933	S18_peak_4347	intron (ENST00000608254, intron 5 of 5)	ENST0000060825 4
PTCHD1-AS	X	22860120	22860285	S18_peak_4348	intron (ENST00000608254, intron 3 of 5)	ENST0000060825 4
NR0B1	X	30429617	30429782	S18_peak_4349	Intergenic between ENST00000378970 and ENST00000378962	ENST0000037897 0
RP11-466I1.1	11	29092476	29092641	S18_peak_435	Intergenic between ENST00000608850 and ENST00000530960	ENST0000053096 0
RP13-444K19.1	X	53833434	53833712	S18_peak_4350	Intergenic between ENST00000455300 and ENST00000364748	ENST0000045530 0
KRT8P17	X	59153340	59153505	S18_peak_4351	Intergenic between ENST00000453110 and ENST00000455793	ENST0000045311 0
RP11-3D23.1	X	62470401	62470596	S18_peak_4352	Intergenic between ENST00000453110 and ENST00000455793	ENST0000045579 3
RP11-3D23.1	X	62522586	62522751	S18_peak_4353	Intergenic between ENST00000453110 and ENST00000455793	ENST0000045579 3
LINC01278	X	63441688	63441853	S18_peak_4354	intron (ENST00000610088, intron 1 of 3)	ENST0000061008 8
RP11-231N9.1	X	65209401	65209578	S18_peak_4355	Intergenic between ENST00000451184 and ENST00000233836	ENST0000045118 4
RP6-22P16.1	X	67140366	67140552	S18_peak_4356	Intergenic between ENST00000410735 and ENST00000441055	ENST0000044105 5
BMI1P1	X	67871434	67871599	S18_peak_4357	Intergenic between ENST00000445879 and ENST00000561973	ENST0000044587 9
EFNB1	X	68998056	68998295	S18_peak_4358	Intergenic between ENST00000204961 and ENST00000374584	ENST0000020496 1
FTX	X	74208668	74208833	S18_peak_4359	intron (ENST00000603672, intron 5 of 5)	ENST0000060367 2
RP11-460B17.3	11	29433803	29434003	S18_peak_436	intron (ENST00000525097, intron 2 of 3)	ENST0000052509 7
MIR325HG	X	76710813	76710978	S18_peak_4360	intron (ENST00000630388, intron 2 of 3)	ENST0000063038 8
MIR325HG	X	76780511	76780676	S18_peak_4361	intron (ENST00000630388, intron 2 of 3)	ENST0000063038 8
RP13-88F20.1	X	93182722	93182983	S18_peak_4362	Intergenic between ENST00000441503 and ENST00000441188	ENST0000044118 8
RP1-241P17.4	X	115731892	11573210 5	S18_peak_4363	Intergenic between ENST00000536192 and ENST00000451869	ENST0000053619 2
RHOXF1-AS1	X	120066583	12006676 6	S18_peak_4364	intron (ENST00000553843, intron 1 of 4)	ENST0000055384 3

RP4-655L22.5	X	120703539	120703785	S18_peak_4365	Intergenic between ENST00000371313 and ENST00000613393	ENST00000613393
RP1-85D24.3	Y	11100414	11100677	S18_peak_4366	Intergenic between ENST00000618128 and ENST00000605663	ENST00000605663
DUX4L17	Y	11318638	11318816	S18_peak_4367	Intergenic between ENST00000557360 and ENST00000553347	ENST00000557360
RP4-562D20.2	11	30136413	30136619	S18_peak_437	Intergenic between ENST00000527819 and ENST00000528537	ENST00000528537
RP4-562D20.2	11	30179077	30179289	S18_peak_438	Intergenic between ENST00000528537 and ENST00000254122	ENST00000528537
ELP4	11	31637537	31637714	S18_peak_439	intron (ENST00000350638, intron 7 of 9)	ENST00000350638
ZCCHC11	1	52506070	52506235	S18_peak_44	intron (ENST00000257177, intron 4 of 29)	ENST00000257177
PAUPAR	11	31861303	31861551	S18_peak_440	Intergenic between ENST00000630360 and ENST00000531962	ENST00000630360
THEM7P	11	32218766	32218980	S18_peak_441	intron (ENST00000419556, intron 2 of 2)	ENST00000419556
THEM7P	11	32325077	32325242	S18_peak_442	intron (ENST00000525689, intron 1 of 1)	ENST00000525689
CCDC73	11	32784949	32785114	S18_peak_443	intron (ENST00000335185, intron 1 of 17)	ENST00000335185
CCDC73	11	32791717	32791963	S18_peak_444	intron (ENST00000335185, intron 1 of 17)	ENST00000335185
DEPDC7	11	33011044	33011397	S18_peak_445	Intergenic between ENST00000363745 and ENST00000241051	ENST00000241051
CSTF3	11	33154593	33154790	S18_peak_446	intron (ENST00000431742, intron 1 of 1)	ENST00000431742
RP1-130L23.1	11	33414067	33414232	S18_peak_447	Intergenic between ENST00000532231 and ENST00000527476	ENST00000532231
CD59	11	33710256	33710421	S18_peak_448	exon (ENST00000395850, exon 4 of 4)	ENST00000395850
RP4-683L5.1	11	35424233	35424405	S18_peak_449	Intergenic between ENST00000534165 and ENST00000622144	ENST00000534165
HNRNPA3P12	1	53989678	53989979	S18_peak_45	Intergenic between ENST00000437410 and ENST00000371362	ENST00000437410
RP4-683L5.1	11	35425521	35425686	S18_peak_450	Intergenic between ENST00000534165 and ENST00000622144	ENST00000534165
PAMR1	11	35457109	35457405	S18_peak_451	intron (ENST00000622144, intron 6 of 11)	ENST00000622144
LDLRAD3	11	36170667	36170863	S18_peak_452	intron (ENST00000315571, intron 4 of 5)	ENST00000315571
LDLRAD3	11	36213538	36213703	S18_peak_453	intron (ENST00000315571, intron 4 of 5)	ENST00000315571
COMMD9	11	36274065	36274237	S18_peak_454	exon (ENST00000263401, exon 6 of 6)	ENST00000263401
PRR5L	11	36303922	36304152	S18_peak_455	intron (ENST00000530639,	ENST0000053063

					intron 1 of 8)	9
TRAF6	11	36480508	36480714	S18_peak_456	Intergenic between ENST00000530627 and ENST00000526995	ENST0000052699 5
RP11-545D22.1	11	36520413	36520654	S18_peak_457	Intergenic between ENST00000624454 and ENST00000299440	ENST0000062445 4
LINC01493	11	38742171	38742336	S18_peak_458	Intergenic between ENST00000534756 and ENST00000486306	ENST0000053475 6
RNU6-99P	11	39343833	39344080	S18_peak_459	Intergenic between ENST00000364721 and ENST00000604322	ENST0000036472 1
RP11-101C11.1	1	55220055	55220354	S18_peak_46	intron (ENST00000451250, intron 1 of 1)	ENST0000045125 0
RNU6-99P	11	39478393	39478558	S18_peak_460	Intergenic between ENST00000364721 and ENST00000604322	ENST0000036472 1
LRRC4C	11	40293769	40293934	S18_peak_461	promoter-TSS (ENST00000278198)	ENST0000027819 8
RP11-58K22.1	11	44493435	44493683	S18_peak_462	Intergenic between ENST00000528183 and ENST00000525465	ENST0000052818 3
RP11-45A12.2	11	44747725	44747890	S18_peak_463	Intergenic between ENST00000530039 and ENST00000525114	ENST0000053003 9
RP11-45A12.2	11	44805605	44805794	S18_peak_464	Intergenic between ENST00000530039 and ENST00000525114	ENST0000053003 9
AC068858.1	11	44920225	44920400	S18_peak_465	Intergenic between ENST00000530039 and ENST00000525114	ENST0000052511 4
CHST1	11	45639346	45639511	S18_peak_466	Intergenic between ENST00000525926 and ENST00000308064	ENST0000030806 4
CRY2	11	45840876	45841117	S18_peak_467	Intergenic between ENST00000534128 and ENST00000417225	ENST0000041722 5
CRY2	11	45861463	45861628	S18_peak_468	intron (ENST00000417225, intron 4 of 11)	ENST0000041722 5
PHF21A	11	45963582	45963747	S18_peak_469	intron (ENST00000323180, intron 9 of 17)	ENST0000032318 0
RP1-158P9.1	1	56135700	56135929	S18_peak_47	Intergenic between ENST00000455010 and ENST00000435828	ENST0000043582 8
RP11-702F3.3	11	46158558	46158832	S18_peak_470	intron (ENST00000635144, intron 2 of 3)	ENST0000063514 4
HARBI1	11	46609956	46610144	S18_peak_471	intron (ENST00000326737, intron 2 of 2)	ENST0000032673 7
C11orf49	11	47100429	47100628	S18_peak_472	intron (ENST00000378618, intron 3 of 8)	ENST0000037861 8
SPI1	11	47369908	47370360	S18_peak_473	intron (ENST00000227163, intron 2 of 4)	ENST0000022716 3
RP11-750H9.5	11	47386839	47387142	S18_peak_474	intron (ENST00000527426, intron 1 of 1)	ENST0000052742 6
CELF1	11	47469762	47469927	S18_peak_475	intron (ENST00000395292, intron 13 of 13)	ENST0000039529 2

AGBL2	11	47685092	47685298	S18_peak_476	intron (ENST00000528609, intron 1 of 8)	ENST0000052860 9
NUP160	11	47776548	47776796	S18_peak_477	Intergenic between ENST00000363220 and ENST00000378460	ENST0000037846 0
OR4B1	11	48174907	48175243	S18_peak_478	Intergenic between ENST00000577977 and ENST00000309562	ENST0000030956 2
OR4S1	11	48297452	48297617	S18_peak_479	Intergenic between ENST00000320048 and ENST00000319988	ENST0000031998 8
RP11-414C16.1	1	58332931	58333096	S18_peak_48	Intergenic between ENST00000394974 and ENST00000371226	ENST0000039497 4
OR4C2P	11	48423007	48423188	S18_peak_480	Intergenic between ENST00000530855 and ENST00000434991	ENST0000053085 5
FOLH1	11	49231452	49231658	S18_peak_481	Intergenic between ENST00000533034 and ENST00000529674	ENST0000053303 4
RP11-707M1.1	11	49810702	49810945	S18_peak_482	TTS (ENST00000527477)	ENST0000052747 7
RP11-574M7.2	11	50619989	50620244	S18_peak_483	Intergenic between ENST00000532521 and ENST00000330155	ENST0000053252 1
RP11-574M7.2	11	51607073	51607241	S18_peak_484	Intergenic between ENST00000532521 and ENST00000330155	ENST0000053252 1
RP11-574M7.2	11	51618087	51618262	S18_peak_485	Intergenic between ENST00000532521 and ENST00000330155	ENST0000053252 1
RP11-574M7.2	11	51735485	51735760	S18_peak_486	Intergenic between ENST00000532521 and ENST00000330155	ENST0000053252 1
RP11-574M7.2	11	51949321	51949486	S18_peak_487	Intergenic between ENST00000532521 and ENST00000330155	ENST0000053252 1
RP11-574M7.2	11	52107912	52108152	S18_peak_488	Intergenic between ENST00000532521 and ENST00000330155	ENST0000053252 1
RP11-574M7.2	11	52481190	52481371	S18_peak_489	Intergenic between ENST00000532521 and ENST00000330155	ENST0000053252 1
MYSM1	1	58677682	58677875	S18_peak_49	intron (ENST00000472487, intron 8 of 19)	ENST0000047248 7
OR4C50P	11	52629236	52629405	S18_peak_490	Intergenic between ENST00000532521 and ENST00000330155	ENST0000033015 5
OR4C50P	11	53547358	53547534	S18_peak_491	Intergenic between ENST00000532521 and ENST00000330155	ENST0000033015 5
OR4C50P	11	54296755	54296979	S18_peak_492	Intergenic between ENST00000532521 and ENST00000330155	ENST0000033015 5
OR4C50P	11	54331643	54331847	S18_peak_493	Intergenic between ENST00000532521 and ENST00000330155	ENST0000033015 5
TRIM48	11	55107627	55107792	S18_peak_494	Intergenic between	ENST0000041754

					ENST00000534364 and ENST00000417545	5
RN7SL605P	11	57524347	57524512	S18_peak_495	Intergenic between ENST00000528450 and ENST00000577229	ENST0000057722 9
AP000662.4	11	57642353	57642614	S18_peak_496	intron (ENST00000528466, intron 1 of 1)	ENST0000052846 6
TMX2-CTNND1	11	57760352	57760517	S18_peak_497	intron (ENST00000528395, intron 2 of 3)	ENST0000052839 5
OR5B15P	11	58384898	58385063	S18_peak_498	Intergenic between ENST00000456493 and ENST00000609961	ENST0000060996 1
RP11-142C4.6	11	59056870	59057214	S18_peak_499	promoter-TSS (ENST00000533954)	ENST0000053395 4
RP5-892F13.2	1	7936245	7936410	S18_peak_5	Intergenic between ENST00000054668 and ENST00000603760	ENST0000060376 0
RP4-794H19.1	1	58911820	58911985	S18_peak_50	Intergenic between ENST00000427292 and ENST00000634502	ENST0000042729 2
OSBP	11	59599773	59599938	S18_peak_500	intron (ENST00000263847, intron 7 of 13)	ENST0000026384 7
OOSP1	11	59934683	59934848	S18_peak_501	Intergenic between ENST00000603021 and ENST00000398992	ENST0000039899 2
RP11-286N22.8	11	61466037	61466272	S18_peak_502	intron (ENST00000544025, intron 3 of 3)	ENST0000054402 5
DAGLA	11	61720690	61720855	S18_peak_503	exon (ENST00000257215, exon 3 of 20)	ENST0000025721 5
MYRF	11	61784563	61784815	S18_peak_504	intron (ENST00000278836, intron 25 of 26)	ENST0000027883 6
BEST1	11	61950248	61950413	S18_peak_505	promoter-TSS (ENST00000378043)	ENST0000037804 3
EEF1G	11	62570968	62571144	S18_peak_506	exon (ENST00000329251, exon 5 of 10)	ENST0000032925 1
SNHG1	11	62851192	62851357	S18_peak_507	TTS (ENST00000537068)	ENST0000053706 8
SLC22A9	11	63426942	63427107	S18_peak_508	Intergenic between ENST00000279178 and ENST00000301790	ENST0000027917 8
RP11-466C23.5	11	63832197	63832476	S18_peak_509	Intergenic between ENST00000294244 and ENST00000538089	ENST0000053808 9
FGGY	1	59556919	59557084	S18_peak_51	intron (ENST00000371218, intron 8 of 16)	ENST0000037121 8
MACROD1	11	64005091	64005303	S18_peak_510	intron (ENST00000255681, intron 4 of 10)	ENST0000025568 1
GPR137	11	64287044	64287270	S18_peak_511	intron (ENST00000411458, intron 4 of 8)	ENST0000041145 8
NAALADL1	11	65052143	65052355	S18_peak_512	intron (ENST00000358658, intron 8 of 17)	ENST0000035865 8
NEAT1_1	11	65422631	65422844	S18_peak_513	Intergenic between ENST00000317568 and ENST00000620348	ENST0000062034 8
mascRNA-menRNA	11	65507148	65507360	S18_peak_514	Intergenic between ENST00000611300 and ENST00000603826	ENST0000061130 0
CCDC85B	11	65891271	65891654	S18_peak_515	promoter-TSS	ENST0000031257

					(ENST00000312579)	9
TSGA10IP	11	65960345	65960529	S18_peak_516	TTS (ENST00000532620)	ENST0000053262 0
RP11-1167A19.6	11	65986266	65986431	S18_peak_517	Intergenic between ENST00000533995 and ENST00000533544	ENST0000053399 5
PACS1	11	66098149	66098476	S18_peak_518	intron (ENST00000320580, intron 1 of 23)	ENST0000032058 0
PACS1	11	66122380	66122545	S18_peak_519	intron (ENST00000320580, intron 1 of 23)	ENST0000032058 0
C1orf87	1	59986774	59986939	S18_peak_52	Intergenic between ENST00000583379 and ENST00000371201	ENST0000037120 1
PACS1	11	66166284	66166471	S18_peak_520	intron (ENST00000320580, intron 1 of 23)	ENST0000032058 0
CD248	11	66322202	66322367	S18_peak_521	Intergenic between ENST00000311330 and ENST00000311320	ENST0000031133 0
CTD-3074O7.11	11	66529245	66529486	S18_peak_522	intron (ENST00000419755, intron 13 of 16)	ENST0000041975 5
RN7SL12P	11	66719246	66719411	S18_peak_523	Intergenic between ENST00000473849 and ENST00000528754	ENST0000047384 9
RPS6KB2	11	67430829	67430994	S18_peak_524	intron (ENST00000312629, intron 4 of 14)	ENST0000031262 9
PTPRCAP	11	67440473	67440790	S18_peak_525	Intergenic between ENST00000326294 and ENST00000312457	ENST0000032629 4
AIP	11	67478614	67478779	S18_peak_526	Intergenic between ENST00000393877 and ENST00000279146	ENST0000027914 6
OR7E145P	11	67727999	67728193	S18_peak_527	Intergenic between ENST00000524910 and ENST00000532901	ENST0000052491 0
ENPP7P7	11	67828735	67828944	S18_peak_528	intron (ENST00000611822, intron 2 of 3)	ENST0000061182 2
TCIRG1	11	68045058	68045223	S18_peak_529	intron (ENST00000265686, intron 10 of 19)	ENST0000026568 6
NFIA	1	61110076	61110241	S18_peak_53	intron (ENST00000407417, intron 2 of 10)	ENST0000040741 7
CHKA	11	68108369	68108534	S18_peak_530	intron (ENST00000265689, intron 1 of 11)	ENST0000026568 9
CTD-2655K5.1	11	68184521	68184686	S18_peak_531	Intergenic between ENST00000530842 and ENST00000304271	ENST0000053084 2
RP11-554A11.8	11	69131940	69132130	S18_peak_532	Intergenic between ENST00000567925 and ENST00000562772	ENST0000056277 2
RP11-554A11.8	11	69132497	69132857	S18_peak_533	Intergenic between ENST00000567925 and ENST00000562772	ENST0000056277 2
RP11-211G23.2	11	69392261	69392426	S18_peak_534	Intergenic between ENST00000561588 and ENST00000562341	ENST0000056158 8
AP000439.3	11	69514605	69514880	S18_peak_535	Intergenic between ENST00000542064 and ENST00000227507	ENST0000054206 4

CCND1	11	69567541	69567706	S18_peak_536	Intergenic between ENST00000542064 and ENST00000227507	ENST0000022750 7
FGF19	11	69720126	69720326	S18_peak_537	Intergenic between ENST00000294312 and ENST00000413963	ENST0000029431 2
PPFIA1	11	70334704	70334869	S18_peak_538	intron (ENST00000253925, intron 10 of 27)	ENST0000025392 5
SHANK2	11	70540843	70541008	S18_peak_539	intron (ENST00000601538, intron 16 of 24)	ENST0000060153 8
PATJ	1	61757188	61757353	S18_peak_54	intron (ENST00000371158, intron 1 of 42)	ENST0000037115 8
SHANK2	11	70695400	70695565	S18_peak_540	intron (ENST00000601538, intron 14 of 24)	ENST0000060153 8
FLJ42102	11	71230088	71230253	S18_peak_541	Intergenic between ENST00000579074 and ENST00000460880	ENST0000046088 0
FLJ42102	11	71237433	71237669	S18_peak_542	Intergenic between ENST00000579074 and ENST00000460880	ENST0000046088 0
FLJ42102	11	71286743	71286995	S18_peak_543	Intergenic between ENST00000579074 and ENST00000460880	ENST0000046088 0
RPS3AP41	11	71659109	71659332	S18_peak_544	Intergenic between ENST00000524675 and ENST00000462701	ENST0000046270 1
DEFB108B	11	71836025	71836190	S18_peak_545	intron (ENST00000328698, intron 1 of 1)	ENST0000032869 8
RNF121	11	71961911	71962291	S18_peak_546	intron (ENST00000361756, intron 3 of 8)	ENST0000036175 6
AP000593.5	11	72252213	72252378	S18_peak_547	Intergenic between ENST00000542775 and ENST00000393668	ENST0000054277 5
ATG16L2	11	72818187	72818389	S18_peak_548	intron (ENST00000321297, intron 3 of 17)	ENST0000032129 7
FCHSD2	11	73022720	73022885	S18_peak_549	intron (ENST00000409418, intron 3 of 19)	ENST0000040941 8
PATJ	1	62056636	62056886	S18_peak_55	intron (ENST00000371158, intron 31 of 42)	ENST0000037115 8
P2RY6	11	73270453	73270618	S18_peak_550	intron (ENST00000349767, intron 1 of 3)	ENST0000034976 7
RP11-707G14.8	11	73933357	73933522	S18_peak_551	Intergenic between ENST00000543613 and ENST00000339764	ENST0000054361 3
RN7SKP297	11	74674975	74675140	S18_peak_552	Intergenic between ENST00000530970 and ENST00000411270	ENST0000041127 0
ARRB1	11	75269967	75270152	S18_peak_553	intron (ENST00000420843, intron 13 of 15)	ENST0000042084 3
ARRB1	11	75294973	75295149	S18_peak_554	intron (ENST00000420843, intron 1 of 15)	ENST0000042084 3
MIR326	11	75360994	75361291	S18_peak_555	Intergenic between ENST00000362220 and ENST00000422465	ENST0000036222 0
RPS3	11	75393297	75393462	S18_peak_556	Intergenic between ENST00000362220 and ENST00000422465	ENST0000042246 5
EMSY	11	76458130	76458295	S18_peak_557	exon (ENST00000525038,	ENST0000052503

					exon 5 of 20)	8
RP11-21L23.4	11	76750505	76750670	S18_peak_558	Intergenic between ENST00000525328 and ENST00000533437	ENST0000053343 7
ALG8	11	78114868	78115033	S18_peak_559	intron (ENST00000299626, intron 5 of 12)	ENST0000029962 6
DOCK7	1	62485003	62485168	S18_peak_56	intron (ENST00000454575, intron 42 of 48)	ENST0000045457 5
MIR5579	11	79467217	79467382	S18_peak_560	Intergenic between ENST00000580400 and ENST00000526851	ENST0000058040 0
RP11-258O13.1	11	79682821	79683104	S18_peak_561	Intergenic between ENST00000526851 and ENST00000623322	ENST0000052685 1
RP11-683O4.1	11	79922686	79922851	S18_peak_562	Intergenic between ENST00000526851 and ENST00000623322	ENST0000062332 2
MIR4300HG	11	82297580	82297745	S18_peak_563	intron (ENST00000500502, intron 3 of 7)	ENST0000050050 2
HNRNPA1P72	11	84463509	84463713	S18_peak_564	Intergenic between ENST00000513668 and ENST00000529511	ENST0000052951 1
HNRNPCP6	11	85030093	85030258	S18_peak_565	Intergenic between ENST00000525816 and ENST00000528836	ENST0000052581 6
RP11-882I15.1	11	85189774	85190038	S18_peak_566	Intergenic between ENST00000525816 and ENST00000528836	ENST0000052883 6
RP11-882I15.1	11	85254731	85254896	S18_peak_567	Intergenic between ENST00000525816 and ENST00000528836	ENST0000052883 6
SYTL2	11	85751747	85752040	S18_peak_568	intron (ENST00000527523, intron 1 of 18)	ENST0000052752 3
Metazoa_SRP	11	86226989	86227154	S18_peak_569	Intergenic between ENST00000618659 and ENST00000263360	ENST0000061865 9
RP4-597J3.1	1	63752945	63753110	S18_peak_57	Intergenic between ENST00000489463 and ENST00000418154	ENST0000041815 4
CCDC81	11	86406638	86407136	S18_peak_570	intron (ENST00000354755, intron 6 of 13)	ENST0000035475 5
CTD-2005H7.2	11	86769321	86769486	S18_peak_571	Intergenic between ENST00000532979 and ENST00000280258	ENST0000053297 9
RP11-736K20.5	11	86920451	86920768	S18_peak_572	promoter-TSS (ENST00000528660)	ENST0000052866 0
HNRNPCP8	11	87017066	87017574	S18_peak_573	Intergenic between ENST00000531827 and ENST00000528764	ENST0000052876 4
TMEM135	11	87078535	87078700	S18_peak_574	intron (ENST00000340353, intron 3 of 13)	ENST0000034035 3
CTD-2028E8.2	11	87495075	87495287	S18_peak_575	Intergenic between ENST00000624220 and ENST00000624107	ENST0000062422 0
RP11-665E10.3	11	87943028	87943193	S18_peak_576	Intergenic between ENST00000533307 and ENST00000532849	ENST0000053330 7

RP11-665E10.3	11	87963908	87964162	S18_peak_577	Intergenic between ENST00000533307 and ENST00000532849	ENST0000053330 7
RP11-164N3.1	11	88018039	88018220	S18_peak_578	Intergenic between ENST00000533307 and ENST00000532849	ENST0000053284 9
RP11-164N3.1	11	88051517	88051718	S18_peak_579	promoter-TSS (ENST00000532849)	ENST0000053284 9
ROR1-AS1	1	64187044	64187209	S18_peak_58	Intergenic between ENST00000424995 and ENST00000611228	ENST0000042499 5
CTSC	11	88350978	88351143	S18_peak_580	Intergenic between ENST00000529974 and ENST00000515281	ENST0000052997 4
GRM5-AS1	11	88521295	88521537	S18_peak_581	intron (ENST00000526448, intron 4 of 5)	ENST0000052644 8
GRM5	11	88720763	88720928	S18_peak_582	intron (ENST00000305447, intron 2 of 8)	ENST0000030544 7
GRM5	11	88727729	88727894	S18_peak_583	intron (ENST00000305447, intron 2 of 8)	ENST0000030544 7
GRM5	11	88885401	88885685	S18_peak_584	intron (ENST00000305447, intron 1 of 8)	ENST0000030544 7
FOLH1B	11	89696489	89696657	S18_peak_585	intron (ENST00000525540, intron 12 of 13)	ENST0000052554 0
RP11-358N4.6	11	89832794	89832959	S18_peak_586	Intergenic between ENST00000507987 and ENST00000532912	ENST0000050798 7
RP11-529A4.4	11	89927197	89927362	S18_peak_587	Intergenic between ENST00000526739 and ENST00000530354	ENST0000053035 4
DISC1FP1	11	90660016	90660281	S18_peak_588	intron (ENST00000562245, intron 3 of 6)	ENST0000056224 5
DISC1FP1	11	90693335	90693597	S18_peak_589	intron (ENST00000562245, intron 3 of 6)	ENST0000056224 5
RP4-700A9.1	1	65204243	65204473	S18_peak_59	Intergenic between ENST00000439662 and ENST00000395325	ENST0000043966 2
RP11-201M22.1	11	91735520	91735685	S18_peak_590	Intergenic between ENST00000623291 and ENST00000577699	ENST0000057769 9
NDUFB11P1	11	92290667	92290842	S18_peak_591	Intergenic between ENST00000497708 and ENST00000455818	ENST0000045581 8
FAT3	11	92569653	92569818	S18_peak_592	intron (ENST00000525166, intron 2 of 26)	ENST0000052516 6
FAT3	11	92766603	92766818	S18_peak_593	intron (ENST00000525166, intron 5 of 26)	ENST0000052516 6
SNRPGP16	11	92946489	92946683	S18_peak_594	Intergenic between ENST00000533275 and ENST00000532770	ENST0000053327 5
RP11-288E14.2	11	93508334	93508534	S18_peak_595	Intergenic between ENST00000623739 and ENST00000533701	ENST0000053370 1
RP11-288E14.2	11	93517763	93517954	S18_peak_596	Intergenic between ENST00000623739 and ENST00000533701	ENST0000053370 1
RP11-288E14.2	11	93523197	93523370	S18_peak_597	Intergenic between ENST00000623739 and	ENST0000053370 1

					ENST00000533701	
RP11-288E14.2	11	93528636	93528816	S18_peak_598	Intergenic between ENST00000623739 and ENST00000533701	ENST0000053370 1
Y_RNA	11	93896676	93896841	S18_peak_599	Intergenic between ENST00000384184 and ENST00000534879	ENST0000038418 4
RNU6-991P	1	8276214	8276379	S18_peak_6	Intergenic between ENST00000424689 and ENST00000516168	ENST0000051616 8
LEPR	1	65446585	65446840	S18_peak_60	intron (ENST00000371059, intron 2 of 19)	ENST0000037105 9
RP11-867G2.8	11	94668315	94668519	S18_peak_600	intron (ENST00000536540, intron 4 of 4)	ENST0000053654 0
RP11-867G2.8	11	94716727	94716968	S18_peak_601	intron (ENST00000536540, intron 1 of 4)	ENST0000053654 0
ENDOD1	11	95122972	95123326	S18_peak_602	intron (ENST00000278505, intron 1 of 1)	ENST0000027850 5
RP11-712B9.2	11	95218026	95218253	S18_peak_603	intron (ENST00000534891, intron 2 of 3)	ENST0000053489 1
RP11-697H10.1	11	95952751	95953160	S18_peak_604	Intergenic between ENST00000410646 and ENST00000472927	ENST0000047292 7
RP11-697H10.1	11	96073290	96073455	S18_peak_605	Intergenic between ENST00000472927 and ENST00000582890	ENST0000047292 7
RP11-697H10.1	11	96112009	96112174	S18_peak_606	Intergenic between ENST00000472927 and ENST00000582890	ENST0000047292 7
MIR1260B	11	96281997	96282162	S18_peak_607	Intergenic between ENST00000472927 and ENST00000582890	ENST0000058289 0
RP11-49K4.2	11	96548961	96549126	S18_peak_608	Intergenic between ENST00000543403 and ENST00000527528	ENST0000054340 3
RP11-49K4.2	11	96549386	96549624	S18_peak_609	Intergenic between ENST00000543403 and ENST00000527528	ENST0000054340 3
SGIP1	1	66514643	66514808	S18_peak_61	Intergenic between ENST00000480109 and ENST00000371037	ENST0000037103 7
RP11-360K13.2	11	96737956	96738207	S18_peak_610	Intergenic between ENST00000529375 and ENST00000528254	ENST0000052937 5
RP11-360K13.2	11	96789263	96789428	S18_peak_611	Intergenic between ENST00000529375 and ENST00000528254	ENST0000052937 5
RP11-693I21.1	11	97047872	97048217	S18_peak_612	Intergenic between ENST00000528254 and ENST00000604675	ENST0000060467 5
RP11-882G5.1	11	97444008	97444173	S18_peak_613	Intergenic between ENST00000529088 and ENST00000362445	ENST0000052908 8
RNA5SP347	11	97574234	97574517	S18_peak_614	Intergenic between ENST00000529088 and ENST00000362445	ENST0000036244 5
RNA5SP347	11	97626680	97626845	S18_peak_615	Intergenic between ENST00000529088 and	ENST0000036244 5

					ENST00000362445	
RNA5SP347	11	97690137	97690358	S18_peak_616	Intergenic between ENST00000362445 and ENST00000526385	ENST0000036244 5
RNA5SP347	11	97731794	97731959	S18_peak_617	Intergenic between ENST00000362445 and ENST00000526385	ENST0000036244 5
RP11-684B20.1	11	98136800	98137168	S18_peak_618	Intergenic between ENST00000531194 and ENST00000527289	ENST0000053119 4
RP11-115E19.1	11	98774388	98774565	S18_peak_619	Intergenic between ENST00000532109 and ENST00000527345	ENST0000053210 9
SGIP1	1	66531315	66531480	S18_peak_62	Intergenic between ENST00000480109 and ENST00000371037	ENST0000037103 7
RP11-99C10.1	11	98889650	98889815	S18_peak_620	Intergenic between ENST00000532109 and ENST00000527345	ENST0000052734 5
RN7SKP53	11	99573378	99573592	S18_peak_621	Intergenic between ENST00000411337 and ENST00000533268	ENST0000041133 7
RN7SKP53	11	99719241	99719516	S18_peak_622	Intergenic between ENST00000411337 and ENST00000533268	ENST0000041133 7
RPA2P3	11	100098716	10009895 1	S18_peak_623	Intergenic between ENST00000411337 and ENST00000533268	ENST0000053326 8
RPA2P3	11	100435576	10043575 9	S18_peak_624	Intergenic between ENST00000533268 and ENST00000476452	ENST0000053326 8
PGR	11	101044634	10104494 1	S18_peak_625	intron (ENST00000325455, intron 6 of 7)	ENST0000032545 5
RP11-788M5.3	11	101297575	10129782 9	S18_peak_626	Intergenic between ENST00000526107 and ENST00000344327	ENST0000052610 7
RP11-748H22.1	11	101647461	10164777 7	S18_peak_627	Intergenic between ENST00000526526 and ENST00000530032	ENST0000052652 6
CEP126	11	101921765	10192193 0	S18_peak_628	intron (ENST00000263468, intron 1 of 10)	ENST0000026346 8
CEP126	11	101985300	10198546 5	S18_peak_629	intron (ENST00000263468, intron 8 of 10)	ENST0000026346 8
IL23R	1	67259009	67259174	S18_peak_63	exon (ENST00000347310, exon 11 of 11)	ENST0000034731 0
YAP1	11	102170653	10217083 1	S18_peak_630	intron (ENST00000524575, intron 3 of 8)	ENST0000052457 5
YAP1	11	102193691	10219385 6	S18_peak_631	intron (ENST00000524575, intron 4 of 8)	ENST0000052457 5
WTAPP1	11	102746632	10274687 6	S18_peak_632	Intergenic between ENST00000236826 and ENST00000371455	ENST0000037145 5
RP11-563P16.1	11	103564201	10356436 6	S18_peak_633	Intergenic between ENST00000528866 and ENST00000533459	ENST0000053345 9
PDGFD	11	103947667	10394783 2	S18_peak_634	intron (ENST00000393158, intron 3 of 6)	ENST0000039315 8

RNU6-277P	11	105960353	105960518	S18_peak_635	Intergenic between ENST00000410731 and ENST00000516272	ENST00000516272
CWF19L2	11	107356884	107357135	S18_peak_636	intron (ENST00000282251, intron 12 of 17)	ENST00000282251
CUL5	11	108059735	108059988	S18_peak_637	intron (ENST00000393094, intron 7 of 18)	ENST00000393094
NPAT	11	108149282	108149447	S18_peak_638	Intergenic between ENST00000299355 and ENST00000278612	ENST00000278612
ATM	11	108338238	108338403	S18_peak_639	Intergenic between ENST00000531525 and ENST00000323468	ENST00000531525
CASP3P1	1	70599015	70599180	S18_peak_64	Intergenic between ENST00000456361 and ENST00000446037	ENST00000446037
DDX10	11	108927301	108927466	S18_peak_640	intron (ENST00000322536, intron 17 of 17)	ENST00000322536
RP11-25I9.2	11	109013001	109013189	S18_peak_641	intron (ENST00000526041, intron 1 of 1)	ENST00000526041
RP11-344L21.1	11	110041673	110041986	S18_peak_642	Intergenic between ENST00000603241 and ENST00000525414	ENST00000525414
RP11-794P6.2	11	111443938	111444103	S18_peak_643	Intergenic between ENST00000528614 and ENST00000635926	ENST00000635926
NXPE4	11	114592289	114592454	S18_peak_644	intron (ENST00000375478, intron 2 of 5)	ENST00000375478
ACA59	11	115075546	115075768	S18_peak_645	Intergenic between ENST00000389586 and ENST00000517061	ENST00000517061
TMPRSS13	11	117902324	117902593	S18_peak_646	intron (ENST00000528626, intron 11 of 11)	ENST00000528626
TMPRSS4-AS1	11	118013442	118013793	S18_peak_647	intron (ENST00000636151, intron 6 of 6)	ENST00000636151
TMPRSS4-AS1	11	118022220	118022452	S18_peak_648	intron (ENST00000636151, intron 3 of 6)	ENST00000636151
SCN4B	11	118139807	118140008	S18_peak_649	intron (ENST00000324727, intron 4 of 4)	ENST00000324727
RNU6-161P	1	76739418	76739688	S18_peak_65	Intergenic between ENST00000420977 and ENST00000363051	ENST00000363051
UBE4A	11	118377317	118377502	S18_peak_650	intron (ENST00000431736, intron 10 of 19)	ENST00000431736
DDX6	11	118786164	118786329	S18_peak_651	exon (ENST00000534980, exon 2 of 14)	ENST00000534980
RN7SL688P	11	118934526	118934731	S18_peak_652	Intergenic between ENST00000471754 and ENST00000264031	ENST00000471754
NECTIN1	11	119687709	119687874	S18_peak_653	intron (ENST00000341398, intron 1 of 7)	ENST00000341398
RP11-831A10.2	11	119926833	119926998	S18_peak_654	Intergenic between ENST00000527539 and ENST00000534055	ENST00000534055
GRIK4	11	120698643	120698808	S18_peak_655	intron (ENST00000438375, intron 2 of 19)	ENST00000438375
GRIK4	11	120796857	120797025	S18_peak_656	intron (ENST00000438375, intron 2 of 19)	ENST00000438375

GRIK4	11	120844699	120844949	S18_peak_657	intron (ENST00000438375, intron 7 of 19)	ENST00000438375
MIR100HG	11	122241627	122241835	S18_peak_658	intron (ENST00000637700, intron 3 of 5)	ENST00000637700
UBASH3B	11	122714027	122714289	S18_peak_659	intron (ENST00000284273, intron 1 of 13)	ENST00000284273
PIGK	1	77146451	77146660	S18_peak_66	intron (ENST00000370812, intron 9 of 10)	ENST00000370812
RPL34P23	11	123042223	123042491	S18_peak_660	Intergenic between ENST00000469983 and ENST00000497201	ENST00000469983
CTD-2216M2.1	11	123226819	123226984	S18_peak_661	intron (ENST00000531681, intron 4 of 4)	ENST00000531681
OR6M3P	11	123869566	123869731	S18_peak_662	Intergenic between ENST00000531567 and ENST00000375026	ENST00000531567
HEPACAM	11	124917507	124917672	S18_peak_663	Intergenic between ENST00000524433 and ENST00000298251	ENST00000298251
PKNOX2	11	125179927	125180260	S18_peak_664	intron (ENST00000298282, intron 1 of 12)	ENST00000298282
PKNOX2	11	125338826	125338991	S18_peak_665	intron (ENST00000298282, intron 3 of 12)	ENST00000298282
PATE1	11	125757265	125757460	S18_peak_666	Intergenic between ENST00000305738 and ENST00000358524	ENST00000305738
CDON	11	126012527	126012723	S18_peak_667	intron (ENST00000392693, intron 7 of 19)	ENST00000392693
FAM118B	11	126245582	126245747	S18_peak_668	intron (ENST00000533050, intron 4 of 8)	ENST00000533050
KIRREL3	11	126556170	126556335	S18_peak_669	intron (ENST00000525144, intron 2 of 16)	ENST00000525144
USP33	1	77756642	77756830	S18_peak_67	intron (ENST00000370794, intron 1 of 23)	ENST00000370794
RP11-702B10.2	11	128153395	128153560	S18_peak_670	Intergenic between ENST00000526843 and ENST00000609911	ENST00000609911
RP11-702B10.1	11	128291155	128291353	S18_peak_671	Intergenic between ENST00000609260 and ENST00000392668	ENST00000609260
ETS1	11	128394330	128394495	S18_peak_672	Intergenic between ENST00000609260 and ENST00000392668	ENST00000392668
FLI1	11	128750594	128750759	S18_peak_673	intron (ENST00000534087, intron 2 of 9)	ENST00000534087
FLI1	11	128810769	128810934	S18_peak_674	exon (ENST00000534087, exon 10 of 10)	ENST00000534087
RNU6-874P	11	129221413	129221578	S18_peak_675	Intergenic between ENST00000362390 and ENST00000531501	ENST00000362390
BARX2	11	129472288	129472453	S18_peak_676	Intergenic between ENST00000281437 and ENST00000492051	ENST00000281437
RP11-507F16.1	11	129725907	129726144	S18_peak_677	Intergenic between ENST00000631195 and ENST00000529684	ENST00000529684
PRDM10	11	129914454	129914619	S18_peak_678	intron (ENST00000358825, intron 18 of 21)	ENST00000358825

PRDM10	11	129948495	129948720	S18_peak_679	intron (ENST00000358825, intron 4 of 21)	ENST00000358825
LINC01362	1	82796505	82796752	S18_peak_68	Intergenic between ENST00000420549 and ENST00000452901	ENST00000452901
ZBTB44	11	130275767	130275932	S18_peak_680	intron (ENST00000445008, intron 1 of 10)	ENST00000445008
ADAMTS8	11	130418933	130419155	S18_peak_681	intron (ENST00000257359, intron 2 of 8)	ENST00000257359
NTM-AS1	11	131628928	131629093	S18_peak_682	Intergenic between ENST00000421650 and ENST00000416725	ENST00000416725
NTM-AS1	11	131680485	131680690	S18_peak_683	Intergenic between ENST00000416725 and ENST00000604842	ENST00000416725
NTM-AS1	11	131685720	131685914	S18_peak_684	Intergenic between ENST00000416725 and ENST00000604842	ENST00000416725
RP11-259P6.3	11	133870044	133870243	S18_peak_685	Intergenic between ENST00000529222 and ENST00000533871	ENST00000529222
ACAD8	11	134255227	134255394	S18_peak_686	promoter-TSS (ENST00000281182)	ENST00000281182
RP11-627G23.1	11	134461210	134461375	S18_peak_687	intron (ENST00000531319, intron 1 of 1)	ENST00000531319
AP004550.1	11	134576798	134576963	S18_peak_688	Intergenic between ENST00000532652 and ENST00000529417	ENST00000532652
WASH7P	12	19075	19281	S18_peak_689	intron (ENST00000400706, intron 2 of 10)	ENST00000400706
RP11-131L23.1	1	85296337	85296502	S18_peak_69	intron (ENST00000427819, intron 2 of 4)	ENST00000427819
KDM5A	12	378505	378924	S18_peak_690	intron (ENST00000399788, intron 3 of 27)	ENST00000399788
CCDC77	12	440941	441106	S18_peak_691	intron (ENST00000540180, intron 10 of 10)	ENST00000540180
WNK1	12	837244	837458	S18_peak_692	intron (ENST00000315939, intron 4 of 27)	ENST00000315939
RAD52	12	931034	931270	S18_peak_693	intron (ENST00000358495, intron 3 of 11)	ENST00000358495
RP11-359B12.2	12	1068614	1068779	S18_peak_694	Intergenic between ENST00000543290 and ENST00000616500	ENST00000543290
RP5-951N9.1	12	1303651	1303816	S18_peak_695	Intergenic between ENST00000616500 and ENST00000513082	ENST00000513082
RP5-951N9.1	12	1355869	1356045	S18_peak_696	Intergenic between ENST00000616500 and ENST00000513082	ENST00000513082
MIR3649	12	1665752	1665917	S18_peak_697	Intergenic between ENST00000577921 and ENST00000357103	ENST00000577921
ADIPOR2	12	1726088	1726253	S18_peak_698	intron (ENST00000357103, intron 1 of 7)	ENST00000357103
CACNA1C	12	2344935	2345141	S18_peak_699	intron (ENST00000335762, intron 3 of 47)	ENST00000335762
UBE4B	1	10174226	10174391	S18_peak_7	intron (ENST00000253251, intron 24 of 26)	ENST00000253251

RP11-131L23.1	1	85331839	85332004	S18_peak_70	intron (ENST00000427819, intron 2 of 4)	ENST00000427819
CACNA1C	12	2658191	2658356	S18_peak_700	intron (ENST00000335762, intron 35 of 47)	ENST00000335762
RP5-1063M23.3	12	3331891	3332056	S18_peak_701	intron (ENST00000635814, intron 1 of 6)	ENST00000635814
CCND2-AS1	12	4260124	4260304	S18_peak_702	intron (ENST00000539135, intron 1 of 1)	ENST00000539135
RP11-500M8.7	12	4657561	4657763	S18_peak_703	intron (ENST00000536588, intron 4 of 6)	ENST00000536588
RP11-319E16.1	12	5204969	5205134	S18_peak_704	Intergenic between ENST00000252321 and ENST00000544717	ENST00000544717
VWF	12	5871642	5871828	S18_peak_705	Intergenic between ENST00000545761 and ENST00000261405	ENST00000261405
VWF	12	5994166	5994331	S18_peak_706	intron (ENST00000261405, intron 36 of 51)	ENST00000261405
RP3-454B23.1	12	6130445	6130610	S18_peak_707	Intergenic between ENST00000468423 and ENST00000539206	ENST00000539206
RP3-454B23.1	12	6174761	6174926	S18_peak_708	Intergenic between ENST00000539206 and ENST00000009180	ENST00000539206
PIANP	12	6702666	6702831	S18_peak_709	Intergenic between ENST00000540656 and ENST00000516377	ENST00000540656
GBP3	1	89001375	89001540	S18_peak_71	Intergenic between ENST00000321792 and ENST00000370481	ENST00000370481
CD4	12	6802827	6802992	S18_peak_710	intron (ENST00000011653, intron 3 of 9)	ENST00000011653
DSTNP2	12	6885369	6885534	S18_peak_711	promoter-TSS (ENST00000602547)	ENST00000602547
LPCAT3	12	7024830	7024995	S18_peak_712	Intergenic between ENST00000261407 and ENST00000468816	ENST00000261407
RP3-461F17.1	12	7035406	7035571	S18_peak_713	Intergenic between ENST00000468816 and ENST00000543250	ENST00000468816
ACSM4	12	7332770	7332935	S18_peak_714	Intergenic between ENST00000399422 and ENST00000313599	ENST00000399422
ACSM4	12	7337192	7337357	S18_peak_715	Intergenic between ENST00000399422 and ENST00000313599	ENST00000399422
GAPDHP31	12	7564427	7564599	S18_peak_716	Intergenic between ENST00000359156 and ENST00000509325	ENST00000509325
NANOGP1	12	7892427	7892592	S18_peak_717	promoter-TSS (ENST00000607111)	ENST00000607111
LINC00937	12	8315884	8316064	S18_peak_718	intron (ENST00000544461, intron 3 of 4)	ENST00000544461
A2M	12	9112231	9112396	S18_peak_719	intron (ENST00000318602, intron 3 of 35)	ENST00000318602
HSP90B3P	1	91623035	91623200	S18_peak_72	Intergenic between ENST00000439897 and ENST00000415545	ENST00000415545

CLEC1B	12	9996747	9996948	S18_peak_720	exon (ENST00000298527, exon 4 of 6)	ENST0000029852 7
PRH1-PRR4	12	10942096	10942261	S18_peak_721	intron (ENST00000536668, intron 4 of 9)	ENST0000053666 8
RP11-434C1.2	12	11534975	11535140	S18_peak_722	Intergenic between ENST00000614160 and ENST00000536492	ENST0000053649 2
LINC01252	12	11601199	11601478	S18_peak_723	Intergenic between ENST00000541162 and ENST00000396373	ENST0000054116 2
ETV6	12	11837035	11837200	S18_peak_724	intron (ENST00000396373, intron 2 of 7)	ENST0000039637 3
BORCS5	12	12393987	12394211	S18_peak_725	intron (ENST00000314565, intron 2 of 3)	ENST0000031456 5
DUSP16	12	12552162	12552374	S18_peak_726	intron (ENST00000298573, intron 1 of 6)	ENST0000029857 3
RPL13AP20	12	12881113	12881278	S18_peak_727	Intergenic between ENST00000459725 and ENST00000014914	ENST0000045972 5
GPRC5A	12	12904275	12904440	S18_peak_728	intron (ENST00000014914, intron 1 of 3)	ENST0000001491 4
GNAI2P1	12	14274963	14275182	S18_peak_729	Intergenic between ENST00000541815 and ENST00000598995	ENST0000054181 5
BCAR3	1	93764428	93764593	S18_peak_73	intron (ENST00000370244, intron 2 of 13)	ENST0000037024 4
RP11-233G1.4	12	14850895	14851081	S18_peak_730	Intergenic between ENST00000444324 and ENST00000539261	ENST0000044432 4
ERP27	12	14930965	14931217	S18_peak_731	intron (ENST00000266397, intron 3 of 6)	ENST0000026639 7
SLC15A5	12	16249998	16250251	S18_peak_732	intron (ENST00000344941, intron 3 of 8)	ENST0000034494 1
SLC15A5	12	16253828	16253993	S18_peak_733	intron (ENST00000344941, intron 3 of 8)	ENST0000034494 1
SUPT16HP1	12	16384071	16384236	S18_peak_734	Intergenic between ENST00000396207 and ENST00000537175	ENST0000053717 5
RP11-239A17.1	12	16706309	16706474	S18_peak_735	intron (ENST00000637435, intron 1 of 6)	ENST0000063743 5
RP11-239A17.1	12	16738406	16738668	S18_peak_736	intron (ENST00000637435, intron 3 of 6)	ENST0000063743 5
RP11-1018J11.1	12	16966605	16966910	S18_peak_737	Intergenic between ENST00000418574 and ENST00000444513	ENST0000044451 3
TIMM17BP1	12	17451901	17452106	S18_peak_738	Intergenic between ENST00000545713 and ENST00000540399	ENST0000054571 3
RP11-871F6.3	12	17471831	17471996	S18_peak_739	Intergenic between ENST00000545713 and ENST00000540399	ENST0000054039 9
LINC01057	1	94813875	94814040	S18_peak_74	intron (ENST00000634870, intron 1 of 5)	ENST0000063487 0
NDFIP1P1	12	18268969	18269134	S18_peak_740	Intergenic between ENST00000538724 and ENST00000543144	ENST0000054314 4
NDFIP1P1	12	18302129	18302306	S18_peak_741	Intergenic between ENST00000538724 and	ENST0000054314 4

					ENST00000543144	
PLCZ1	12	18612201	18612410	S18_peak_742	Intergenic between ENST00000543668 and ENST00000266505	ENST0000026650 5
PLCZ1	12	18694173	18694390	S18_peak_743	intron (ENST00000266505, intron 12 of 14)	ENST0000026650 5
RP11-841C19.1	12	19556020	19556185	S18_peak_744	Intergenic between ENST00000498395 and ENST00000364015	ENST0000049839 5
RP11-405A12.2	12	19921084	19921260	S18_peak_745	intron (ENST00000535764, intron 1 of 4)	ENST0000053576 4
PDE3A	12	20532814	20532979	S18_peak_746	intron (ENST00000359062, intron 1 of 15)	ENST0000035906 2
PDE3A	12	20533526	20533722	S18_peak_747	intron (ENST00000359062, intron 1 of 15)	ENST0000035906 2
RP11-545J16.1	12	20936502	20936667	S18_peak_748	intron (ENST00000381541, intron 3 of 13)	ENST0000038154 1
PYROXD1	12	21449795	21449960	S18_peak_749	intron (ENST00000240651, intron 4 of 11)	ENST0000024065 1
LINC01057	1	94849265	94849430	S18_peak_75	intron (ENST00000634339, intron 1 of 5)	ENST0000063433 9
RP11-114G22.1	12	22699580	22699745	S18_peak_750	Intergenic between ENST00000612441 and ENST00000413794	ENST0000041379 4
RP11-114G22.1	12	22954769	22954934	S18_peak_751	intron (ENST00000538317, intron 3 of 4)	ENST0000053831 7
SOX5	12	23707332	23707497	S18_peak_752	intron (ENST00000451604, intron 6 of 14)	ENST0000045160 4
RP11-444D3.1	12	24250048	24250232	S18_peak_753	intron (ENST00000540811, intron 4 of 4)	ENST0000054081 1
RP11-444D3.1	12	24256823	24256988	S18_peak_754	intron (ENST00000540811, intron 4 of 4)	ENST0000054081 1
C12orf77	12	24989847	24990012	S18_peak_755	Intergenic between ENST00000396004 and ENST00000623395	ENST0000062339 5
C12orf77	12	25011225	25011416	S18_peak_756	Intergenic between ENST00000623395 and ENST00000354454	ENST0000062339 5
RP11-707G18.1	12	25374241	25374477	S18_peak_757	Intergenic between ENST00000620933 and ENST00000622671	ENST0000062267 1
MIR4302	12	25855711	25856011	S18_peak_758	Intergenic between ENST00000545704 and ENST00000638058	ENST0000063805 8
RP11-283G6.4	12	26242006	26242171	S18_peak_759	intron (ENST00000540392, intron 2 of 3)	ENST0000054039 2
ALG14	1	95068927	95069092	S18_peak_76	intron (ENST00000370205, intron 1 of 3)	ENST0000037020 5
ITPR2	12	26596389	26596720	S18_peak_760	intron (ENST00000381340, intron 31 of 56)	ENST0000038134 0
ITPR2	12	26663087	26663312	S18_peak_761	intron (ENST00000381340, intron 15 of 56)	ENST0000038134 0
ARNTL2	12	27398134	27398299	S18_peak_762	intron (ENST00000546179, intron 7 of 13)	ENST0000054617 9
RP11-874G11.1	12	28629885	28630159	S18_peak_763	Intergenic between ENST00000463466 and ENST00000537327	ENST0000046346 6

RP11-874G11.1	12	28635774	28635950	S18_peak_764	Intergenic between ENST00000463466 and ENST00000537327	ENST0000046346 6
RP11-977P2.1	12	28895136	28895464	S18_peak_765	Intergenic between ENST00000537327 and ENST00000614376	ENST0000053732 7
RP11-31A23.1	12	28967393	28967620	S18_peak_766	Intergenic between ENST00000537327 and ENST00000614376	ENST0000061437 6
RP11-31A23.1	12	28972312	28972477	S18_peak_767	Intergenic between ENST00000537327 and ENST00000614376	ENST0000061437 6
OVCH1	12	29497733	29497898	S18_peak_768	Intergenic between ENST00000318184 and ENST00000256062	ENST0000031818 4
RP11-776A13.4	12	30064973	30065138	S18_peak_769	Intergenic between ENST00000480369 and ENST00000618803	ENST0000061880 3
RP4-586O15.1	1	95330640	95330861	S18_peak_77	Intergenic between ENST00000423410 and ENST00000438093	ENST0000042341 0
RP11-776A13.4	12	30081680	30081933	S18_peak_770	Intergenic between ENST00000480369 and ENST00000618803	ENST0000061880 3
RNA5SP356	12	30302818	30303003	S18_peak_771	Intergenic between ENST00000551287 and ENST00000515972	ENST0000051597 2
RP11-776A13.2	12	30500977	30501286	S18_peak_772	Intergenic between ENST00000551867 and ENST00000256079	ENST0000055186 7
RP11-77I22.4	12	30843064	30843245	S18_peak_773	Intergenic between ENST00000551972 and ENST00000550612	ENST0000055061 2
RP11-771K4.1	12	31357583	31357831	S18_peak_774	Intergenic between ENST00000313737 and ENST00000541749	ENST0000054174 9
DENND5B	12	31548332	31548497	S18_peak_775	intron (ENST00000389082, intron 1 of 20)	ENST0000038908 2
RPL12P32	12	31930194	31930359	S18_peak_776	Intergenic between ENST00000498198 and ENST00000484010	ENST0000048401 0
RP11-843B15.1	12	32074768	32074933	S18_peak_777	Intergenic between ENST00000547280 and ENST00000605512	ENST0000054728 0
RP11-843B15.1	12	32077270	32077441	S18_peak_778	Intergenic between ENST00000547280 and ENST00000605512	ENST0000054728 0
YARS2	12	32753172	32753367	S18_peak_779	intron (ENST00000324868, intron 2 of 4)	ENST0000032486 8
RP11-411H5.1	1	101631474	10163176 0	S18_peak_78	Intergenic between ENST00000444327 and ENST00000415532	ENST0000041553 2
RP11-267D19.2	12	32957843	32958081	S18_peak_780	Intergenic between ENST00000462203 and ENST00000553060	ENST0000055306 0
RP11-267D19.1	12	33139421	33139586	S18_peak_781	Intergenic between ENST00000549660 and ENST00000228567	ENST0000054966 0

SYT10	12	33341080	33341245	S18_peak_782	Intergenic between ENST00000549660 and ENST00000228567	ENST0000022856 7
SYT10	12	33397945	33398110	S18_peak_783	intron (ENST00000228567, intron 3 of 6)	ENST0000022856 7
RNU6-472P	12	33869151	33869316	S18_peak_784	Intergenic between ENST00000603103 and ENST00000384299	ENST0000038429 9
RP11-847H18.2	12	34038817	34039101	S18_peak_785	intron (ENST00000501954, intron 1 of 1)	ENST0000050195 4
AK6P1	12	34350191	34350356	S18_peak_786	Intergenic between ENST00000540219 and ENST00000547456	ENST0000054021 9
AK6P1	12	34372838	34373003	S18_peak_787	Intergenic between ENST00000540219 and ENST00000547456	ENST0000054021 9
AK6P1	12	34500175	34500350	S18_peak_788	Intergenic between ENST00000540219 and ENST00000547456	ENST0000054021 9
AK6P1	12	34556619	34556976	S18_peak_789	Intergenic between ENST00000540219 and ENST00000547456	ENST0000054021 9
RP11-202K23.1	1	102534994	10253515 9	S18_peak_79	Intergenic between ENST00000447916 and ENST00000414418	ENST0000044791 6
AK6P1	12	34589330	34589503	S18_peak_790	Intergenic between ENST00000540219 and ENST00000547456	ENST0000054021 9
AK6P1	12	34910136	34910303	S18_peak_791	Intergenic between ENST00000540219 and ENST00000547456	ENST0000054021 9
AK6P1	12	34953212	34953403	S18_peak_792	Intergenic between ENST00000540219 and ENST00000547456	ENST0000054021 9
AK6P1	12	35557981	35558155	S18_peak_793	Intergenic between ENST00000540219 and ENST00000547456	ENST0000054021 9
ZNF970P	12	35967663	35967843	S18_peak_794	Intergenic between ENST00000540219 and ENST00000547456	ENST0000054745 6
ZNF970P	12	36270132	36270301	S18_peak_795	Intergenic between ENST00000540219 and ENST00000547456	ENST0000054745 6
ZNF970P	12	36293317	36293489	S18_peak_796	Intergenic between ENST00000540219 and ENST00000547456	ENST0000054745 6
ZNF970P	12	36306445	36306705	S18_peak_797	Intergenic between ENST00000540219 and ENST00000547456	ENST0000054745 6
ZNF970P	12	36372693	36372858	S18_peak_798	Intergenic between ENST00000540219 and ENST00000547456	ENST0000054745 6
ZNF970P	12	37027628	37027793	S18_peak_799	Intergenic between ENST00000540219 and ENST00000547456	ENST0000054745 6
KIF1B	1	10319159	10319324	S18_peak_8	intron (ENST00000263934, intron 20 of 46)	ENST0000026393 4
RP11-202K23.1	1	102545846	10254601	S18_peak_80	Intergenic between	ENST0000044791

			1		ENST00000447916 and ENST00000414418	6
AK6P2	12	37873233	37873398	S18_peak_800	Intergenic between ENST00000547456 and ENST00000549020	ENST0000054902 0
CLUHP8	12	38111665	38111841	S18_peak_801	Intergenic between ENST00000547527 and ENST00000620973	ENST0000054752 7
pRNA	12	38137210	38137375	S18_peak_802	Intergenic between ENST00000547527 and ENST00000620973	ENST0000062097 3
FAM166AP9	12	38205344	38205539	S18_peak_803	promoter-TSS (ENST00000548633)	ENST0000054863 3
CPNE8	12	38797634	38797911	S18_peak_804	intron (ENST00000331366, intron 6 of 19)	ENST0000033136 6
KIF21A	12	39280183	39280436	S18_peak_805	Intergenic between ENST00000603311 and ENST00000361961	ENST0000036196 1
KIF21A	12	39316654	39316819	S18_peak_806	intron (ENST00000361961, intron 28 of 36)	ENST0000036196 1
KIF21A	12	39451429	39451594	S18_peak_807	Intergenic between ENST00000541463 and ENST00000396414	ENST0000054146 3
SLC2A13	12	39926699	39926864	S18_peak_808	intron (ENST00000280871, intron 4 of 9)	ENST0000028087 1
PDZRN4	12	41313521	41313686	S18_peak_809	intron (ENST00000402685, intron 3 of 9)	ENST0000040268 5
RP5-936J12.1	1	102773348	10277353 6	S18_peak_81	intron (ENST00000414418, intron 2 of 6)	ENST0000041441 8
PDZRN4	12	41346217	41346382	S18_peak_810	intron (ENST00000402685, intron 3 of 9)	ENST0000040268 5
MTND2P17	12	41695638	41695803	S18_peak_811	Intergenic between ENST00000516555 and ENST00000550281	ENST0000055028 1
MTND1P24	12	41719188	41719381	S18_peak_812	Intergenic between ENST00000552175 and ENST00000552712	ENST0000055217 5
RP11-440G2.2	12	42022999	42023231	S18_peak_813	Intergenic between ENST00000546669 and ENST00000604396	ENST0000060439 6
RP11-440G2.2	12	42072493	42072658	S18_peak_814	Intergenic between ENST00000604396 and ENST00000398675	ENST0000060439 6
PPHLN1	12	42371185	42371456	S18_peak_815	intron (ENST00000395580, intron 5 of 10)	ENST0000039558 0
PRICKLE1	12	42491632	42491885	S18_peak_816	intron (ENST00000345127, intron 1 of 7)	ENST0000034512 7
MRPS36P5	12	43101343	43101558	S18_peak_817	Intergenic between ENST00000551909 and ENST00000553211	ENST0000055190 9
ADAMTS20	12	43483935	43484100	S18_peak_818	intron (ENST00000389420, intron 7 of 38)	ENST0000038942 0
EEF1A1P17	12	43671684	43671936	S18_peak_819	Intergenic between ENST00000547505 and ENST00000416848	ENST0000054750 5
CDK4PS	1	105105180	10510536 0	S18_peak_82	Intergenic between ENST00000432567 and ENST00000454213	ENST0000045421 3

PUS7L	12	43705442	43705607	S18_peak_820	Intergenic between ENST00000547505 and ENST00000416848	ENST0000041684 8
TMEM117	12	43953657	43953822	S18_peak_821	intron (ENST00000551577, intron 3 of 6)	ENST0000055157 7
TMEM117	12	43955081	43955246	S18_peak_822	intron (ENST00000551577, intron 3 of 6)	ENST0000055157 7
NELL2	12	44585249	44585416	S18_peak_823	intron (ENST00000429094, intron 15 of 19)	ENST0000042909 4
NELL2	12	44911006	44911171	S18_peak_824	intron (ENST00000333837, intron 1 of 20)	ENST0000033383 7
RNA5SP361	12	45126072	45126237	S18_peak_825	Intergenic between ENST00000362686 and ENST00000256692	ENST0000036268 6
PLEKHA8P1	12	45178454	45178619	S18_peak_826	intron (ENST00000256692, intron 1 of 2)	ENST0000025669 2
PLEKHA8P1	12	45209244	45209436	S18_peak_827	intron (ENST00000256692, intron 1 of 2)	ENST0000025669 2
RP11-352M15.1	12	45651710	45651900	S18_peak_828	Intergenic between ENST00000549223 and ENST00000609803	ENST0000054922 3
RP11-352M15.2	12	45686201	45686366	S18_peak_829	Intergenic between ENST00000549223 and ENST00000609803	ENST0000060980 3
RP11-356N1.2	1	108045066	10804523 1	S18_peak_83	intron (ENST00000419428, intron 2 of 4)	ENST0000041942 8
MIR4494	12	47378314	47378560	S18_peak_830	Intergenic between ENST00000577654 and ENST00000552320	ENST0000057765 4
SENP1	12	48053339	48053504	S18_peak_831	intron (ENST00000552189, intron 14 of 18)	ENST0000055218 9
RP11-370I10.2	12	48258766	48259069	S18_peak_832	intron (ENST00000547523, intron 2 of 3)	ENST0000054752 3
ZNF641	12	48343401	48343566	S18_peak_833	exon (ENST00000448928, exon 5 of 5)	ENST0000044892 8
OR8S1	12	48515112	48515361	S18_peak_834	Intergenic between ENST00000494130 and ENST00000310194	ENST0000031019 4
RNU6-600P	12	48890678	48890843	S18_peak_835	Intergenic between ENST00000309739 and ENST00000362524	ENST0000036252 4
Y_RNA	12	49158368	49158569	S18_peak_836	Intergenic between ENST00000363439 and ENST00000295766	ENST0000036343 9
TUBA1A	12	49183227	49183515	S18_peak_837	Intergenic between ENST00000363439 and ENST00000295766	ENST0000029576 6
Metazoa_SRP	12	49193580	49193787	S18_peak_838	Intergenic between ENST00000295766 and ENST00000616942	ENST0000061694 2
FMNL3	12	49683446	49683667	S18_peak_839	intron (ENST00000335154, intron 1 of 25)	ENST0000033515 4
FAM102B	1	108604906	10860521 3	S18_peak_84	intron (ENST00000370035, intron 2 of 10)	ENST0000037003 5
TMBIM6	12	49767294	49767459	S18_peak_840	Intergenic between ENST00000267115 and ENST00000552208	ENST0000026711 5
FAIM2	12	49887166	49887440	S18_peak_841	intron (ENST00000320634,	ENST0000032063

					intron 11 of 11)	4
AQP6	12	49973432	49973597	S18_peak_842	exon (ENST00000489786, exon 4 of 7)	ENST0000048978 6
DIP2B	12	50522219	50522384	S18_peak_843	intron (ENST00000301180, intron 1 of 37)	ENST0000030118 0
BIN2	12	51294320	51294511	S18_peak_844	intron (ENST00000615107, intron 9 of 12)	ENST0000061510 7
GALNT6	12	51378273	51378723	S18_peak_845	intron (ENST00000543196, intron 2 of 10)	ENST0000054319 6
GALNT6	12	51386321	51386486	S18_peak_846	intron (ENST00000356317, intron 2 of 11)	ENST0000035631 7
RP11-923I11.4	12	51824676	51824895	S18_peak_847	intron (ENST00000637934, intron 3 of 4)	ENST0000063793 4
RP11-923I11.4	12	51838158	51838365	S18_peak_848	intron (ENST00000637934, intron 4 of 4)	ENST0000063793 4
KRT87P	12	52253102	52253385	S18_peak_849	exon (ENST00000534226, exon 6 of 9)	ENST0000053422 6
FNDC7	1	108741027	10874119 2	S18_peak_85	intron (ENST00000370017, intron 11 of 12)	ENST0000037001 7
KRT81	12	52293534	52293699	S18_peak_850	Intergenic between ENST00000327741 and ENST00000552441	ENST0000032774 1
KRT72	12	52586436	52586737	S18_peak_851	intron (ENST00000537672, intron 8 of 9)	ENST0000053767 2
KRT3	12	52786957	52787122	S18_peak_852	Intergenic between ENST00000547968 and ENST00000417996	ENST0000041799 6
RP11-983P16.4	12	53043328	53043593	S18_peak_853	intron (ENST00000552905, intron 2 of 2)	ENST0000055290 5
RP11-793H13.8	12	53458056	53458223	S18_peak_854	intron (ENST00000547717, intron 7 of 12)	ENST0000054771 7
RP11-793H13.10	12	53518768	53518980	S18_peak_855	intron (ENST00000591834, intron 11 of 12)	ENST0000059183 4
CALCOCO1	12	53718806	53718971	S18_peak_856	intron (ENST00000430117, intron 7 of 13)	ENST0000043011 7
HOXC13-AS	12	53890041	53890336	S18_peak_857	Intergenic between ENST00000517244 and ENST00000512916	ENST0000051291 6
HOXC13-AS	12	53927480	53927645	S18_peak_858	Intergenic between ENST00000517244 and ENST00000512916	ENST0000051291 6
HOXC4	12	54042018	54042206	S18_peak_859	intron (ENST00000303406, intron 1 of 3)	ENST0000030340 6
GSTM2	1	109669360	10966952 5	S18_peak_86	promoter-TSS (ENST00000241337)	ENST0000024133 7
HOXC4	12	54042738	54042948	S18_peak_860	intron (ENST00000303406, intron 1 of 3)	ENST0000030340 6
RP11-753H16.3	12	54447270	54447435	S18_peak_861	intron (ENST00000550474, intron 3 of 3)	ENST0000055047 4
OR6C1	12	55323929	55324143	S18_peak_862	Intergenic between ENST00000379668 and ENST00000379667	ENST0000037966 8
OR10P1	12	55631535	55631718	S18_peak_863	Intergenic between ENST00000607990 and ENST00000309675	ENST0000030967 5
RP11-644F5.16	12	55643375	55643665	S18_peak_864	intron (ENST00000556606, intron 1 of 1)	ENST0000055660 6

TIMELESS	12	56415189	56415408	S18_peak_865	Intergenic between ENST00000620583 and ENST00000553532	ENST0000055353 2
RNU6-879P	12	57377426	57377595	S18_peak_866	Intergenic between ENST00000548184 and ENST00000364328	ENST0000036432 8
RP11-756H6.1	12	57426177	57426342	S18_peak_867	Intergenic between ENST00000364328 and ENST00000547552	ENST0000054755 2
RP11-471N19.1	12	60980591	60980756	S18_peak_868	Intergenic between ENST00000546569 and ENST00000548612	ENST0000054861 2
RP11-769N19.2	12	62116457	62116622	S18_peak_869	Intergenic between ENST00000487417 and ENST00000547084	ENST0000054708 4
LINC01356	1	112790355	11279052 0	S18_peak_87	Intergenic between ENST00000458353 and ENST00000433505	ENST0000043350 5
USP15	12	62394539	62394704	S18_peak_870	intron (ENST00000353364, intron 18 of 20)	ENST0000035336 4
PPM1H	12	62742672	62742837	S18_peak_871	intron (ENST00000228705, intron 4 of 9)	ENST0000022870 5
PPM1H	12	62830159	62830324	S18_peak_872	intron (ENST00000228705, intron 2 of 9)	ENST0000022870 5
RP11-715H19.2	12	63206897	63207062	S18_peak_873	Intergenic between ENST00000624438 and ENST00000552536	ENST0000062443 8
RP11-415I12.3	12	63738211	63738376	S18_peak_874	intron (ENST00000509615, intron 1 of 1)	ENST0000050961 5
RPS11P6	12	64290817	64290982	S18_peak_875	intron (ENST00000535684, intron 2 of 4)	ENST0000053568 4
RPS11P6	12	64346818	64346983	S18_peak_876	intron (ENST00000535684, intron 2 of 4)	ENST0000053568 4
RP11-230G5.2	12	65523790	65523955	S18_peak_877	intron (ENST00000537250, intron 2 of 3)	ENST0000053725 0
RP11-123O10.3	12	67097613	67097833	S18_peak_878	Intergenic between ENST00000502700 and ENST00000545905	ENST0000050270 0
GGTA2P	12	67221350	67221630	S18_peak_879	Intergenic between ENST00000545905 and ENST00000543442	ENST0000054344 2
RP4-590F24.2	1	114030123	11403028 8	S18_peak_88	Intergenic between ENST00000604433 and ENST00000632517	ENST0000063251 7
IFNG-AS1	12	68208421	68208586	S18_peak_880	intron (ENST00000536914, intron 5 of 5)	ENST0000053691 4
RP11-81H14.3	12	68464252	68464580	S18_peak_881	Intergenic between ENST00000542875 and ENST00000216407	ENST0000021640 7
LINC01481	12	70182508	70182691	S18_peak_882	intron (ENST00000549419, intron 2 of 2)	ENST0000054941 9
CTD-2021H9.1	12	70976899	70977123	S18_peak_883	Intergenic between ENST00000384657 and ENST00000552098	ENST0000055209 8
TRHDE	12	72326437	72326647	S18_peak_884	intron (ENST00000261180, intron 2 of 18)	ENST0000026118 0
RP11-314D7.3	12	73341660	73341825	S18_peak_885	Intergenic between ENST00000548029 and	ENST0000054802 9

					ENST00000474343	
RP11-166B14.1	12	73541269	73541434	S18_peak_886	Intergenic between ENST00000548029 and ENST00000474343	ENST0000047434 3
ATXN7L3B	12	74535695	74535917	S18_peak_887	Intergenic between ENST00000546480 and ENST00000519948	ENST0000051994 8
RP11-361A23.3	12	74820742	74820983	S18_peak_888	Intergenic between ENST00000547218 and ENST00000549762	ENST0000054721 8
RP11-114H23.1	12	75615663	75615899	S18_peak_889	intron (ENST00000552856, intron 4 of 4)	ENST0000055285 6
DENND2C	1	114582524	11458268 9	S18_peak_89	TTS (ENST00000393276)	ENST0000039327 6
ZDHHC17	12	76788866	76789470	S18_peak_890	intron (ENST00000426126, intron 1 of 16)	ENST0000042612 6
RP11-781A6.1	12	77755408	77755573	S18_peak_891	Intergenic between ENST00000552736 and ENST00000549993	ENST0000054999 3
RP11-754N21.1	12	78400201	78400366	S18_peak_892	intron (ENST00000552230, intron 1 of 2)	ENST0000055223 0
RP11-123M21.2	12	78722891	78723056	S18_peak_893	Intergenic between ENST00000547089 and ENST00000546724	ENST0000054672 4
PPP1R12A	12	79823716	79823881	S18_peak_894	intron (ENST00000450142, intron 5 of 24)	ENST0000045014 2
PPFIA2	12	81393538	81393761	S18_peak_895	intron (ENST00000407050, intron 5 of 28)	ENST0000040705 0
TMTC2	12	82841498	82841745	S18_peak_896	intron (ENST00000321196, intron 1 of 11)	ENST0000032119 6
TMTC2	12	82907147	82907312	S18_peak_897	intron (ENST00000321196, intron 3 of 11)	ENST0000032119 6
SNORA3	12	84000146	84000311	S18_peak_898	Intergenic between ENST00000547795 and ENST00000408221	ENST0000040822 1
RP11-701B6.1	12	84592054	84592377	S18_peak_899	Intergenic between ENST00000550884 and ENST00000546698	ENST0000054669 8
DISP3	1	11526527	11526702	S18_peak_9	intron (ENST00000294484, intron 12 of 20)	ENST0000029448 4
AMPD1	1	114692767	11469293 2	S18_peak_90	intron (ENST00000520113, intron 2 of 15)	ENST0000052011 3
LRRIQ1	12	85148303	85148468	S18_peak_900	intron (ENST00000393217, intron 19 of 26)	ENST0000039321 7
RP11-324H9.1	12	86948782	86949007	S18_peak_901	Intergenic between ENST00000550014 and ENST00000624306	ENST0000062430 6
TMTC3	12	88193998	88194163	S18_peak_902	intron (ENST00000266712, intron 13 of 13)	ENST0000026671 2
RP11-654D12.2	12	89862987	89863168	S18_peak_903	Intergenic between ENST00000517014 and ENST00000547370	ENST0000054737 0
RP11-753N8.1	12	90216893	90217098	S18_peak_904	Intergenic between ENST00000548722 and ENST00000549313	ENST0000054931 3
RP11-121E16.1	12	91395714	91395895	S18_peak_905	Intergenic between ENST00000551571 and ENST00000610536	ENST0000055157 1

LINC01619	12	91959229	91959632	S18_peak_906	Intergenic between ENST00000551075 and ENST00000551843	ENST0000055184 3
RP11-756G20.1	12	92300712	92300931	S18_peak_907	intron (ENST00000615716, intron 2 of 4)	ENST0000061571 6
RP11-511B23.2	12	93197490	93197655	S18_peak_908	intron (ENST00000549930, intron 1 of 2)	ENST0000054993 0
CRADD	12	93686851	93687046	S18_peak_909	intron (ENST00000332896, intron 2 of 2)	ENST0000033289 6
HSD3B1	1	119503868	11950420 0	S18_peak_91	Intergenic between ENST00000438477 and ENST00000369413	ENST0000036941 3
RP11-74K11.1	12	94083226	94083391	S18_peak_910	Intergenic between ENST00000411194 and ENST00000548294	ENST0000054829 4
CEP83	12	94363612	94363777	S18_peak_911	intron (ENST00000339839, intron 10 of 15)	ENST0000033983 9
TMCC3	12	94646699	94646864	S18_peak_912	intron (ENST00000261226, intron 1 of 3)	ENST0000026122 6
KRT19P2	12	94894979	94895327	S18_peak_913	Intergenic between ENST00000405395 and ENST00000327772	ENST0000040539 5
FGD6	12	95075104	95075269	S18_peak_914	Intergenic between ENST00000330677 and ENST00000343958	ENST0000034395 8
VEZT	12	95291405	95291621	S18_peak_915	intron (ENST00000436874, intron 9 of 11)	ENST0000043687 4
NTN4	12	95703944	95704203	S18_peak_916	intron (ENST00000343702, intron 5 of 9)	ENST0000034370 2
ELK3	12	96256200	96256365	S18_peak_917	intron (ENST00000228741, intron 3 of 4)	ENST0000022874 1
RP11-690J15.1	12	98242695	98242860	S18_peak_918	intron (ENST00000548344, intron 1 of 4)	ENST0000054834 4
RP11-406H4.1	12	99189326	99189550	S18_peak_919	Intergenic between ENST00000547633 and ENST00000324341	ENST0000054763 3
RP11-114O18.1	1	120073812	12007399 4	S18_peak_92	Intergenic between ENST00000602566 and ENST00000608123	ENST0000060812 3
GOLGA2P5	12	100150962	10015122 9	S18_peak_920	Intergenic between ENST00000550886 and ENST00000304813	ENST0000030481 3
SLC17A8	12	100439910	10044015 2	S18_peak_921	Intergenic between ENST00000392989 and ENST00000392986	ENST0000039298 9
RNA5SP369	12	101849816	10185000 6	S18_peak_922	Intergenic between ENST00000411114 and ENST00000517054	ENST0000041111 4
RNU6-1183P	12	101864813	10186497 8	S18_peak_923	Intergenic between ENST00000411114 and ENST00000517054	ENST0000051705 4
CCDC53	12	102044008	10204417 3	S18_peak_924	intron (ENST00000240079, intron 4 of 6)	ENST0000024007 9
HELLPAR	12	102369373	10236953 8	S18_peak_925	exon (ENST00000626826, exon 1 of 1)	ENST0000062682 6
PAH	12	102856586	10285688 1	S18_peak_926	intron (ENST00000553106, intron 5 of 12)	ENST0000055310 6
STAB2	12	103575706	10357599	S18_peak_927	Intergenic between	ENST0000038888

			0		ENST00000551174 and ENST00000388887	7
GLT8D2	12	104043080	104043245	S18_peak_928	intron (ENST00000360814, intron 1 of 10)	ENST00000360814
HCFC2	12	104092602	104092773	S18_peak_929	intron (ENST00000229330, intron 9 of 14)	ENST00000229330
RP11-114O18.1	1	120080648	120080813	S18_peak_93	Intergenic between ENST00000608123 and ENST00000384420	ENST00000608123
RP11-818F20.5	12	104240849	104241014	S18_peak_930	Intergenic between ENST00000547554 and ENST00000549807	ENST00000549807
CHST11	12	104667634	104667799	S18_peak_931	intron (ENST00000303694, intron 2 of 2)	ENST00000303694
CKAP4	12	106220004	106220169	S18_peak_932	Intergenic between ENST00000548943 and ENST00000378026	ENST00000378026
LINC01498	12	108484492	108484657	S18_peak_933	intron (ENST00000502160, intron 2 of 17)	ENST00000502160
CORO1C	12	108702632	108702797	S18_peak_934	intron (ENST00000261401, intron 1 of 10)	ENST00000261401
SVOP	12	108943766	108943931	S18_peak_935	intron (ENST00000610966, intron 7 of 15)	ENST00000610966
SVOP	12	109015695	109015991	S18_peak_936	intron (ENST00000610966, intron 1 of 15)	ENST00000610966
ACACB	12	109190365	109190530	S18_peak_937	Intergenic between ENST00000541704 and ENST00000538526	ENST00000538526
MMAB	12	109548739	109548961	S18_peak_938	Intergenic between ENST00000340074 and ENST00000545712	ENST00000545712
GIT2	12	109988554	109988719	S18_peak_939	intron (ENST00000355312, intron 4 of 19)	ENST00000355312
CH17-472G23.1	1	120196911	120197115	S18_peak_94	promoter-TSS (ENST00000612320)	ENST00000612320
IFT81	12	110110524	110110708	S18_peak_940	Intergenic between ENST00000623996 and ENST00000361948	ENST00000361948
SNORD50	12	110497245	110497410	S18_peak_941	Intergenic between ENST00000365465 and ENST00000623894	ENST00000365465
RAD9B	12	110515598	110515763	S18_peak_942	intron (ENST00000392672, intron 6 of 11)	ENST00000392672
CUX2	12	111086011	111086288	S18_peak_943	intron (ENST00000261726, intron 1 of 21)	ENST00000261726
CUX2	12	111209070	111209235	S18_peak_944	intron (ENST00000261726, intron 1 of 21)	ENST00000261726
RP3-473L9.4	12	111396651	111396816	S18_peak_945	intron (ENST00000552663, intron 2 of 2)	ENST00000552663
ATXN2	12	111534841	111535153	S18_peak_946	intron (ENST00000377617, intron 5 of 24)	ENST00000377617
ATXN2	12	111536850	111537060	S18_peak_947	intron (ENST00000377617, intron 5 of 24)	ENST00000377617
BRAP	12	111669167	111669396	S18_peak_948	intron (ENST00000419234, intron 5 of 11)	ENST00000419234
RPL7AP60	12	112299836	112300148	S18_peak_949	Intergenic between ENST00000364558 and ENST00000469026	ENST00000469026

CH17-472G23.4	1	120638191	120638427	S18_peak_95	intron (ENST00000612480, intron 3 of 41)	ENST00000612480
PTPN11	12	112456351	112456549	S18_peak_950	intron (ENST00000392597, intron 6 of 10)	ENST00000392597
PTPN11	12	112477152	112477317	S18_peak_951	intron (ENST00000392597, intron 7 of 10)	ENST00000392597
PTPN11	12	112555779	112555955	S18_peak_952	Intergenic between ENST00000392597 and ENST00000637932	ENST00000392597
TPCN1	12	113298131	113298296	S18_peak_953	exon (ENST00000335509, exon 28 of 28)	ENST00000335509
MIR6762	12	113324578	113324805	S18_peak_954	Intergenic between ENST00000613340 and ENST00000545182	ENST00000613340
SDSL	12	113444118	113444361	S18_peak_955	Intergenic between ENST00000403593 and ENST00000261731	ENST00000403593
LINC01234	12	113625039	113625232	S18_peak_956	Intergenic between ENST00000551357 and ENST00000550905	ENST00000550905
RBM19	12	113917869	113918058	S18_peak_957	intron (ENST00000545145, intron 20 of 24)	ENST00000545145
RBM19	12	113934142	113934450	S18_peak_958	intron (ENST00000545145, intron 16 of 24)	ENST00000545145
MED13L	12	115916723	115917020	S18_peak_959	Intergenic between ENST00000363242 and ENST00000281928	ENST00000281928
MAP1LC3B2	12	116568991	116569319	S18_peak_960	intron (ENST00000556529, intron 1 of 1)	ENST00000556529
RP11-497G19.3	12	116602785	116602982	S18_peak_961	intron (ENST00000550742, intron 1 of 1)	ENST00000550742
RP11-497G19.3	12	116629482	116629647	S18_peak_962	Intergenic between ENST00000550742 and ENST00000546835	ENST00000550742
RP11-497G19.2	12	116701140	116701305	S18_peak_963	intron (ENST00000552718, intron 1 of 1)	ENST00000552718
NOS1	12	117233333	117233498	S18_peak_964	intron (ENST00000317775, intron 21 of 28)	ENST00000317775
TAOK3	12	118266206	118266392	S18_peak_965	intron (ENST00000392533, intron 2 of 20)	ENST00000392533
HSPB8	12	119192375	119192589	S18_peak_966	intron (ENST00000281938, intron 2 of 2)	ENST00000281938
RP11-768F21.1	12	119618009	119618174	S18_peak_967	intron (ENST00000537366, intron 1 of 3)	ENST00000537366
GCN1	12	120180273	120180438	S18_peak_968	intron (ENST00000300648, intron 5 of 57)	ENST00000300648
SIRT4	12	120308933	120309126	S18_peak_969	intron (ENST00000202967, intron 2 of 3)	ENST00000202967
RP11-344P13.1	1	121768657	121768830	S18_peak_97	Intergenic between ENST00000450546 and ENST00000612151	ENST00000450546
HNF1A	12	121000092	121000257	S18_peak_970	intron (ENST00000257555, intron 9 of 9)	ENST00000257555
RNF34	12	121404572	121404737	S18_peak_971	intron (ENST00000361234, intron 1 of 5)	ENST00000361234
RNF34	12	121411109	121411274	S18_peak_972	intron (ENST00000361234, intron 1 of 5)	ENST00000361234
KDM2B	12	121478484	12147864	S18_peak_973	intron (ENST00000377069,	ENST0000037706

			9		intron 12 of 22)	9
RP11-7M8.2	12	121859623	121859827	S18_peak_974	Intergenic between ENST00000543848 and ENST00000618674	ENST00000543848
RP11-87C12.5	12	122012776	122012941	S18_peak_975	intron (ENST00000538710, intron 1 of 1)	ENST00000538710
BCL7A	12	122034652	122034817	S18_peak_976	intron (ENST00000538010, intron 2 of 5)	ENST00000538010
KNTC1	12	122586133	122586358	S18_peak_977	intron (ENST00000333479, intron 37 of 63)	ENST00000333479
PITPNM2	12	123104807	123104972	S18_peak_978	promoter-TSS (ENST00000451868)	ENST00000451868
MPHOSPH9	12	123181924	123182122	S18_peak_979	intron (ENST00000606320, intron 13 of 23)	ENST00000606320
RNA5SP375	12	123280722	123280887	S18_peak_980	Intergenic between ENST00000602352 and ENST00000362975	ENST00000362975
DDX55	12	123617816	123617981	S18_peak_981	intron (ENST00000238146, intron 11 of 13)	ENST00000238146
ATP6V0A2	12	123730196	123730409	S18_peak_982	intron (ENST00000330342, intron 6 of 19)	ENST00000330342
CCDC92	12	123954794	123954979	S18_peak_983	intron (ENST00000238156, intron 1 of 4)	ENST00000238156
RP11-214K3.25	12	124040145	124040349	S18_peak_984	intron (ENST00000618862, intron 2 of 2)	ENST00000618862
RP11-83B20.1	12	124499503	124499701	S18_peak_985	Intergenic between ENST00000545095 and ENST00000543970	ENST00000543970
RNU6-927P	12	124901961	124902126	S18_peak_986	TTS (ENST00000516199)	ENST00000516199
RPL22P19	12	124933380	124933649	S18_peak_987	Intergenic between ENST00000583467 and ENST00000480427	ENST00000480427
BRI3BP	12	125010908	125011085	S18_peak_988	intron (ENST00000341446, intron 1 of 2)	ENST00000341446
RP11-158L12.6	12	125172823	125172988	S18_peak_989	Intergenic between ENST00000623279 and ENST00000622998	ENST00000623279
RP3-446N13.2	12	126108471	126108636	S18_peak_990	Intergenic between ENST00000624756 and ENST00000623191	ENST00000624756
LINC00944	12	126726982	126727147	S18_peak_991	Intergenic between ENST00000545535 and ENST00000540684	ENST00000540684
LINC00507	12	127912660	127912881	S18_peak_992	Intergenic between ENST00000614177 and ENST00000544645	ENST00000544645
TMEM132D	12	129365432	129365597	S18_peak_993	intron (ENST00000422113, intron 3 of 8)	ENST00000422113
RP11-474D1.4	12	129991448	129991726	S18_peak_994	Intergenic between ENST00000502221 and ENST00000561864	ENST00000561864
PIWIL1	12	130362746	130363056	S18_peak_995	intron (ENST00000245255, intron 17 of 20)	ENST00000245255
RAN	12	130878844	130879170	S18_peak_996	Intergenic between ENST00000392367 and ENST00000384123	ENST00000392367

RP11-897M7.3	12	131439097	131439262	S18_peak_997	Intergenic between ENST00000623945 and ENST00000618927	ENST00000623945
RP11-292I17.1	12	131576945	131577166	S18_peak_998	Intergenic between ENST00000624926 and ENST00000541343	ENST00000541343
RP11-495K9.10	12	131621118	131621323	S18_peak_999	Intergenic between ENST00000624871 and ENST00000542797	ENST00000624871

Supplementary Data 11, ChIRP-qPCR of seRNA LOC100506178 in S18 cells

Samples	Human hnRNPK promoter (Ct)	Mean (Ct)	Fold Enrichment
Negative control	34.76<br>35.07<br>35.11	34.98	1.00
LOC100506178	35.23<br>35.15<br>34.68	35.02	0.96
Input	34.12<br>33.89<br>33.59	33.86	

ChIRP-qPCR assay was used to detect the interactions between LOC100506178 and the promoter of hnRNPK. The fold enrichment of hnRNPK promoter with LOC100506178 was less than one. Therefore, the LOC100506178 did not bind with the promoter of hnRNPK gene.

The primer sequences for qPCR
HnRNPK promoter-F TTTCCTGTCGGATCCGGGTG
HnRNPK promoter-R TATGTGCTCCACTGGCCGAG
